# Stability of Radiomic Features across Different Region of Interest Sizes—A CT and MR Phantom Study

**DOI:** 10.3390/tomography7020022

**Published:** 2021-06-08

**Authors:** Laura J. Jensen, Damon Kim, Thomas Elgeti, Ingo G. Steffen, Bernd Hamm, Sebastian N. Nagel

**Affiliations:** Department of Radiology, Corporate Member of Freie Universität and Humboldt-Universität zu Berlin, Charité–Universitätsmedizin Berlin, Hindenburgdamm 30, 12203 Berlin, Germany; damon.kim@charite.de (D.K.); thomas.elgeti@charite.de (T.E.); ingo.steffen@charite.de (I.G.S.); bernd.hamm@charite.de (B.H.); sebastian.nagel@charite.de (S.N.N.)

**Keywords:** radiomics, texture analysis, magnetic resonance imaging, computed tomography, phantom, reproducibility, robustness

## Abstract

We aimed to evaluate radiomic features’ stability across different region of interest (ROI) sizes in CT and MR images. We chose a phantom with a homogenous internal structure so no differences for a feature extracted from ROIs of different sizes would be expected. For this, we scanned a plastic cup filled with sodium chloride solution ten times in CT and per MR sequence (T1-weighted-gradient-echo and T2-weighted-turbo-inversion-recovery-magnitude). We placed sphere-shaped ROIs of different diameters (4, 8, and 16 mm, and 4, 8, and 16 pixels) into the phantom’s center. Features were extracted using PyRadiomics. We assessed feature stability across ROI sizes with overall concordance correlation coefficients (OCCCs). Differences were tested for significance with the Mann–Whitney U-test. Of 93 features, 87 T1w-derived, 87 TIRM-derived, and 70 CT-derived features were significantly different between ROI sizes. Among MR-derived features, OCCCs showed excellent (>0.90) agreement for mean, median, and root mean squared for ROI sizes between 4 and 16 mm and pixels. We further observed excellent agreement for 10th and 90th percentile in T1w and 10th percentile in T2w TIRM images. There was no excellent agreement among the OCCCs of CT-derived features. In summary, many features indicated significant differences and only few showed excellent agreement across varying ROI sizes, although we examined a homogenous phantom. Since we considered a small phantom in an experimental setting, further studies to investigate this size effect would be necessary for a generalization. Nevertheless, we believe knowledge about this effect is crucial in interpreting radiomics studies, as features that supposedly discriminate disease entities may only indicate a systematic difference in ROI size.

## 1. Introduction

Radiomics, i.e., the extraction of various texture features from radiologic images, is an emerging and rapidly evolving technique. The aim is to detect subtle changes in imaging data imperceptible to the human eye [1].

After image acquisition, preprocessing, and segmentation of a lesion or a tumor, different subgroups of radiomic features can be extracted: shape features that describe the shape and geometry [2,3], first-order features that provide information on global characteristics of the gray level intensity distribution [4] without considering spatial relationships [3], as well as second- and higher-order features, which are derived using complex functions to describe the spatial arrangement of voxel intensity values [2,5].

Explorative analysis and modeling of these data attempt to correlate radiomic features with prediction targets, such as clinical endpoints and genomic features [6]. Especially for numerous malignant entities and solid tumors, e.g., brain tumors [7], head and neck cancer [8], renal tumors [9], or prostate cancer [10], correlations between radiomic features, histopathology, and outcome have been shown recently. Although there is a growing body of data on the application of radiomics as “quantitative imaging biomarkers” [11], the reliability of the data is not yet fully assured [12]. However, reproducibility is an essential property of a quantitative biomarker [13,14].

Radiomic feature extraction from medical images requires segmentation of the volume of interest. Variability in the segmentation process can already bias radiomic features [14,15,16]. Besides the segmentation process, voxel size in computed tomography (CT) impacts a substantial number of radiomic features [17]. Additionally, inter-scanner and inter-vendor variability of numerous radiomic features have been reported for CT [18] and MR imaging [19]. Overall, published data suggest that all steps prior to a radiomics analysis can affect feature values, including image acquisition, preprocessing, reconstruction algorithms, and applied software [6,12,20,21,22,23], increasing the demand for a standardization of radiomics studies [11]. Additionally, Berenguer et al. suspected CT-based radiomics of being fundamentally influenced by noise [24], which Lu et al. were recently able to disprove for individual features [25]. As other improvements, Van Timmeren et al. suggested test-retest strategies to select robust radiomic features [26]. Kalpathy-Cramer et al. recommended training on phantoms to counteract variations due to different segmentation [16].

Additionally, it has already been addressed that the size of the segmented volume influences radiomic features: Different first-order features (energy, total energy, root mean squared) are confounded by volume, because—in generalized terms—the pixels’ gray levels in a region of interest (ROI) are summed, i.e., a ROI with more pixels leads to a higher feature value and vice versa [2]. Additionally, the first-order feature variance is supposed to be influenced by ROI size [27]. Therefore, these features cannot reliably distinguish between different pathologies unless they are derived from identically sized ROIs. For example, in a study investigating radiation-induced lung disease in CT scans, Choi et al. found that only 16 of 27 texture features were robust across different tumor sizes [28]. Roy et al. found 16 radiomic features dependent on tumor size in breast cancer lesions and suggested normalization for volume dependency to be used for the confounded features [29]. Traverso et al. investigated volume-confounding in 841 radiomic features derived from lung and head and neck tumors and found nearly 30% strongly correlated with tumor volume [30]. Thus, the question arose of which features remain stable when the ROI size varies.

Therefore, this study aimed to identify stable radiomic features in CT and MR images when extracted from ROIs of variable size considering a homogenous phantom. In this way, we intended to observe solely the effects of the different ROI sizes on the features, as the phantom’s structure remains identical throughout.

## 2. Materials and Methods

### 2.1. Phantom and Image Acquisition

We have considered a phantom with no internal structure, so any differences in the results of the feature extraction would be attributable to the varying ROI size. Conversely, no differences for ROIs of varying size would be expected for a stable feature because the texture does not change throughout the phantom. To also test if the behavior is modality-specific, image acquisition should be performed on CT and MR scanners.

We, therefore, used a plastic cup containing 100 mL of sodium chloride solution as a phantom with the desired homogenous inner structure to acquire images for the analysis. All scans were performed with the same phantom on CT and MRI machines from clinical routine and repeated ten times to address potential outliers.

MR scans were performed on a 3 Tesla MRI scanner (Magnetom Skyra, Siemens Healthineers, Erlangen, Germany). The phantom was placed in the isocenter with a head coil carefully positioned on top, removed after each scan, and repositioned for the next acquisition. We selected two MRI sequences with a resolution that is considered suitable for MRI radiomics according to Mayerhoefer et al. [31]. The T2-weighted turbo inversion recovery magnitude (T2w TIRM) sequence was selected as it was assumed to be the most robust MRI sequence for radiomic analysis [12]. The T1-weighted (T1w) sequence was acquired as a counterpart to the T2w TIRM sequence. Sequence parameters are listed in Table 1.

All CT acquisitions were performed on a 320-detector row CT scanner (Aquillion ONE, Canon Medical Systems, Neuss, Germany) using the small field of view. The phantom was placed in the isocenter, removed after each scan, and repositioned for the next acquisition. Scan parameters are listed in Table 2.

### 2.2. Image Analysis

**Figure 1:**
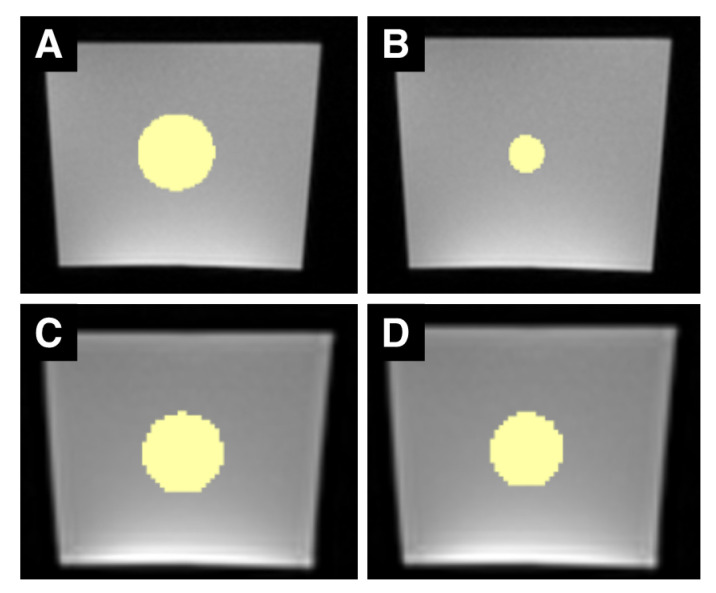
Sample set of MRI ROIs. (**A**–**D**) are examples of MR images of the phantom with differently sized ROIs (in yellow). (**A**,**B**) are images acquired with the T1w sequence, (**C**,**D**) are images acquired with the T2w TIRM sequence. The left column shows ROIs with a 16 mm diameter, the right column ROIs with a 16-pixel diameter. Due to the different spatial resolutions, the 16-pixel ROI in the T2w TIRM image has a greater diameter than the 16-pixel ROI in the T1w image. Both T1w and T2w TIRM-weighted images show typical, gradient-like inhomogeneities within the phantom (brighter at the bottom, darker at the top).

**Figure 2:**
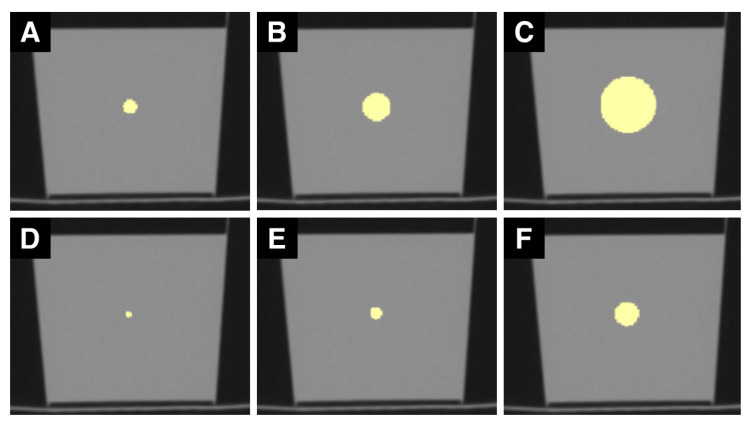
Sample set of CT ROIs. (**A**–**F**) are CT images of the phantom. (**A**) shows a ROI with a 4 mm diameter, (**B**) with 8 mm, and (**C**) with 16 mm. (**D**) illustrates one slice of the 4-pixel diameter ROI, (**E**) of the 8-pixel, and (**F**) of the 16-pixel diameter ROI. mm sized ROIs are generally larger than px sized ROIs.

ROIs were drawn as spheres using 3D Slicer (3D Slicer, Version 4.10.2, http://www.slicer.org (accessed on 8 August 2020)) into the center of the images of all ten scans. ROI diameters were set to 4, 8, and 16 pixels (px; ROI_px_) as well as to 4, 8, and 16 mm (mm, ROI_mm_). A millimeter-wise analysis was done because metric units are the standard of measure used for reporting. However, the number of pixels in the same mm sized ROI varies with the resolution of the radiologic image (lower resolution: fewer pixels, higher resolution: more pixels). On the opposite, the number of pixels in a px sized ROI is independent of the image resolution. We therefore also conducted a pixel-wise analysis. Figure 1 and Figure 2 show examples of ROIs placed in MR and CT images.

All ROIs were drawn twice by one reader and once more by another reader to determine intra- and interrater agreement.

All features except for the shape features were extracted using PyRadiomics (Version 3.0) [32] with settings suggested by the developers: for CT: imageType: Original: {} \featureClass: firstorder:, glcm:, glrlm:, glszm:, gldm:, ngtdm: \setting: binWidth: 25, voxelArrayShift: 1000, correctMask: true; for MR: imageType: Original: {}\featureClass: firstorder:, glcm:, glrlm:, glszm:, gldm:, ngtdm: \setting: binWidth: 5, voxelArrayShift: 300, correctMask: true).

Shape features were not considered because all ROIs were spheres with a defined size and geometry. The first-order features included were energy, total energy, entropy, minimum, maximum, mean, median, interquartile range (IQR), range, mean absolute deviation (MAD), robust mean absolute deviation (RMAD), root mean squared (RMS), skewness, kurtosis, variance, uniformity, 10th percentile, and 90th percentile. In addition, the second- and higher-order feature classes were comprised of: 24 gray level co-occurrence matrix (GLCM) features, which describe combinations of gray levels of neighboring pixels [33,34]; 14 gray level dependence matrix (GLDM) features, which quantify gray level dependencies in an image [2]; 16 gray level run-length matrix (GLRLM) features, which quantify gray level runs (defined as the number of pixels that have the same gray level value) [2]; 16 gray level size zone matrix (GLSZM) features, which quantify gray level zones in an image (defined as the number of connected voxels that share the same gray level intensity) [2]; as well as 5 neighboring gray tone difference matrix (NGTDM) features to quantify the difference between a gray value and the average gray value of its neighbors within a certain distance [2].

The classification of features into first-, second-, and higher-order features is based on the system proposed by the developers of PyRadiomics [2].

### 2.3. Statistical Analysis

Statistical analysis was performed using SPSS (SPSS Statistics for Windows, version 26.0, IBM Corp. Armonk, NY, USA) and R (version 3.5.1) [35].

To check whether the variation in ROI size can lead to significant differences of the feature value, the results were evaluated with a pairwise Mann–Whitney U (MWU)-test with Bonferroni correction in R. The three possible pairs were tested for millimeter- and pixel-sized ROIs (4 vs. 8 mm/px, 4 vs. 16 mm/px, and 8 vs. 16 mm/px). A *p*-value < 0.05 was considered statistically significant.

Overall concordance correlation coefficients (OCCCs) for agreement of continuous measures according to Lin et al. [36] and Barnhart et al. [37] were calculated using the epiR package for R [38]. While the OCCC is equivalent to the generalized CCC [37], it can be used to measure agreement between more than two variables of interest. Concordance coefficient values range from 1 to −1, with −1 indicating reverse agreement [15]. OCCCs ≥ 0.90 were defined to indicate excellent reproducibility, consistent with reported studies [12,15].

OCCCs were calculated twice: once to assess agreement among the ROI sizes 4, 8, and 16 px/mm (OCCCs_4–16_) and once for the ROI sizes 8 and 16 px/mm (OCCCs_8,16_). This was done to obtain results without the 4 mm and 4 px ROIs, to check if a threshold value should be considered and to determine whether results can be degraded by a small ROI size.

We created Bland–Altman and correlation plots for the ROI sizes of 8 and 16 millimeters and pixels to illustrate numerical data distribution points.

For assessment of interrater agreement, intraclass correlation coefficient (ICC) estimates and their 95% confidence intervals (CIs) were computed using a mean-rating (k = 2), absolute-agreement, 2-way random-effects model. Intrarater agreement was assessed by calculating ICC estimates and their 95% CIs using a mean-rating (k = 2), absolute-agreement, 2-way mixed-effect model. Intra- and interrater reliability was classified as poor to excellent (ICC: <0.5 poor, 0.5–0.75 moderate, 0.75–0.9 good, >0.9 excellent) [39].

## 3. Results

### 3.1. MWU-Test

#### 3.1.1. T1w MR Images

Of the 18 first-order features, RMAD, entropy, range, uniformity, energy, and total energy showed a significant difference for all pairs of ROI sizes in both ROI_mm_ and ROI_px_. On the contrary, no significant differences were observed for mean, median, RMS, 10th percentile, and skewness. The remaining first-order features were different in at least one compound of ROI sizes (mm or pixel).

Of the 24 GLCM features, ten features were significantly different in each possible combination. Seven out of fourteen GLDM features were different in all possible combinations; the others showed differences for at least one pair. Nine of sixteen GLRLM features were different for all possible pairs, while all of the features showed differences for at least one pair. Of the 16 GLSZM features, nine features were different in all combinations, with only one feature (small area emphasis) showing no differences in any compound. Three of five NGTDM features were different in all varieties, while all of the features showed differences for at least one pair.

In total, in T1w images, out of 93 analyzed features, 44 were different in every pairing and 43 in at least one pairing; only 6 features did not show differences in any combination.

For the total of 558 ROI pairs (4,8 and 4,16 and 8,16 in mm or px: six combinations for 93 features), we recognized 221 significant differences in ROI_mm_ and 185 in ROI_px_.

Results of the MWU-test for T1w images sorted by feature class are shown in Supplementary Materials 1 (see Table S1).

#### 3.1.2. T2w TIRM Images

Of the first-order features, uniformity, RMAD, MAD, IQR, variance, entropy, range, total energy, and energy were significantly different for all pairs of ROI sizes. Mean, median, RMS, 10th percentile, 90th percentile, and skewness showed no differences for any combination. The remaining first-order parameters were significantly different in at least one compound.

A total of 13 GLCM, 9 GLDM-, 11 GLRLM, 9 GLSZM, and 2 NGTDM features were different in all combinations, while all of the features showed differences for at least one pair.

In summary, in the T2w TIRM images, a significant difference occurred in 53 of 93 features in all combinations and 34 features in at least one pair. Only 6 of the features (6 first-order features) showed no significant differences in all possible variations.

There were 221 significant differences in ROI_mm_ and 212 in ROI_px_.

Results of the MWU-test for T2w TIRM images are shown in Supplementary Materials 2 (see Table S2).

#### 3.1.3. CT Images

Compared to the MR images, only a few first-order features were significantly different between ROI sizes: range, energy, and total energy showed significant differences in all possible ROI combinations; 10th percentile, variance, MAD, and minimum showed difference in at least one compound. The features mean, median, RMS, entropy, uniformity, skewness, 90th percentile, RMAD, and IQR did not show differences in any pair.

Of 24 GLCM features, 13 features were different in at least one compound. The remaining 11 GLCM features showed no differences. A total of 6 GLDM- and 5-GLSZM features showed significant differences in all possible combinations and one feature with significant differences for at least one pair. 10 GLRLM- and two NGTDM features were significantly different in all compounds, while all of the features showed differences for at least one pair.

In total, 21 of 93 CT-derived features did not show significant differences in any pair. Twenty-six features were significantly different in all compounds, and 44 features in at least one combination.

We found 128 significant differences for ROI_mm_ and 137 for ROI_px_.

Results of the MWU-test for the CT images are shown in Supplementary Materials 3 (see Table S3).

**Figure 3:**
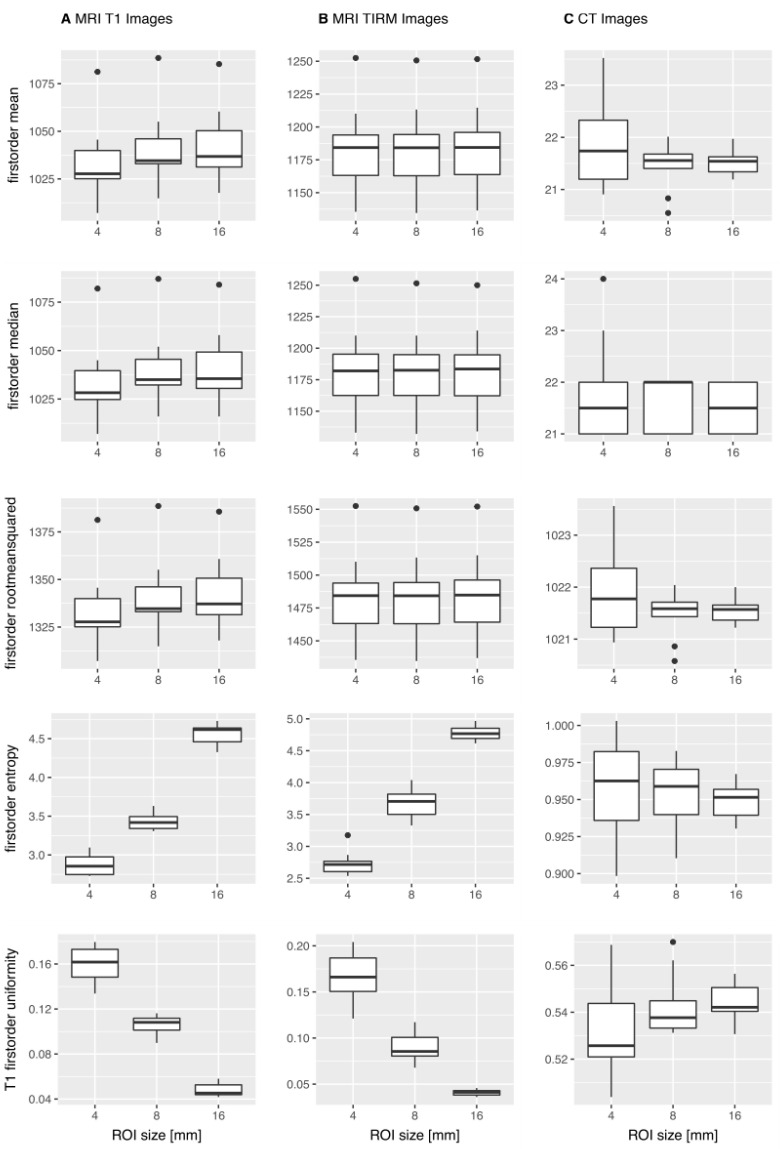
Exemplary boxplots of first-order features mean, median, root mean squared, entropy, and uniformity across the 10 repetitive scans. For the T1w MR images (**A**) and the T2w TIRM MR images (**B**), mean, median, and RMS showed no significant difference in the Mann–Whitney U-test, whereas entropy and uniformity were significantly different. For the CT images (**C**), the illustrated features did not show significantly different results, i.e., the features do not simulate significant differences.

Figure 3 shows exemplary boxplots of the features mean, median, RMS, entropy, and uniformity for T1w, T2w TIRM, and CT images.

### 3.2. OCCCs

OCCCs_4–16_ showed excellent agreement for the features mean, median, and RMS extracted from T1w and T2w TIRM MR images for ROIs_mm_ and ROIs_px_.

The features 90th percentile and 10th percentile showed excellent agreement for T1w ROIs_px_ but not for ROIs_mm_. In T2w TIRM MR images, the 10th percentile showed excellent agreement also only for ROIs_px_.

In the OCCCs_8,16_ agreement was consistent for MR images, besides that the 10th percentile in T2w TIRM no longer showed excellent agreement, either in ROIs_mm_ or in ROIs_px_, despite a high agreement of 0.88 in ROIs_px_.

None of the first-order features derived from CT showed excellent agreement based on OCCCs_4–16_ and OCCCs_8,16_. Median showed the best agreement with 0.8 in ROIs_mm_.

Considering second-order and higher-order features, none of the features, either extracted from CT or from MR images, showed excellent agreement.

**Figure 4:**
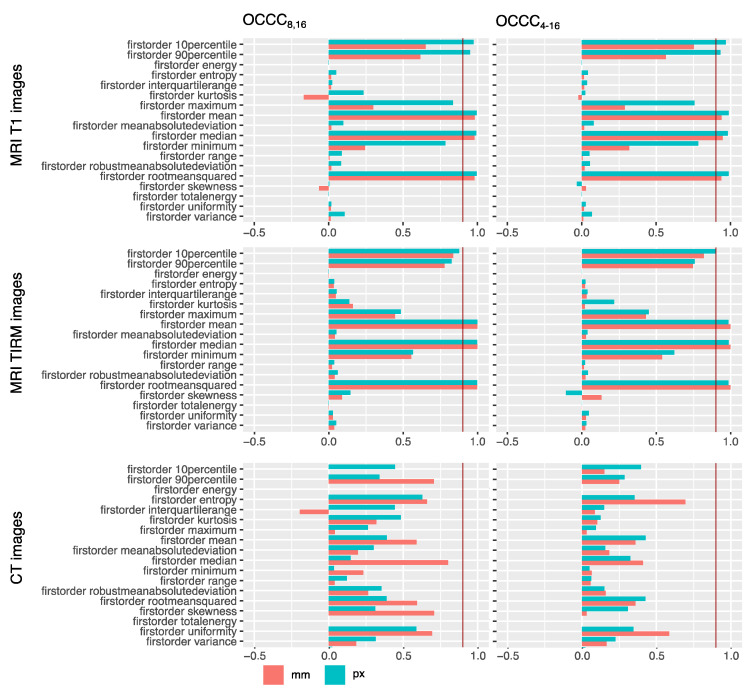
OCCCs_8,16_ and OCCCs_4–16_ for ROIs drawn in millimeters (**red**) and pixels (**green**). Excellent agreement of 0.9 is marked with a red line. Second- and higher-order features as well as numerical values can be found in the Supplementary Materials (Figures S1–S3, Tables S1–S3). Mean, median, and root mean squared concordantly showed excellent agreement across ROI sizes for MR images. None of the features showed excellent agreement for CT images; here, the best agreement of 0.8 is shown by the feature median for the millimeter sized ROI OCCCs_8,16_.

Results for the OCCCs of first-order features are compiled as bar plots in Figure 4. OCCCs of all included features are illustrated in the Supplementary Materials (see Figures S1–S3). Numerical data of OCCCs of all features are also provided in the Supplementary Materials together with results of MWU-test (see Tables S1–S3).

**Figure 5:**
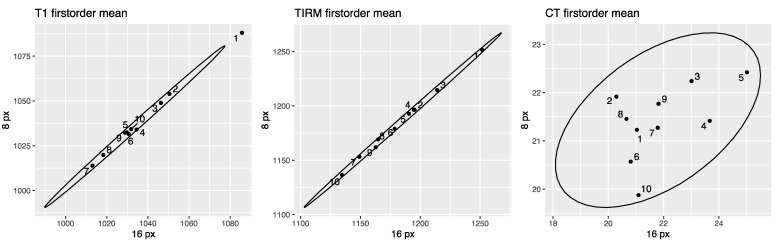
Correlation plots for the first-order feature mean for ROI sizes 8 and 16 pixels in CT and MR images. T1w MR-derived mean on the left, T2w TIRM MR-derived mean in the middle, and CT-derived mean on the right. Data points are defined by the values for the 8-pixel ROI (ordinate) and the 16-pixel ROI (abscissa). The narrower ellipse of the MR data compared to the CT data illustrates that the discrepancy between the ROI sizes is smaller. Correlation plots of all features are provided in the Supplementary Materials (see Figure S4).

**Figure 6:**
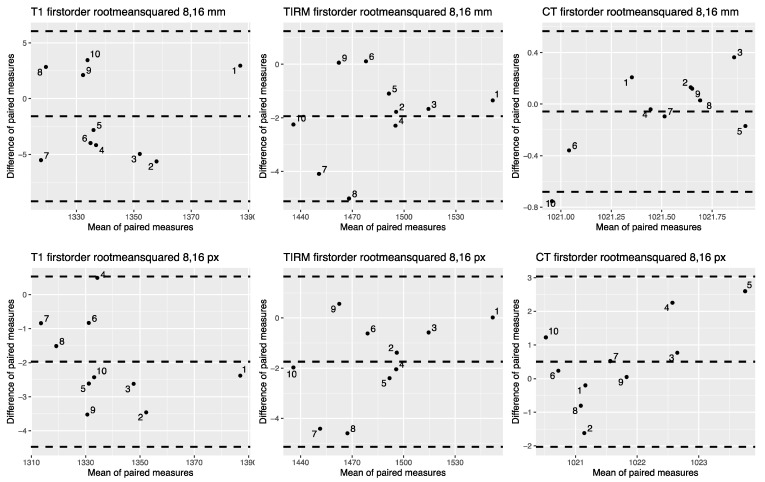
Bland–Altman plots for the first-order feature root mean squared for pixel-sized and mm-sized ROIs from CT and MR images. Bland–Altman plots for T1w MR images on the left, for T2w TIRM images in the middle, and for CT-derived images on the right side. The upper row shows root mean squared extracted from mm sized ROIs, the lower row root mean squared extracted from px sized ROIs. Bland–Altman plots for all features are shown in the Supplementary Materials (see Figure S5).

Figure 5 shows correlation plots for the first-order feature mean for ROI sizes 8 and 16 pixels. 2D correlation plots of all included features for ROI sizes 8 and 16 mm and px are provided in the Supplementary Materials (see Figure S4). Figure 6 shows Bland–Altman plots for the first-order feature RMS. Bland–Altman plots of all included features are provided in the Supplementary Materials (see Figure S5).

### 3.3. Intra- and Interrater Agreement

Intra- and interrater agreement was calculated for first-order features to rule out reader dependency of results.

Except for skewness and kurtosis, both intra- and interrater agreement was excellent, demonstrating that the obtained results are not attributable to the individual reader. Skewness was the only feature for which agreement was moderate. Kurtosis was the only feature for which agreement was poor. This may be attributable to intrinsic properties of these parameters, which are known to be prone to outliers [12].

Results are summarized in Table 3.

### 3.4. Summary of the Results

Compared to the CT-derived features, more MR-derived features were significantly different between ROI sizes in the MWU-test. Most of the few features for MR images without significant differences (mean, median, RMS, 10th percentile, skewness, and in T2w TIRM images additionally 90th percentile) showed excellent OCCCs.

For CT, in total fewer features were significantly different between ROI sizes, especially considering the first-order and the GLCM features. However, none of the CT-derived OCCCs showed excellent agreement.

For the MR images, more features from ROIs drawn in millimeters showed significant differences than from ROIs drawn in pixels. In CT images, slightly more features from ROIs drawn in pixels were significantly different.

## 4. Discussion

Of all features extracted from our homogenous phantom, the first-order parameters mean, median, and RMS proved robust to a ROI size variation of 4–16 mm and pixels in MR images. Thus, a lesion could vary in size between 4 and 16 mm or pixels without altering these three radiomic features. Agreement in absolute numbers, however, was better when only the two largest ROIs were analyzed.

Considering the Mann–Whitney U-test results, it is interesting that differences between the ROI sizes were significant for a substantial number of features. When transferring this to clinical studies, a feature could be classified as helpful in differentiating a disease entity or condition, even though it may only indicate a systematic difference in lesion size. Our observations on the homogenous phantom showed more MR than CT-derived features with a significant difference between ROI sizes.

We intentionally chose a phantom without an internal structure to acquire images that remain identical for all ROI sizes. We decided to analyze three different spherical ROI sizes in our study to mimic three lesions of the same homogenous composition, but with different volumes. Although a 4 mm ROI is relatively small, it is not entirely unusual in clinical routine (e.g., small pulmonary nodules). Still, it is more likely to encounter larger lesions of clinical relevance, corresponding to ROIs with diameters of 8 to 16 pixels or mm. Nevertheless, we can deduct from our results, that the features we consider stable provide congruent information from 8 to 16 mm/px and 4 to 16 mm/px resp.

Our results for RMS—a measure of the magnitude of intensity values [2]—as a robust feature are rather unexpected since the developers of PyRadiomics themselves refer to RMS as a volume-confounded parameter [2]. Yet, the results confirm a lack of reproducibility across different ROI sizes for energy and total energy, congruent to the developers’ statement. Our stable parameters in T1w and T2w TIRM images, mean and median, were already reported as stable in lung CTs by Choi et al. [28]; however, in our study these parameters did not show excellent OCCCs when derived from CT images.

None of the second- or higher-order features extracted from MR images of our homogenous phantom achieved excellent agreement in the OCCCs. These parameters identified as volume confounded in our study were also reported unstable in the in vivo MRI study by Roy et al., who investigated stability across different tumor volumes on breast cancer patients with T1w and T2w MR sequences [29]. Therefore, these features do not seem reliable for use in MRI-based texture analysis from differently sized ROIs, and studies based on MR-derived second- and higher-order features should be scrutinized.

Unlike Baessler et al. [12], who reported TIRM (FLAIR) images to be most robust in reproducing radiomic features in fruits, we observed no crucial differences between T2w TIRM and T1w images with T1w even yielding slightly better results in our homogenous phantom.

Moreover, we found the reproducibility of MRI-derived 90th and 10th percentiles dependent on whether we measured ROI size in pixels or millimeters, showing excellent agreement only for ROIs_px_. In contrast, mean, median, and RMS were robust to ROI size irrespective of whether we used pixels or millimeters. By comparison, there are more pixels included in ROIs_mm_ than in the respective ROIs_px_. This fact may increase the number of outliers in the ROIs_mm_ by which the percentiles shift slightly, which may be enough to reduce stability. Percentiles are known to be strongly influenced by single-pixel outliers [12]; however, this also applies to mean, which proved to be less susceptible in our study.

Our results for CT-derived features are not surprising since several studies have approved that many CT texture features lack reproducibility, even under constant examination conditions [24,40,41]. In our homogenous phantom, none of the CT-derived features had an excellent OCCC_8,16_ or OCCC_4–16_. In contrast, we must also highlight that fewer CT than MR-derived features showed significant differences. Therefore, CT-derived radiomics seem to be volume confounded in our setting, but not distorted enough to simulate significant differences.

One reason for the high number of features prone to ROI size variation could be that most of the radiomic features were initially developed for non-medical applications and planar images, while typically three-dimensional lesions are investigated in radiological imaging [17].

Our study has some limitations. One is that only one scanner per modality was used to acquire the images used for the analysis. Thus, as already outlined in the introduction, results may be different for other reconstruction algorithms, manufacturers, and settings, especially for MRI [12]. Taking these issues into account was beyond the scope of this study. Nevertheless, we have aimed for reproducible settings with examination parameters taken from the clinical routine. Furthermore, the smallest ROIs in this study (especially ROI_px_) comprise a relatively low number of pixels, which may render the results prone to outliers. We tried to compensate for that by considering multiple acquisitions (10 acquisitions per CT/MR sequence) and comparing values under the exclusion of the smallest ROI by applying the OCCCs_8,16_. In this context, the consideration of only two readers for the estimation of the interobserver variability should also be mentioned. More readers would lead to an even more reliable assessment.

Apart from that, the comparability of T1w and T2w TIRM MR images is limited because the slightly different slice thicknesses lead to different voxel depths and hence differences in spatial resolution in this direction. Additionally, we used different PyRadiomics settings for the extraction from CT and MR images. However, the use of identical parameters without consideration of modality-specific characteristics would again have been associated with limitations.

It may also be seen as a drawback that intensity inhomogeneities in the MR images of our phantom are already visible to the naked eye and may influence radiomic features. However, we believe that similar effects are likely to be encountered in clinical images as well. And although they may not be obvious, there are probably minor inhomogeneities in CT images as well due to repositioning and rotating the phantom, since the wall of the cup is unlikely to be absolutely uniform.

Moreover, it can be considered a limitation that our phantom has no internal structure and hence may not be applicable for texture analysis. In addition, images from a homogenous phantom likely reflect mainly image noise. However, clinical images are not expected to be entirely free of similar effects and homogenous structures are not generally excluded from texture analysis. Nevertheless, it should be kept in mind that the results obtained from our phantom may not be directly translatable to clinical routine.

Despite the already known myriad of factors influencing radiomic features, our results underline that the ROI size is another factor to be considered in radiomics studies. In our study, more MR than CT-derived features were stable across ROI sizes and less susceptible to whether ROI size was measured in millimeters or pixels. On the contrary, less CT than MR-derived features were significantly different between the ROI sizes.

In many studies, lesions were marked with ROIs, but the lesions and consecutively the ROIs had different sizes. Considering our results, however, it has to be validated if the ROI size is a pivotal influencing factor in radiomics, for example, by sorting lesions by volume and voxel size and comparing heterogeneities of the radiomic features or by normalizing the features by voxel count or volume [17,29,42]. Thus, before applying radiomics in clinical routine, volume as a confounding factor needs to be investigated further.

## 5. Conclusions

In conclusion, when considering a phantom with a homogenous structure, the only features robust to a variation in ROI diameter from 4 to 16 mm and pixels were mean, median, and RMS extracted from MR images. Moreover, many features also showed significant differences between the ROI sizes, but this was more frequent for MR than CT images. Since we considered a small phantom in an experimental setting, further studies to investigate this size effect would be necessary for a generalization. Nevertheless, we believe knowledge about this effect is crucial in interpreting radiomics studies, as features that supposedly discriminate disease entities may only indicate a systematic difference in ROI size.

## Figures and Tables

**Table 1:** Scanning details—MRI.

Parameter	T1w GRE	T2w TIRM
TR/TE (ms)	250/3.43	9000/85
Flip angle (°)	70	150
Slice thickness (mm)	5	4
Matrix	512 × 410	256 × 218
Field of view (mm)	240 × 240	230 × 230

GRE: gradient echo. TIRM: turbo inversion recovery magnitude. TR: repetition time. TE: echo time.

**Table 2:** Scanning details—CT.

Parameter	
Tube voltage (kVp)	120
X-ray tube current (mA)	50
Exposure time (s)	0.5
Single collimation width	0.5
Total collimation width	100
Reconstruction kernel	Body
Slice thickness (mm)	0.5
Pixel spacing (mm)	0.430\0.430
Matrix	512 × 512
Field of view (mm)	220 × 220

kVp: peak kilovoltage. mA: milliampere.

**Table 3:** Intrarater and interrater agreement.

Intrarater			
Parameter	ICC	95% CI	*p*
Energy	1.000	1.000–1.000	<0.001
Total energy	1.000	1.000–1.000	<0.001
Entropy	0.998	0.997–0.998	<0.001
Minimum	1.000	1.000–1.000	<0.001
Maximum	1.000	1.000–1.000	<0.001
Mean	1.000	1.000–1.000	<0.001
Median	1.000	1.000–1.000	<0.001
IQR	0.990	0.989–0.991	<0.001
Range	0.997	0.997–0.997	<0.001
MAD	0.996	0.995–0.996	<0.001
RMAD	0.993	0.992–0.994	<0.001
RMS	1.000	1.000–1.000	<0.001
Skewness	0.726	0.695–0.755	<0.001
Kurtosis	0.482	0.422–0.536	<0.001
Variance	0.993	0.992–0.993	<0.001
Uniformity	0.992	0.991–0.993	<0.001
10th percentile	1.000	1.000–1.000	<0.001
90th percentile	1.000	1.000–1.000	<0.001
**Interrater**			
**Parameter**	**ICC**	**95% CI**	***p***
Energy	1.000	1.000–1.000	<0.001
Total energy	1.000	1.000–1.000	<0.001
Entropy	0.994	0.993–0.995	<0.001
Minimum	1.000	1.000–1.000	<0.001
Maximum	1.000	1.000–1.000	<0.001
Mean	1.000	1.000–1.000	<0.001
Median	1.000	1.000–1.000	<0.001
IQR	0.981	0.987–0.984	<0.001
Range	0.987	0.985–0.989	<0.001
MAD	0.983	0.979–0.985	<0.001
RMAD	0.982	0.979–0.985	<0.001
RMS	1.000	1.000–1.000	<0.001
Skewness	0.525	0.471–0.575	<0.001
Kurtosis	0.319	0.240–0.389	<0.001
Variance	0.962	0.956–0.966	<0.001
Uniformity	0.988	0.986–0.990	<0.001
10th percentile	1.000	1.000–1.000	<0.001

ICC: intraclass correlation coefficient. 95% CI: 95% confidence interval. *p*: significance level. Intra- and interrater agreement shows excellent agreement for all first-order parameters except for skewness and kurtosis, which are known to be prone to outliers.

## Supplementary Materials

The following are available online at https://www.mdpi.com/article/10.3390/tomography7020022/s1, Supplementary Table S1: MWU-test and OCCCs for T1w MR images, Supplementary Table S2: MWU-test and OCCCs for T2w TIRM MR images, Supplementary Table S3: MWU-test and OCCCs for CT images, Supplementary Figure S1: OCCCs for T1w MR Images of all features, Supplementary Figure S2: OCCCs for T2w TIRM MR images of all features, Supplementary Figure S3: OCCCs for CT images of all features, Supplementary Figure S4: Correlation plots for all features, Supplementary Figure S5: Bland–Altman plots for all features.
