# Supplementary material for: Stability of Radiomic Features across Different Region of Interest Sizes—A CT and MR Phantom Study
Source: Tomography. 2021 Jun 8;7(2):238–52. doi: 10.3390/tomography7020022 (PMC8293351; doi:10.3390/tomography7020022)
Supplement: Supplementary file 1 [file tomography-07-00022-s001.zip › figure_S4.pdf]

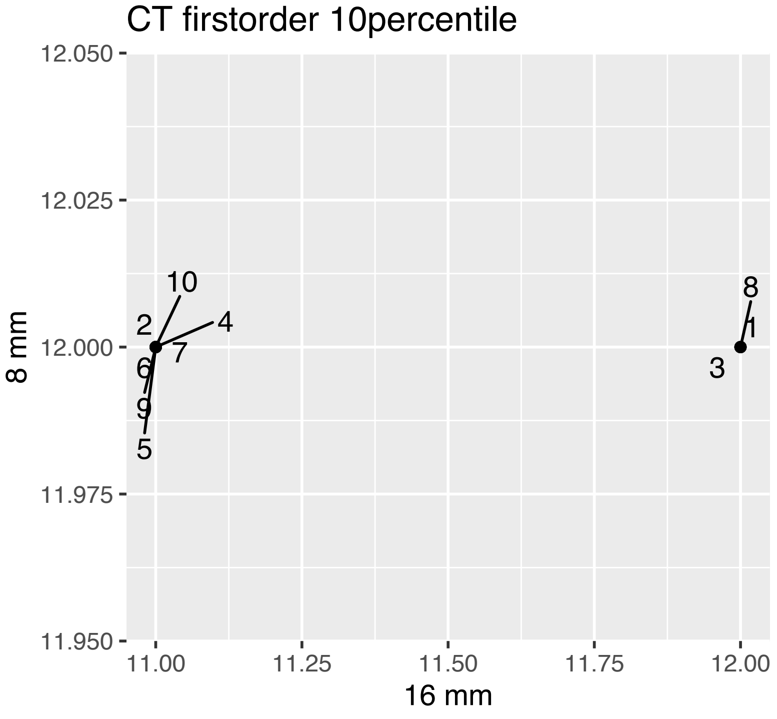
CT firstorder 10percentile
8 mm
16 mm
12.050
12.025
12.000
11.975
11.950
11.00
11.25
11.50
11.75
12.00

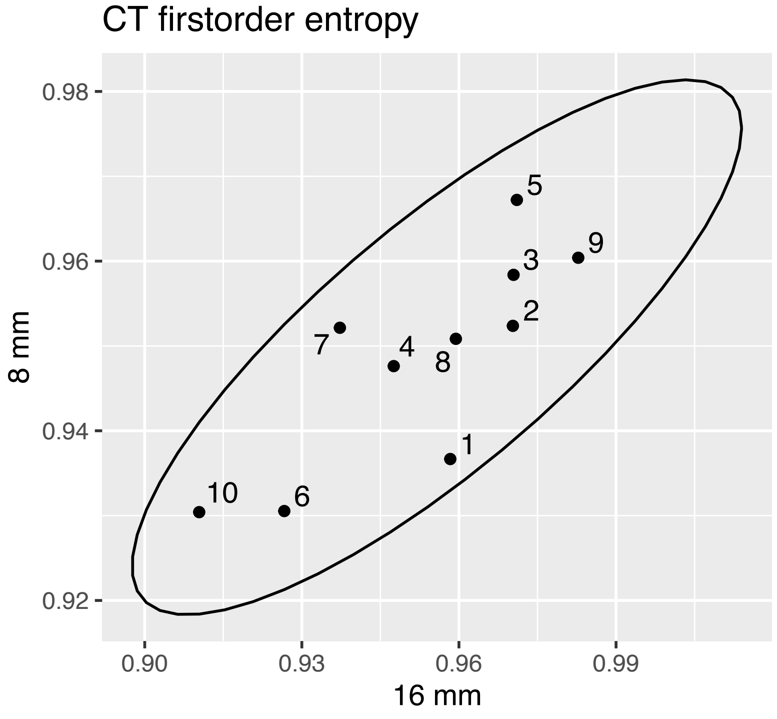
CT firstorder entropy
8 mm
16 mm
0.98
0.96
0.94
0.92
0.90
0.93
0.96
0.99

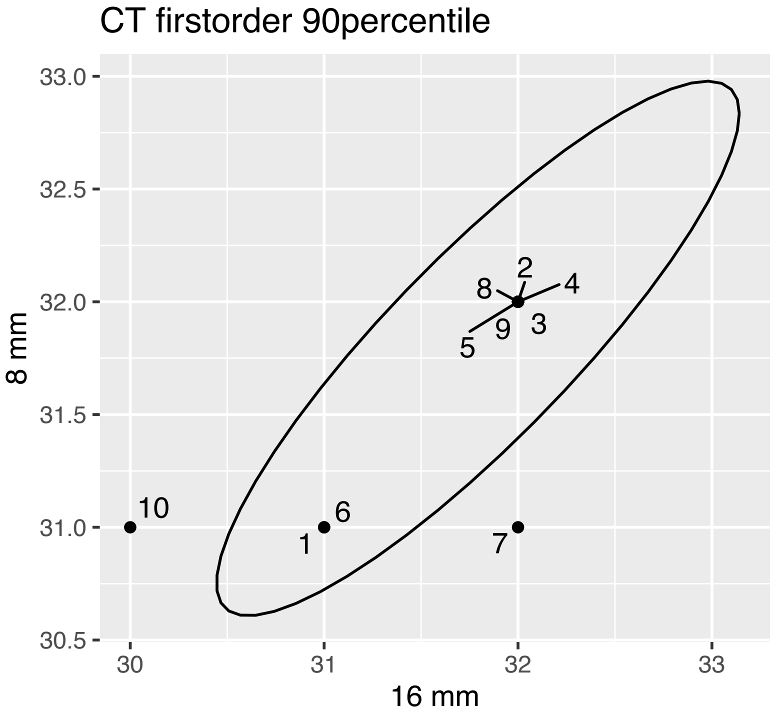
CT firstorder 90percentile
8 mm
16 mm
33.0
32.5
32.0
31.5
31.0
30.5
30
31
32
33

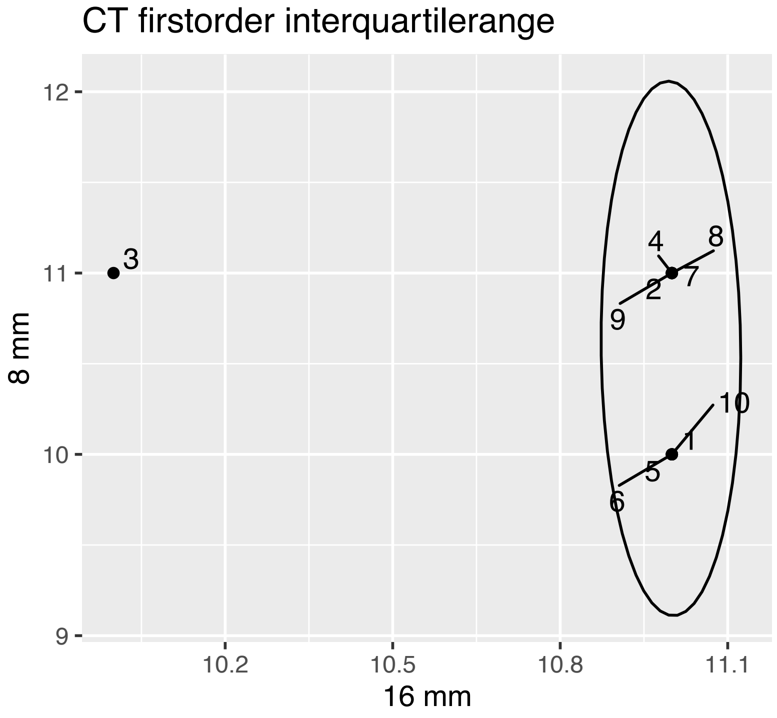
CT firstorder interquartilerange
8 mm
16 mm
12
11
10
9
10.2
10.5
10.8
11.1

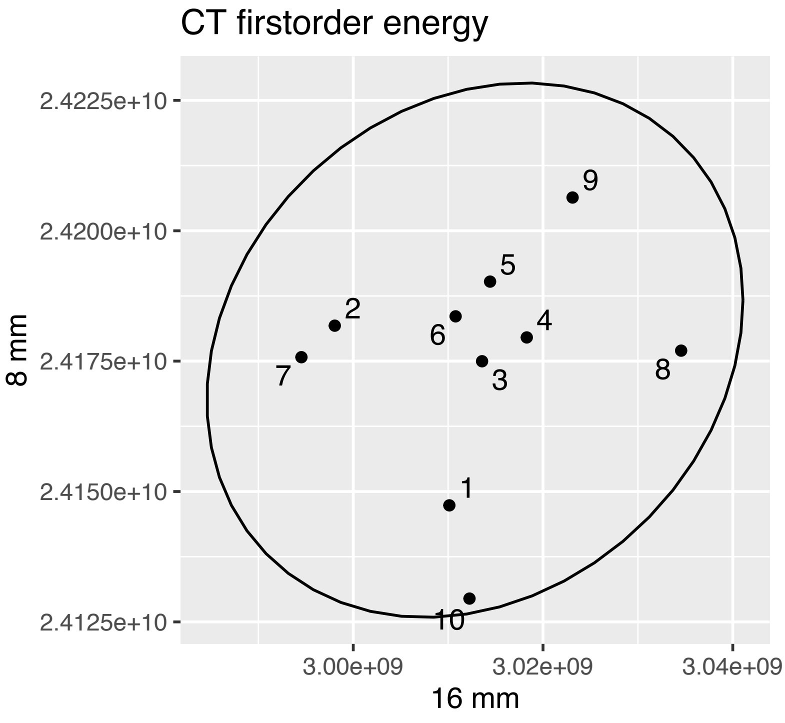
CT firstorder energy
8 mm
16 mm
2.4225e+10
2.4200e+10
2.4175e+10
2.4150e+10
2.4125e+10
3.00e+09
3.02e+09
3.04e+09

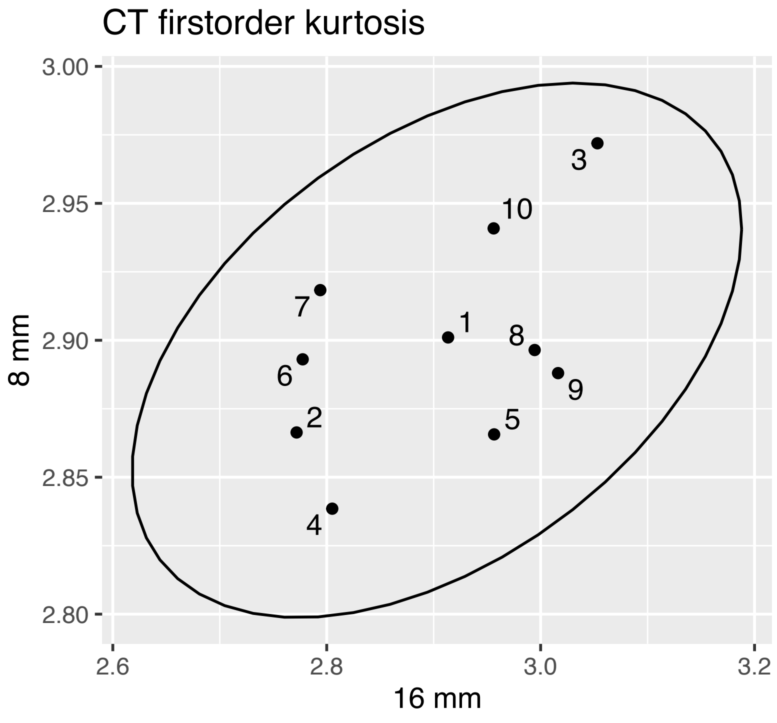
CT firstorder kurtosis
8 mm
16 mm
3.00
2.95
2.90
2.85
2.80
2.6
2.8
3.0
3.2

CT firstorder maximum

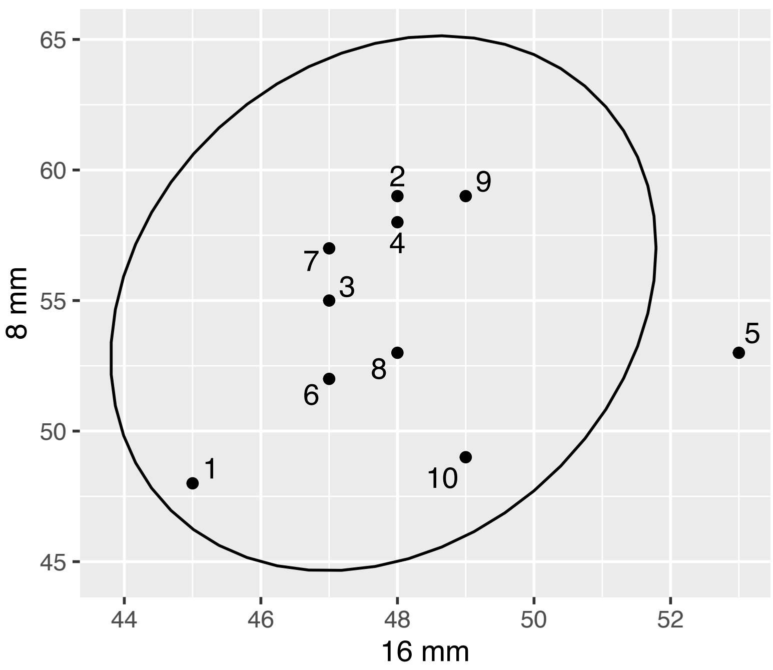

CT firstorder median

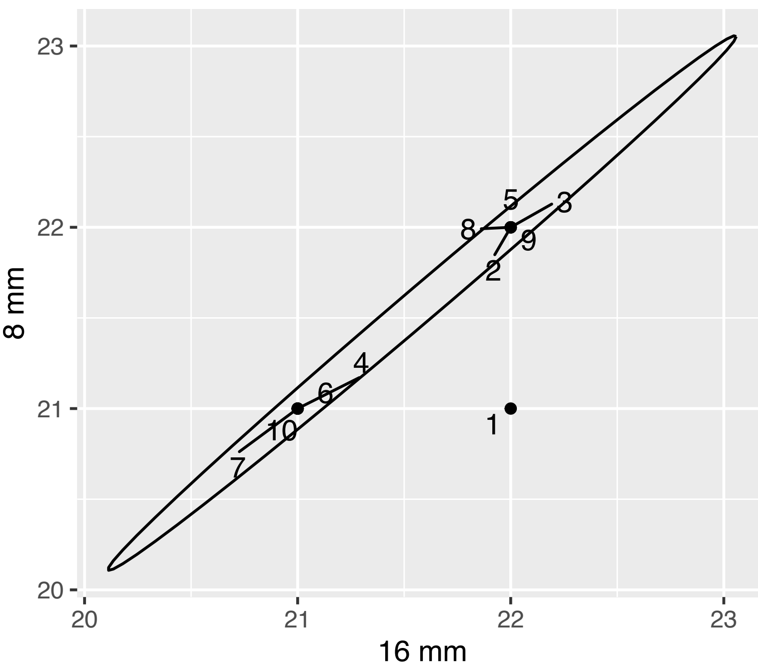

CT firstorder meanabsolutedeviation

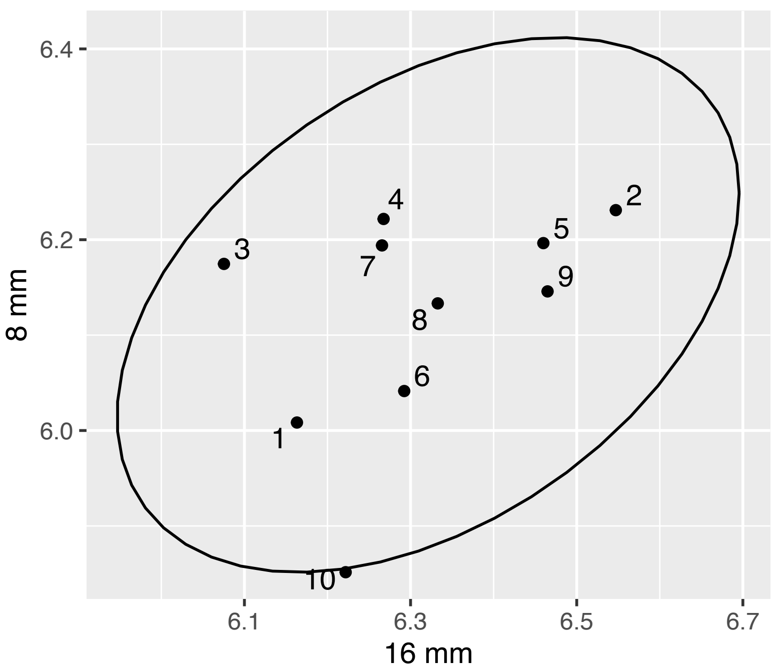

CT firstorder minimum

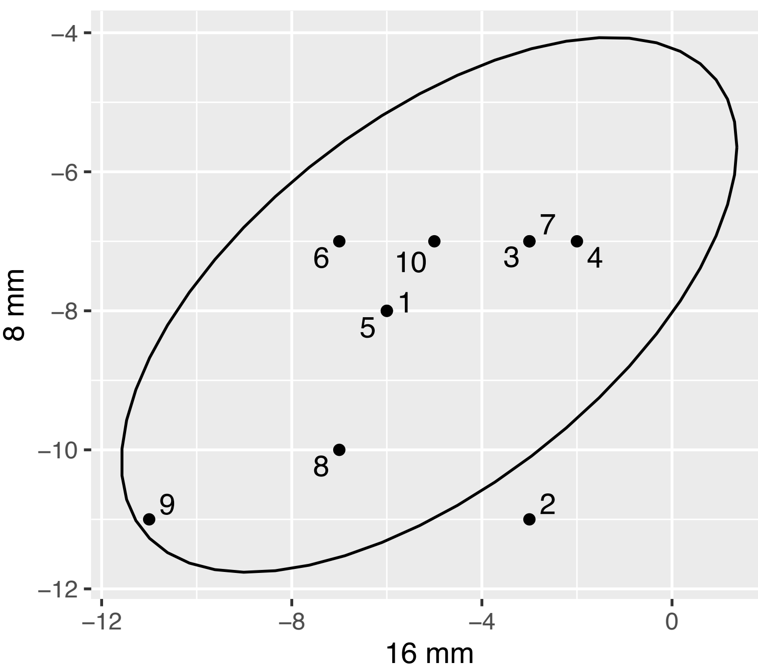

CT firstorder mean

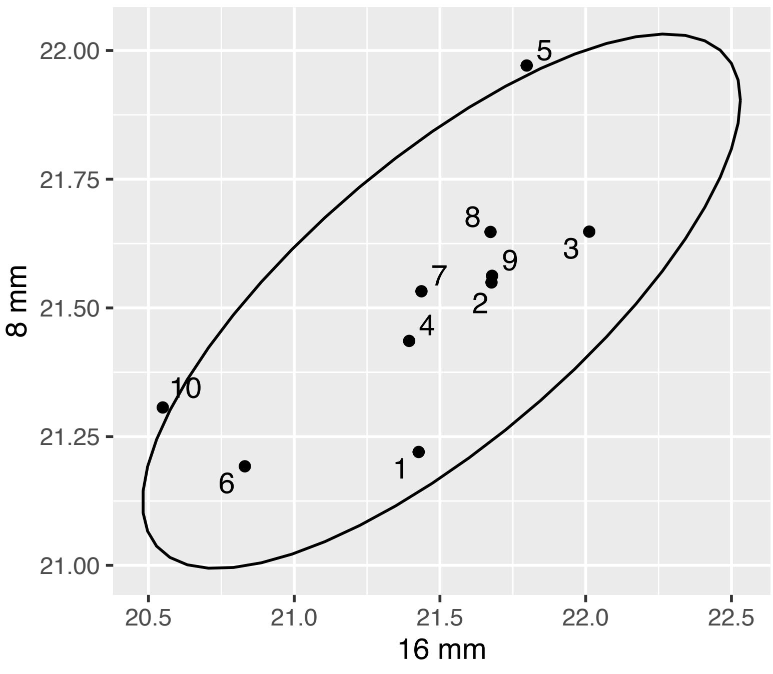

CT firstorder range

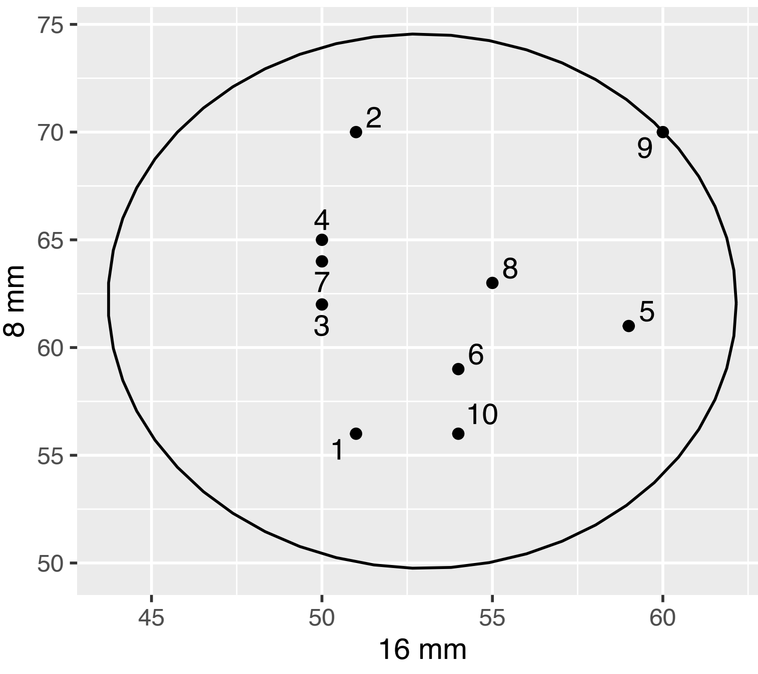

CT firstorder robustmeanabsolutedeviation

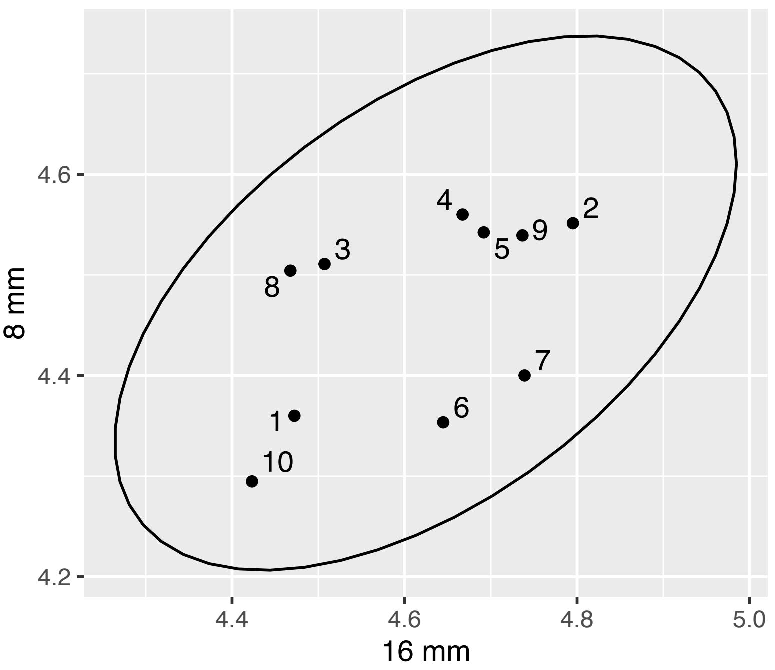

CT firstorder totalenergy

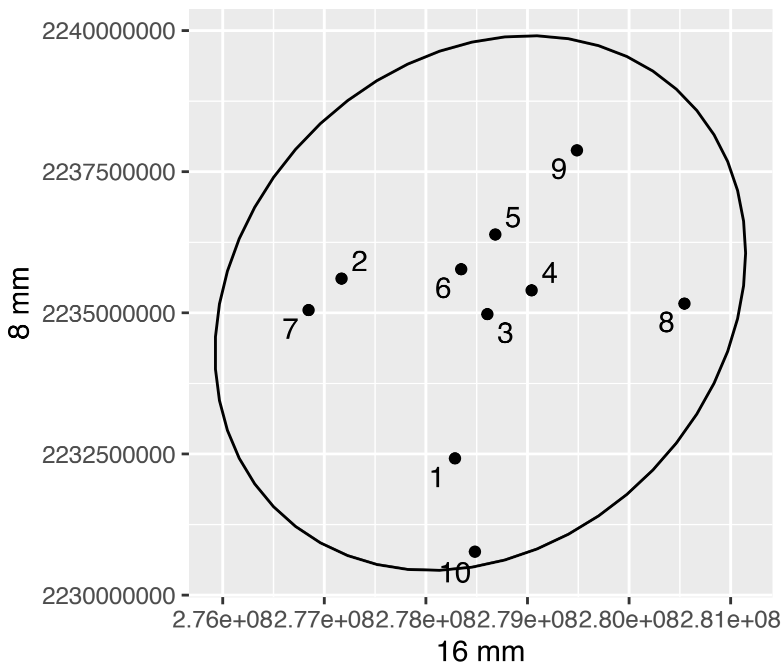

CT firstorder rootmeansquared

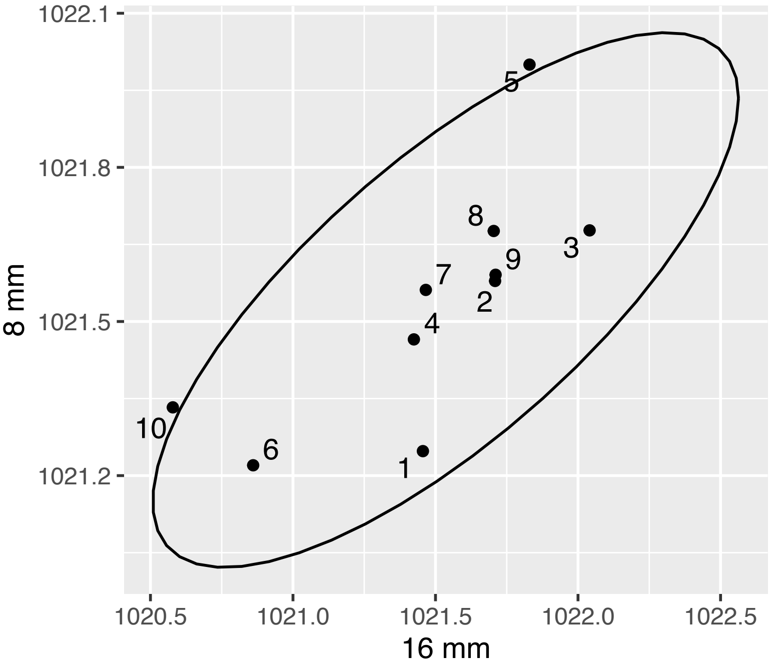

CT firstorder uniformity

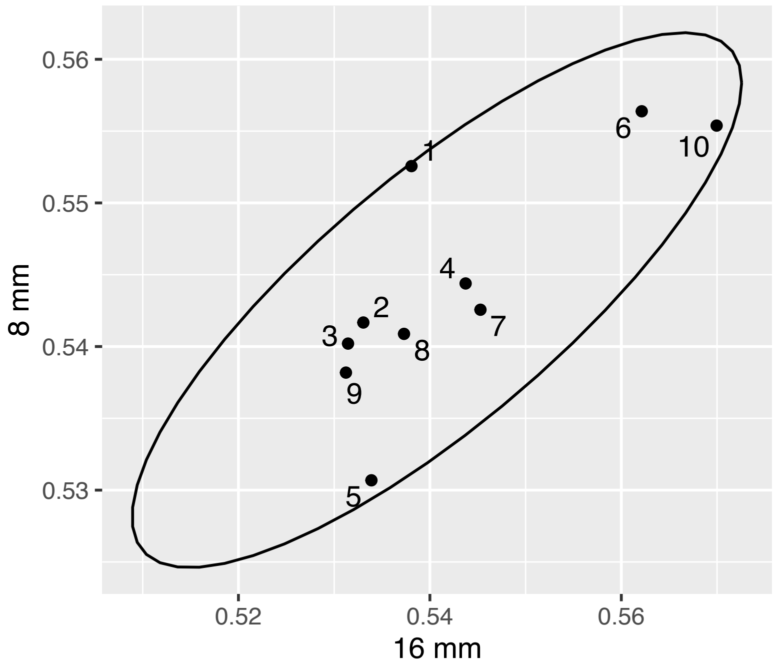

CT firstorder skewness

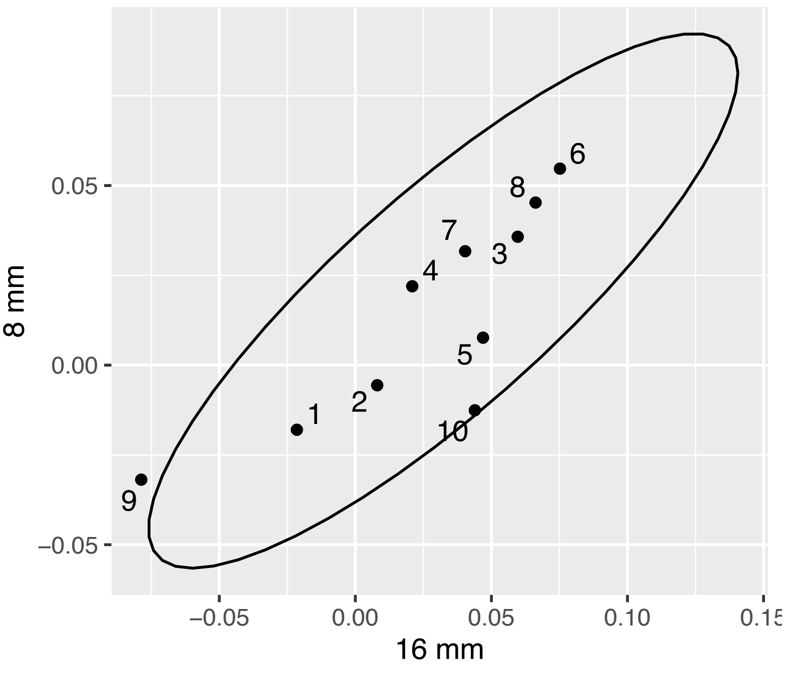

CT firstorder variance

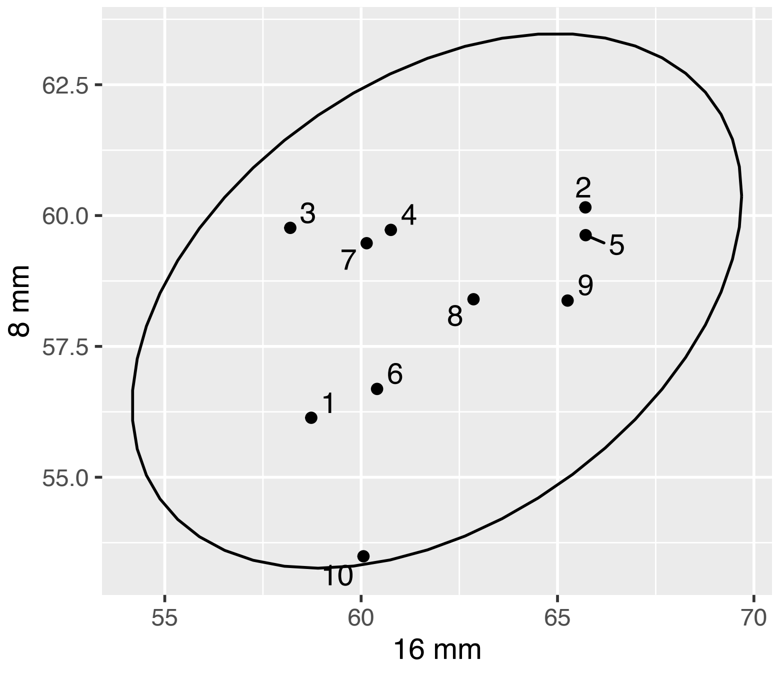

CT glcm autocorrelation

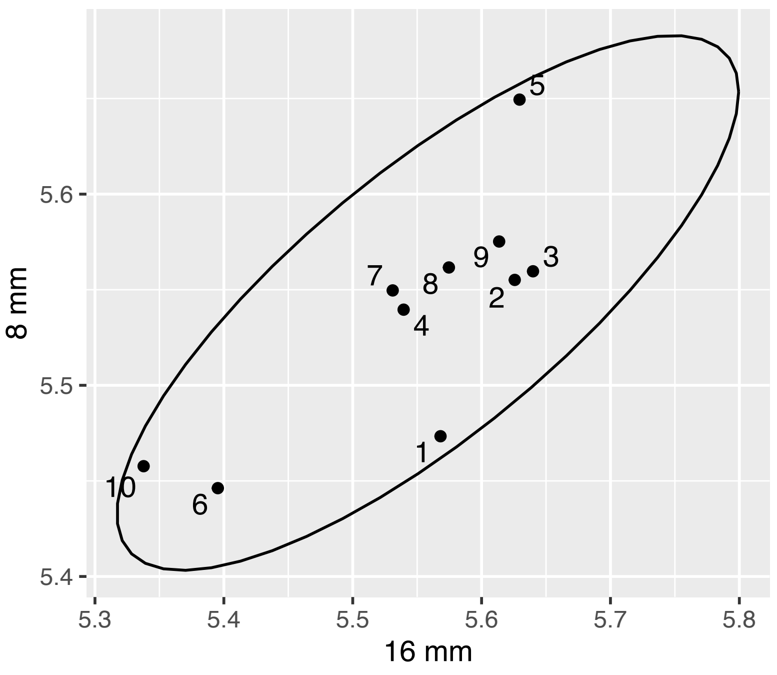

CT glcm clustertendency

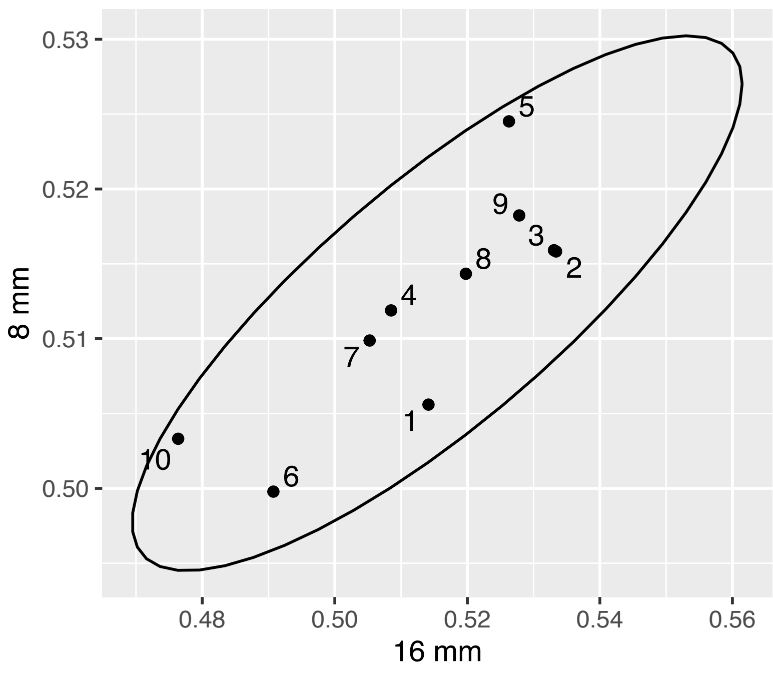

CT glcm clusterprominence

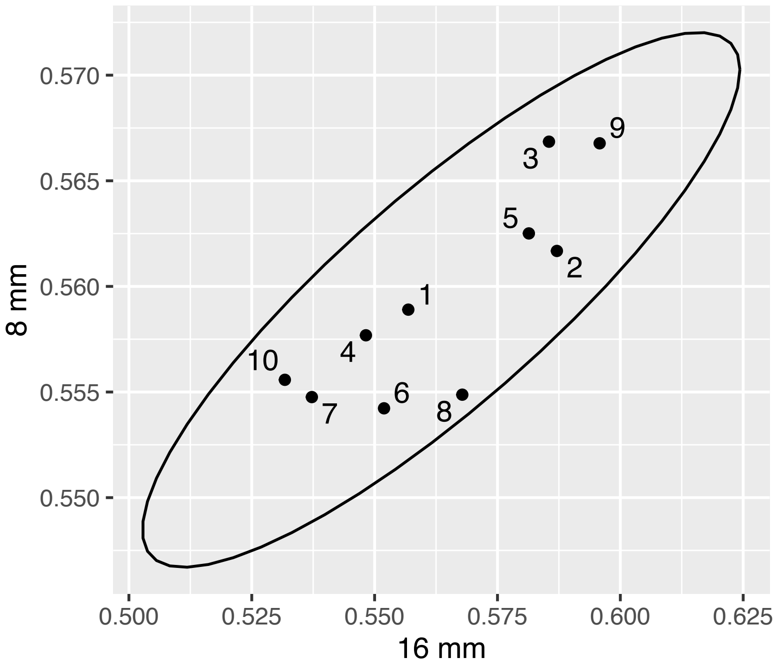

CT glcm contrast

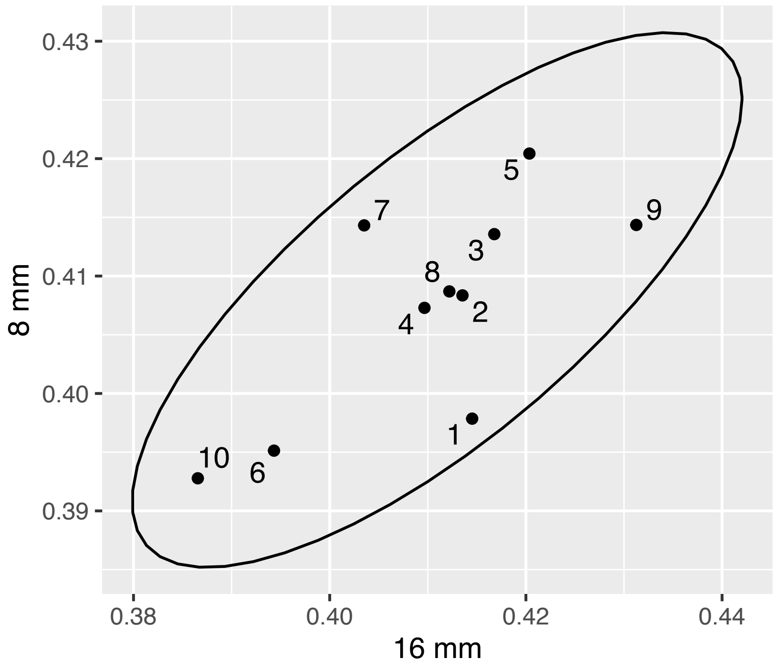

CT glcm clustershade

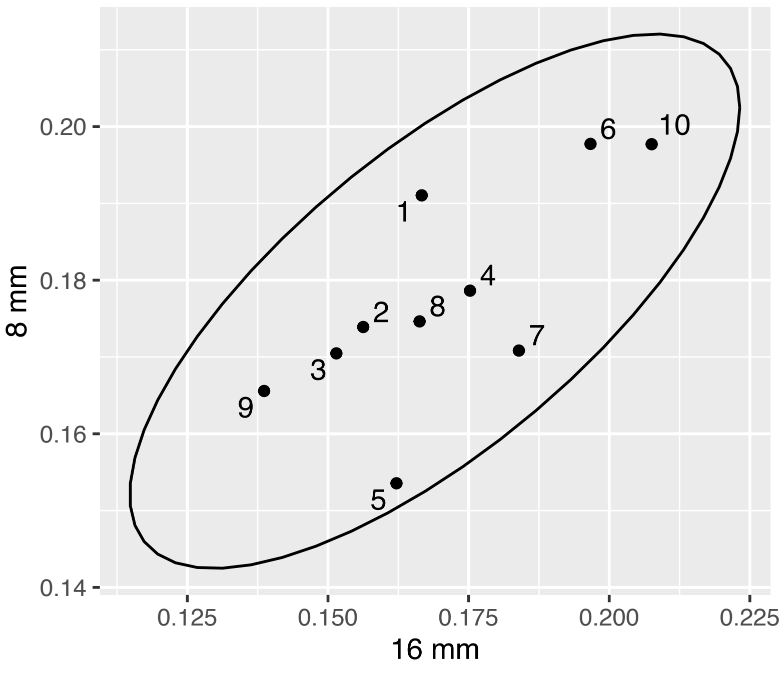

CT glcm correlation

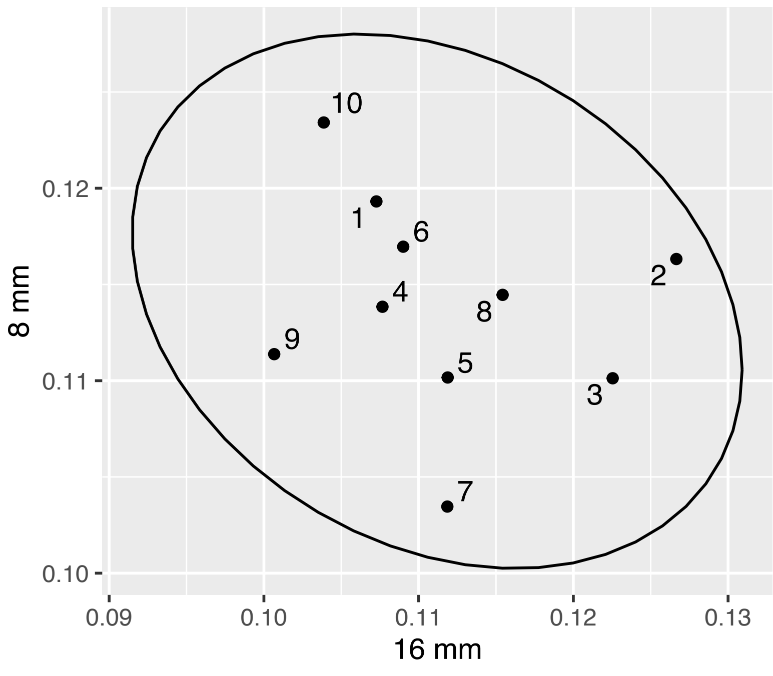

CT glcm differenceaverage

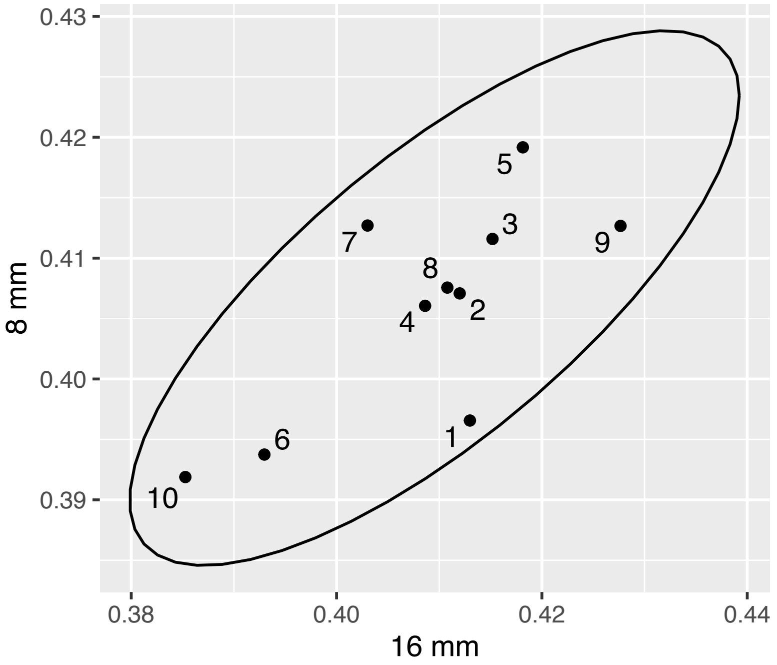

CT glcm id

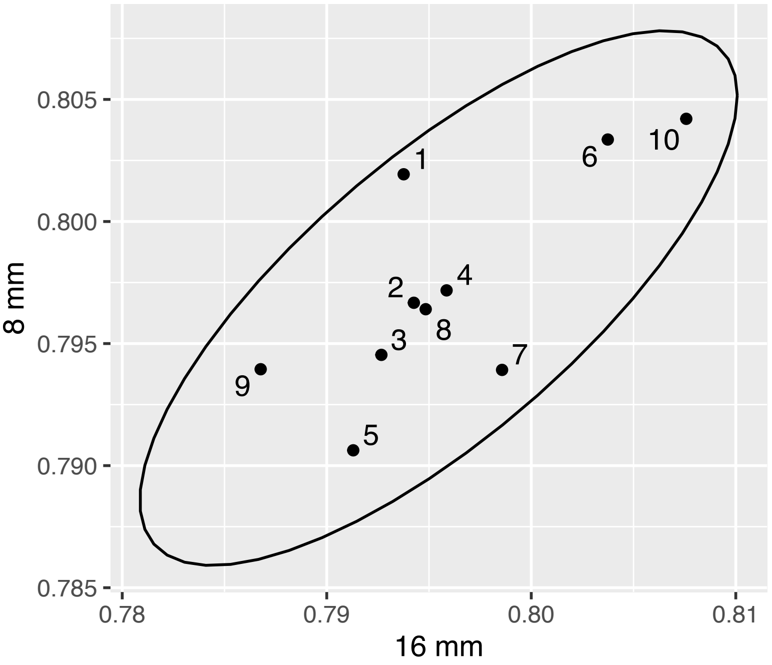

CT glcm differenceentropy

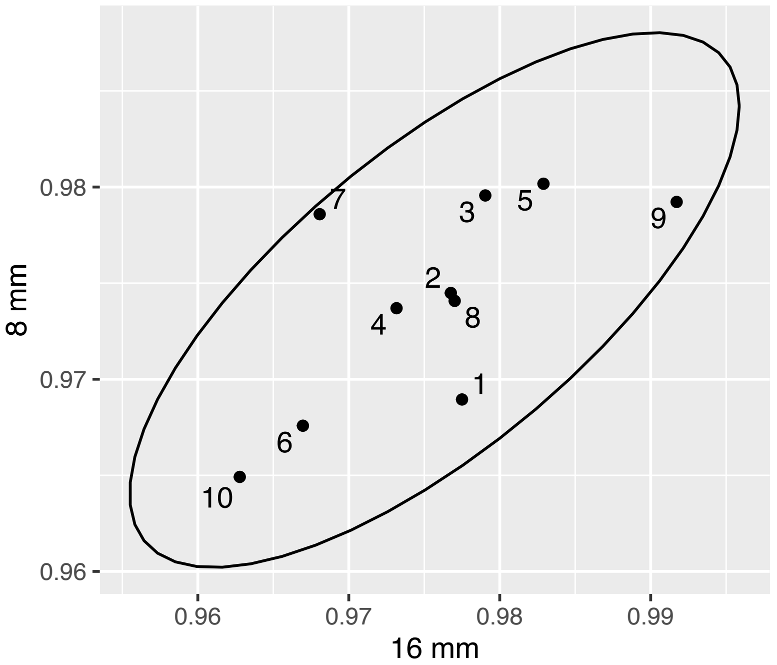

CT glcm idm

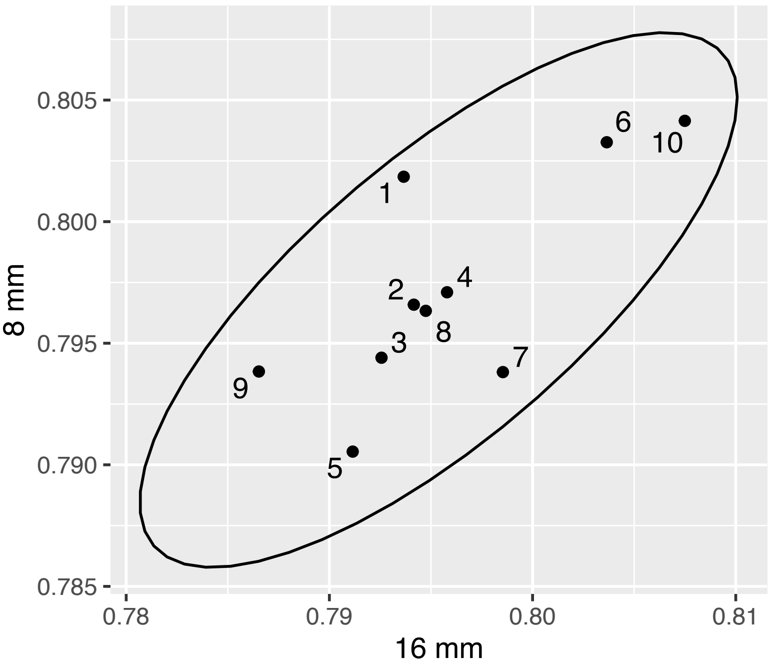

CT glcm differencevariance

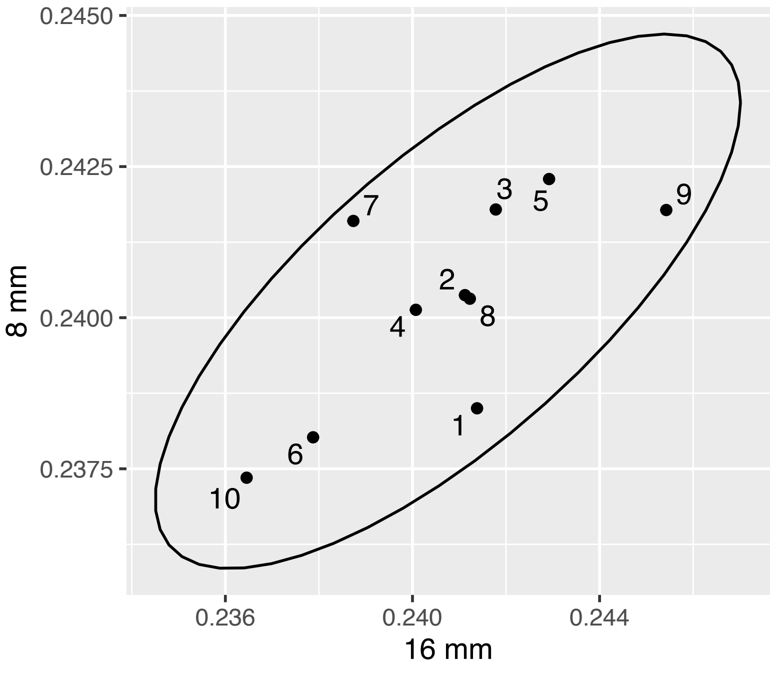

CT glcm idmn

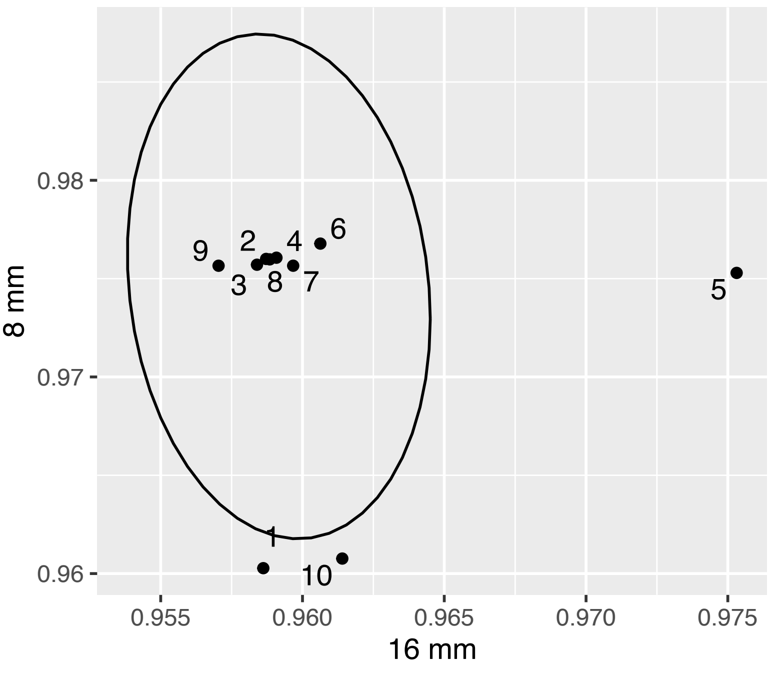

CT glcm idn

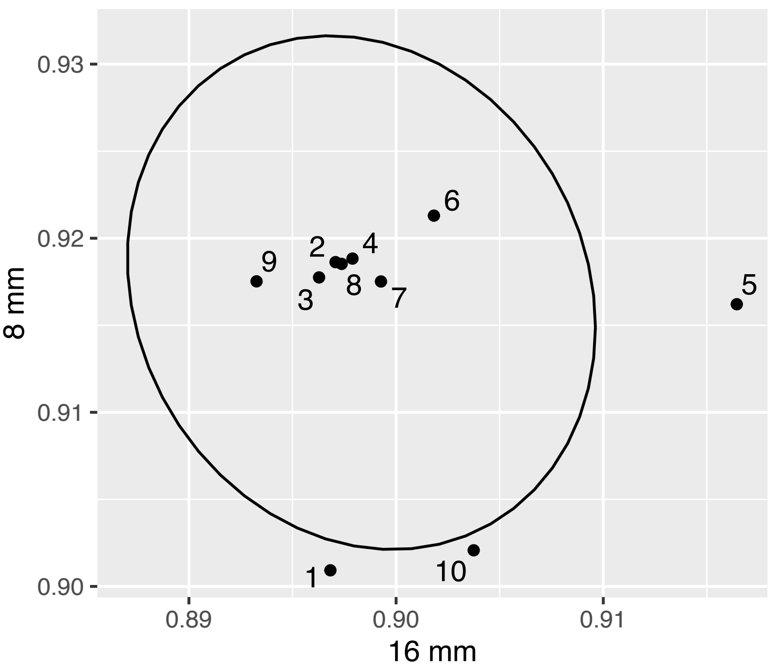

CT glcm inversevariance

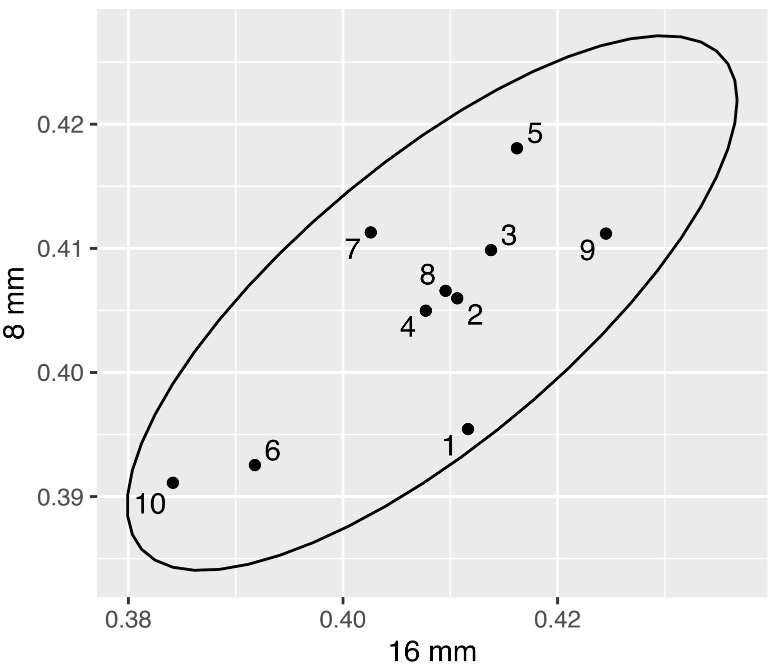

CT glcm imc1

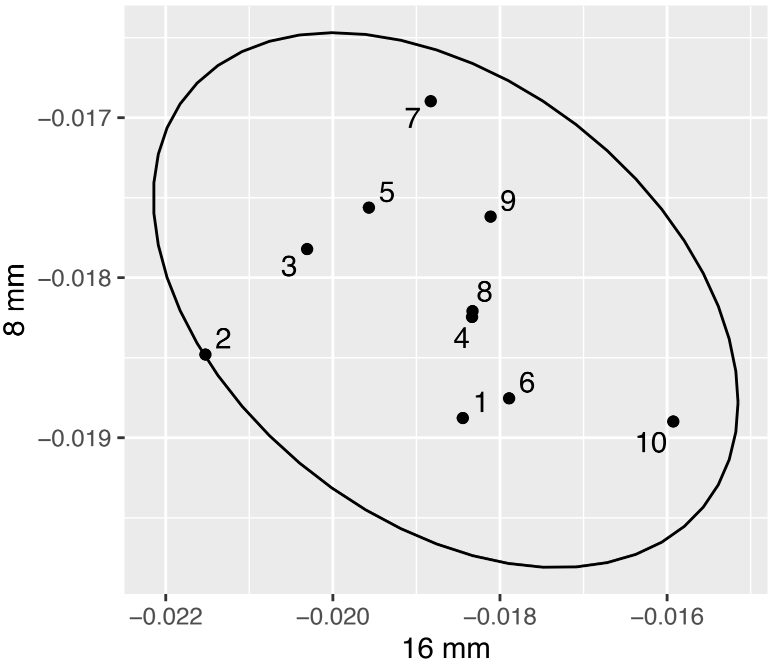

CT glcm jointaverage

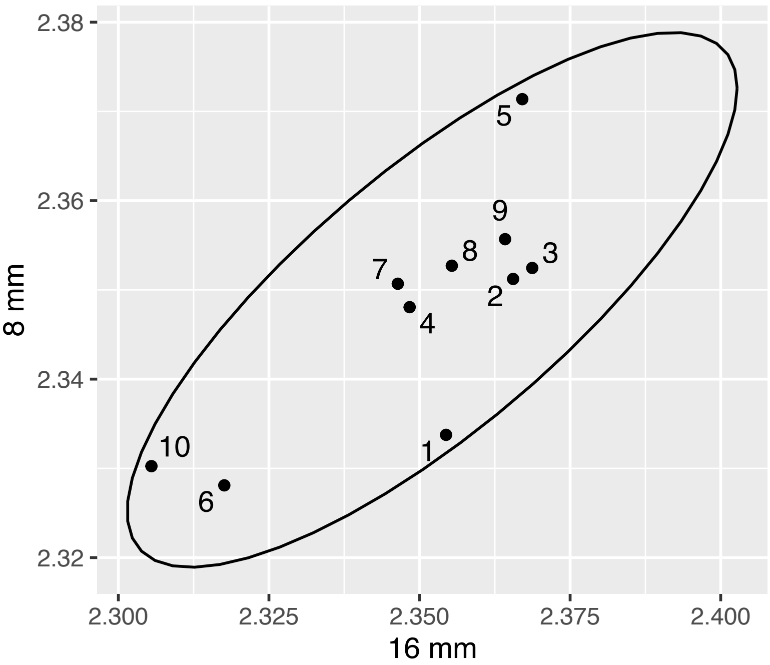

CT glcm imc2

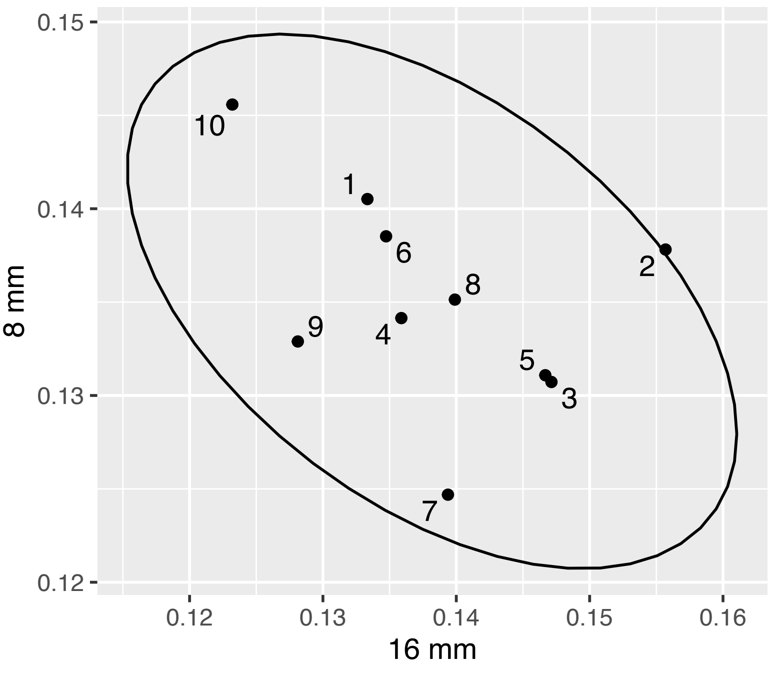

CT glcm jointenergy

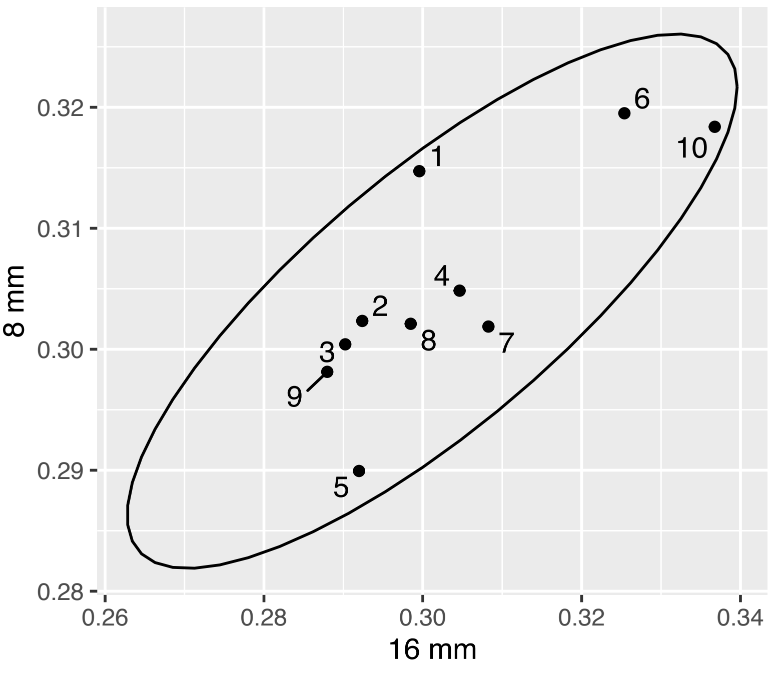

CT glcm jointentropy

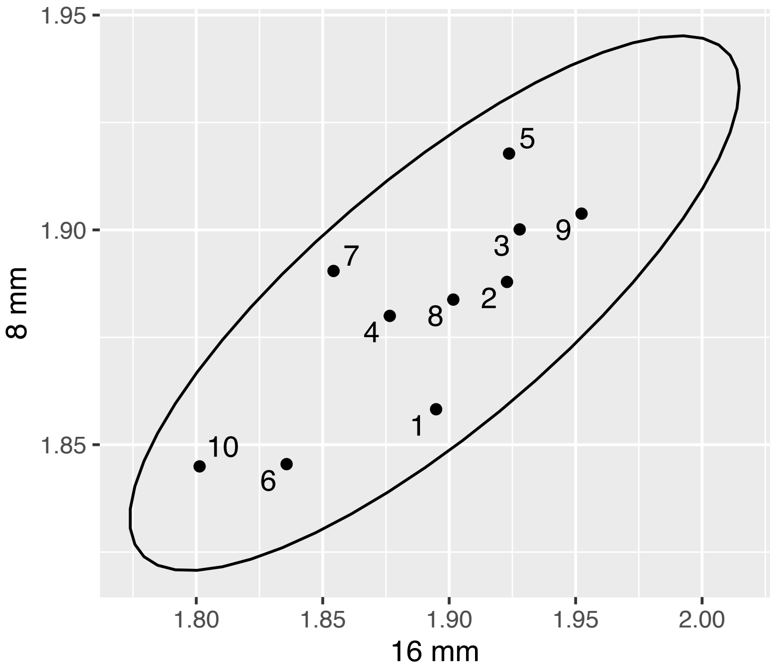

CT glcm sumaverage

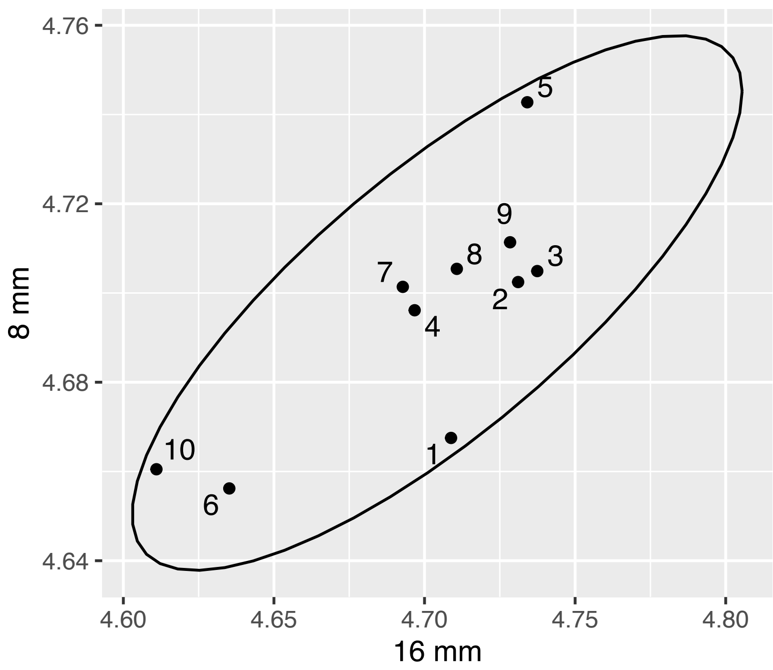

CT glcm mcc

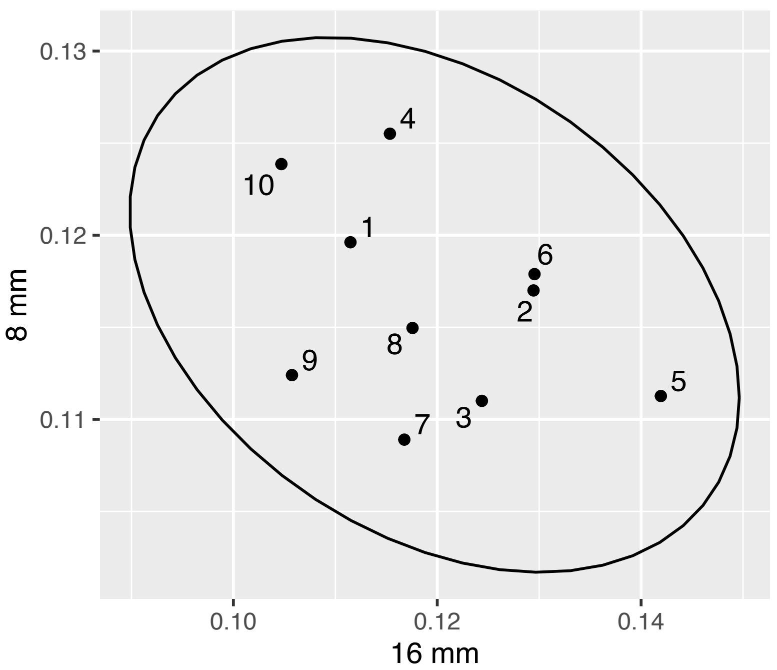

CT glcm sumentropy

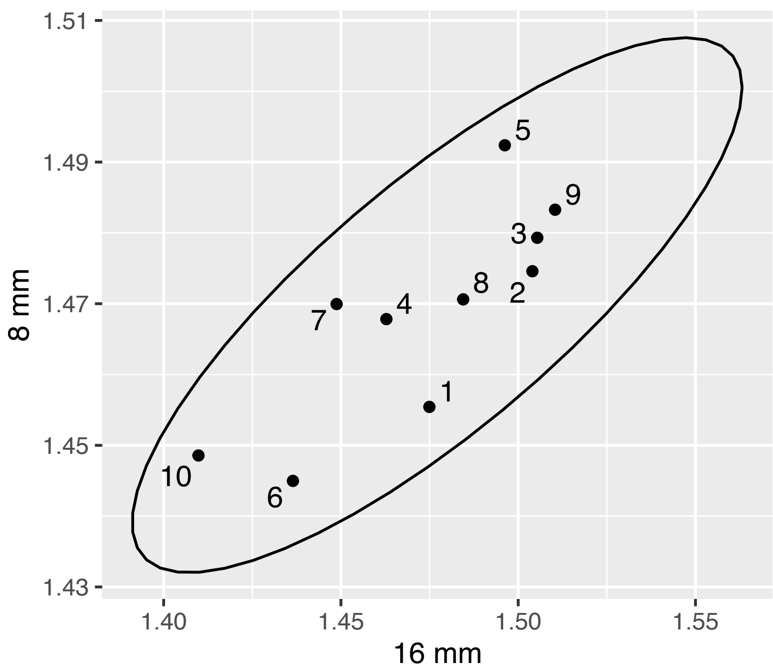

CT glcm maximumprobability

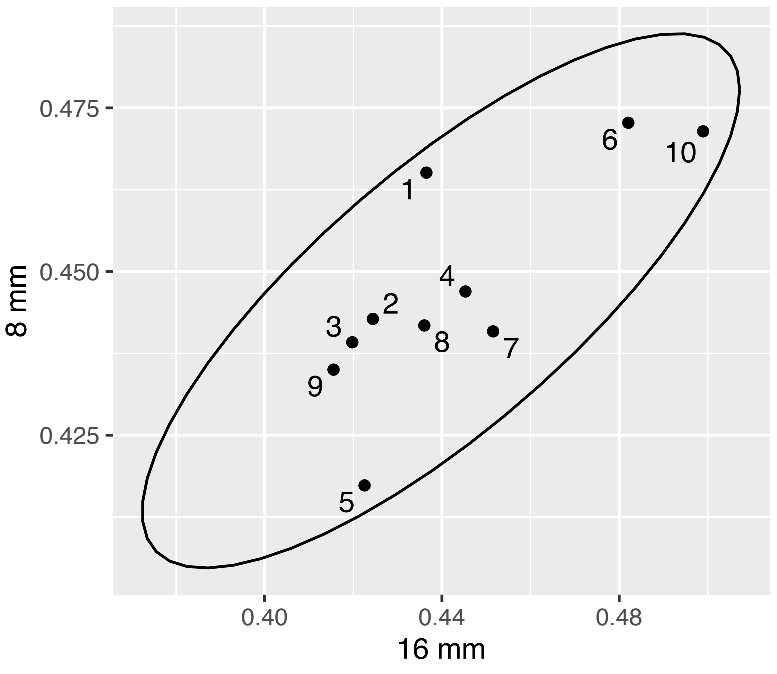

CT glcm sumsquares

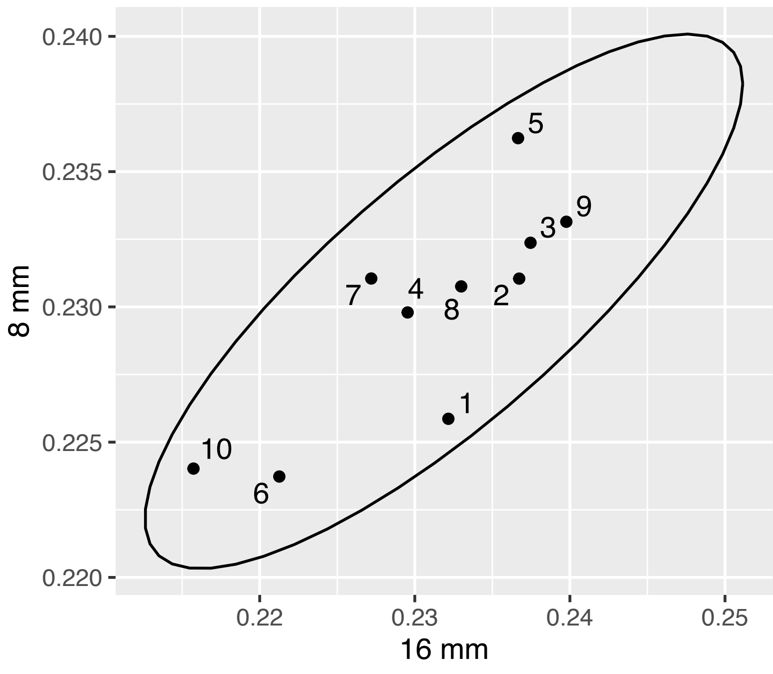

CT glrlm graylevelnonuniformity

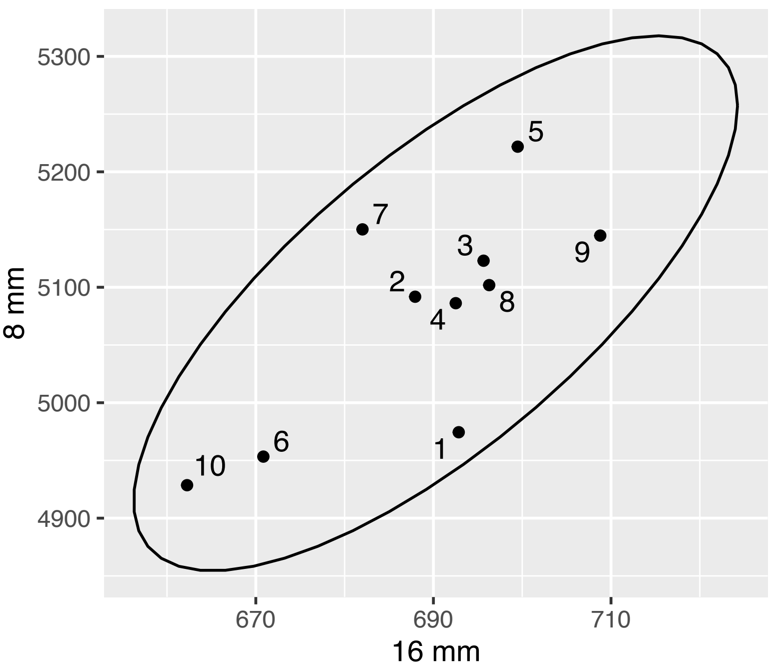

CT glrlm highgraylevelrunemphasis

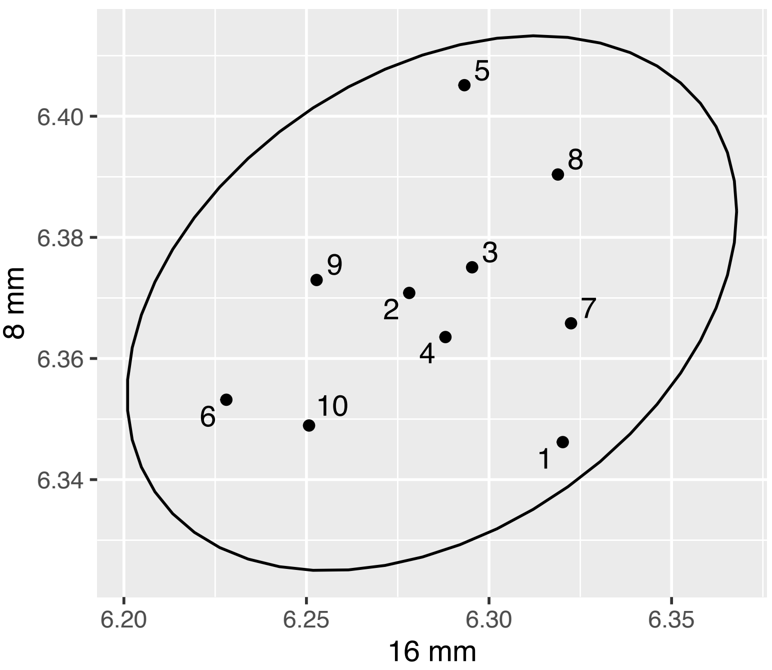

CT glrlm graylevelnonuniformitynormalized

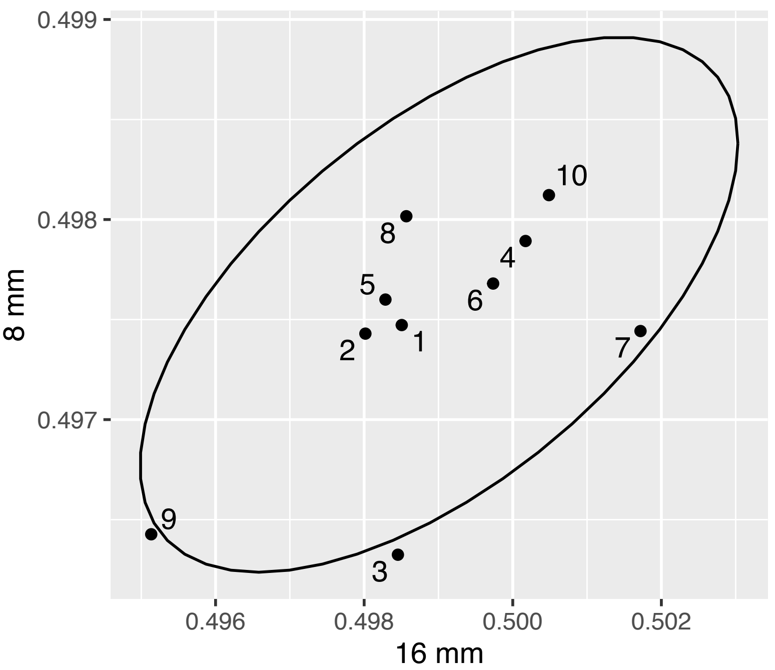

CT glrlm longrunemphasis

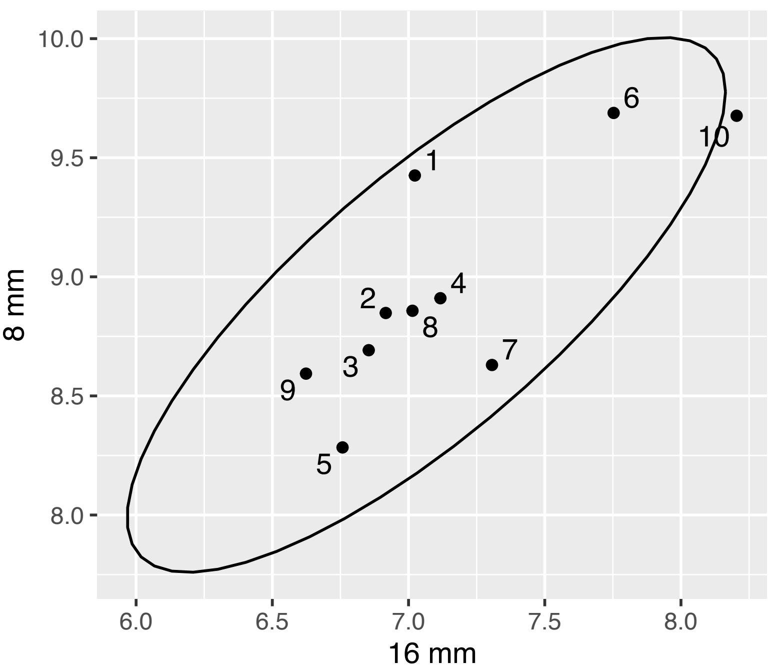

CT glrlm graylevelvariance

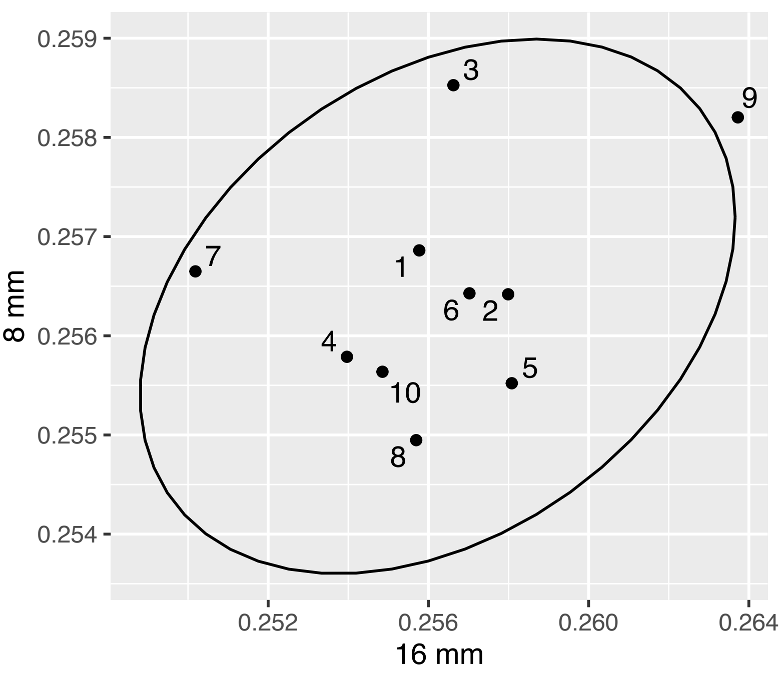

CT glrlm longrunhighgraylevelemphasis

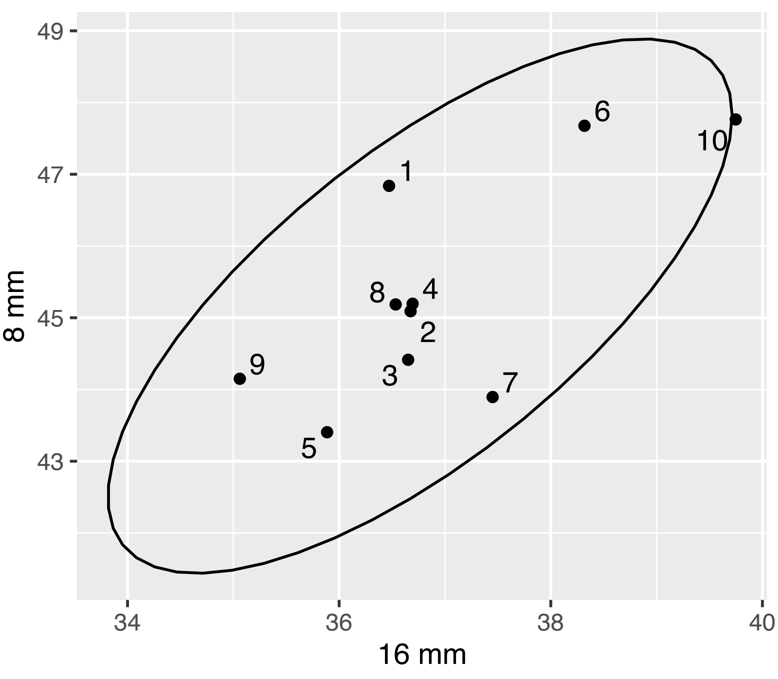

CT glrlm longrunlowgraylevelemphasis

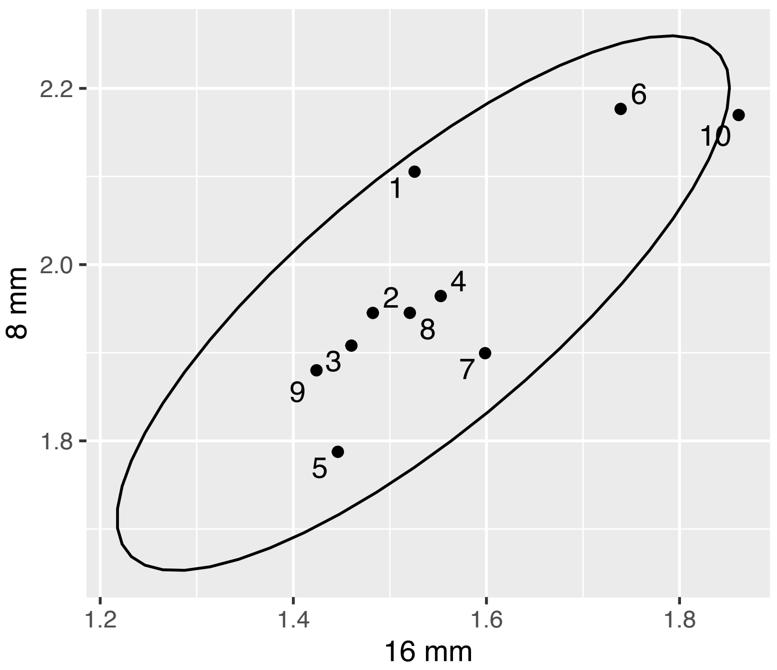

CT glrlm runlengthnonuniformity

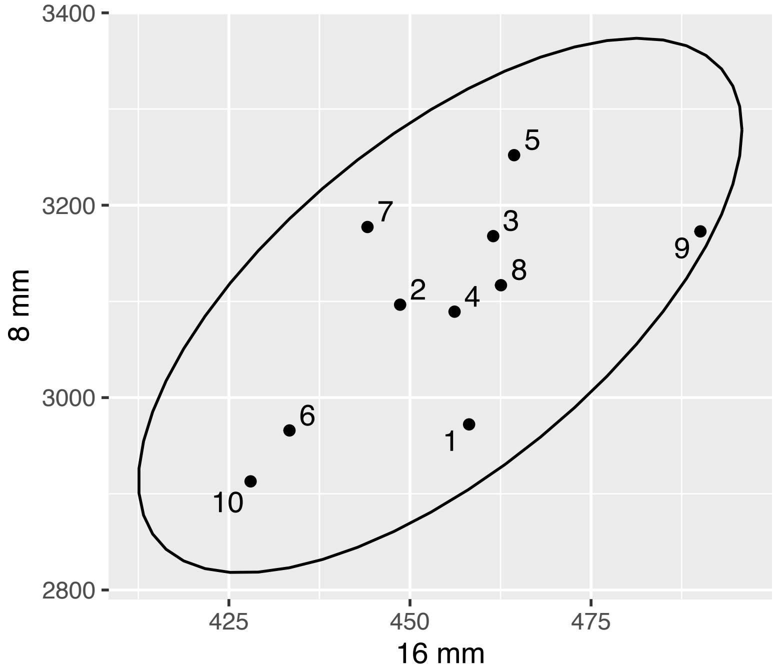

CT glrlm lowgraylevelrunemphasis

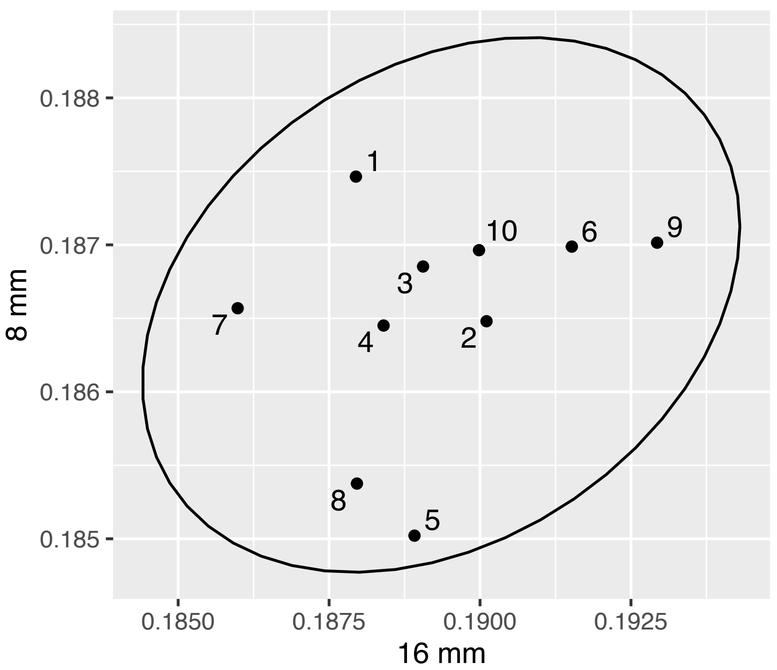

CT glrlm runlengthnonuniformitynormalized

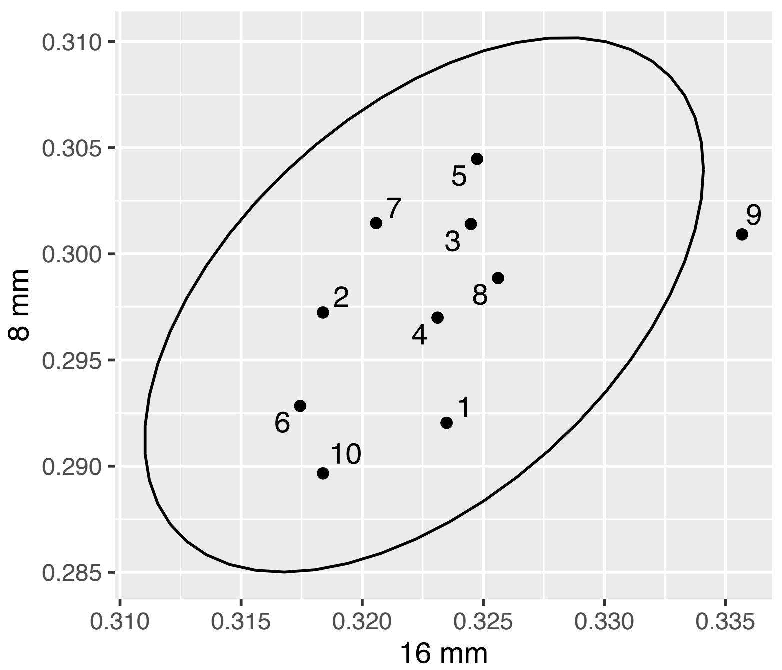

CT glrlm runentropy

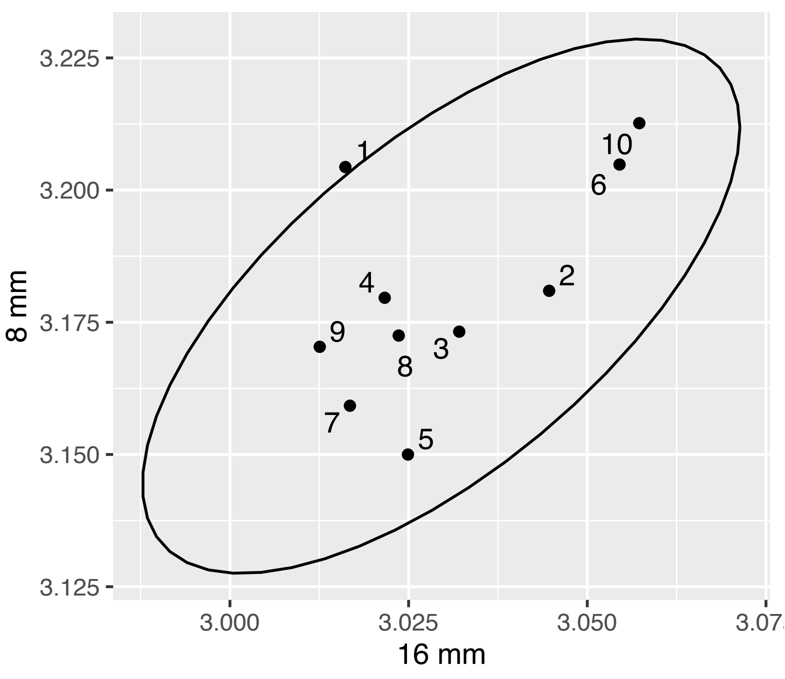

CT glrlm runpercentage

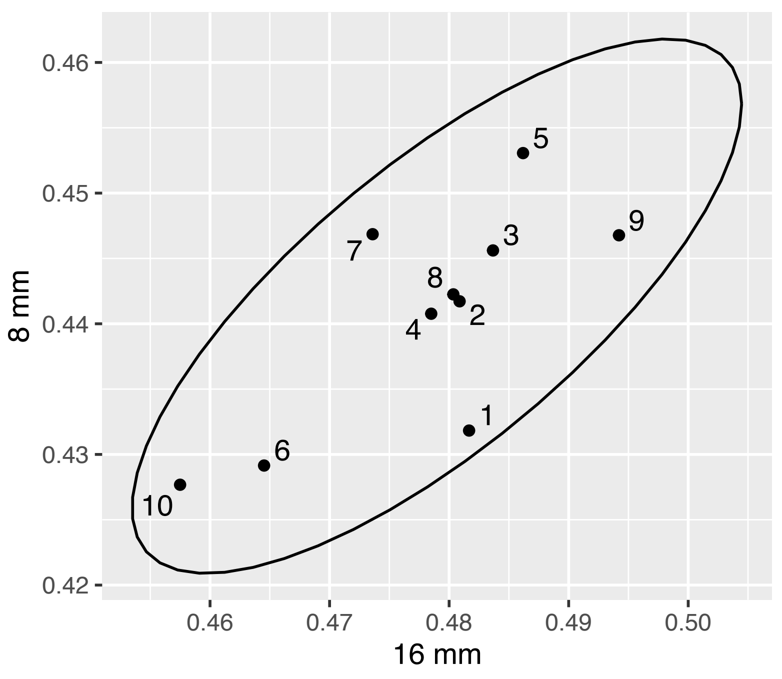

CT glrlm runvariance

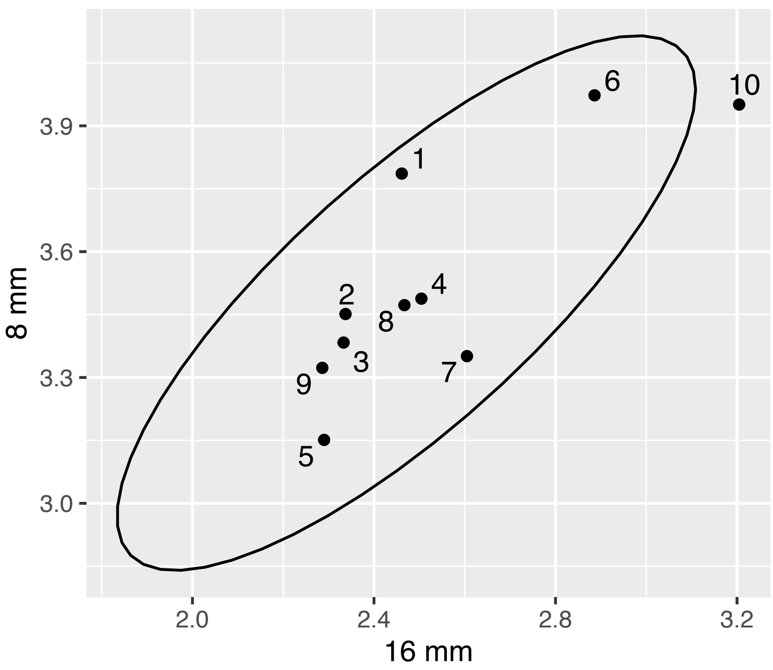

CT glrlm shortrunlowgraylevelemphasis

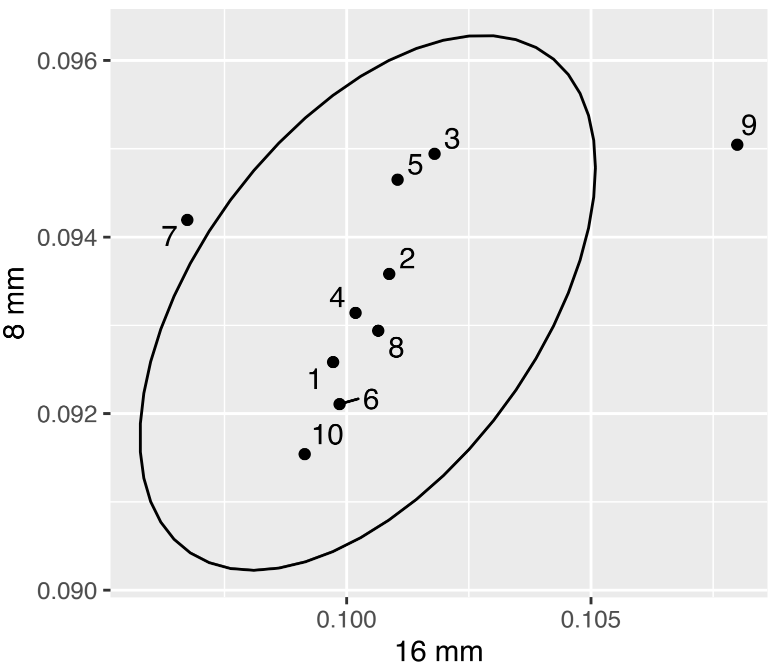

CT glrlm shortrunemphasis

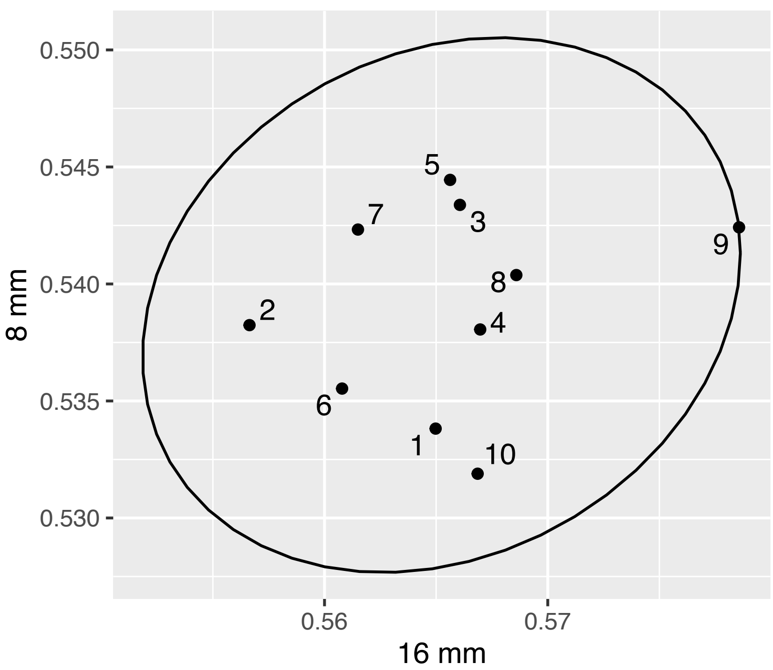

CT glszm graylevelnonuniformity

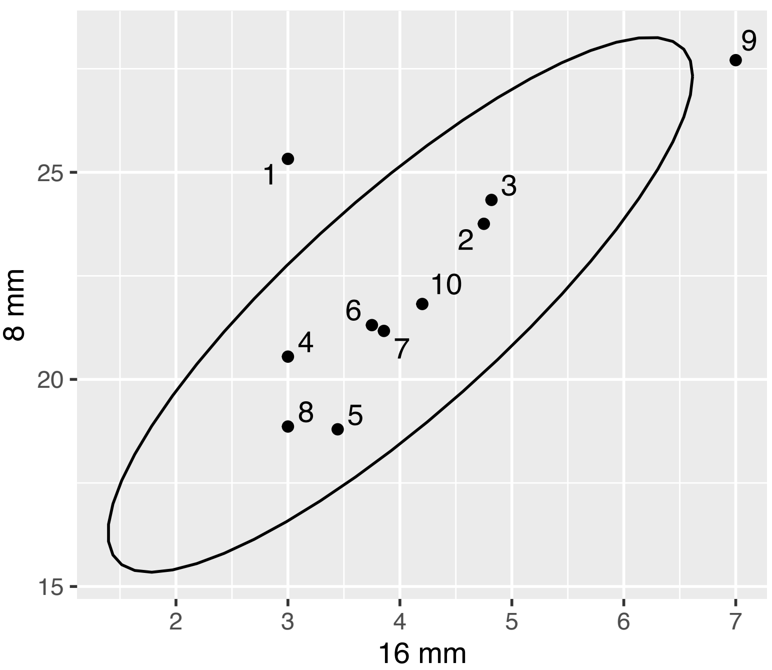

CT glrlm shortrunhighgraylevelemphasis

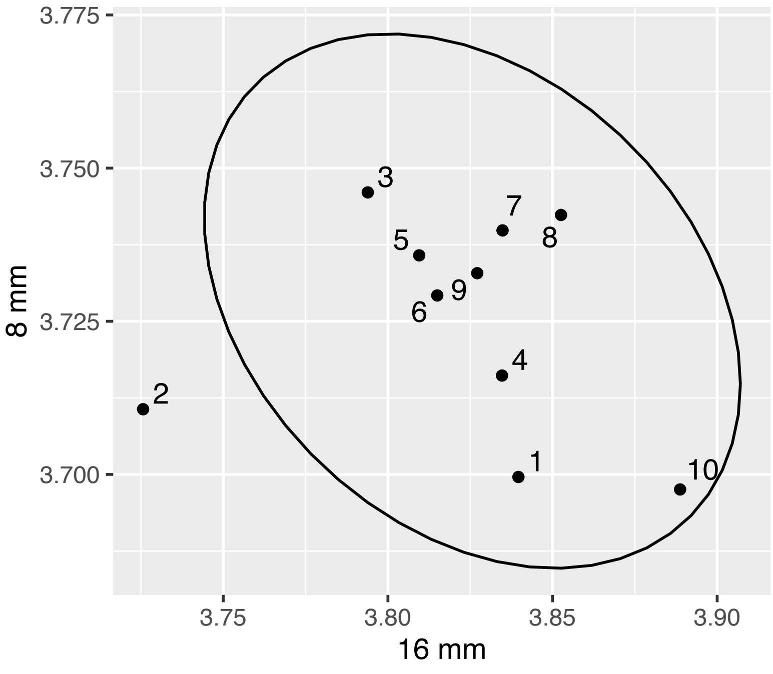

CT glszm graylevelnonuniformitynormalized

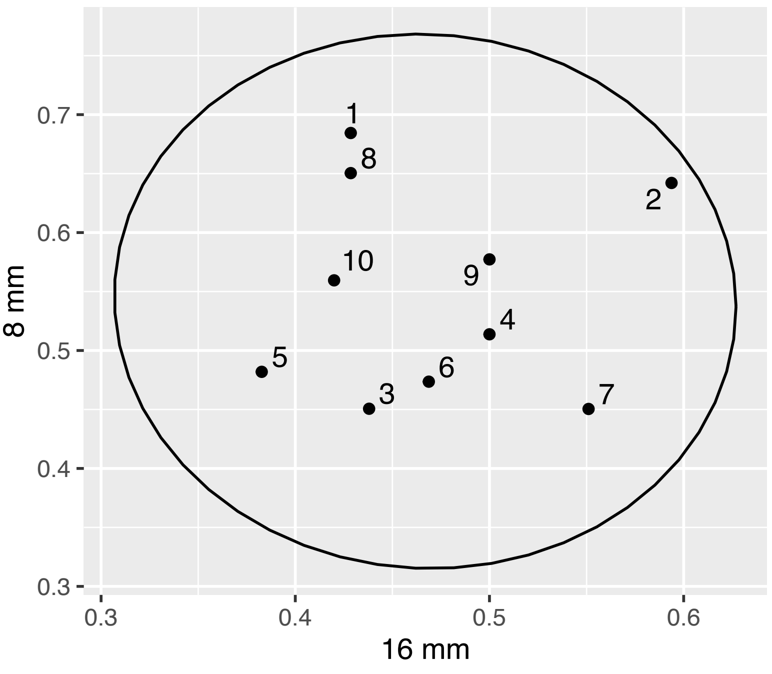

CT glszm graylevelvariance

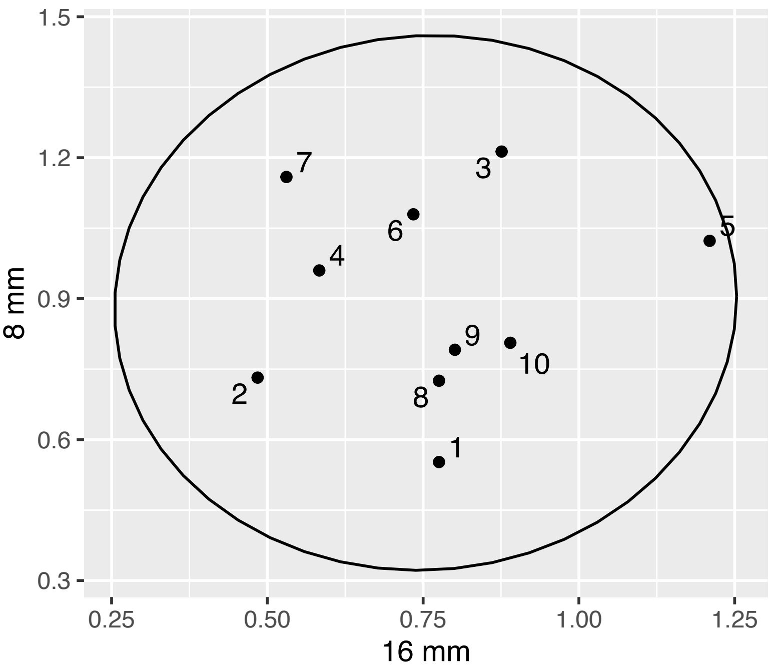

CT glszm largeareahighgraylevelemphasis

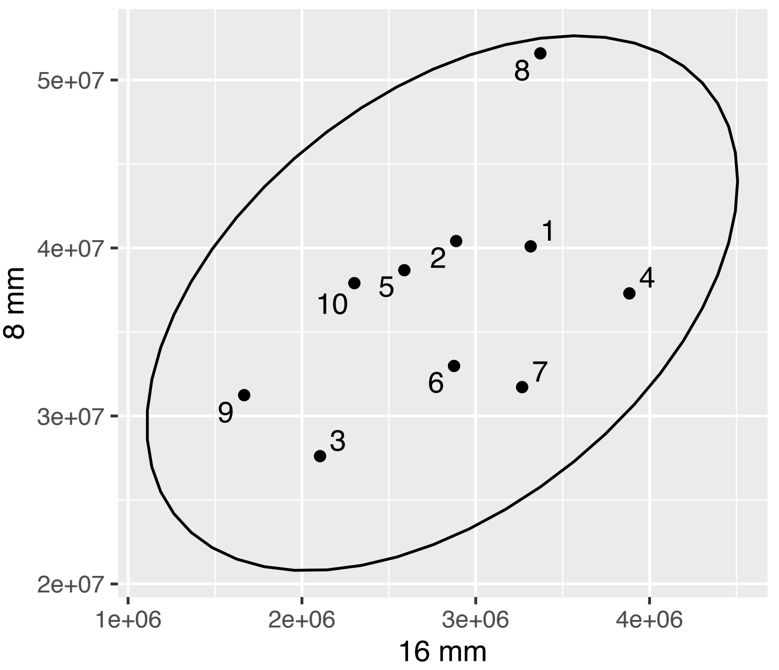

CT glszm highgraylevelzoneemphasis

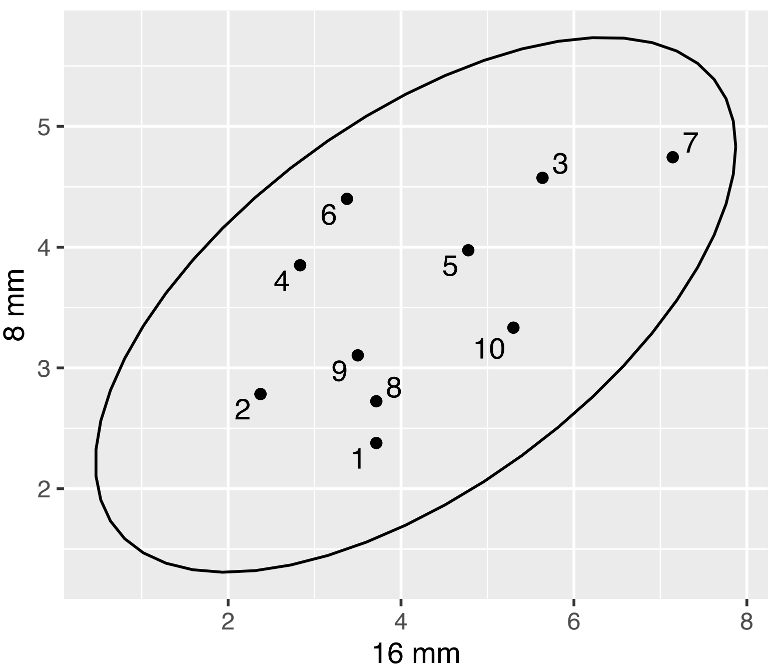

CT glszm largearealowgraylevelemphasis

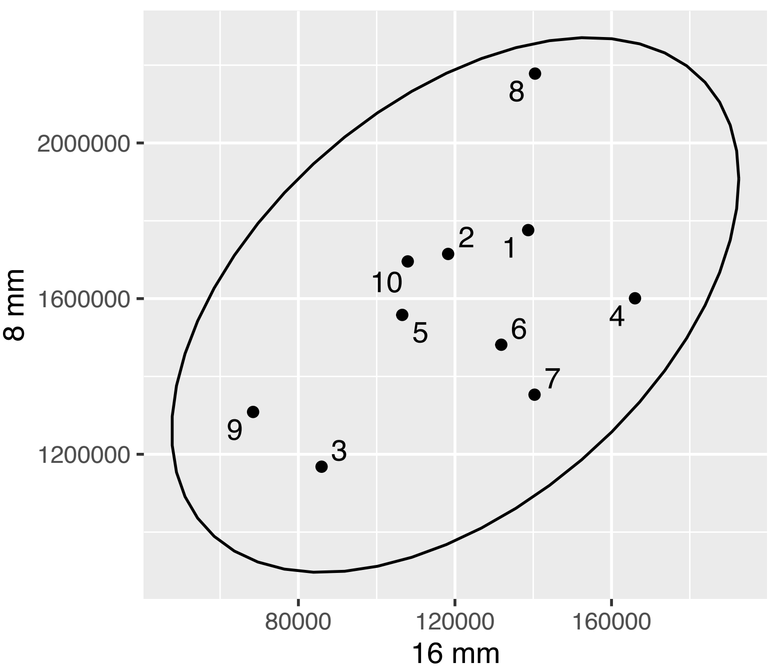

CT glszm largeareaemphasis

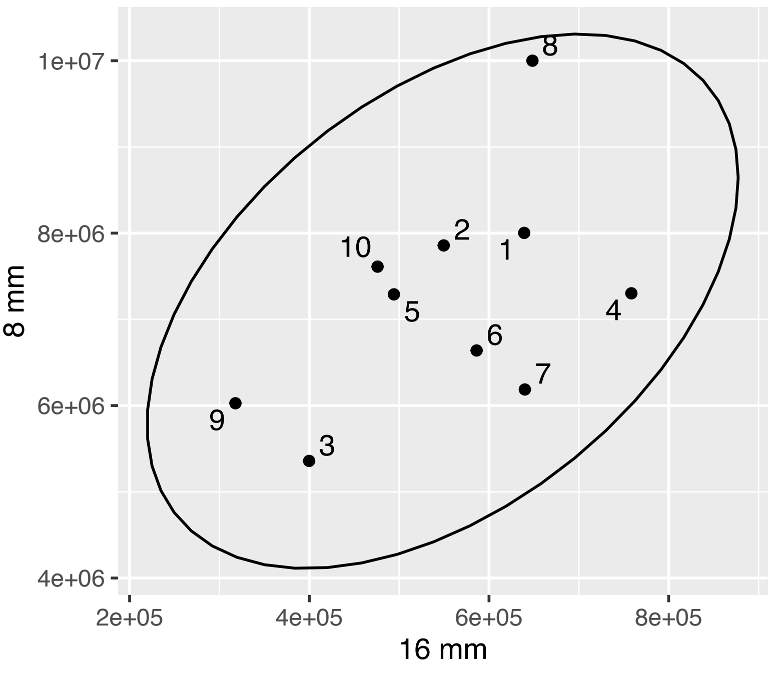

CT glszm lowgraylevelzoneemphasis

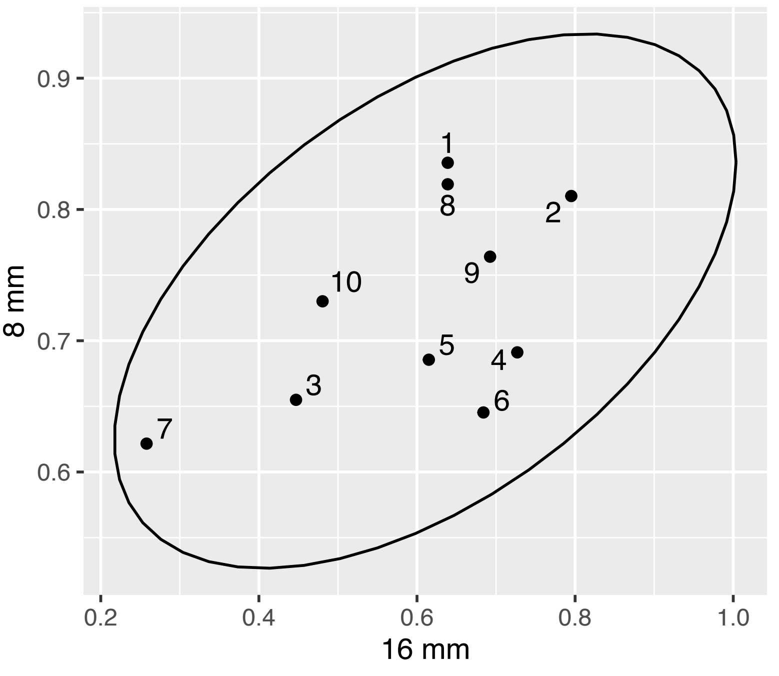

CT glszm sizezonenonuniformity

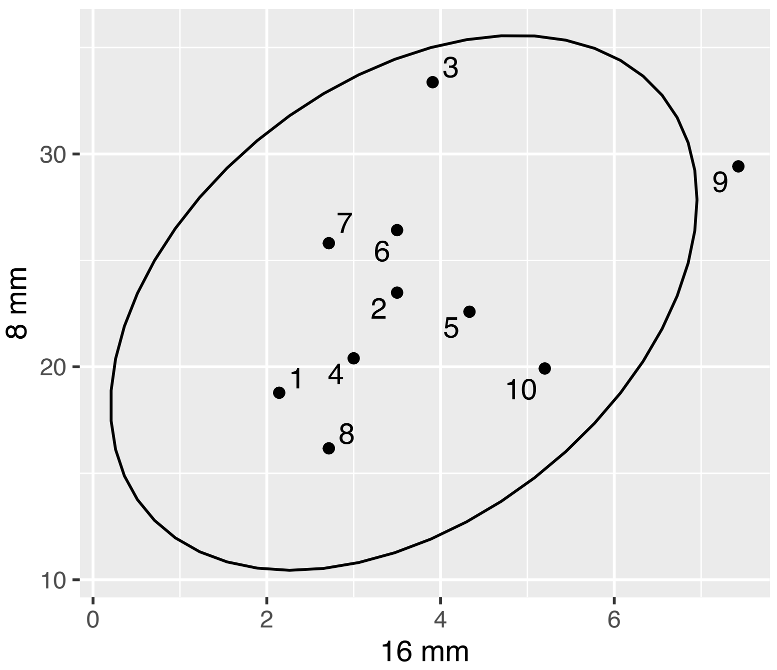

CT glszm smallareahighgraylevelemphasis

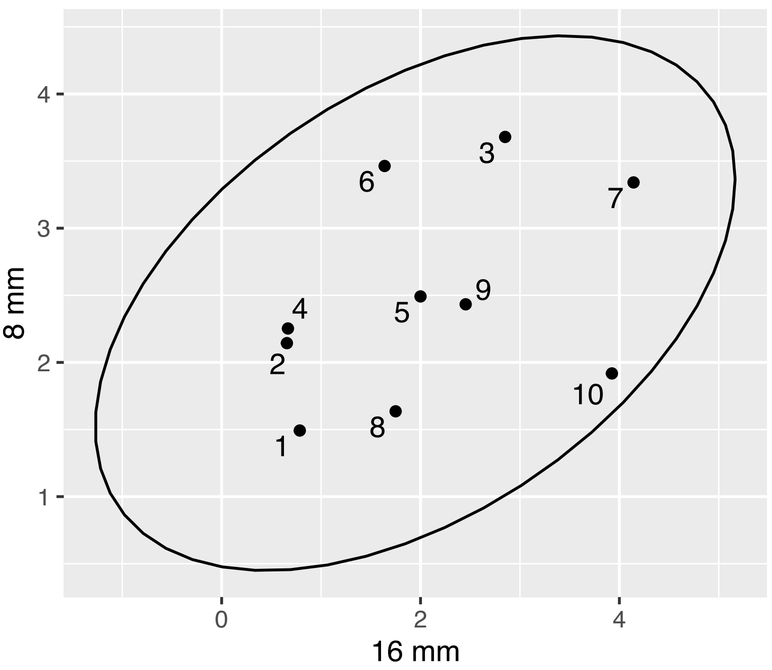

CT glszm sizezonenonuniformitynormalized

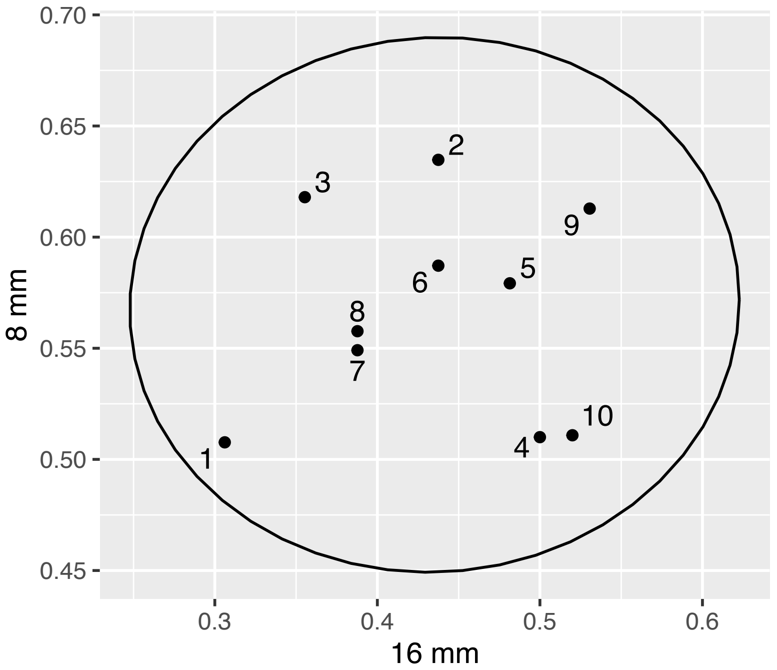

CT glszm smallarealowgraylevelemphasis

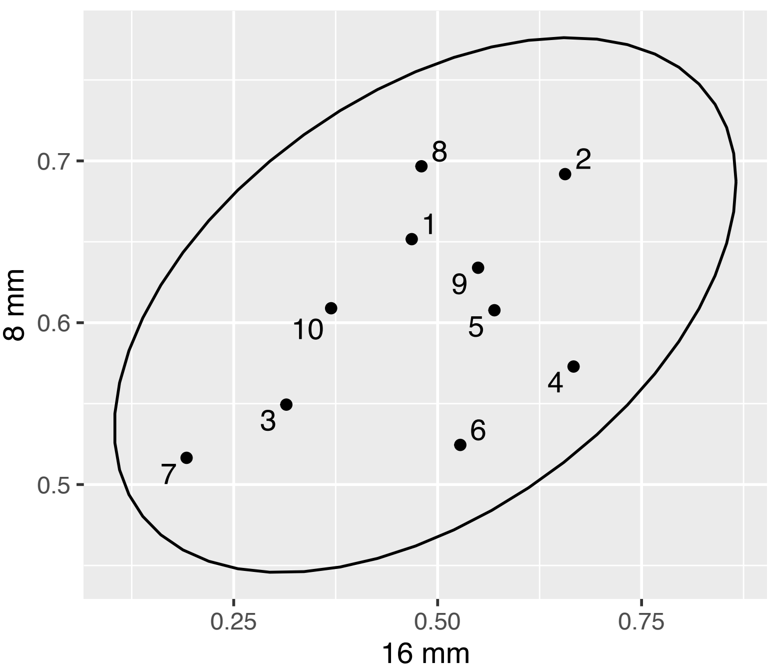

CT glszm smallareaemphasis

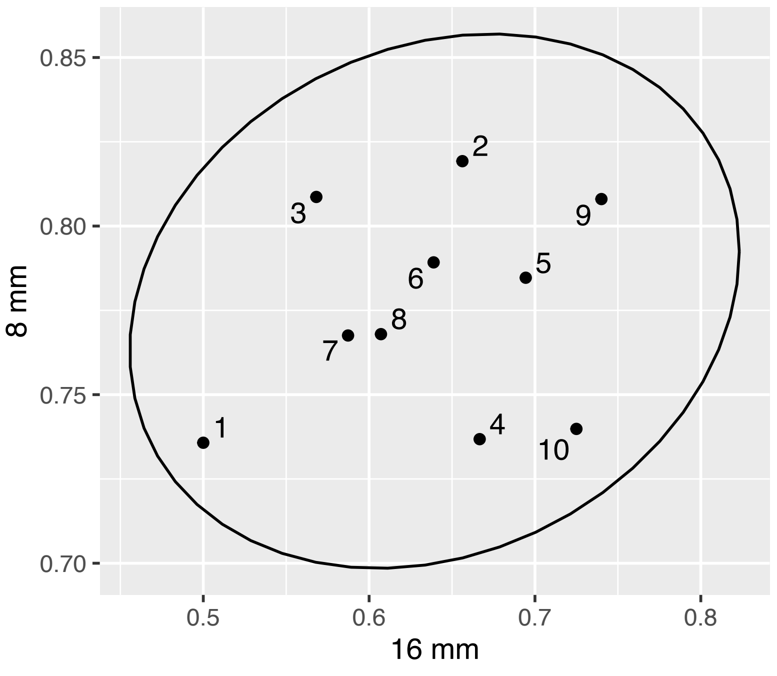

CT glszm zoneentropy

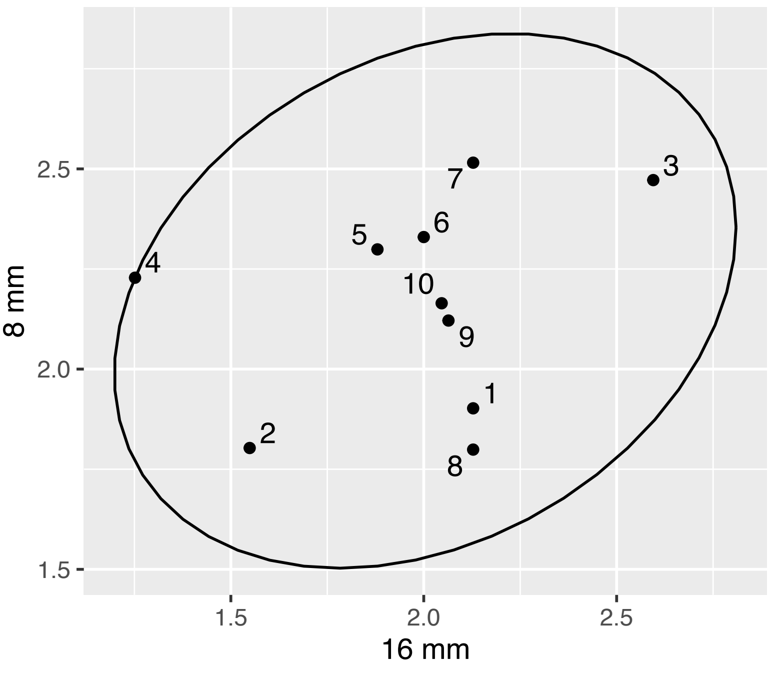

CT glszm zonepercentage

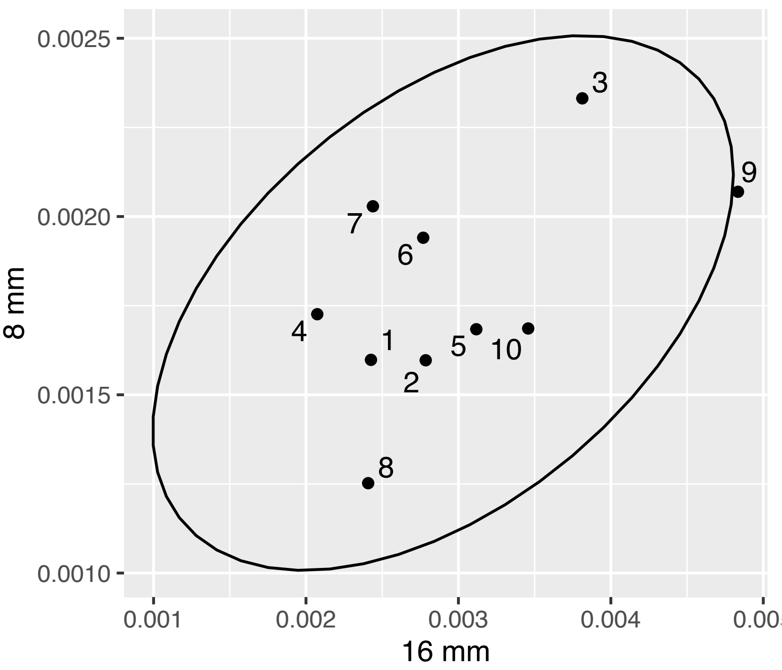

CT gldm dependencenonuniformity

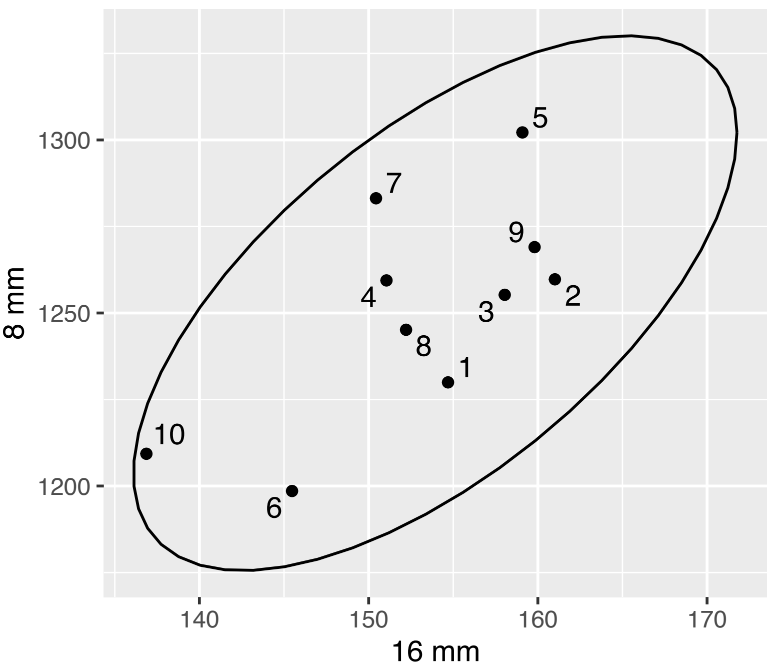

CT glszm zonevariance

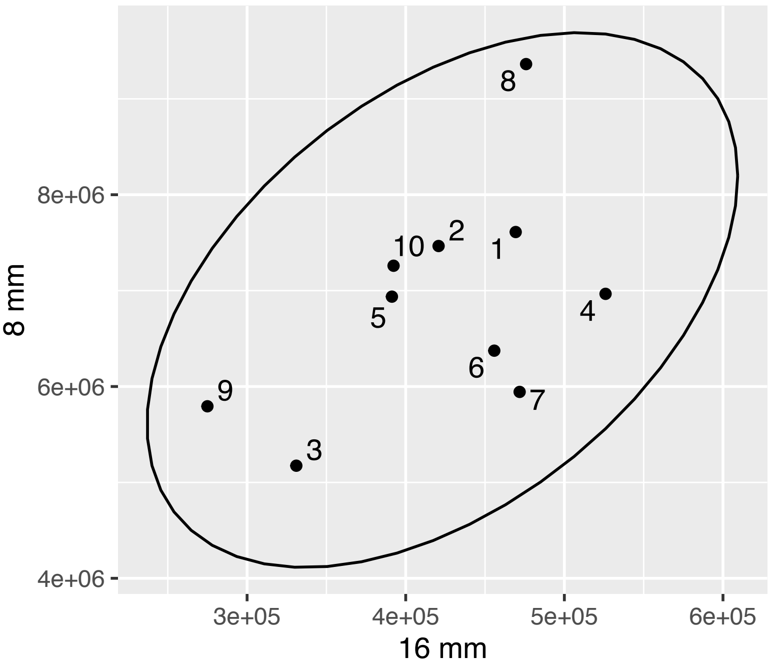

CT gldm dependencenonuniformitynormaliz

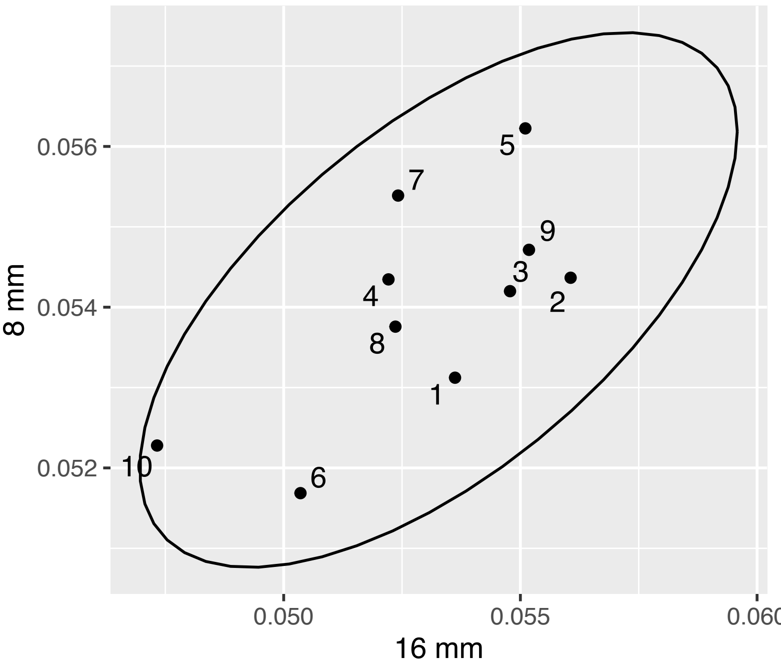

CT gldm dependenceentropy

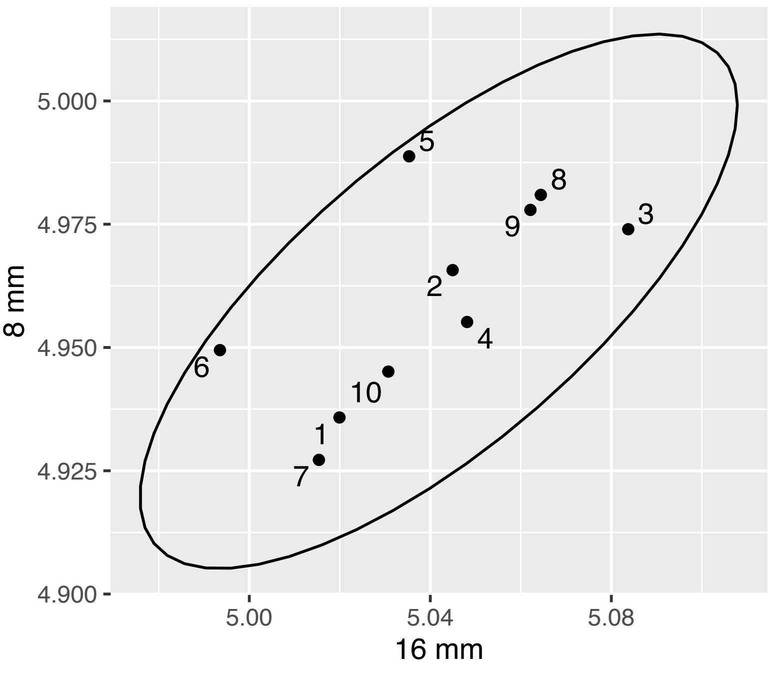

CT gldm dependencevariance

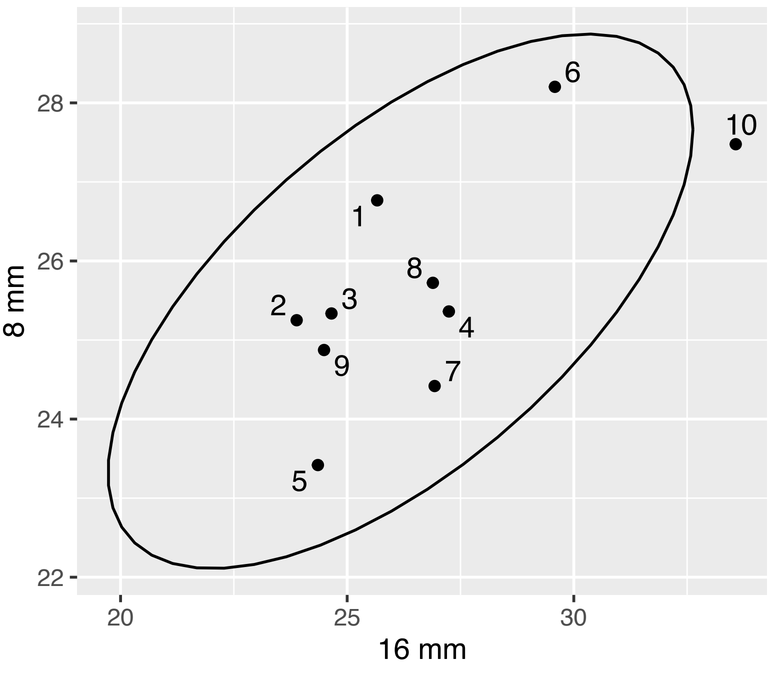

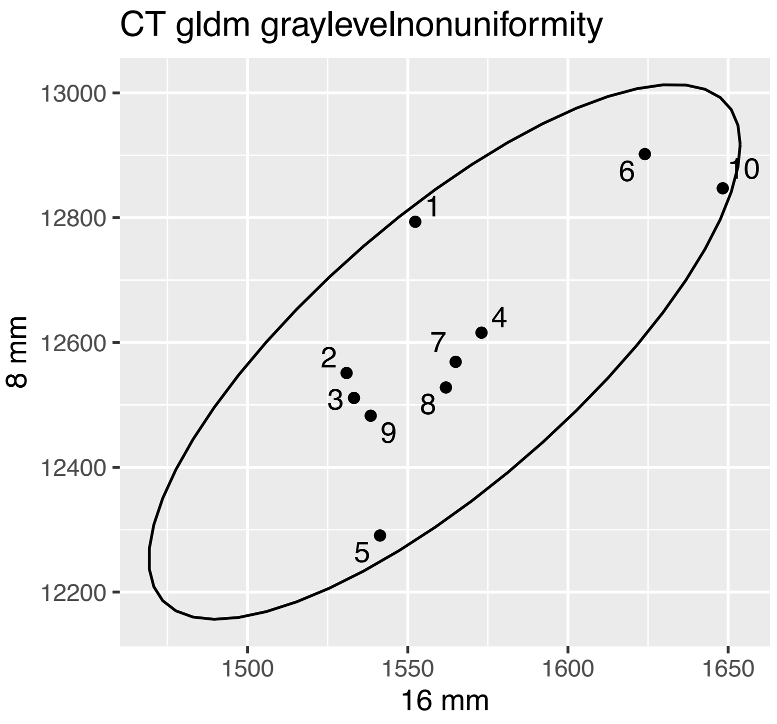
CT gldm graylevelnonuniformity
8 mm
16 mm

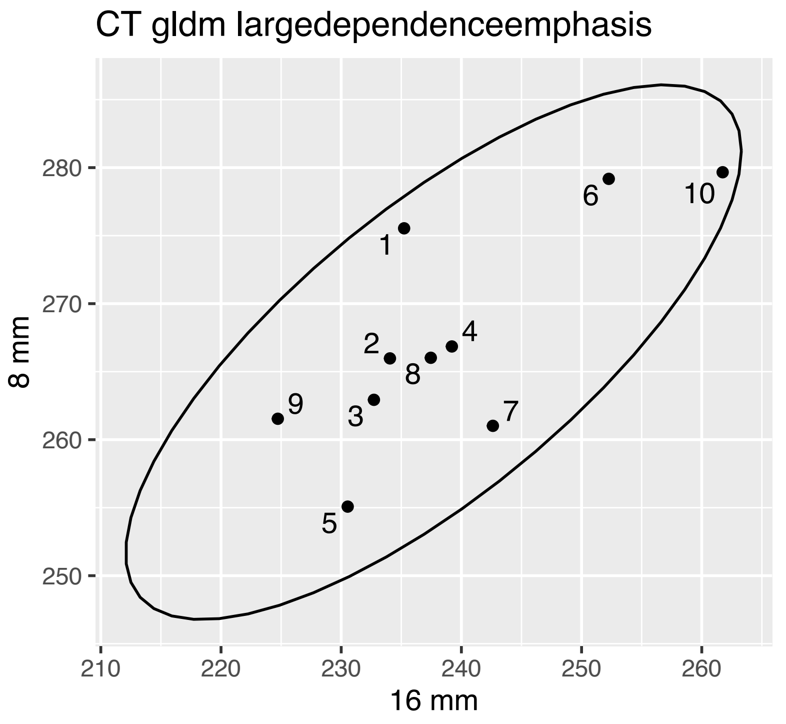
CT gldm largedependenceemphasis
8 mm
16 mm

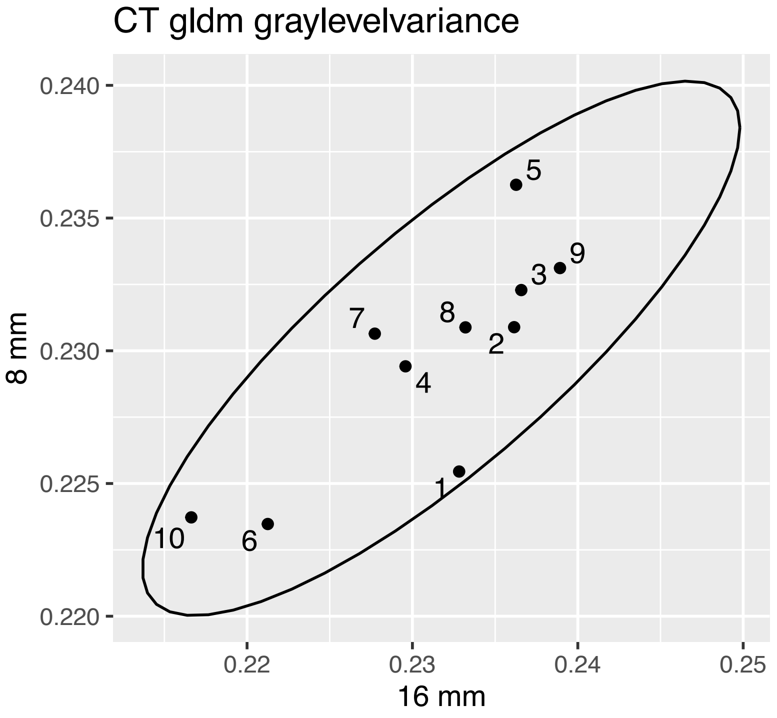
CT gldm graylevelvariance
8 mm
16 mm

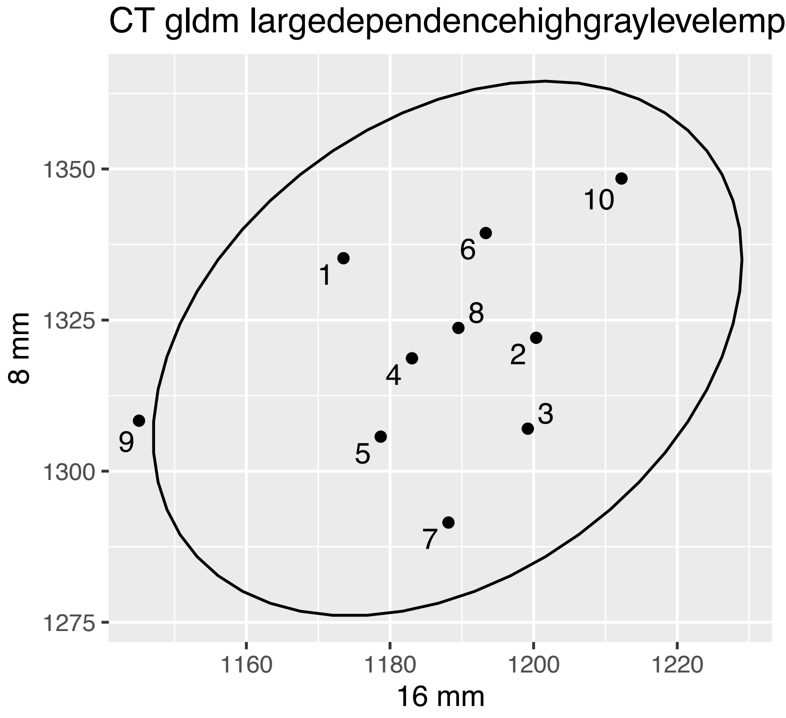
CT gldm largedependencehighgraylevelemp
8 mm
16 mm

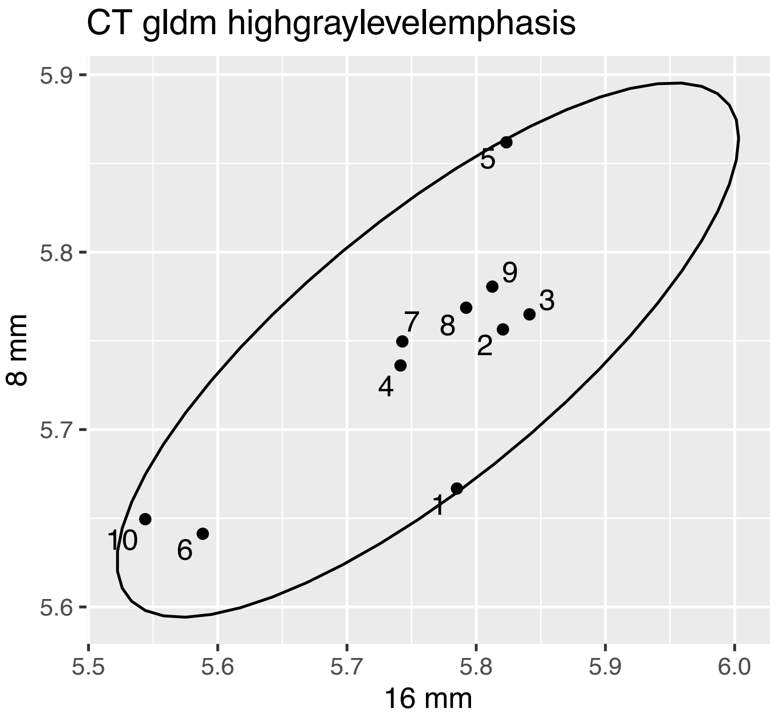
CT gldm highgraylevelemphasis
8 mm
16 mm

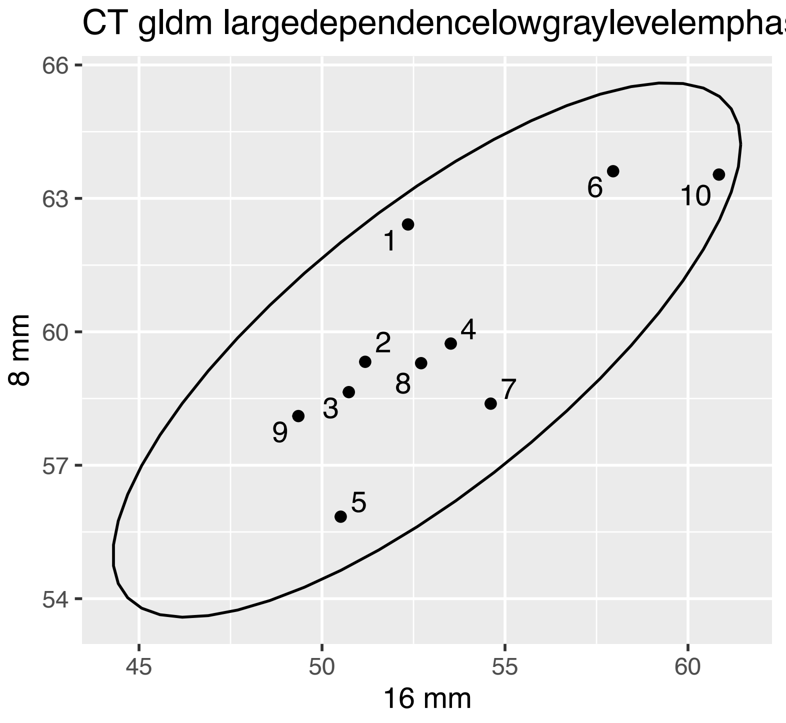
CT gldm largedependencelowgraylevelemphas
8 mm
16 mm

CT gldm lowgraylevelemphasis

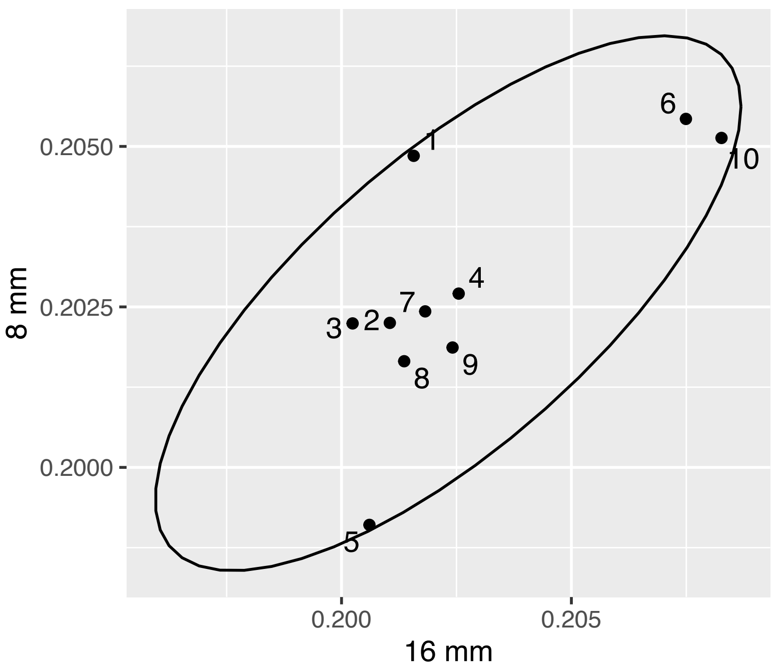

CT gldm smalldependencelowgraylevelemp

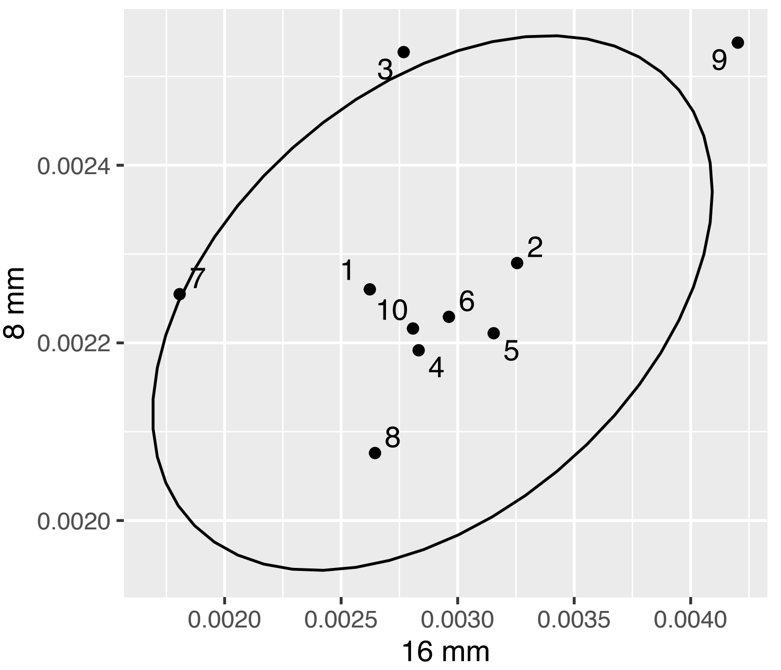

CT gldm smalldependenceemphasis

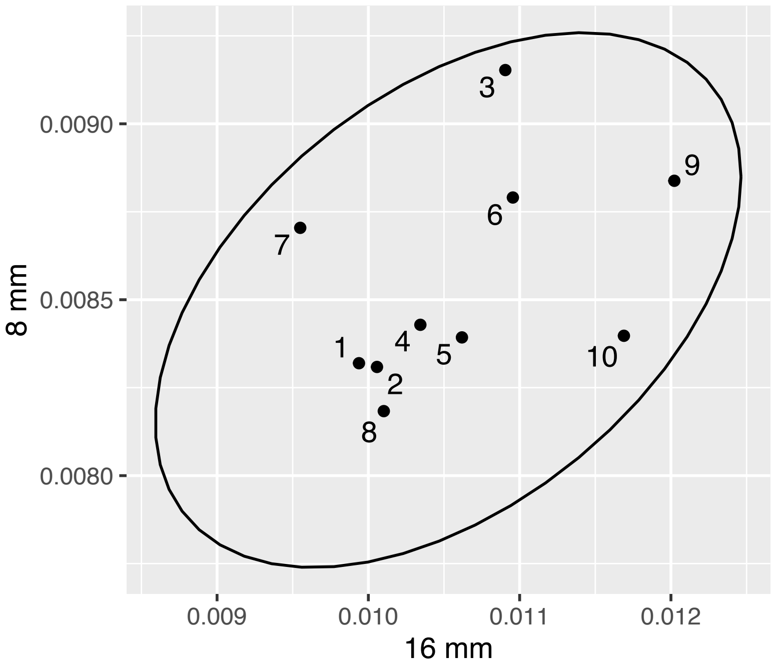

CT ngtdm busyness

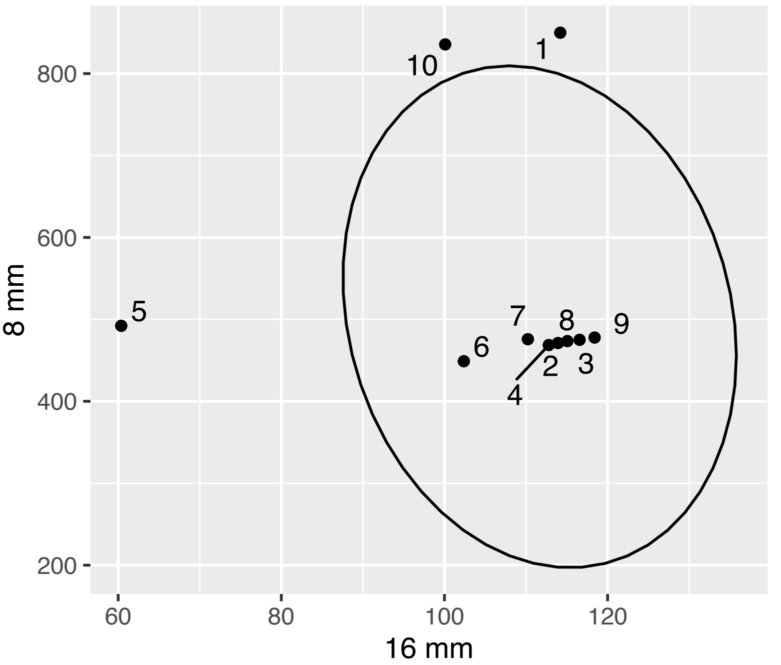

CT gldm smalldependencehighgraylevelemp

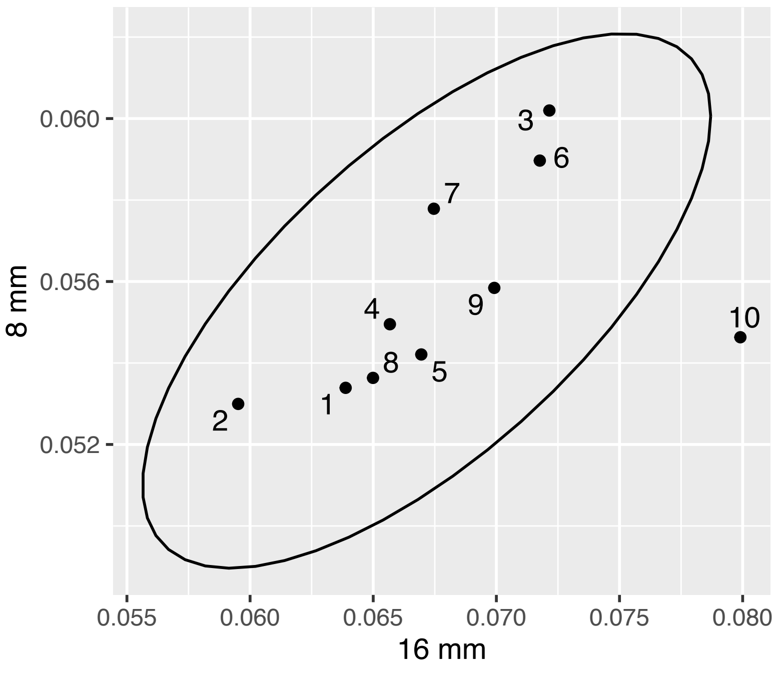

CT ngtdm coarseness

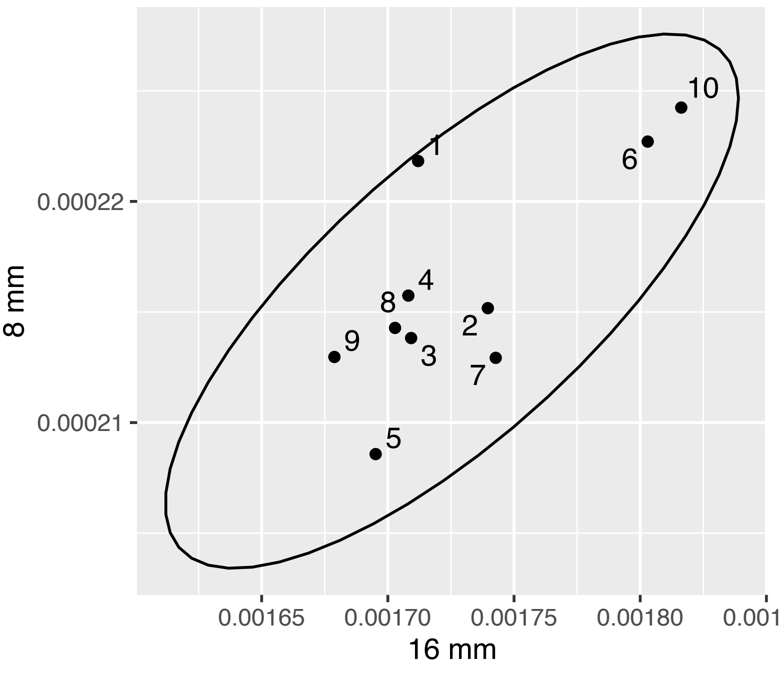

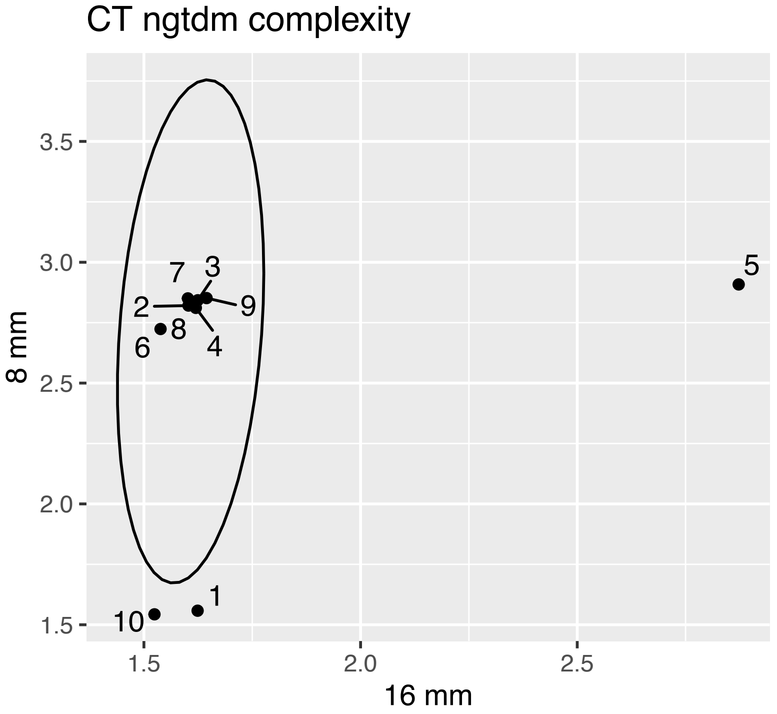
CT ngtdm complexity
8 mm
16 mm

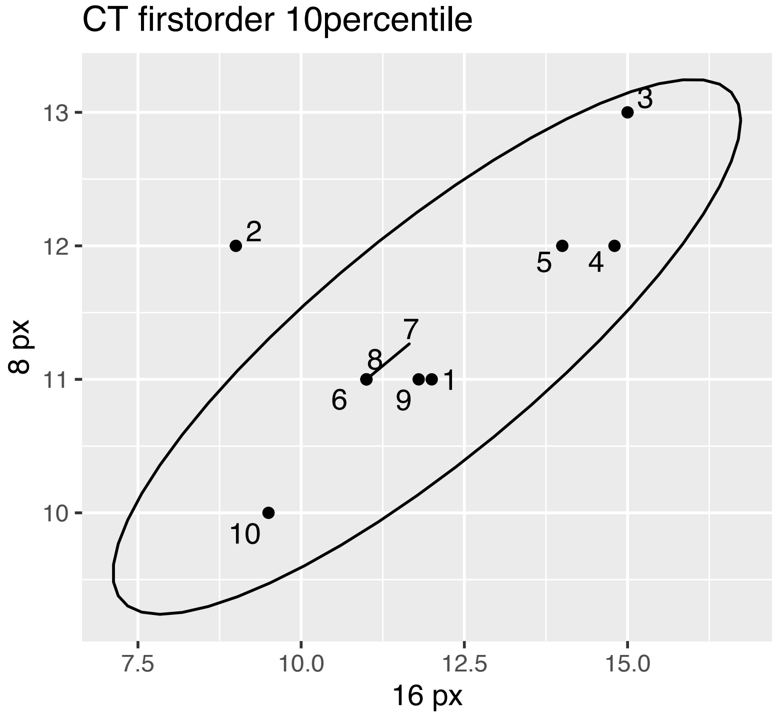
CT firstorder 10percentile
8 px
16 px

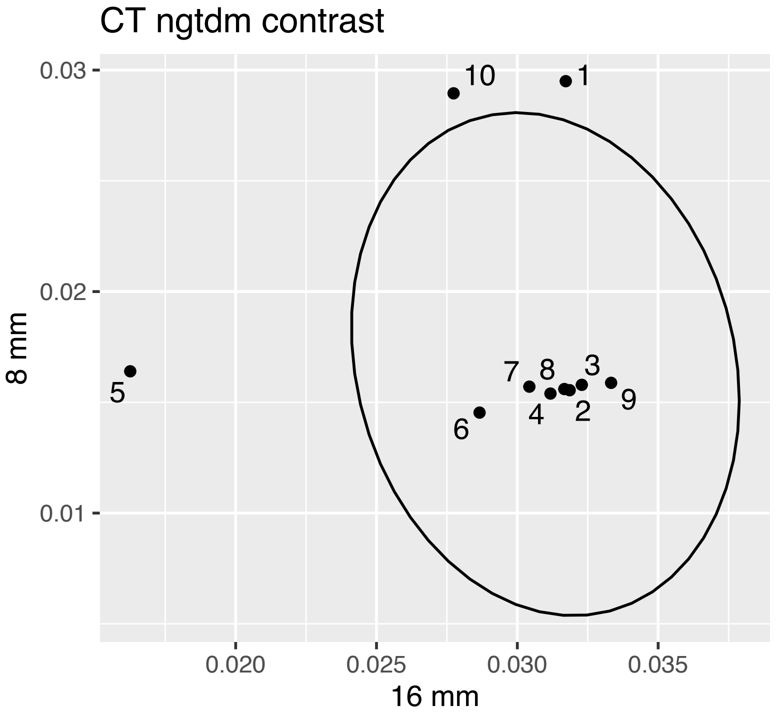
CT ngtdm contrast
8 mm
16 mm

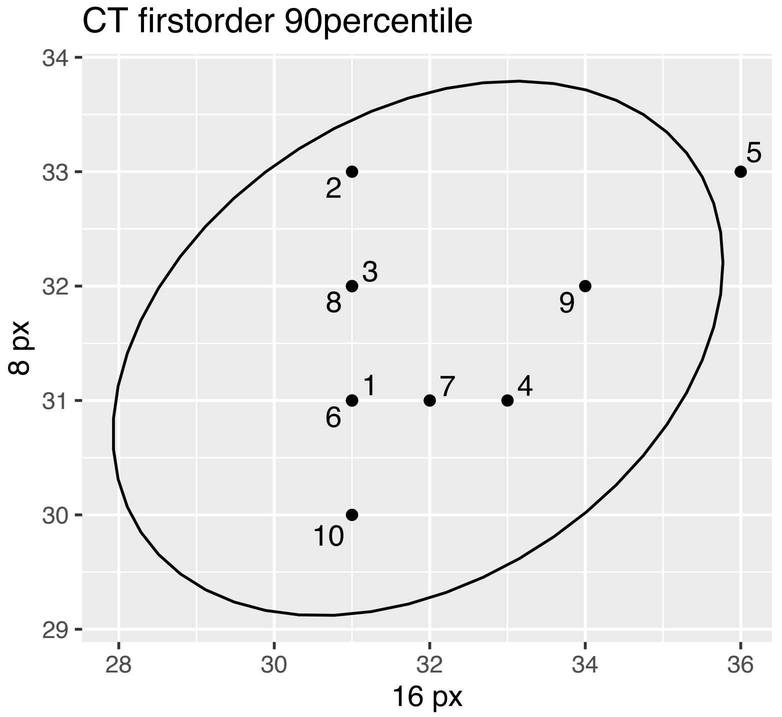
CT firstorder 90percentile
8 px
16 px

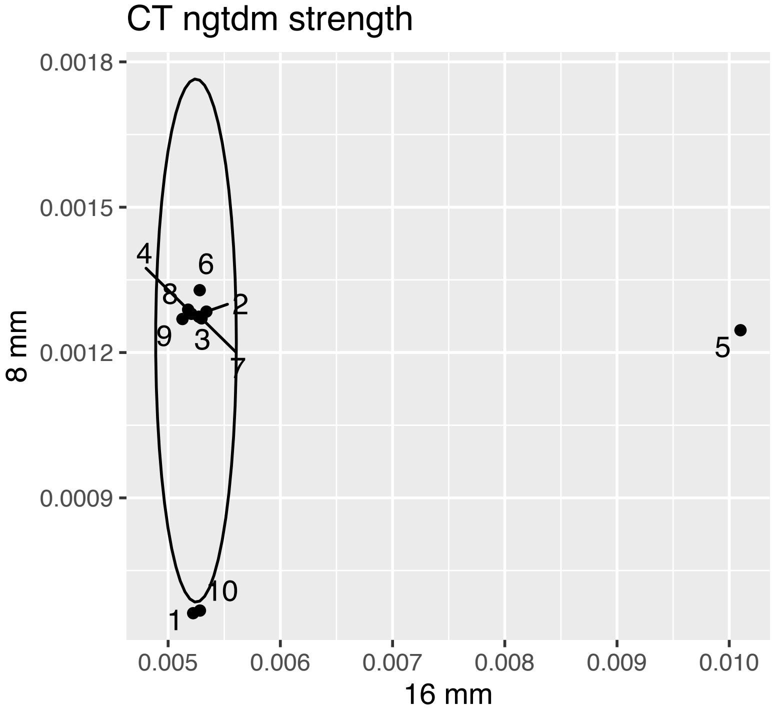
CT ngtdm strength
8 mm
16 mm

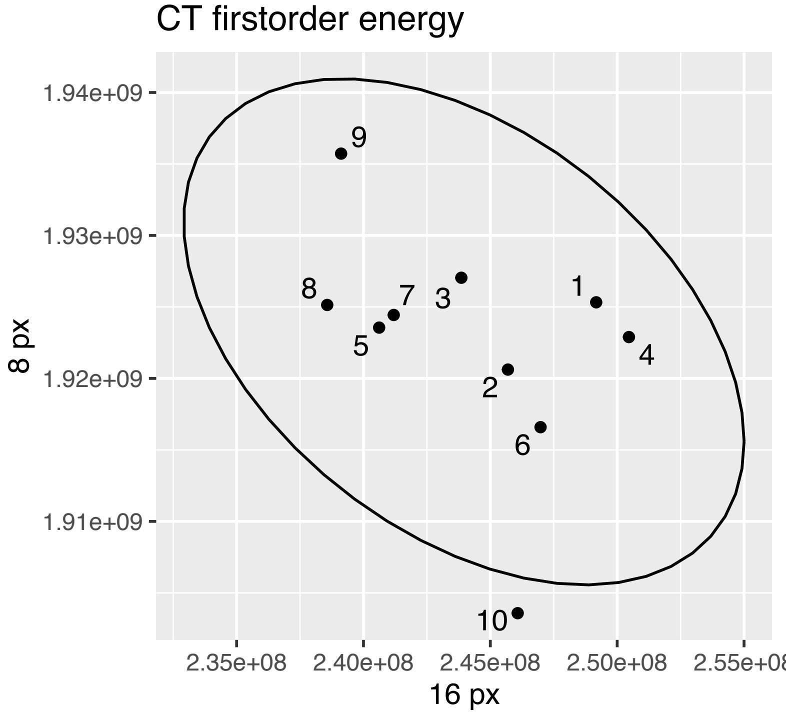
CT firstorder energy
8 px
16 px

CT firstorder entropy

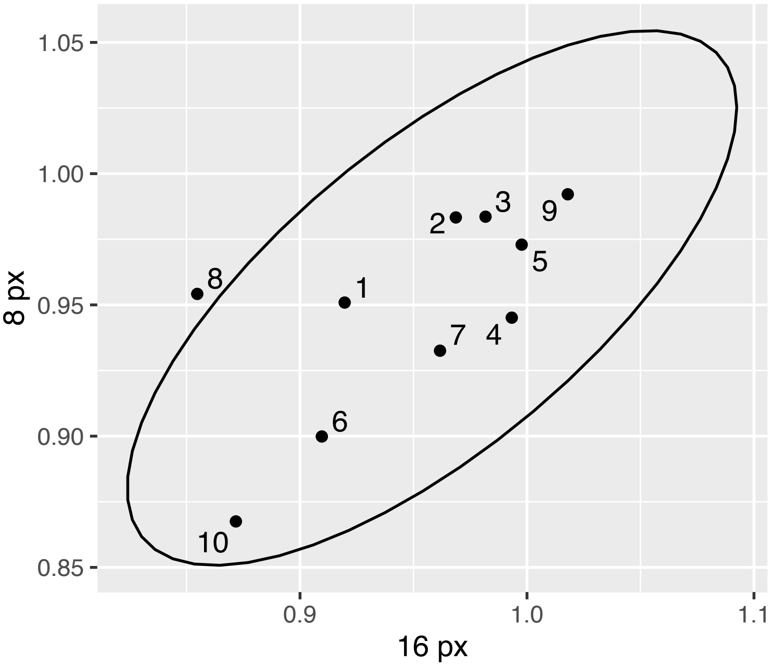

CT firstorder maximum

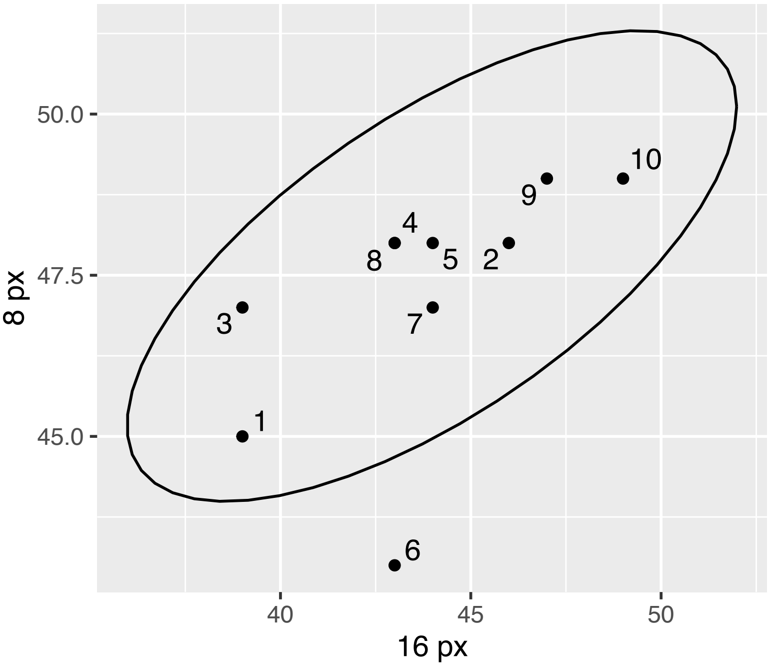

CT firstorder interquartilerange

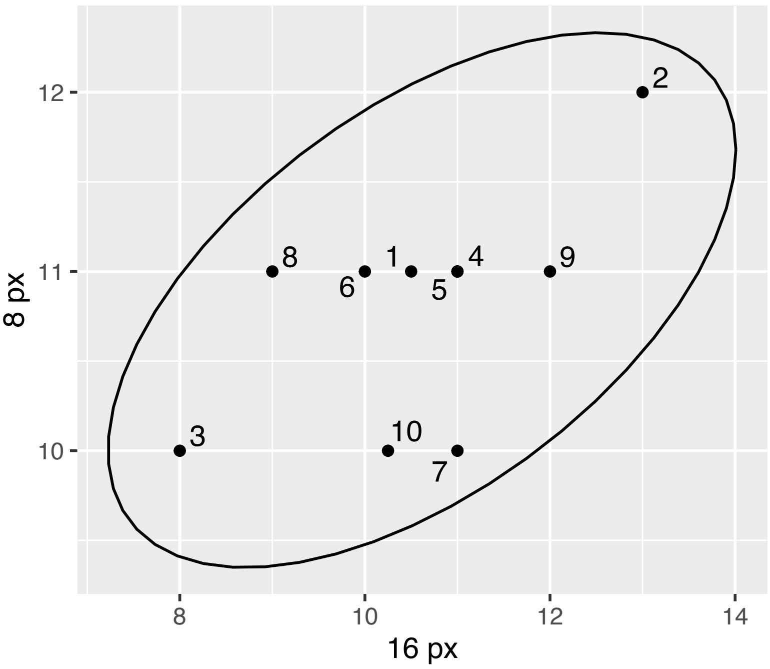

CT firstorder meanabsolutedeviation

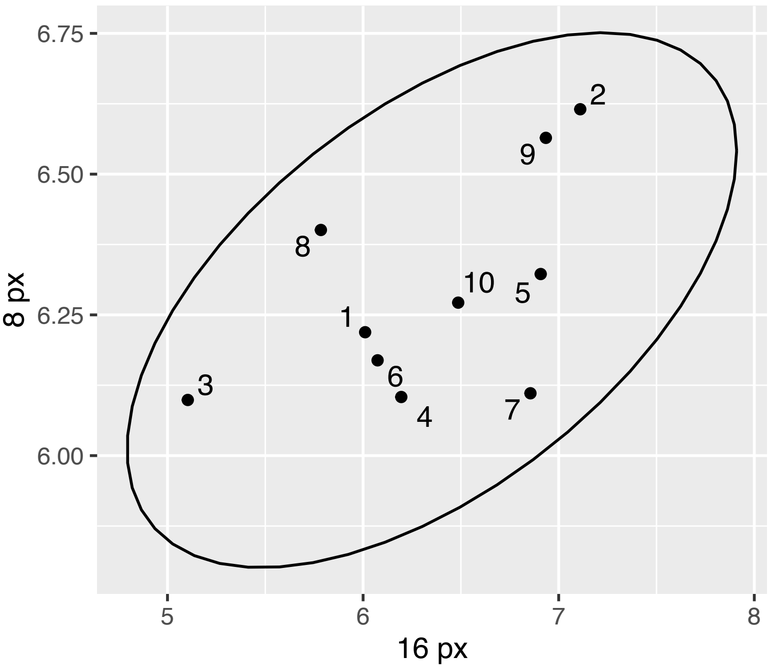

CT firstorder kurtosis

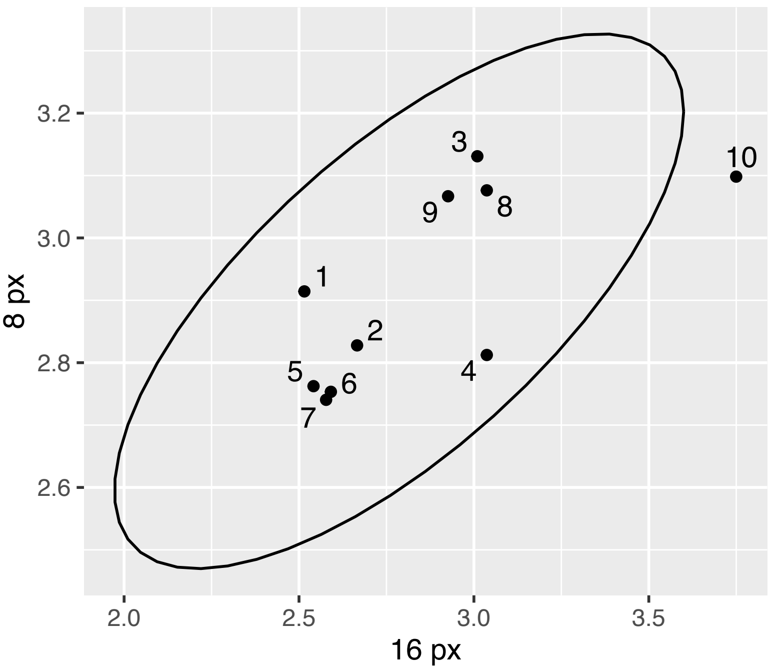

CT firstorder mean

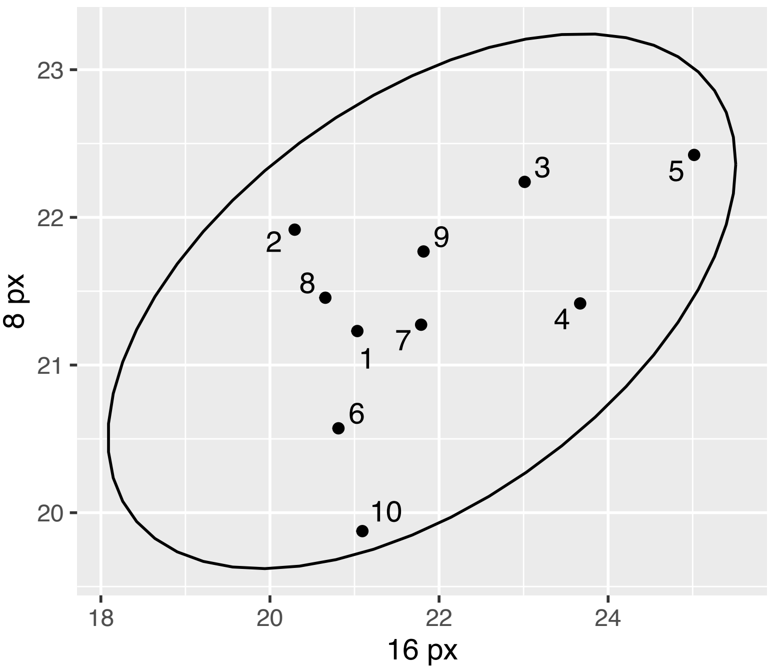

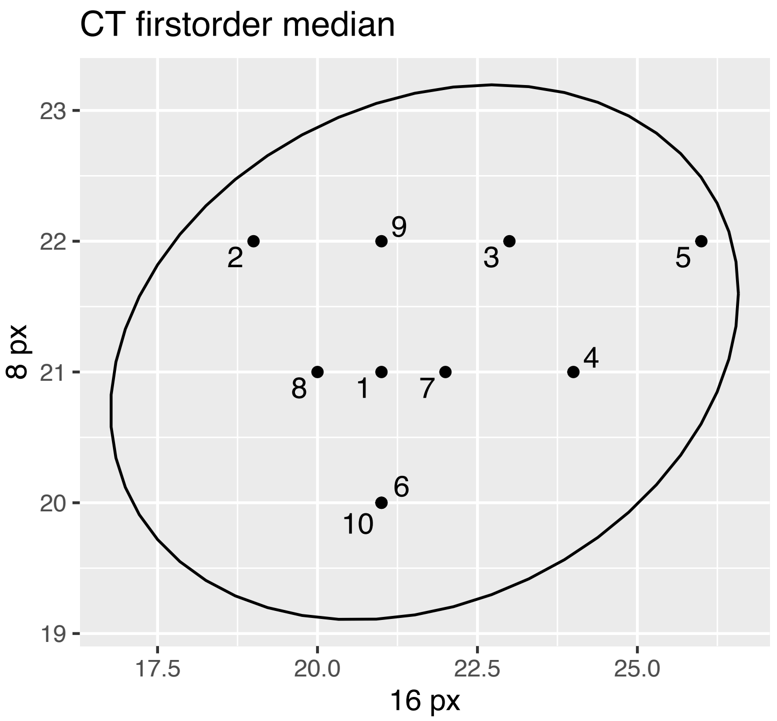
CT firstorder median
8 px
16 px
23
22
21
20
19
17.5
20.0
22.5
25.0
1
2
3
4
5
6
7
8
9
10

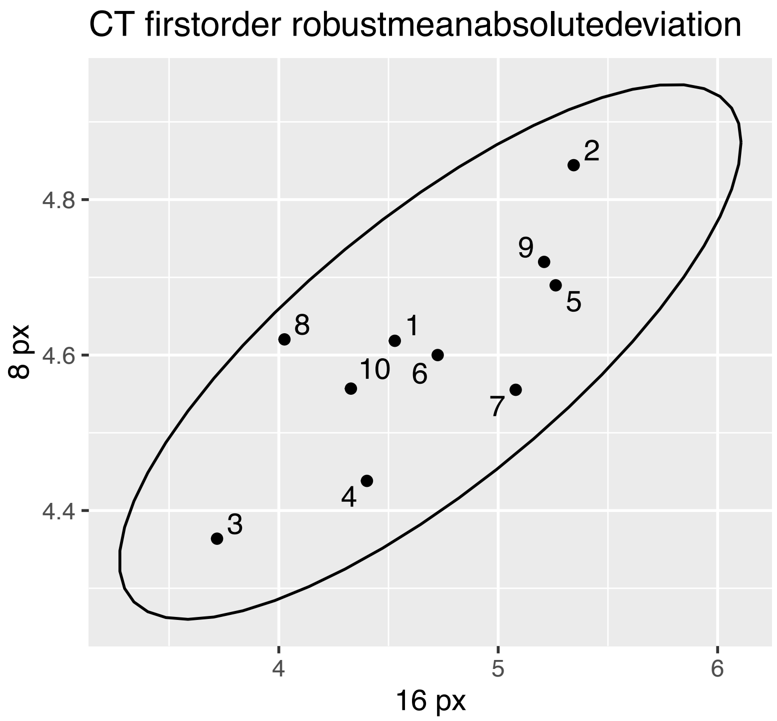
CT firstorder robustmeanabsolutedeviation
8 px
16 px
4.8
4.6
4.4
4
5
6
1
2
3
4
5
6
7
8
9
10

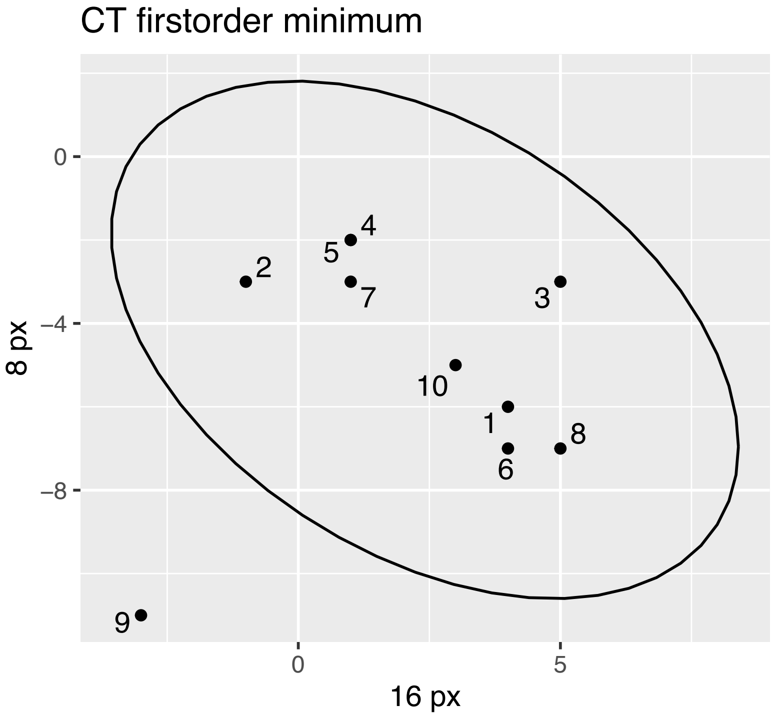
CT firstorder minimum
8 px
16 px
0
−4
−8
0
5
1
2
3
4
5
6
7
8
9
10

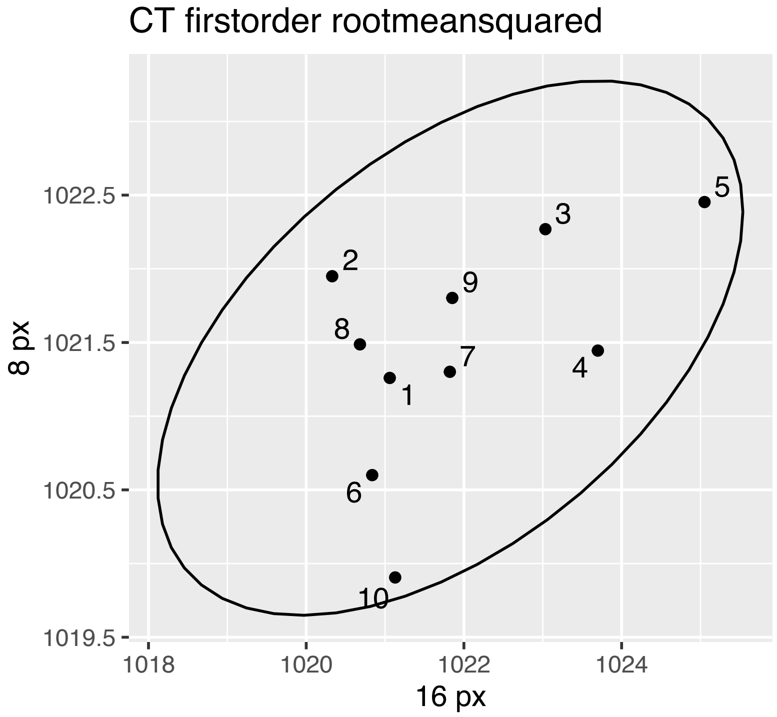
CT firstorder rootmeansquared
8 px
16 px
1022.5
1021.5
1020.5
1019.5
1018
1020
1022
1024
1
2
3
4
5
6
7
8
9
10

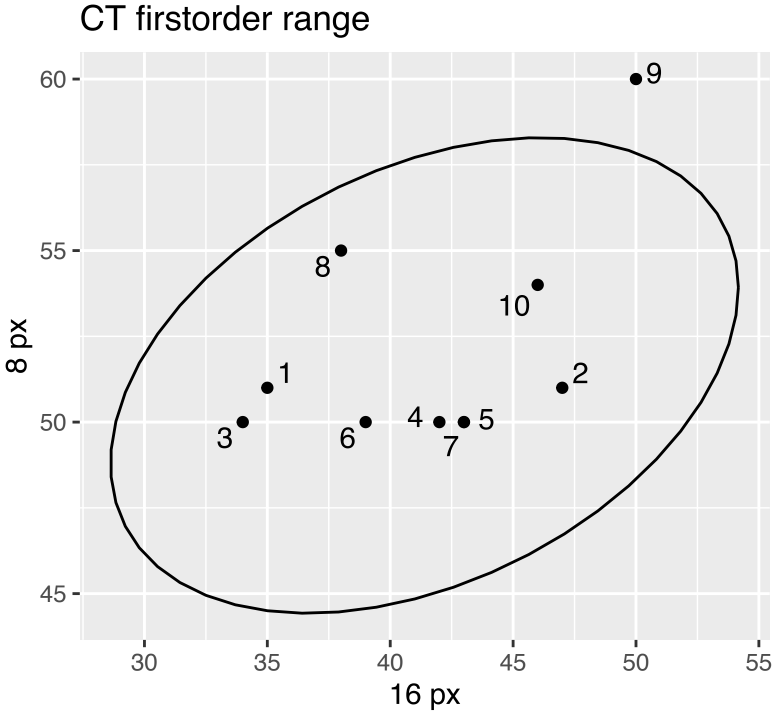
CT firstorder range
8 px
16 px
60
55
50
45
30
35
40
45
50
55
1
2
3
4
5
6
7
8
9
10

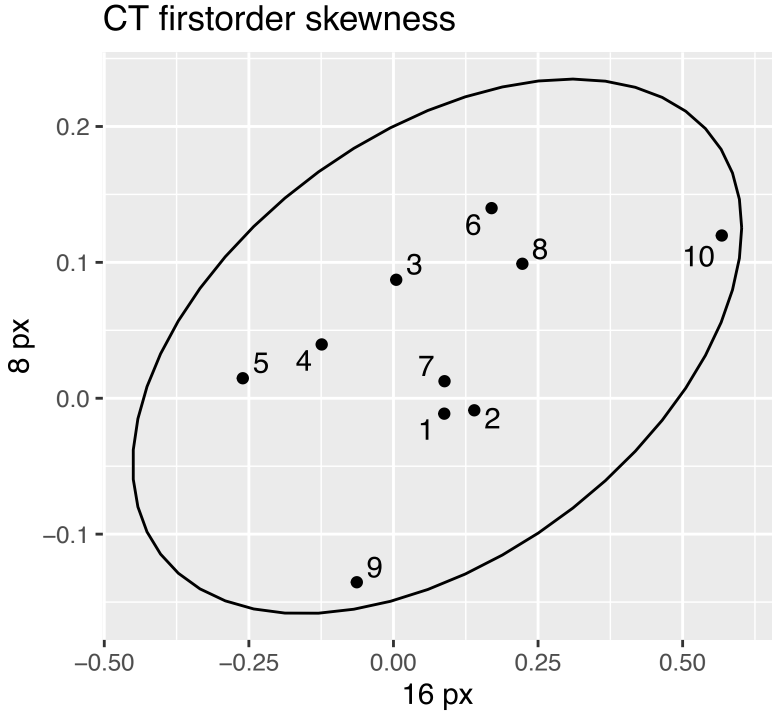
CT firstorder skewness
8 px
16 px
0.2
0.1
0.0
−0.1
−0.50
−0.25
0.00
0.25
0.50
1
2
3
4
5
6
7
8
9
10

CT firstorder totalenergy

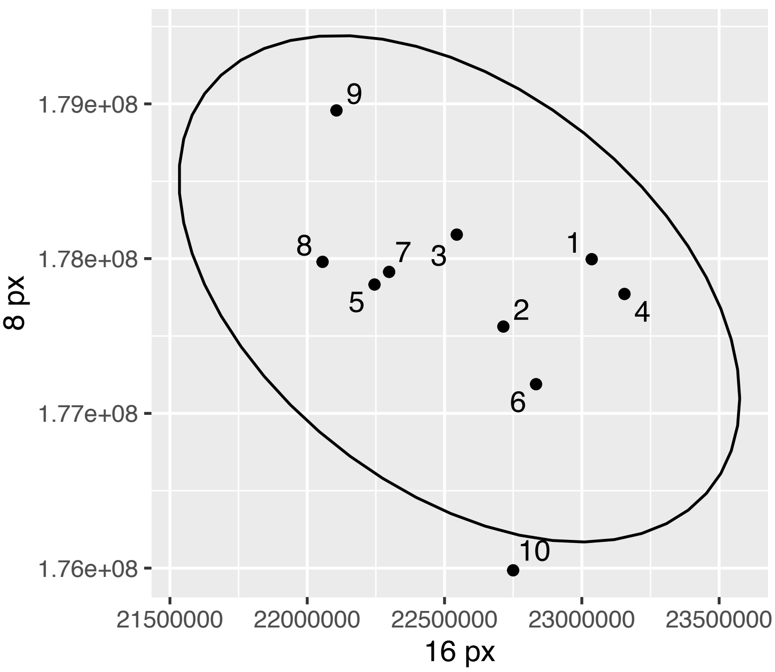

CT glcm autocorrelation

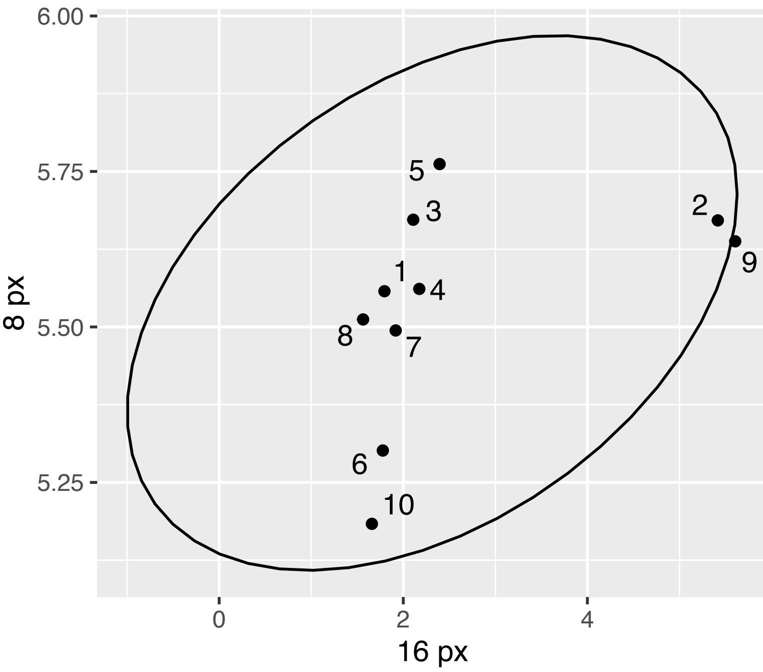

CT firstorder uniformity

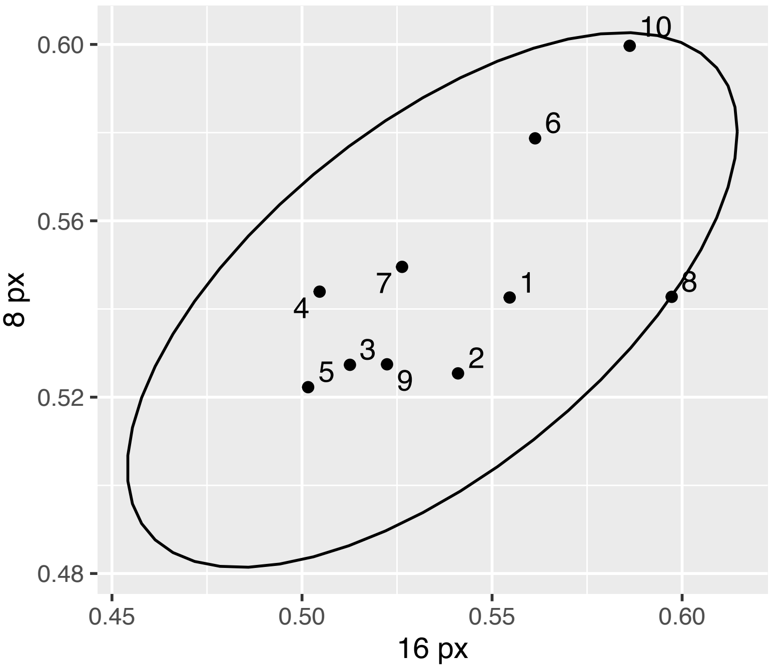

CT glcm clusterprominence

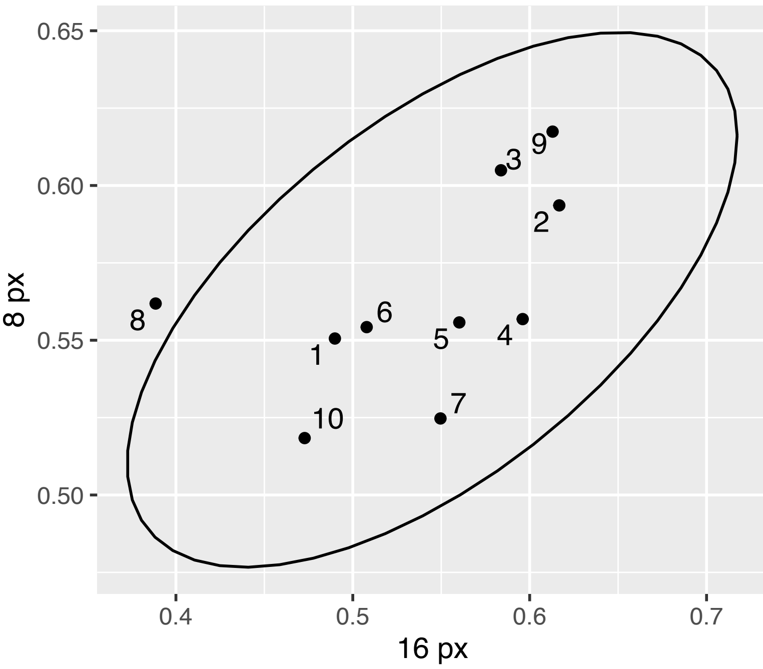

CT firstorder variance

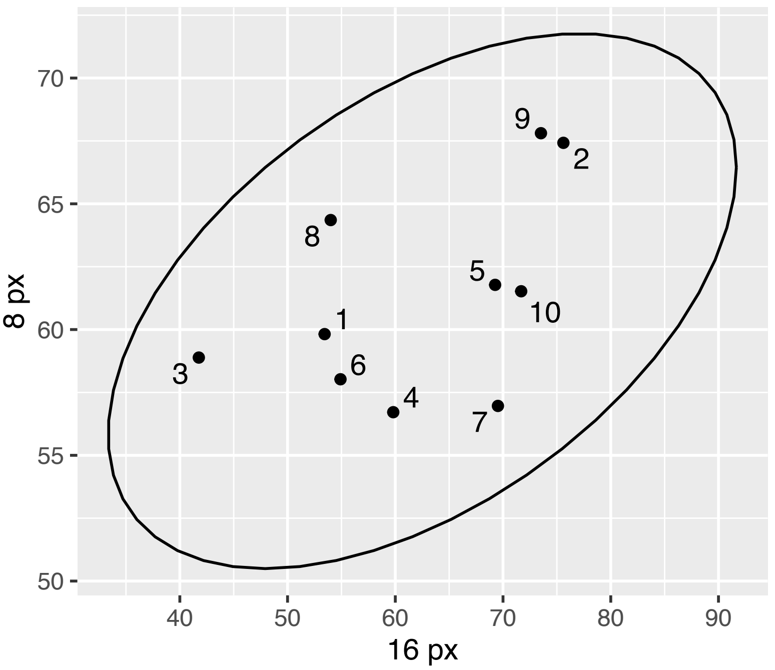

CT glcm clustershade

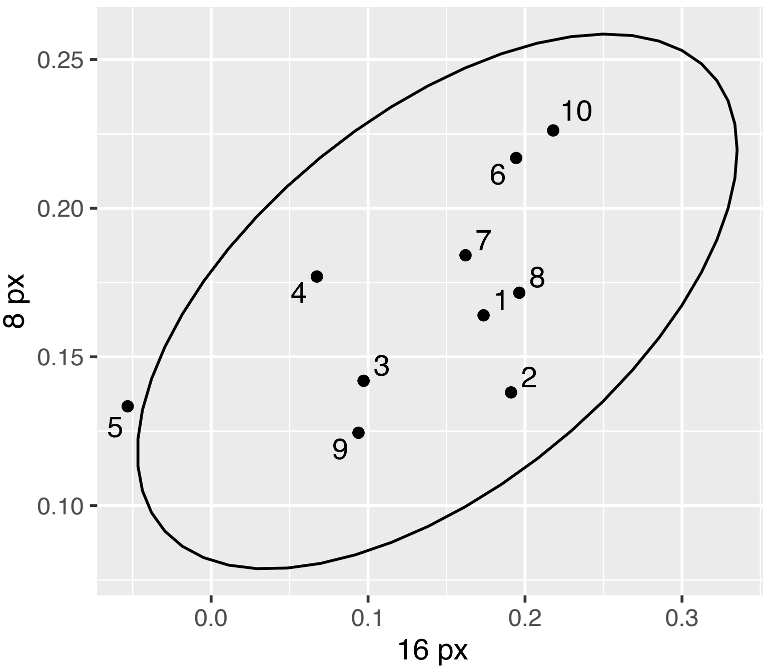

CT glcm clustertendency

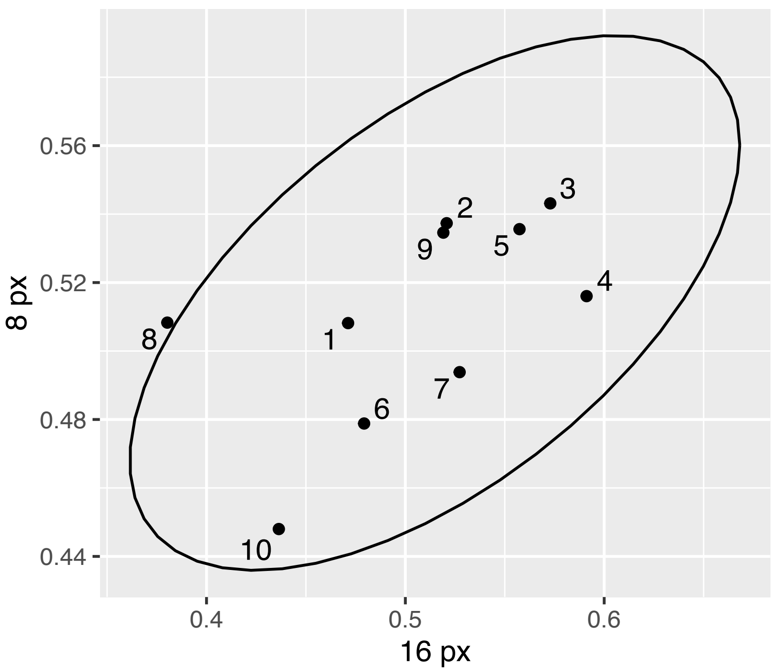

CT glcm differenceaverage

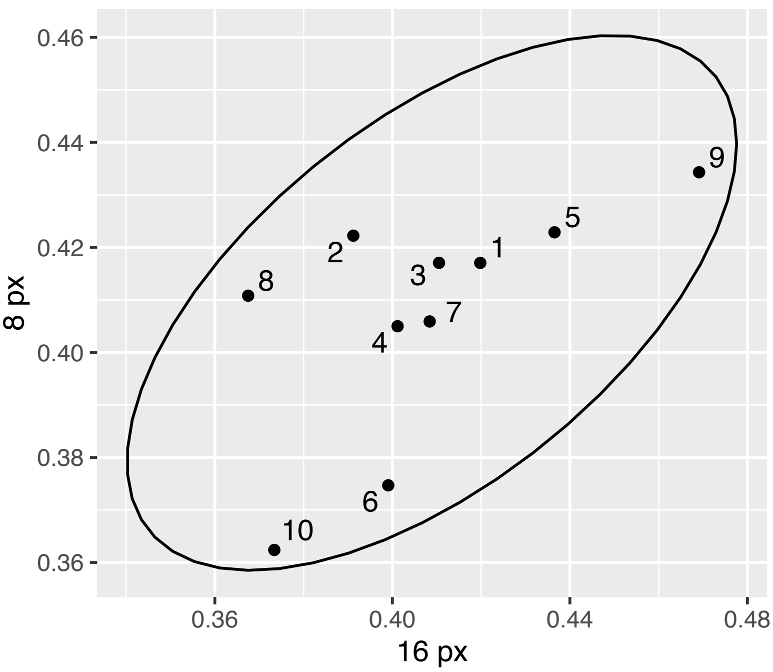

CT glcm contrast

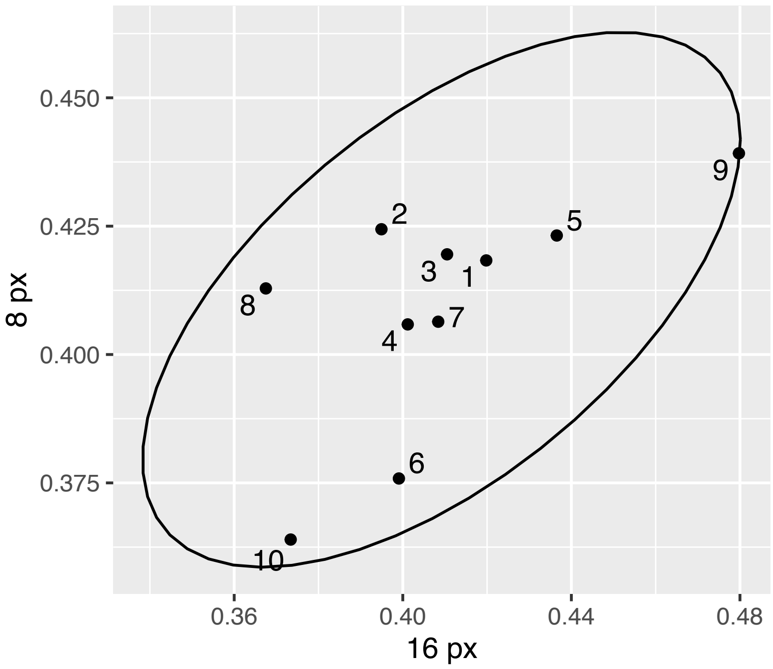

CT glcm differenceentropy

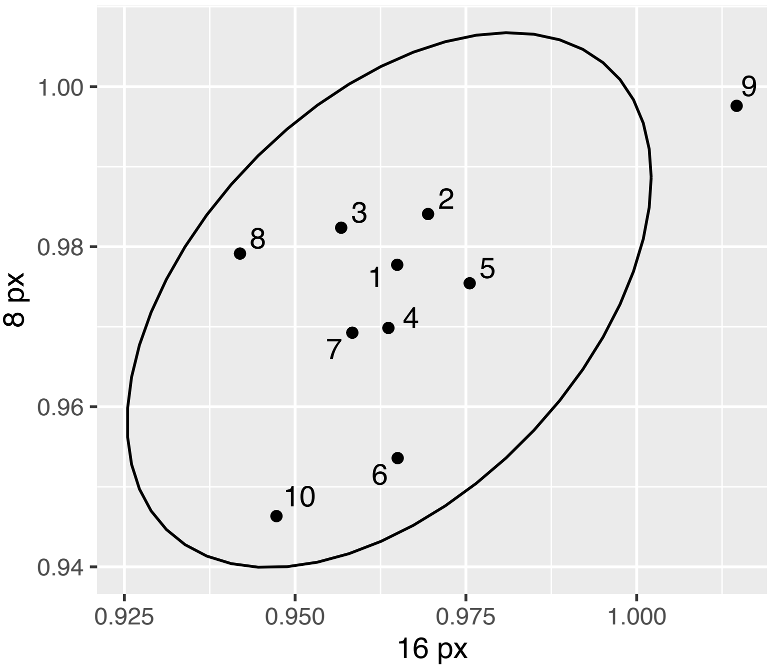

CT glcm correlation

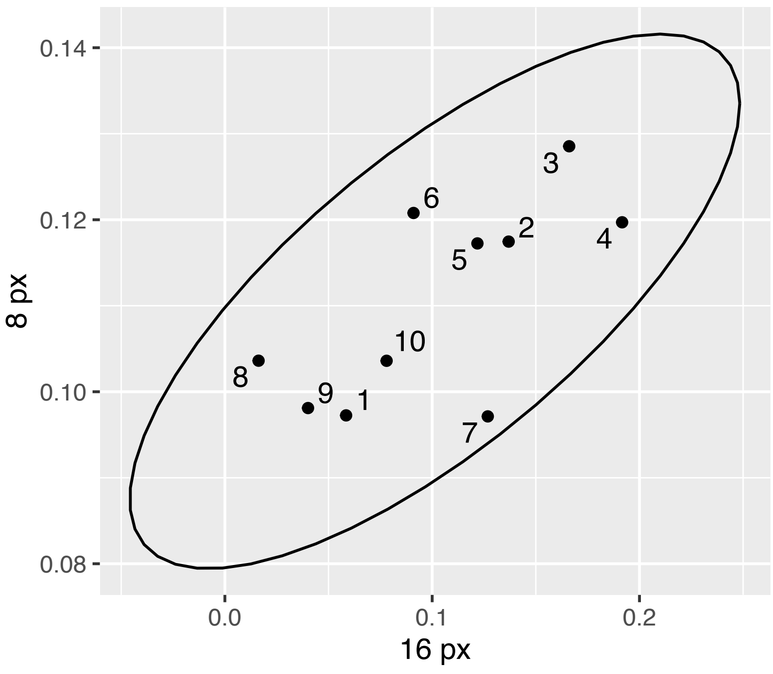

CT glcm differencevariance

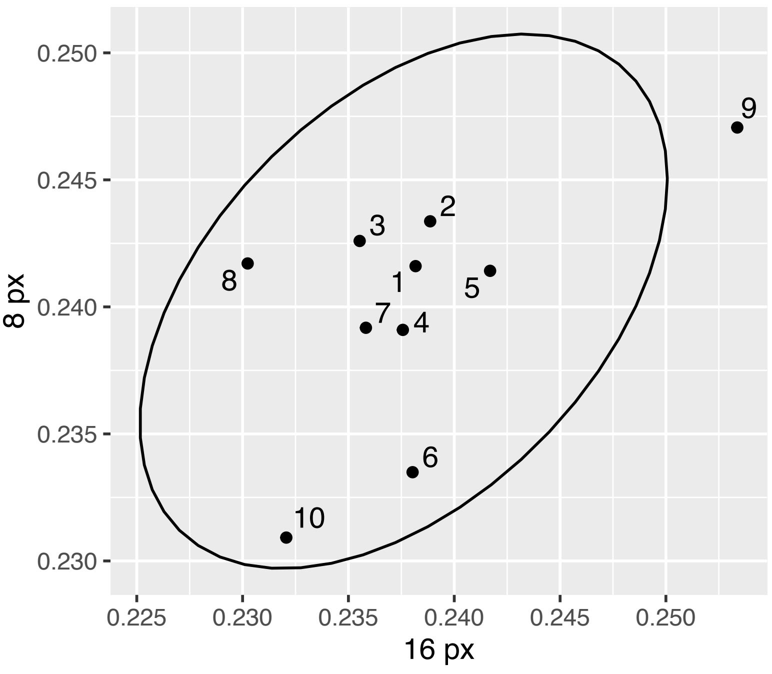

CT glcm id

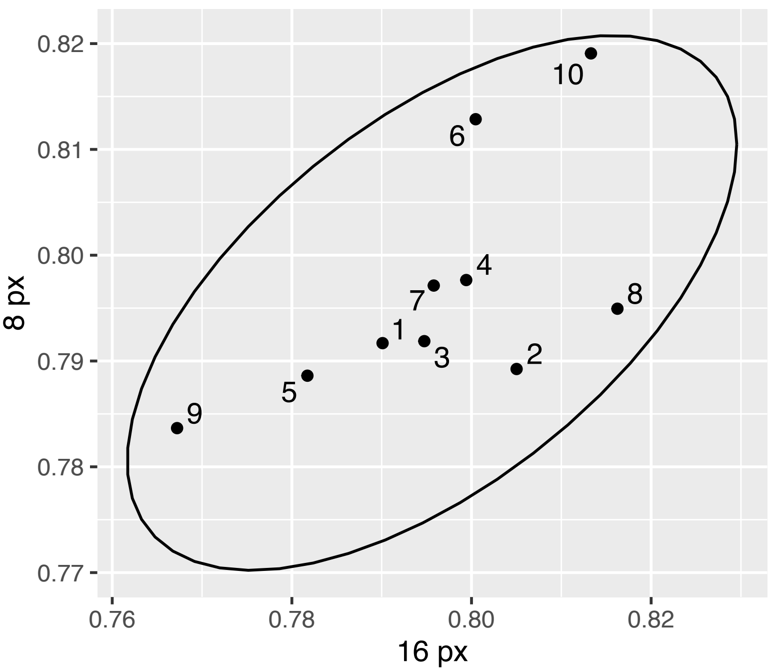

CT glcm idn

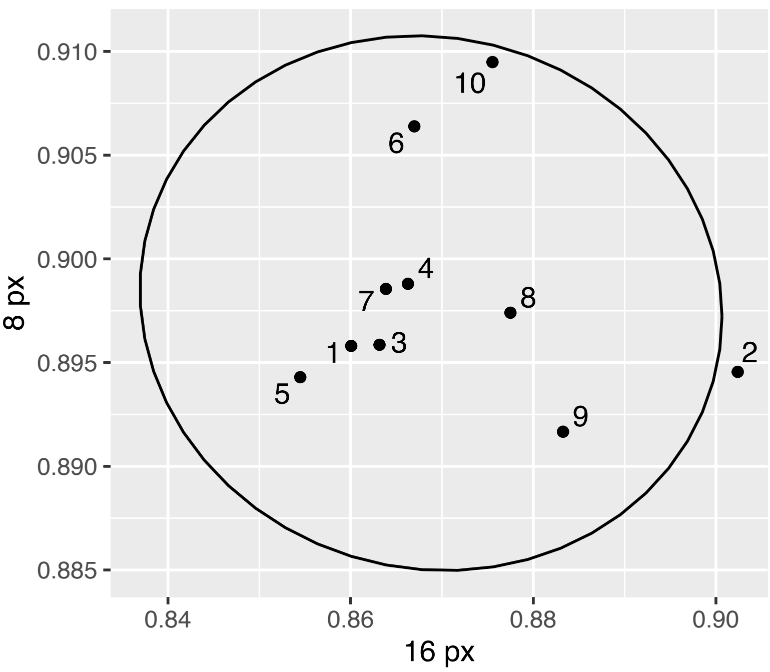

CT glcm idm

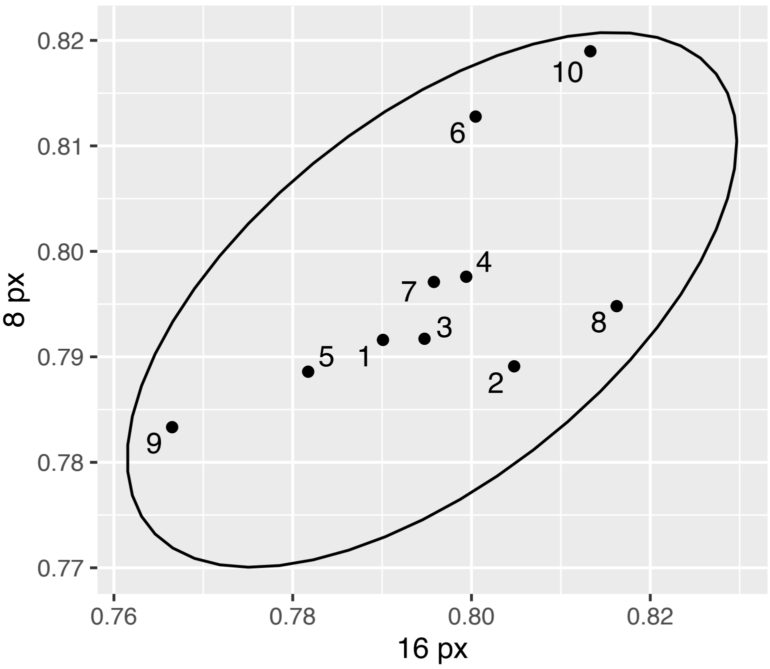

CT glcm imc1

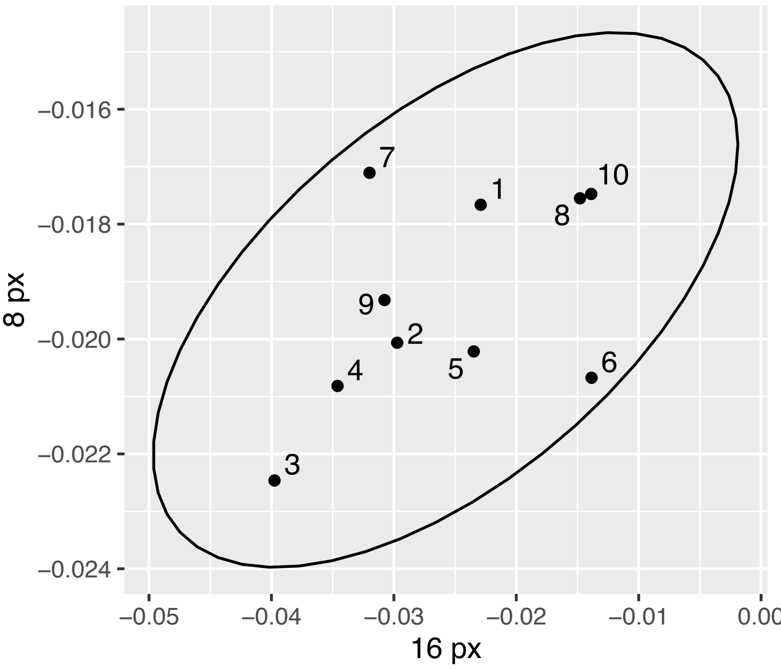

CT glcm idmn

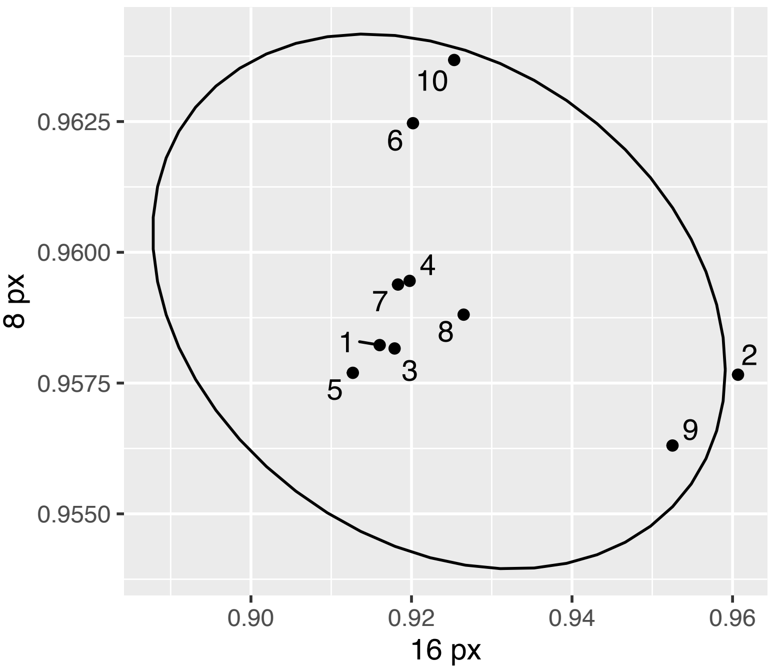

CT glcm imc2

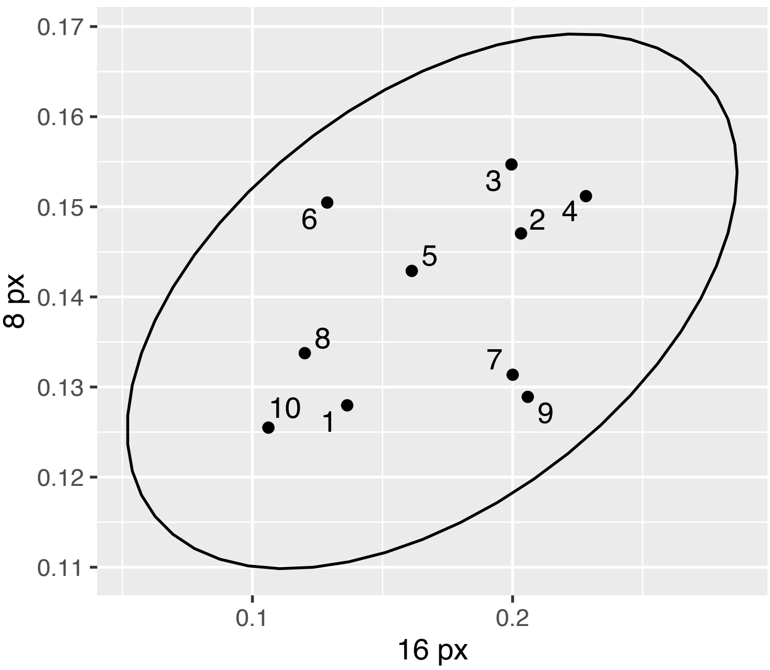

CT glcm inversevariance

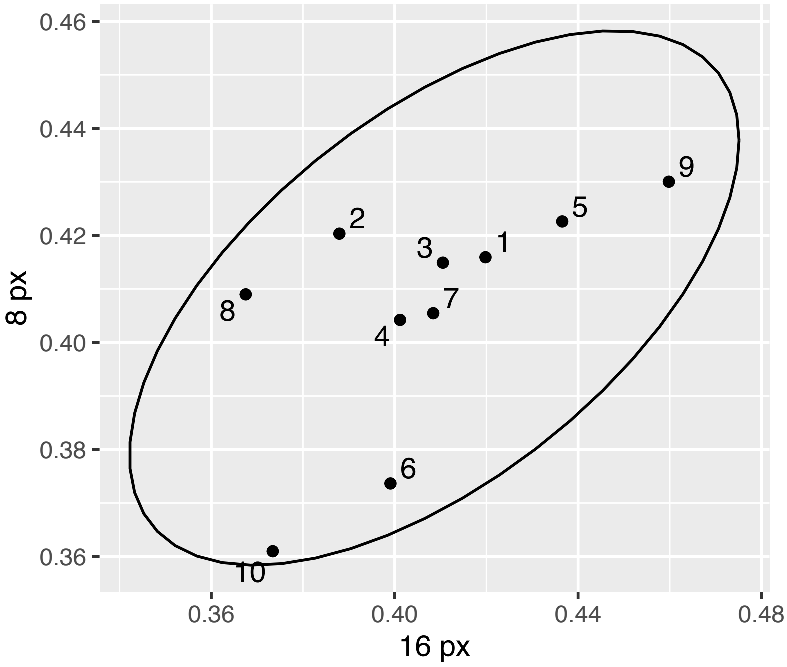

CT glcm jointentropy

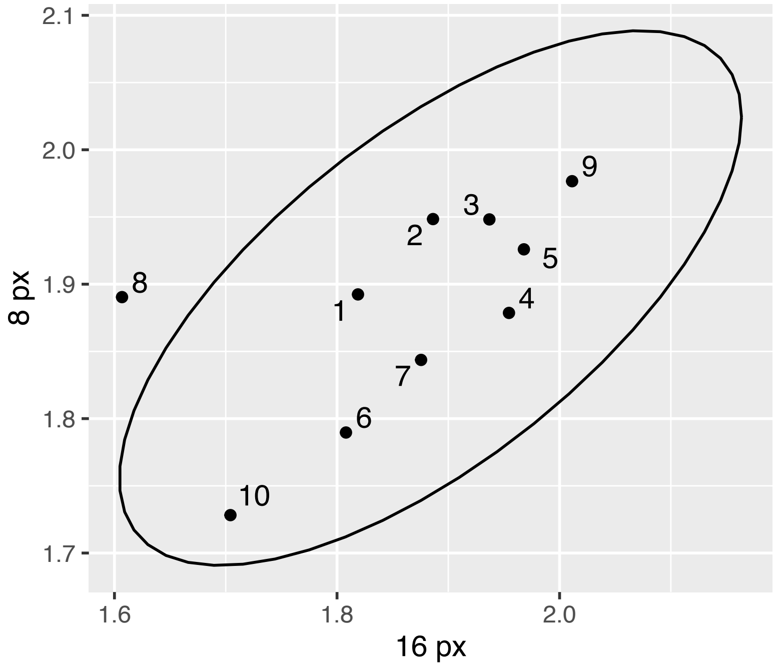

CT glcm jointaverage

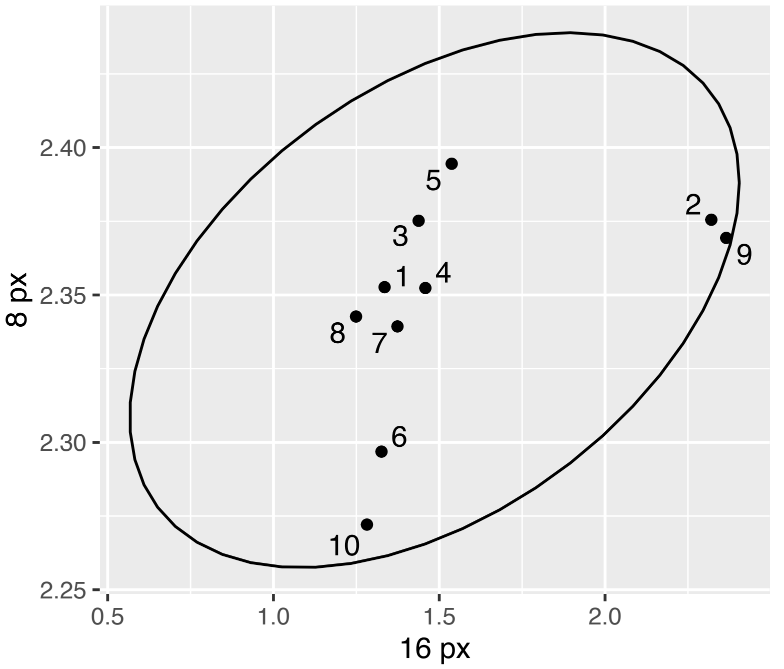

CT glcm mcc

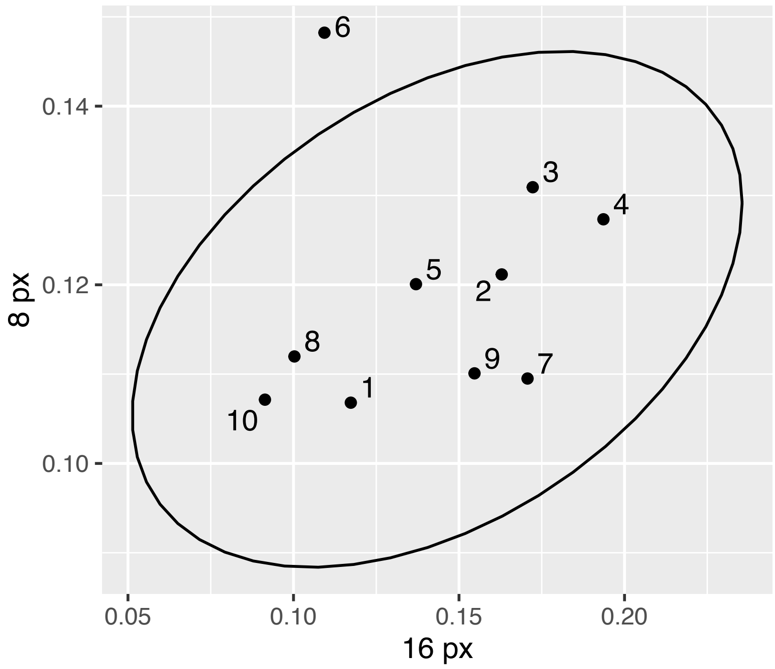

CT glcm jointenergy

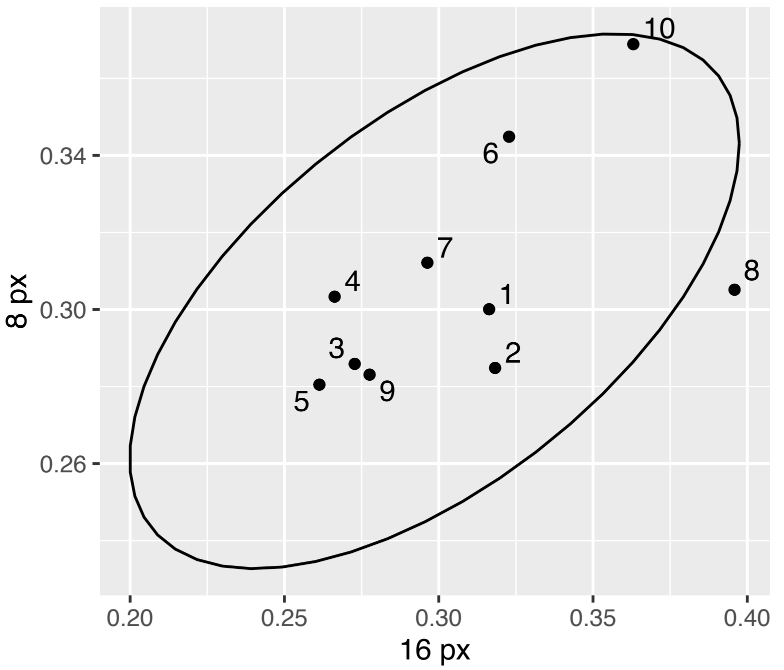

CT glcm maximumprobability

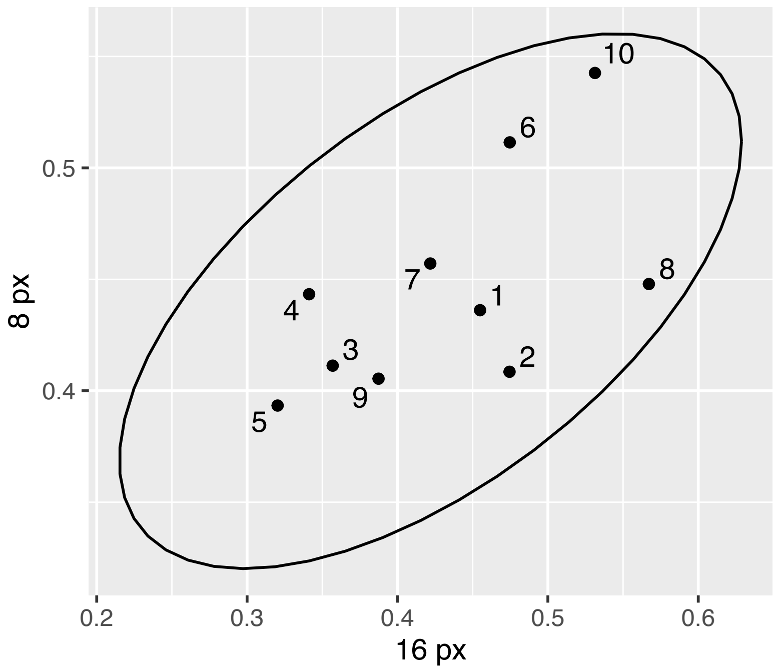

CT glcm sumaverage

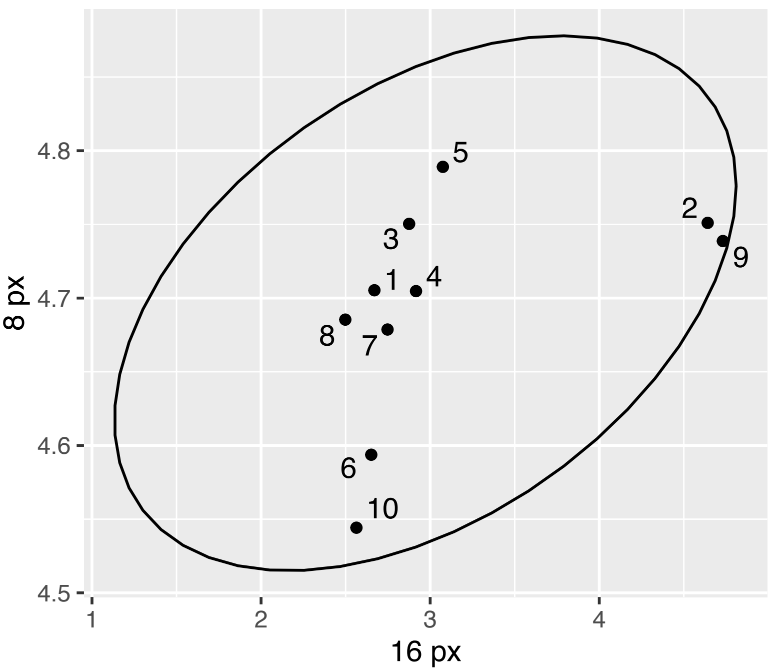

CT glrlm graylevelnonuniformity

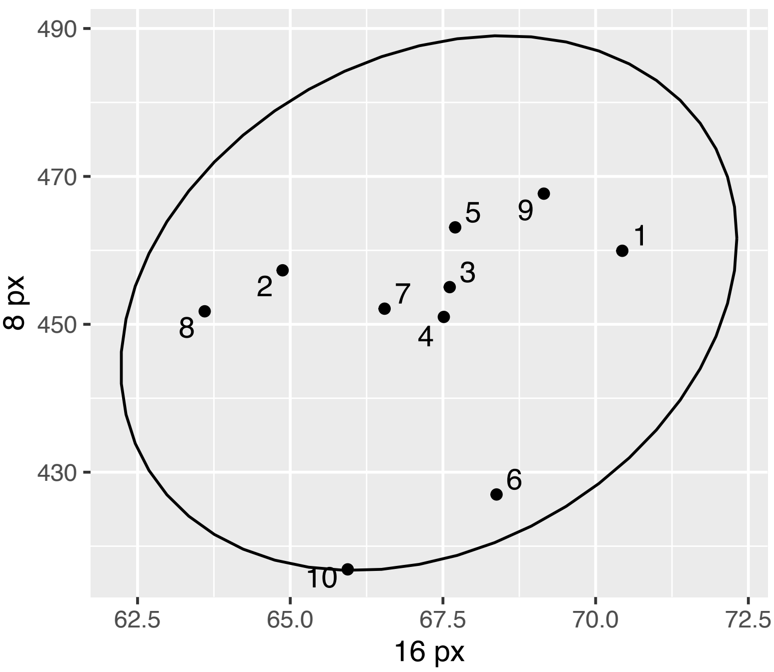

CT glcm sumentropy

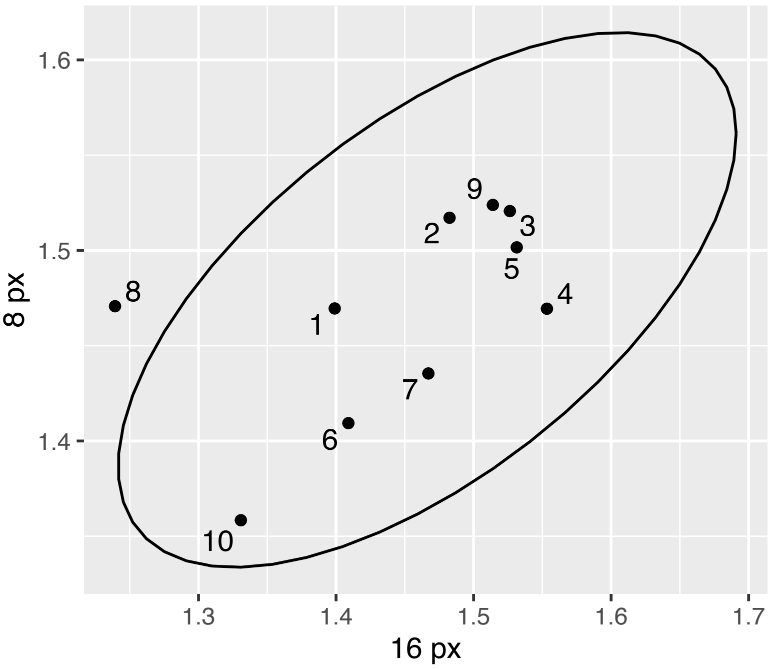

CT glrlm graylevelnonuniformitynormalized

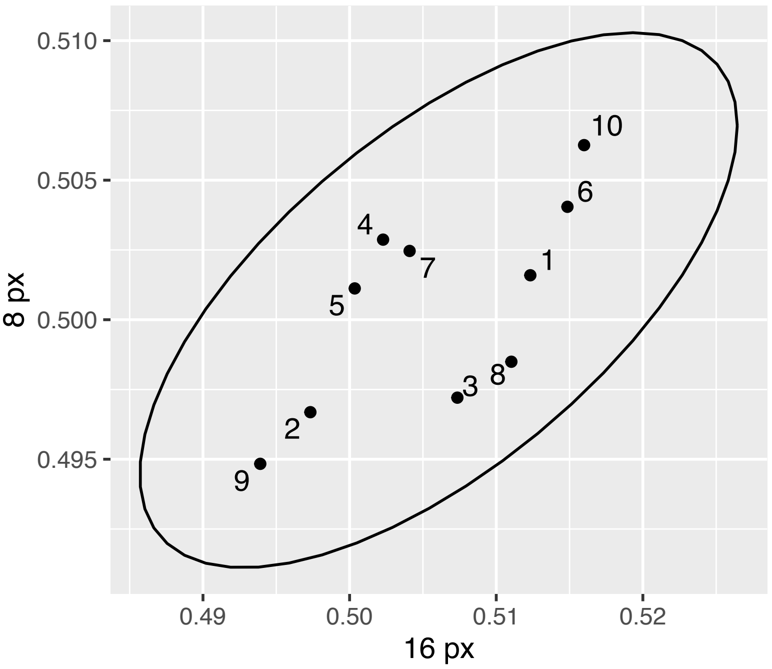

CT glcm sumsquares

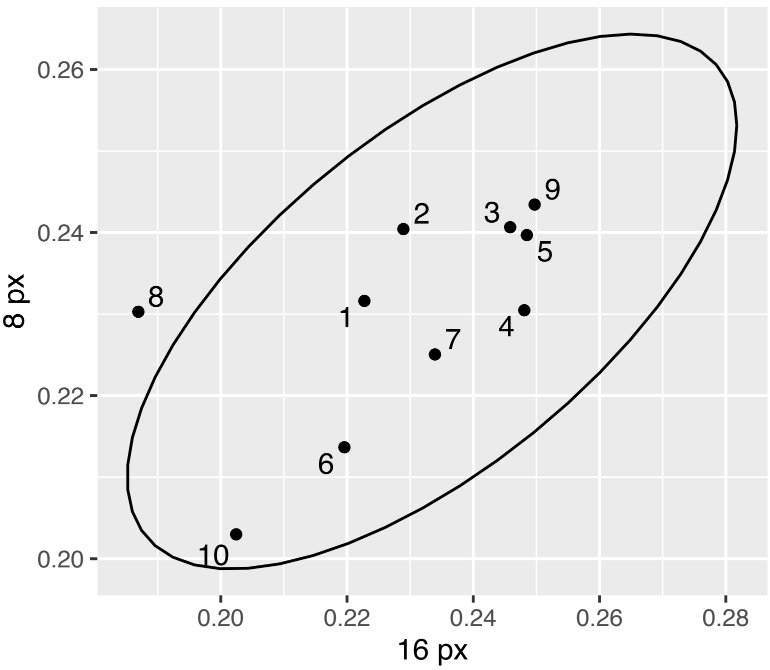

CT glrlm graylevelvariance

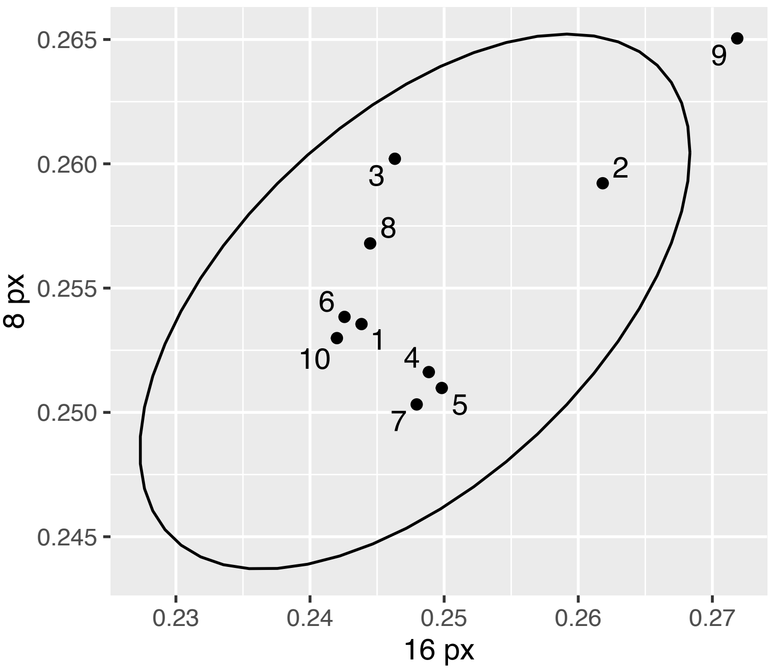

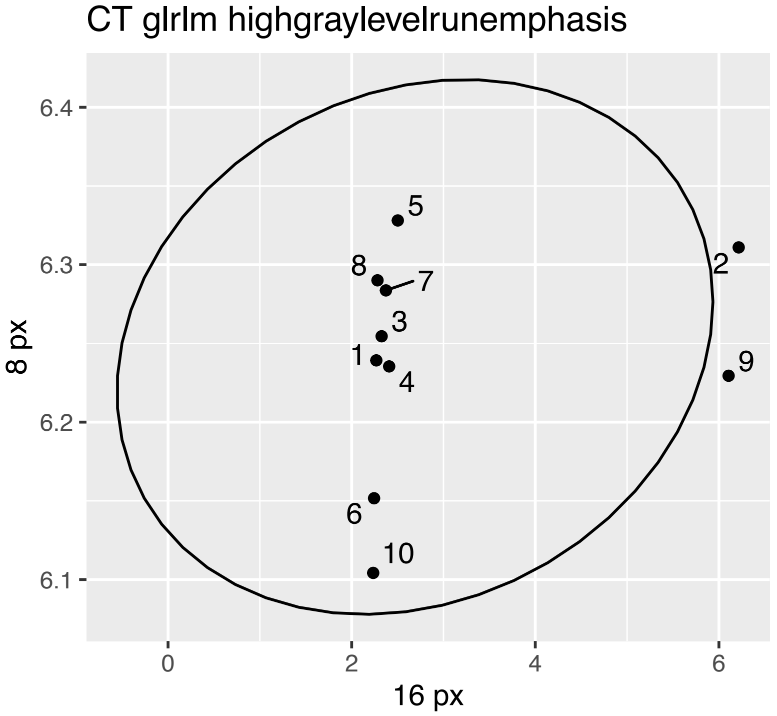
CT glrlm highgraylevelrunemphasis
8 px
16 px
6.4
6.3
6.2
6.1
0
2
4
6
1
2
3
4
5
6
7
8
9
10

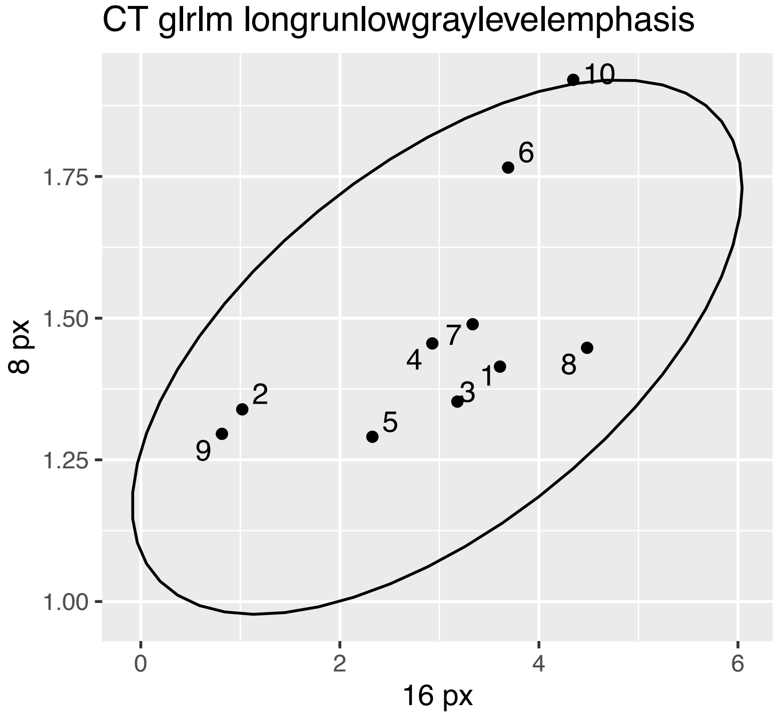
CT glrlm longrunlowgraylevelemphasis
8 px
16 px
1.75
1.50
1.25
1.00
0
2
4
6
1
2
3
4
5
6
7
8
9
10

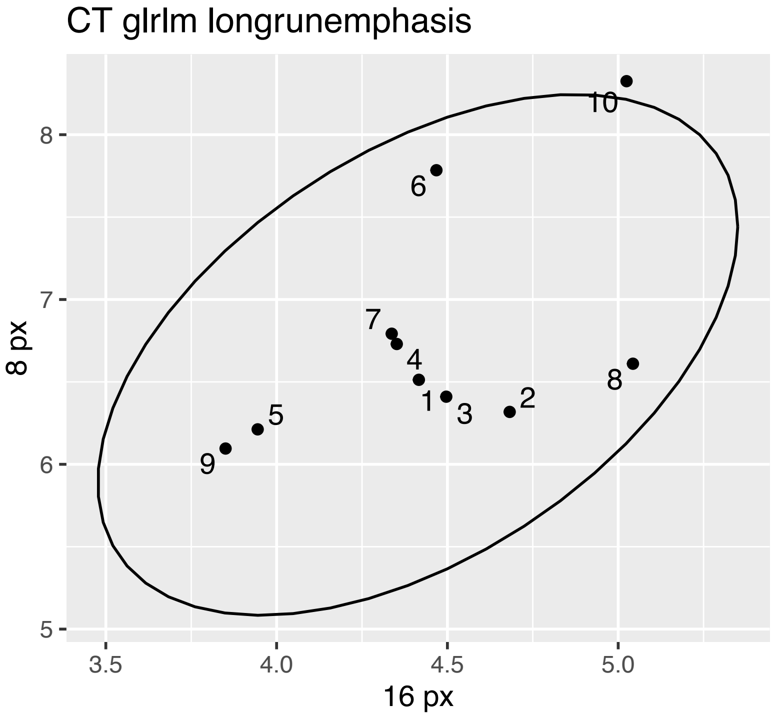
CT glrlm longrunemphasis
8 px
16 px
8
7
6
5
3.5
4.0
4.5
5.0
1
2
3
4
5
6
7
8
9
10

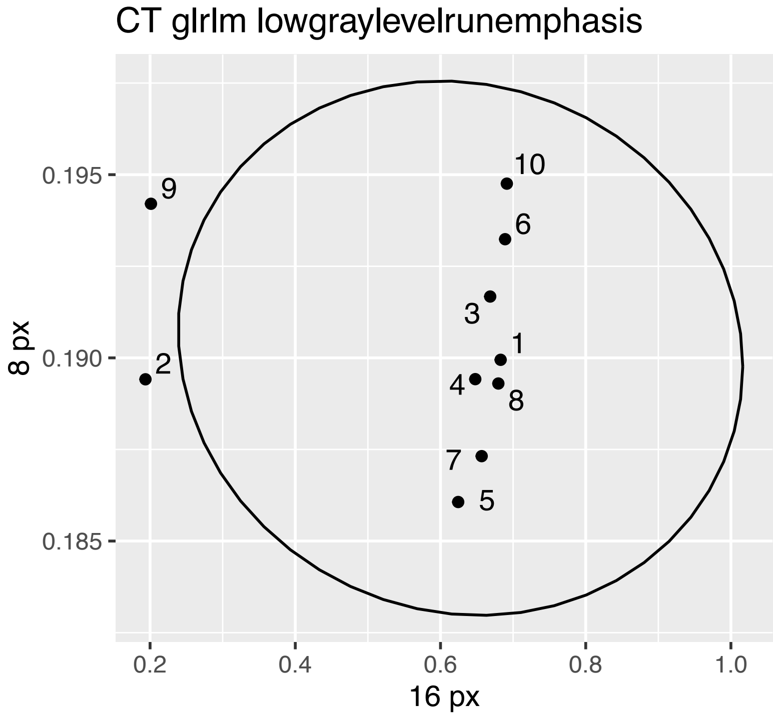
CT glrlm lowgraylevelrunemphasis
8 px
16 px
0.195
0.190
0.185
0.2
0.4
0.6
0.8
1.0
1
2
3
4
5
6
7
8
9
10

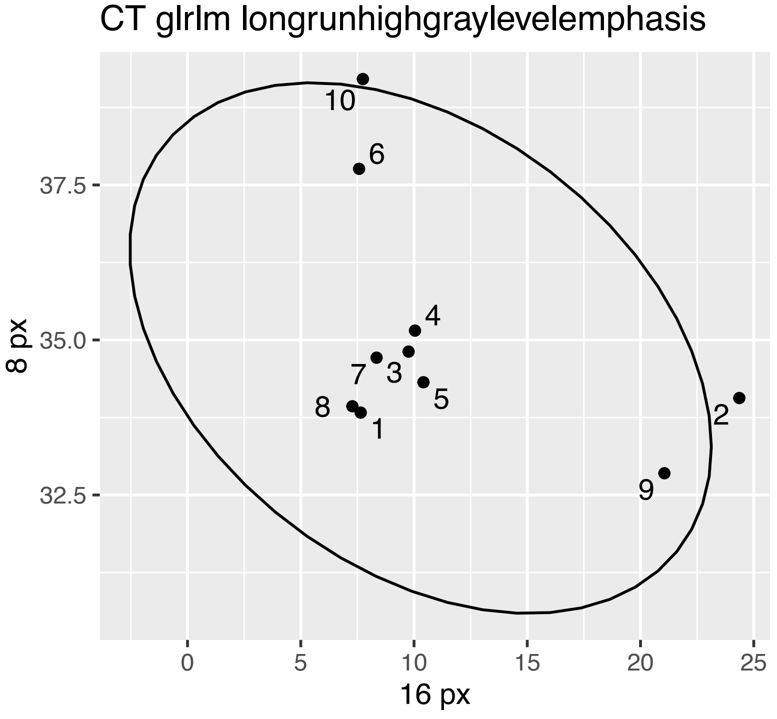
CT glrlm longrunhighgraylevelemphasis
8 px
16 px
37.5
35.0
32.5
0
5
10
15
20
25
1
2
3
4
5
6
7
8
9
10

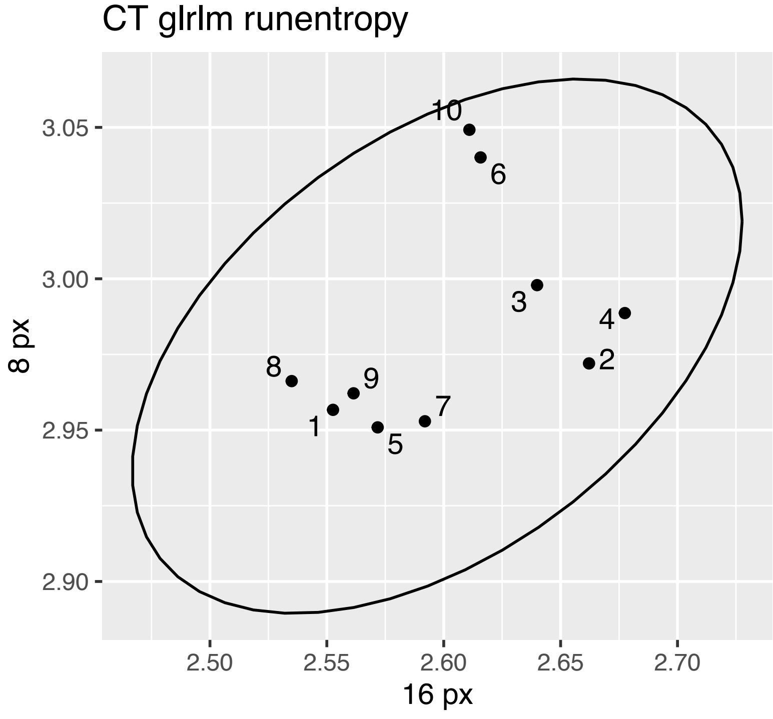
CT glrlm runentropy
8 px
16 px
3.05
3.00
2.95
2.90
2.50
2.55
2.60
2.65
2.70
1
2
3
4
5
6
7
8
9
10

CT glrlm runlengthnonuniformity

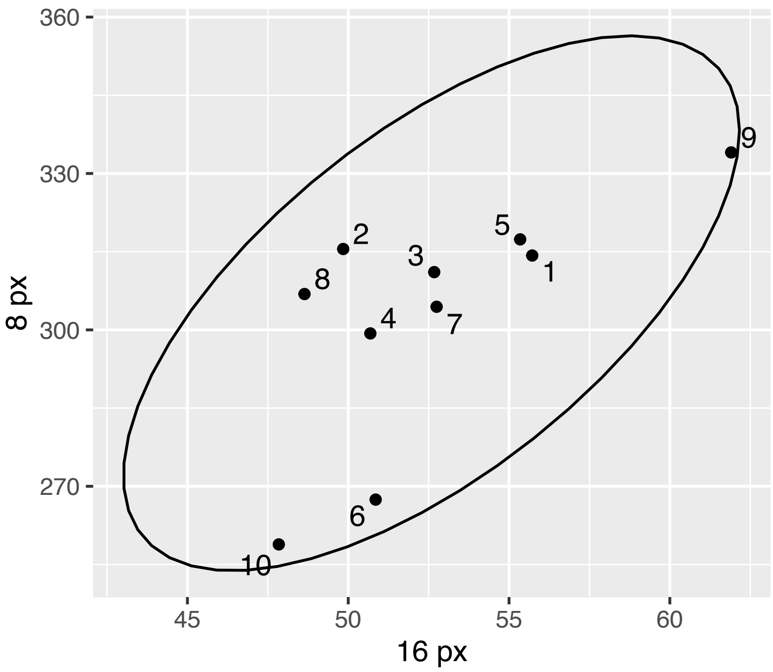

CT glrlm runvariance

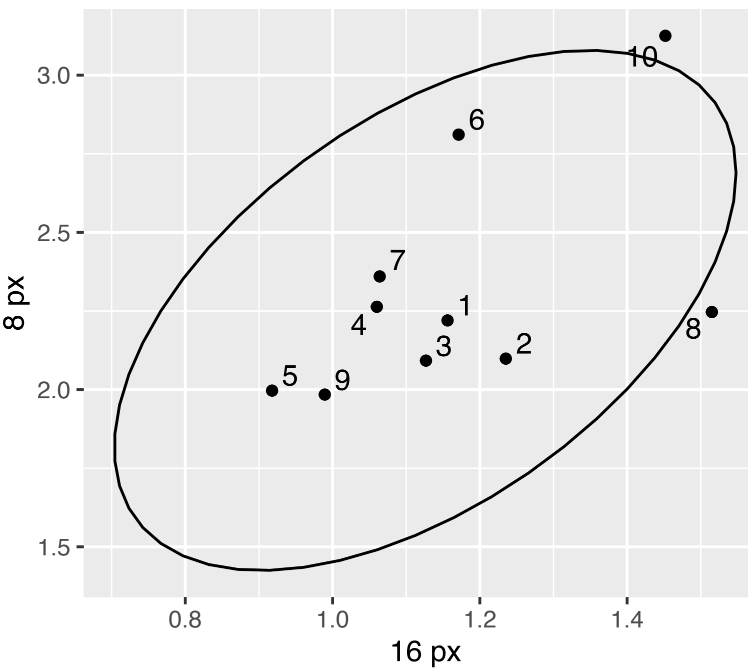

CT glrlm runlengthnonuniformitynormalized

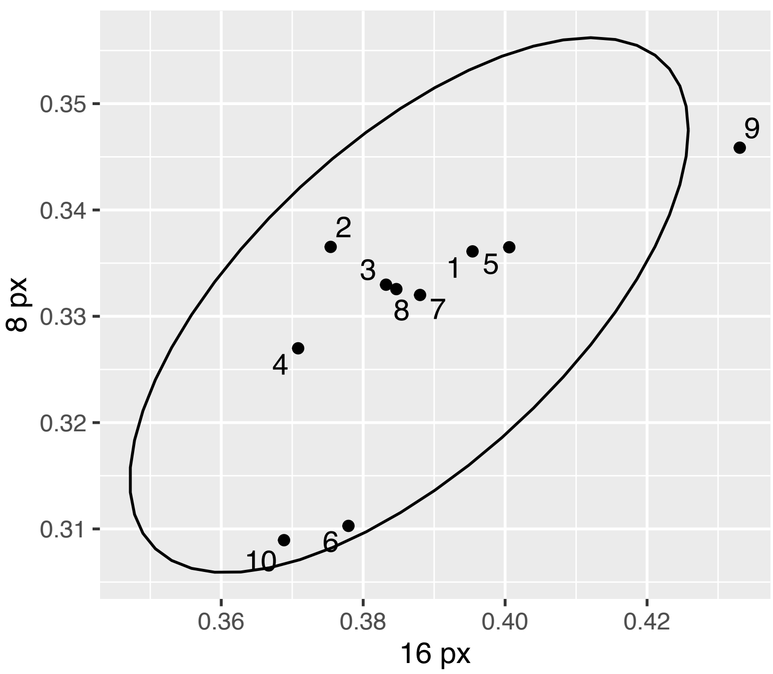

CT glrlm shortrunemphasis

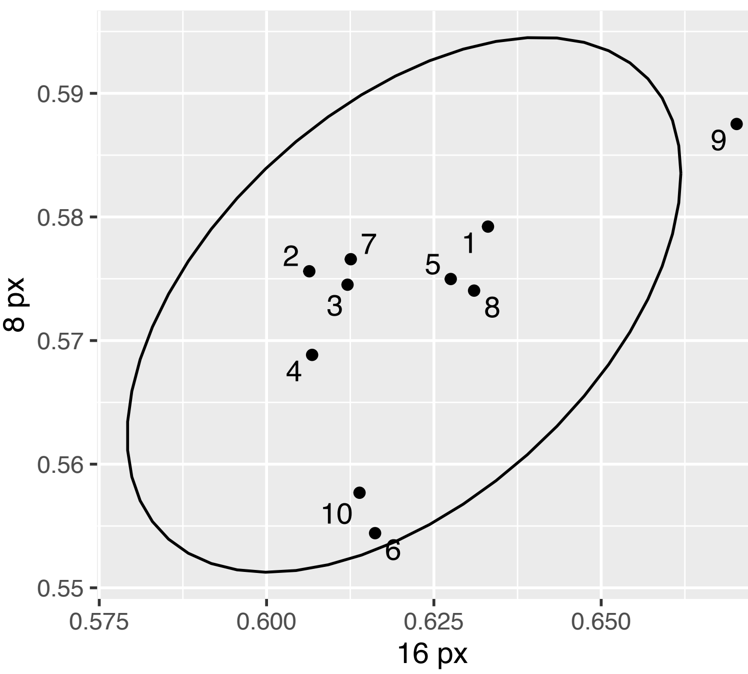

CT glrlm runpercentage

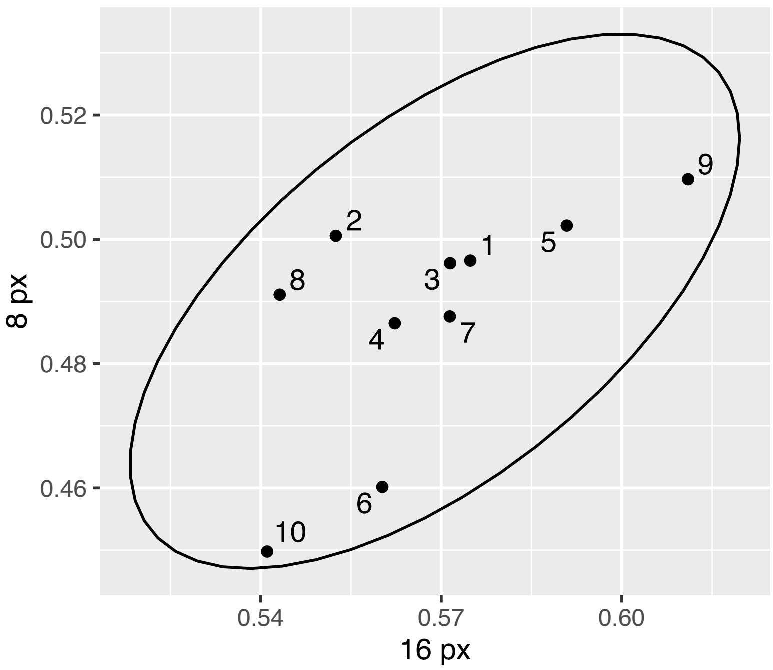

CT glrlm shortrunhighgraylevelemphasis

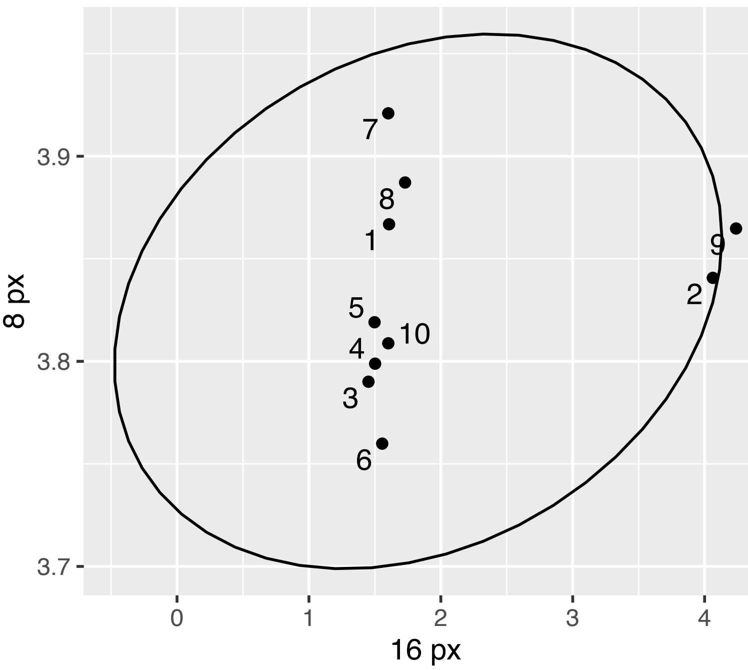

CT glrlm shortrunlowgraylevelemphasis

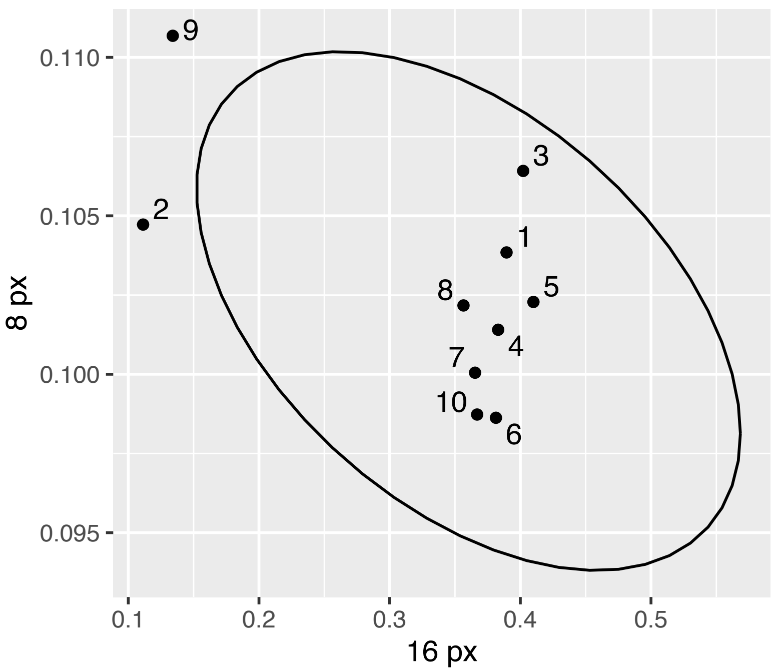

CT glszm graylevelvariance

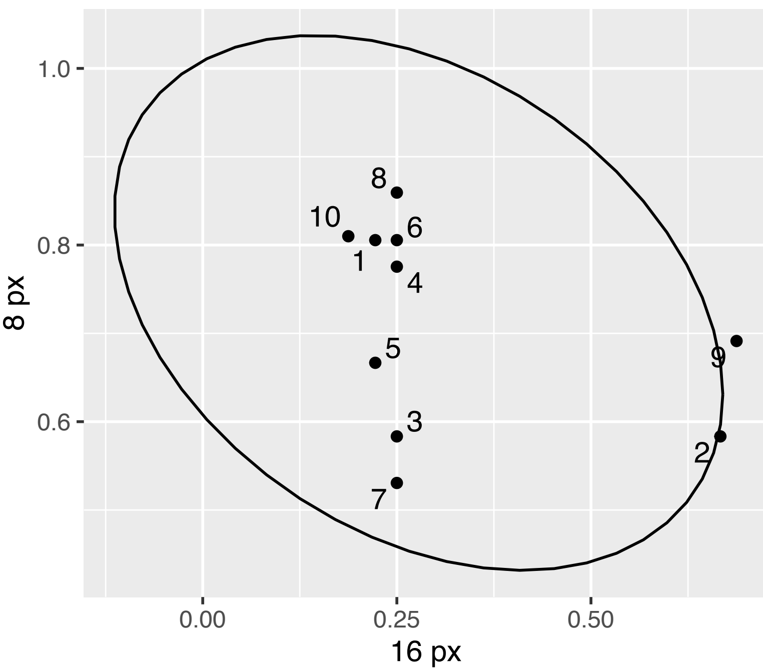

CT glszm graylevelnonuniformity

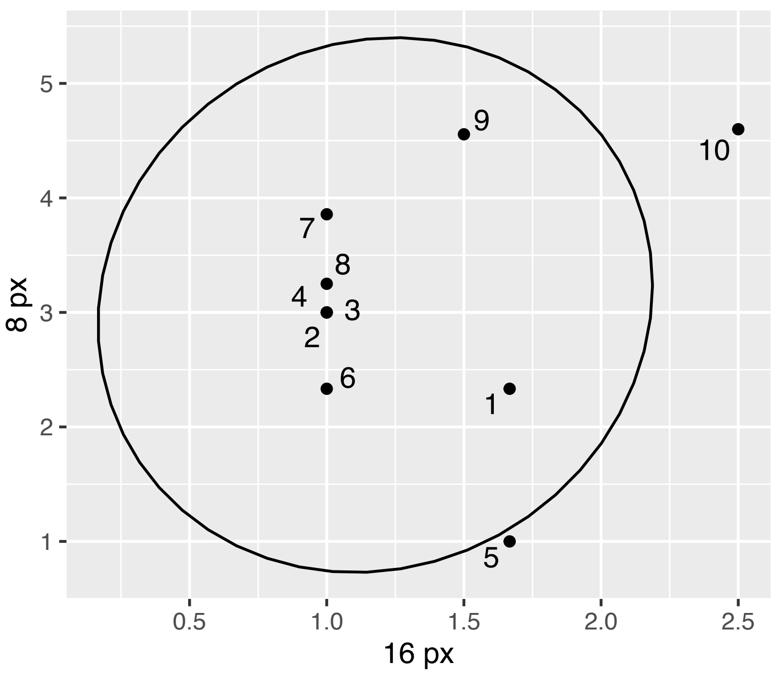

CT glszm highgraylevelzoneemphasis

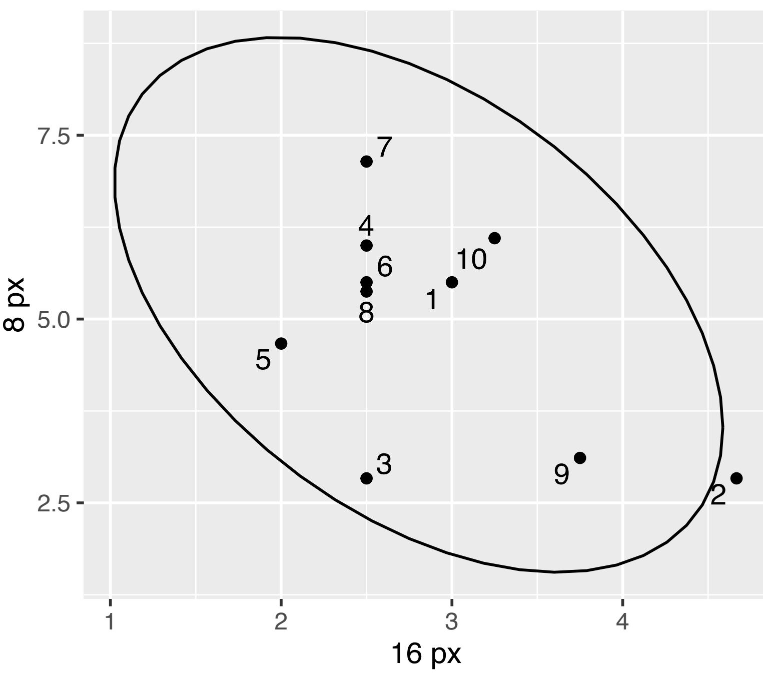

CT glszm graylevelnonuniformitynormalized

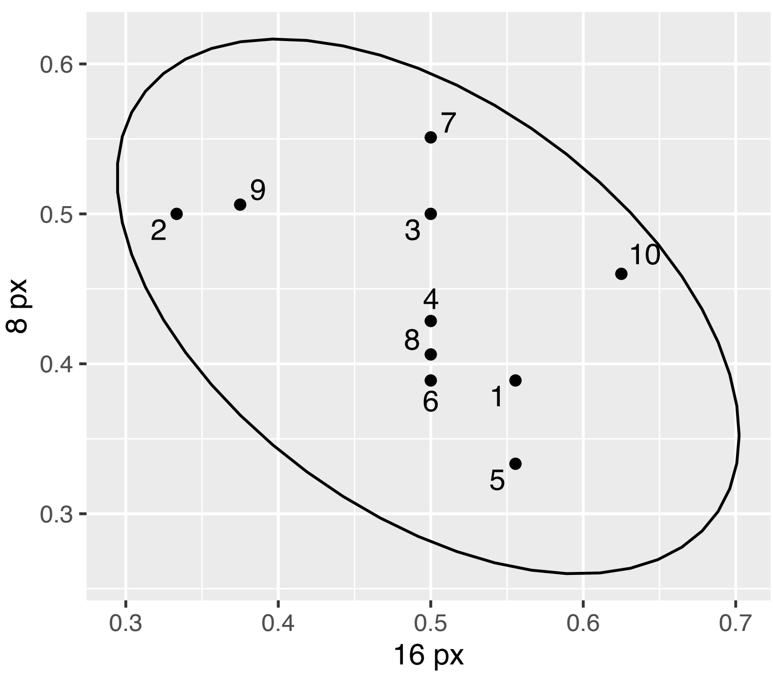

CT glszm largeareaemphasis

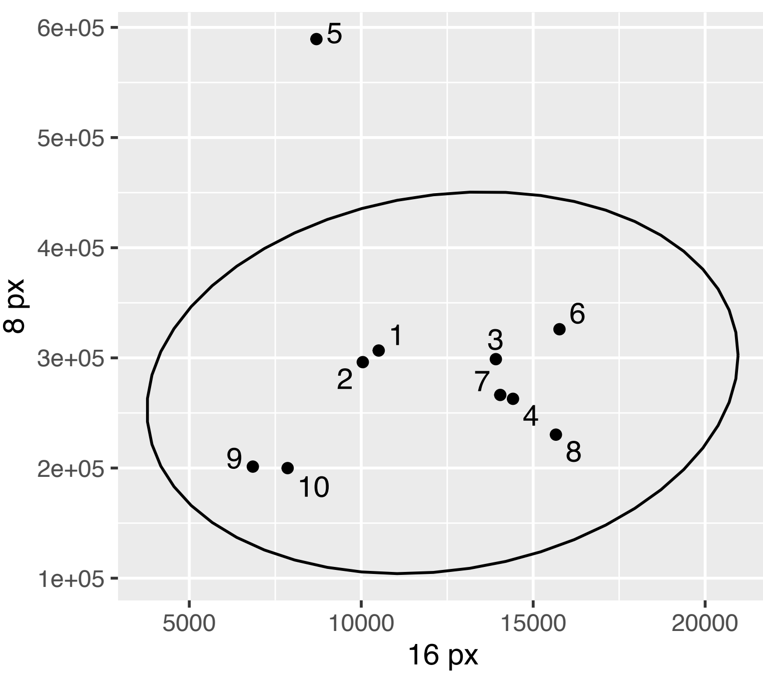

CT glszm largeareahighgraylevelemphasis

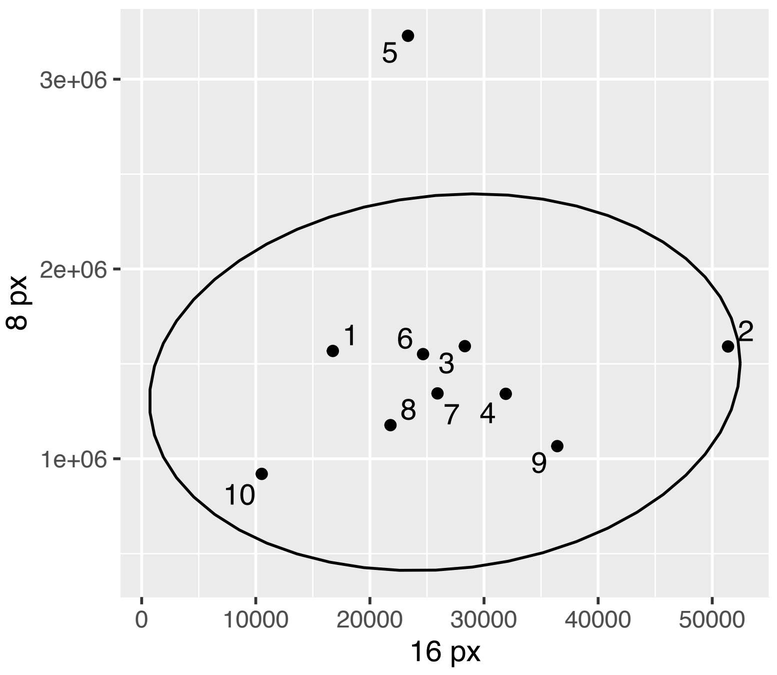

CT glszm sizezonenonuniformity

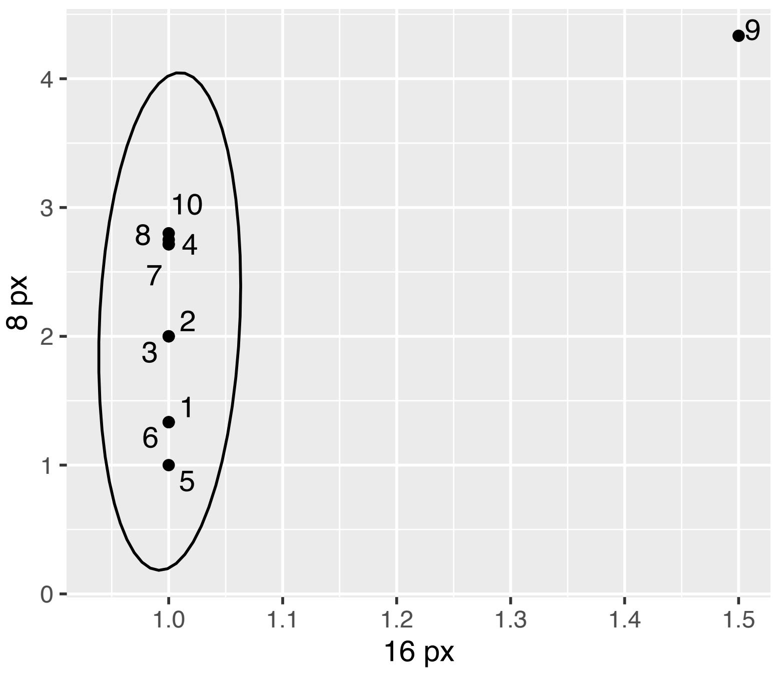

CT glszm largearealowgraylevelemphasis

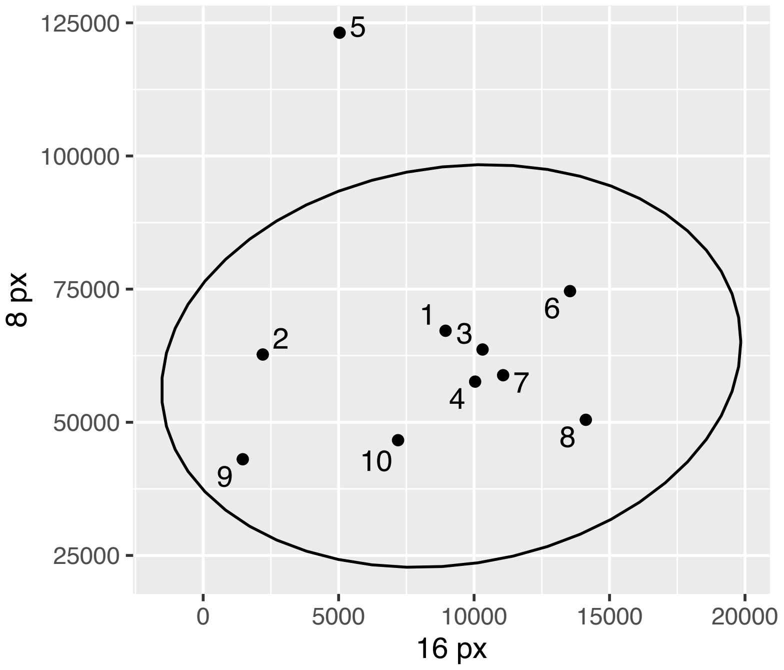

CT glszm sizezonenonuniformitynormalized

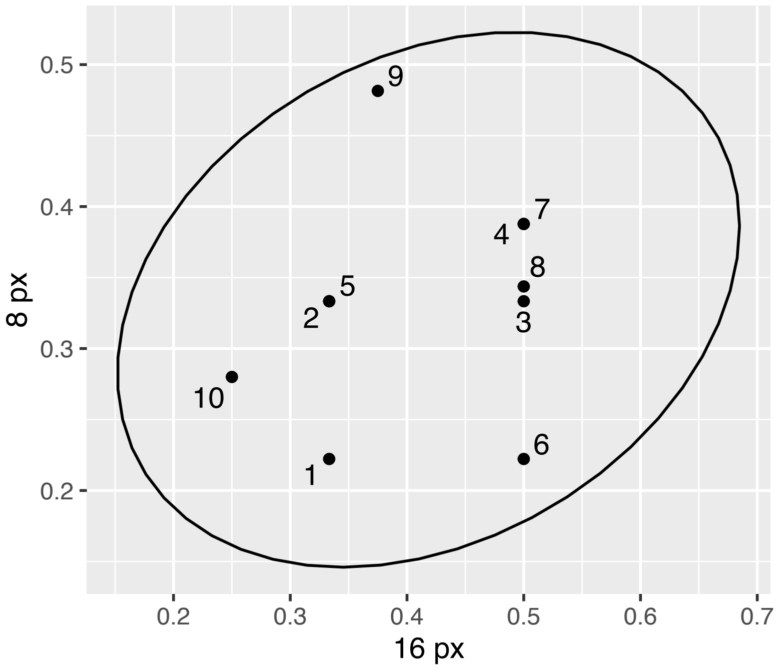

CT glszm lowgraylevelzoneemphasis

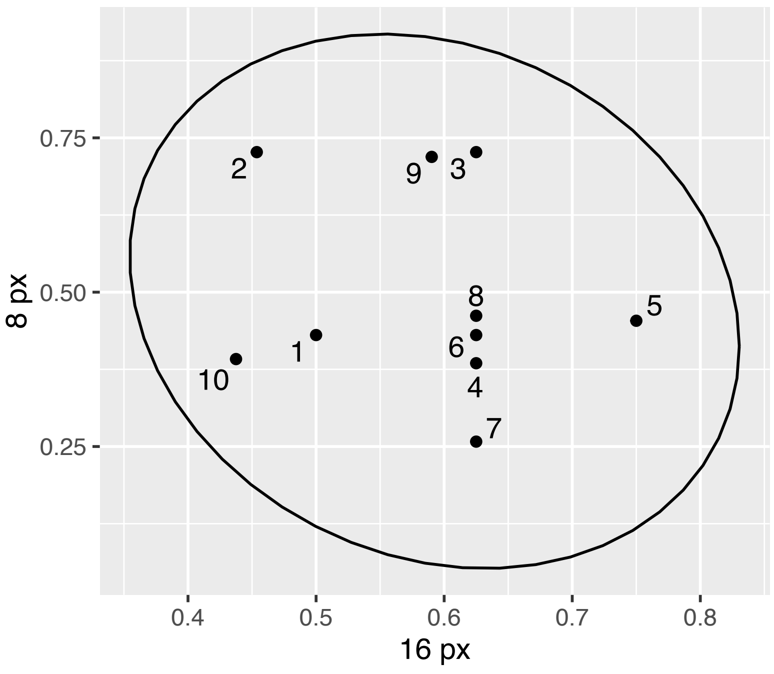

CT glszm smallareaemphasis

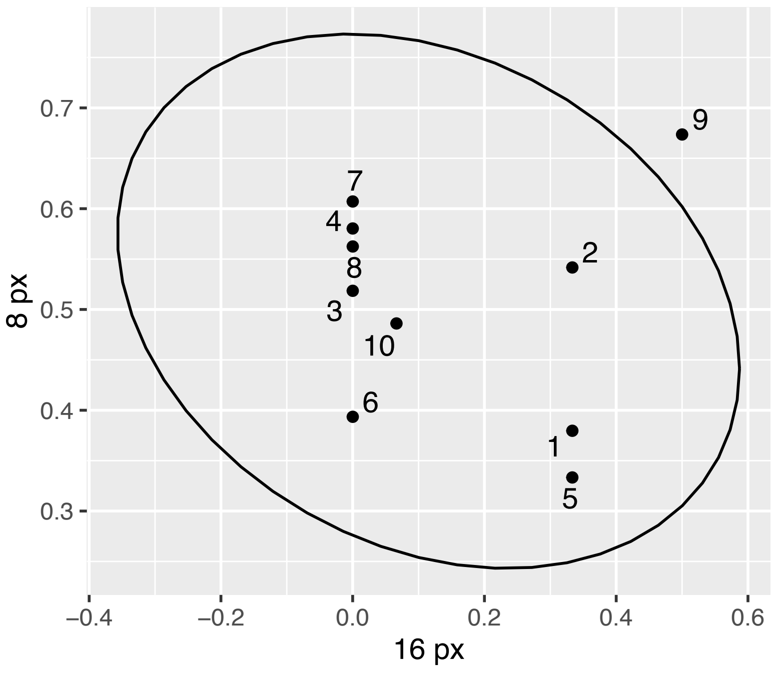

CT glszm smallareahighgraylevelemphasis

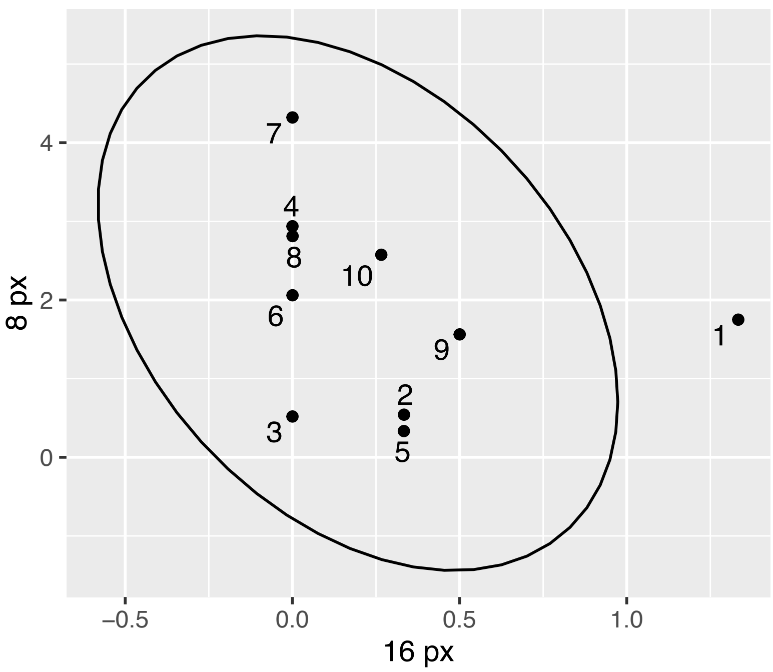

CT glszm zonepercentage

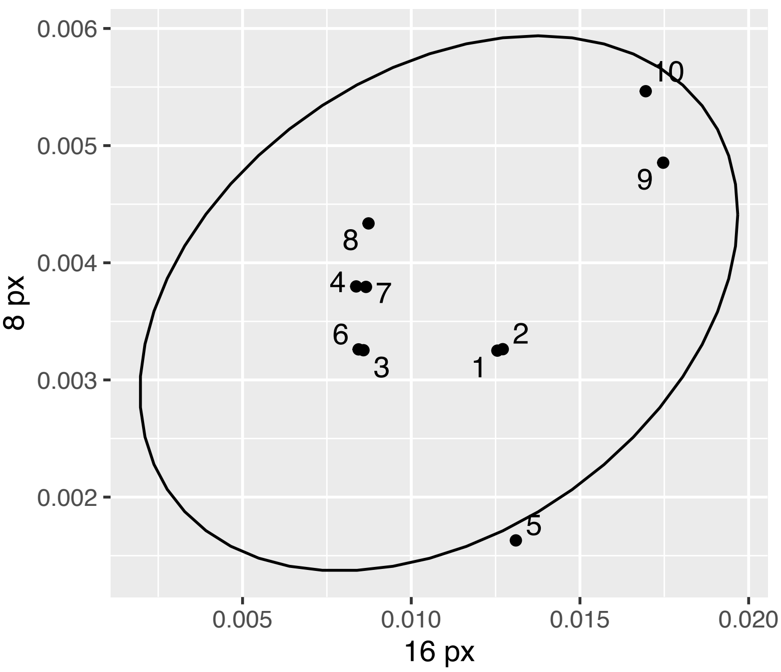

CT glszm smallarealowgraylevelemphasis

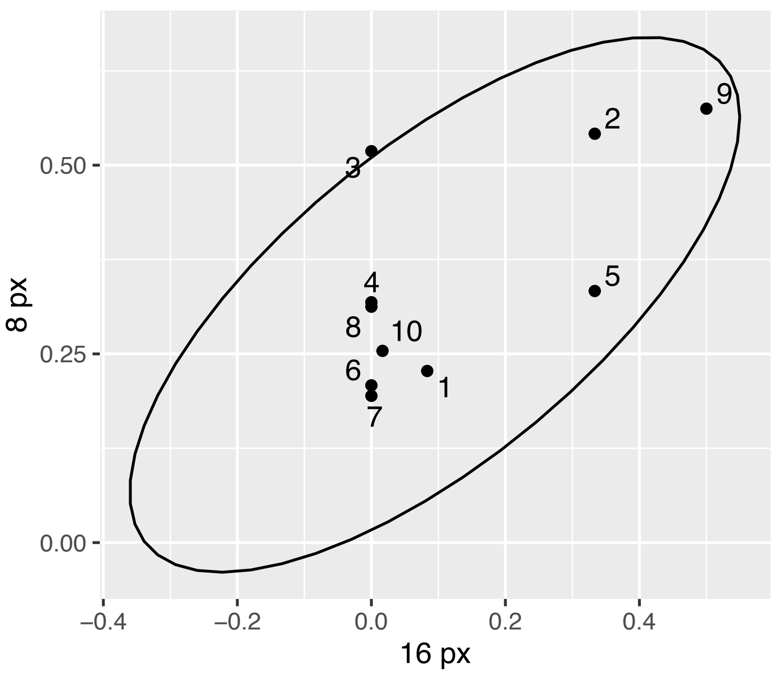

CT glszm zonevariance

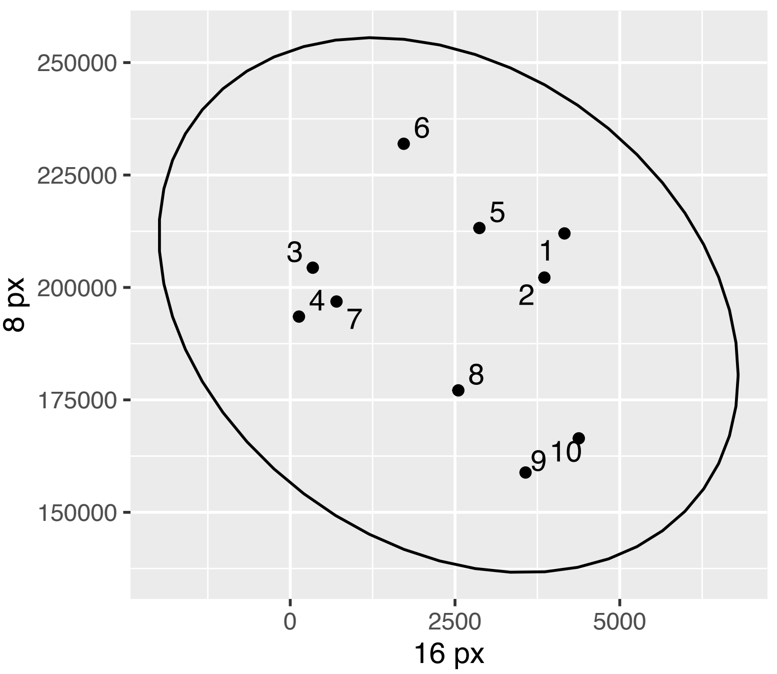

CT glszm zoneentropy

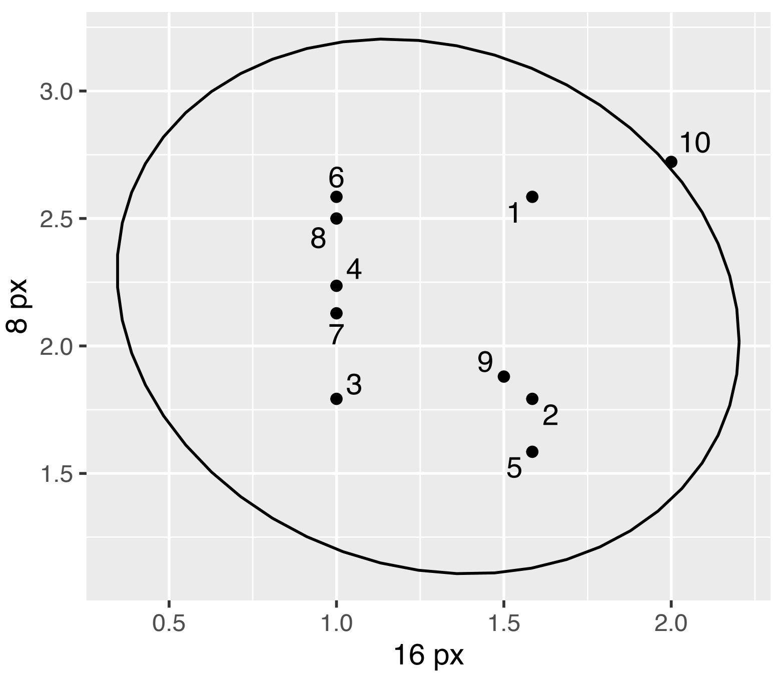

CT gldm dependenceentropy

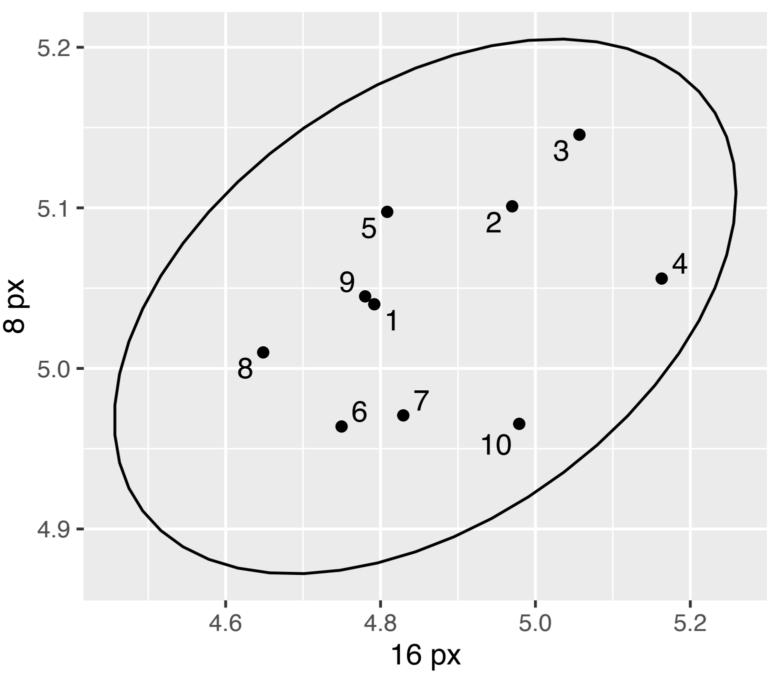

CT gldm dependencenonuniformity

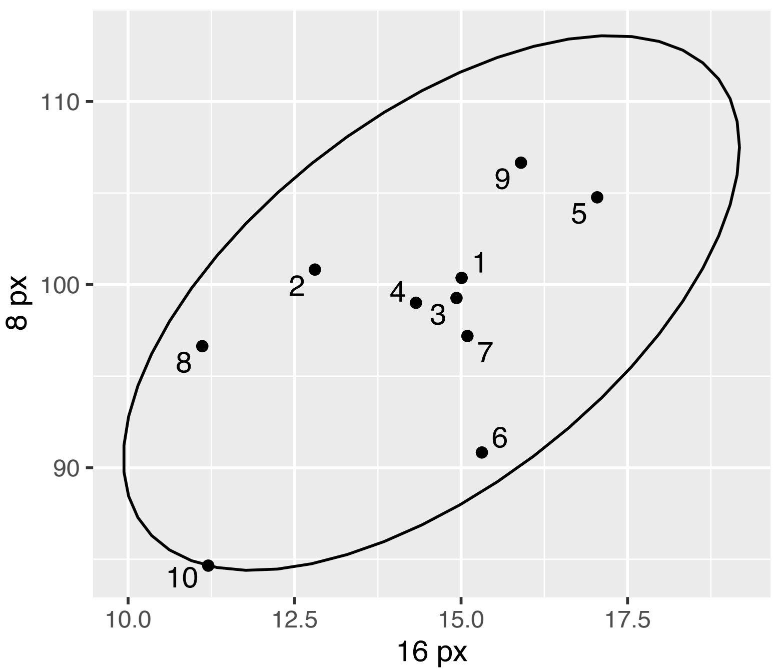

CT gldm graylevelnonuniformity

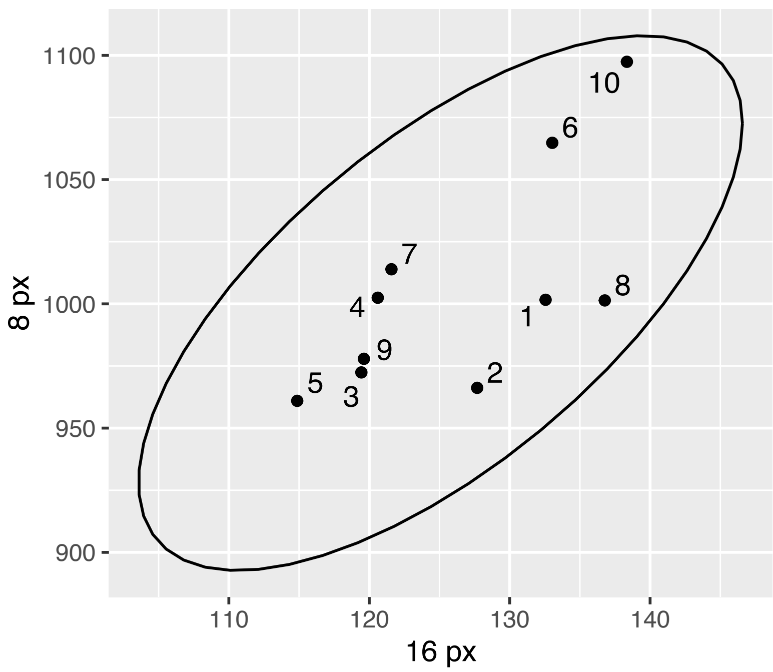

CT gldm dependencenonuniformitynormaliz

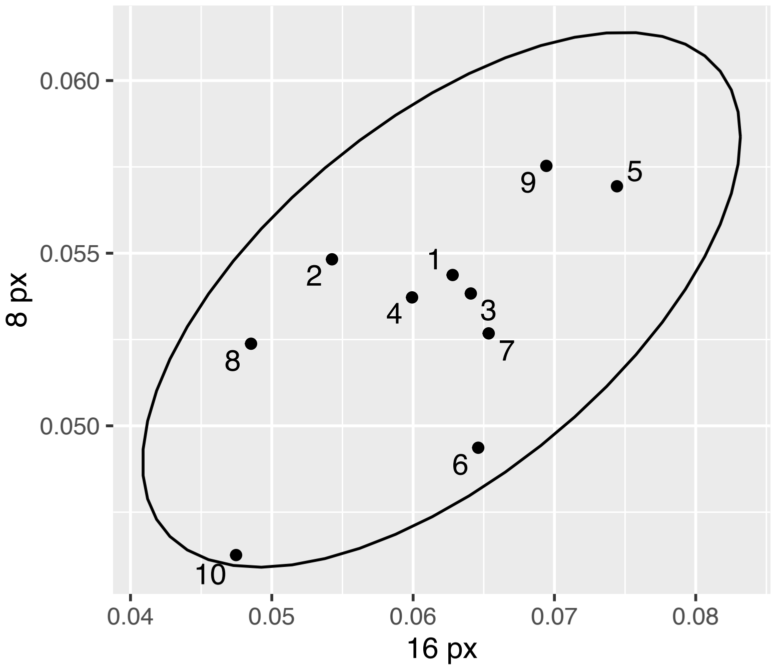

CT gldm graylevelvariance

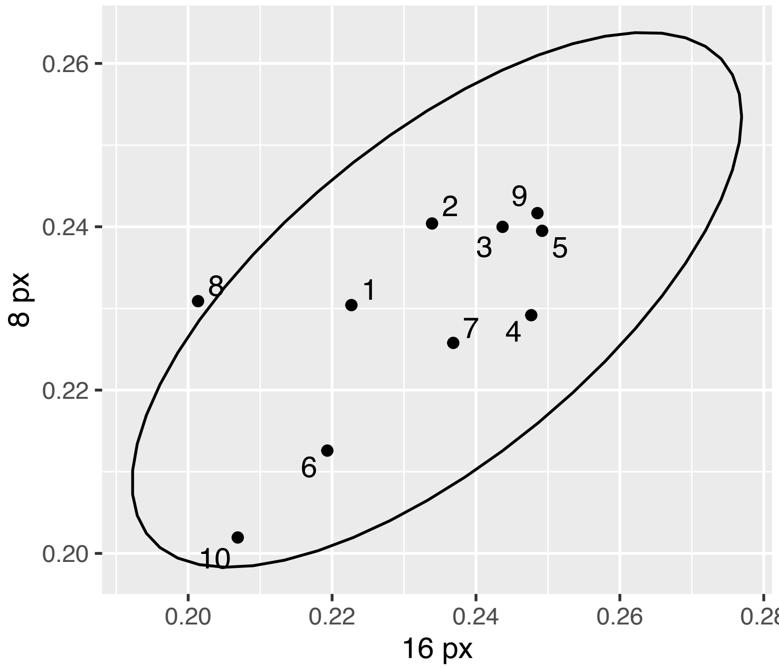

CT gldm dependencevariance

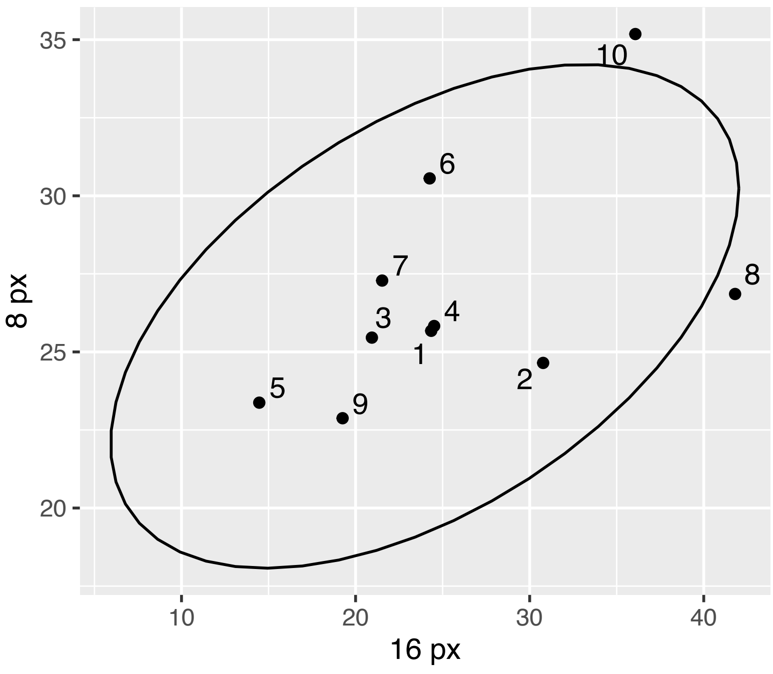

CT gldm highgraylevelemphasis

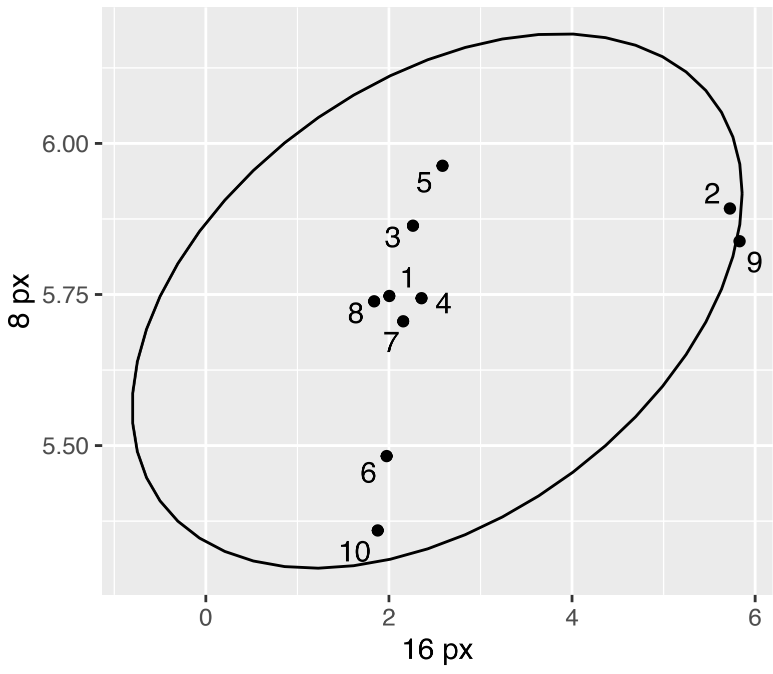

CT gldm largedependenceemphasis

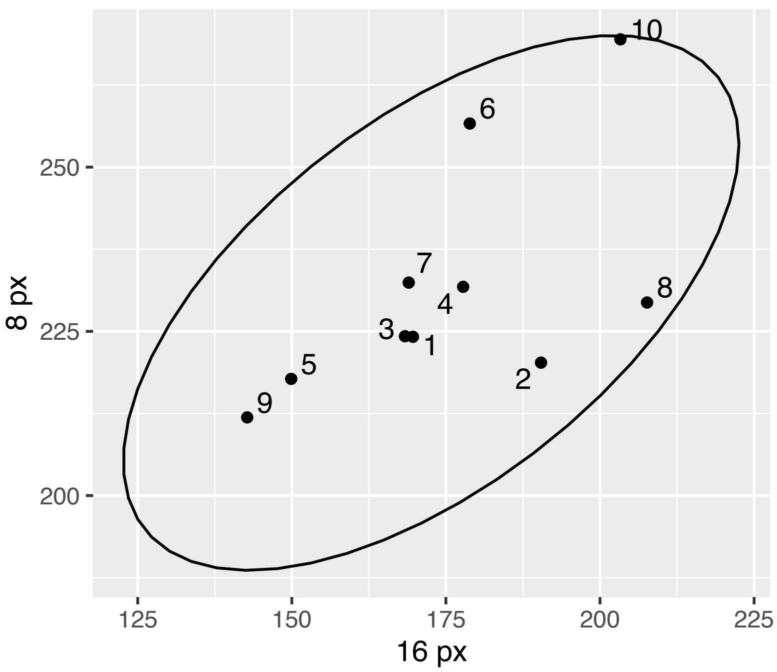

CT gldm lowgraylevelemphasis

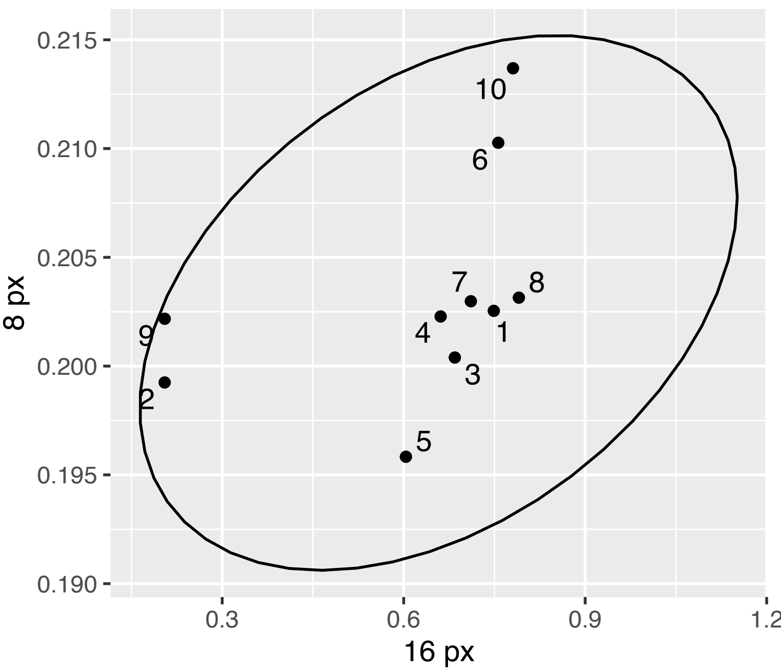

CT gldm largedependencehighgraylevelemp

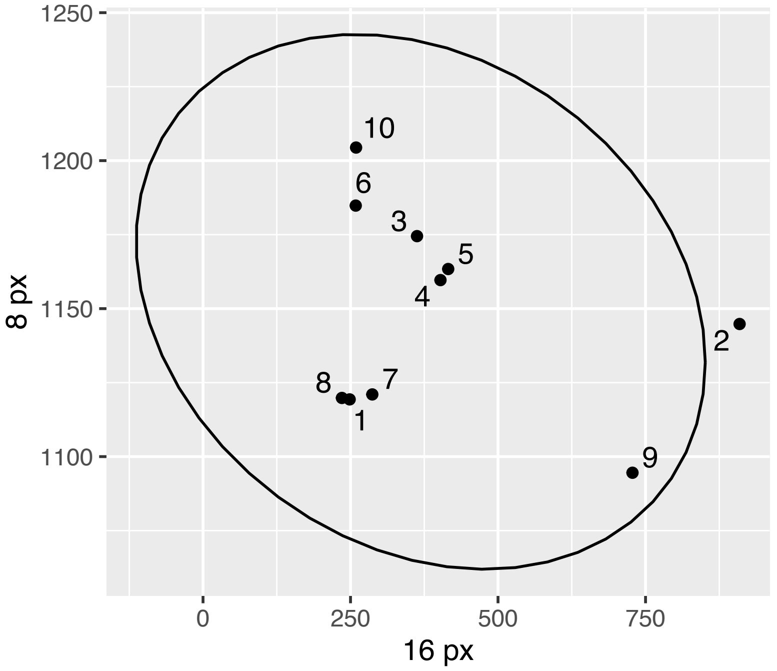

CT gldm smalldependenceemphasis

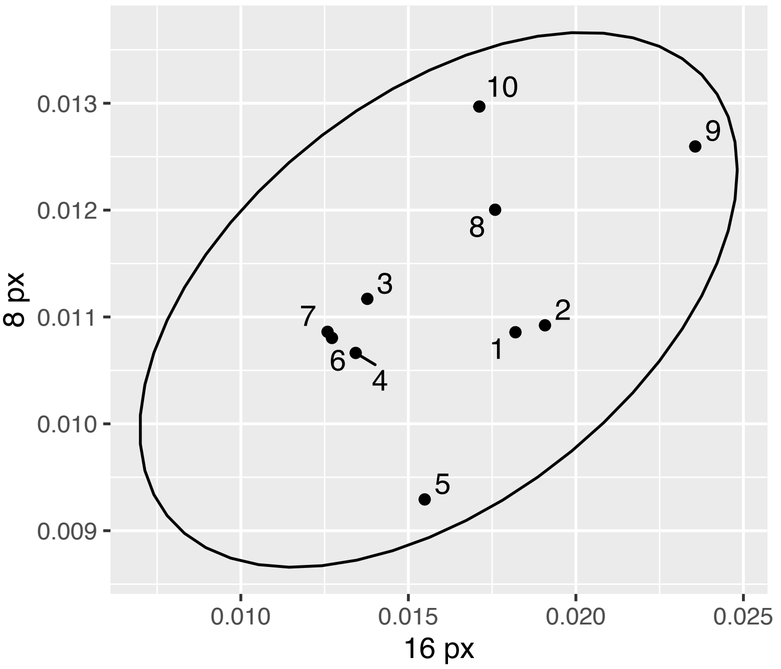

CT gldm largedependencelowgraylevelempha

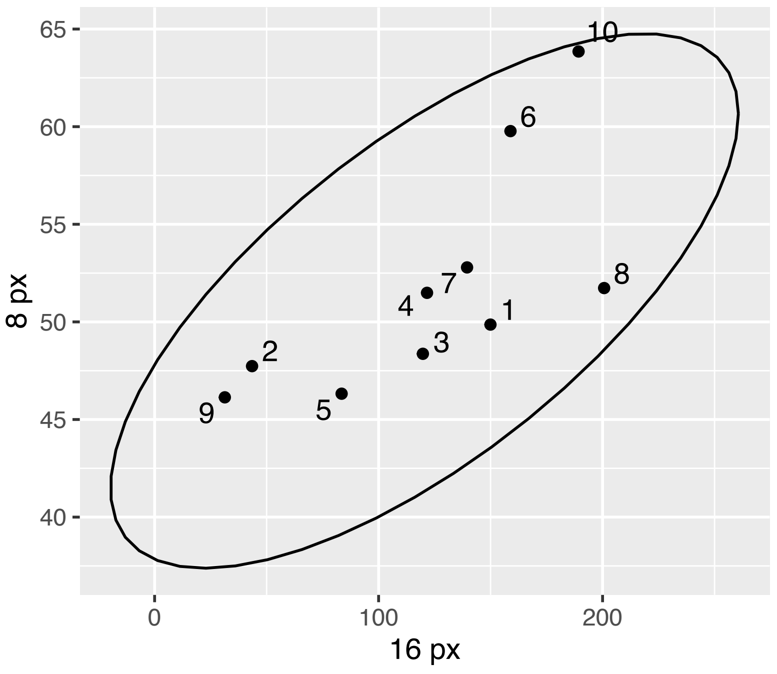

CT gldm smalldependencehighgraylevelemp

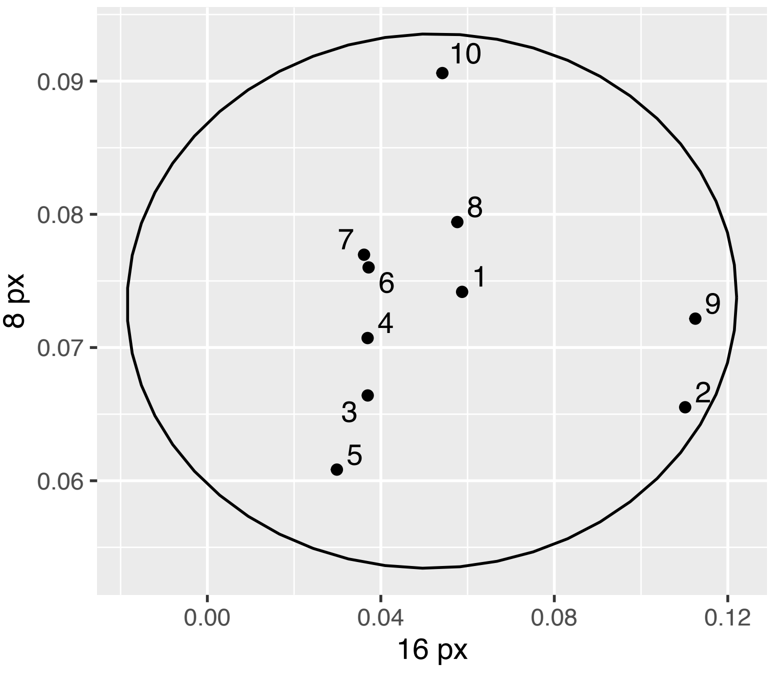

CT gldm smalldependencelowgraylevelempl

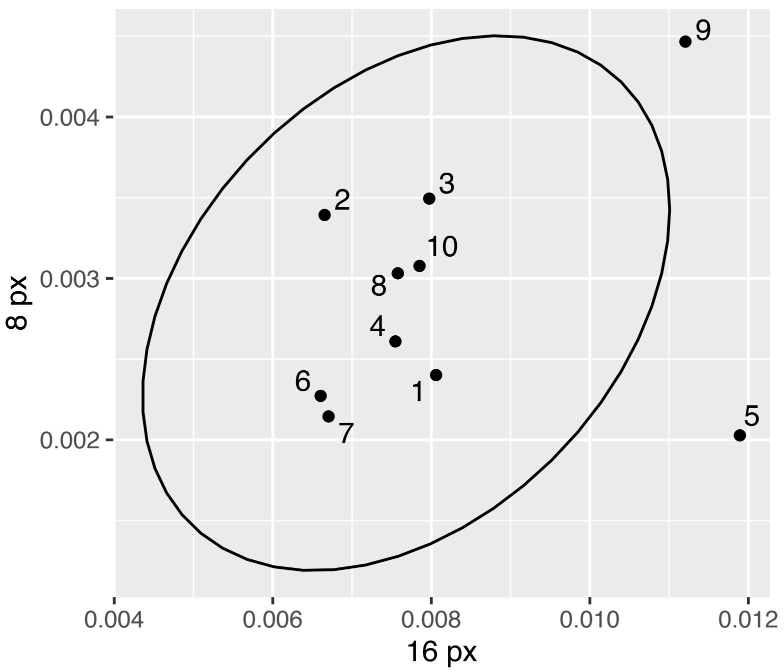

CT ngtdm complexity

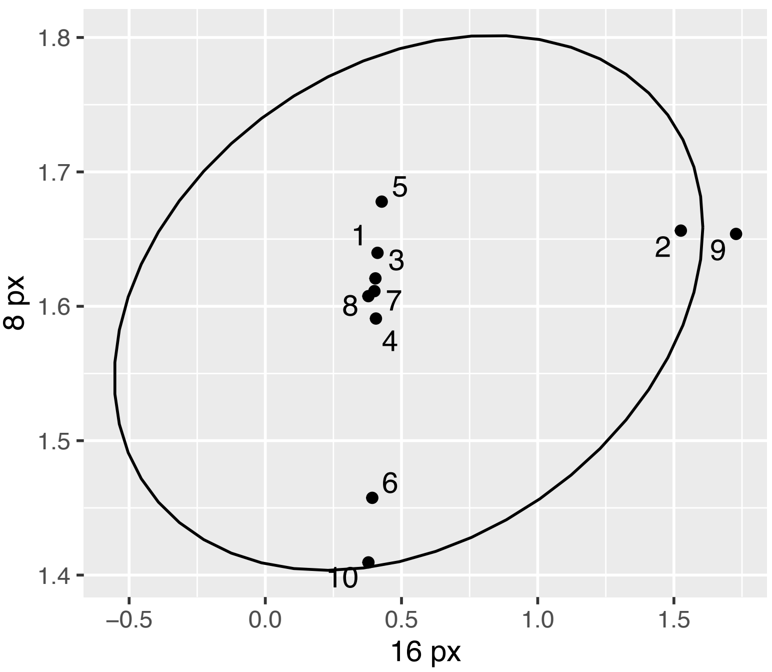

CT ngtdm busyness

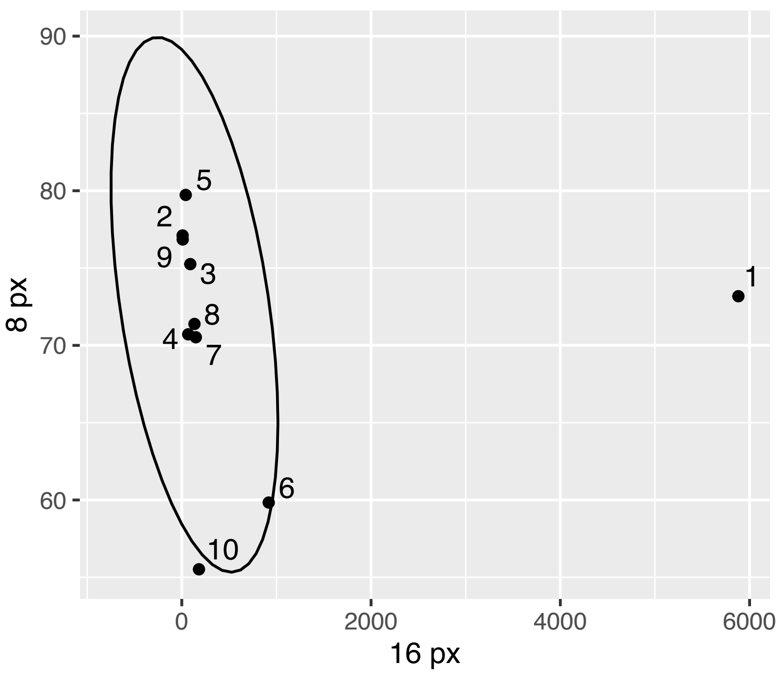

CT ngtdm contrast

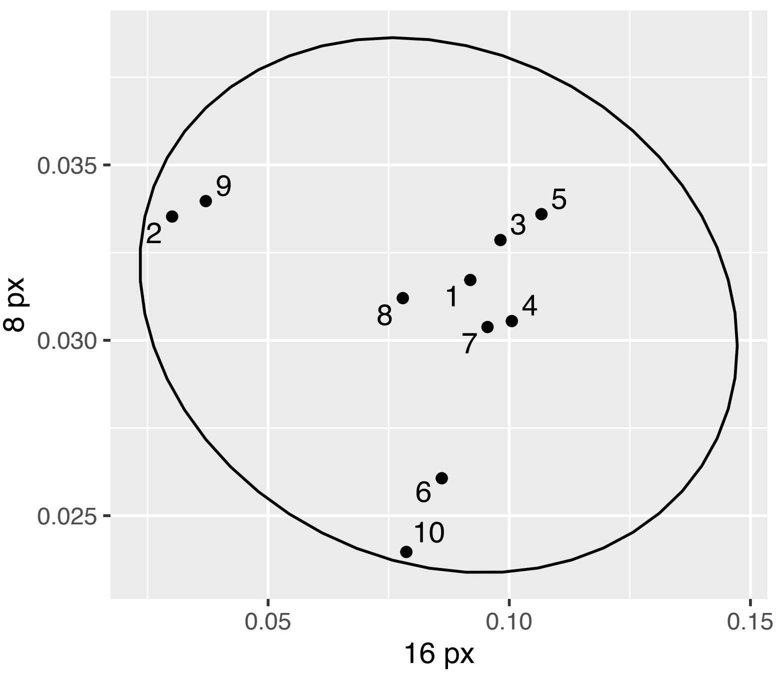

CT ngtdm coarseness

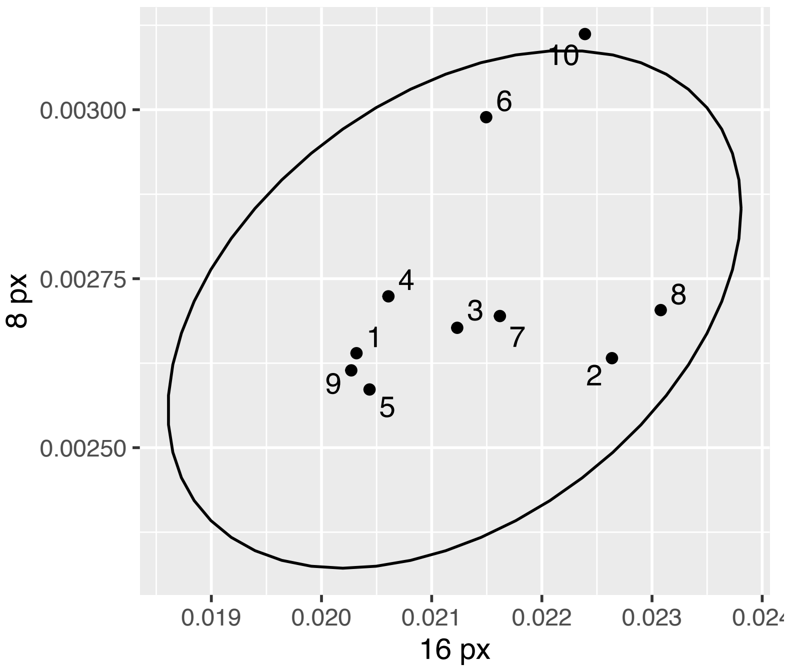

CT ngtdm strength

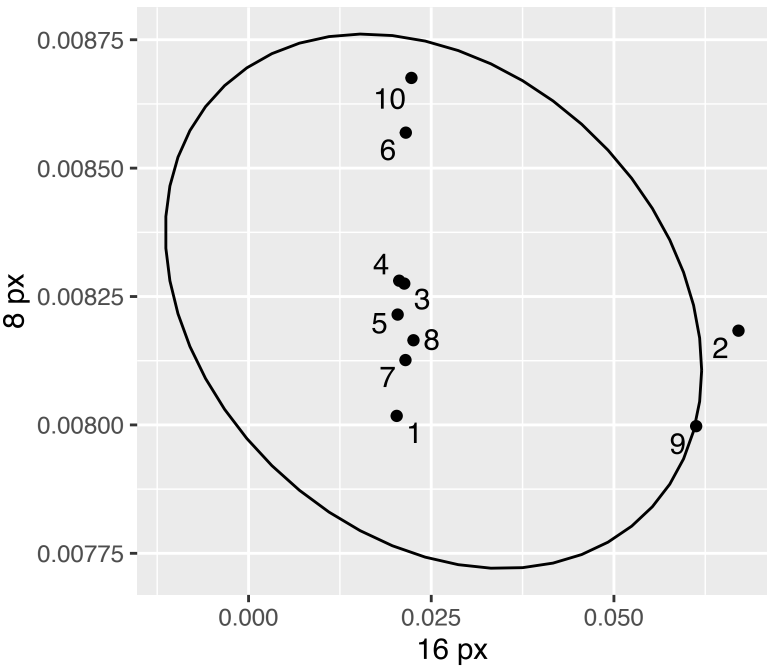

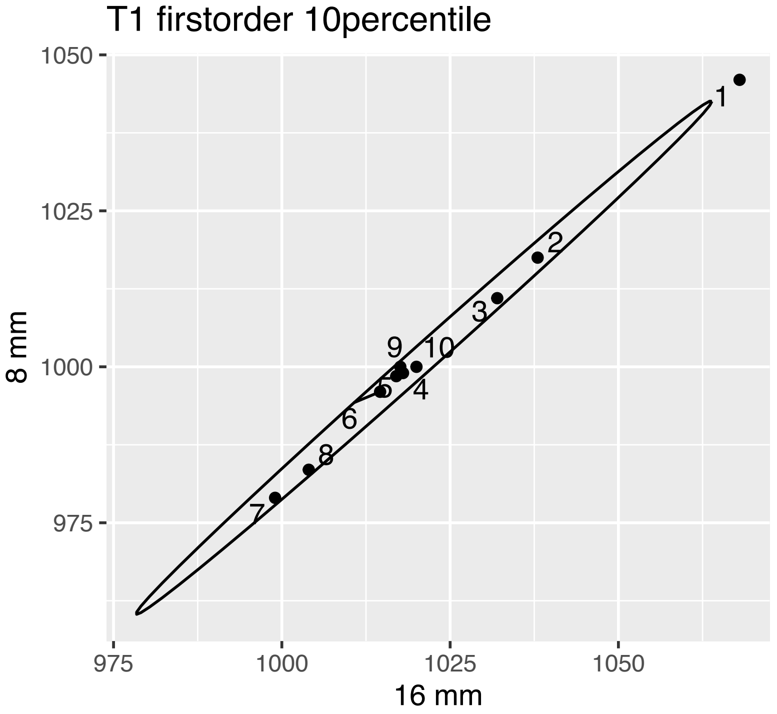
T1 firstorder 10percentile
8 mm
16 mm
1050
1025
1000
975
975
1000
1025
1050
1
2
3
4
5
6
7
8
9
10

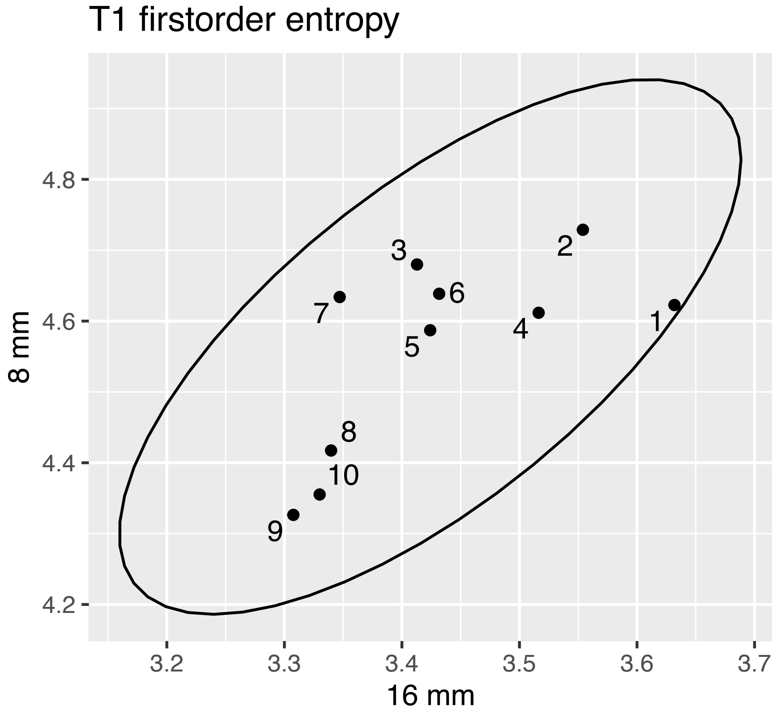
T1 firstorder entropy
8 mm
16 mm
4.8
4.6
4.4
4.2
3.2
3.3
3.4
3.5
3.6
3.7
1
2
3
4
5
6
7
8
9
10

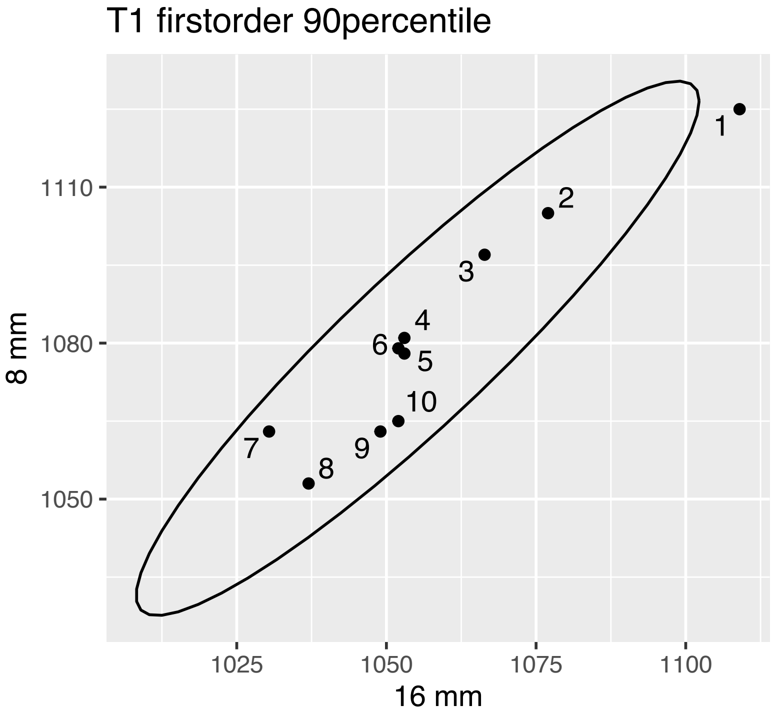
T1 firstorder 90percentile
8 mm
16 mm
1110
1080
1050
1025
1050
1075
1100
1
2
3
4
5
6
7
8
9
10

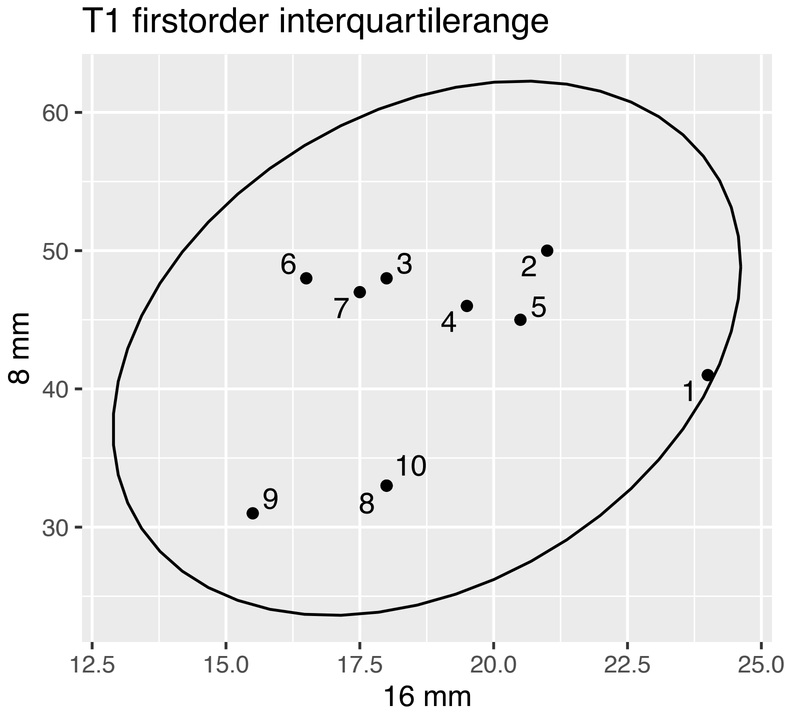
T1 firstorder interquartilerange
8 mm
16 mm
60
50
40
30
12.5
15.0
17.5
20.0
22.5
25.0
1
2
3
4
5
6
7
8
9
10

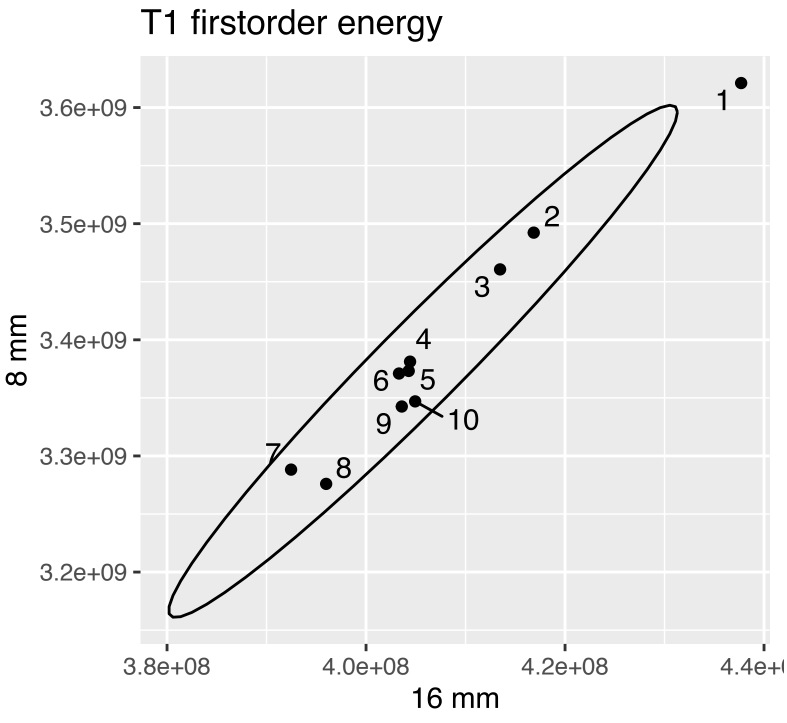
T1 firstorder energy
8 mm
16 mm
3.6e+09
3.5e+09
3.4e+09
3.3e+09
3.2e+09
3.8e+08
4.0e+08
4.2e+08
1
2
3
4
5
6
7
8
9
10

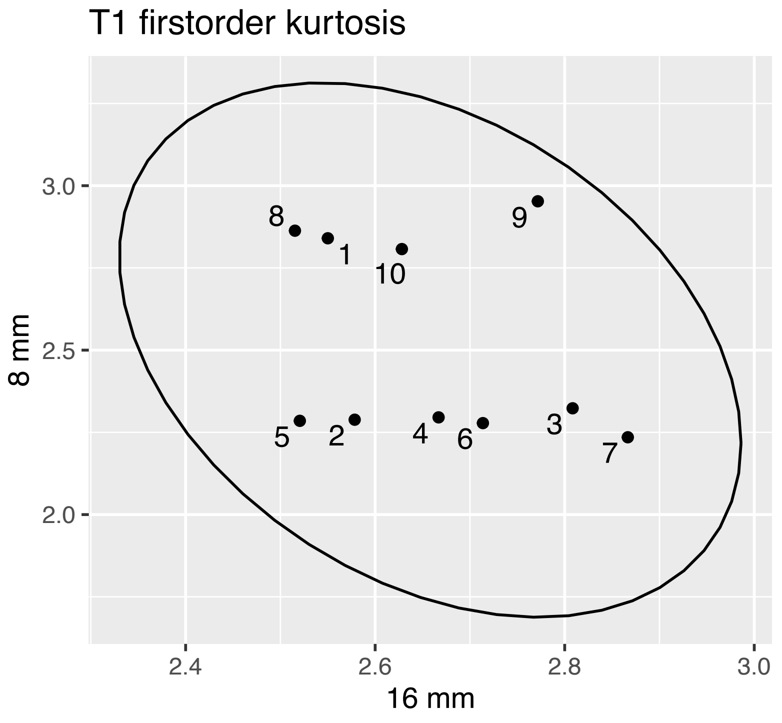
T1 firstorder kurtosis
8 mm
16 mm
3.0
2.5
2.0
2.4
2.6
2.8
3.0
1
2
3
4
5
6
7
8
9
10

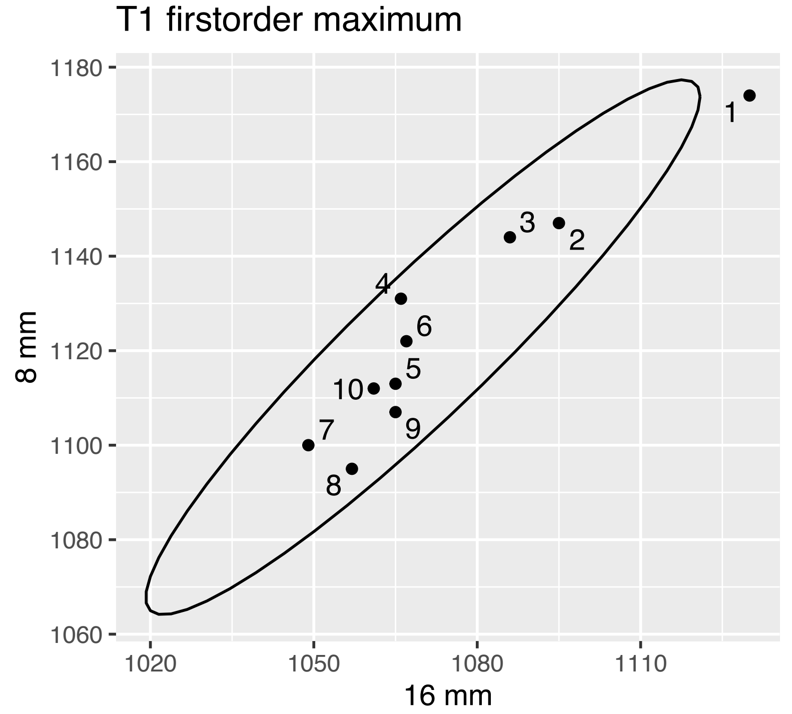
T1 firstorder maximum
8 mm
16 mm
1060
1080
1100
1120
1140
1160
1180
1020
1050
1080
1110
1
2
3
4
5
6
7
8
9
10

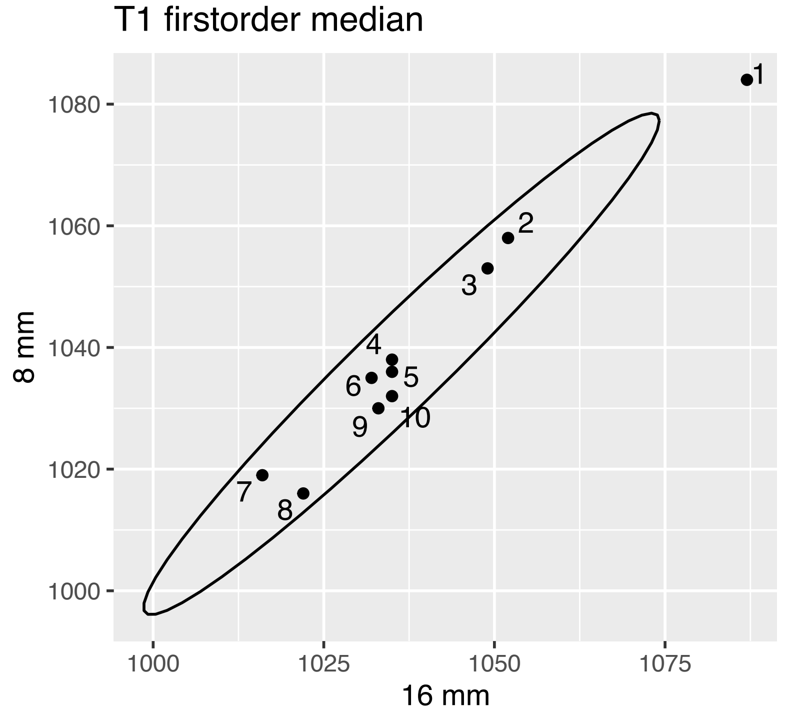
T1 firstorder median
8 mm
16 mm
1000
1020
1040
1060
1080
1000
1025
1050
1075
1
2
3
4
5
6
7
8
9
10

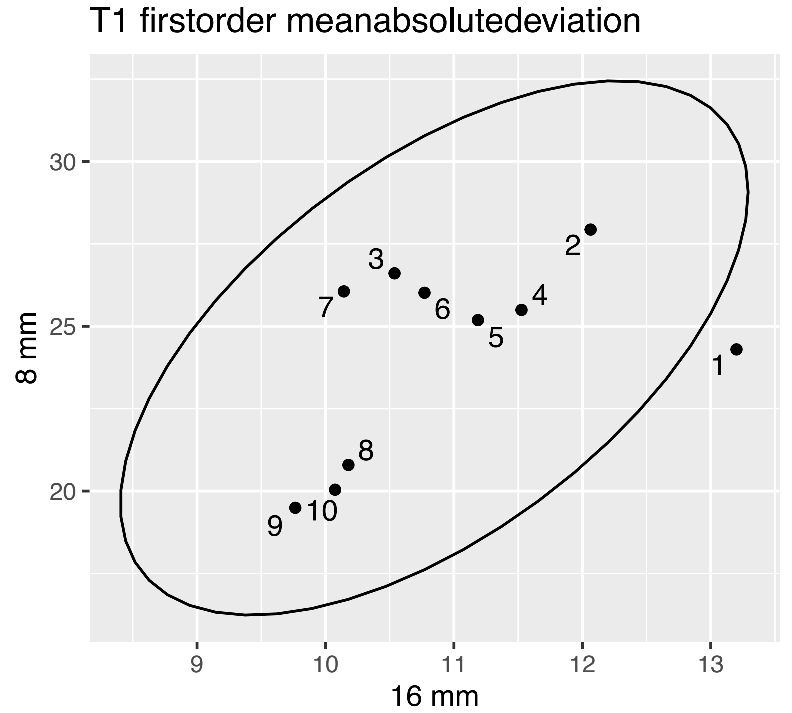
T1 firstorder meanabsolutedeviation
8 mm
16 mm
20
25
30
9
10
11
12
13
1
2
3
4
5
6
7
8
9
10

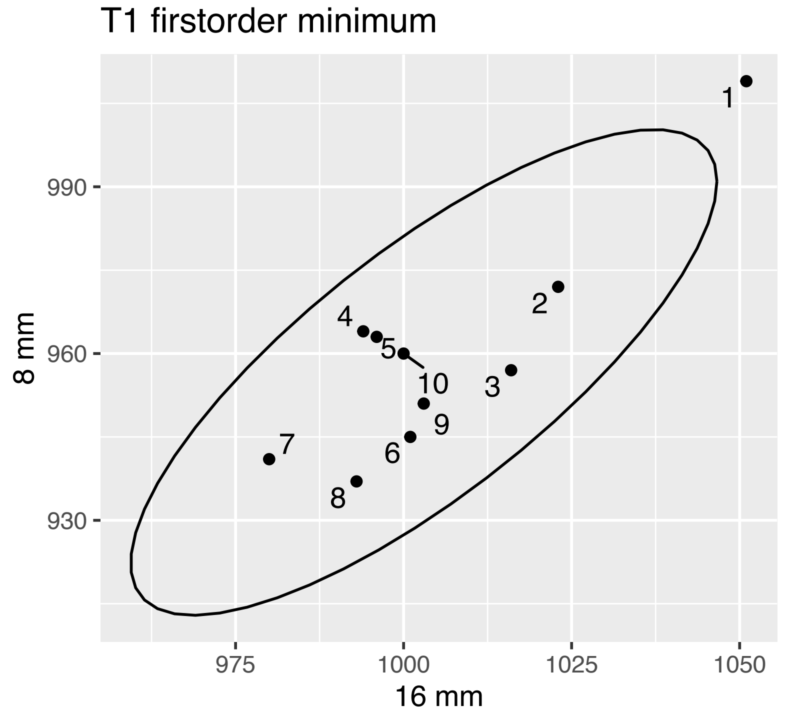
T1 firstorder minimum
8 mm
16 mm
930
960
990
975
1000
1025
1050
1
2
3
4
5
6
7
8
9
10

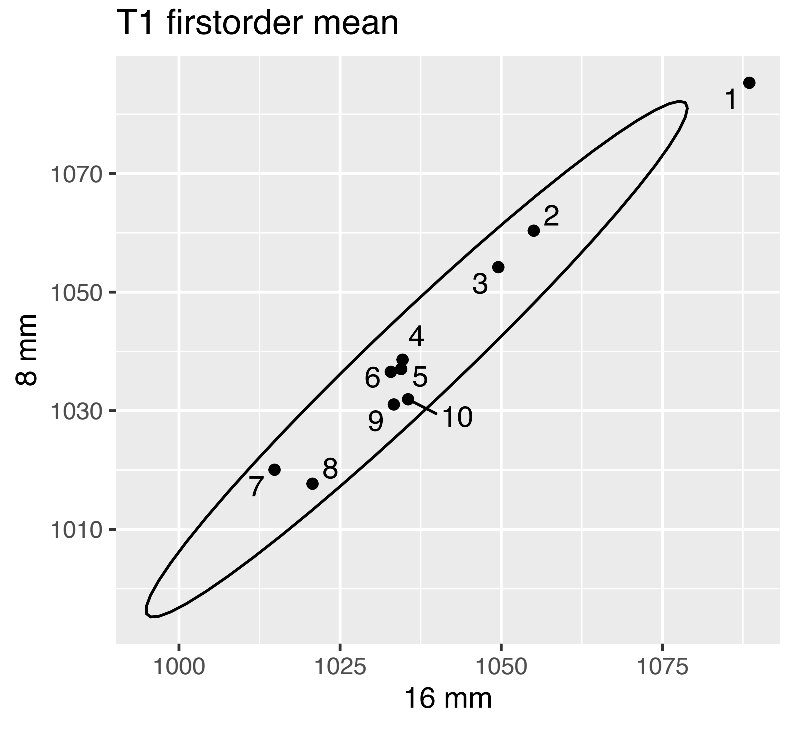
T1 firstorder mean
8 mm
16 mm
1010
1030
1050
1070
1000
1025
1050
1075
1
2
3
4
5
6
7
8
9
10

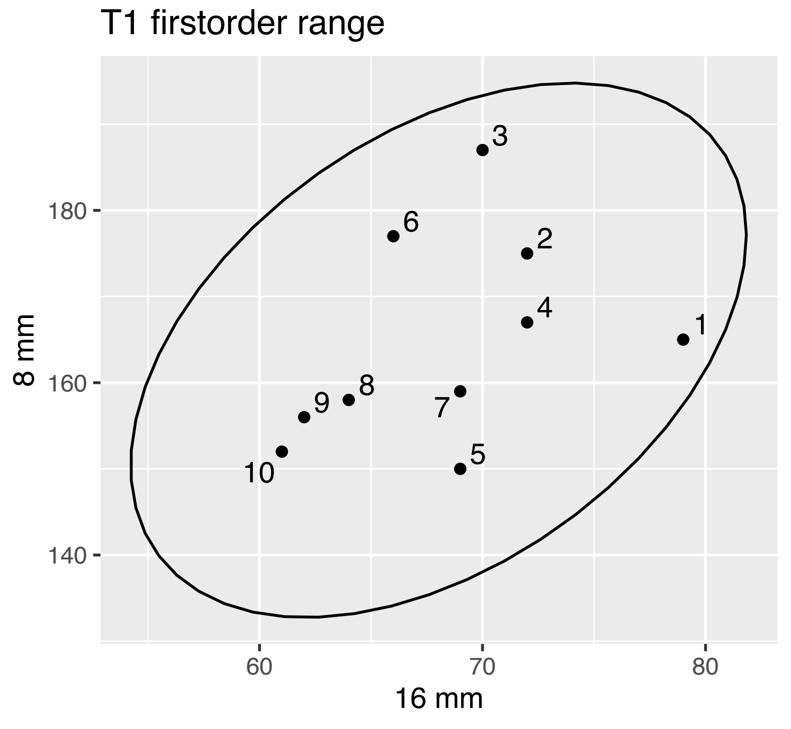
T1 firstorder range
8 mm
16 mm
140
160
180
60
70
80
1
2
3
4
5
6
7
8
9
10

T1 firstorder robustmeanabsolutedeviation

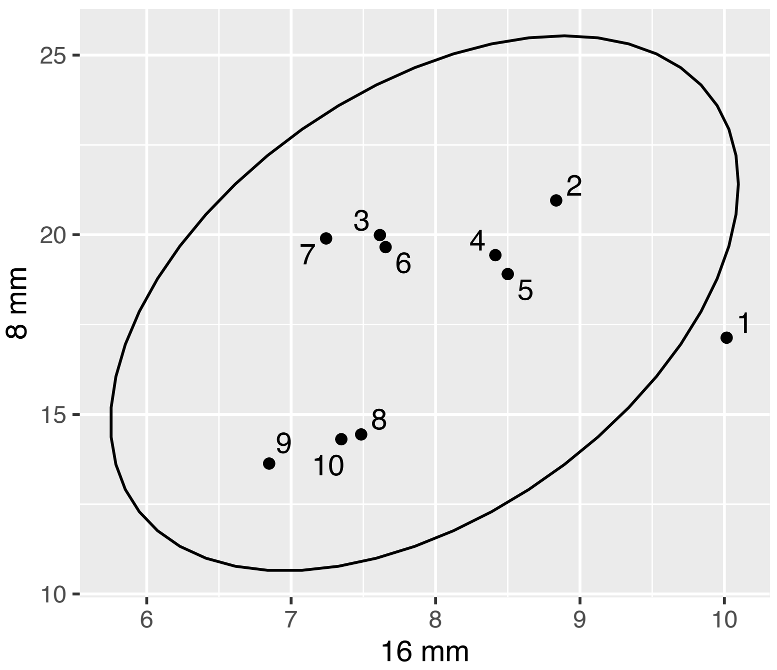

T1 firstorder totalenergy

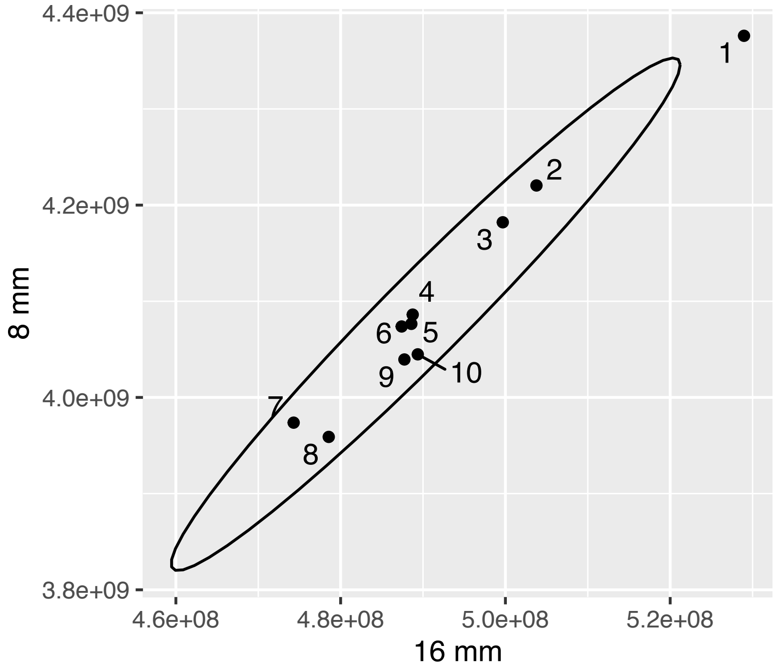

T1 firstorder rootmeansquared

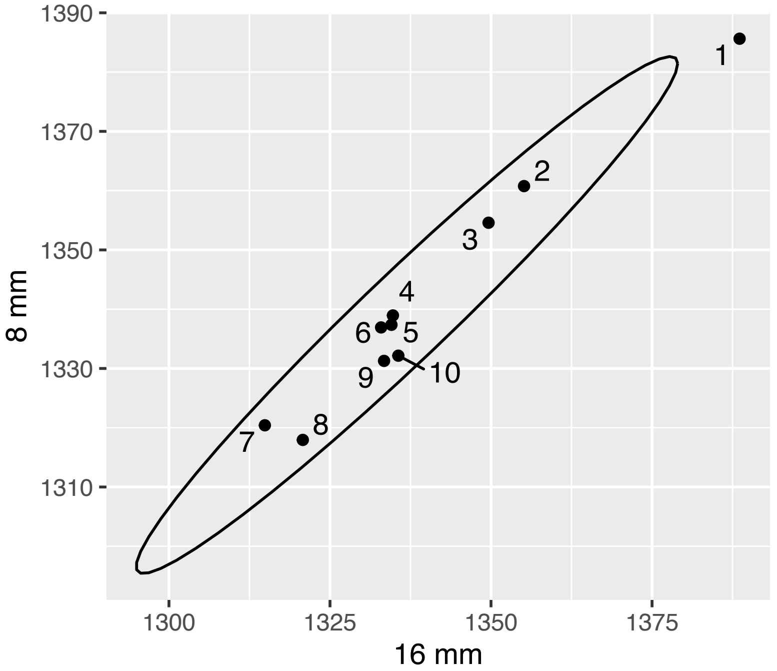

T1 firstorder uniformity

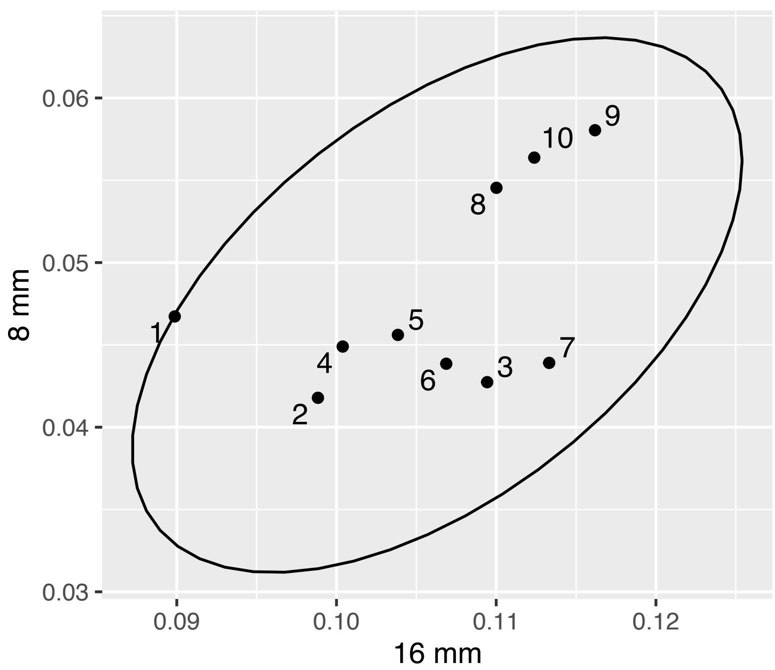

T1 firstorder skewness

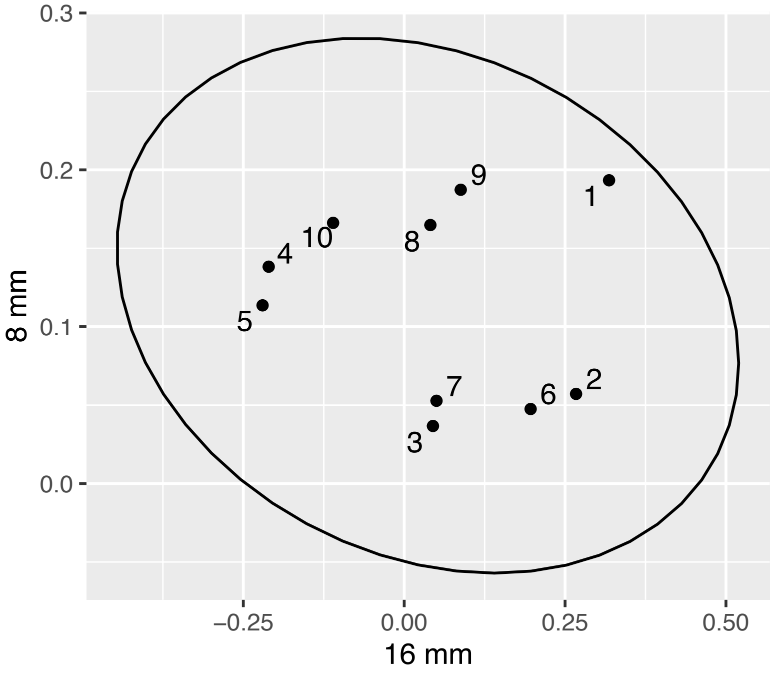

T1 firstorder variance

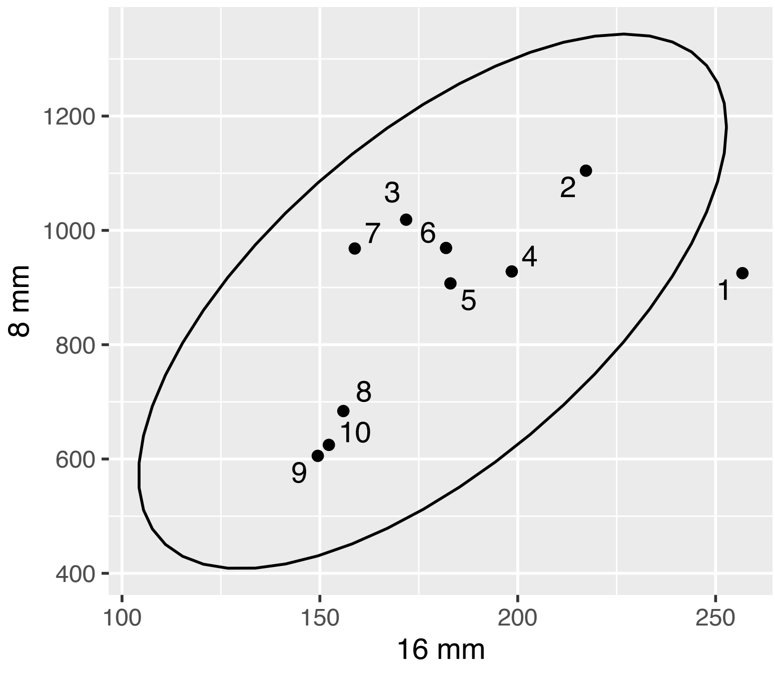

T1 glcm autocorrelation

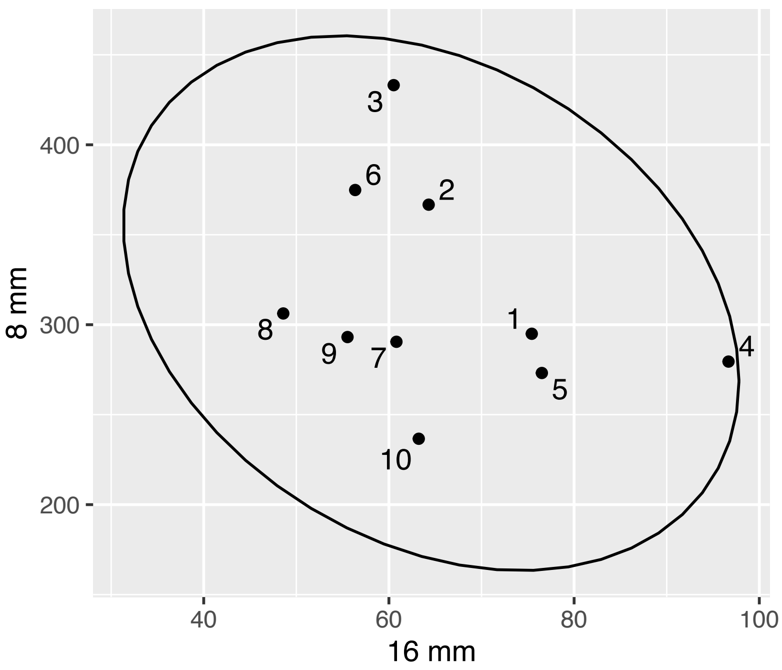

T1 glcm clustertendency

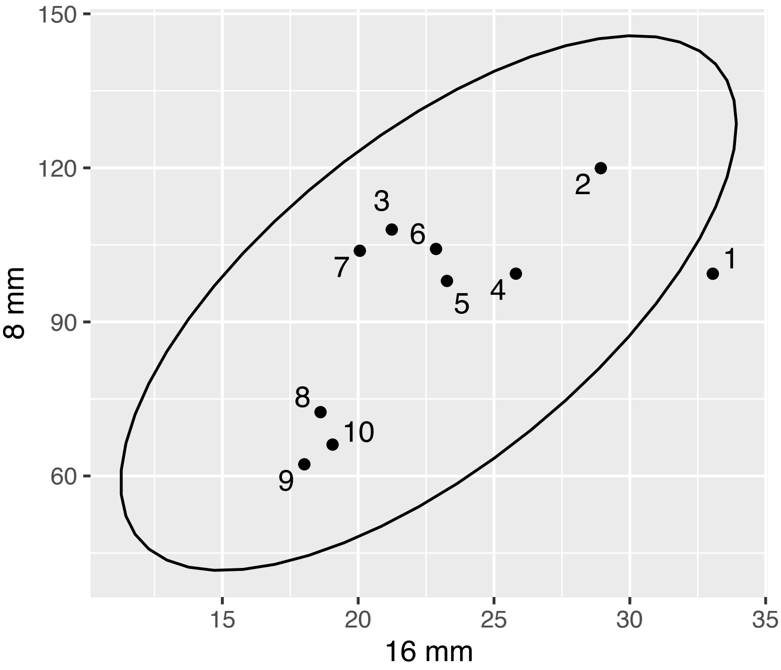

T1 glcm clusterprominence

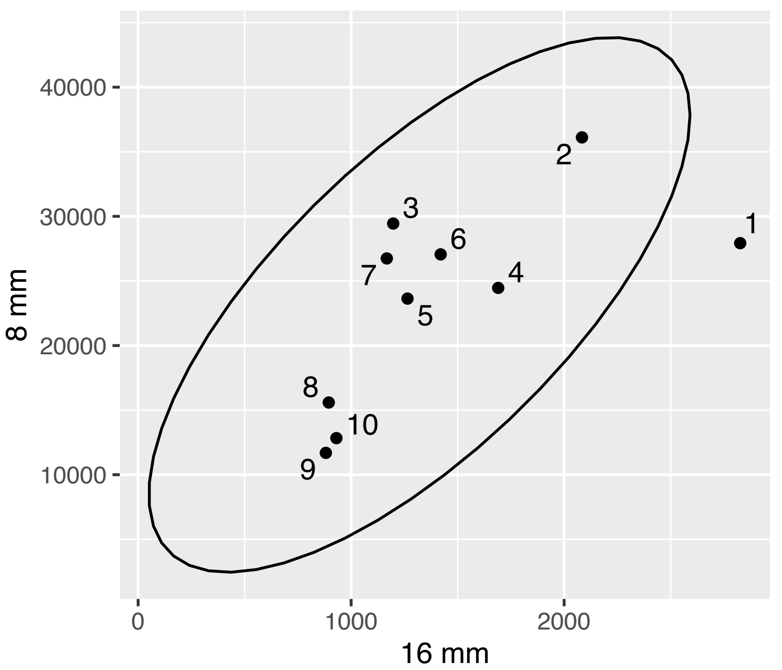

T1 glcm contrast

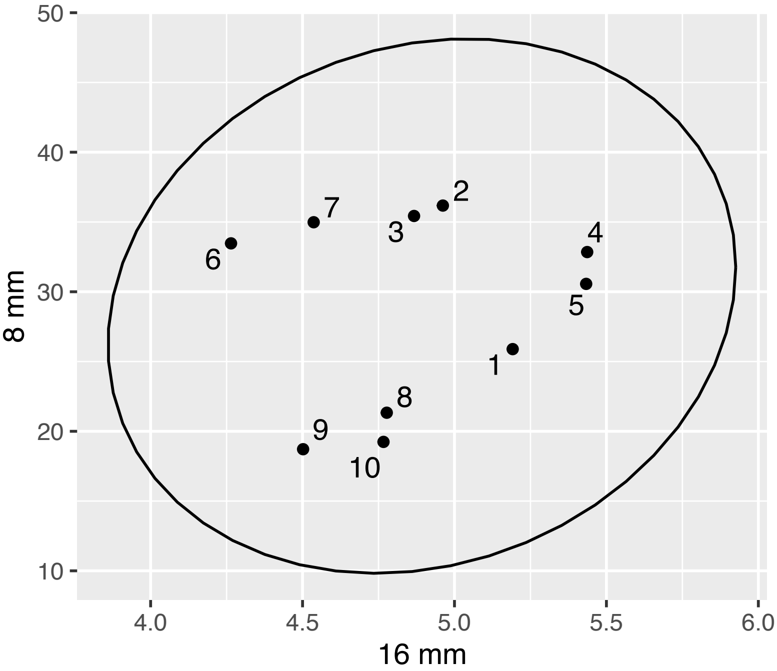

T1 glcm clustershade

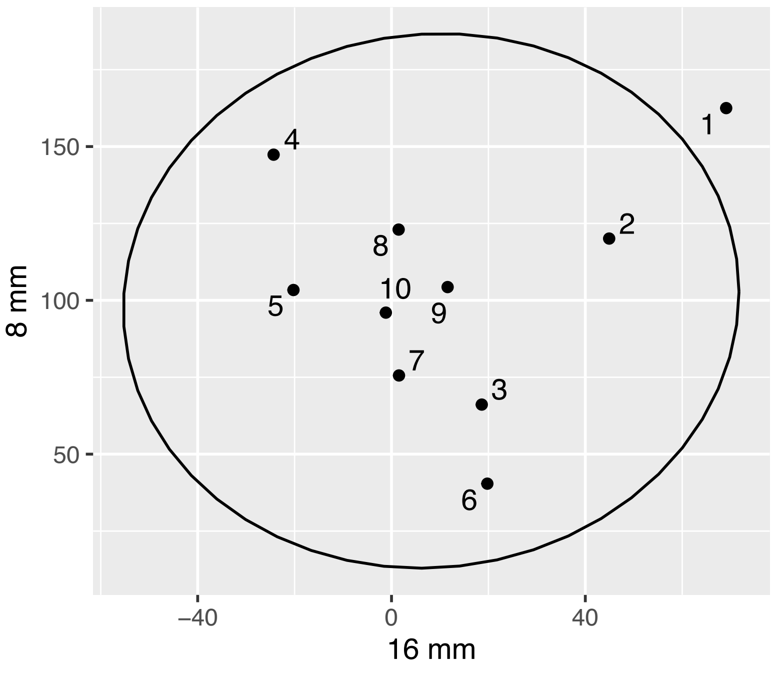

T1 glcm correlation

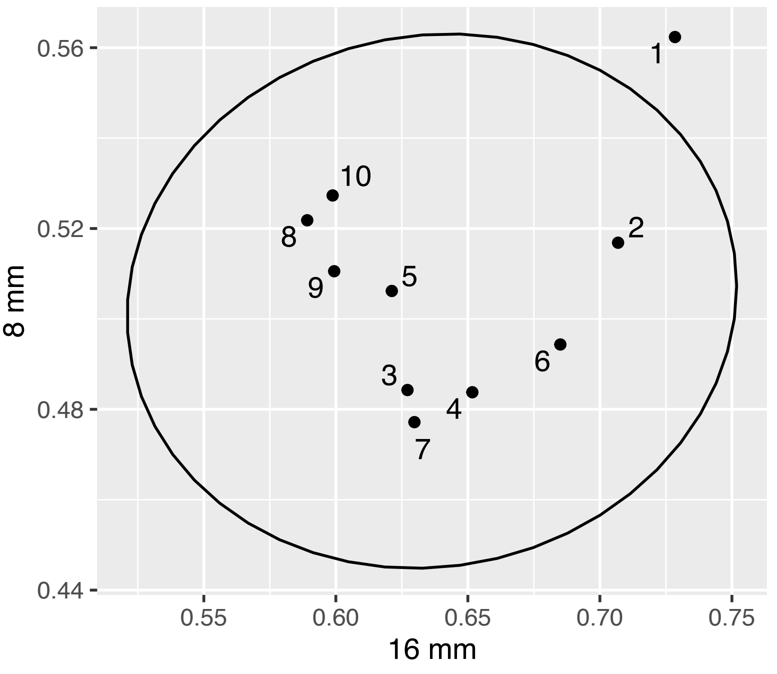

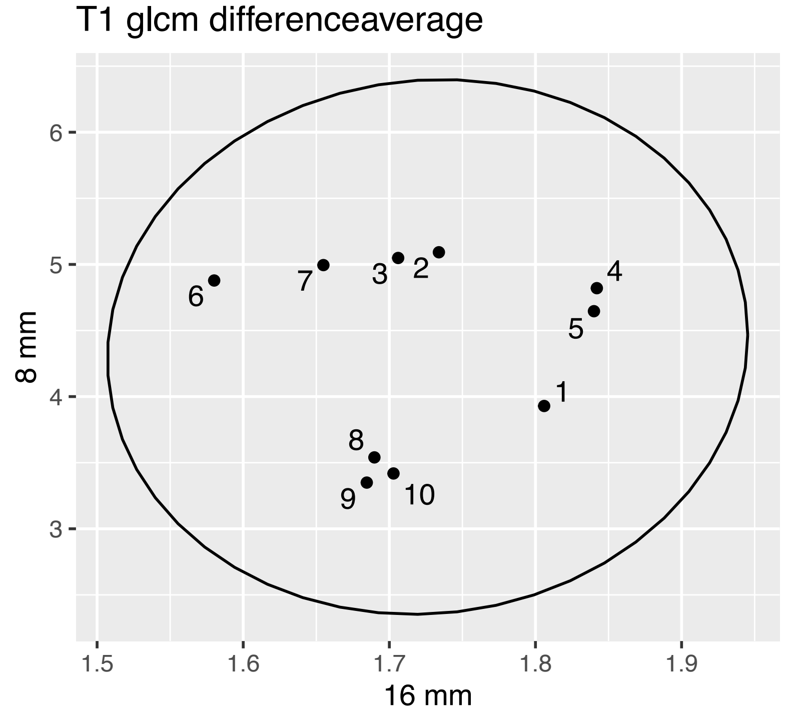
T1 glcm differenceaverage
8 mm
16 mm
6
5
4
3
1.5
1.6
1.7
1.8
1.9
6
7
3
2
4
5
1
8
9
10

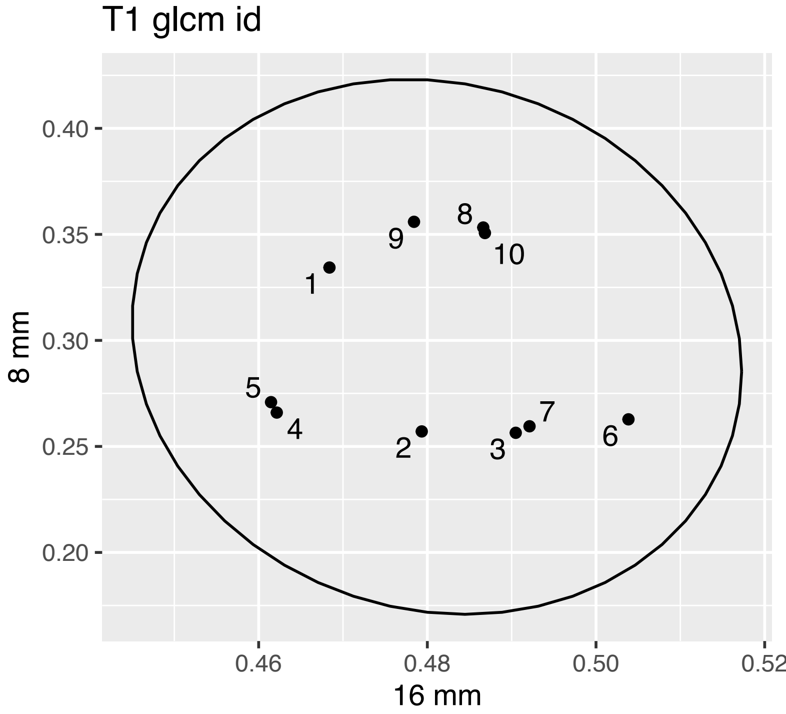
T1 glcm id
8 mm
16 mm
0.40
0.35
0.30
0.25
0.20
0.46
0.48
0.50
0.52
8
9
10
1
5
4
2
3
7
6

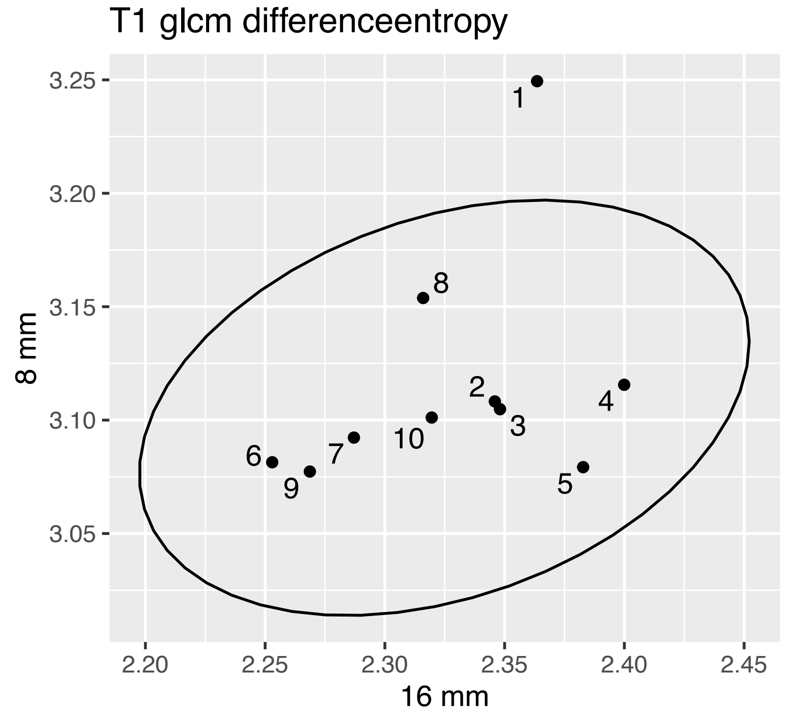
T1 glcm differenceentropy
8 mm
16 mm
3.25
3.20
3.15
3.10
3.05
2.20
2.25
2.30
2.35
2.40
2.45
1
8
2
3
4
10
7
6
9
5

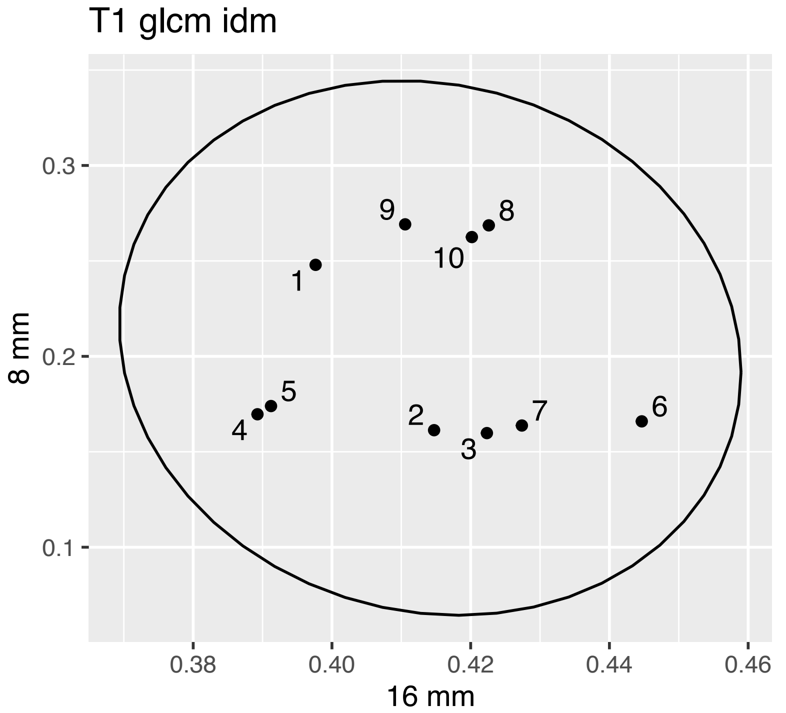
T1 glcm idm
8 mm
16 mm
0.3
0.2
0.1
0.38
0.40
0.42
0.44
0.46
9
8
10
1
5
4
2
3
7
6

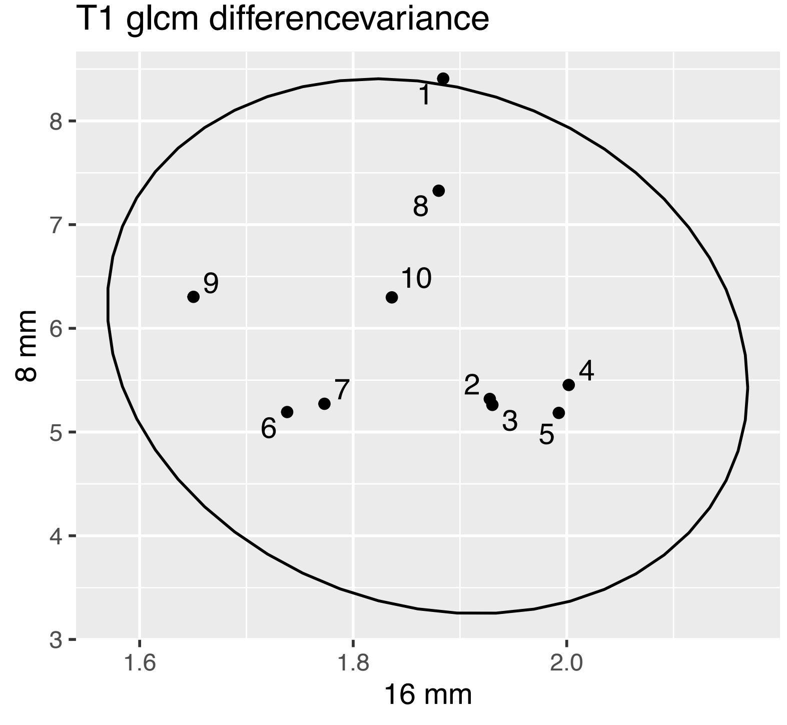
T1 glcm differencevariance
8 mm
16 mm
8
7
6
5
4
3
1.6
1.8
2.0
1
8
9
10
7
6
2
3
4
5

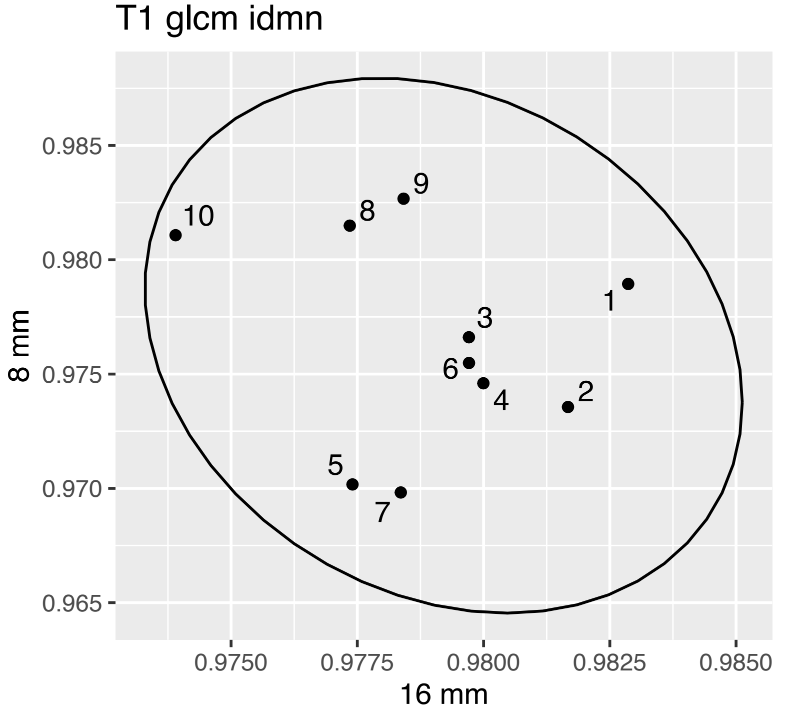
T1 glcm idmn
8 mm
16 mm
0.985
0.980
0.975
0.970
0.965
0.9750
0.9775
0.9800
0.9825
0.9850
10
8
9
1
3
6
4
2
5
7

T1 glcm idn

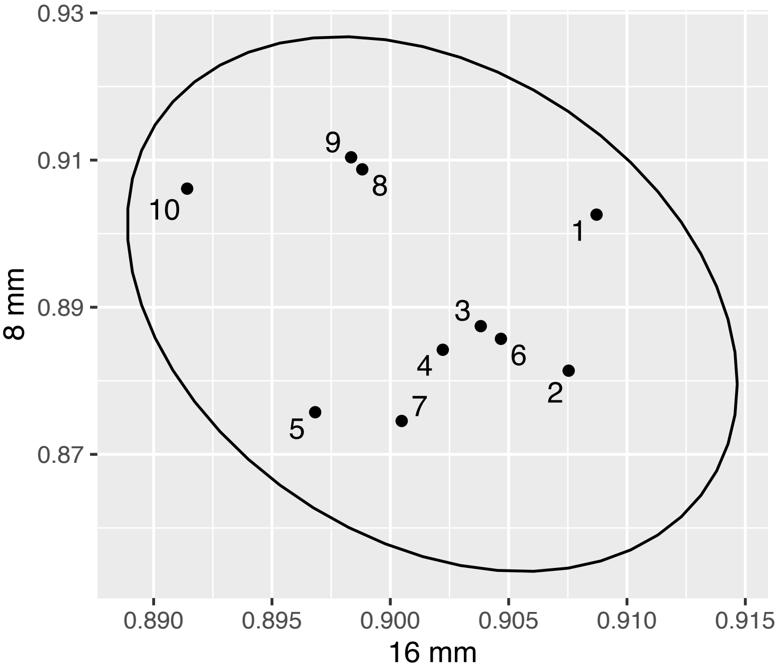

T1 glcm inversevariance

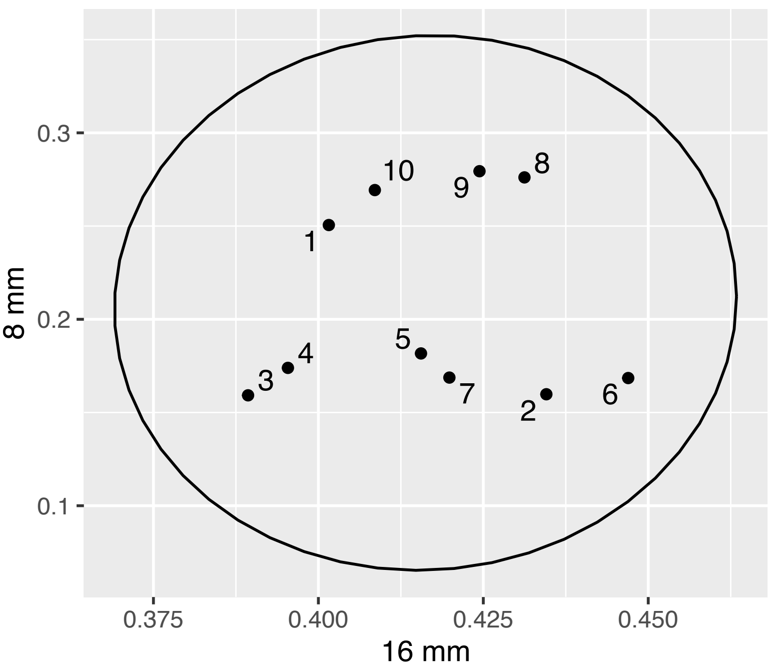

T1 glcm imc1

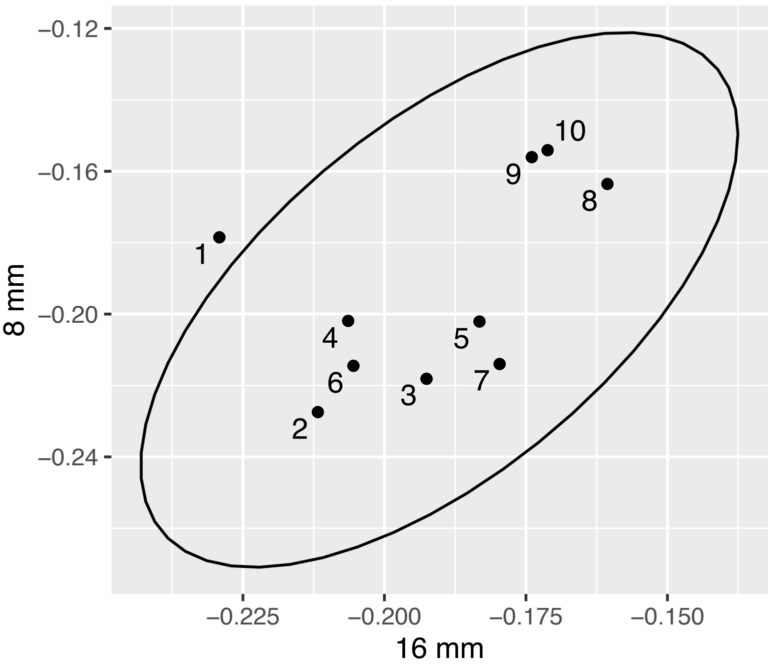

T1 glcm jointaverage

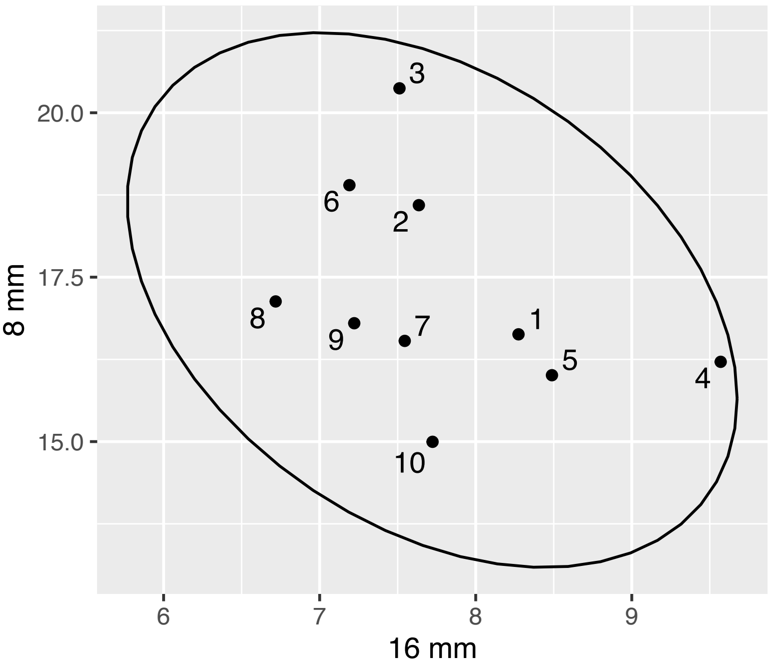

T1 glcm imc2

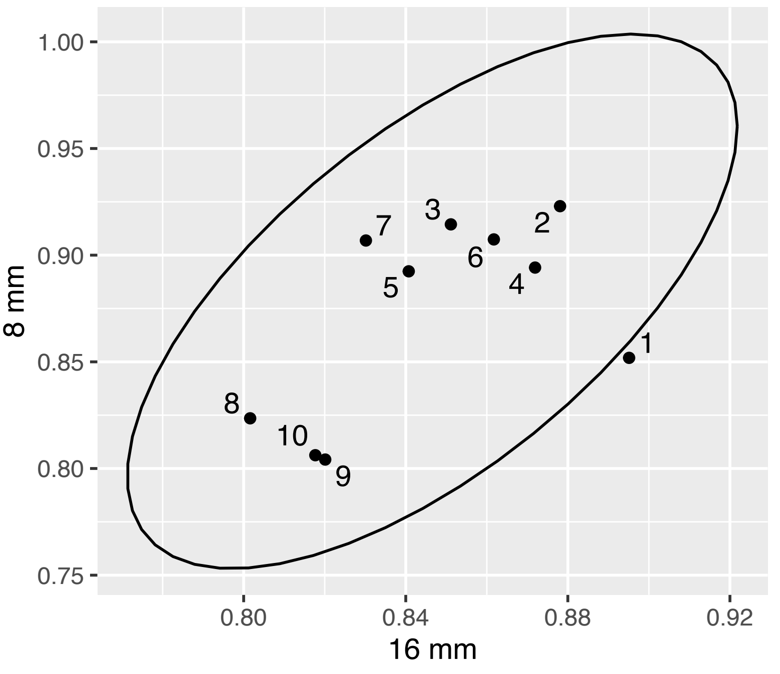

T1 glcm jointenergy

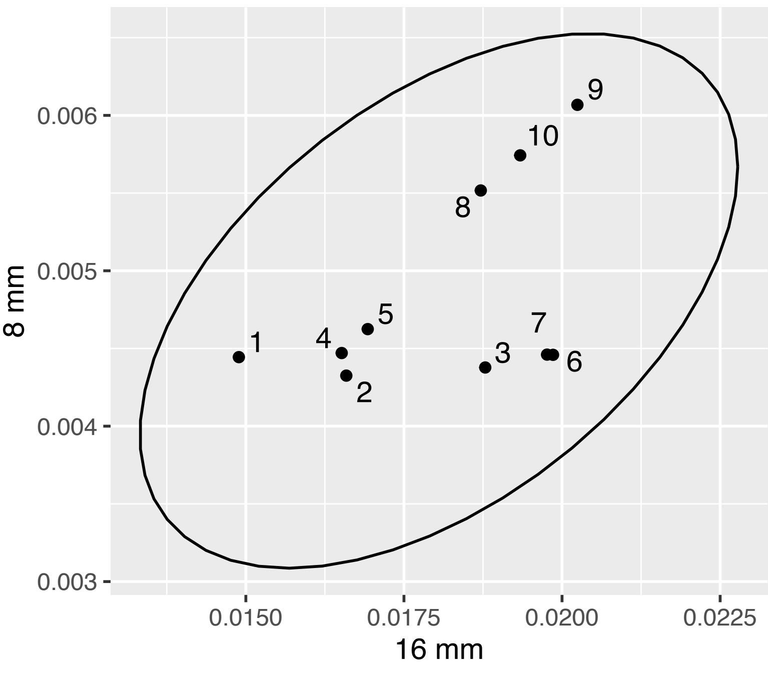

T1 glcm jointentropy

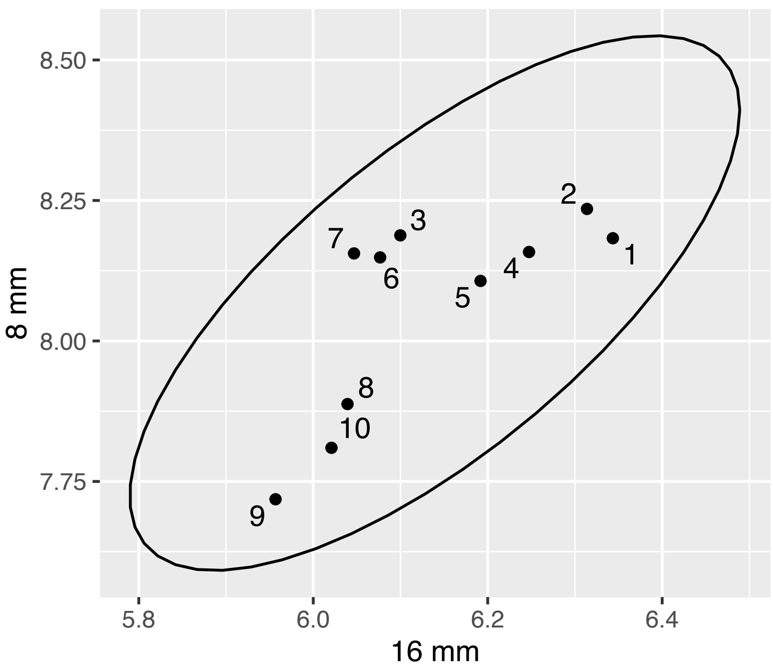

T1 glcm sumaverage

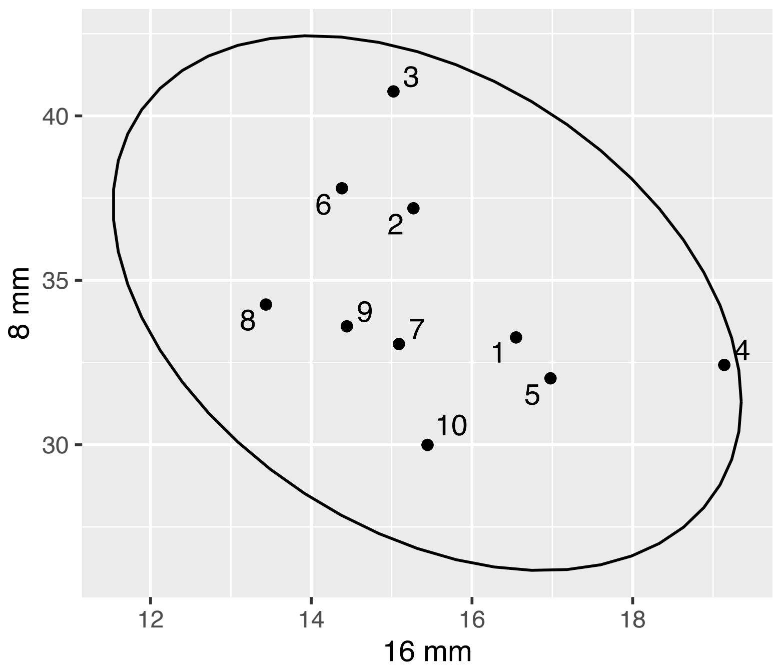

T1 glcm mcc

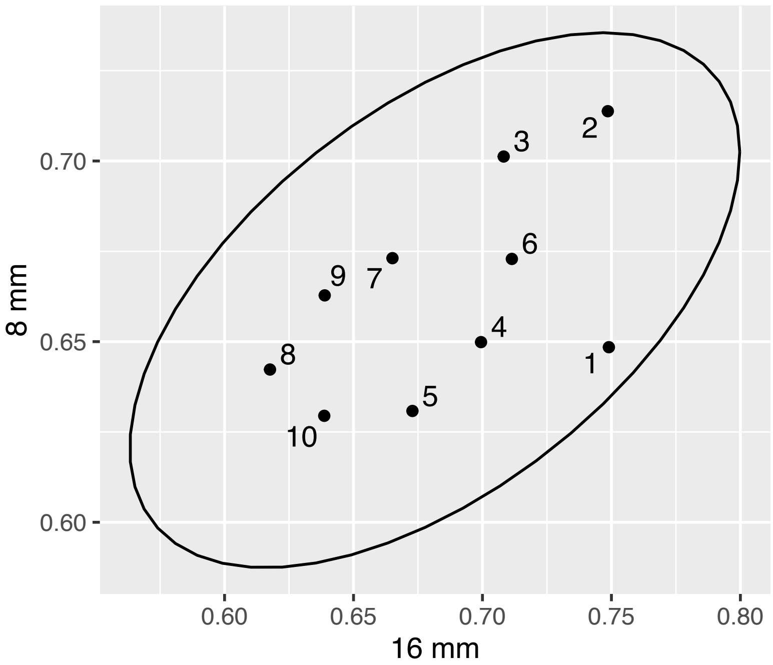

T1 glcm sumentropy

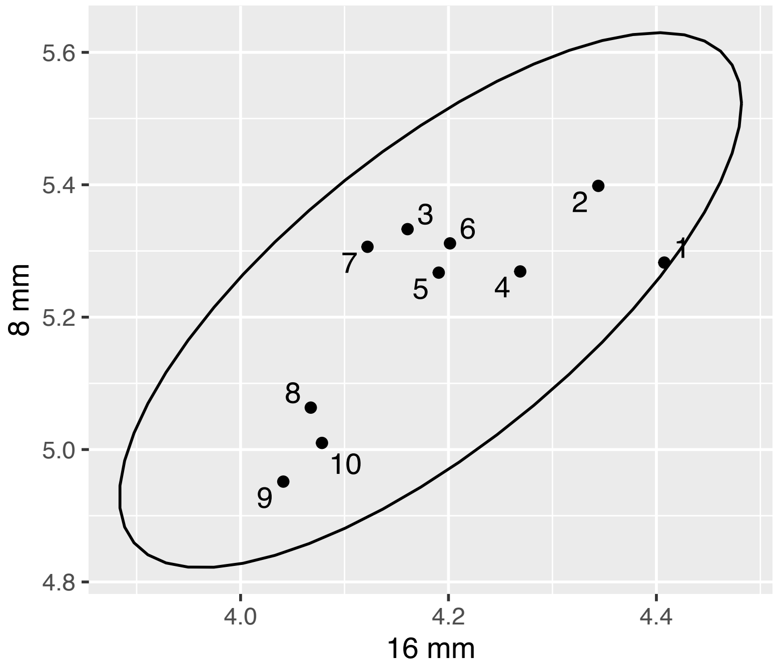

T1 glcm maximumprobability

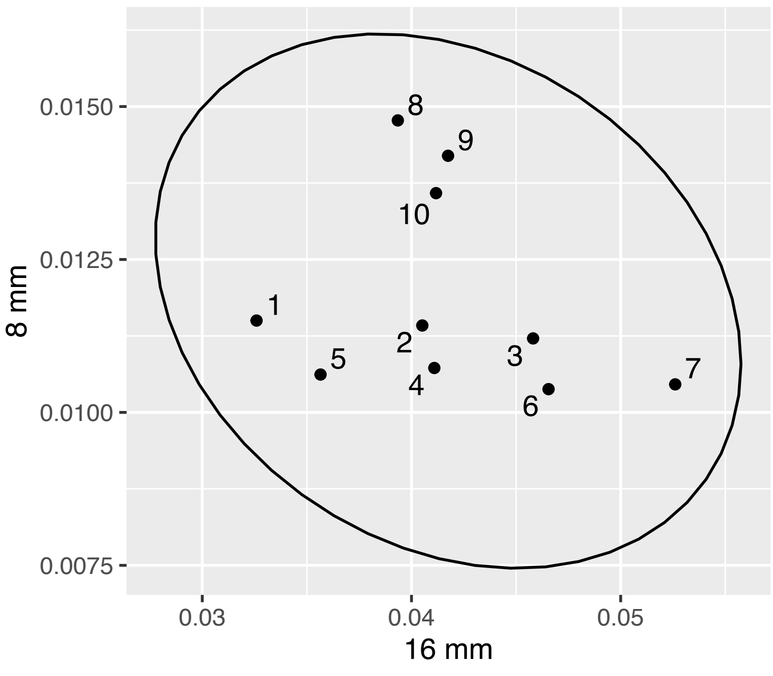

T1 glcm sumsquares

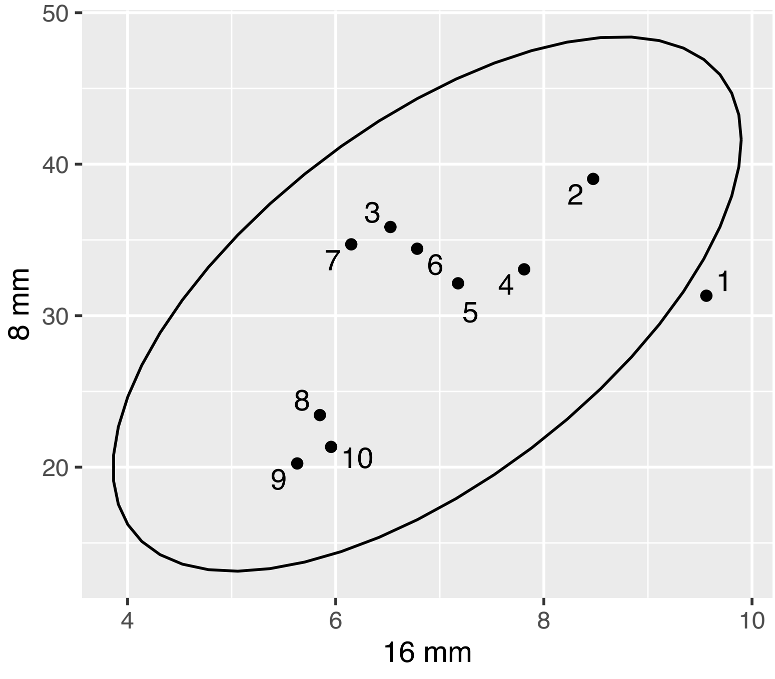

T1 glrlm graylevelnonuniformity

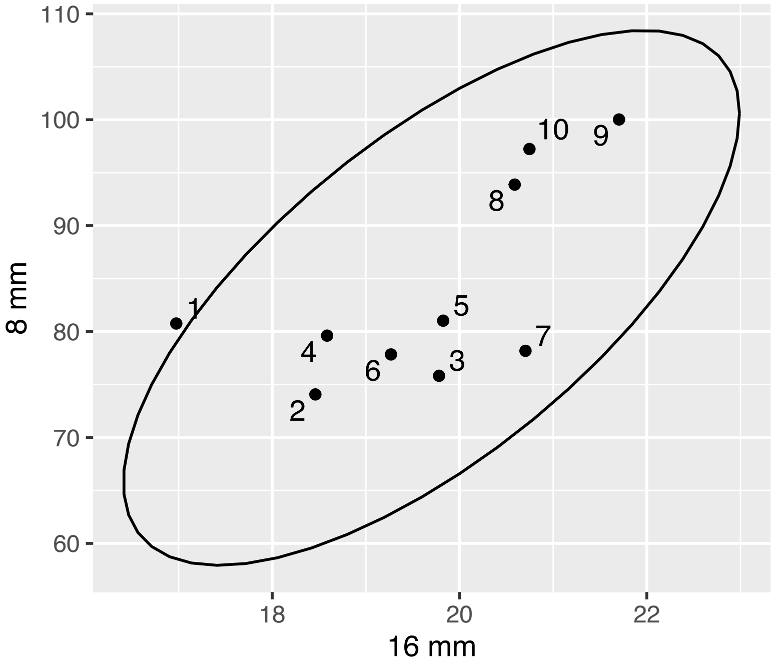

T1 glrlm highgraylevelrunemphasis

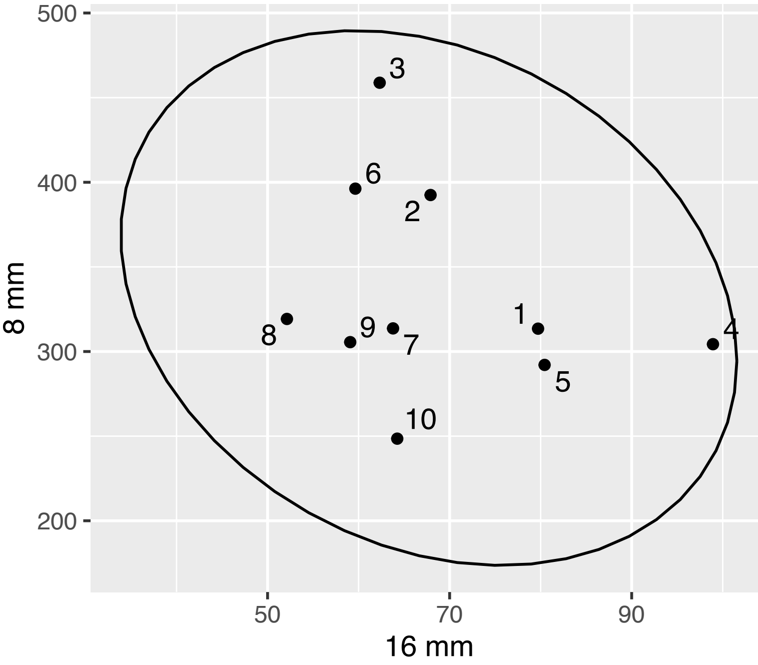

T1 glrlm graylevelnonuniformitynormalized

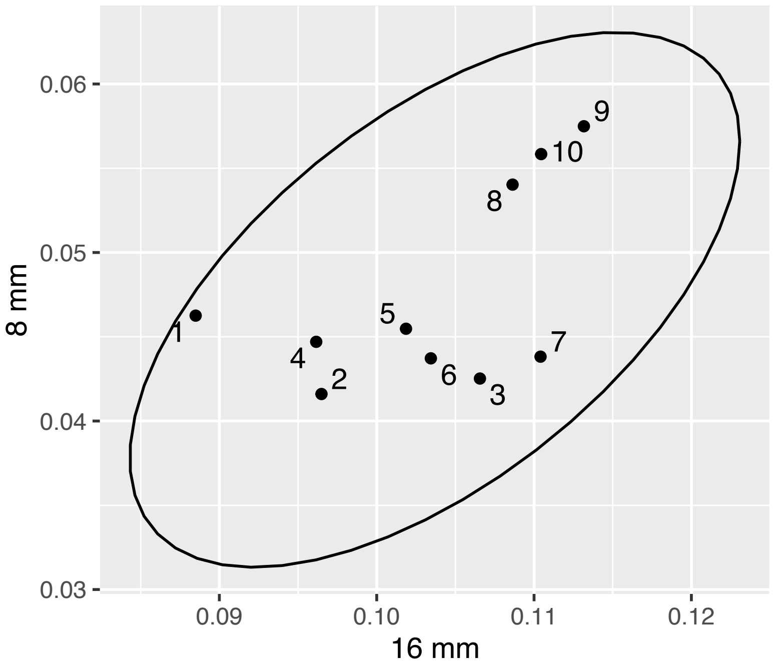

T1 glrlm longrunemphasis

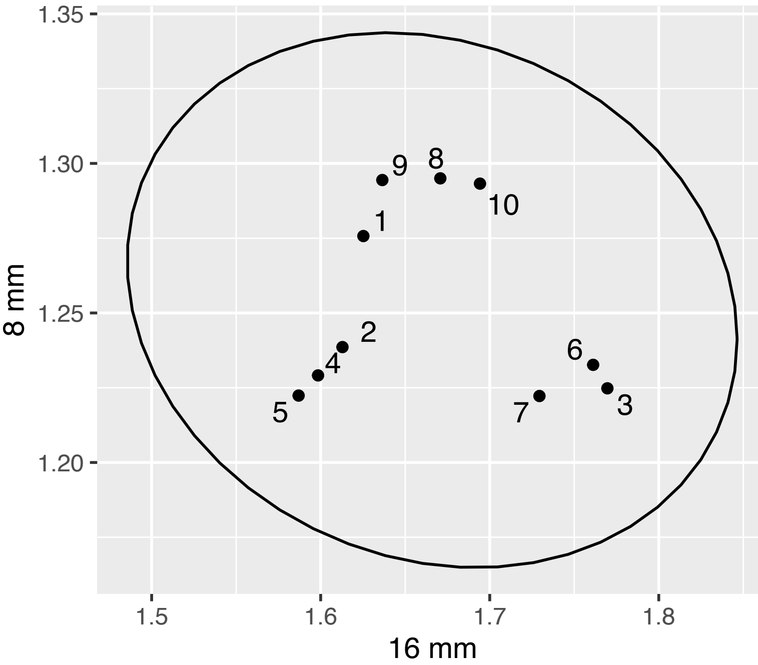

T1 glrlm graylevelvariance

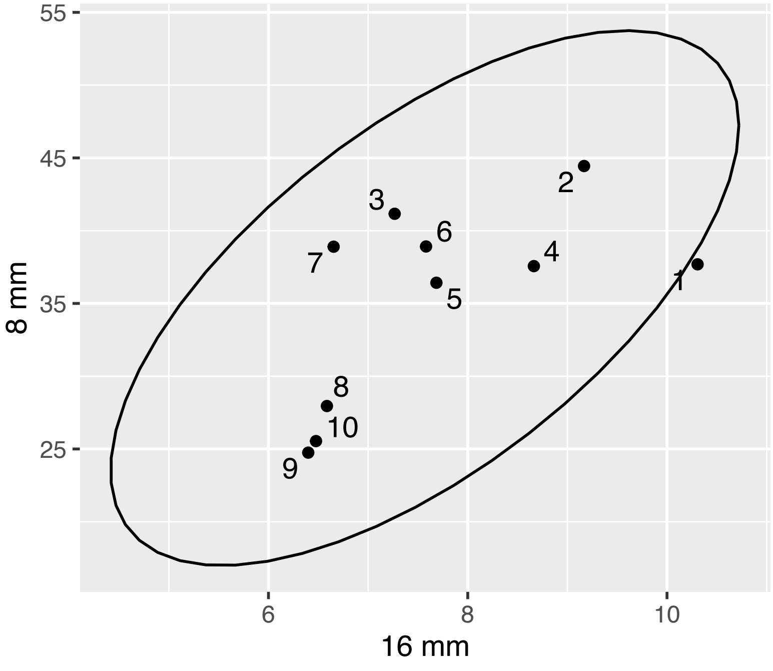

T1 glrlm longrunhighgraylevelemphasis

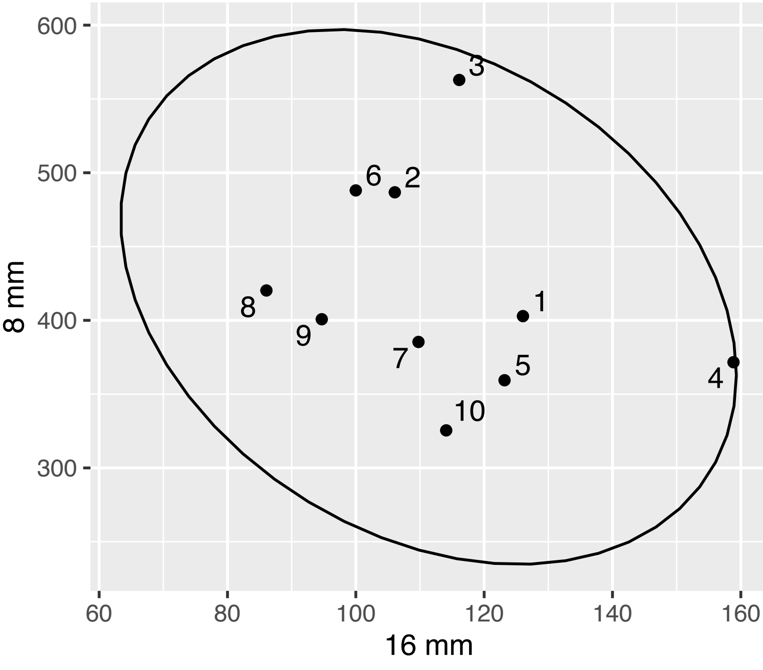

T1 glrlm longrunlowgraylevelemphasis

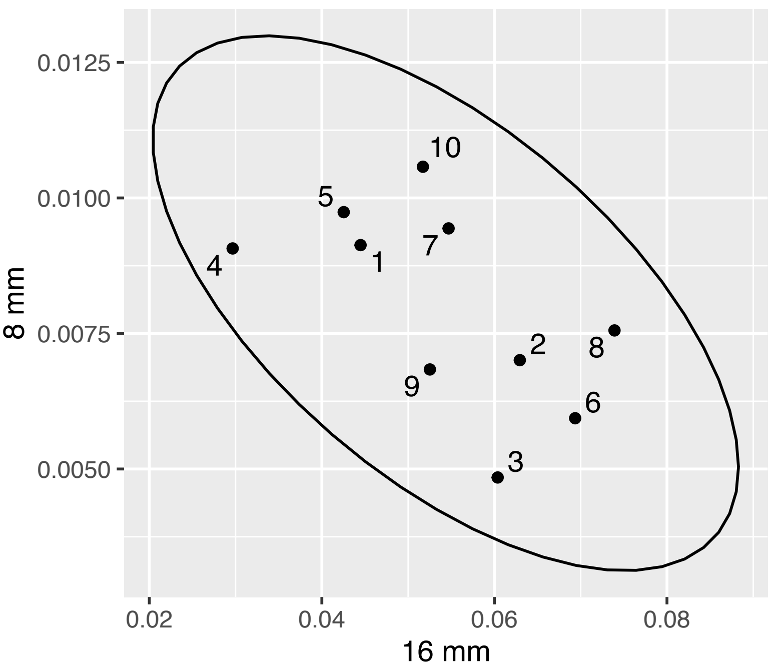

T1 glrlm runlengthnonuniformity

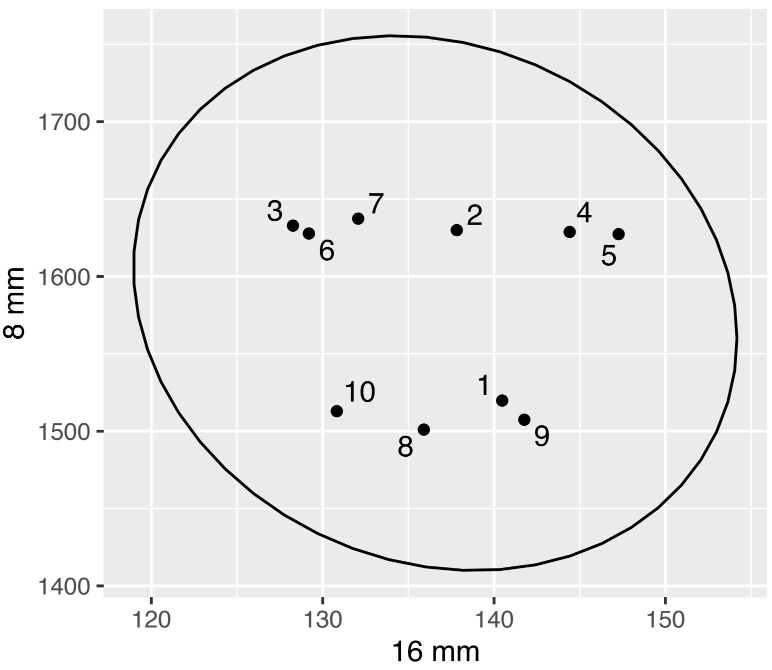

T1 glrlm lowgraylevelrunemphasis

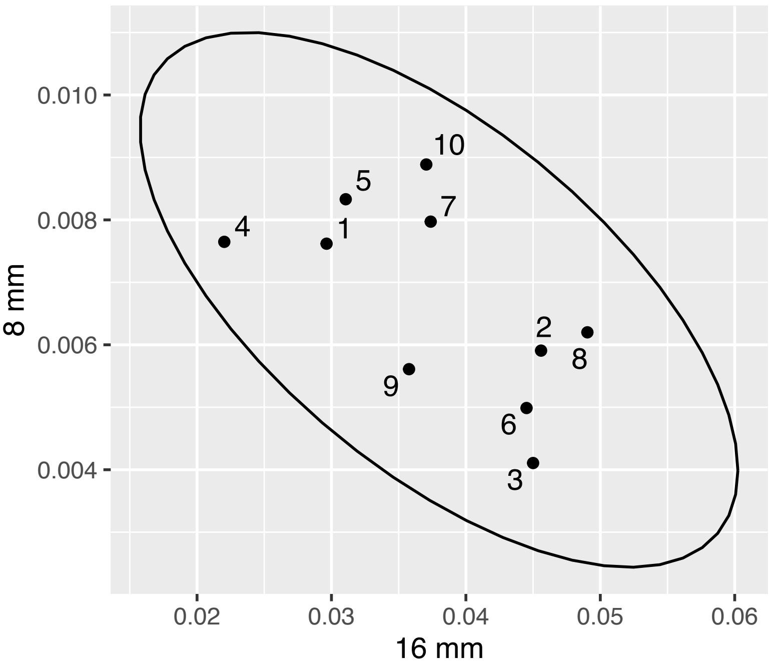

T1 glrlm runlengthnonuniformitynormalized

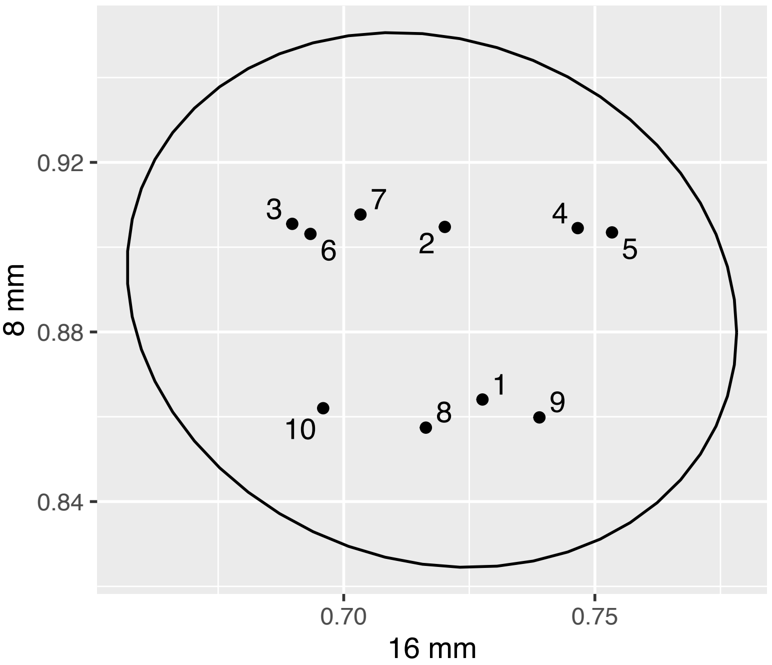

T1 glrlm runentropy

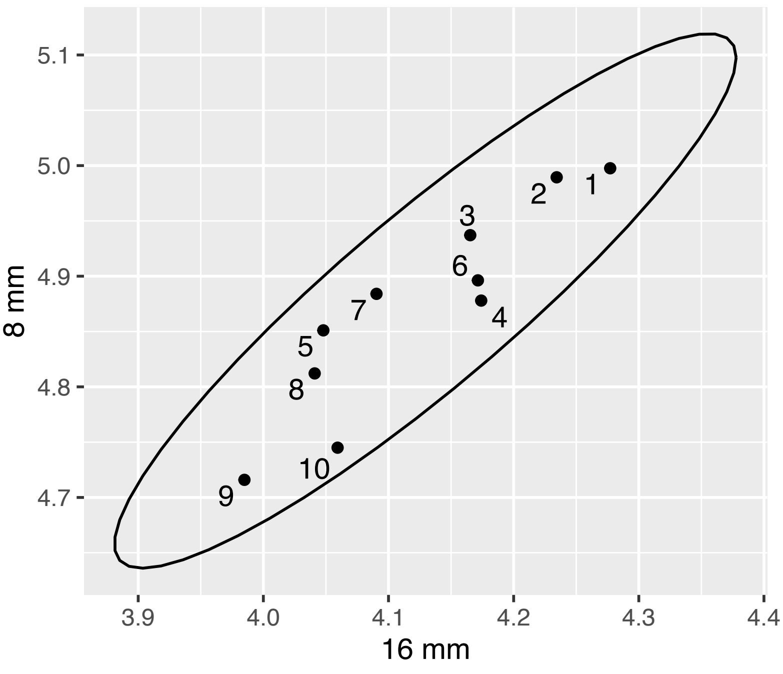

T1 glrlm runpercentage

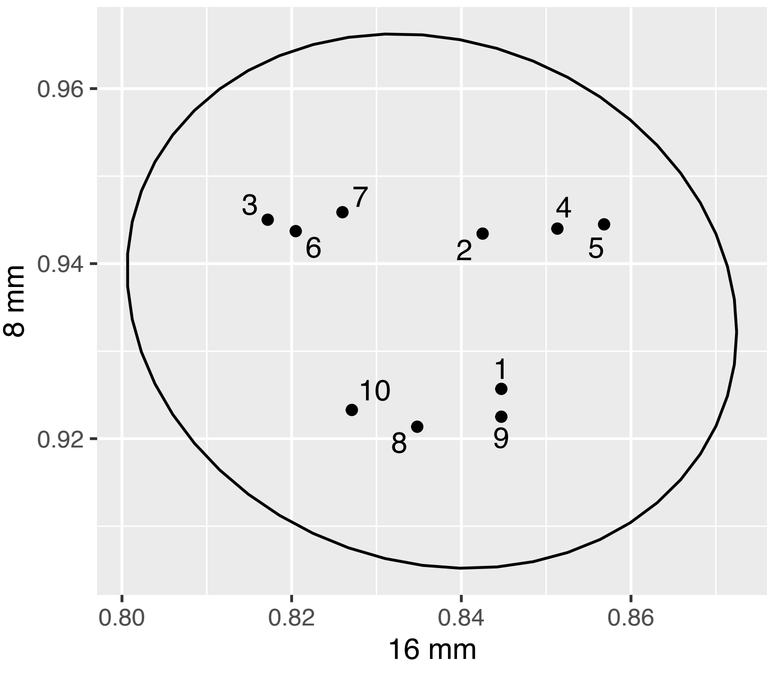

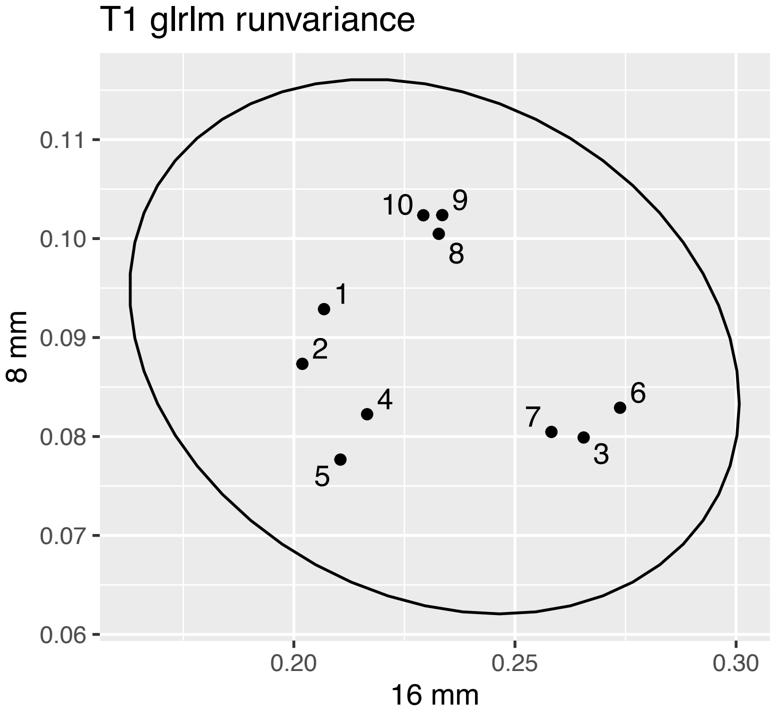
T1 glrlm runvariance
8 mm
16 mm
0.06
0.07
0.08
0.09
0.10
0.11
0.20
0.25
0.30
10
9
8
1
2
4
5
7
6
3

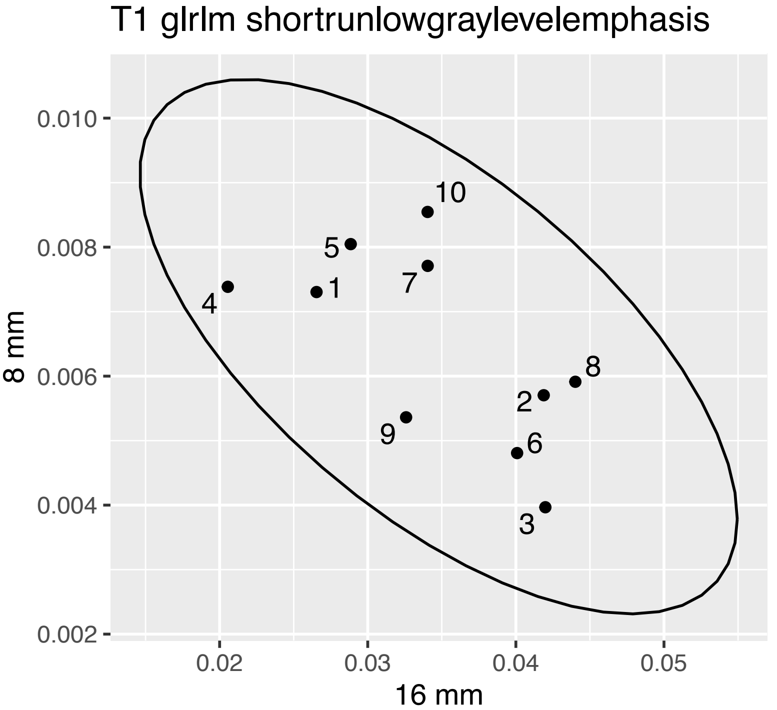
T1 glrlm shortrunlowgraylevelemphasis
8 mm
16 mm
0.002
0.004
0.006
0.008
0.010
0.02
0.03
0.04
0.05
10
5
7
4
1
8
2
9
6
3

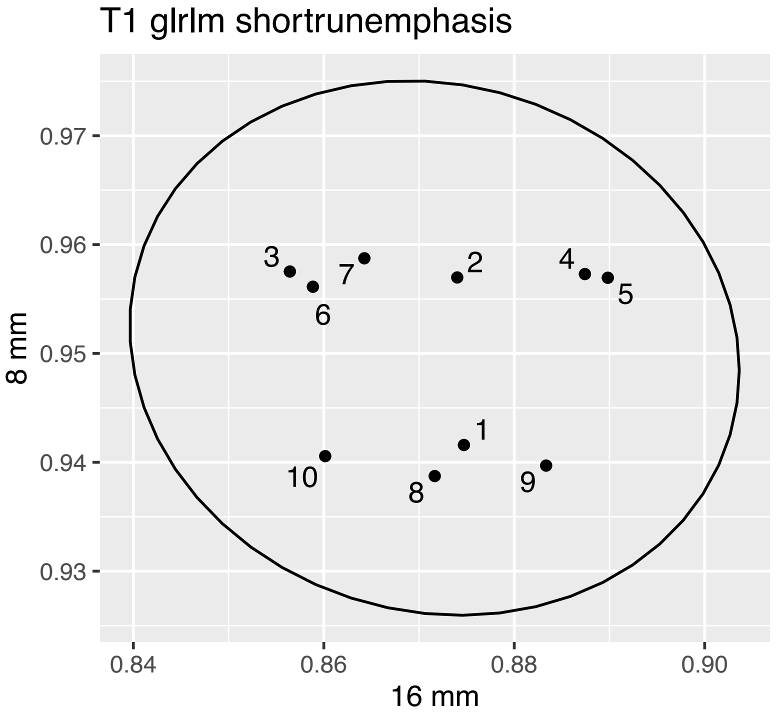
T1 glrlm shortrunemphasis
8 mm
16 mm
0.93
0.94
0.95
0.96
0.97
0.84
0.86
0.88
0.90
3
7
6
2
4
5
1
10
8
9

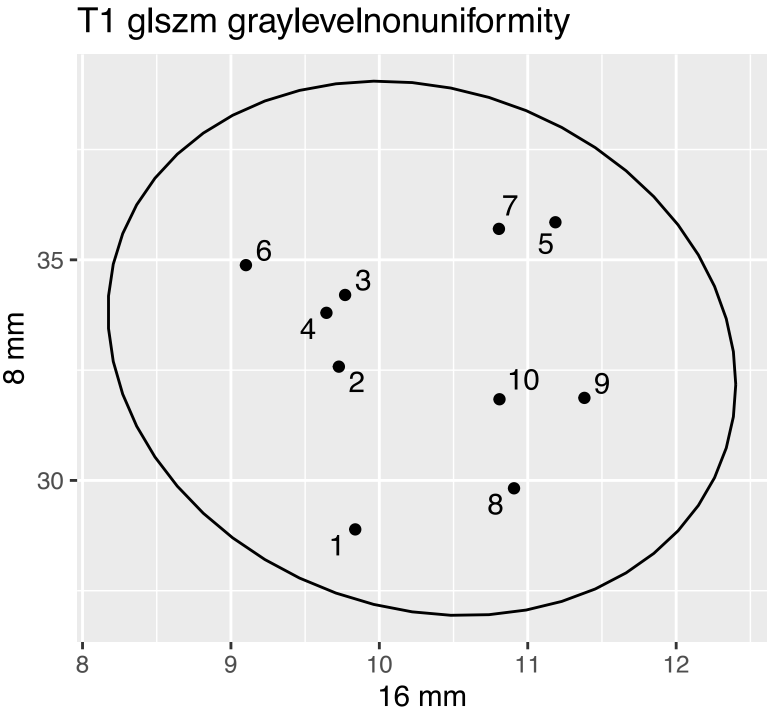
T1 glszm graylevelnonuniformity
8 mm
16 mm
30
35
8
9
10
11
12
7
5
6
3
4
2
10
9
8
1

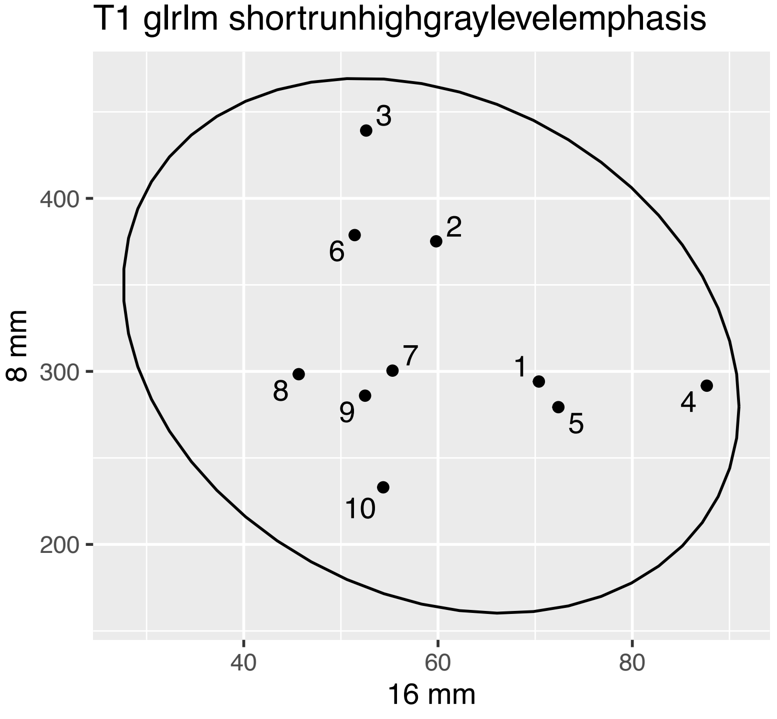
T1 glrlm shortrunhighgraylevelemphasis
8 mm
16 mm
200
300
400
40
60
80
3
6
2
7
8
9
1
5
4
10

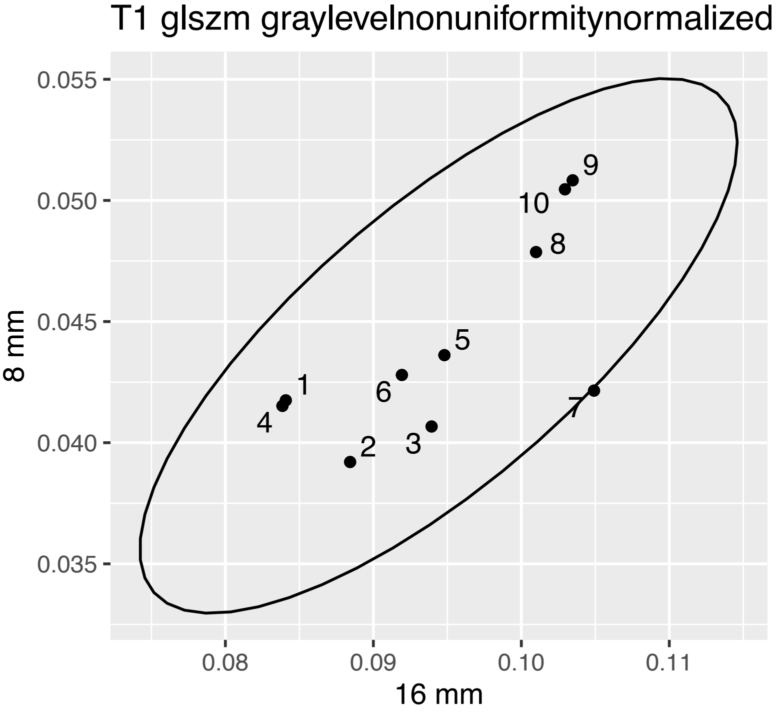
T1 glszm graylevelnonuniformitynormalized
8 mm
16 mm
0.035
0.040
0.045
0.050
0.055
0.08
0.09
0.10
0.11
9
10
8
5
1
6
4
2
3
7

T1 glszm graylevelvariance

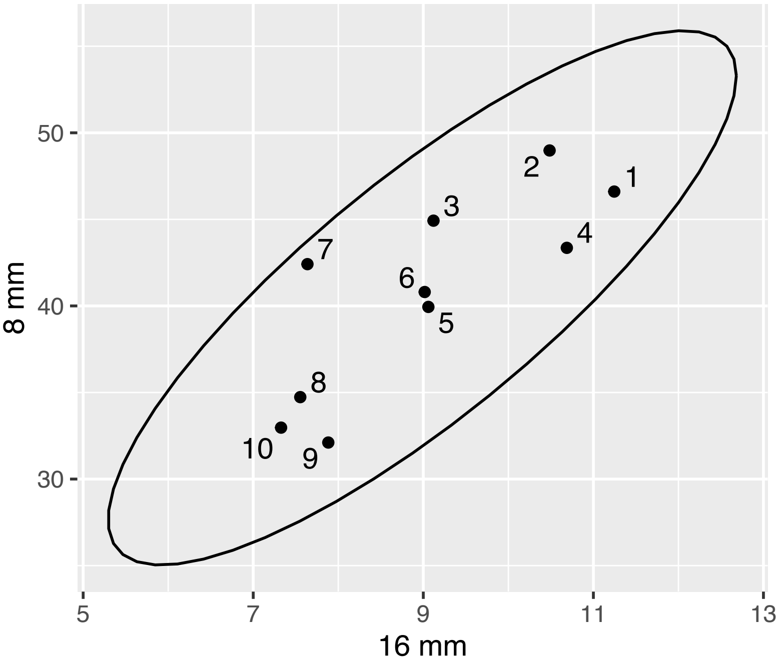

T1 glszm largeareahighgraylevelemphasis

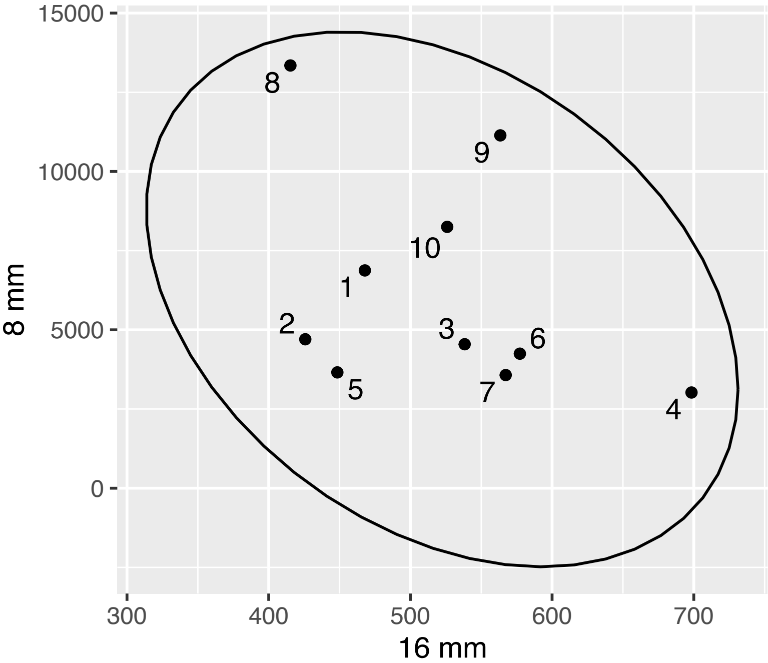

T1 glszm highgraylevelzoneemphasis

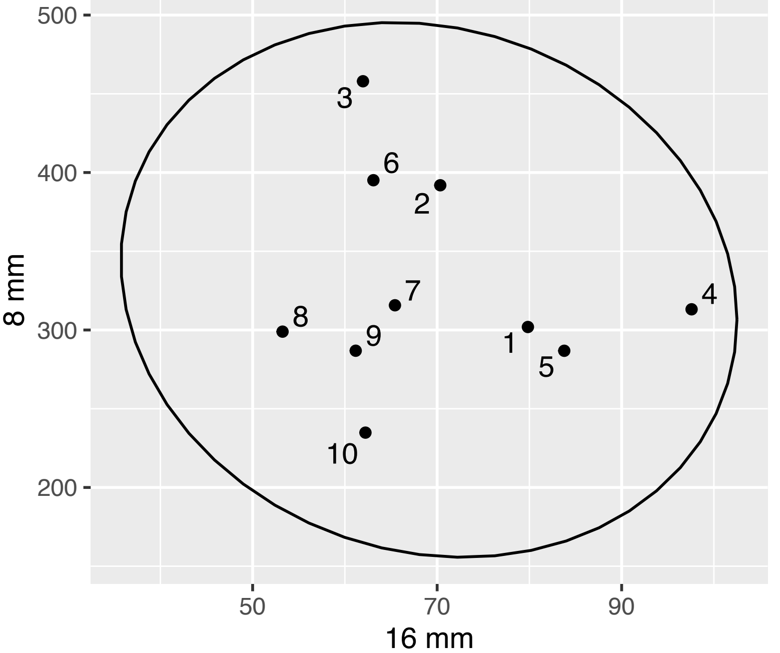

T1 glszm largearealowgraylevelemphasis

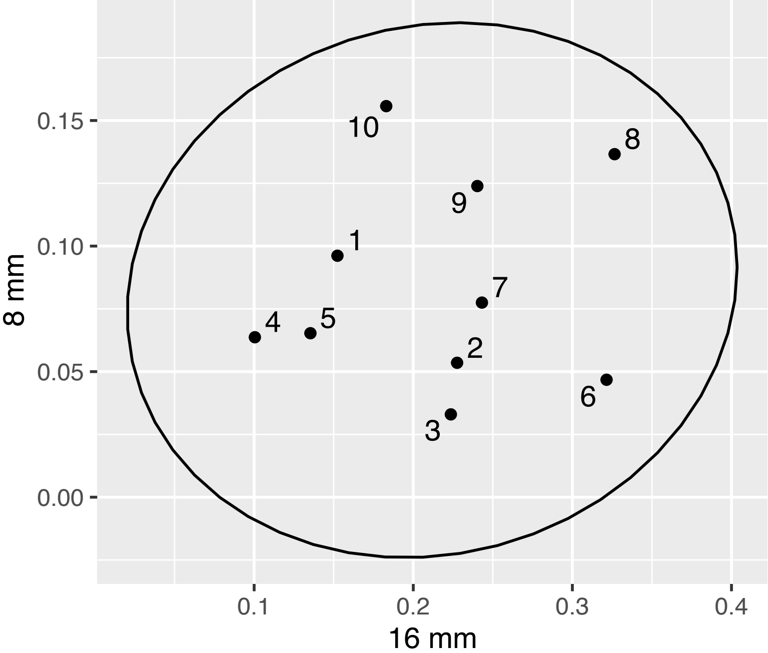

T1 glszm largeareaemphasis

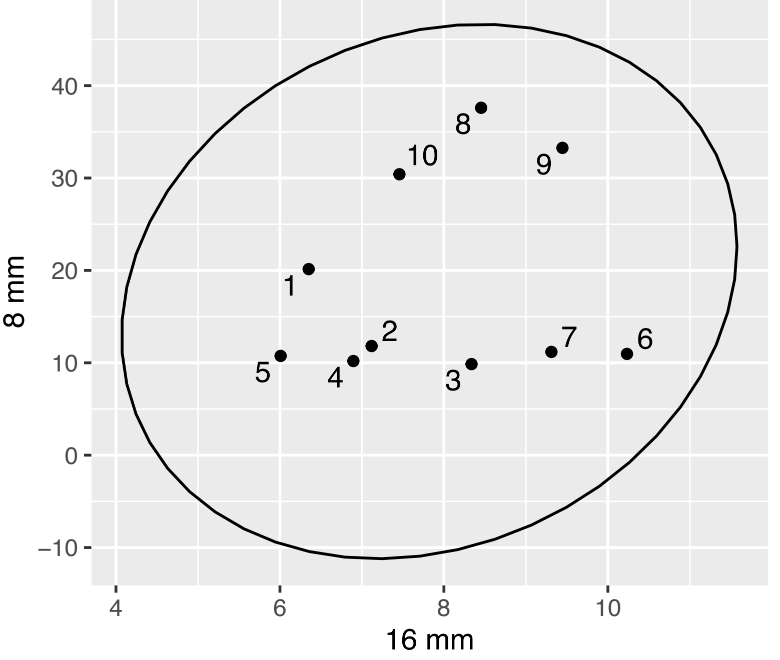

T1 glszm lowgraylevelzoneemphasis

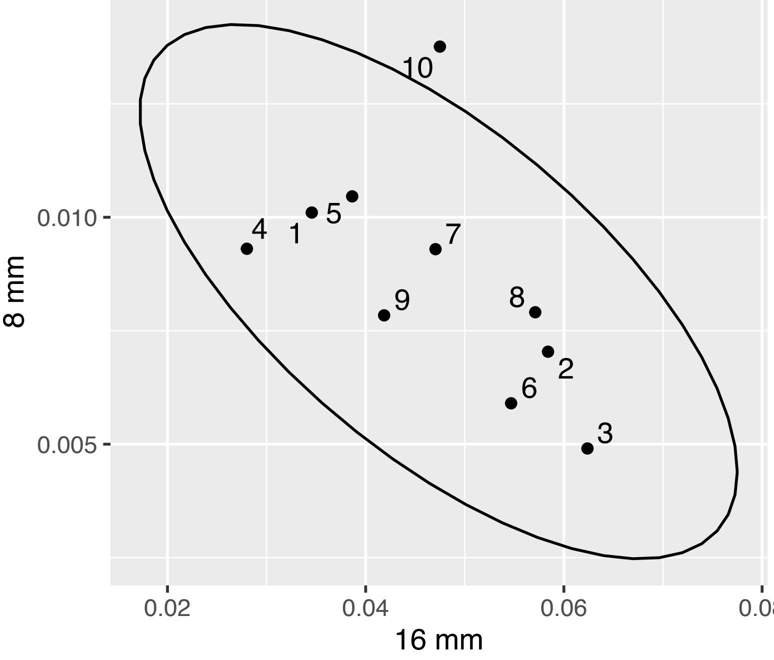

T1 glszm sizezonenonuniformity

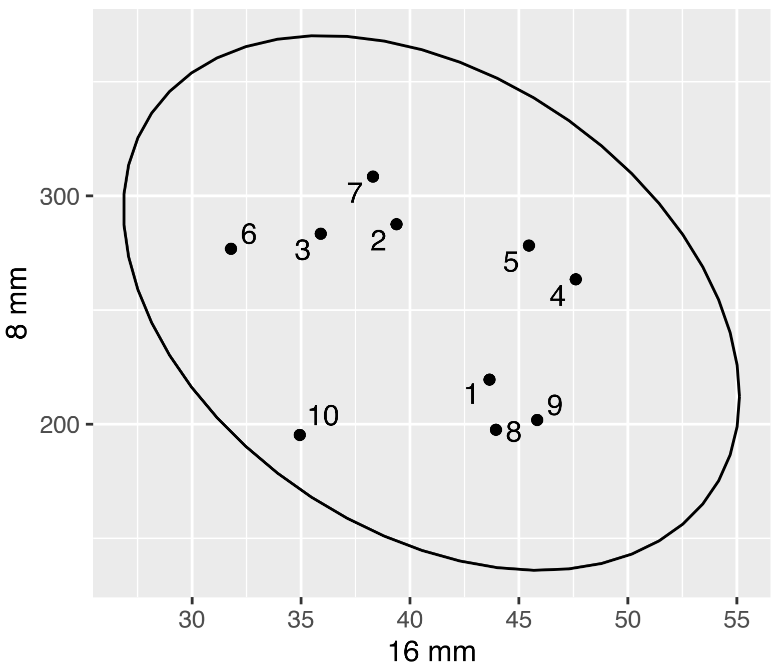

T1 glszm smallareahighgraylevelemphasis

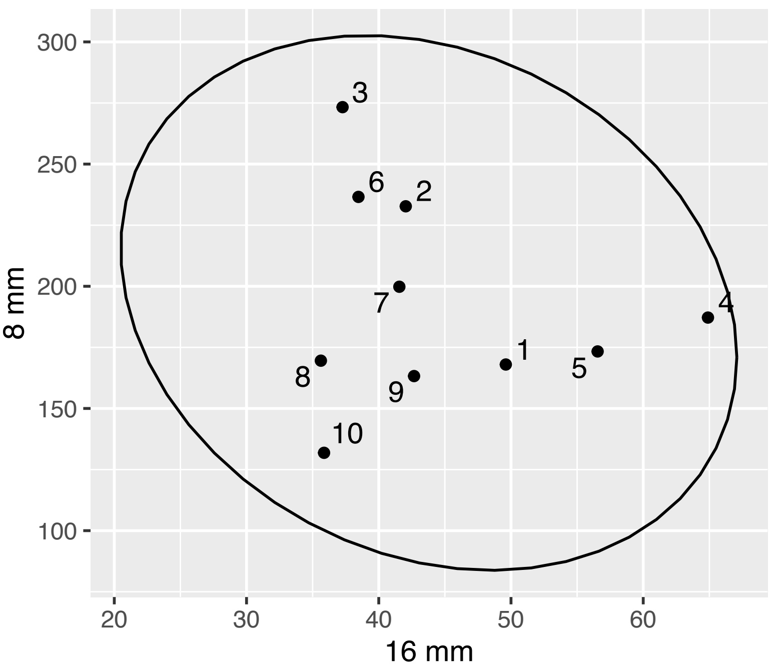

T1 glszm sizezonenonuniformitynormalized

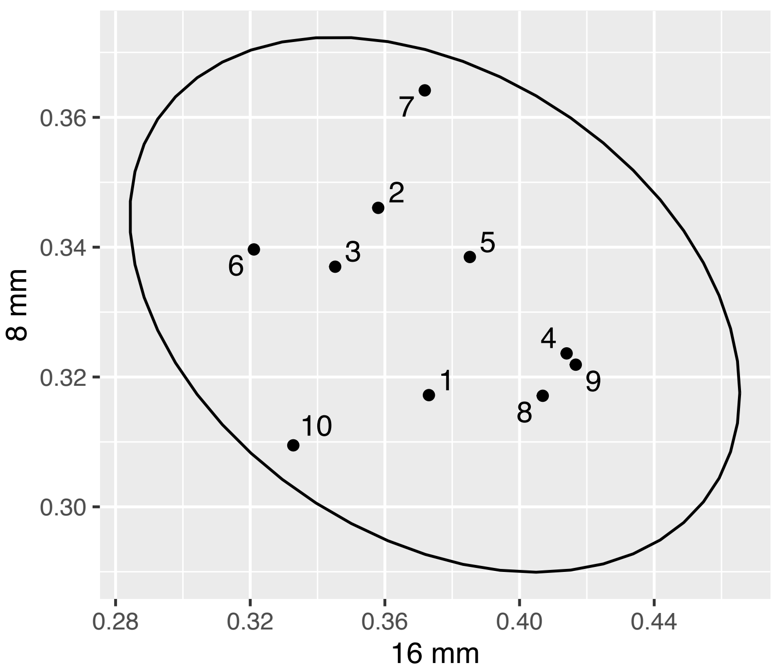

T1 glszm smallarealowgraylevelemphasis

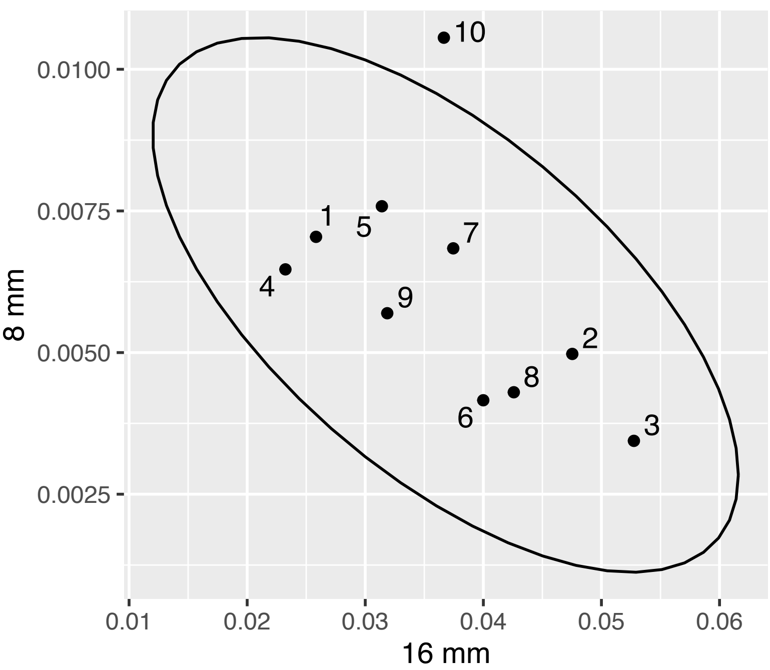

T1 glszm smallareaemphasis

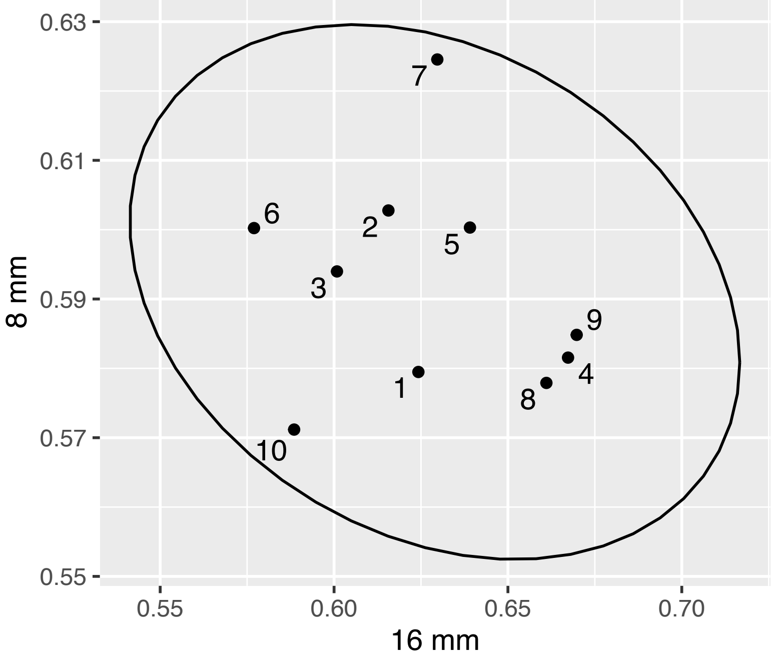

T1 glszm zoneentropy

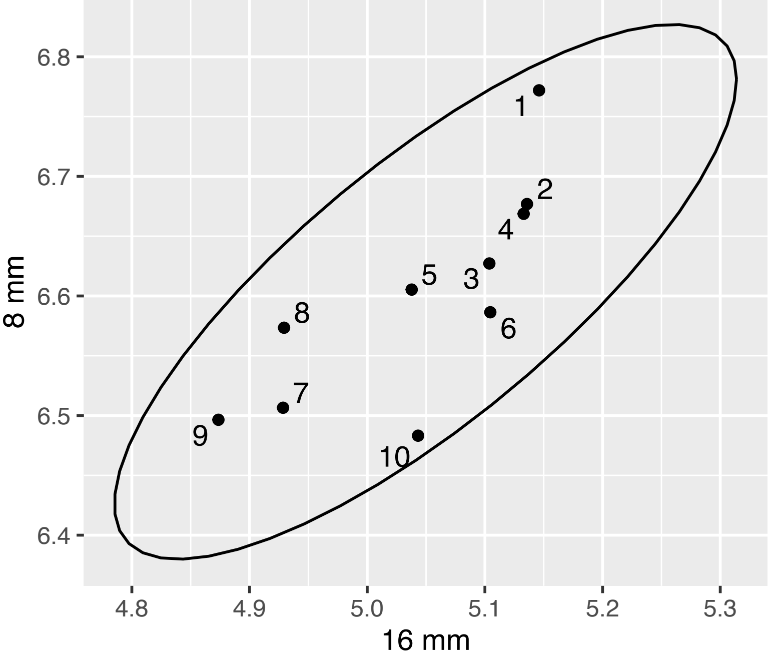

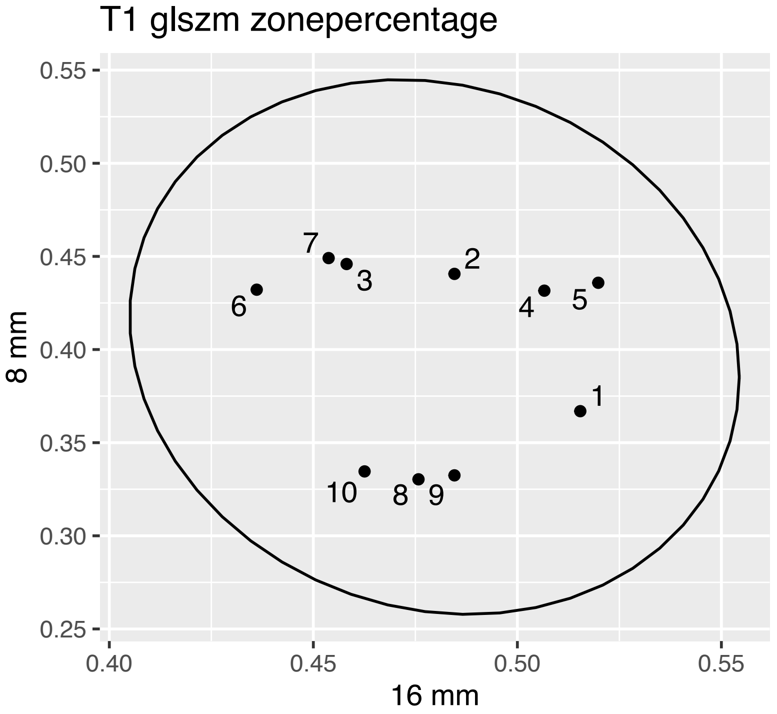
T1 glszm zonepercentage
8 mm
16 mm

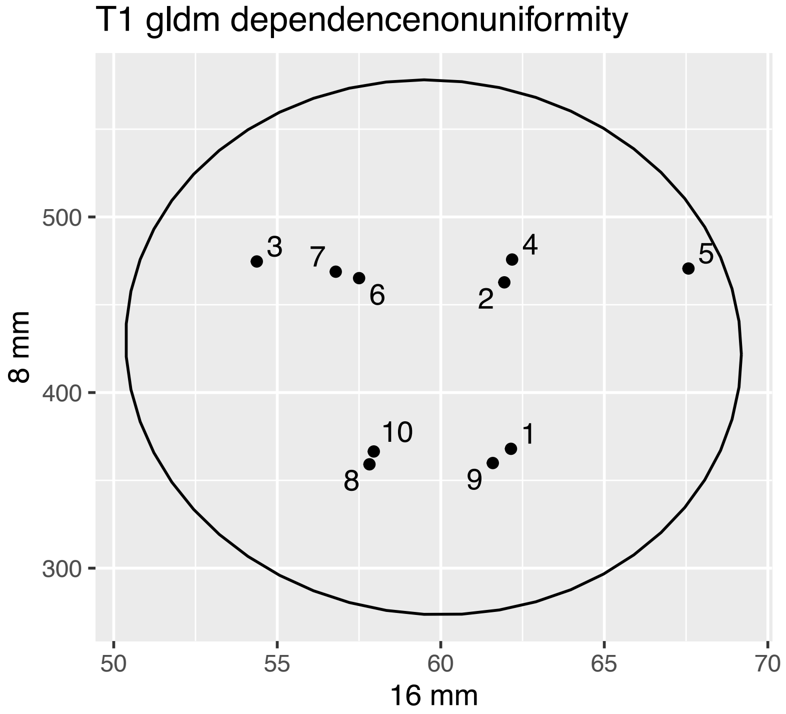
T1 gldm dependencenonuniformity
8 mm
16 mm

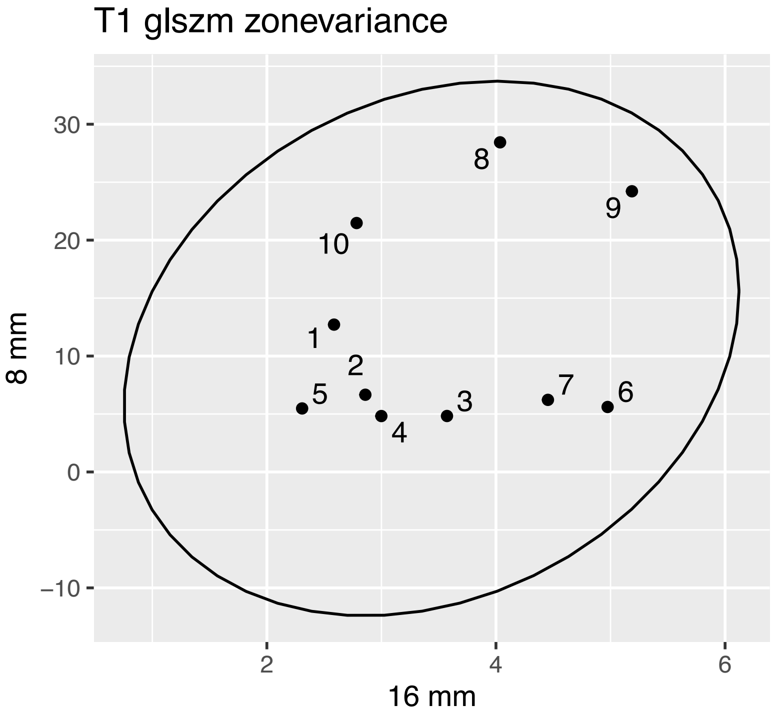
T1 glszm zonevariance
8 mm
16 mm

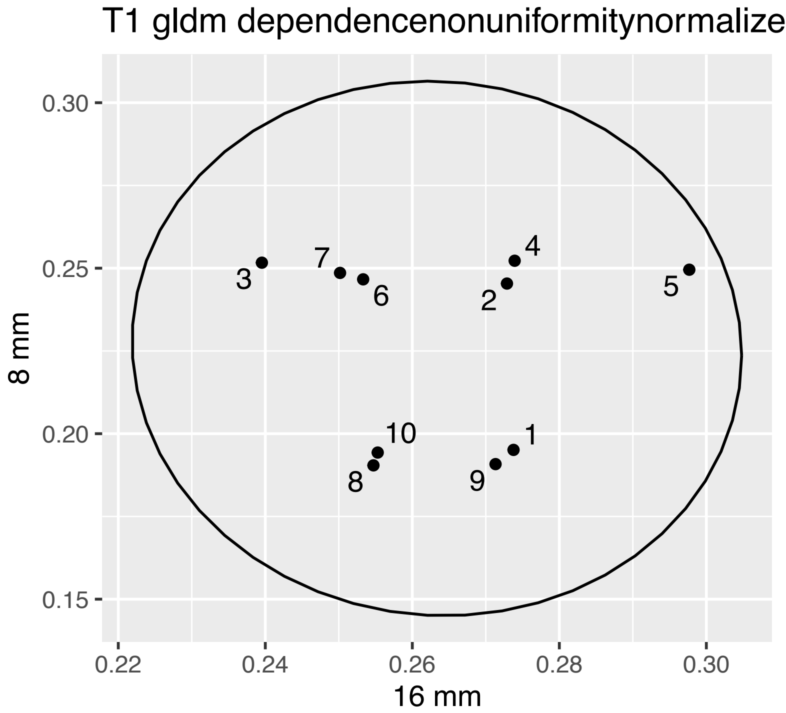
T1 gldm dependencenonuniformitynormalize
8 mm
16 mm

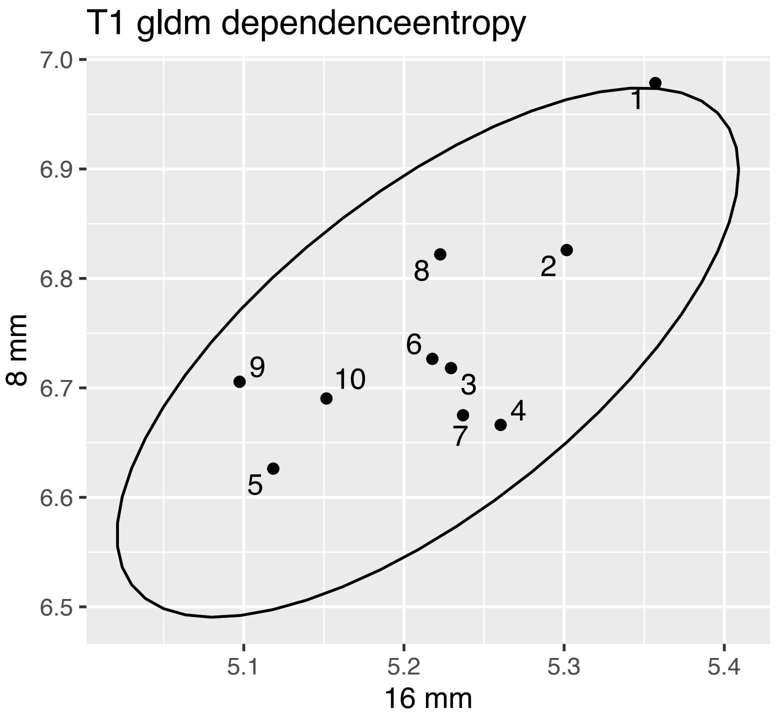
T1 gldm dependenceentropy
8 mm
16 mm

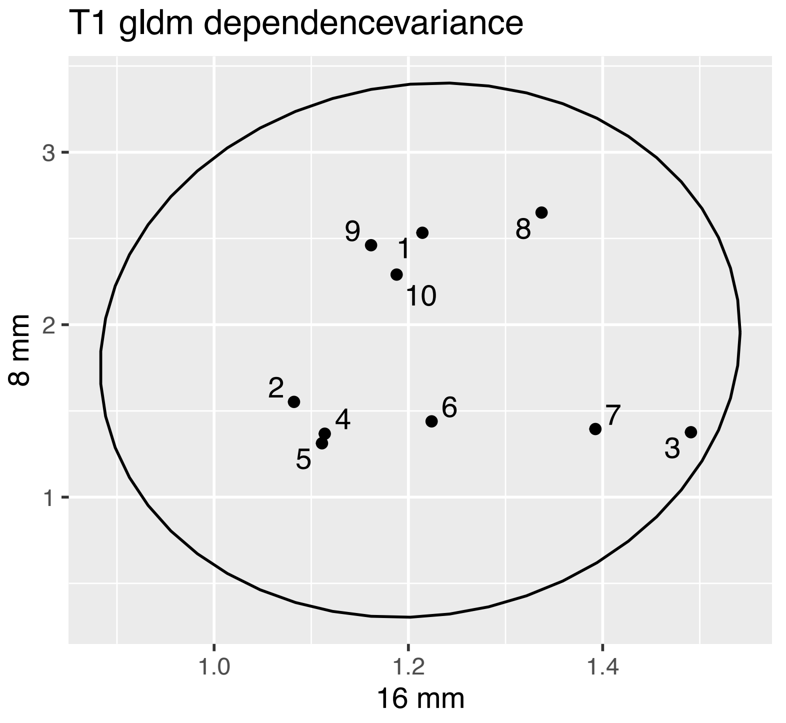
T1 gldm dependencevariance
8 mm
16 mm

T1 gldm graylevelnonuniformity

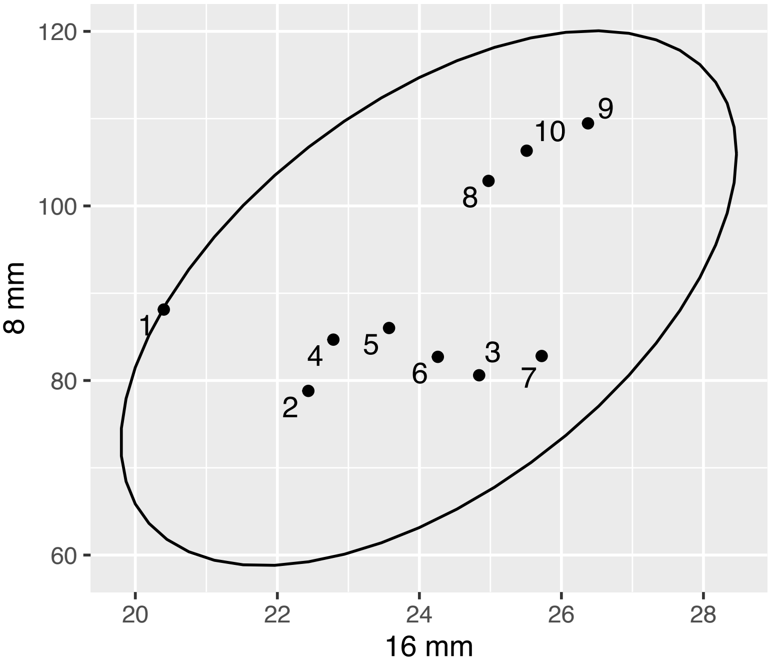

T1 gldm largedependenceemphasis

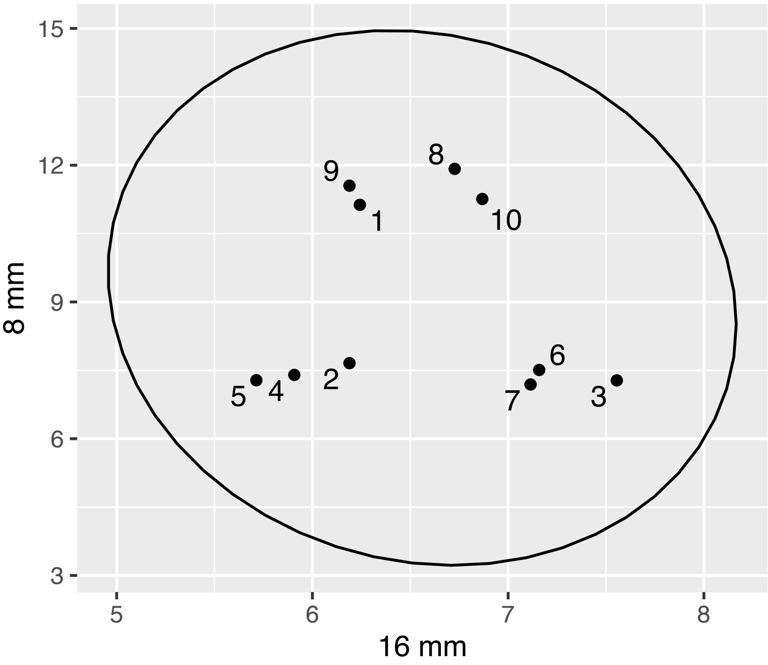

T1 gldm graylevelvariance

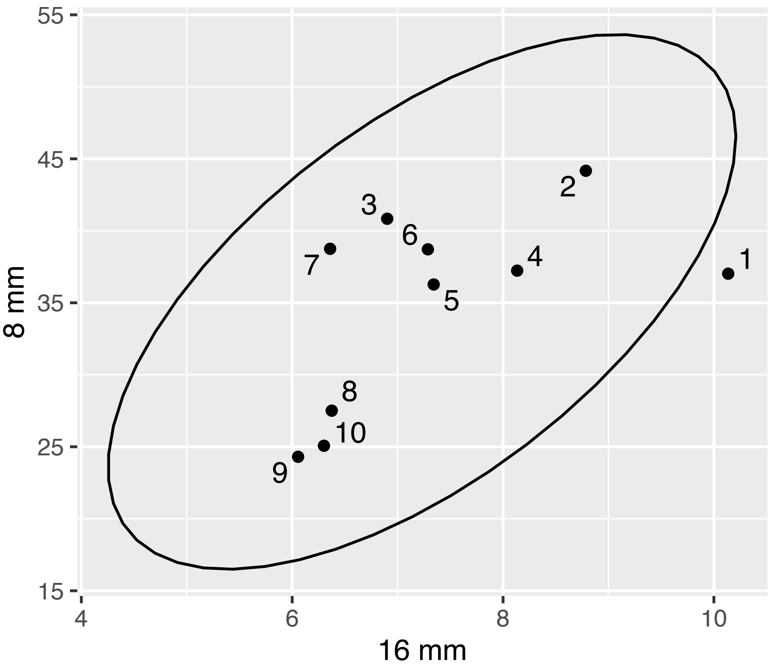

T1 gldm largedependencehighgraylevelempl

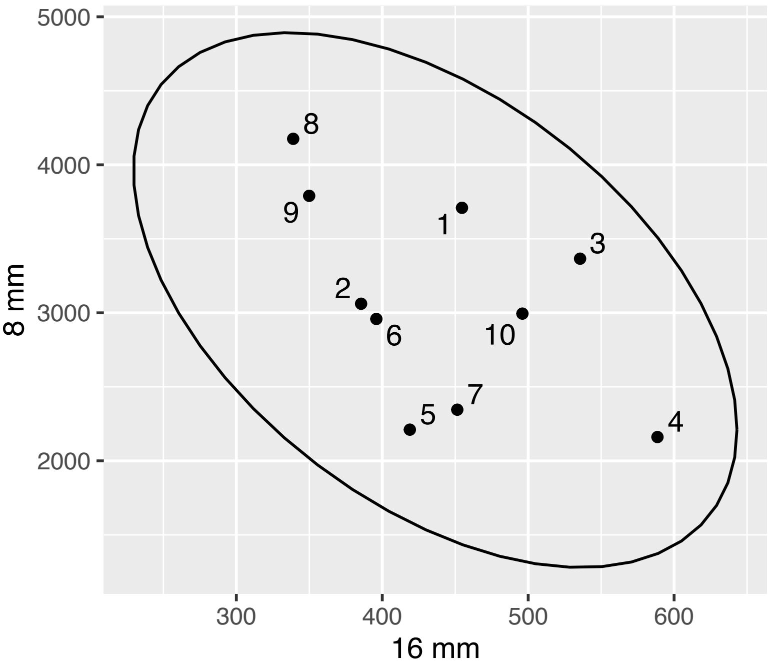

T1 gldm highgraylevelemphasis

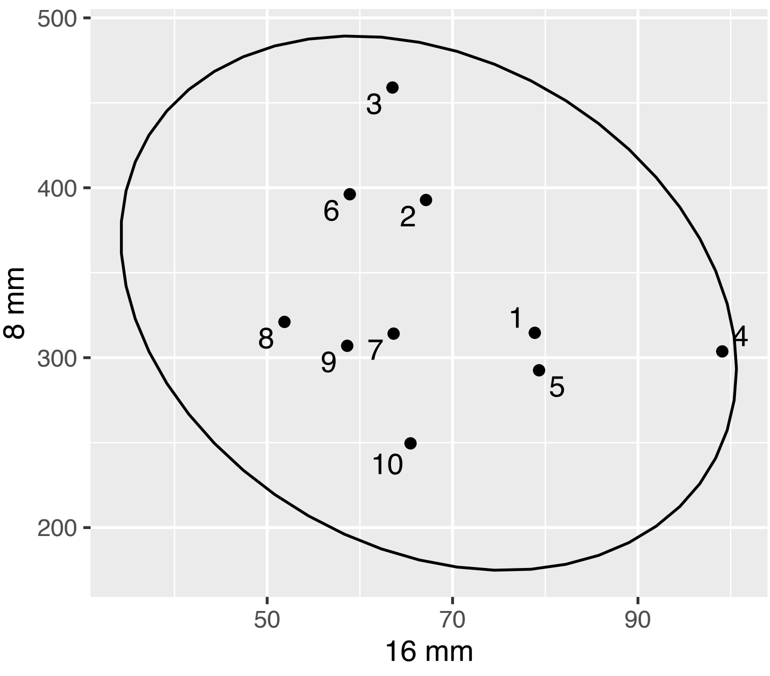

T1 gldm largedependencelowgraylevelempha

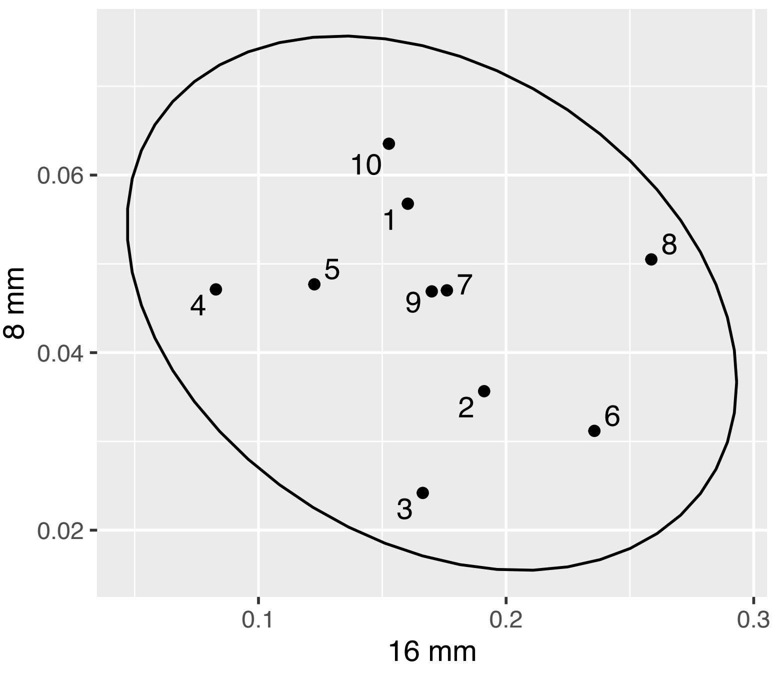

T1 gldm lowgraylevelemphasis

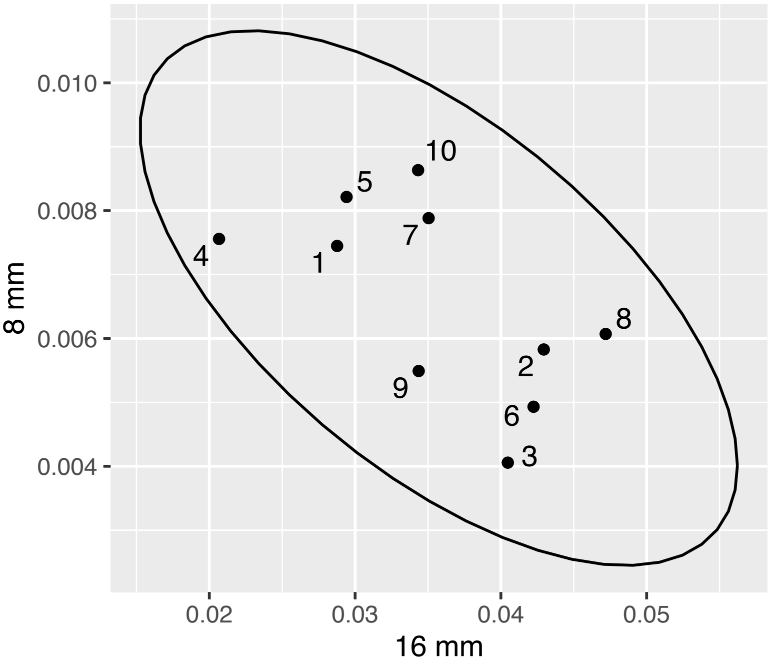

T1 gldm smalldependencelowgraylevelemph

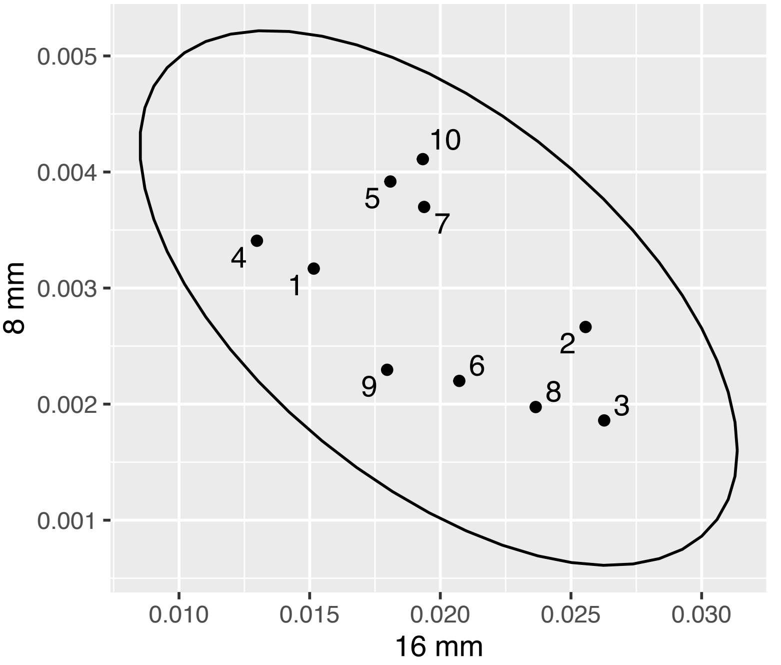

T1 gldm smalldependenceemphasis

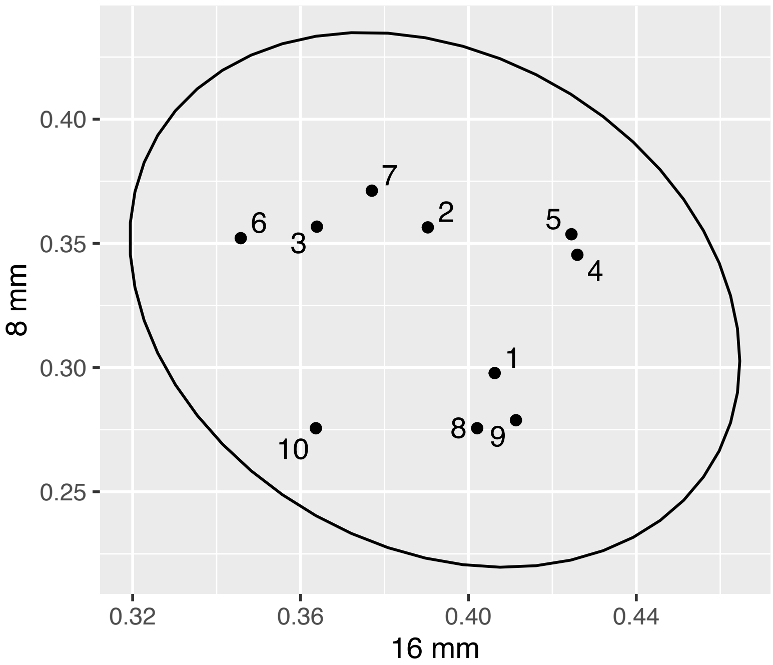

T1 ngtdm busyness

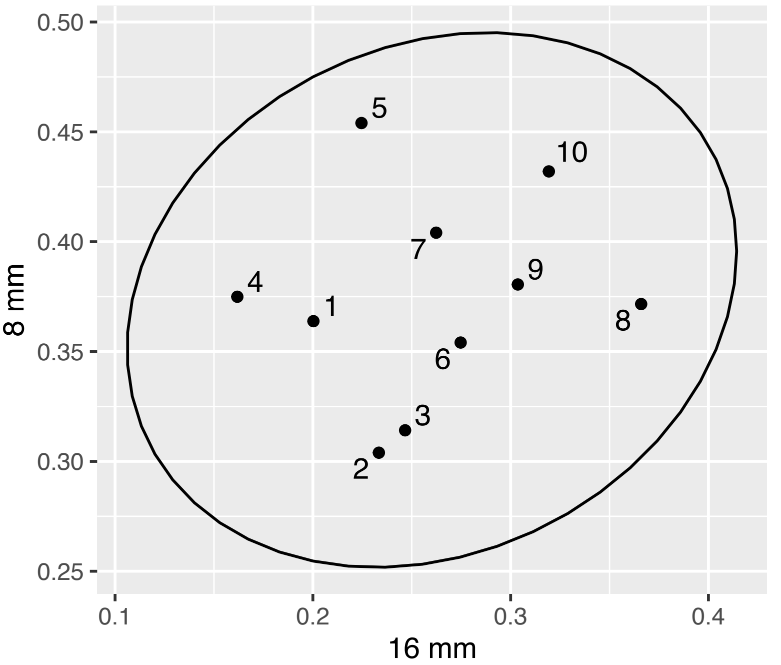

T1 gldm smalldependencehighgraylevelemph

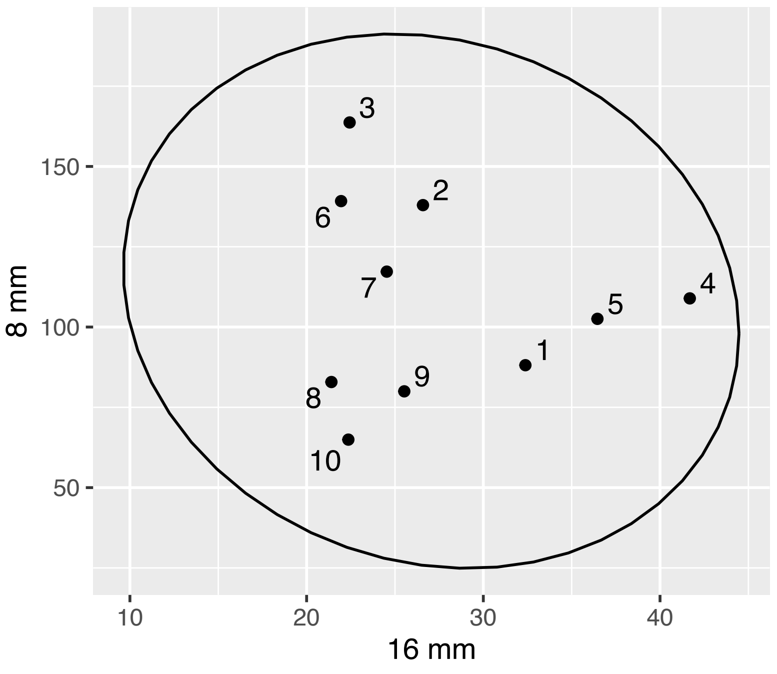

T1 ngtdm coarseness

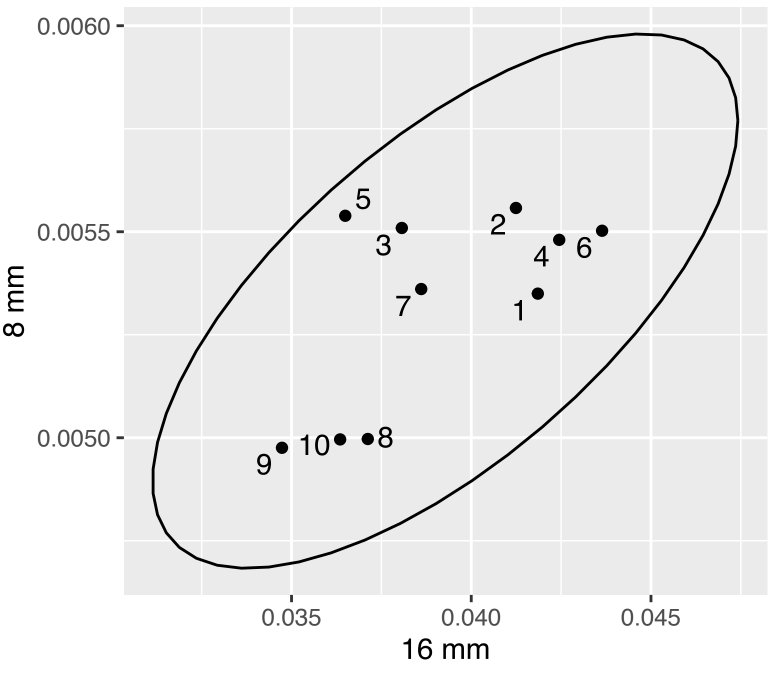

T1 ngtdm complexity

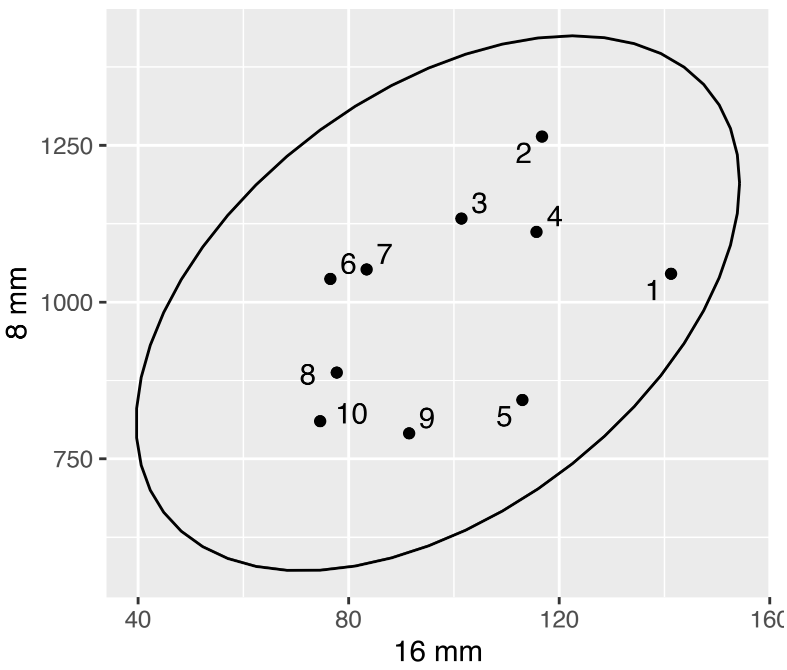

T1 firstorder 10percentile

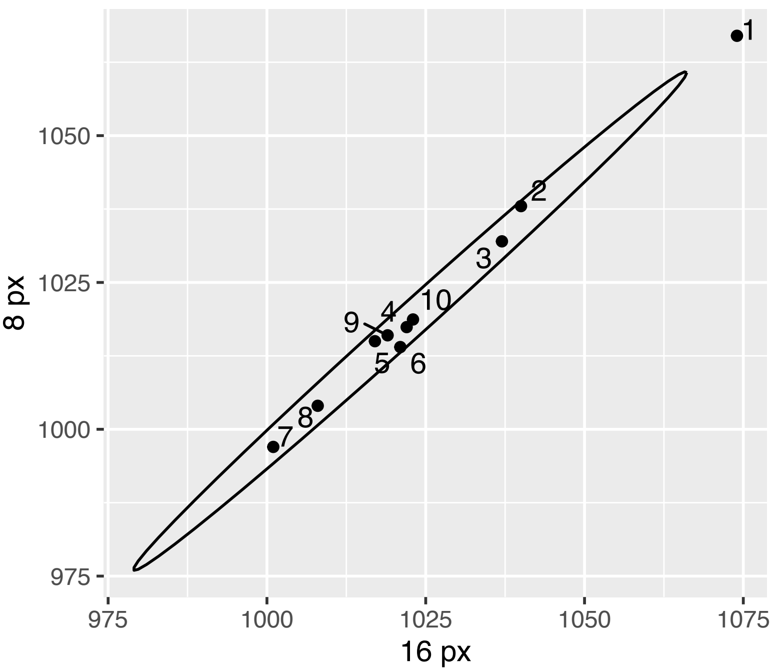

T1 ngtdm contrast

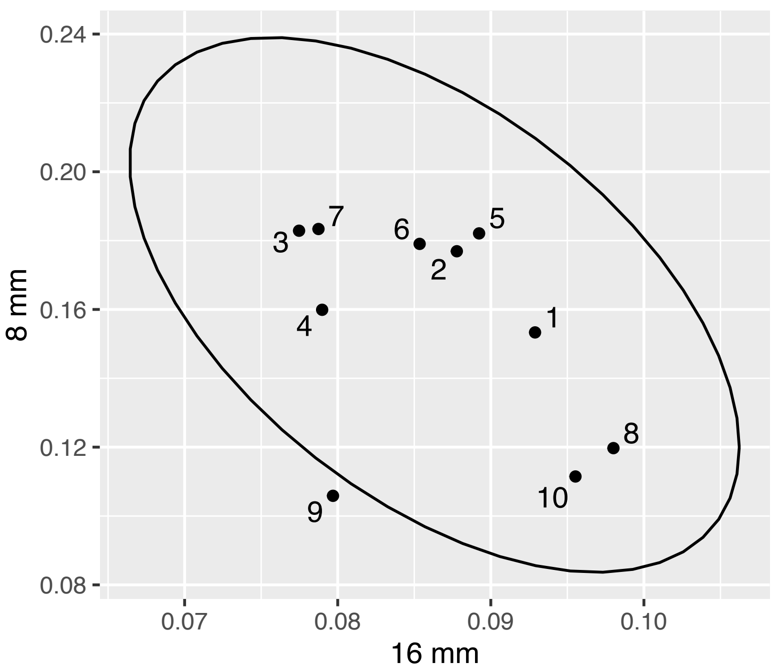

T1 firstorder 90percentile

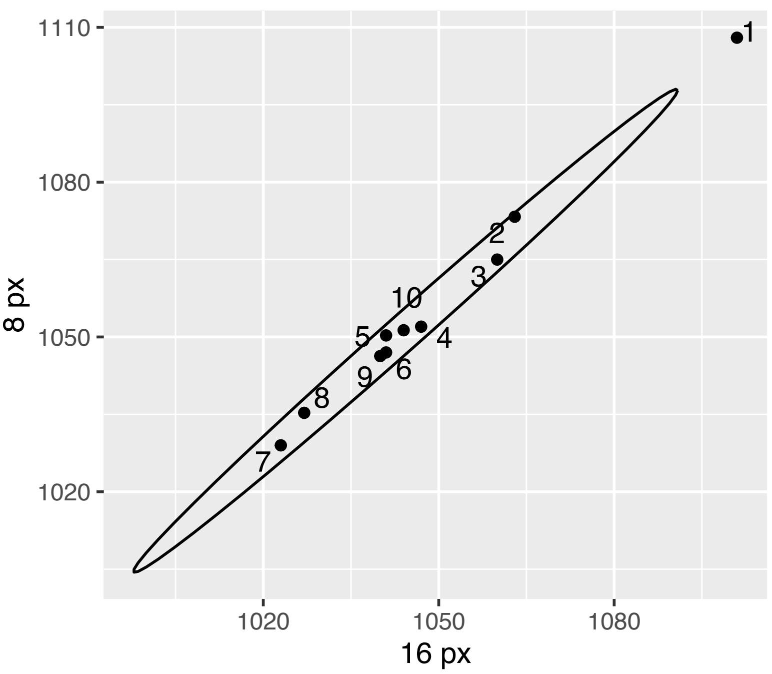

T1 ngtdm strength

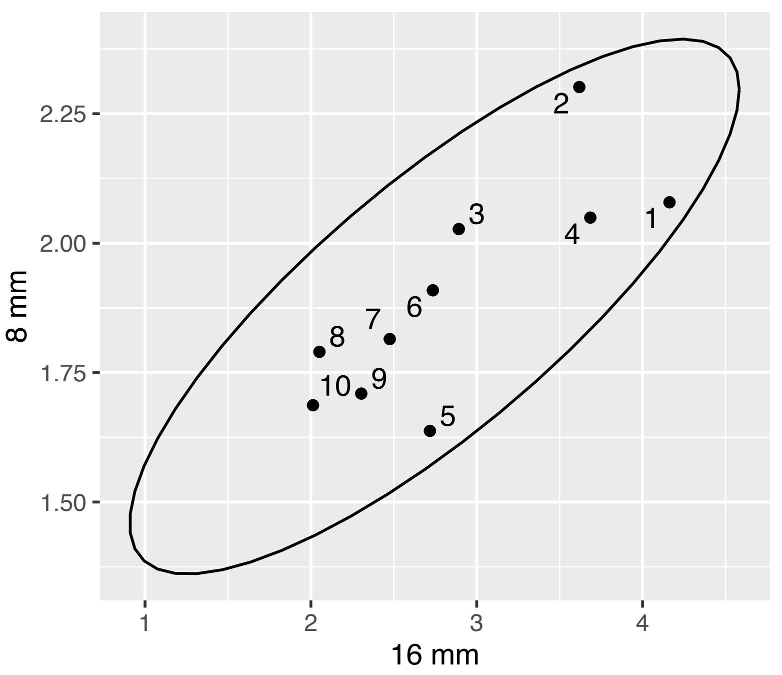

T1 firstorder energy

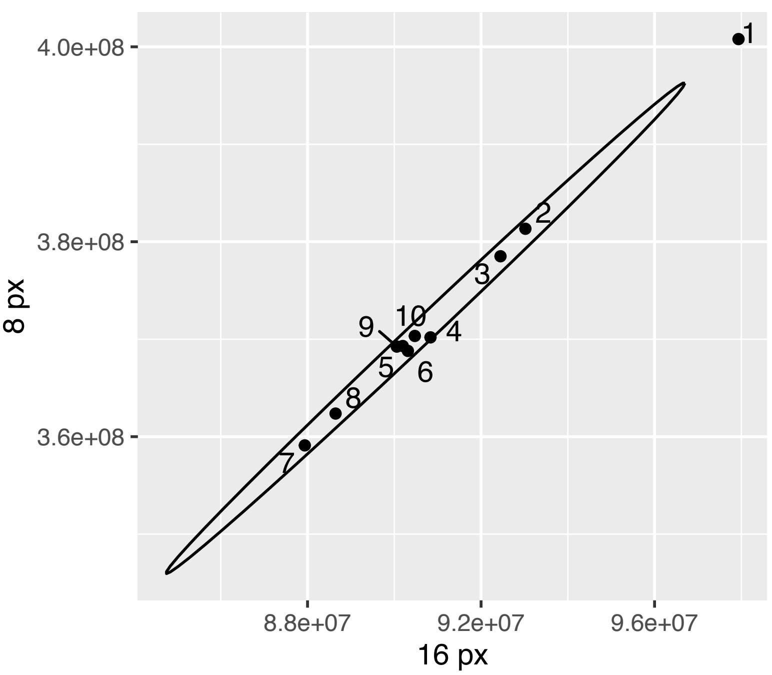

T1 firstorder entropy

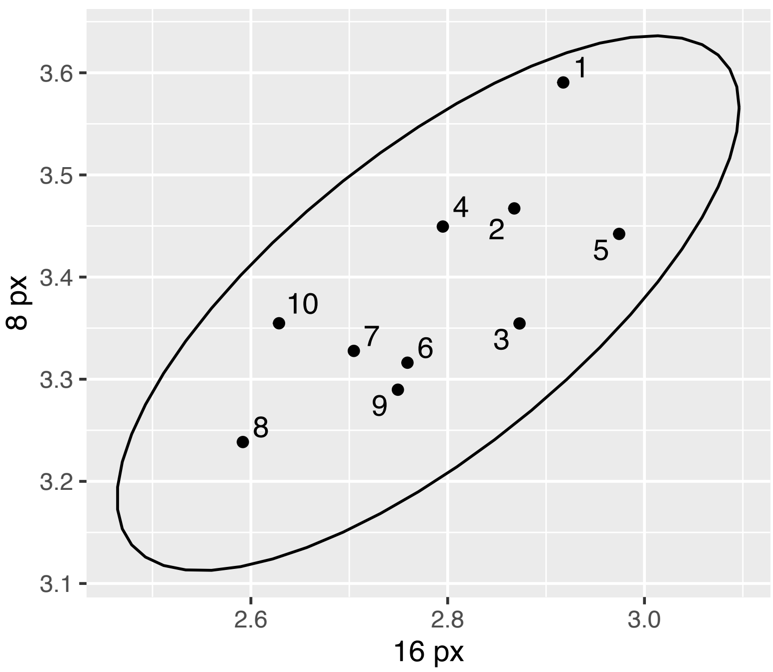

T1 firstorder maximum

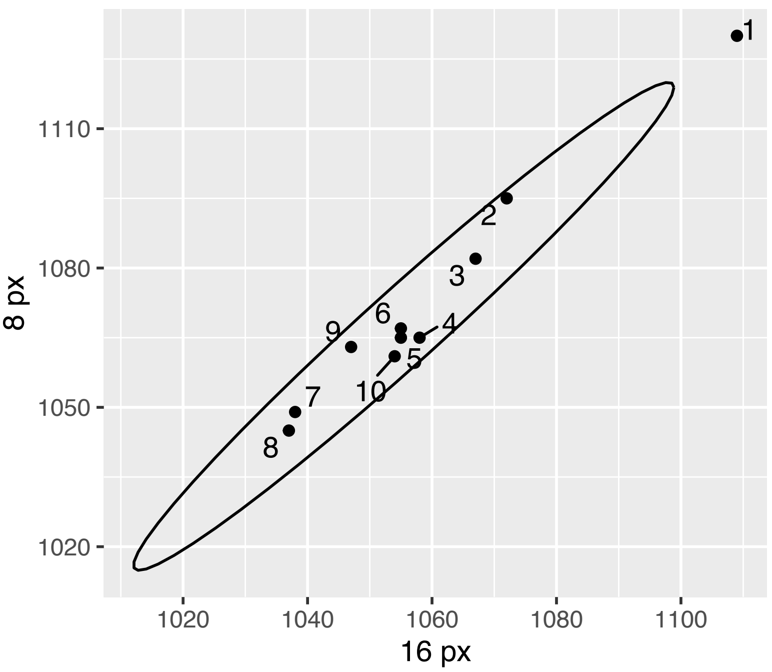

T1 firstorder interquartilerange

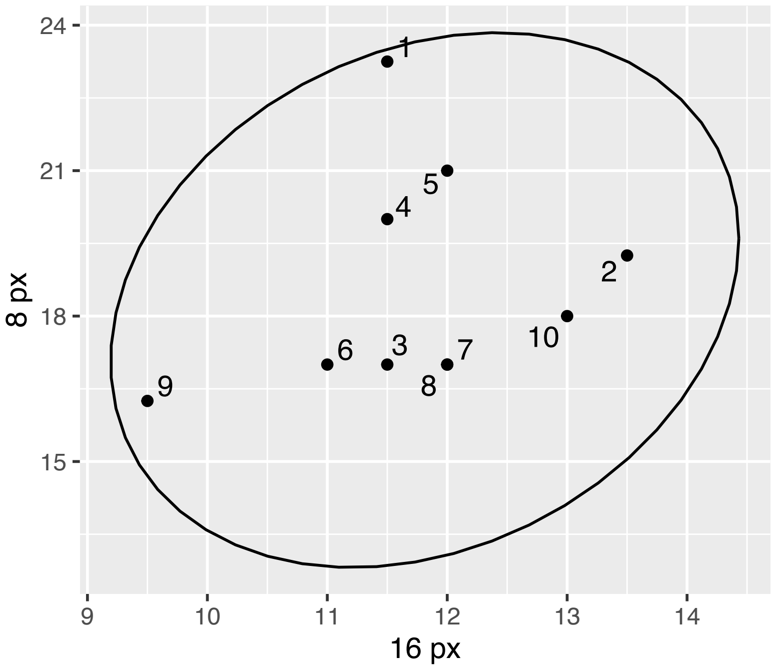

T1 firstorder meanabsolutedeviation

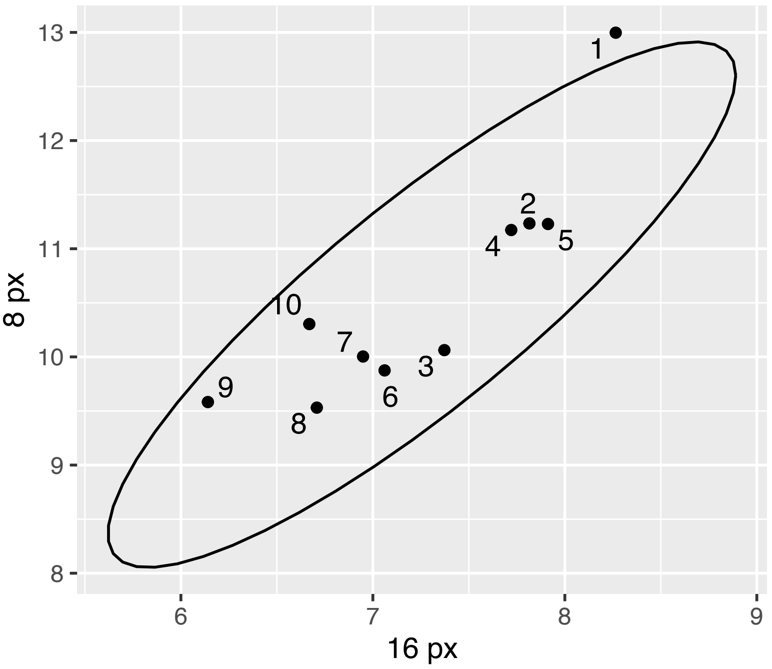

T1 firstorder kurtosis

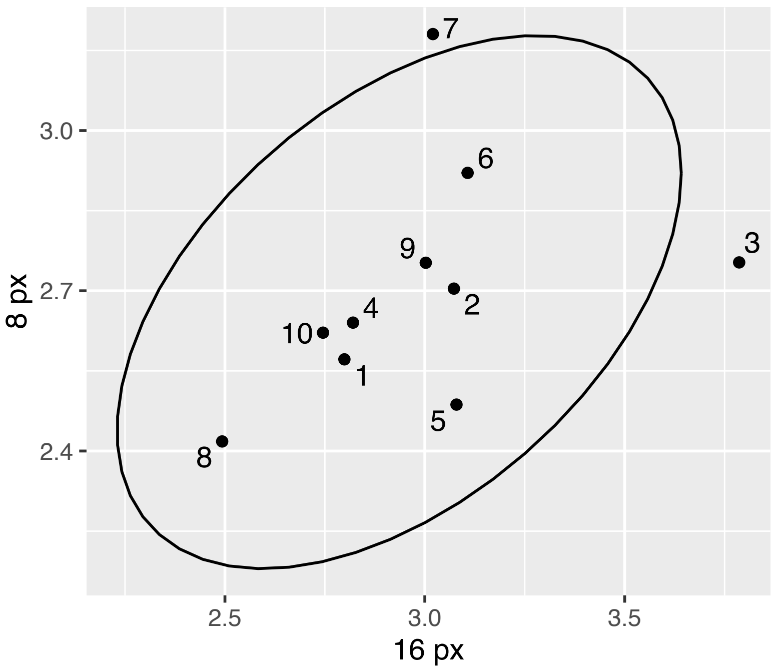

T1 firstorder mean

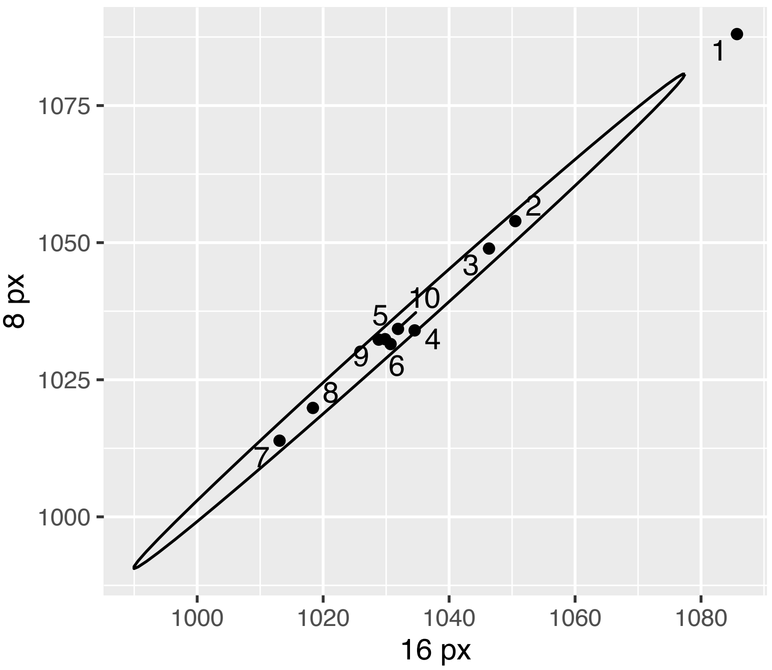

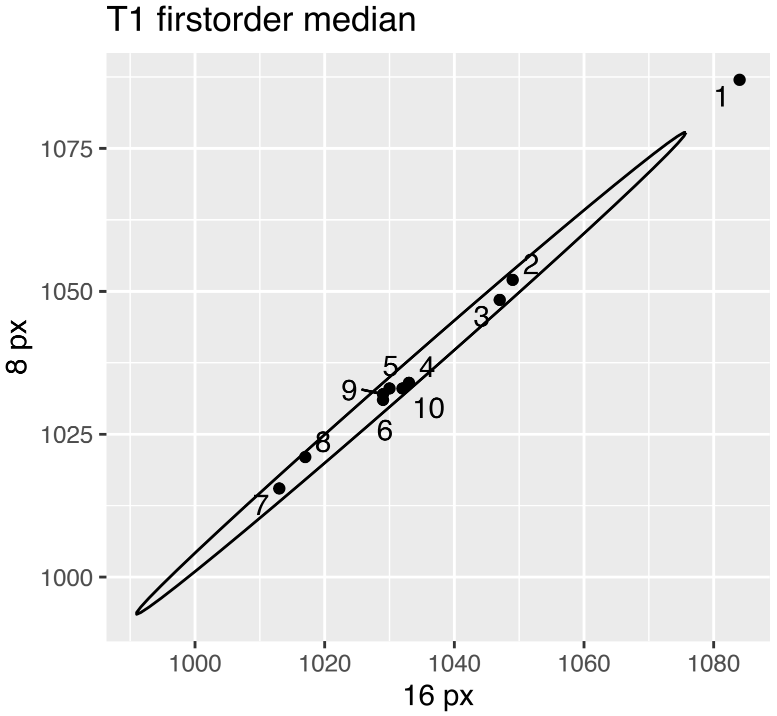
T1 firstorder median
8 px
16 px
1000
1020
1040
1060
1080
1075
1050
1025
1000
1
2
3
4
5
6
7
8
9
10

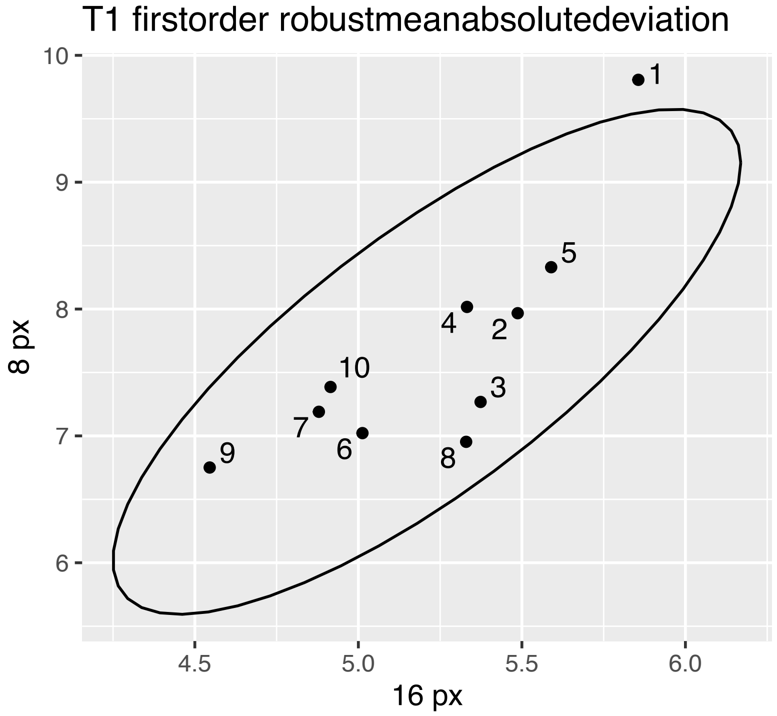
T1 firstorder robustmeanabsolutedeviation
8 px
16 px
4.5
5.0
5.5
6.0
10
9
8
7
6
1
2
3
4
5
6
7
8
9
10

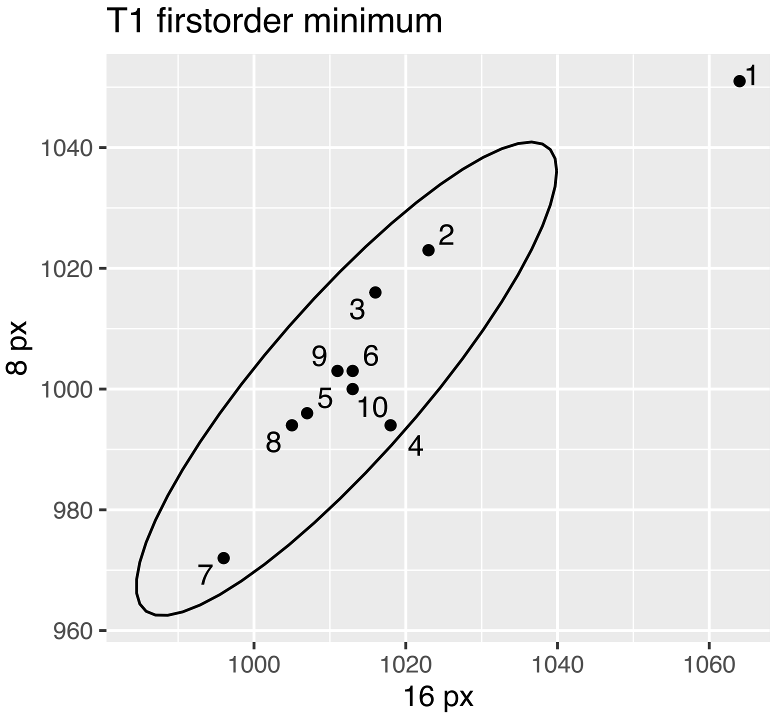
T1 firstorder minimum
8 px
16 px
1000
1020
1040
1060
1040
1020
1000
980
960
1
2
3
4
5
6
7
8
9
10

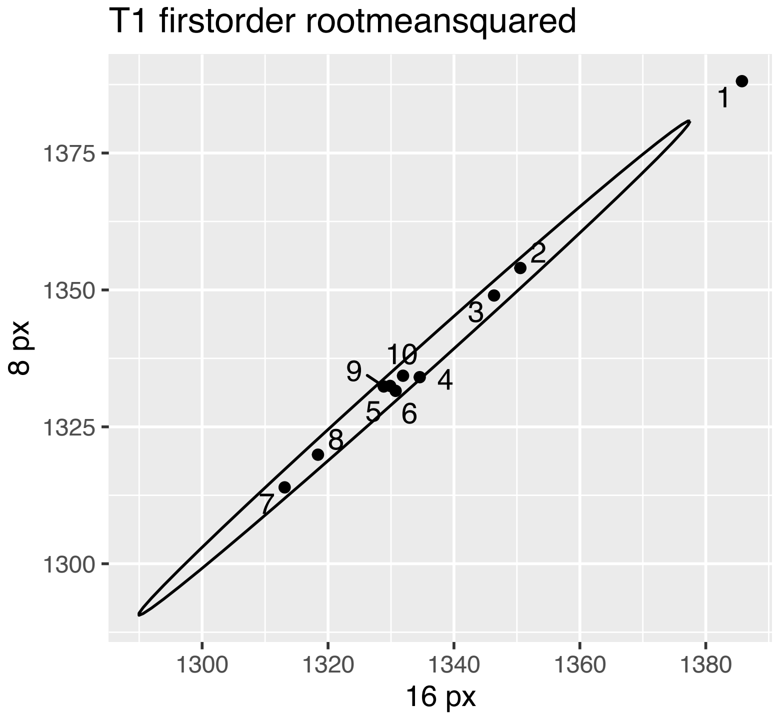
T1 firstorder rootmeansquared
8 px
16 px
1300
1320
1340
1360
1380
1375
1350
1325
1300
1
2
3
4
5
6
7
8
9
10

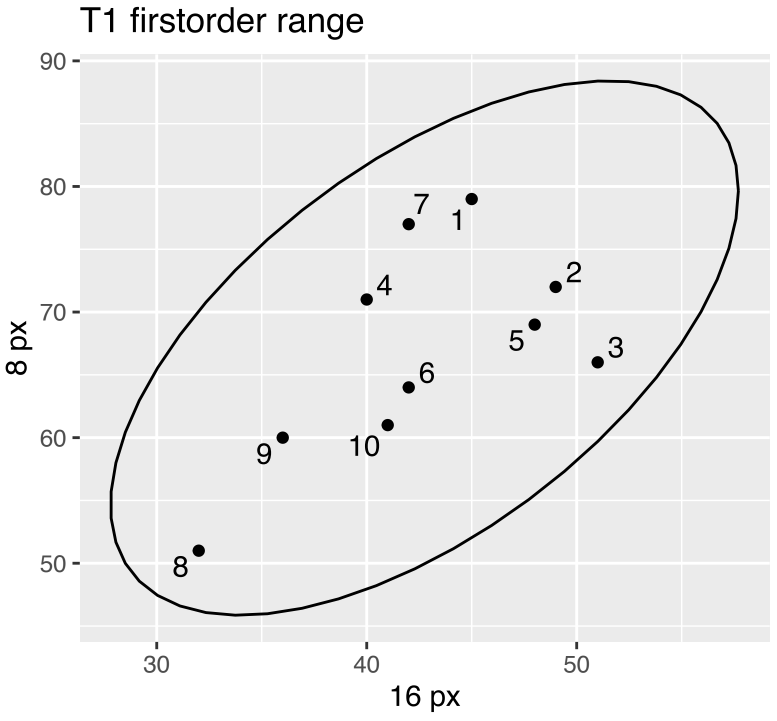
T1 firstorder range
8 px
16 px
30
40
50
90
80
70
60
50
1
2
3
4
5
6
7
8
9
10

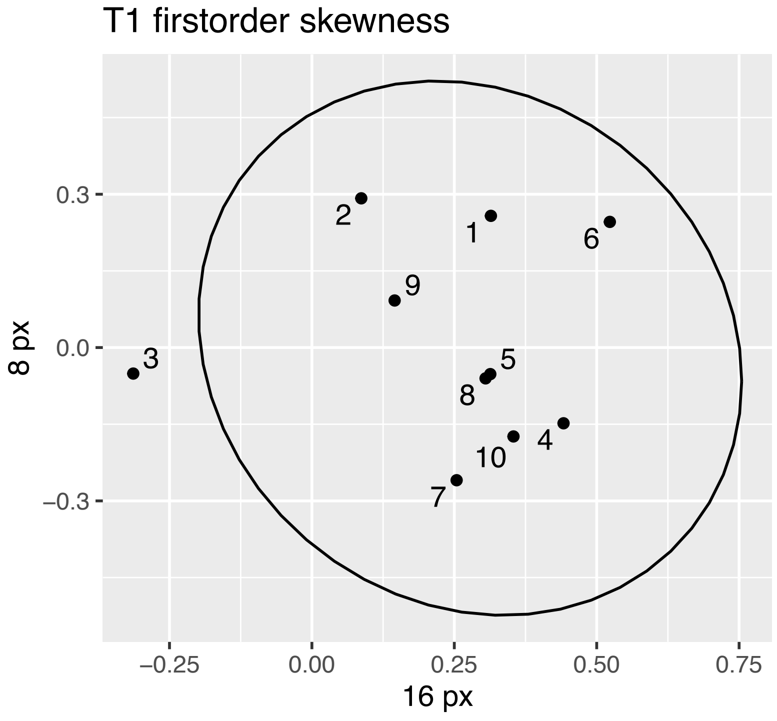
T1 firstorder skewness
8 px
16 px
−0.25
0.00
0.25
0.50
0.75
0.3
0.0
−0.3
1
2
3
4
5
6
7
8
9
10

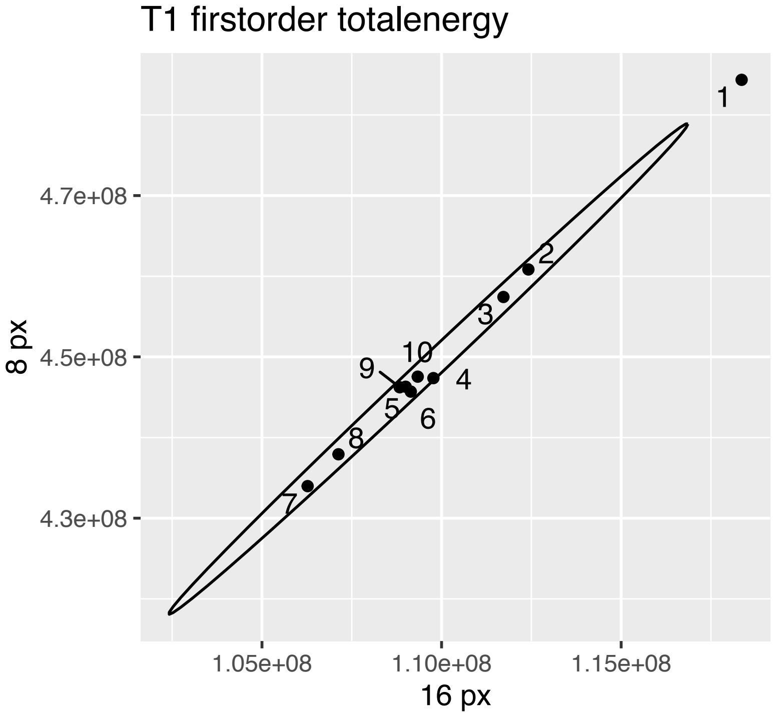
T1 firstorder totalenergy
4.7e+08
4.5e+08
4.3e+08
8 px
1.05e+08
1.10e+08
1.15e+08
16 px
1
2
3
4
5
6
7
8
9
10

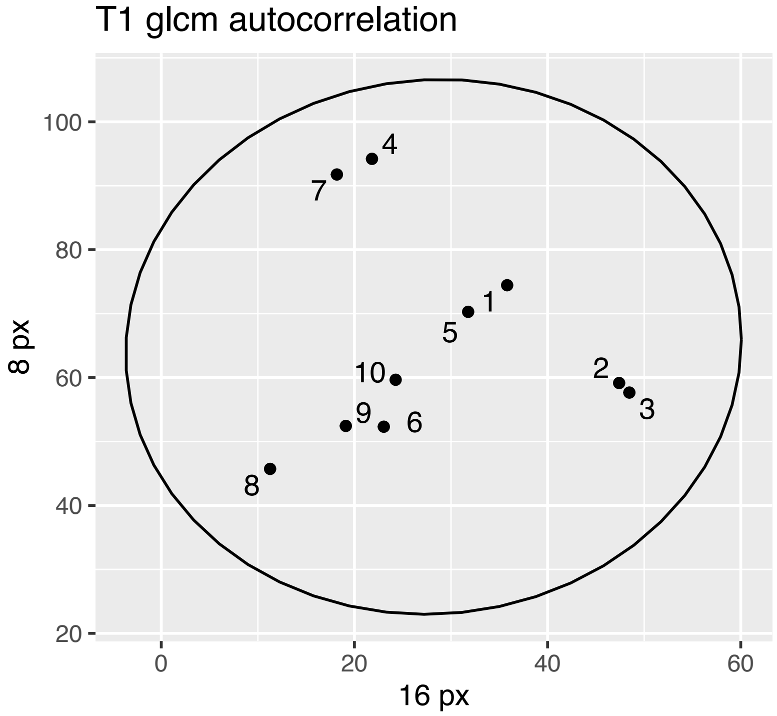
T1 glcm autocorrelation
100
80
60
40
20
8 px
0
20
40
60
16 px
1
2
3
4
5
6
7
8
9
10

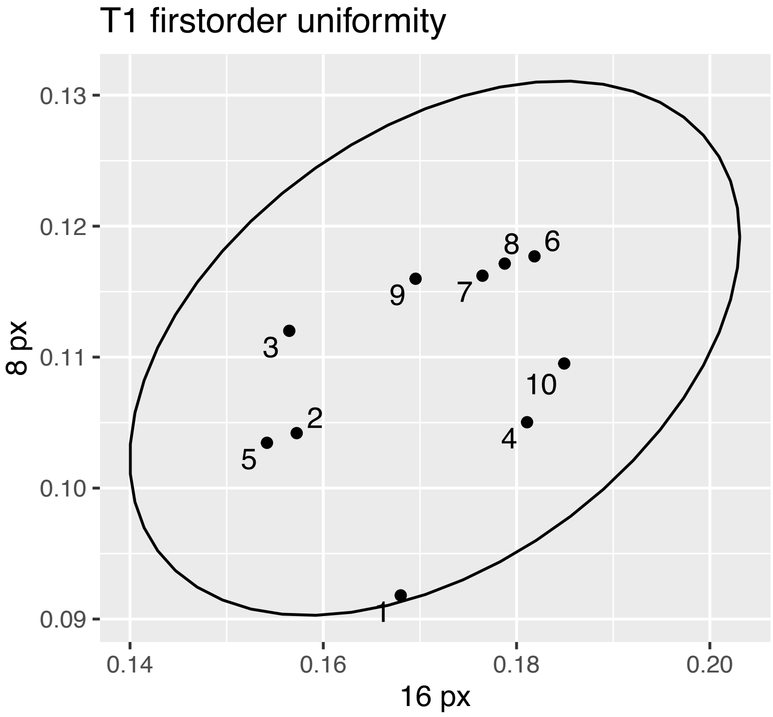
T1 firstorder uniformity
0.13
0.12
0.11
0.10
0.09
8 px
0.14
0.16
0.18
0.20
16 px
1
2
3
4
5
6
7
8
9
10

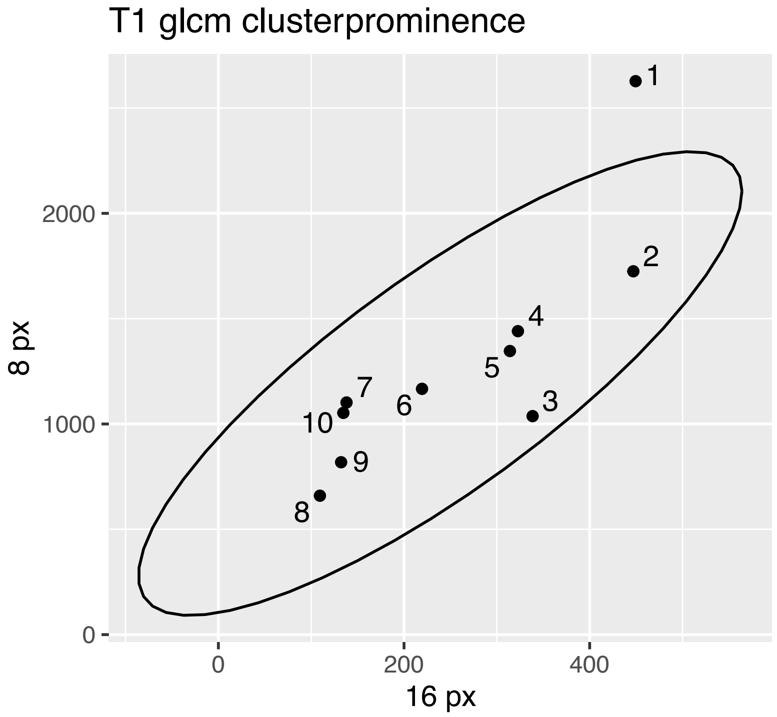
T1 glcm clusterprominence
2000
1000
0
8 px
0
200
400
16 px
1
2
3
4
5
6
7
8
9
10

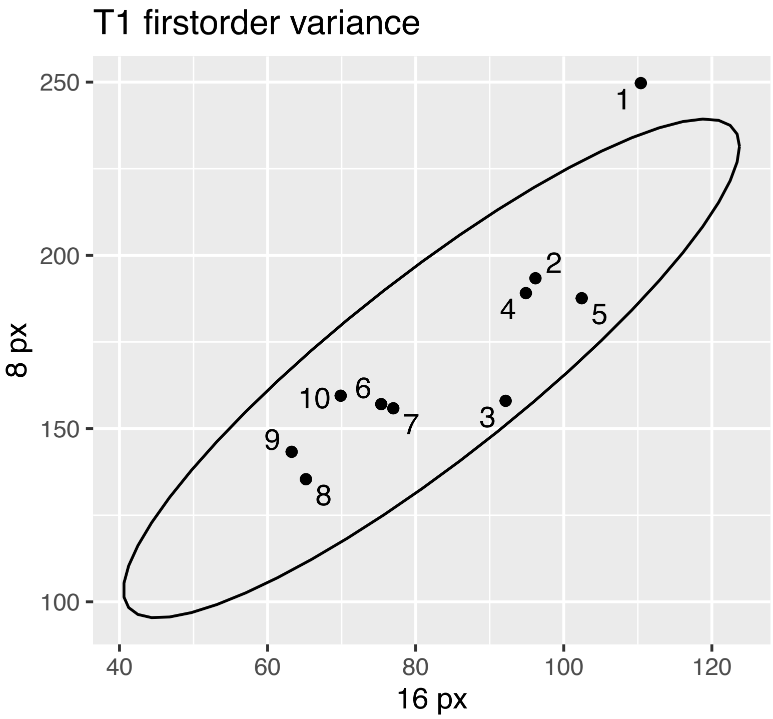
T1 firstorder variance
250
200
150
100
8 px
40
60
80
100
120
16 px
1
2
3
4
5
6
7
8
9
10

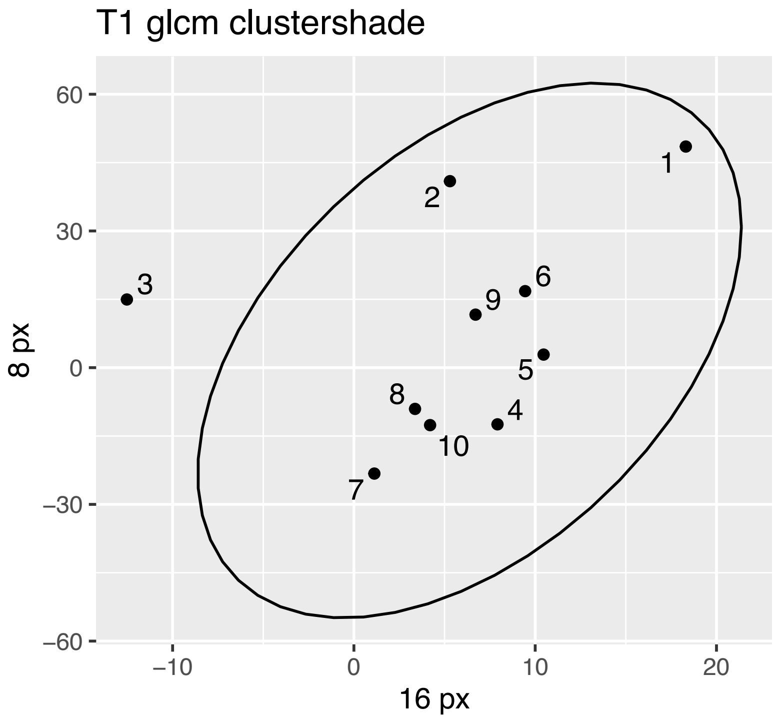
T1 glcm clustershade
60
30
0
−30
−60
8 px
−10
0
10
20
16 px
1
2
3
4
5
6
7
8
9
10

T1 glcm clustertendency

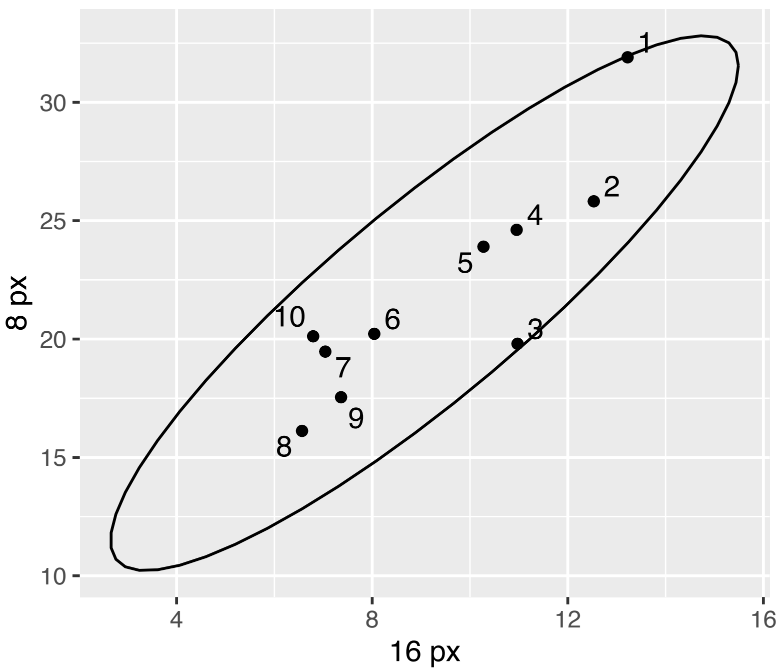

T1 glcm differenceaverage

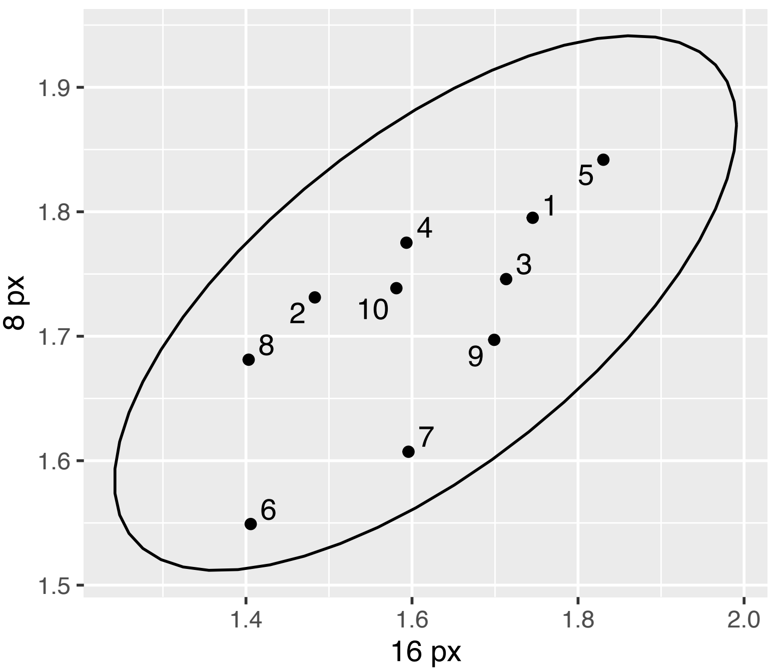

T1 glcm contrast

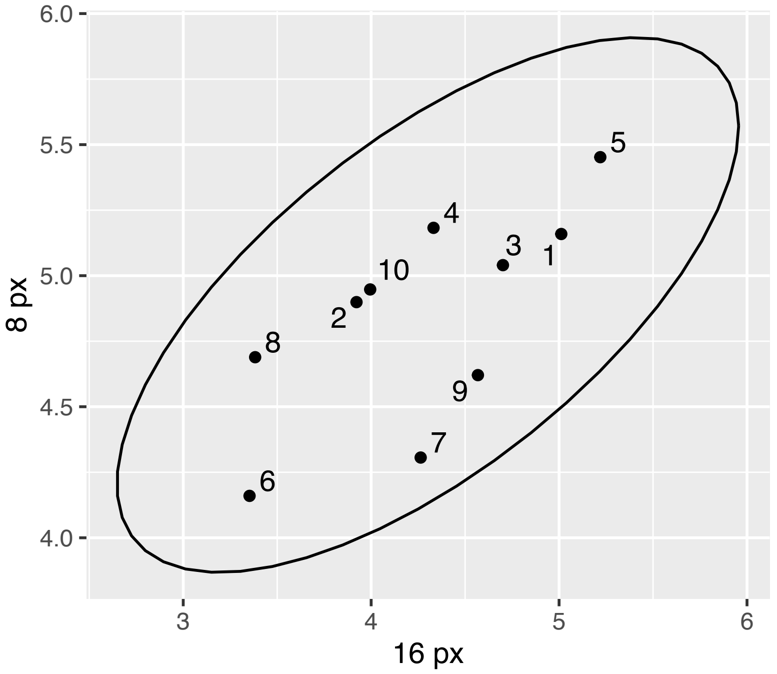

T1 glcm differenceentropy

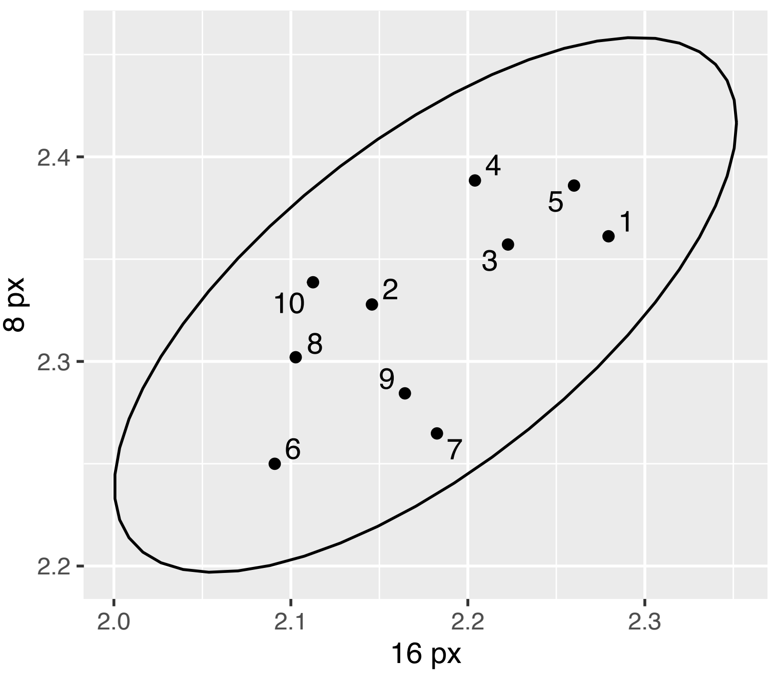

T1 glcm correlation

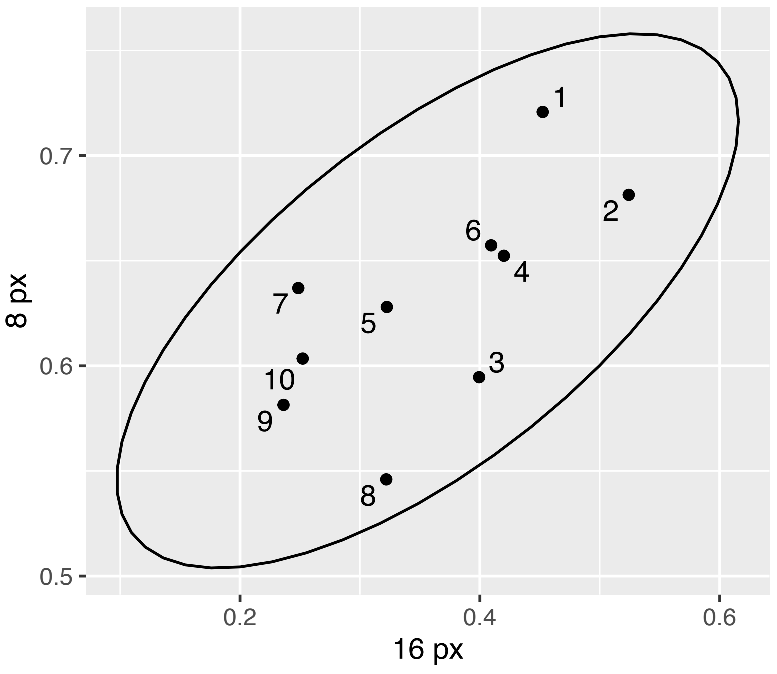

T1 glcm differencevariance

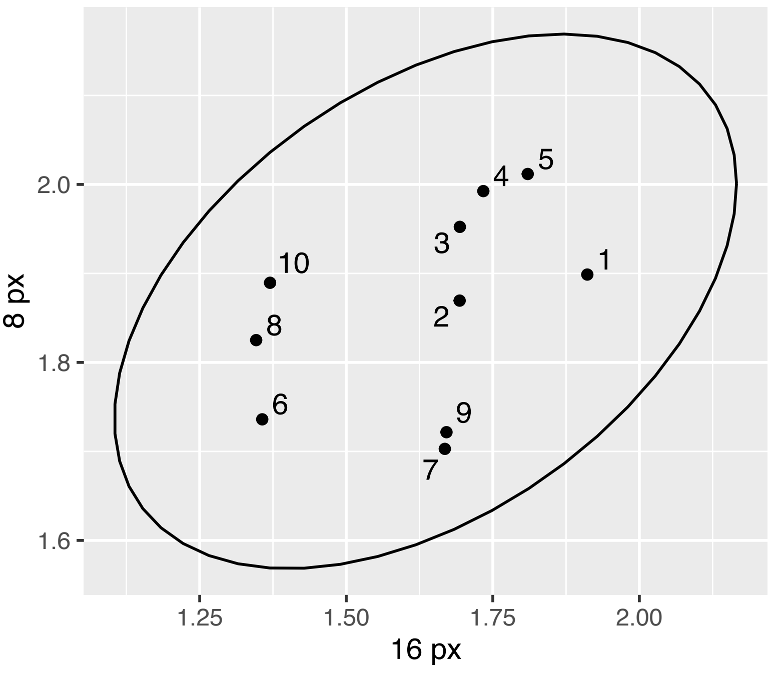

T1 glcm id

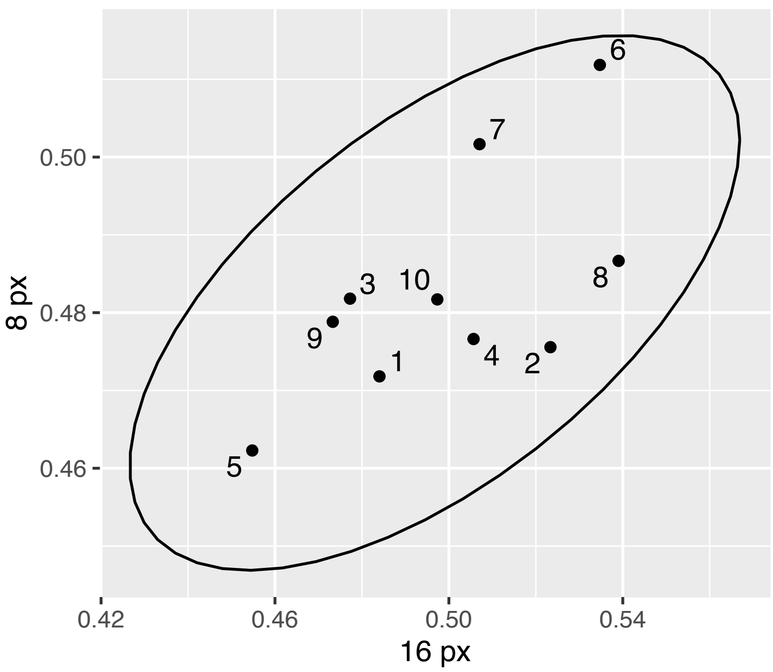

T1 glcm idn

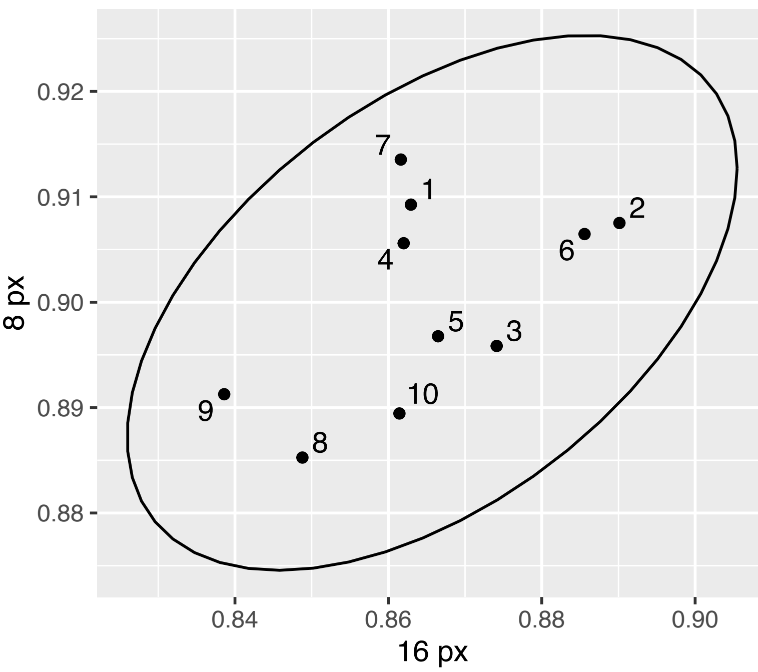

T1 glcm idm

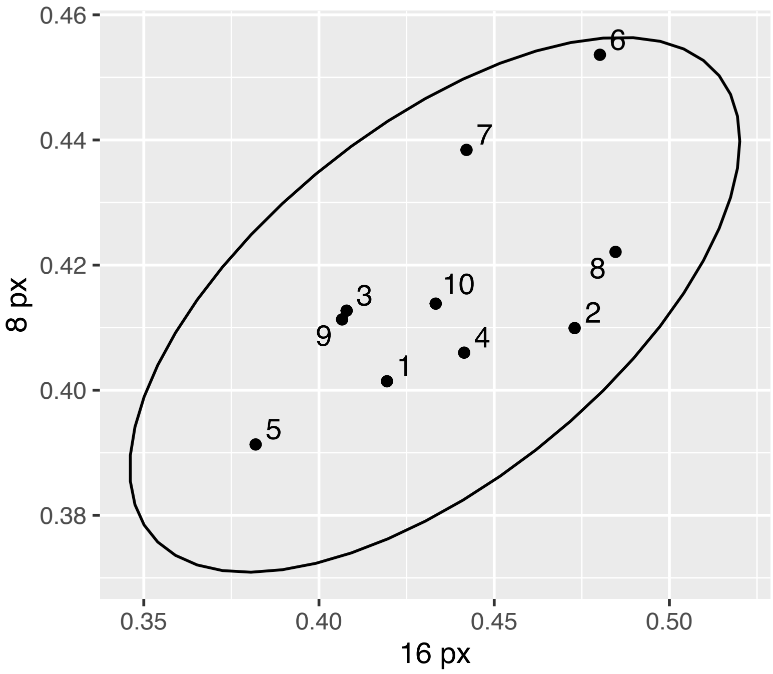

T1 glcm imc1

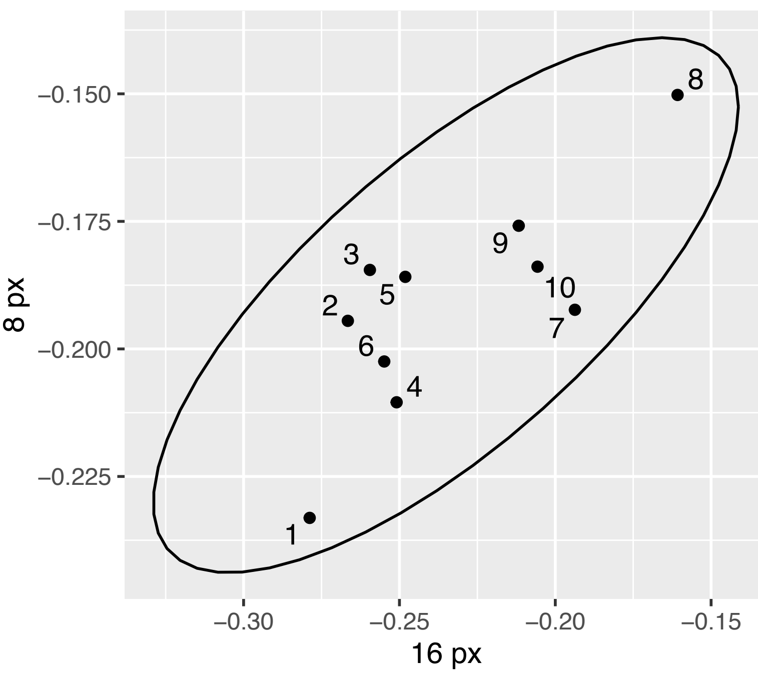

T1 glcm idmn

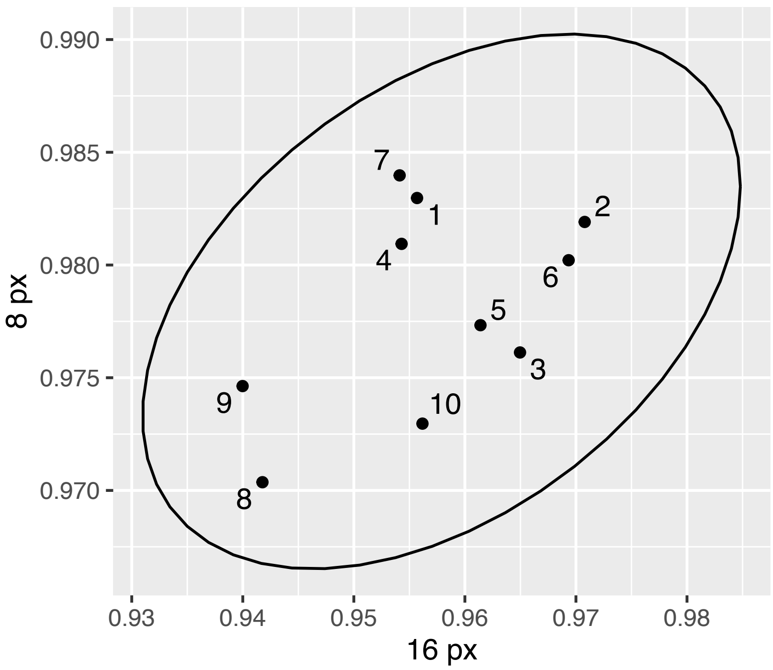

T1 glcm imc2

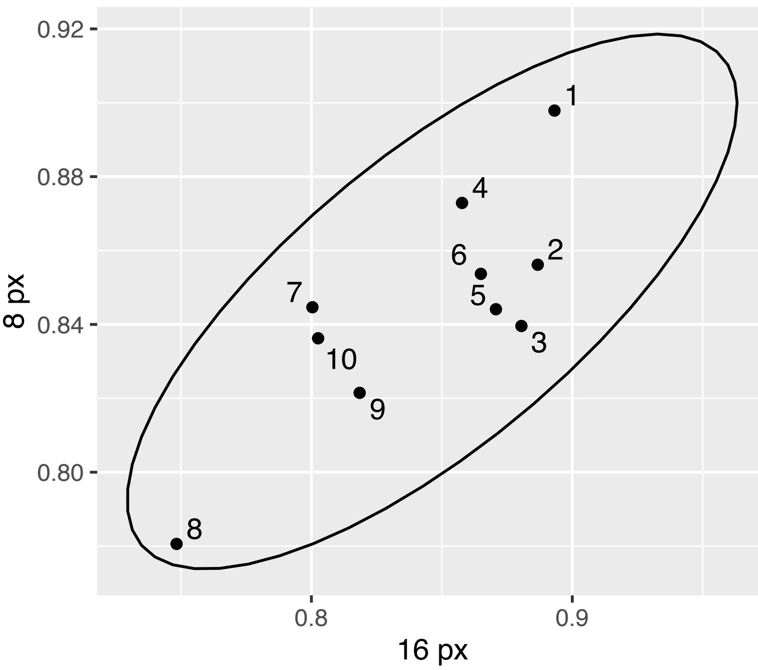

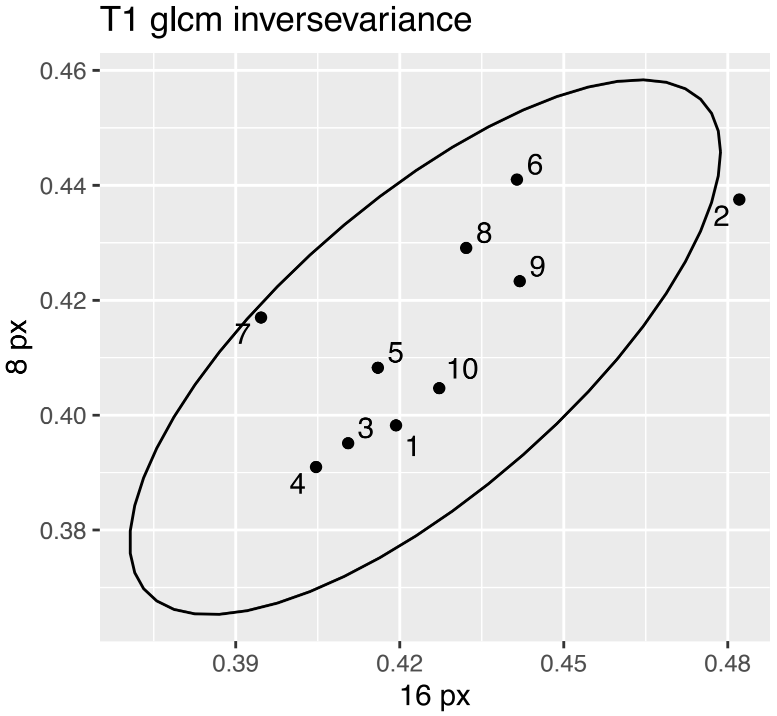
T1 glcm inversevariance
0.46
0.44
0.42
0.40
0.38
8 px
0.39
0.42
0.45
0.48
16 px

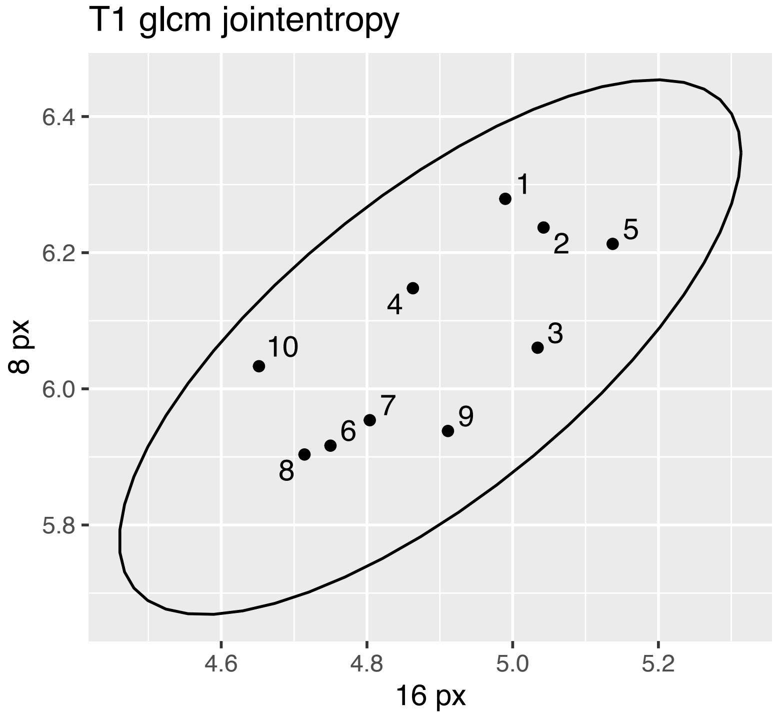
T1 glcm jointentropy
6.4
6.2
6.0
5.8
8 px
4.6
4.8
5.0
5.2
16 px

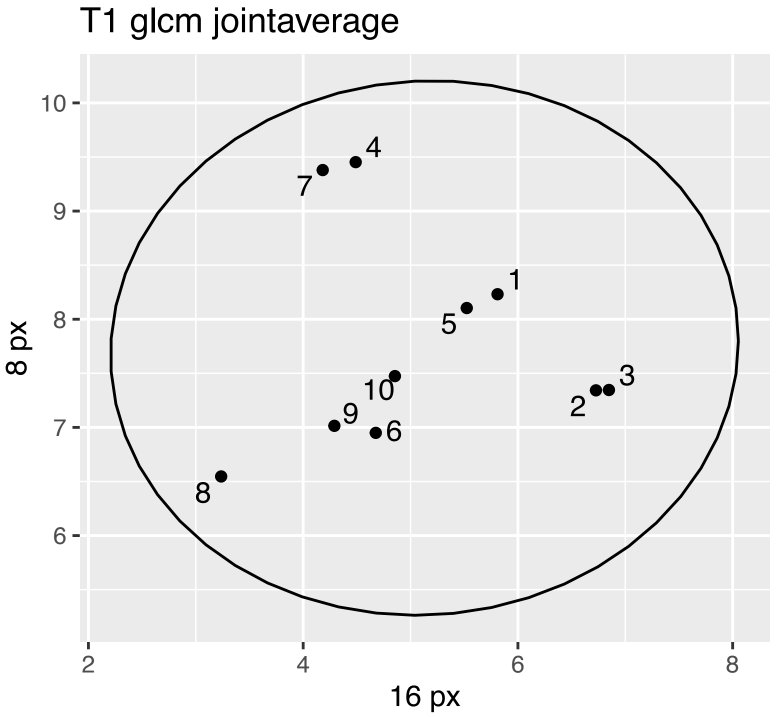
T1 glcm jointaverage
10
9
8
7
6
8 px
2
4
6
8
16 px

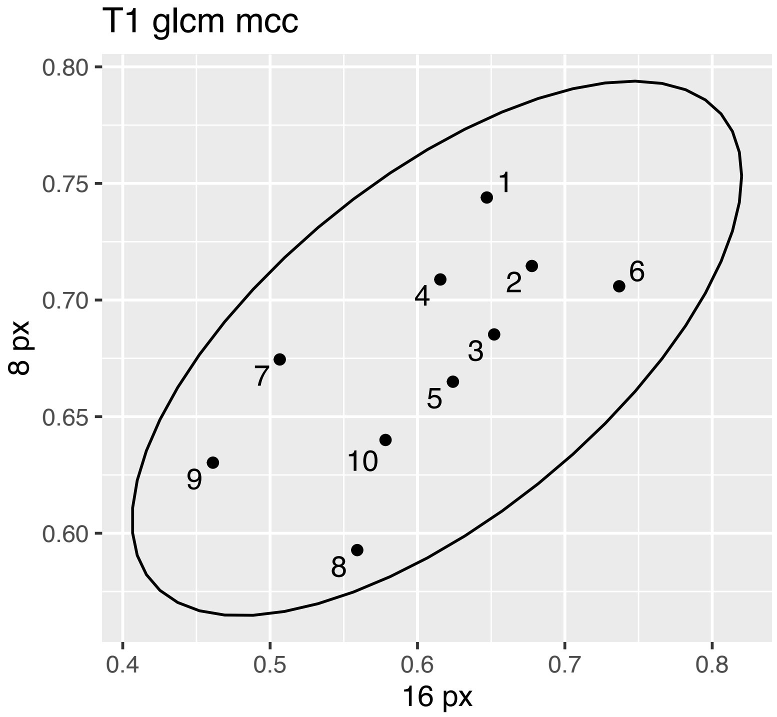
T1 glcm mcc
0.80
0.75
0.70
0.65
0.60
8 px
0.4
0.5
0.6
0.7
0.8
16 px

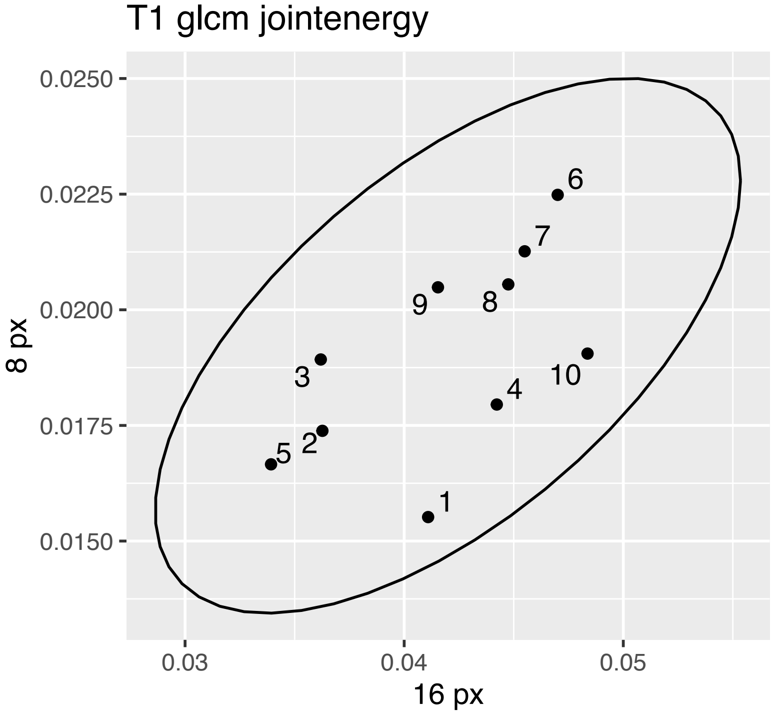
T1 glcm jointenergy
0.0250
0.0225
0.0200
0.0175
0.0150
8 px
0.03
0.04
0.05
16 px

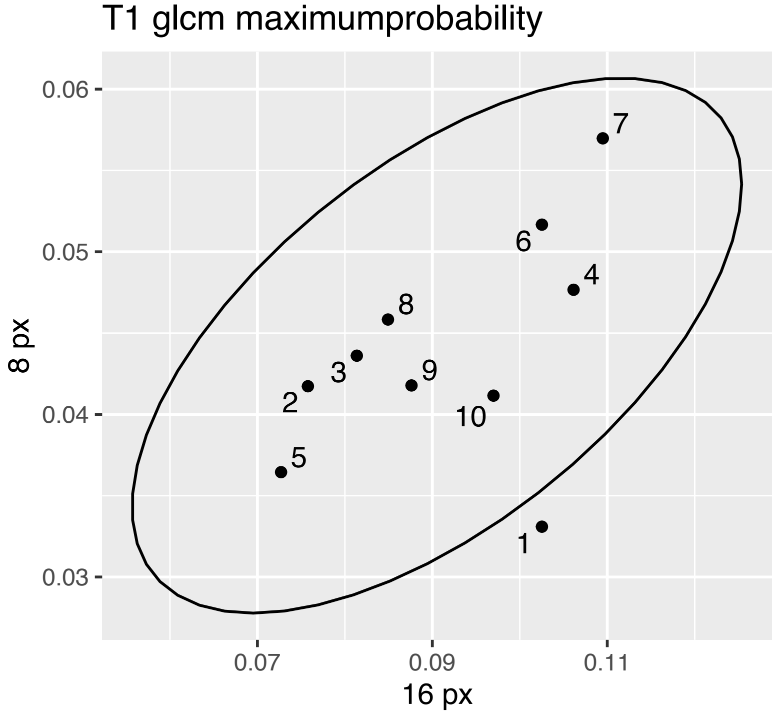
T1 glcm maximumprobability
0.06
0.05
0.04
0.03
8 px
0.07
0.09
0.11
16 px

T1 glcm sumaverage

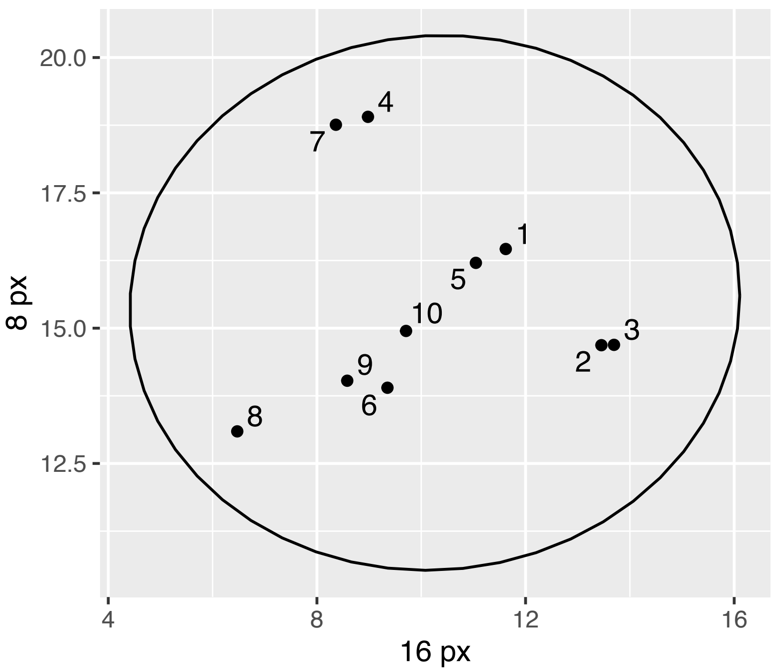

T1 glrlm graylevelnonuniformity

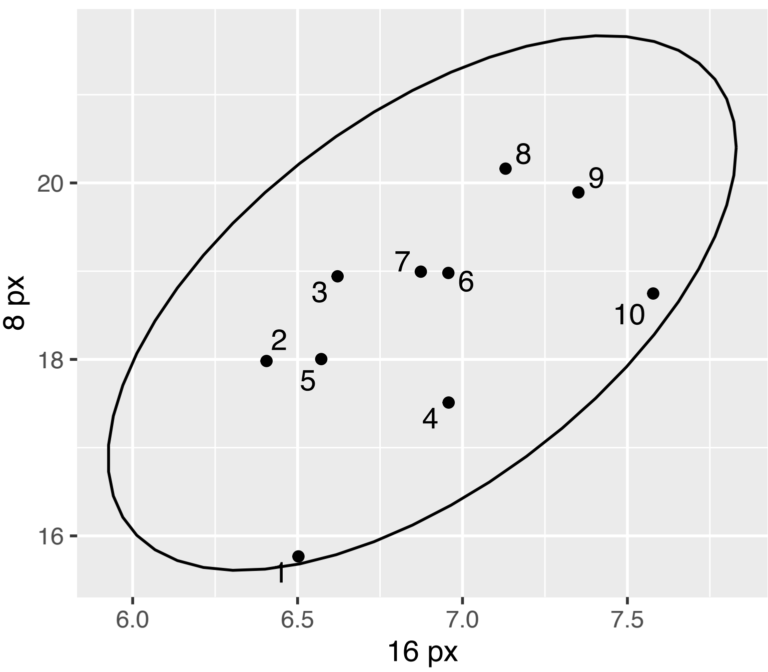

T1 glcm sumentropy

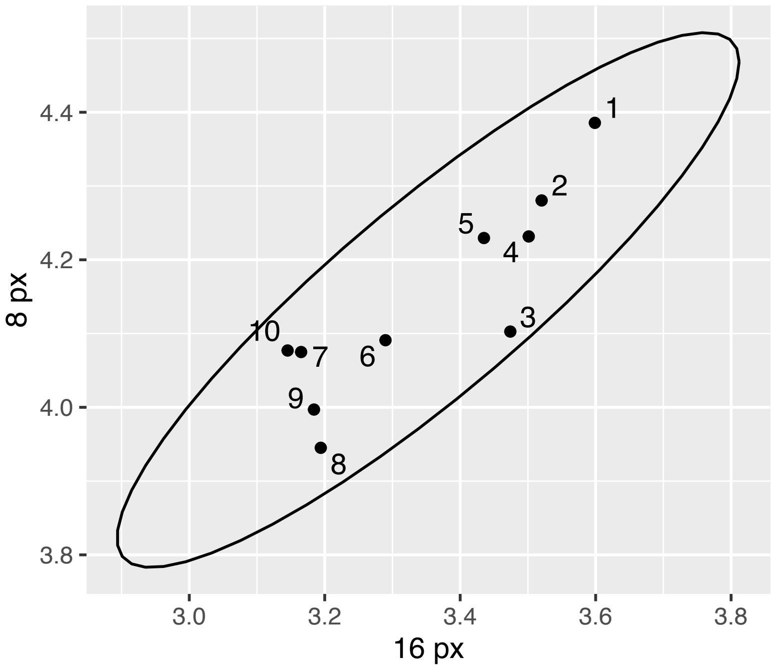

T1 glrlm graylevelnonuniformitynormalized

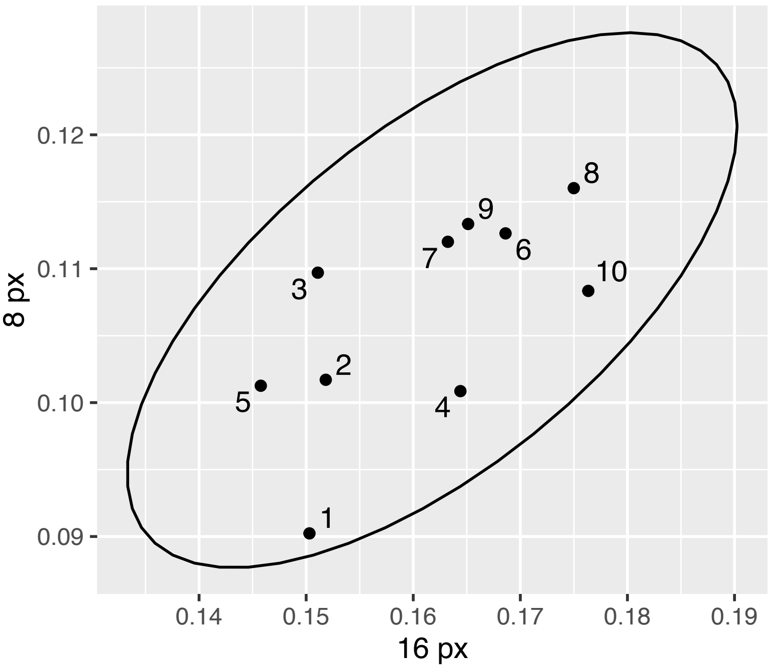

T1 glcm sumsquares

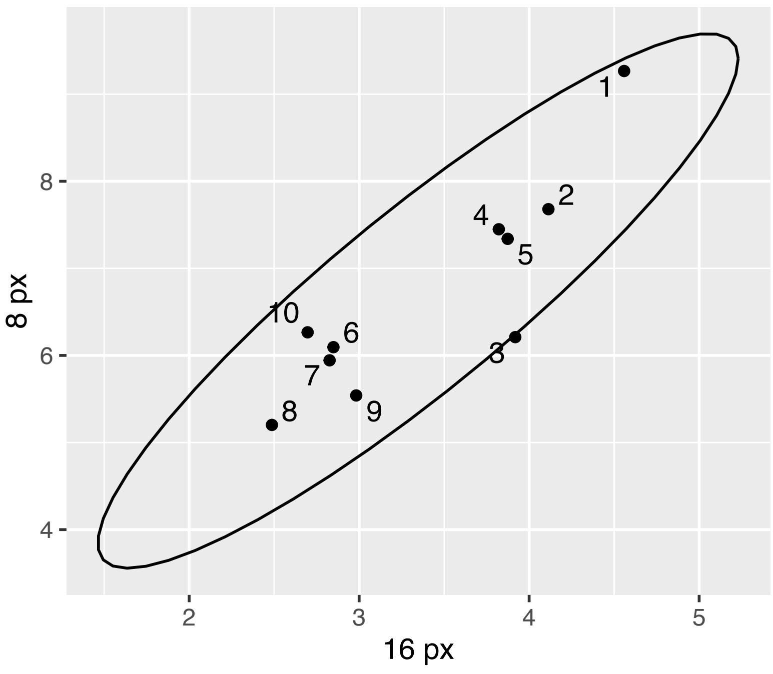

T1 glrlm graylevelvariance

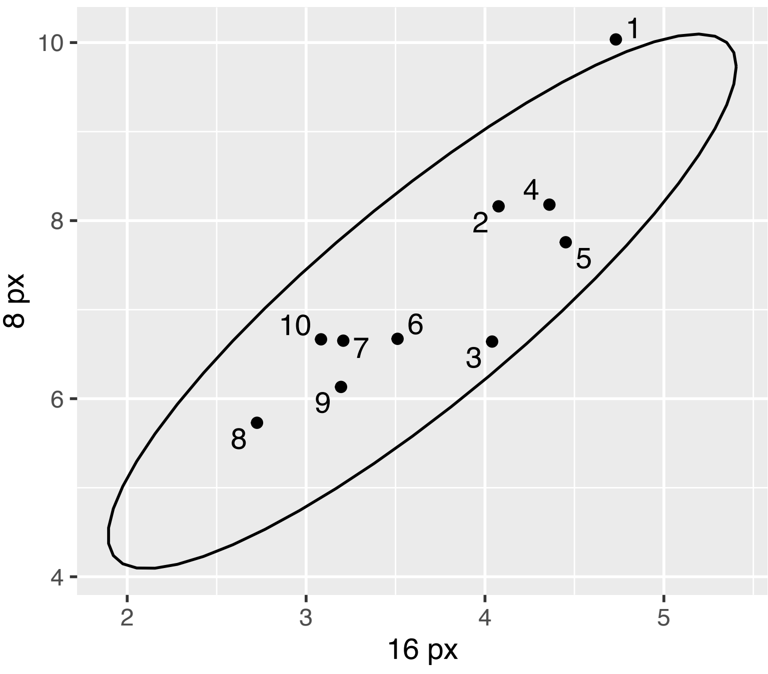

T1 glrlm highgraylevelrunemphasis

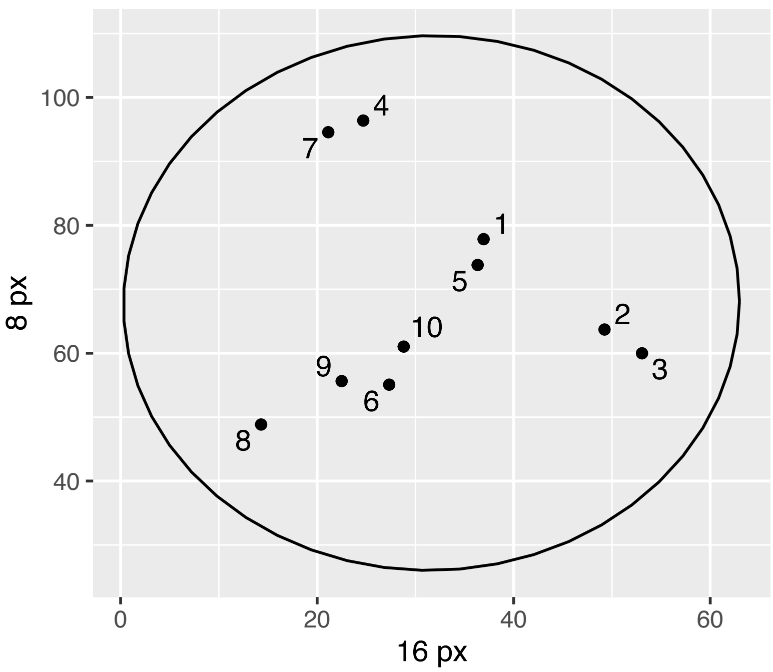

T1 glrlm longrunlowgraylevelemphasis

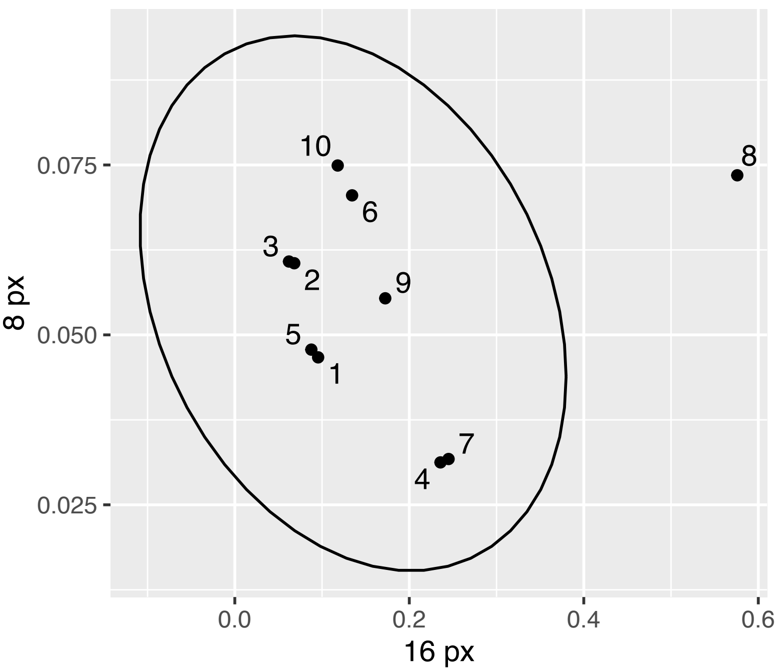

T1 glrlm longrunemphasis

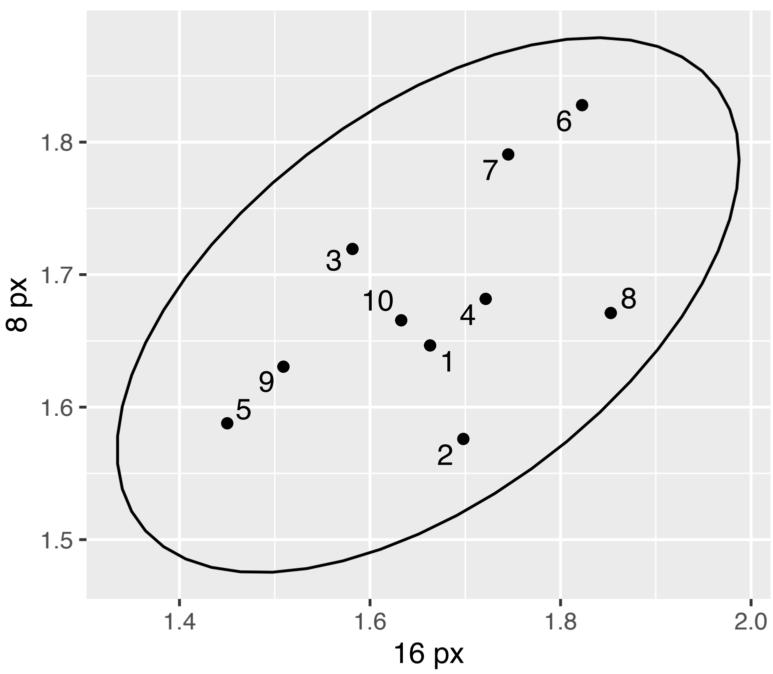

T1 glrlm lowgraylevelrunemphasis

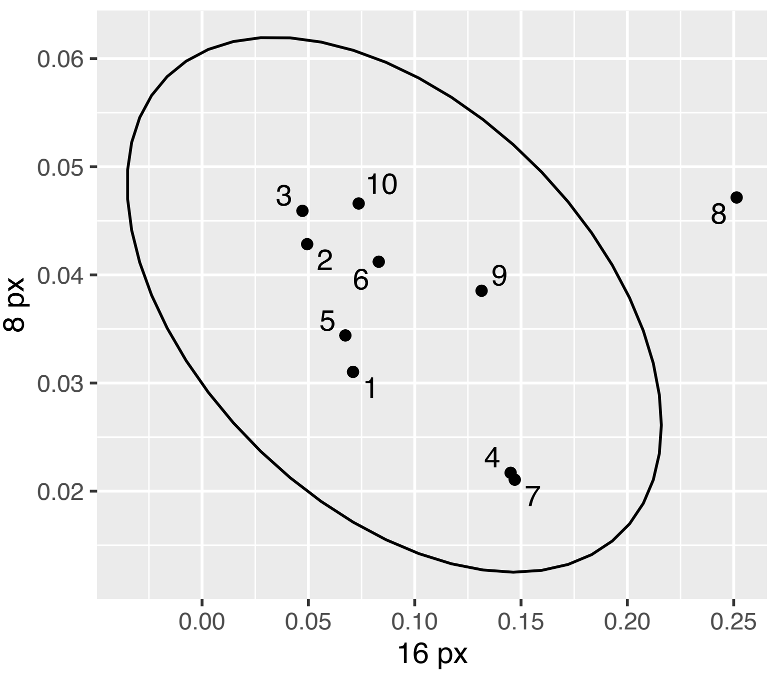

T1 glrlm longrunhighgraylevelemphasis

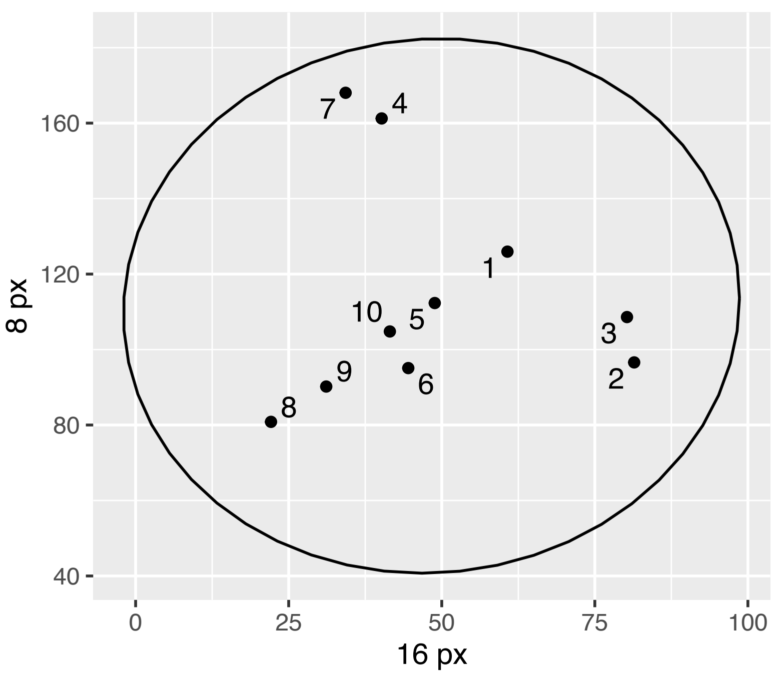

T1 glrlm runentropy

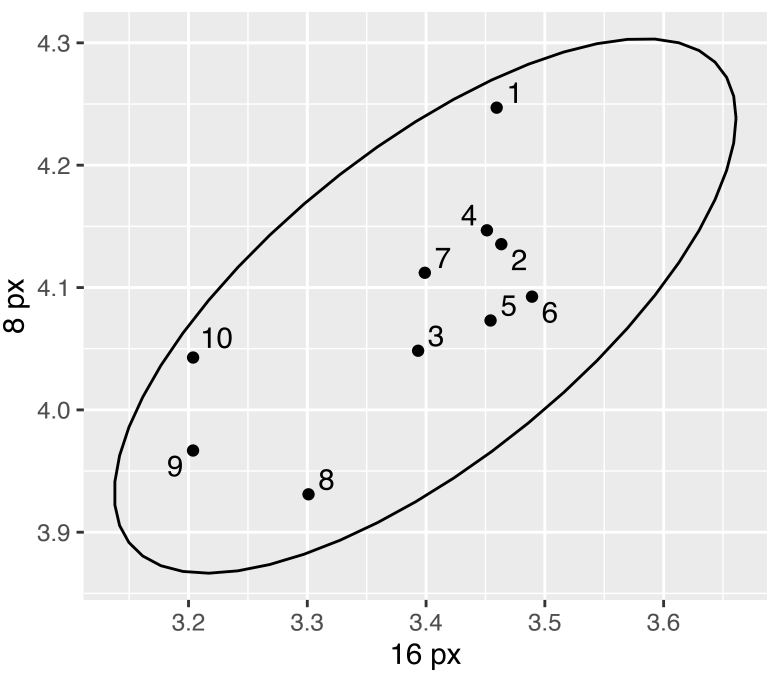

T1 glrlm runlengthnonuniformity

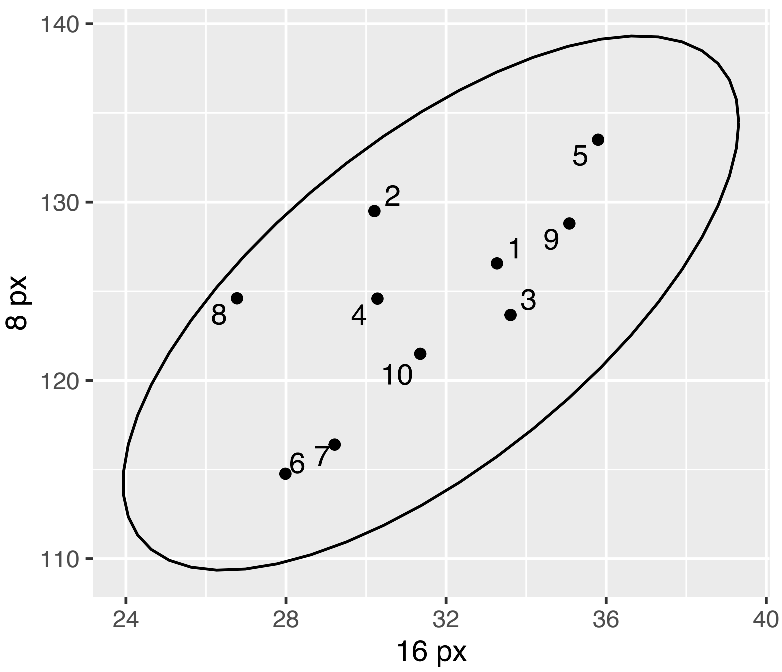

T1 glrlm runvariance

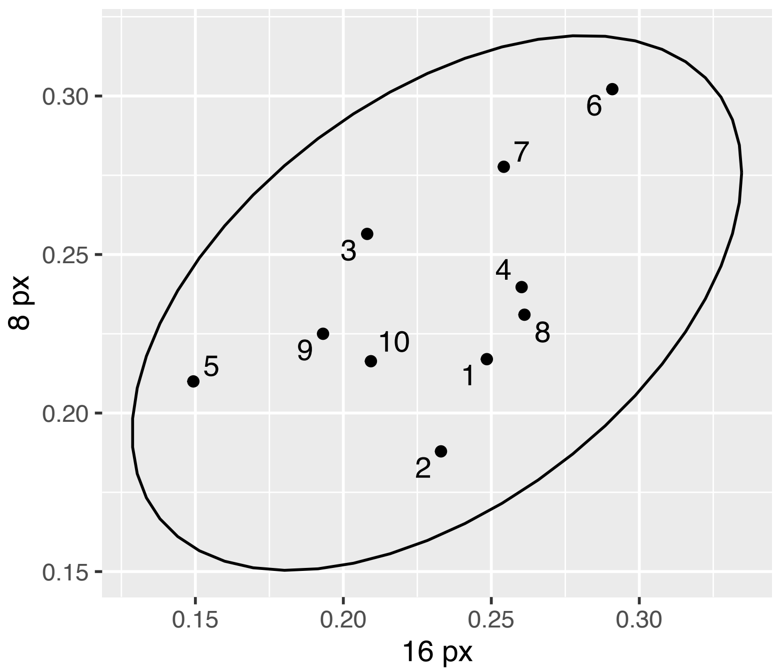

T1 glrlm runlengthnonuniformitynormalized

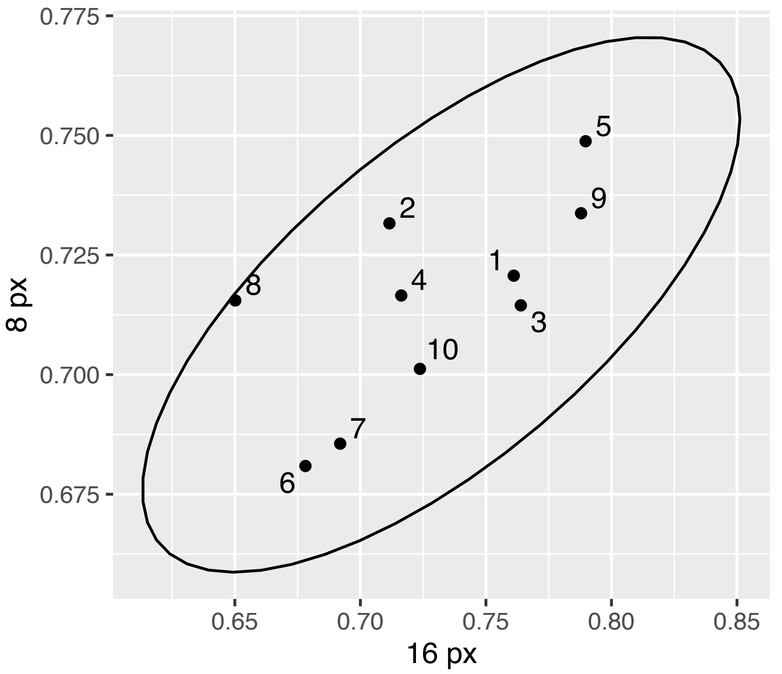

T1 glrlm shortrunemphasis

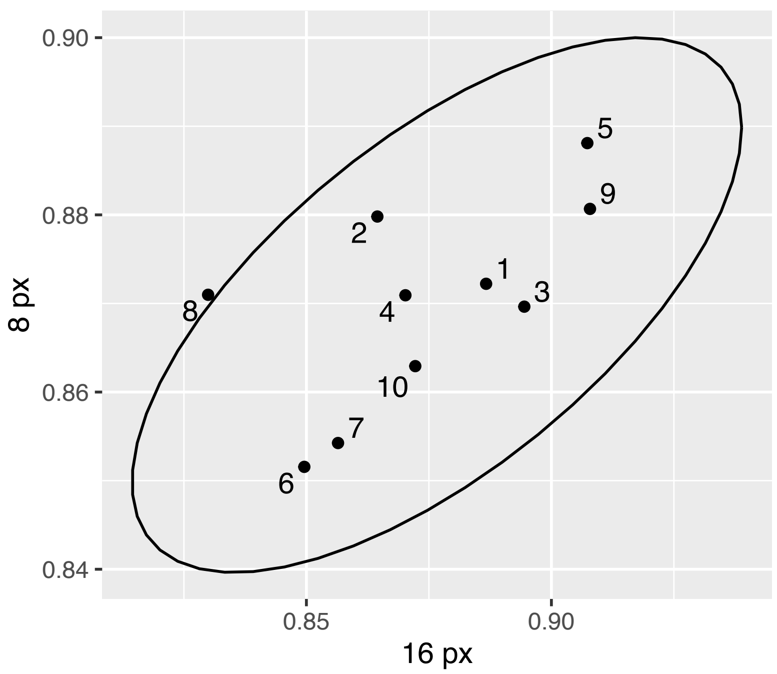

T1 glrlm runpercentage

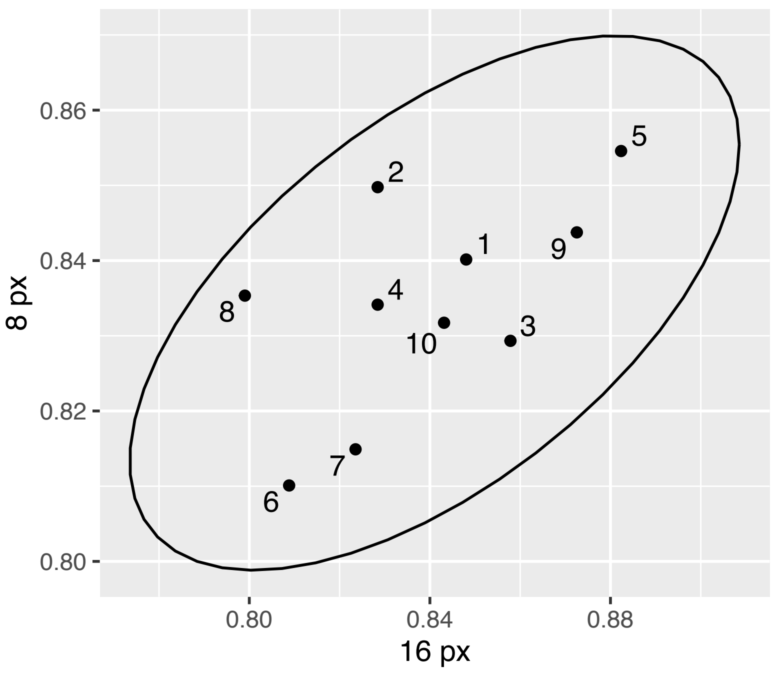

T1 glrlm shortrunhighgraylevelemphasis

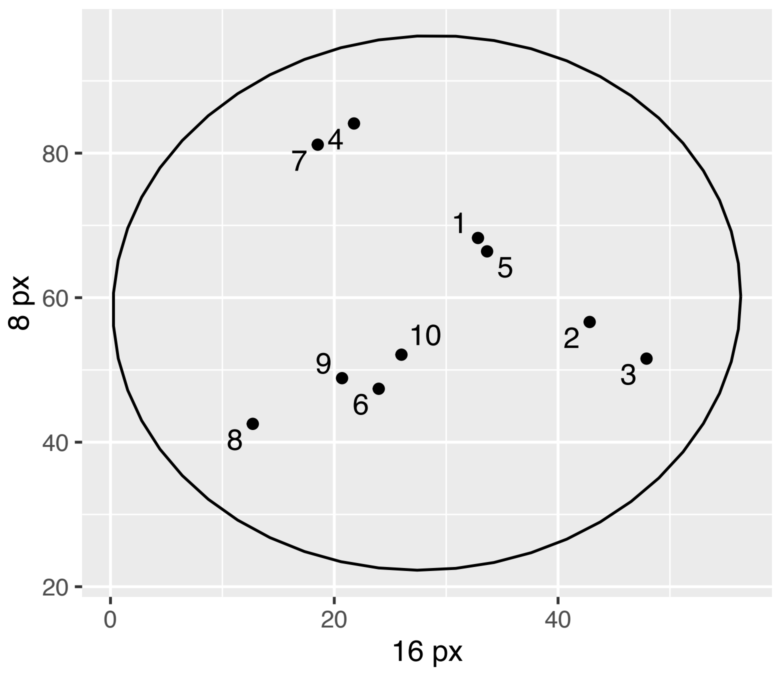

T1 glrlm shortrunlowgraylevelemphasis

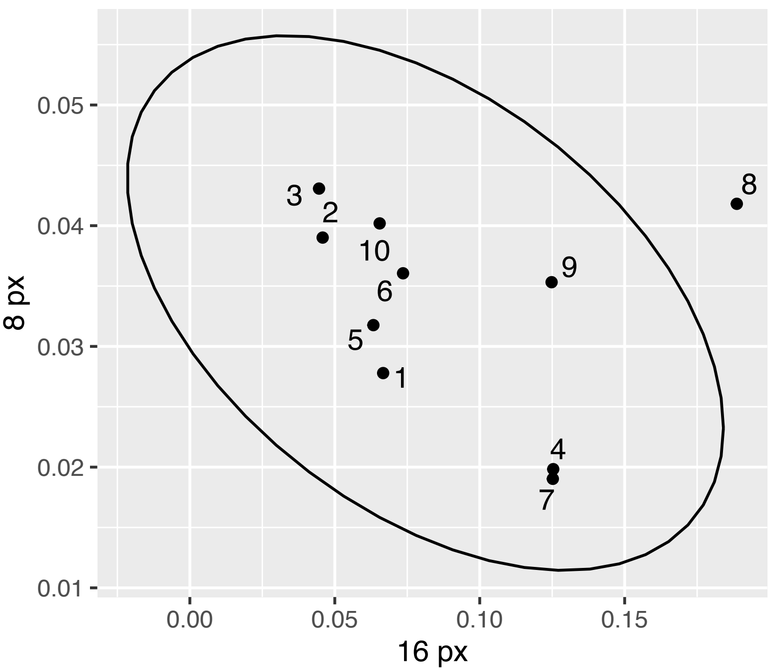

T1 glszm graylevelvariance

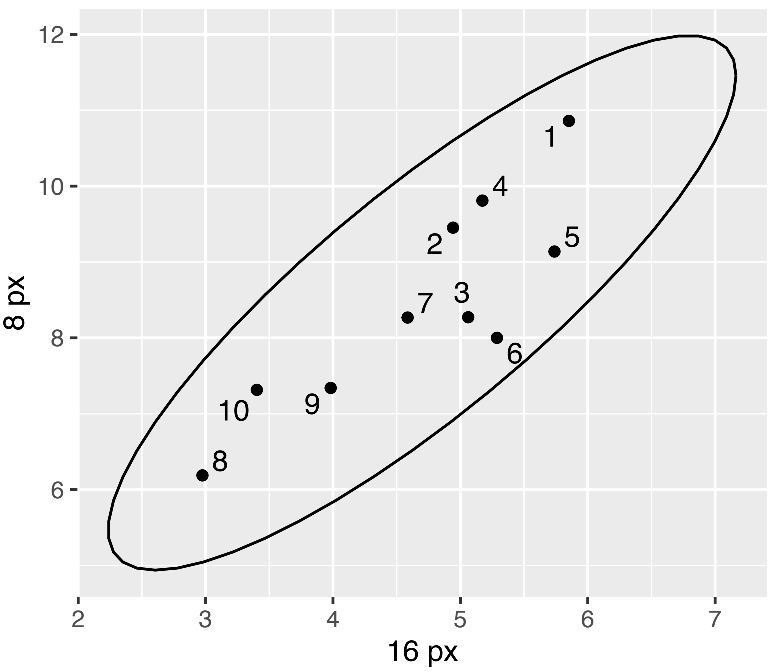

T1 glszm graylevelnonuniformity

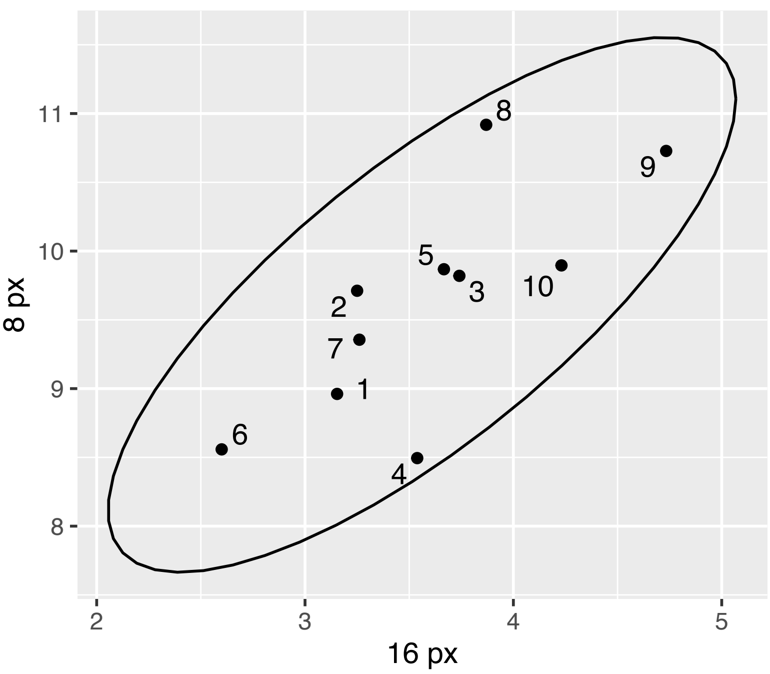

T1 glszm highgraylevelzoneemphasis

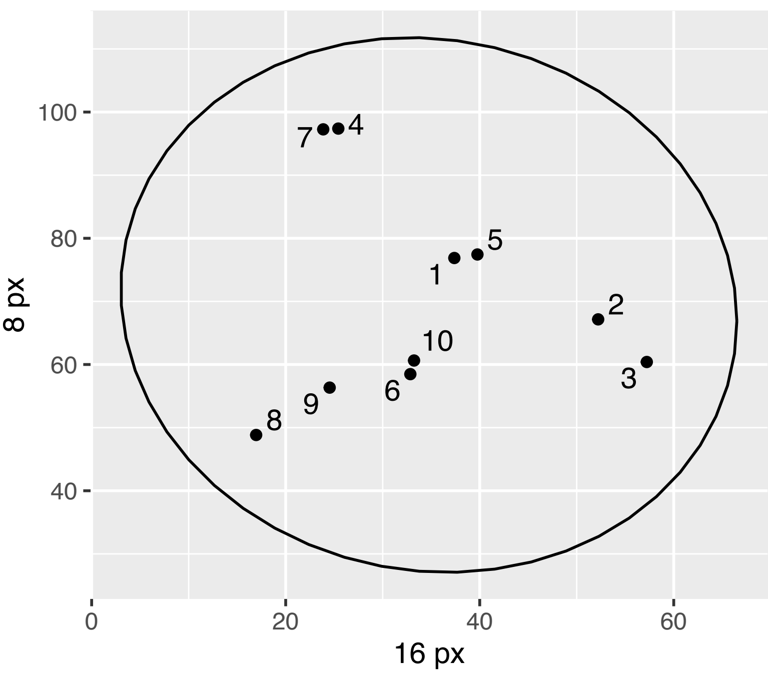

T1 glszm graylevelnonuniformitynormalized

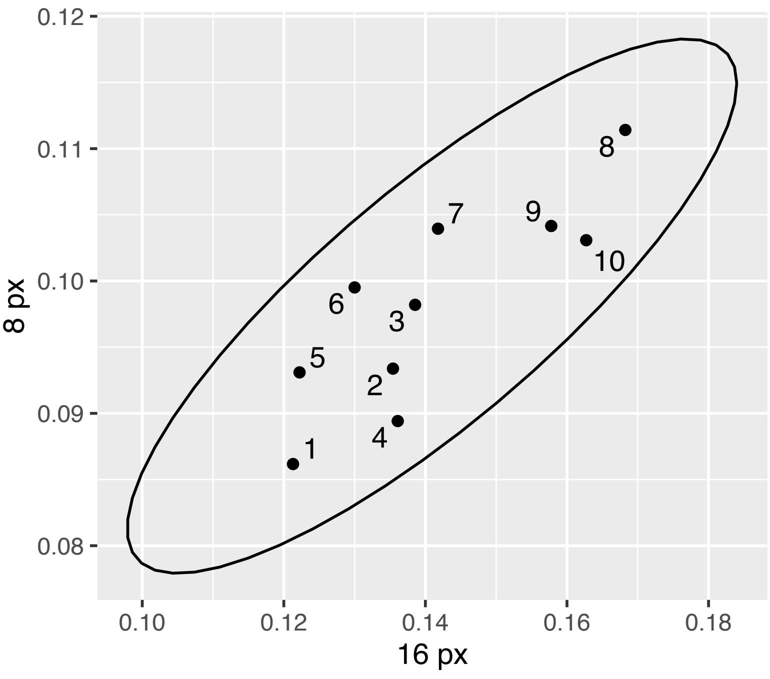

T1 glszm largeareaemphasis

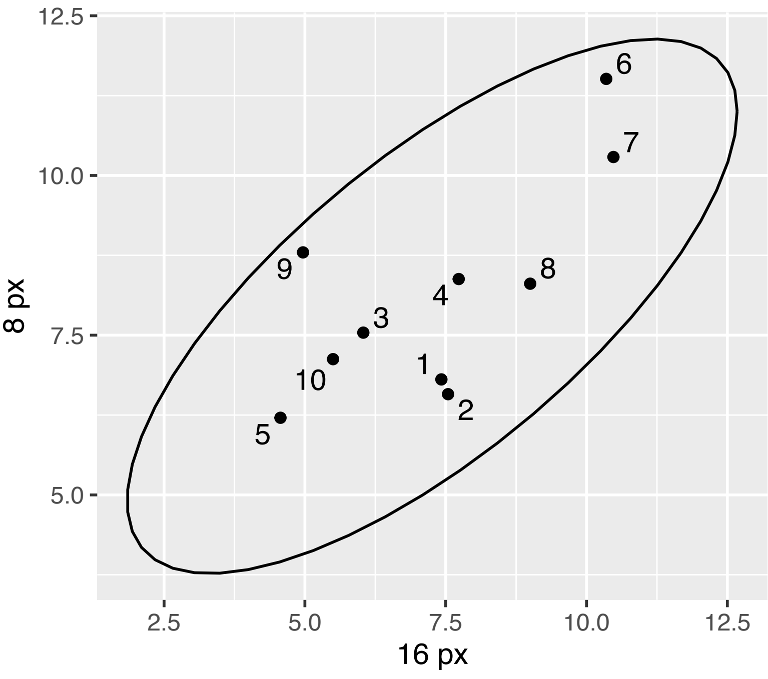

T1 glszm largeareahighgraylevelemphasis

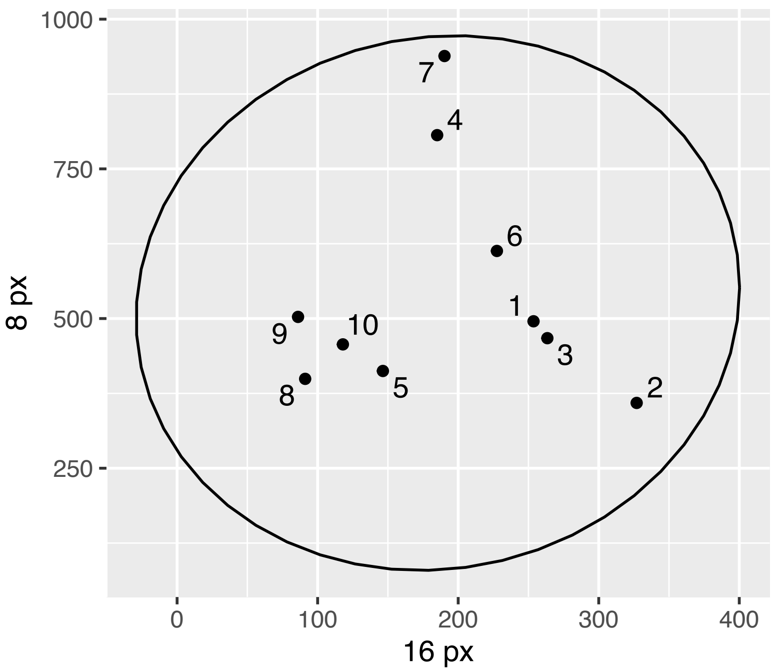

T1 glszm sizezonenonuniformity

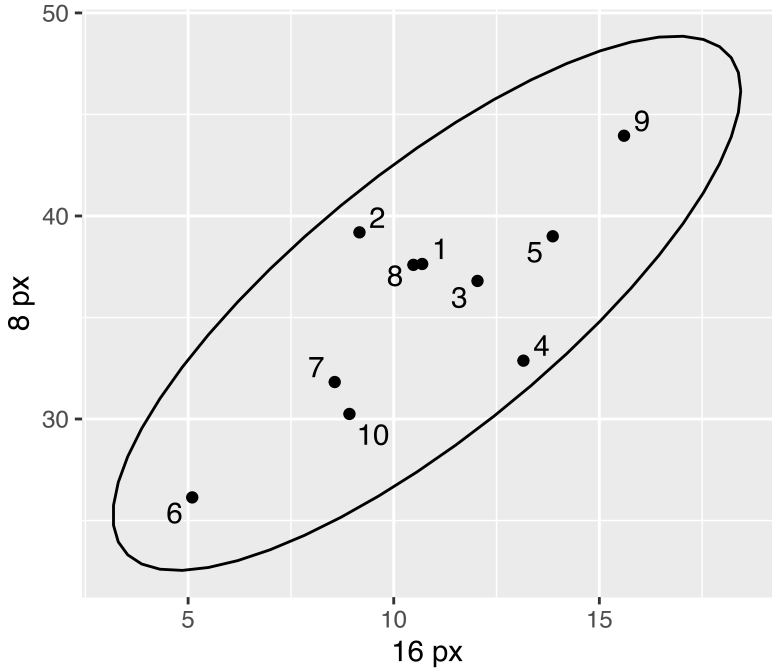

T1 glszm largearealowgraylevelemphasis

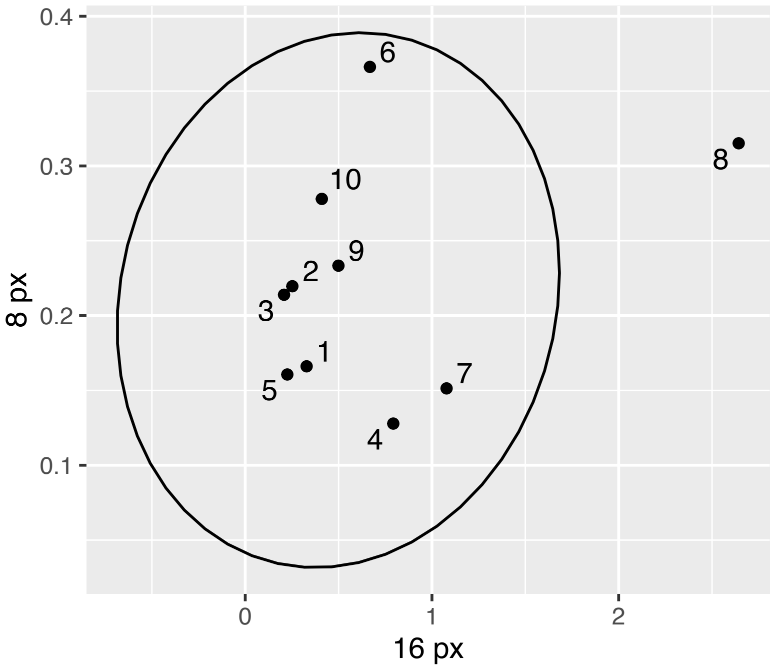

T1 glszm sizezonenonuniformitynormalized

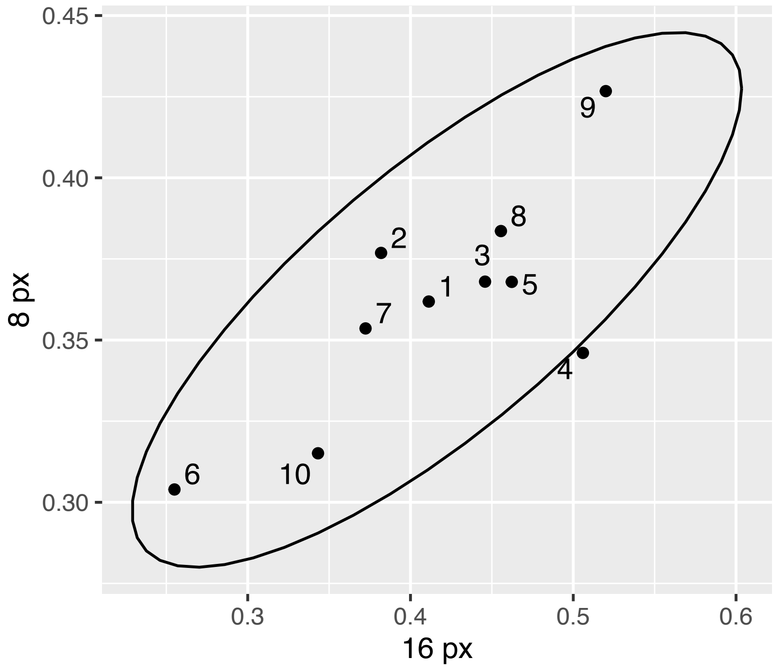

T1 glszm lowgraylevelzoneemphasis

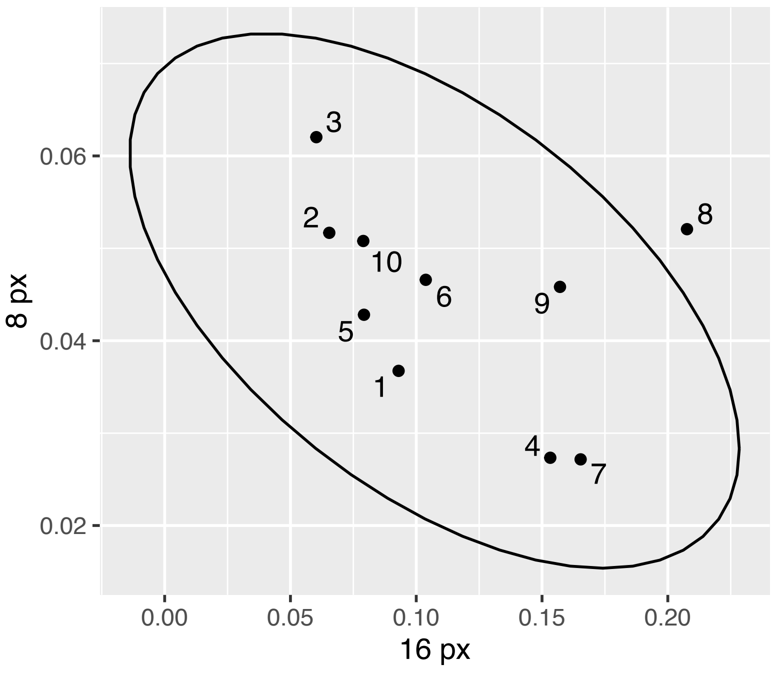

T1 glszm smallareaemphasis

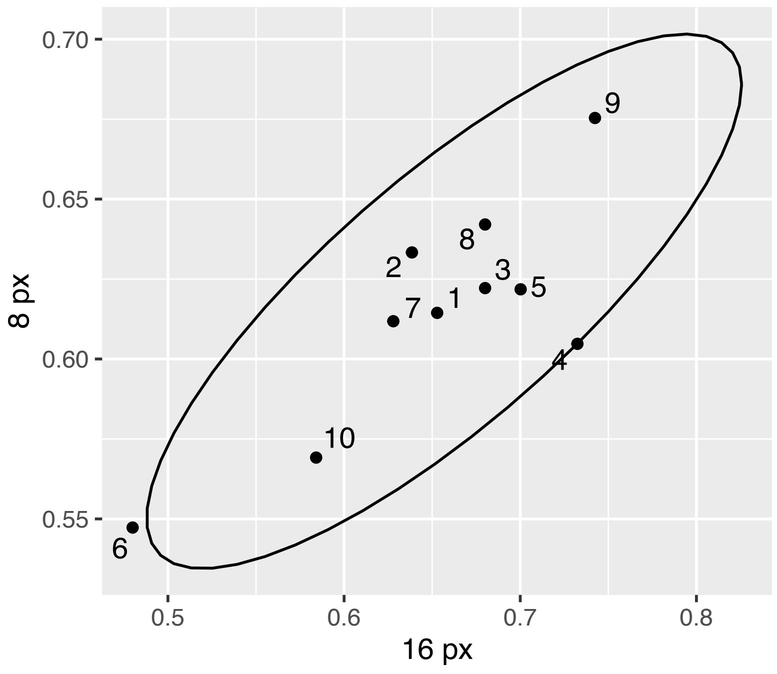

T1 glszm smallareahighgraylevelemphasis

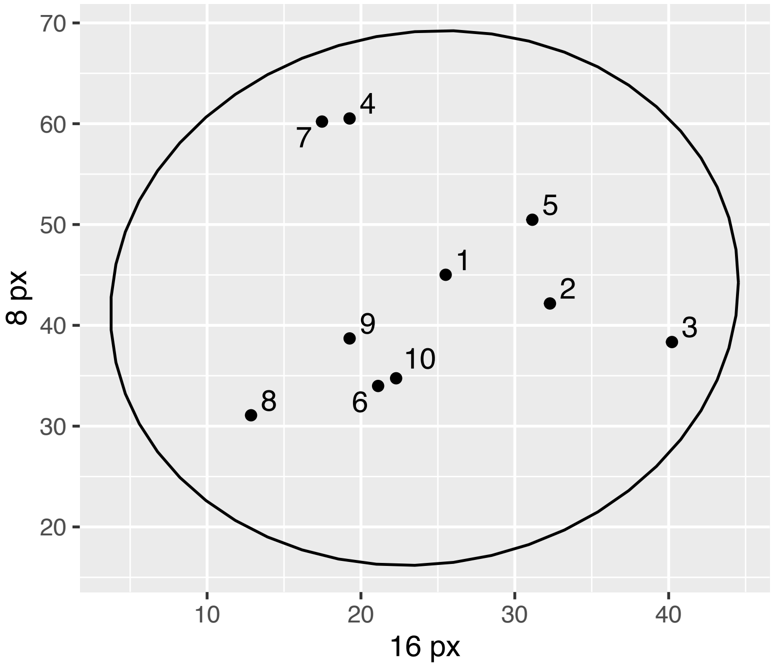

T1 glszm zonepercentage

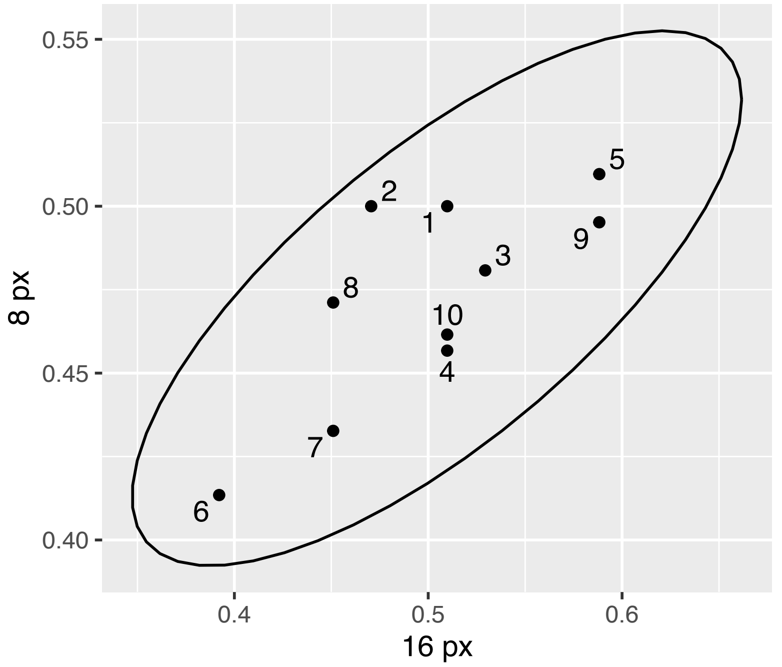

T1 glszm smallarealowgraylevelemphasis

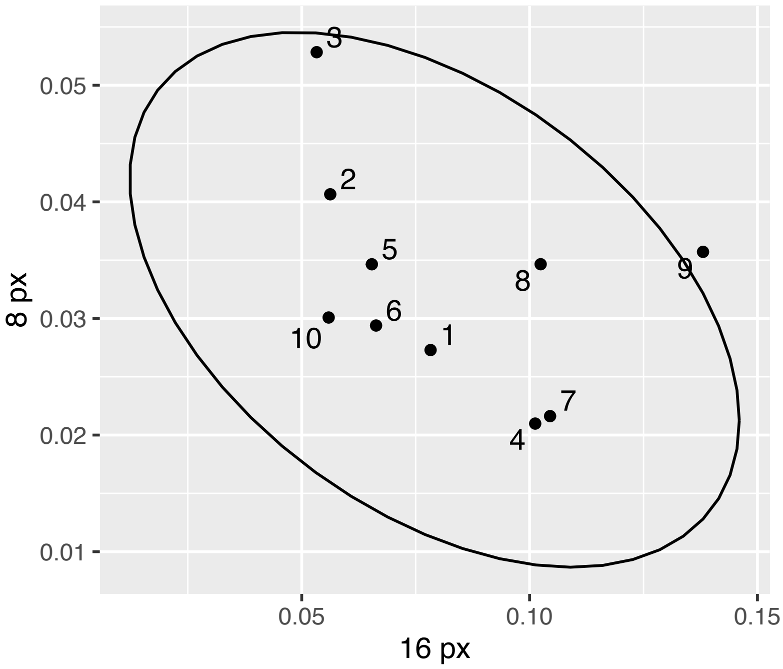

T1 glszm zonevariance

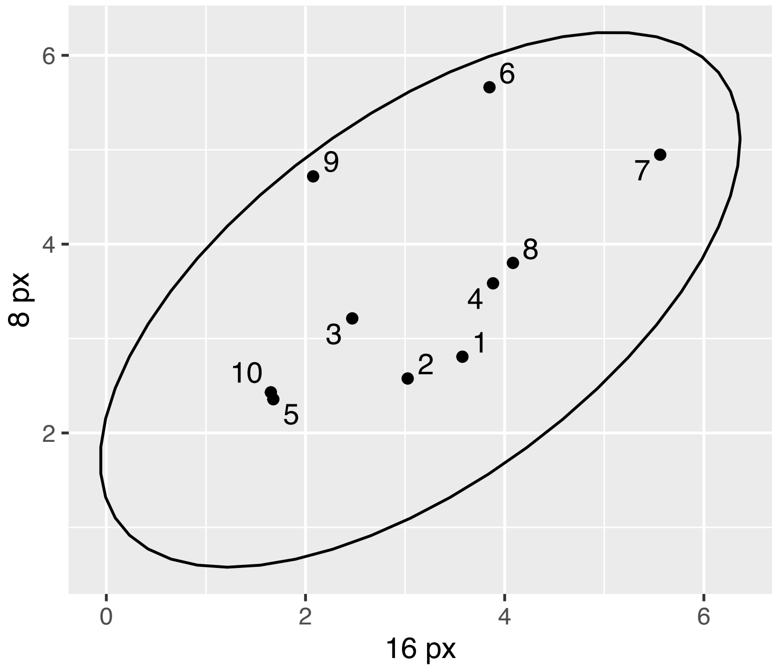

T1 glszm zoneentropy

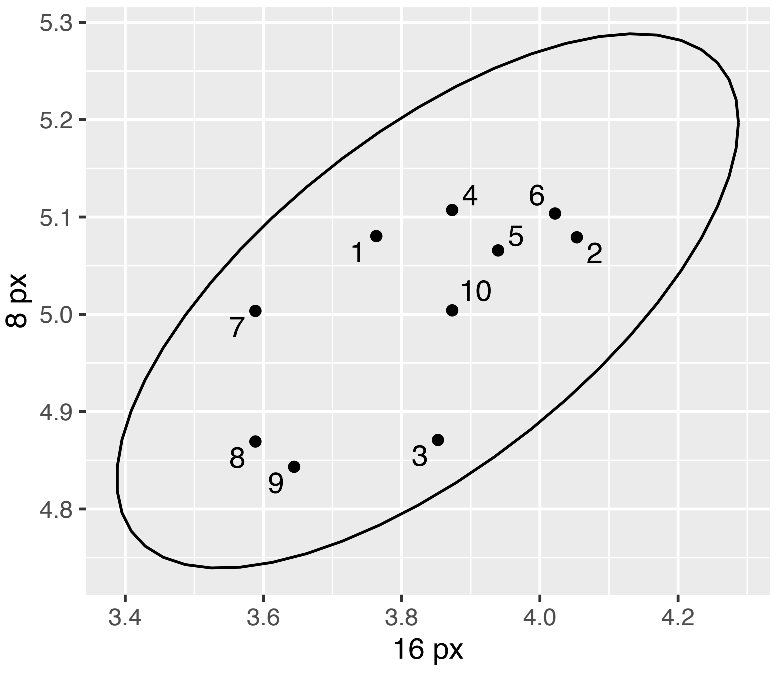

T1 gldm dependenceentropy

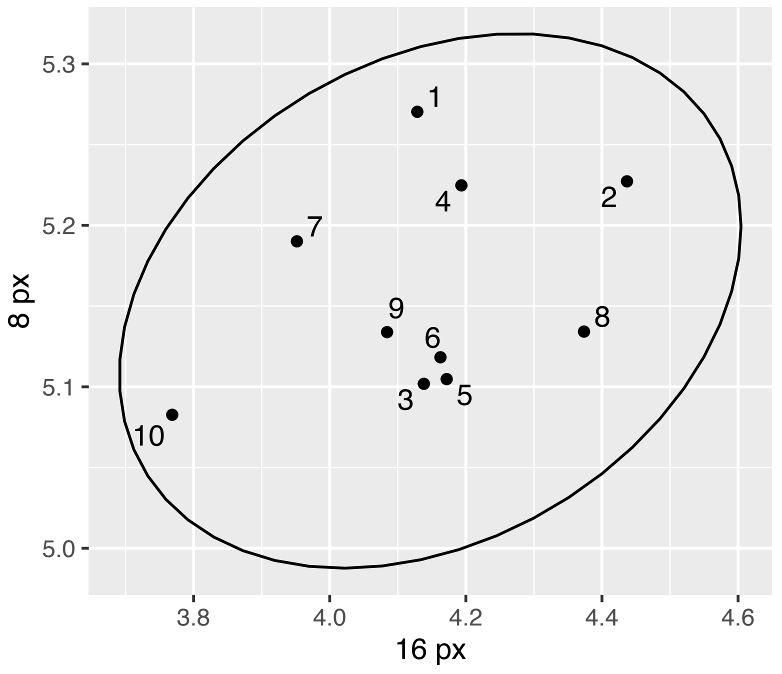

T1 gldm dependencenonuniformity

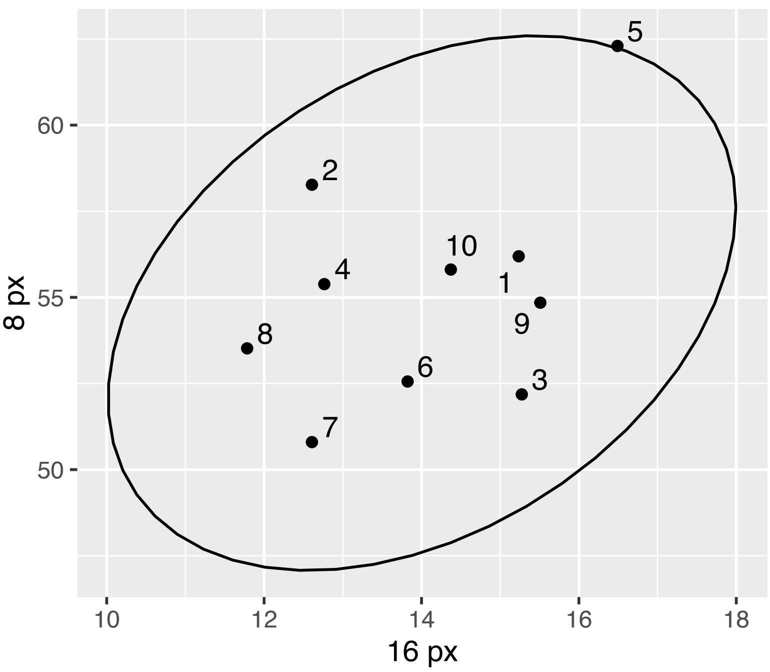

T1 gldm graylevelnonuniformity

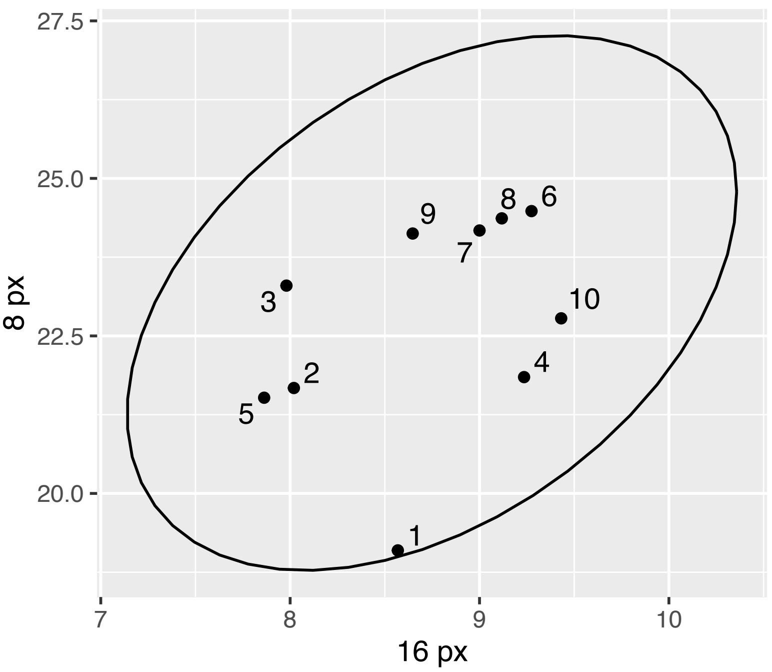

T1 gldm dependencenonuniformitynormalize

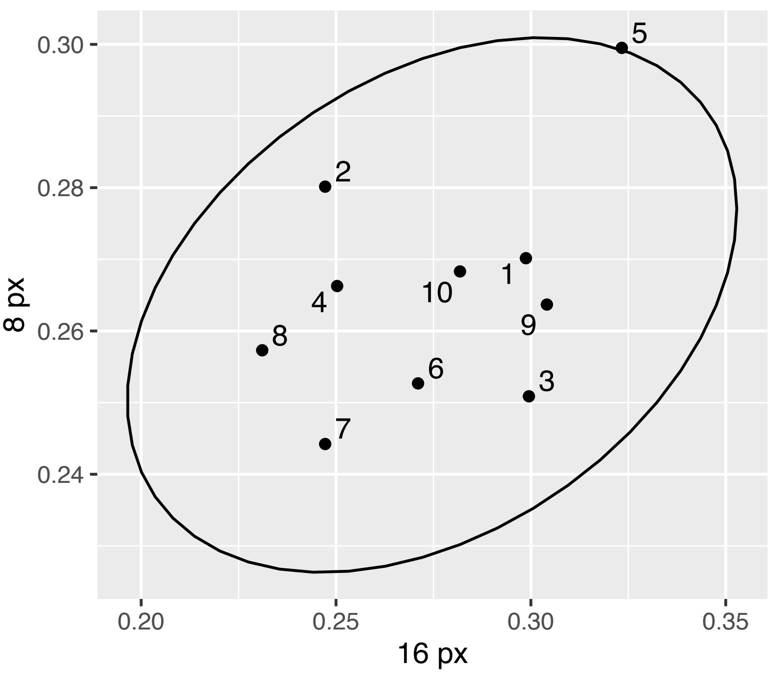

T1 gldm graylevelvariance

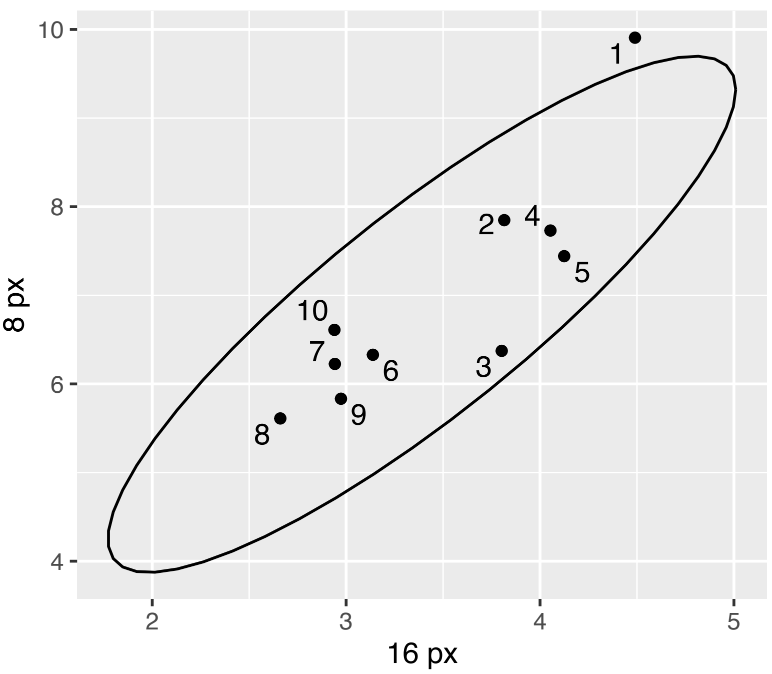

T1 gldm dependencevariance

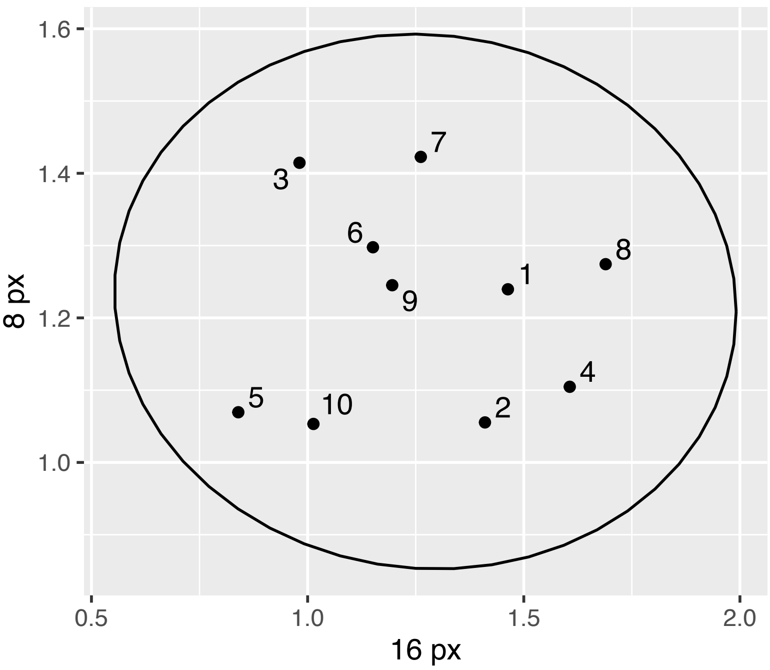

T1 gldm highgraylevelemphasis

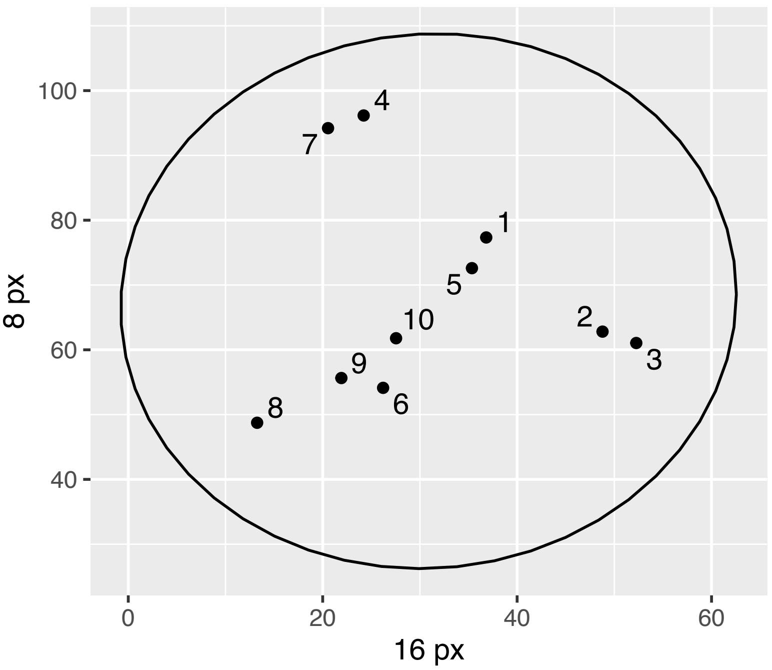

T1 gldm largedependenceemphasis

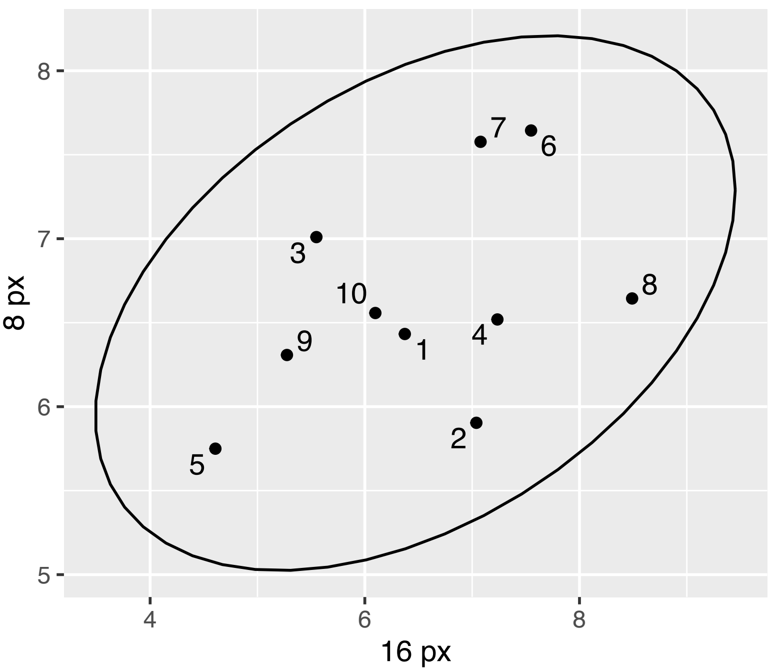

T1 gldm lowgraylevelemphasis

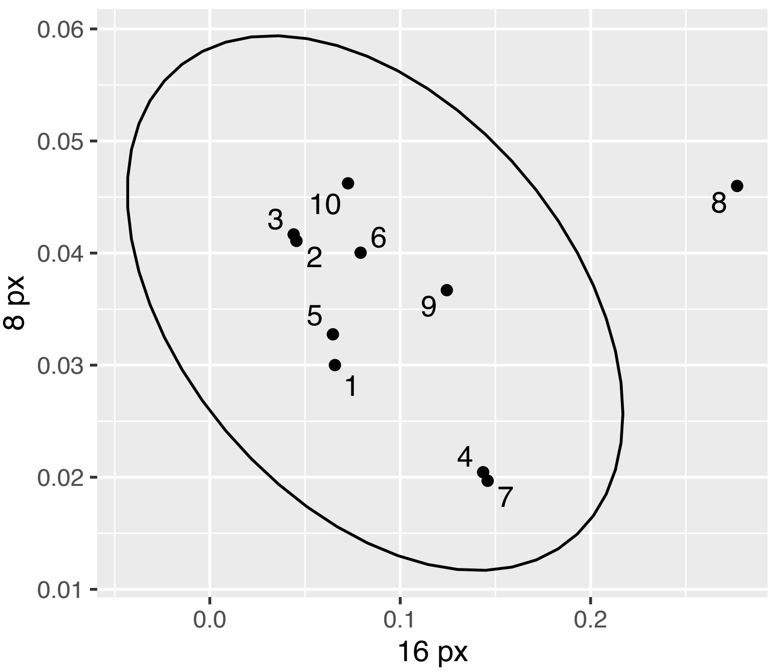

T1 gldm largedependencehighgraylevelemph

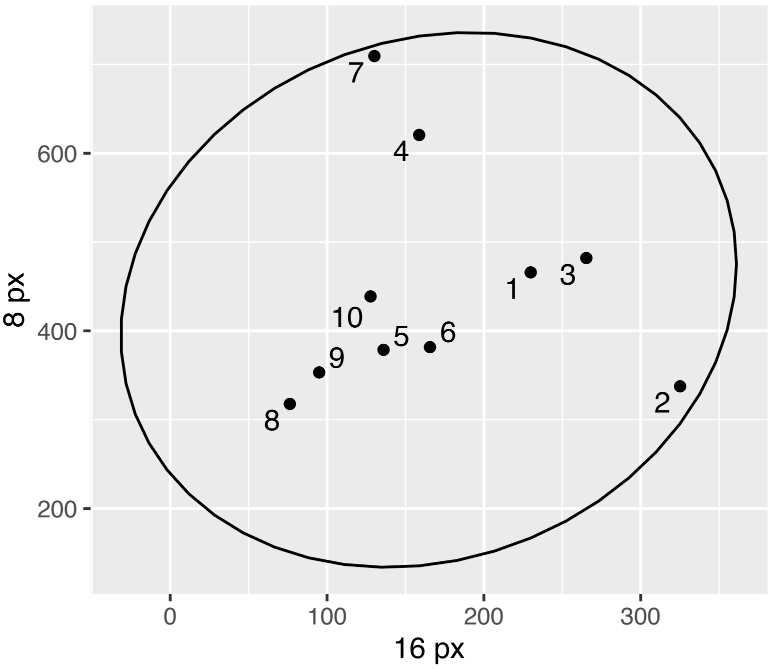

T1 gldm smalldependenceemphasis

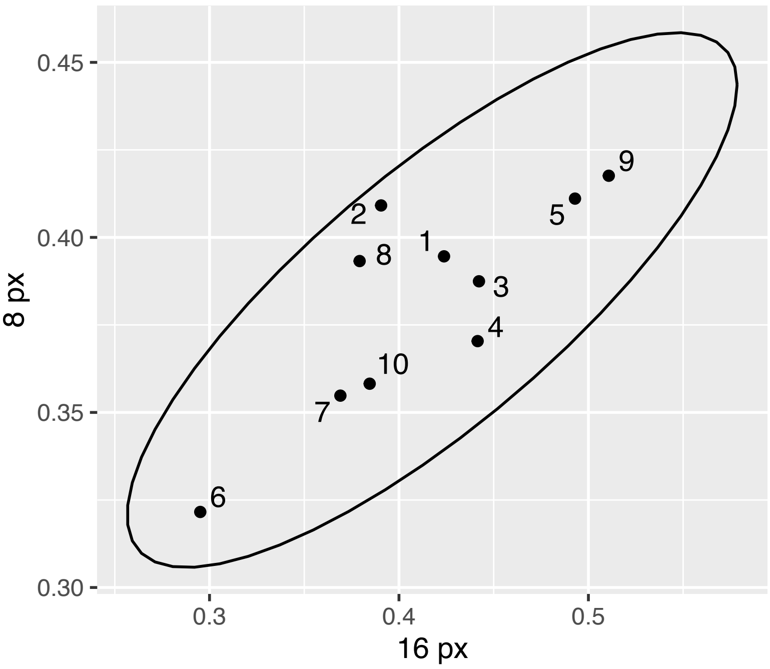

T1 gldm largedependencelowgraylevelempha

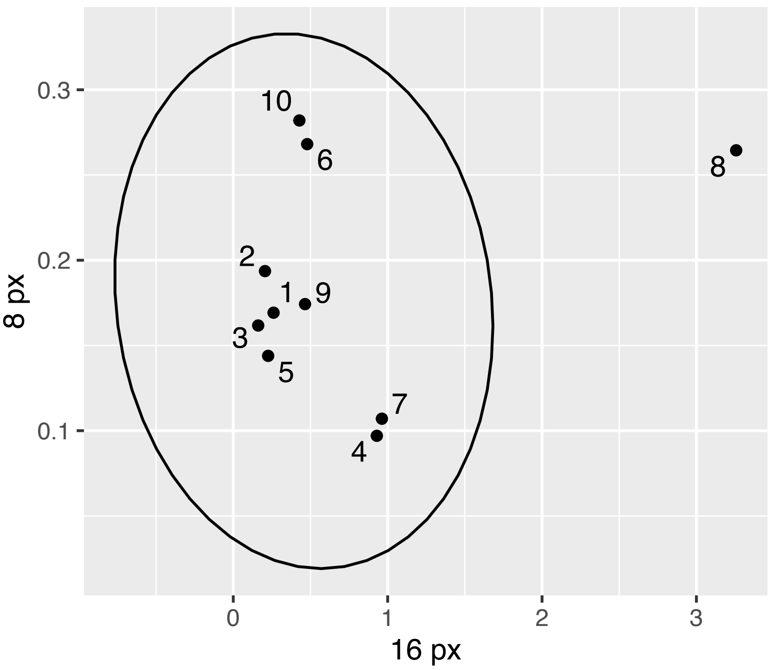

T1 gldm smalldependencehighgraylevelempha

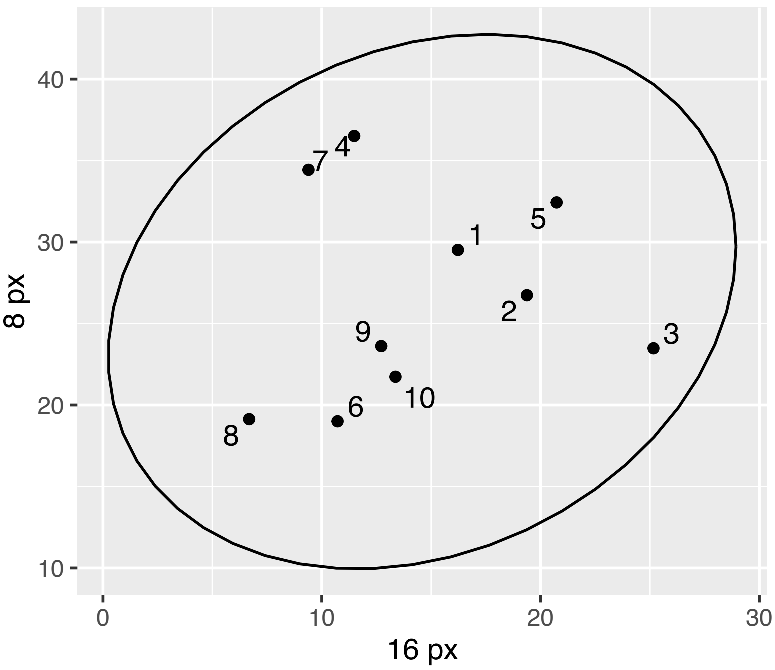

T1 gldm smalldependencelowgraylevelempha

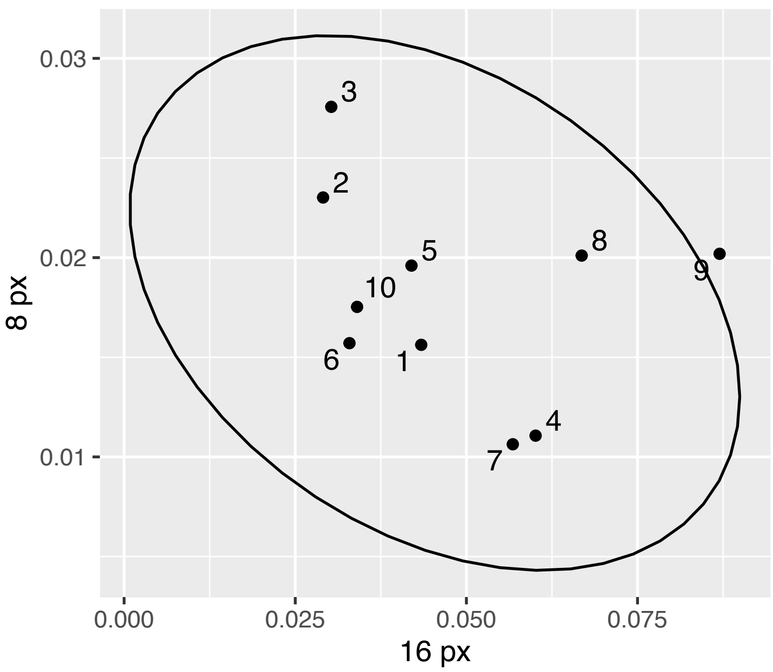

T1 ngtdm complexity

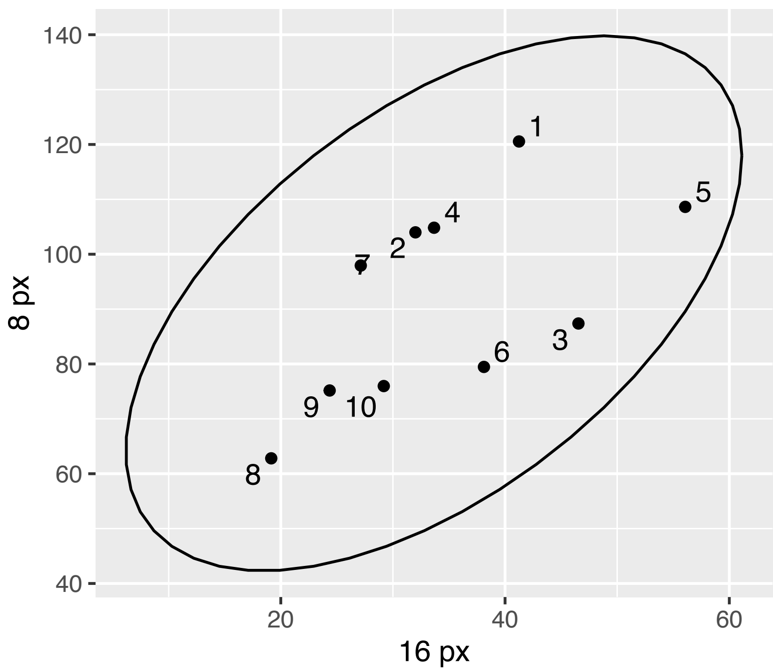

T1 ngtdm busyness

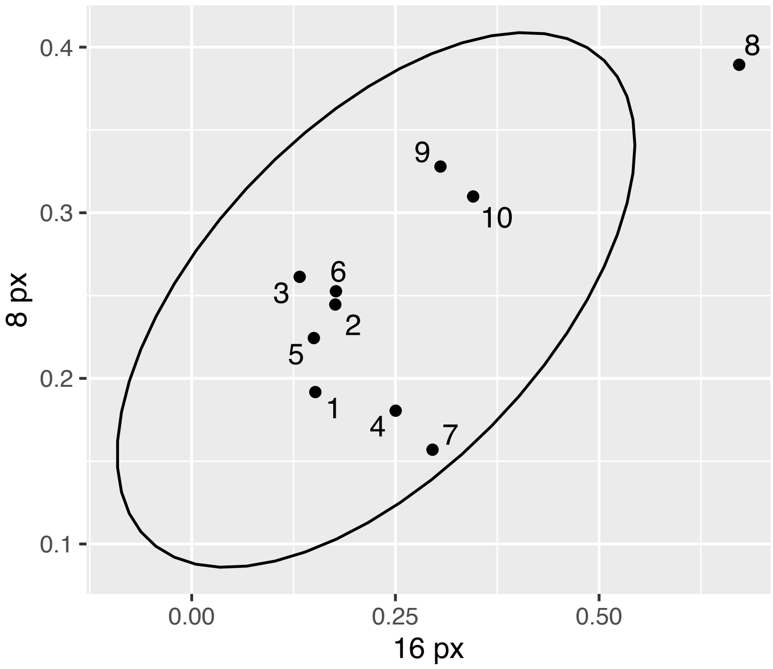

T1 ngtdm contrast

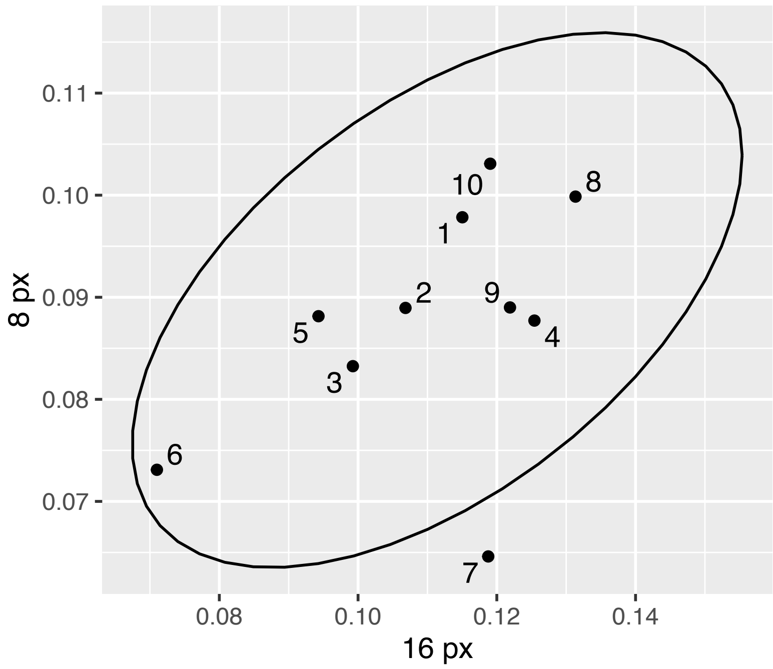

T1 ngtdm coarseness

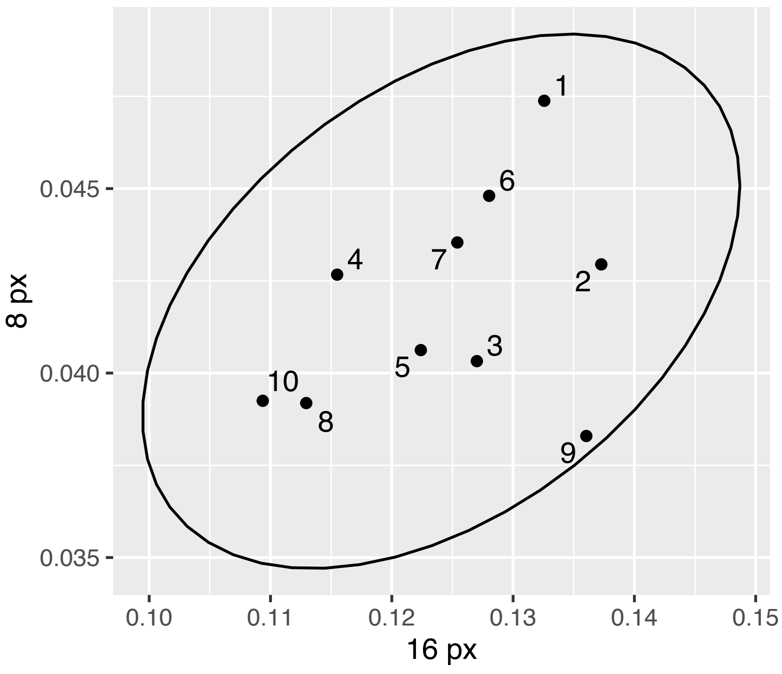

T1 ngtdm strength

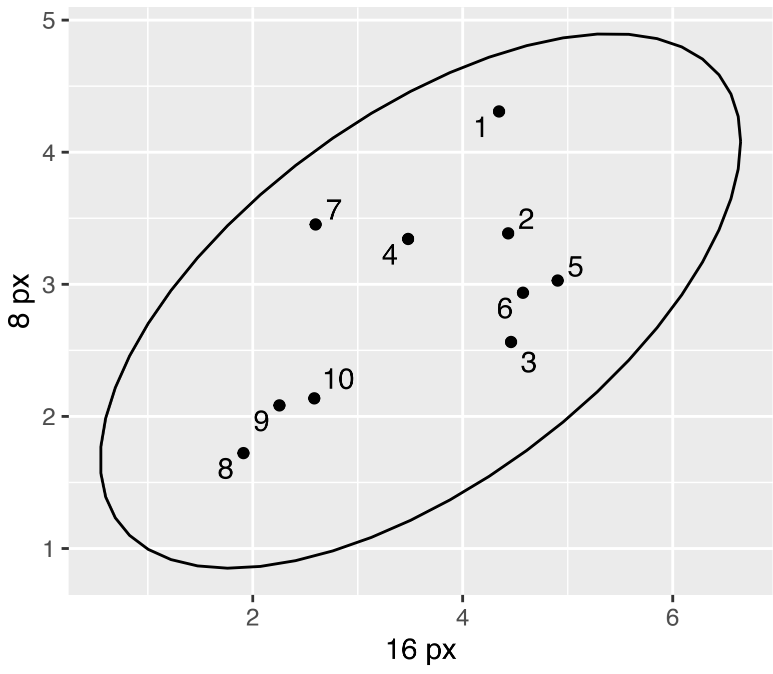

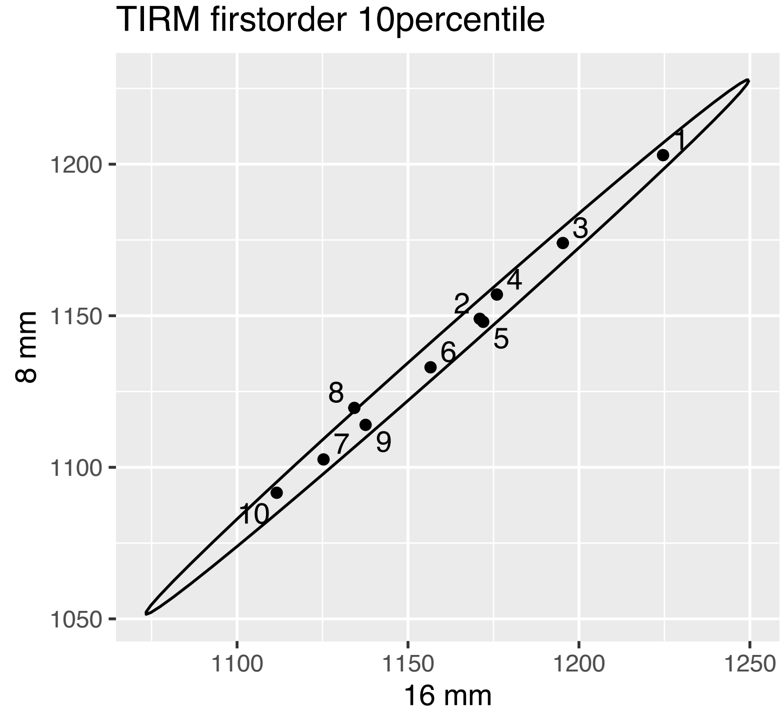
TIRM firstorder 10percentile
8 mm
16 mm
1050
1100
1150
1200
1100
1150
1200
1250

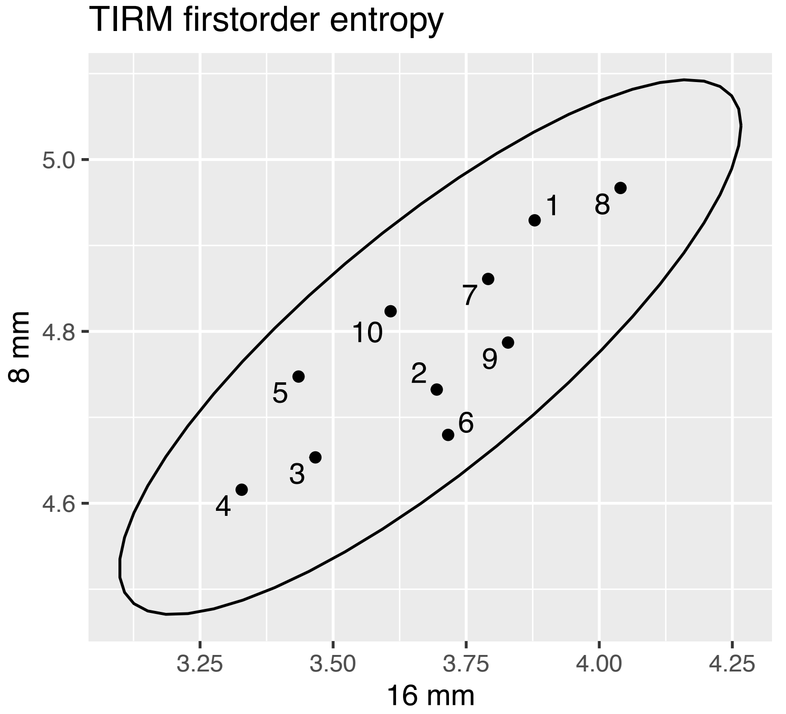
TIRM firstorder entropy
8 mm
16 mm
4.6
4.8
5.0
3.25
3.50
3.75
4.00
4.25

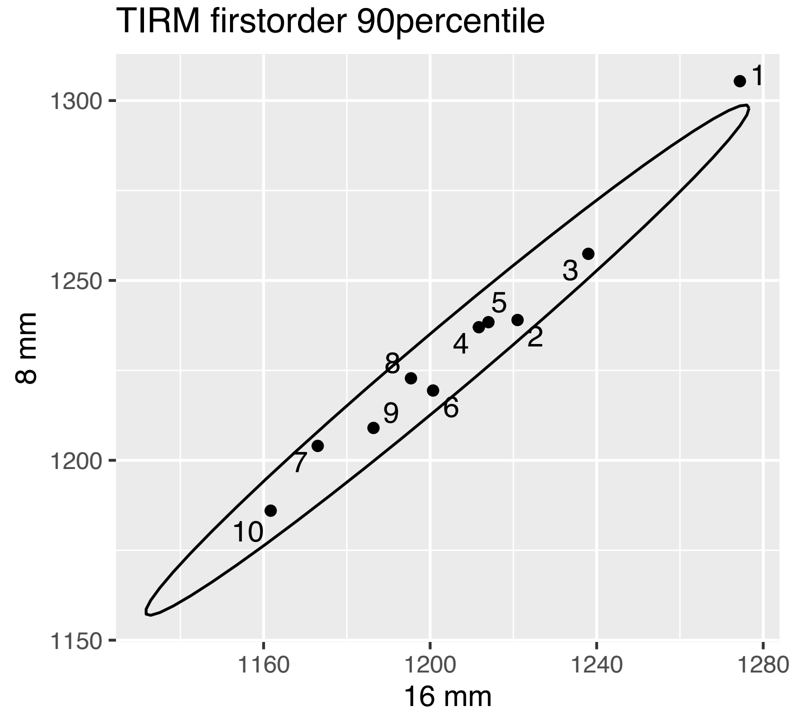
TIRM firstorder 90percentile
8 mm
16 mm
1150
1200
1250
1300
1160
1200
1240
1280

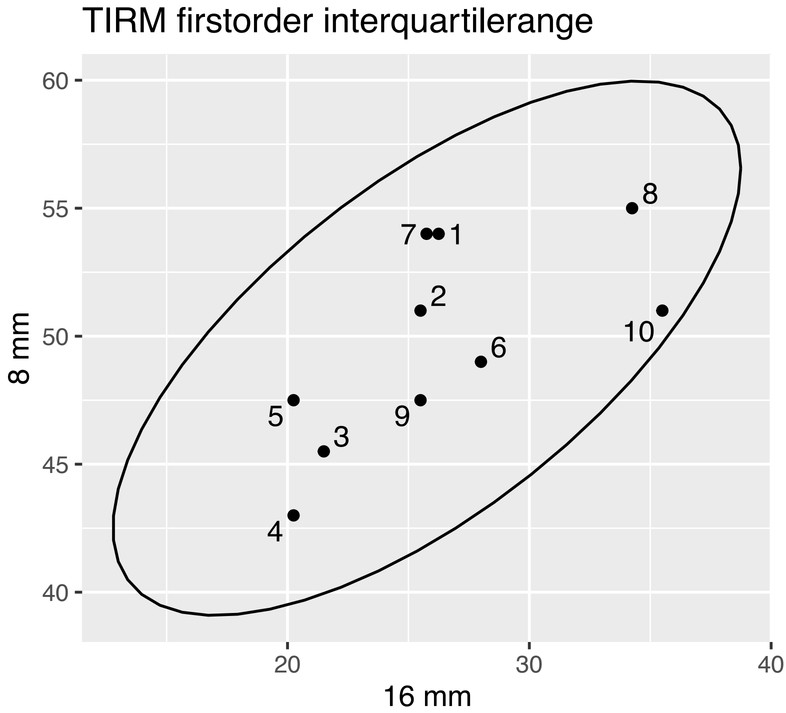
TIRM firstorder interquartilerange
8 mm
16 mm
40
45
50
55
60
20
30
40

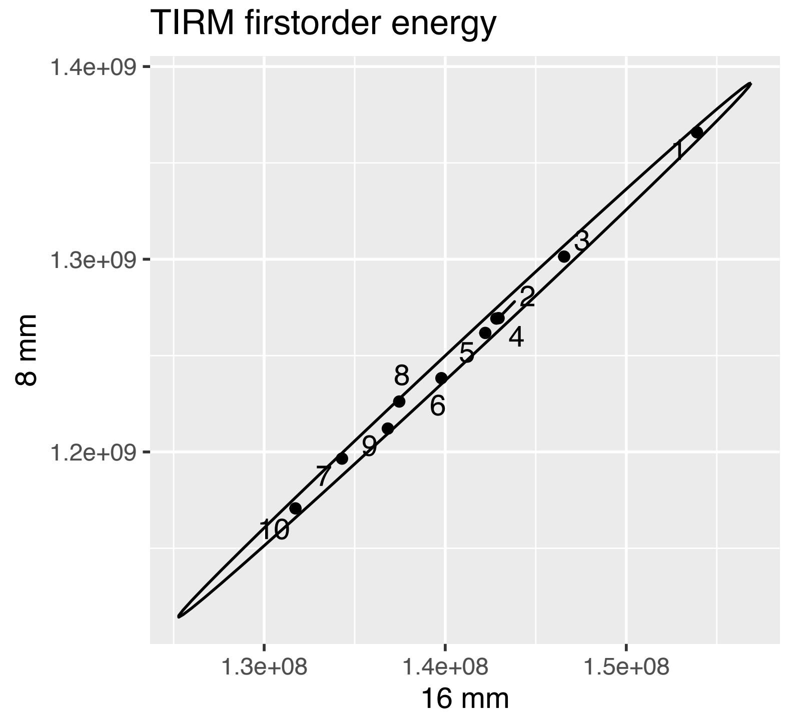
TIRM firstorder energy
8 mm
16 mm
1.2e+09
1.3e+09
1.4e+09
1.3e+08
1.4e+08
1.5e+08

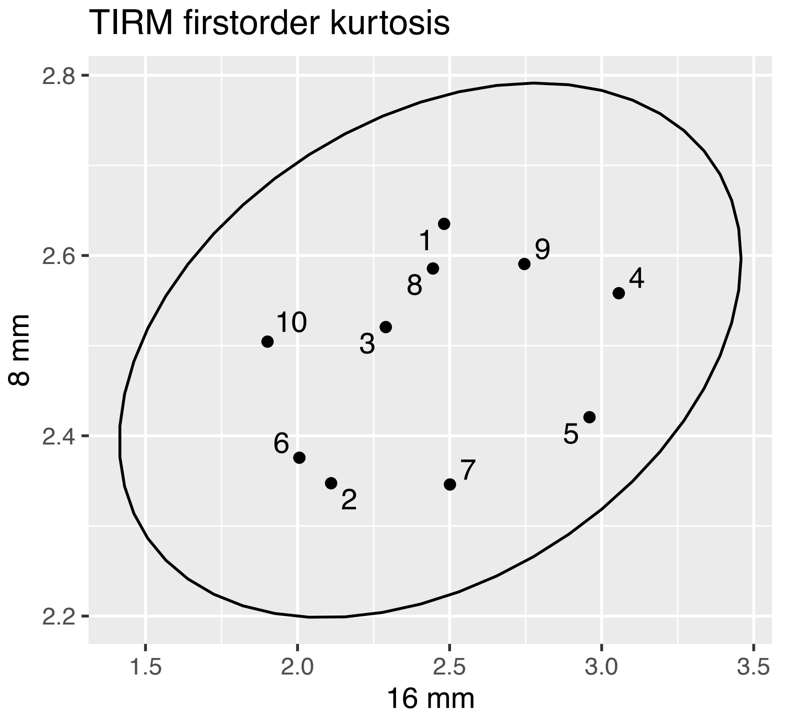
TIRM firstorder kurtosis
8 mm
16 mm
2.2
2.4
2.6
2.8
1.5
2.0
2.5
3.0
3.5

TIRM firstorder maximum

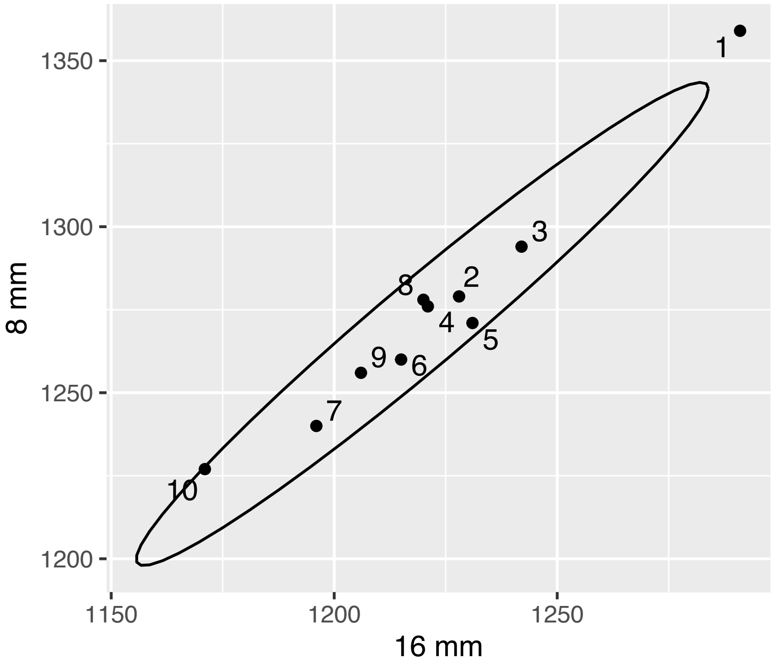

TIRM firstorder median

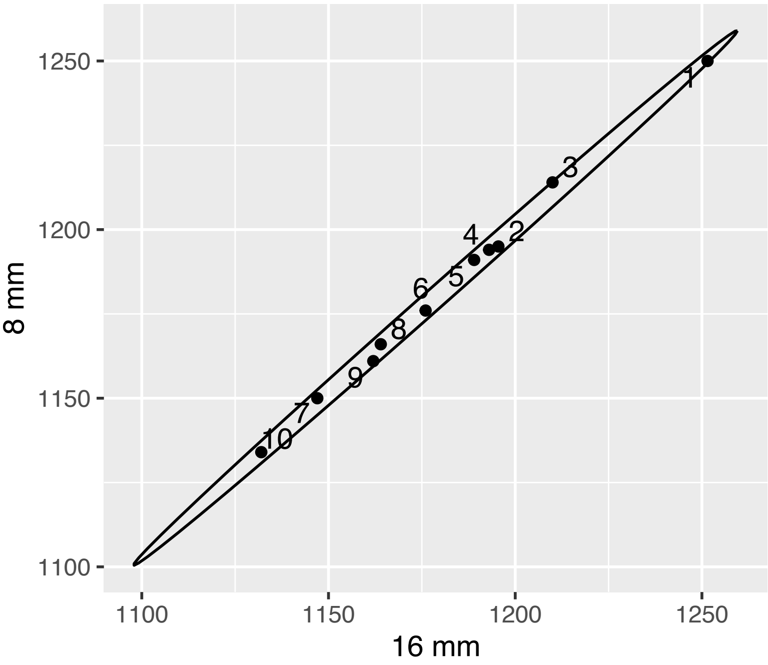

TIRM firstorder meanabsolutedeviation

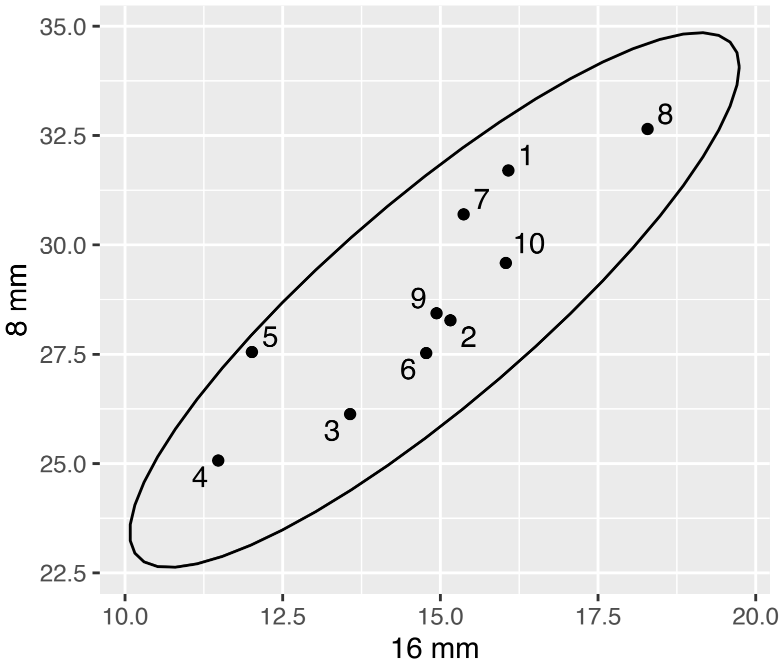

TIRM firstorder minimum

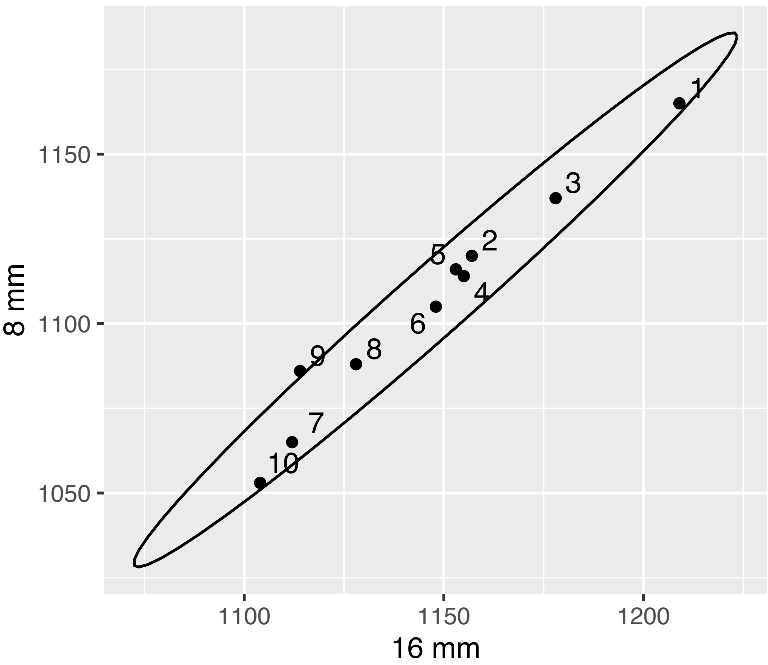

TIRM firstorder mean

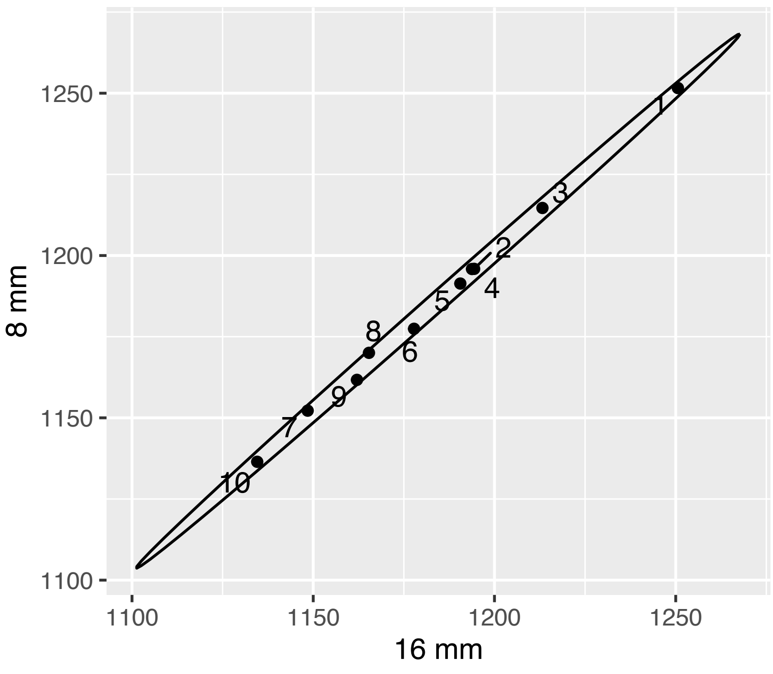

TIRM firstorder range

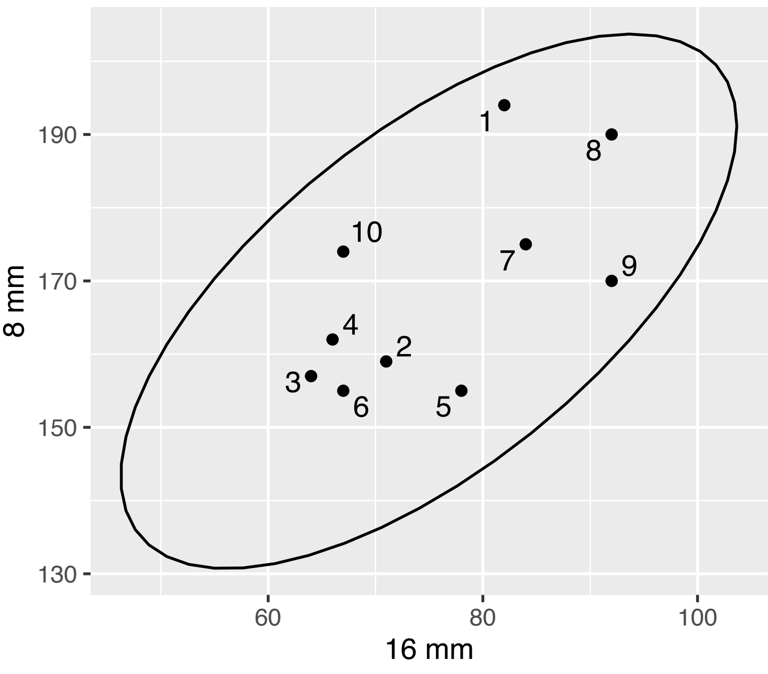

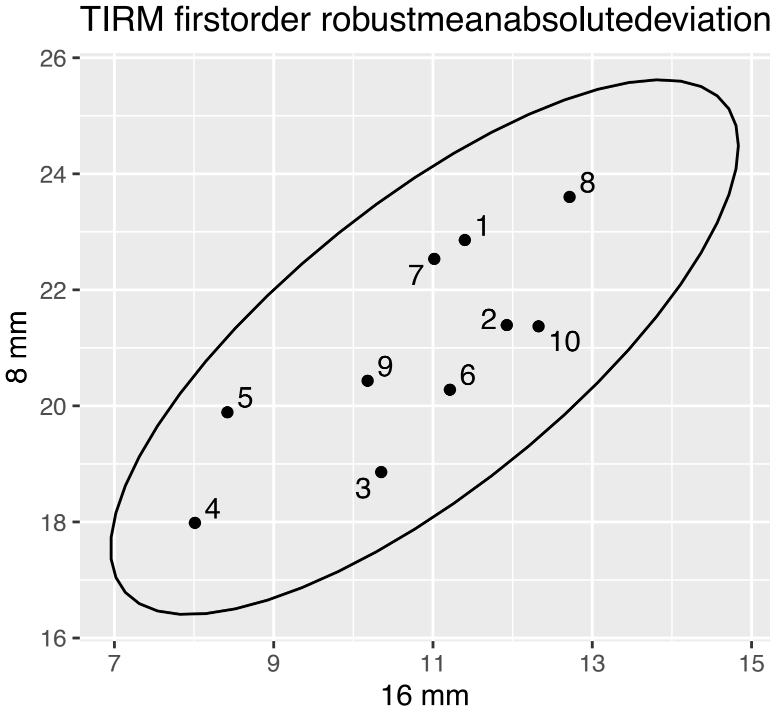

TIRM firstorder robustmeanabsolutedeviation
8 mm
16 mm

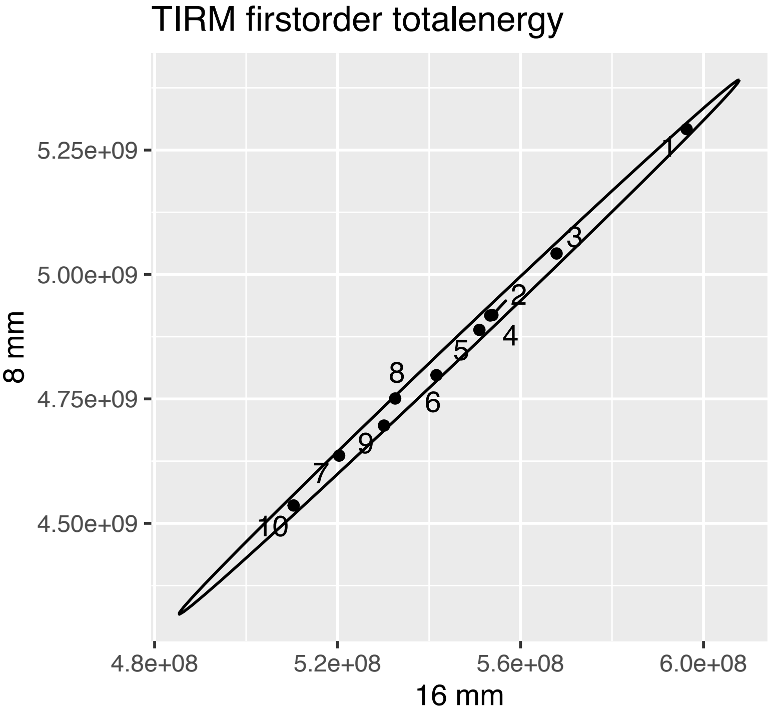

TIRM firstorder totalenergy
8 mm
16 mm

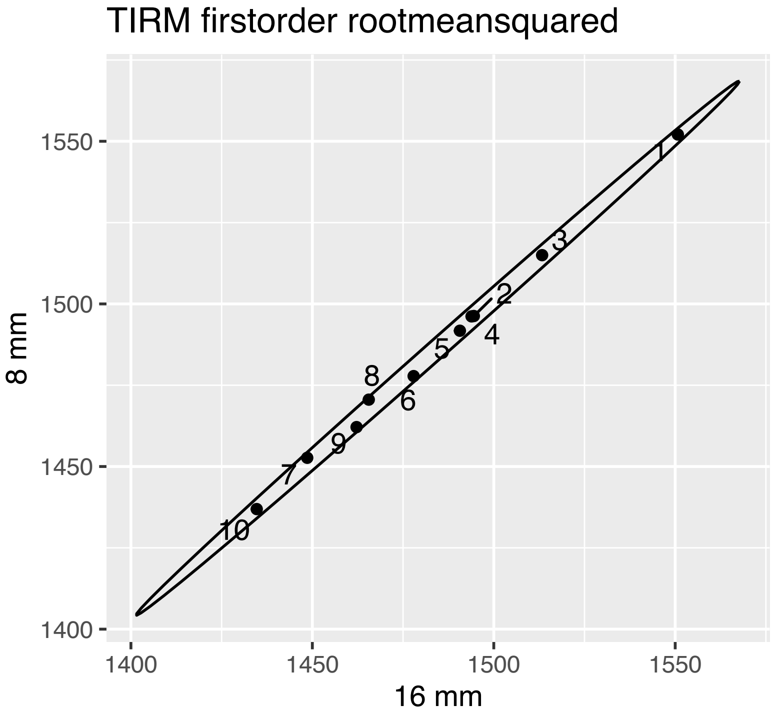

TIRM firstorder rootmeansquared
8 mm
16 mm

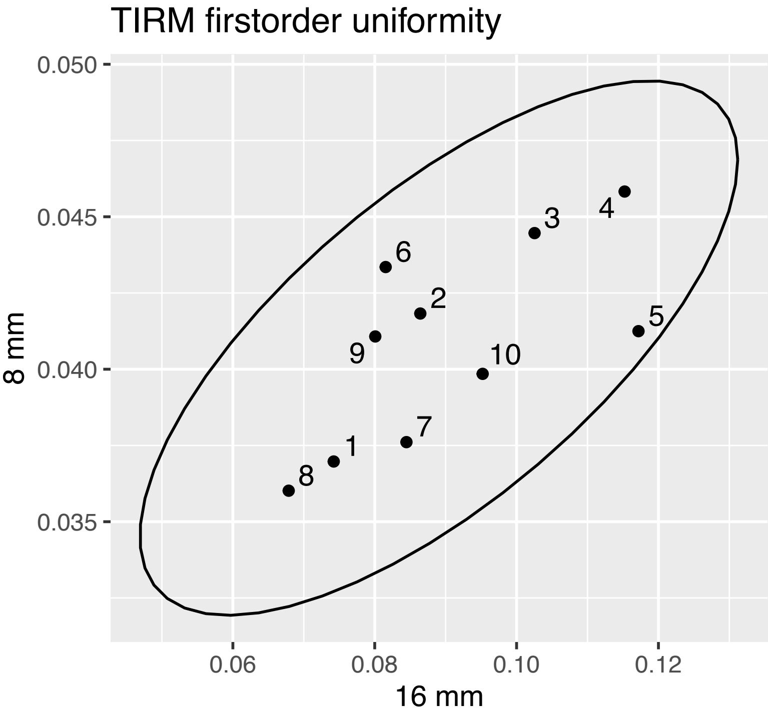

TIRM firstorder uniformity
8 mm
16 mm

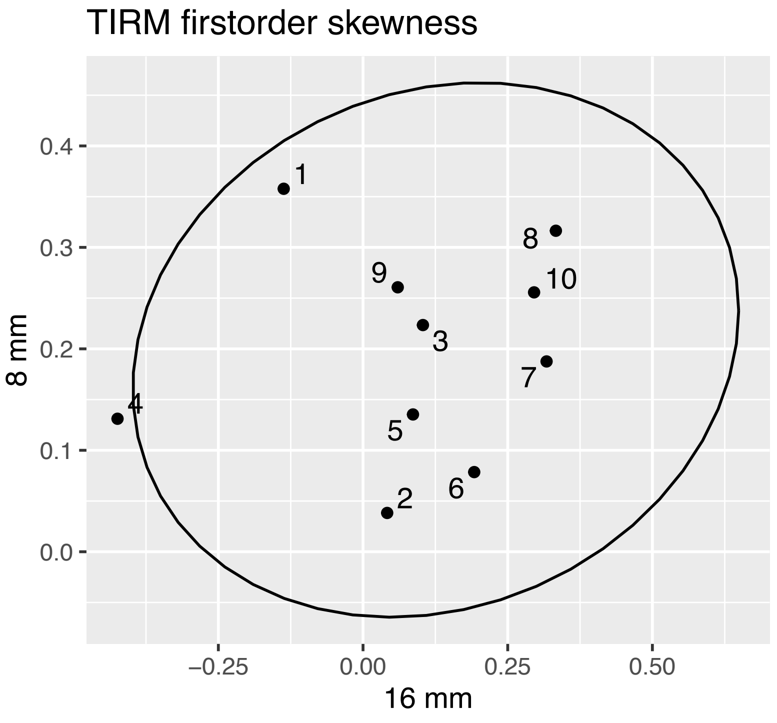

TIRM firstorder skewness
8 mm
16 mm

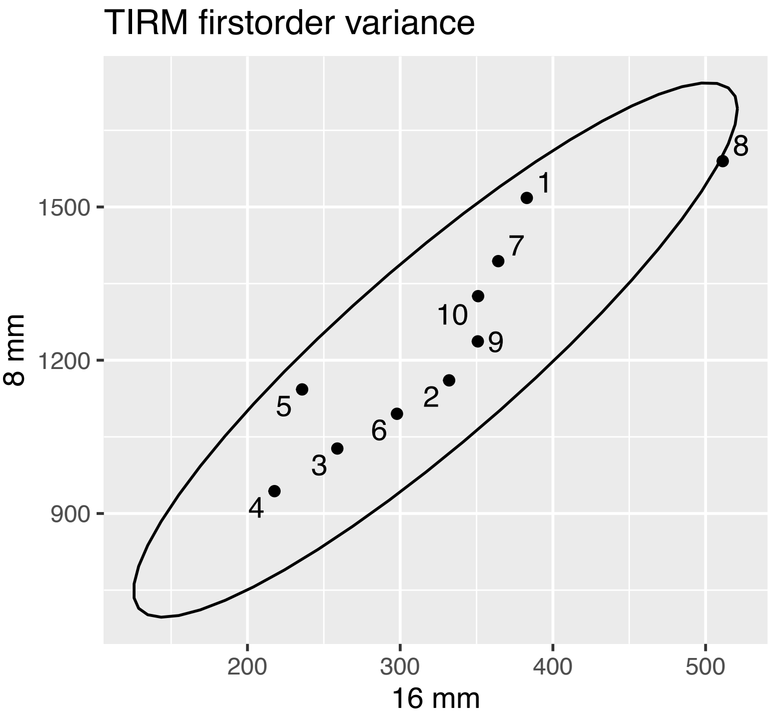

TIRM firstorder variance
8 mm
16 mm

TIRM glcm autocorrelation

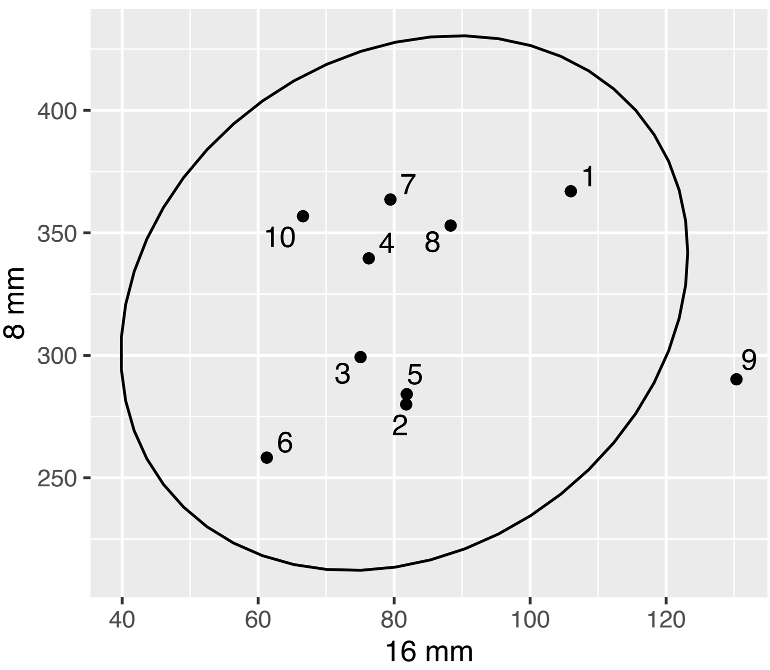

TIRM glcm clustertendency

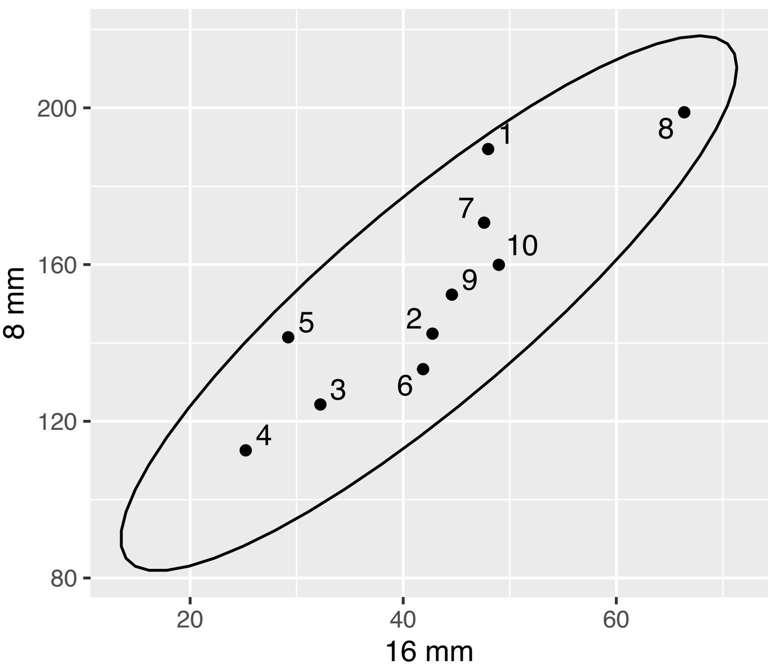

TIRM glcm clusterprominence

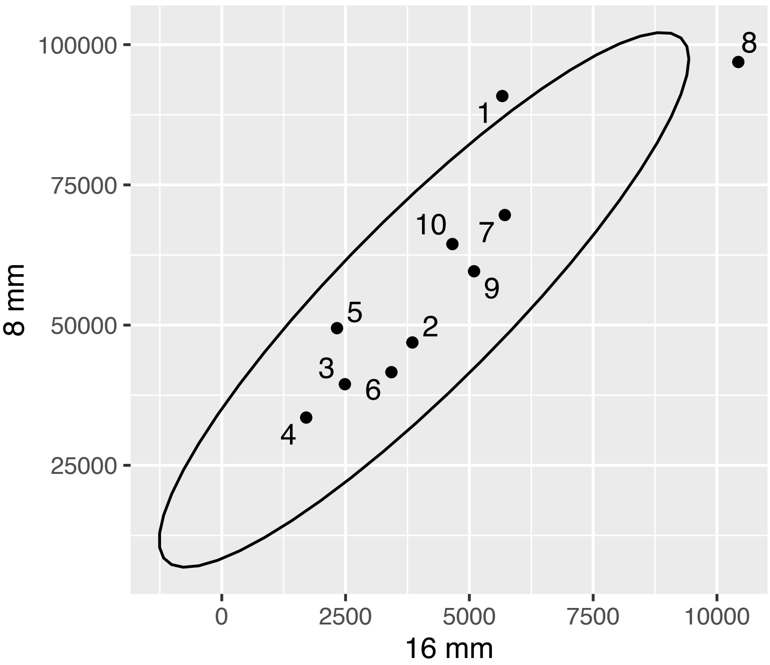

TIRM glcm contrast

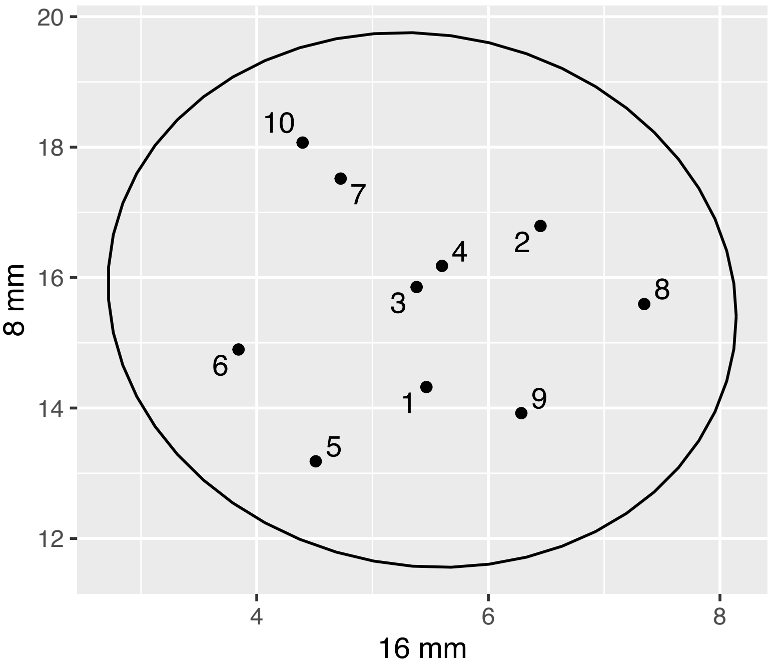

TIRM glcm clustershade

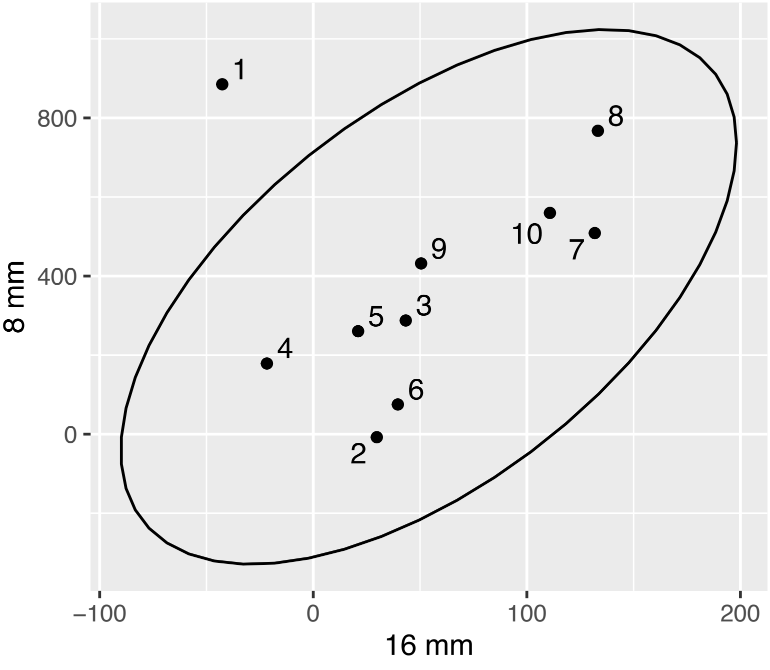

TIRM glcm correlation

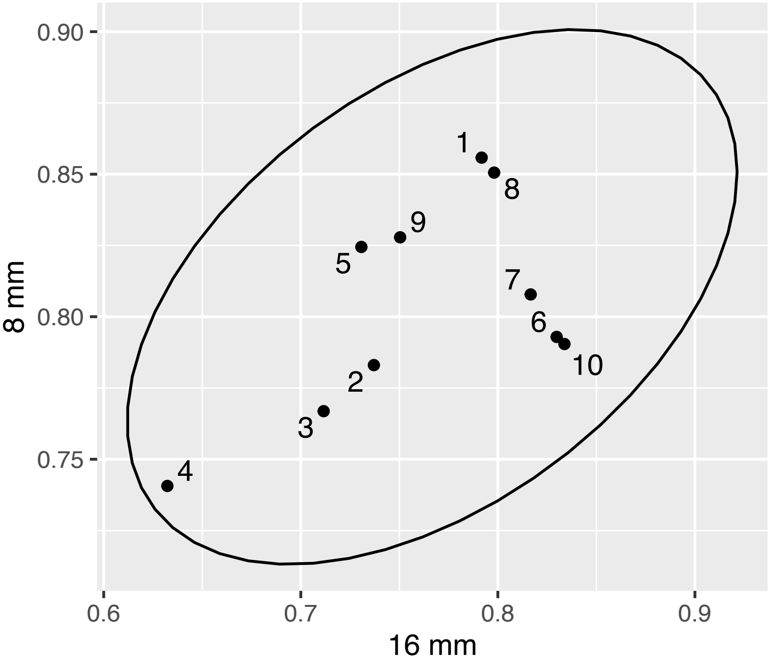

TIRM glcm differenceaverage

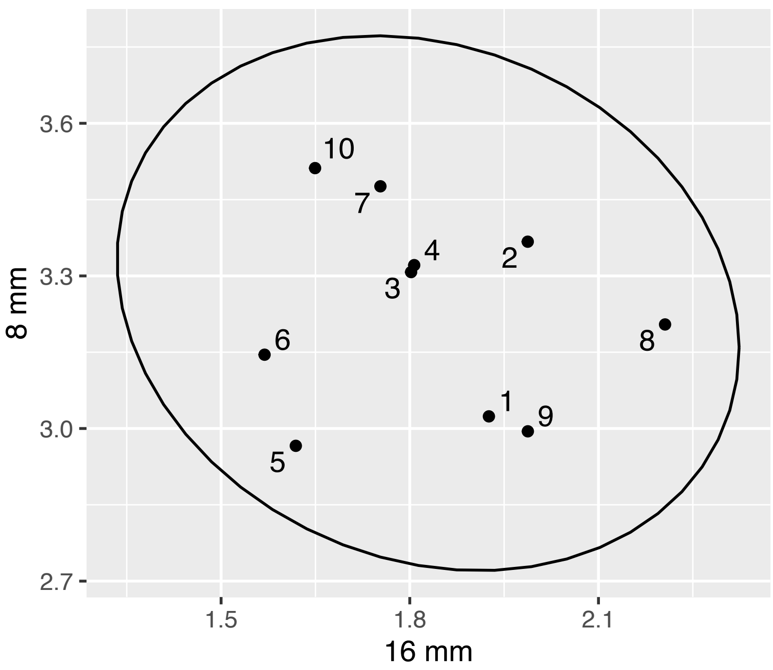

TIRM glcm id

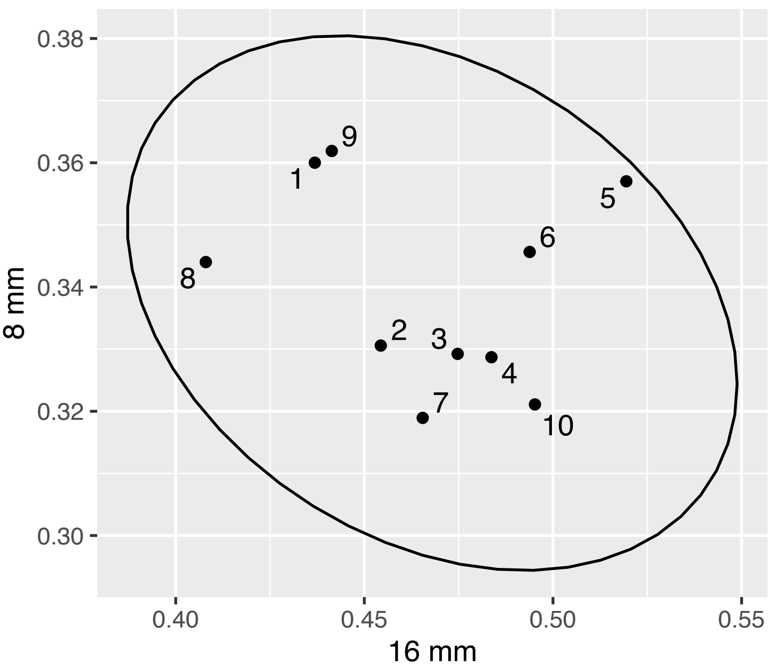

TIRM glcm differenceentropy

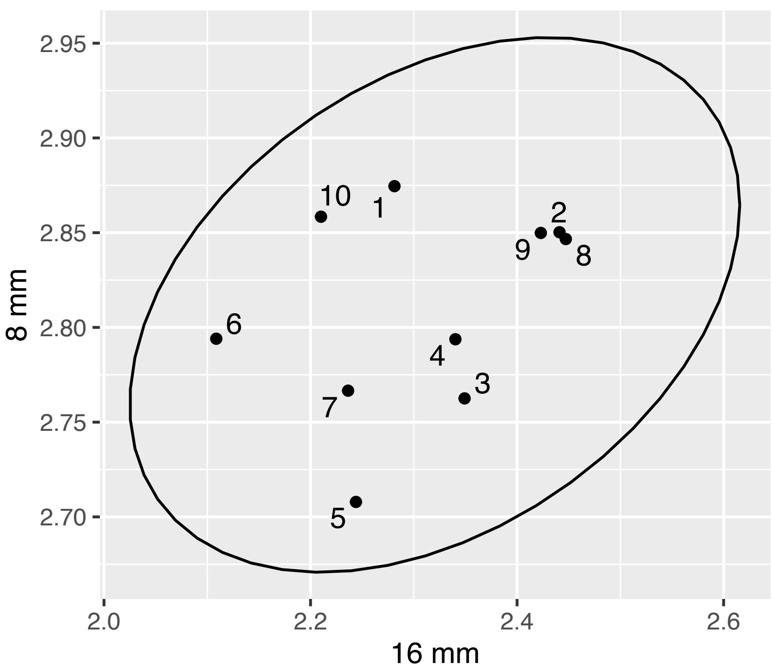

TIRM glcm idm

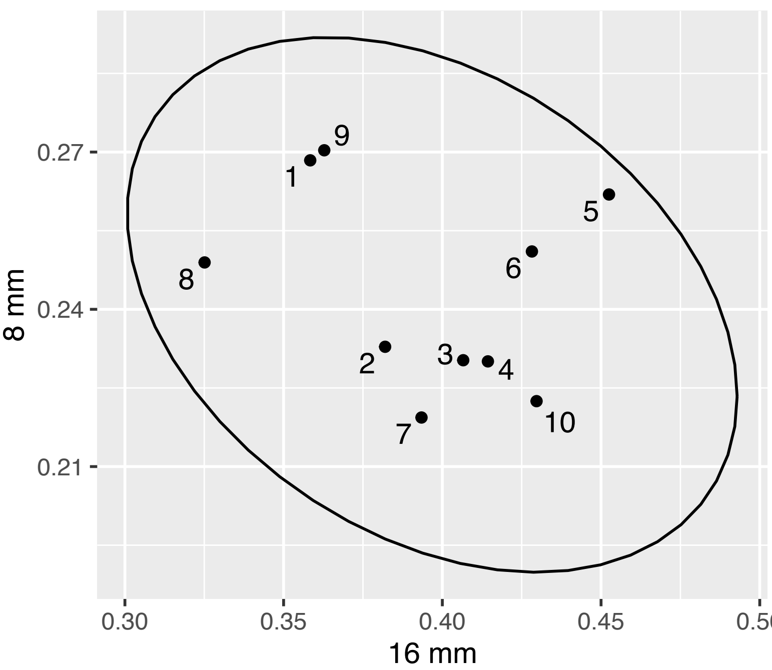

TIRM glcm differencevariance

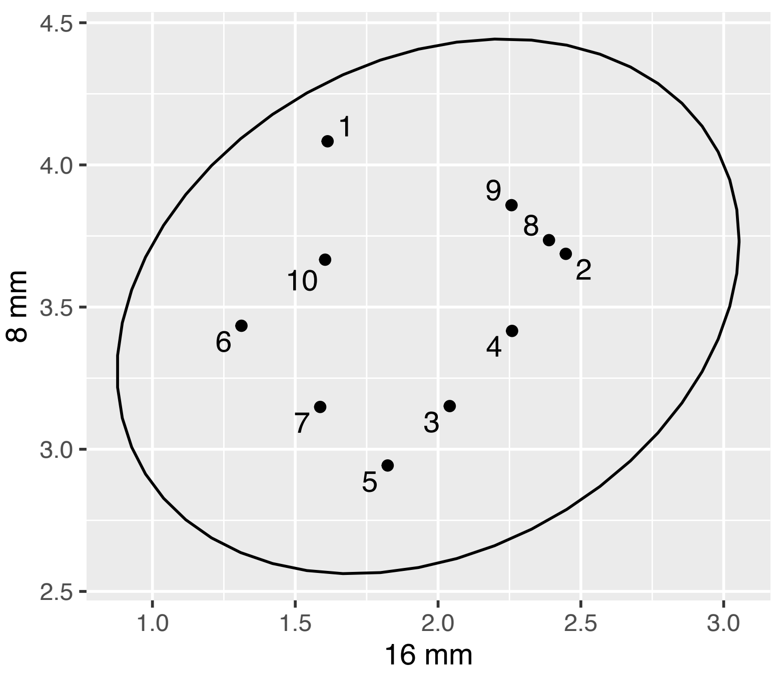

TIRM glcm idmn

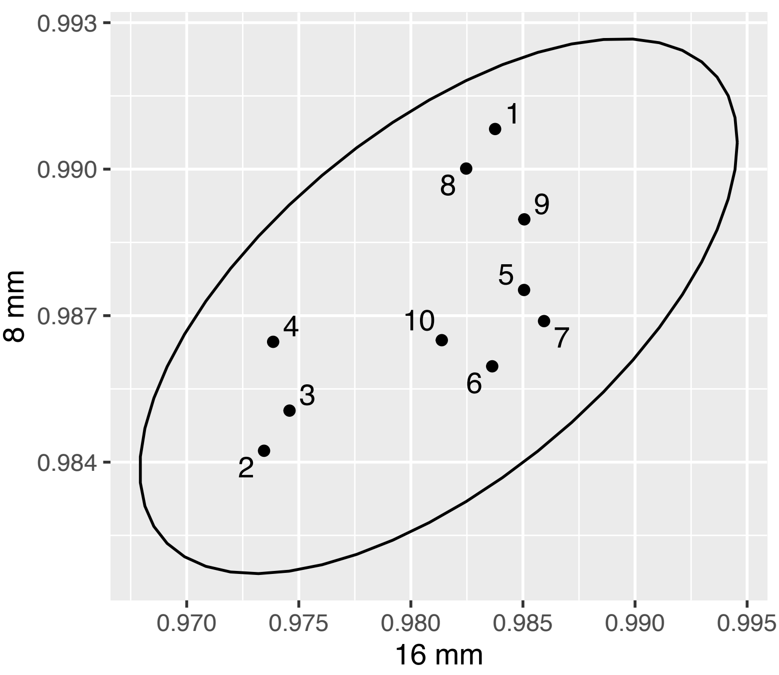

TIRM glcm idn

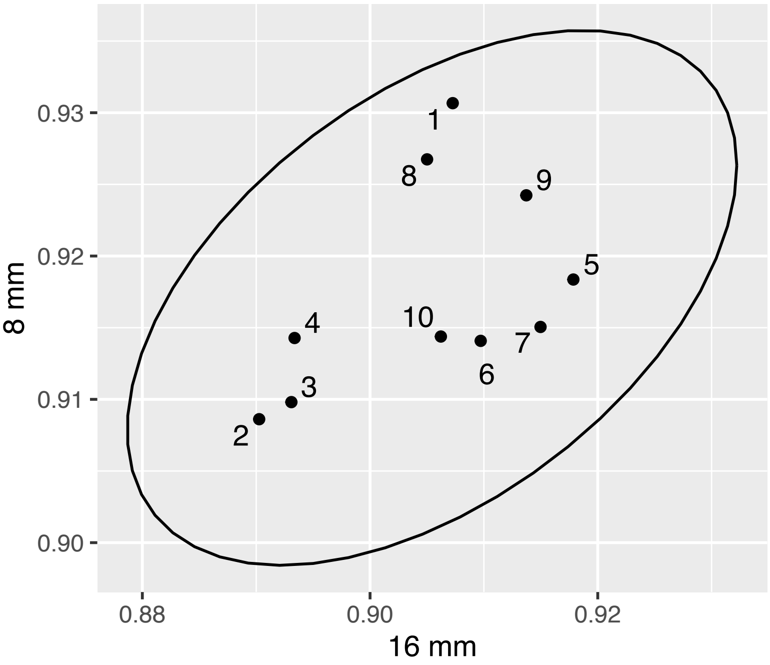

TIRM glcm inversevariance

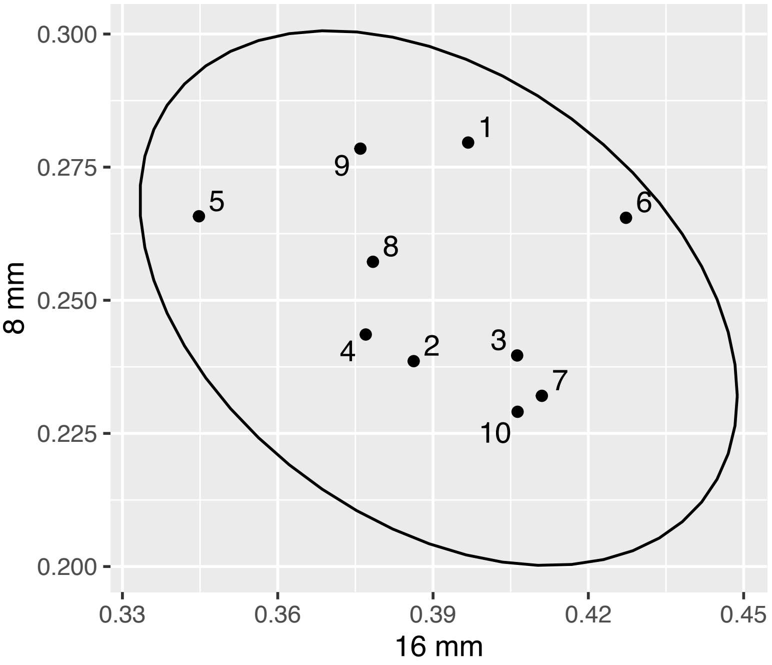

TIRM glcm imc1

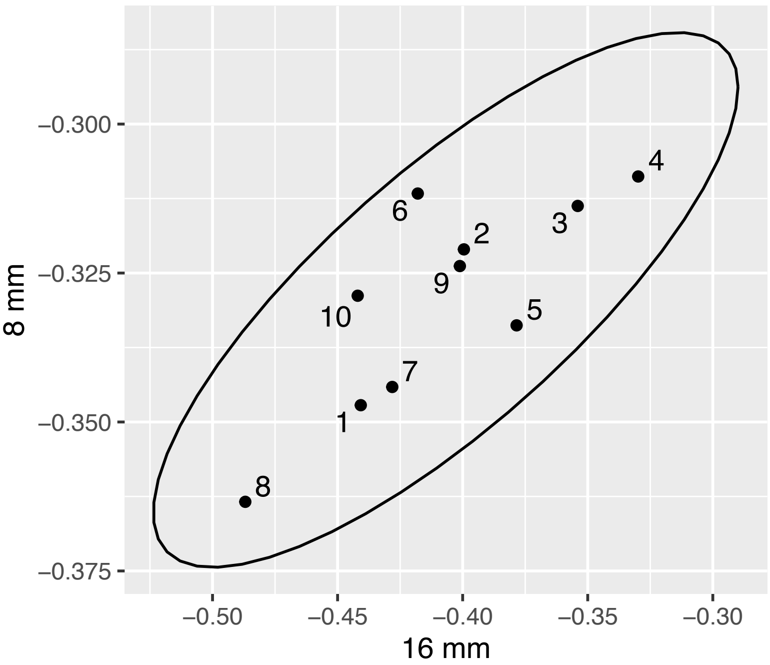

TIRM glcm jointaverage

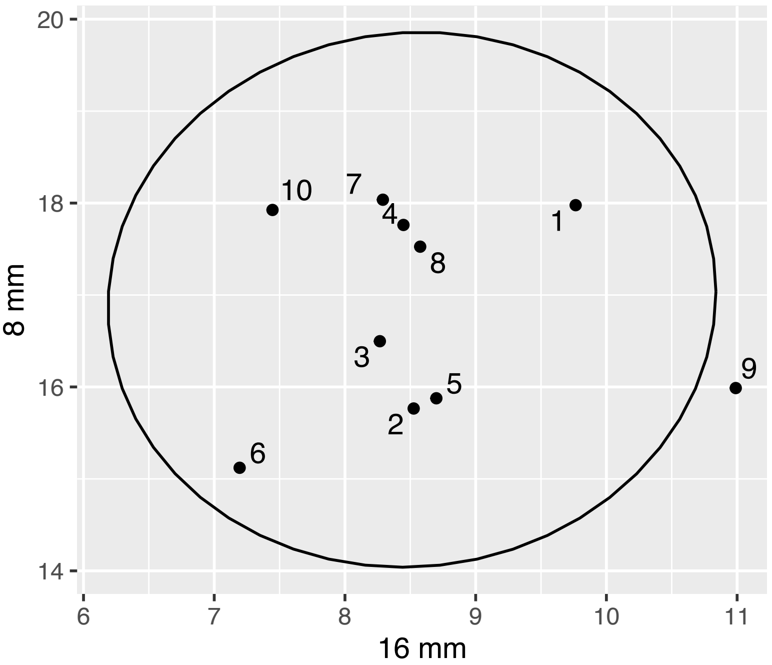

TIRM glcm imc2

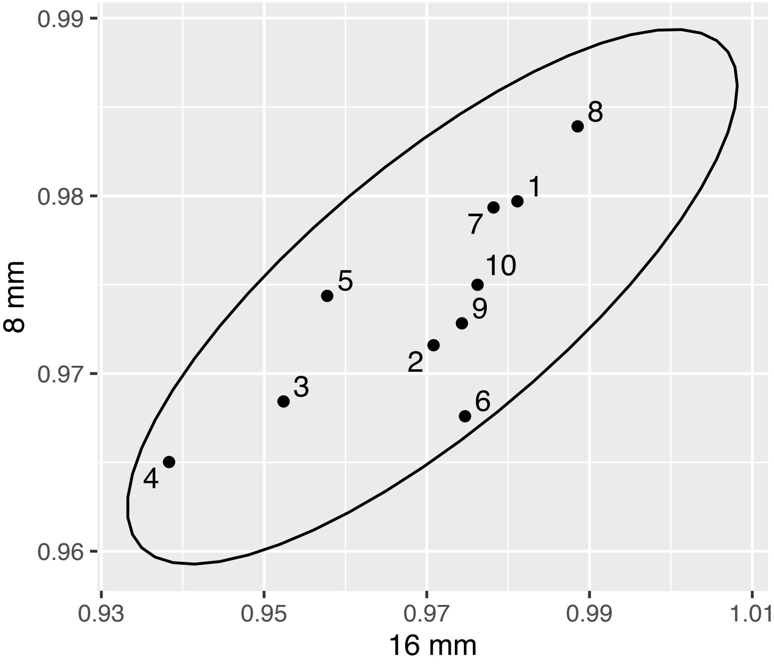

TIRM glcm jointenergy

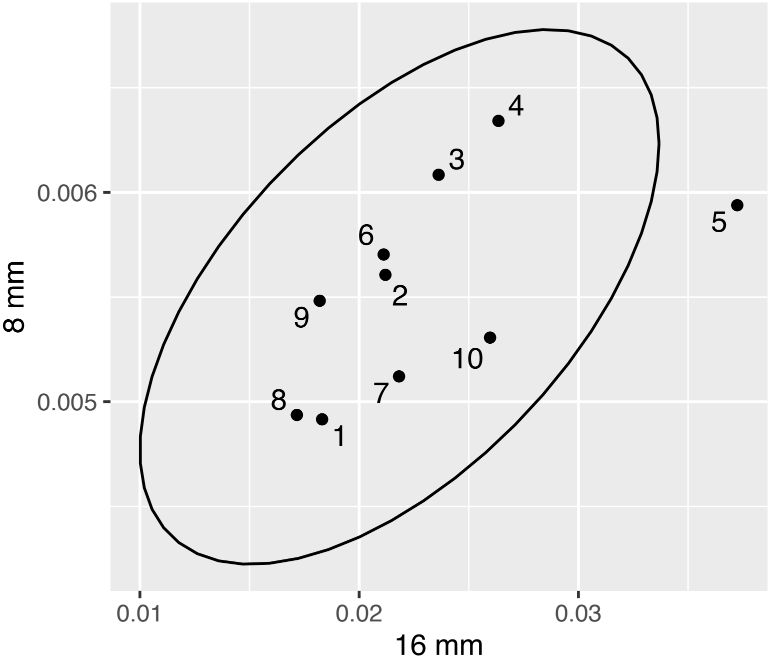

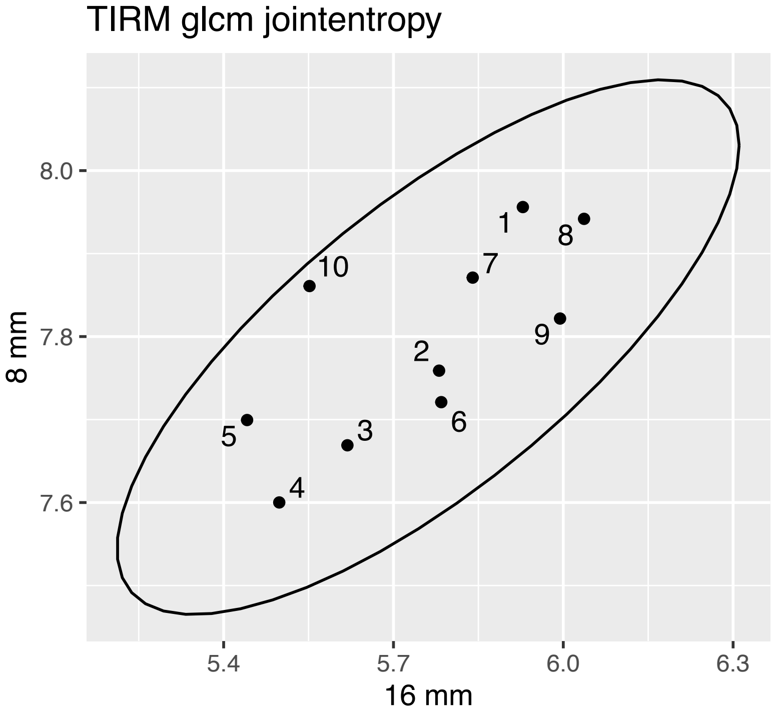
TIRM glcm jointentropy
8 mm
16 mm

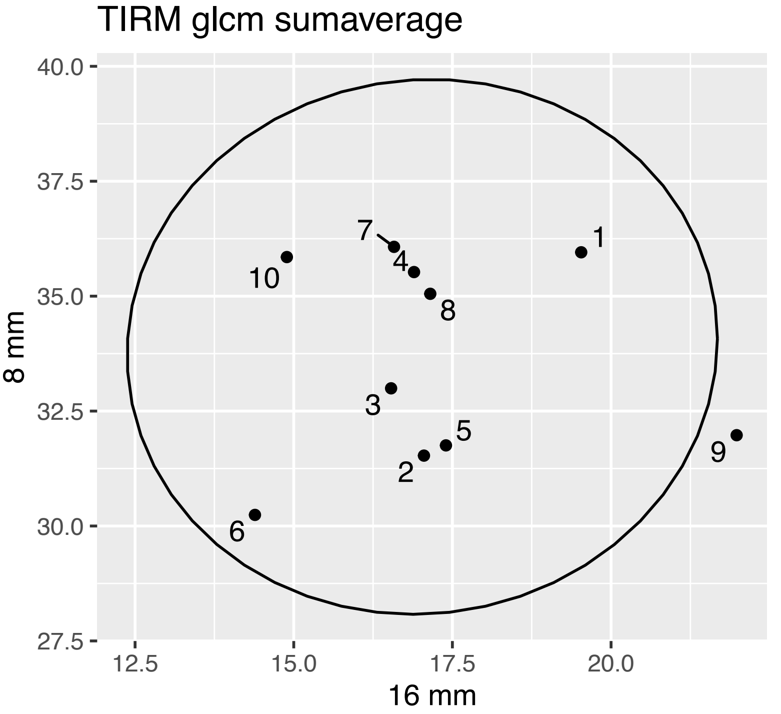
TIRM glcm sumaverage
8 mm
16 mm

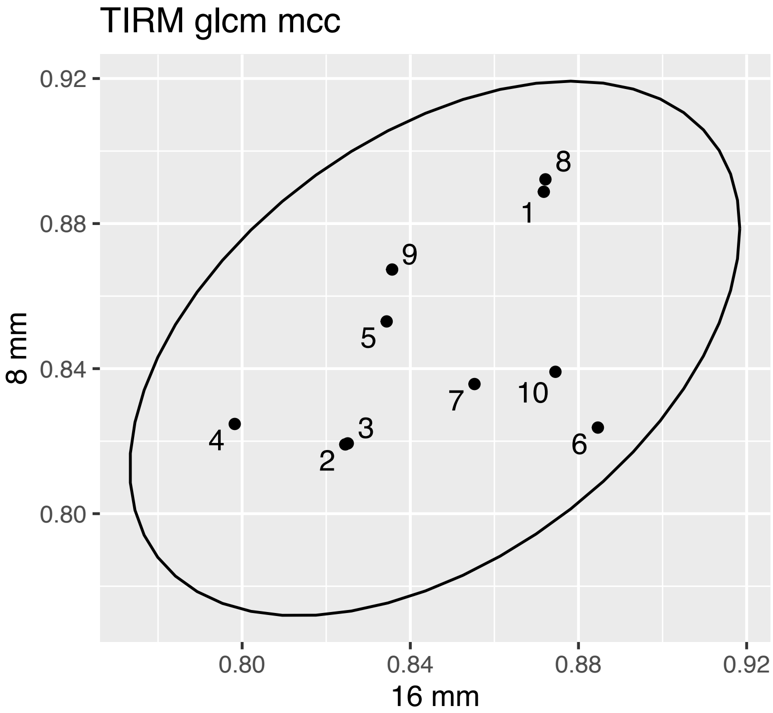
TIRM glcm mcc
8 mm
16 mm

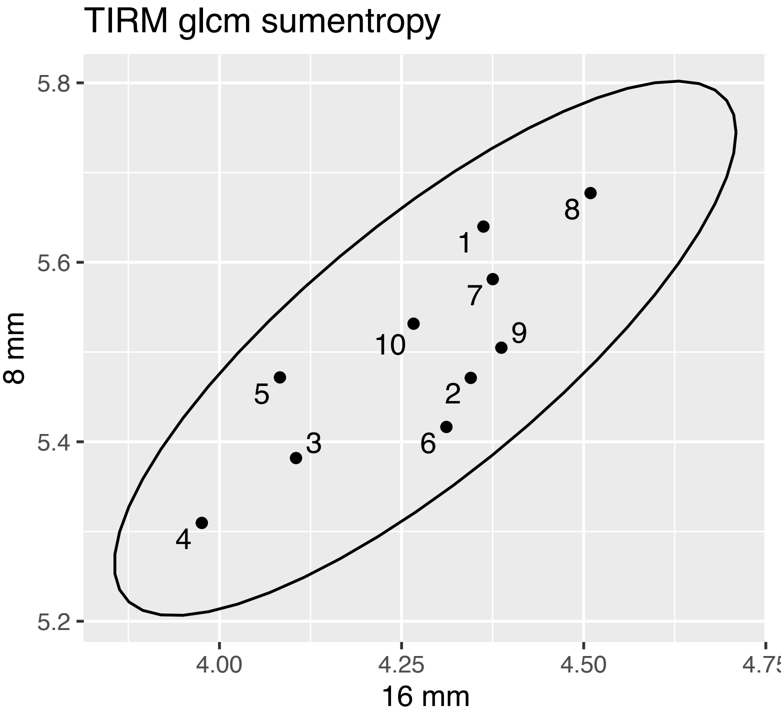
TIRM glcm sumentropy
8 mm
16 mm

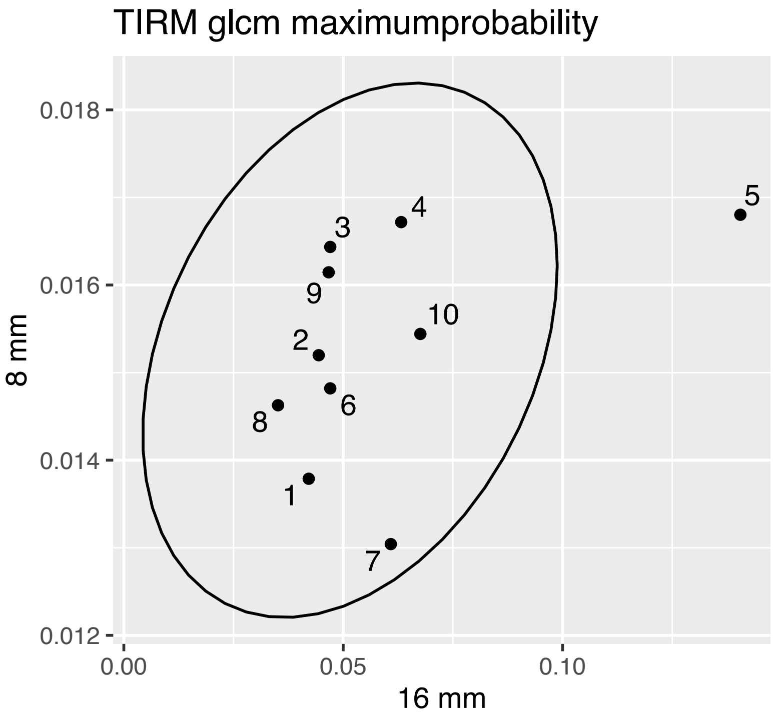
TIRM glcm maximumprobability
8 mm
16 mm

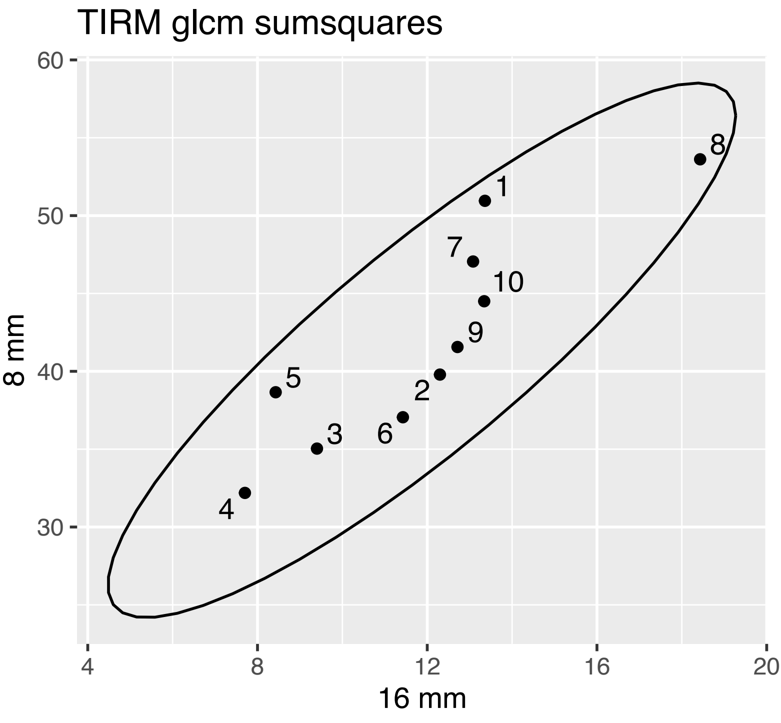
TIRM glcm sumsquares
8 mm
16 mm

TIRM glrlm graylevelnonuniformity

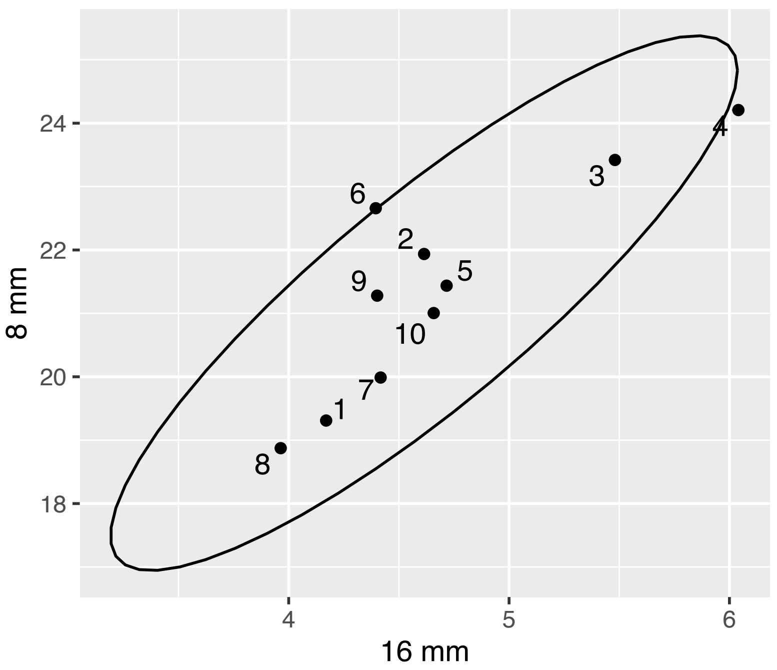

TIRM glrlm highgraylevelrunemphasis

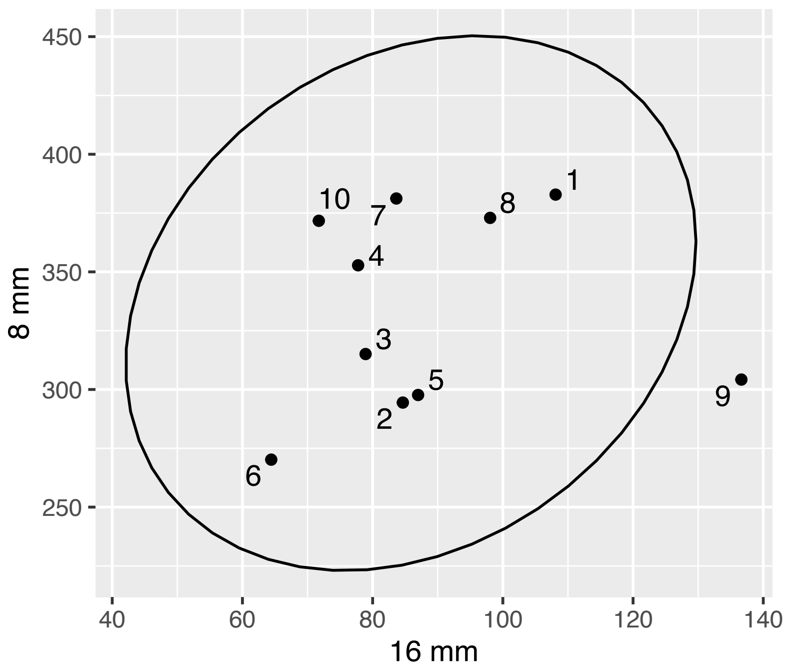

TIRM glrlm graylevelnonuniformitynormalize

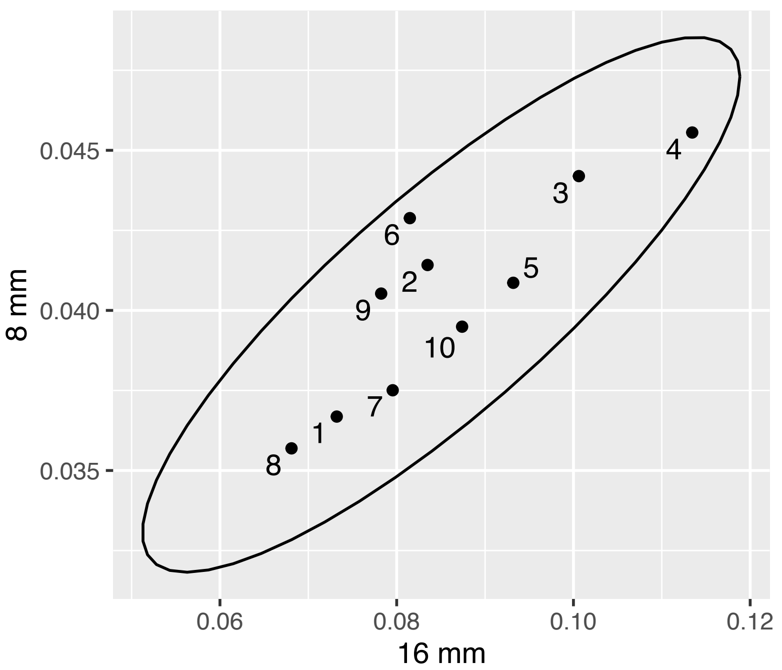

TIRM glrlm longrunemphasis

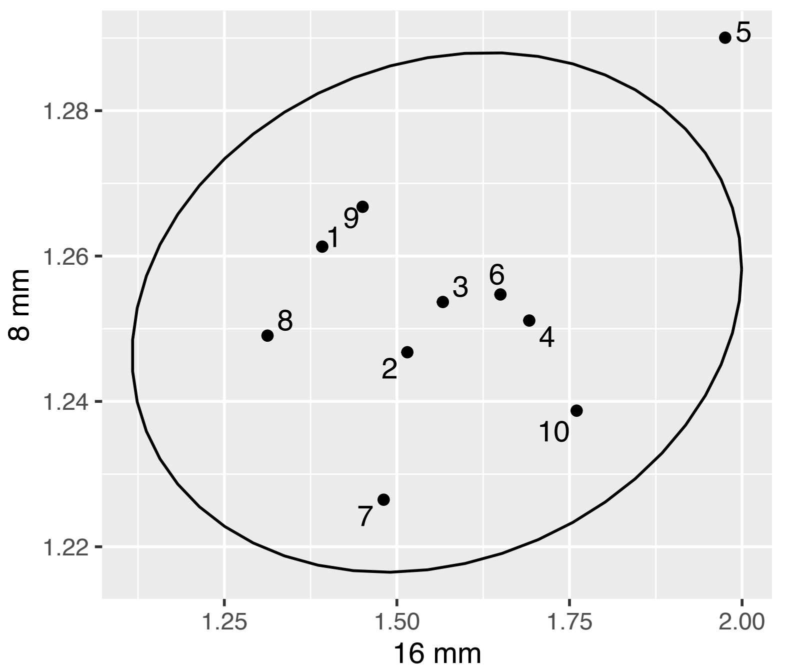

TIRM glrlm graylevelvariance

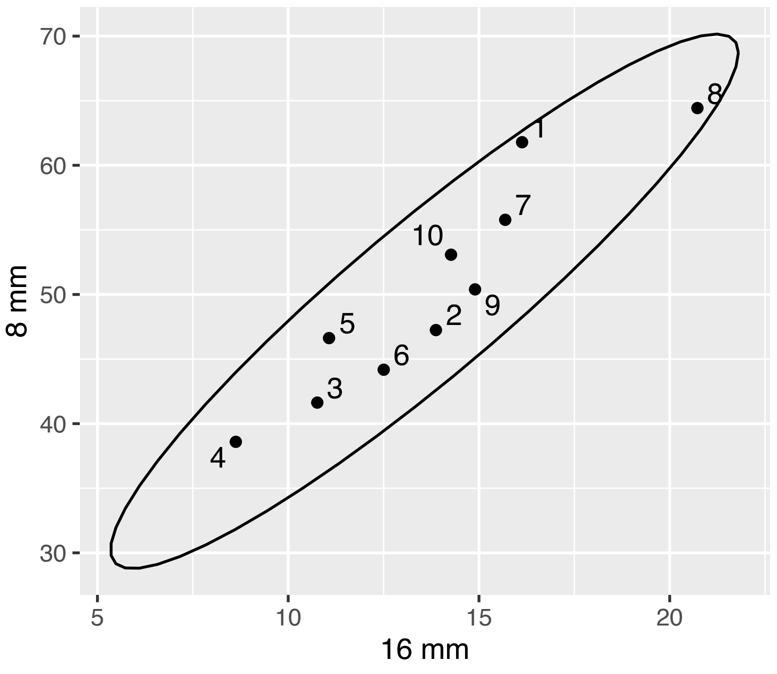

TIRM glrlm longrunhighgraylevelemphasis

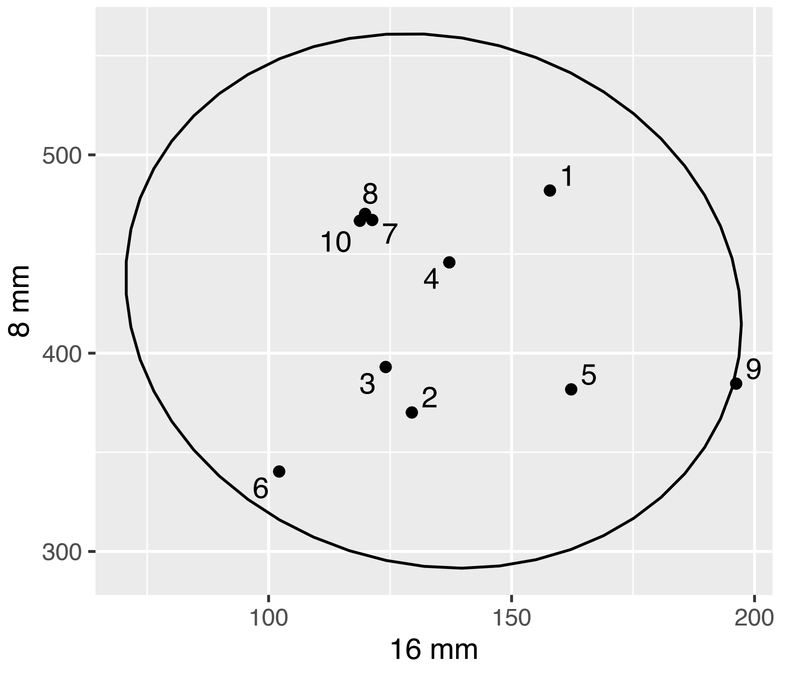

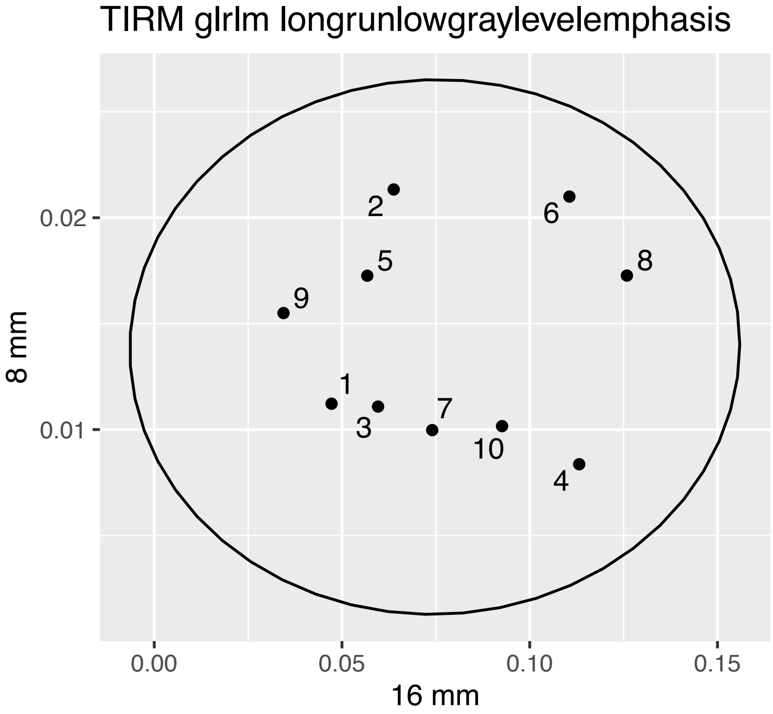
TIRM glrlm longrunlowgraylevelemphasis
8 mm
16 mm
0.02
0.01
0.00
0.05
0.10
0.15
1
2
3
4
5
6
7
8
9
10

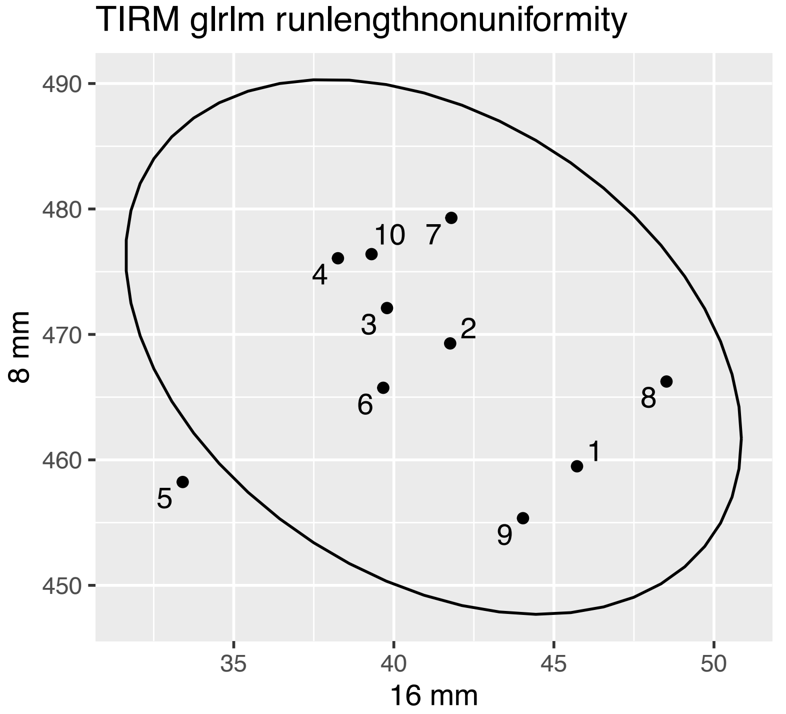
TIRM glrlm runlengthnonuniformity
8 mm
16 mm
490
480
470
460
450
35
40
45
50
1
2
3
4
5
6
7
8
9
10

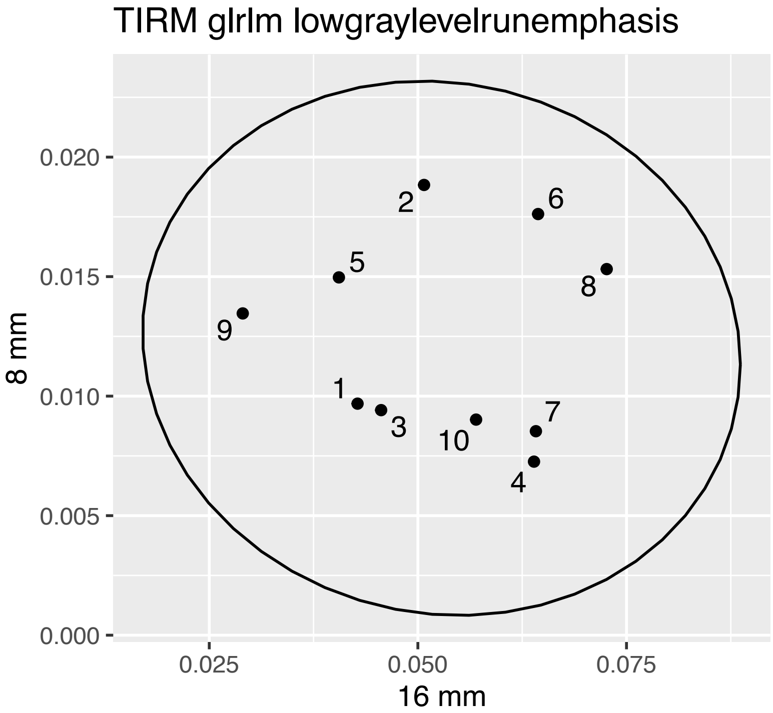
TIRM glrlm lowgraylevelrunemphasis
8 mm
16 mm
0.020
0.015
0.010
0.005
0.000
0.025
0.050
0.075
1
2
3
4
5
6
7
8
9
10

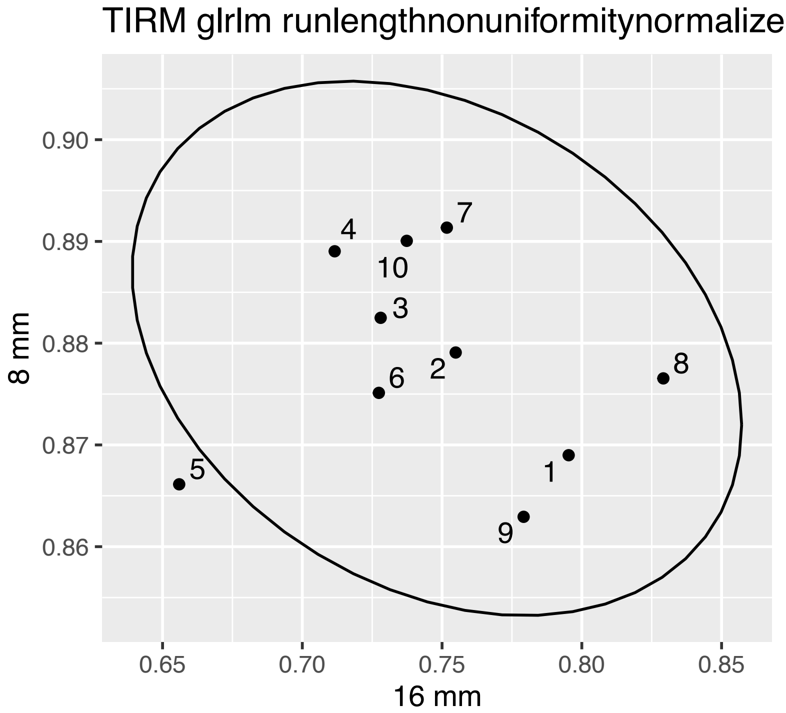
TIRM glrlm runlengthnonuniformitynormalize
8 mm
16 mm
0.90
0.89
0.88
0.87
0.86
0.65
0.70
0.75
0.80
0.85
1
2
3
4
5
6
7
8
9
10

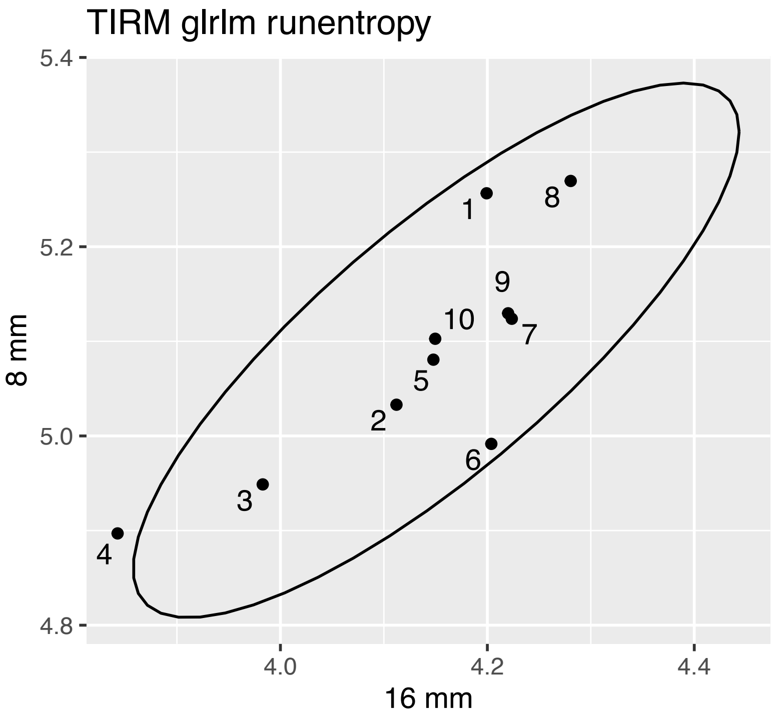
TIRM glrlm runentropy
8 mm
16 mm
5.4
5.2
5.0
4.8
4.0
4.2
4.4
1
2
3
4
5
6
7
8
9
10

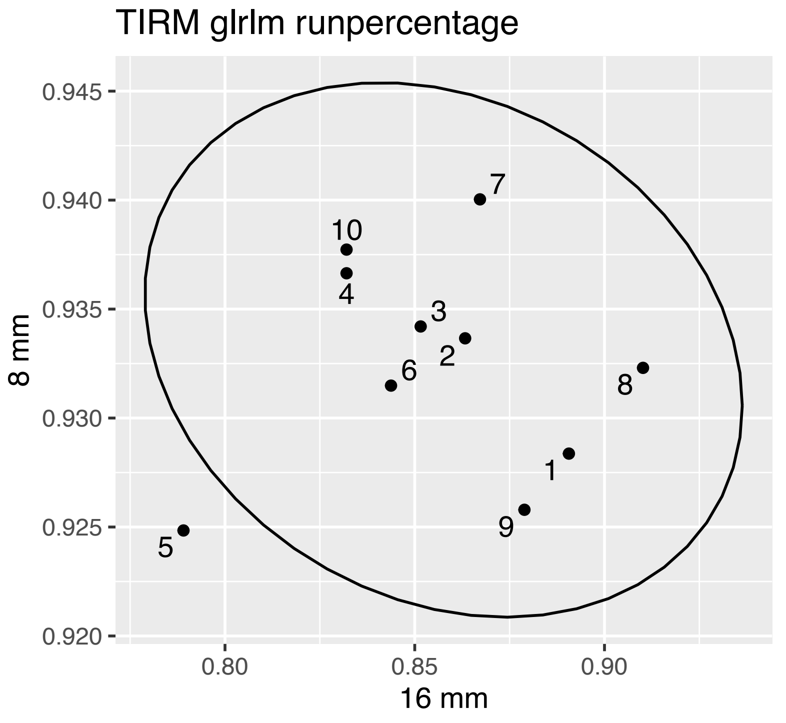
TIRM glrlm runpercentage
8 mm
16 mm
0.945
0.940
0.935
0.930
0.925
0.920
0.80
0.85
0.90
1
2
3
4
5
6
7
8
9
10

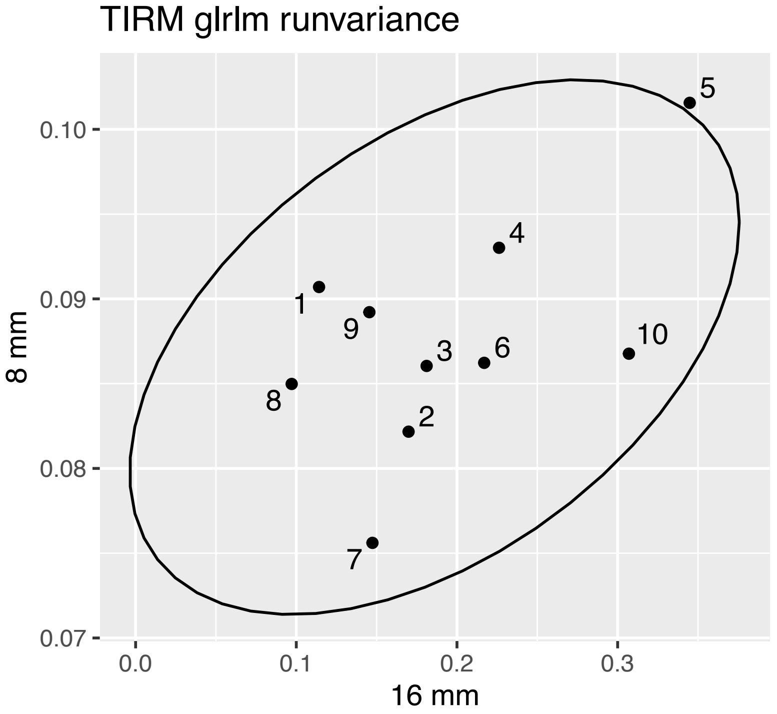
TIRM glrlm runvariance
8 mm
16 mm
0.10
0.09
0.08
0.07
0.0
0.1
0.2
0.3

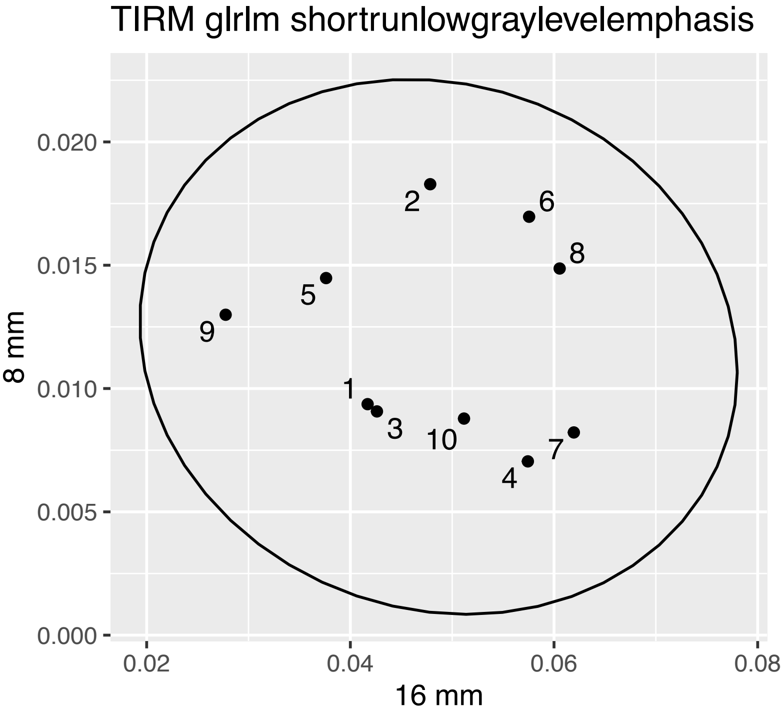
TIRM glrlm shortrunlowgraylevelemphasis
8 mm
16 mm
0.020
0.015
0.010
0.005
0.000
0.02
0.04
0.06
0.08

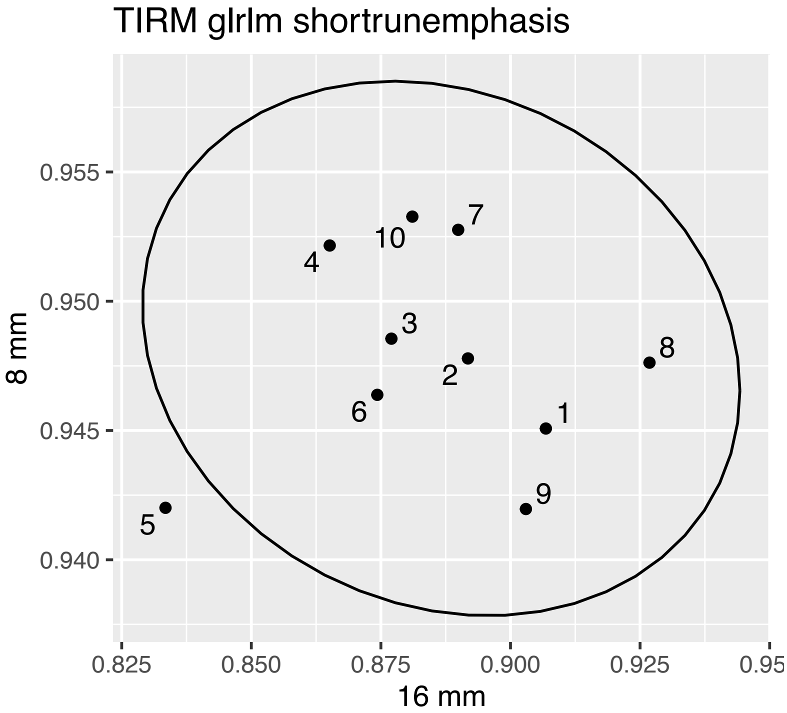
TIRM glrlm shortrunemphasis
8 mm
16 mm
0.955
0.950
0.945
0.940
0.825
0.850
0.875
0.900
0.925
0.95

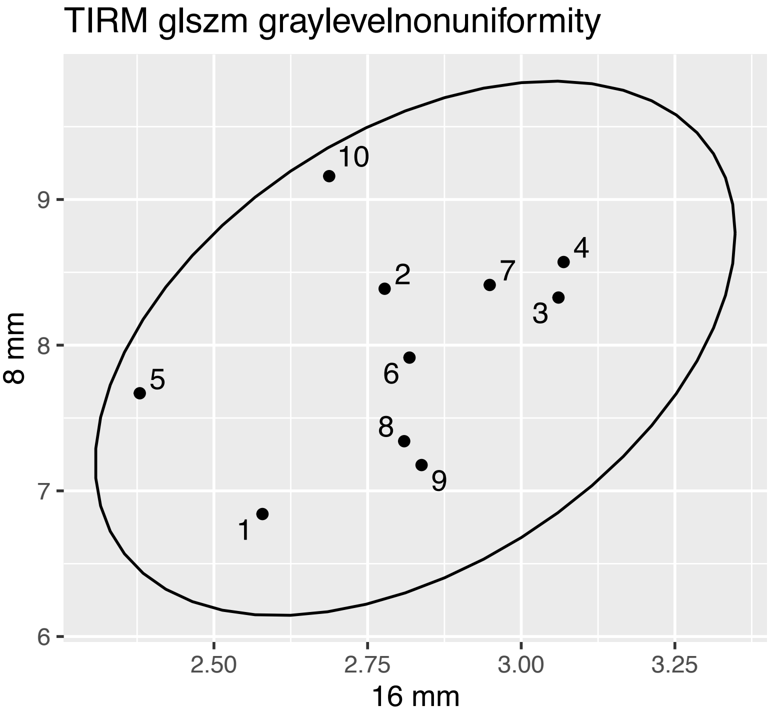
TIRM glszm graylevelnonuniformity
8 mm
16 mm
9
8
7
6
2.50
2.75
3.00
3.25

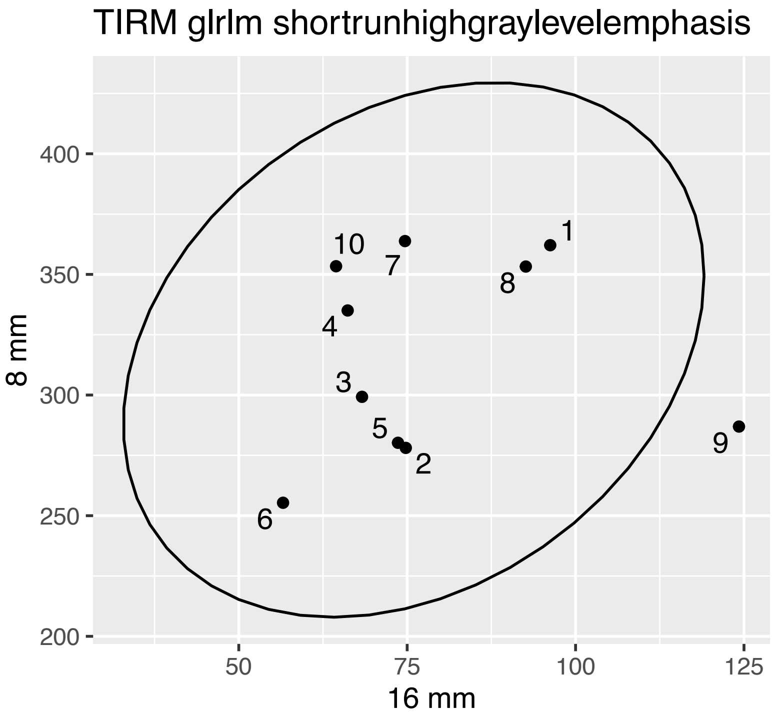
TIRM glrlm shortrunhighgraylevelemphasis
8 mm
16 mm
400
350
300
250
200
50
75
100
125

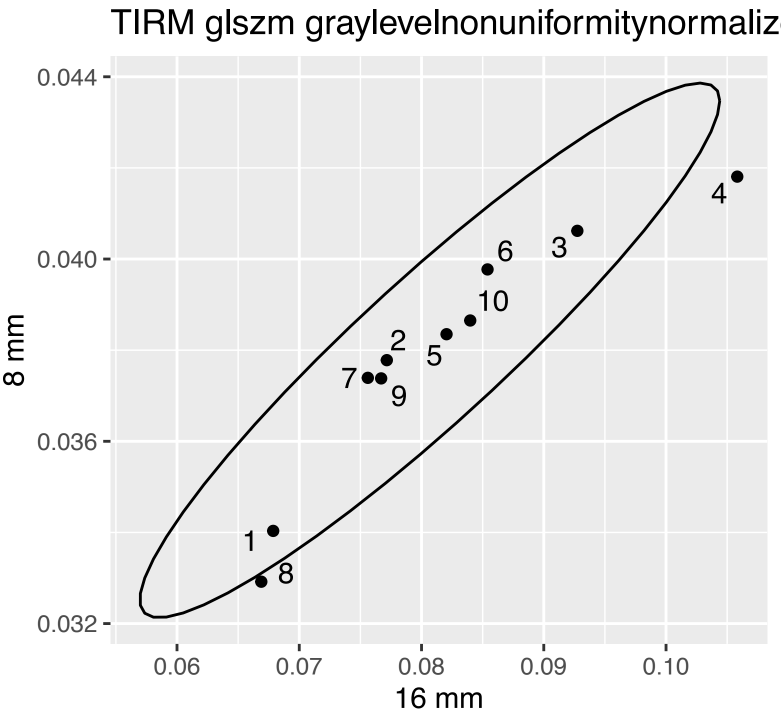
TIRM glszm graylevelnonuniformitynormaliz
8 mm
16 mm
0.044
0.040
0.036
0.032
0.06
0.07
0.08
0.09
0.10

TIRM glszm graylevelvariance

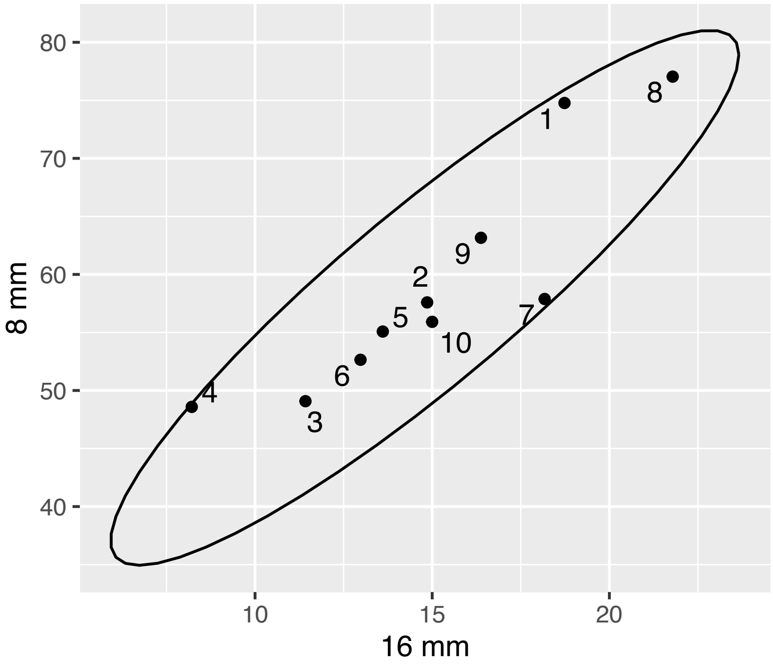

TIRM glszm largeareahighgraylevelemphasis

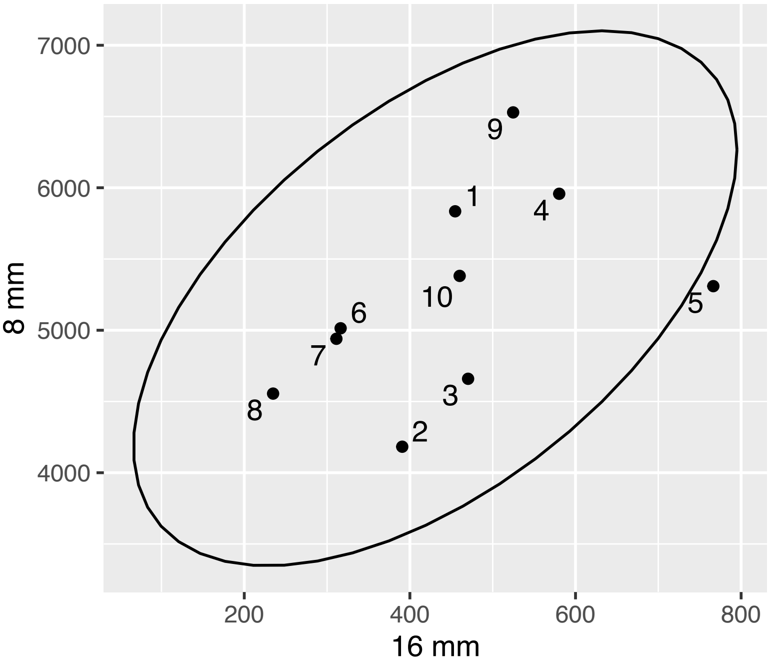

TIRM glszm highgraylevelzoneemphasis

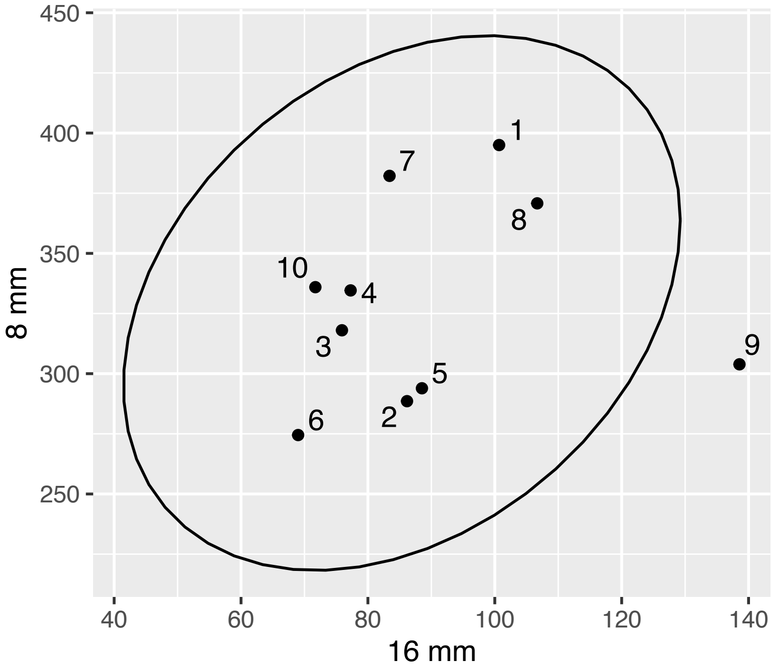

TIRM glszm largearealowgraylevelemphasis

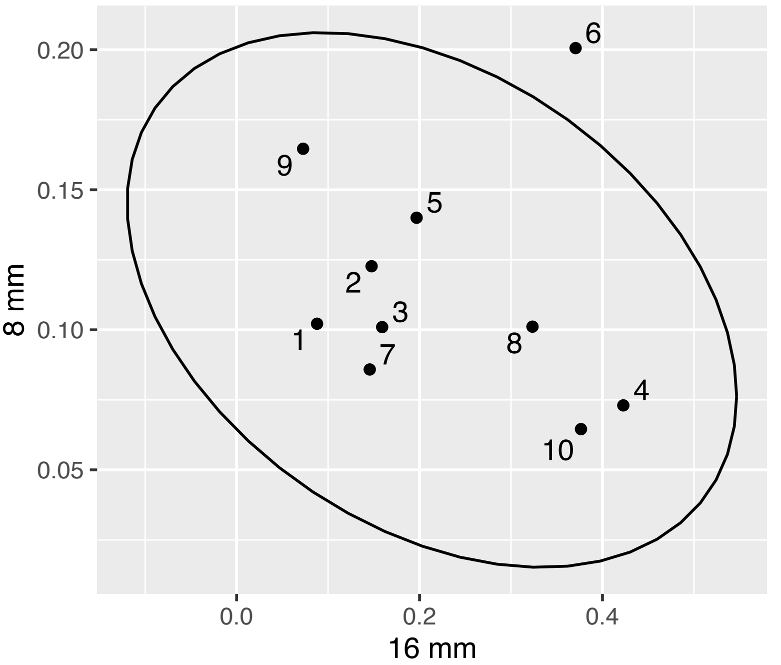

TIRM glszm largeareaemphasis

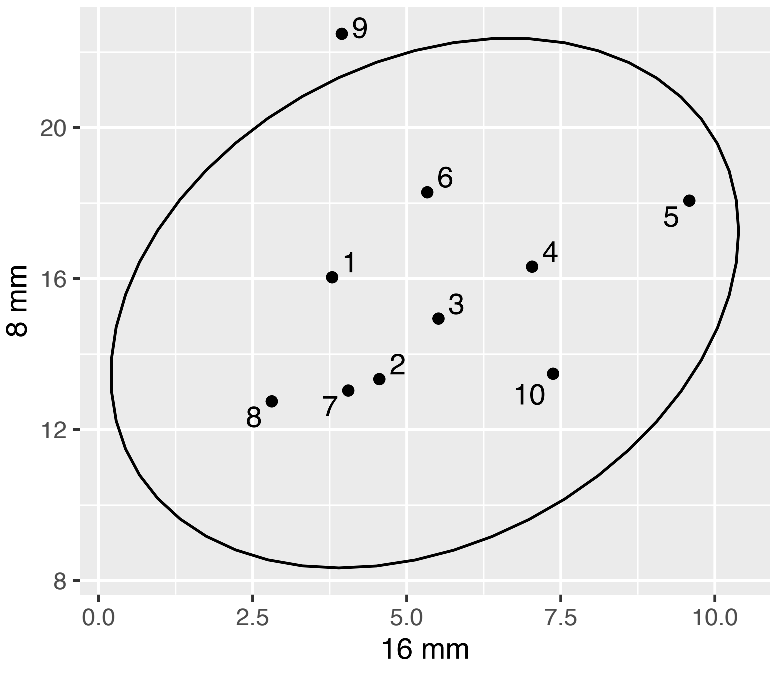

TIRM glszm lowgraylevelzoneemphasis

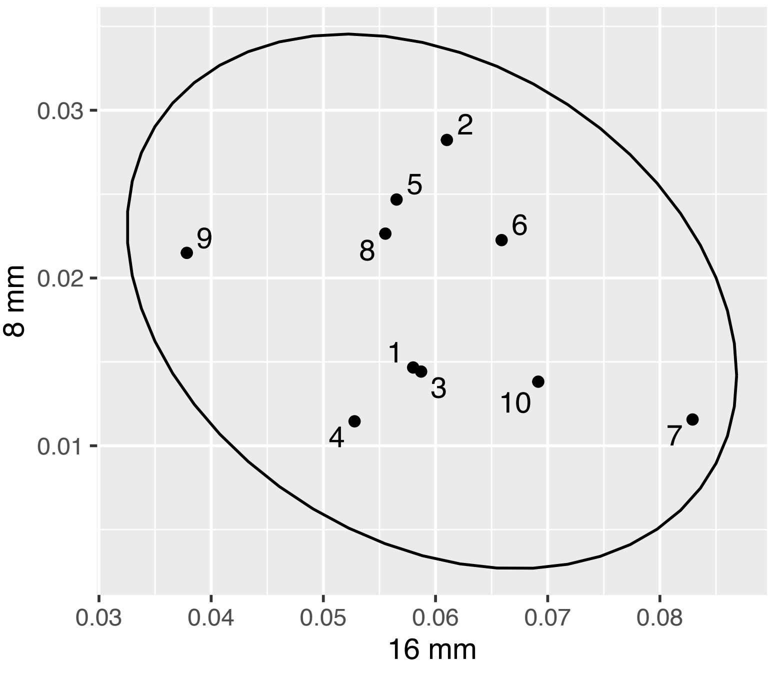

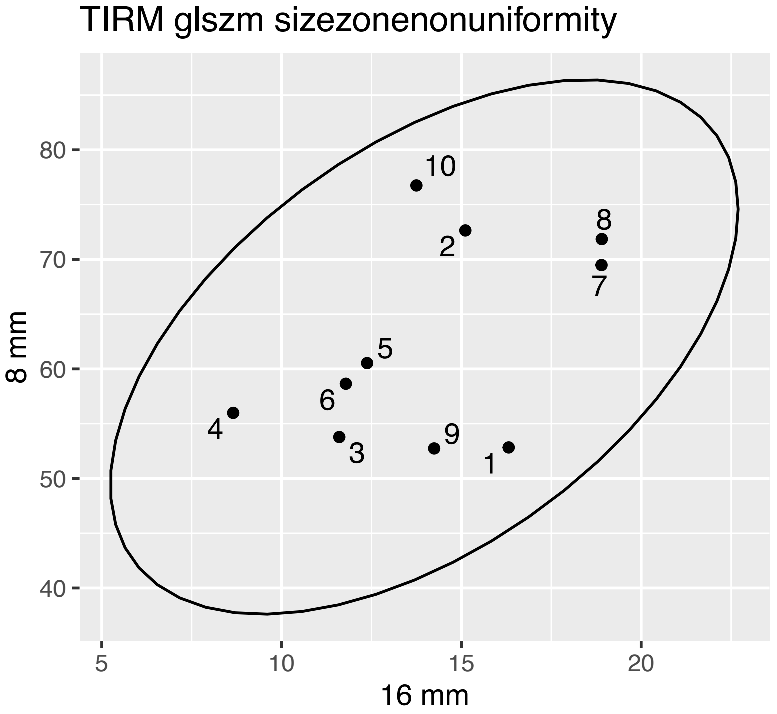
TIRM glszm sizezonenonuniformity
8 mm
16 mm

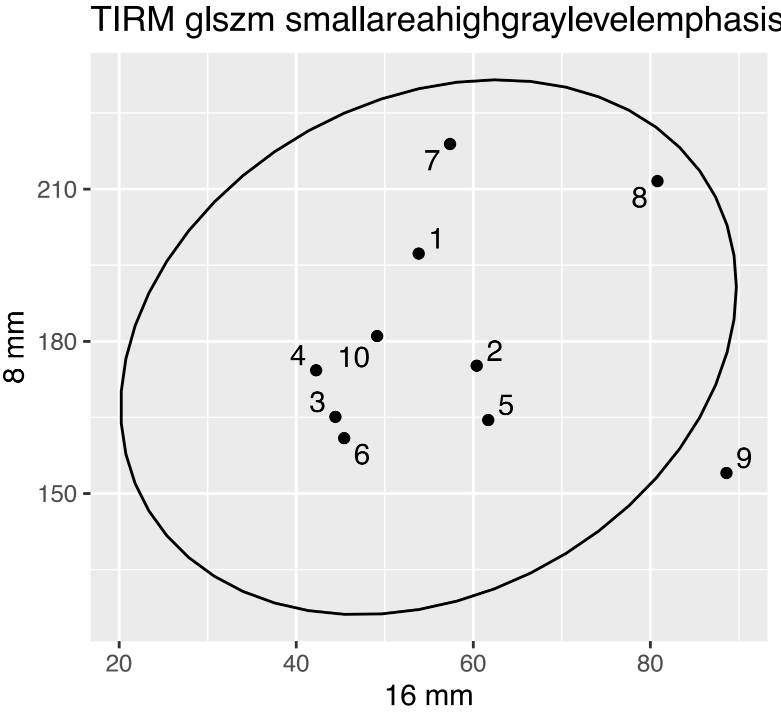
TIRM glszm smallareahighgraylevelemphasis
8 mm
16 mm

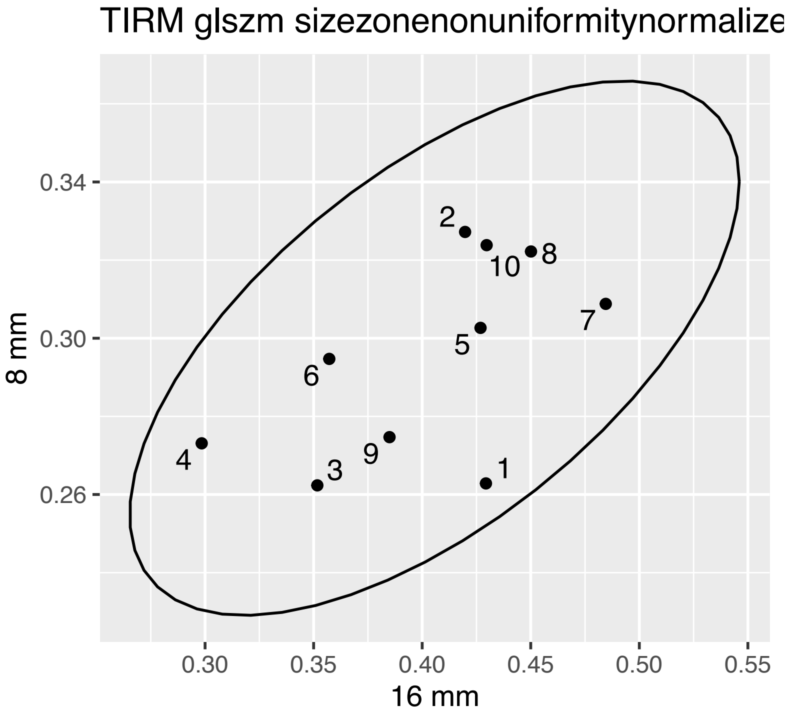
TIRM glszm sizezonenonuniformitynormalize
8 mm
16 mm

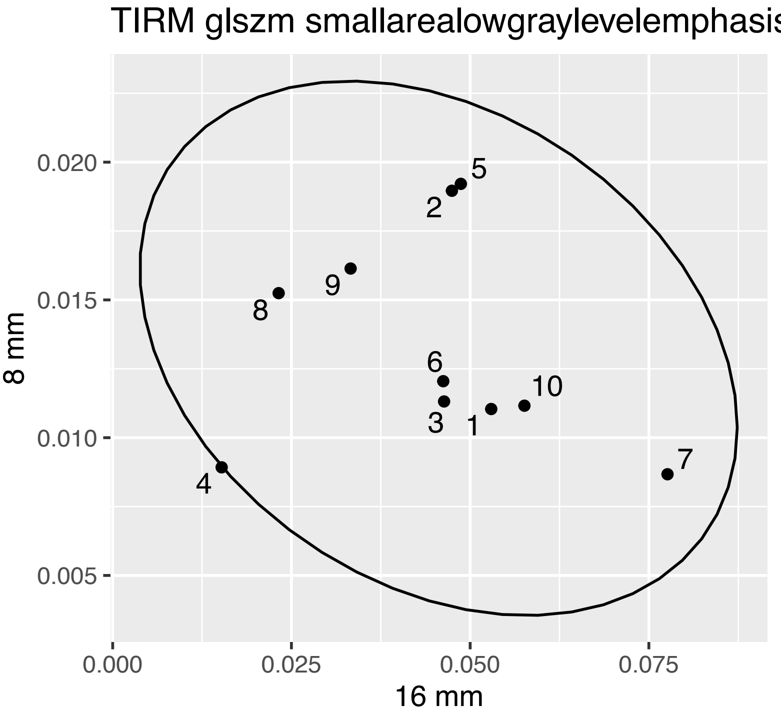
TIRM glszm smallarealowgraylevelemphasis
8 mm
16 mm

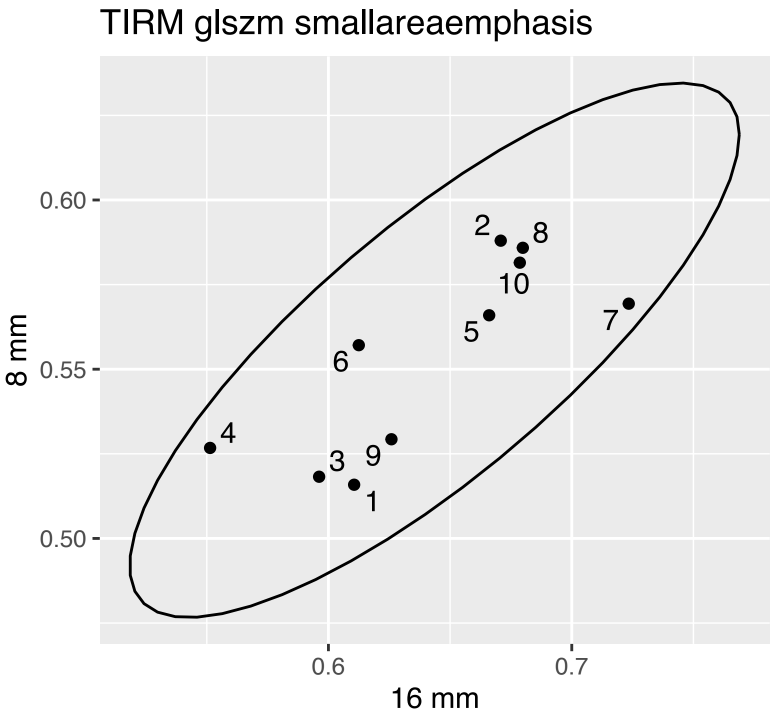
TIRM glszm smallareaemphasis
8 mm
16 mm

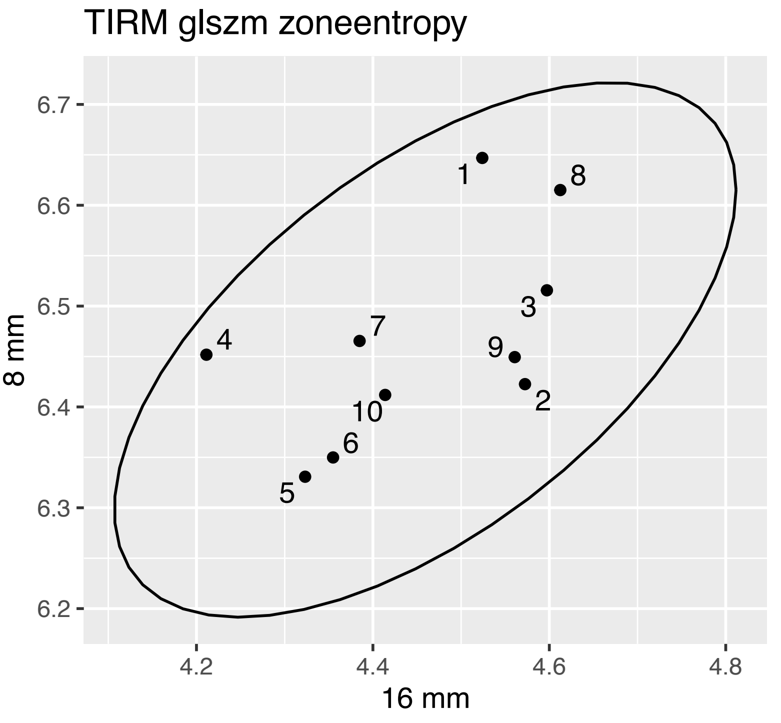
TIRM glszm zoneentropy
8 mm
16 mm

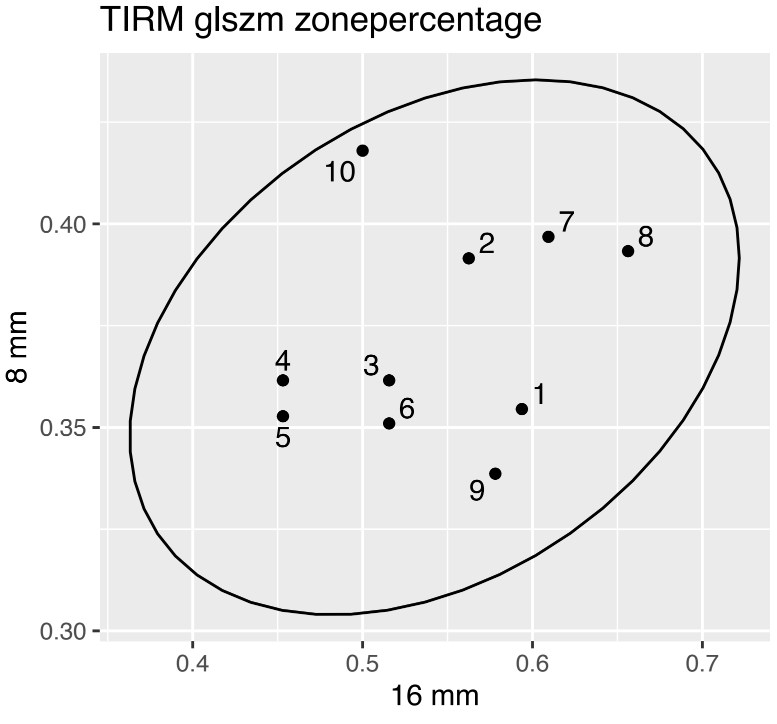
TIRM glszm zonepercentage
8 mm
16 mm

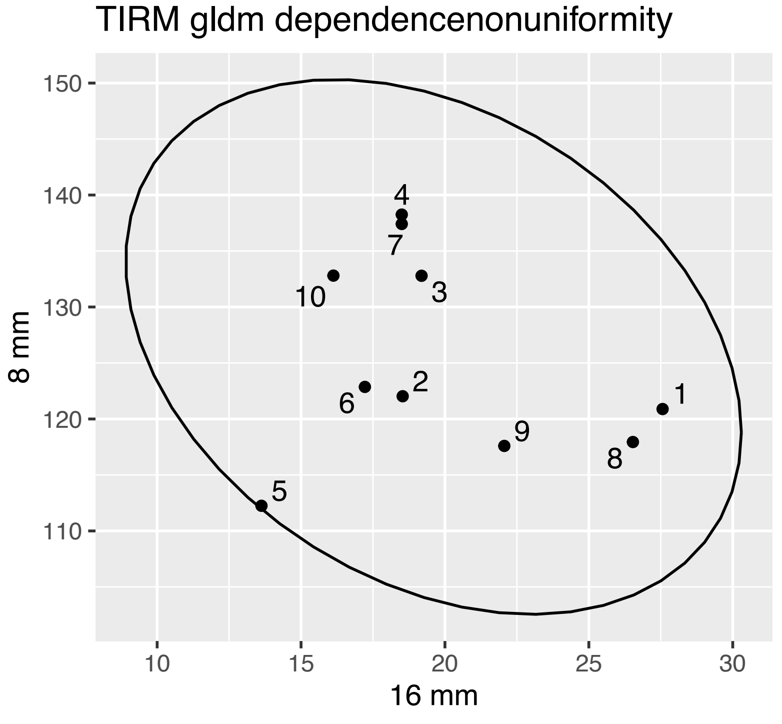
TIRM gldm dependencenonuniformity
8 mm
16 mm

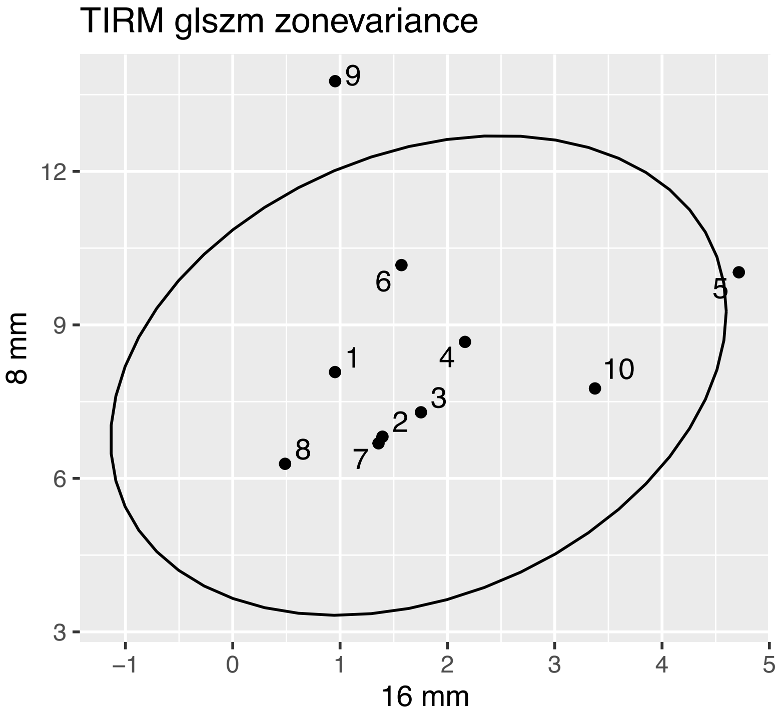
TIRM glszm zonevariance
8 mm
16 mm

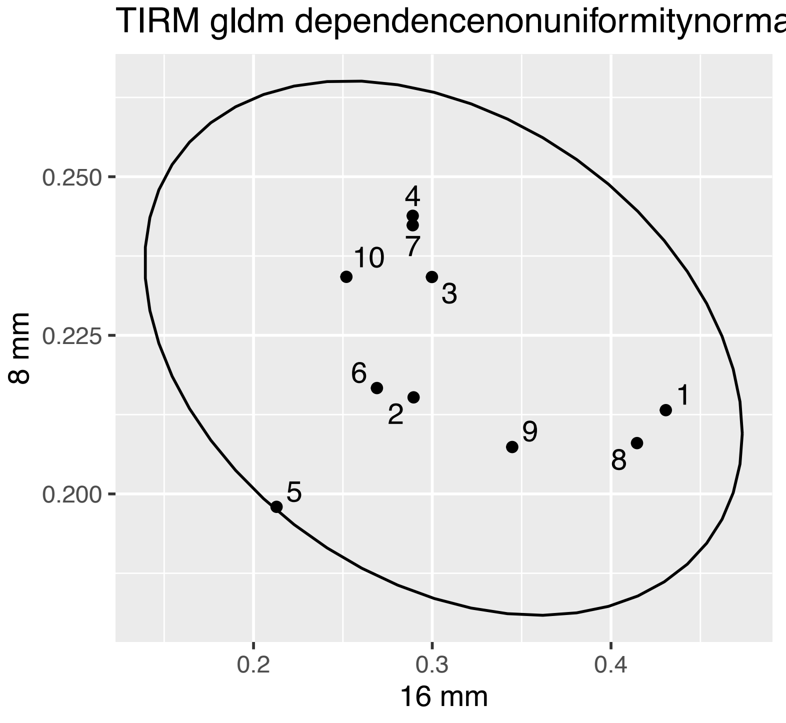
TIRM gldm dependencenonuniformitynorma
8 mm
16 mm

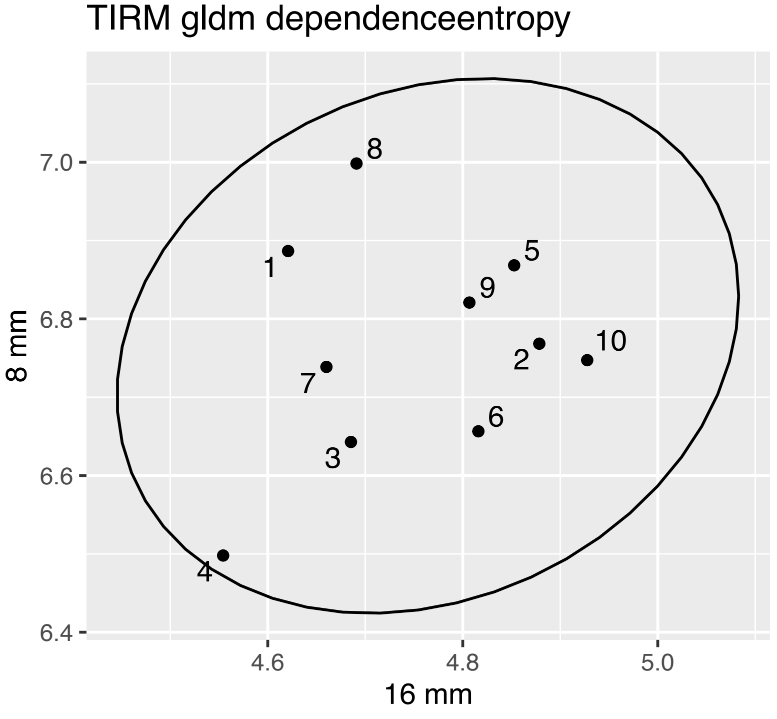
TIRM gldm dependenceentropy
8 mm
16 mm

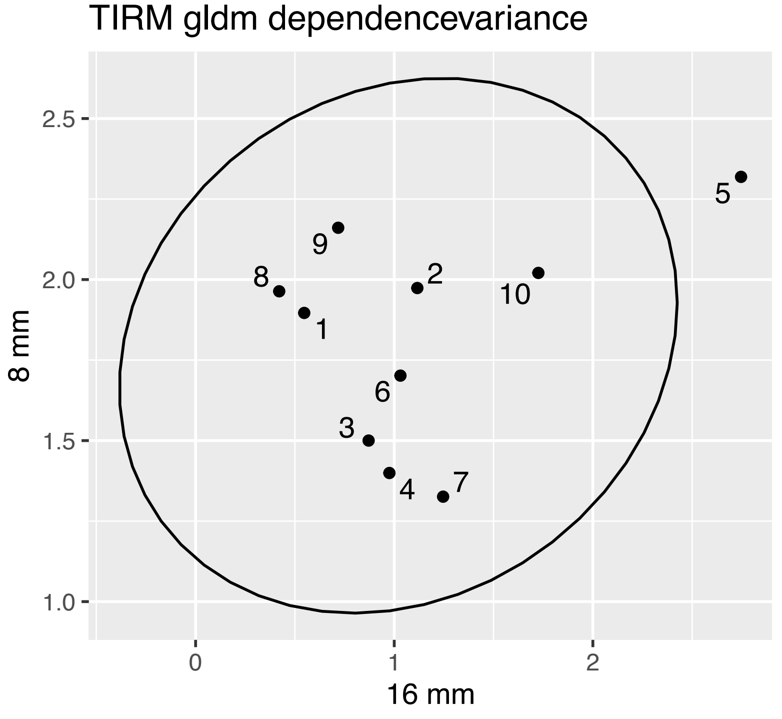
TIRM gldm dependencevariance
8 mm
16 mm

TIRM gldm graylevelnonuniformity

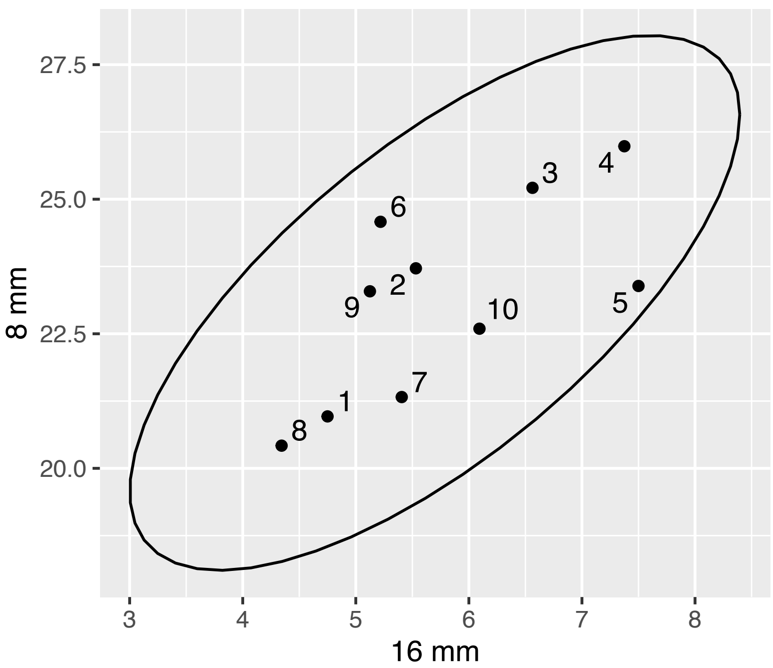

TIRM gldm largedependenceemphasis

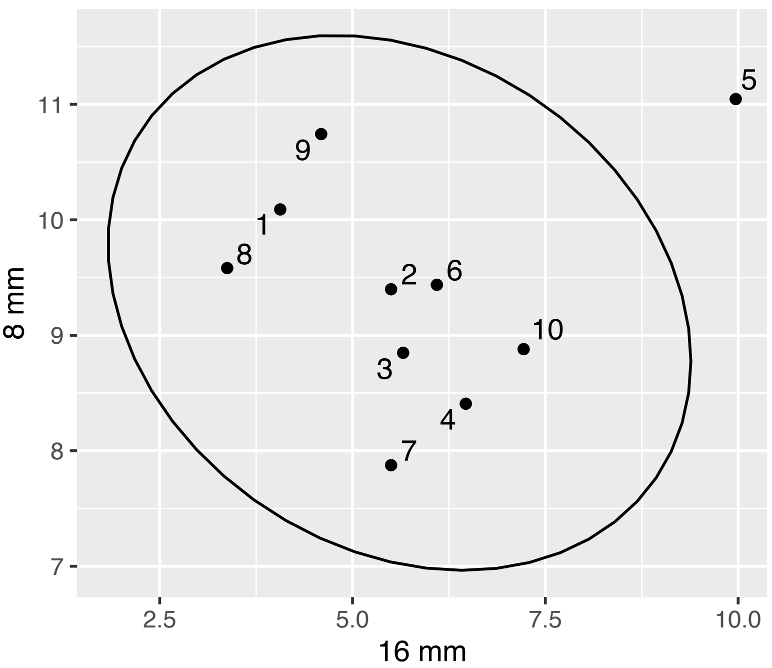

TIRM gldm graylevelvariance

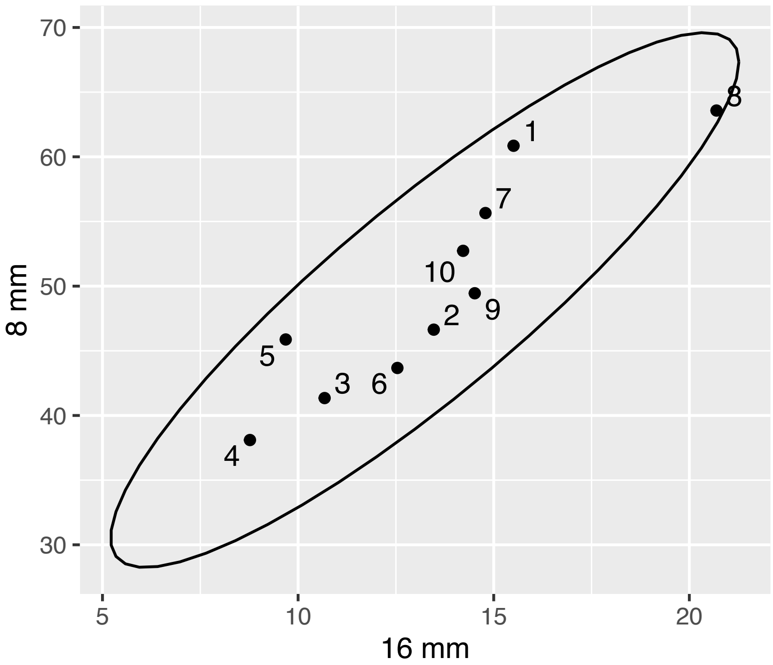

TIRM gldm largedependencehighgraylevelem

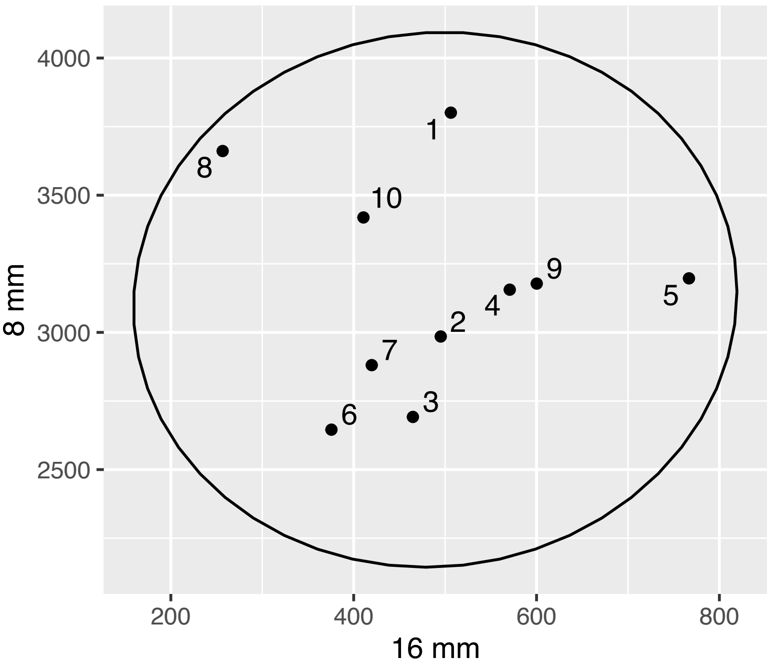

TIRM gldm highgraylevelemphasis

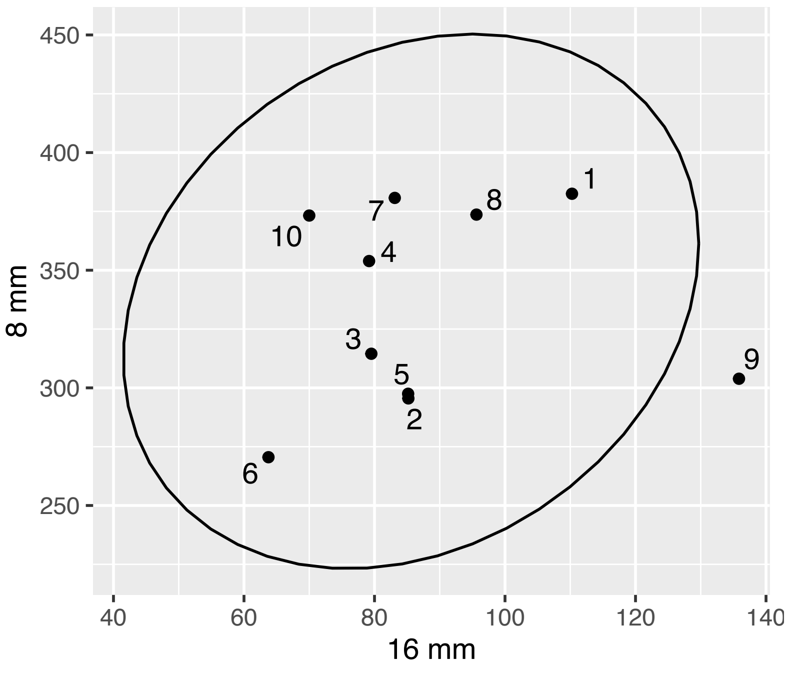

TIRM gldm largedependencelowgraylevelem

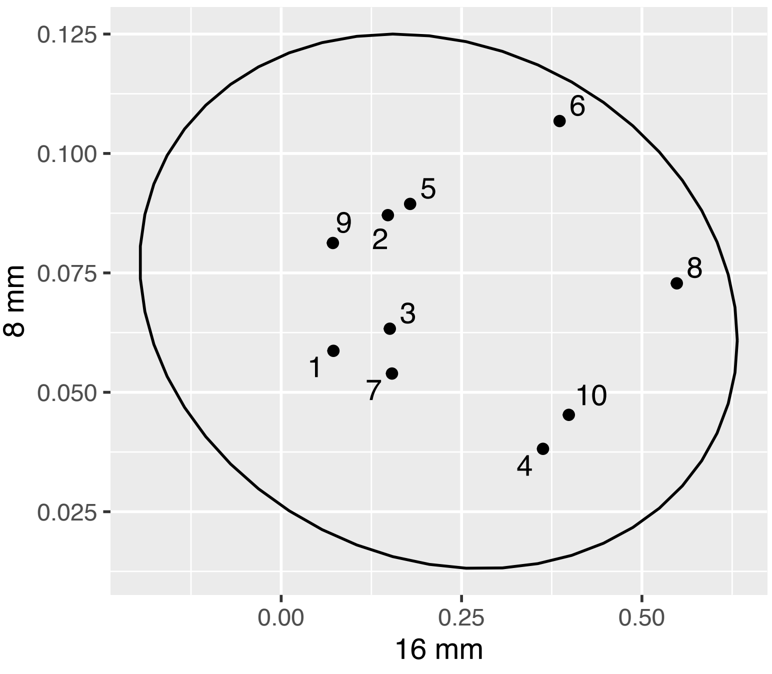

TIRM gldm lowgraylevelemphasis

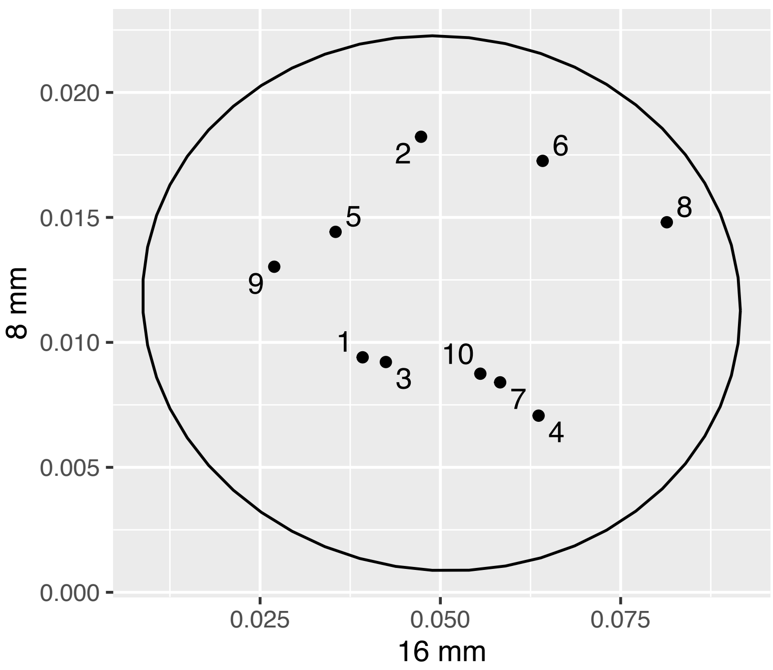

TIRM gldm smalldependencelowgraylevele

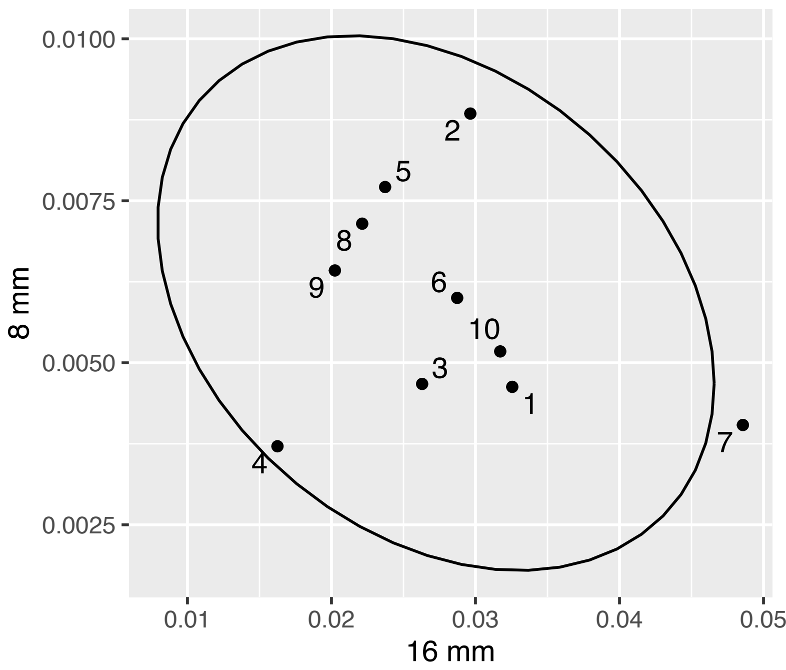

TIRM gldm smalldependenceemphasis

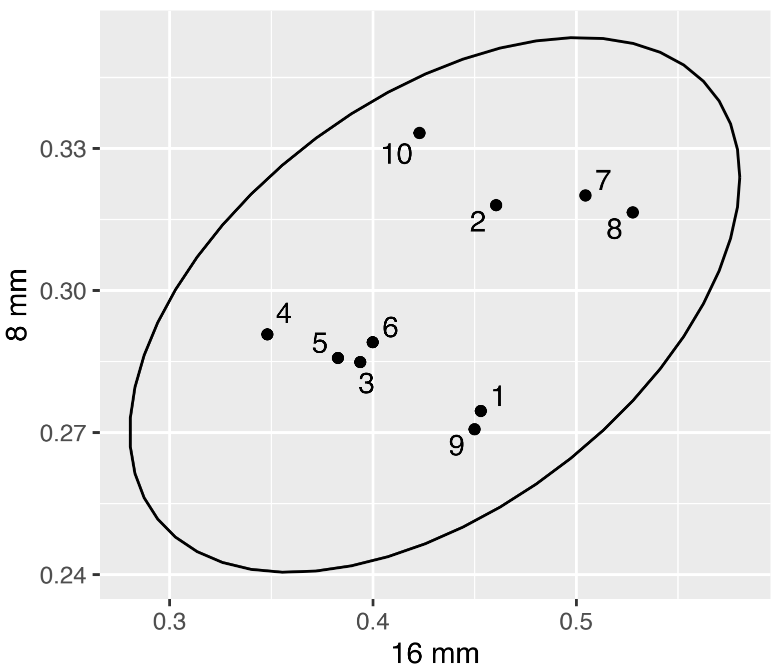

TIRM ngtdm busyness

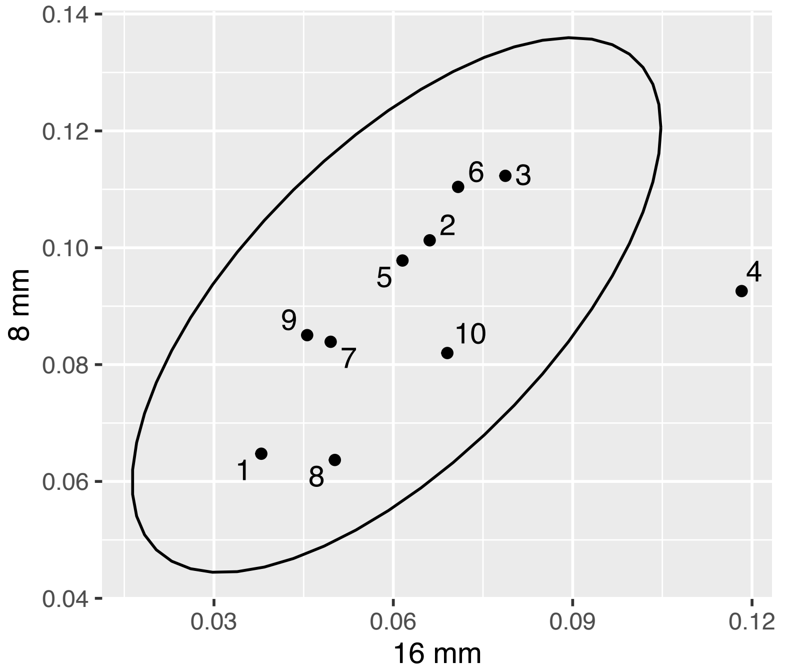

TIRM gldm smalldependencehighgraylevelem

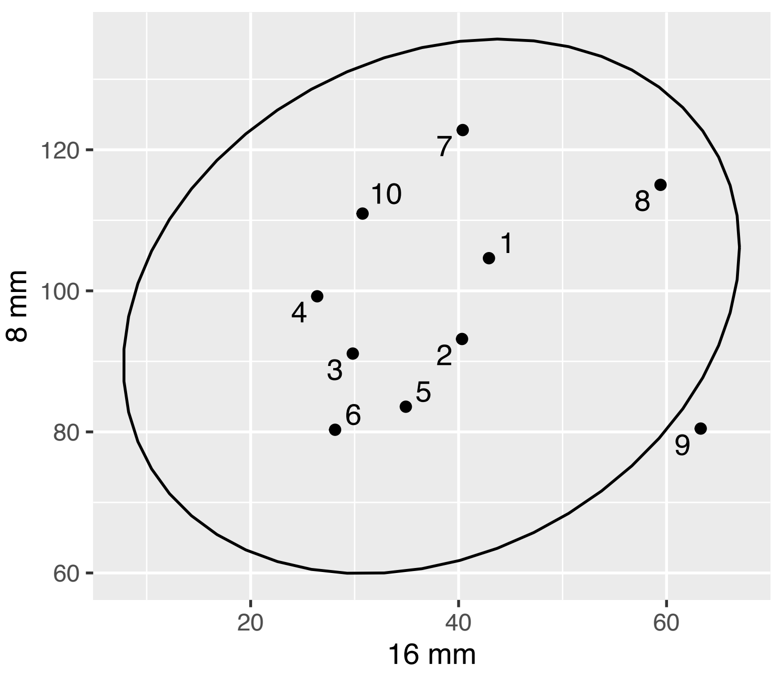

TIRM ngtdm coarseness

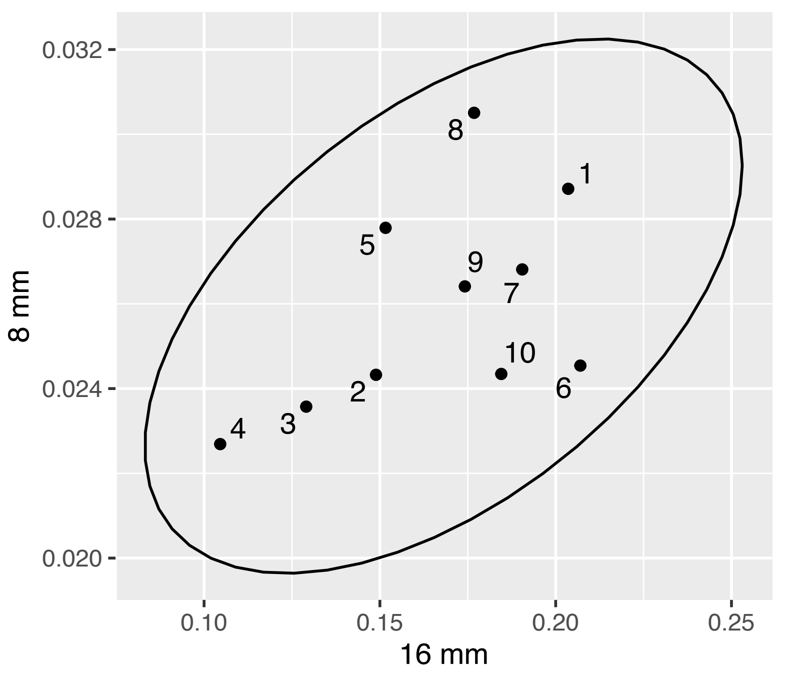

TIRM ngtdm complexity

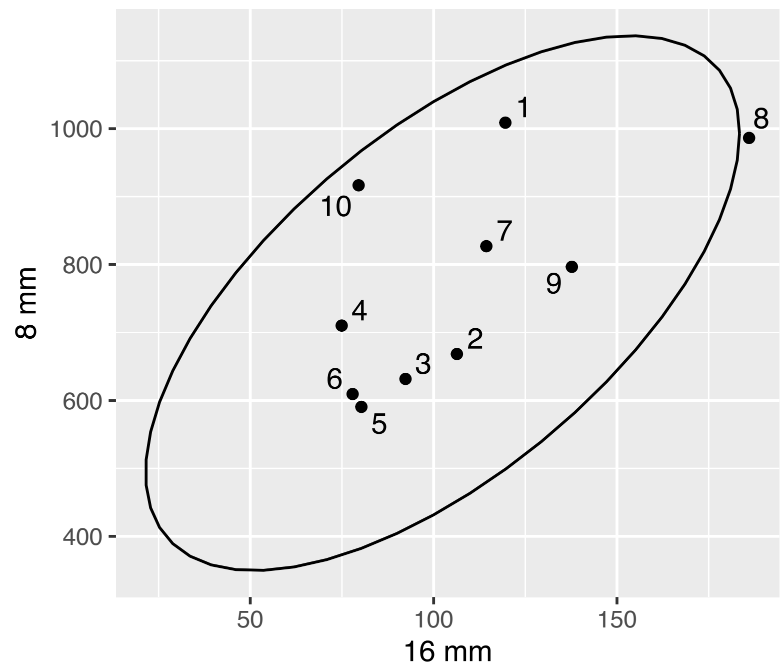

TIRM firstorder 10percentile

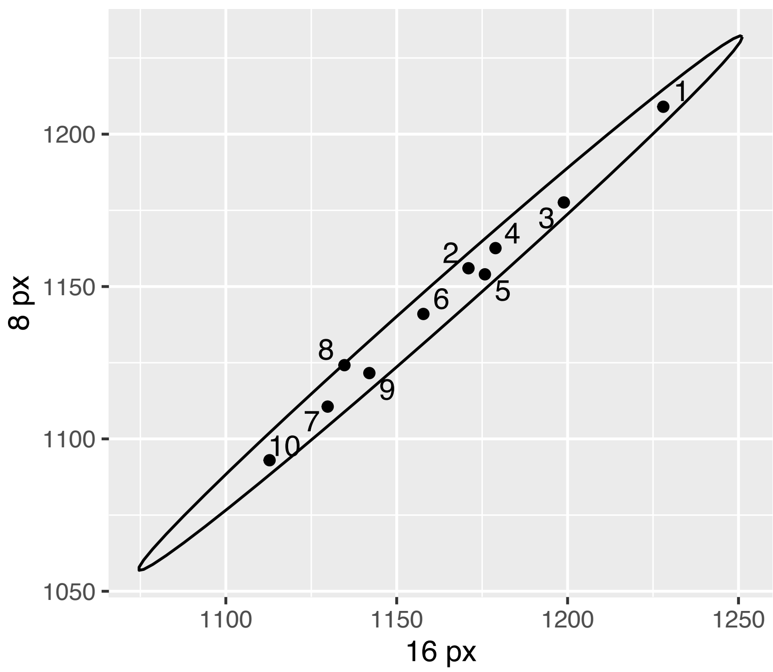

TIRM ngtdm contrast

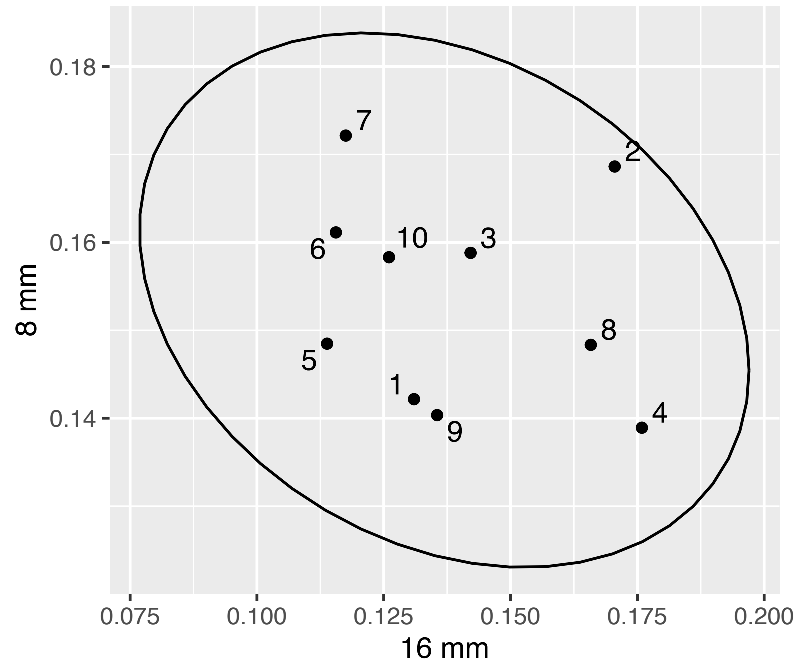

TIRM firstorder 90percentile

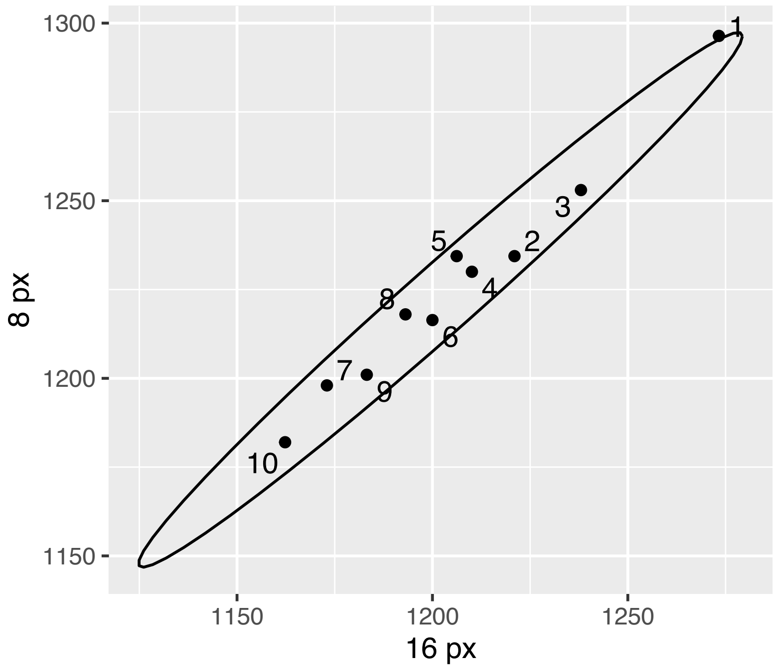

TIRM ngtdm strength

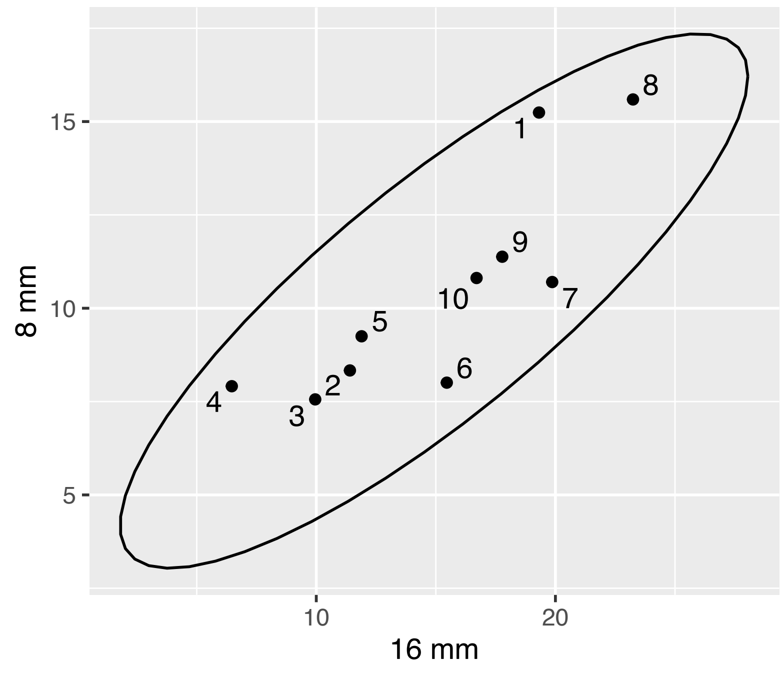

TIRM firstorder energy

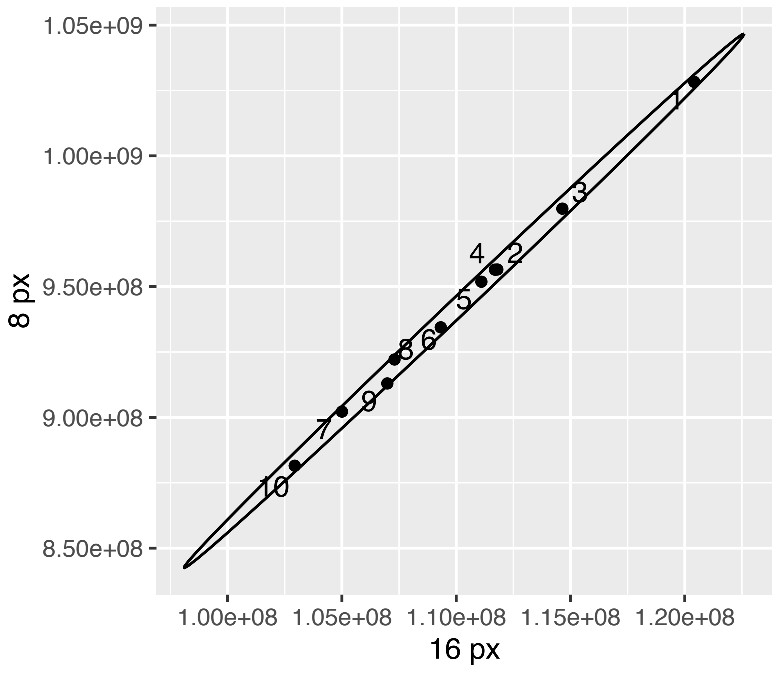

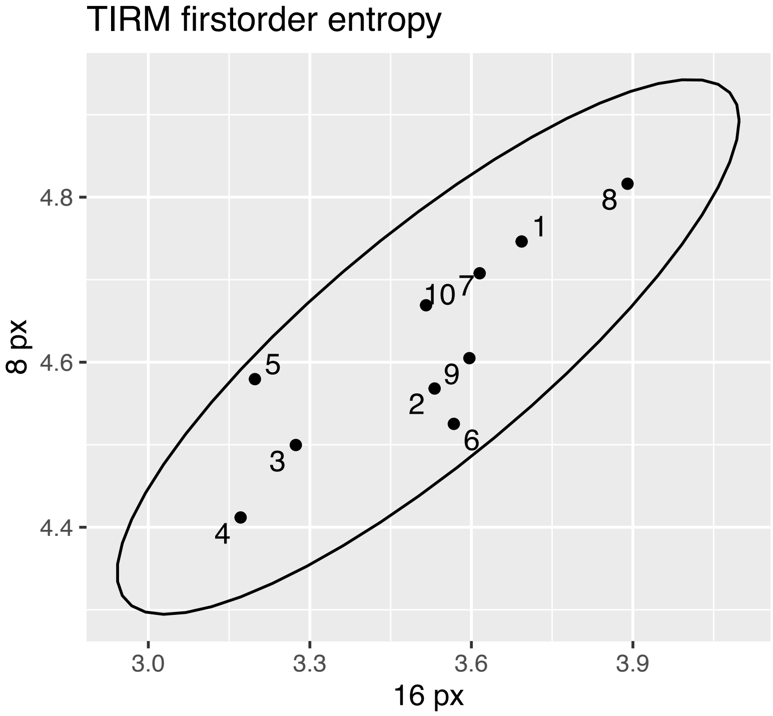
TIRM firstorder entropy
8 px
16 px
4.8
4.6
4.4
3.0
3.3
3.6
3.9
1
2
3
4
5
6
7
8
9
10

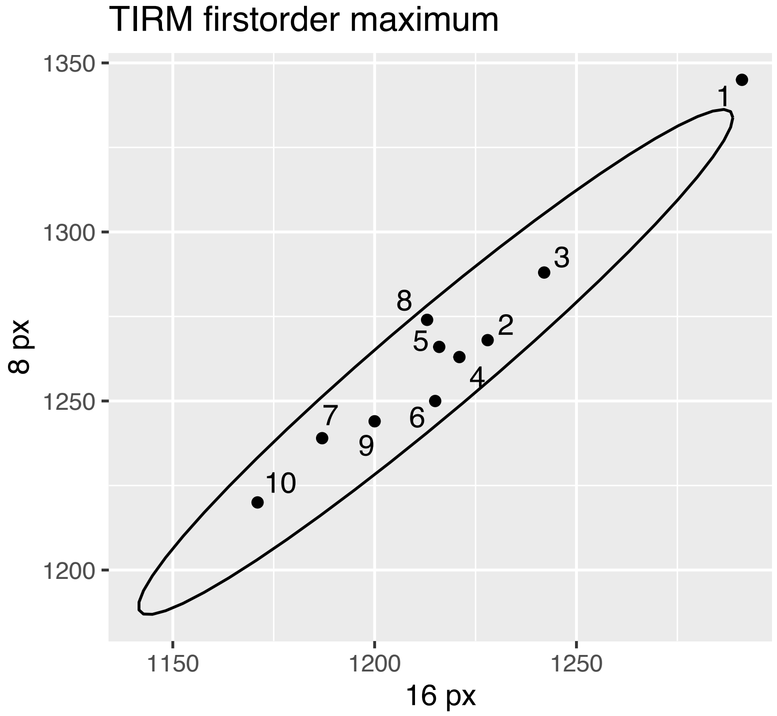
TIRM firstorder maximum
8 px
16 px
1350
1300
1250
1200
1150
1200
1250
1
2
3
4
5
6
7
8
9
10

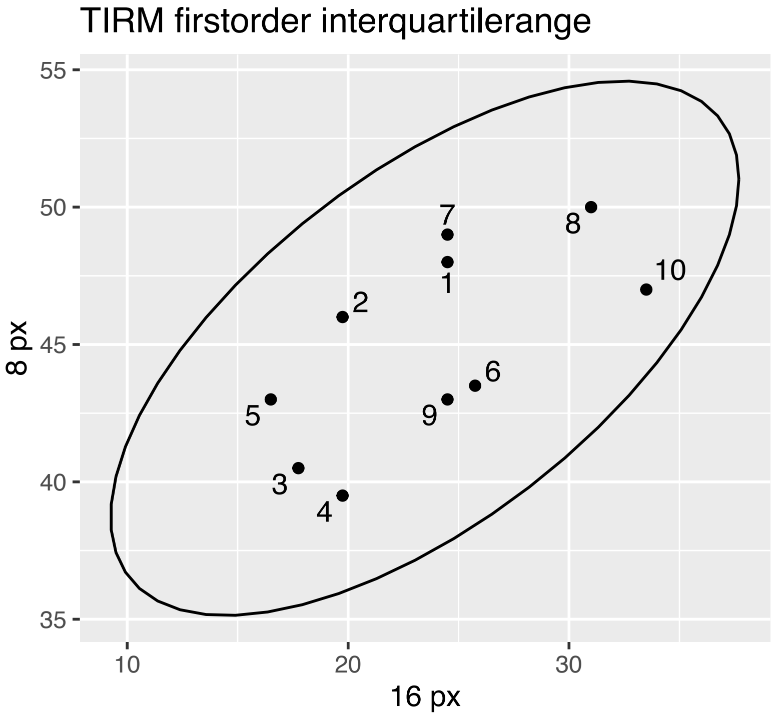
TIRM firstorder interquartilerange
8 px
16 px
55
50
45
40
35
10
20
30
1
2
3
4
5
6
7
8
9
10

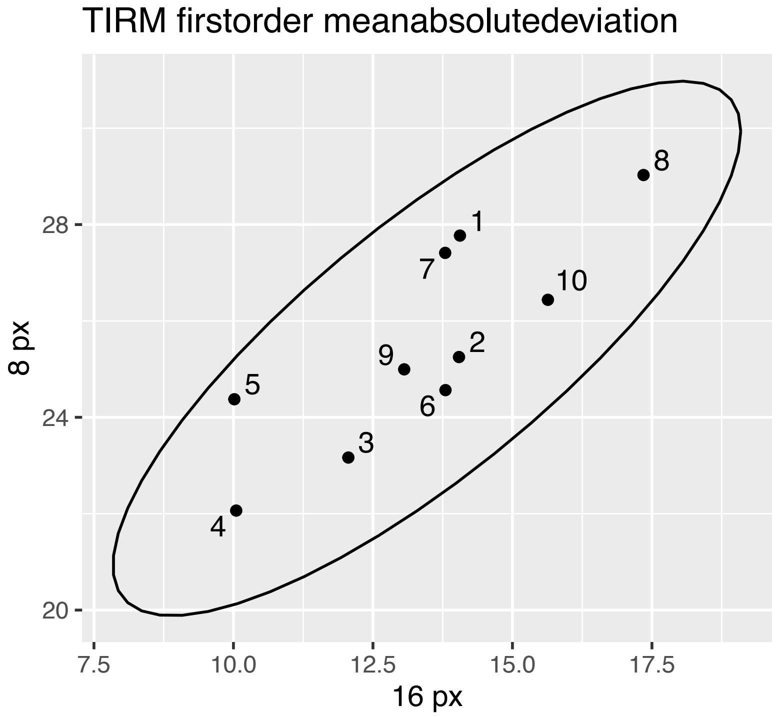
TIRM firstorder meanabsolutedeviation
8 px
16 px
28
24
20
7.5
10.0
12.5
15.0
17.5
1
2
3
4
5
6
7
8
9
10

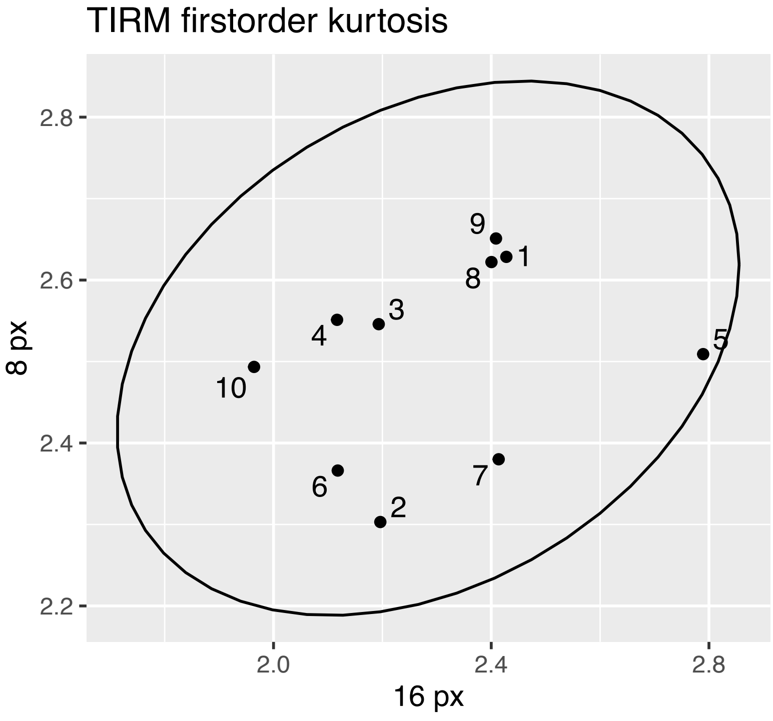
TIRM firstorder kurtosis
8 px
16 px
2.8
2.6
2.4
2.2
2.0
2.4
2.8
1
2
3
4
5
6
7
8
9
10

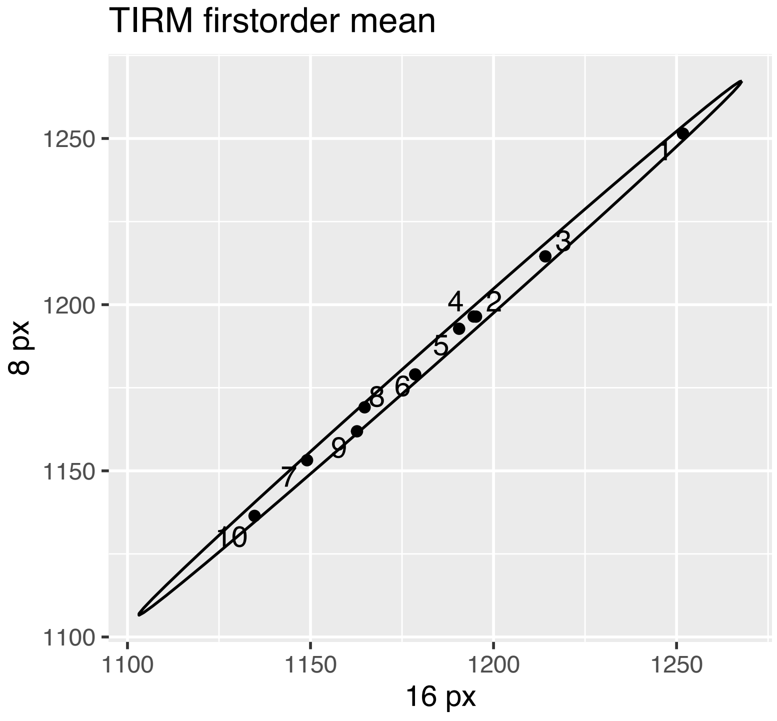
TIRM firstorder mean
8 px
16 px
1250
1200
1150
1100
1100
1150
1200
1250
1
2
3
4
5
6
7
8
9
10

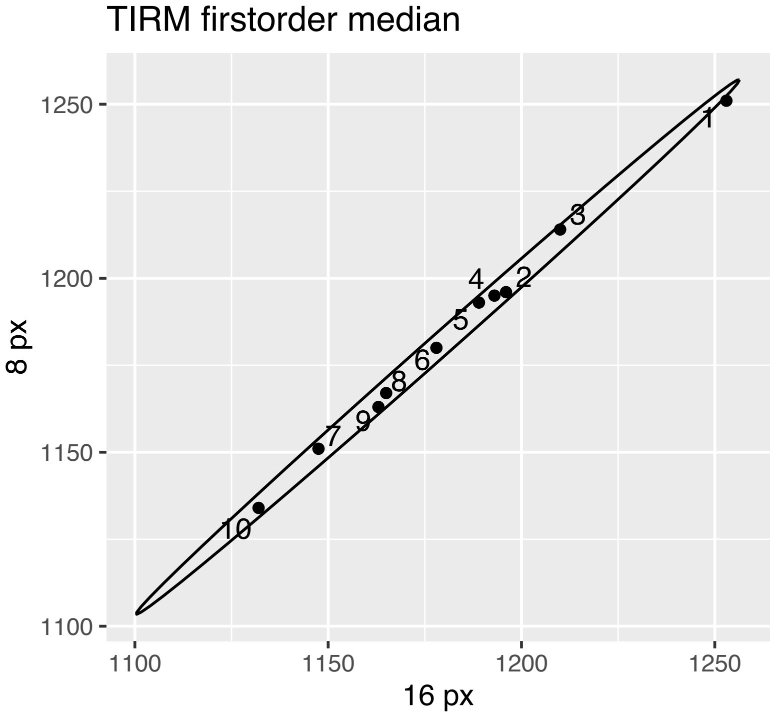
TIRM firstorder median
8 px
16 px
1100
1150
1200
1250
1
2
3
4
5
6
7
8
9
10

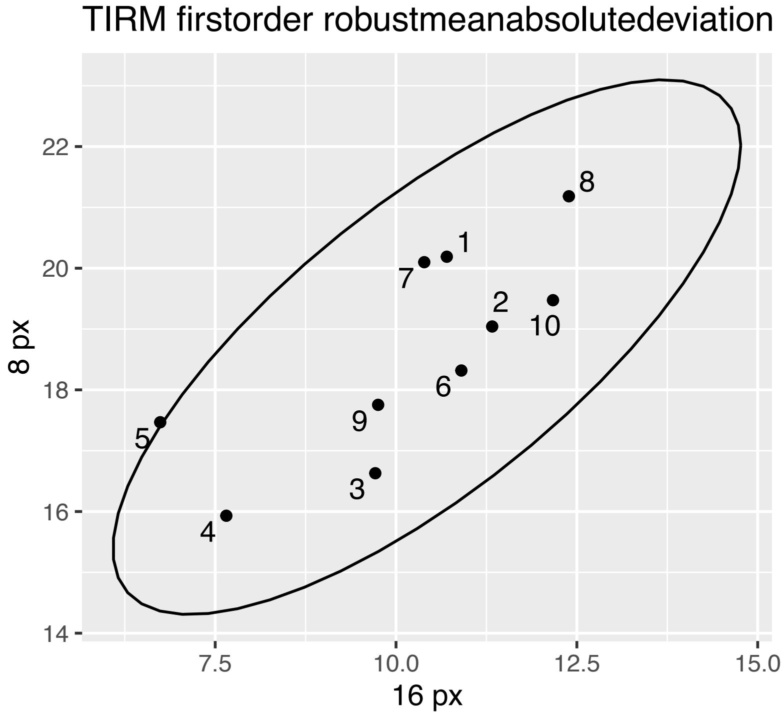
TIRM firstorder robustmeanabsolutedeviation
8 px
16 px
7.5
10.0
12.5
15.0
14
16
18
20
22
1
2
3
4
5
6
7
8
9
10

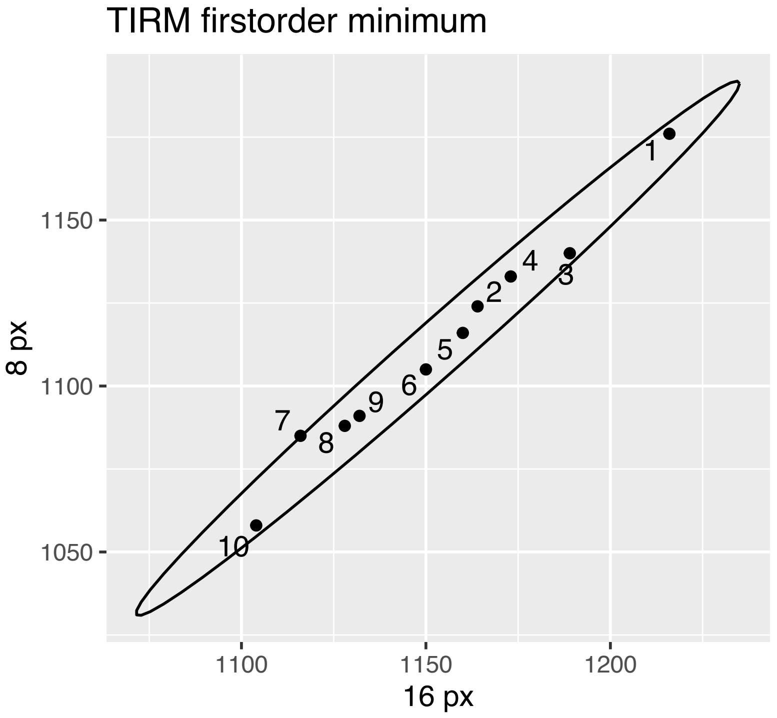
TIRM firstorder minimum
8 px
16 px
1100
1150
1200
1050
1
2
3
4
5
6
7
8
9
10

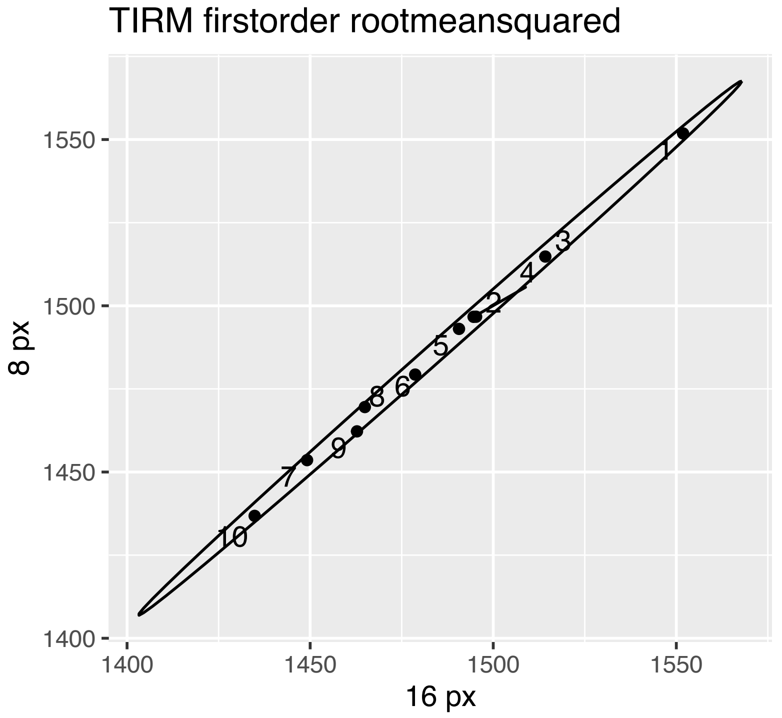
TIRM firstorder rootmeansquared
8 px
16 px
1400
1450
1500
1550
1
2
3
4
5
6
7
8
9
10

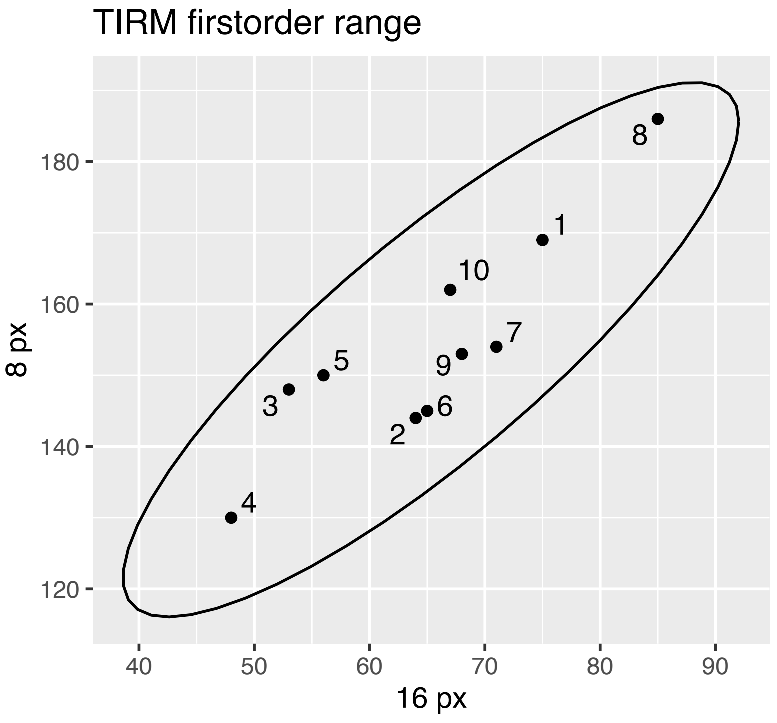
TIRM firstorder range
8 px
16 px
40
50
60
70
80
90
120
140
160
180
1
2
3
4
5
6
7
8
9
10

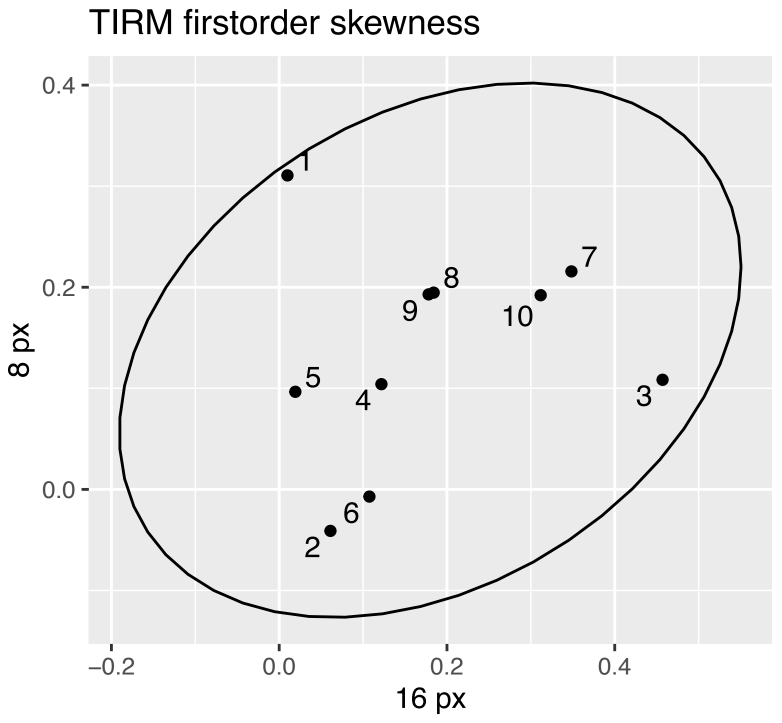
TIRM firstorder skewness
8 px
16 px
−0.2
0.0
0.2
0.4
1
2
3
4
5
6
7
8
9
10

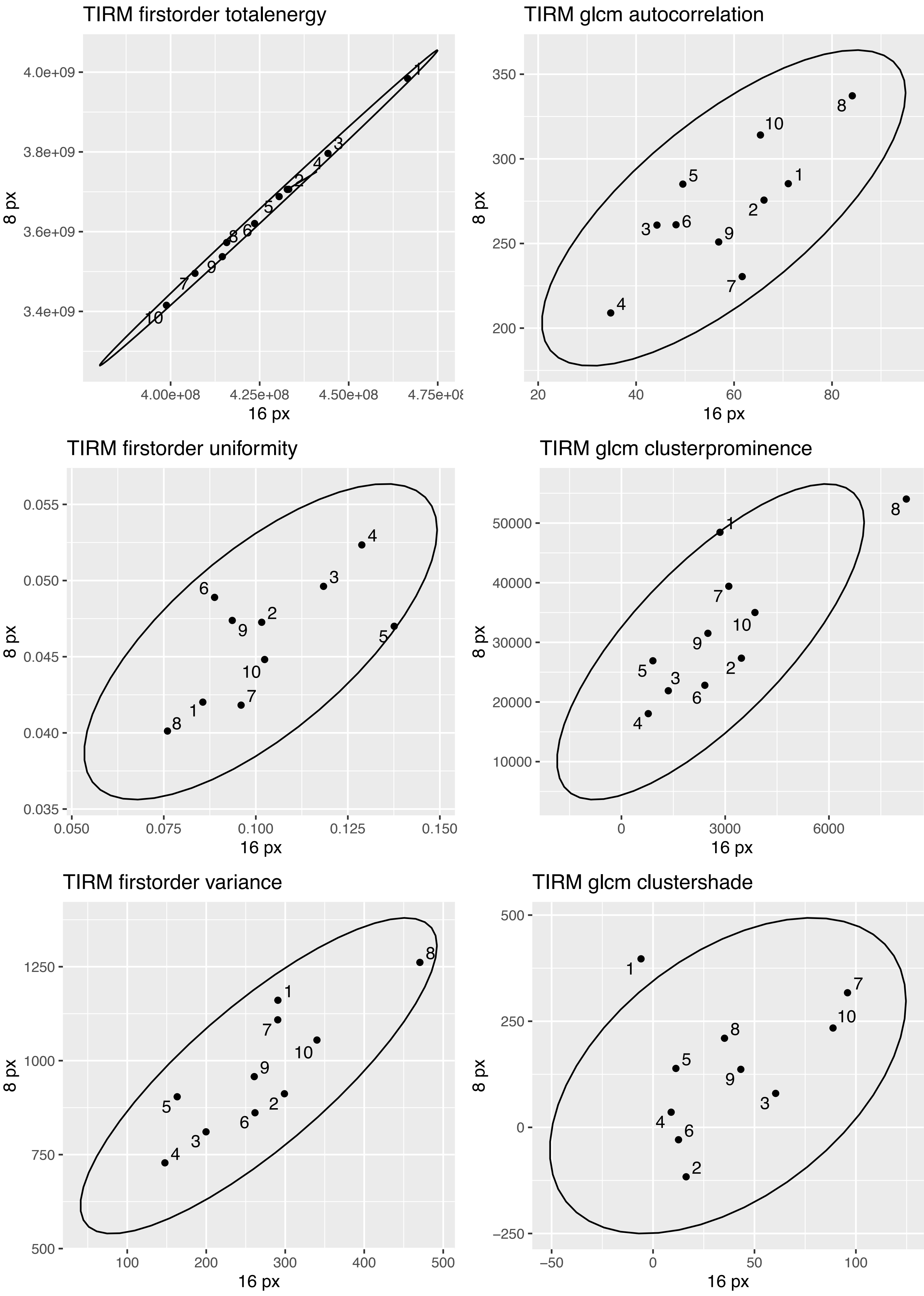
TIRM firstorder totalenergy
8 px
16 px
4.0e+09
3.8e+09
3.6e+09
3.4e+09
4.00e+08
4.25e+08
4.50e+08
4.75e+08
TIRM glcm autocorrelation
350
300
250
200
20
40
60
80
TIRM firstorder uniformity
0.055
0.050
0.045
0.040
0.035
0.050
0.075
0.100
0.125
0.150
TIRM glcm clusterprominence
50000
40000
30000
20000
10000
0
3000
6000
TIRM firstorder variance
1250
1000
750
500
100
200
300
400
500
TIRM glcm clustershade
500
250
0
−250
−50
0
50
100

TIRM glcm clustertendency

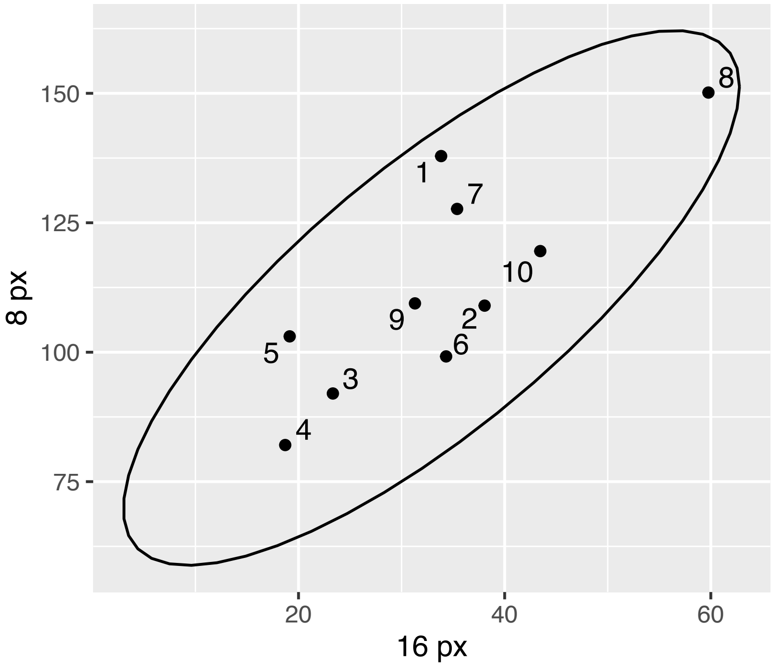

TIRM glcm differenceaverage

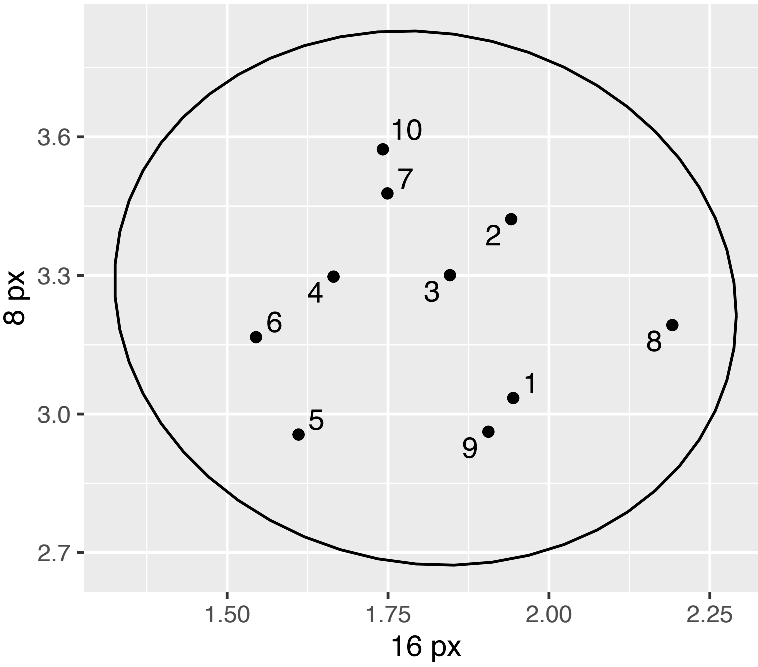

TIRM glcm contrast

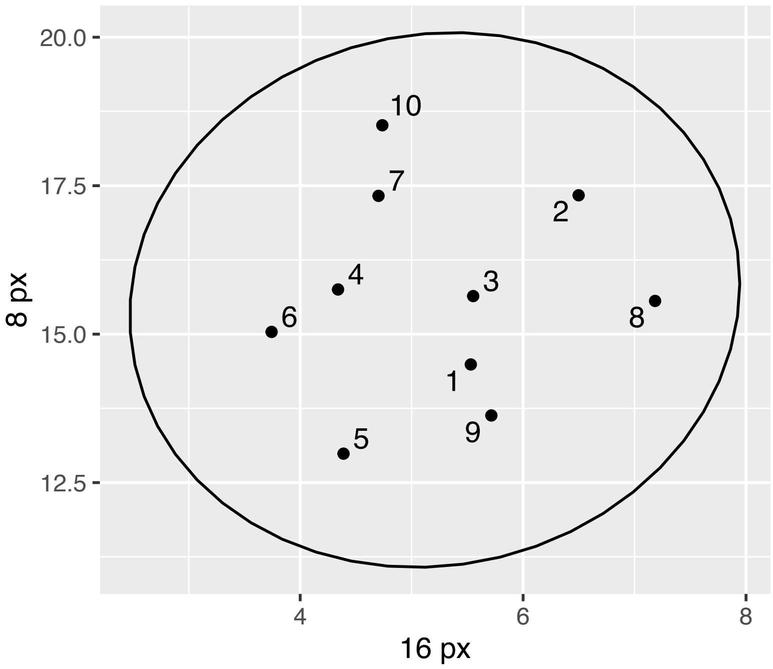

TIRM glcm differenceentropy

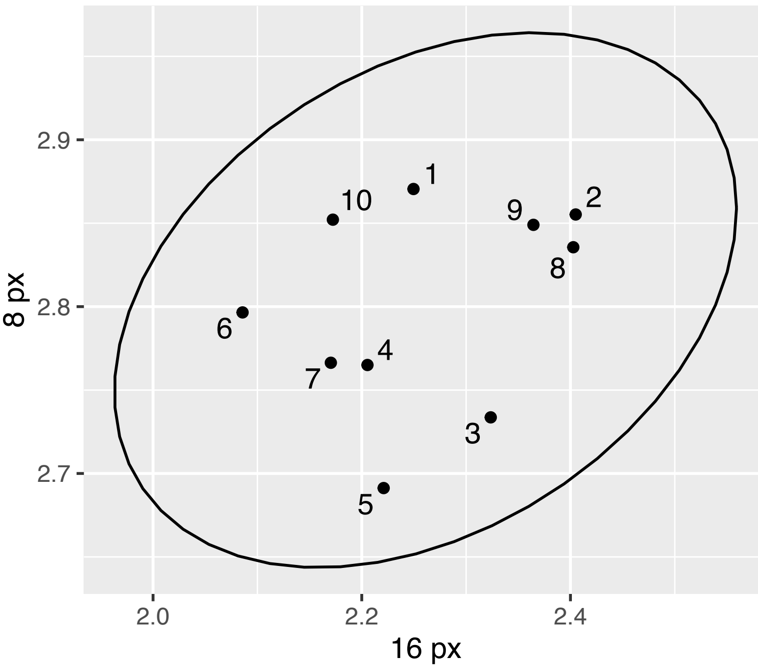

TIRM glcm correlation

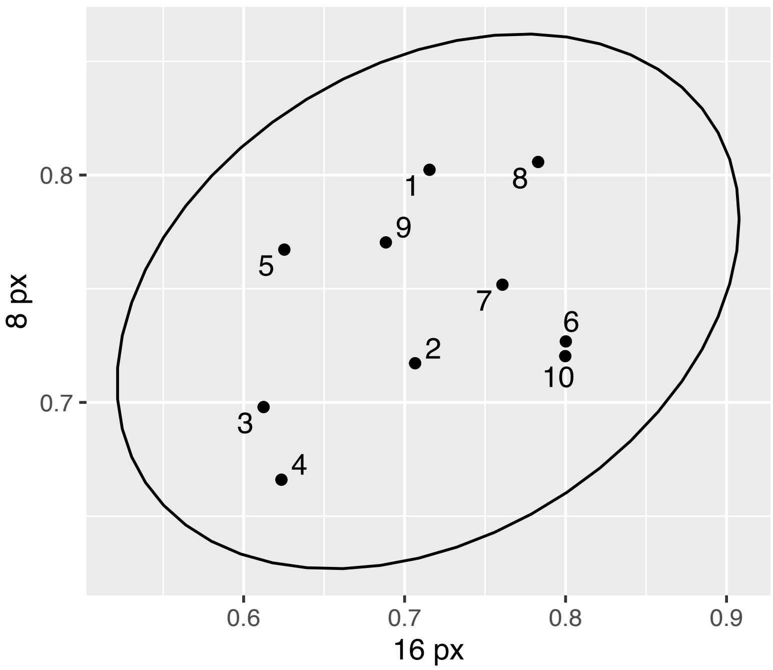

TIRM glcm differencevariance

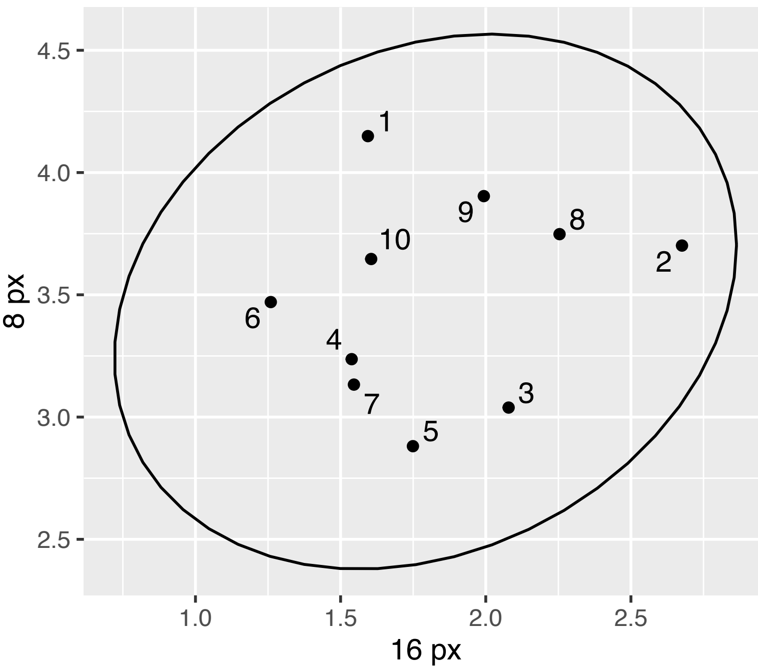

TIRM glcm id

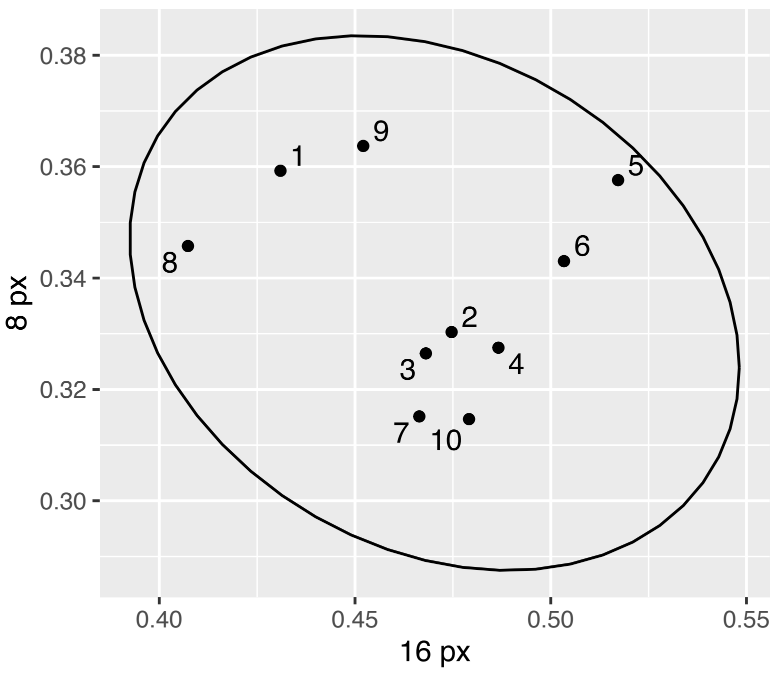

TIRM glcm idn

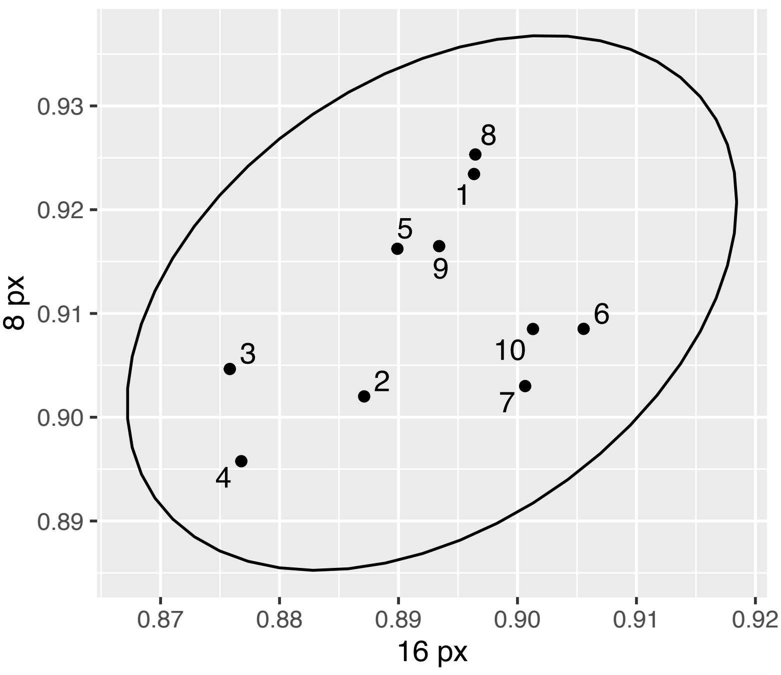

TIRM glcm idm

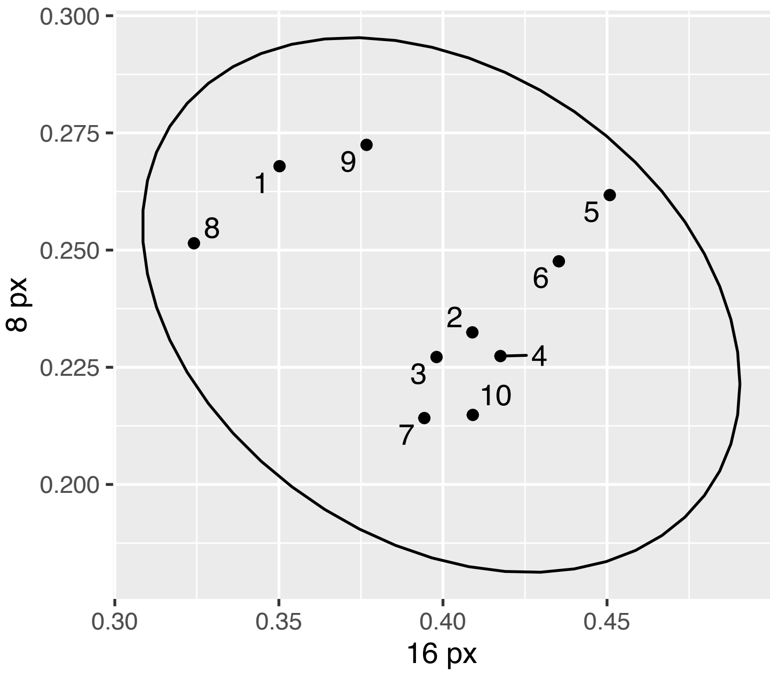

TIRM glcm imc1

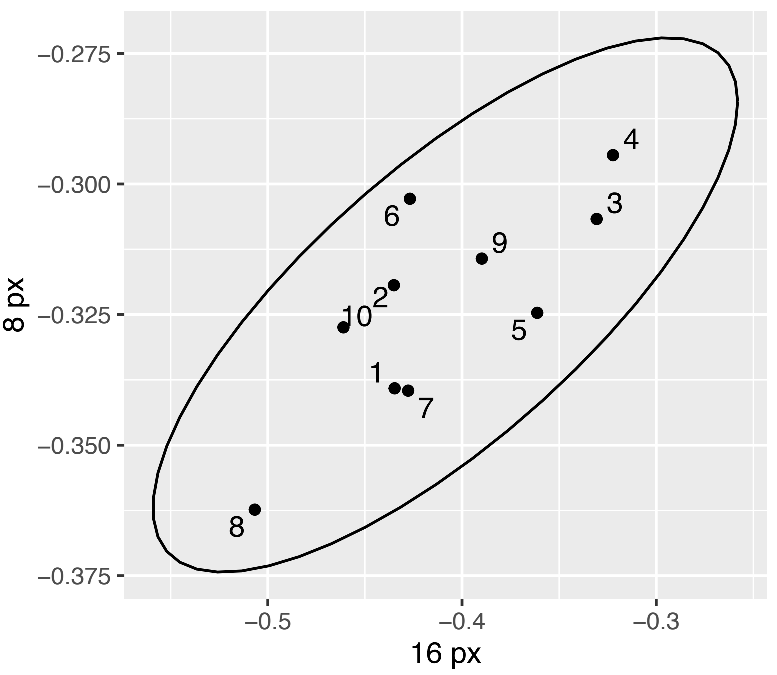

TIRM glcm idmn

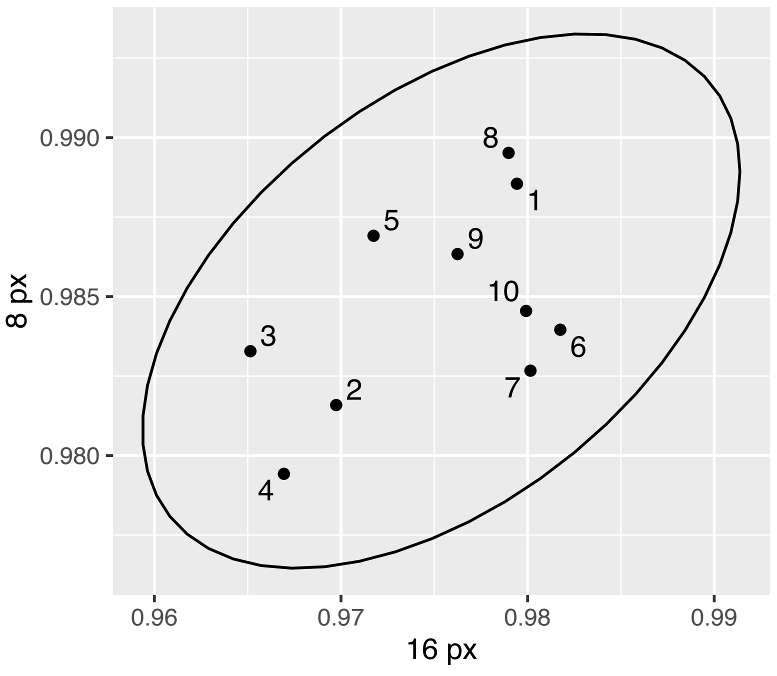

TIRM glcm imc2

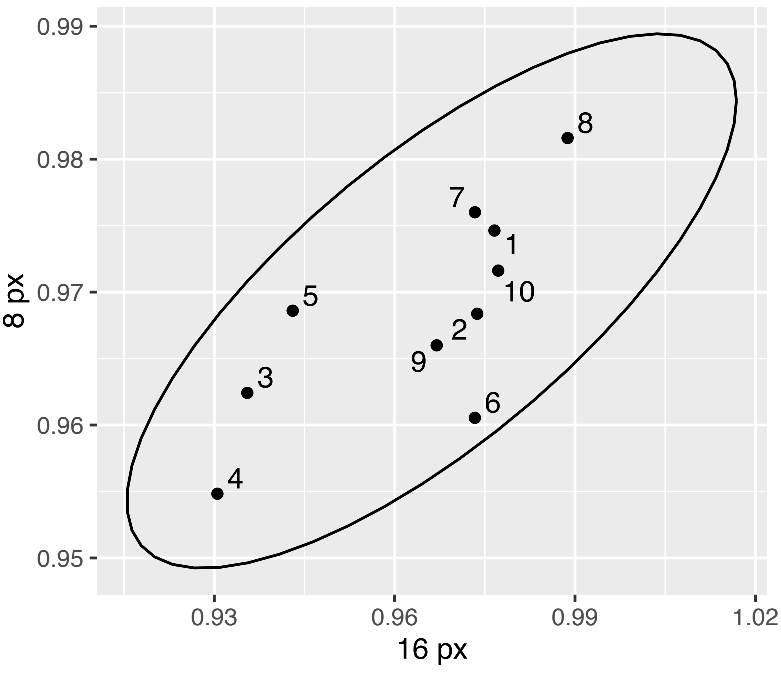

TIRM glcm inversevariance

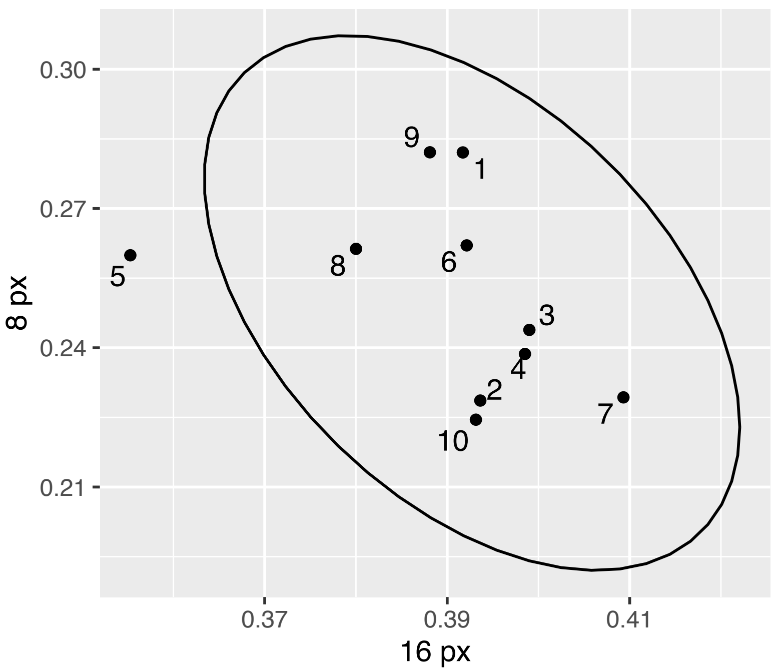

TIRM glcm jointentropy

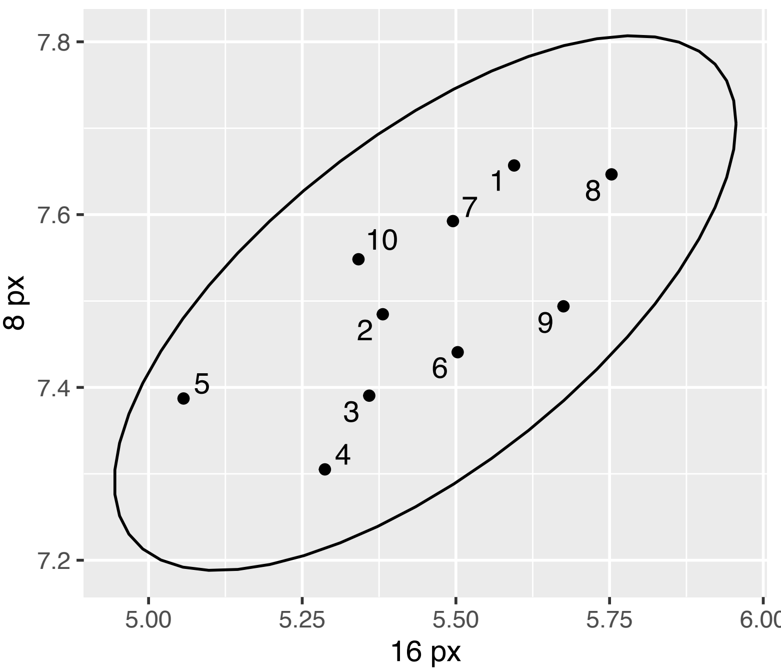

TIRM glcm jointaverage

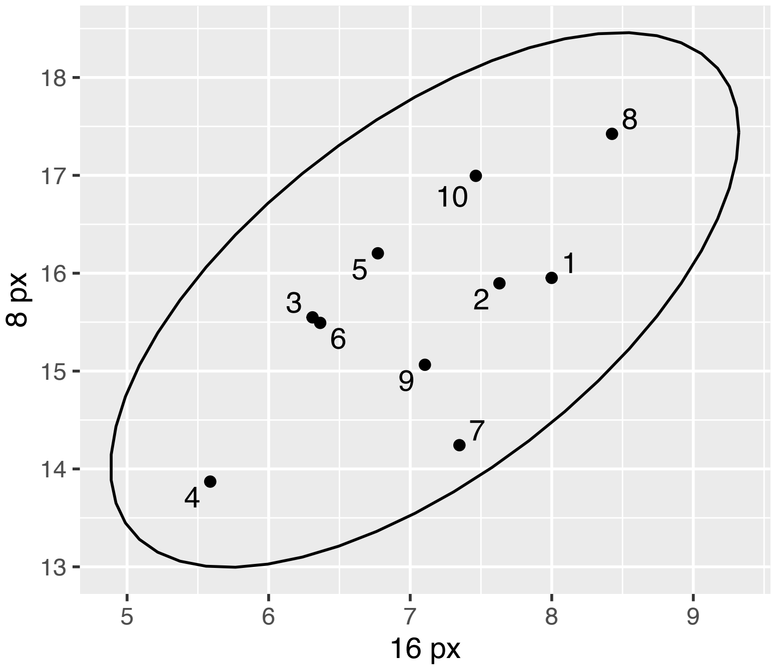

TIRM glcm mcc

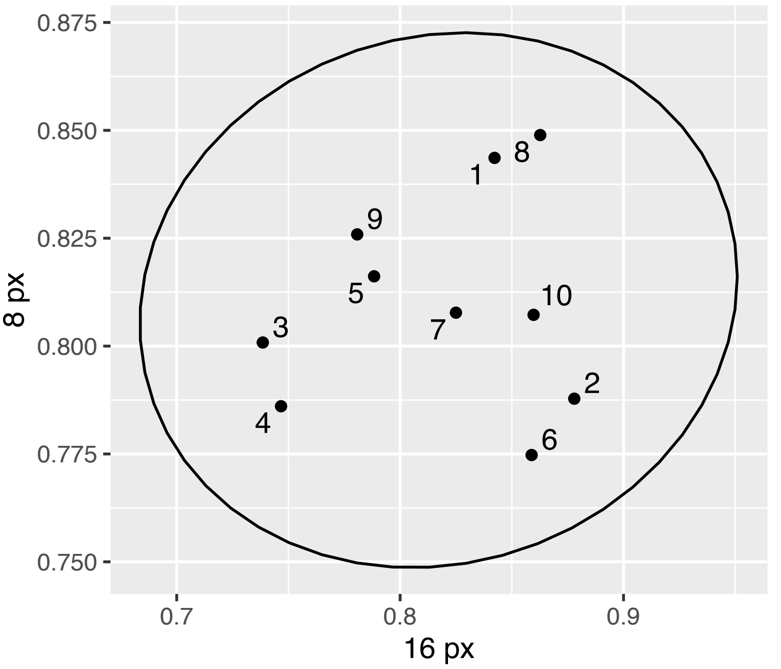

TIRM glcm jointenergy

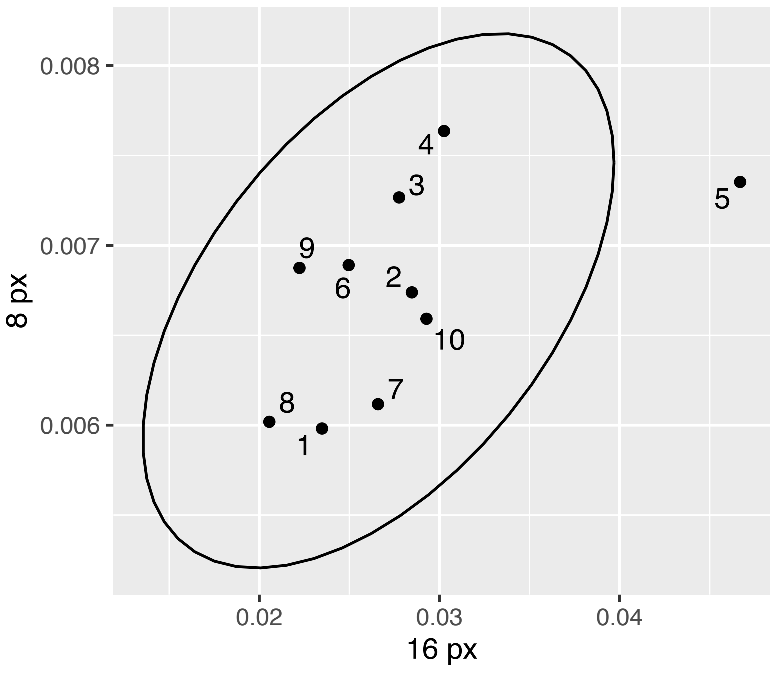

TIRM glcm maximumprobability

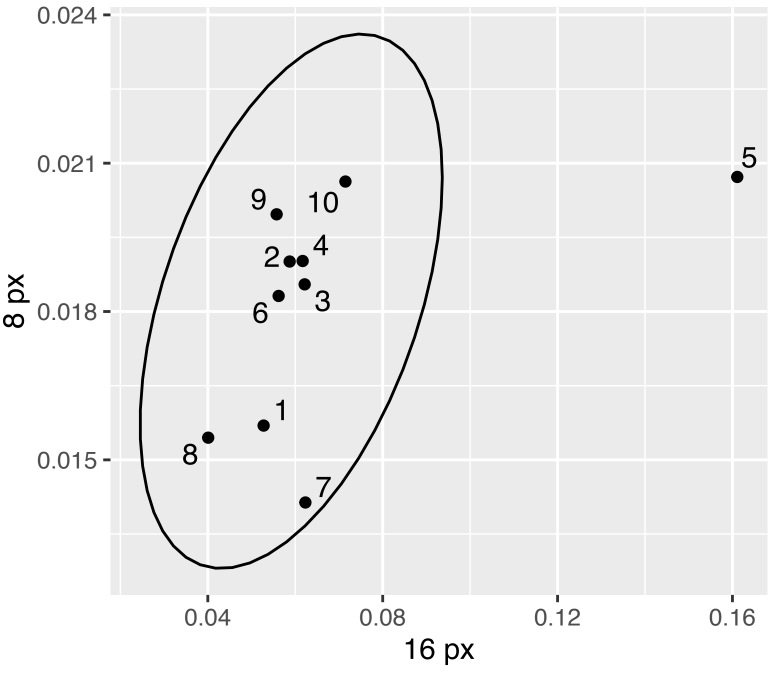

TIRM glcm sumaverage

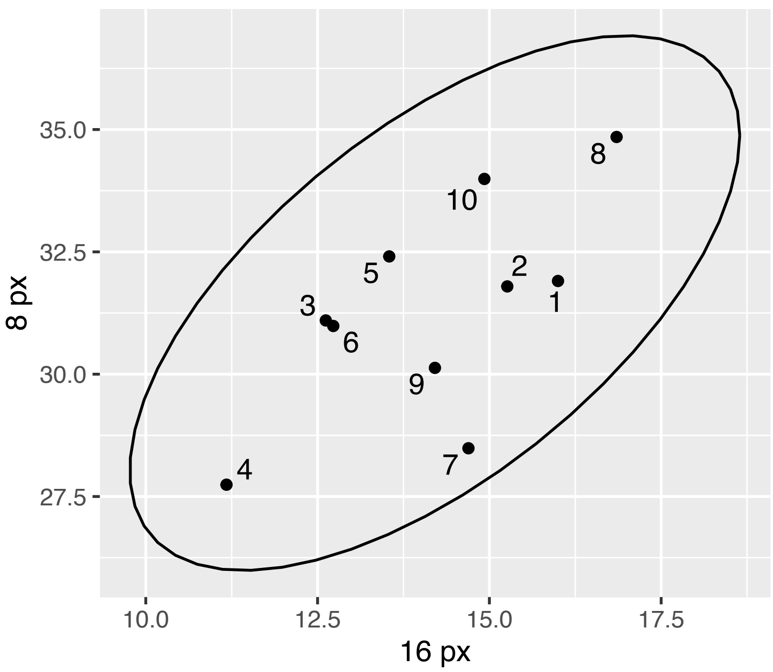

TIRM glrlm graylevelnonuniformity

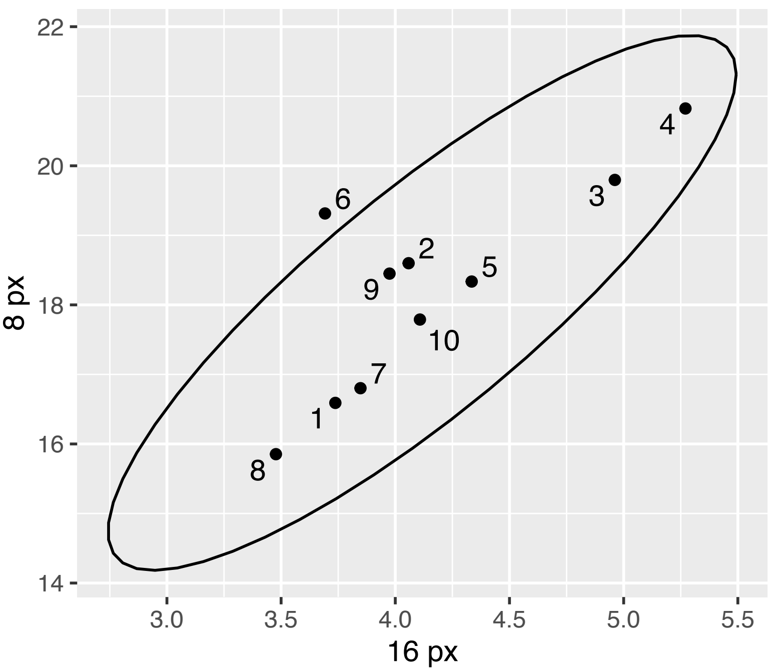

TIRM glcm sumentropy

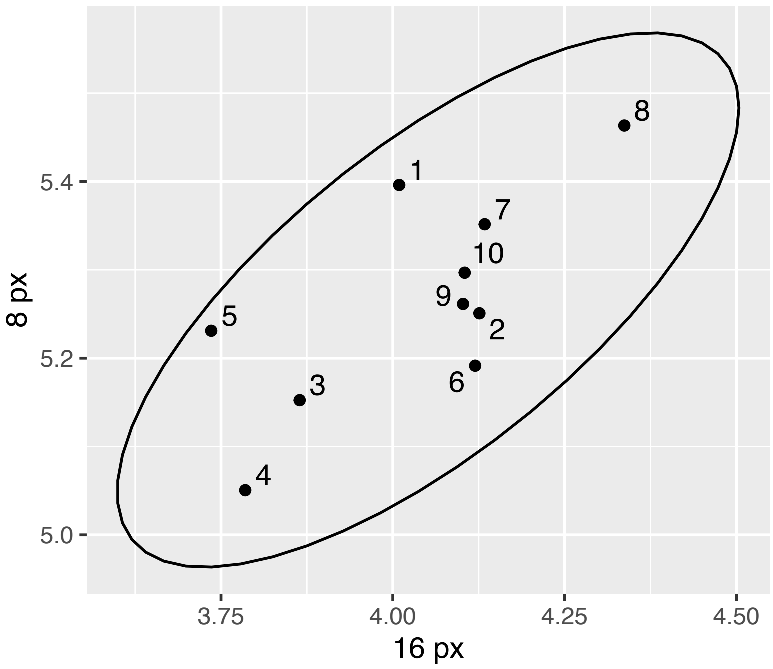

TIRM glrlm graylevelnonuniformitynormalize

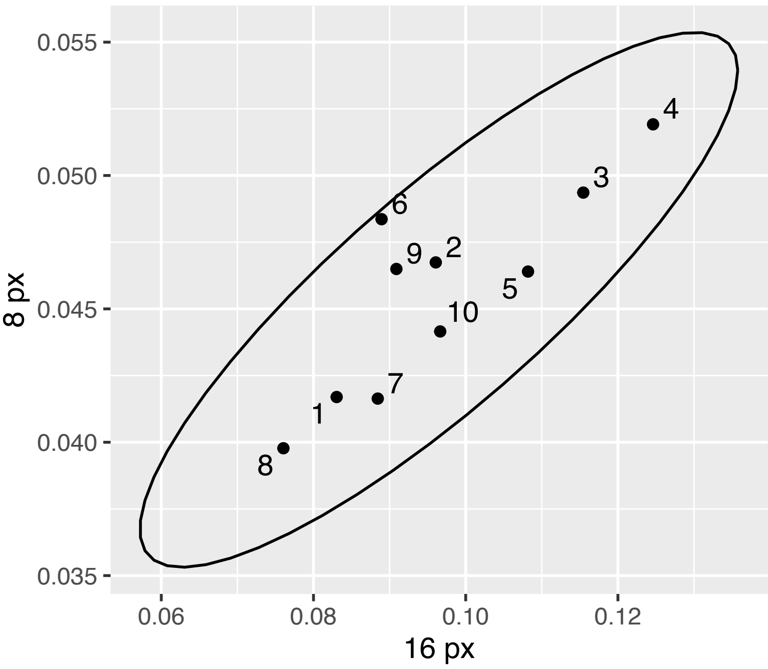

TIRM glcm sumsquares

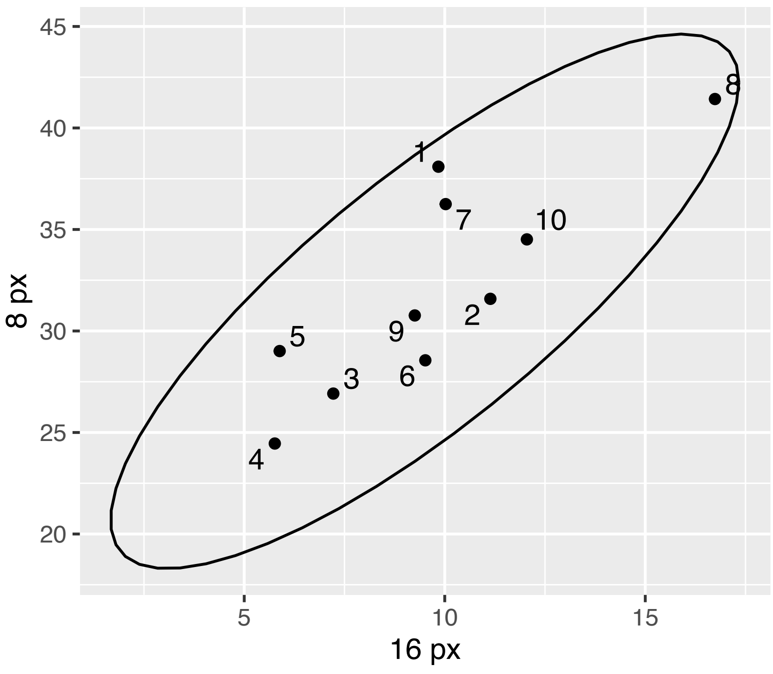

TIRM glrlm graylevelvariance

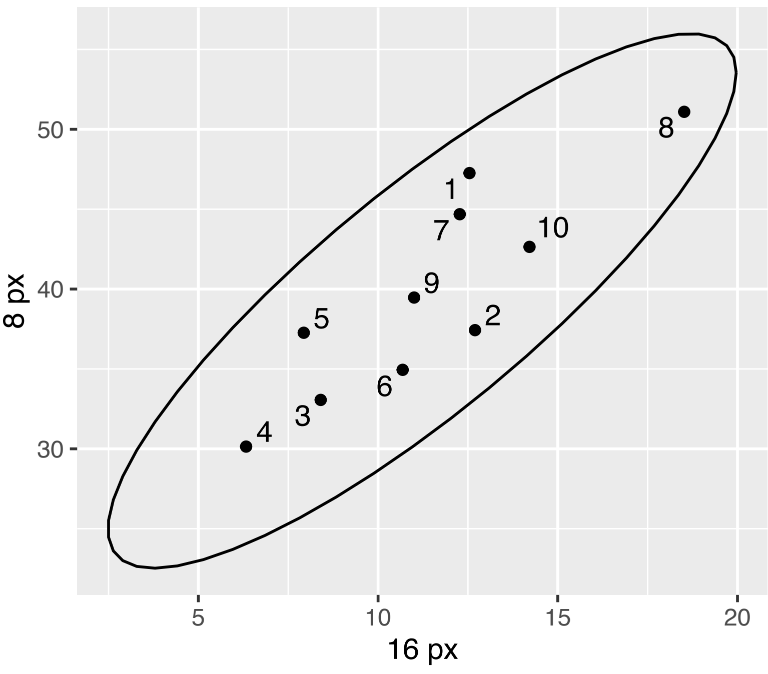

TIRM glrlm highgraylevelrunemphasis

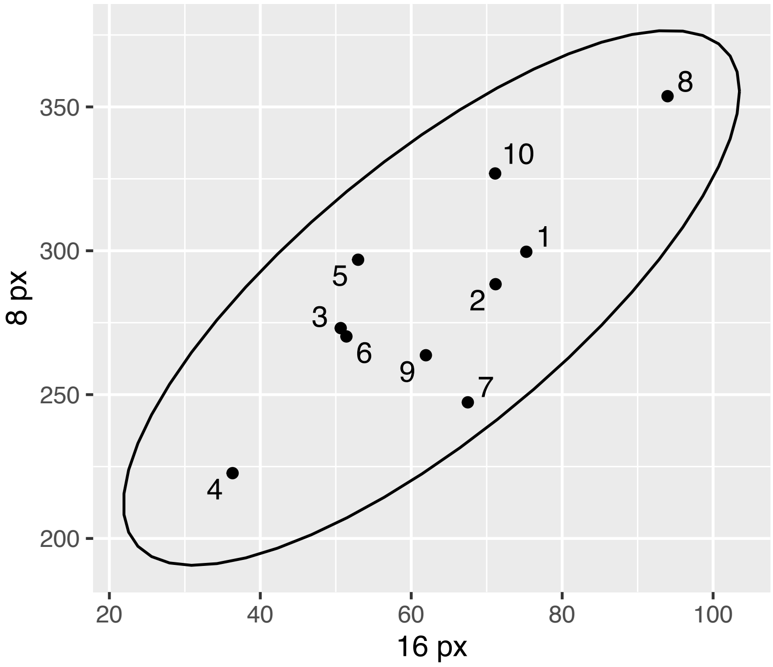

TIRM glrlm longrunlowgraylevelemphasis

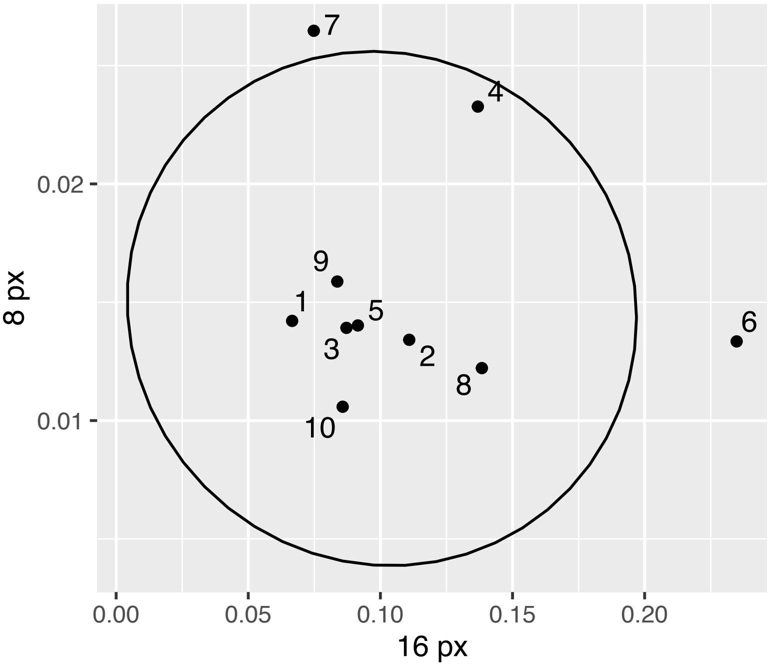

TIRM glrlm longrunemphasis

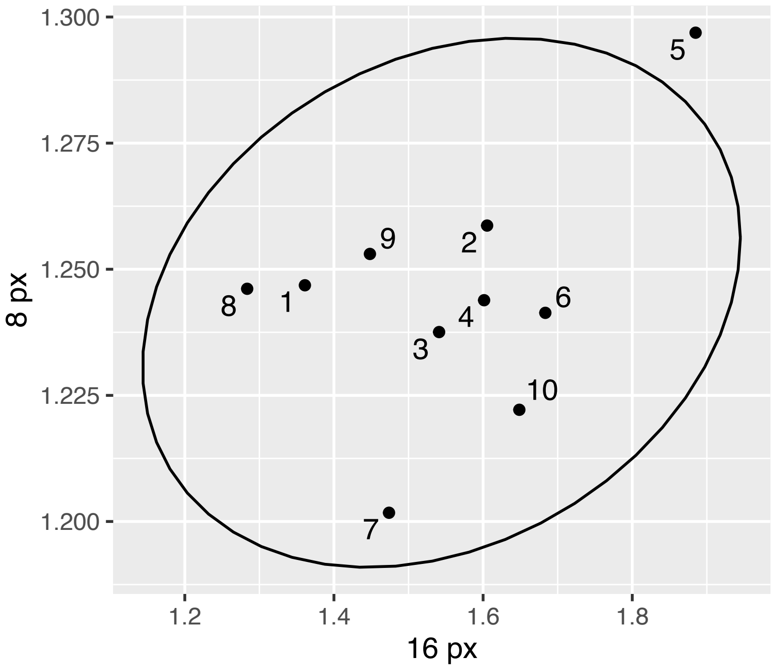

TIRM glrlm lowgraylevelrunemphasis

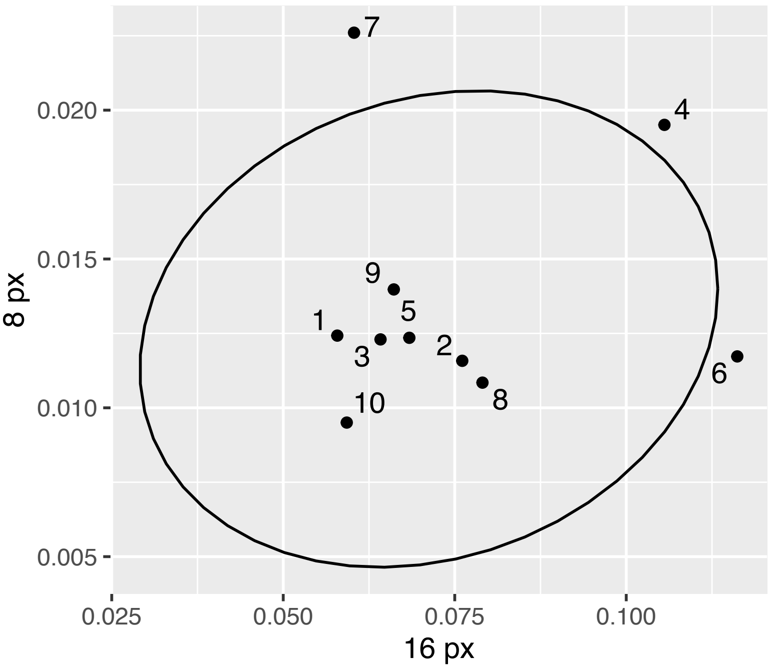

TIRM glrlm longrunhighgraylevelemphasis

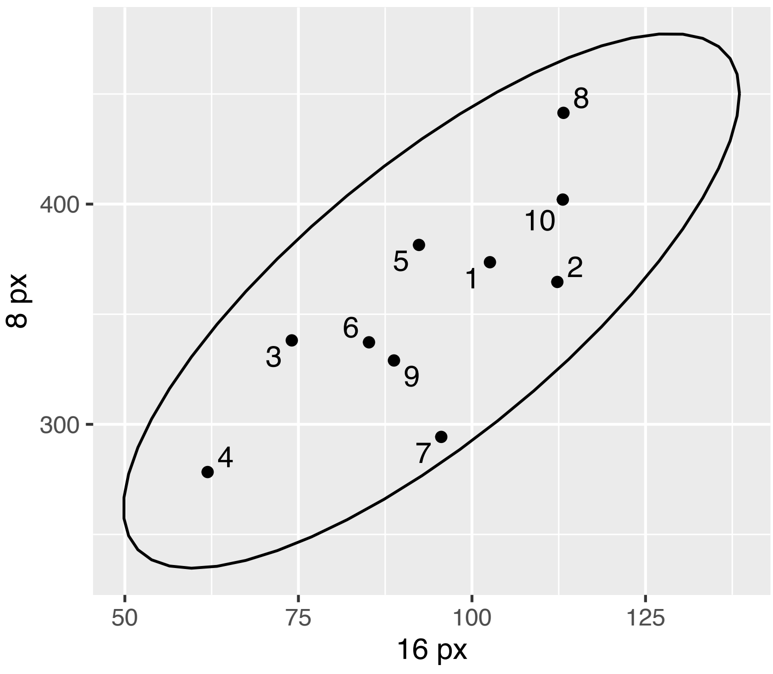

TIRM glrlm runentropy

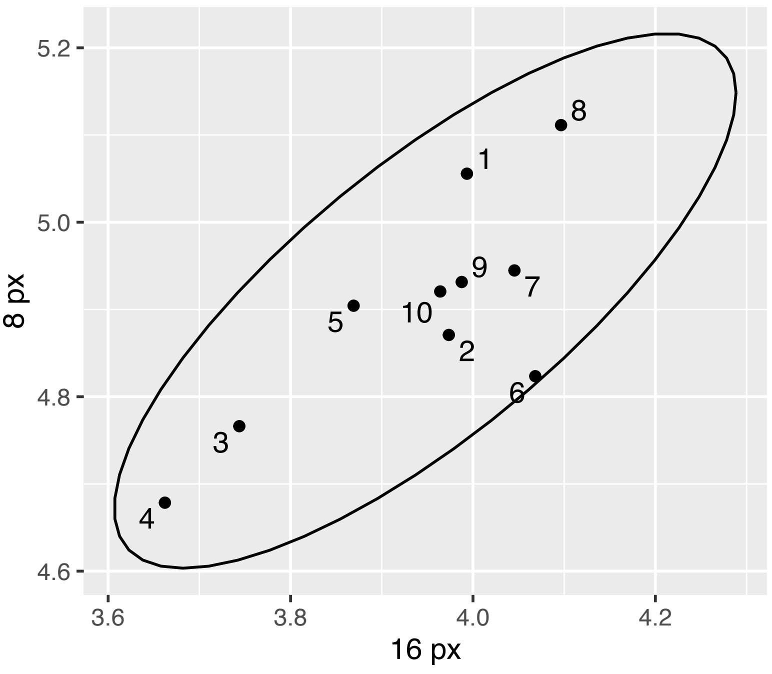

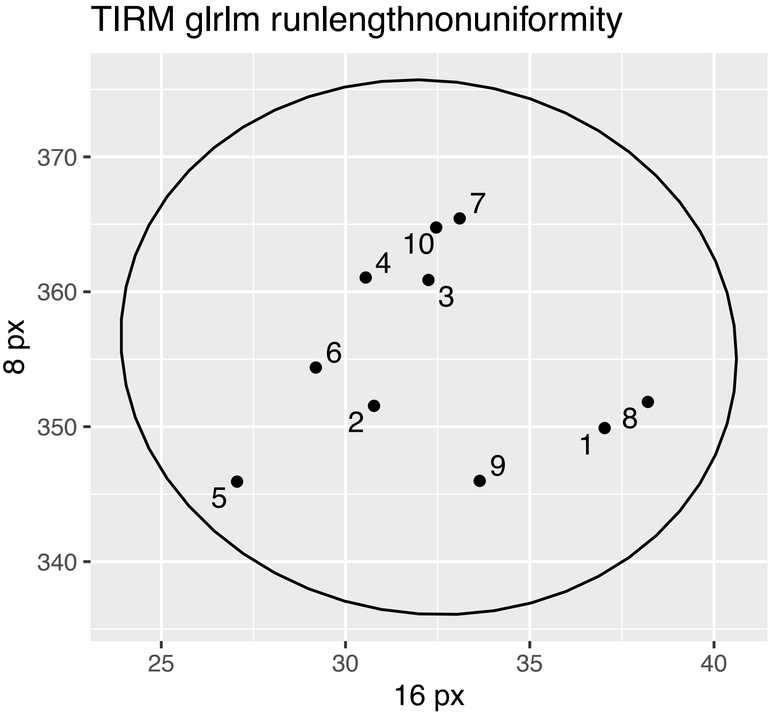
TIRM glrlm runlengthnonuniformity
8 px
16 px

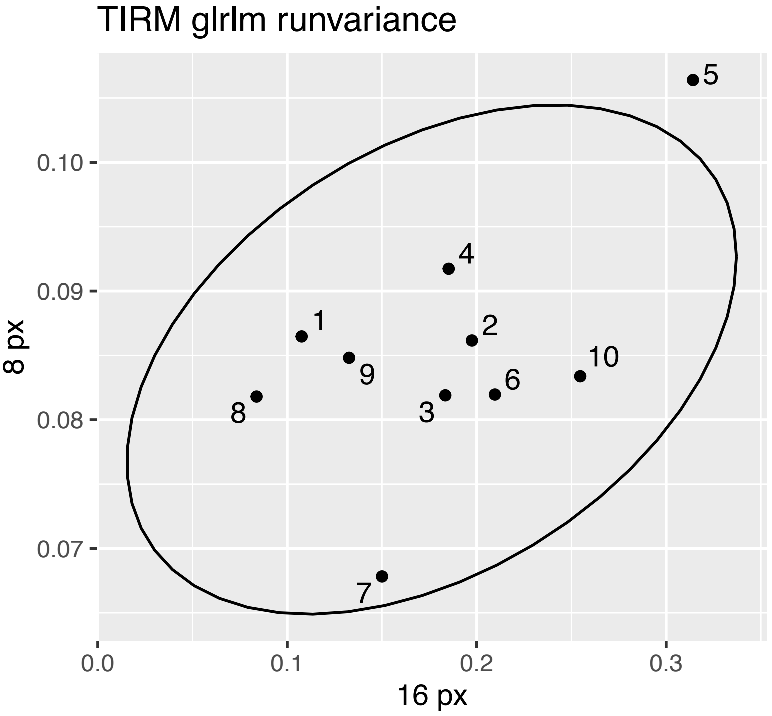
TIRM glrlm runvariance
8 px
16 px

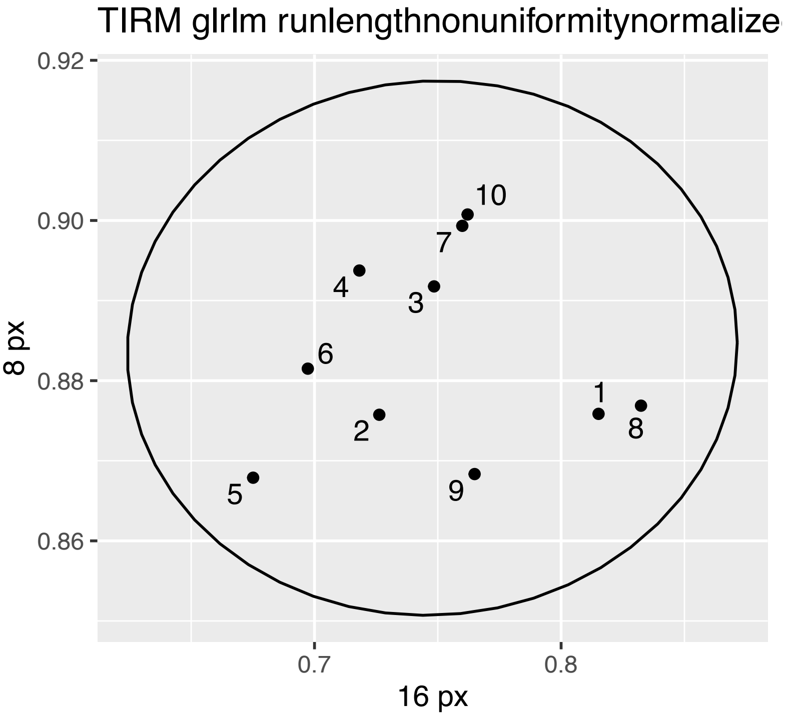
TIRM glrlm runlengthnonuniformitynormalize
8 px
16 px

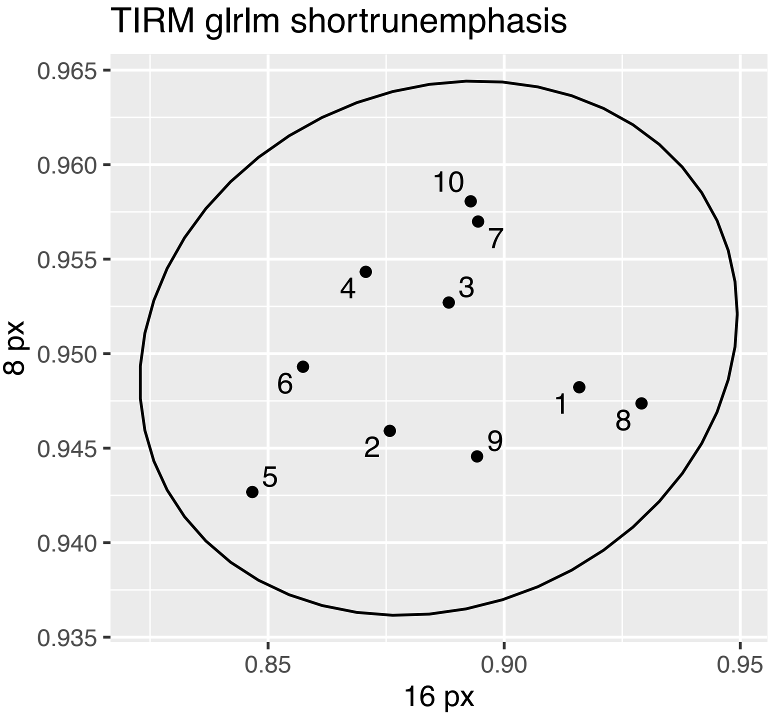
TIRM glrlm shortrunemphasis
8 px
16 px

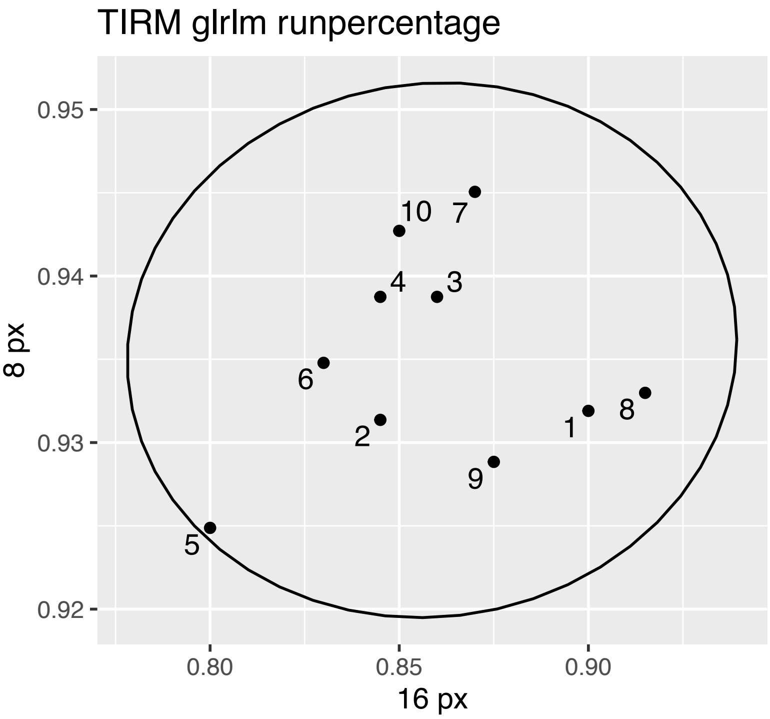
TIRM glrlm runpercentage
8 px
16 px

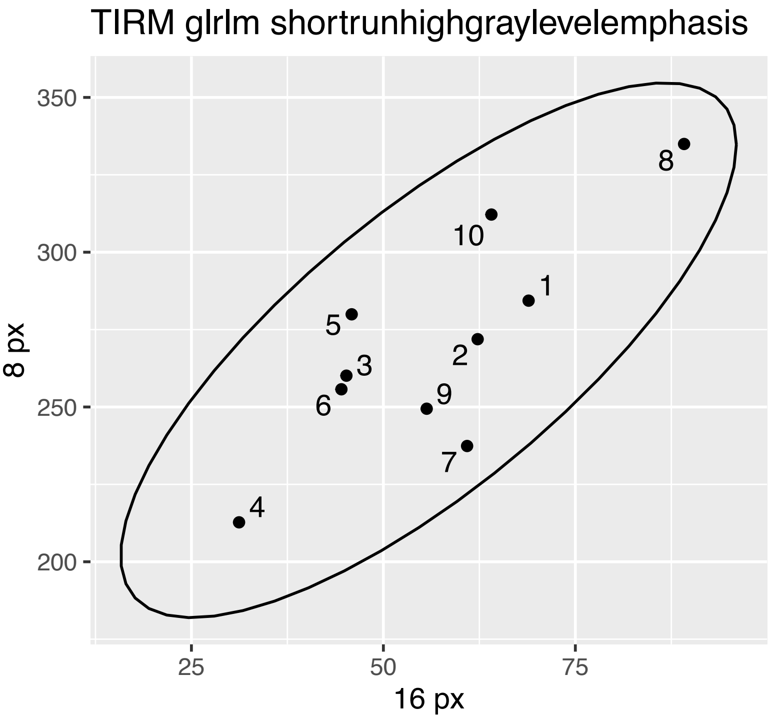
TIRM glrlm shortrunhighgraylevelemphasis
8 px
16 px

TIRM glrlm shortrunlowgraylevelemphasis

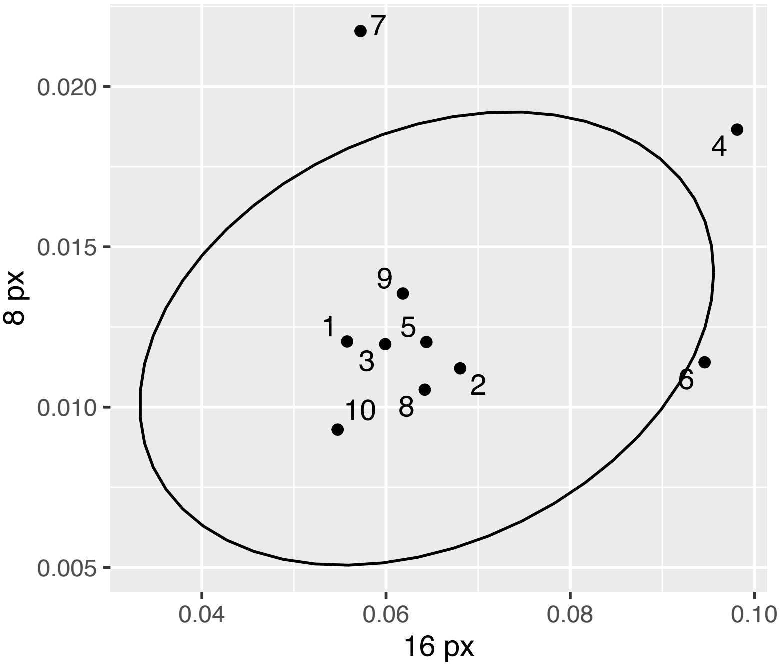

TIRM glszm graylevelvariance

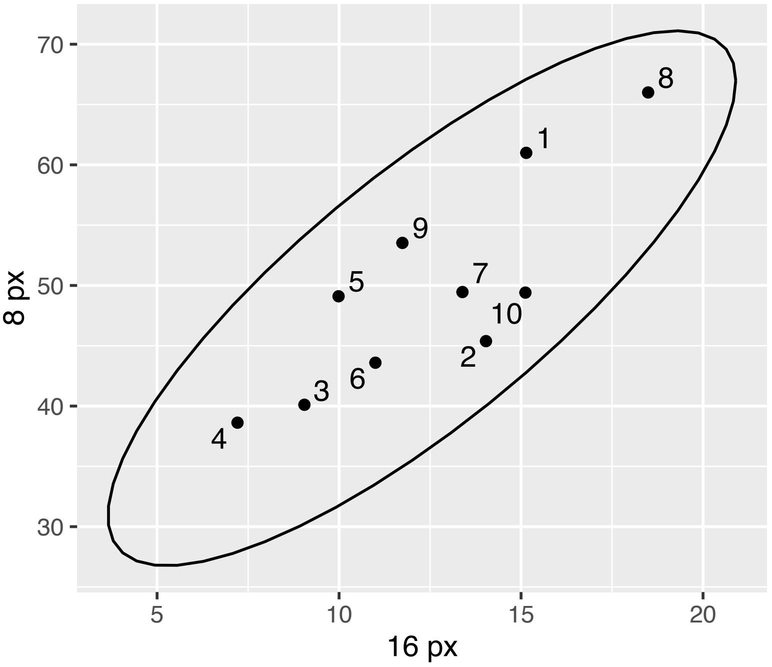

TIRM glszm graylevelnonuniformity

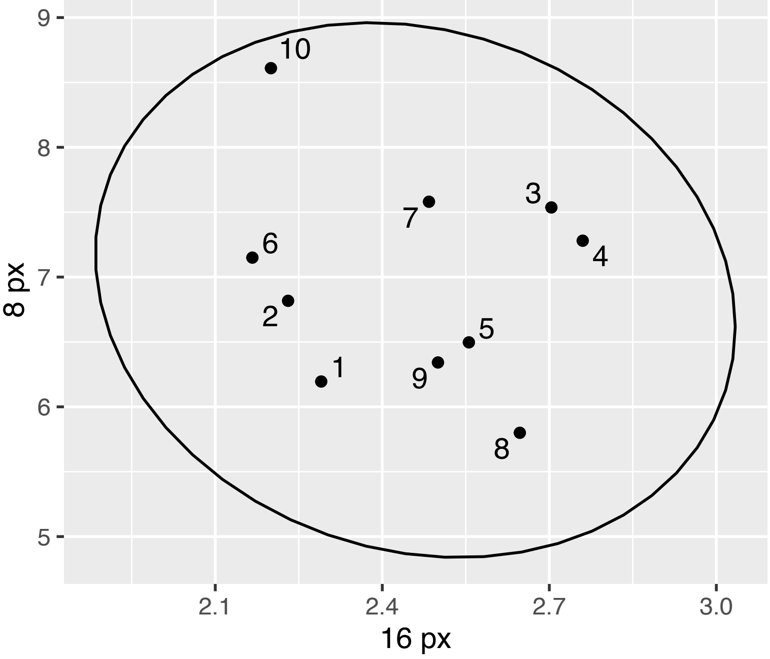

TIRM glszm highgraylevelzoneemphasis

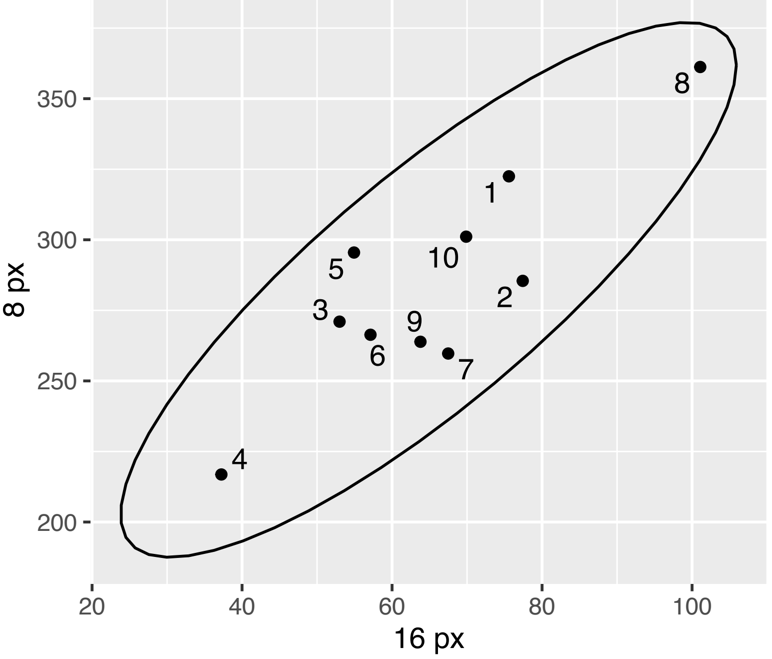

TIRM glszm graylevelnonuniformitynormaliz

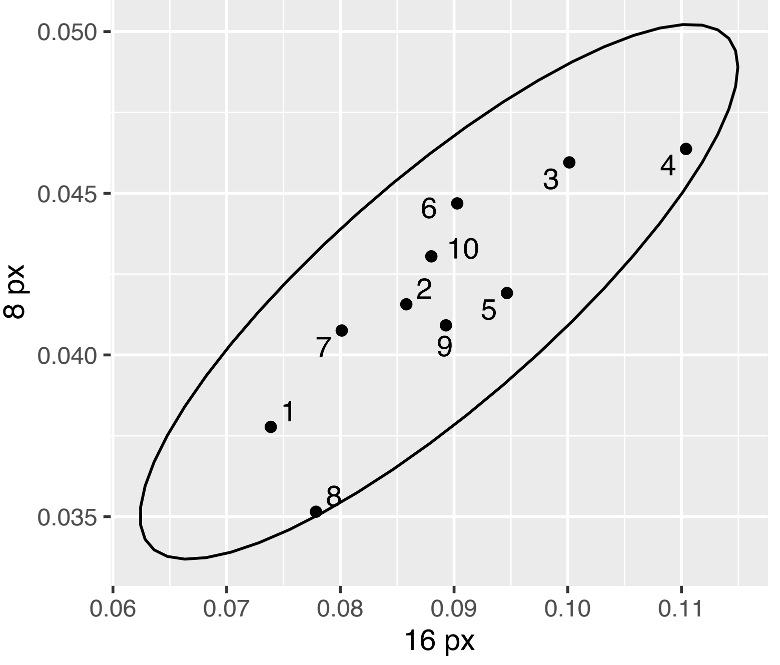

TIRM glszm largeareaemphasis

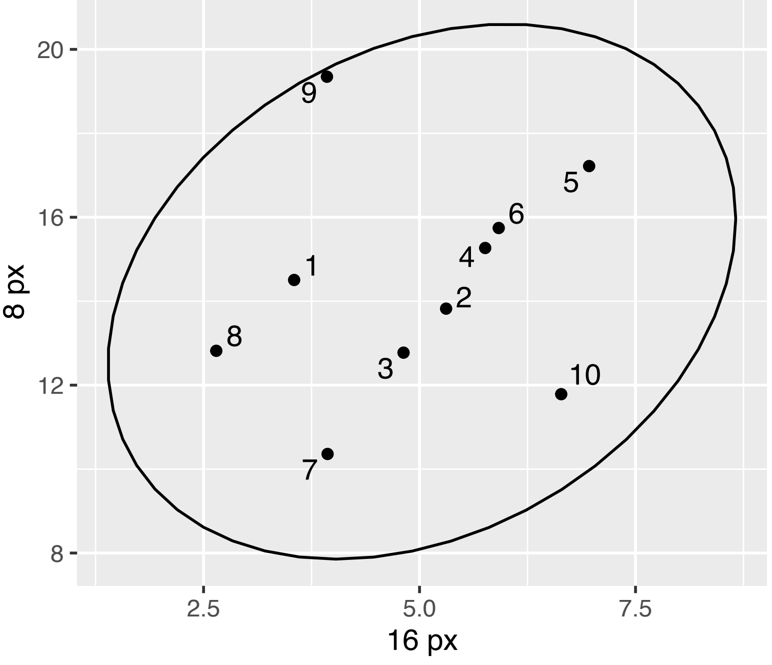

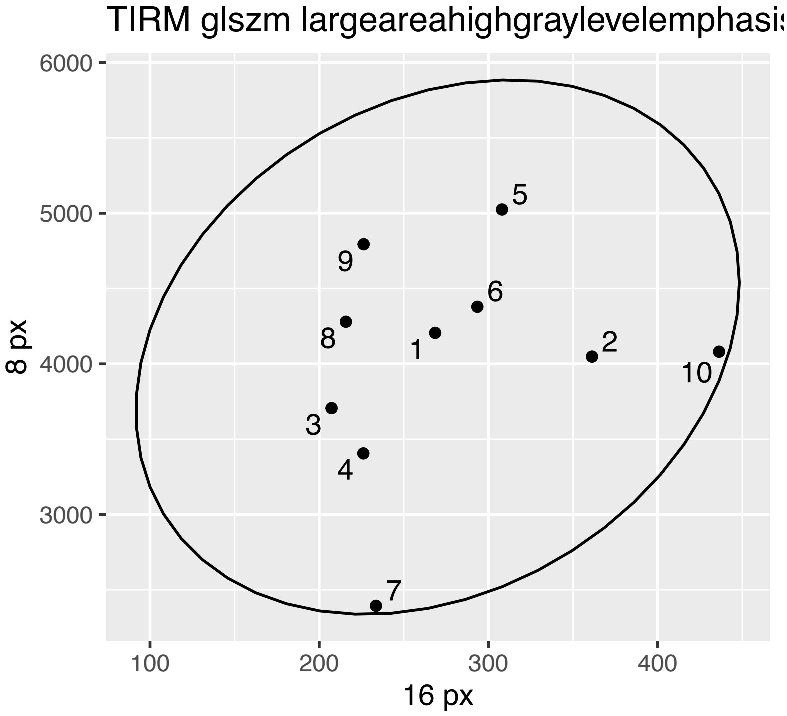
TIRM glszm largeareahighgraylevelemphasis
6000
5000
4000
3000
8 px
100
200
300
400
16 px
1 2 3 4 5 6 7 8 9 10

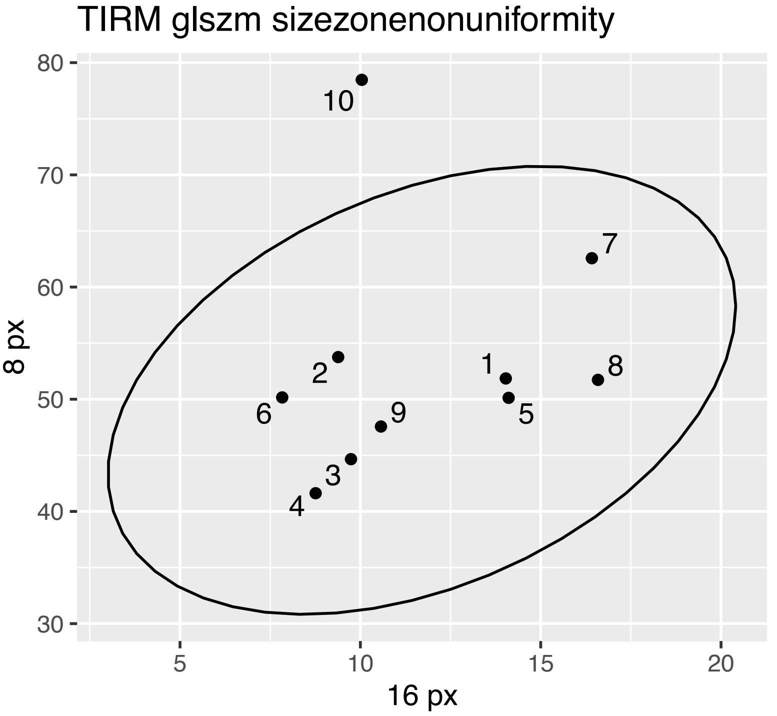
TIRM glszm sizezonenonuniformity
80
70
60
50
40
30
8 px
5
10
15
20
16 px
1 2 3 4 5 6 7 8 9 10

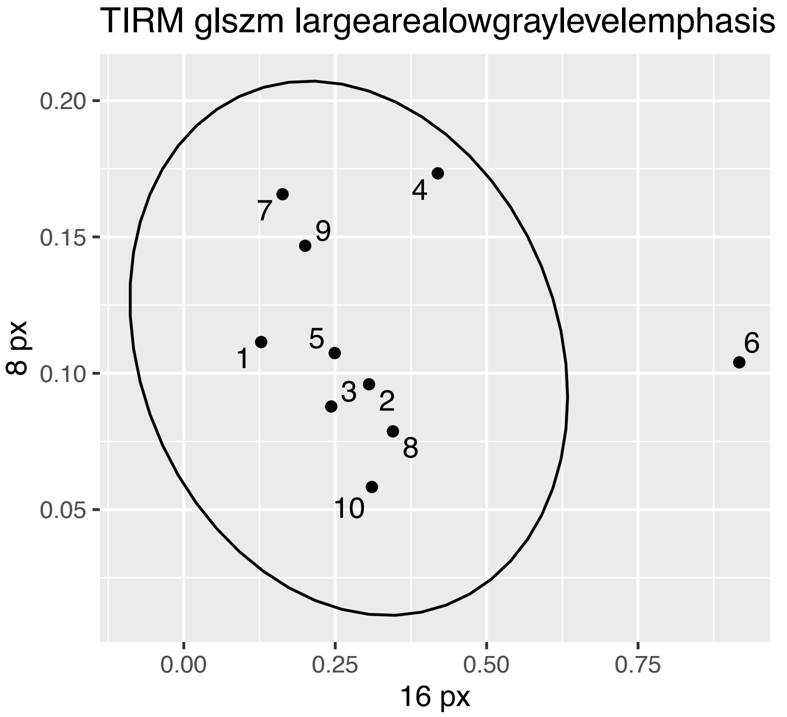
TIRM glszm largearealowgraylevelemphasis
0.20
0.15
0.10
0.05
8 px
0.00
0.25
0.50
0.75
16 px
1 2 3 4 5 6 7 8 9 10

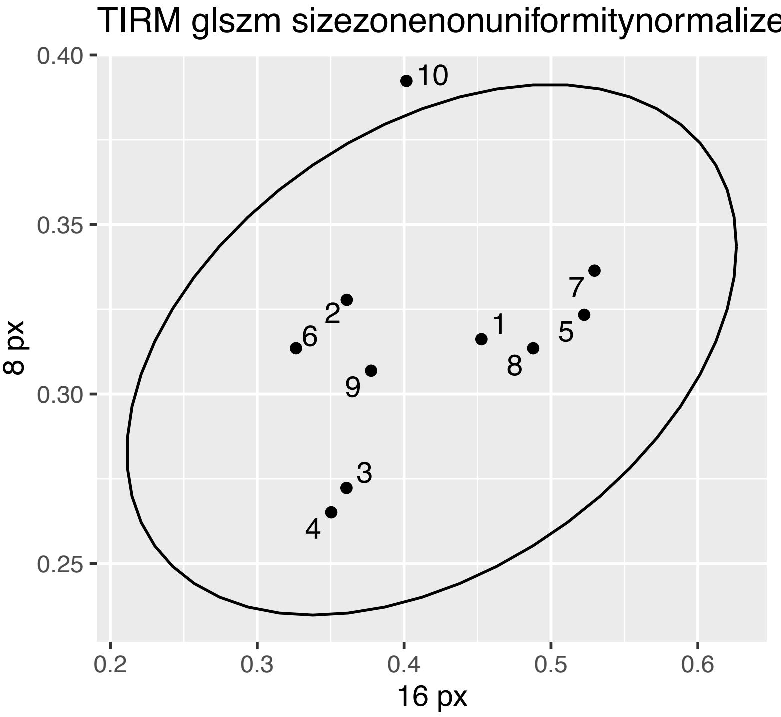
TIRM glszm sizezonenonuniformitynormalize
0.40
0.35
0.30
0.25
8 px
0.2
0.3
0.4
0.5
0.6
16 px
1 2 3 4 5 6 7 8 9 10

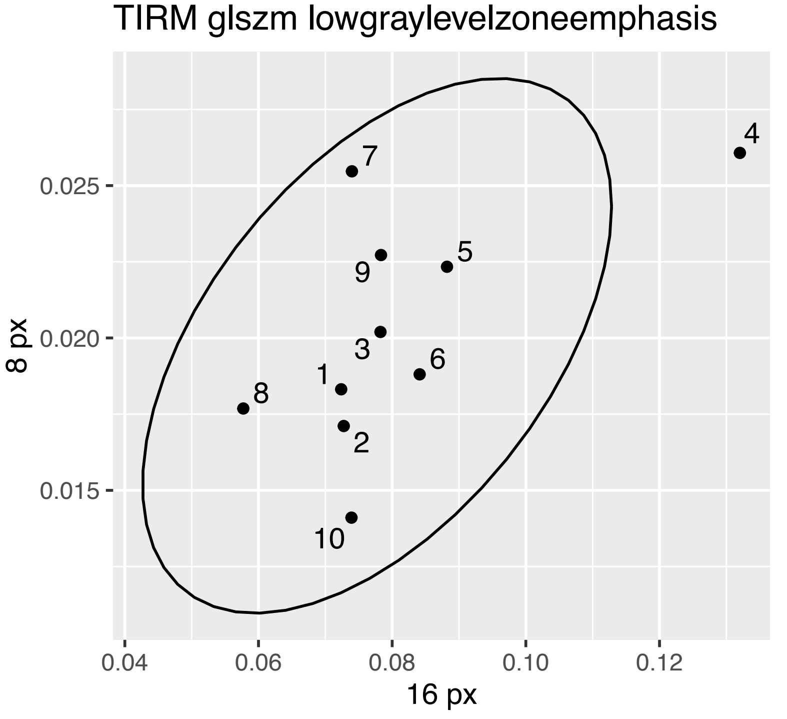
TIRM glszm lowgraylevelzoneemphasis
0.025
0.020
0.015
8 px
0.04
0.06
0.08
0.10
0.12
16 px
1 2 3 4 5 6 7 8 9 10

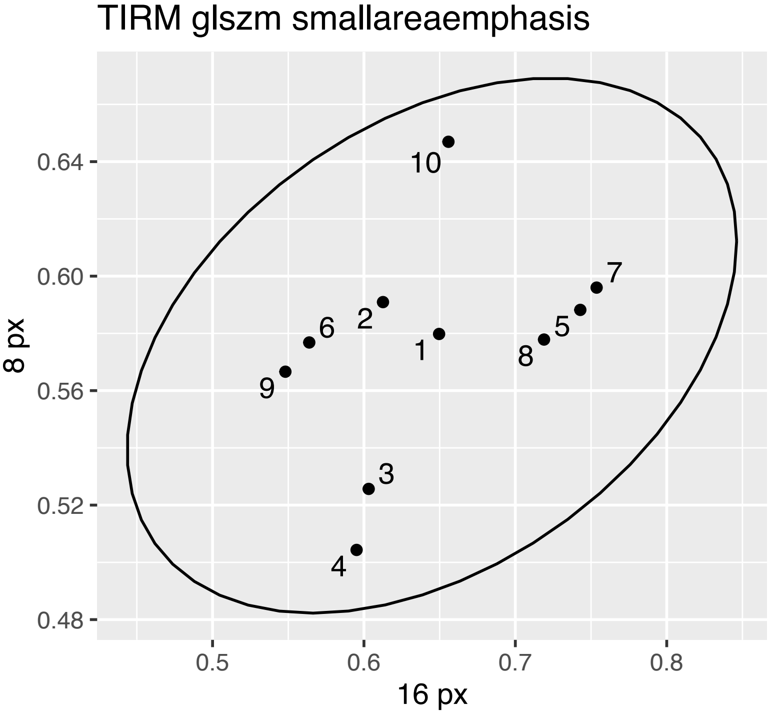
TIRM glszm smallareaemphasis
0.64
0.60
0.56
0.52
0.48
8 px
0.5
0.6
0.7
0.8
16 px
1 2 3 4 5 6 7 8 9 10

TIRM glszm smallareahighgraylevelemphasis

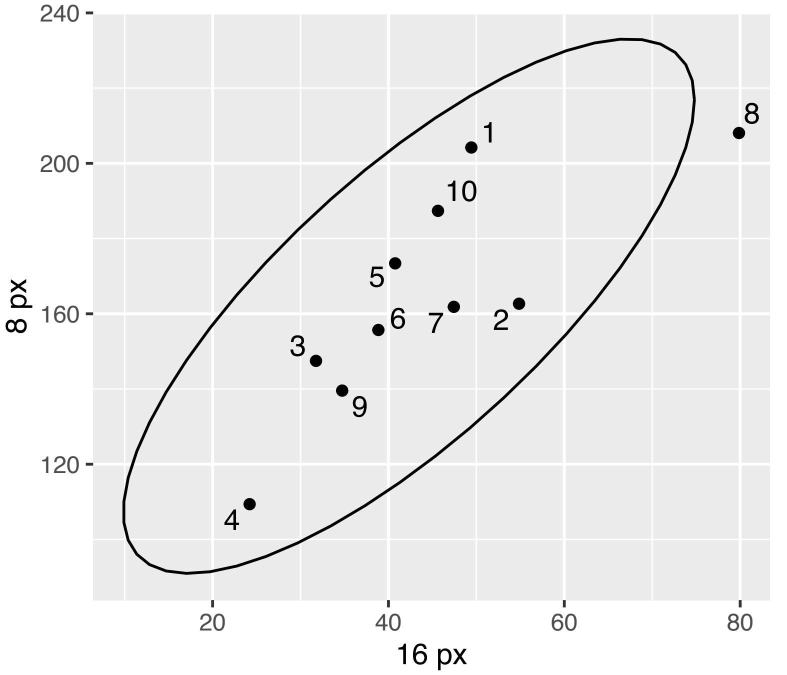

TIRM glszm zonepercentage

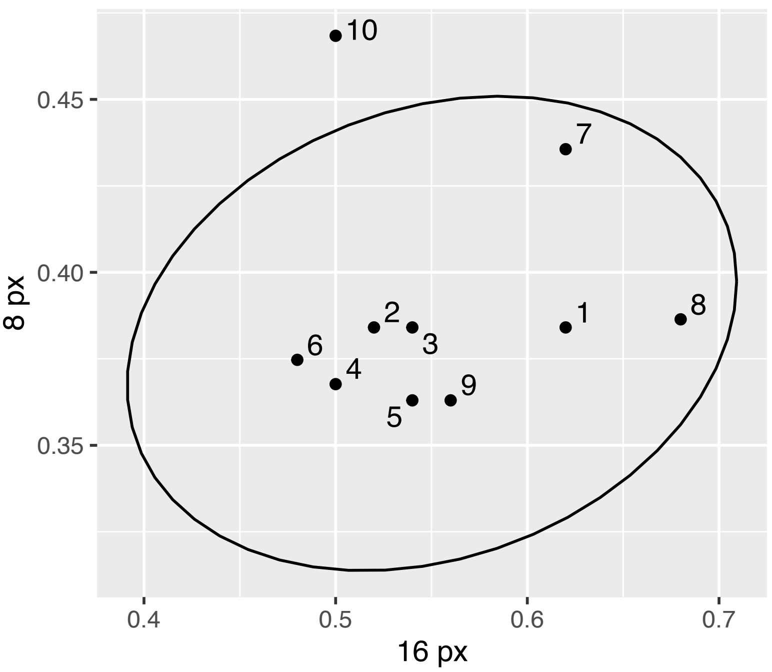

TIRM glszm smallarealowgraylevelemphasis

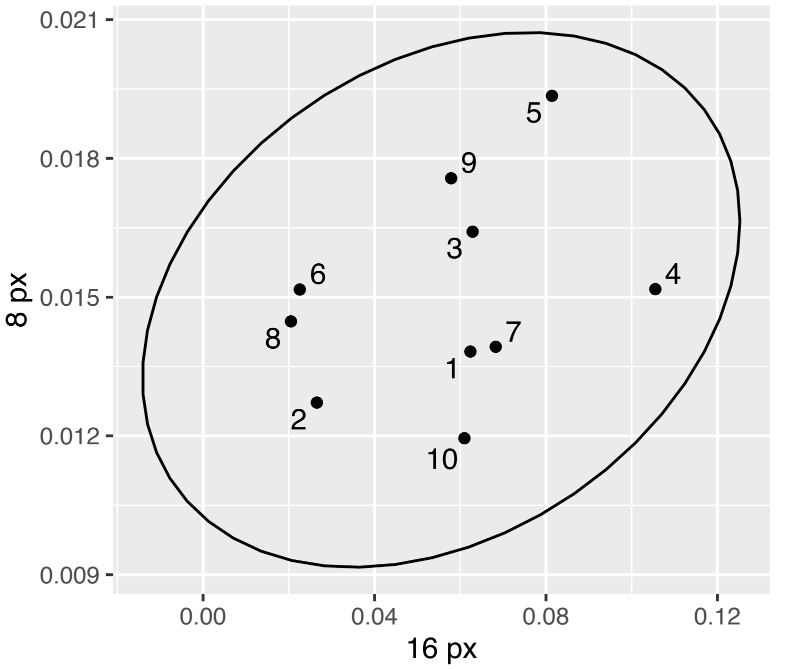

TIRM glszm zonevariance

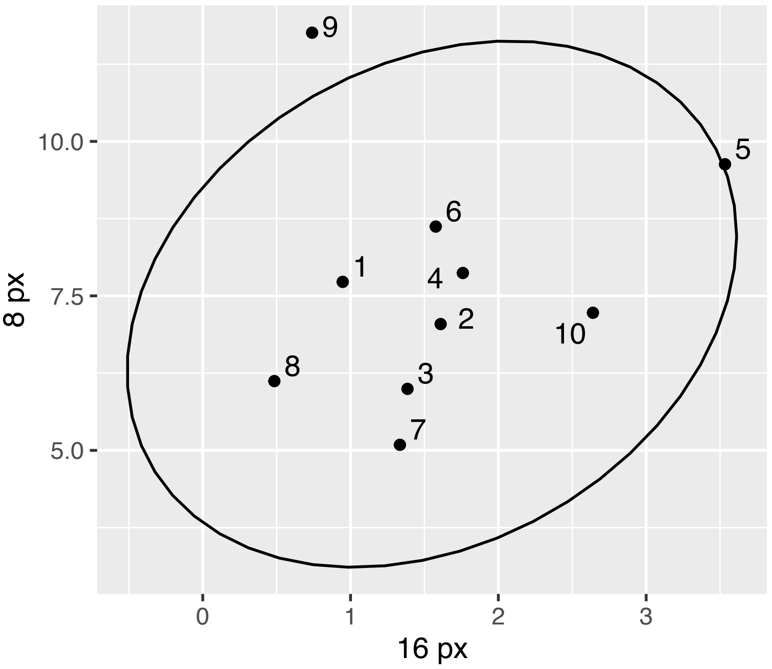

TIRM glszm zoneentropy

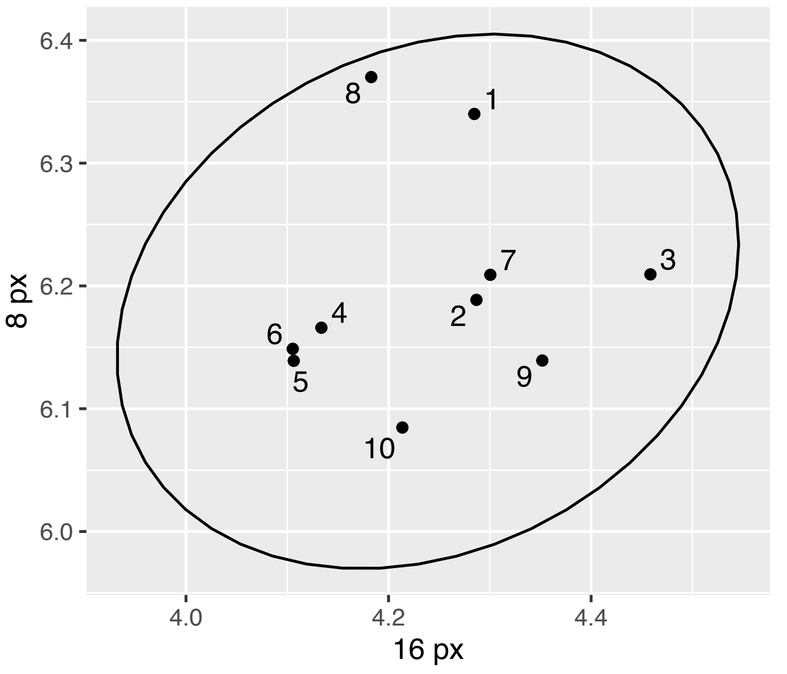

TIRM gldm dependenceentropy

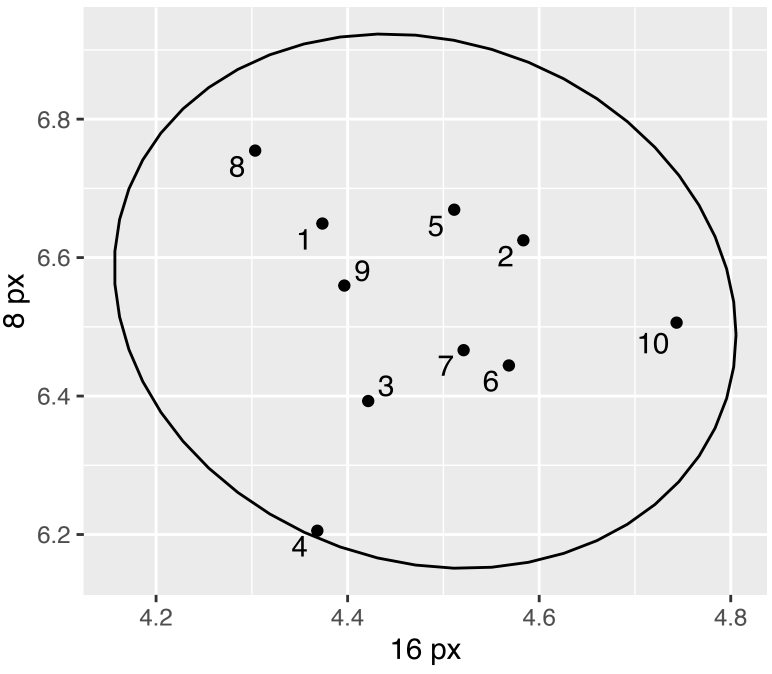

TIRM gldm dependencenonuniformity

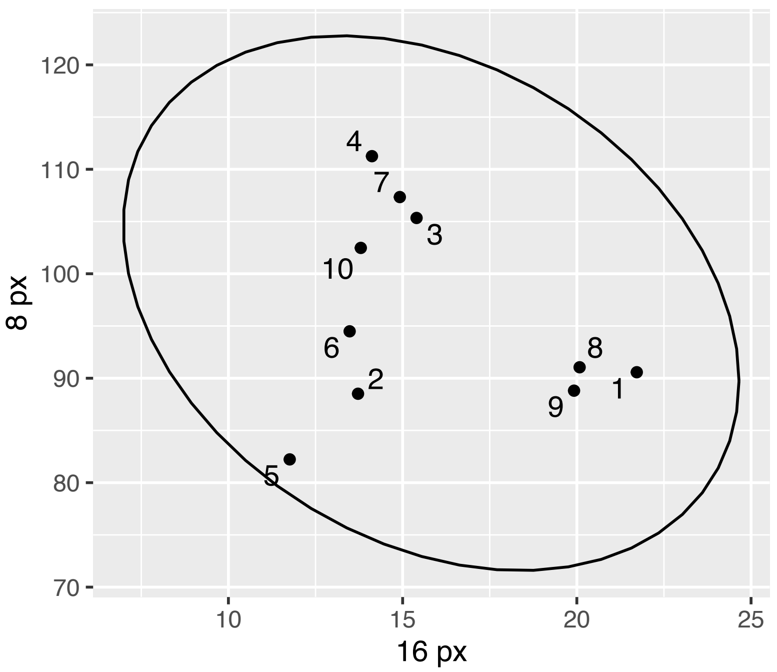

TIRM gldm graylevelnonuniformity

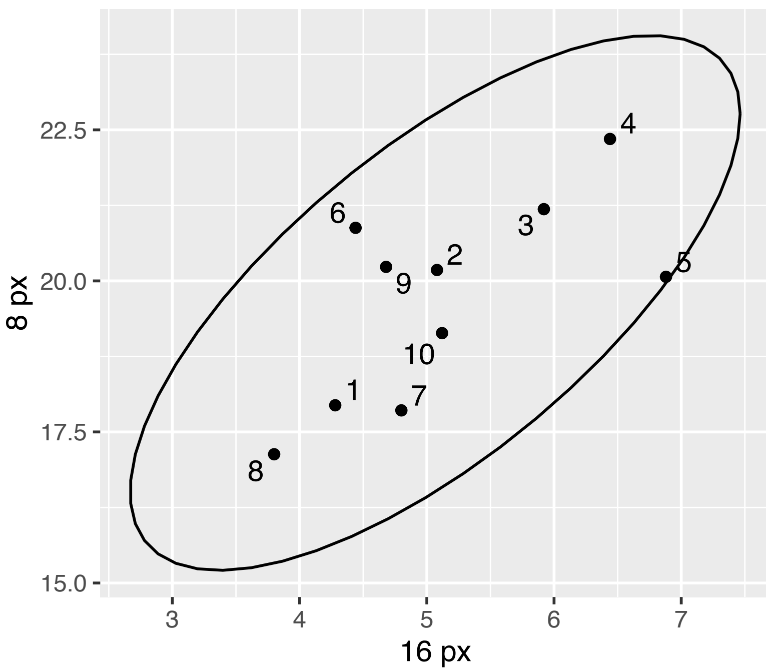

TIRM gldm dependencenonuniformitynormal

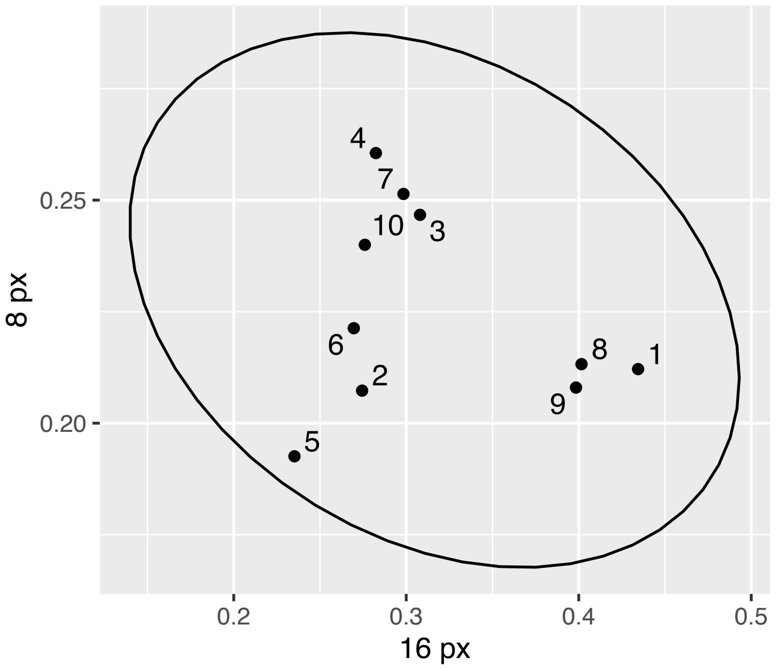

TIRM gldm graylevelvariance

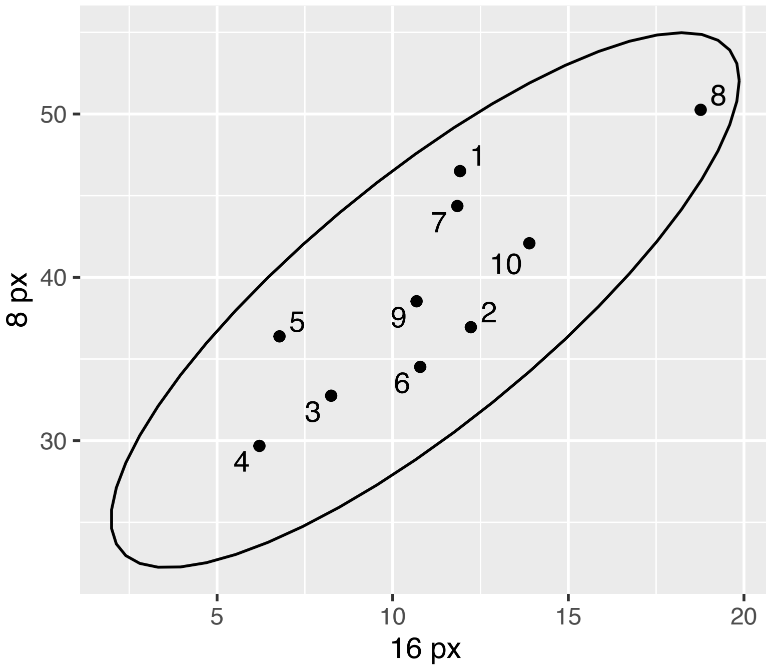

TIRM gldm dependencevariance

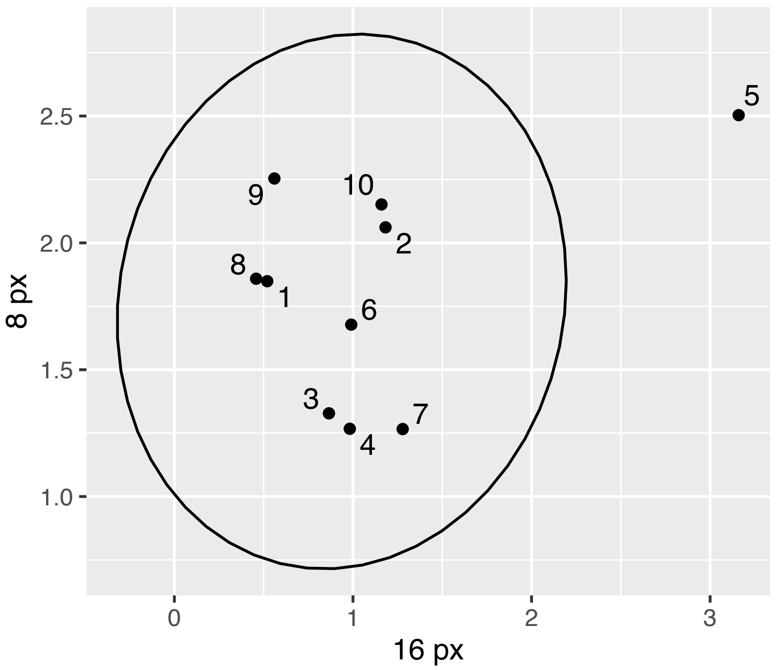

TIRM gldm highgraylevelemphasis

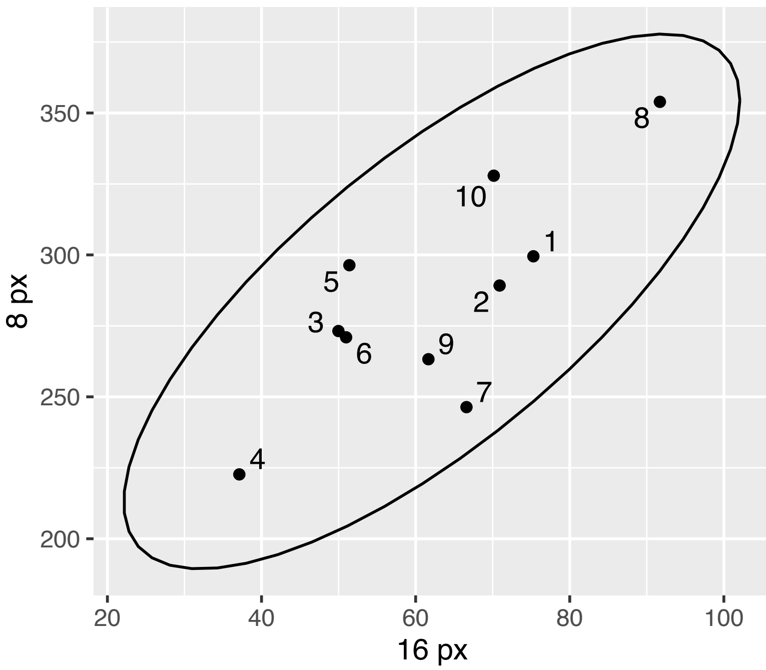

TIRM gldm largedependenceemphasis

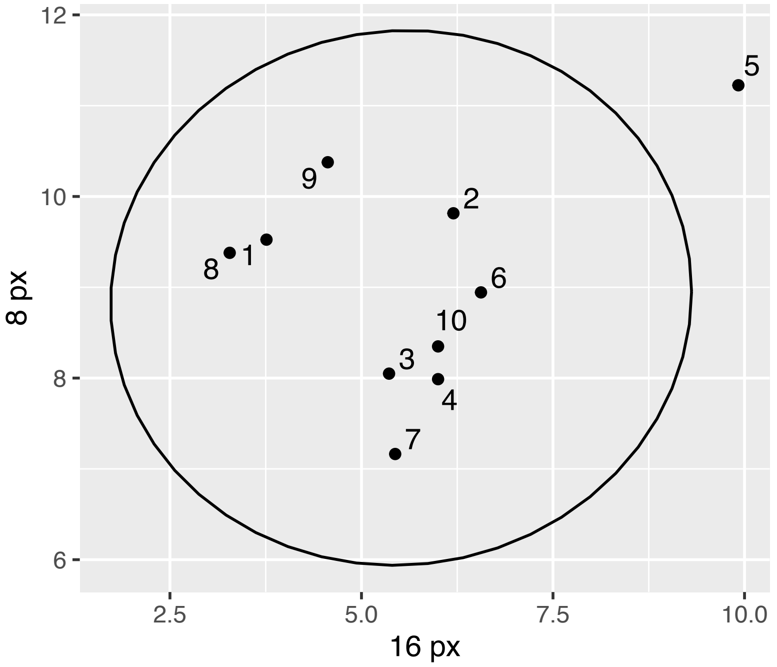

TIRM gldm lowgraylevelemphasis

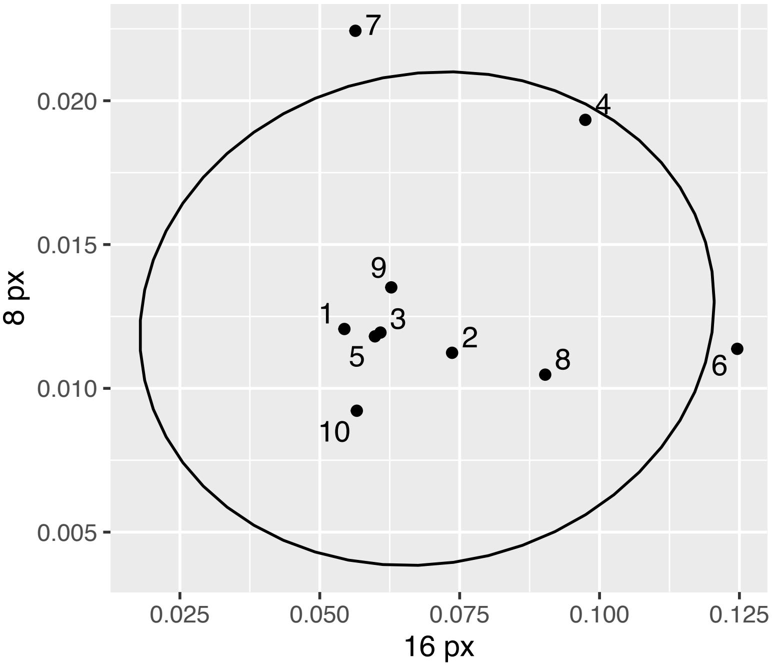

TIRM gldm largedependencehighgrayleveler

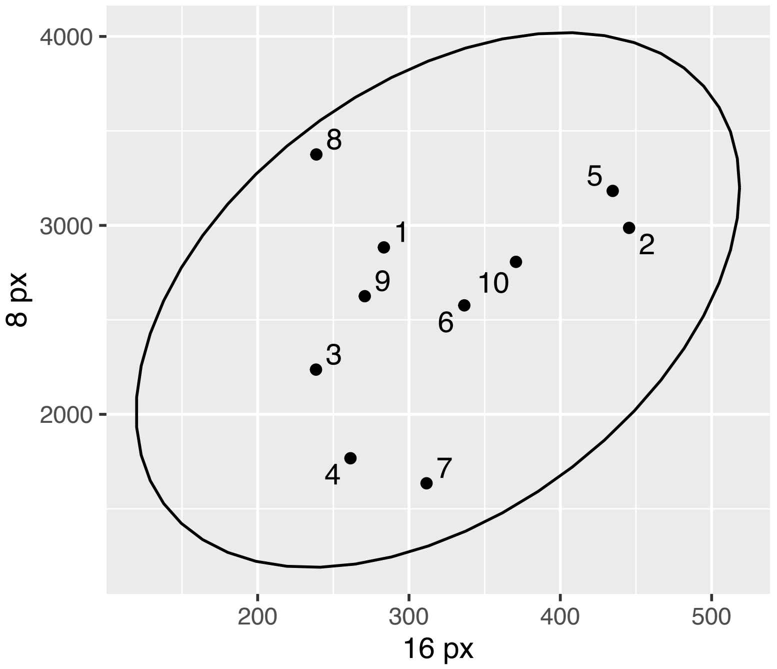

TIRM gldm smalldependenceemphasis

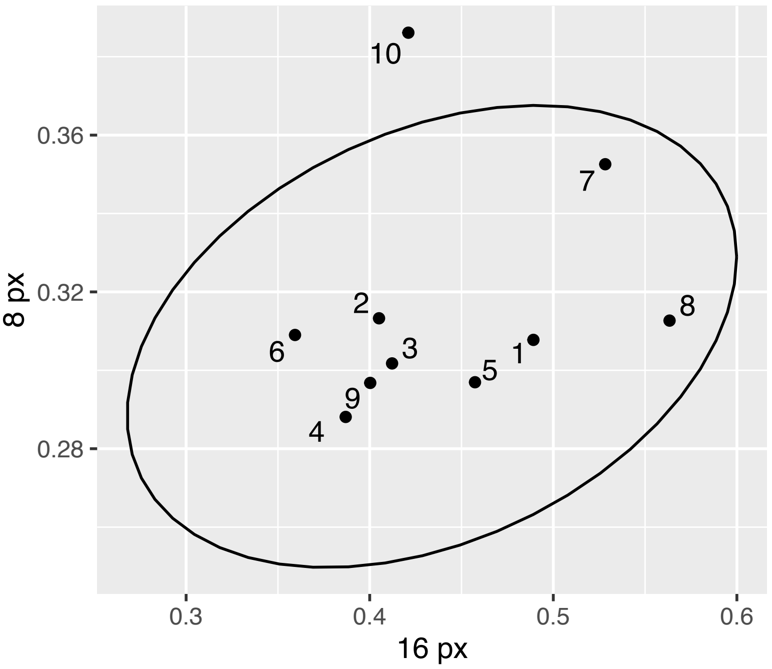

TIRM gldm largedependencelowgraylevelem

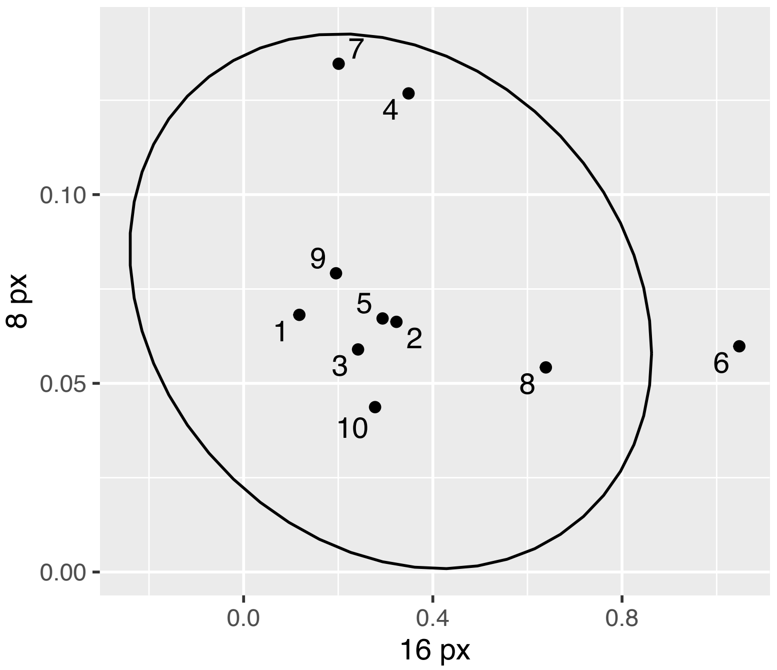

TIRM gldm smalldependencehighgraylevelem

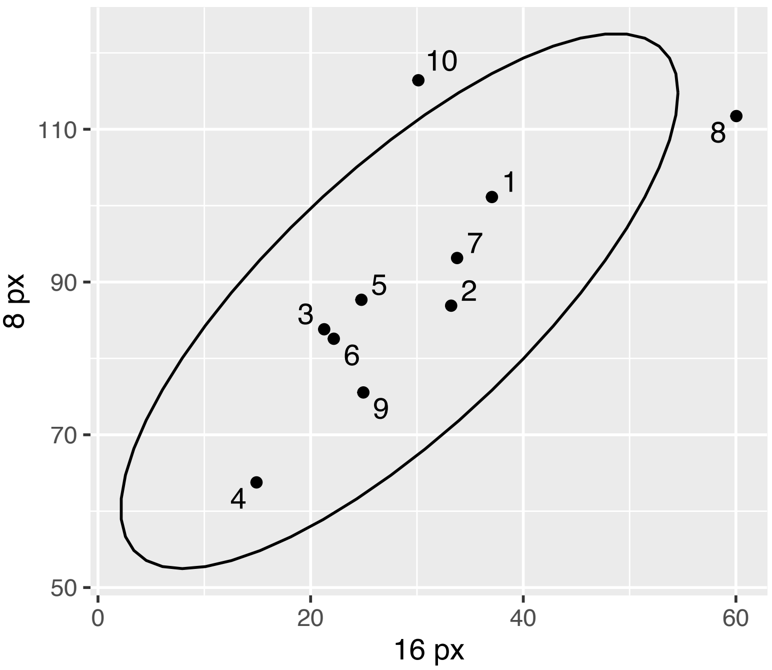

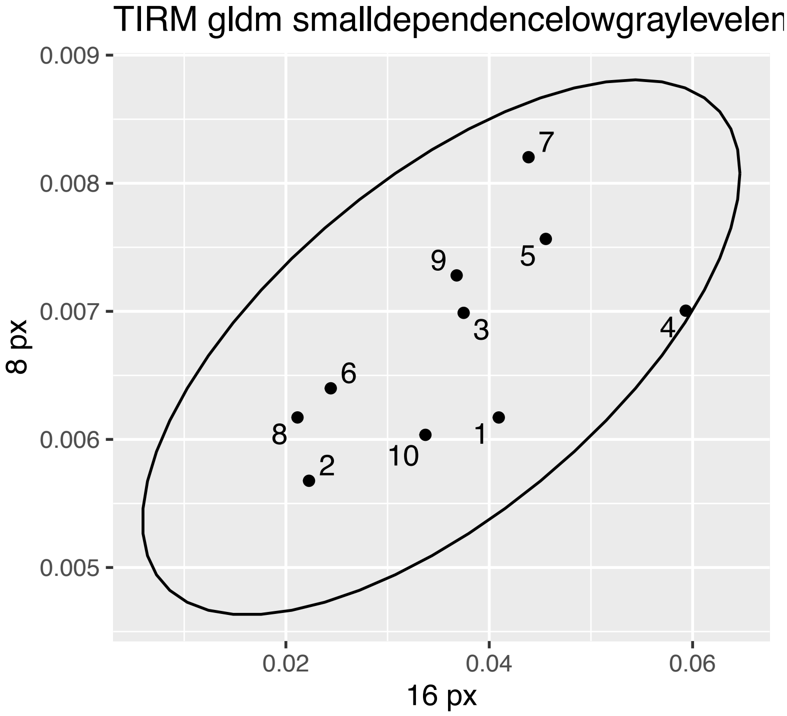
TIRM gldm smalldependencelowgraylevelen
0.009
0.008
0.007
0.006
0.005
0.02
0.04
0.06
8 px
16 px
7
9
5
3
4
6
8
2
10
1

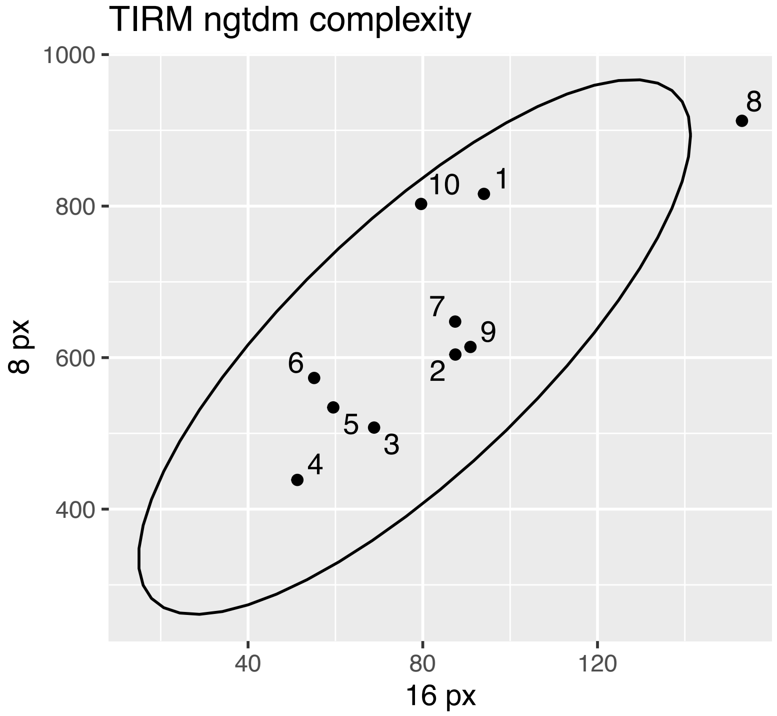
TIRM ngtdm complexity
1000
800
600
400
40
80
120
8 px
16 px
8
10
1
7
9
2
6
5
3
4

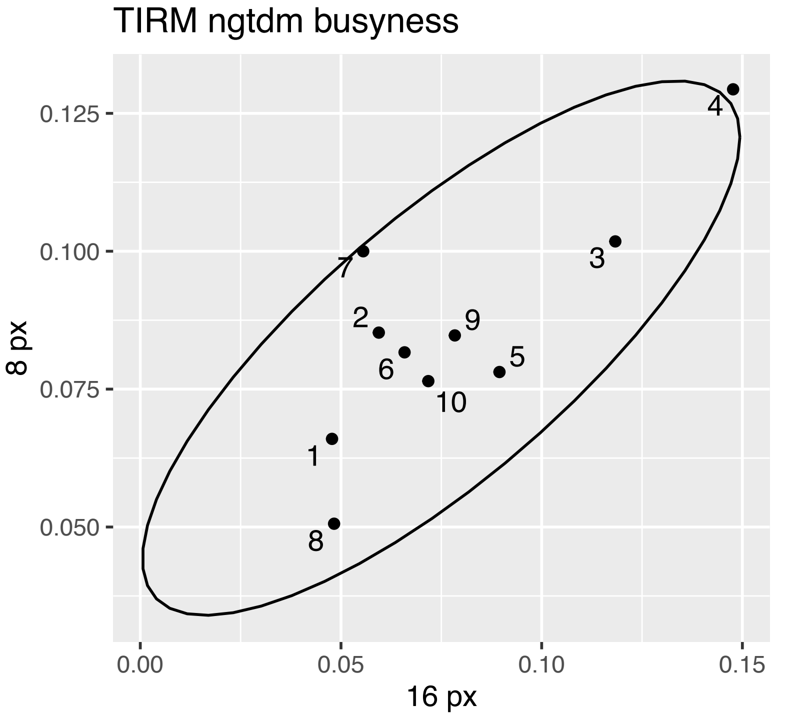
TIRM ngtdm busyness
0.125
0.100
0.075
0.050
0.00
0.05
0.10
0.15
8 px
16 px
4
7
3
2
9
6
5
10
1
8

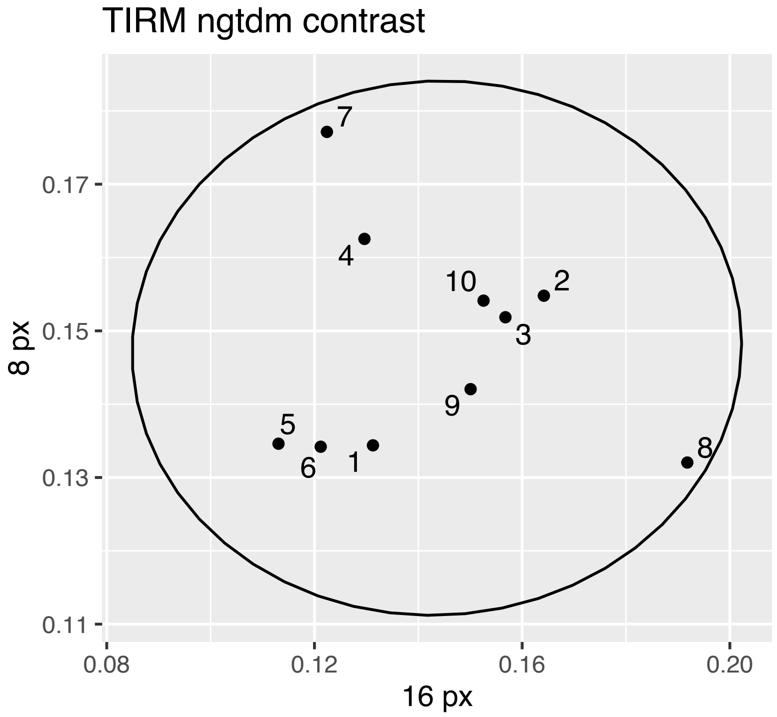
TIRM ngtdm contrast
0.17
0.15
0.13
0.11
0.08
0.12
0.16
0.20
8 px
16 px
7
4
10
2
3
9
5
6
1
8

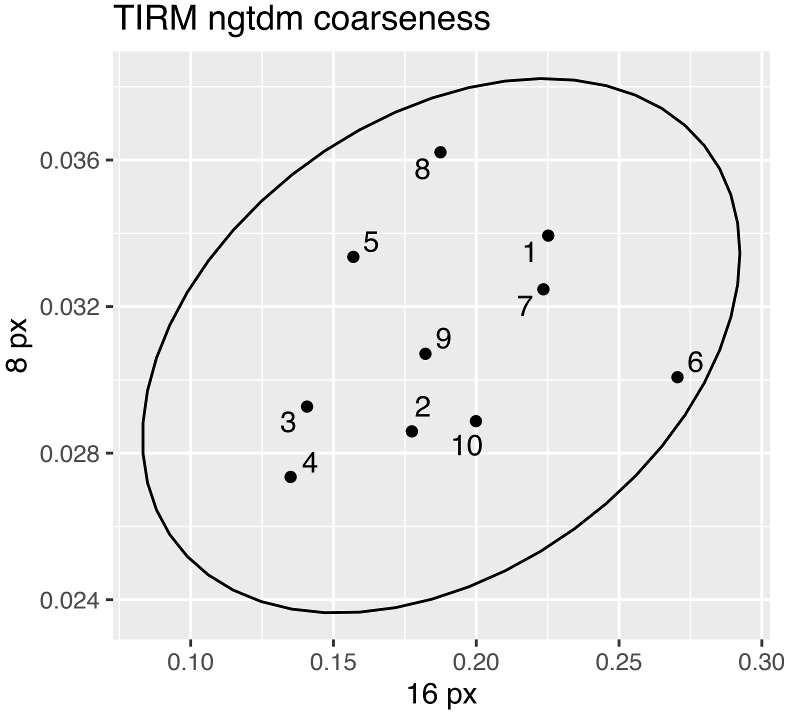
TIRM ngtdm coarseness
0.036
0.032
0.028
0.024
0.10
0.15
0.20
0.25
0.30
8 px
16 px
8
5
1
7
9
6
3
2
10
4

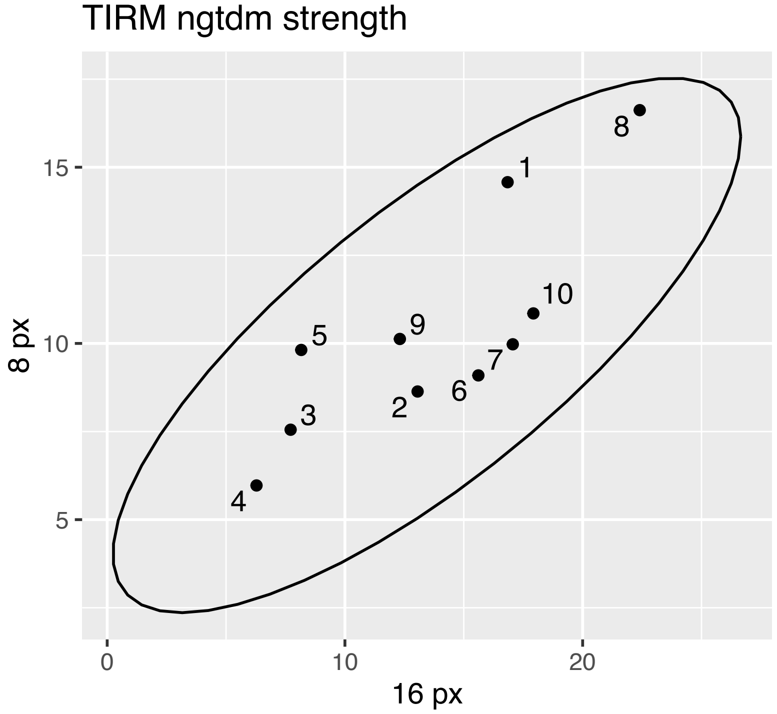
TIRM ngtdm strength
15
10
5
0
10
20
8 px
16 px
8
1
10
5
9
7
2
6
3
4
