# Supplementary material for: Stability of Radiomic Features across Different Region of Interest Sizes—A CT and MR Phantom Study
Source: Tomography. 2021 Jun 8;7(2):238–52. doi: 10.3390/tomography7020022 (PMC8293351; doi:10.3390/tomography7020022)
Supplement: Supplementary file 1 [file tomography-07-00022-s001.zip › figure_S5.pdf]

CT firstorder 10percentile 8,16 mm

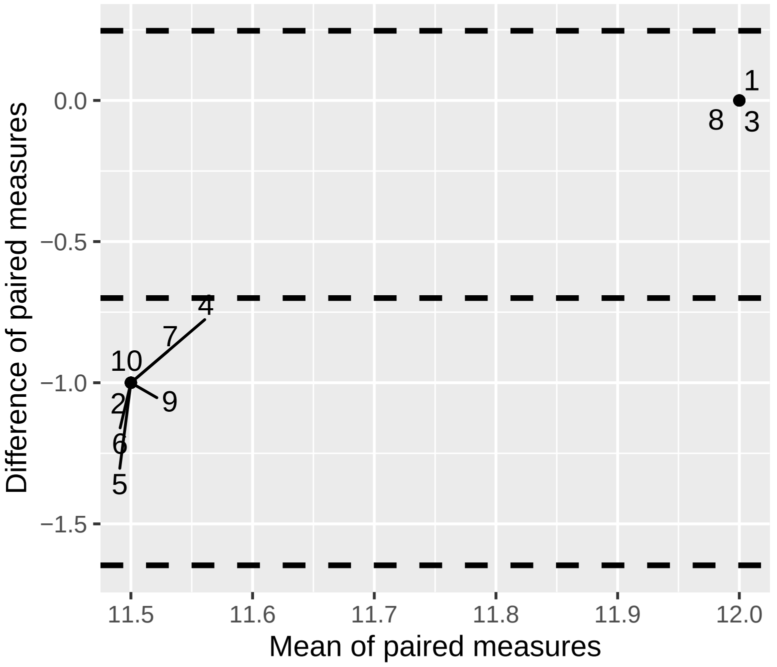


CT firstorder entropy 8,16 mm

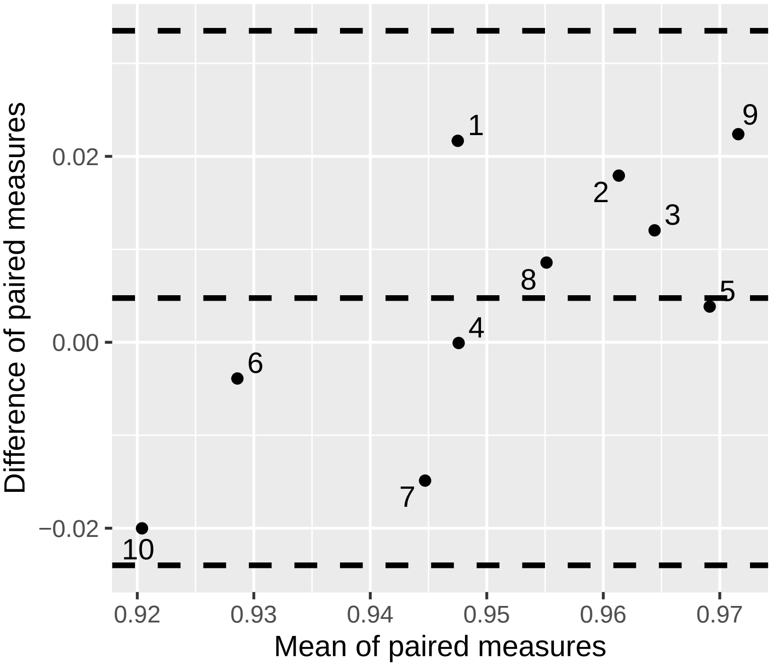


CT firstorder 90percentile 8,16 mm

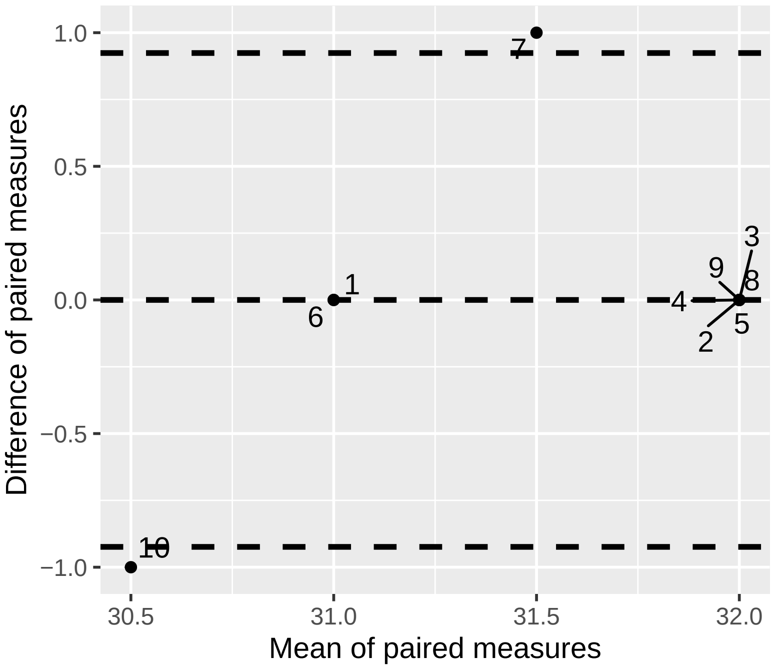


CT firstorder interquartilerange 8,16 mm

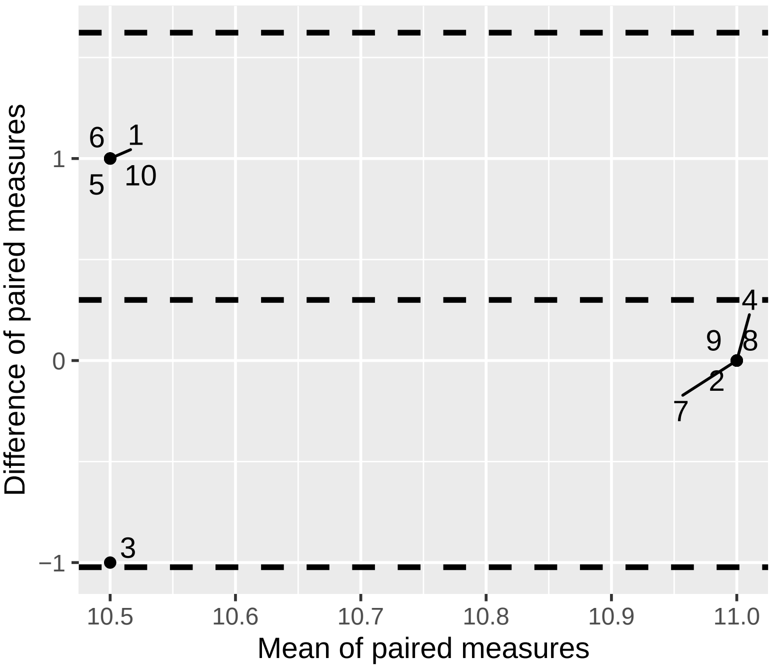


CT firstorder energy 8,16 mm

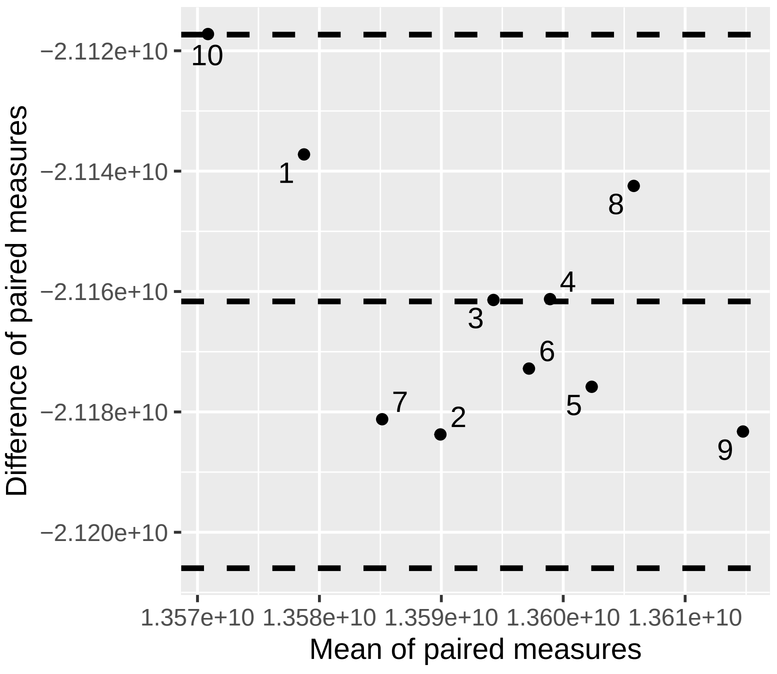


CT firstorder kurtosis 8,16 mm

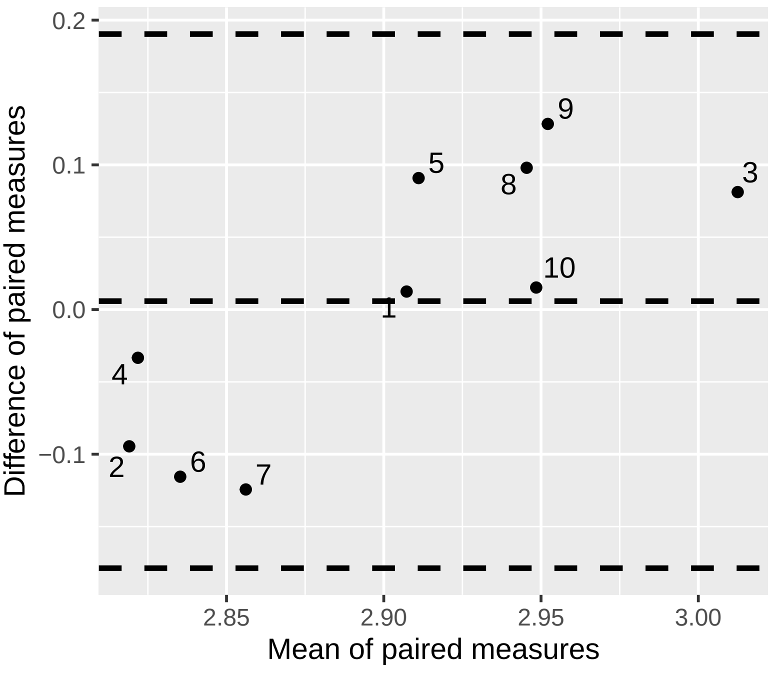

CT firstorder maximum 8,16 mm

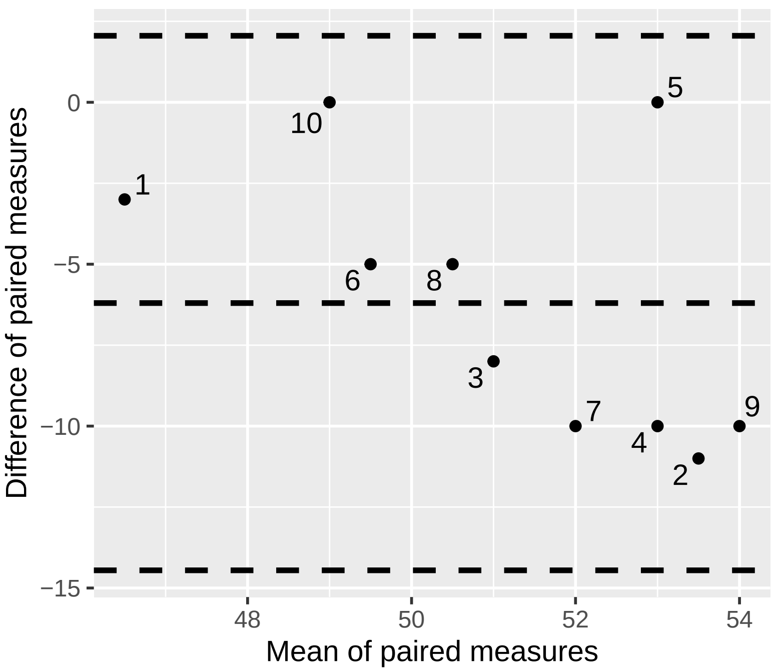


CT firstorder median 8,16 mm

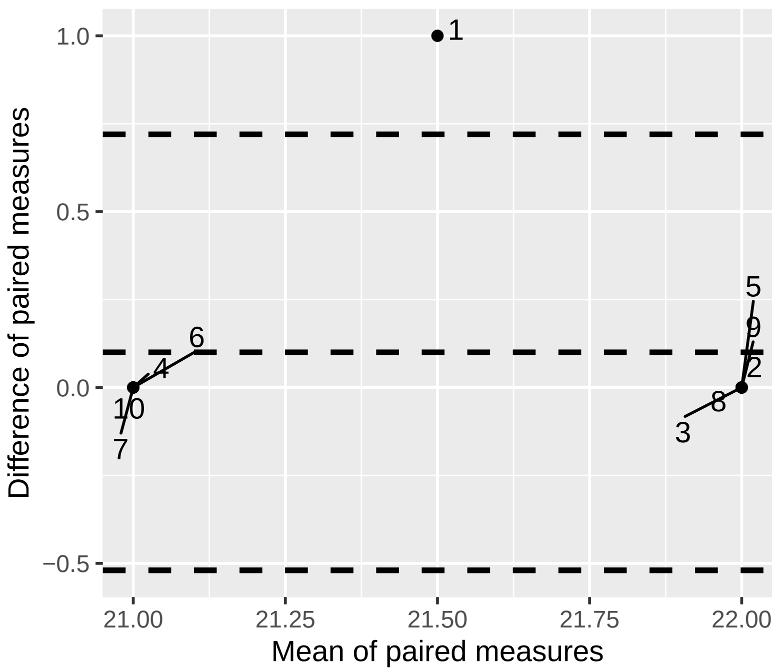


CT firstorder meanabsolutedeviation 8,16 mr

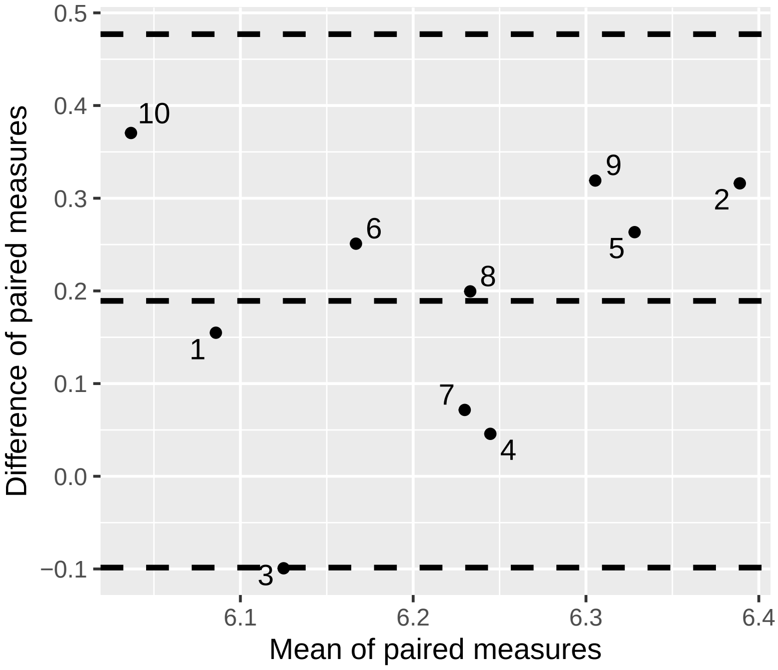


CT firstorder minimum 8,16 mm

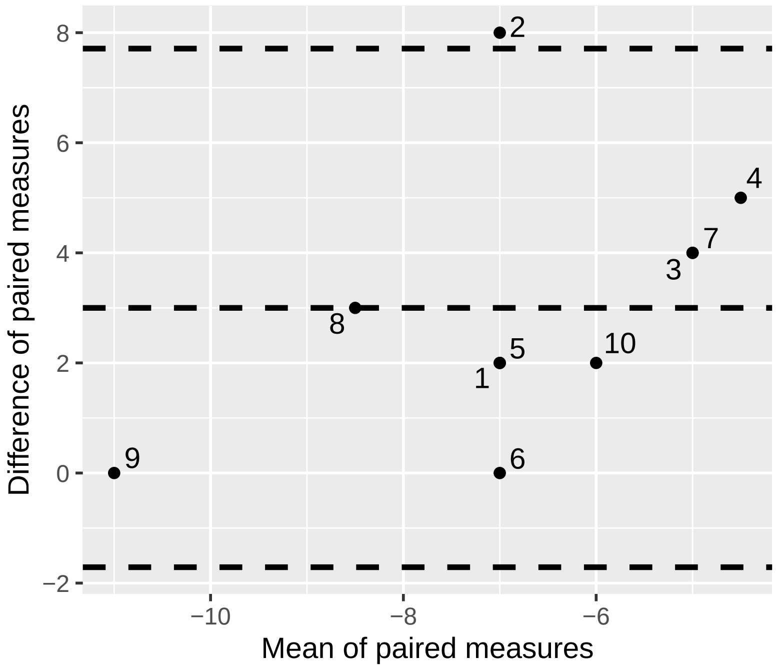


CT firstorder mean 8,16 mm

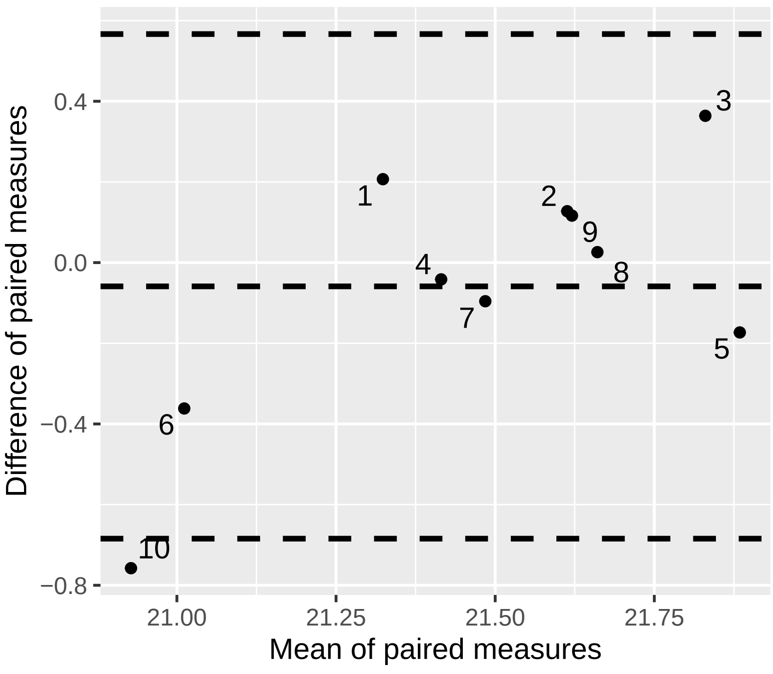


CT firstorder range 8,16 mm

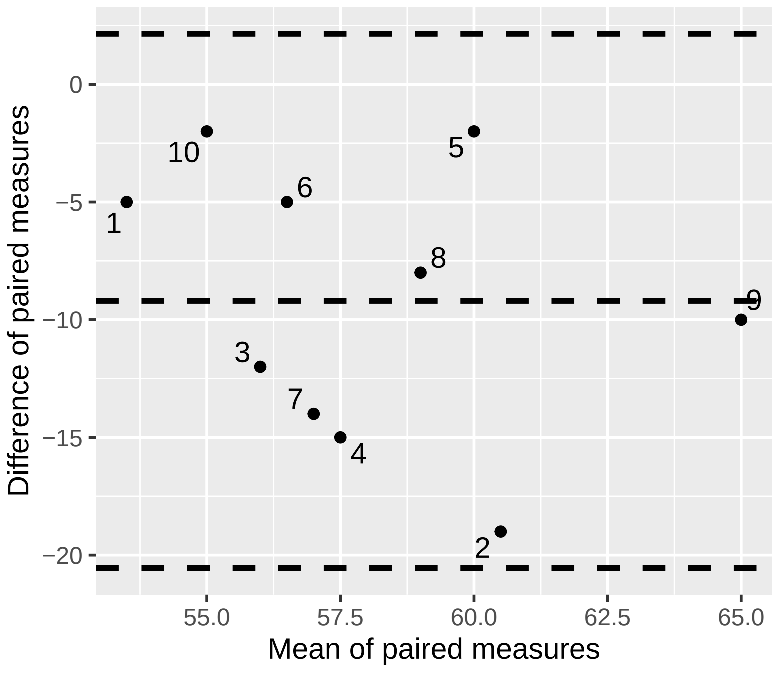

CT firstorder robustmeanabsolutedeviation 8

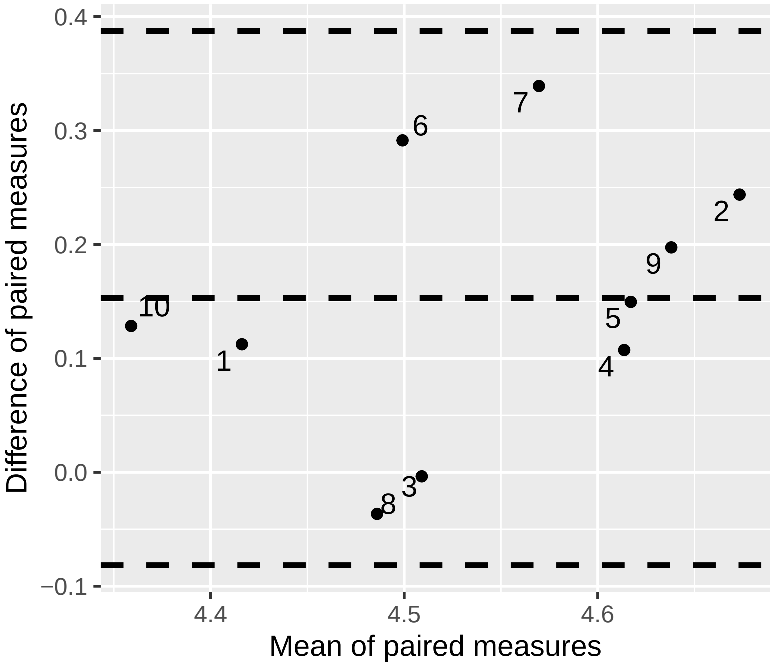


CT firstorder totalenergy 8,16 mm

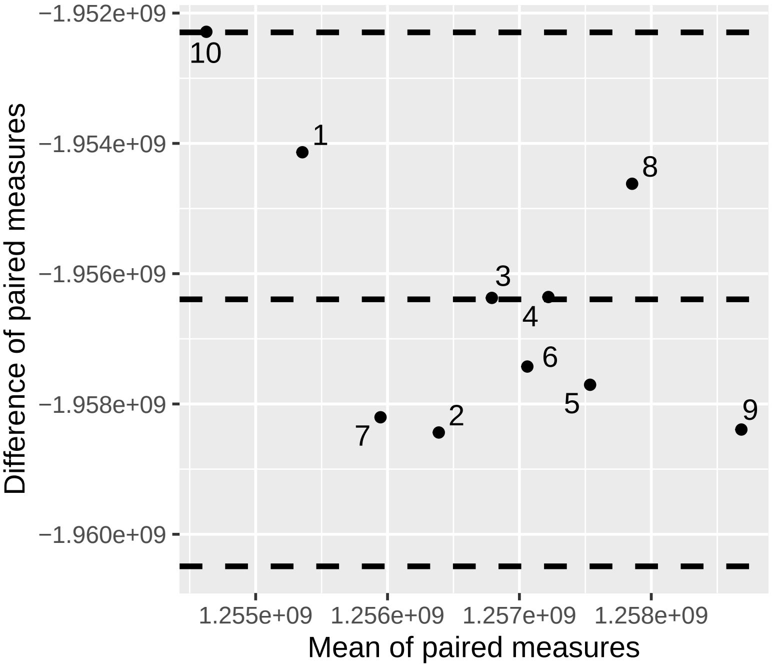


CT firstorder rootmeansquared 8,16 mm

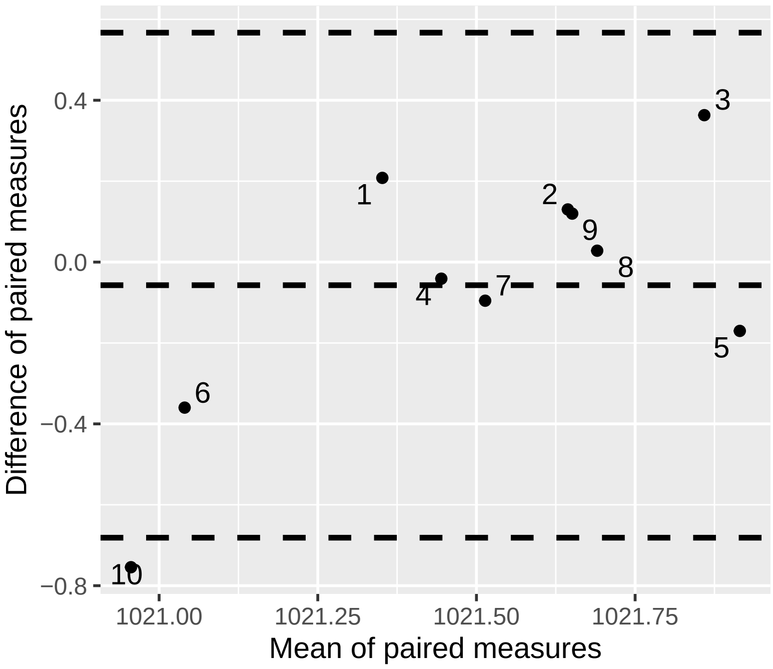


CT firstorder uniformity 8,16 mm

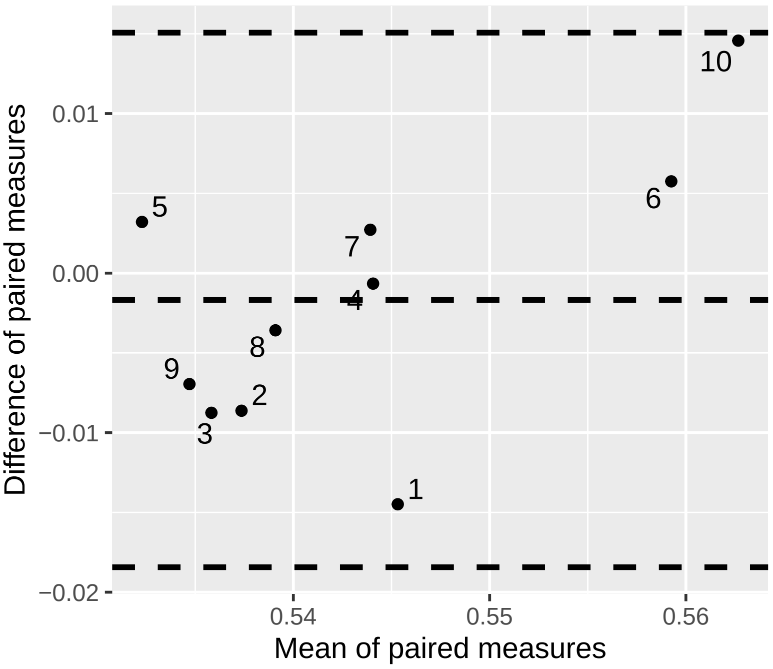


CT firstorder skewness 8,16 mm

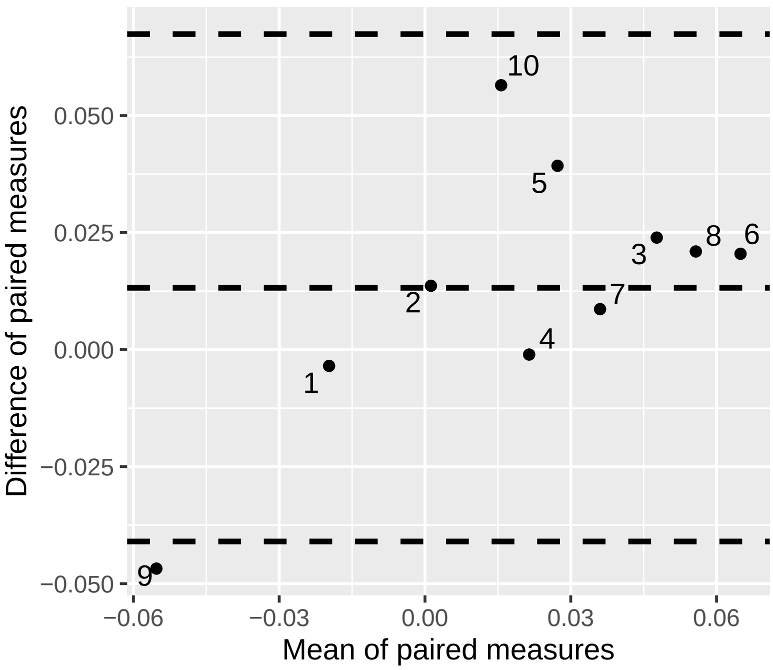


CT firstorder variance 8,16 mm

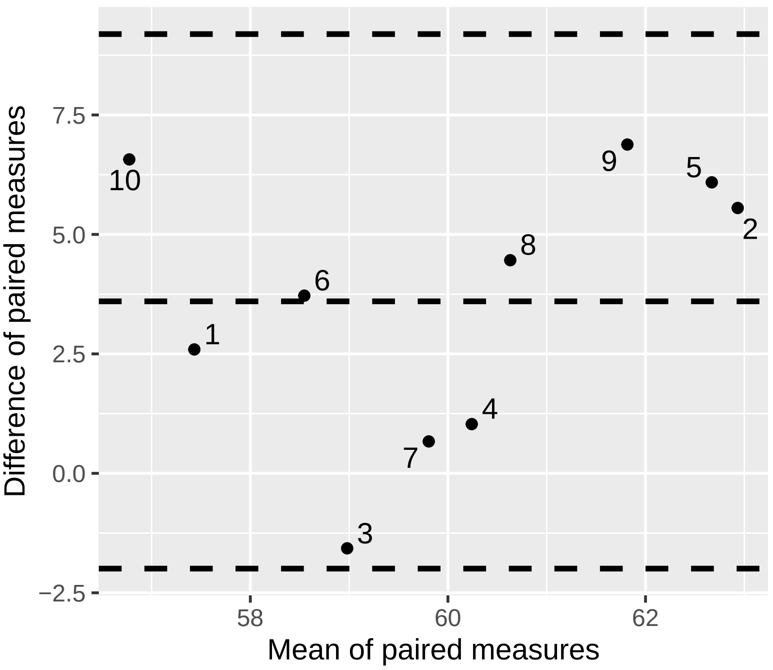

CT glcm autocorrelation 8,16 mm

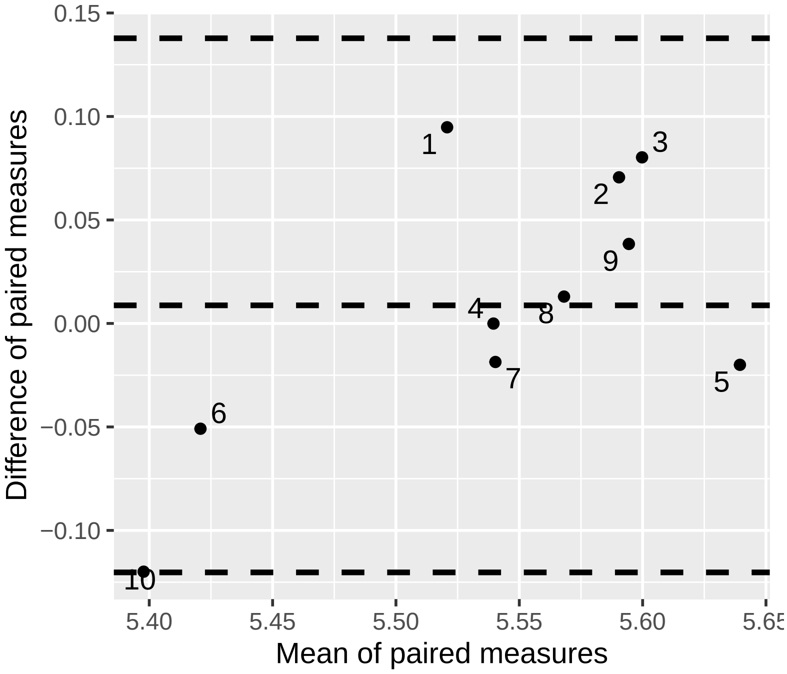


CT glcm clustertendency 8,16 mm

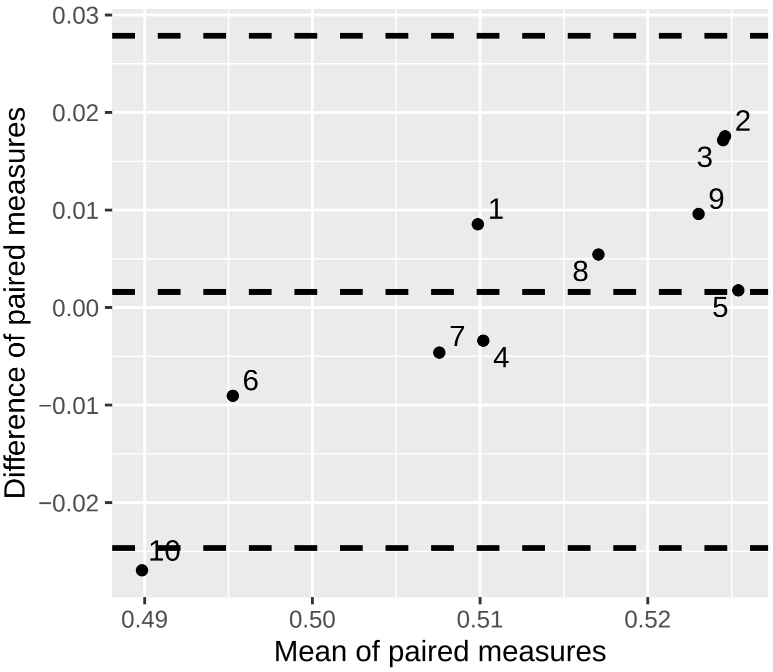


CT glcm clusterprominence 8,16 mm

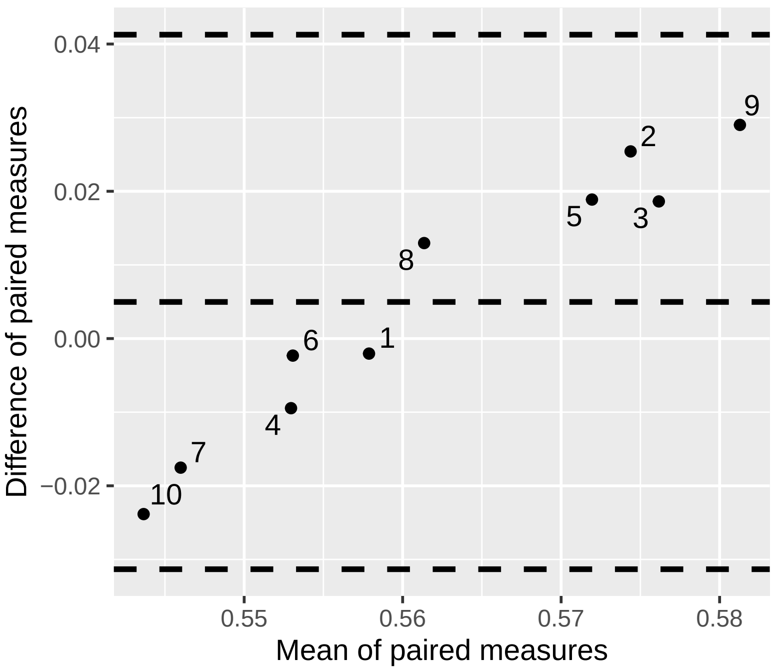


CT glcm contrast 8,16 mm

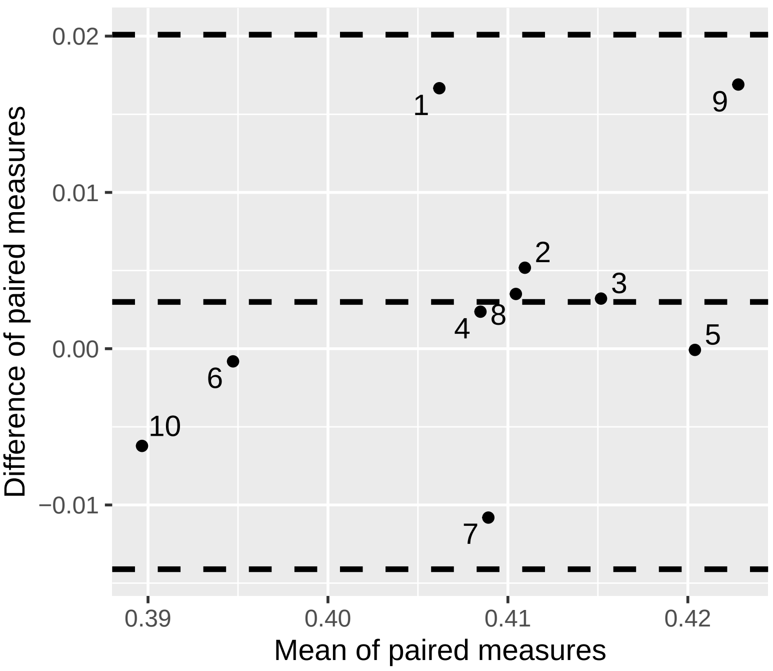


CT glcm clustershade 8,16 mm

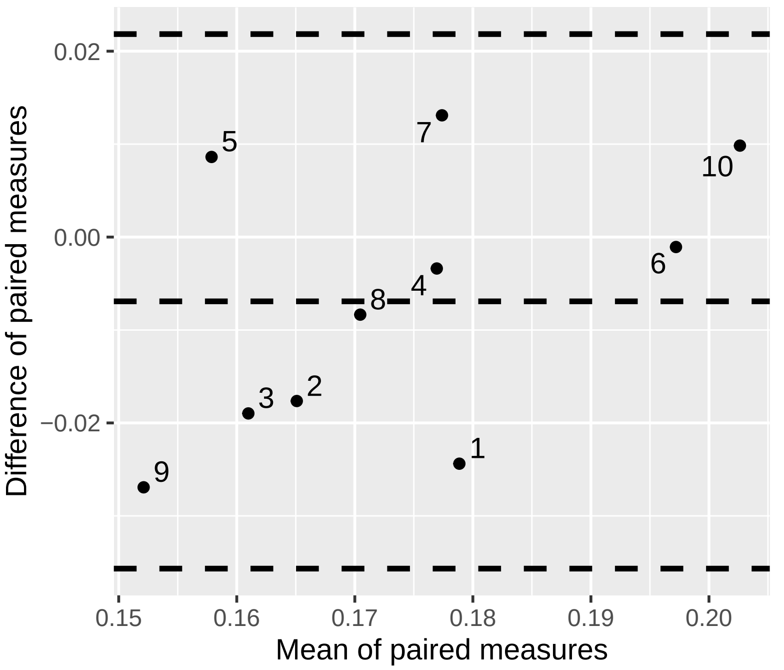


CT glcm correlation 8,16 mm

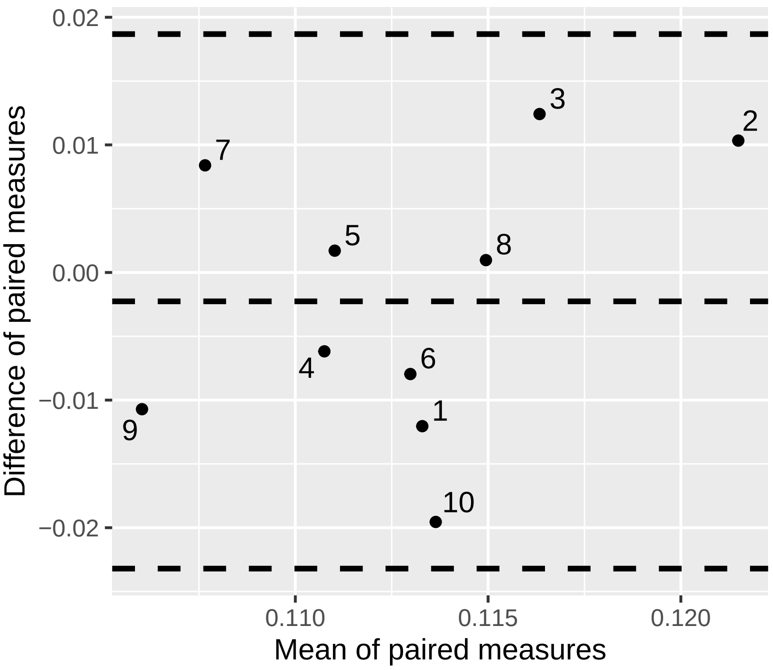

CT glcm differenceaverage 8,16 mm

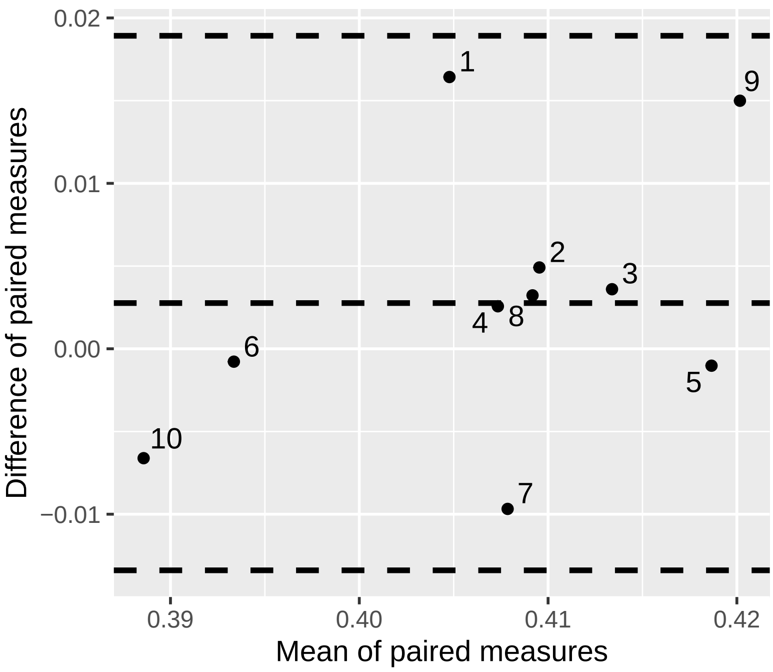


CT glcm id 8,16 mm

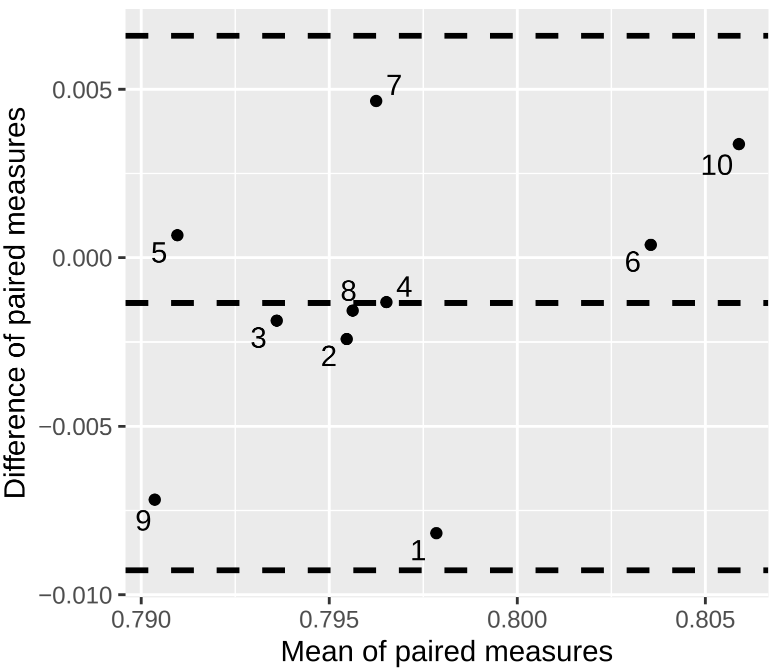


CT glcm differenceentropy 8,16 mm

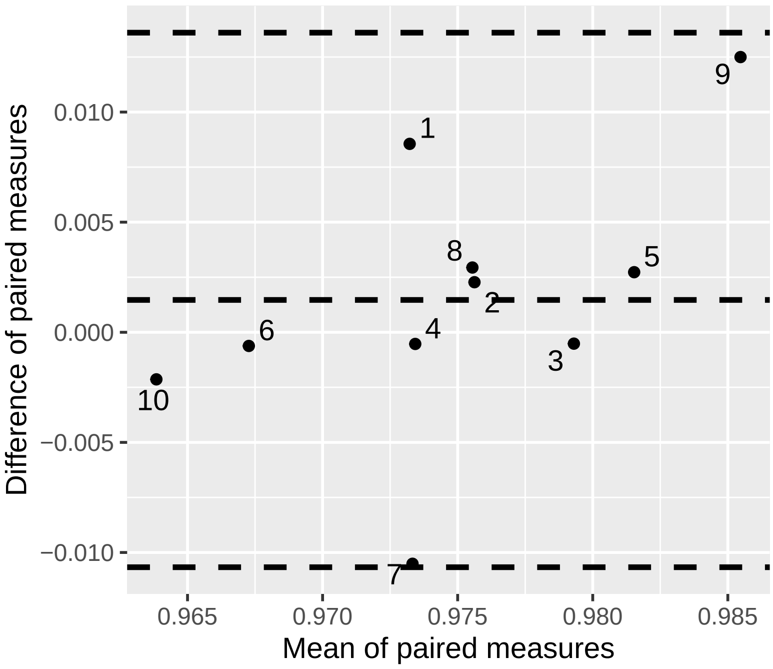


CT glcm idm 8,16 mm

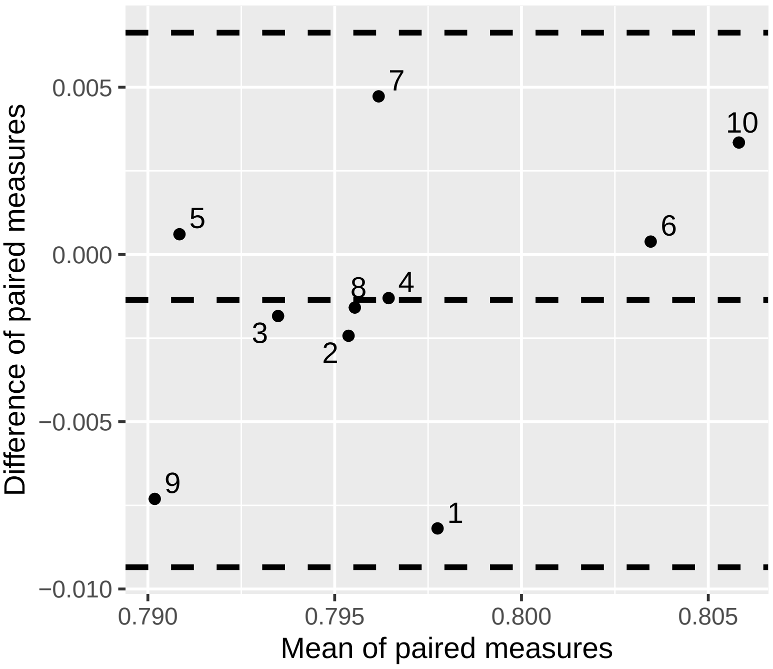


CT glcm differencevariance 8,16 mm

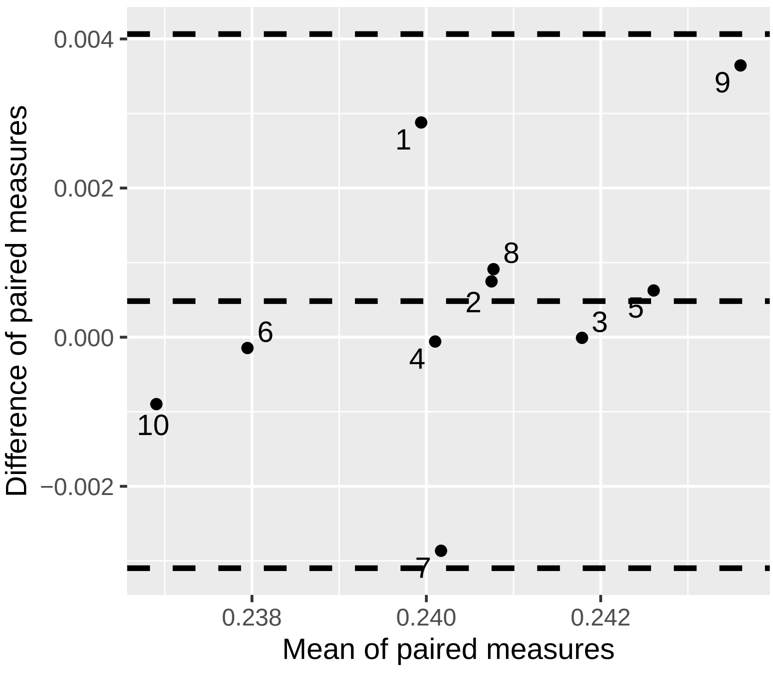


CT glcm idmn 8,16 mm

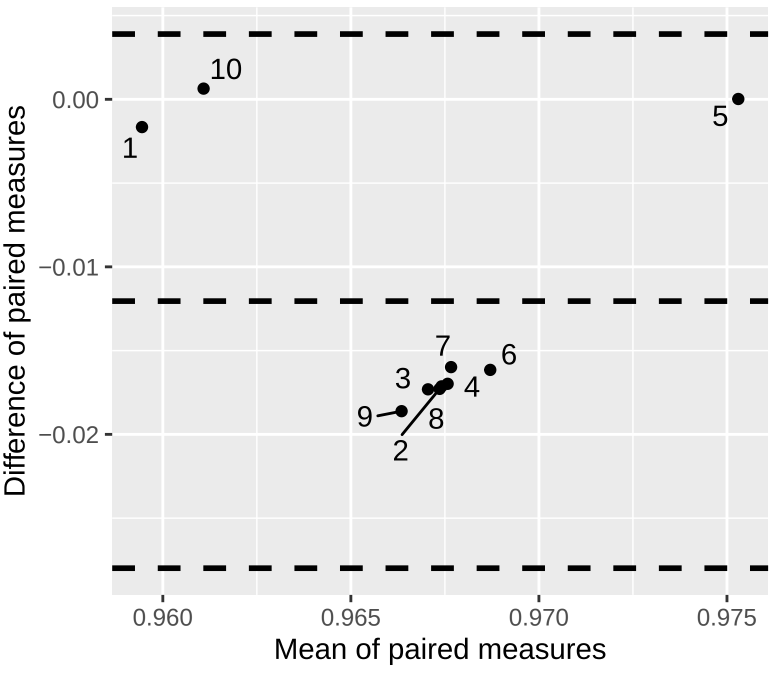

CT glcm idn 8,16 mm

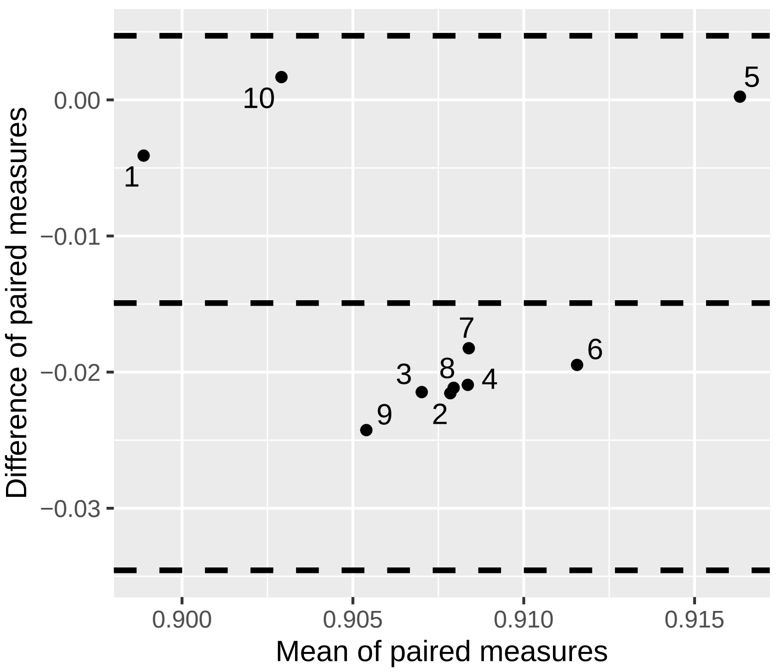


CT glcm inversevariance 8,16 mm

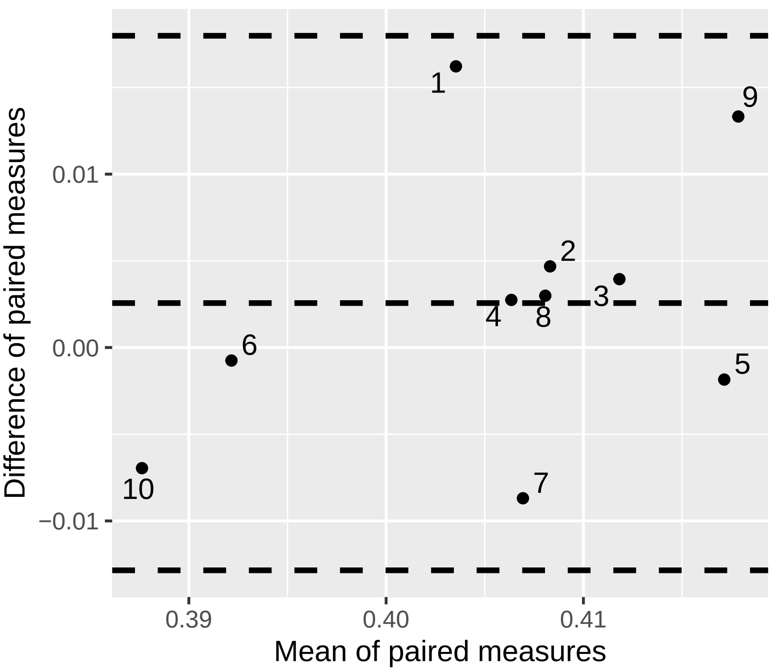


CT glcm imc1 8,16 mm

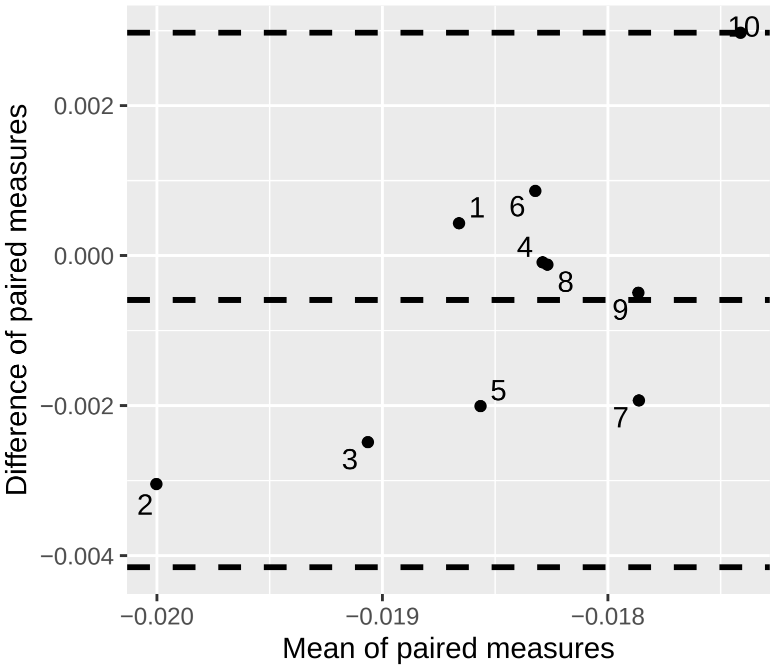


CT glcm jointaverage 8,16 mm

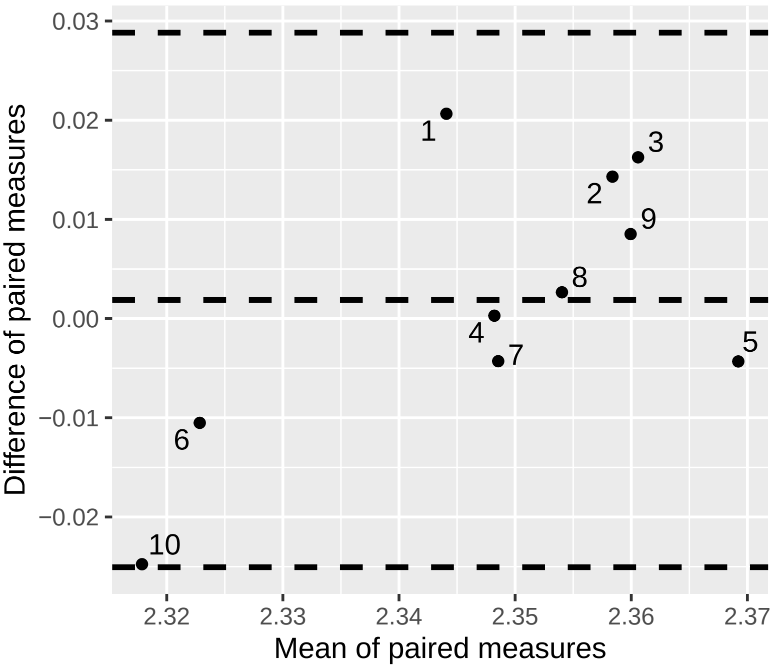


CT glcm imc2 8,16 mm

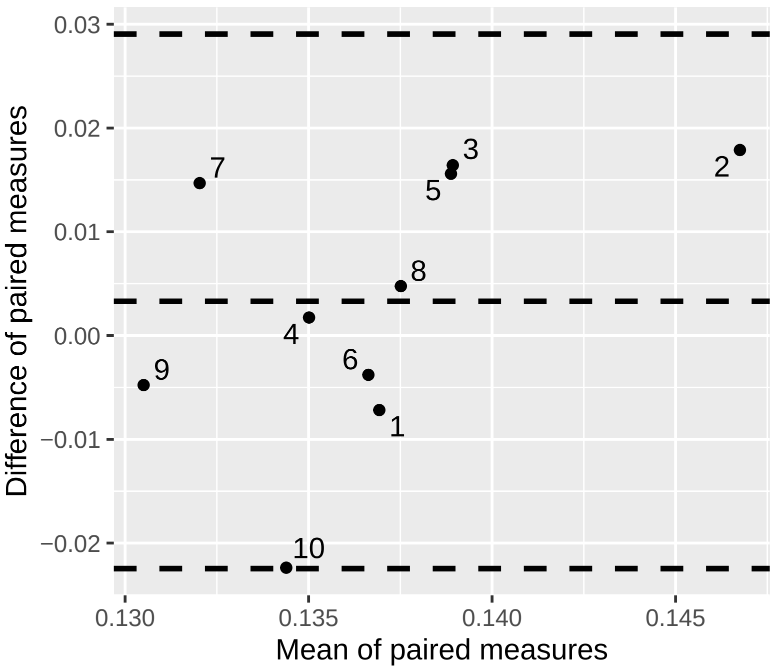


CT glcm jointenergy 8,16 mm

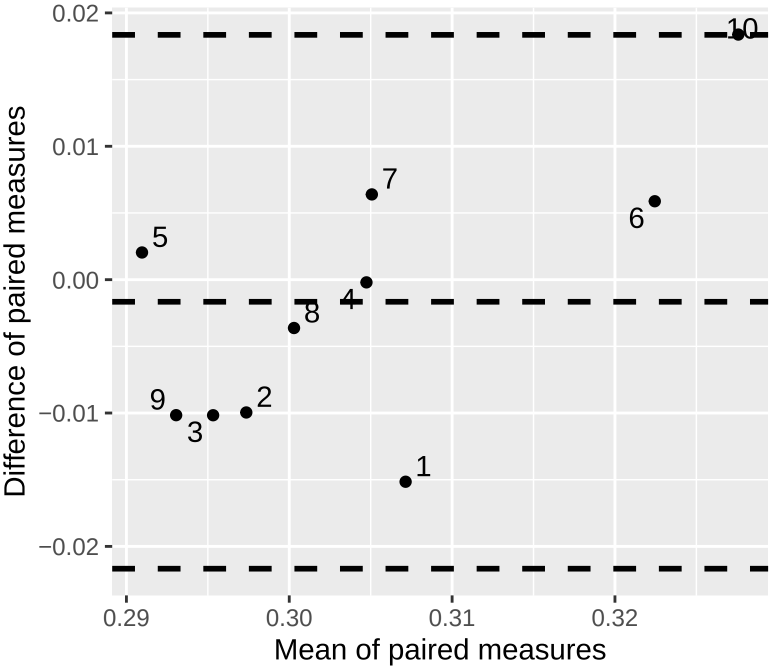

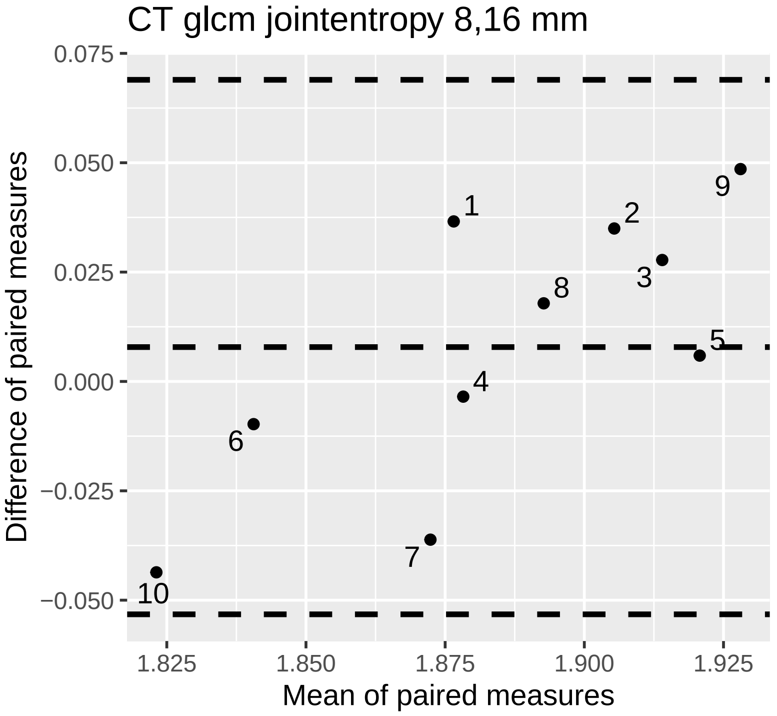
CT glcm jointentropy 8,16 mm
Difference of paired measures
Mean of paired measures
0.075
0.050
0.025
0.000
−0.025
−0.050
1.825
1.850
1.875
1.900
1.925

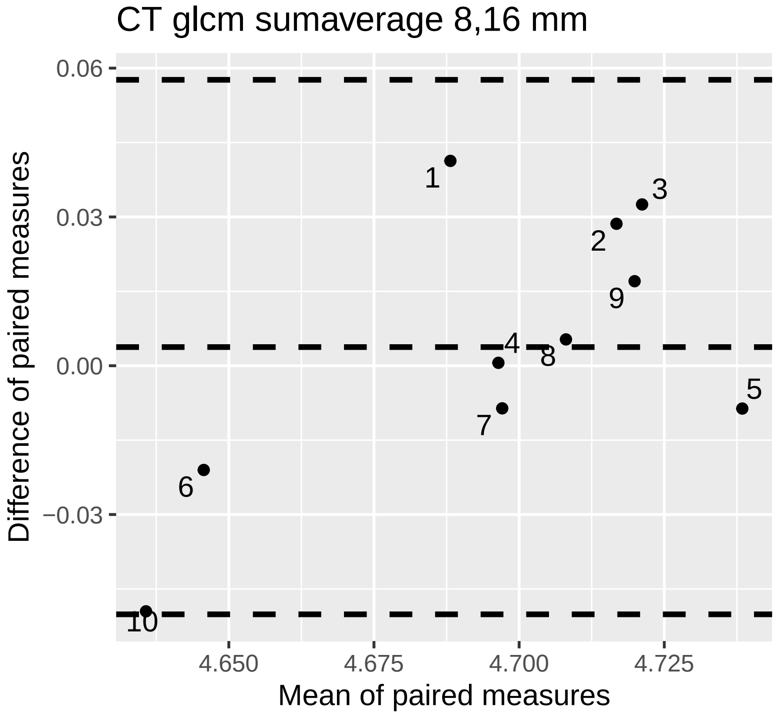
CT glcm sumaverage 8,16 mm
Difference of paired measures
Mean of paired measures
0.06
0.03
0.00
−0.03
4.650
4.675
4.700
4.725

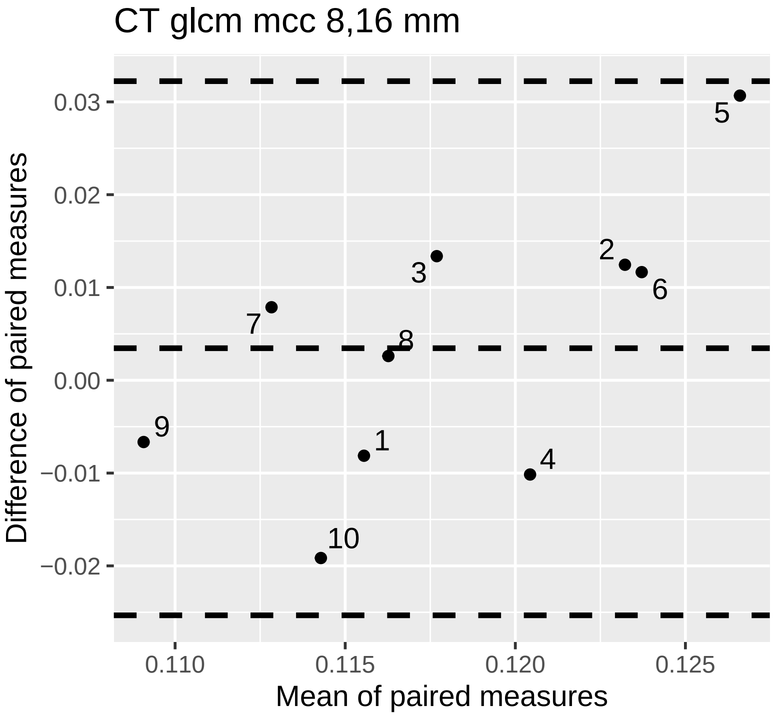
CT glcm mcc 8,16 mm
Difference of paired measures
Mean of paired measures
0.03
0.02
0.01
0.00
−0.01
−0.02
0.110
0.115
0.120
0.125

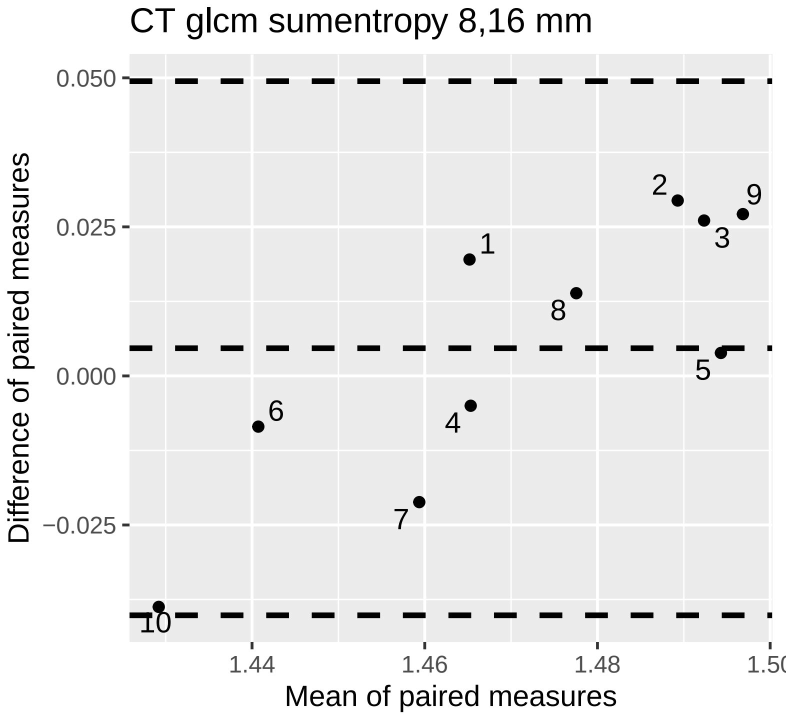
CT glcm sumentropy 8,16 mm
Difference of paired measures
Mean of paired measures
0.050
0.025
0.000
−0.025
1.44
1.46
1.48

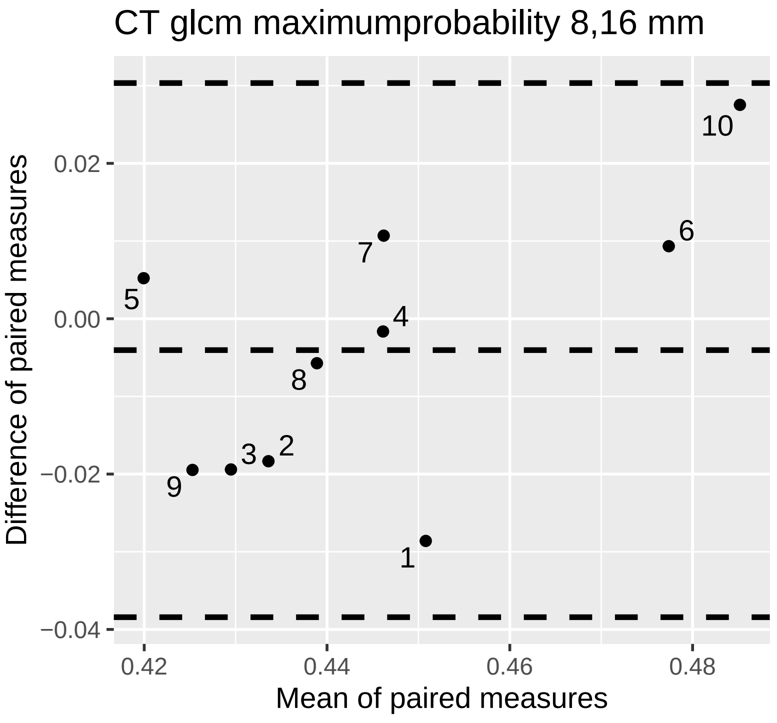
CT glcm maximumprobability 8,16 mm
Difference of paired measures
Mean of paired measures
0.02
0.00
−0.02
−0.04
0.42
0.44
0.46
0.48

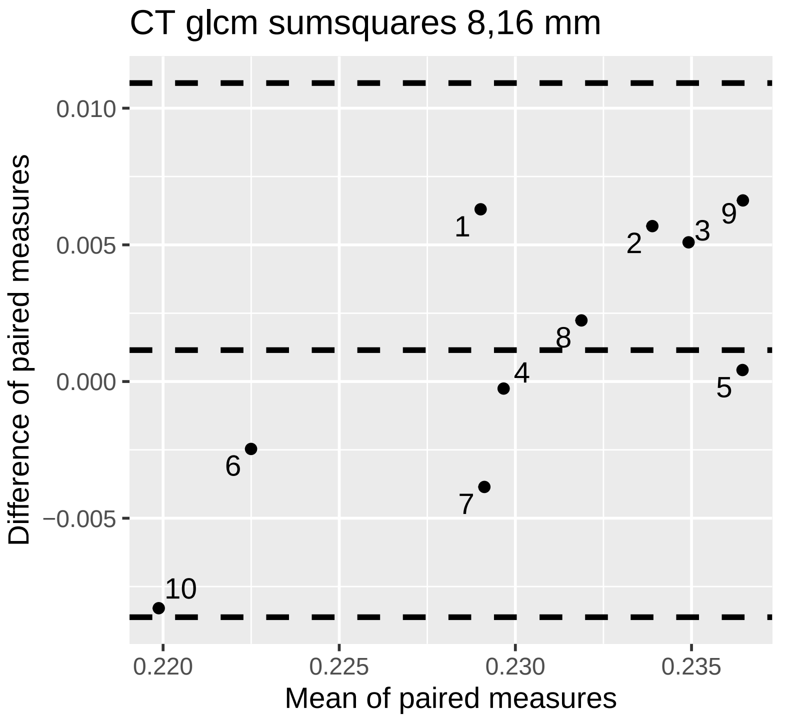
CT glcm sumsquares 8,16 mm
Difference of paired measures
Mean of paired measures
0.010
0.005
0.000
−0.005
0.220
0.225
0.230
0.235

CT glrlm graylevelnonuniformity 8,16 mm

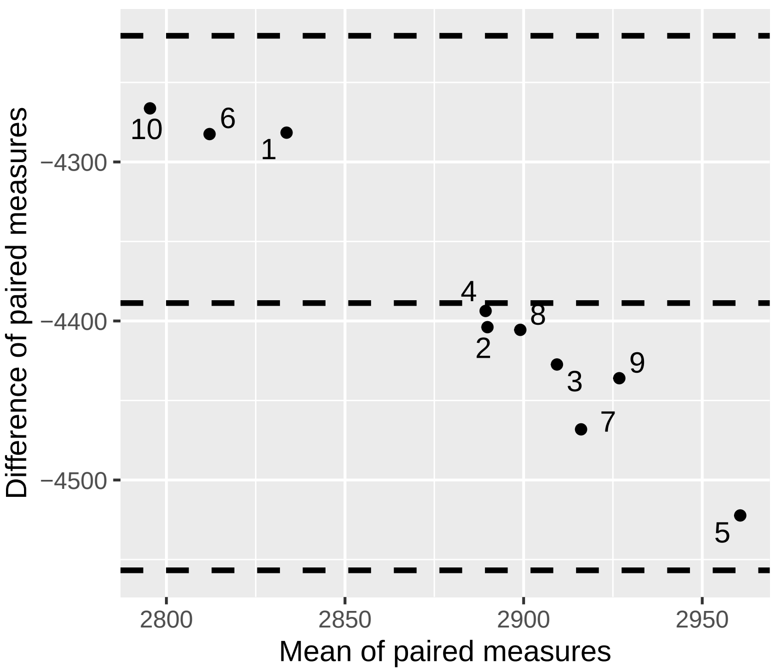


CT glrlm highgraylevelrunemphasis 8,16 mm

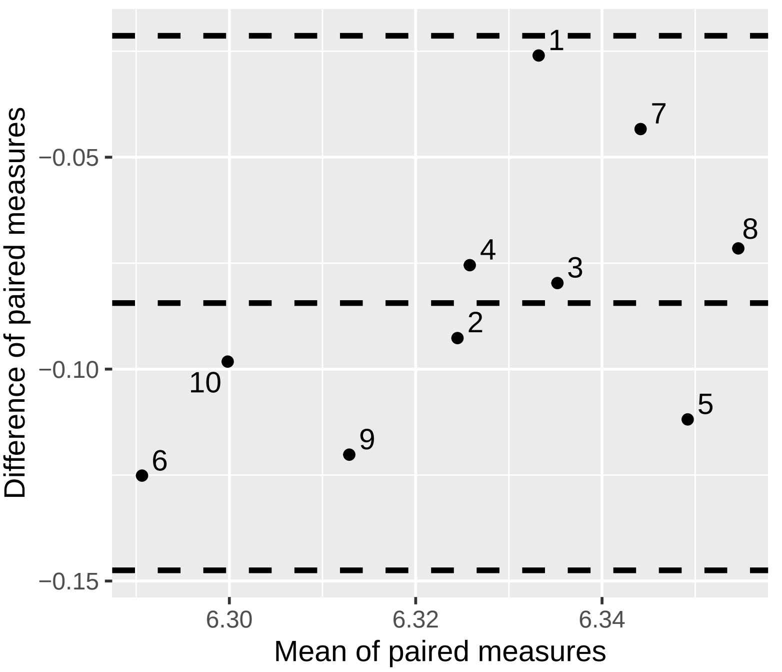


CT glrlm graylevelnonuniformitynormalized 8

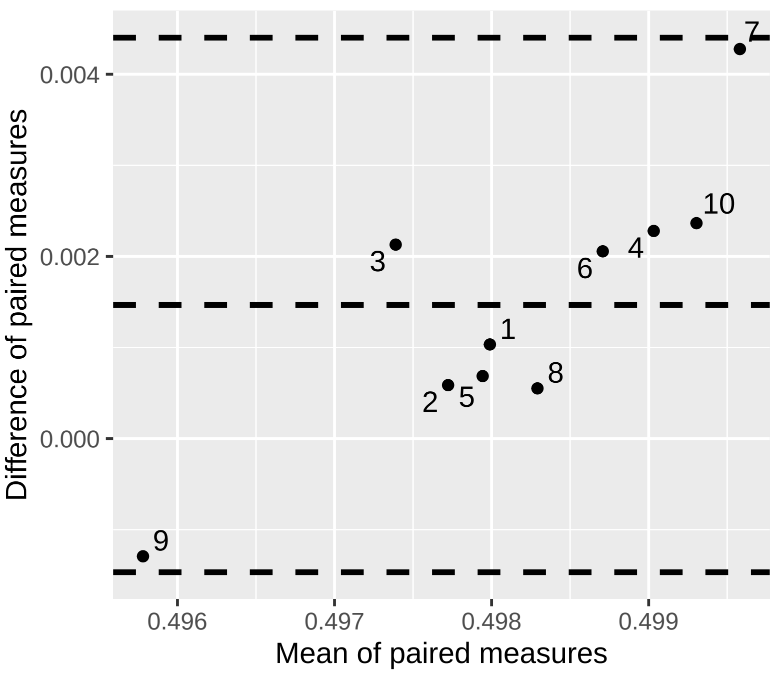


CT glrlm longrunemphasis 8,16 mm

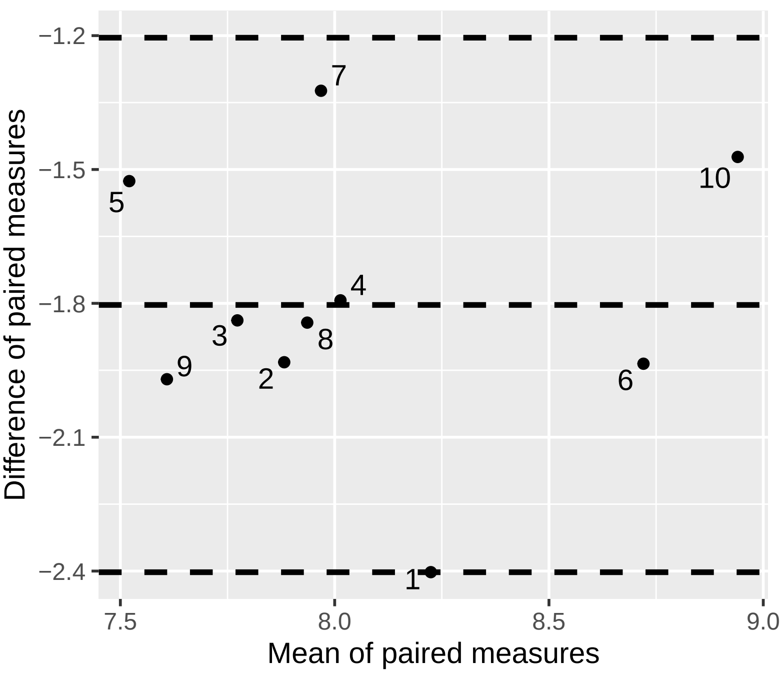


CT glrlm graylevelvariance 8,16 mm

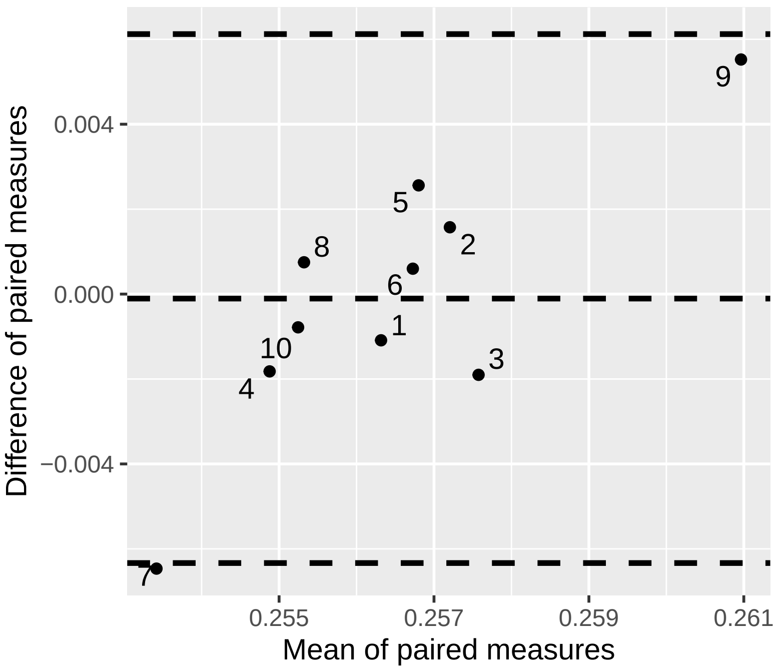


CT glrlm longrunhighgraylevelemphasis 8,16

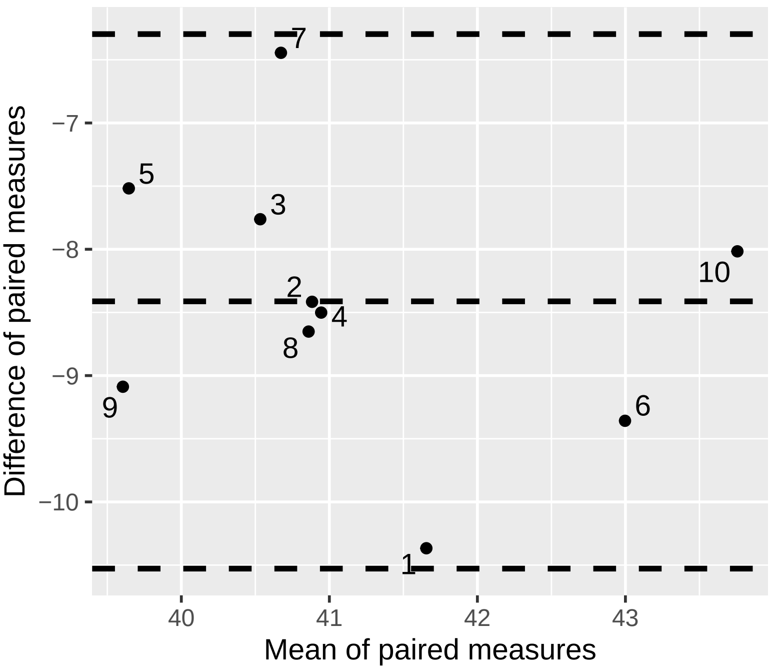

CT glrlm longrunlowgraylevelemphasis 8,16 [illegible]

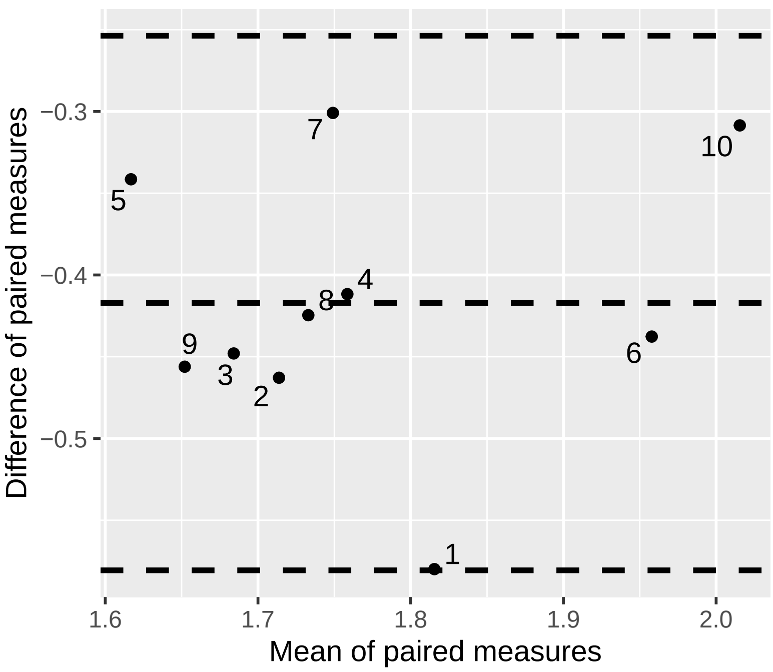


CT glrlm runlengthnonuniformity 8,16 mm

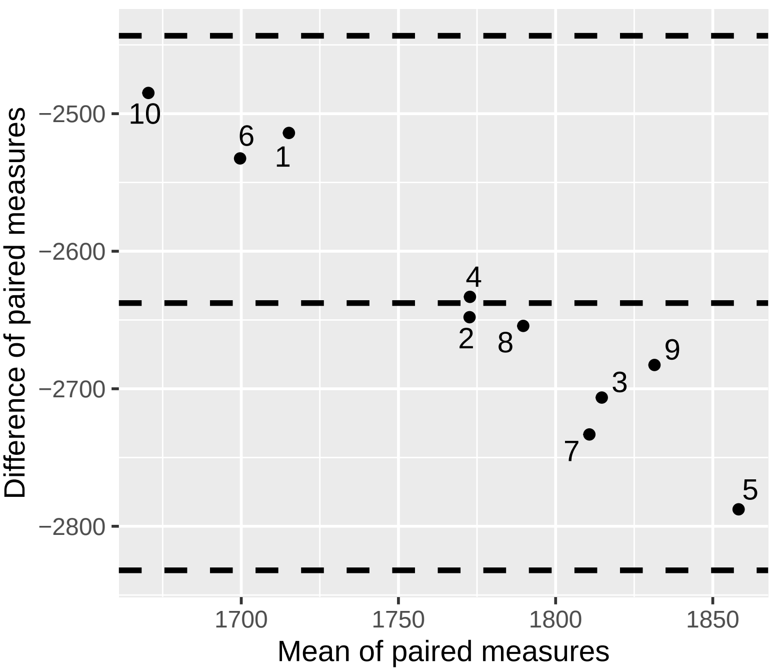


CT glrlm lowgraylevelrunemphasis 8,16 mm

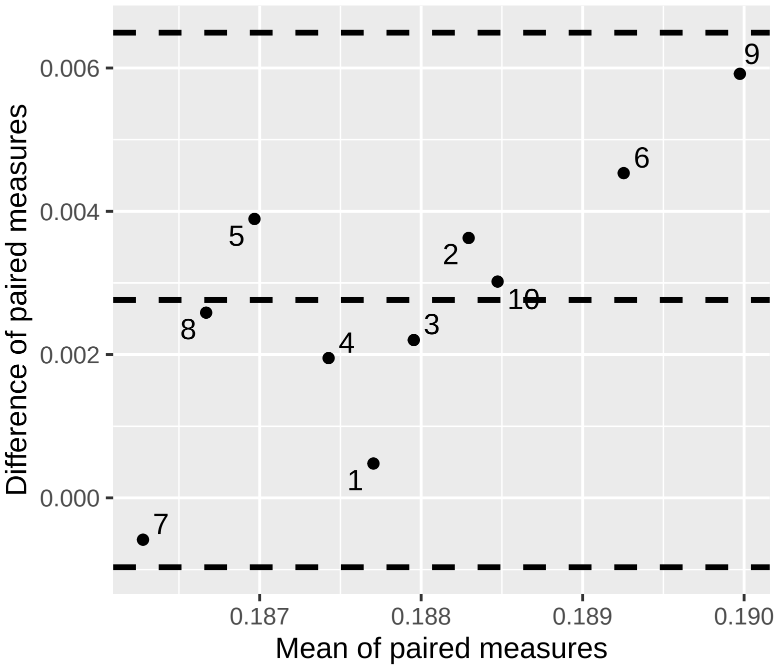


CT glrlm runlengthnonuniformitynormalized

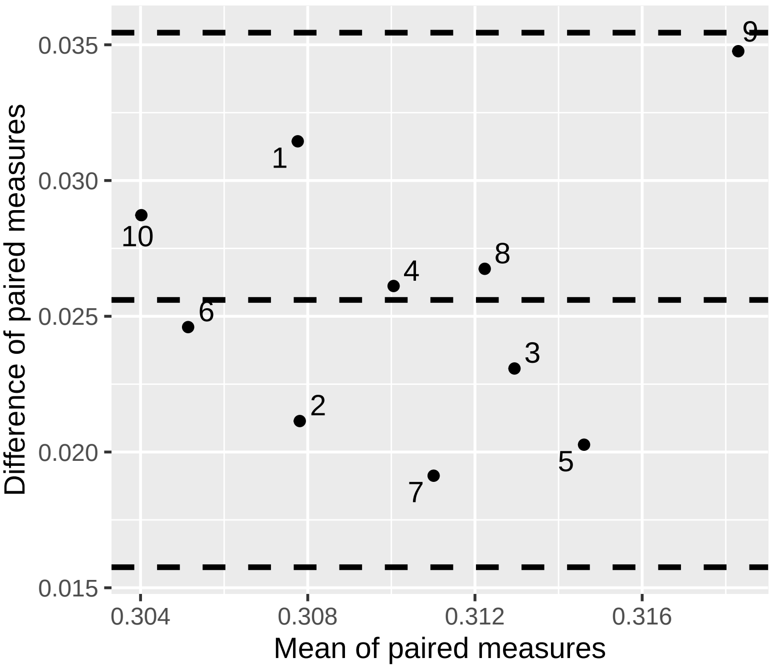


CT glrlm runentropy 8,16 mm

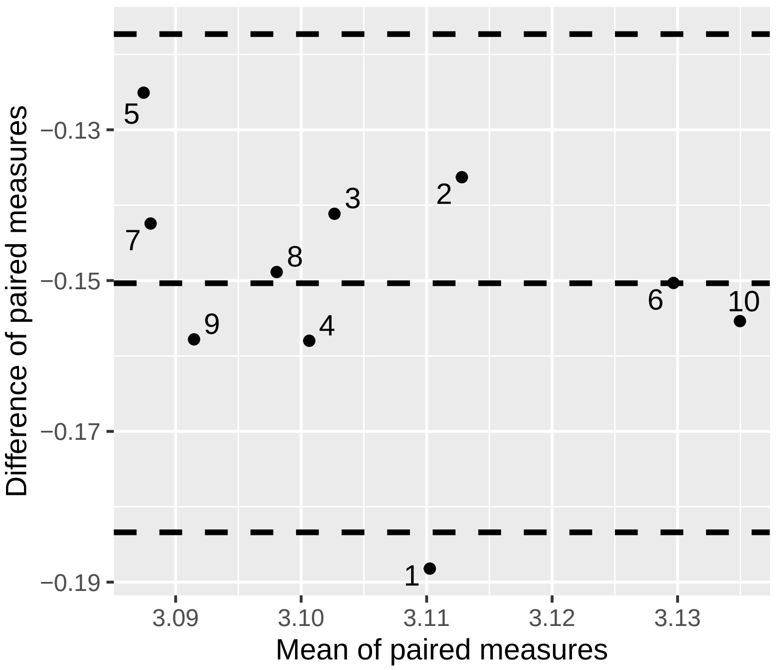


CT glrlm runpercentage 8,16 mm

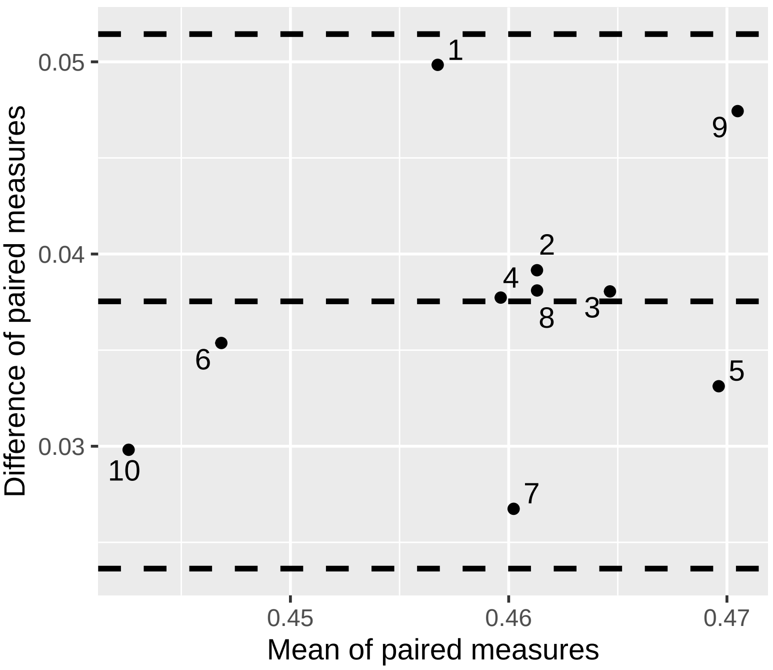

CT glrlm runvariance 8,16 mm

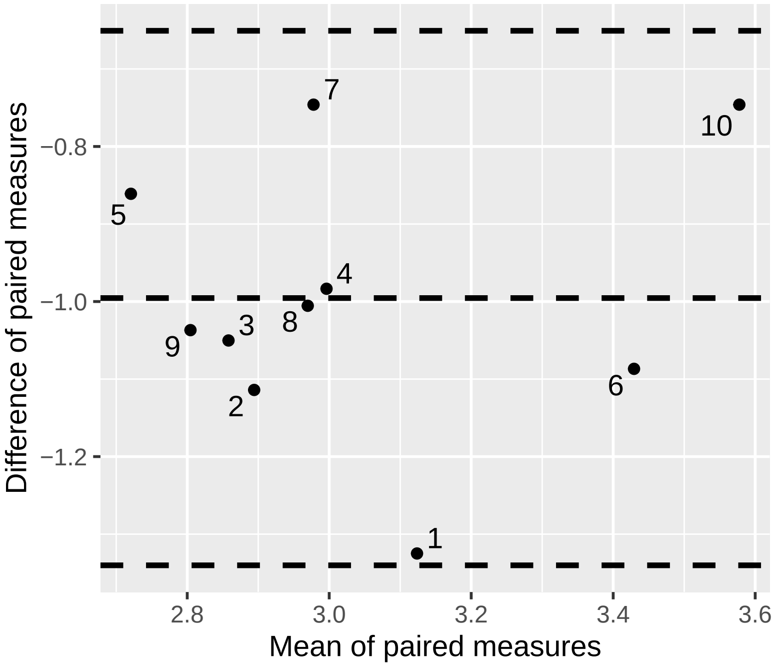


CT glrlm shortrunlowgraylevelemphasis 8,1

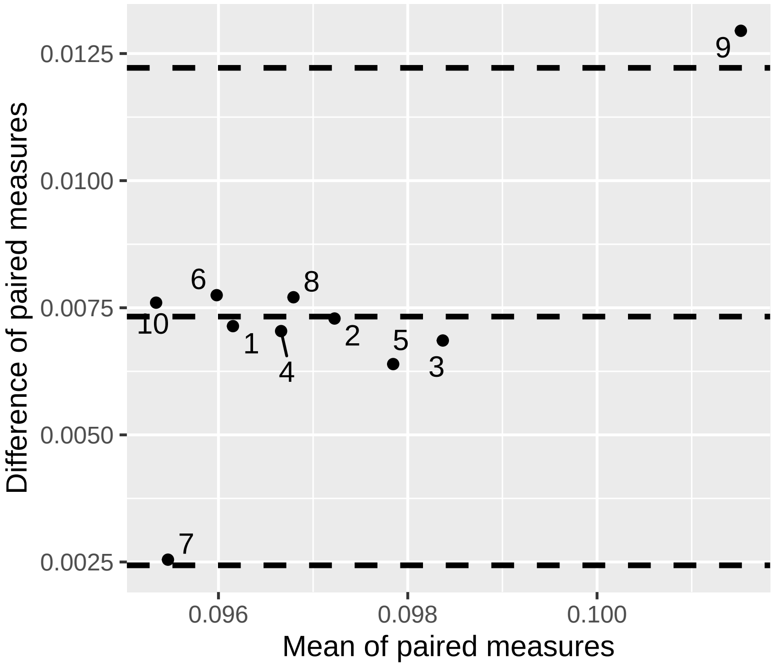


CT glrlm shortrunemphasis 8,16 mm

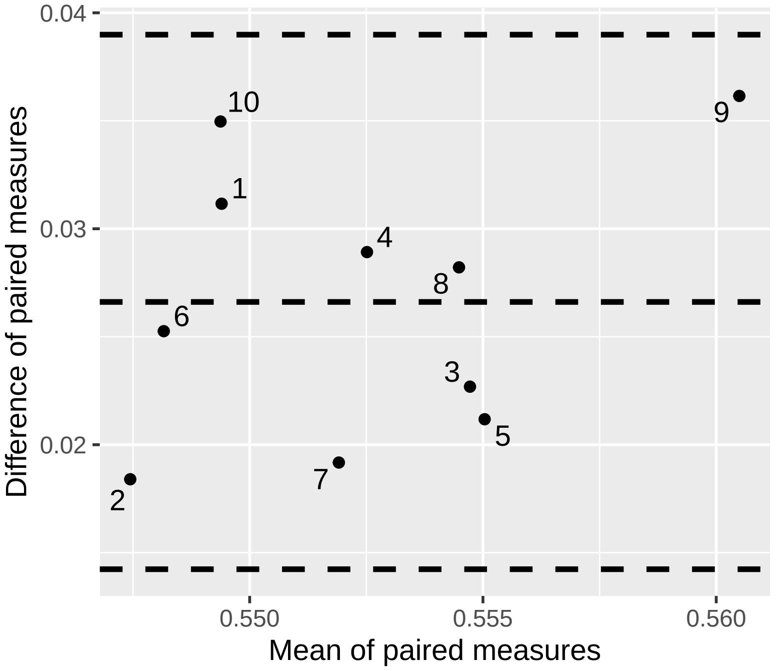


CT glszm graylevelnonuniformity 8,16 mm

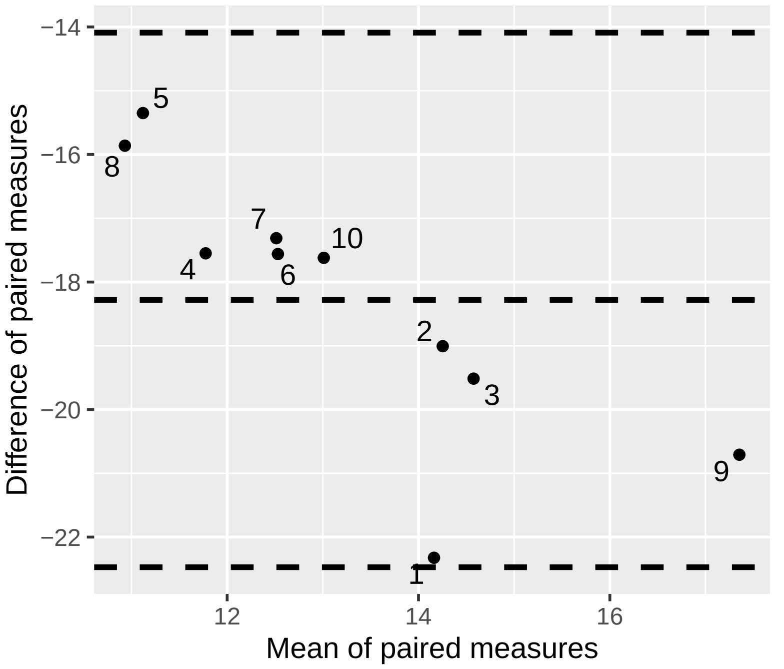


CT glrlm shortrunhighgraylevelemphasis 8,1

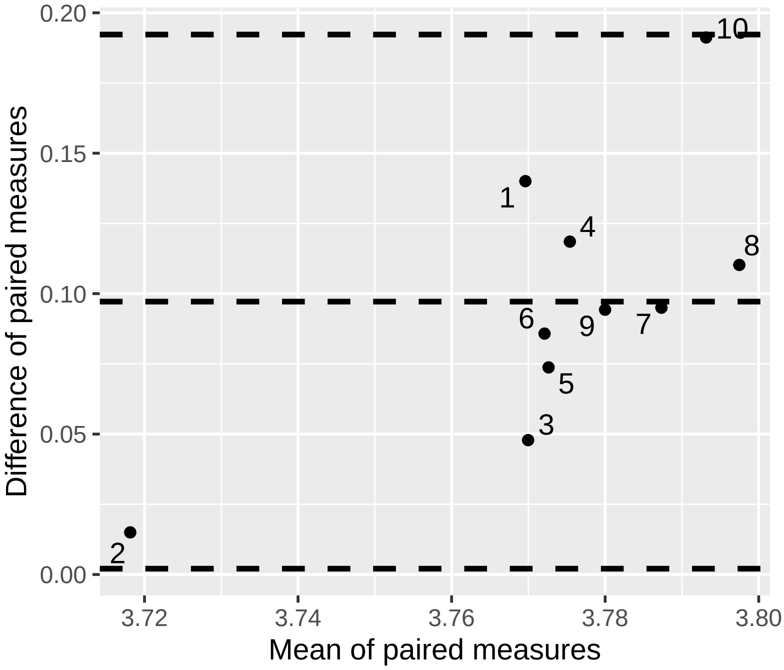


CT glszm graylevelnonuniformitynormalized 8

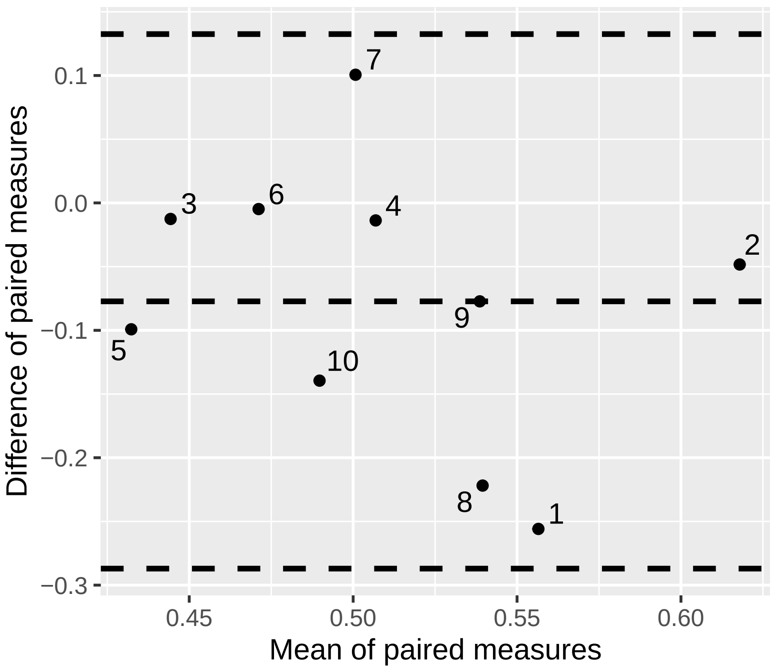

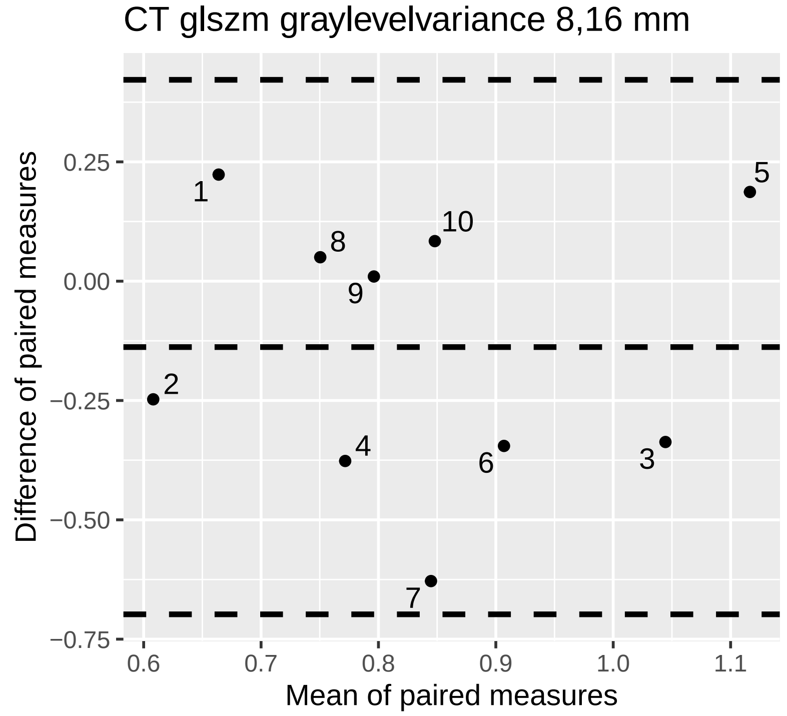
CT glszm graylevelvariance 8,16 mm
Difference of paired measures
Mean of paired measures

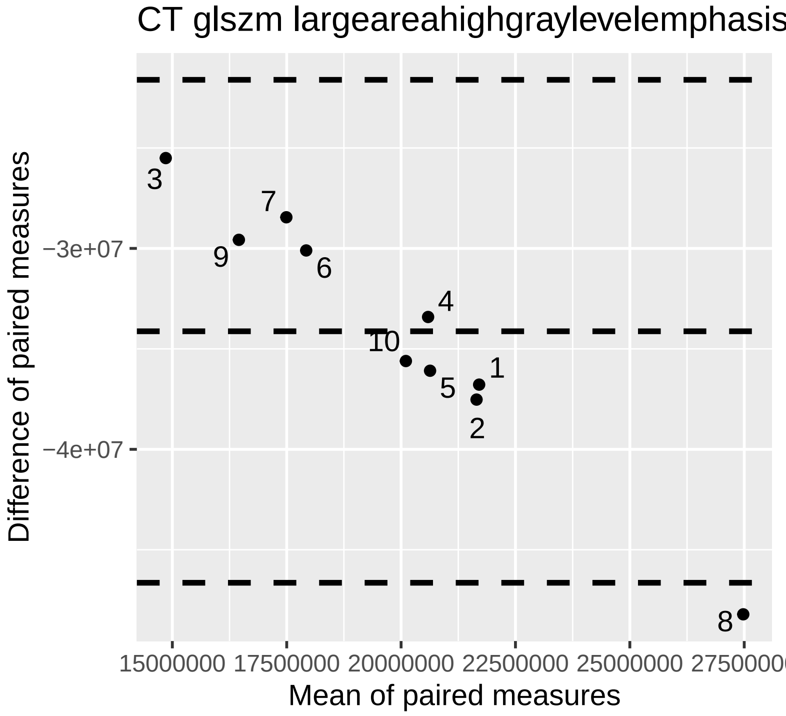
CT glszm largeareahighgraylevelemphasis
Difference of paired measures
Mean of paired measures

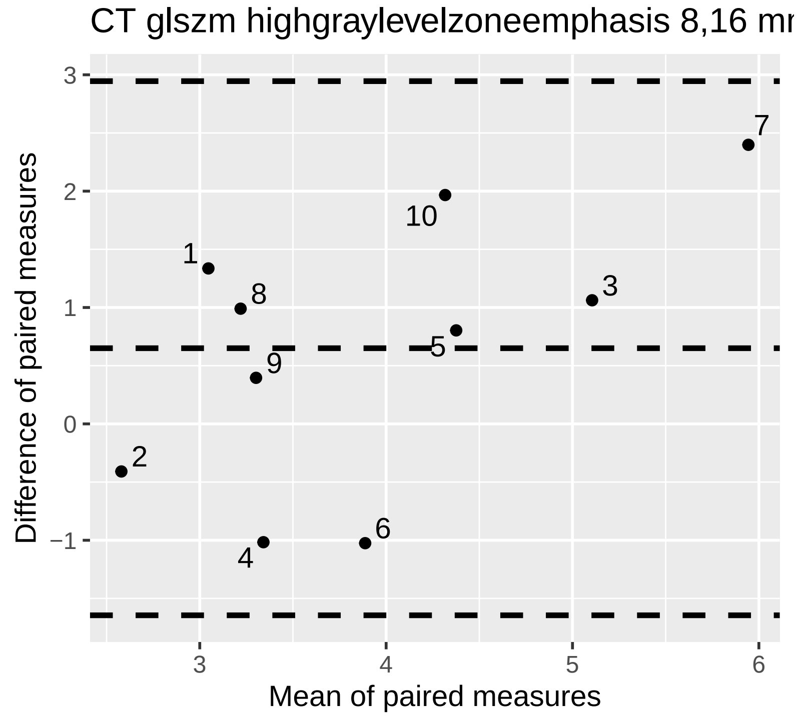
CT glszm highgraylevelzoneemphasis 8,16 mm
Difference of paired measures
Mean of paired measures

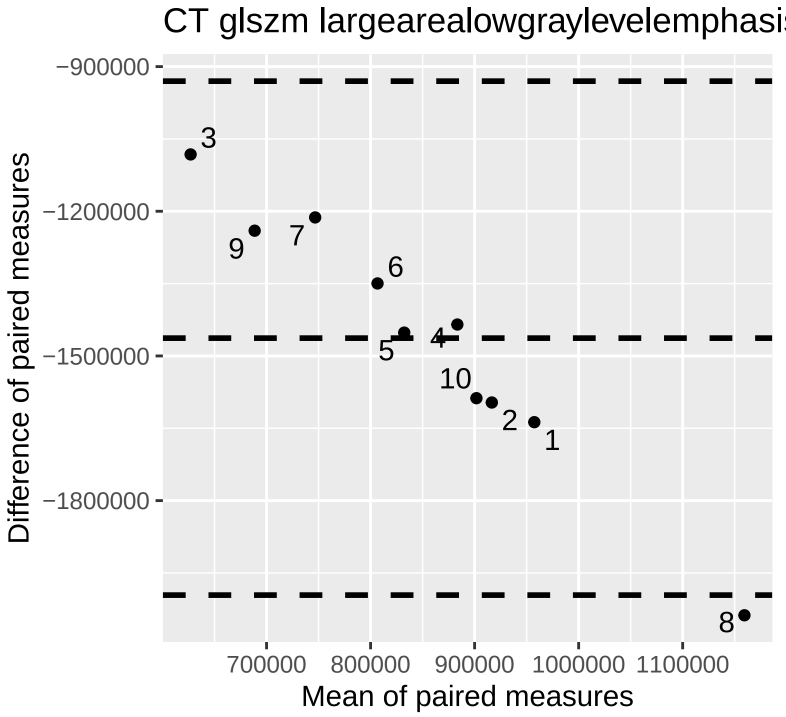
CT glszm largearealowgraylevelemphasis
Difference of paired measures
Mean of paired measures

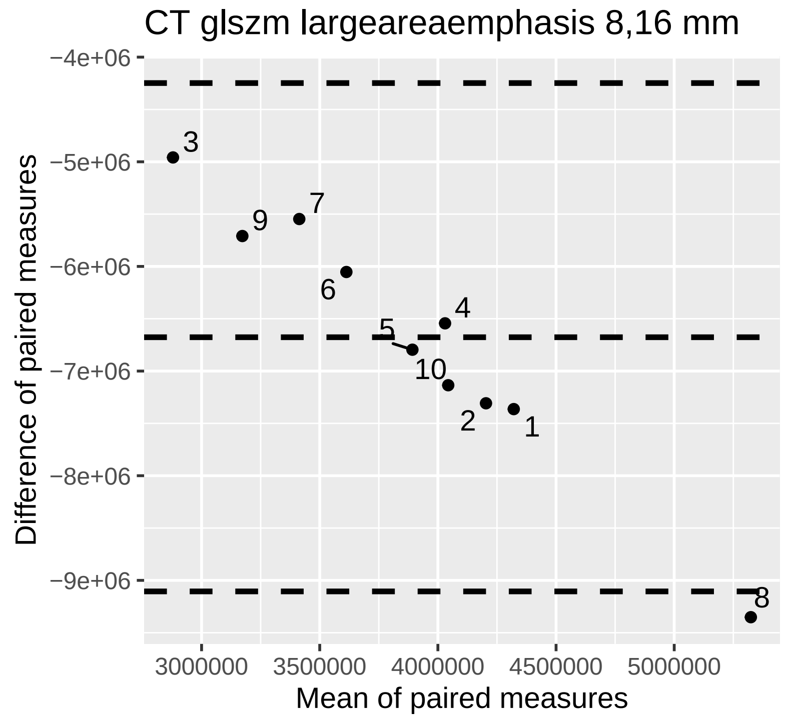
CT glszm largeareaemphasis 8,16 mm
Difference of paired measures
Mean of paired measures

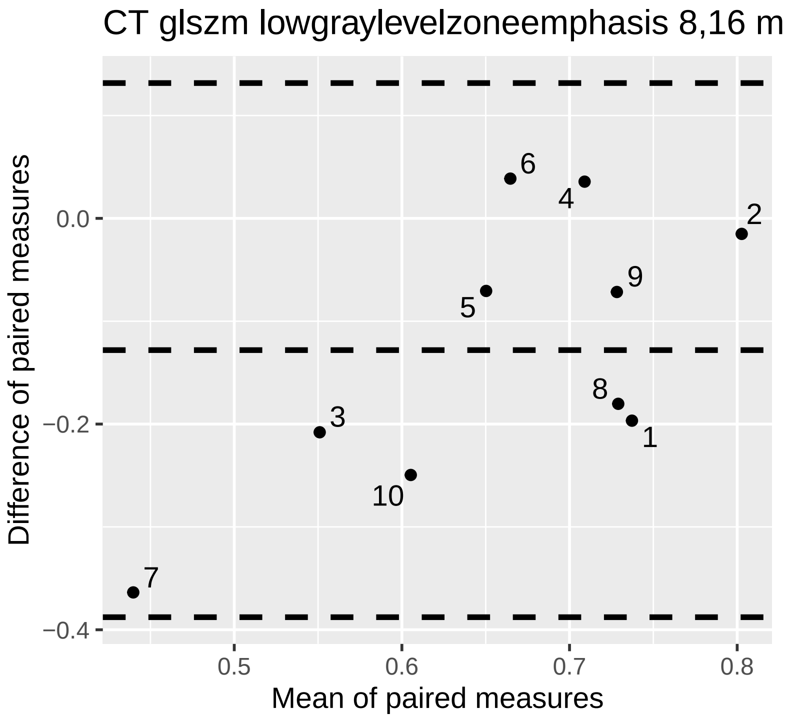
CT glszm lowgraylevelzoneemphasis 8,16 m
Difference of paired measures
Mean of paired measures

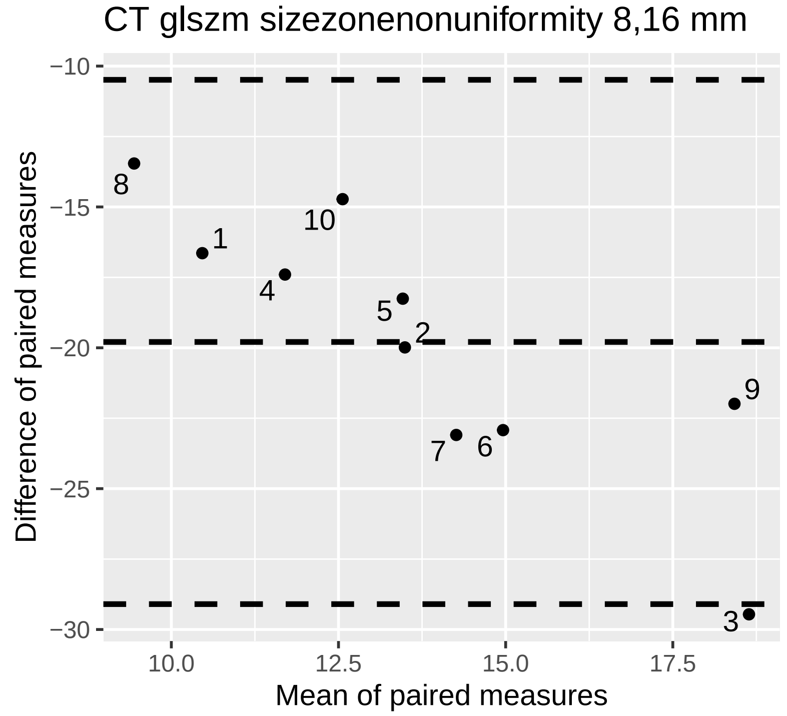
CT glszm sizezonenonuniformity 8,16 mm
Difference of paired measures
Mean of paired measures
−10
−15
−20
−25
−30
10.0
12.5
15.0
17.5

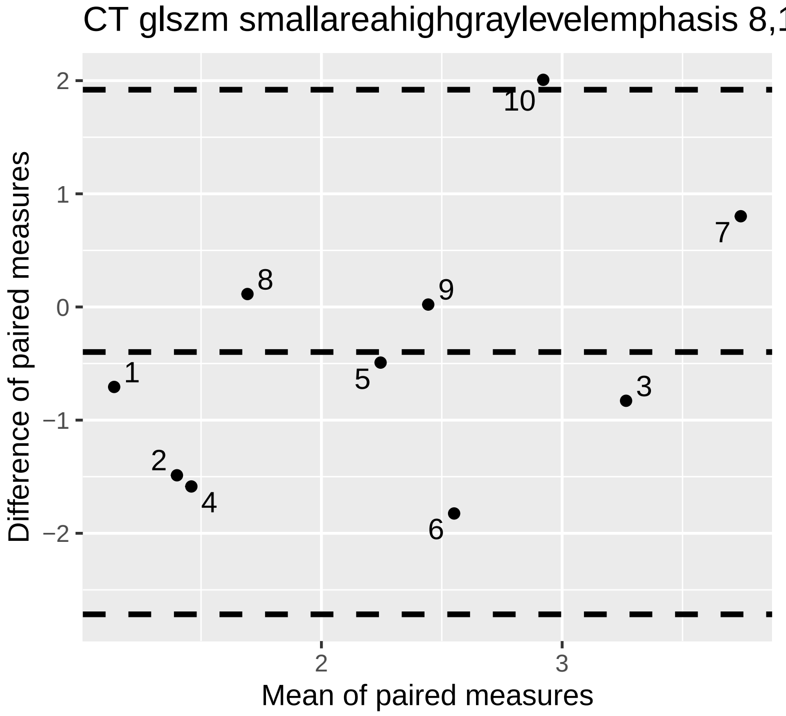
CT glszm smallareahighgraylevelemphasis 8,1
Difference of paired measures
Mean of paired measures
2
1
0
−1
−2
2
3

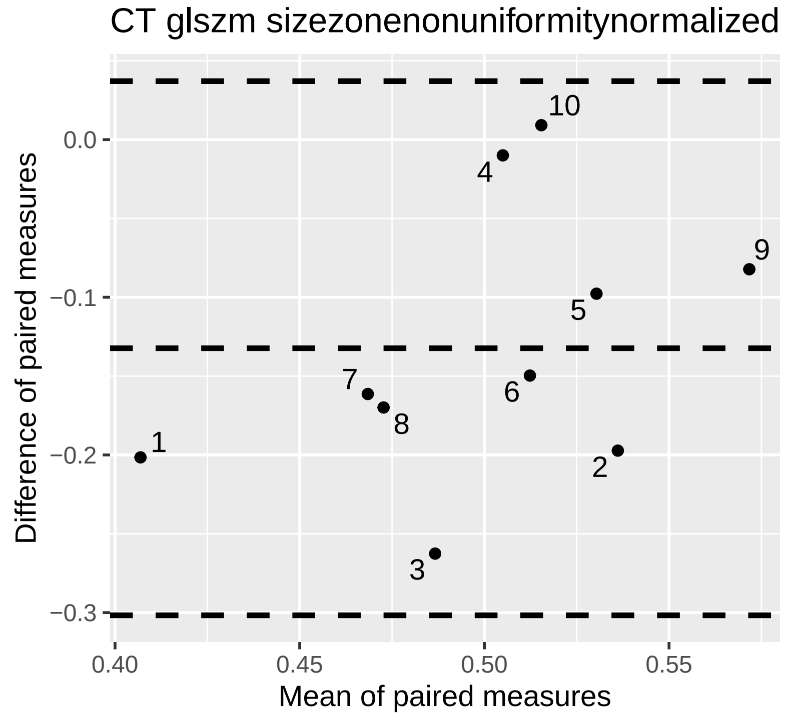
CT glszm sizezonenonuniformitynormalized 8
Difference of paired measures
Mean of paired measures
0.0
−0.1
−0.2
−0.3
0.40
0.45
0.50
0.55

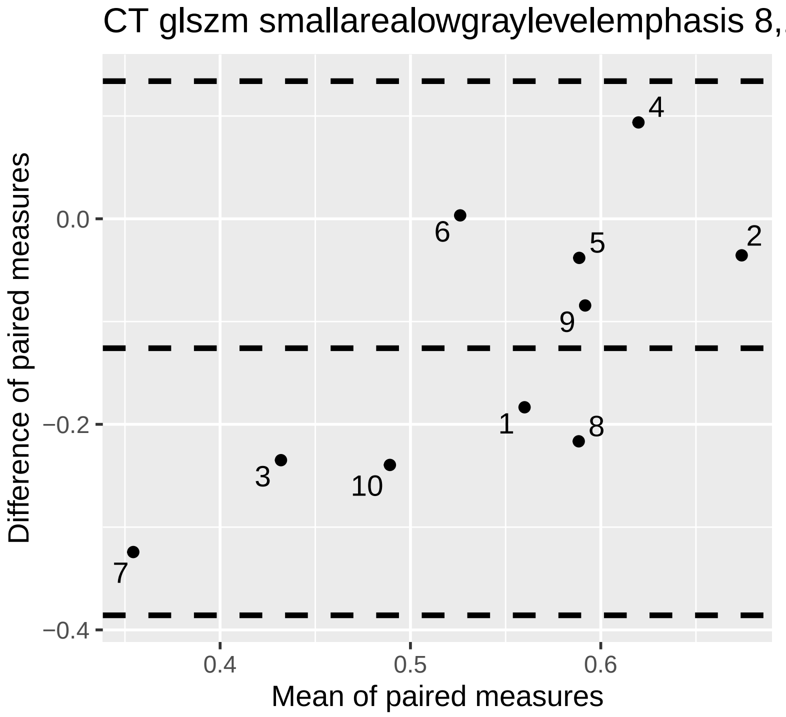
CT glszm smallarealowgraylevelemphasis 8,
Difference of paired measures
Mean of paired measures
0.0
−0.2
−0.4
0.4
0.5
0.6

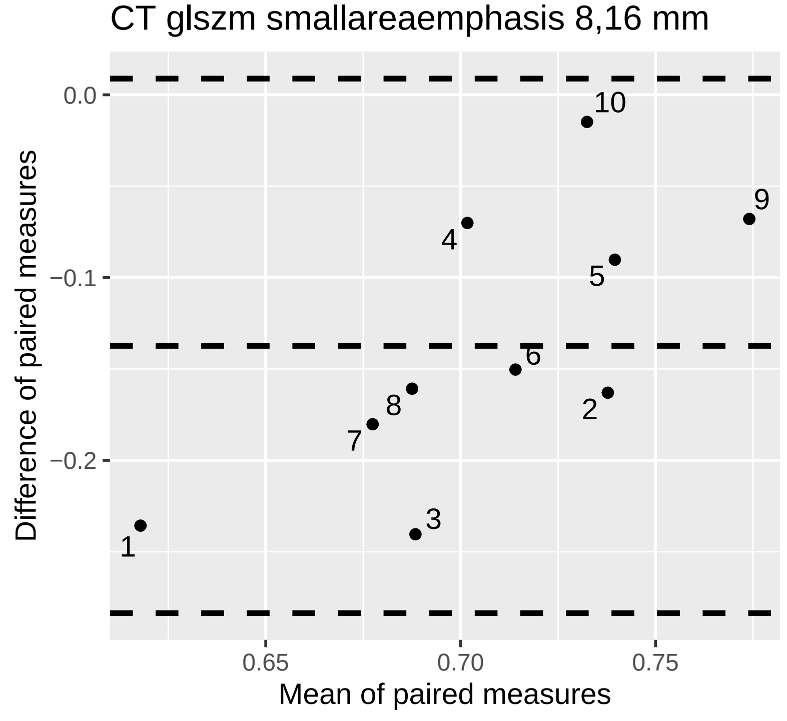
CT glszm smallareaemphasis 8,16 mm
Difference of paired measures
Mean of paired measures
0.0
−0.1
−0.2
0.65
0.70
0.75

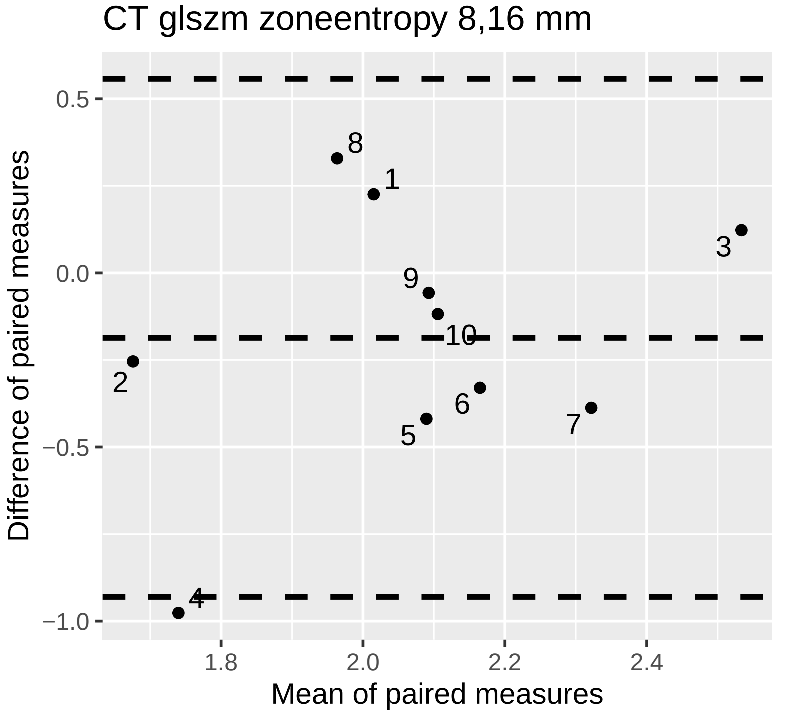
CT glszm zoneentropy 8,16 mm
Difference of paired measures
Mean of paired measures
0.5
0.0
−0.5
−1.0
1.8
2.0
2.2
2.4

CT glszm zonepercentage 8,16 mm

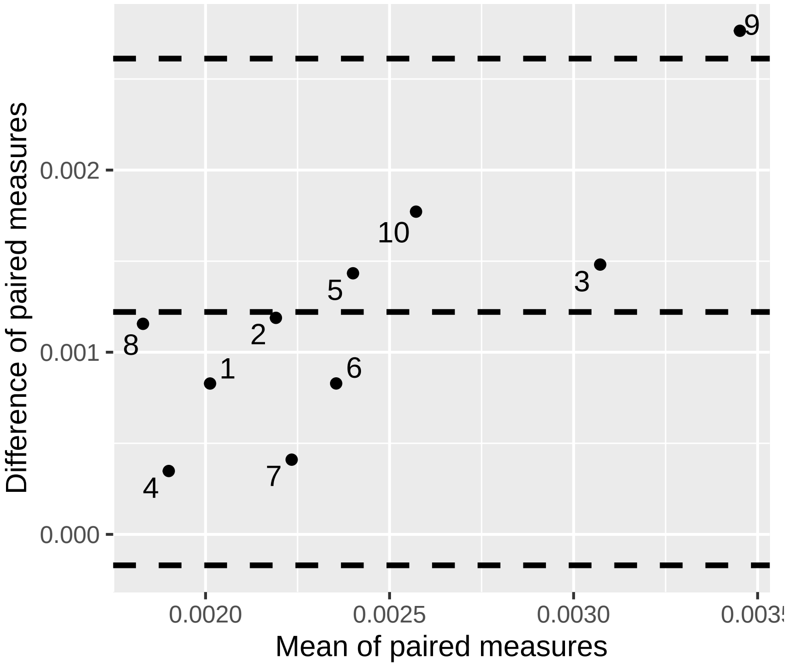


CT gldm dependencenonuniformity 8,16 mm

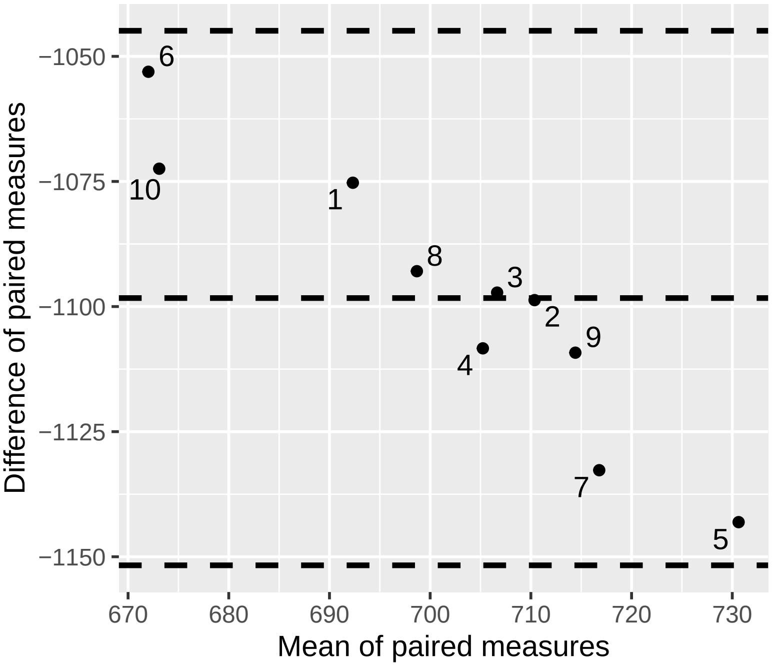


CT glszm zonevariance 8,16 mm

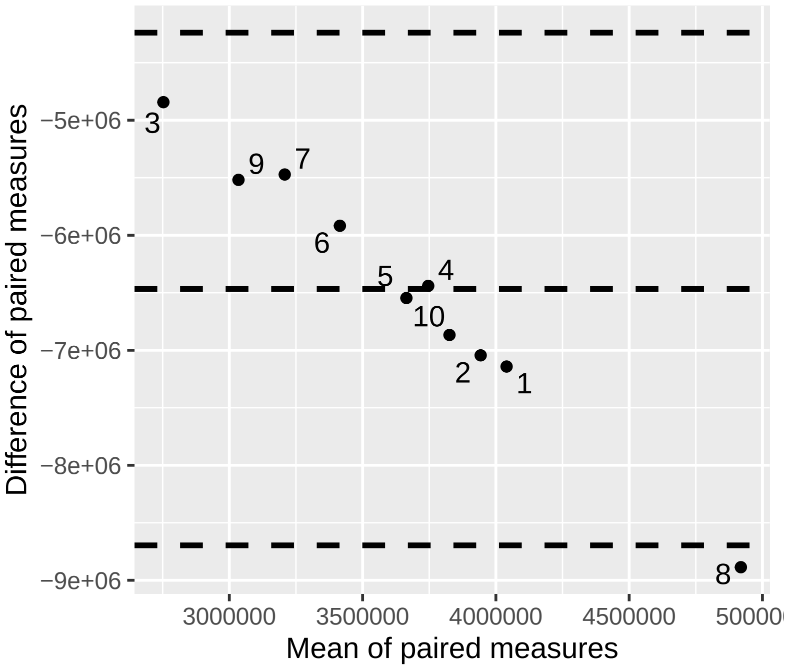


CT gldm dependencenonuniformitynormal

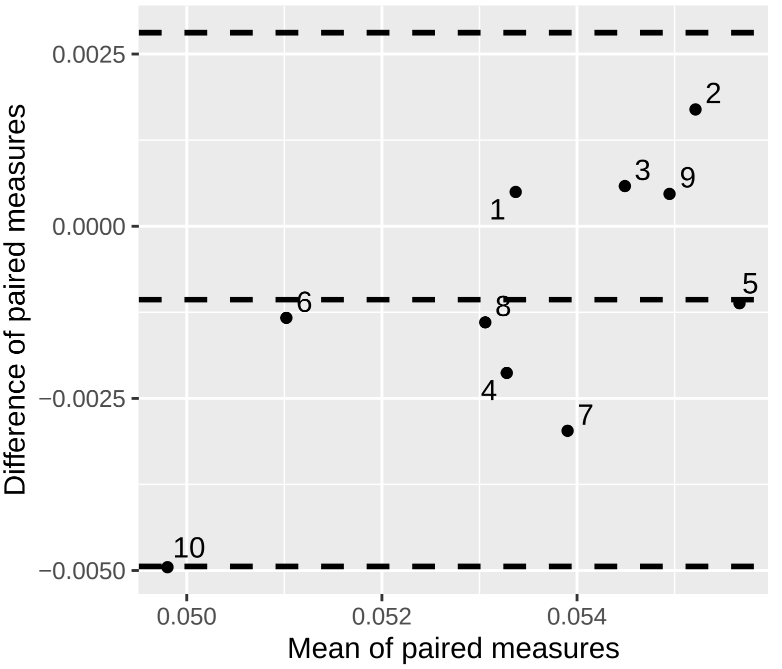


CT gldm dependenceentropy 8,16 mm

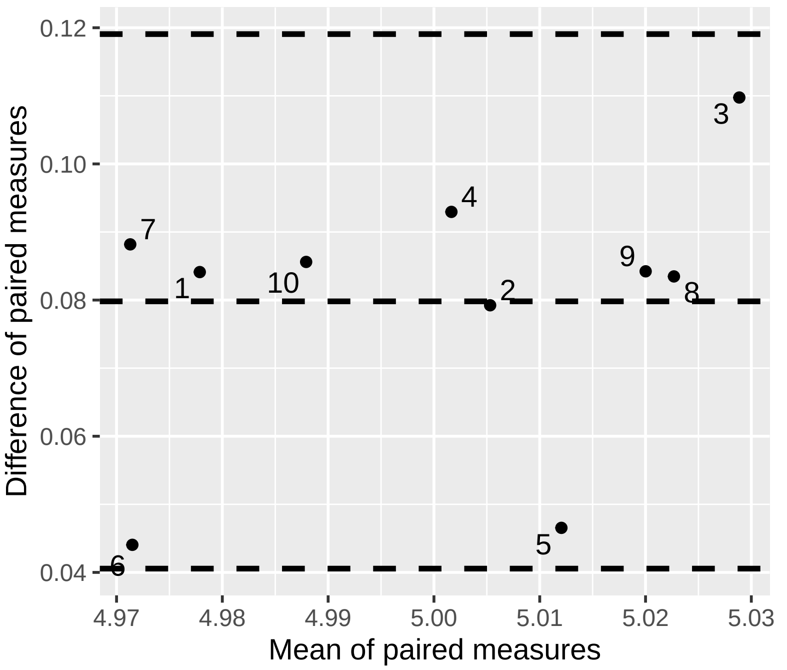


CT gldm dependencevariance 8,16 mm

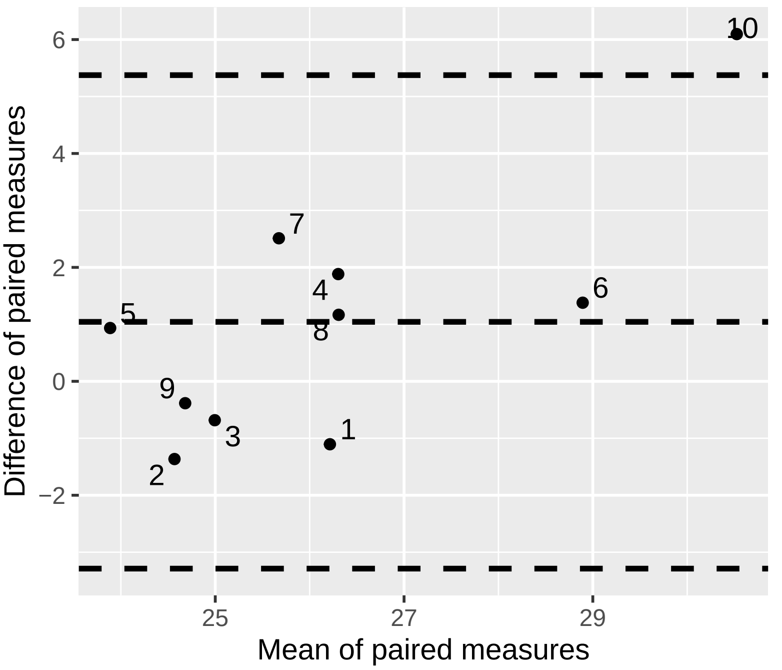

CT gldm graylevelnonuniformity 8,16 mm

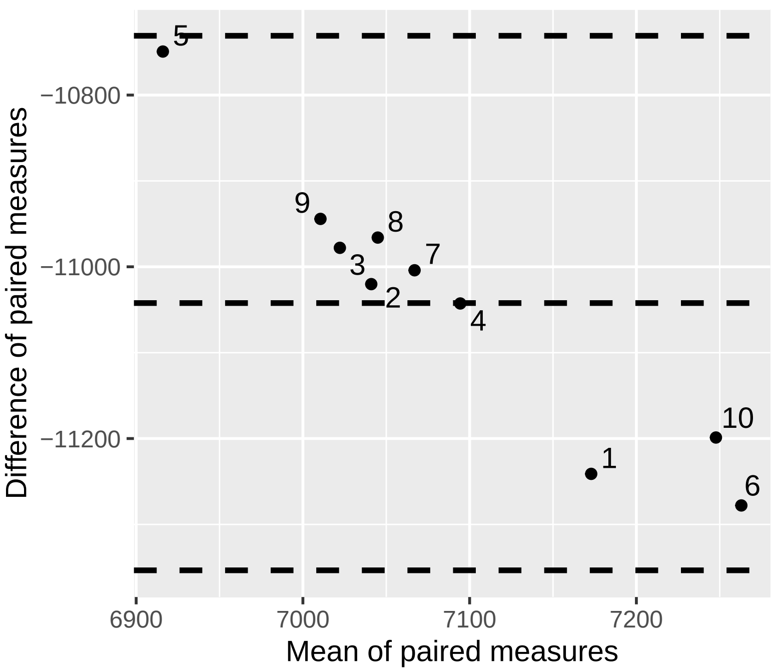


CT gldm largedependenceemphasis 8,16 mm

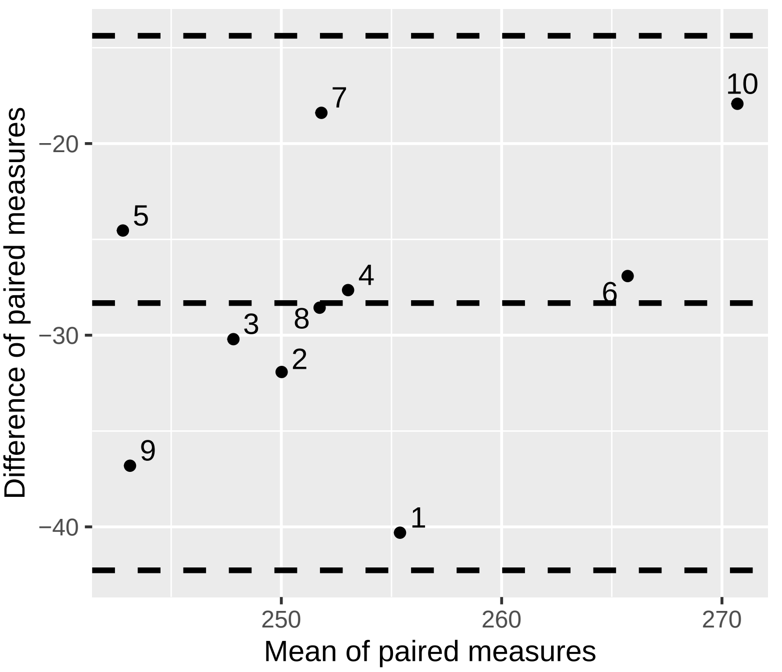


CT gldm graylevelvariance 8,16 mm

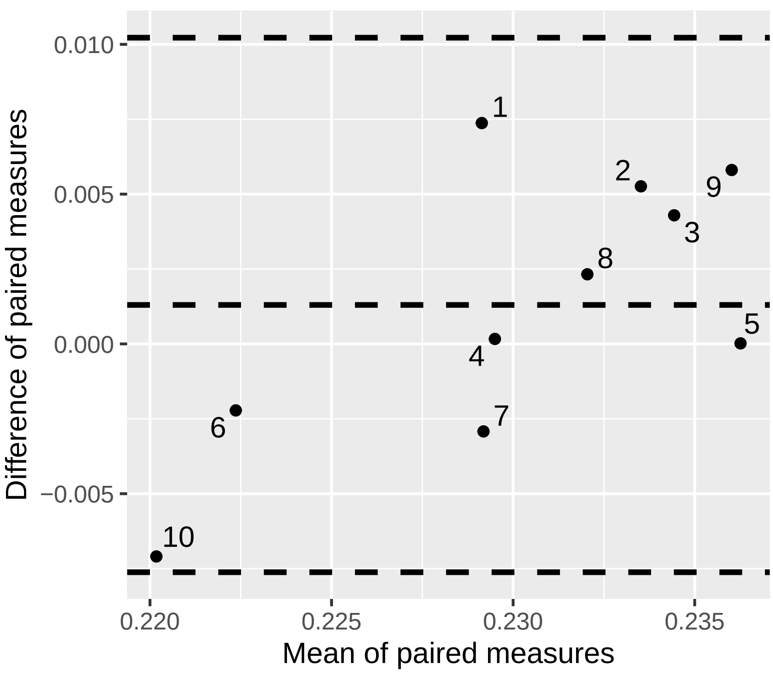


CT gldm largedependencehighgraylevelemp

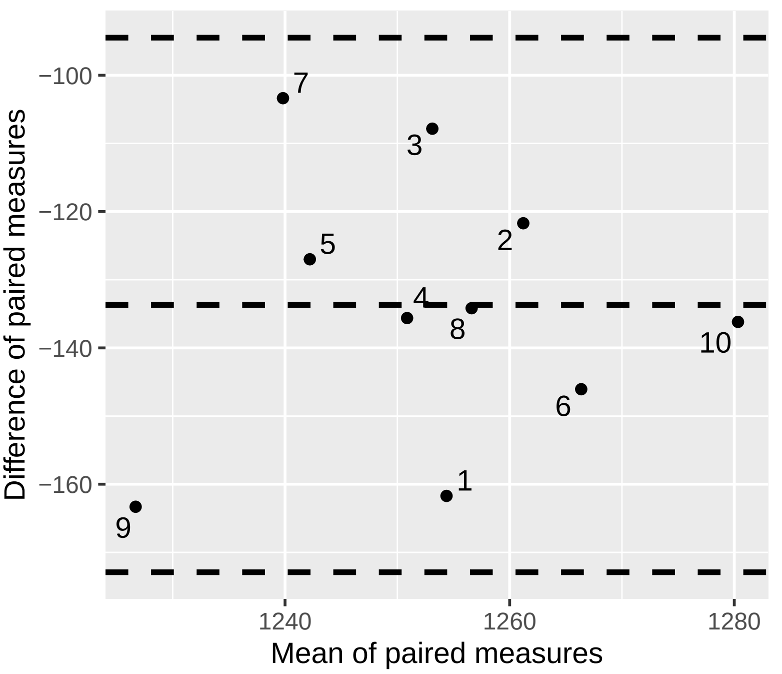


CT gldm highgraylevelemphasis 8,16 mm

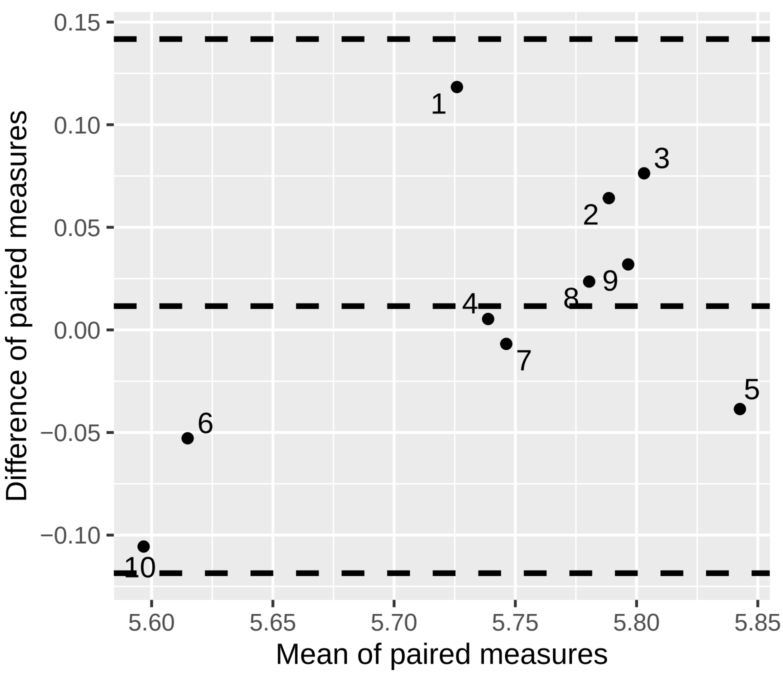


CT gldm largedependencelowgraylevelempha

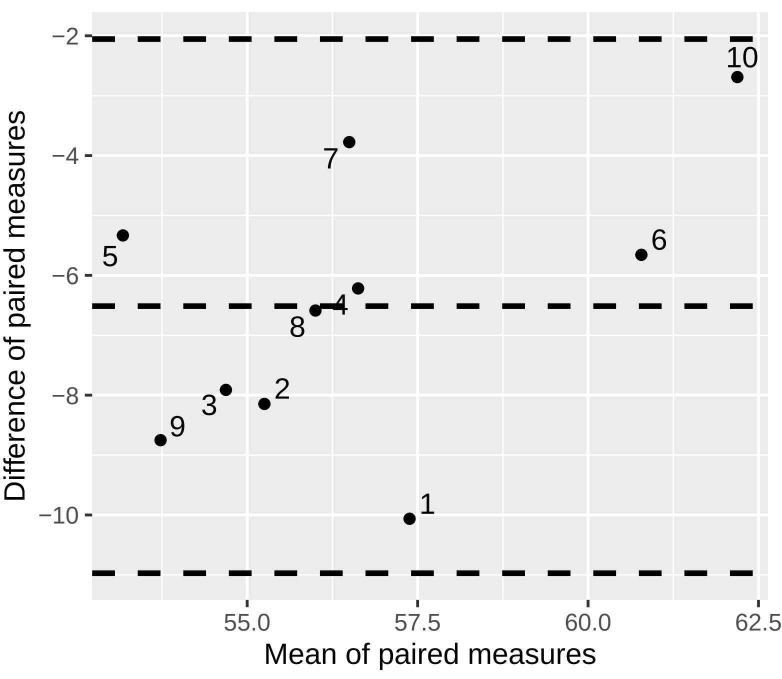

CT gldm lowgraylevelemphasis 8,16 mm

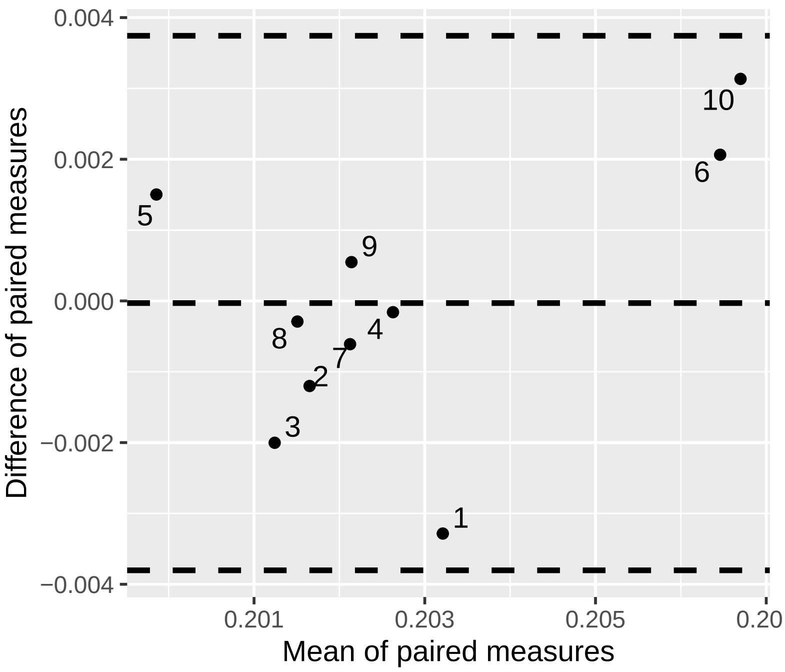


CT gldm smalldependencelowgraylevelem

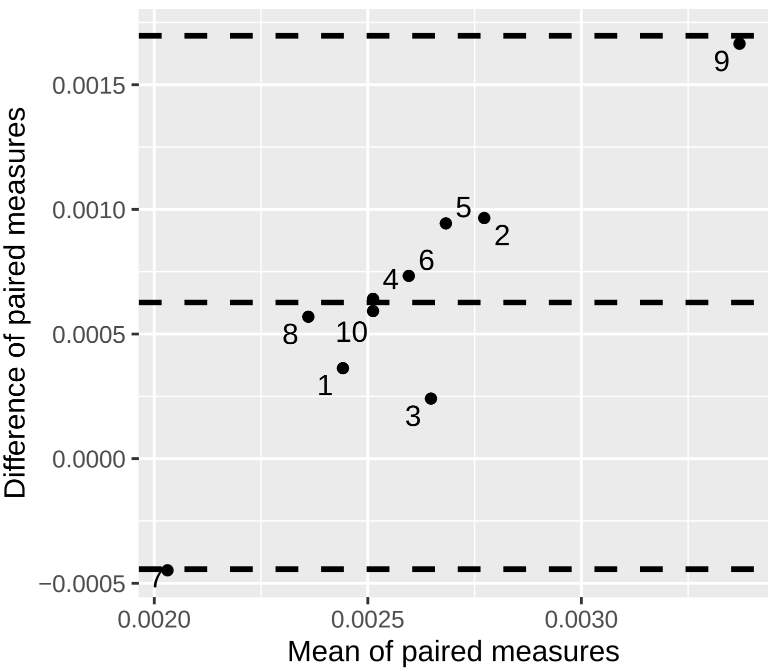


CT gldm smalldependenceemphasis 8,16 m

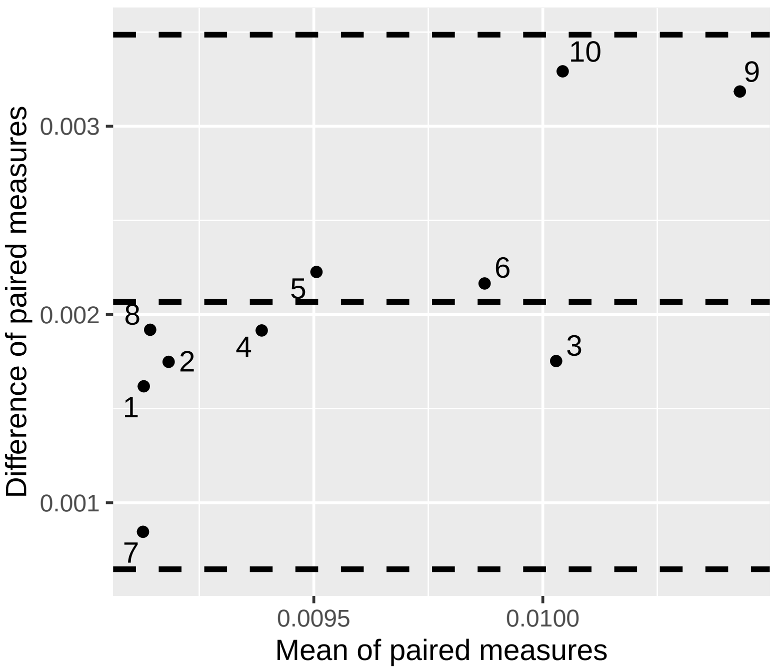


CT ngtdm busyness 8,16 mm

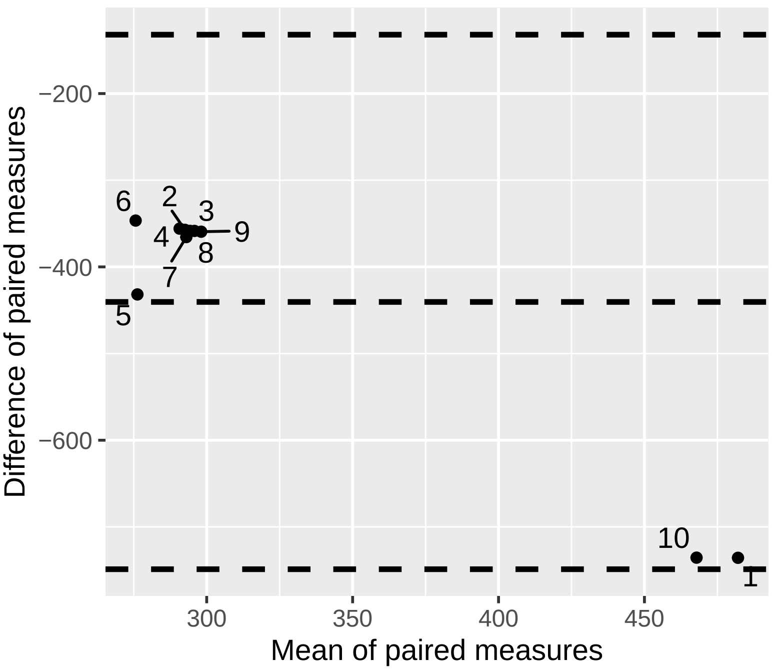


CT gldm smalldependencehighgraylevelem

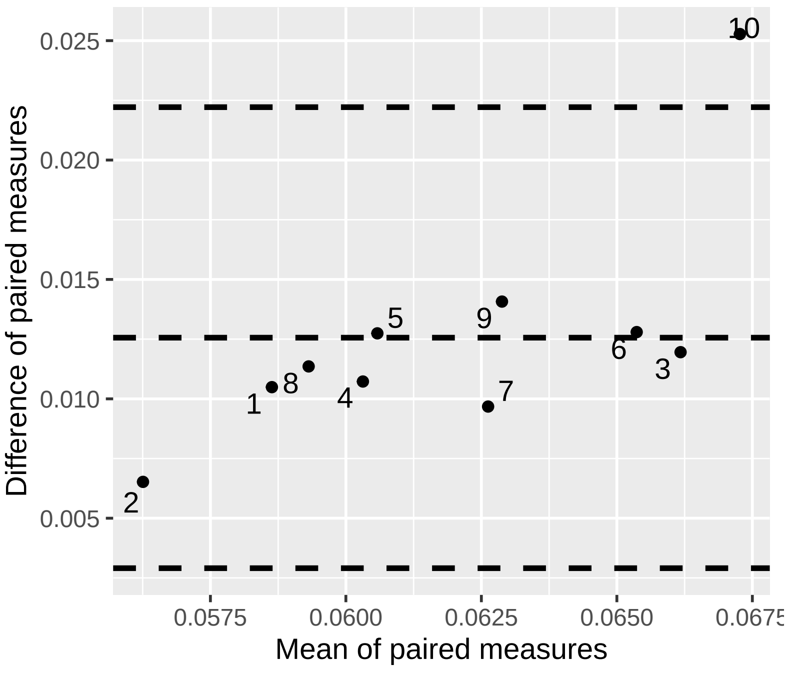


CT ngtdm coarseness 8,16 mm

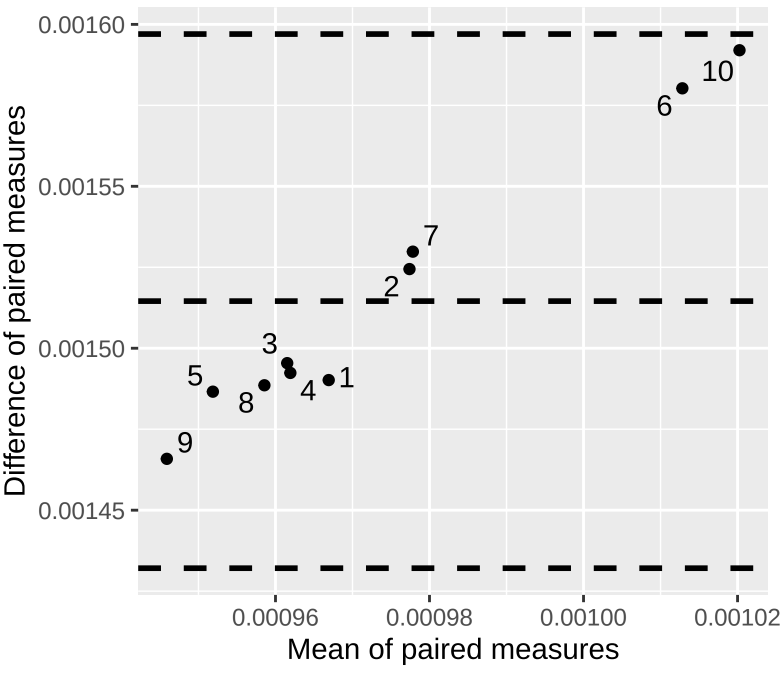

CT ngtdm complexity 8,16 mm

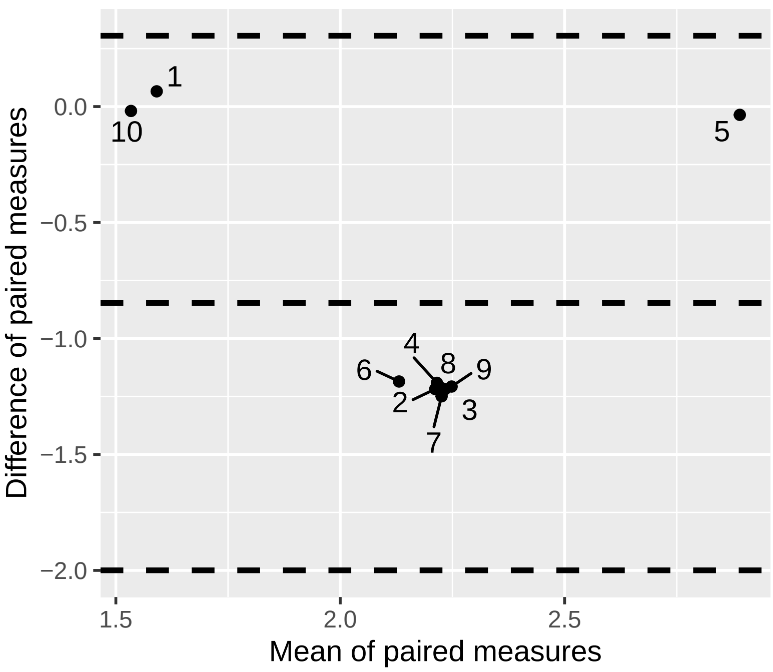


CT firstorder 10percentile 8,16 px

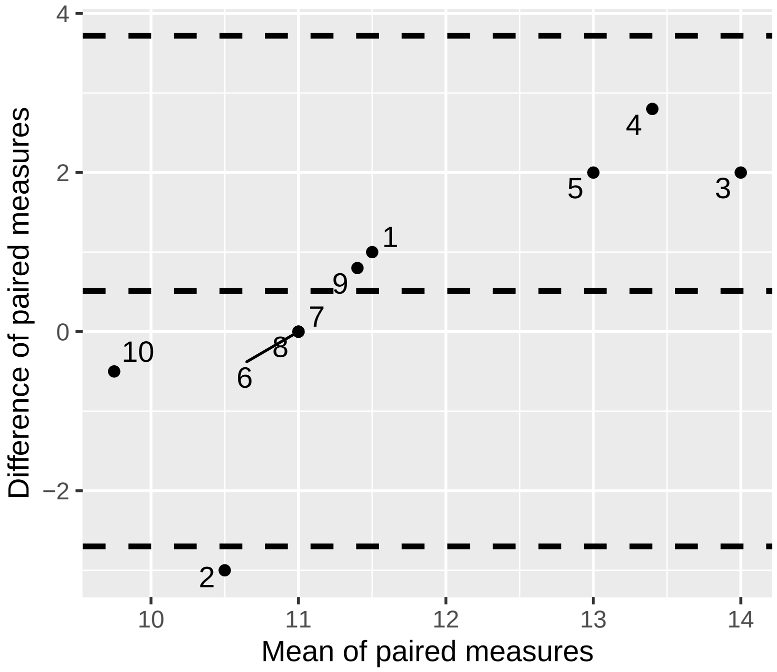


CT ngtdm contrast 8,16 mm

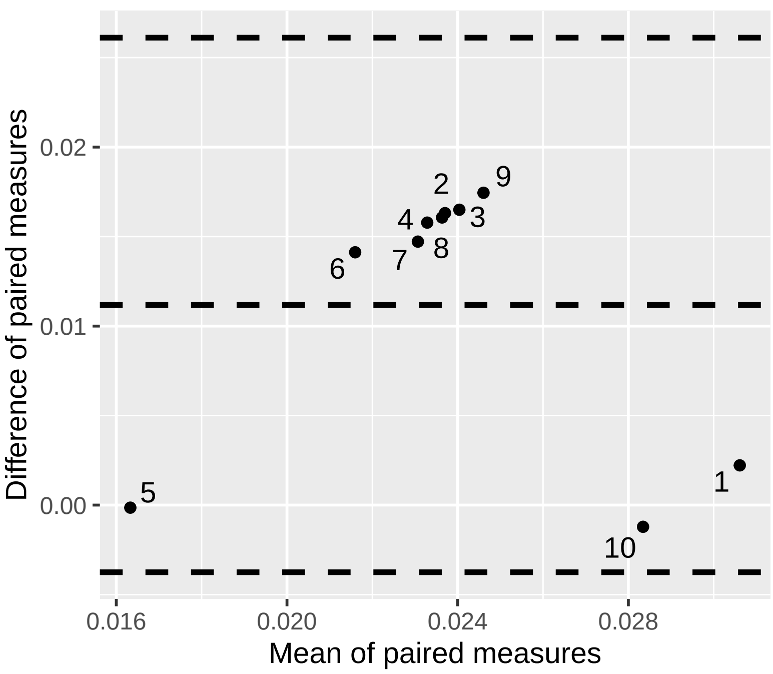


CT firstorder 90percentile 8,16 px

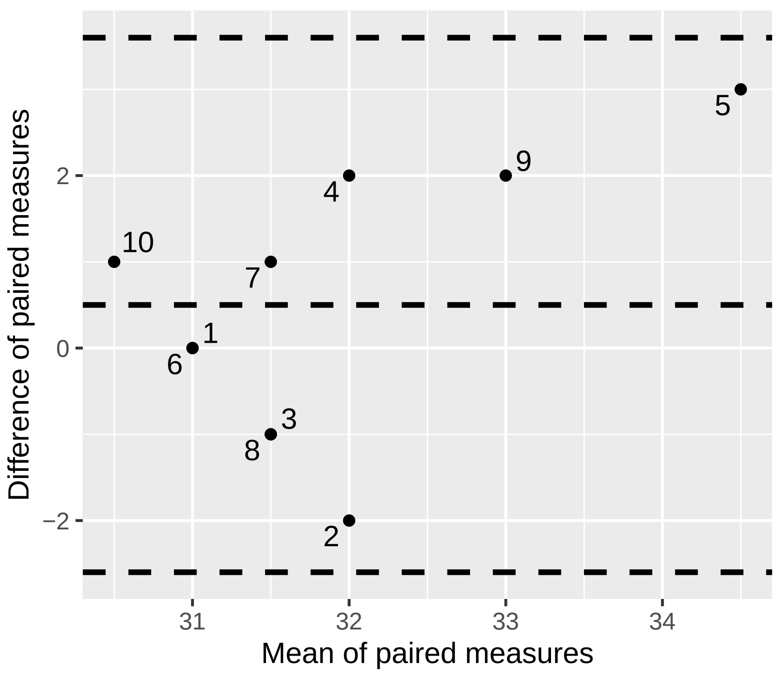


CT ngtdm strength 8,16 mm

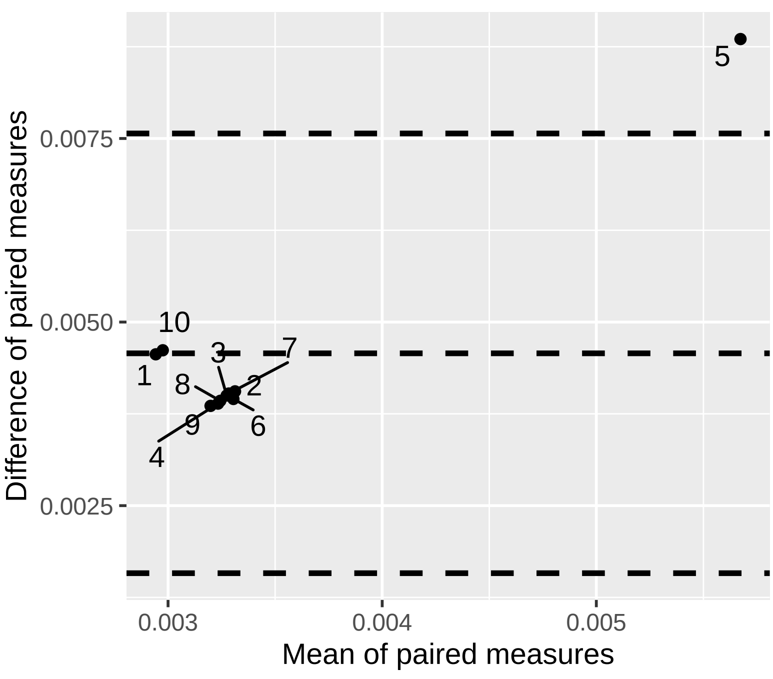


CT firstorder energy 8,16 px

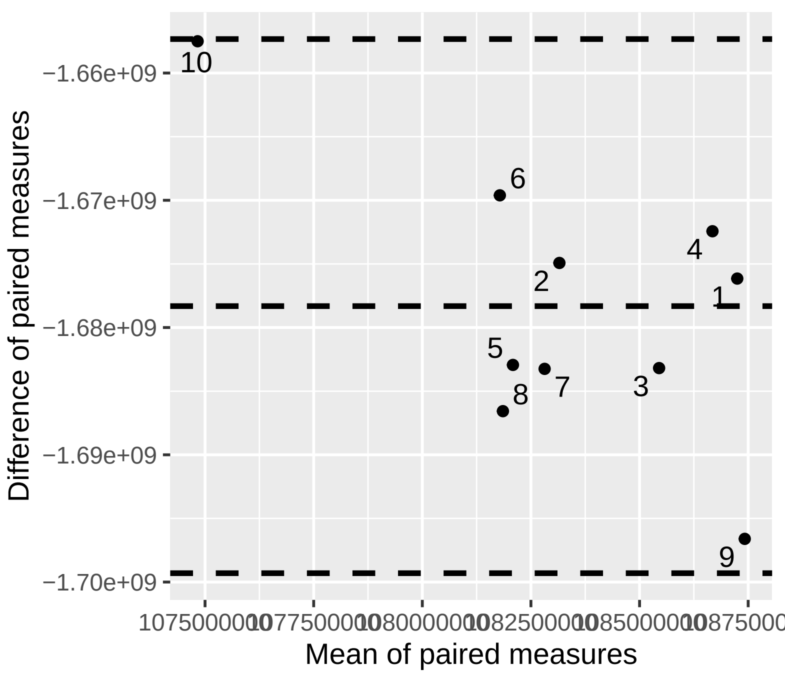

CT firstorder entropy 8,16 px

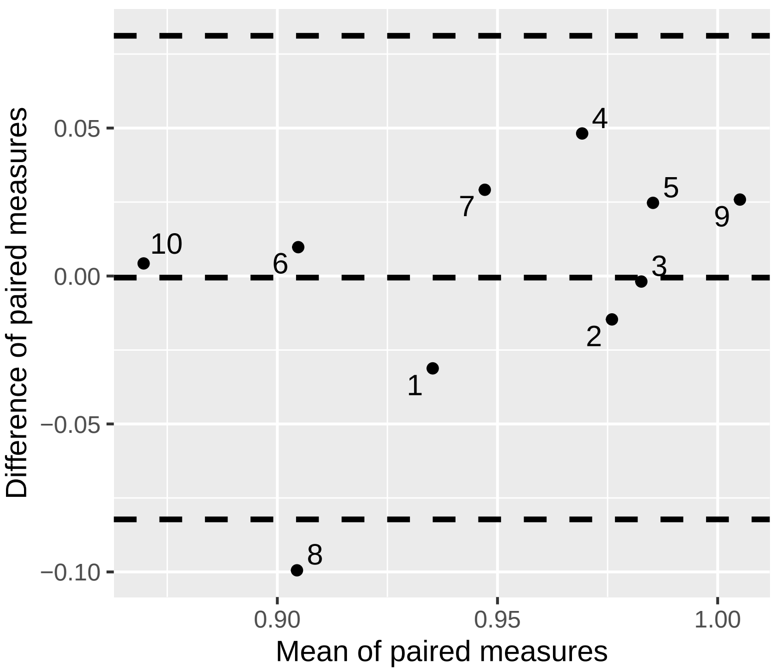


CT firstorder maximum 8,16 px

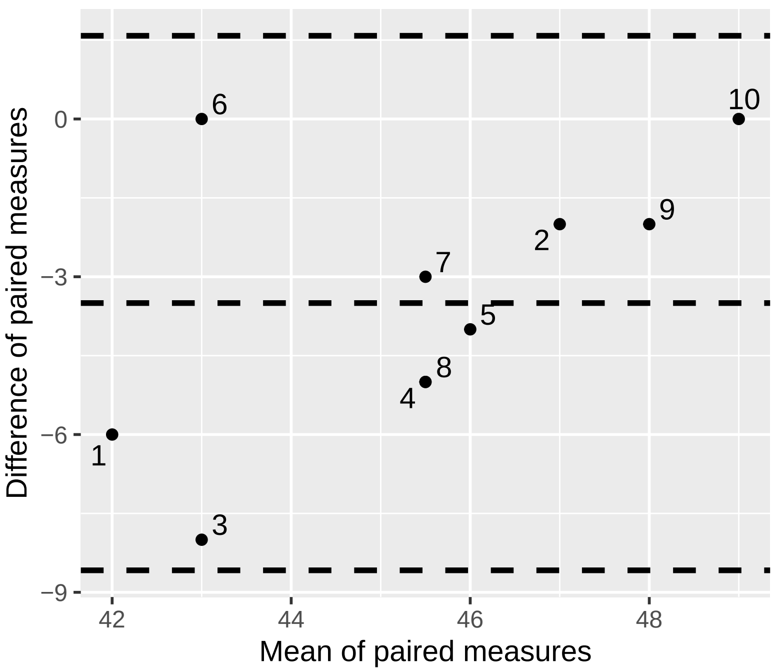


CT firstorder interquartilerange 8,16 px

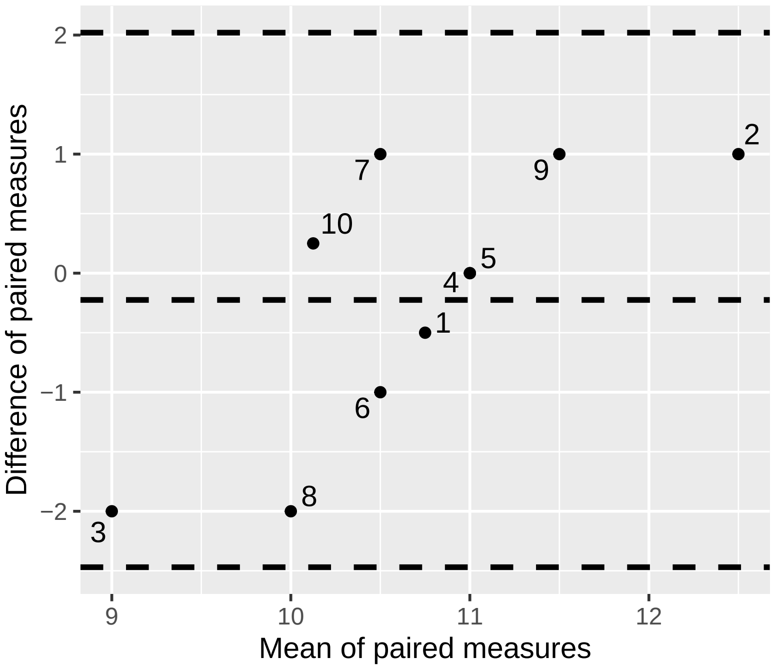


CT firstorder meanabsolutedeviation 8,16 px

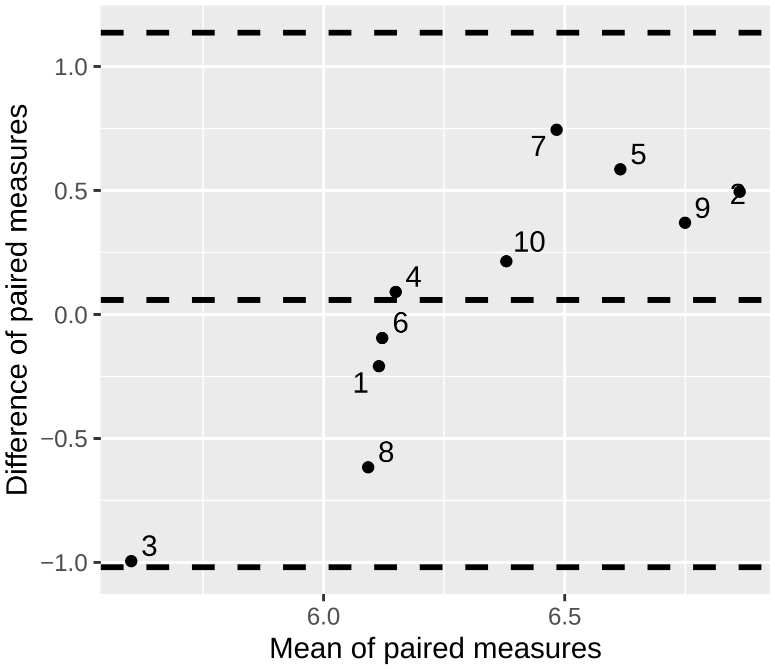


CT firstorder kurtosis 8,16 px

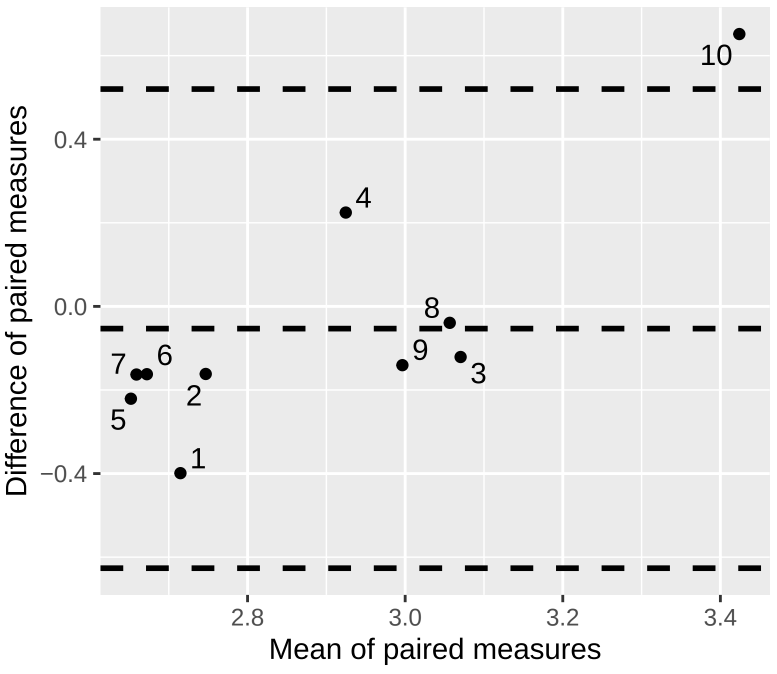


CT firstorder mean 8,16 px

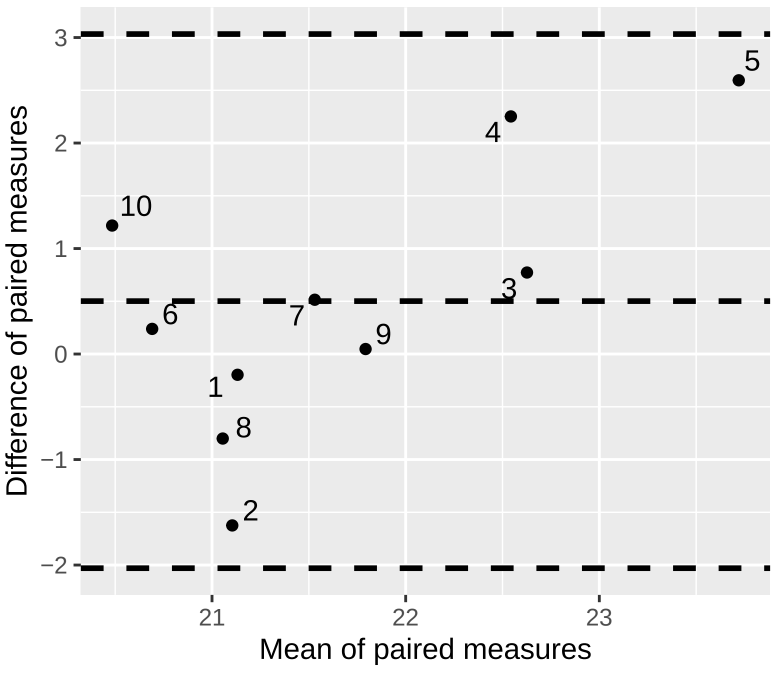

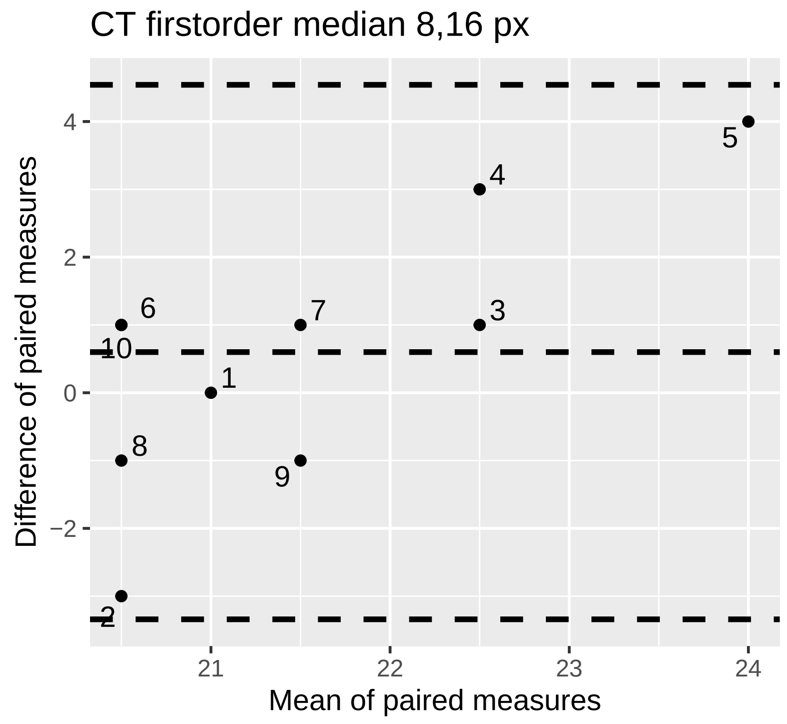
CT firstorder median 8,16 px
Difference of paired measures
Mean of paired measures

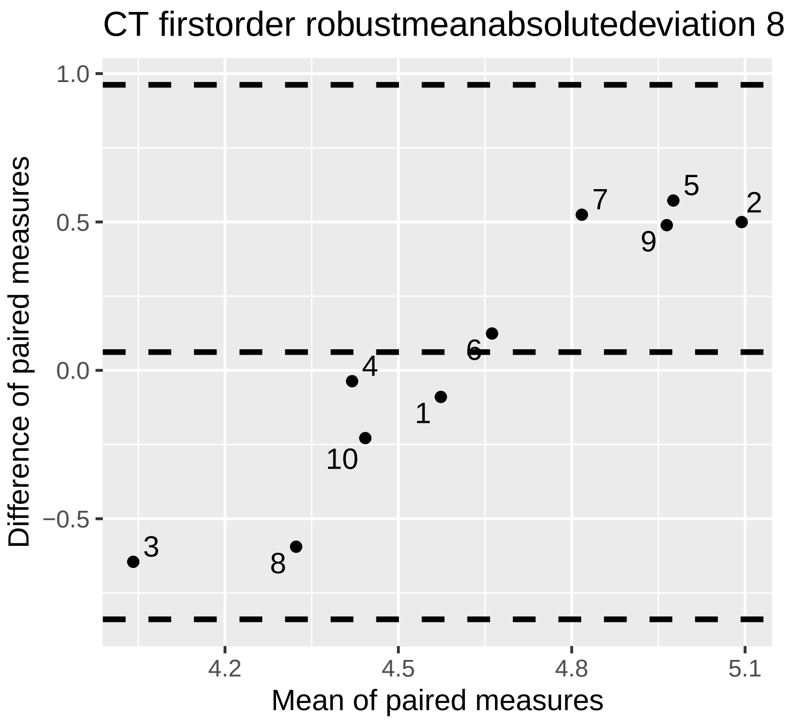
CT firstorder robustmeanabsolutedeviation 8
Difference of paired measures
Mean of paired measures

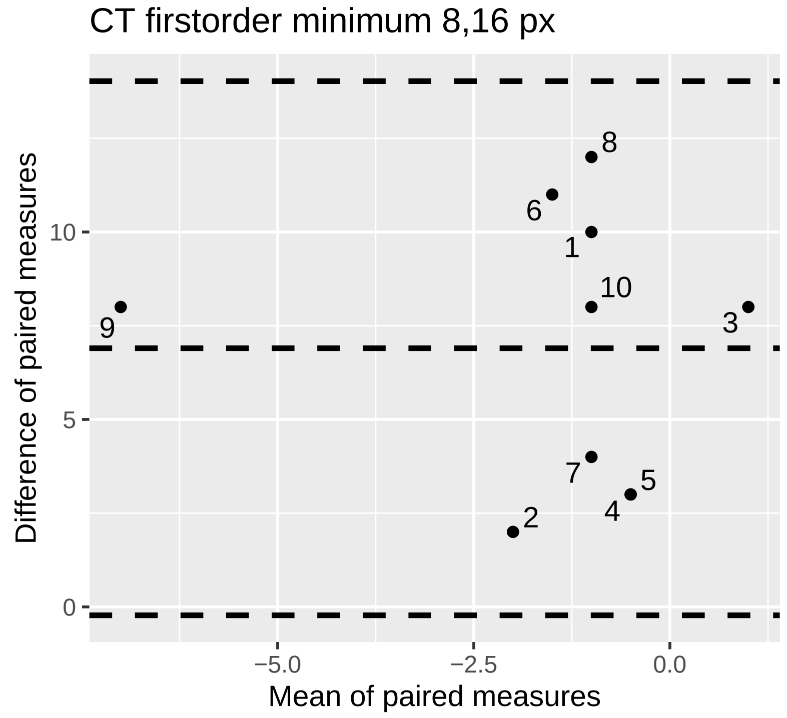
CT firstorder minimum 8,16 px
Difference of paired measures
Mean of paired measures

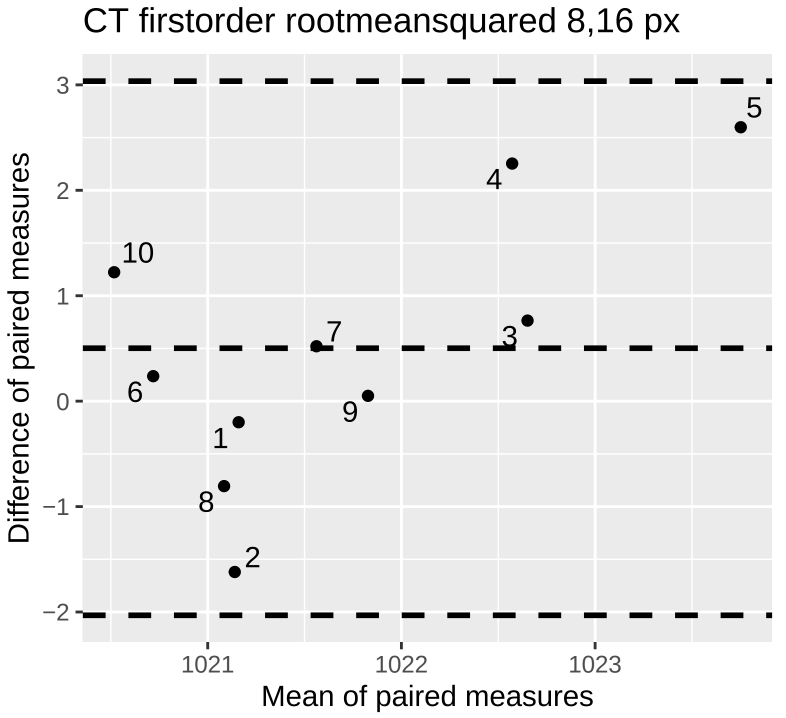
CT firstorder rootmeansquared 8,16 px
Difference of paired measures
Mean of paired measures

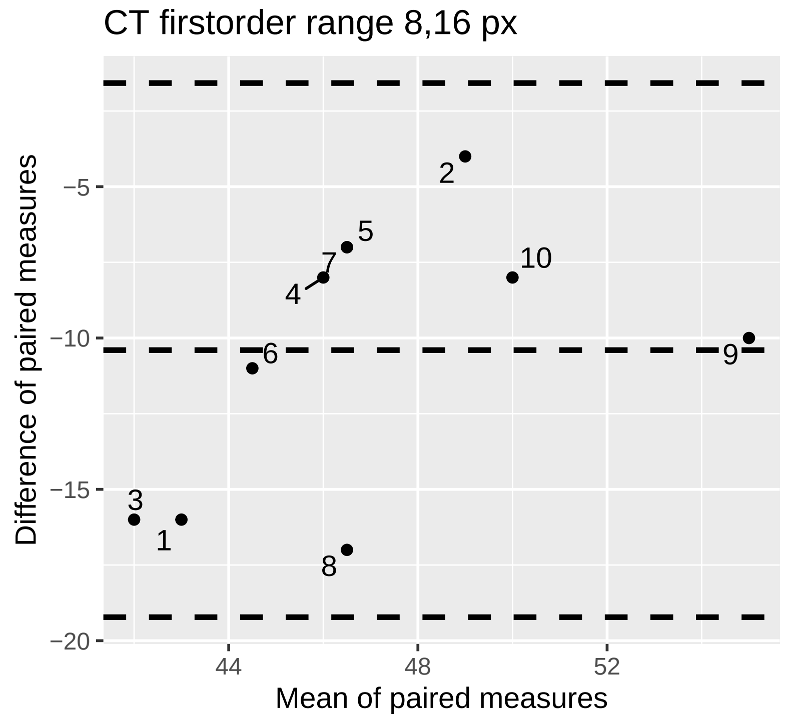
CT firstorder range 8,16 px
Difference of paired measures
Mean of paired measures

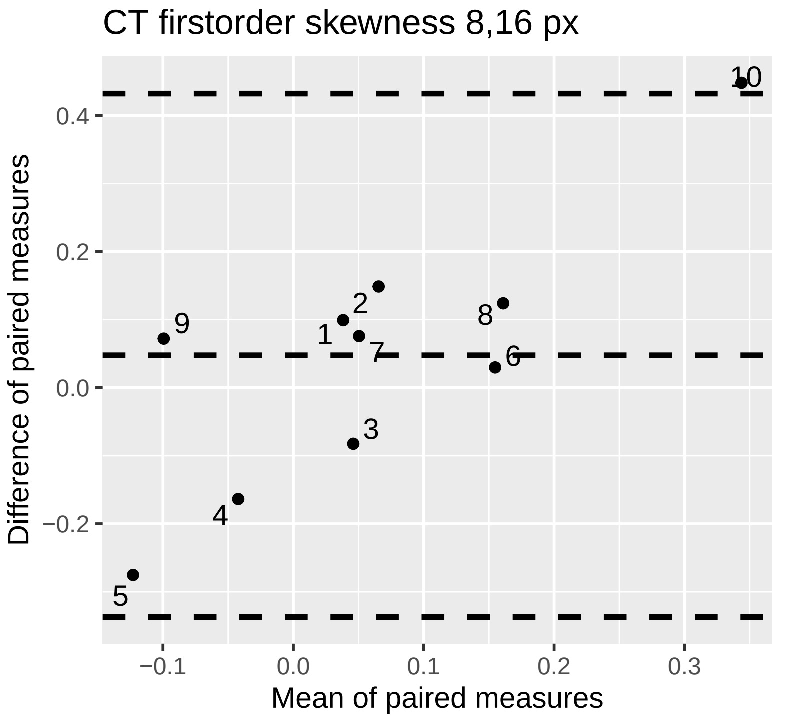
CT firstorder skewness 8,16 px
Difference of paired measures
Mean of paired measures

CT firstorder totalenergy 8,16 px

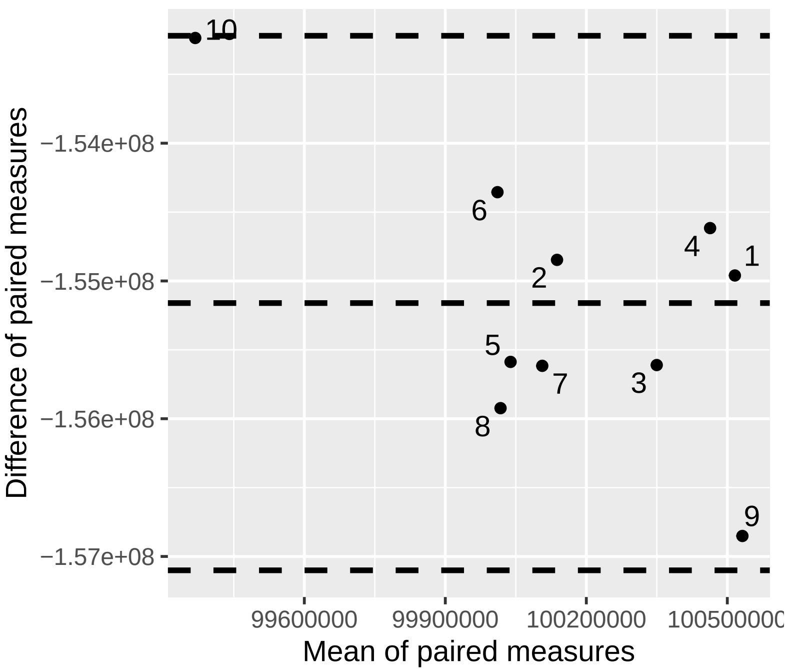


CT glcm autocorrelation 8,16 px

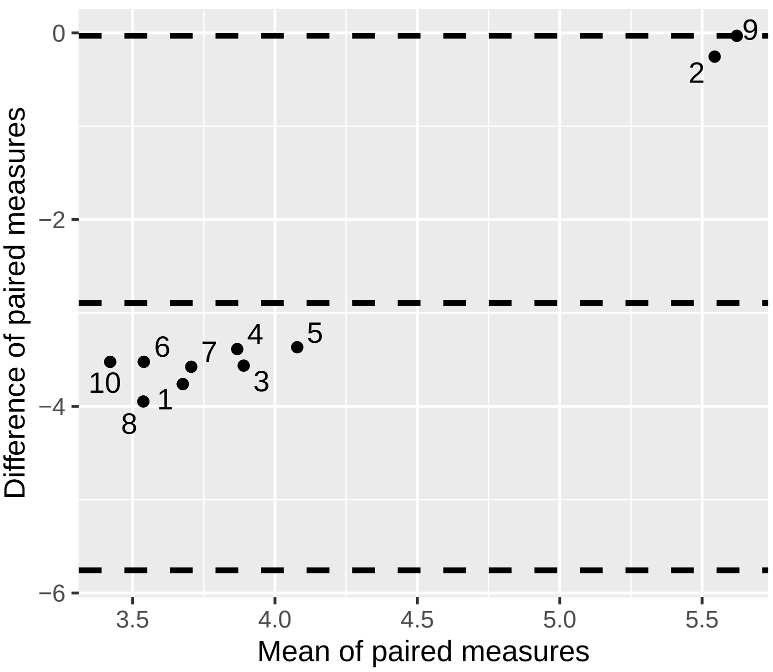


CT firstorder uniformity 8,16 px

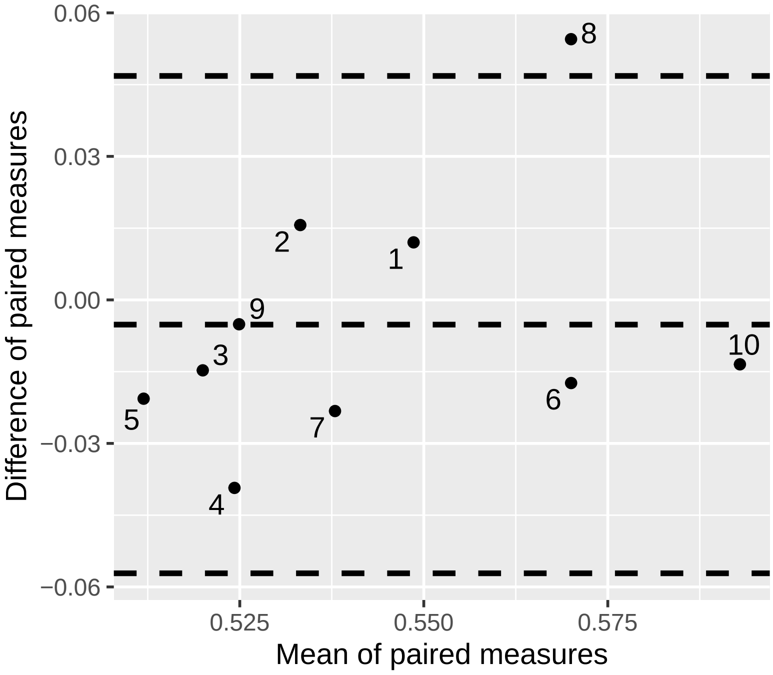


CT glcm clusterprominence 8,16 px

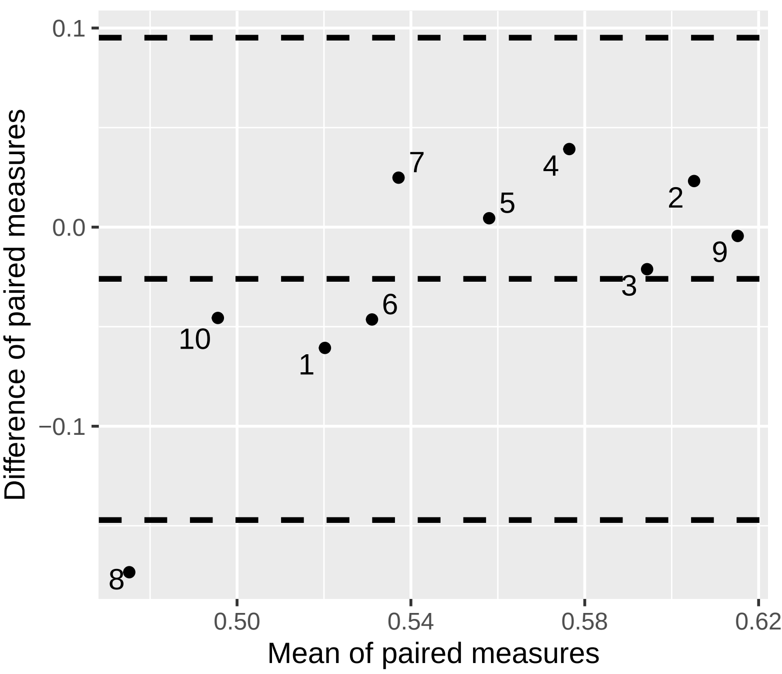


CT firstorder variance 8,16 px

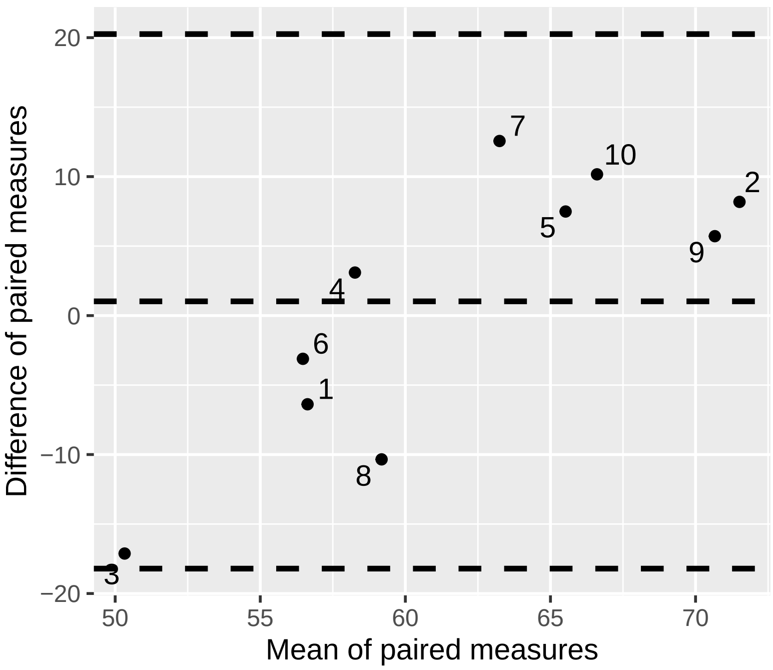


CT glcm clustershade 8,16 px

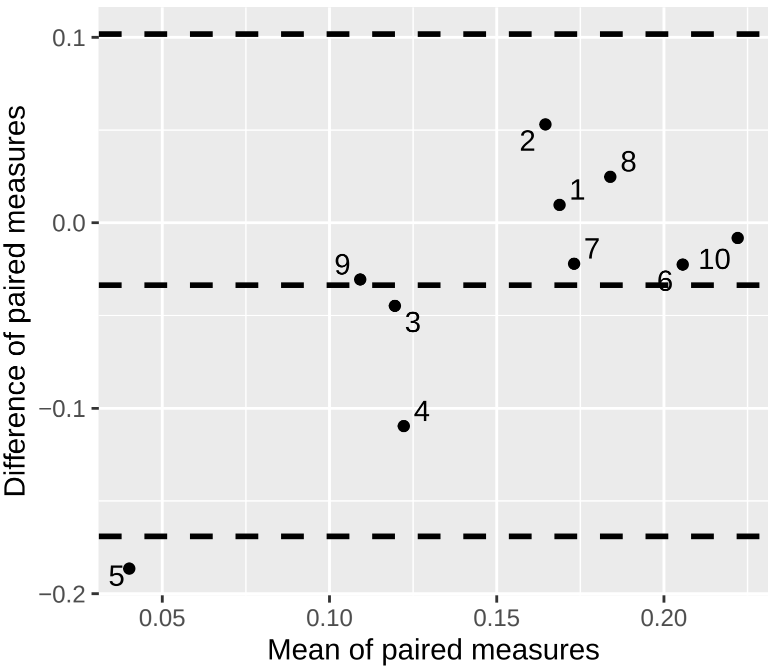

CT glcm clustertendency 8,16 px

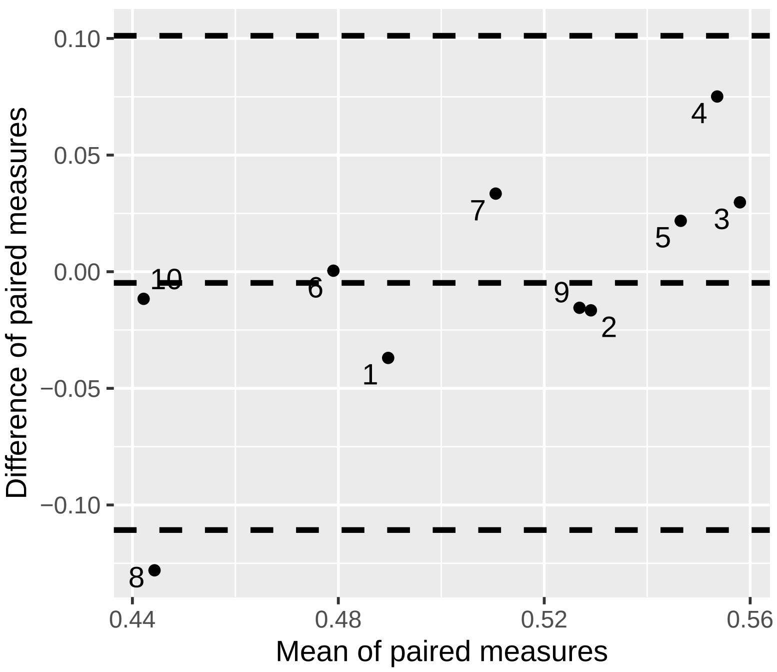


CT glcm differenceaverage 8,16 px

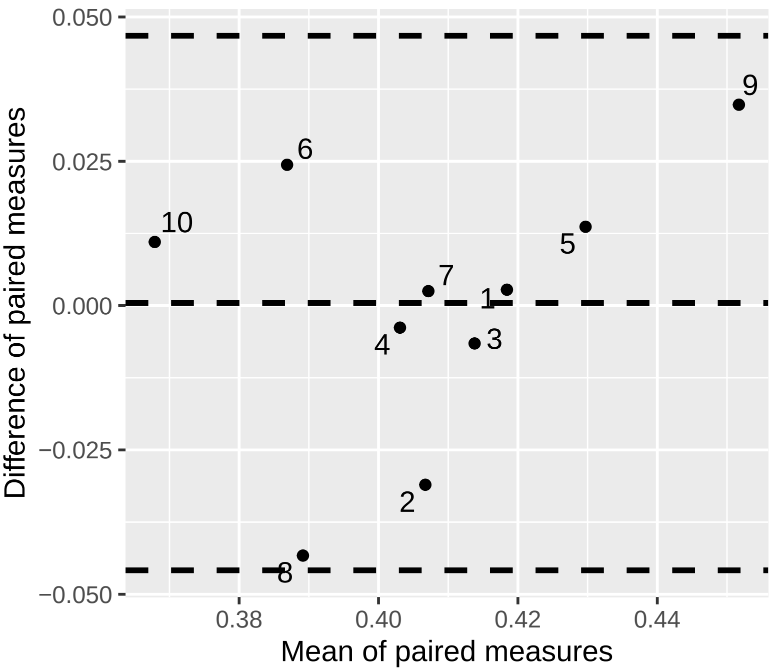


CT glcm contrast 8,16 px

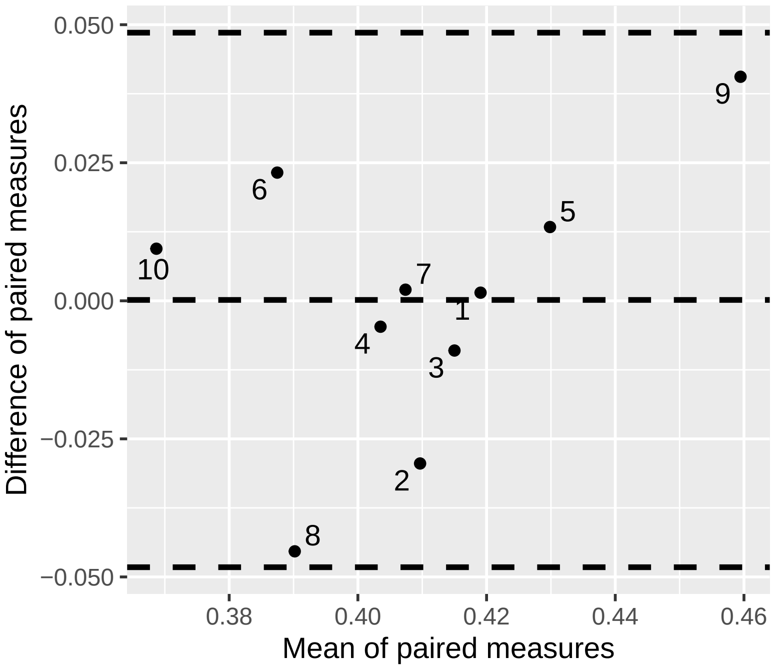


CT glcm differenceentropy 8,16 px

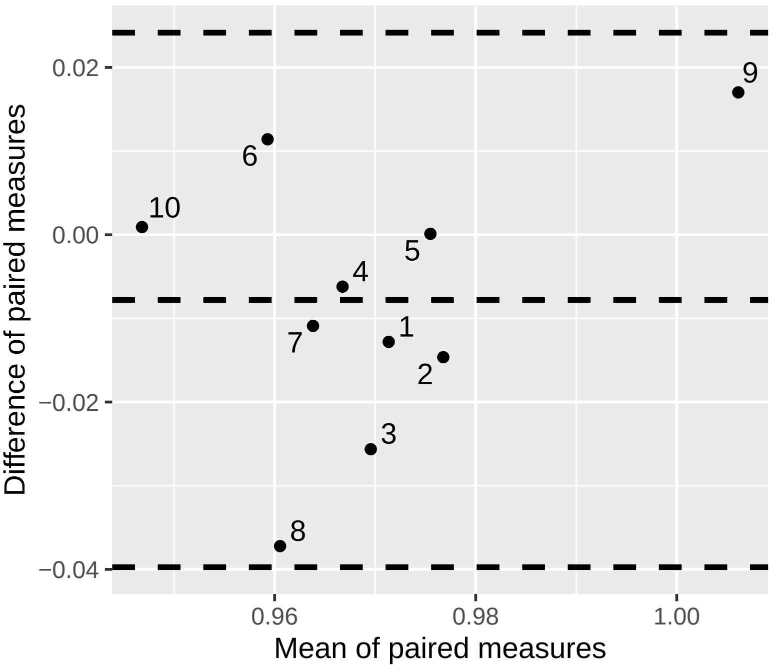


CT glcm correlation 8,16 px

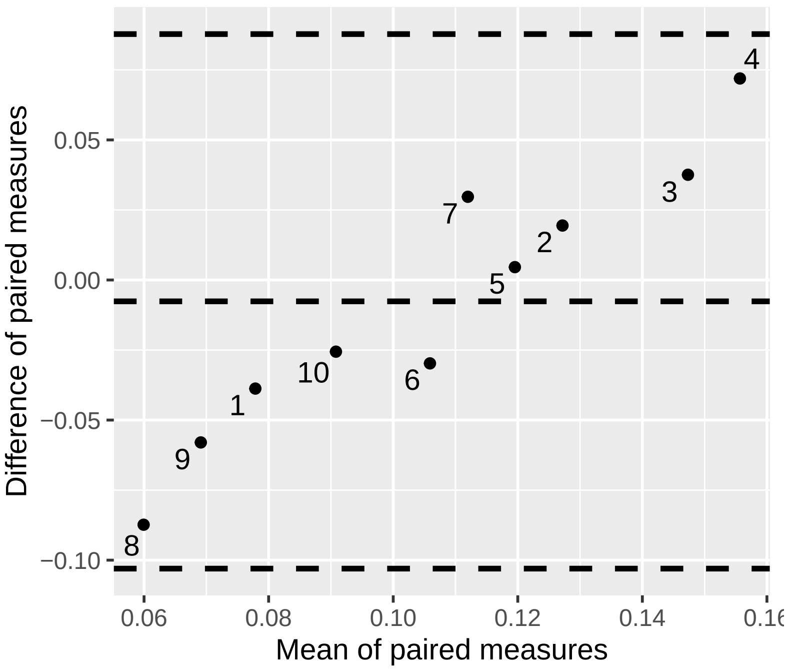


CT glcm differencevariance 8,16 px

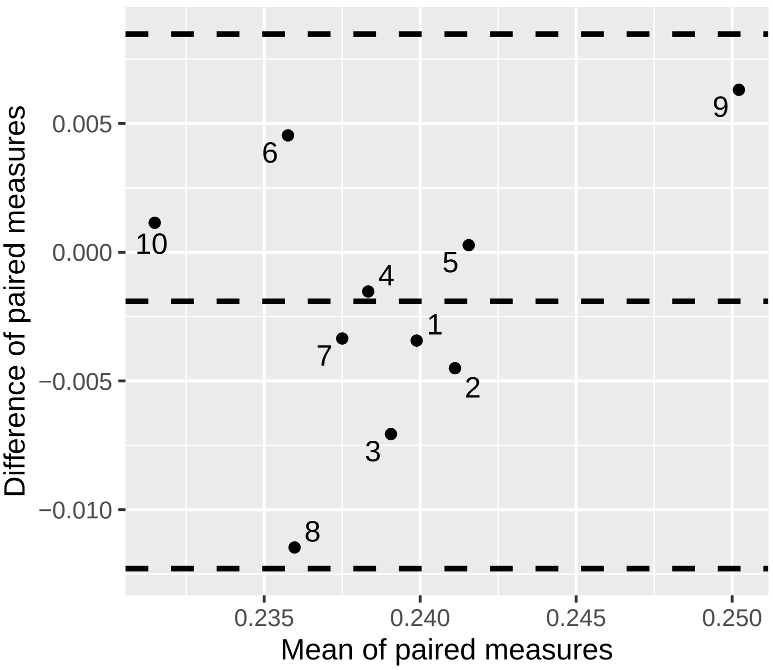

CT glcm id 8,16 px

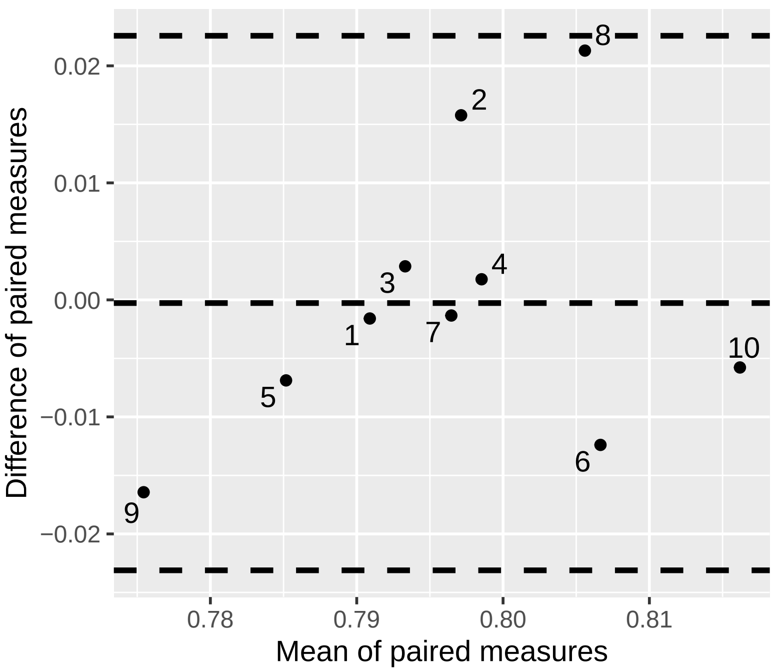


CT glcm idn 8,16 px

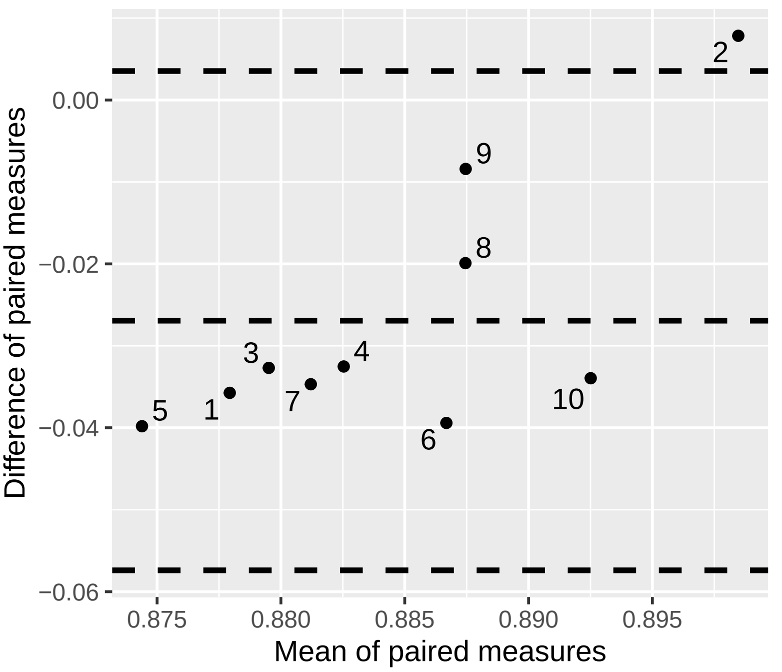


CT glcm idm 8,16 px

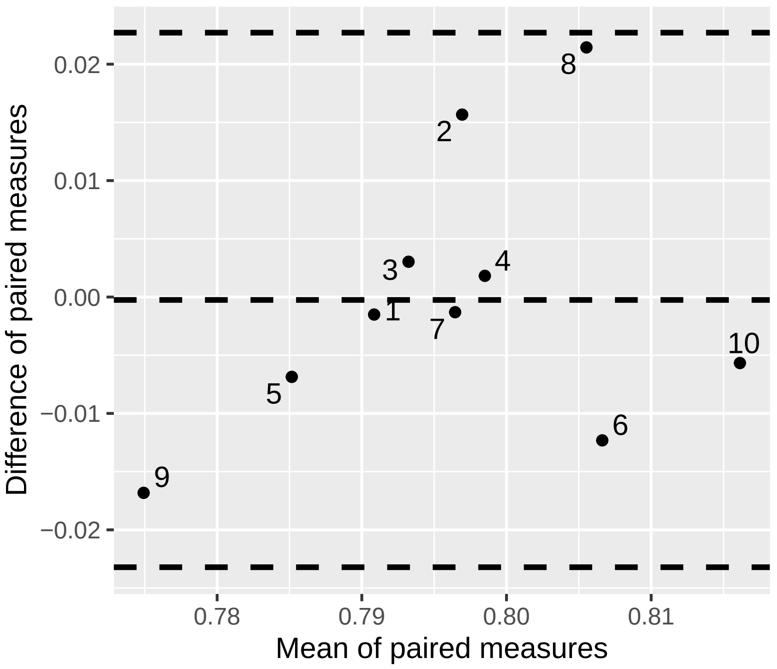


CT glcm imc1 8,16 px

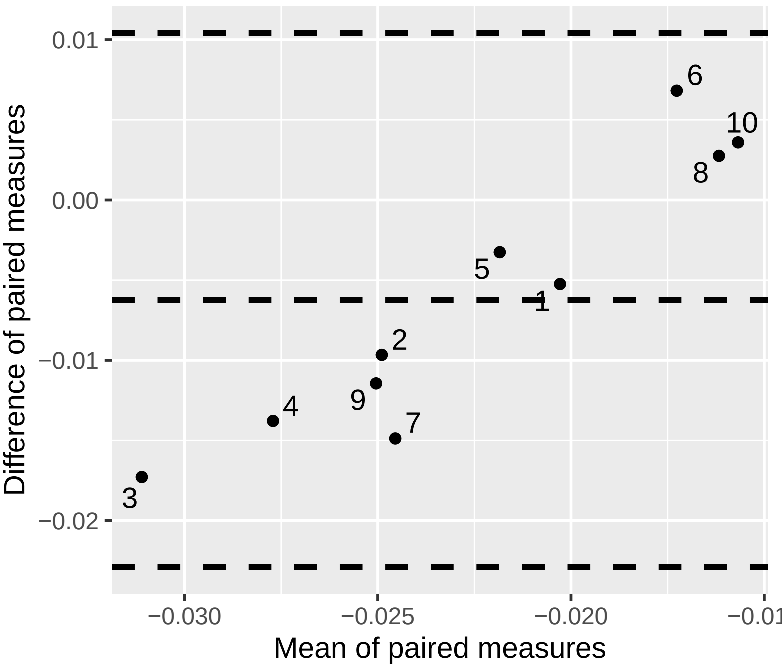


CT glcm idmn 8,16 px

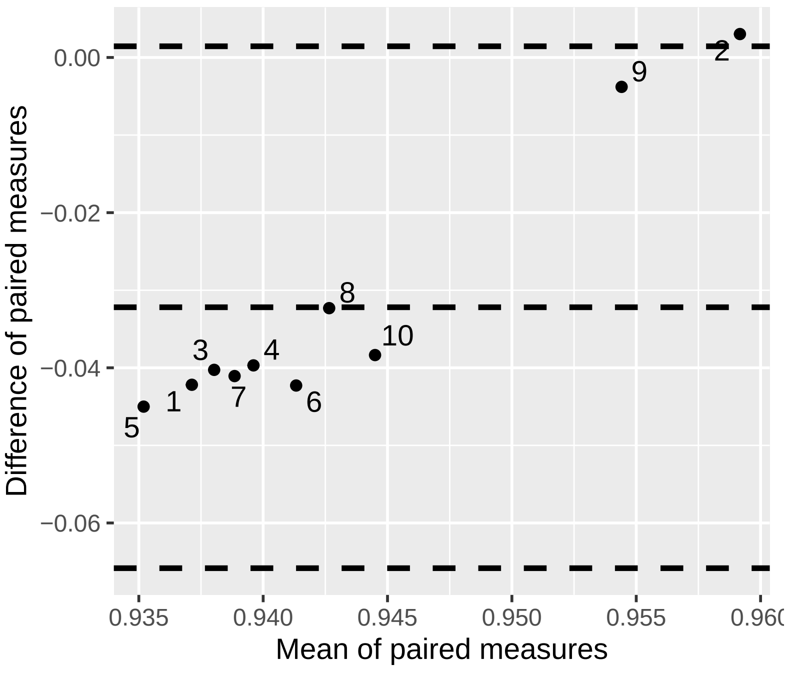


CT glcm imc2 8,16 px

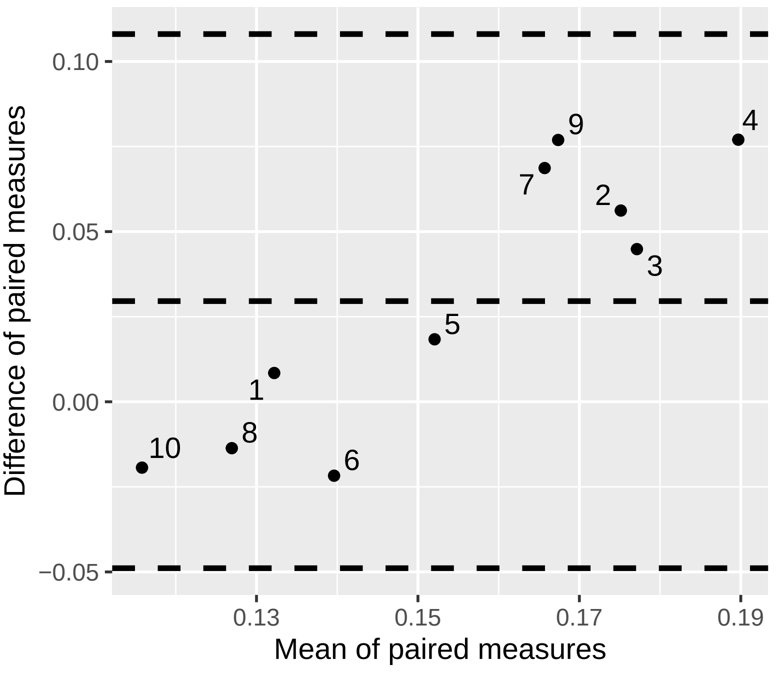

CT glcm inversevariance 8,16 px

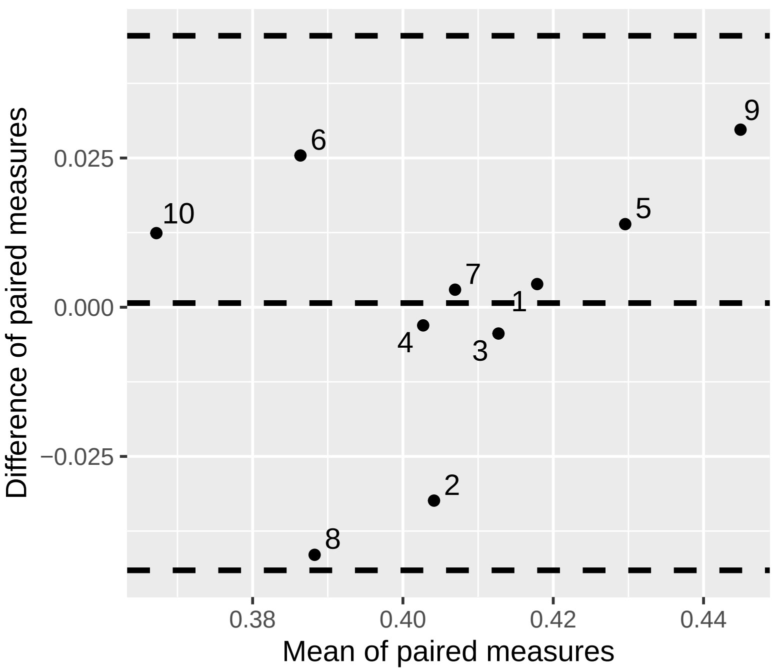


CT glcm jointentropy 8,16 px

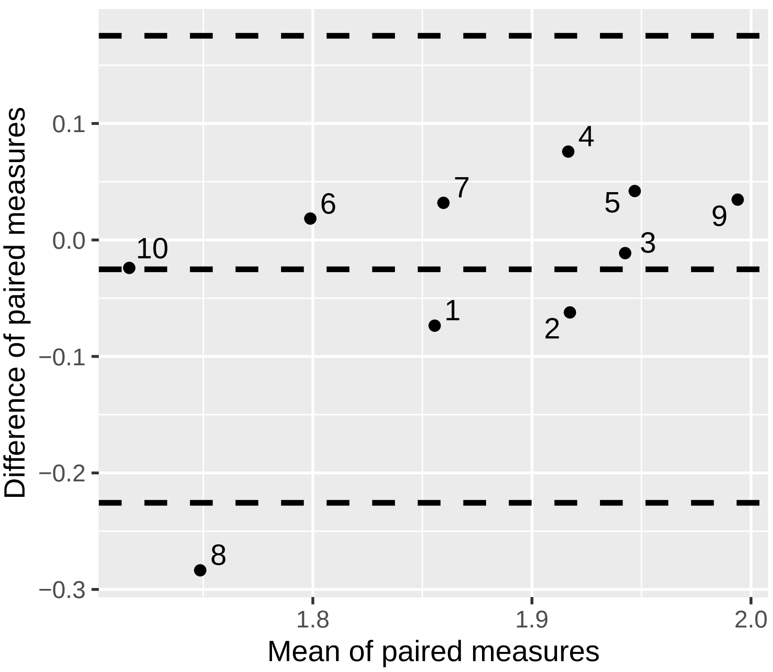


CT glcm jointaverage 8,16 px

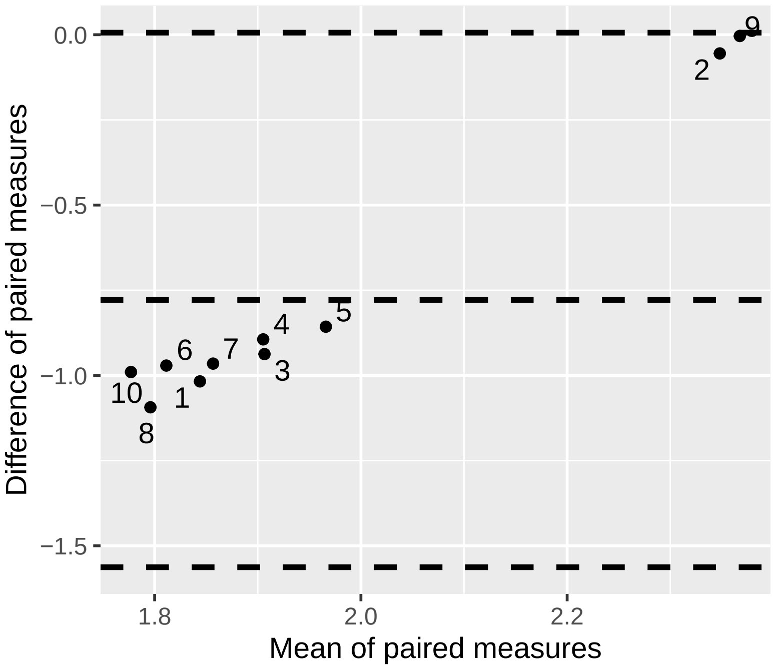


CT glcm mcc 8,16 px

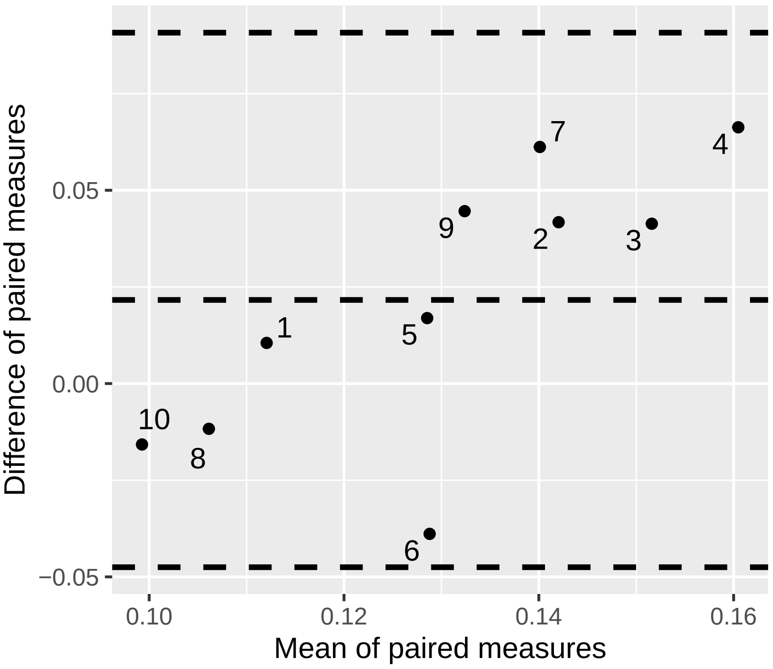


CT glcm jointenergy 8,16 px

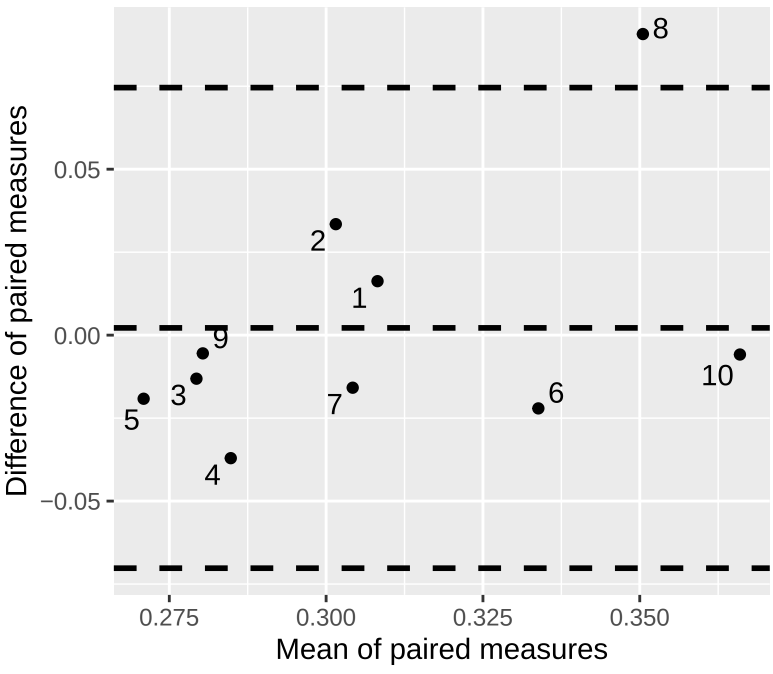


CT glcm maximumprobability 8,16 px

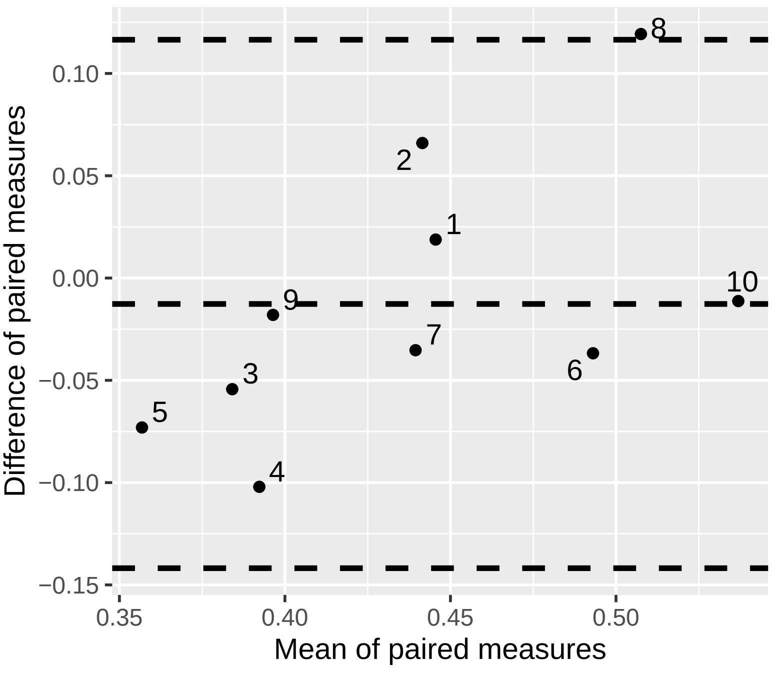

CT glcm sumaverage 8,16 px

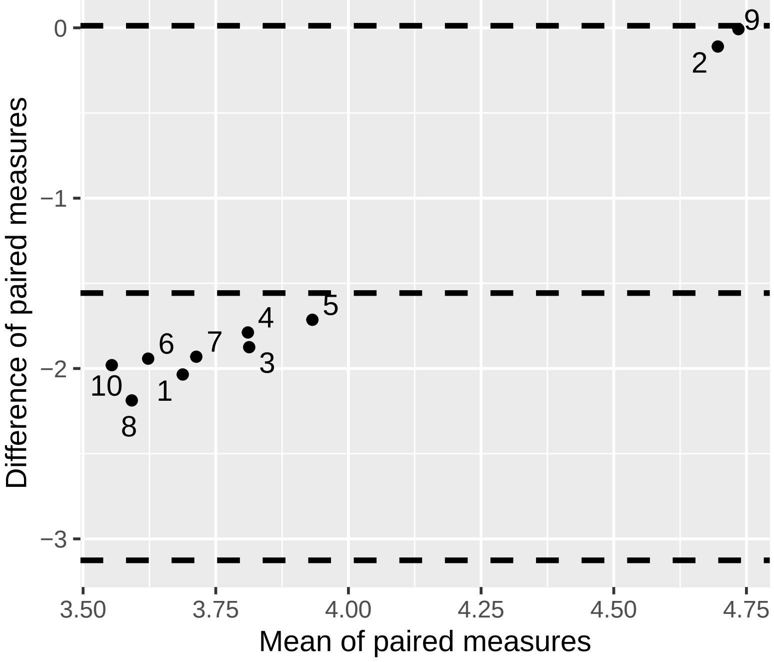


CT glrlm graylevelnonuniformity 8,16 px

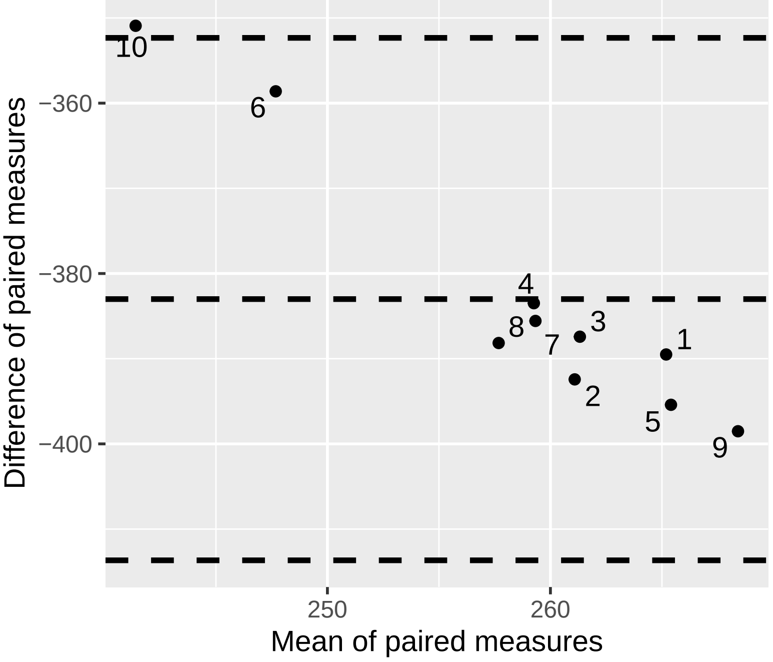


CT glcm sumentropy 8,16 px

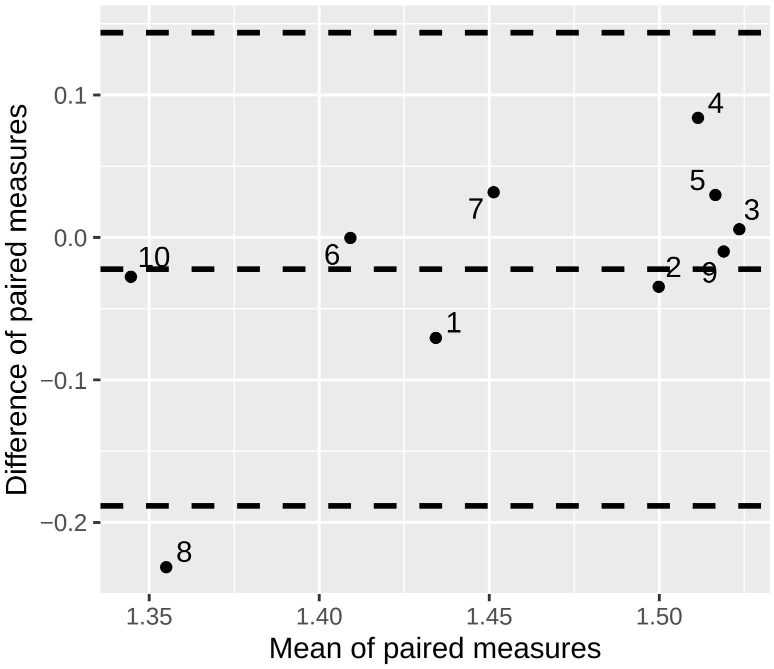


CT glrlm graylevelnonuniformitynormalized

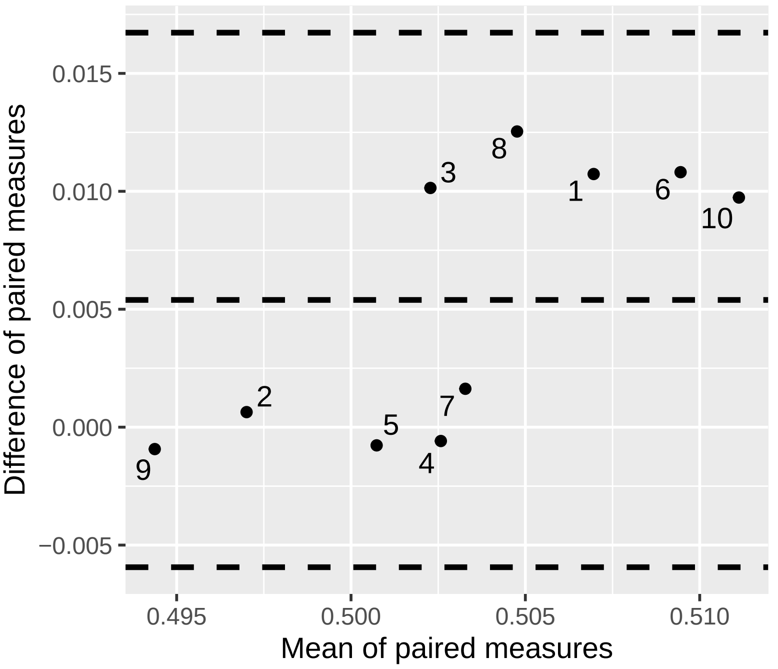


CT glcm sumsquares 8,16 px

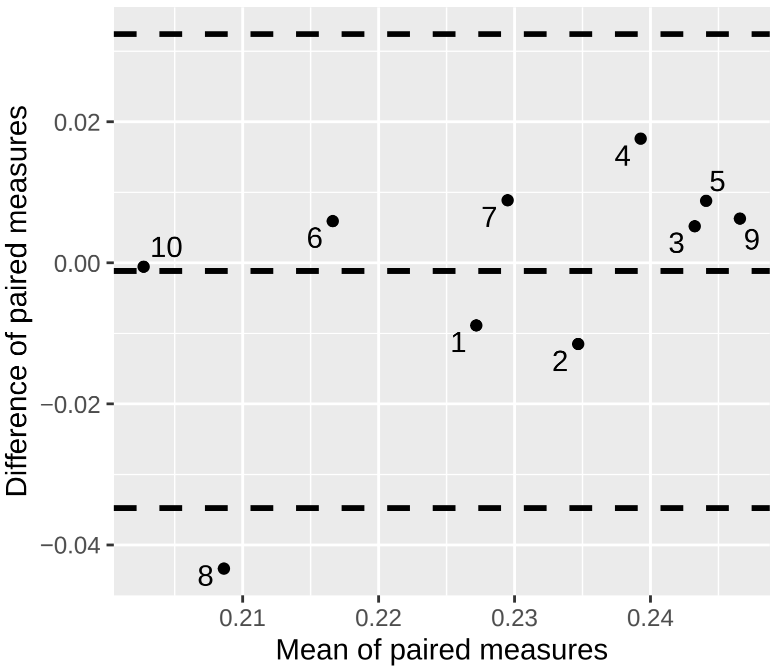


CT glrlm graylevelvariance 8,16 px

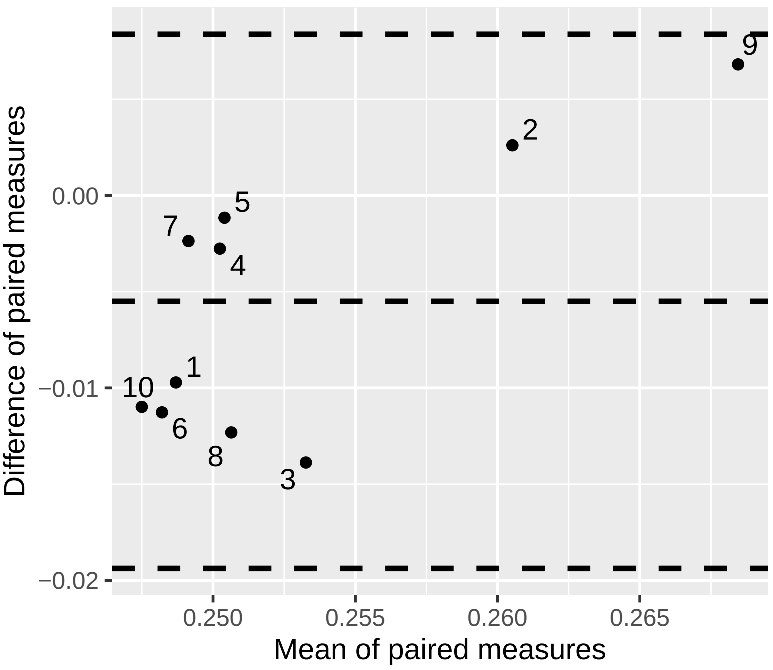

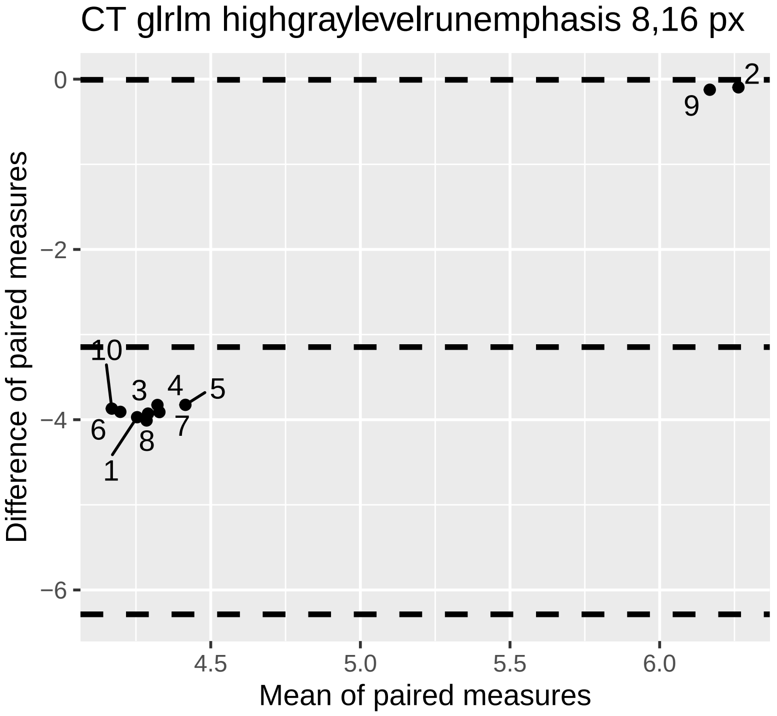
CT glrlm highgraylevelrunemphasis 8,16 px
Difference of paired measures
Mean of paired measures

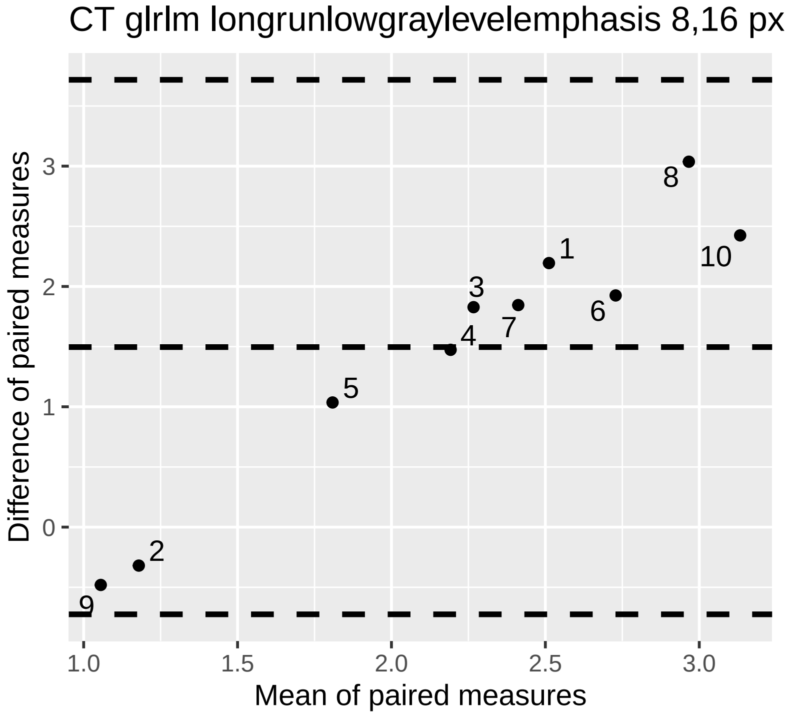
CT glrlm longrunlowgraylevelemphasis 8,16 px
Difference of paired measures
Mean of paired measures

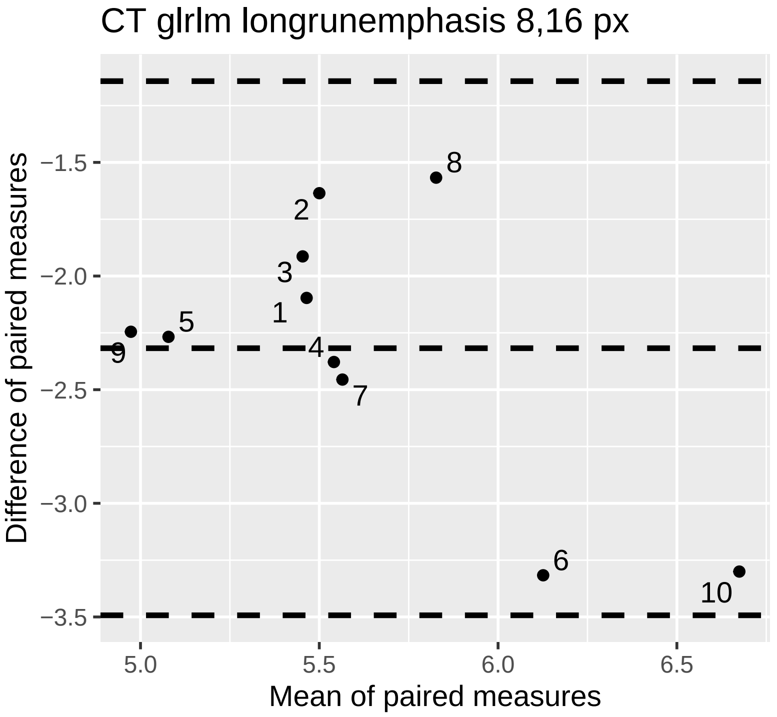
CT glrlm longrunemphasis 8,16 px
Difference of paired measures
Mean of paired measures

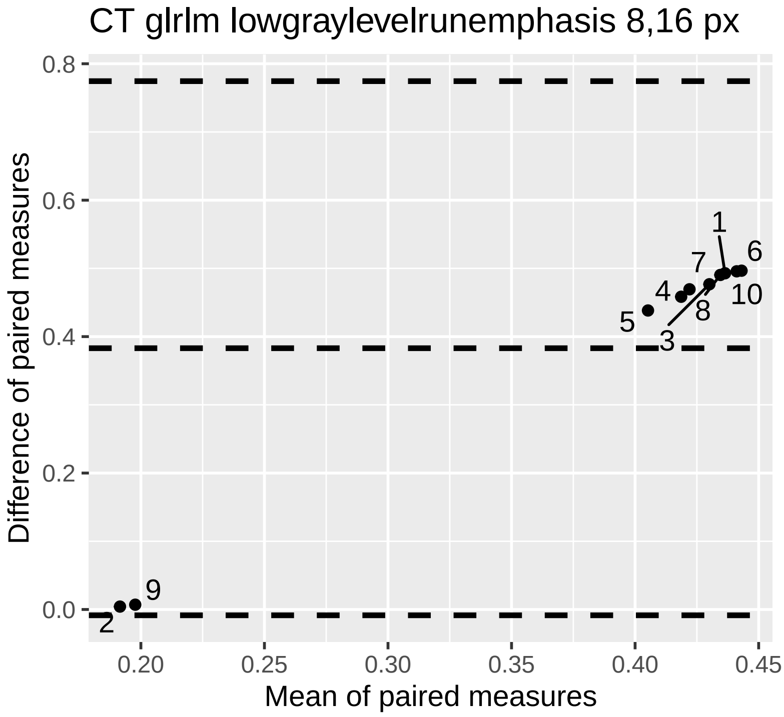
CT glrlm lowgraylevelrunemphasis 8,16 px
Difference of paired measures
Mean of paired measures

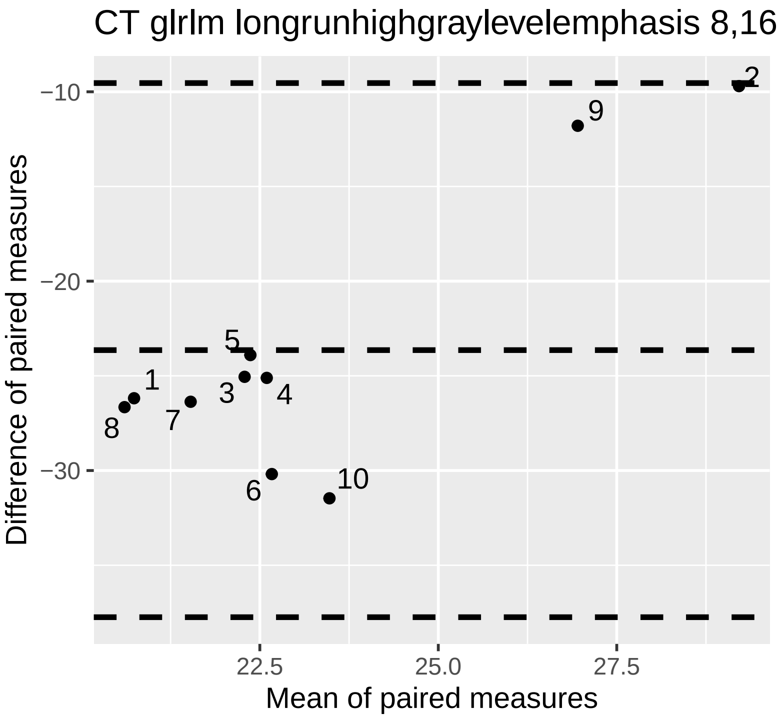
CT glrlm longrunhighgraylevelemphasis 8,16
Difference of paired measures
Mean of paired measures

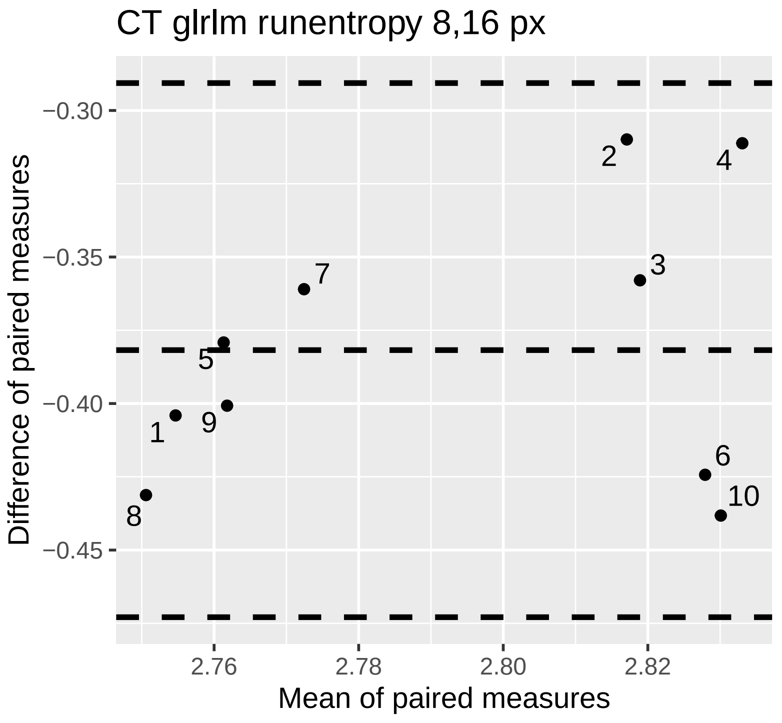
CT glrlm runentropy 8,16 px
Difference of paired measures
Mean of paired measures

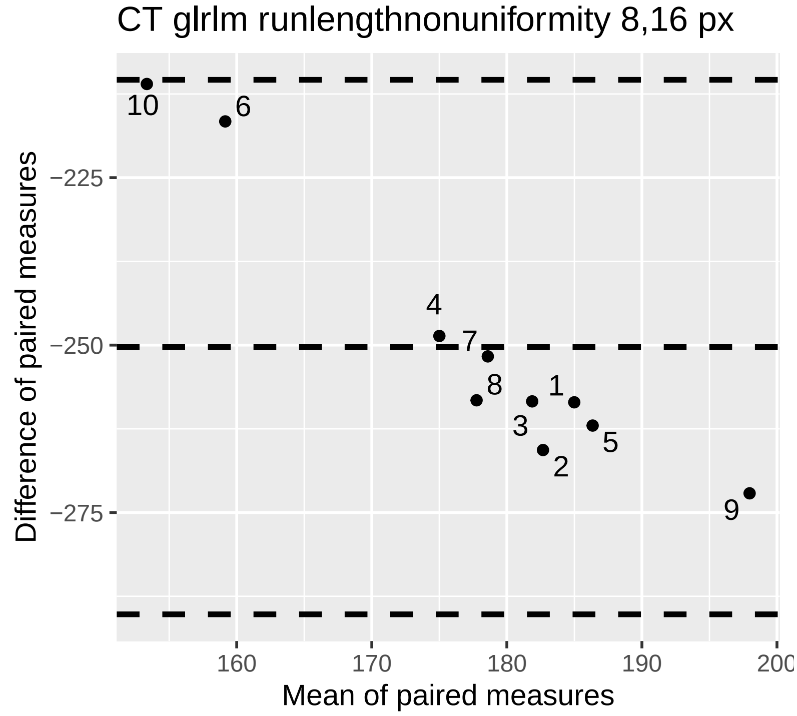
CT glrlm runlengthnonuniformity 8,16 px
Difference of paired measures
Mean of paired measures
−225
−250
−275
160
170
180
190
200
10
6
4
7
8
1
3
2
5
9

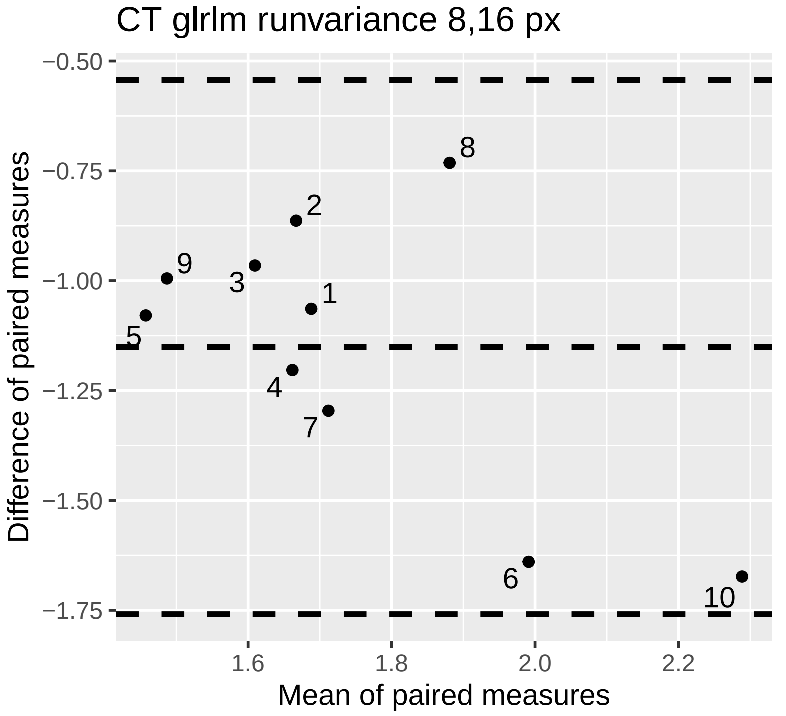
CT glrlm runvariance 8,16 px
Difference of paired measures
Mean of paired measures
−0.50
−0.75
−1.00
−1.25
−1.50
−1.75
1.6
1.8
2.0
2.2
8
2
9
3
1
5
4
7
6
10

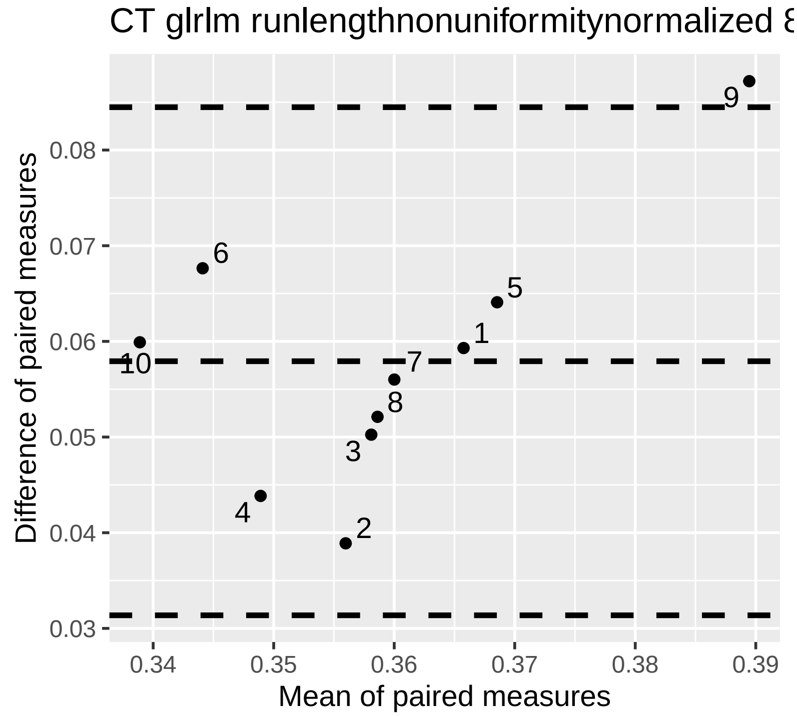
CT glrlm runlengthnonuniformitynormalized 8
Difference of paired measures
Mean of paired measures
0.08
0.07
0.06
0.05
0.04
0.03
0.34
0.35
0.36
0.37
0.38
0.39
9
6
5
1
10
7
8
3
4
2

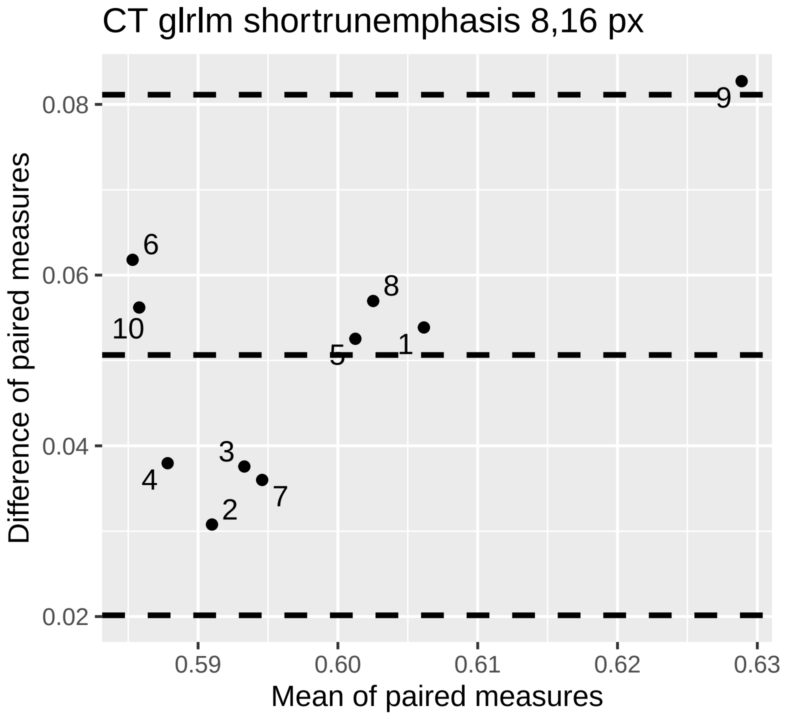
CT glrlm shortrunemphasis 8,16 px
Difference of paired measures
Mean of paired measures
0.08
0.06
0.04
0.02
0.59
0.60
0.61
0.62
0.63
9
6
8
10
5
1
3
4
7
2

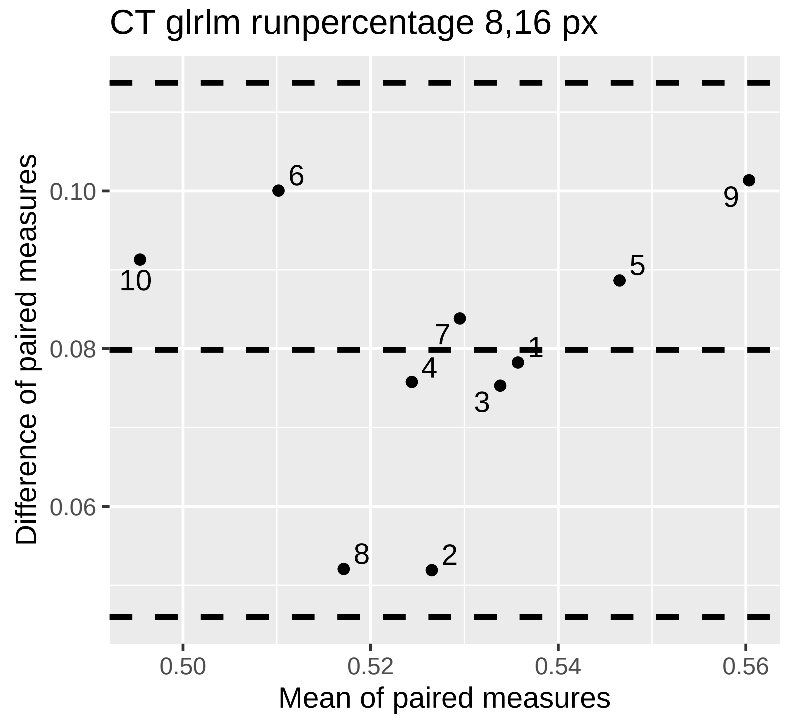
CT glrlm runpercentage 8,16 px
Difference of paired measures
Mean of paired measures
0.10
0.08
0.06
0.50
0.52
0.54
0.56
6
9
10
5
7
1
4
3
8
2

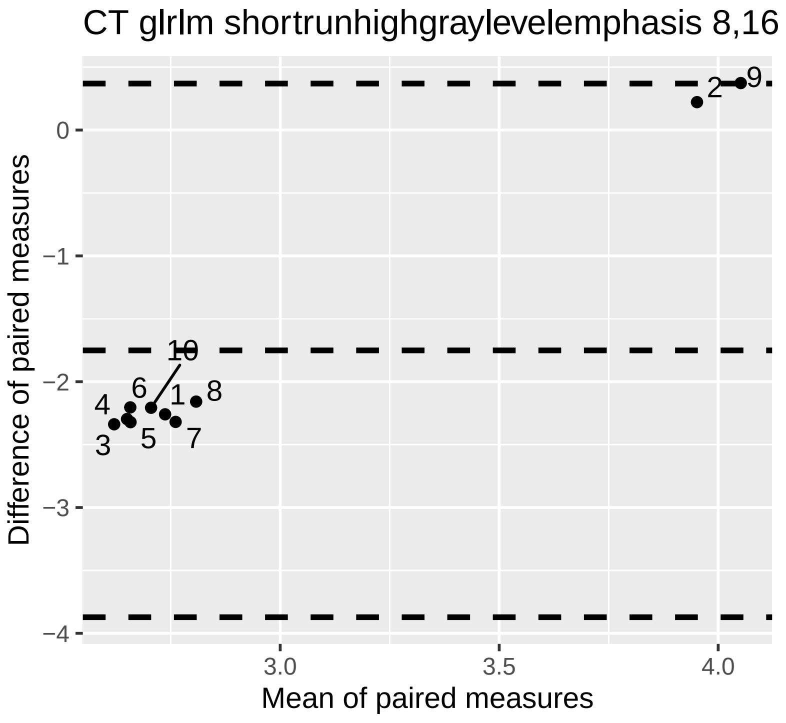
CT glrlm shortrunhighgraylevelemphasis 8,16
Difference of paired measures
Mean of paired measures
0
−1
−2
−3
−4
3.0
3.5
4.0
2
9
10
6
4
1
8
3
5
7

CT glrlm shortrunlowgraylevelemphasis 8,16 px

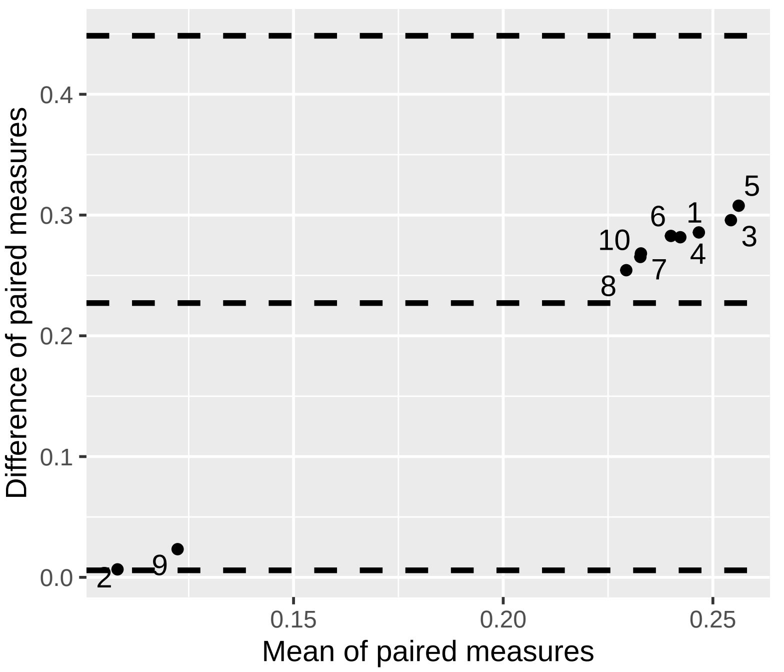


CT glszm graylevelvariance 8,16 px

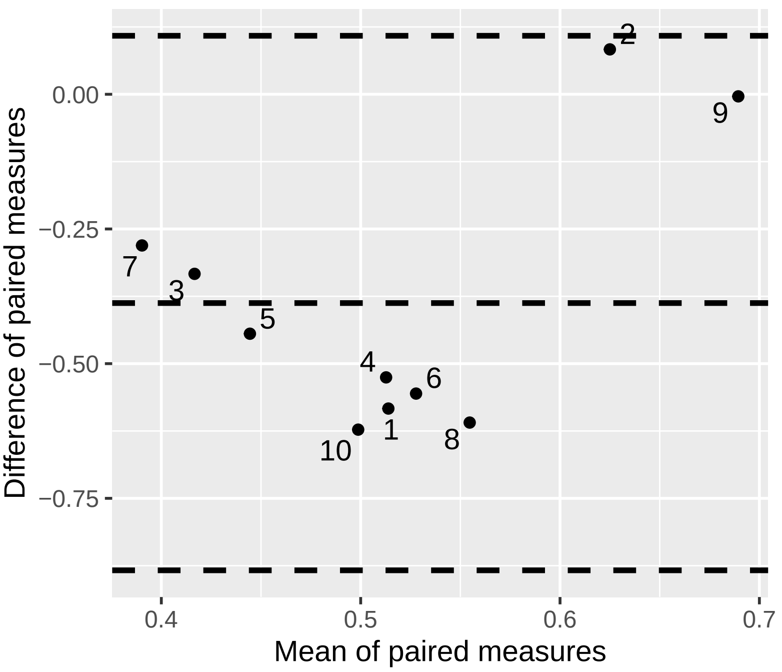


CT glszm graylevelnonuniformity 8,16 px

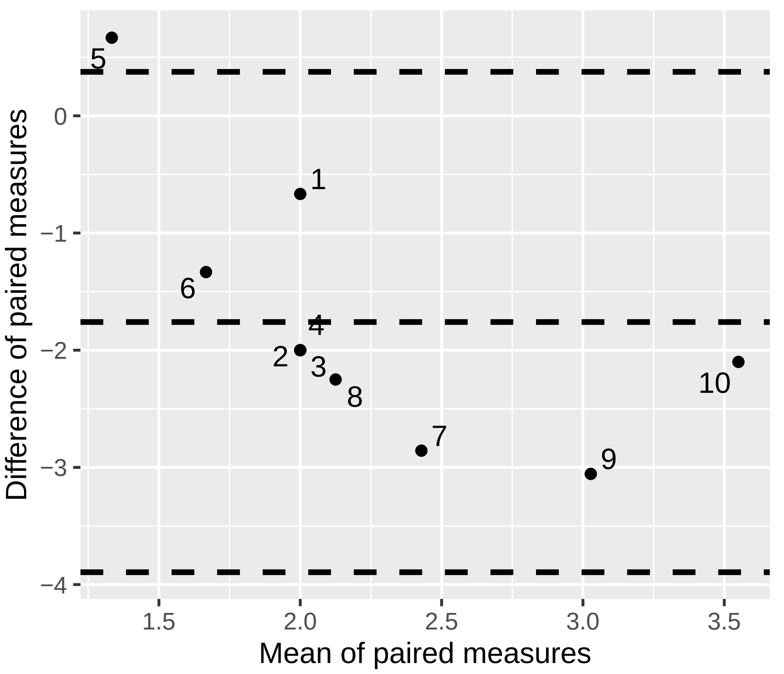


CT glszm highgraylevelzoneemphasis 8,16 px

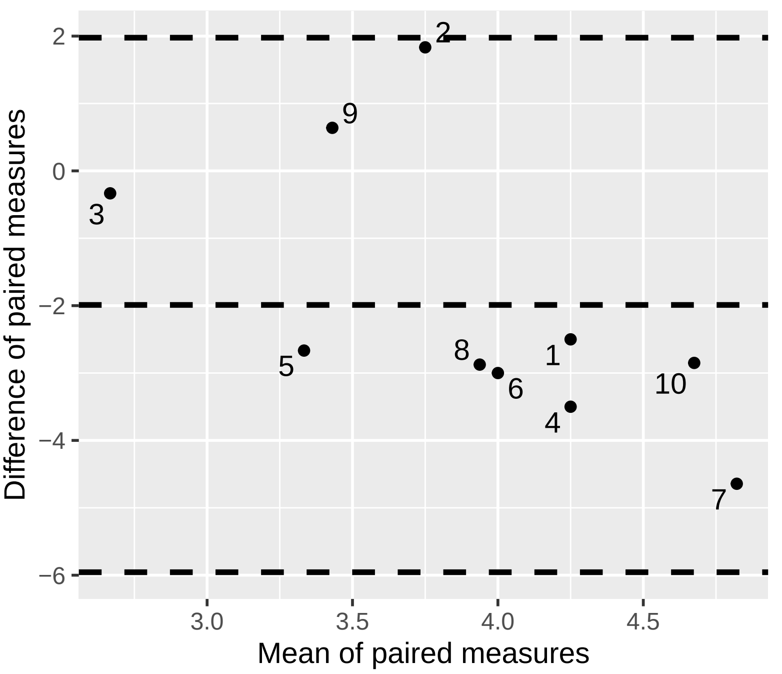


CT glszm graylevelnonuniformitynormalized 8

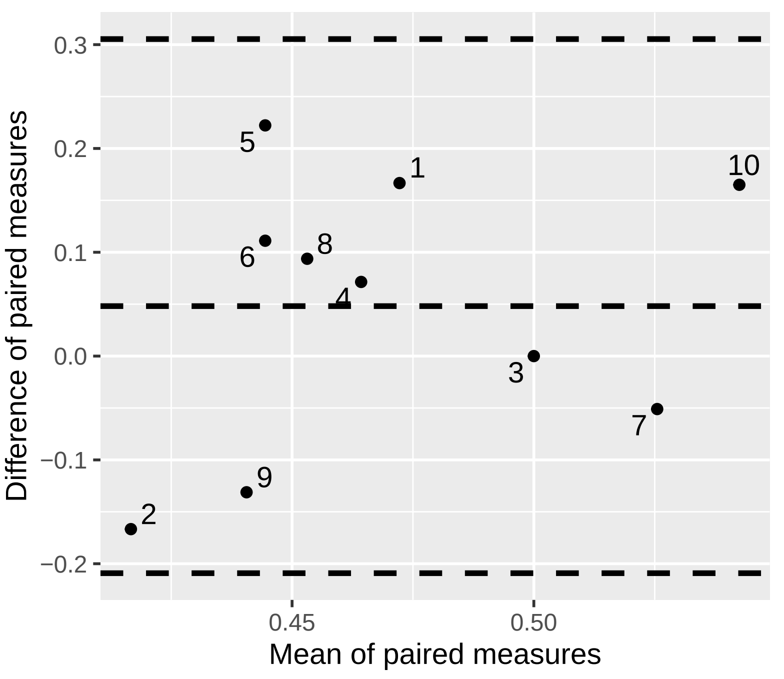


CT glszm largeareaemphasis 8,16 px

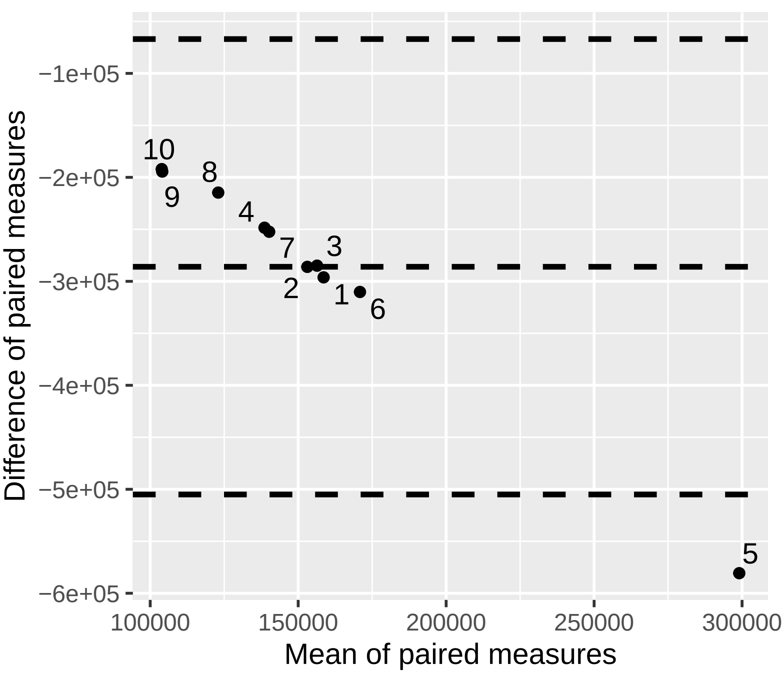

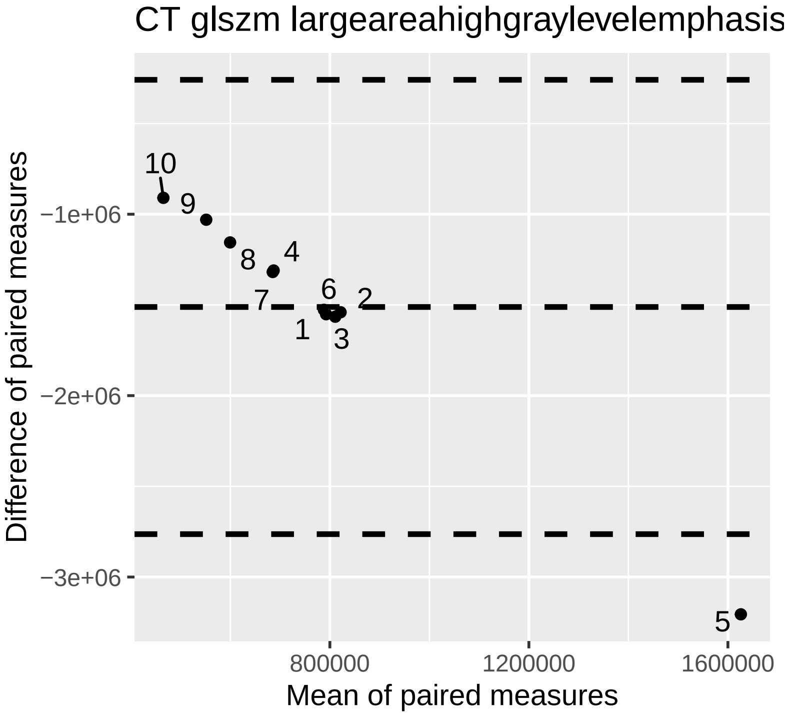
CT glszm largeareahighgraylevelemphasis
Difference of paired measures
−1e+06
−2e+06
−3e+06
800000
1200000
1600000
Mean of paired measures

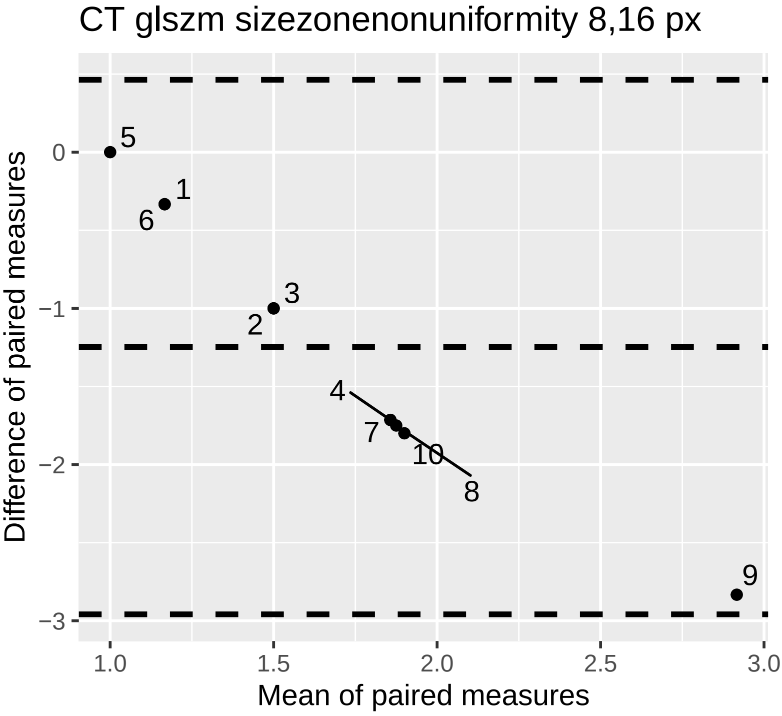
CT glszm sizezonenonuniformity 8,16 px
Difference of paired measures
0
−1
−2
−3
1.0
1.5
2.0
2.5
3.0
Mean of paired measures

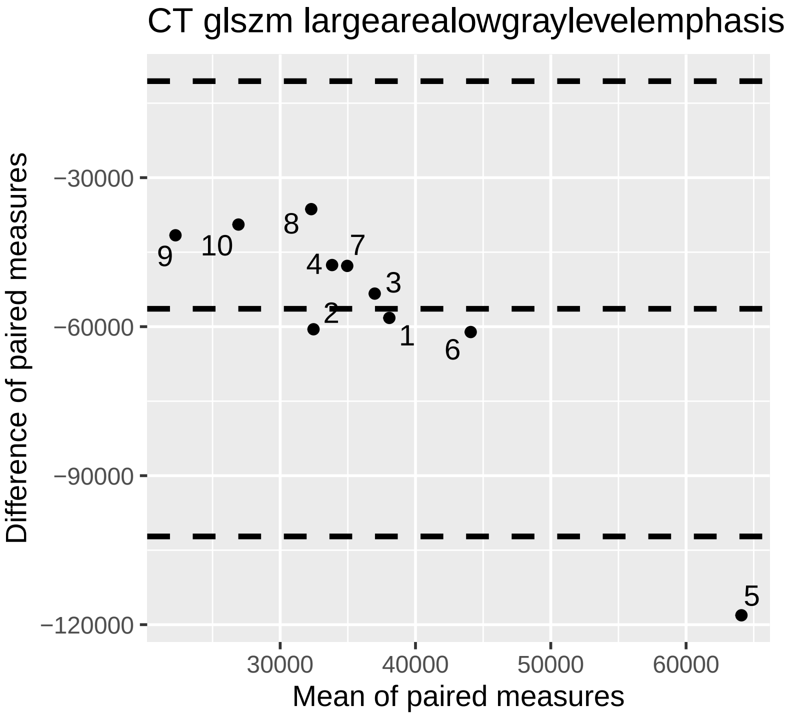
CT glszm largearealowgraylevelemphasis
Difference of paired measures
−30000
−60000
−90000
−120000
30000
40000
50000
60000
Mean of paired measures

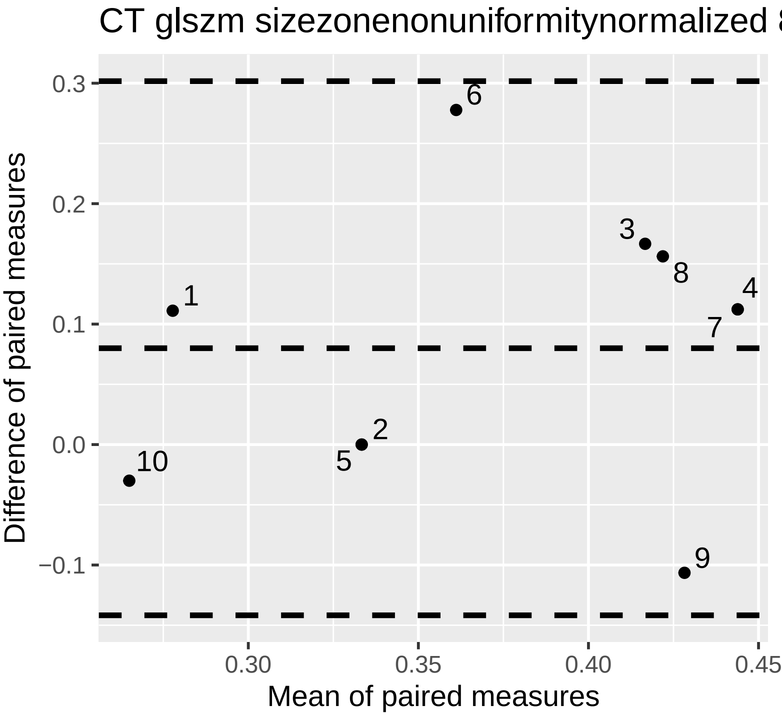
CT glszm sizezonenonuniformitynormalized 8
Difference of paired measures
0.3
0.2
0.1
0.0
−0.1
0.30
0.35
0.40
0.45
Mean of paired measures

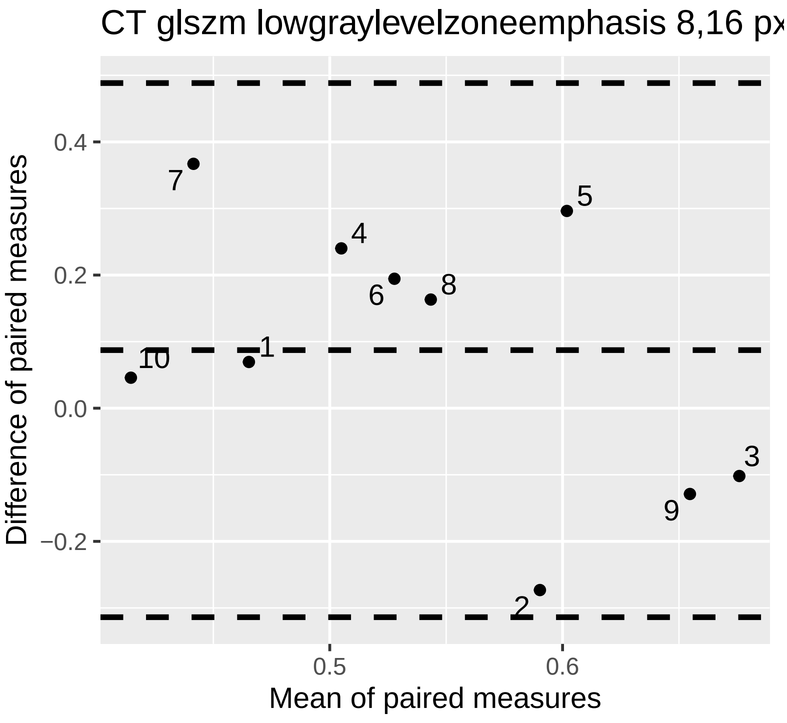
CT glszm lowgraylevelzoneemphasis 8,16 px
Difference of paired measures
0.4
0.2
0.0
−0.2
0.5
0.6
Mean of paired measures

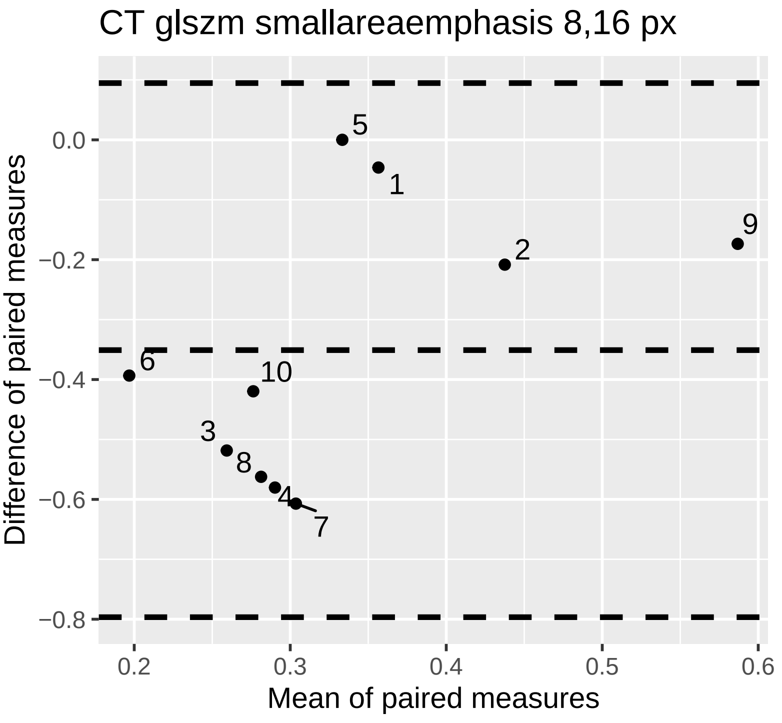
CT glszm smallareaemphasis 8,16 px
Difference of paired measures
0.0
−0.2
−0.4
−0.6
−0.8
0.2
0.3
0.4
0.5
0.6
Mean of paired measures

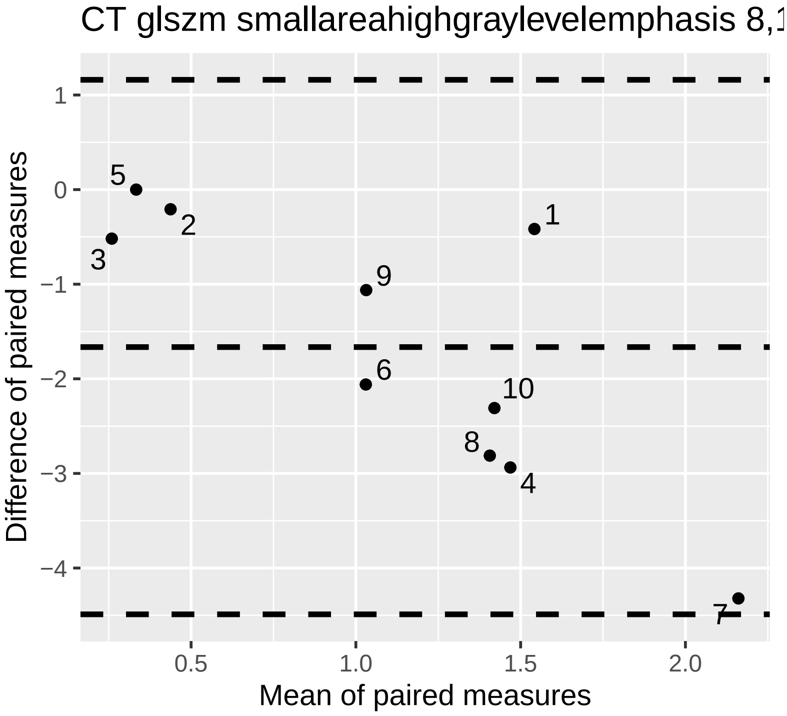
CT glszm smallareahighgraylevelemphasis 8,1
Difference of paired measures
Mean of paired measures

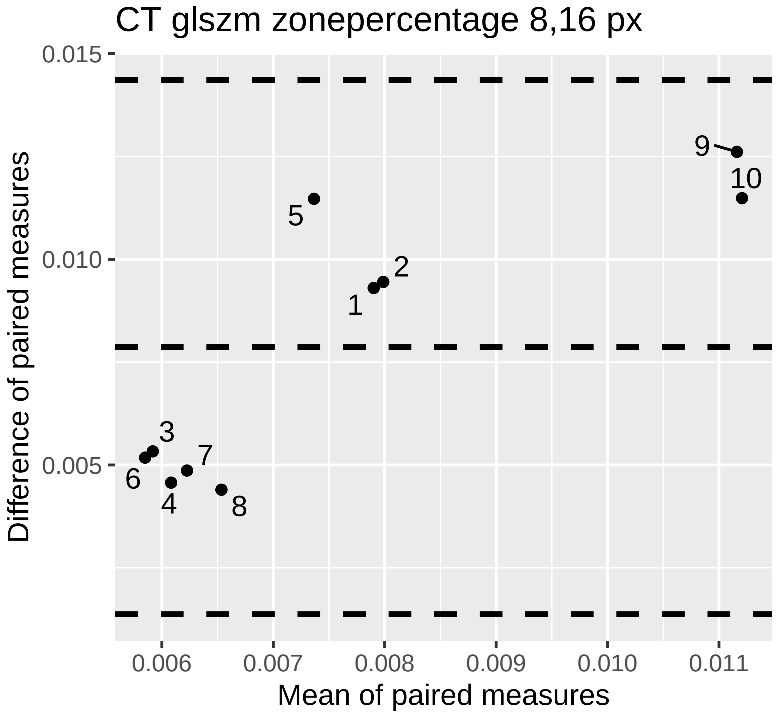
CT glszm zonepercentage 8,16 px
Difference of paired measures
Mean of paired measures

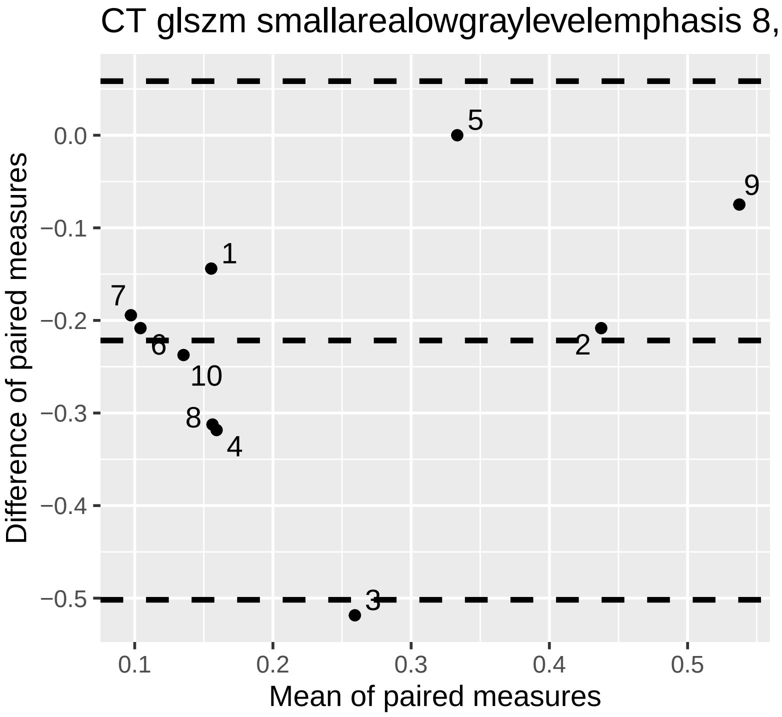
CT glszm smallarealowgraylevelemphasis 8,
Difference of paired measures
Mean of paired measures

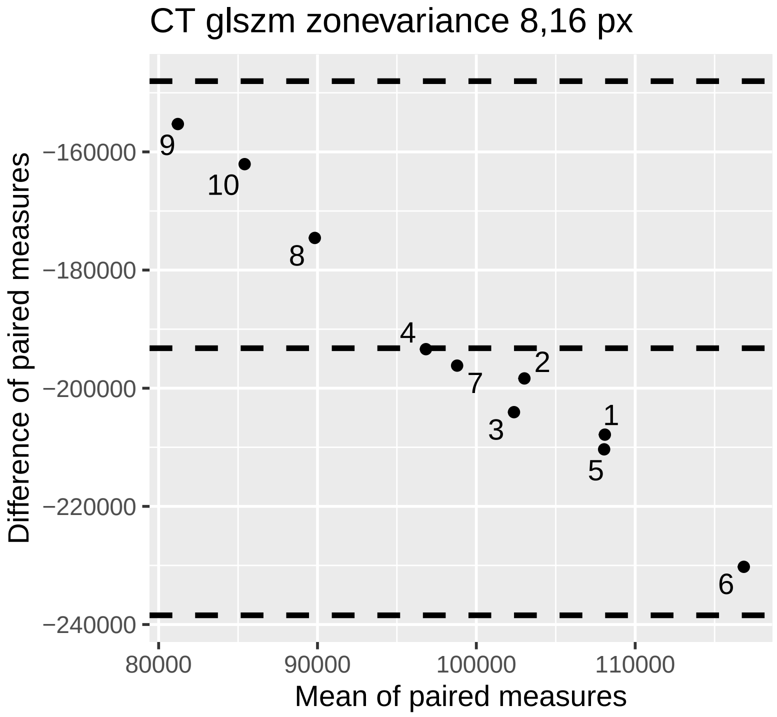
CT glszm zonevariance 8,16 px
Difference of paired measures
Mean of paired measures

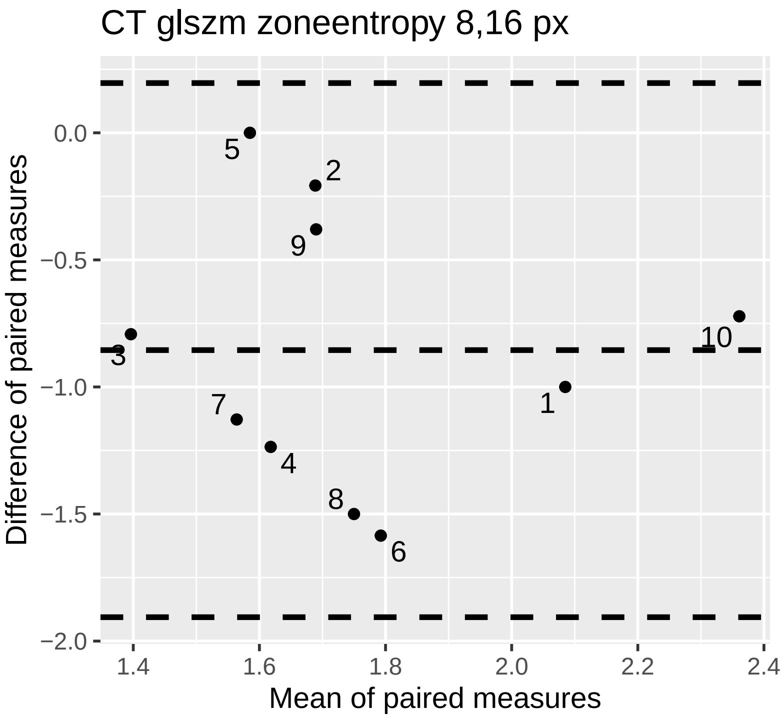
CT glszm zoneentropy 8,16 px
Difference of paired measures
Mean of paired measures

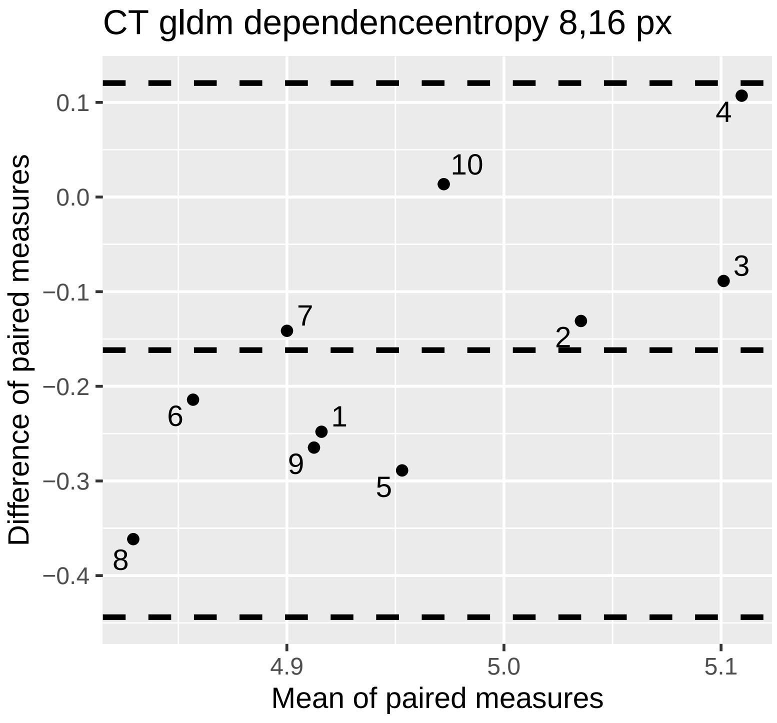
CT gldm dependenceentropy 8,16 px
Difference of paired measures
Mean of paired measures

CT gldm dependencenonuniformity 8,16 px

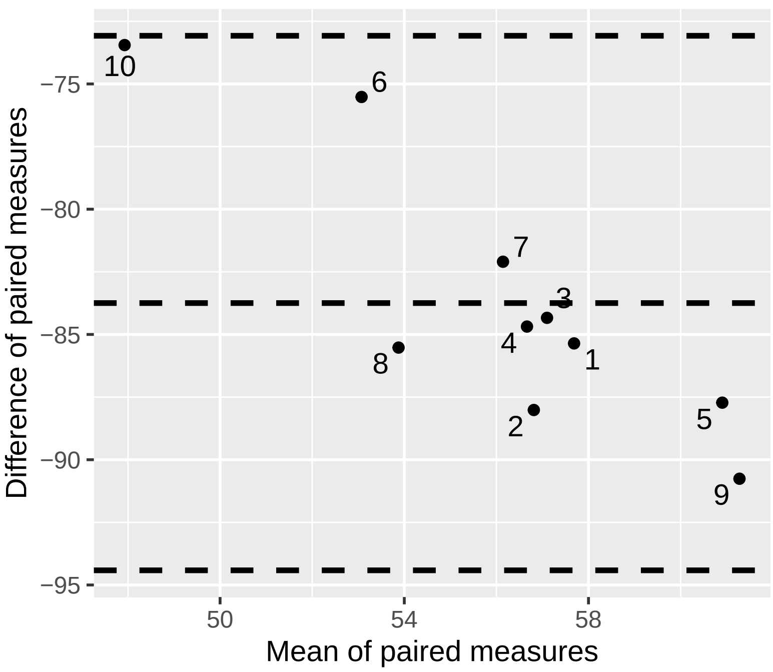


CT gldm graylevelnonuniformity 8,16 px

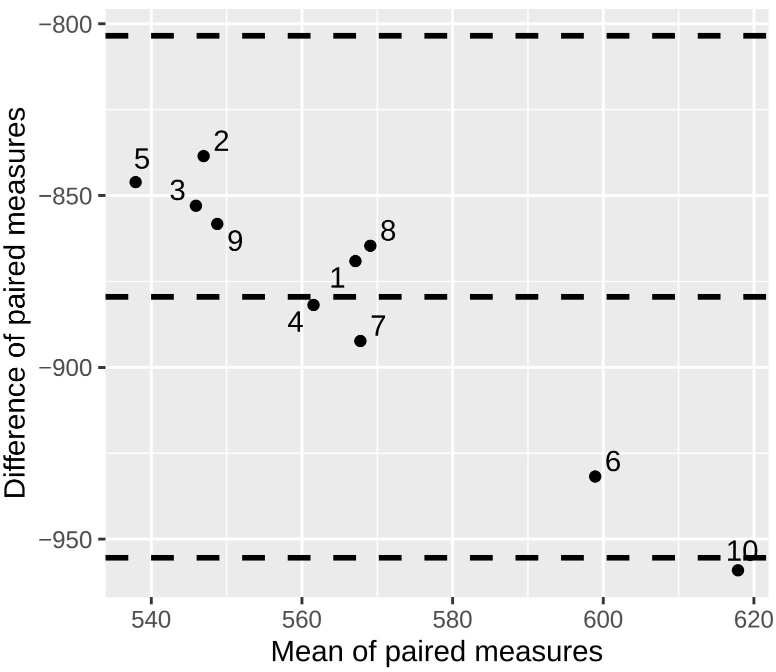


CT gldm dependencenonuniformitynormalize

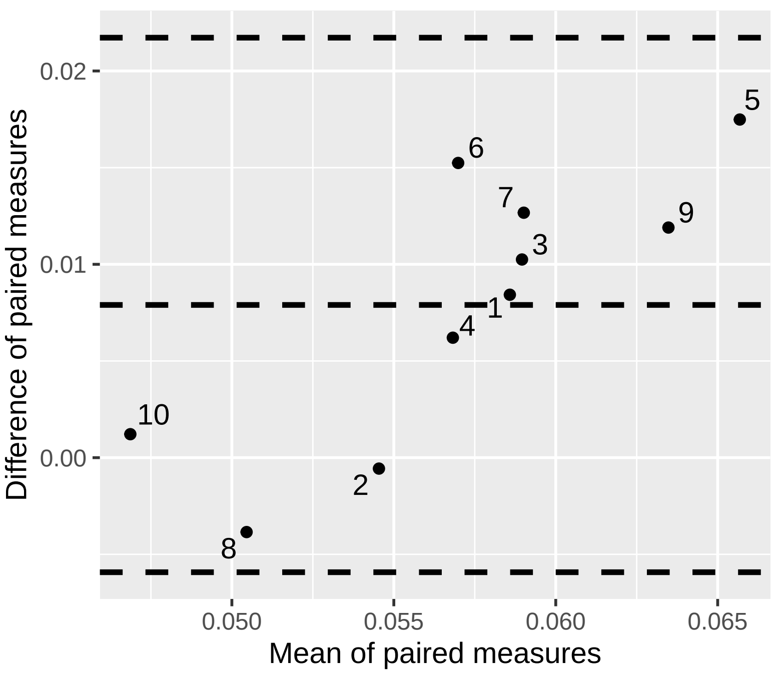


CT gldm graylevelvariance 8,16 px

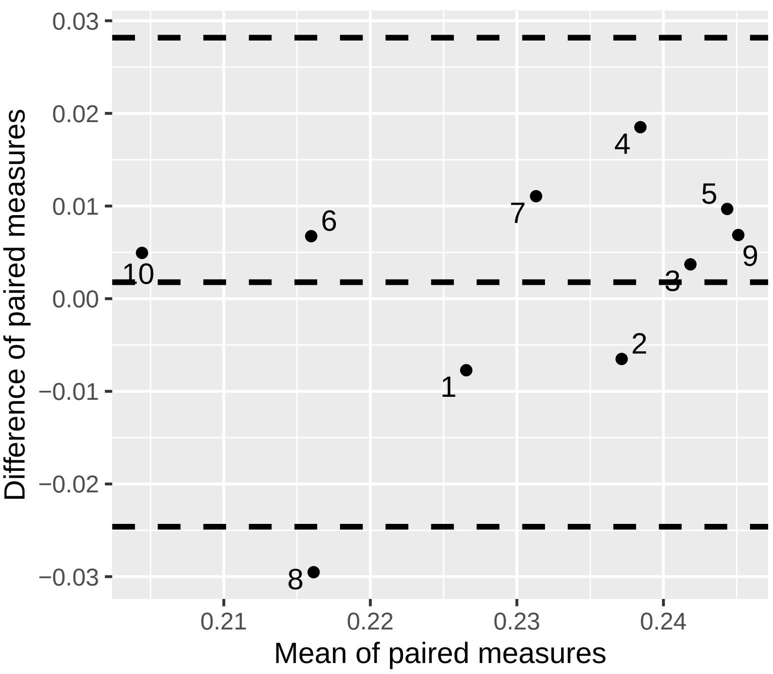


CT gldm dependencevariance 8,16 px

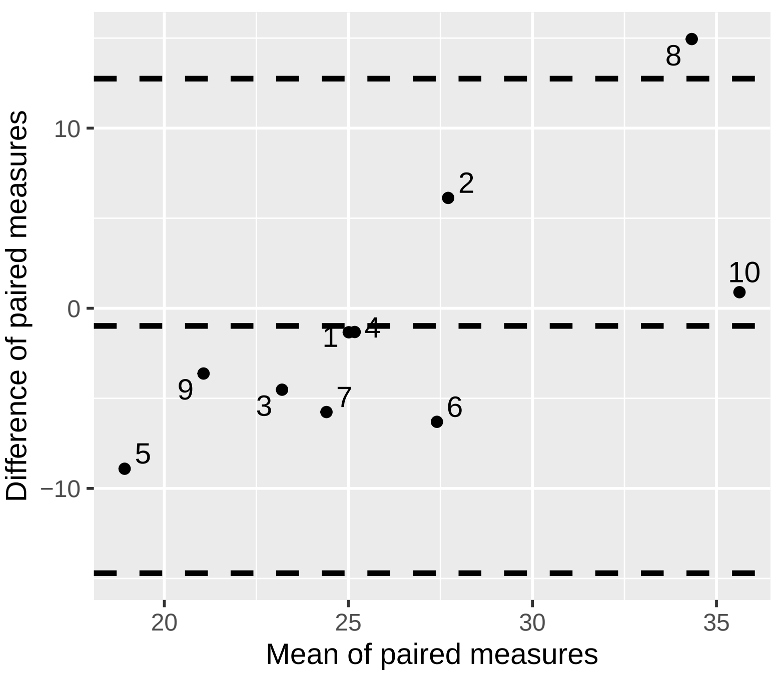


CT gldm highgraylevelemphasis 8,16 px

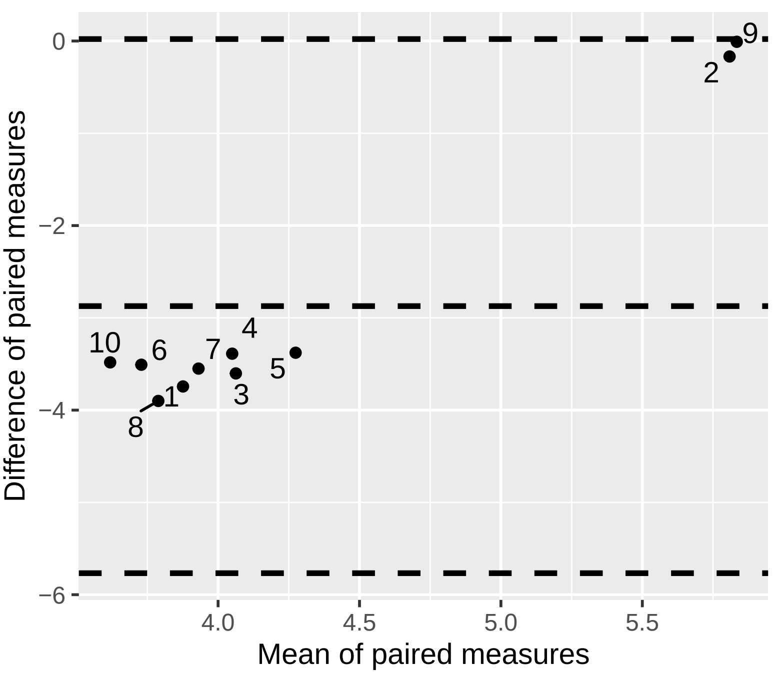

CT gldm largedependenceemphasis 8,16 px

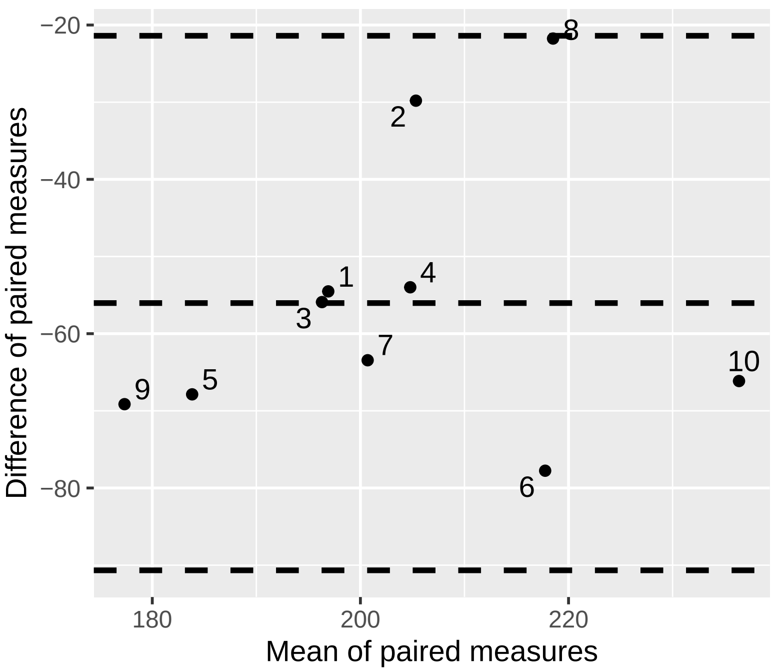


CT gldm lowgraylevelemphasis 8,16 px

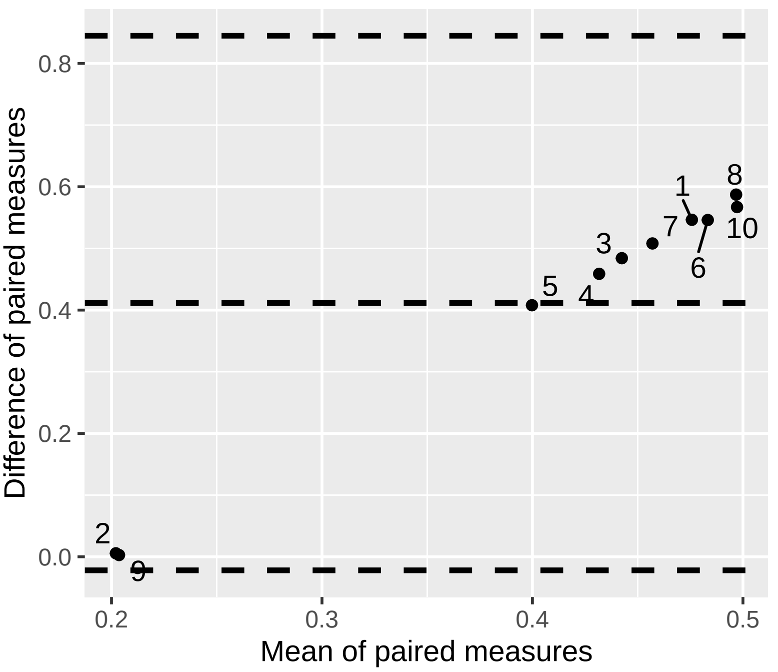


CT gldm largedependencehighgraylevelemp

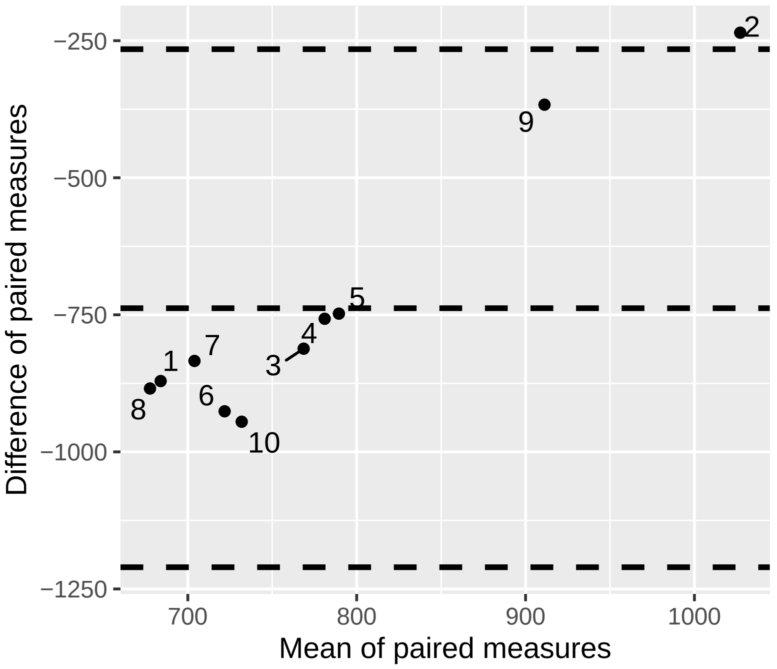


CT gldm smalldependenceemphasis 8,16 p

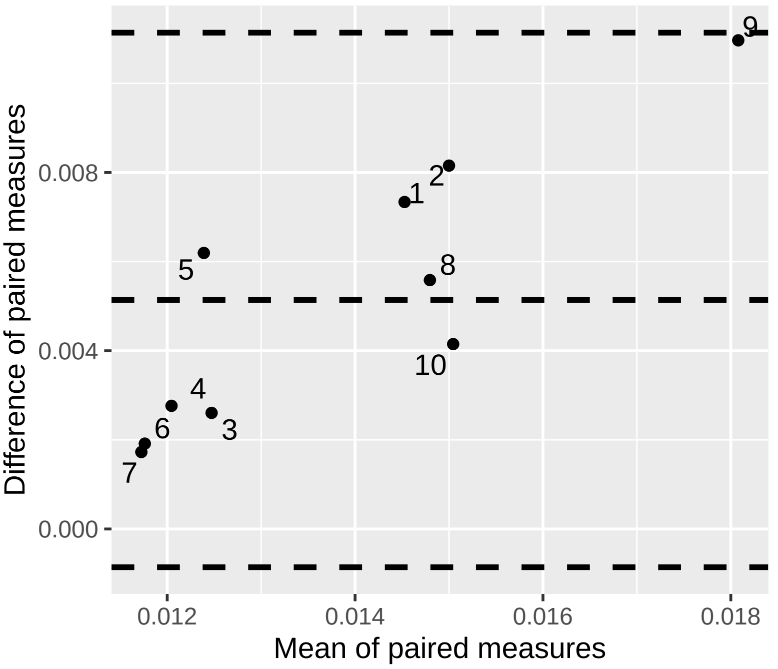


CT gldm largedependencelowgraylevelempha

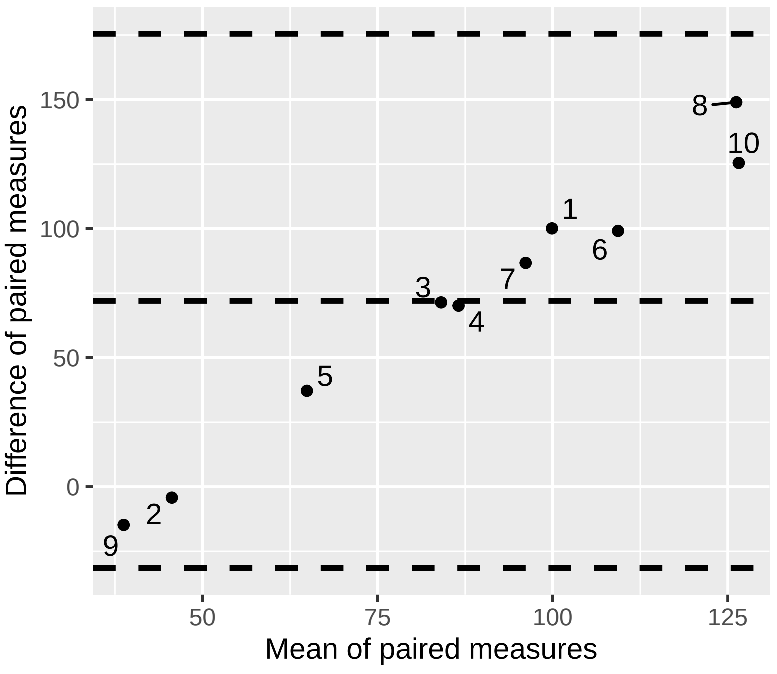


CT gldm smalldependencehighgraylevelemp

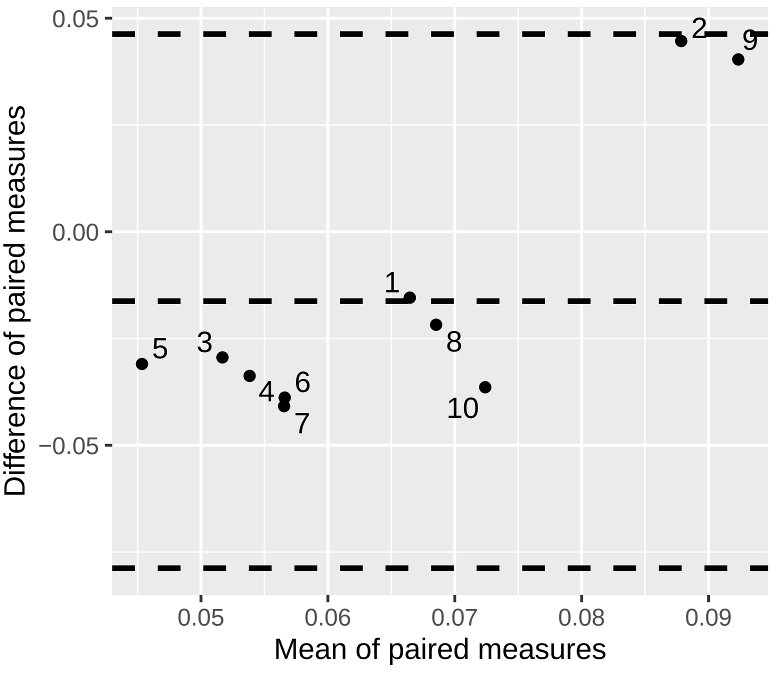

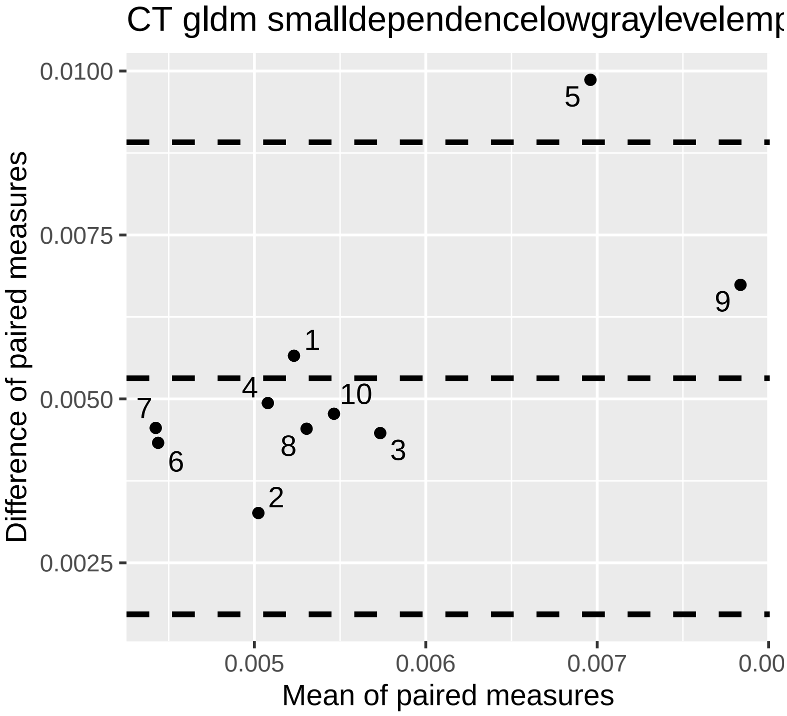
CT gldm smalldependencelowgraylevelemp
Difference of paired measures
Mean of paired measures
0.0100
0.0075
0.0050
0.0025
0.005
0.006
0.007
0.00
1
2
3
4
5
6
7
8
9
10

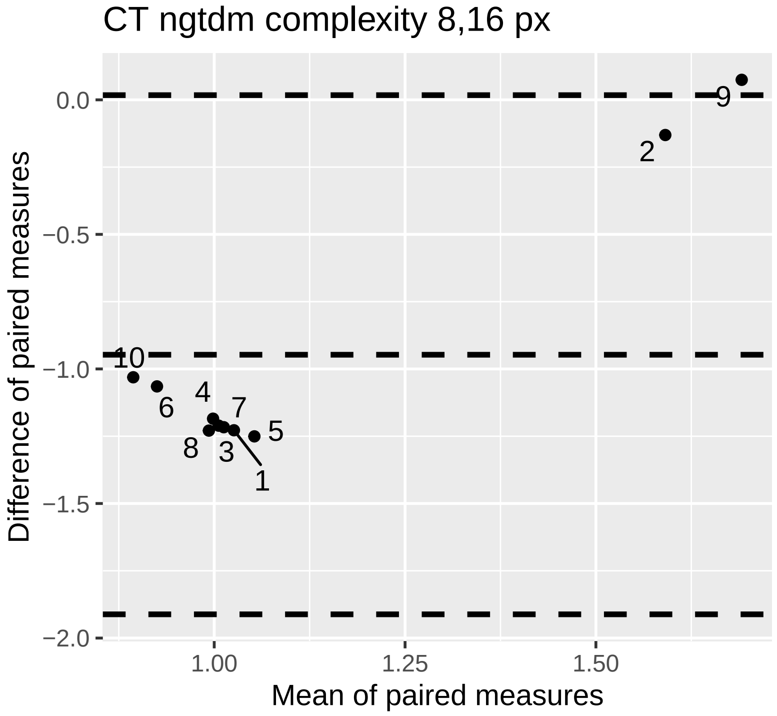
CT ngtdm complexity 8,16 px
Difference of paired measures
Mean of paired measures
0.0
−0.5
−1.0
−1.5
−2.0
1.00
1.25
1.50
1
2
3
4
5
6
7
8
9
10

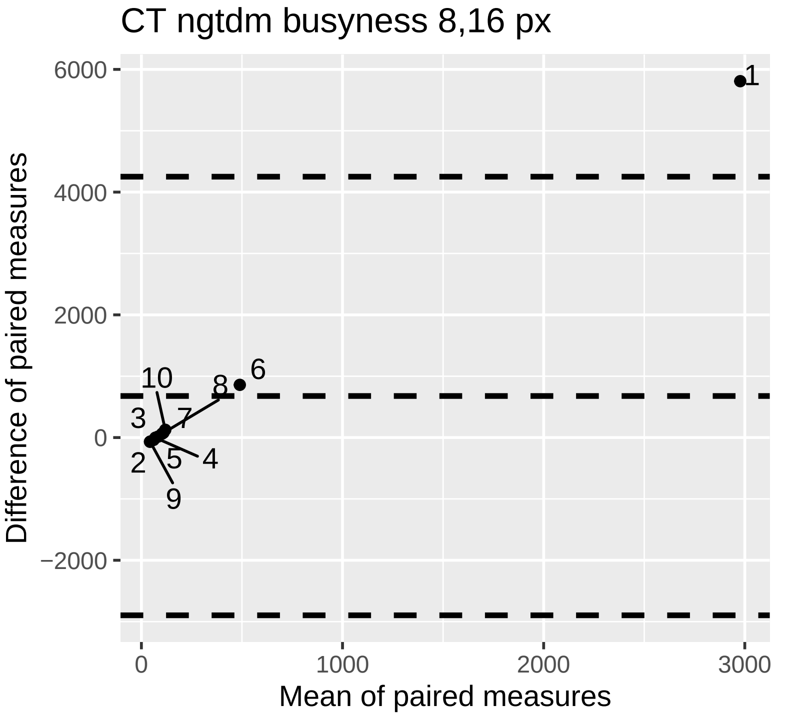
CT ngtdm busyness 8,16 px
Difference of paired measures
Mean of paired measures
6000
4000
2000
0
−2000
0
1000
2000
3000
1
2
3
4
5
6
7
8
9
10

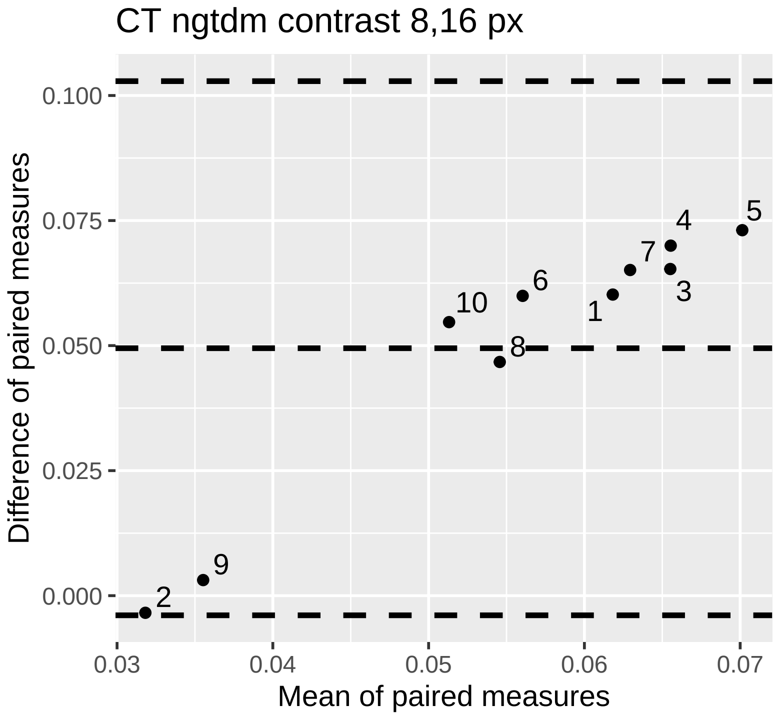
CT ngtdm contrast 8,16 px
Difference of paired measures
Mean of paired measures
0.100
0.075
0.050
0.025
0.000
0.03
0.04
0.05
0.06
0.07
1
2
3
4
5
6
7
8
9
10

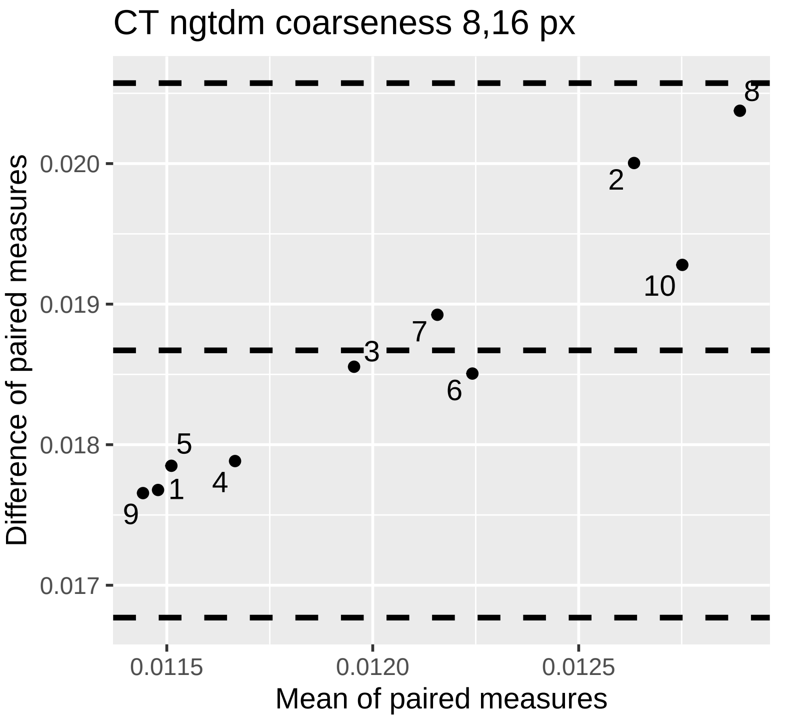
CT ngtdm coarseness 8,16 px
Difference of paired measures
Mean of paired measures
0.020
0.019
0.018
0.017
0.0115
0.0120
0.0125
1
2
3
4
5
6
7
8
9
10

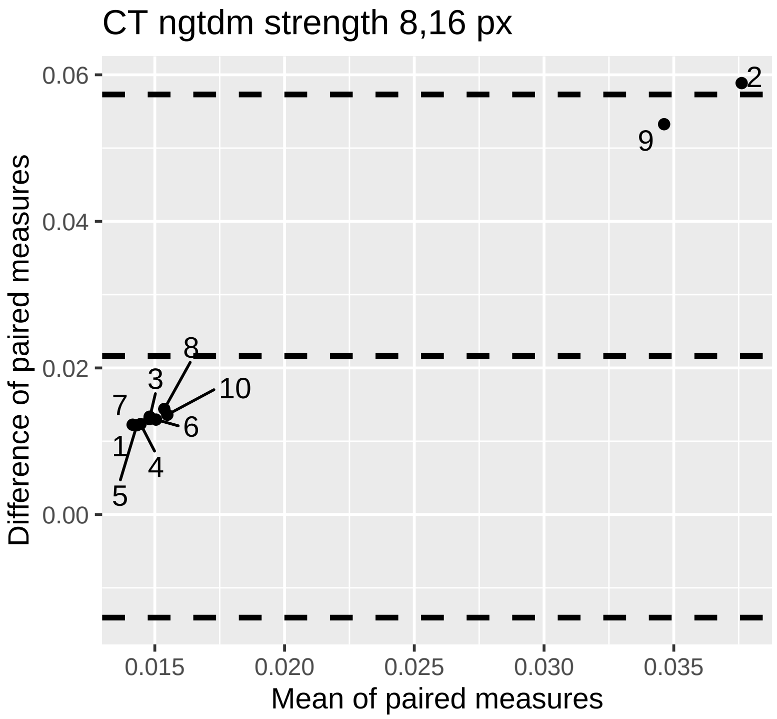
CT ngtdm strength 8,16 px
Difference of paired measures
Mean of paired measures
0.06
0.04
0.02
0.00
0.015
0.020
0.025
0.030
0.035
1
2
3
4
5
6
7
8
9
10

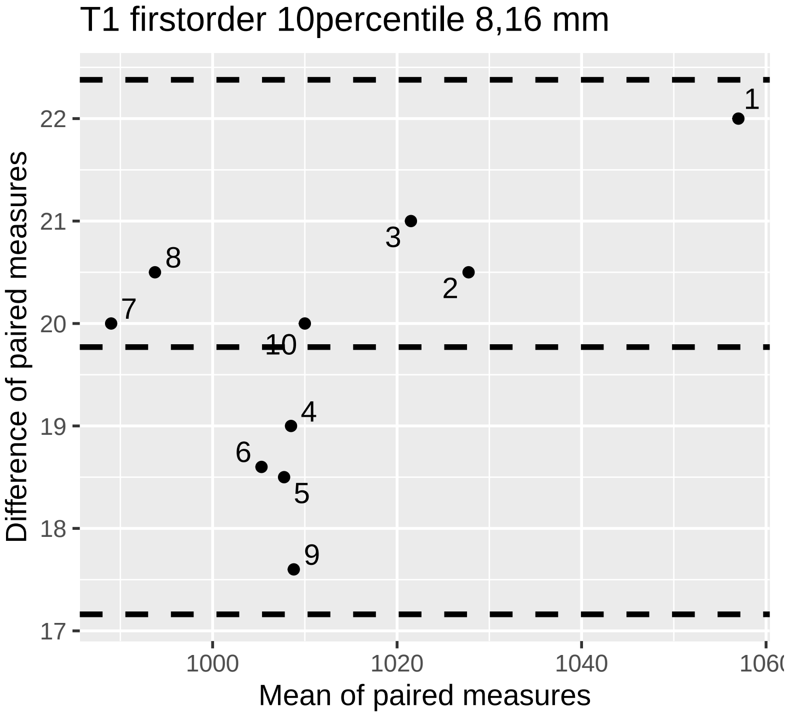
T1 firstorder 10percentile 8,16 mm
Difference of paired measures
Mean of paired measures
22
21
20
19
18
17
1000
1020
1040
1060
1
2
3
4
5
6
7
8
9
10

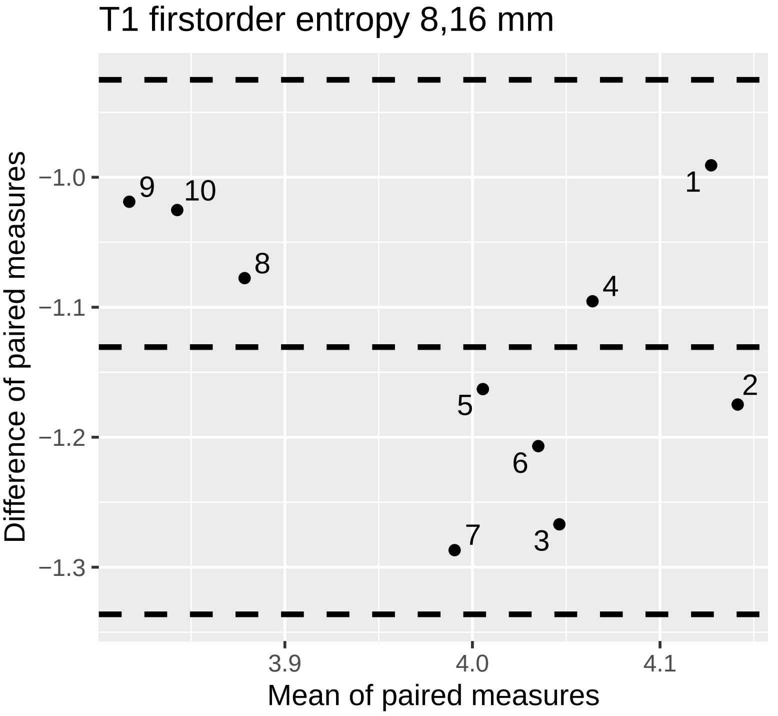
T1 firstorder entropy 8,16 mm
Difference of paired measures
Mean of paired measures
−1.0
−1.1
−1.2
−1.3
3.9
4.0
4.1
1
2
3
4
5
6
7
8
9
10

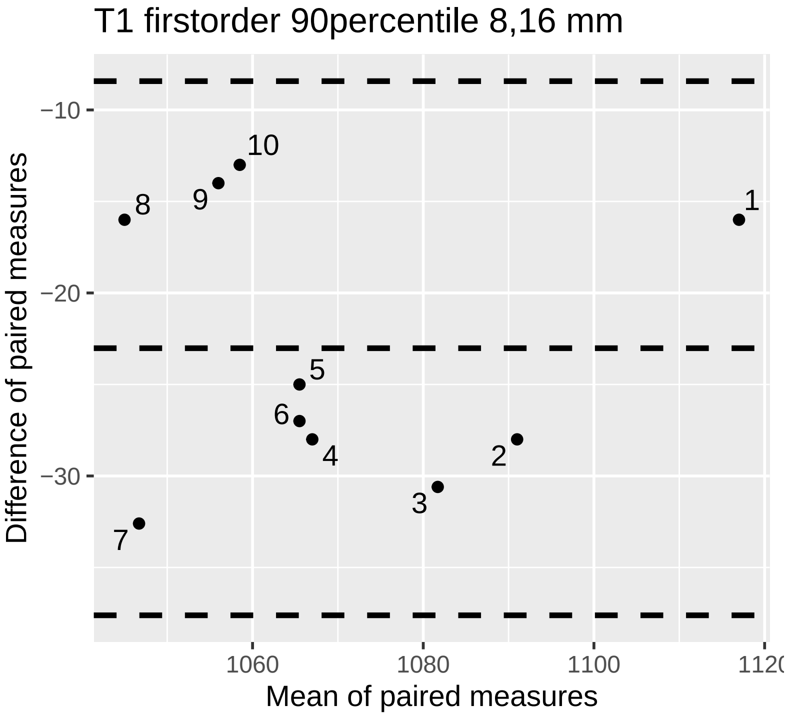
T1 firstorder 90percentile 8,16 mm
Difference of paired measures
Mean of paired measures
−10
−20
−30
1060
1080
1100
1120
1
2
3
4
5
6
7
8
9
10

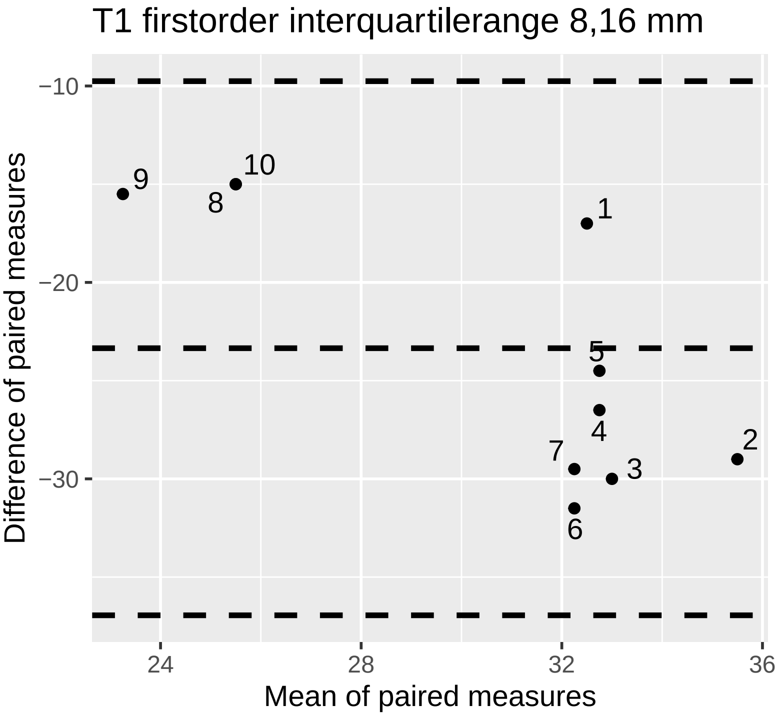
T1 firstorder interquartilerange 8,16 mm
Difference of paired measures
Mean of paired measures
−10
−20
−30
24
28
32
36
1
2
3
4
5
6
7
8
9
10

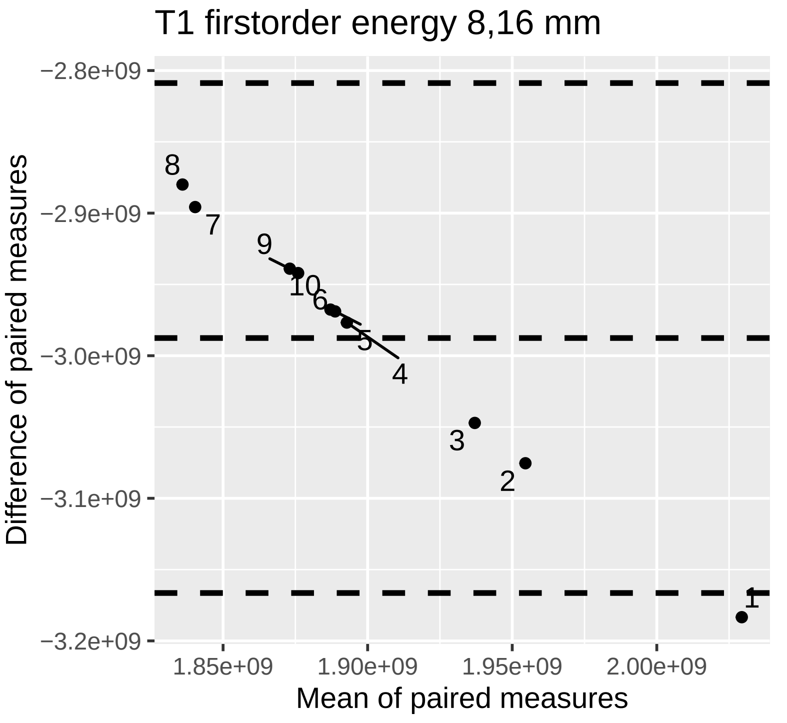
T1 firstorder energy 8,16 mm
Difference of paired measures
Mean of paired measures
−2.8e+09
−2.9e+09
−3.0e+09
−3.1e+09
−3.2e+09
1.85e+09
1.90e+09
1.95e+09
2.00e+09
1
2
3
4
5
6
7
8
9
10

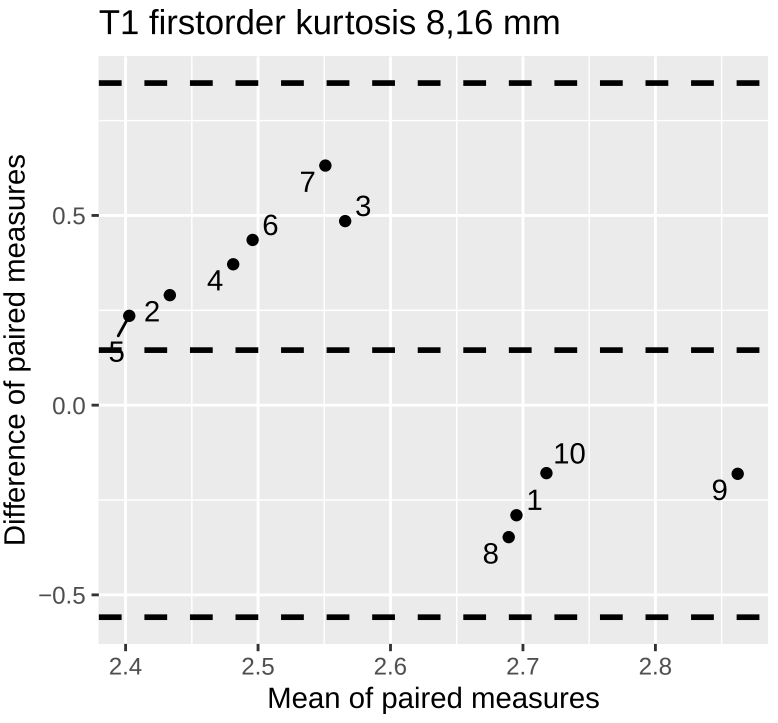
T1 firstorder kurtosis 8,16 mm
Difference of paired measures
Mean of paired measures
0.5
0.0
−0.5
2.4
2.5
2.6
2.7
2.8
1
2
3
4
5
6
7
8
9
10

T1 firstorder maximum 8,16 mm

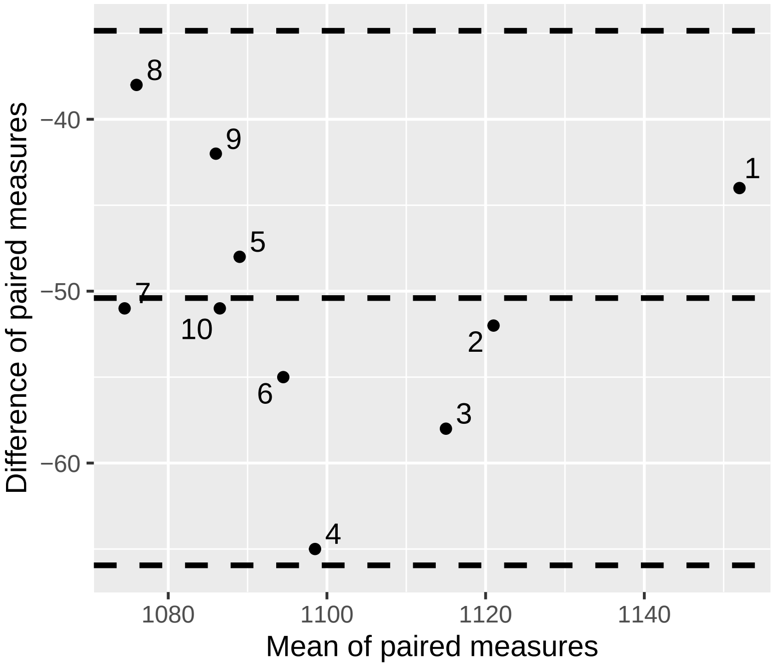


T1 firstorder median 8,16 mm

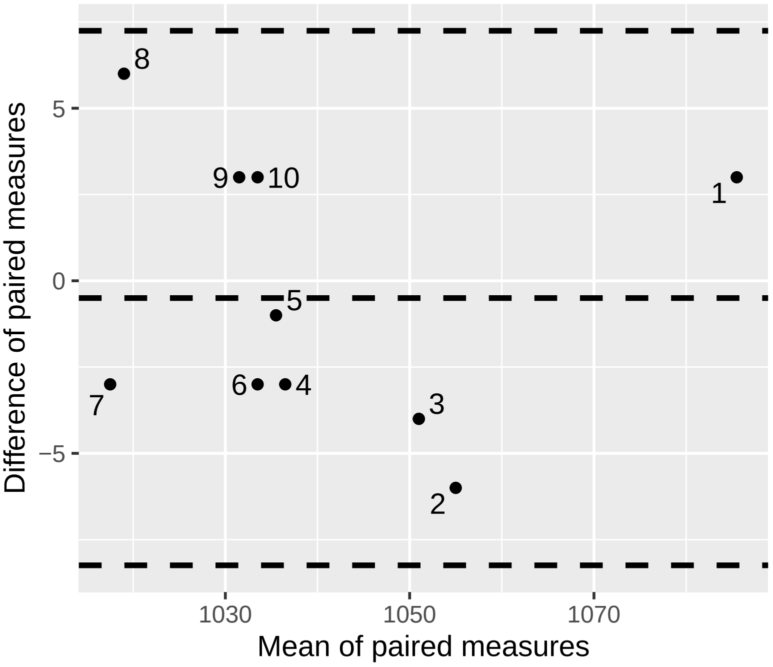


T1 firstorder meanabsolutedeviation 8,16 m

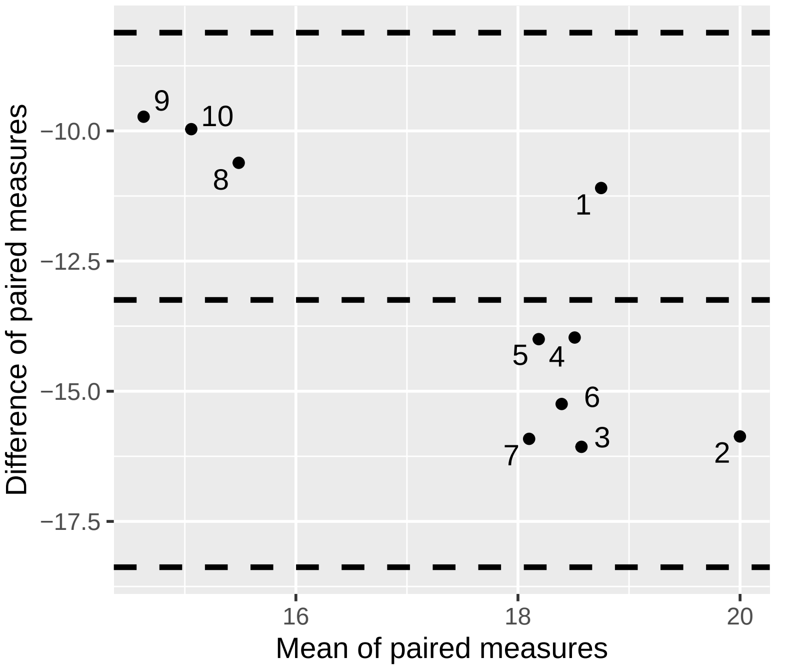


T1 firstorder minimum 8,16 mm

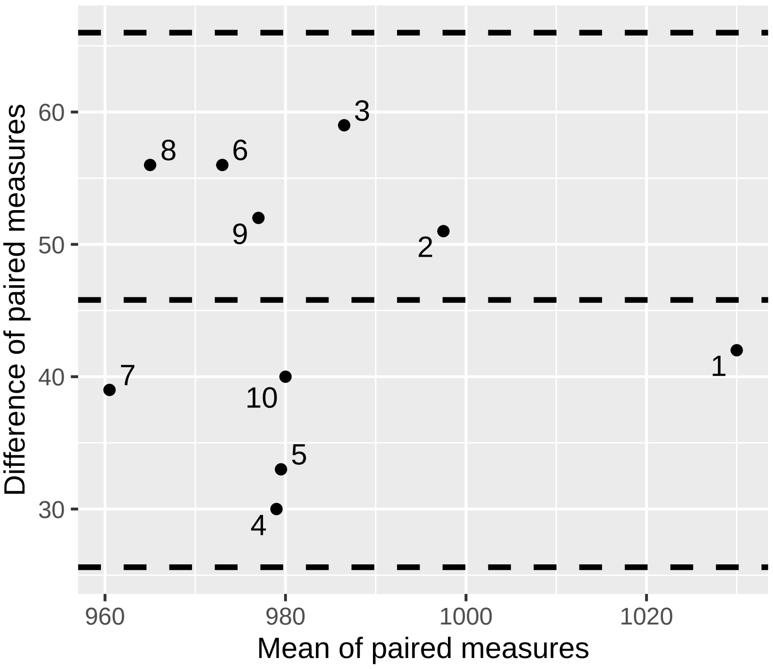


T1 firstorder mean 8,16 mm

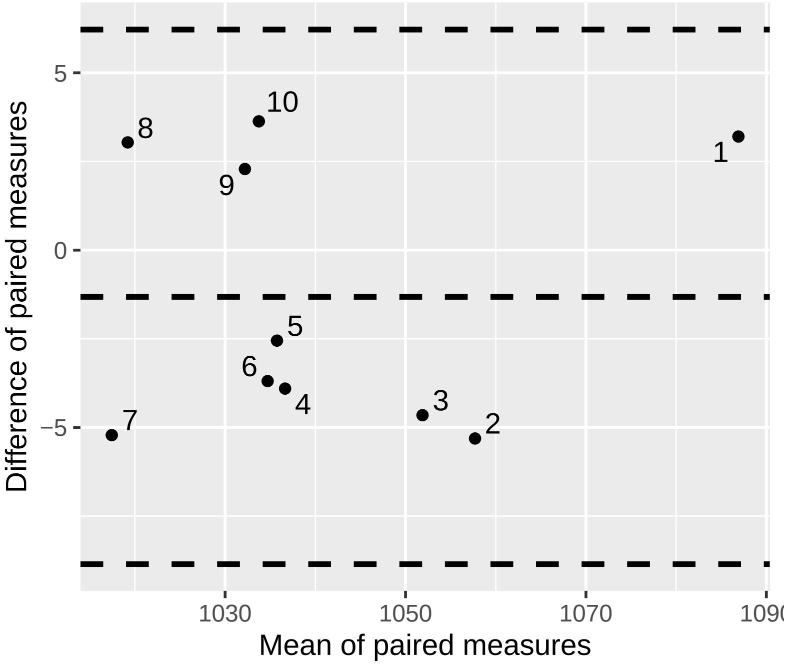


T1 firstorder range 8,16 mm

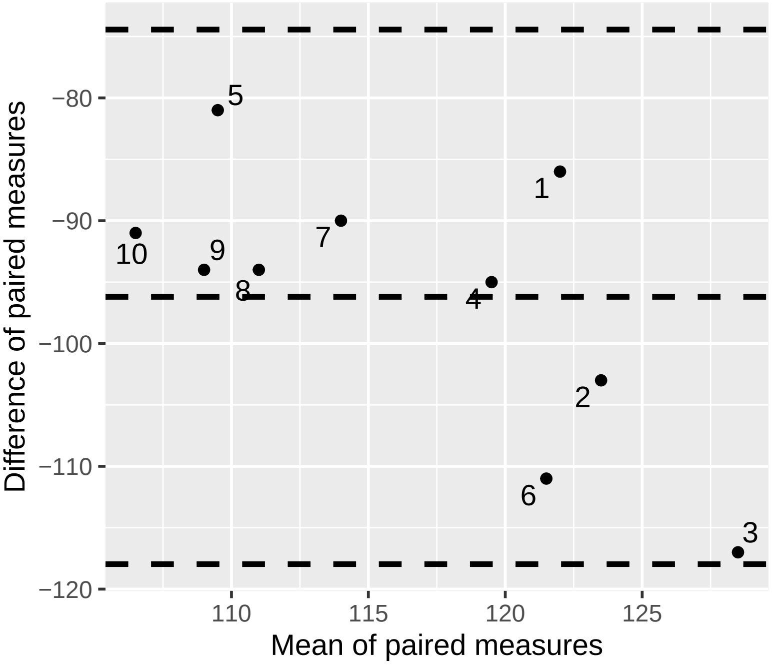

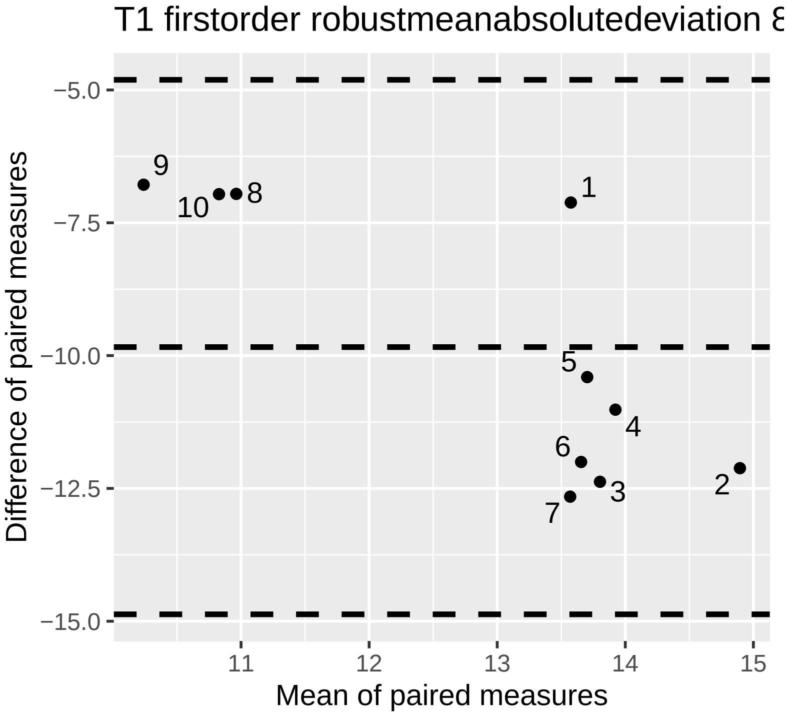
T1 firstorder robustmeanabsolutedeviation 8
Difference of paired measures
Mean of paired measures

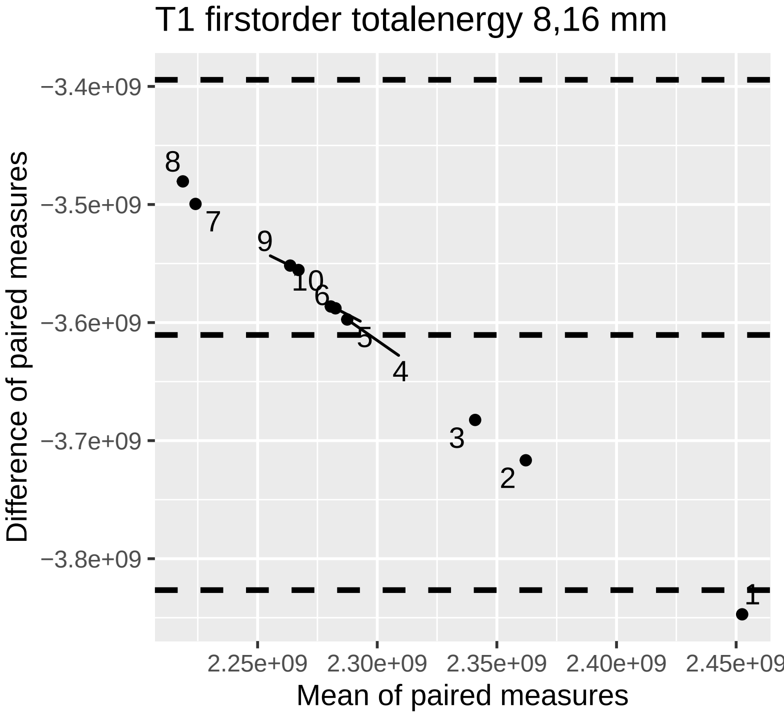
T1 firstorder totalenergy 8,16 mm
Difference of paired measures
Mean of paired measures

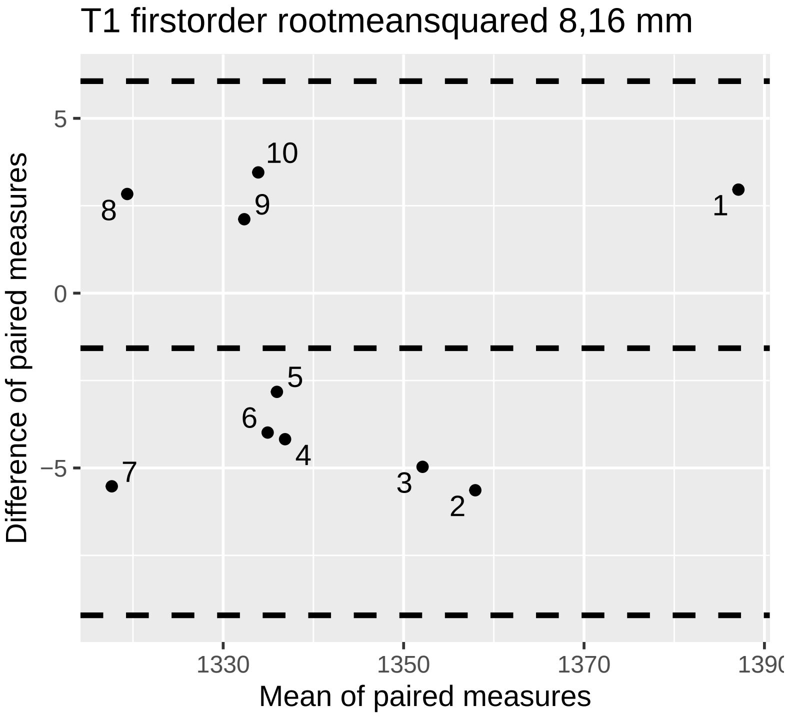
T1 firstorder rootmeansquared 8,16 mm
Difference of paired measures
Mean of paired measures

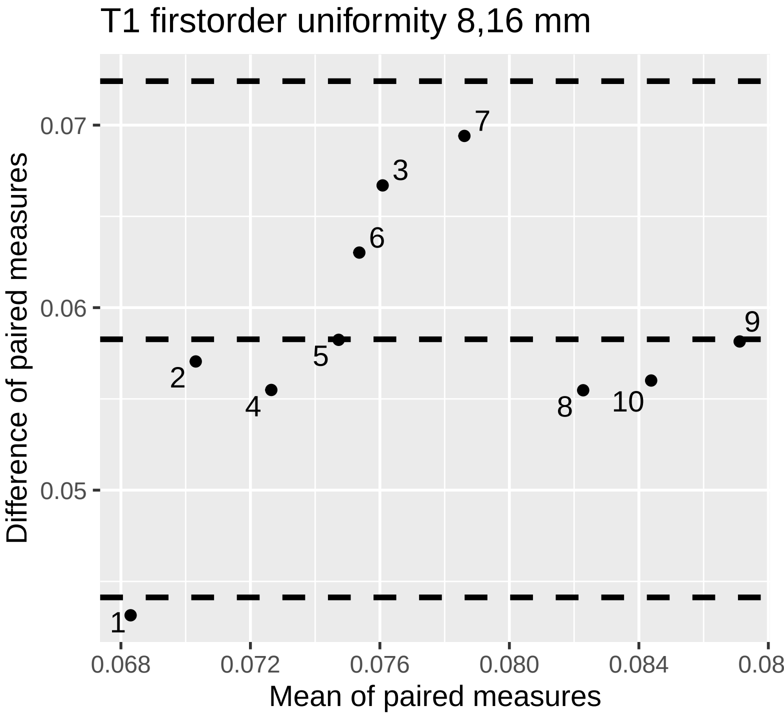
T1 firstorder uniformity 8,16 mm
Difference of paired measures
Mean of paired measures

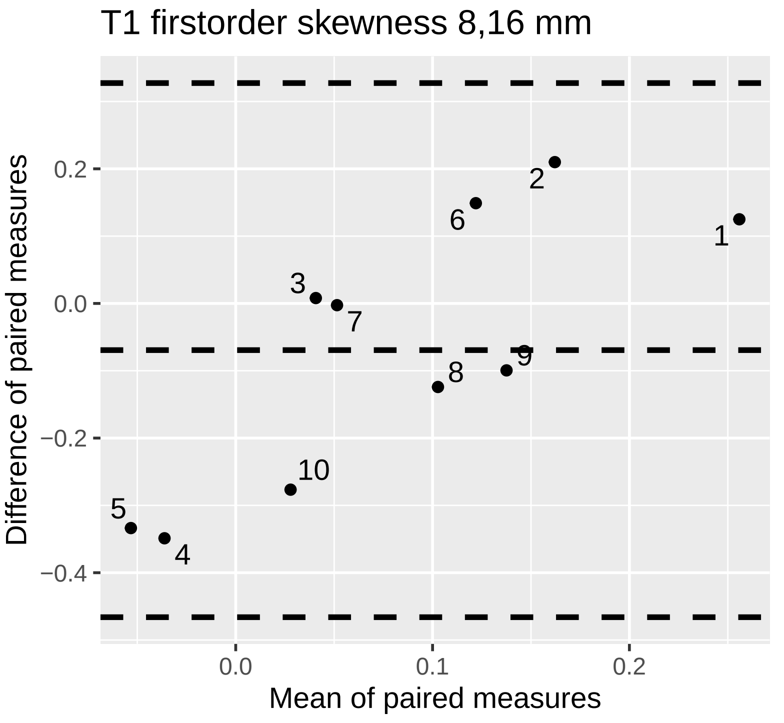
T1 firstorder skewness 8,16 mm
Difference of paired measures
Mean of paired measures

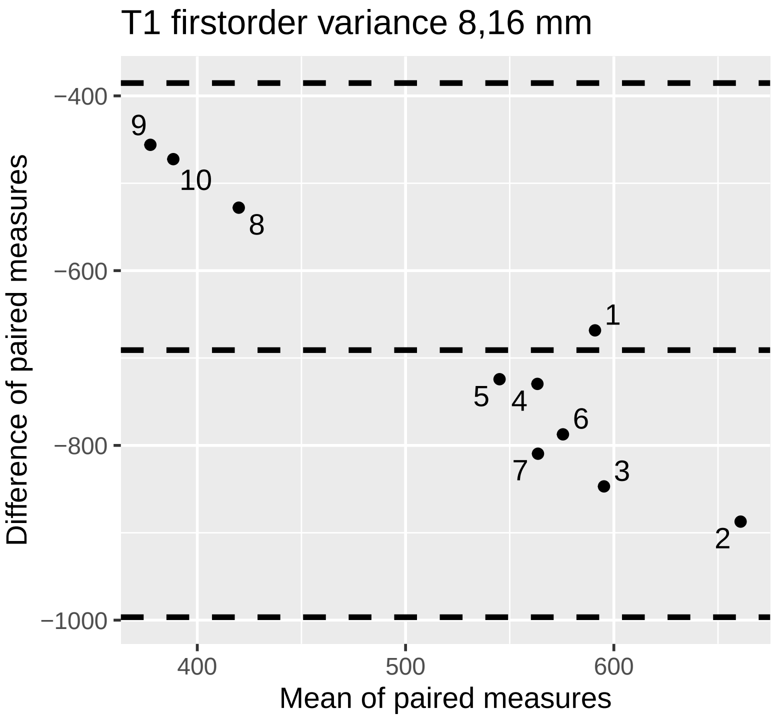
T1 firstorder variance 8,16 mm
Difference of paired measures
Mean of paired measures

## T1 glcm autocorrelation 8,16 mm

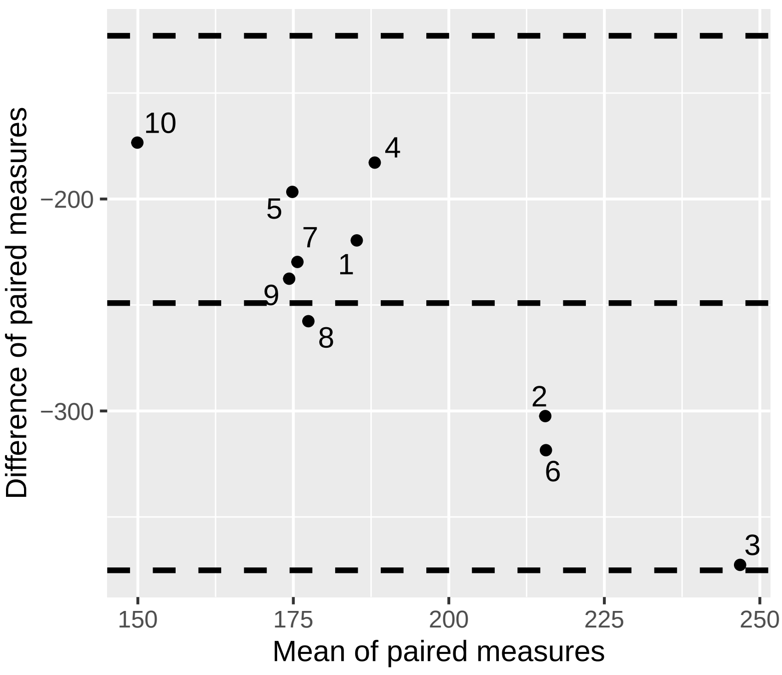


## T1 glcm clustertendency 8,16 mm

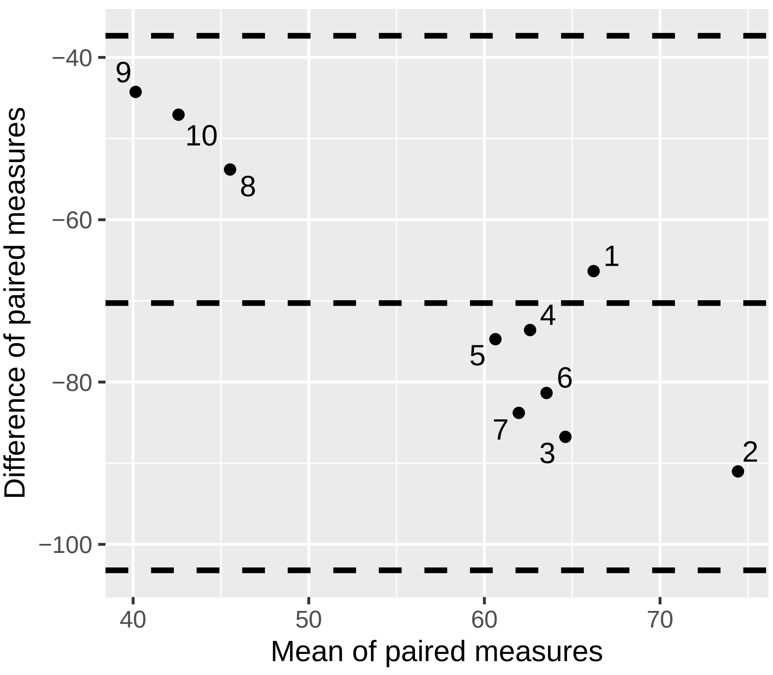


## T1 glcm clusterprominence 8,16 mm

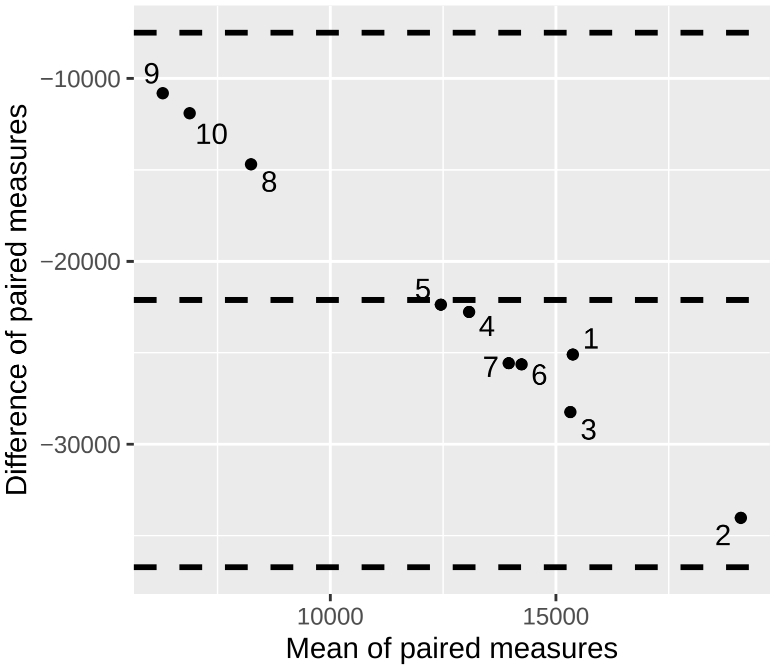


## T1 glcm contrast 8,16 mm

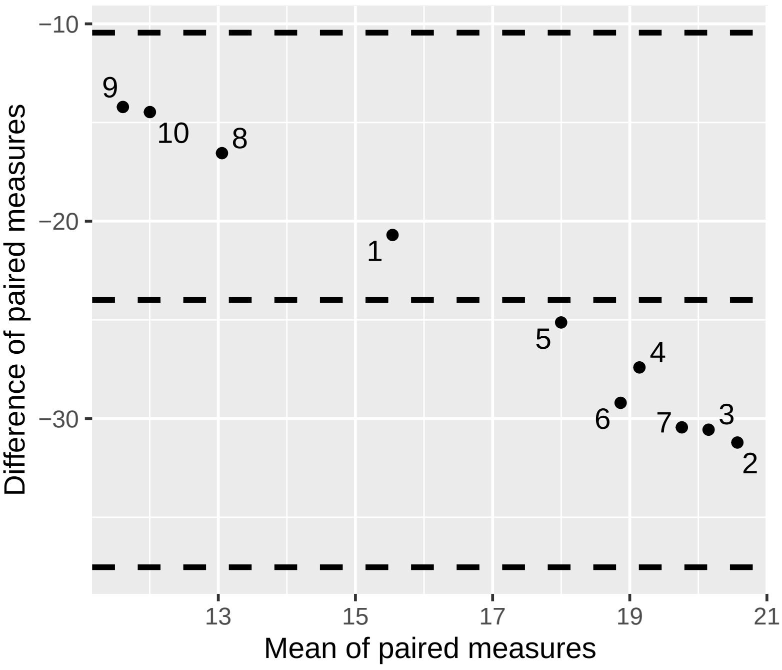


## T1 glcm clustershade 8,16 mm

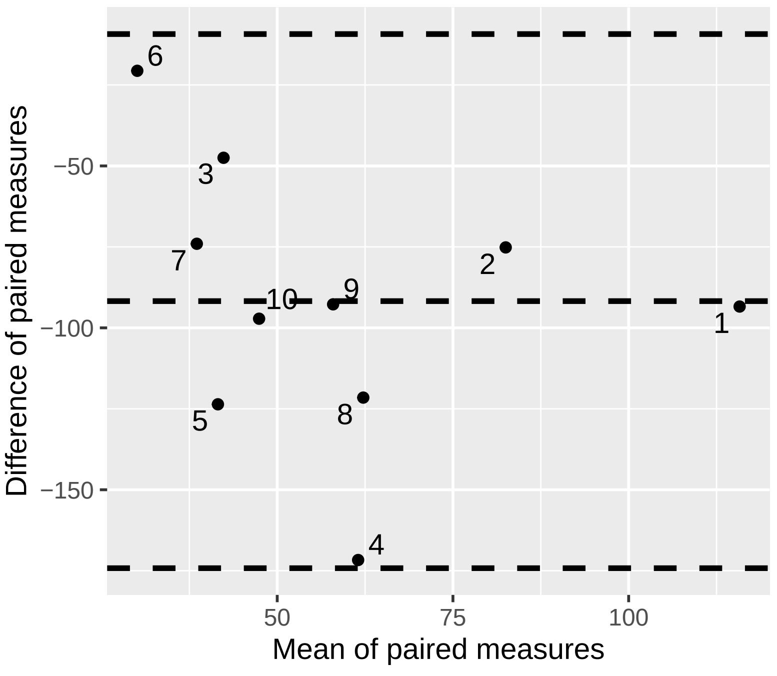


## T1 glcm correlation 8,16 mm

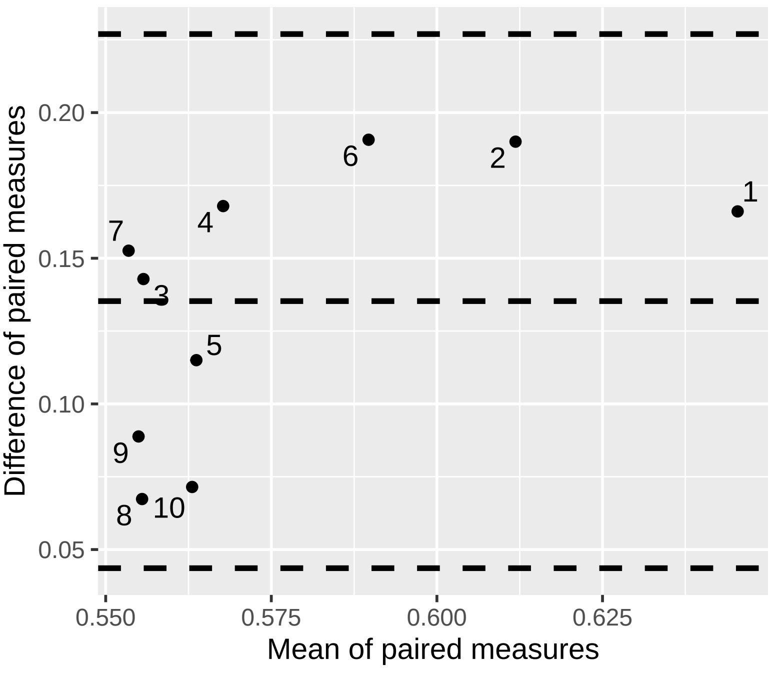

### T1 glcm differenceaverage 8,16 mm

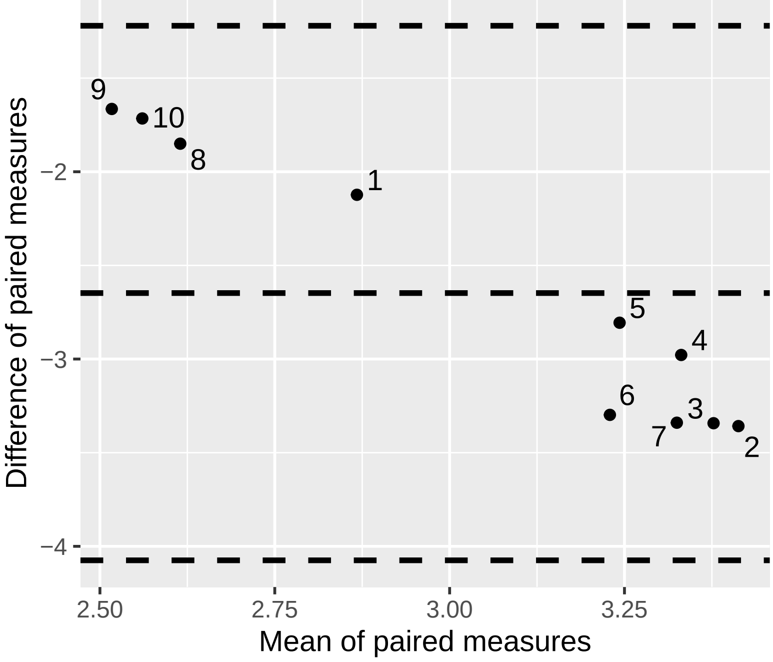


### T1 glcm id 8,16 mm

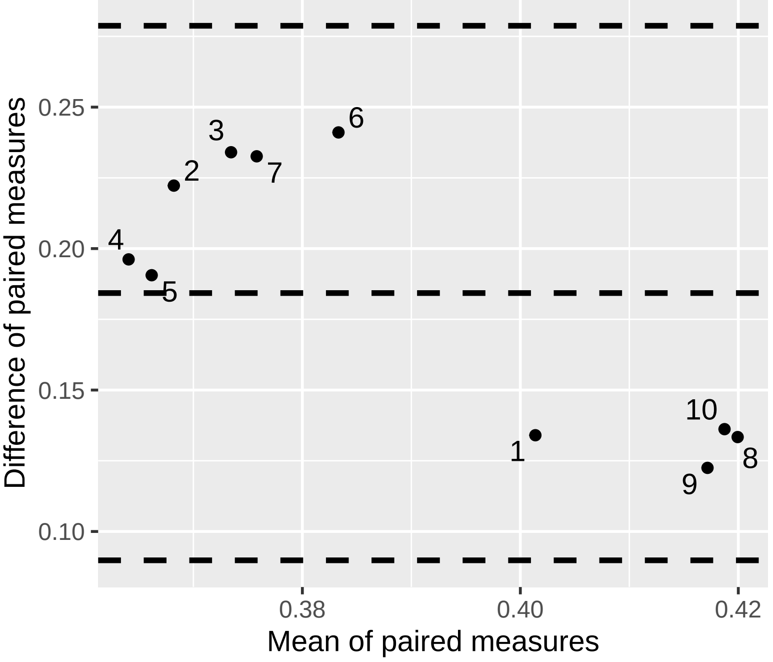


### T1 glcm differenceentropy 8,16 mm

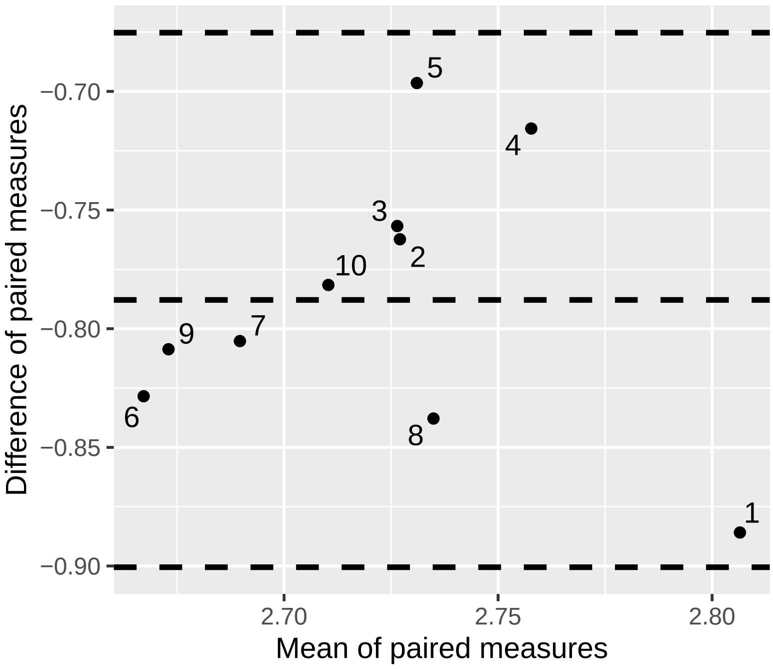


### T1 glcm idm 8,16 mm

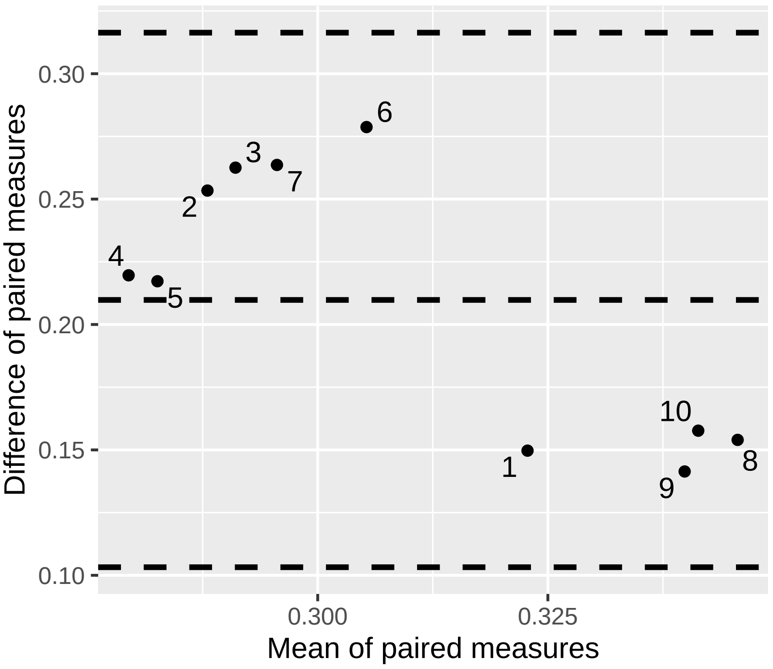


### T1 glcm differencevariance 8,16 mm

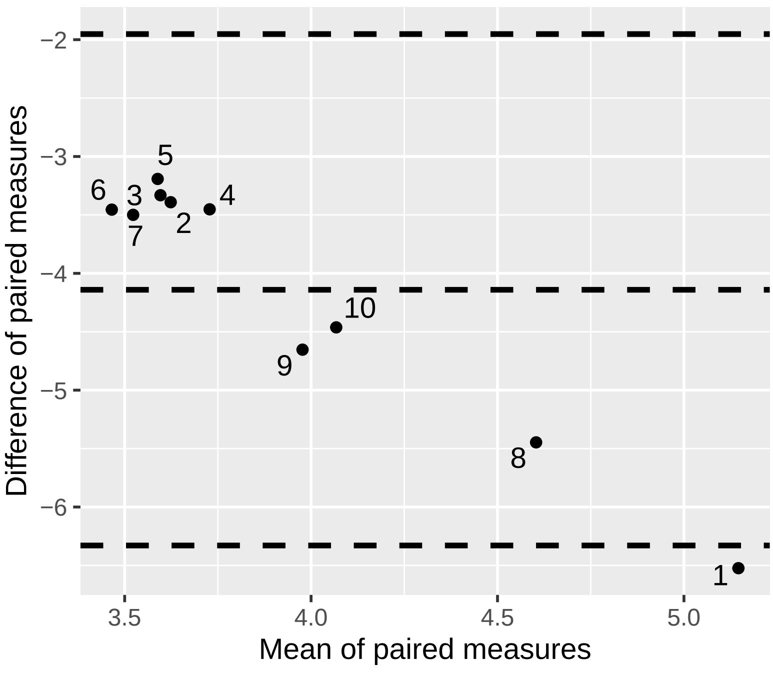


### T1 glcm idmn 8,16 mm

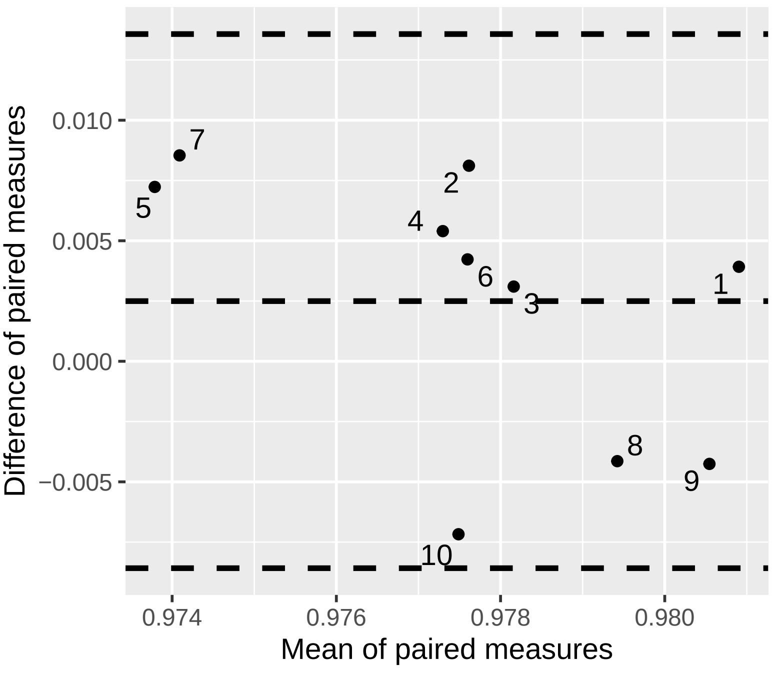

T1 glcm idn 8,16 mm

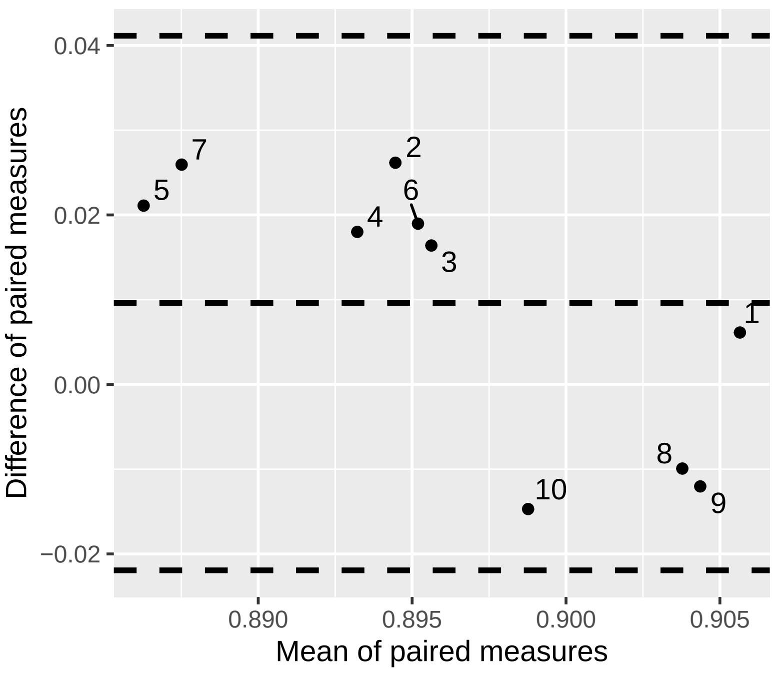


T1 glcm inversevariance 8,16 mm

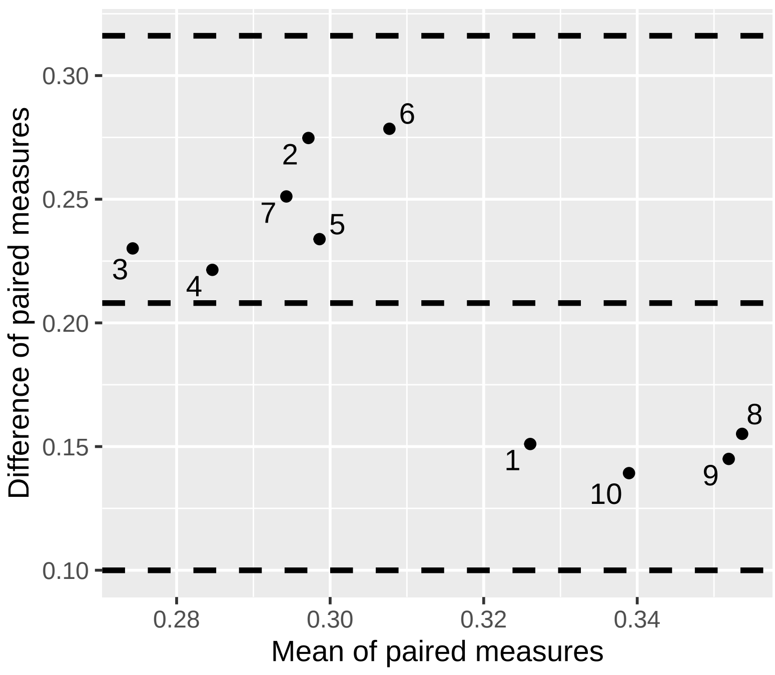


T1 glcm imc1 8,16 mm

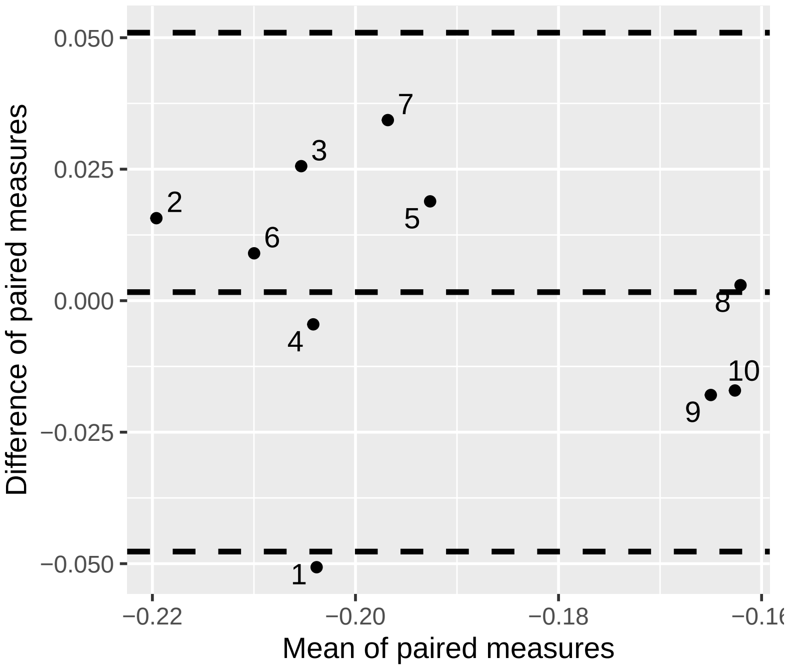


T1 glcm jointaverage 8,16 mm

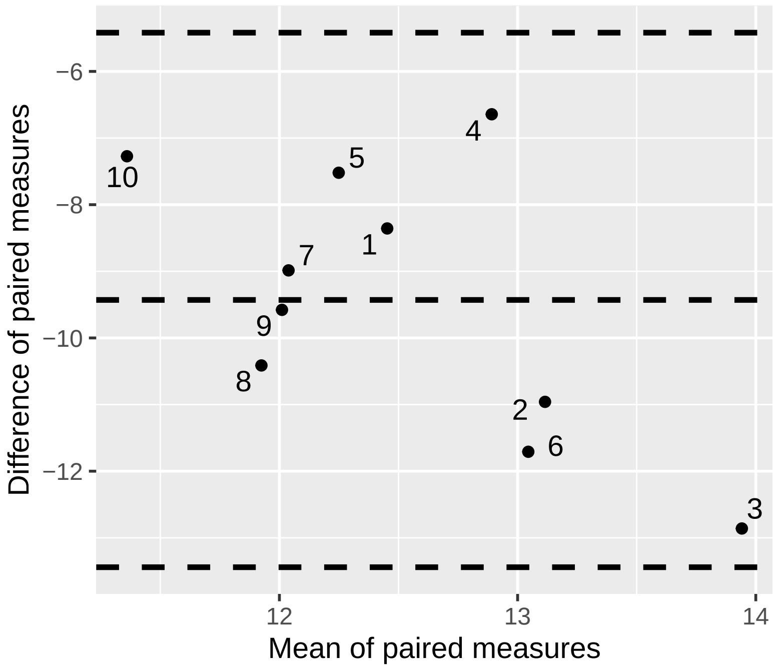


T1 glcm imc2 8,16 mm

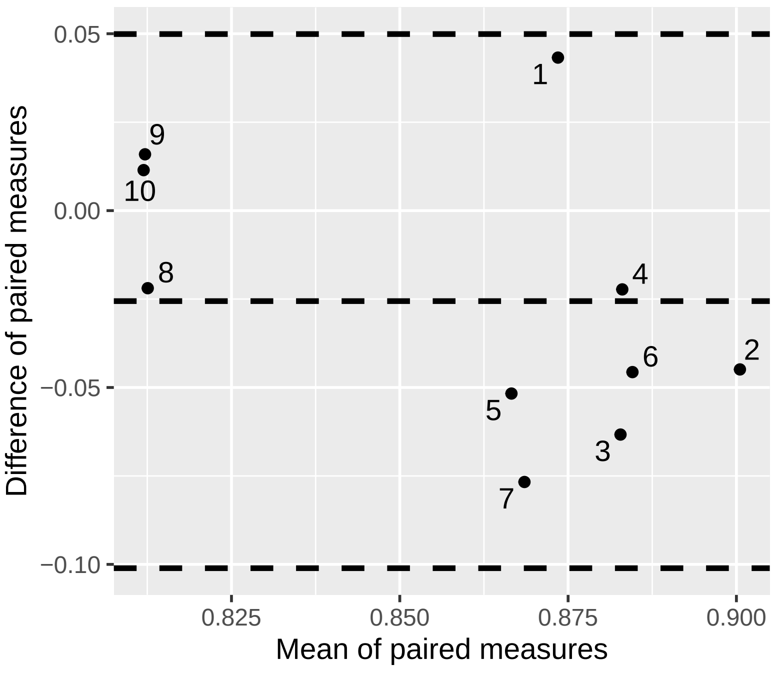


T1 glcm jointenergy 8,16 mm

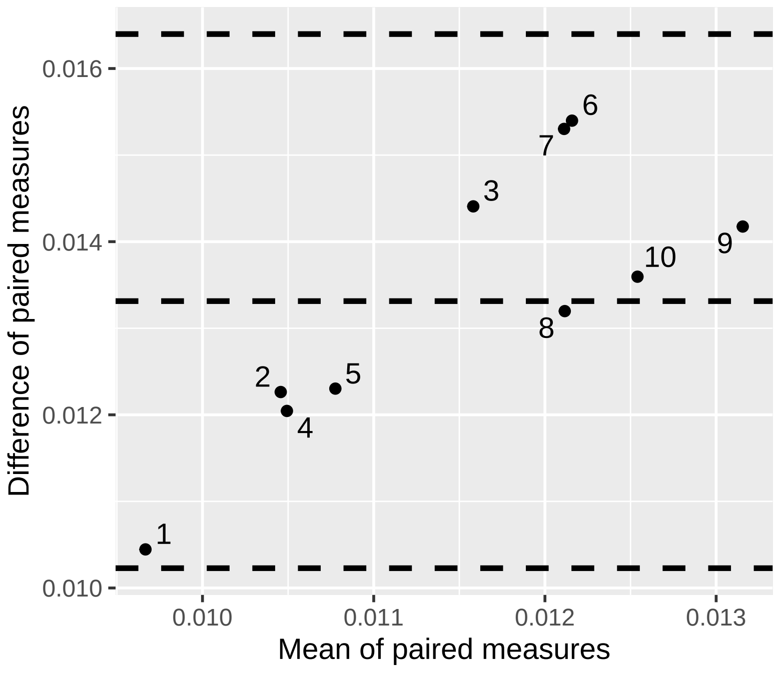

T1 glcm jointentropy 8,16 mm

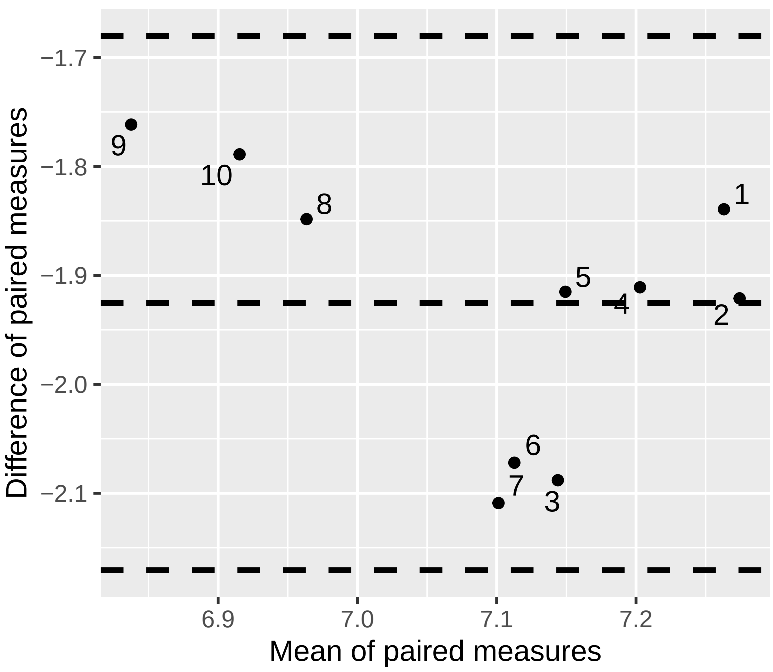


T1 glcm sumaverage 8,16 mm

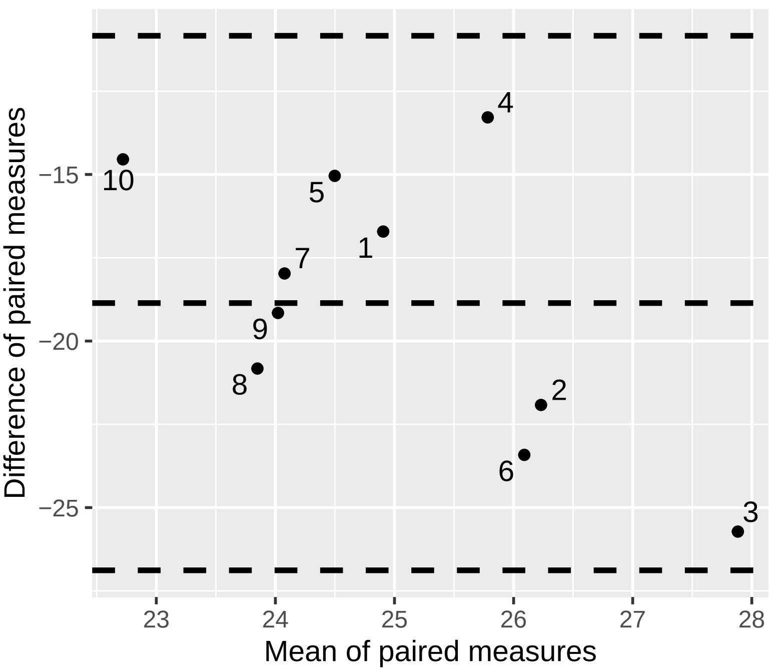


T1 glcm mcc 8,16 mm

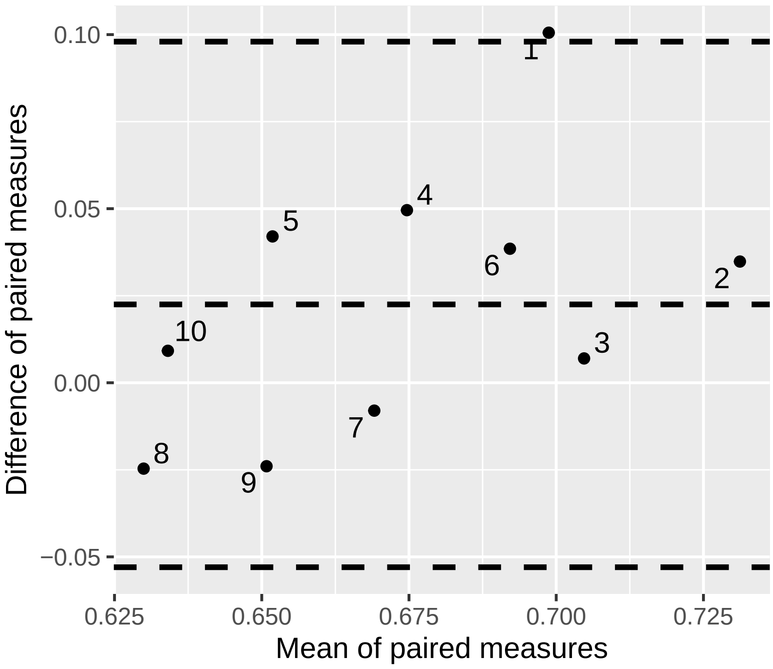


T1 glcm sumentropy 8,16 mm

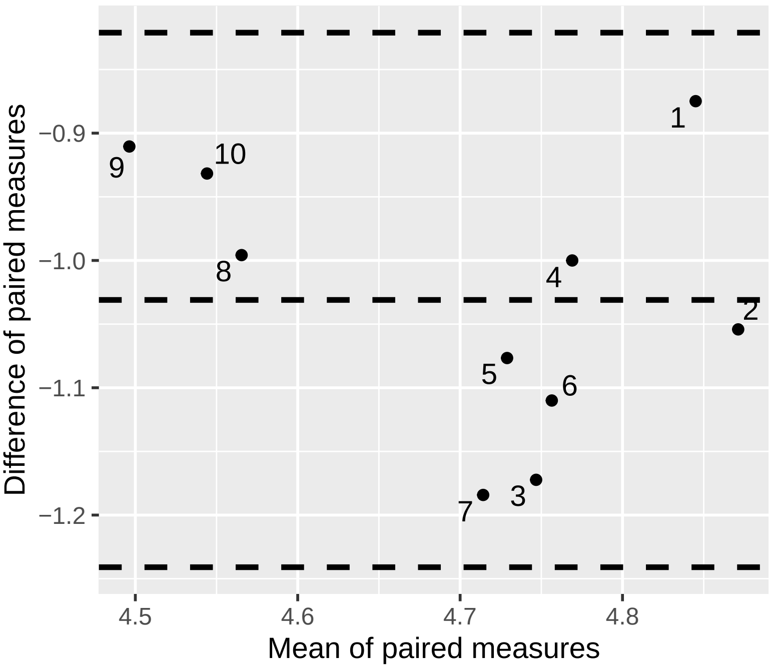


T1 glcm maximumprobability 8,16 mm

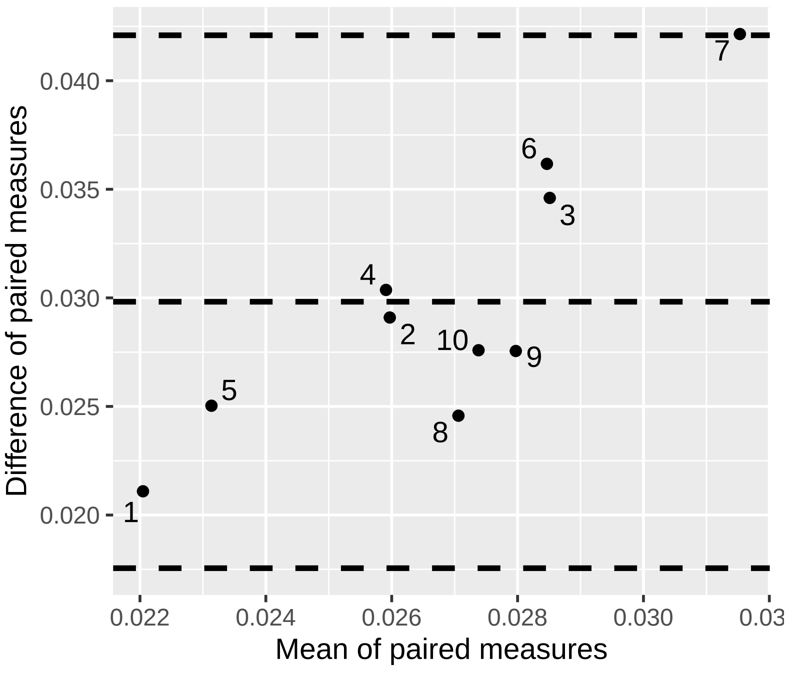


T1 glcm sumsquares 8,16 mm

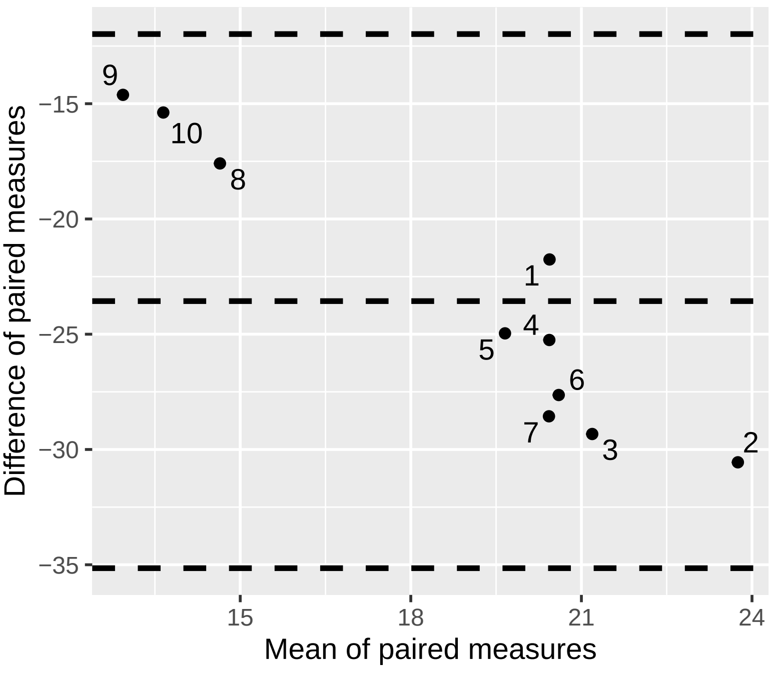

T1 glrlm graylevelnonuniformity 8,16 mm

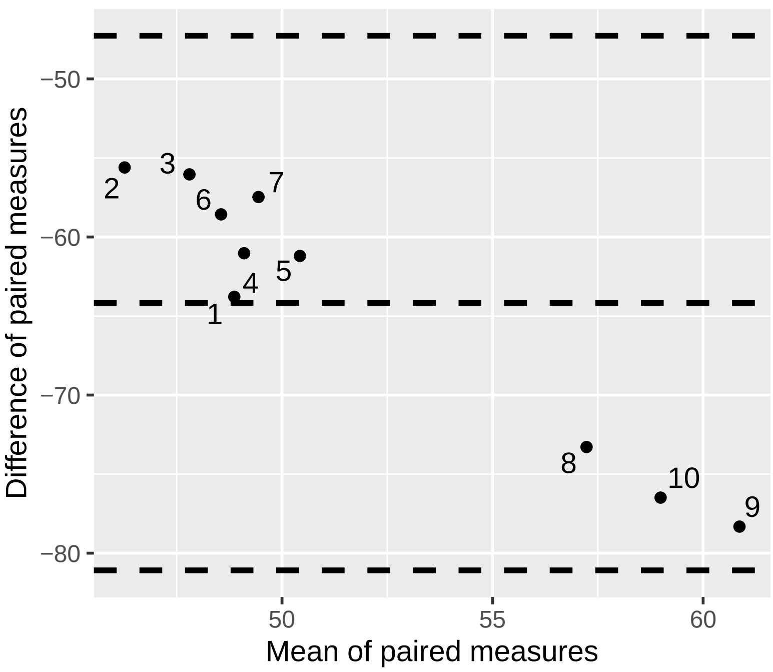


T1 glrlm highgraylevelrunemphasis 8,16 mm

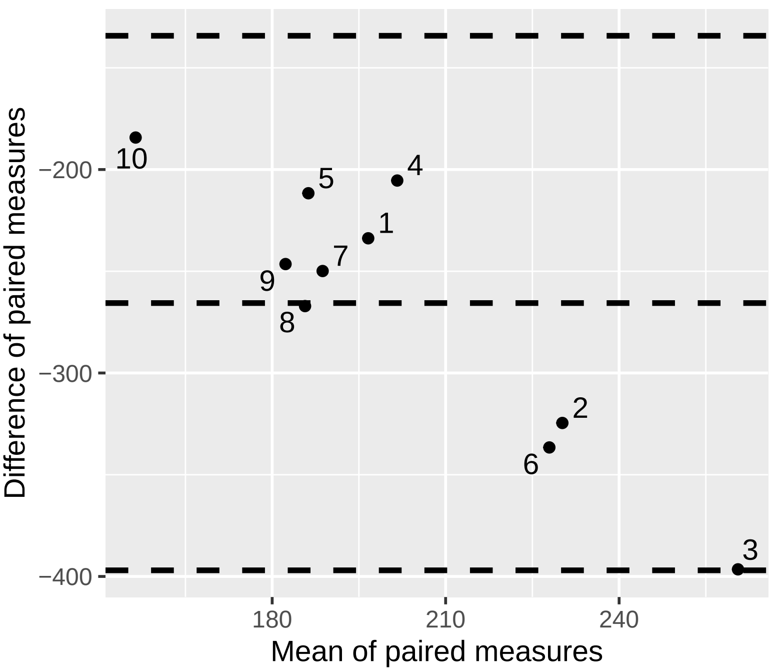


T1 glrlm graylevelnonuniformitynormalized 8,

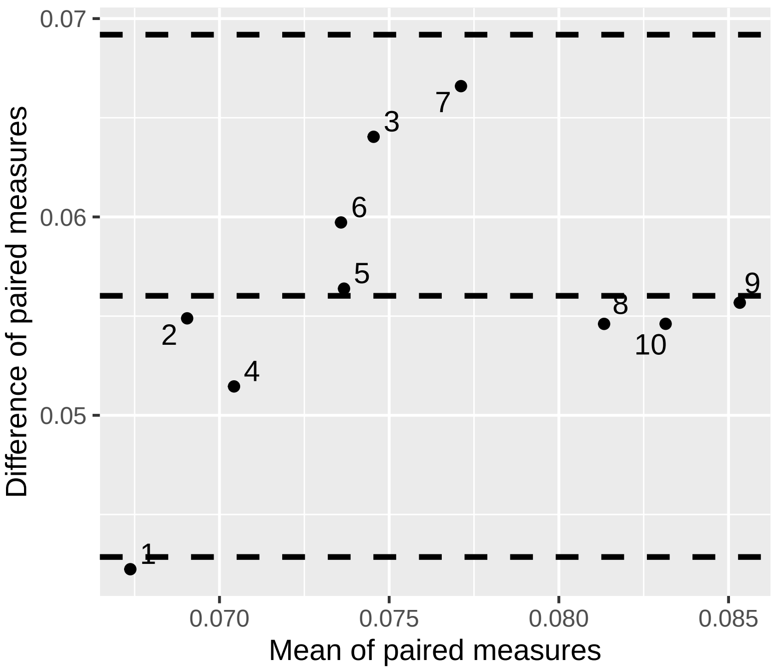


T1 glrlm longrunemphasis 8,16 mm

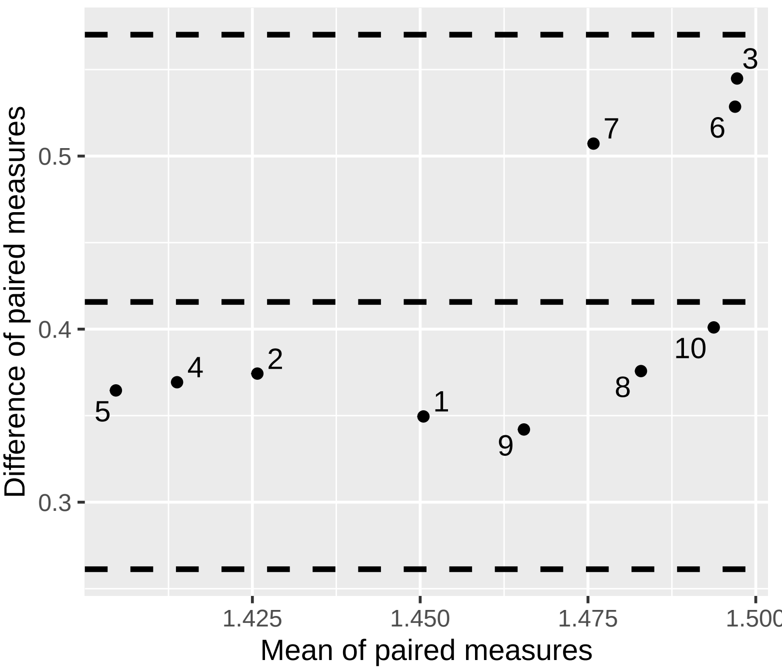


T1 glrlm graylevelvariance 8,16 mm

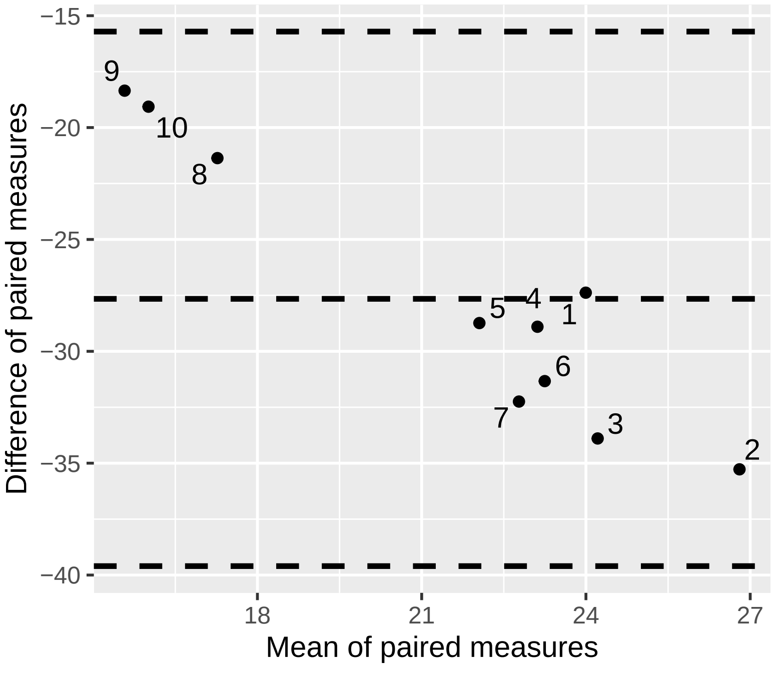


T1 glrlm longrunhighgraylevelemphasis 8,16

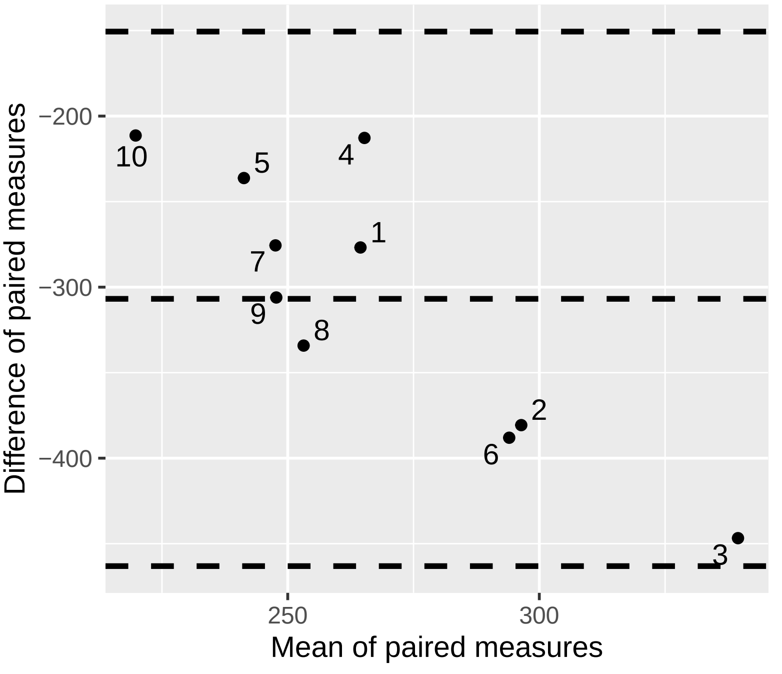

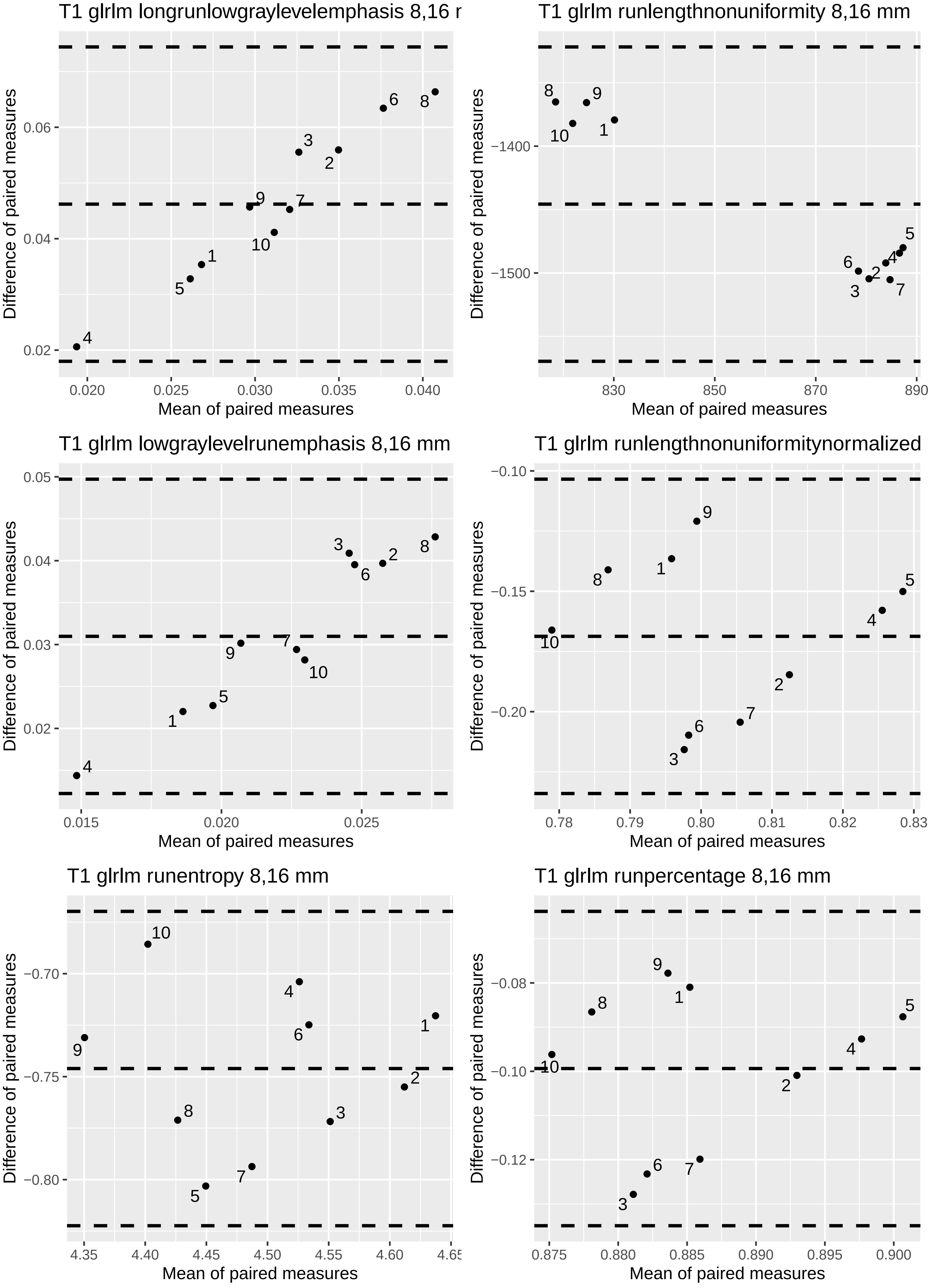
T1 glrlm longrunlowgraylevelemphasis 8,16 r
T1 glrlm runlengthnonuniformity 8,16 mm
T1 glrlm lowgraylevelrunemphasis 8,16 mm
T1 glrlm runlengthnonuniformitynormalized 8
T1 glrlm runentropy 8,16 mm
T1 glrlm runpercentage 8,16 mm
Difference of paired measures
Mean of paired measures

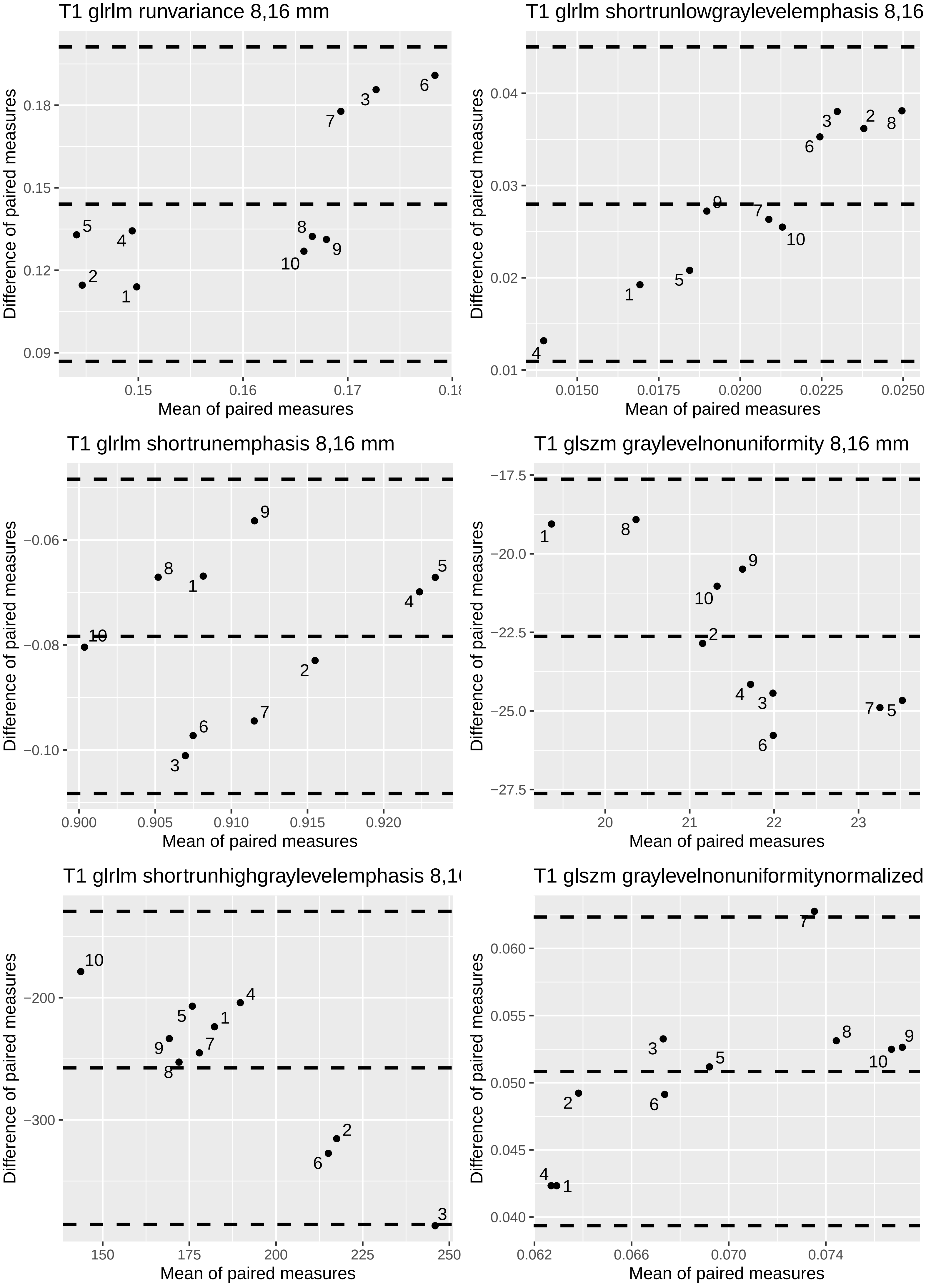
T1 glrlm runvariance 8,16 mm
T1 glrlm shortrunlowgraylevelemphasis 8,16
T1 glrlm shortrunemphasis 8,16 mm
T1 glszm graylevelnonuniformity 8,16 mm
T1 glrlm shortrunhighgraylevelemphasis 8,1
T1 glszm graylevelnonuniformitynormalized
Difference of paired measures
Mean of paired measures

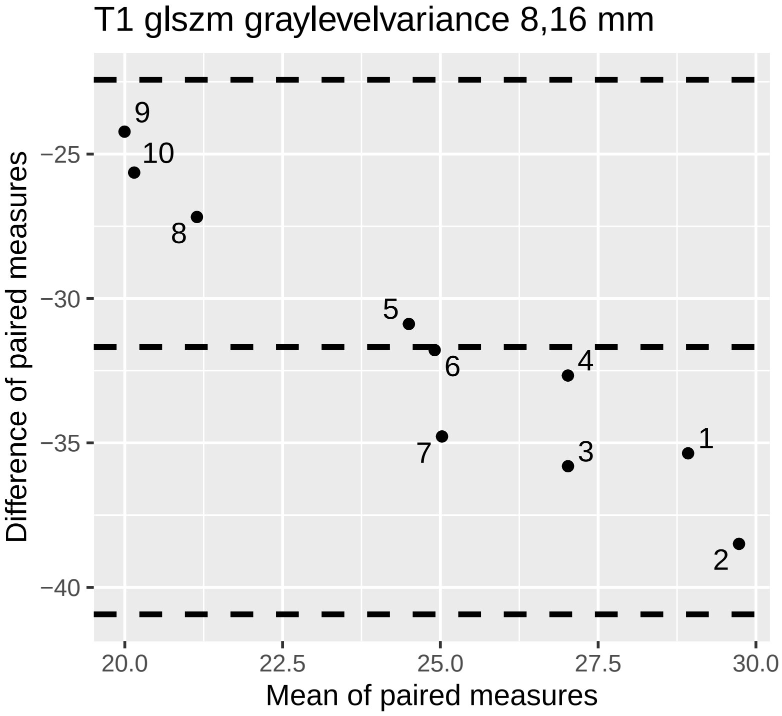
T1 glszm graylevelvariance 8,16 mm
Difference of paired measures
Mean of paired measures
−25
−30
−35
−40
20.0
22.5
25.0
27.5
30.0

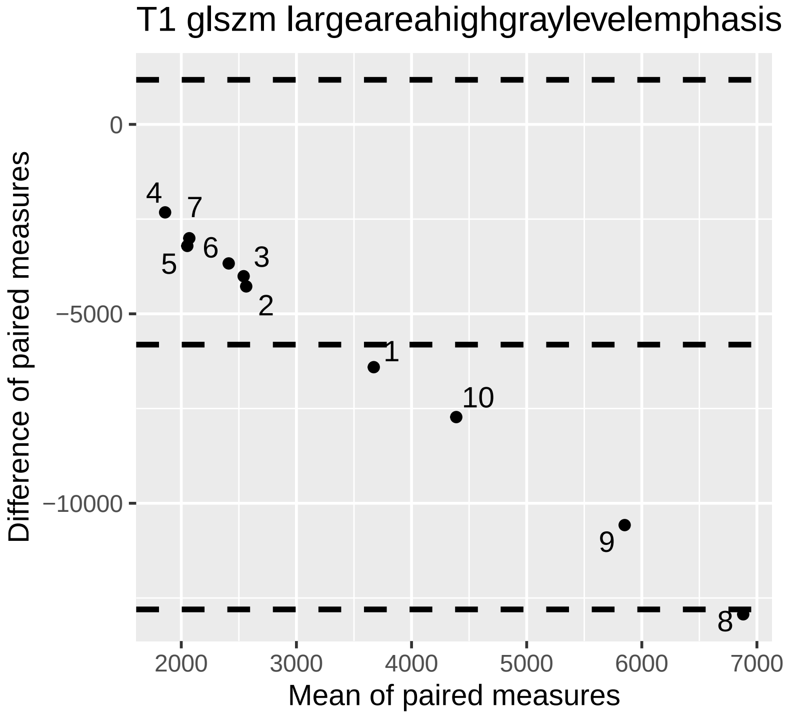
T1 glszm largeareahighgraylevelemphasis
Difference of paired measures
Mean of paired measures
0
−5000
−10000
2000
3000
4000
5000
6000
7000

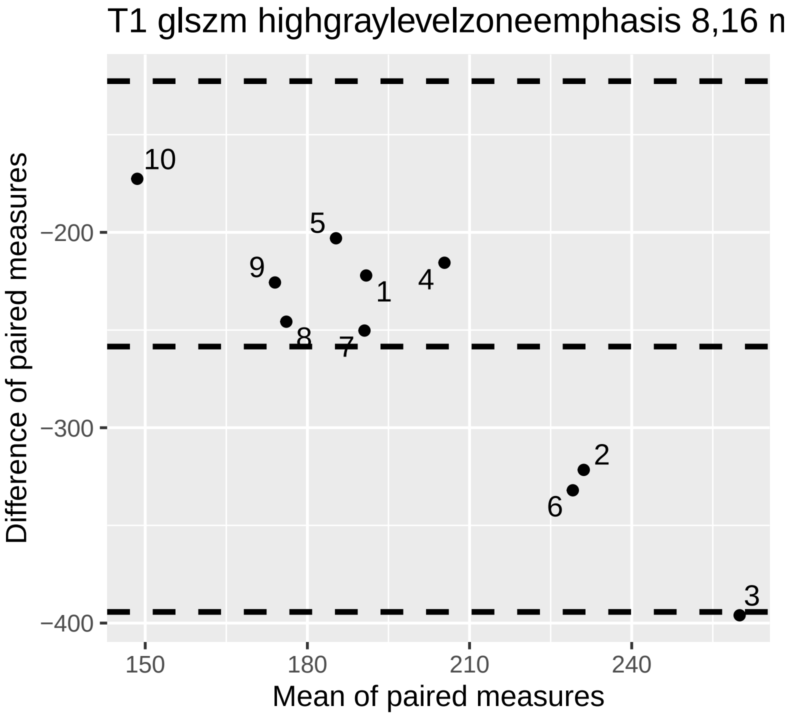
T1 glszm highgraylevelzoneemphasis 8,16 m
Difference of paired measures
Mean of paired measures
−200
−300
−400
150
180
210
240

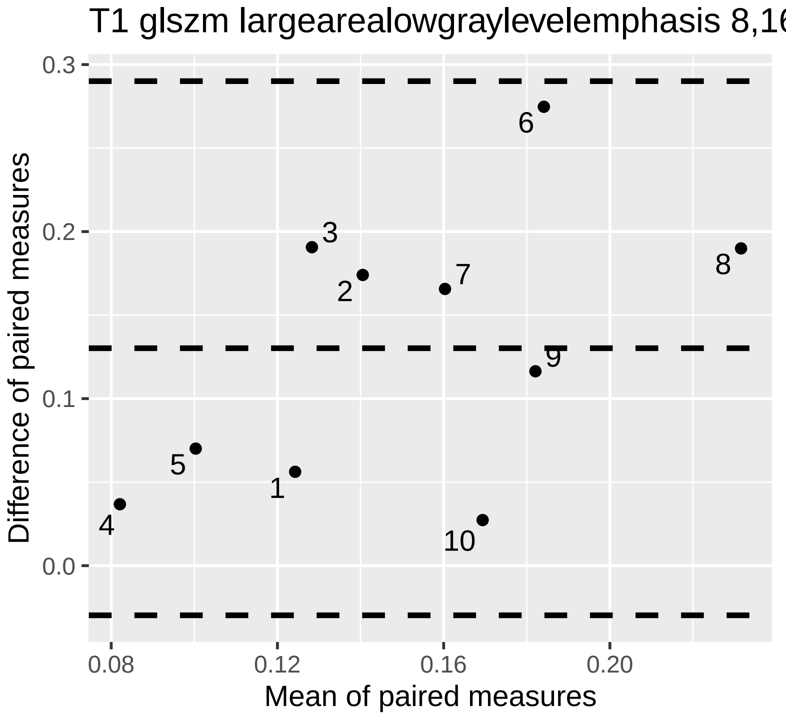
T1 glszm largearealowgraylevelemphasis 8,1
Difference of paired measures
Mean of paired measures
0.3
0.2
0.1
0.0
0.08
0.12
0.16
0.20

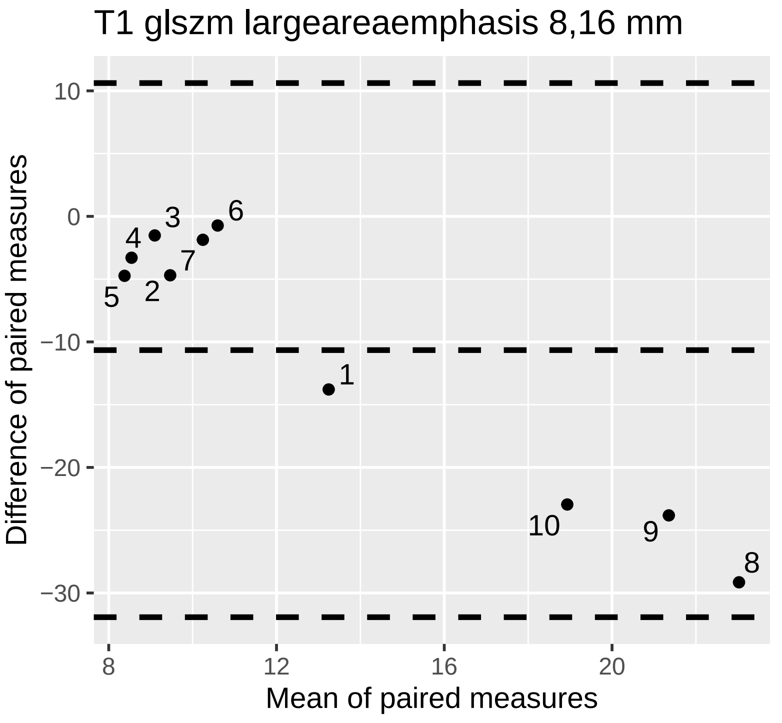
T1 glszm largeareaemphasis 8,16 mm
Difference of paired measures
Mean of paired measures
10
0
−10
−20
−30
8
12
16
20

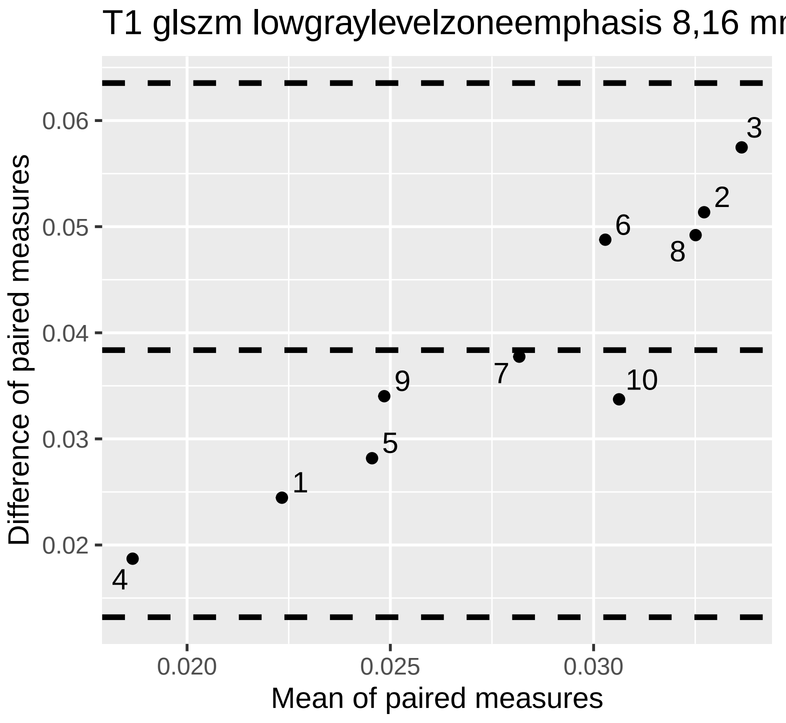
T1 glszm lowgraylevelzoneemphasis 8,16 m
Difference of paired measures
Mean of paired measures
0.06
0.05
0.04
0.03
0.02
0.020
0.025
0.030

T1 glszm sizezonenonuniformity 8,16 mm

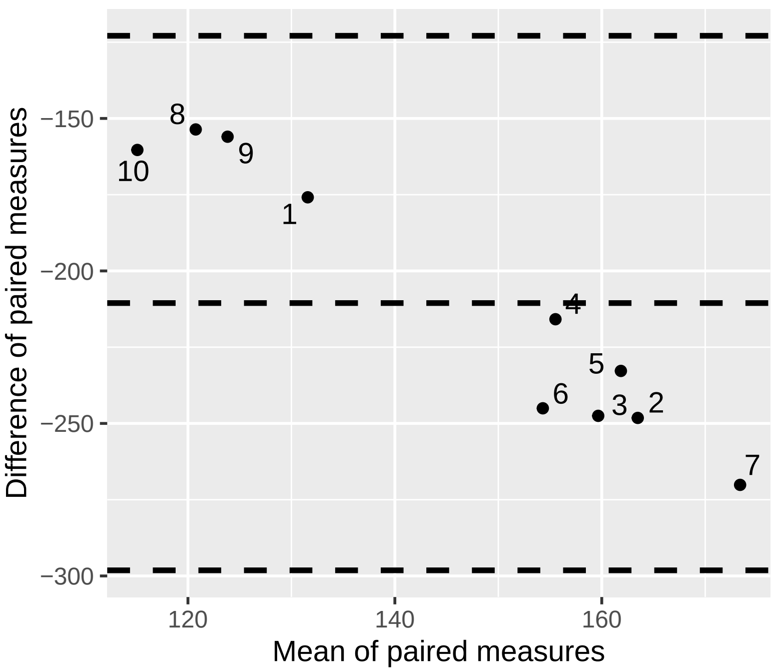


T1 glszm smallareahighgraylevelemphasis 8

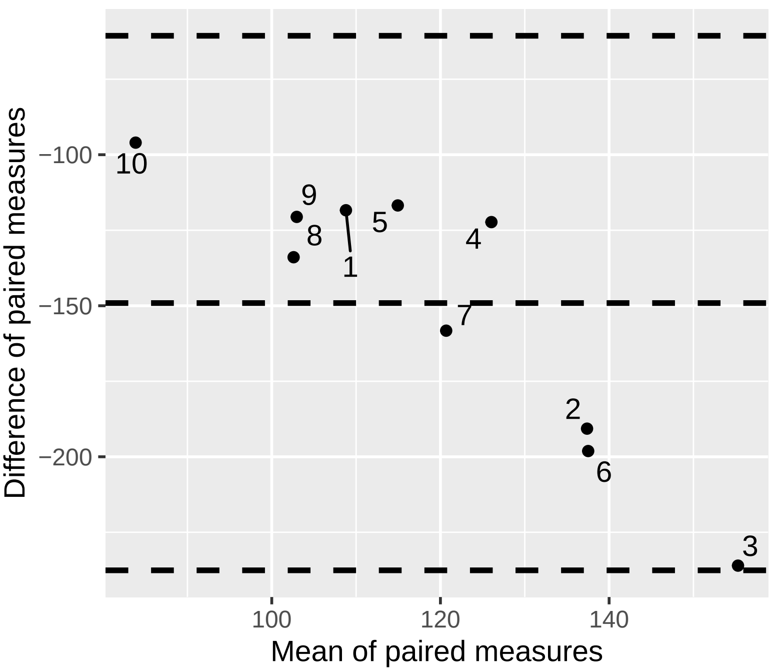


T1 glszm sizezonenonuniformitynormalized 8

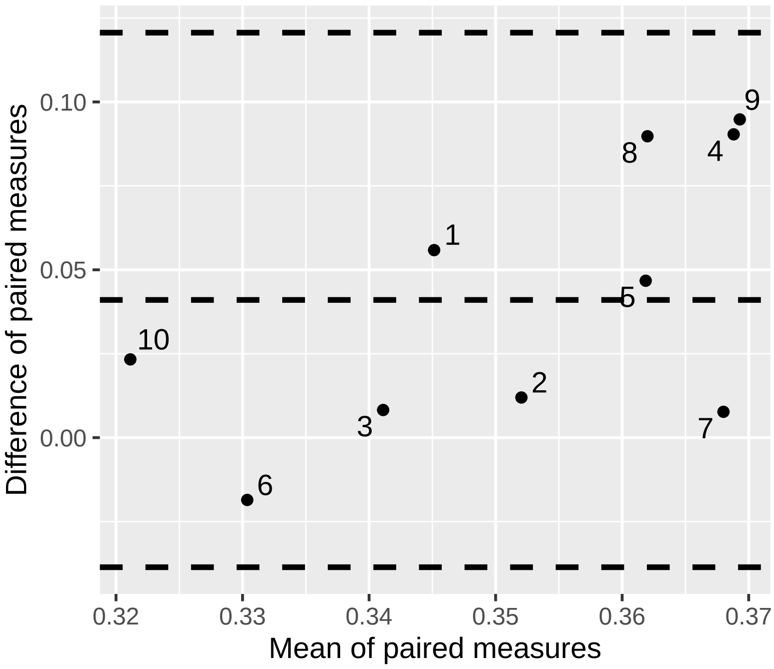


T1 glszm smallarealowgraylevelemphasis 8,1

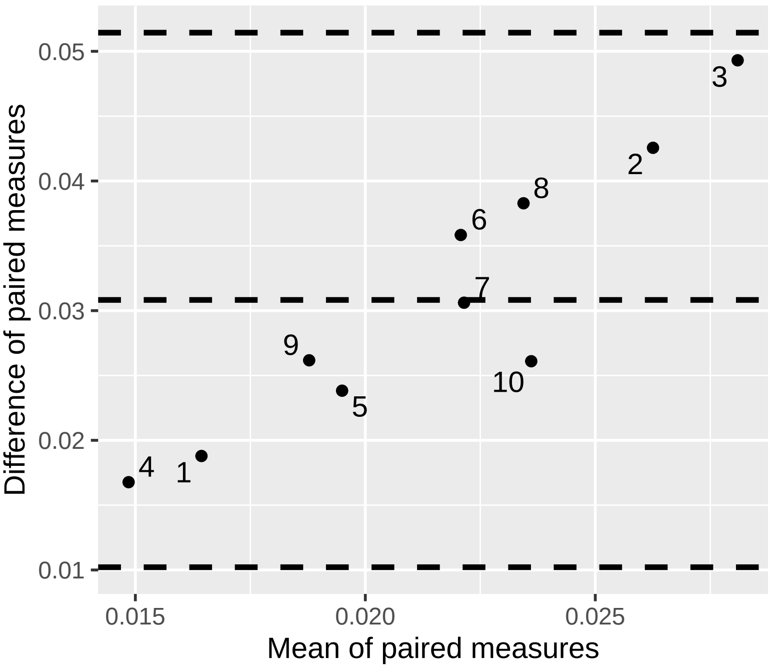


T1 glszm smallareaemphasis 8,16 mm

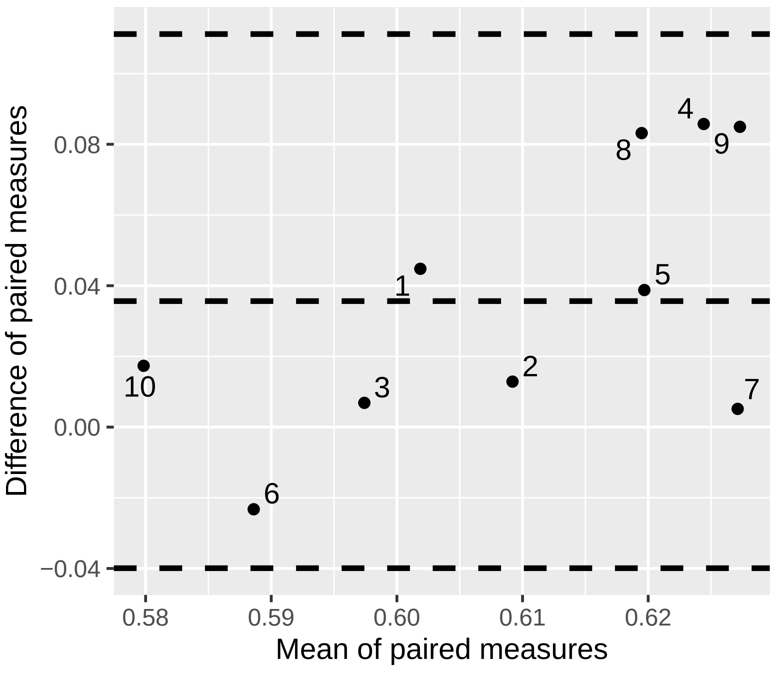


T1 glszm zoneentropy 8,16 mm

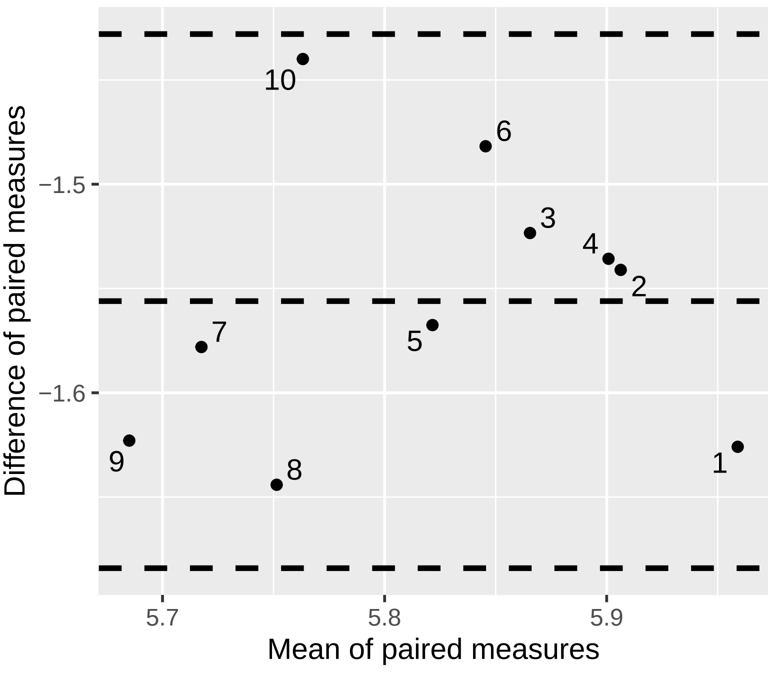

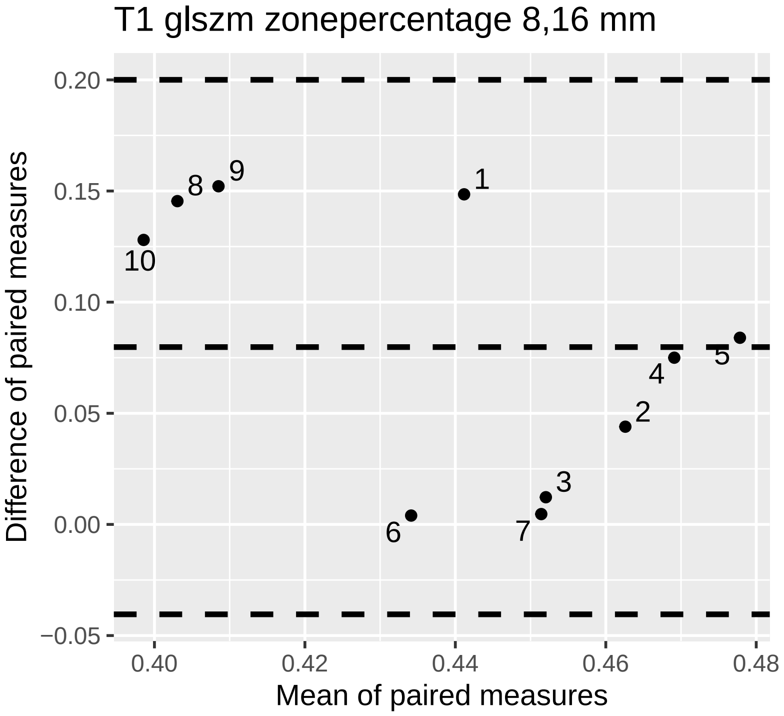
T1 glszm zonepercentage 8,16 mm
Difference of paired measures
Mean of paired measures
0.20
0.15
0.10
0.05
0.00
−0.05
0.40
0.42
0.44
0.46
0.48

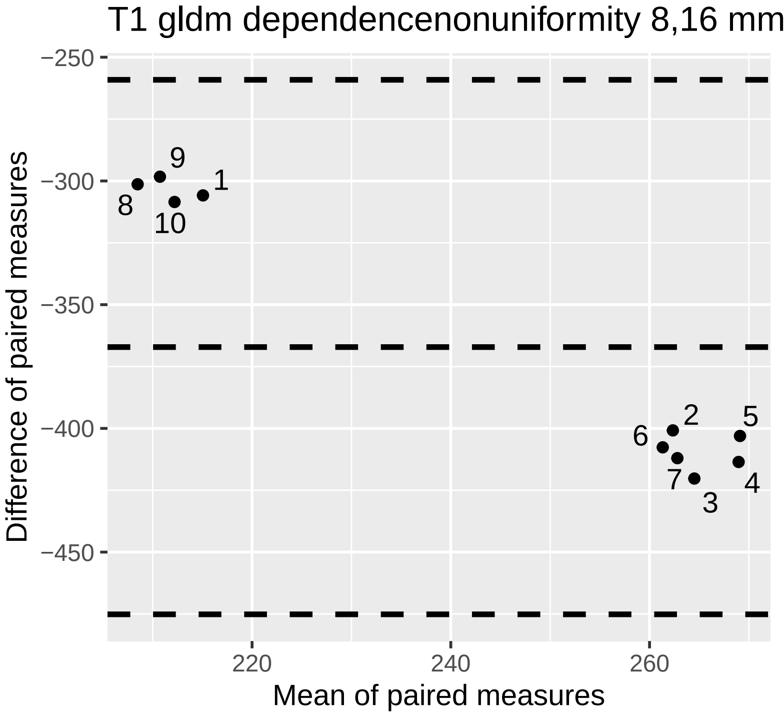
T1 gldm dependencenonuniformity 8,16 mm
Difference of paired measures
Mean of paired measures
−250
−300
−350
−400
−450
220
240
260

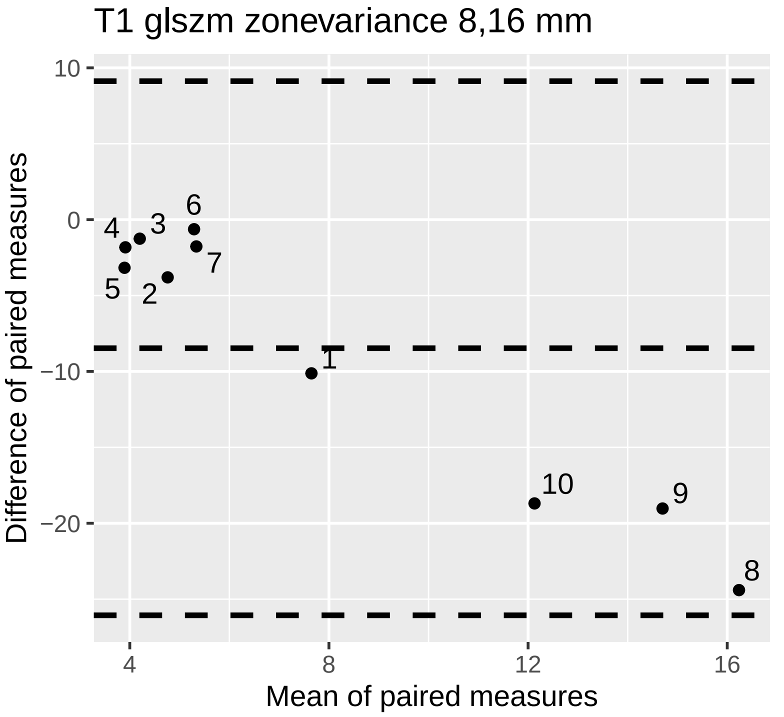
T1 glszm zonevariance 8,16 mm
Difference of paired measures
Mean of paired measures
10
0
−10
−20
4
8
12
16

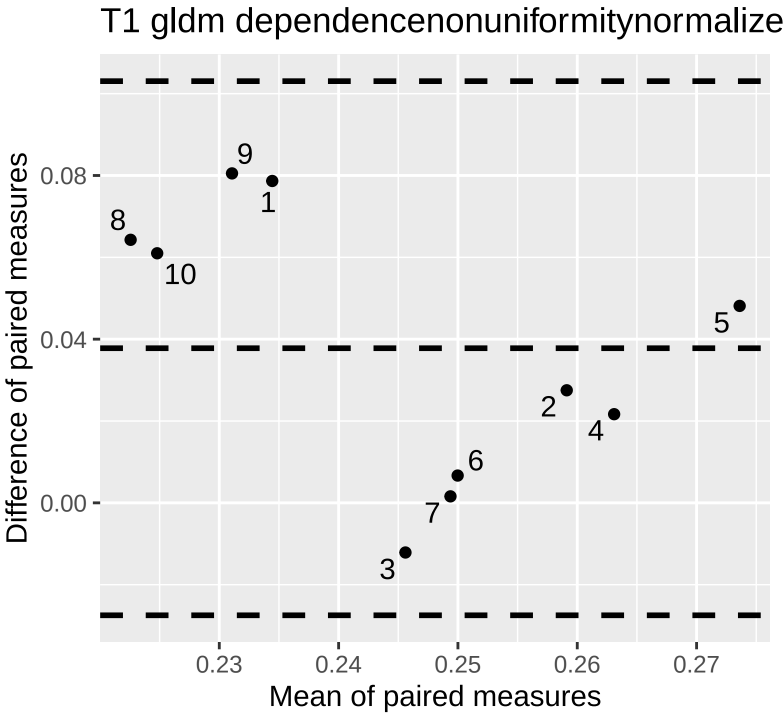
T1 gldm dependencenonuniformitynormalize
Difference of paired measures
Mean of paired measures
0.08
0.04
0.00
0.23
0.24
0.25
0.26
0.27

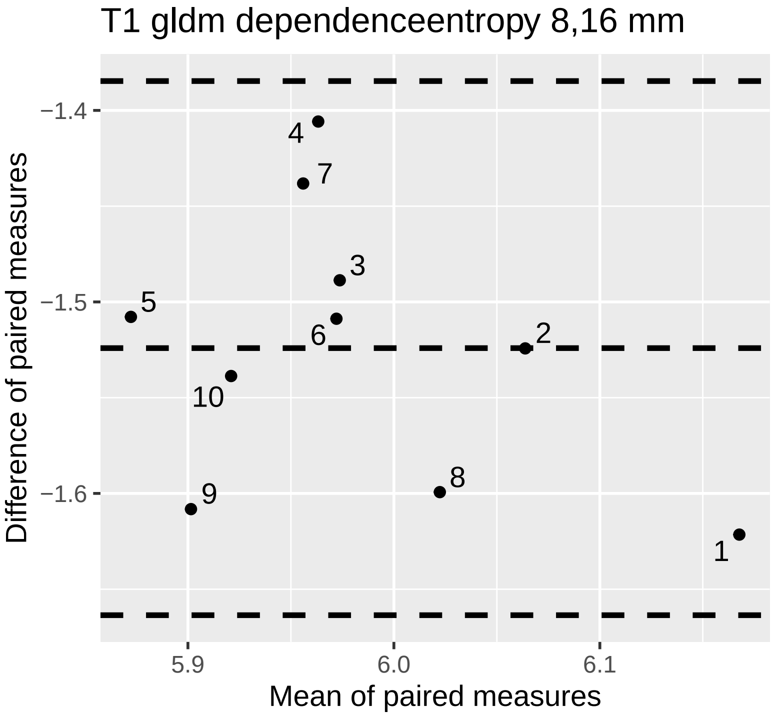
T1 gldm dependenceentropy 8,16 mm
Difference of paired measures
Mean of paired measures
−1.4
−1.5
−1.6
5.9
6.0
6.1

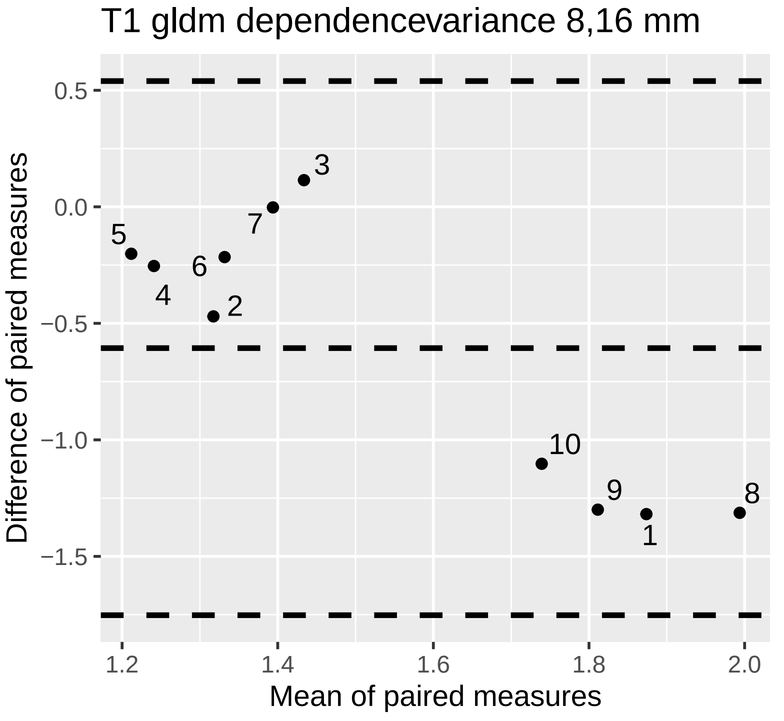
T1 gldm dependencevariance 8,16 mm
Difference of paired measures
Mean of paired measures
0.5
0.0
−0.5
−1.0
−1.5
1.2
1.4
1.6
1.8
2.0

T1 gldm graylevelnonuniformity 8,16 mm

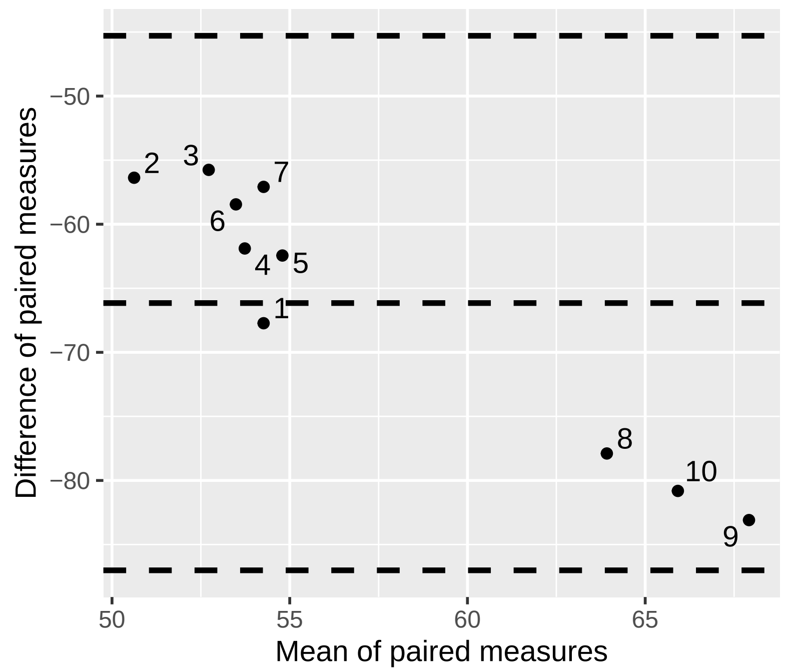


T1 gldm largedependenceemphasis 8,16 mm

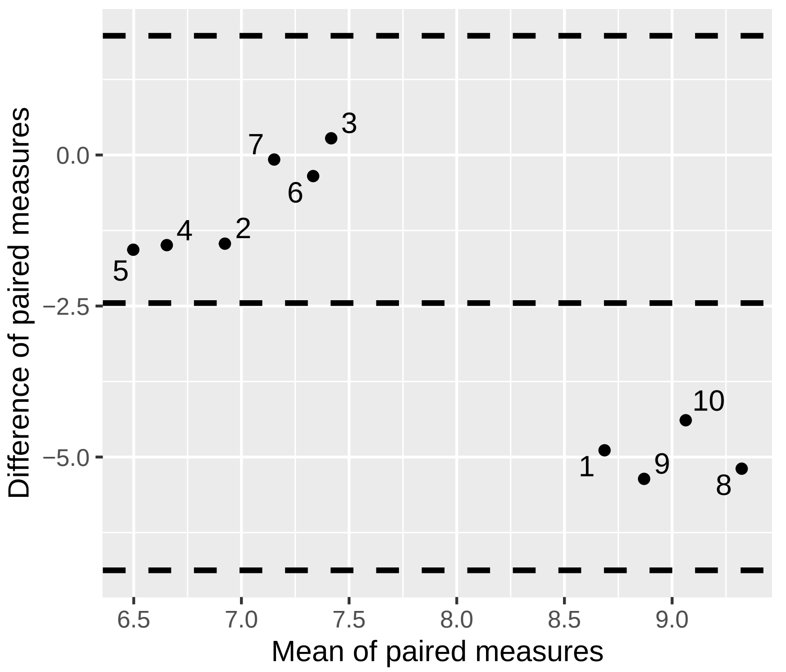


T1 gldm graylevelvariance 8,16 mm

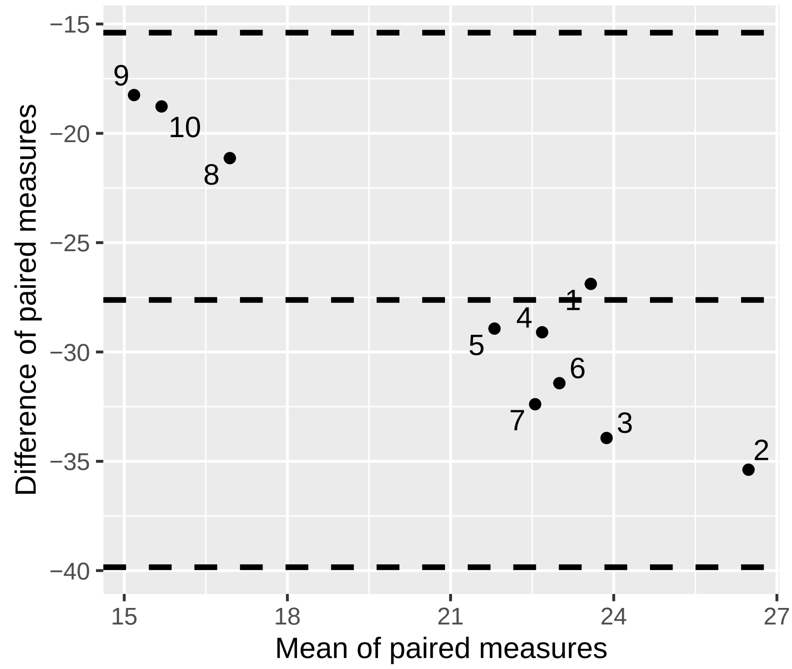


T1 gldm largedependencehighgraylevelemp

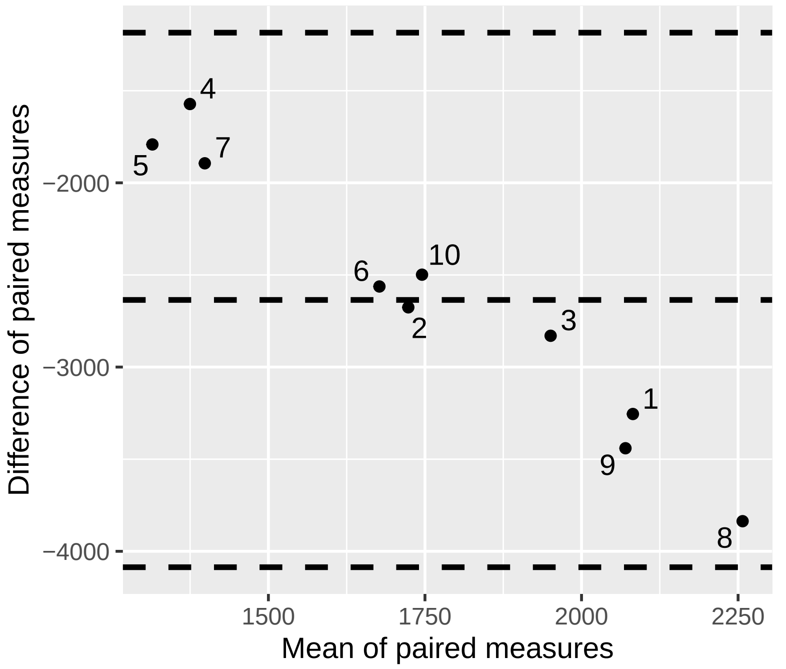


T1 gldm highgraylevelemphasis 8,16 mm

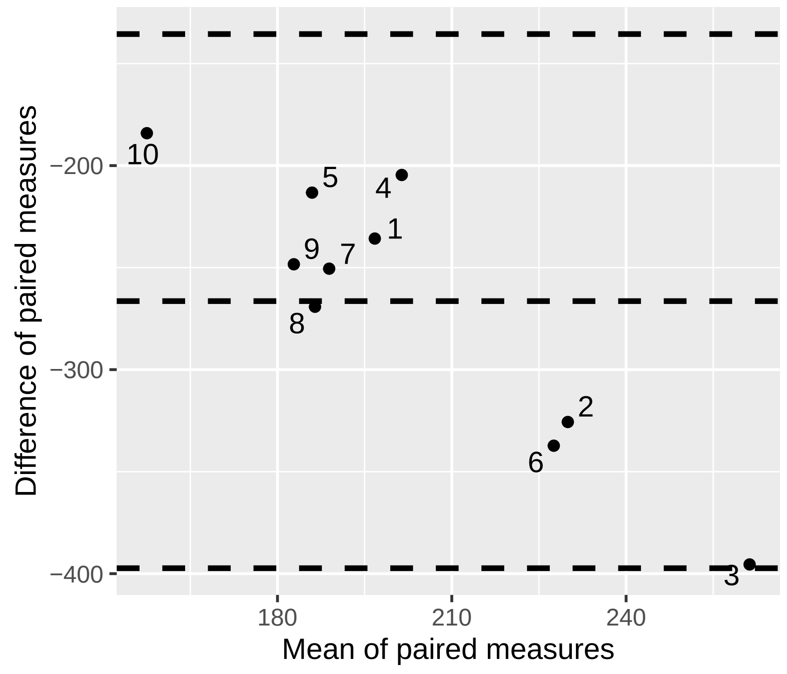


T1 gldm largedependencelowgraylevelempha

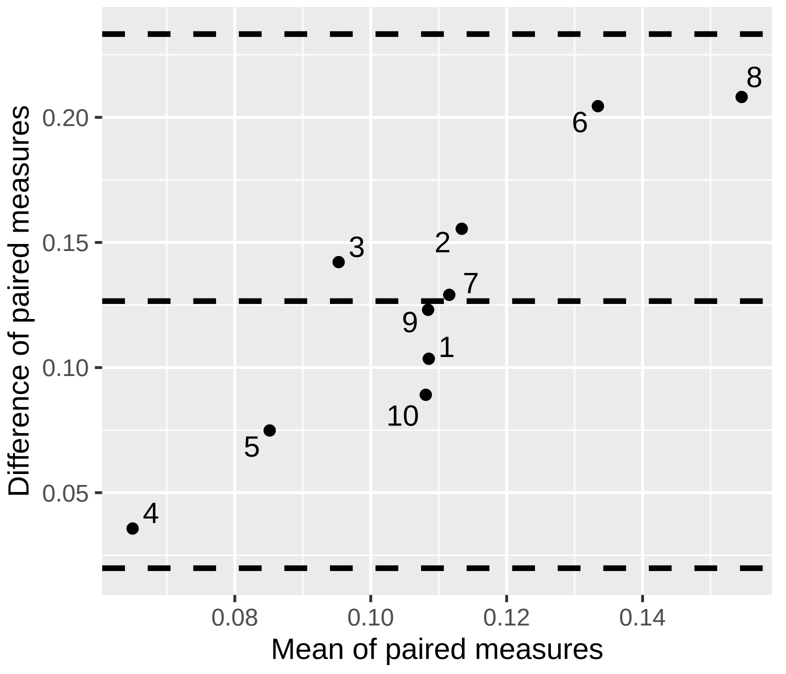

T1 gldm lowgraylevelemphasis 8,16 mm

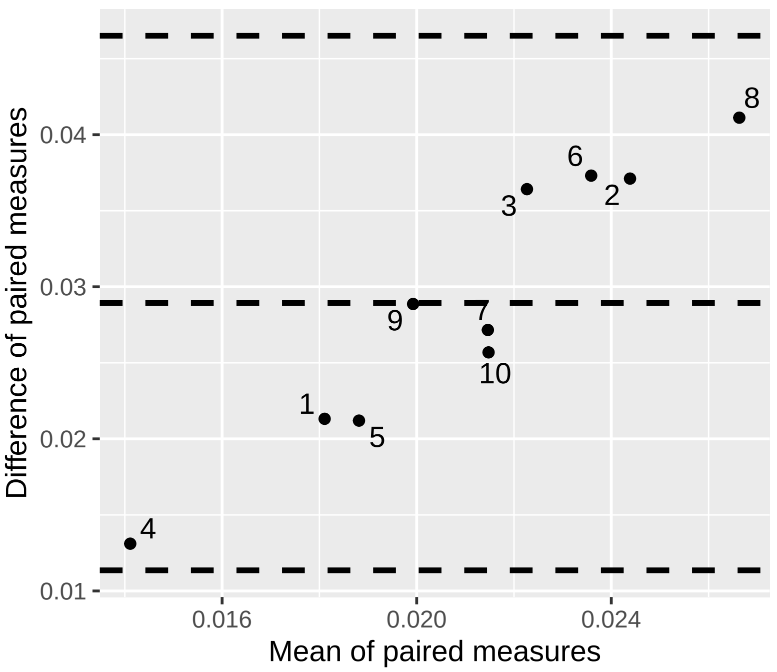


T1 gldm smalldependencelowgraylevelemph

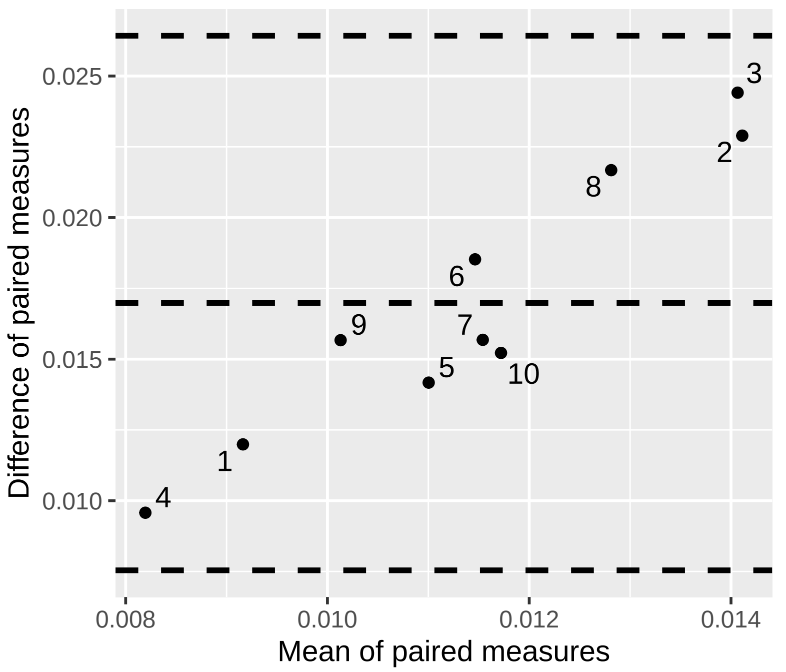


T1 gldm smalldependenceemphasis 8,16 mn

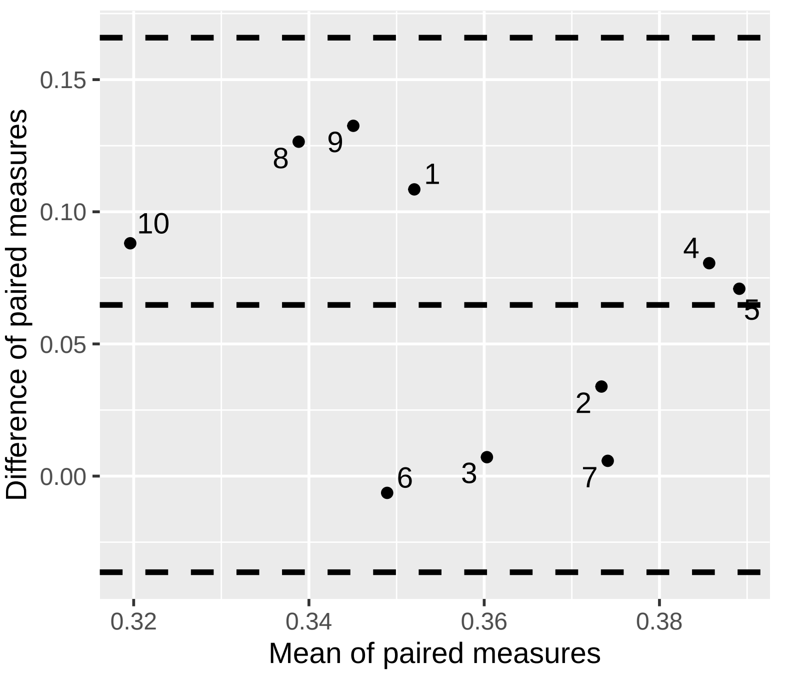


T1 ngtdm busyness 8,16 mm

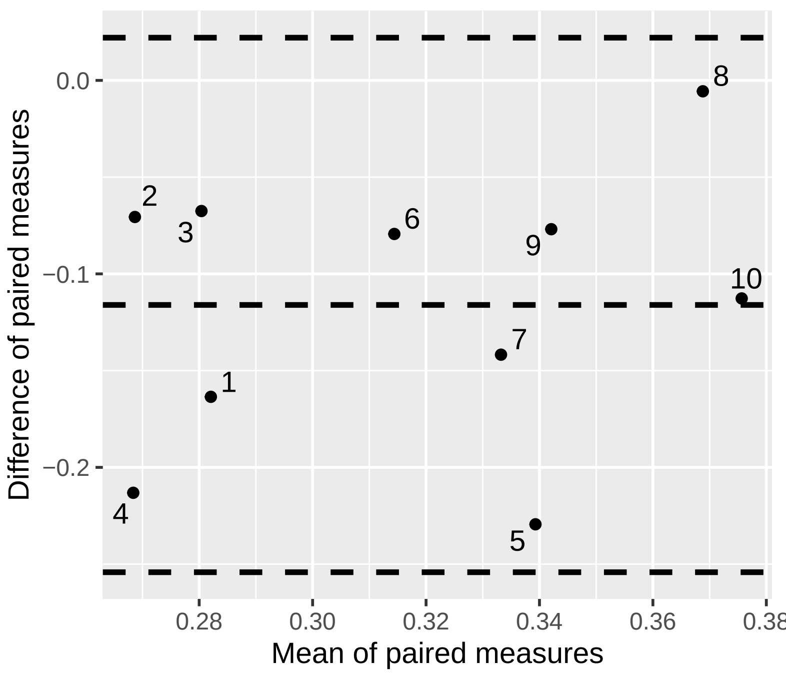


T1 gldm smalldependencehighgraylevelemp

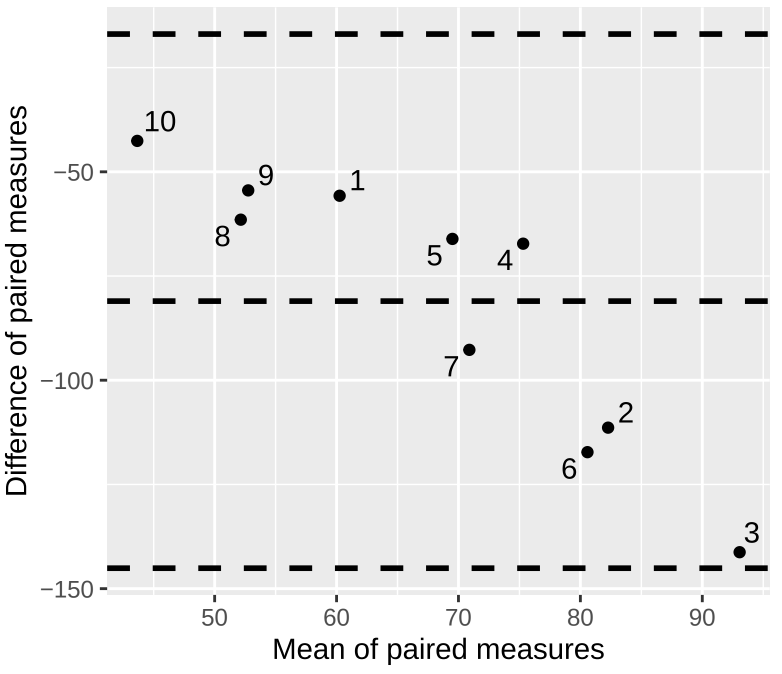


T1 ngtdm coarseness 8,16 mm

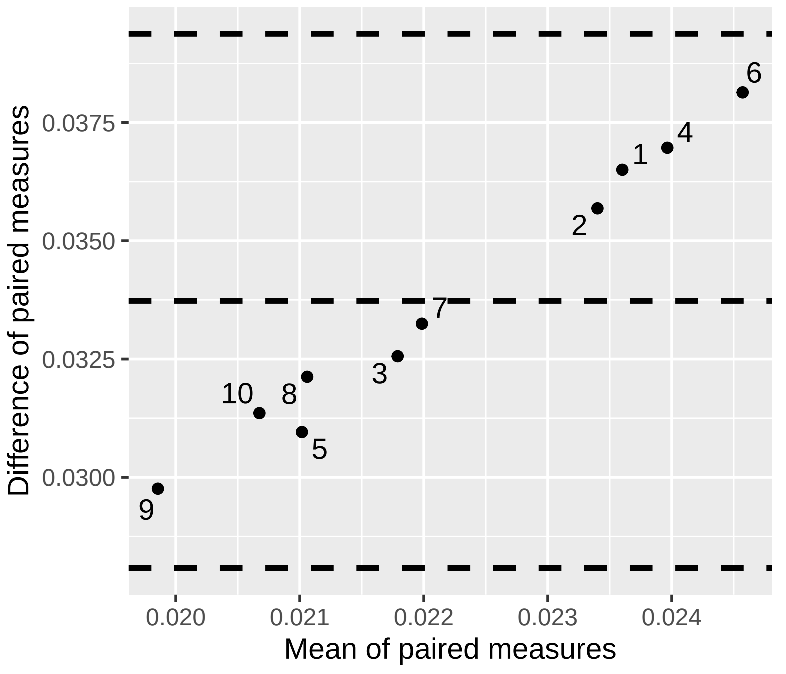

T1 ngtdm complexity 8,16 mm

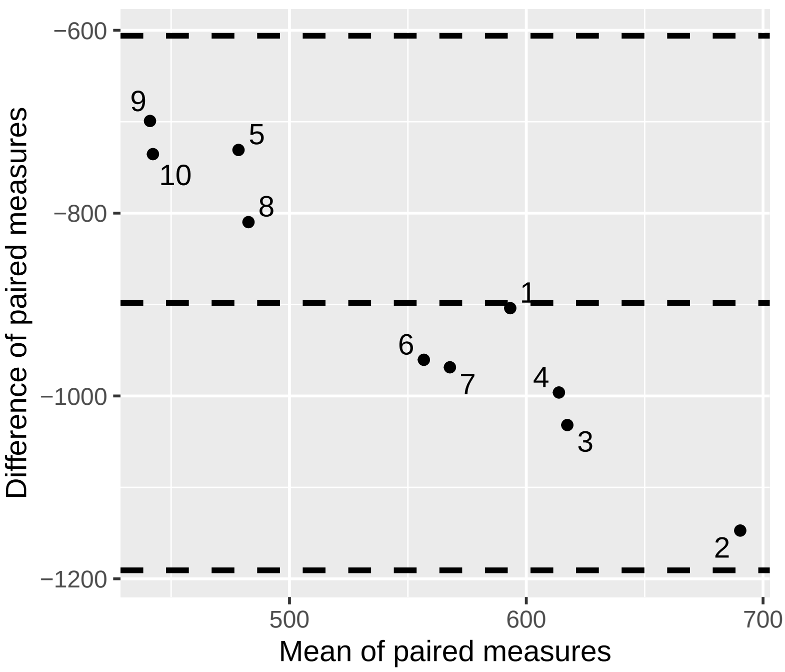


T1 firstorder 10percentile 8,16 px

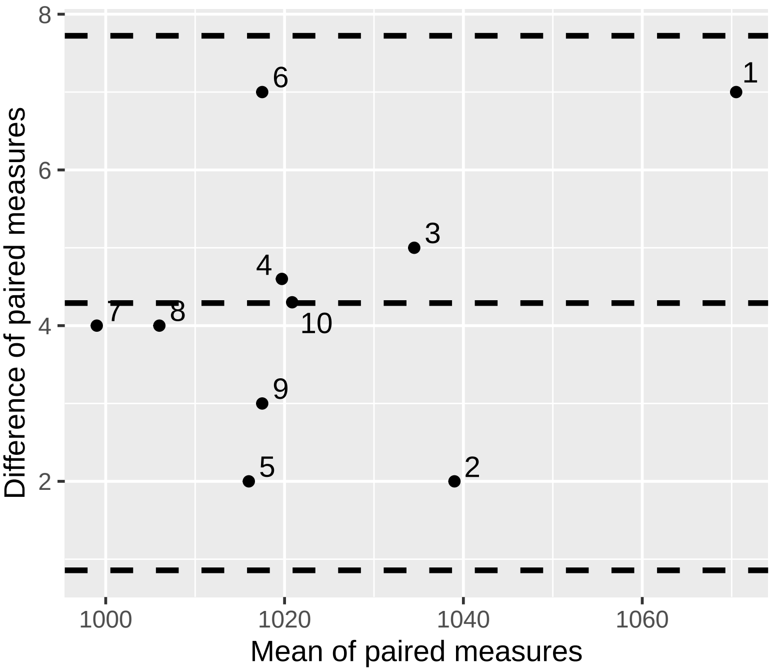


T1 ngtdm contrast 8,16 mm

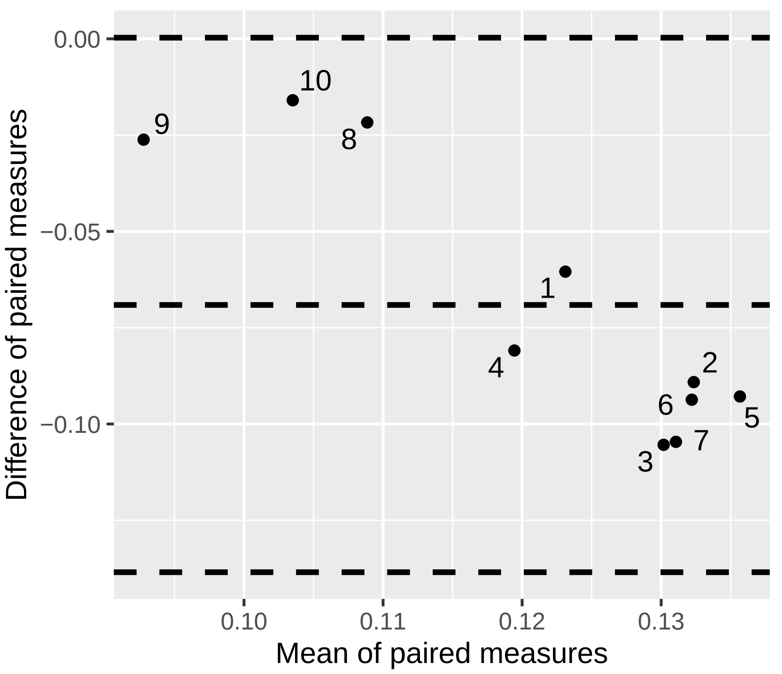


T1 firstorder 90percentile 8,16 px

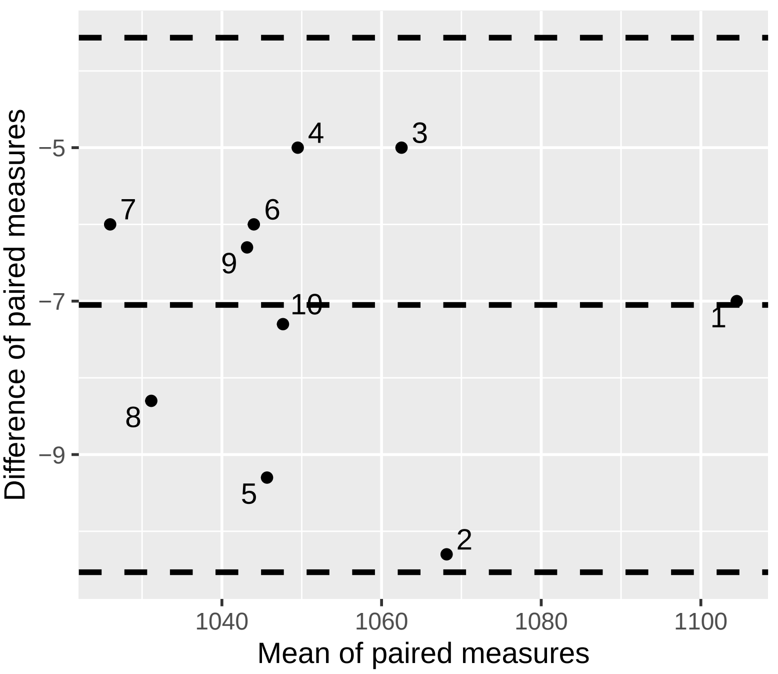


T1 ngtdm strength 8,16 mm

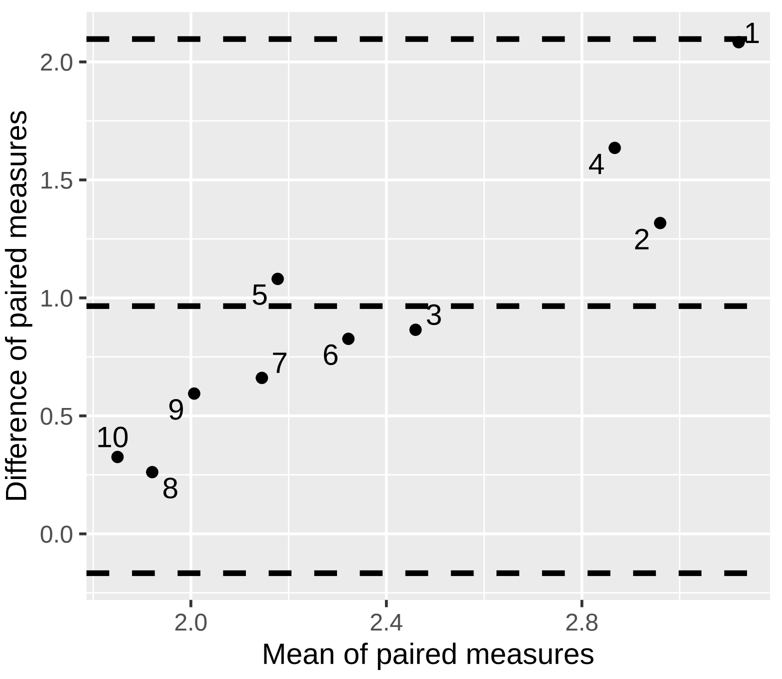


T1 firstorder energy 8,16 px

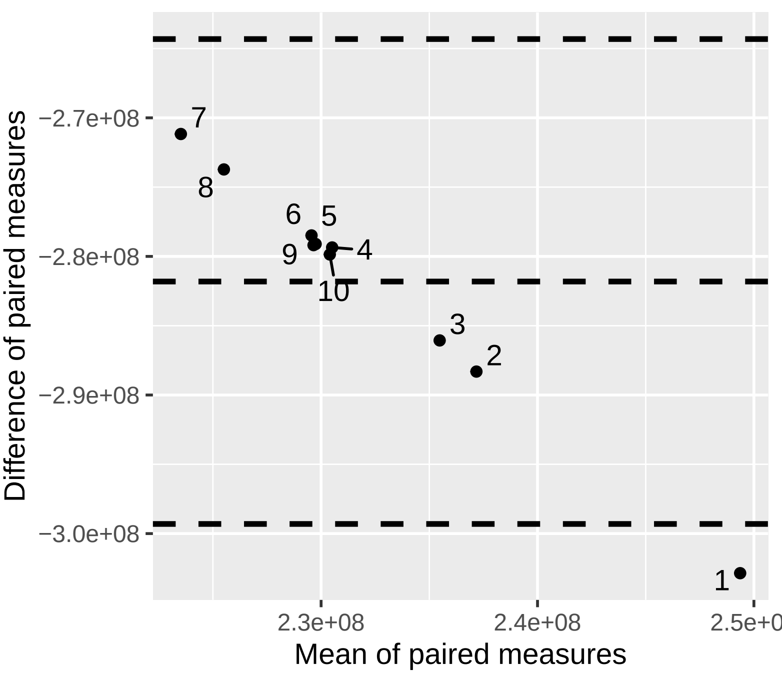

## T1 firstorder entropy 8,16 px

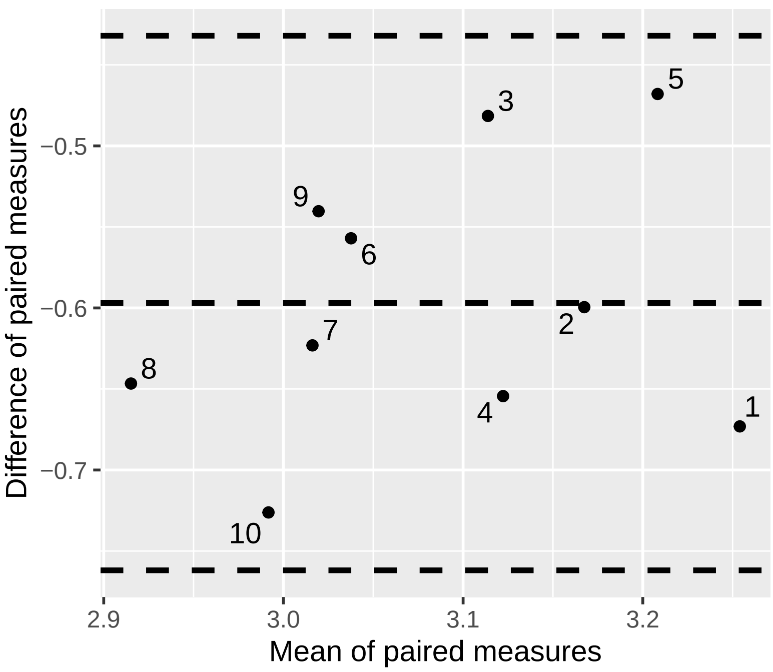


## T1 firstorder maximum 8,16 px

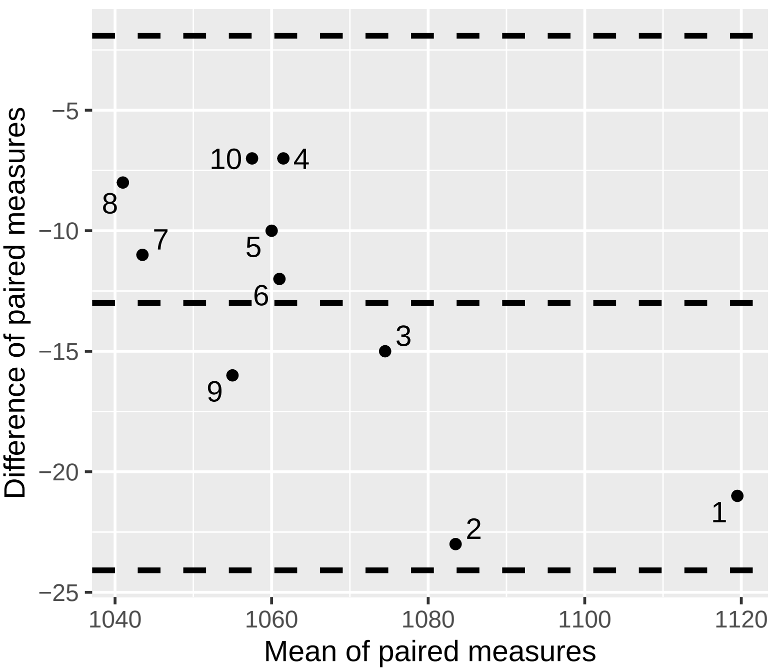


## T1 firstorder interquartilerange 8,16 px

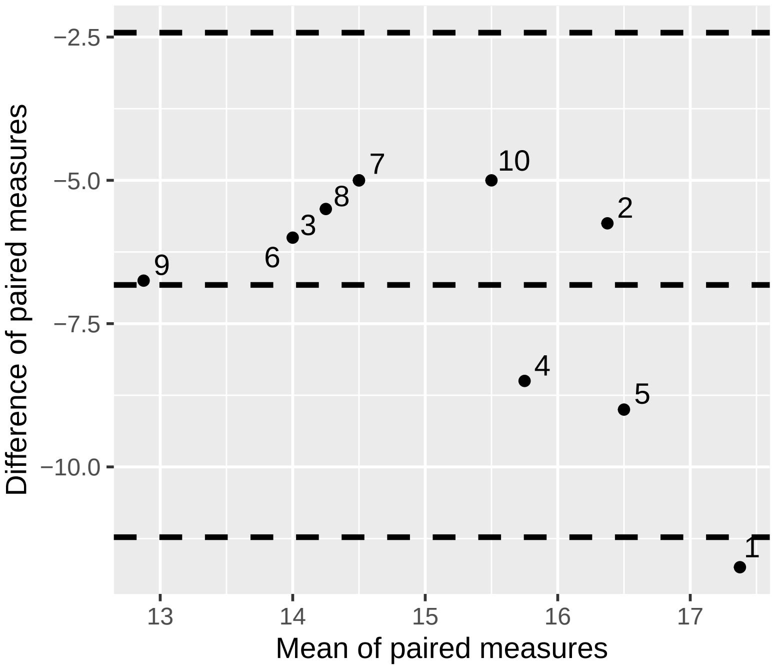


## T1 firstorder meanabsolutedeviation 8,16 px

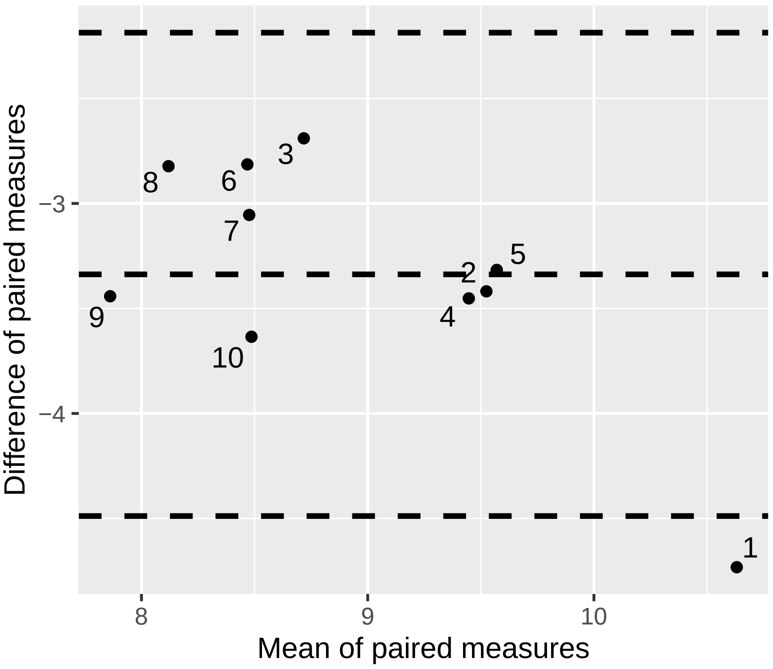


## T1 firstorder kurtosis 8,16 px

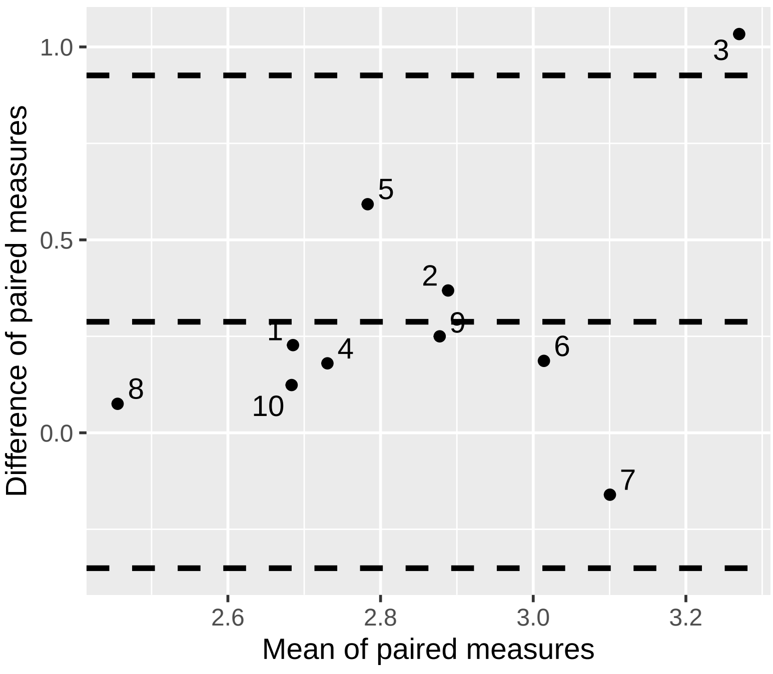


## T1 firstorder mean 8,16 px

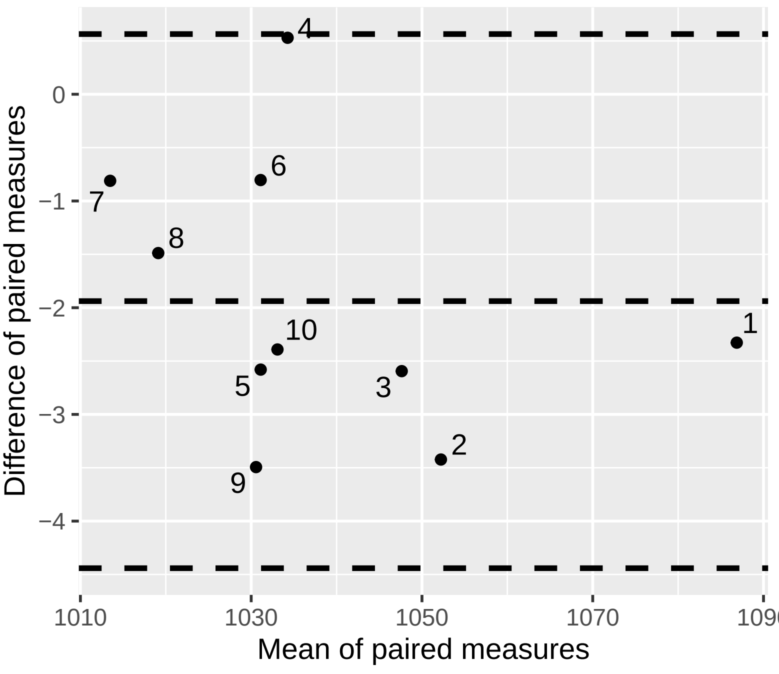

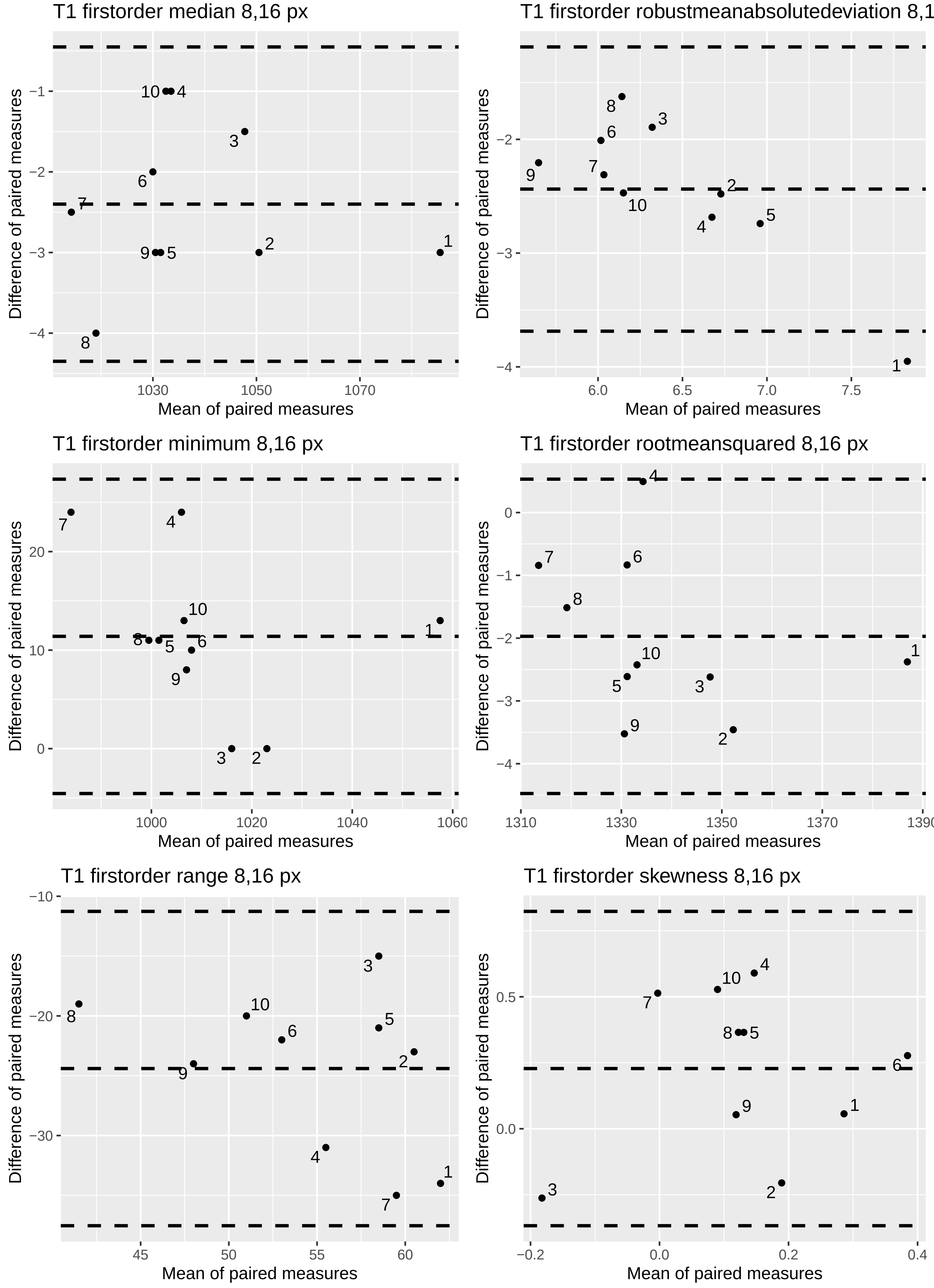
T1 firstorder median 8,16 px
Difference of paired measures
Mean of paired measures
T1 firstorder robustmeanabsolutedeviation 8,1
Difference of paired measures
Mean of paired measures
T1 firstorder minimum 8,16 px
Difference of paired measures
Mean of paired measures
T1 firstorder rootmeansquared 8,16 px
Difference of paired measures
Mean of paired measures
T1 firstorder range 8,16 px
Difference of paired measures
Mean of paired measures
T1 firstorder skewness 8,16 px
Difference of paired measures
Mean of paired measures

T1 firstorder totalenergy 8,16 px

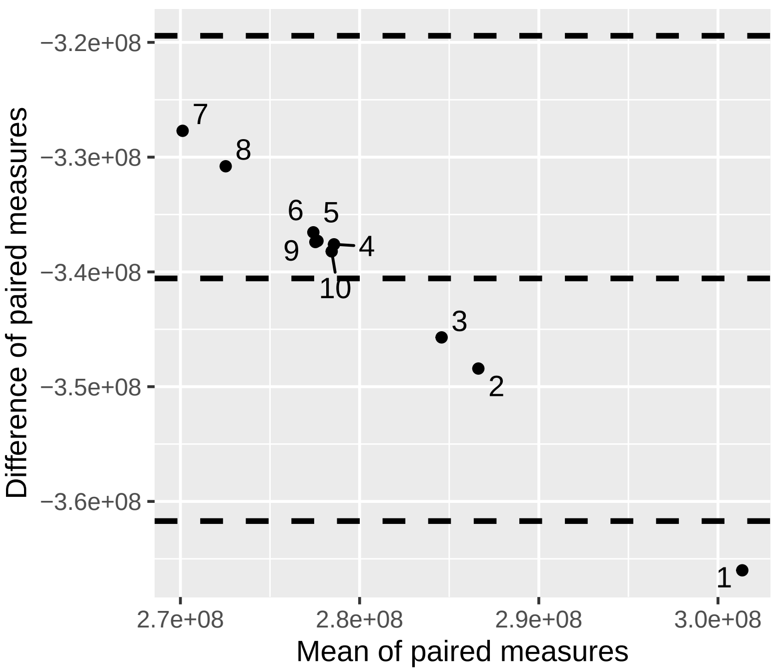


T1 glcm autocorrelation 8,16 px

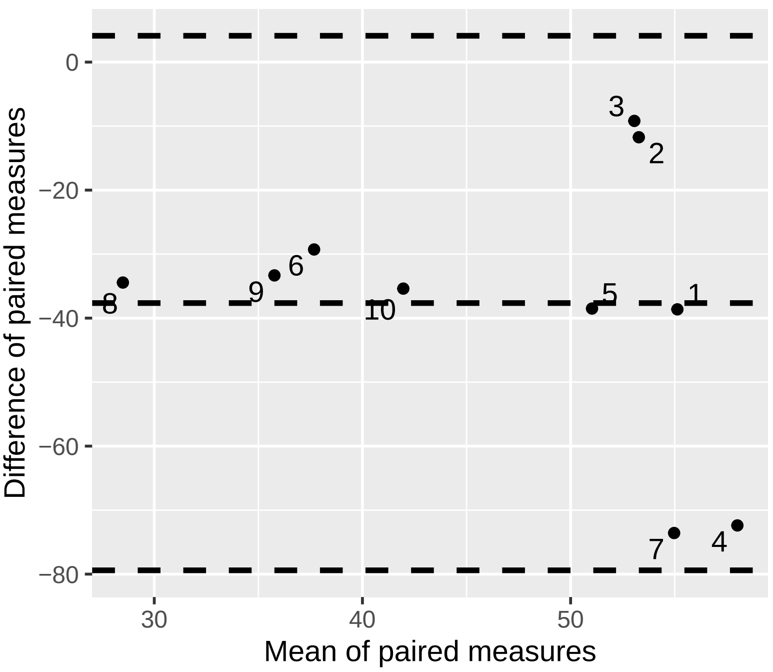


T1 firstorder uniformity 8,16 px

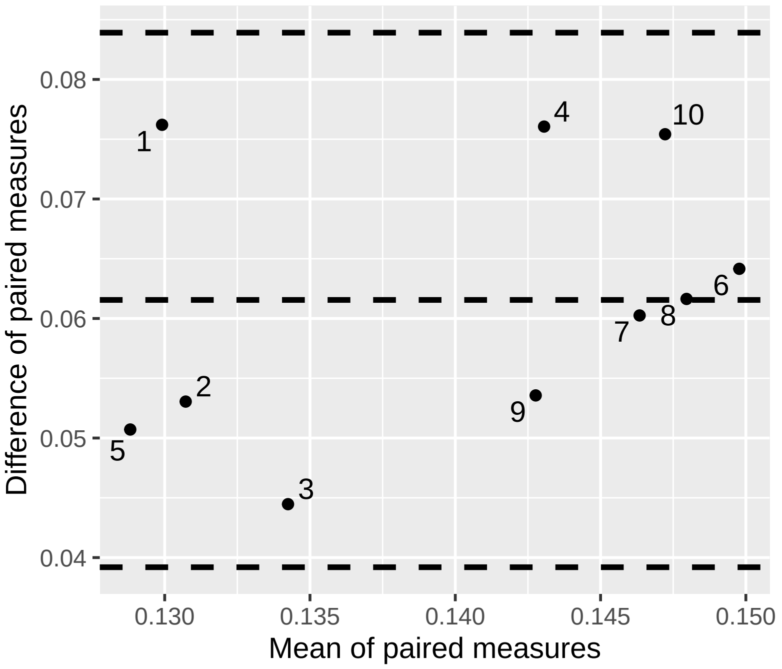


T1 glcm clusterprominence 8,16 px

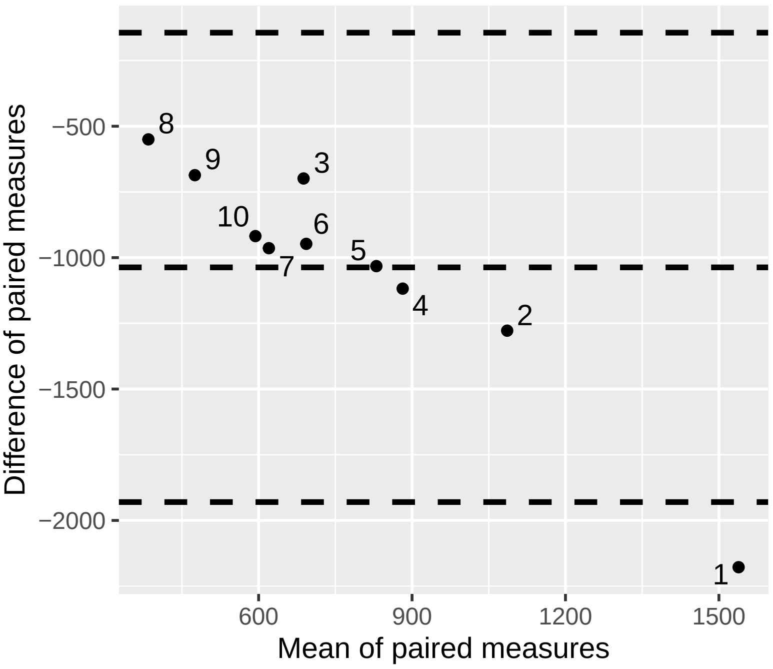


T1 firstorder variance 8,16 px

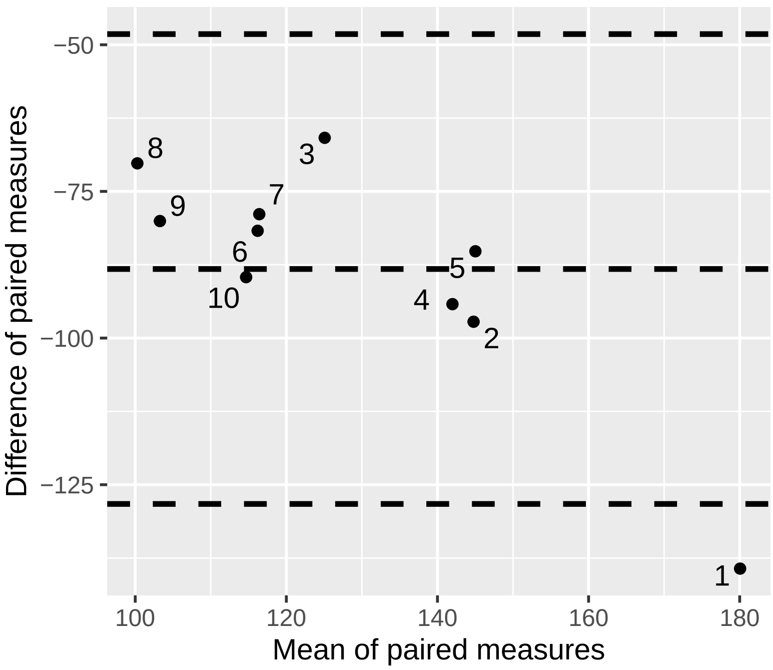


T1 glcm clustershade 8,16 px

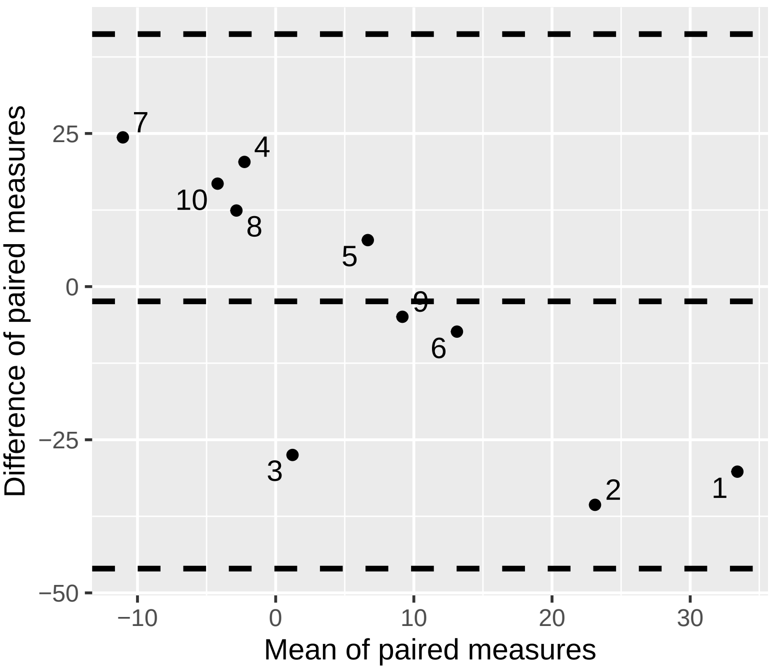

T1 glcm clustertendency 8,16 px

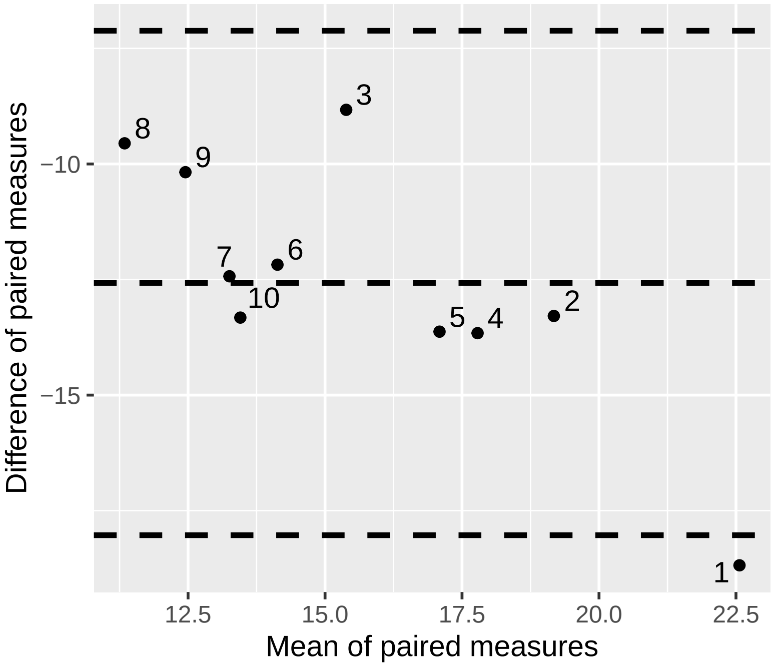


T1 glcm differenceaverage 8,16 px

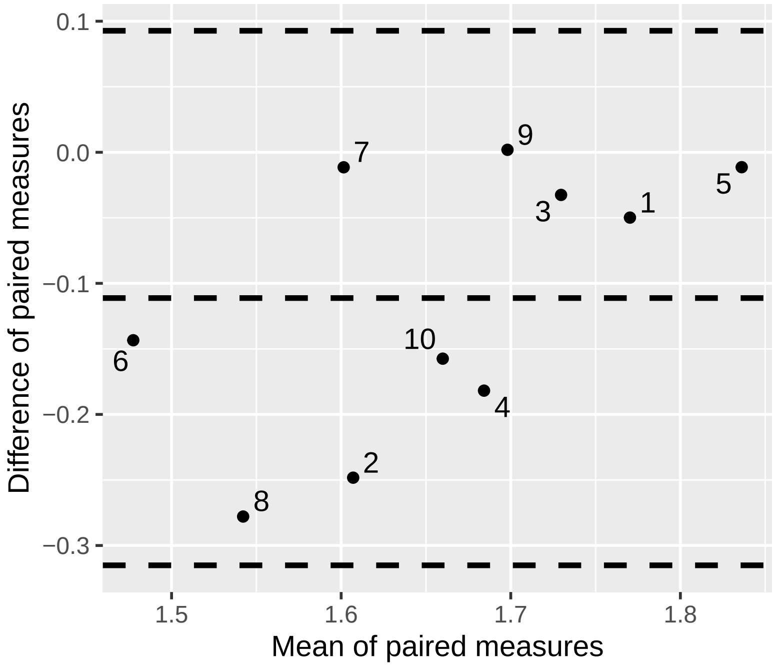


T1 glcm contrast 8,16 px

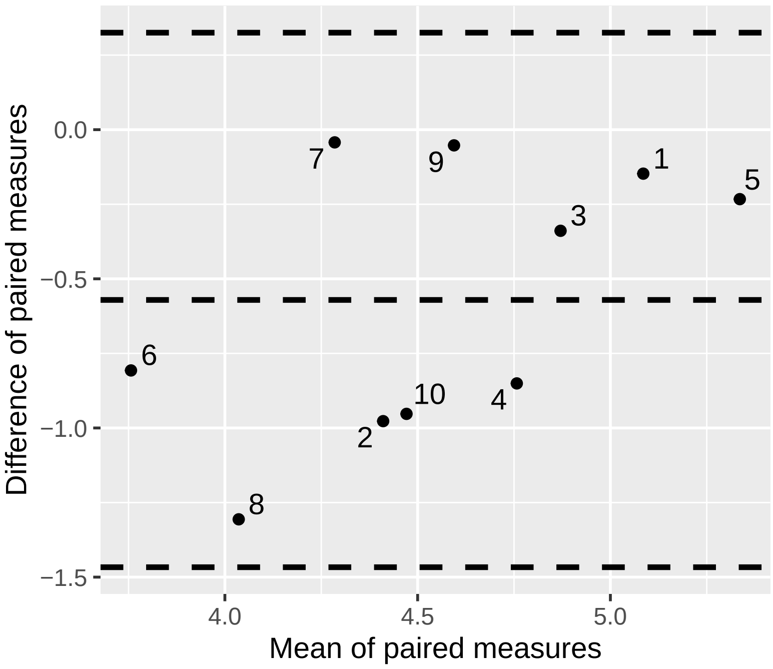


T1 glcm differenceentropy 8,16 px

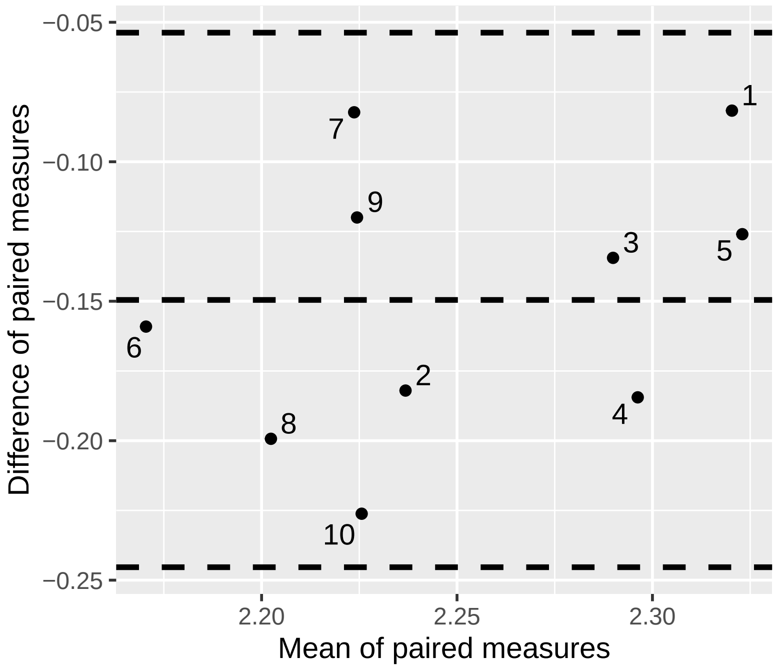


T1 glcm correlation 8,16 px

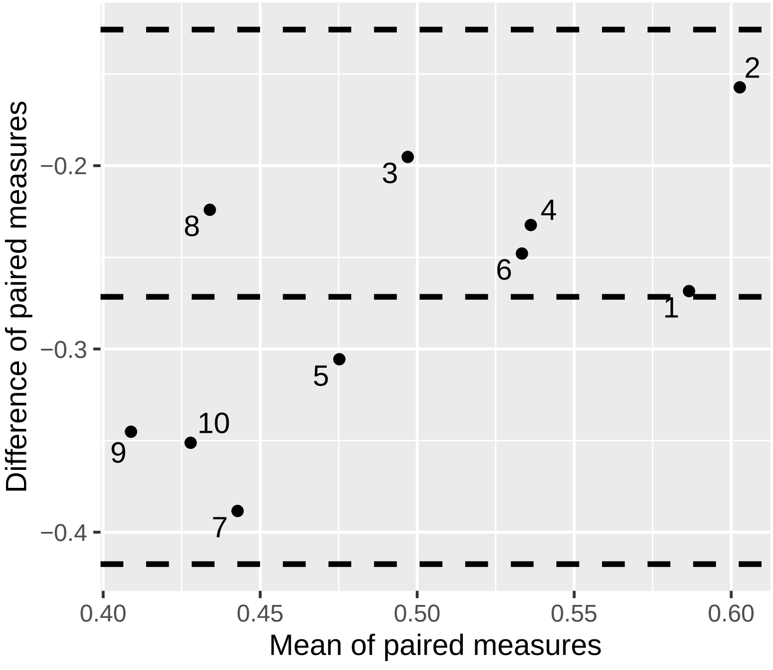


T1 glcm differencevariance 8,16 px

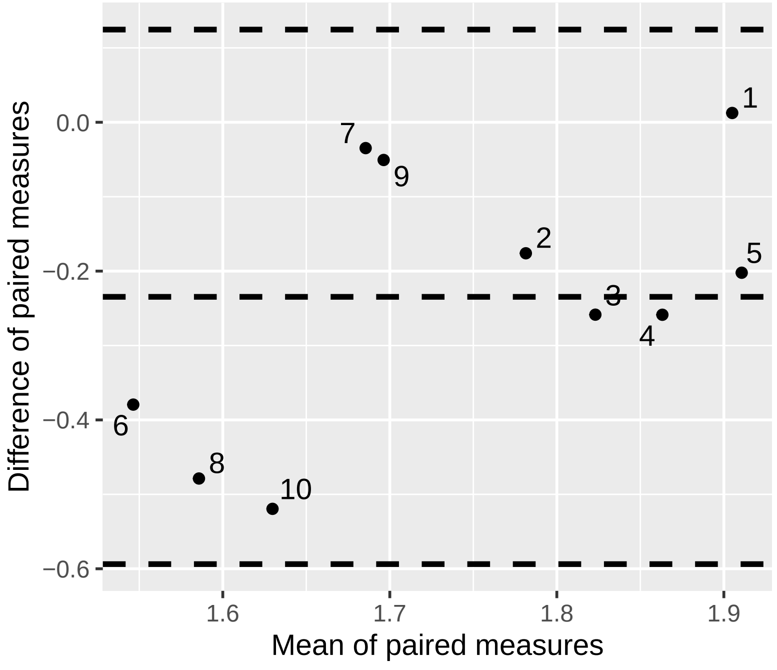

T1 glcm id 8,16 px

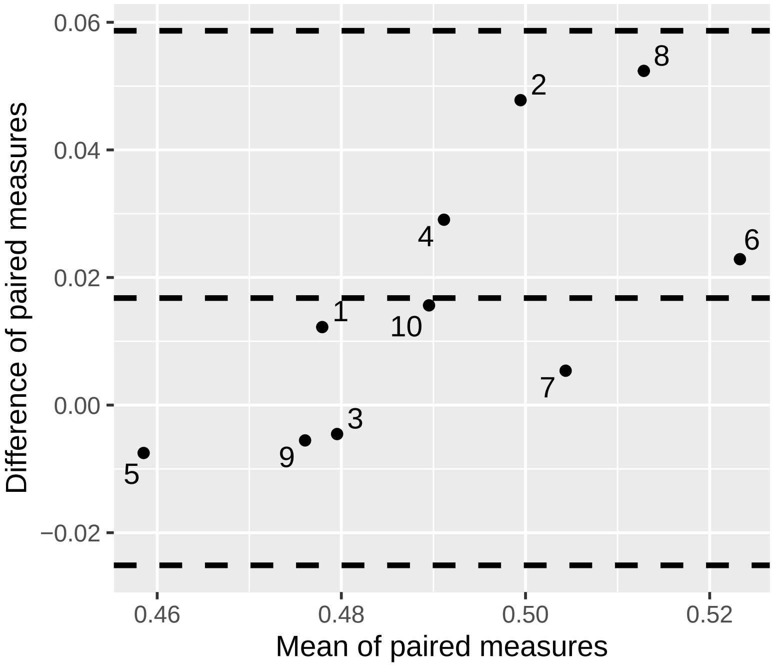


T1 glcm idn 8,16 px

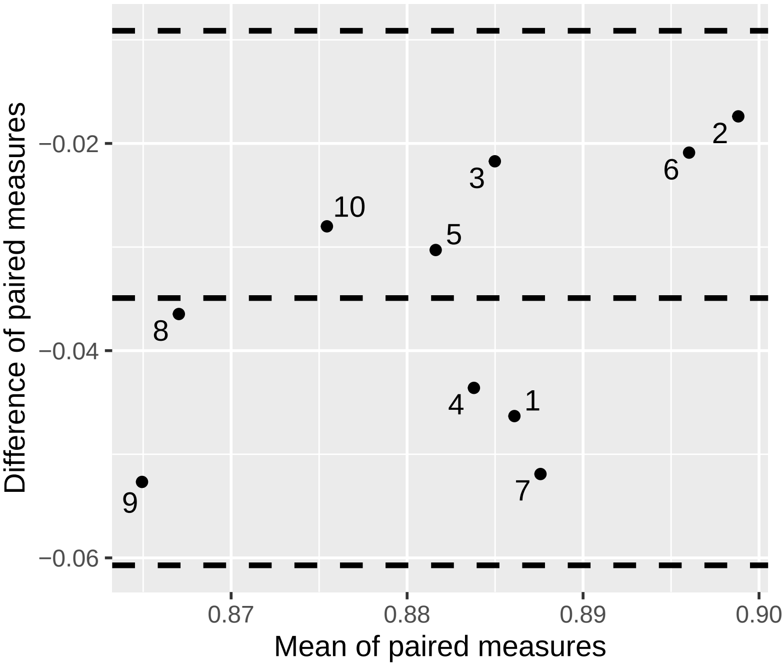


T1 glcm idm 8,16 px

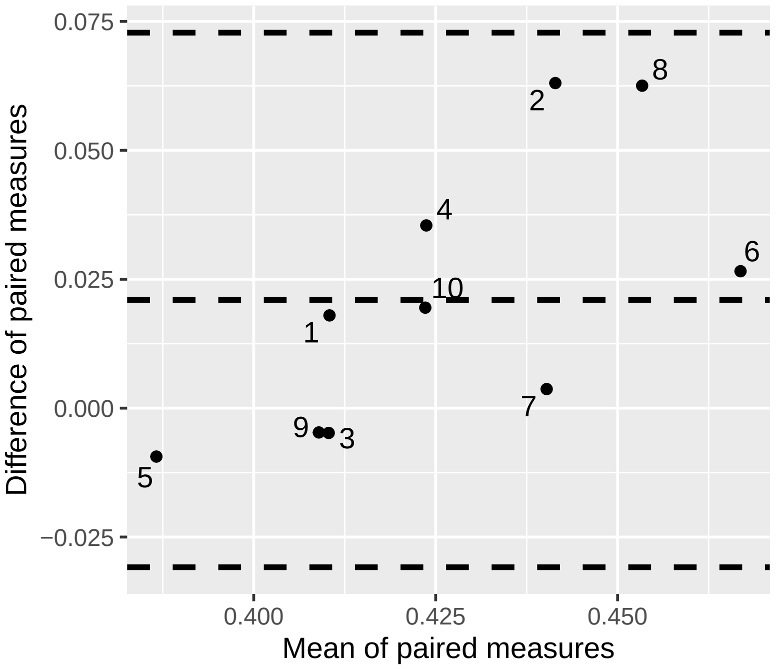


T1 glcm imc1 8,16 px

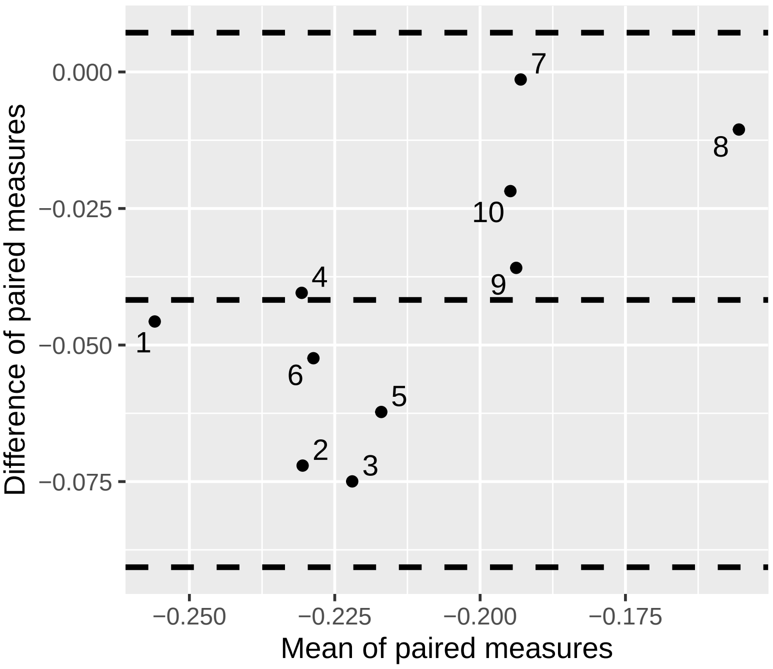


T1 glcm idmn 8,16 px

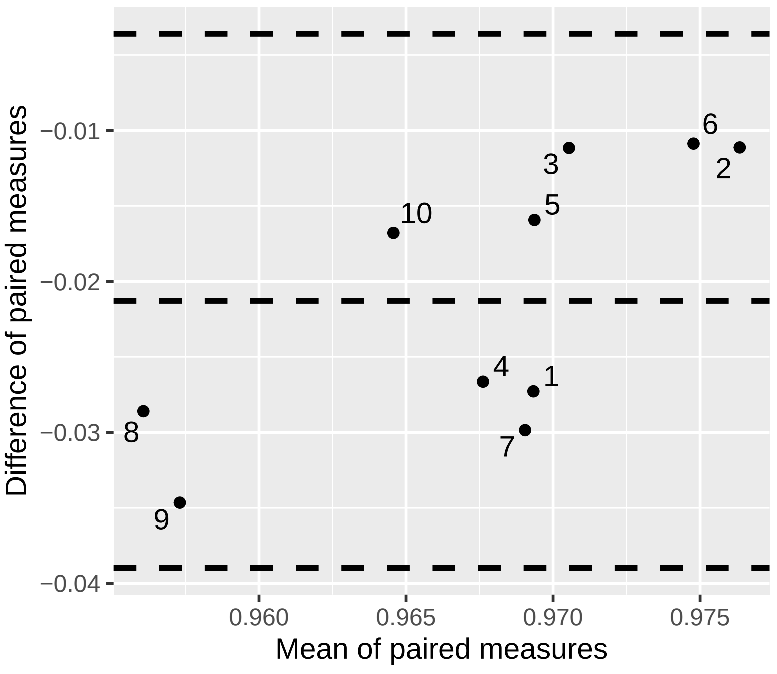


T1 glcm imc2 8,16 px

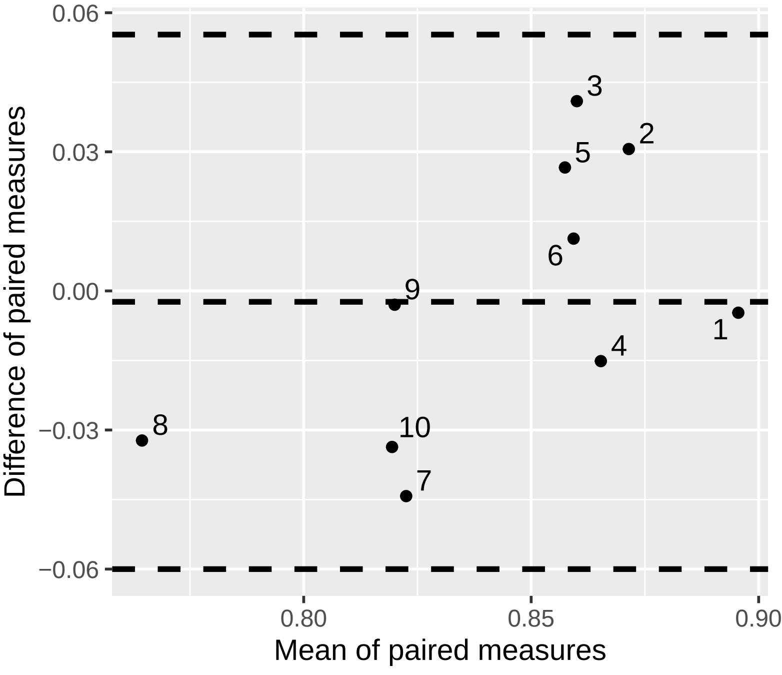

T1 glcm inversevariance 8,16 px

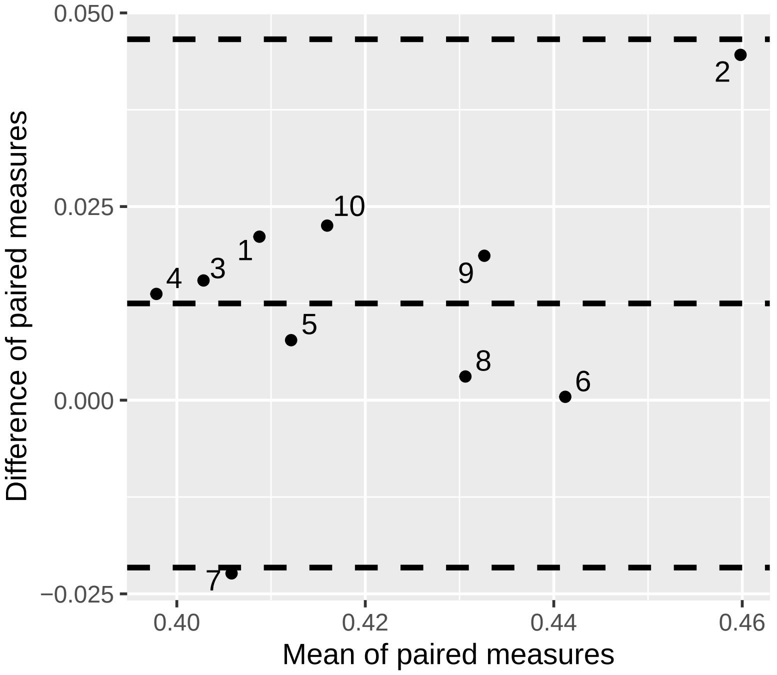


T1 glcm jointentropy 8,16 px

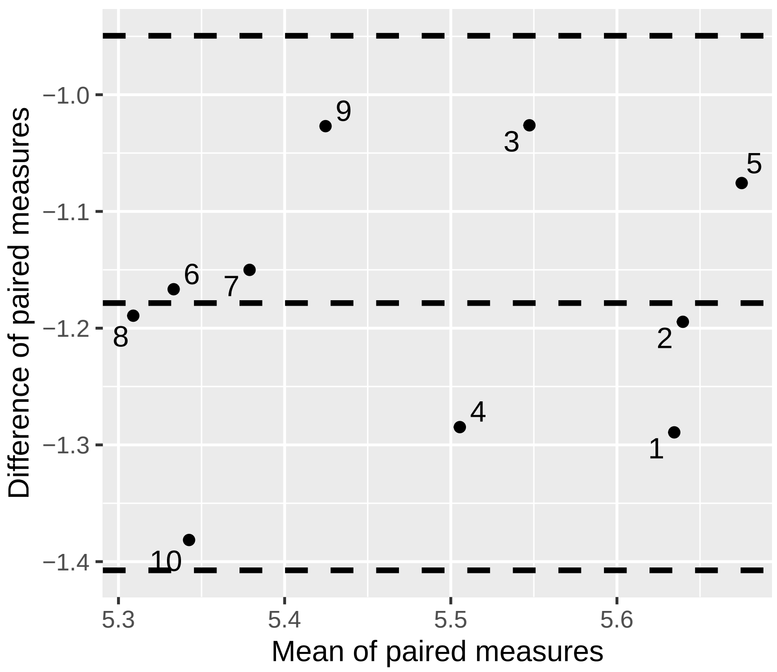


T1 glcm jointaverage 8,16 px

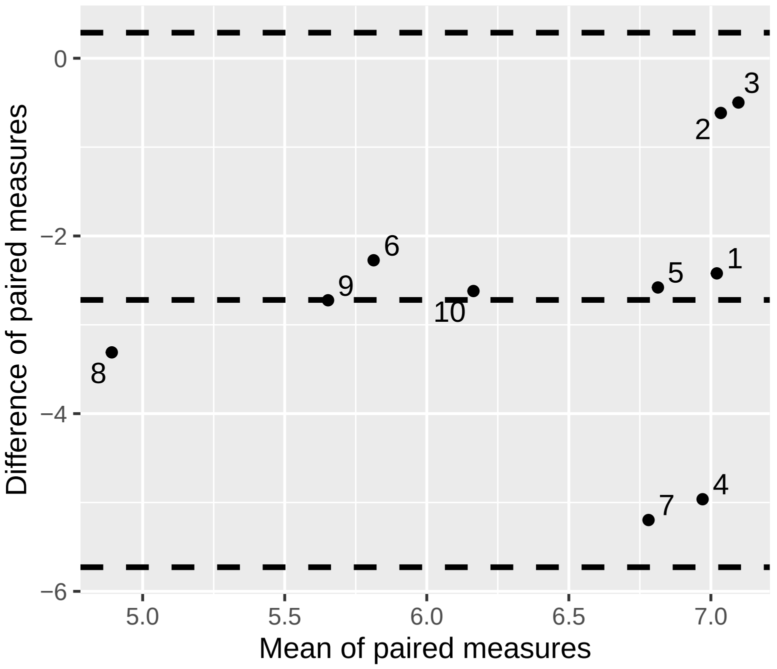


T1 glcm mcc 8,16 px

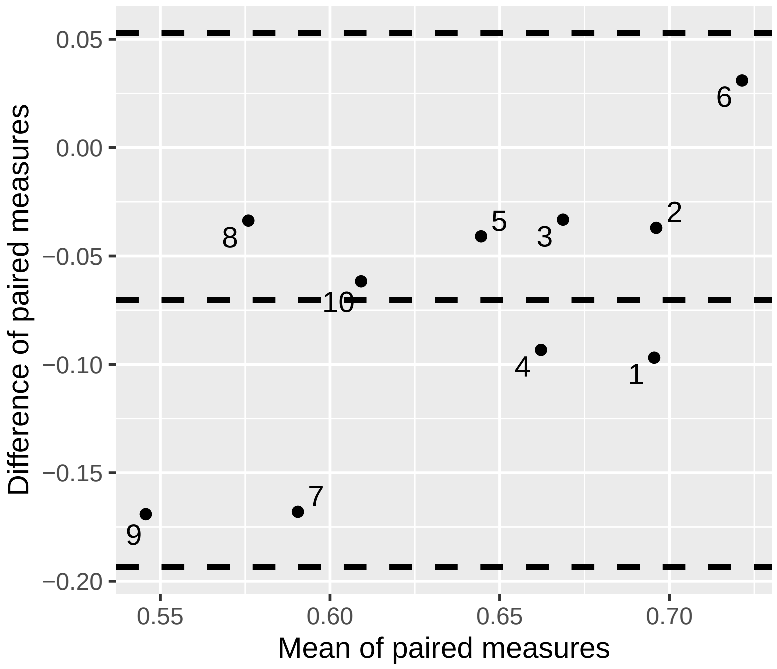


T1 glcm jointenergy 8,16 px

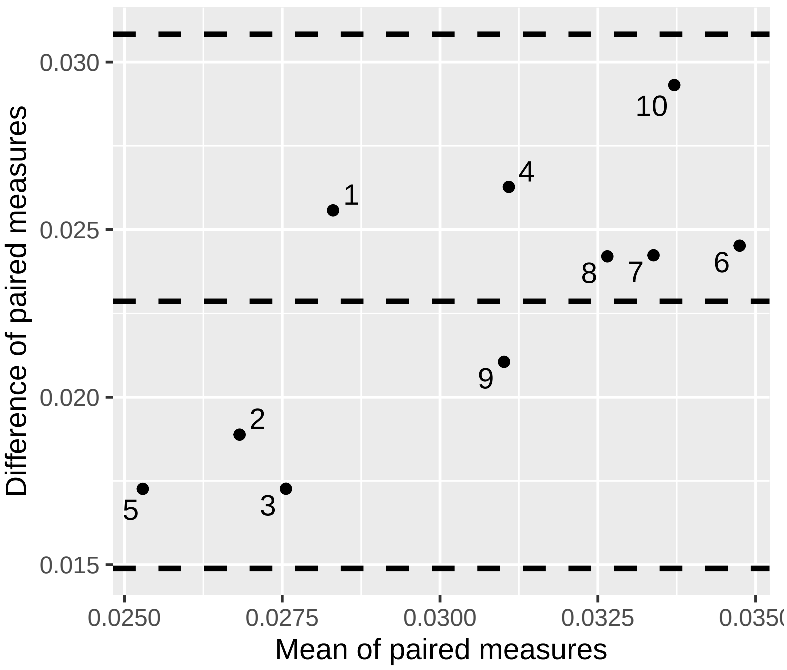


T1 glcm maximumprobability 8,16 px

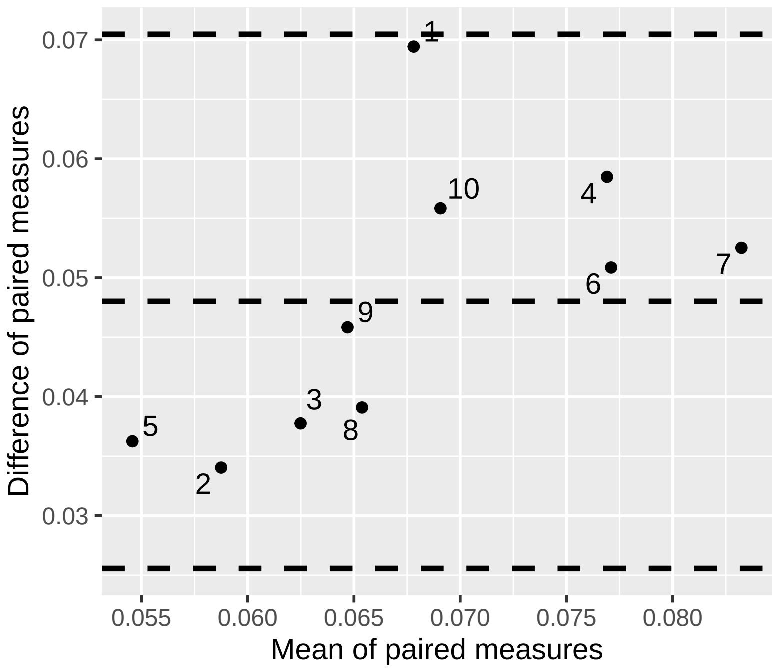

T1 glcm sumaverage 8,16 px

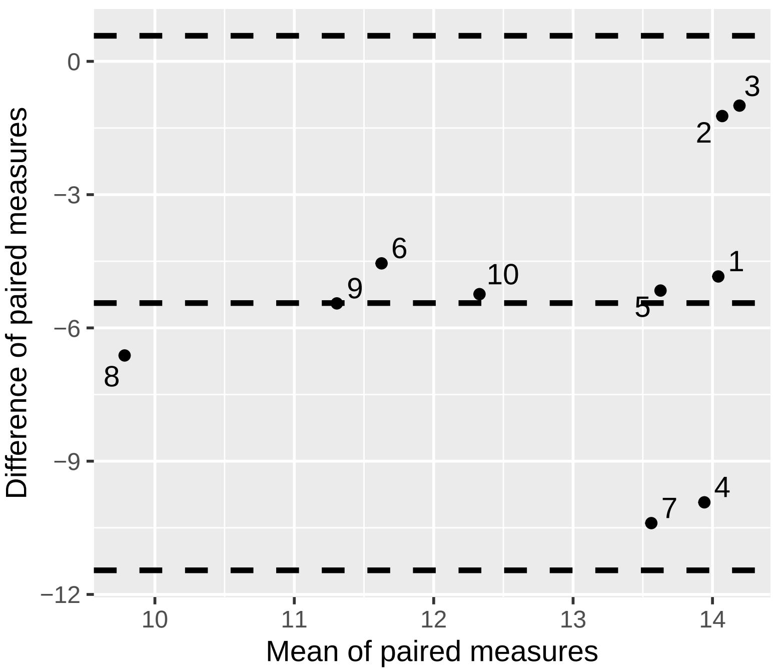


T1 glrlm graylevelnonuniformity 8,16 px

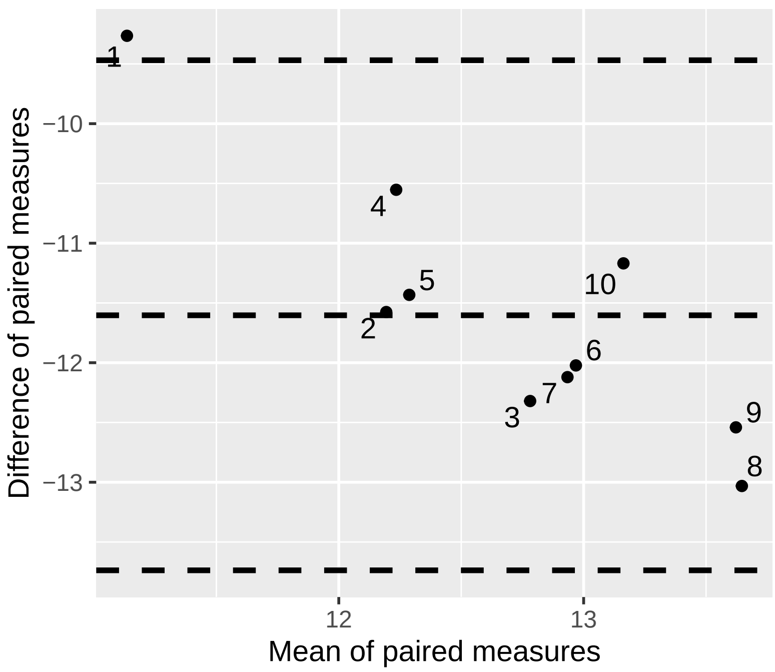


T1 glcm sumentropy 8,16 px

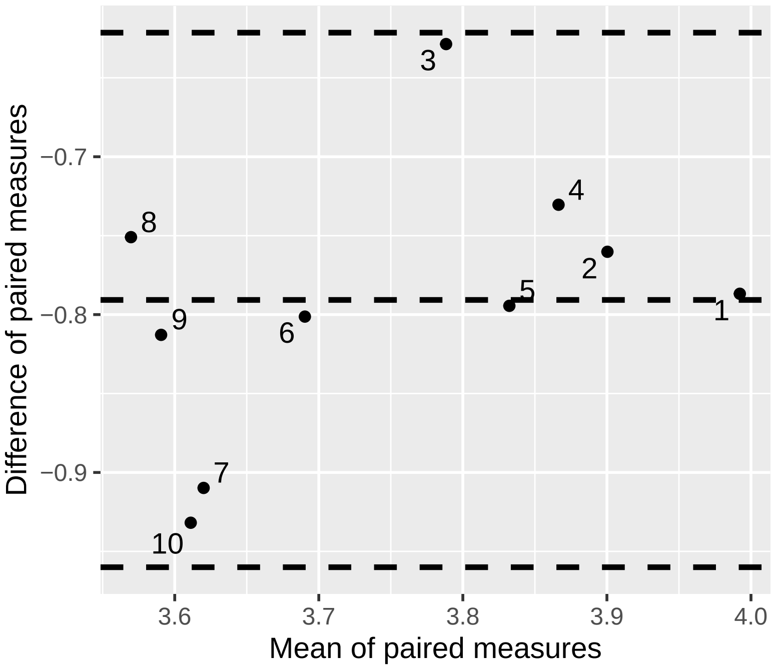


T1 glrlm graylevelnonuniformitynormalized 8,

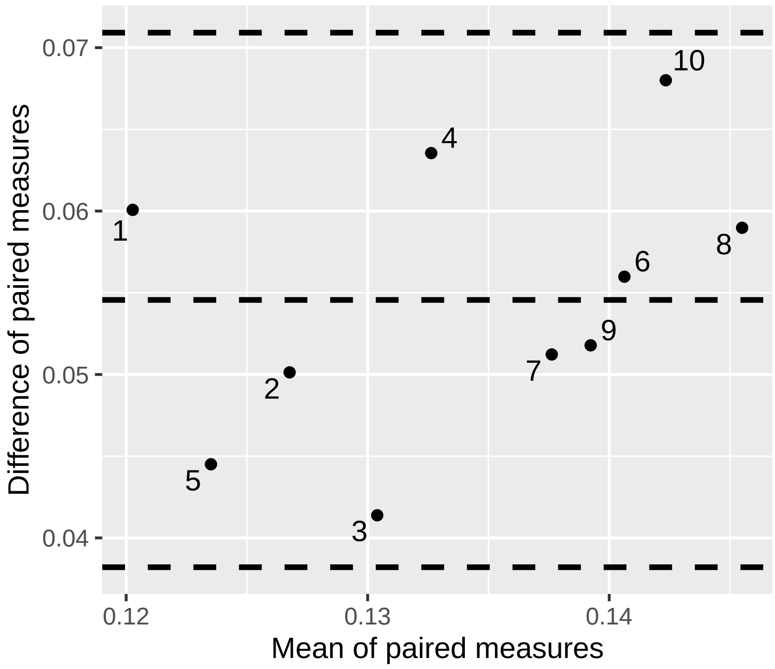


T1 glcm sumsquares 8,16 px

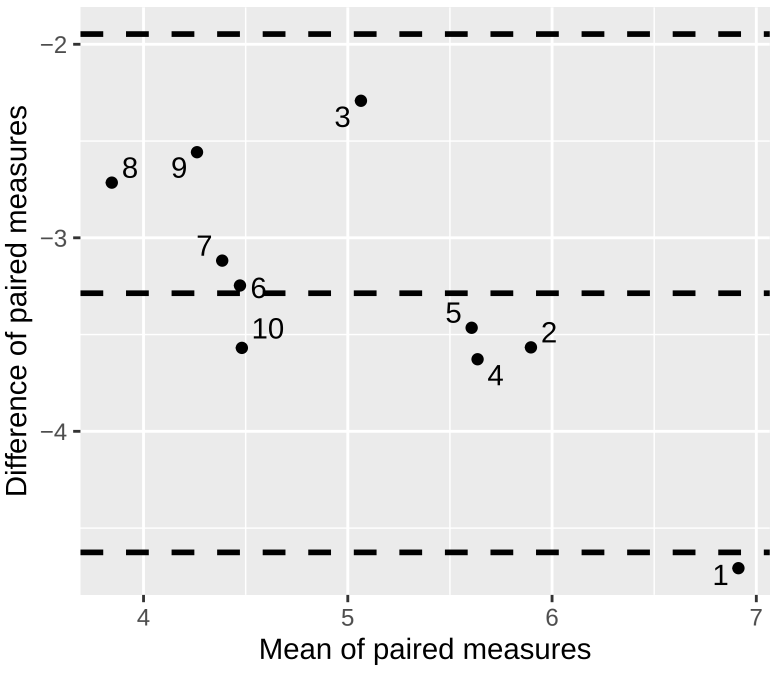


T1 glrlm graylevelvariance 8,16 px

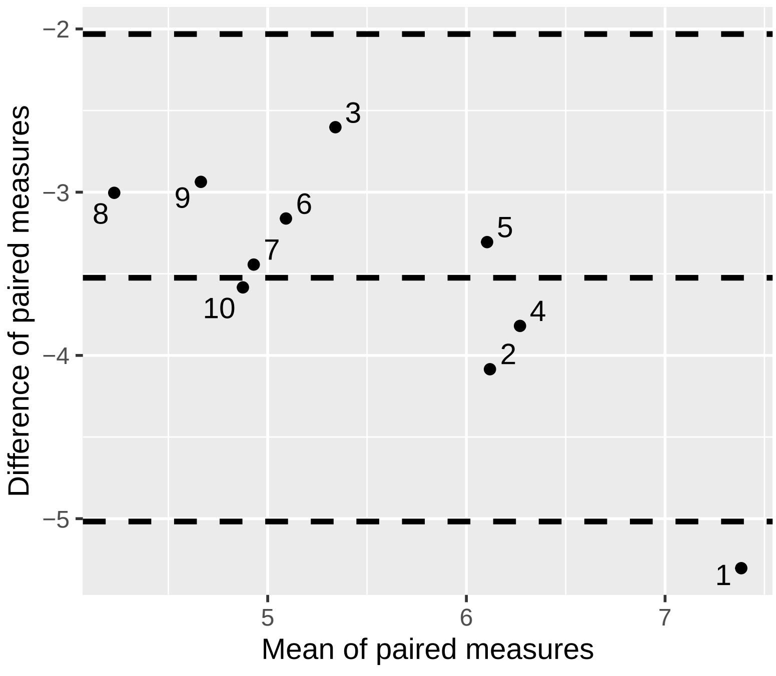

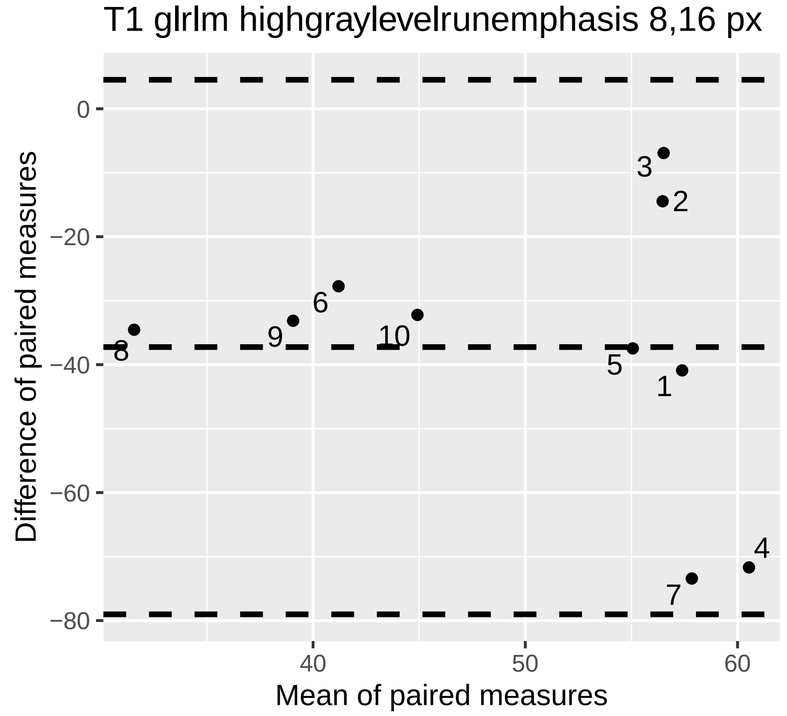
T1 glrlm highgraylevelrunemphasis 8,16 px
Difference of paired measures
Mean of paired measures
0
−20
−40
−60
−80
40
50
60

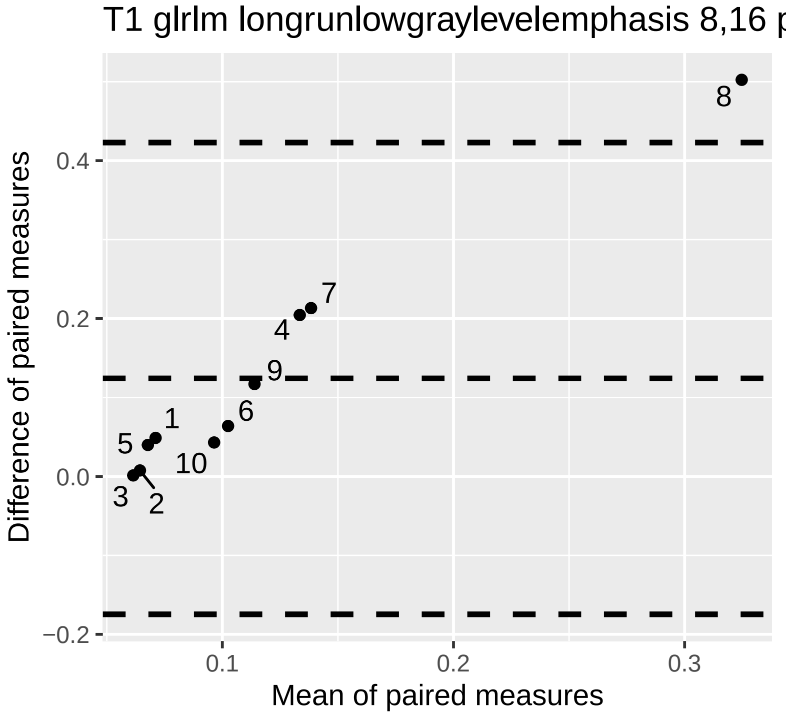
T1 glrlm longrunlowgraylevelemphasis 8,16 p
Difference of paired measures
Mean of paired measures
0.4
0.2
0.0
−0.2
0.1
0.2
0.3

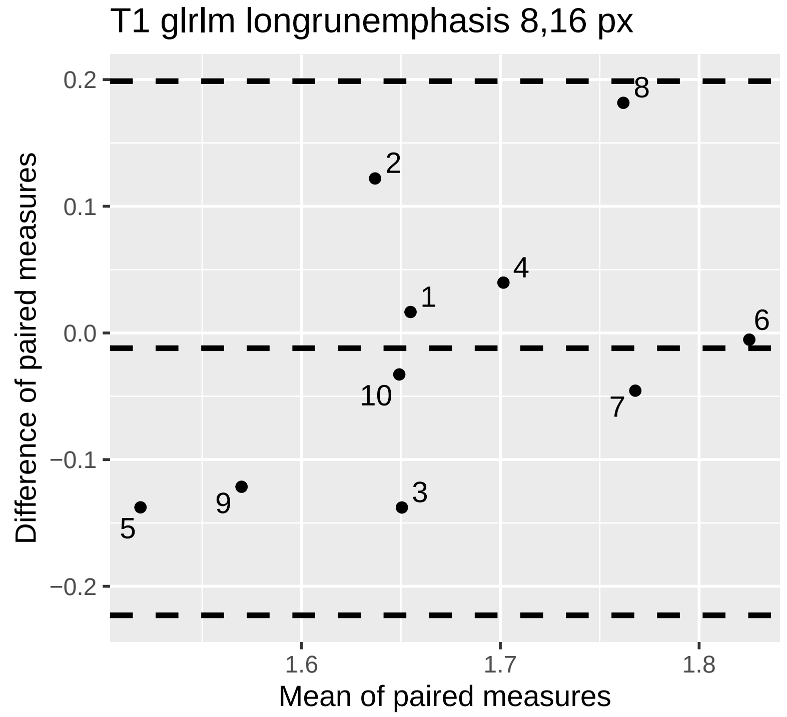
T1 glrlm longrunemphasis 8,16 px
Difference of paired measures
Mean of paired measures
0.2
0.1
0.0
−0.1
−0.2
1.6
1.7
1.8

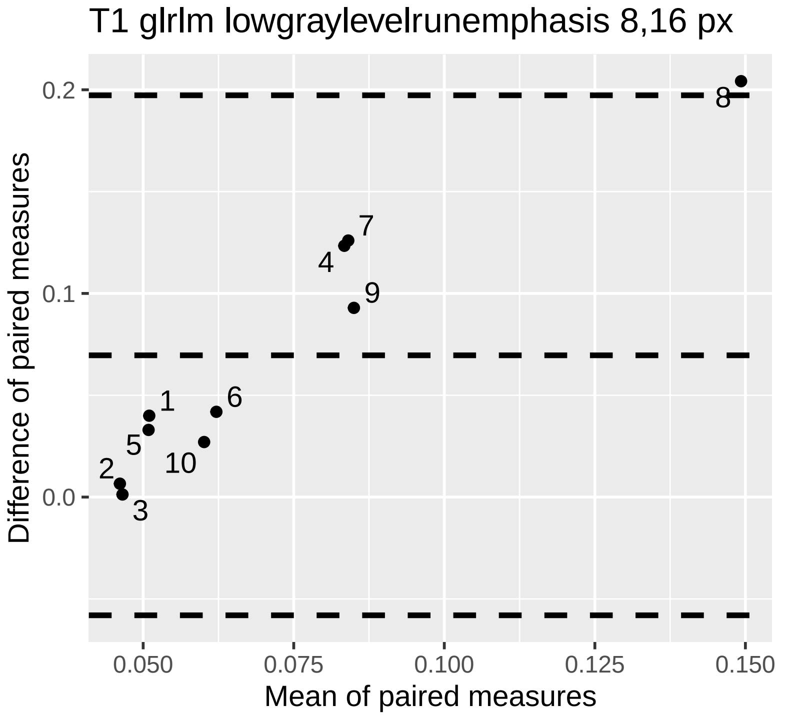
T1 glrlm lowgraylevelrunemphasis 8,16 px
Difference of paired measures
Mean of paired measures
0.2
0.1
0.0
0.050
0.075
0.100
0.125
0.150

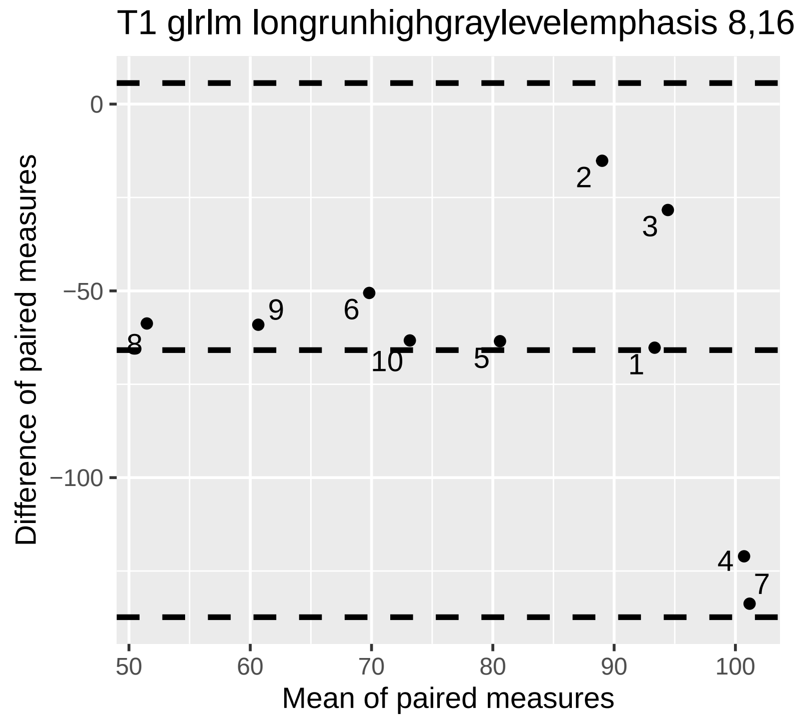
T1 glrlm longrunhighgraylevelemphasis 8,16
Difference of paired measures
Mean of paired measures
0
−50
−100
50
60
70
80
90
100

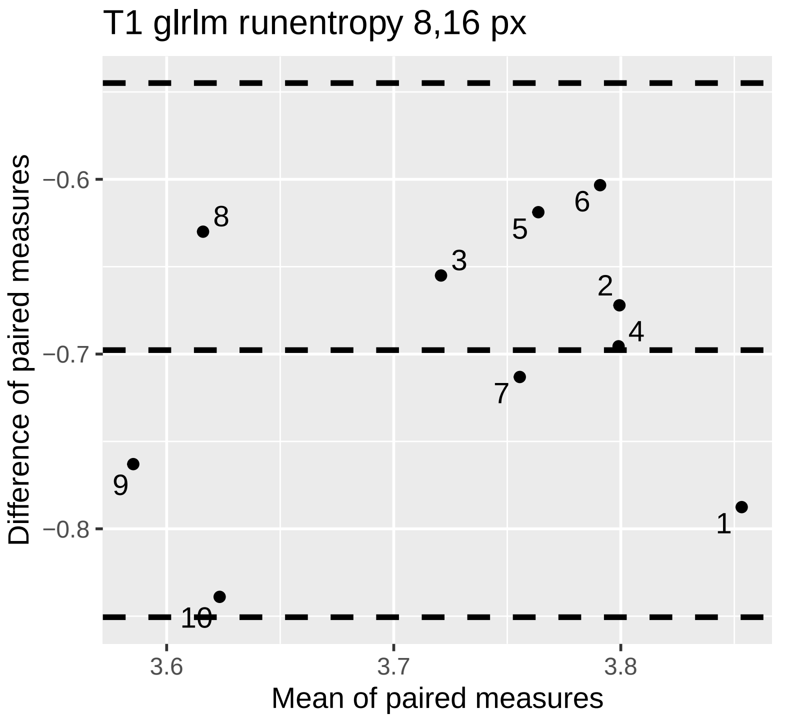
T1 glrlm runentropy 8,16 px
Difference of paired measures
Mean of paired measures
−0.6
−0.7
−0.8
3.6
3.7
3.8

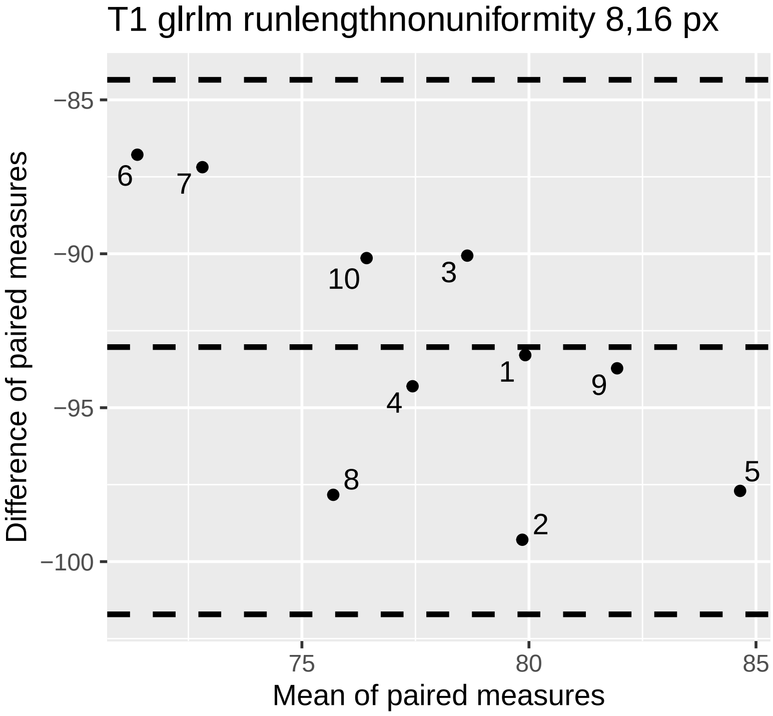
T1 glrlm runlengthnonuniformity 8,16 px
Difference of paired measures
Mean of paired measures

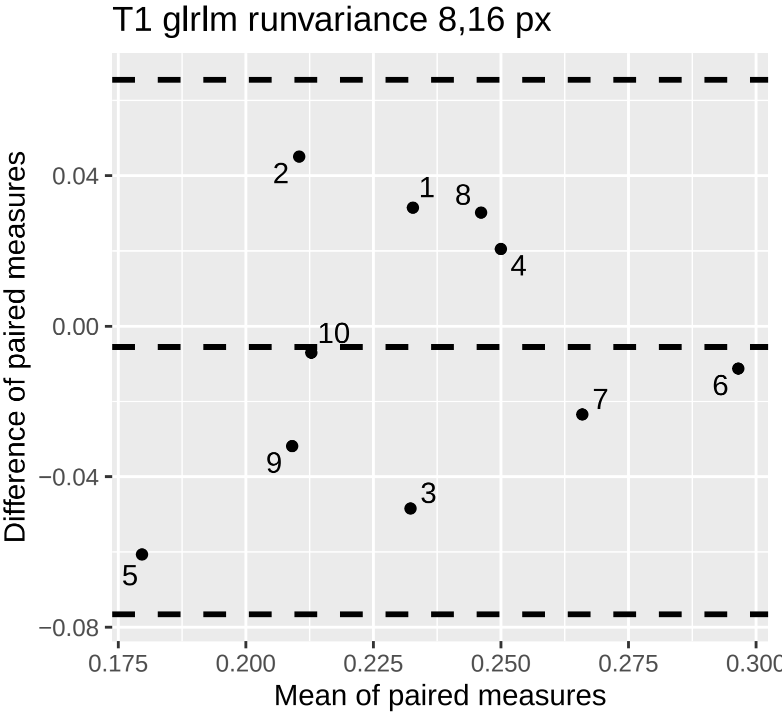
T1 glrlm runvariance 8,16 px
Difference of paired measures
Mean of paired measures

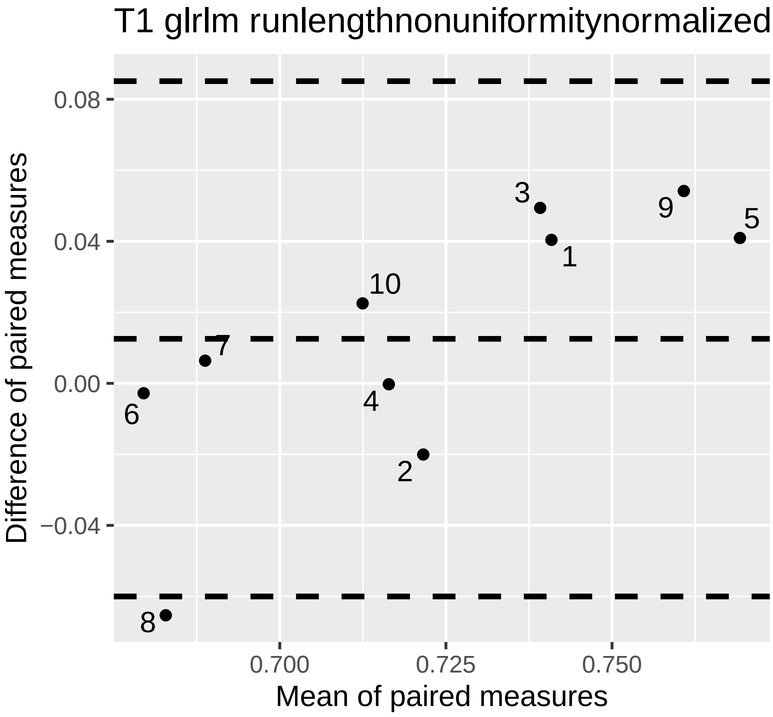
T1 glrlm runlengthnonuniformitynormalized
Difference of paired measures
Mean of paired measures

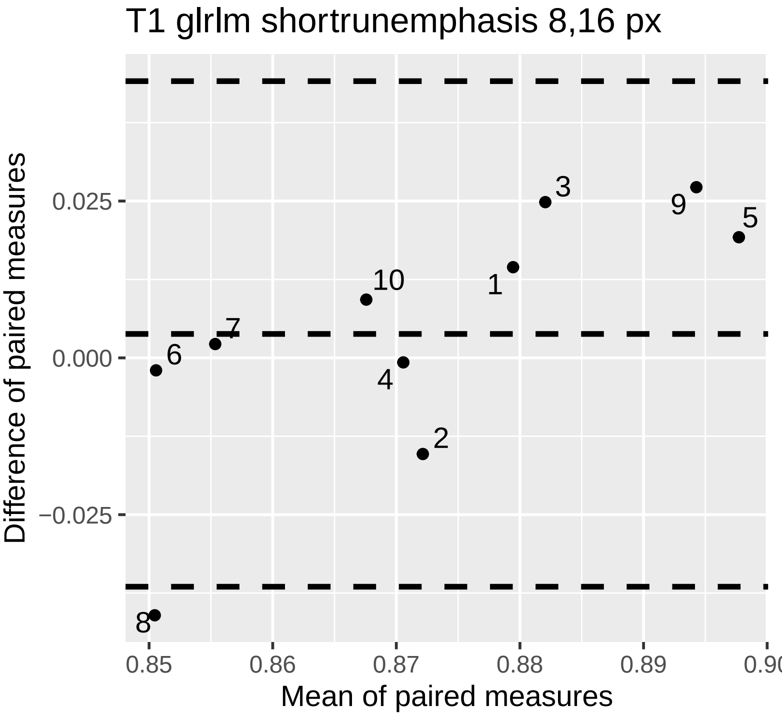
T1 glrlm shortrunemphasis 8,16 px
Difference of paired measures
Mean of paired measures

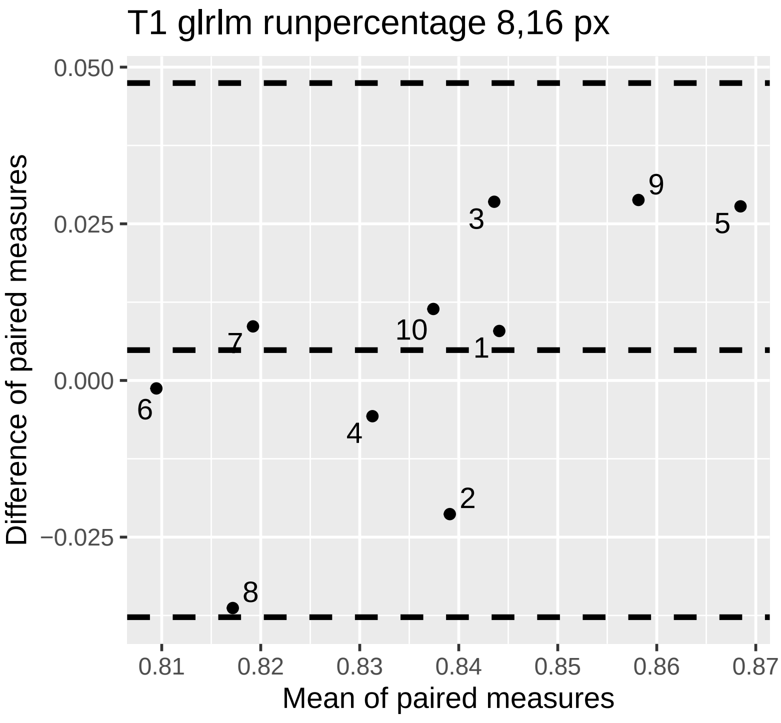
T1 glrlm runpercentage 8,16 px
Difference of paired measures
Mean of paired measures

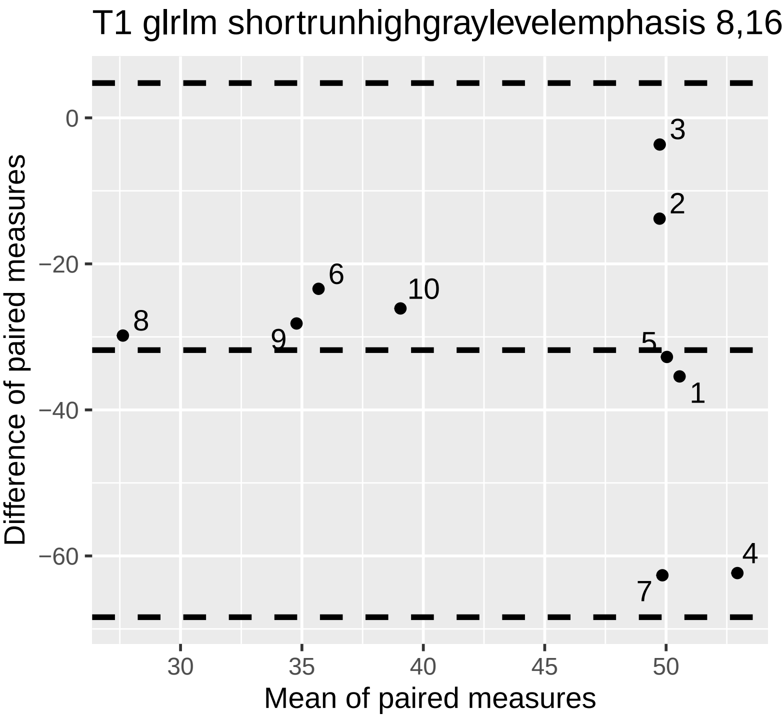
T1 glrlm shortrunhighgraylevelemphasis 8,16
Difference of paired measures
Mean of paired measures

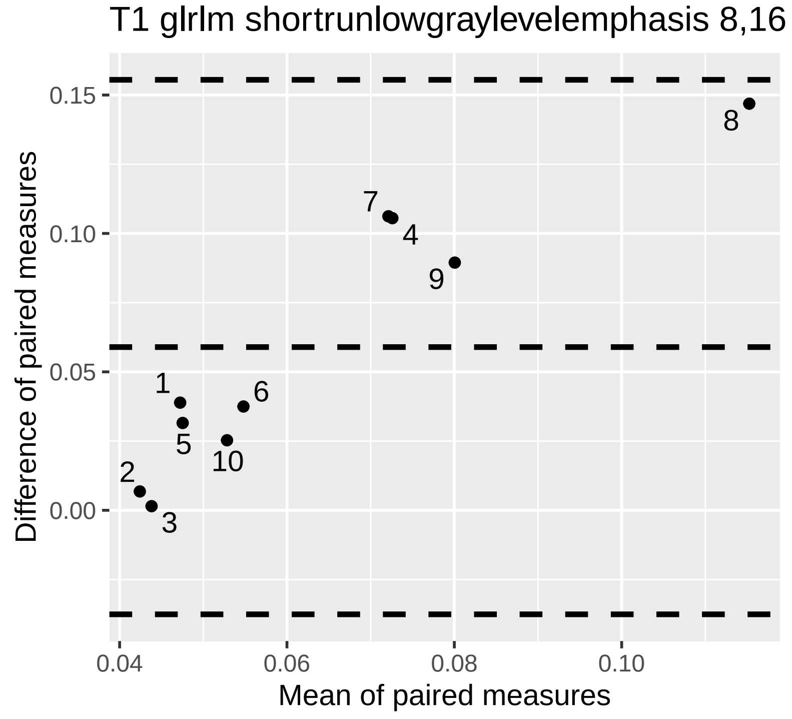
T1 glrlm shortrunlowgraylevelemphasis 8,16
Difference of paired measures
0.15
0.10
0.05
0.00
0.04
0.06
0.08
0.10
Mean of paired measures
1
2
3
4
5
6
7
8
9
10

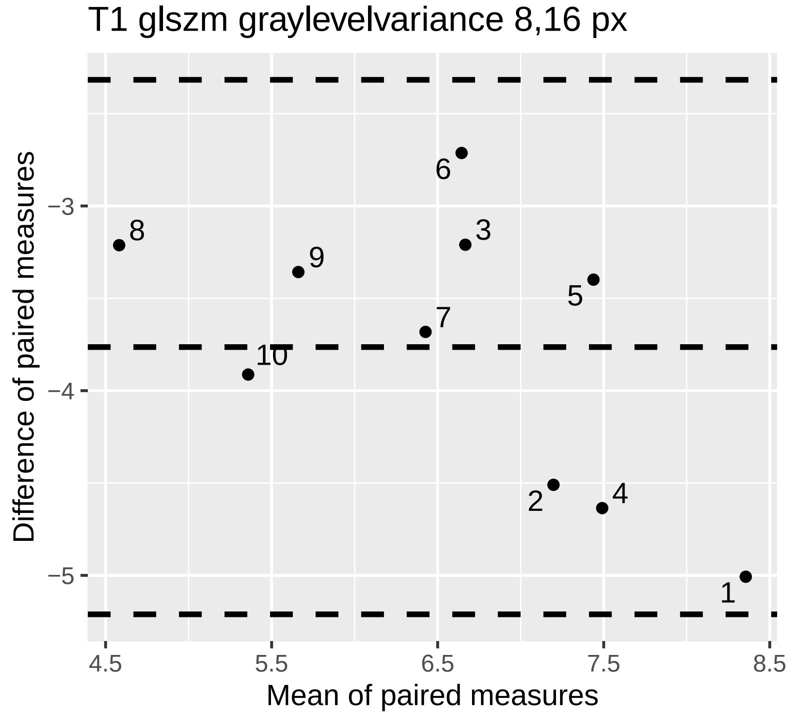
T1 glszm graylevelvariance 8,16 px
Difference of paired measures
−3
−4
−5
4.5
5.5
6.5
7.5
8.5
Mean of paired measures
1
2
3
4
5
6
7
8
9
10

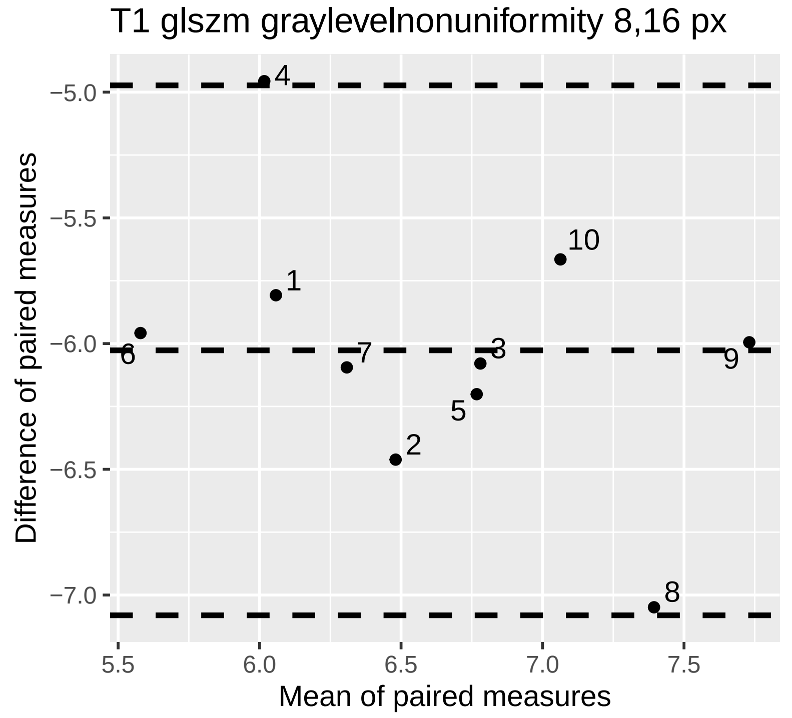
T1 glszm graylevelnonuniformity 8,16 px
Difference of paired measures
−5.0
−5.5
−6.0
−6.5
−7.0
5.5
6.0
6.5
7.0
7.5
Mean of paired measures
1
2
3
4
5
6
7
8
9
10

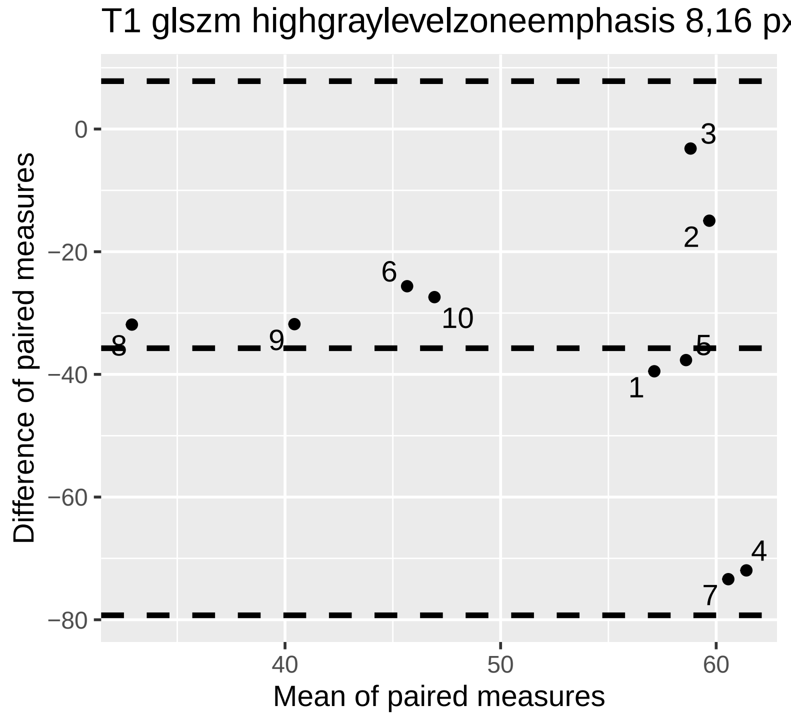
T1 glszm highgraylevelzoneemphasis 8,16 px
Difference of paired measures
0
−20
−40
−60
−80
40
50
60
Mean of paired measures
1
2
3
4
5
6
7
8
9
10

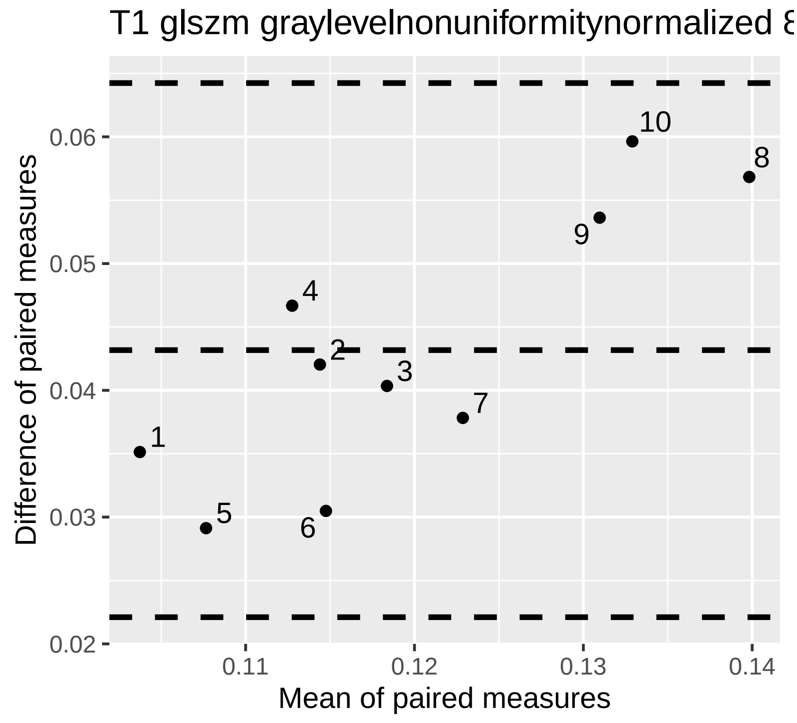
T1 glszm graylevelnonuniformitynormalized 8
Difference of paired measures
0.06
0.05
0.04
0.03
0.02
0.11
0.12
0.13
0.14
Mean of paired measures
1
2
3
4
5
6
7
8
9
10

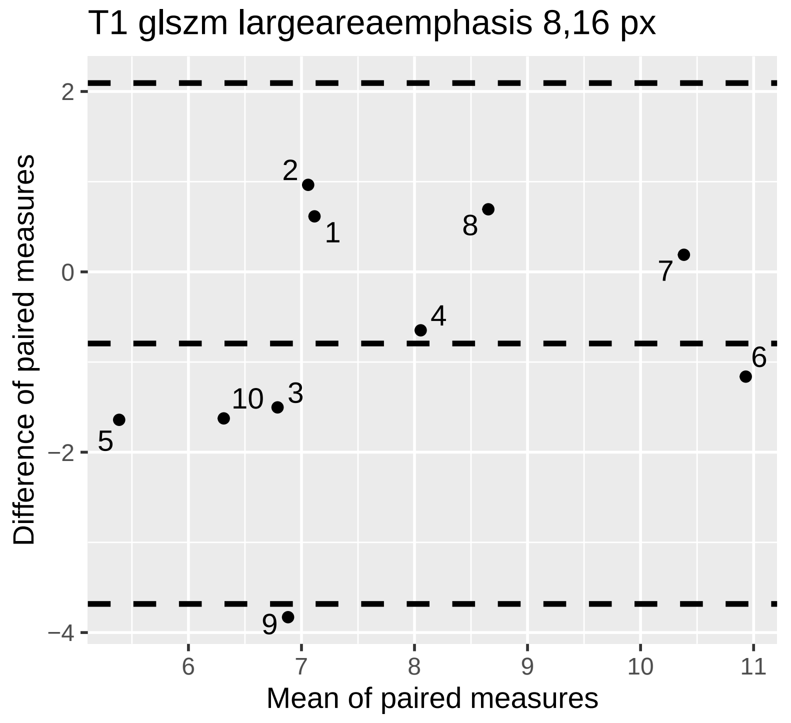
T1 glszm largeareaemphasis 8,16 px
Difference of paired measures
2
0
−2
−4
6
7
8
9
10
11
Mean of paired measures
1
2
3
4
5
6
7
8
9
10

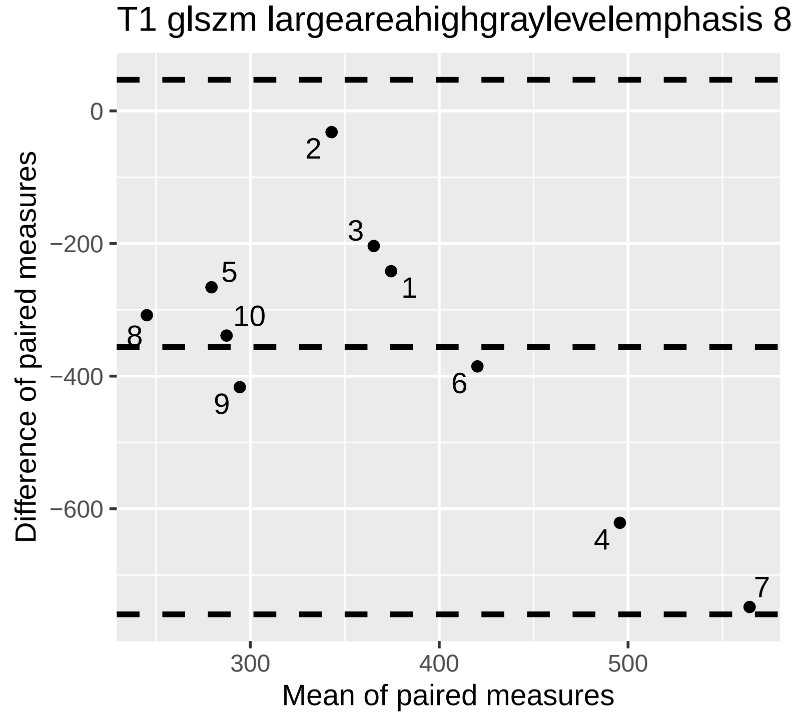
T1 glszm largeareahighgraylevelemphasis 8
Difference of paired measures
Mean of paired measures
0
−200
−400
−600
300
400
500

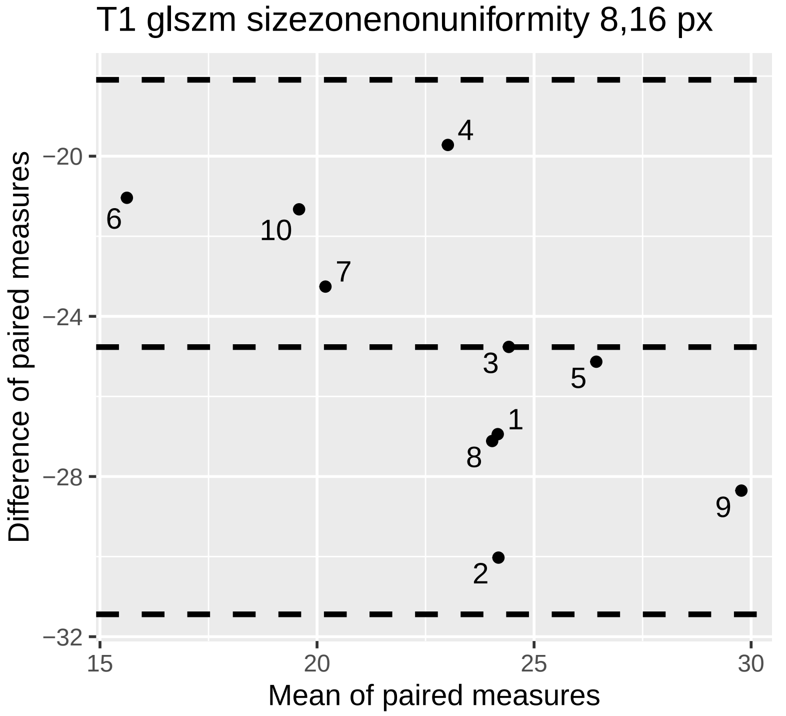
T1 glszm sizezonenonuniformity 8,16 px
Difference of paired measures
Mean of paired measures
−20
−24
−28
−32
15
20
25
30

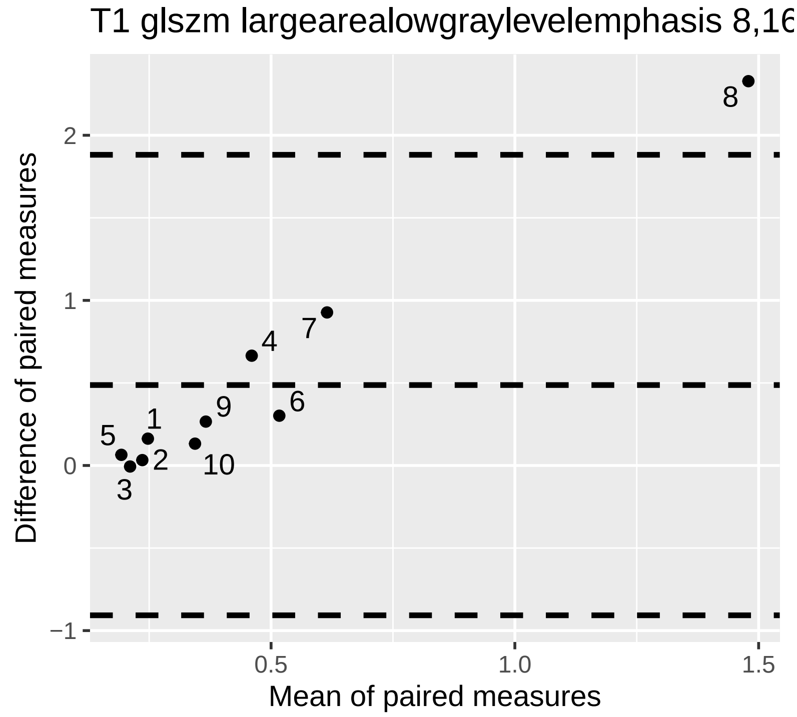
T1 glszm largearealowgraylevelemphasis 8,16
Difference of paired measures
Mean of paired measures
2
1
0
−1
0.5
1.0
1.5

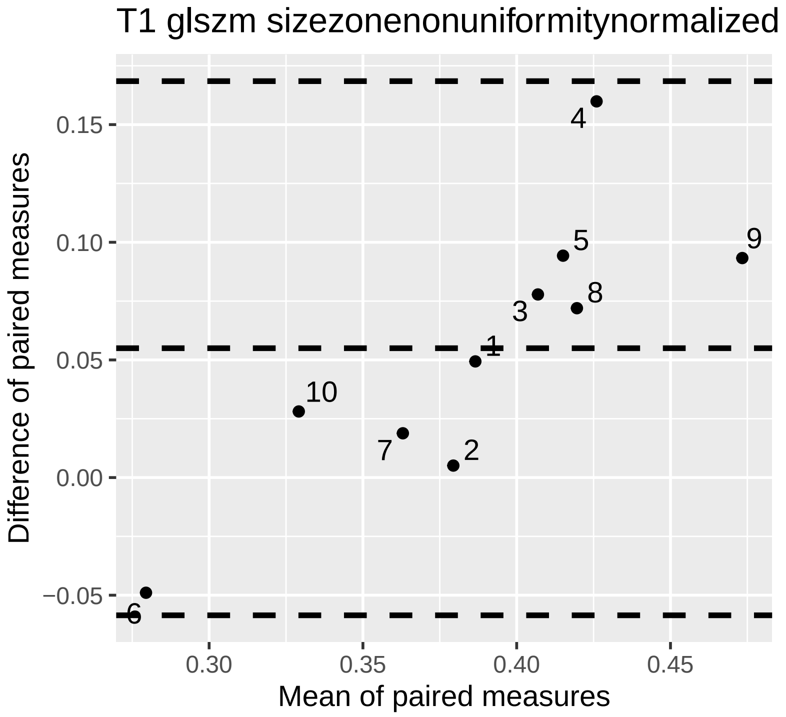
T1 glszm sizezonenonuniformitynormalized
Difference of paired measures
Mean of paired measures
0.15
0.10
0.05
0.00
−0.05
0.30
0.35
0.40
0.45

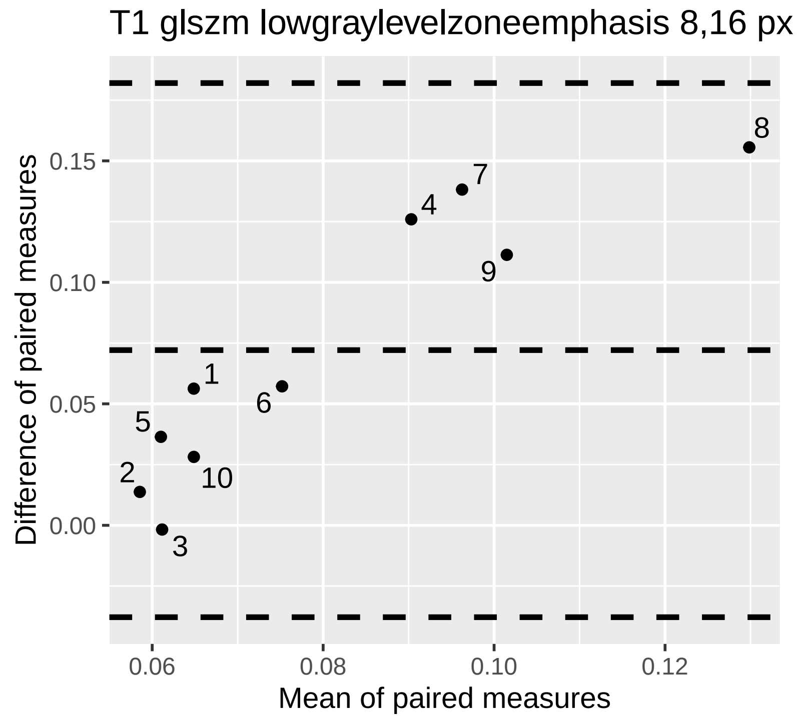
T1 glszm lowgraylevelzoneemphasis 8,16 px
Difference of paired measures
Mean of paired measures
0.15
0.10
0.05
0.00
0.06
0.08
0.10
0.12

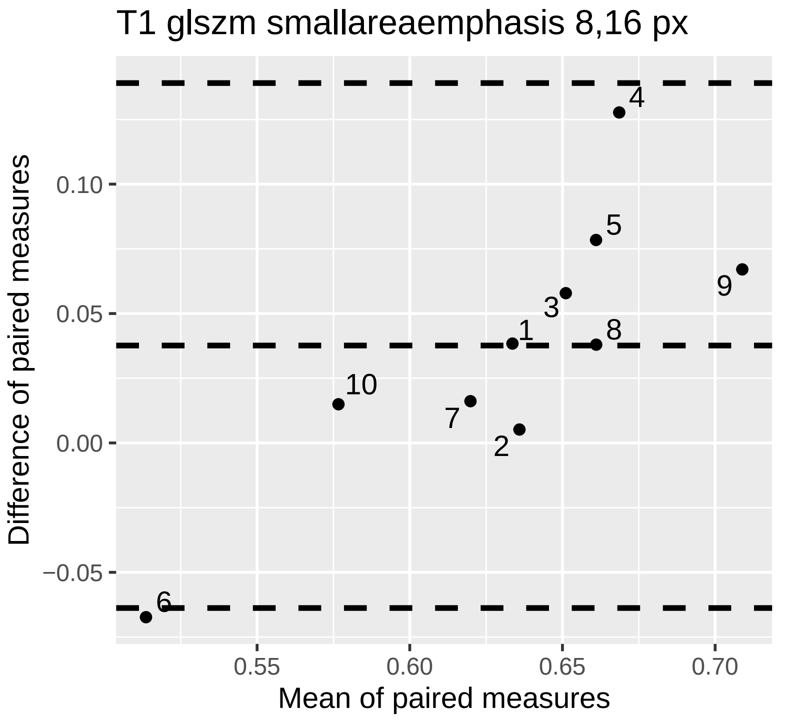
T1 glszm smallareaemphasis 8,16 px
Difference of paired measures
Mean of paired measures
0.10
0.05
0.00
−0.05
0.55
0.60
0.65
0.70

T1 glszm smallareahighgraylevelemphasis 8,

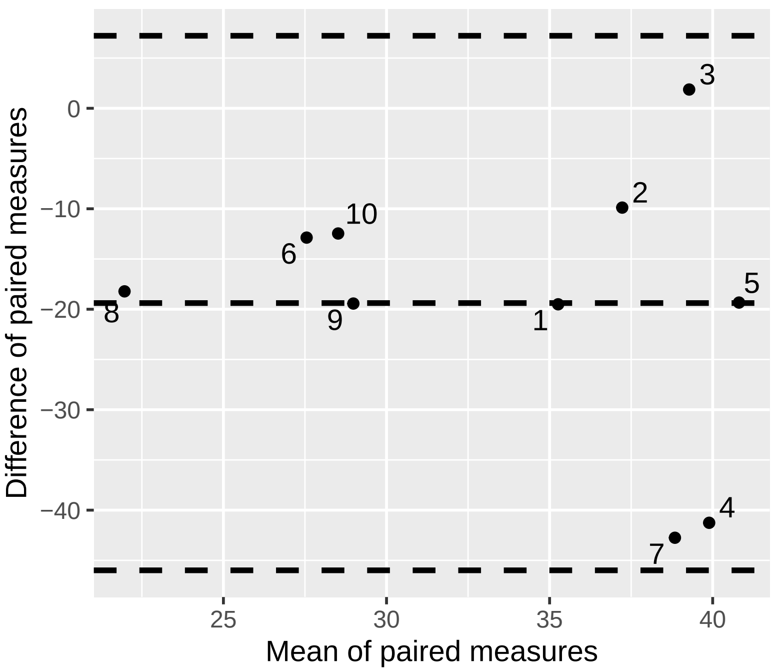


T1 glszm zonepercentage 8,16 px

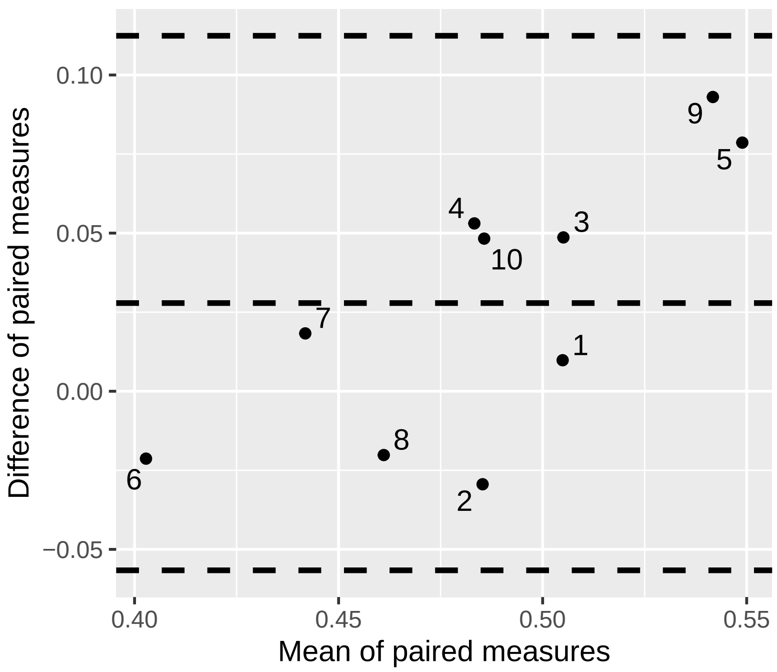


T1 glszm smallarealowgraylevelemphasis 8,1

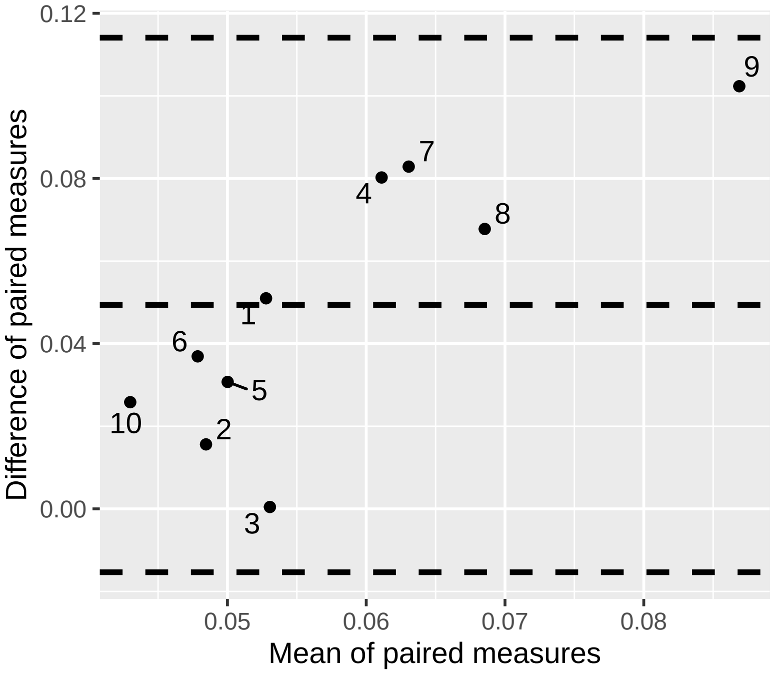


T1 glszm zonevariance 8,16 px

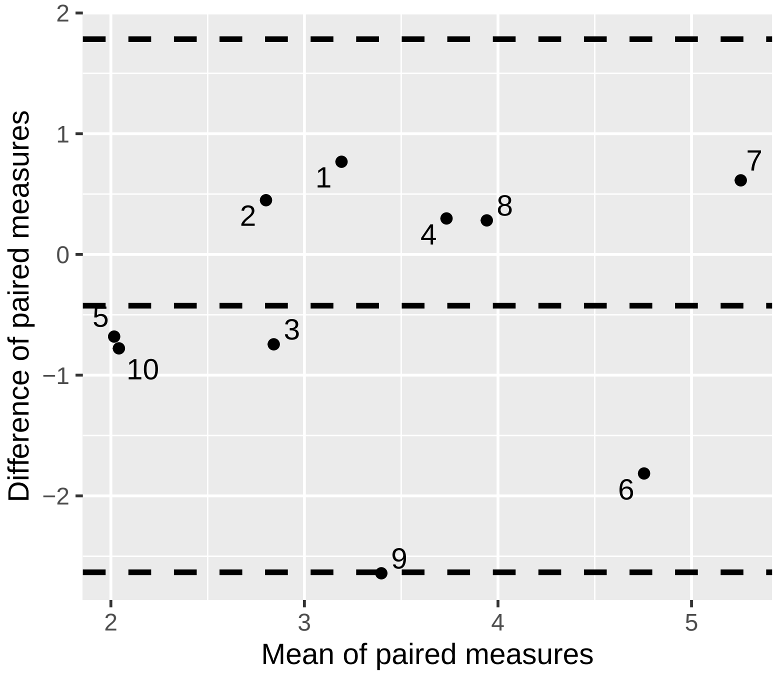


T1 glszm zoneentropy 8,16 px

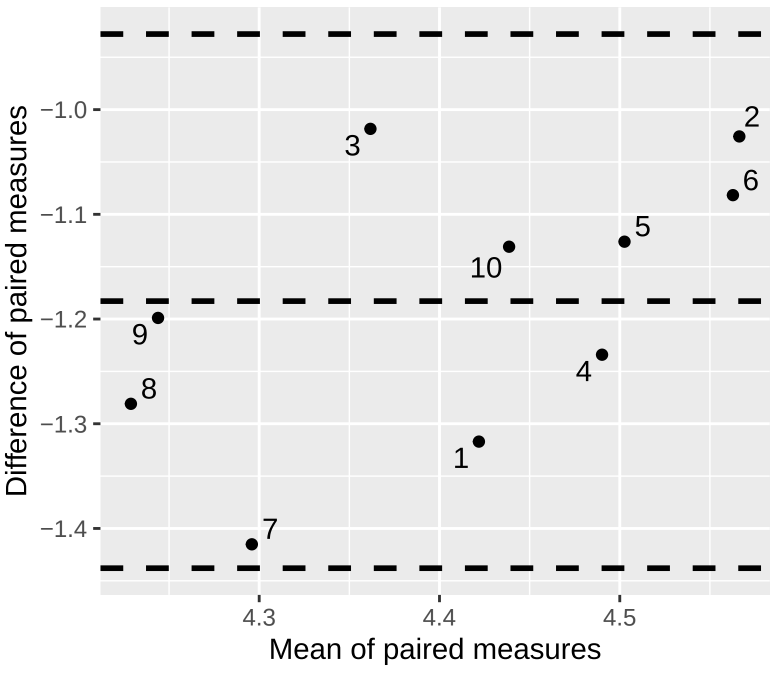


T1 gldm dependenceentropy 8,16 px

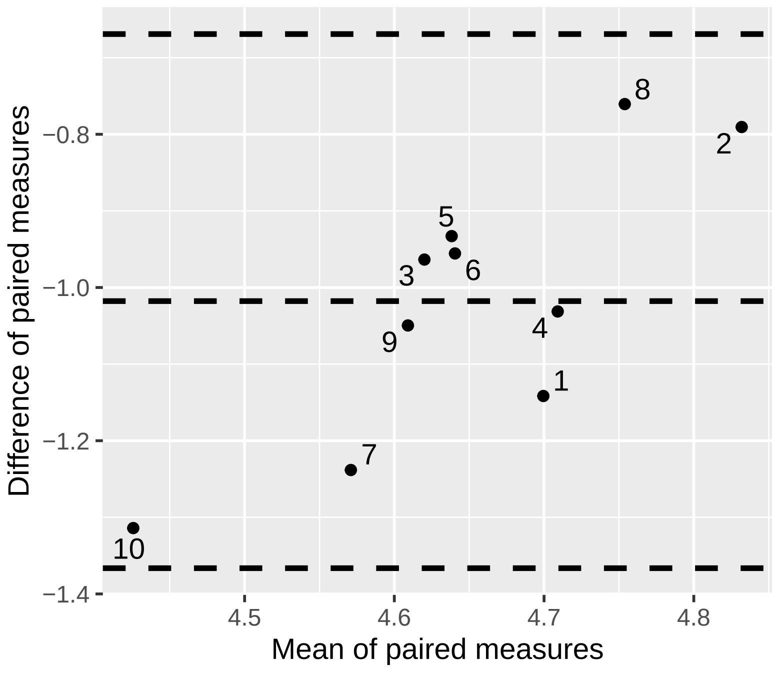

T1 gldm dependencenonuniformity 8,16 px

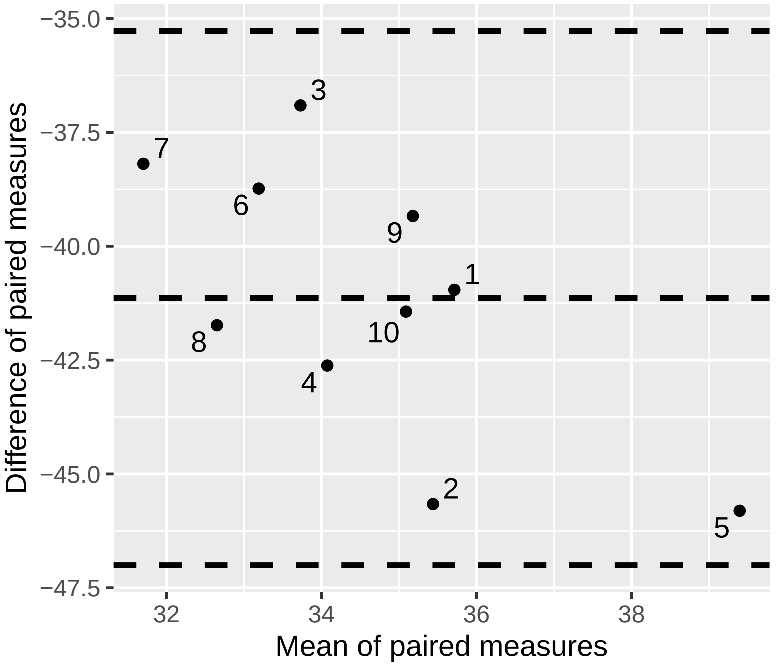


T1 gldm graylevelnonuniformity 8,16 px

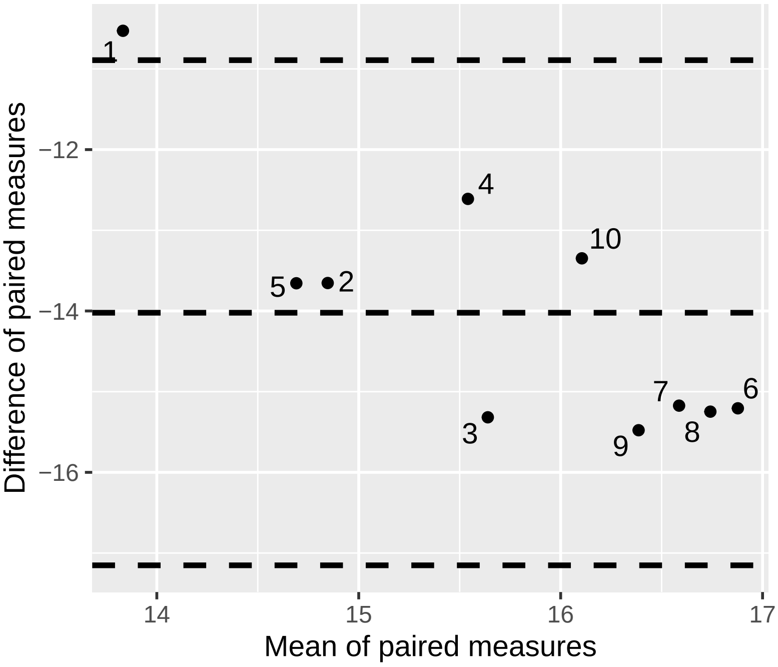


T1 gldm dependencenonuniformitynormaliz

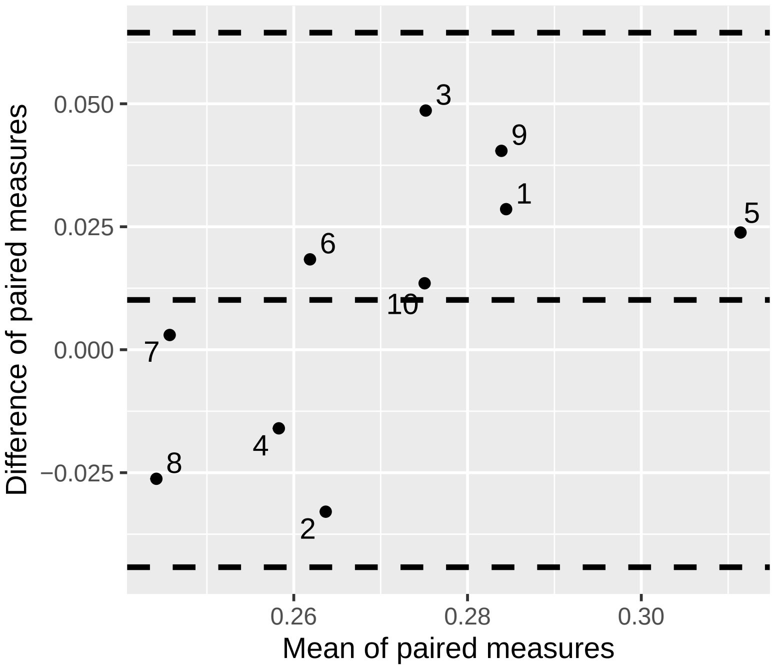


T1 gldm graylevelvariance 8,16 px

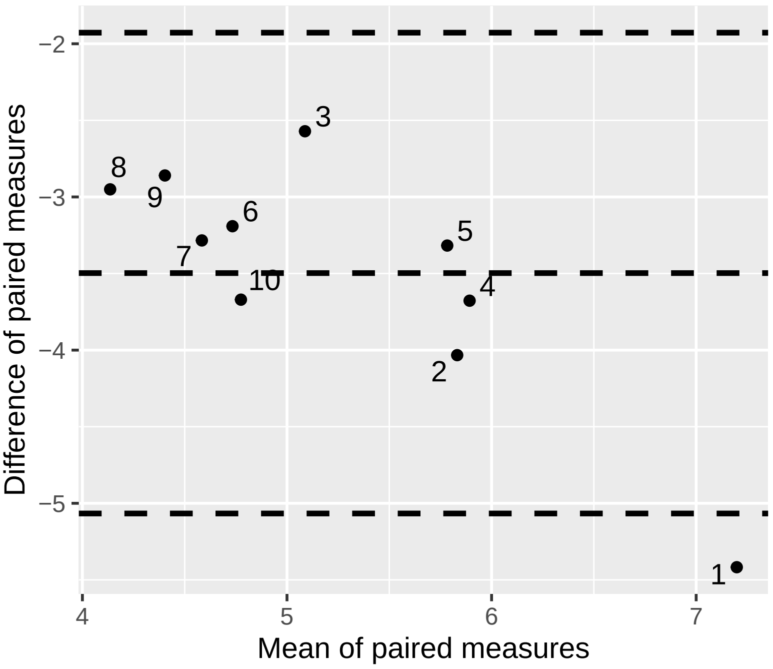


T1 gldm dependencevariance 8,16 px

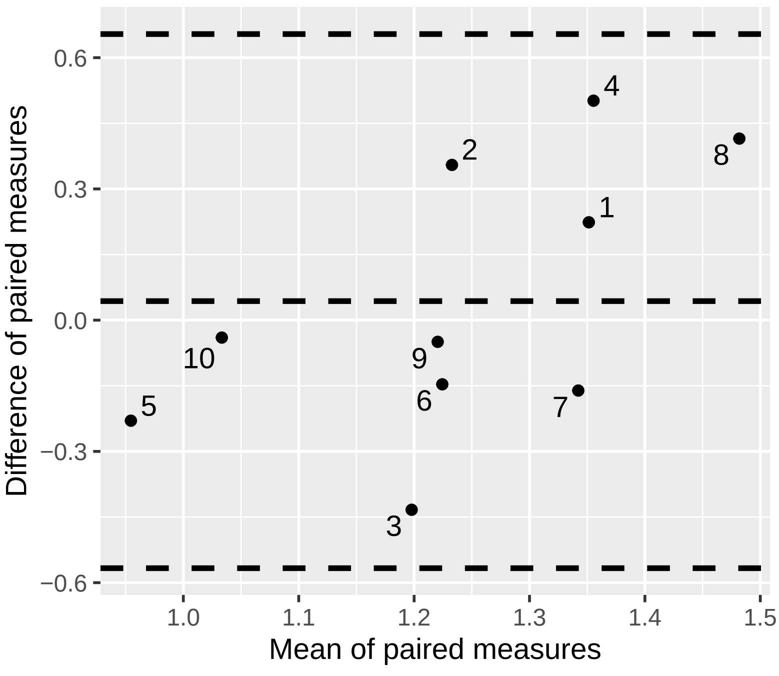


T1 gldm highgraylevelemphasis 8,16 px

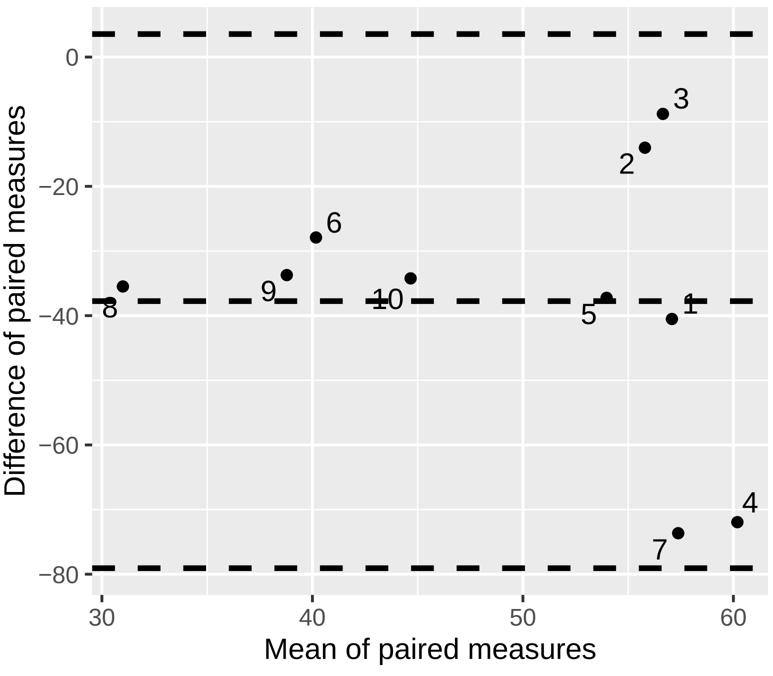

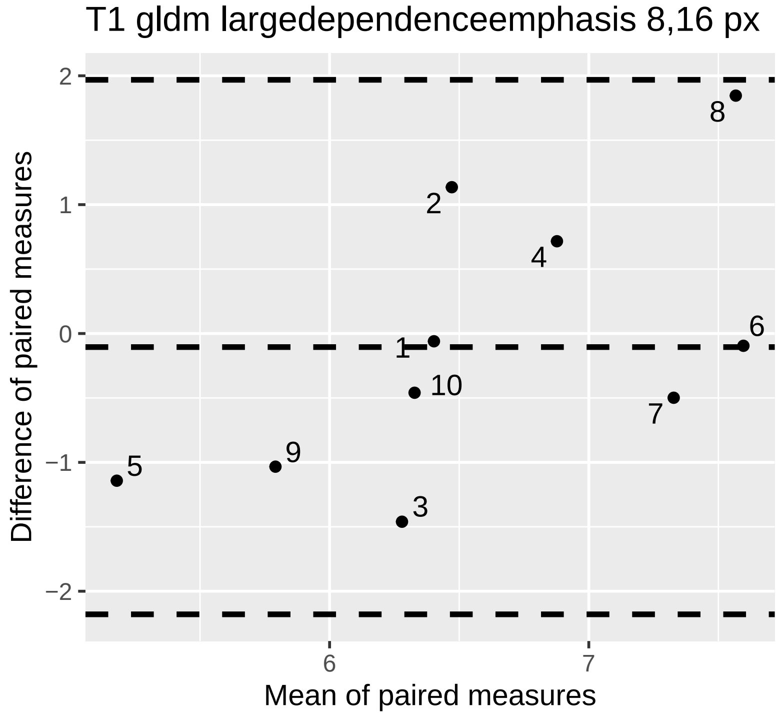
T1 gldm largedependenceemphasis 8,16 px
Difference of paired measures
2
1
0
−1
−2
6
7
Mean of paired measures

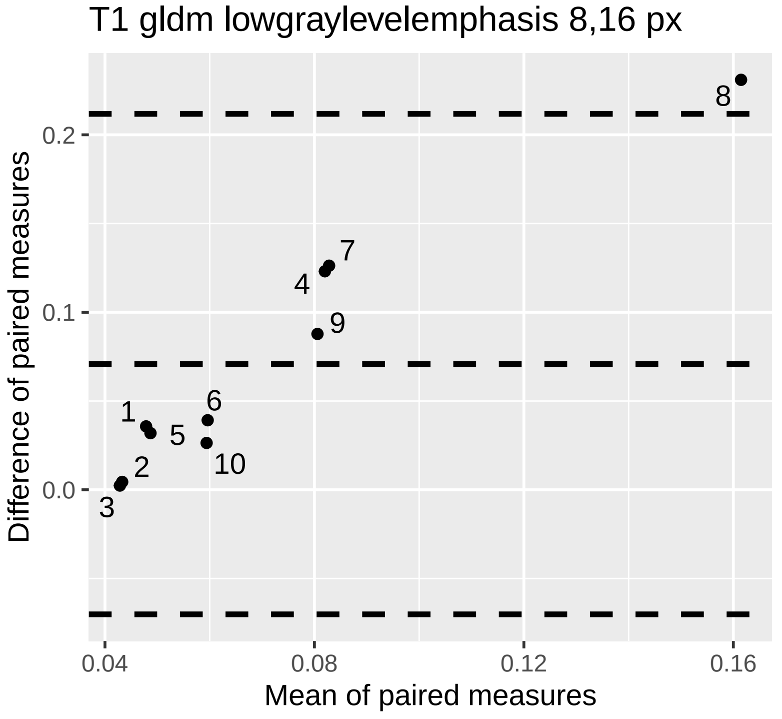
T1 gldm lowgraylevelemphasis 8,16 px
Difference of paired measures
0.2
0.1
0.0
0.04
0.08
0.12
0.16
Mean of paired measures

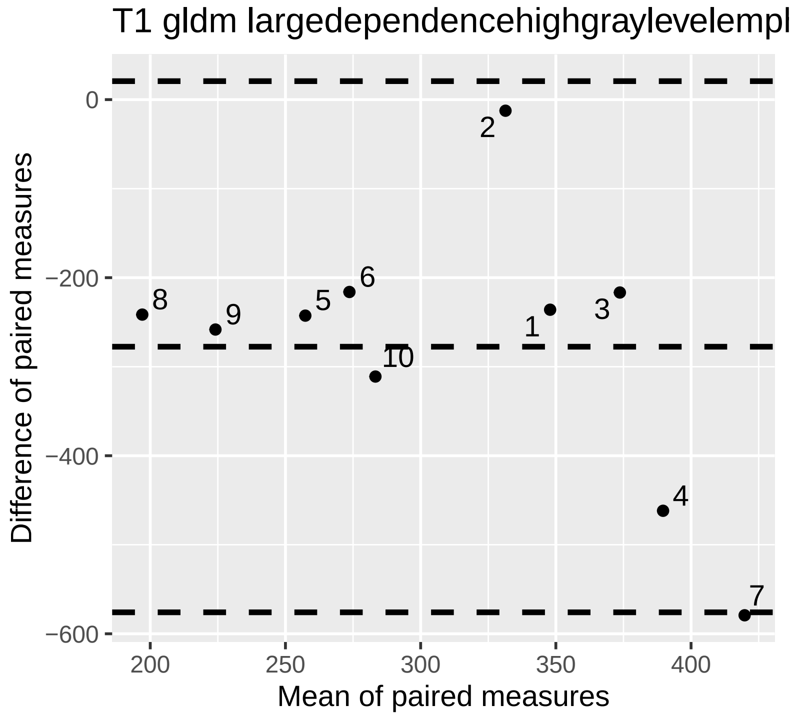
T1 gldm largedependencehighgraylevelempl
Difference of paired measures
0
−200
−400
−600
200
250
300
350
400
Mean of paired measures

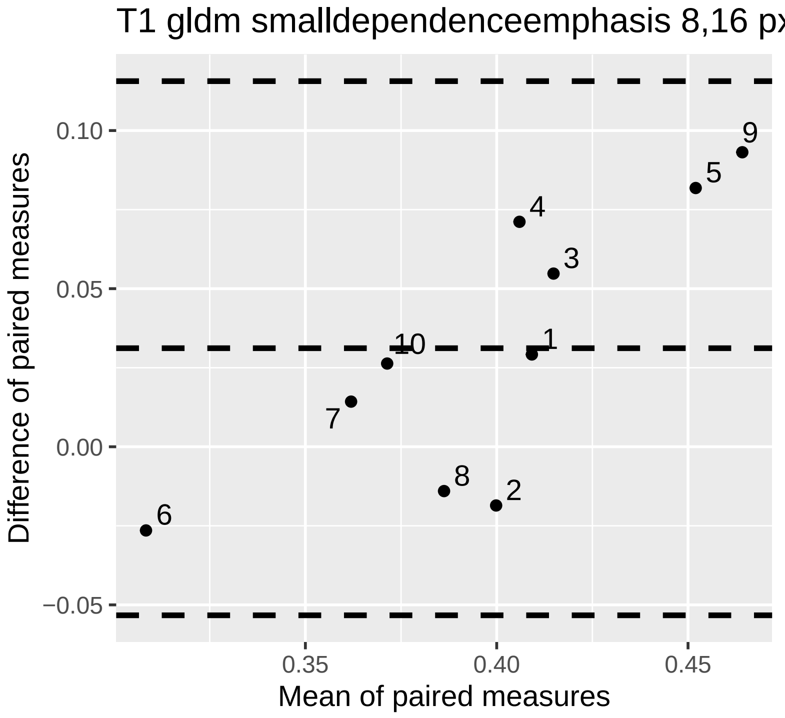
T1 gldm smalldependenceemphasis 8,16 px
Difference of paired measures
0.10
0.05
0.00
−0.05
0.35
0.40
0.45
Mean of paired measures

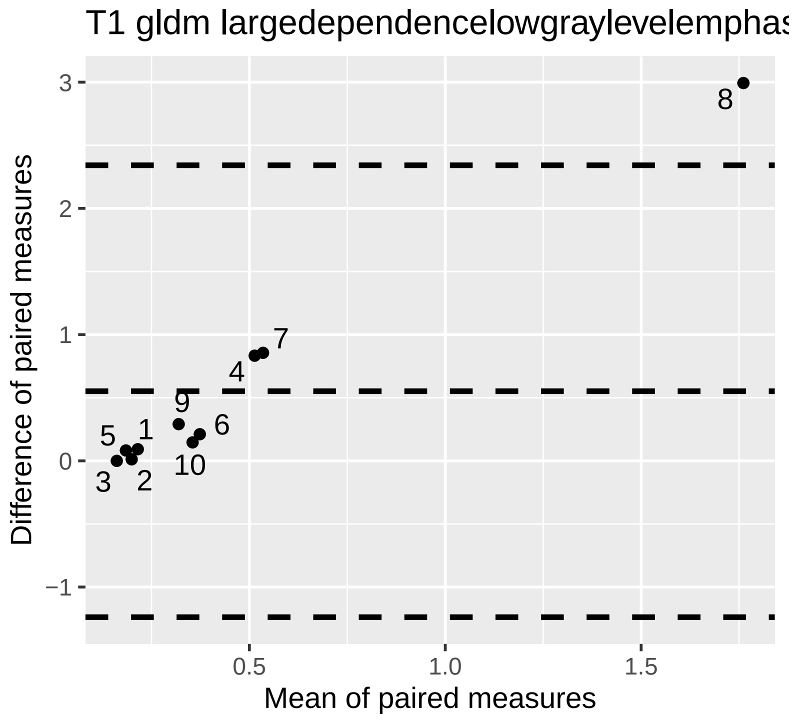
T1 gldm largedependencelowgraylevelemphas
Difference of paired measures
3
2
1
0
−1
0.5
1.0
1.5
Mean of paired measures

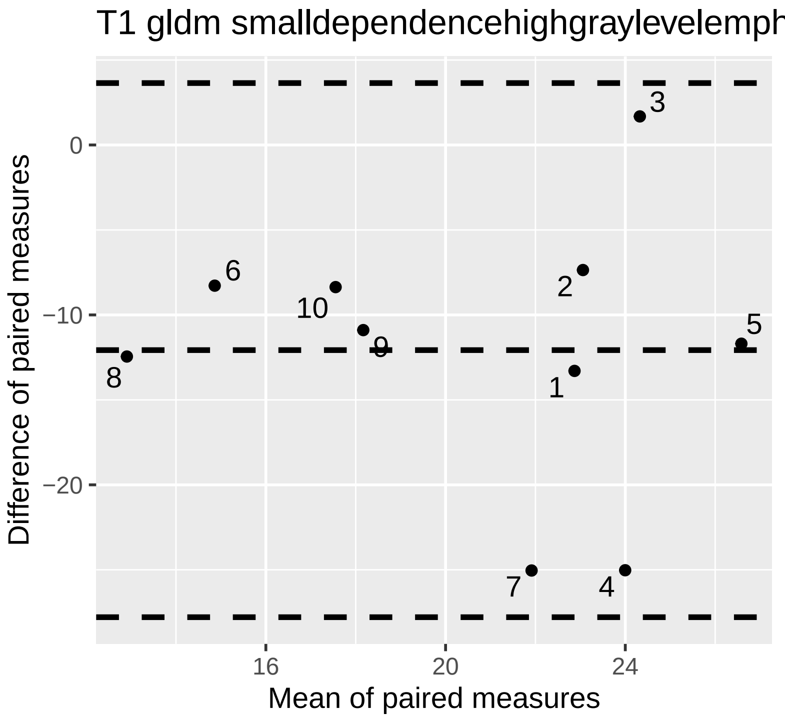
T1 gldm smalldependencehighgraylevelemph
Difference of paired measures
0
−10
−20
16
20
24
Mean of paired measures

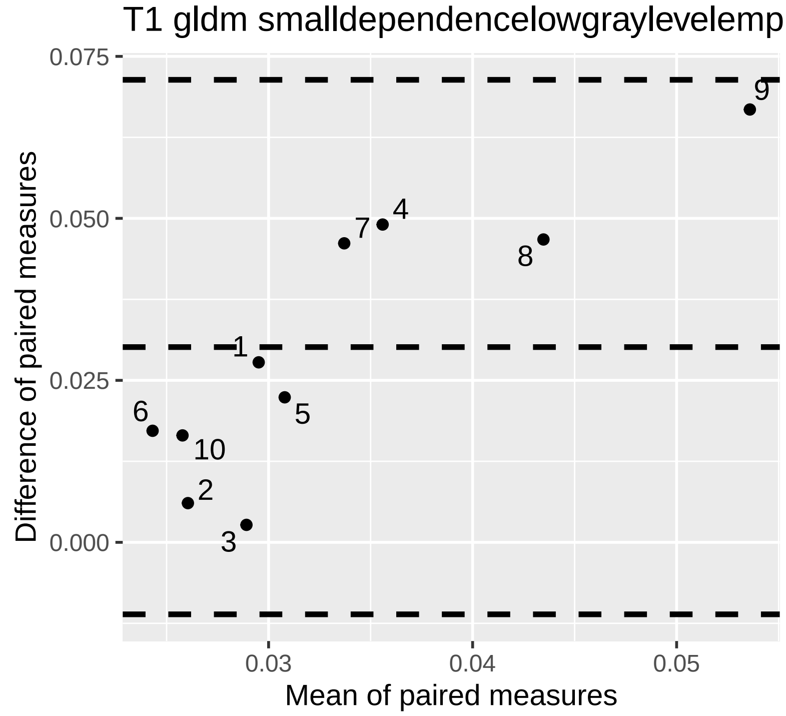
T1 gldm smalldependencelowgraylevelemph
Difference of paired measures
Mean of paired measures
0.075
0.050
0.025
0.000
0.03
0.04
0.05

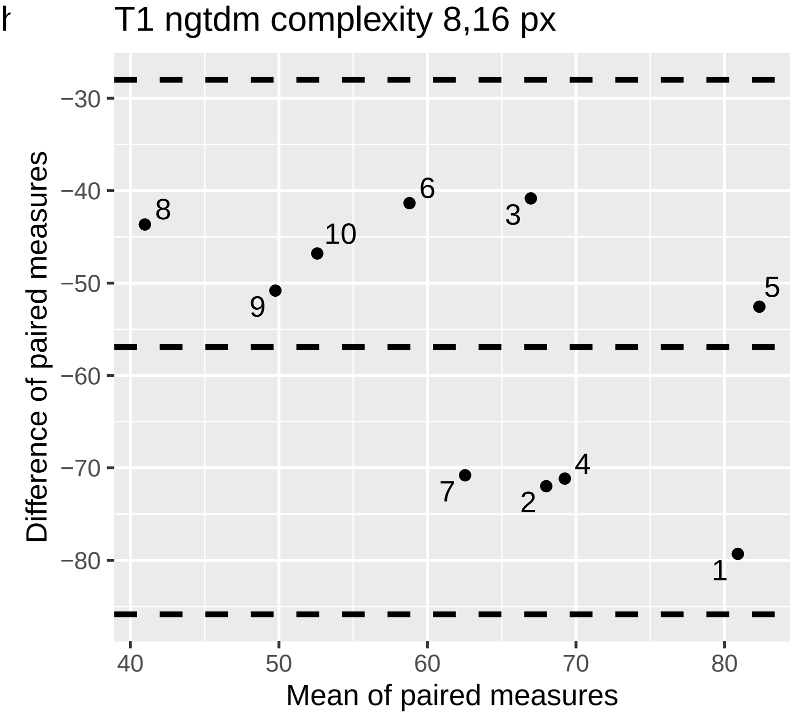
T1 ngtdm complexity 8,16 px
Difference of paired measures
Mean of paired measures
−30
−40
−50
−60
−70
−80
40
50
60
70
80

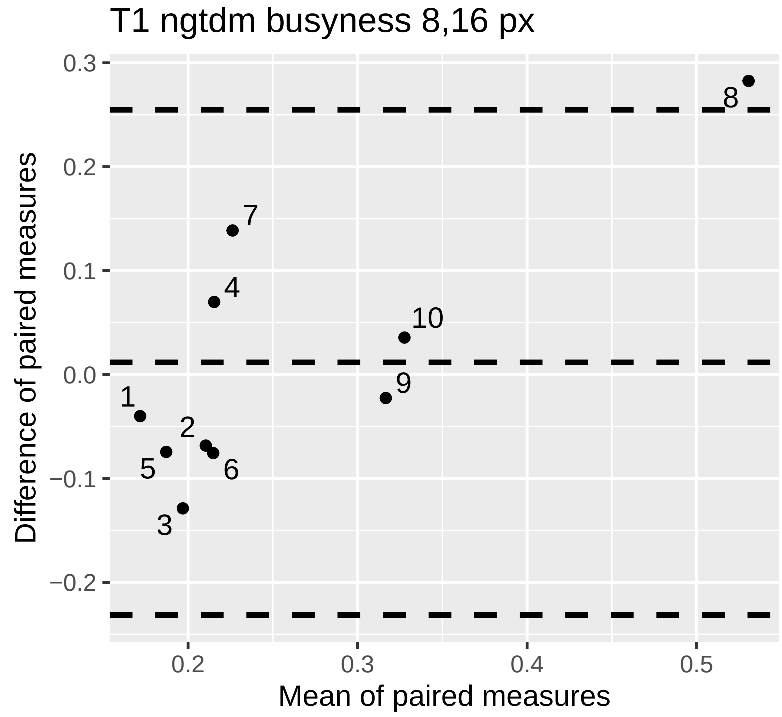
T1 ngtdm busyness 8,16 px
Difference of paired measures
Mean of paired measures
0.3
0.2
0.1
0.0
−0.1
−0.2
0.2
0.3
0.4
0.5

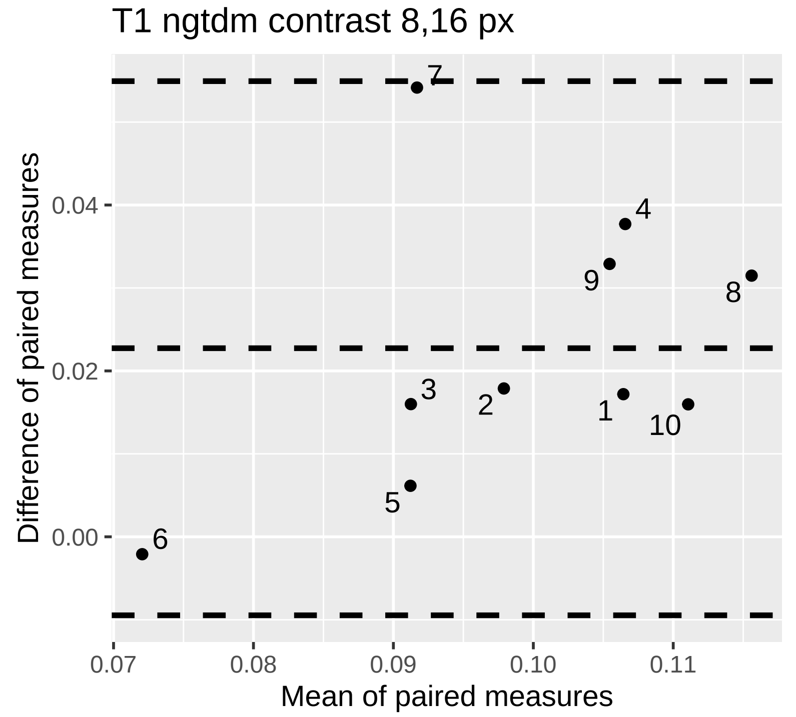
T1 ngtdm contrast 8,16 px
Difference of paired measures
Mean of paired measures
0.04
0.02
0.00
0.07
0.08
0.09
0.10
0.11

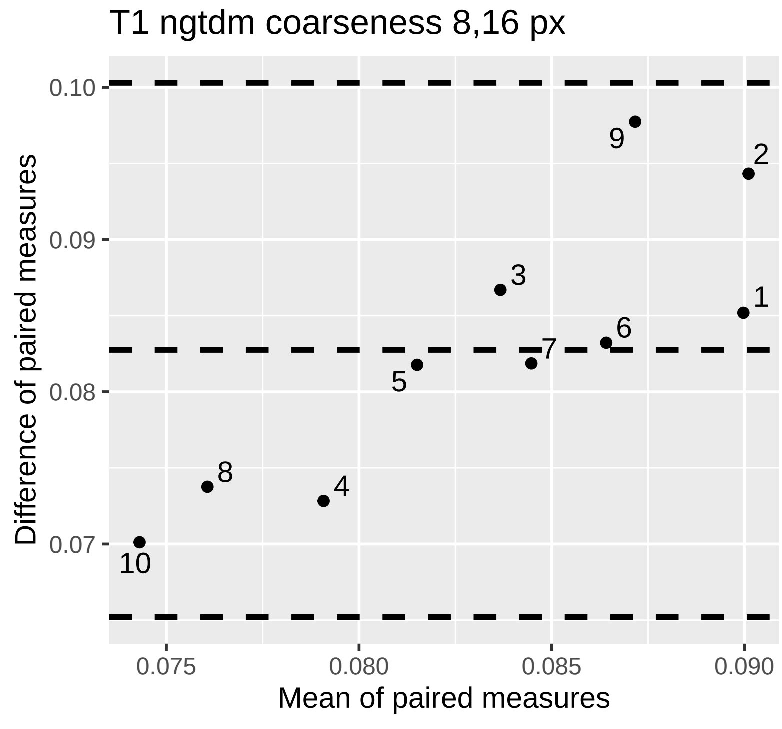
T1 ngtdm coarseness 8,16 px
Difference of paired measures
Mean of paired measures
0.10
0.09
0.08
0.07
0.075
0.080
0.085
0.090

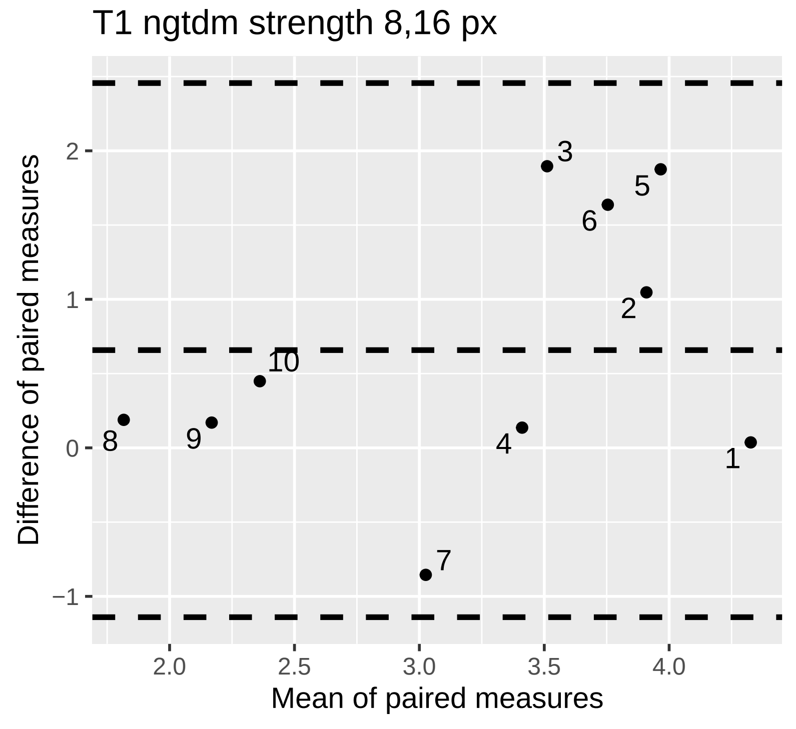
T1 ngtdm strength 8,16 px
Difference of paired measures
Mean of paired measures
2
1
0
−1
2.0
2.5
3.0
3.5
4.0

TIRM firstorder 10percentile 8,16 mm

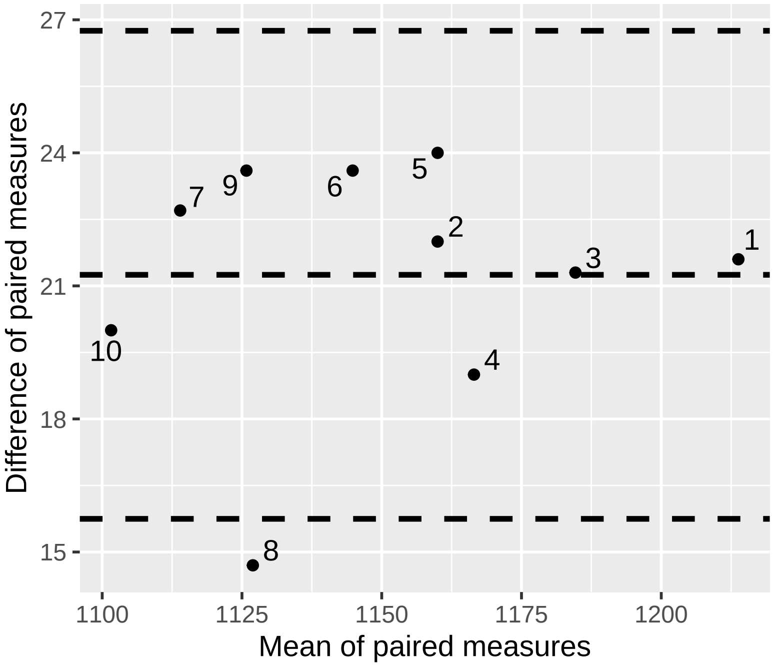


TIRM firstorder entropy 8,16 mm

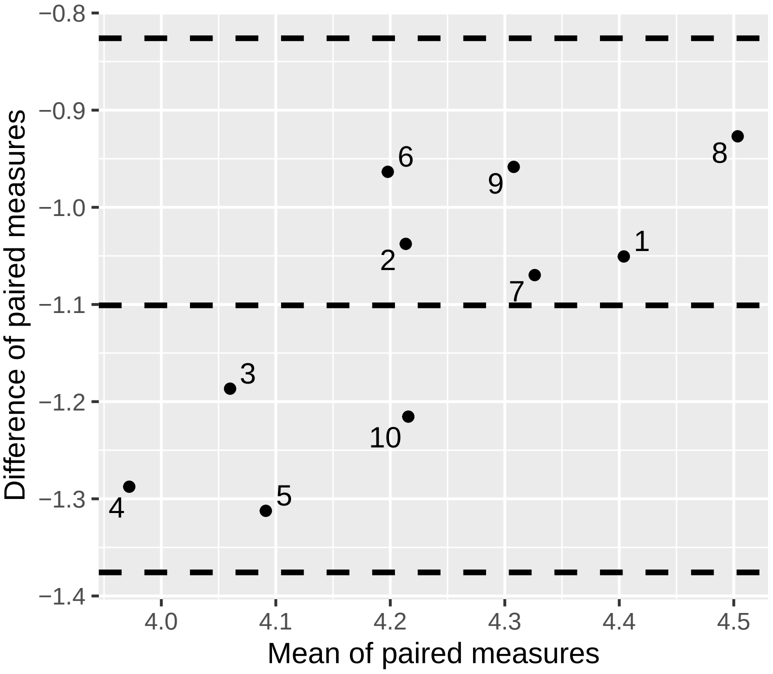


TIRM firstorder 90percentile 8,16 mm

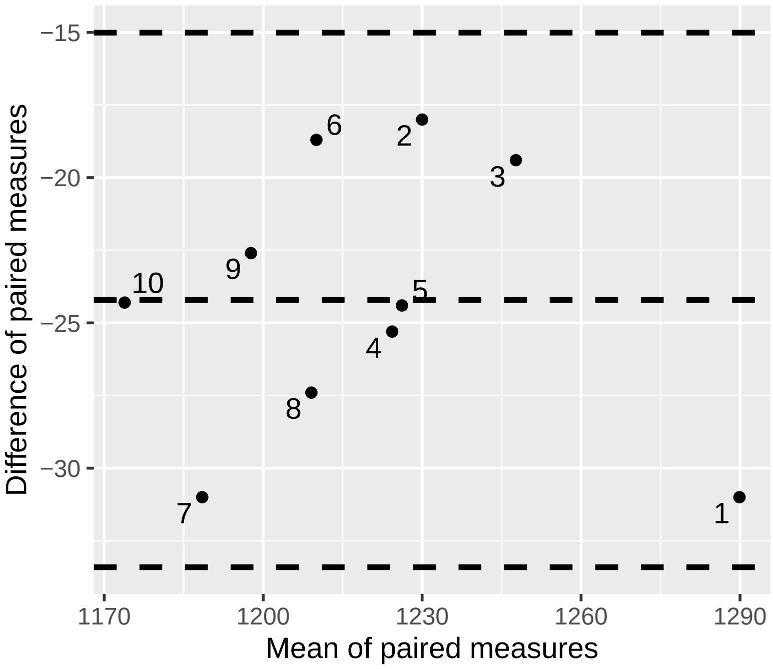


TIRM firstorder interquartilerange 8,16 mm

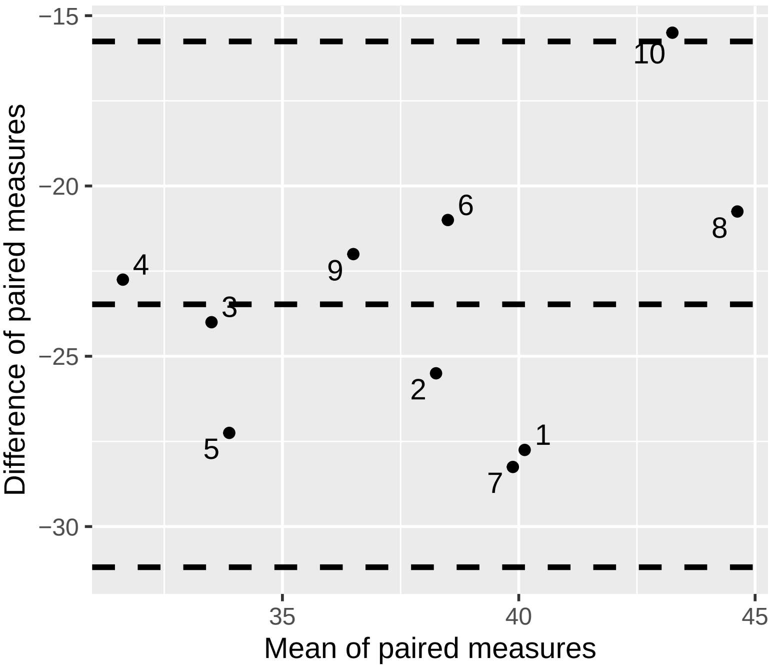


TIRM firstorder energy 8,16 mm

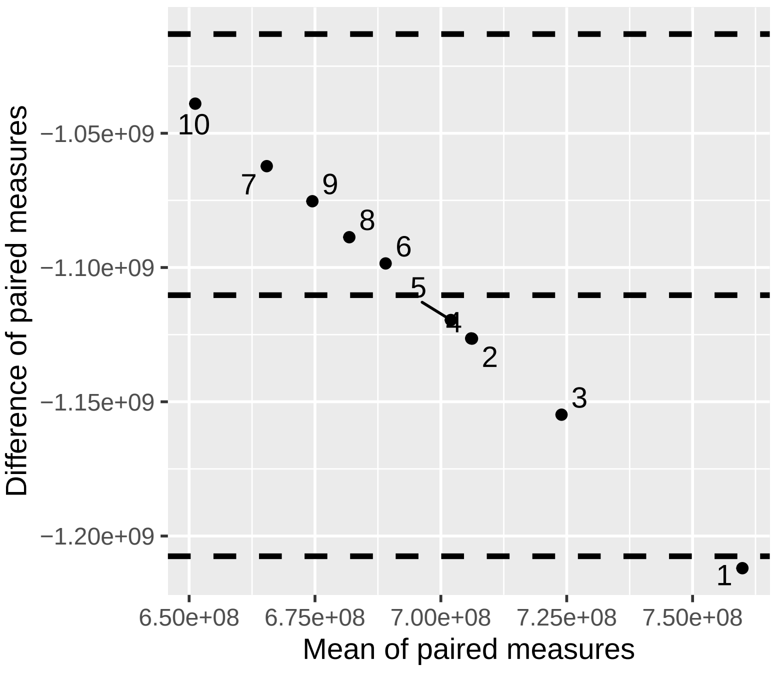


TIRM firstorder kurtosis 8,16 mm

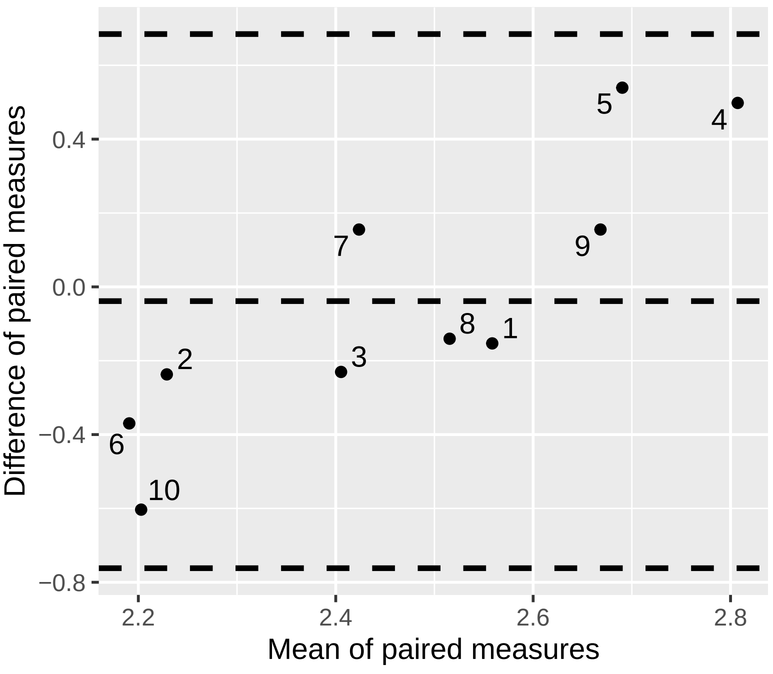

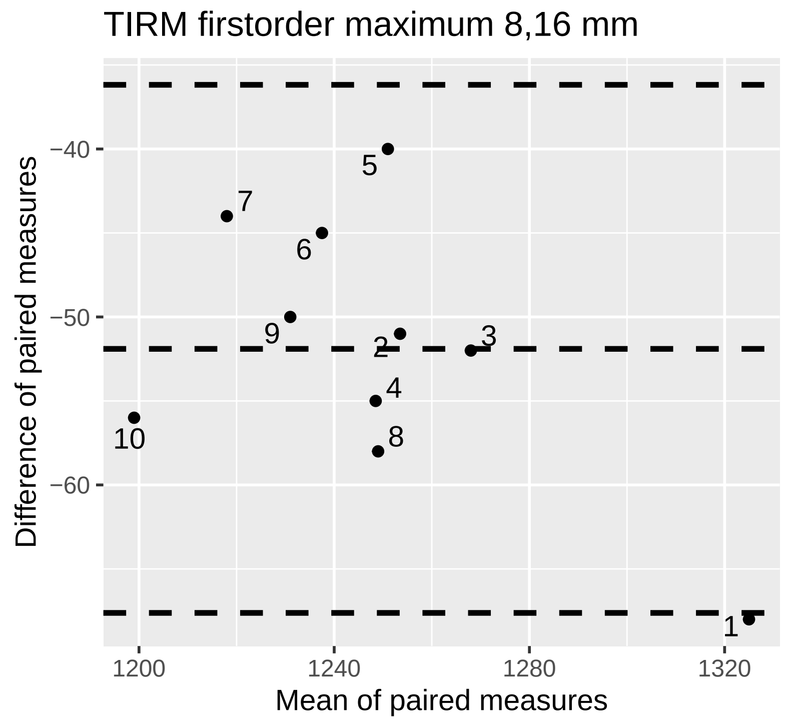
TIRM firstorder maximum 8,16 mm
Difference of paired measures
Mean of paired measures
−40
−50
−60
1200
1240
1280
1320

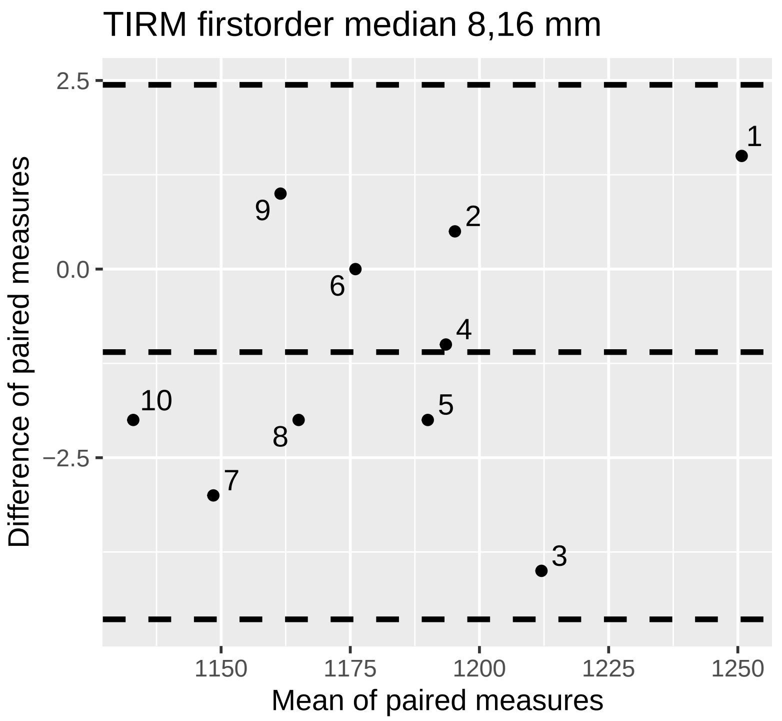
TIRM firstorder median 8,16 mm
Difference of paired measures
Mean of paired measures
2.5
0.0
−2.5
1150
1175
1200
1225
1250

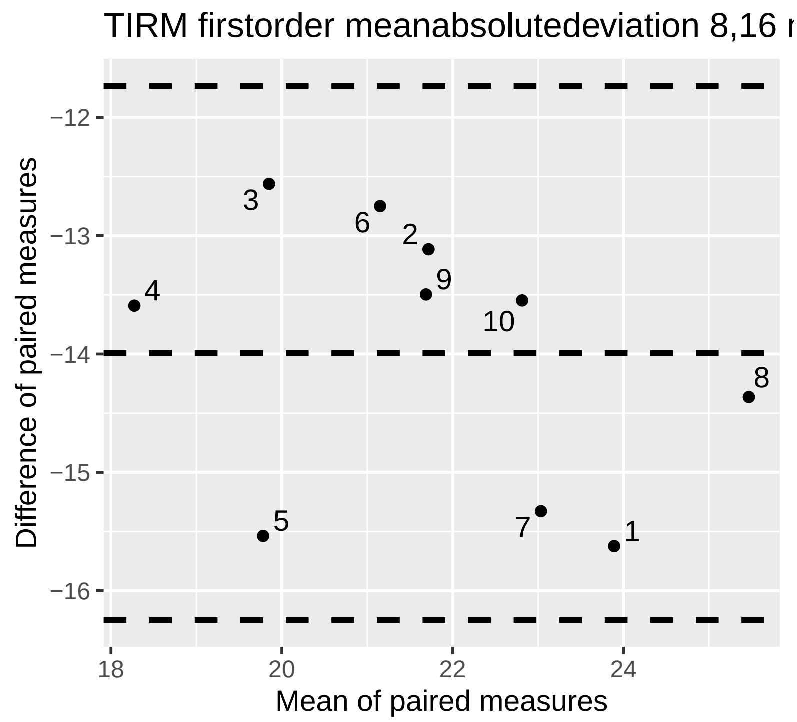
TIRM firstorder meanabsolutedeviation 8,16 r
Difference of paired measures
Mean of paired measures
−12
−13
−14
−15
−16
18
20
22
24

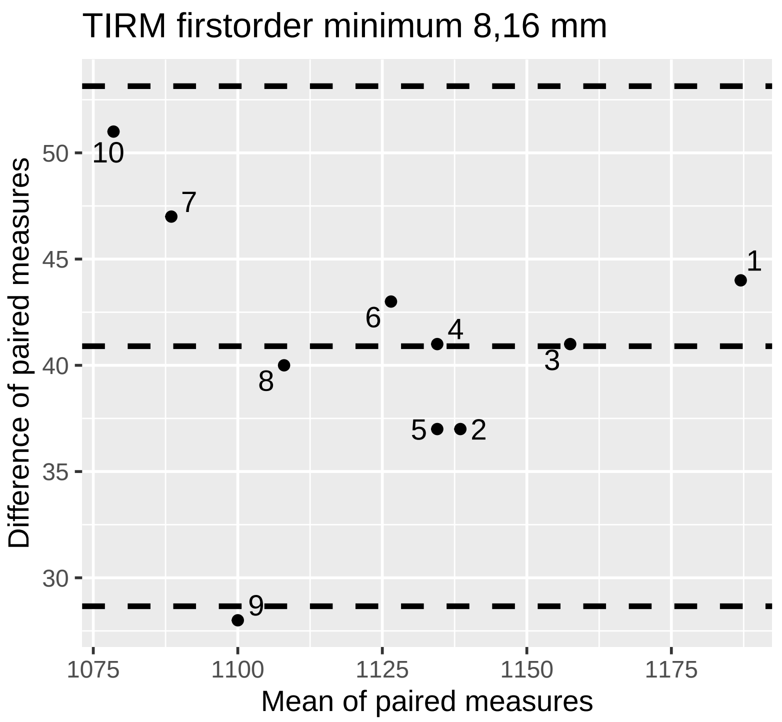
TIRM firstorder minimum 8,16 mm
Difference of paired measures
Mean of paired measures
50
45
40
35
30
1075
1100
1125
1150
1175

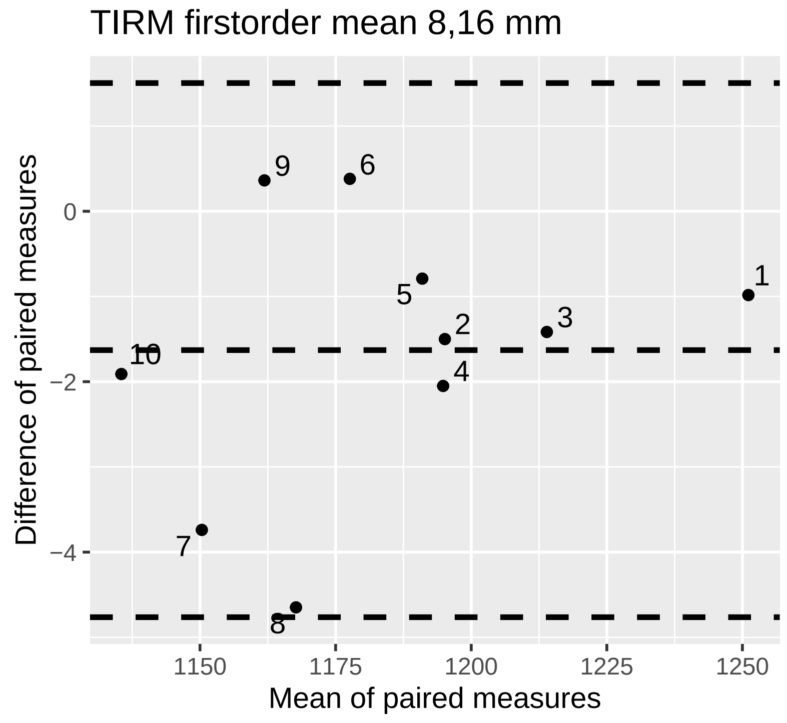
TIRM firstorder mean 8,16 mm
Difference of paired measures
Mean of paired measures
0
−2
−4
1150
1175
1200
1225
1250

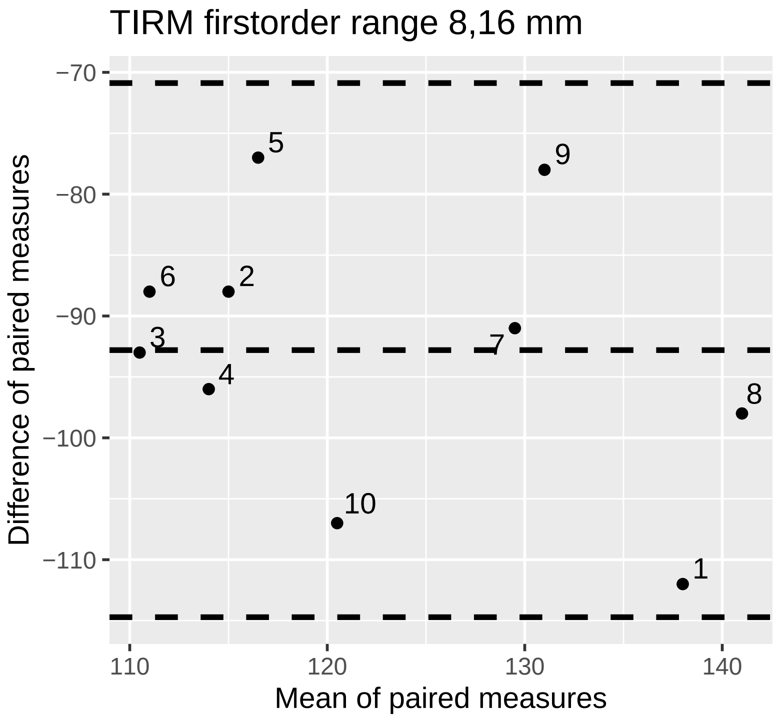
TIRM firstorder range 8,16 mm
Difference of paired measures
Mean of paired measures
−70
−80
−90
−100
−110
110
120
130
140

TIRM firstorder robustmeanabsolutedeviation

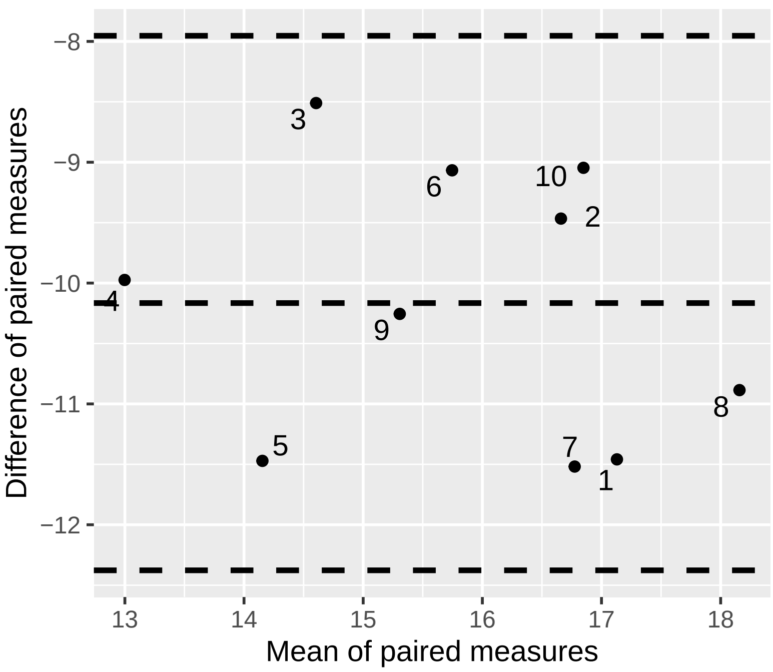


TIRM firstorder totalenergy 8,16 mm

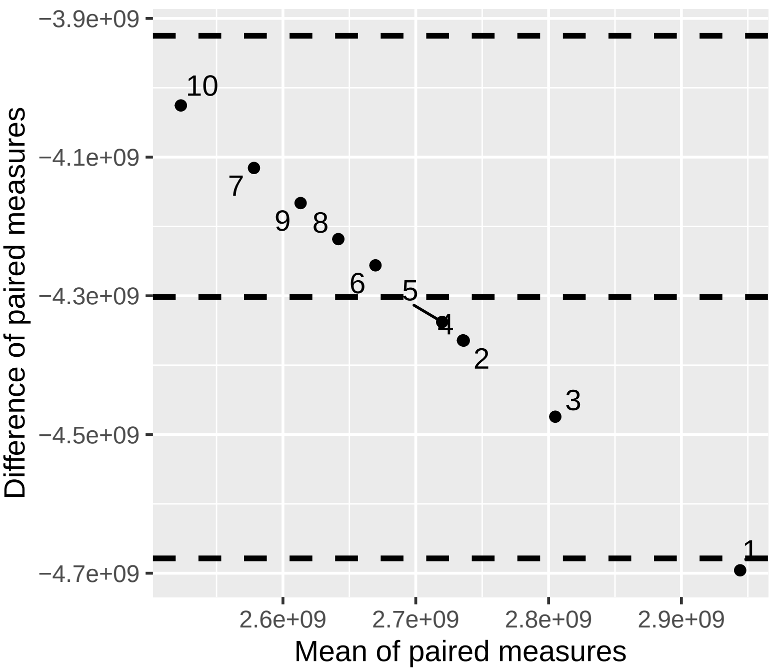


TIRM firstorder rootmeansquared 8,16 mm

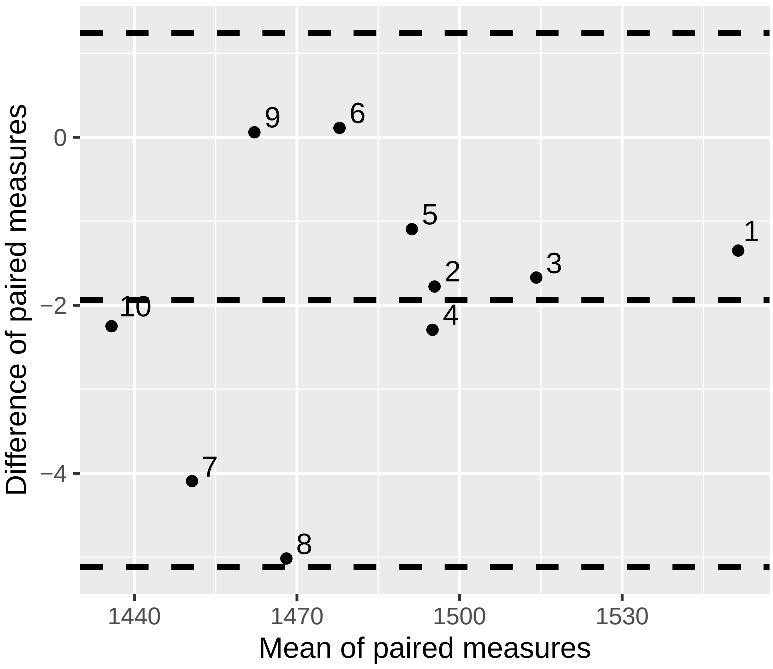


TIRM firstorder uniformity 8,16 mm

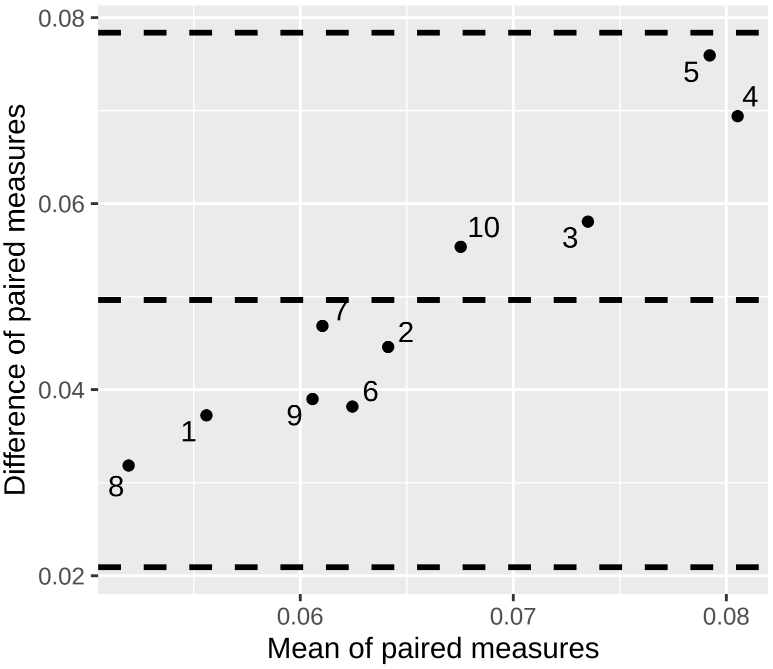


TIRM firstorder skewness 8,16 mm

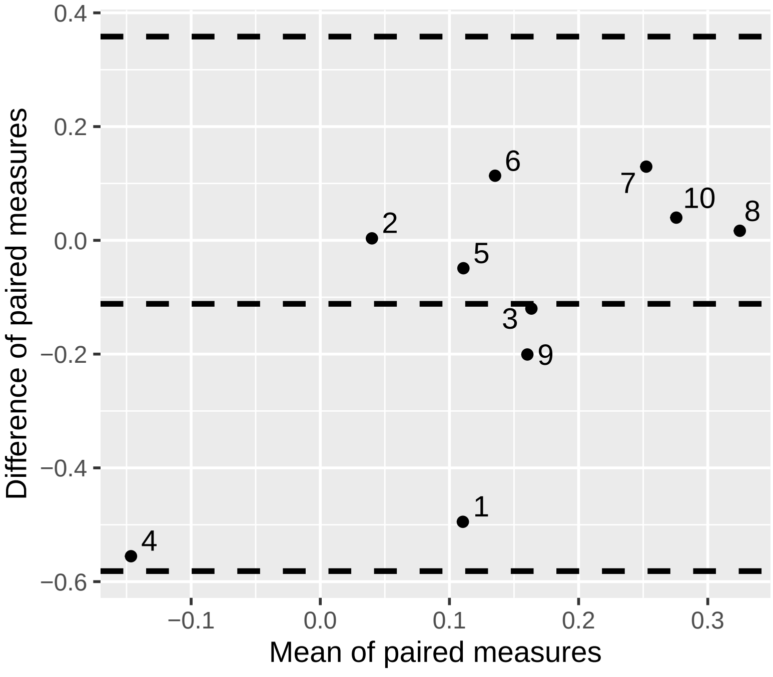


TIRM firstorder variance 8,16 mm

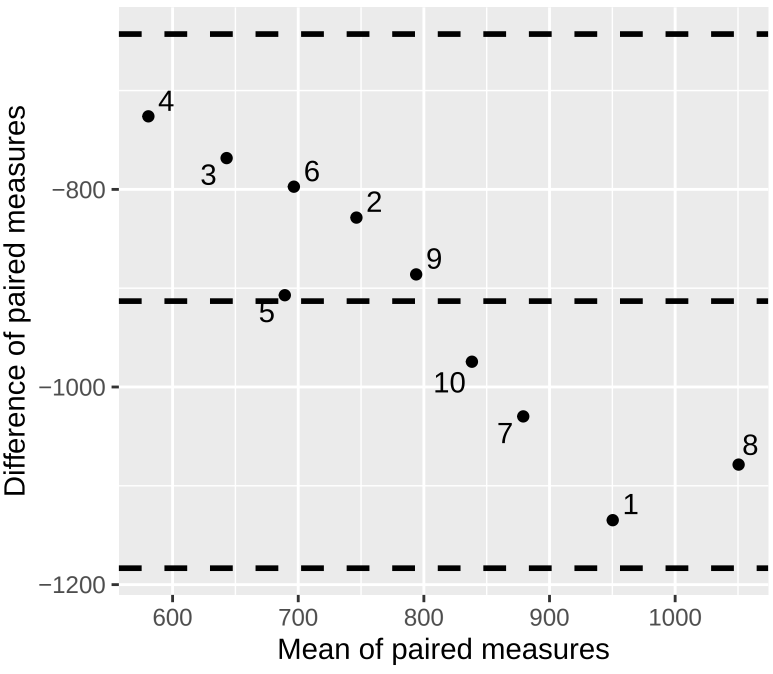

TIRM glcm autocorrelation 8,16 mm

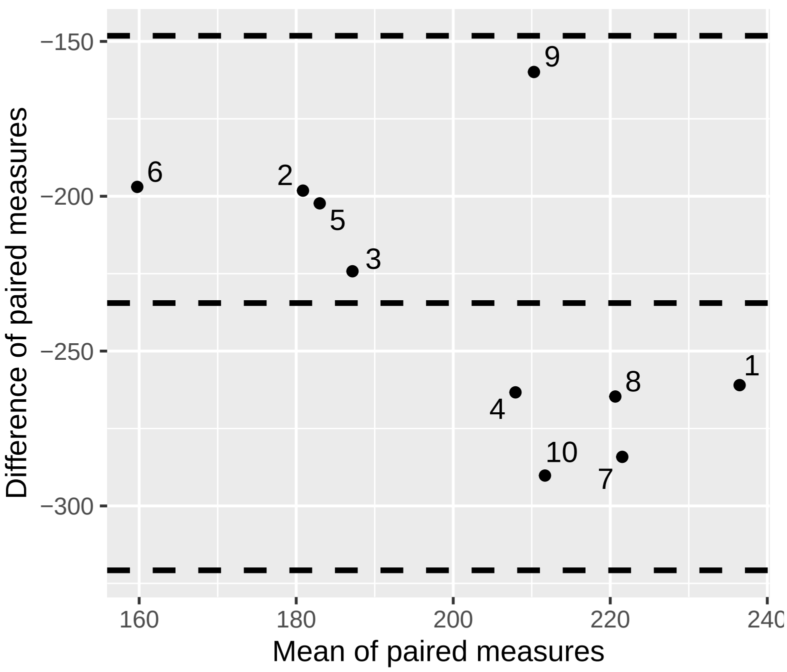


TIRM glcm clustertendency 8,16 mm

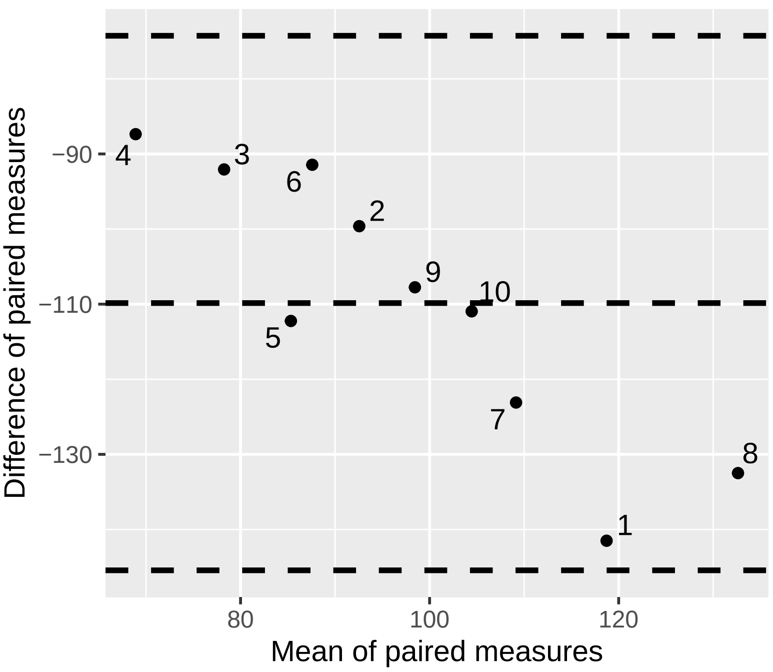


TIRM glcm clusterprominence 8,16 mm

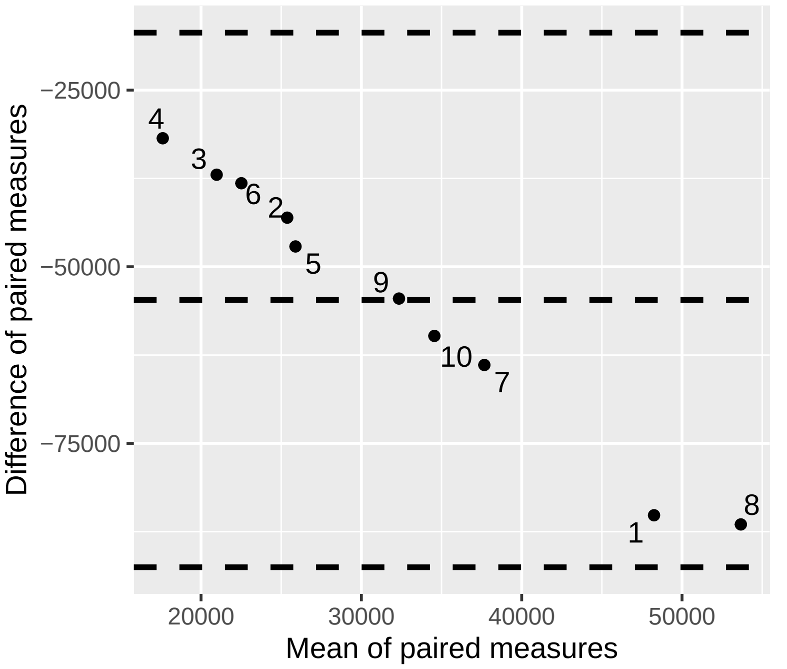


TIRM glcm contrast 8,16 mm

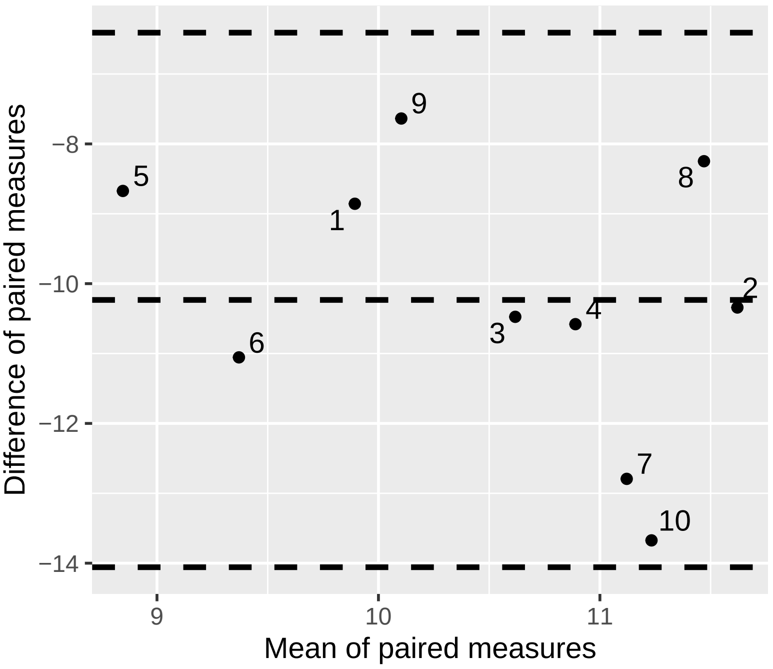


TIRM glcm clustershade 8,16 mm

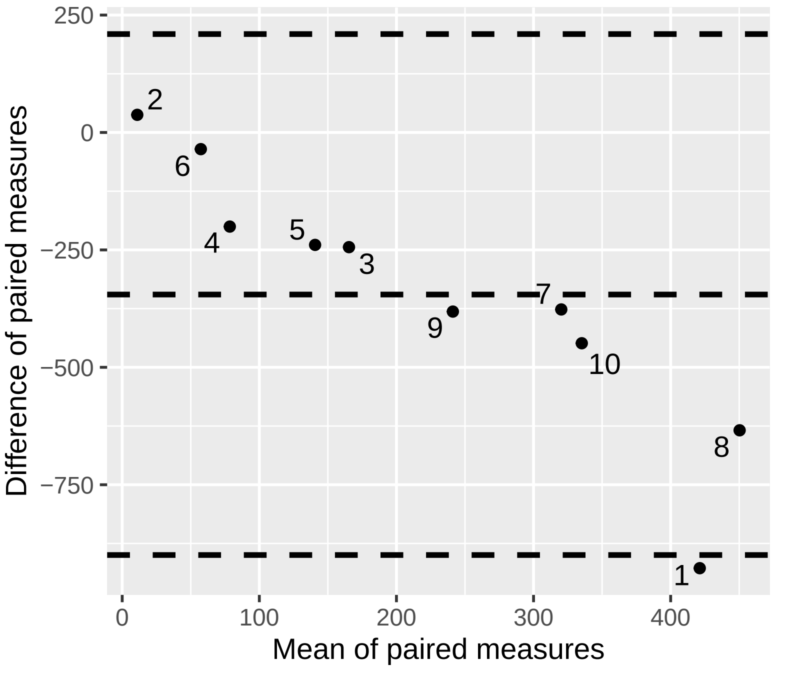


TIRM glcm correlation 8,16 mm

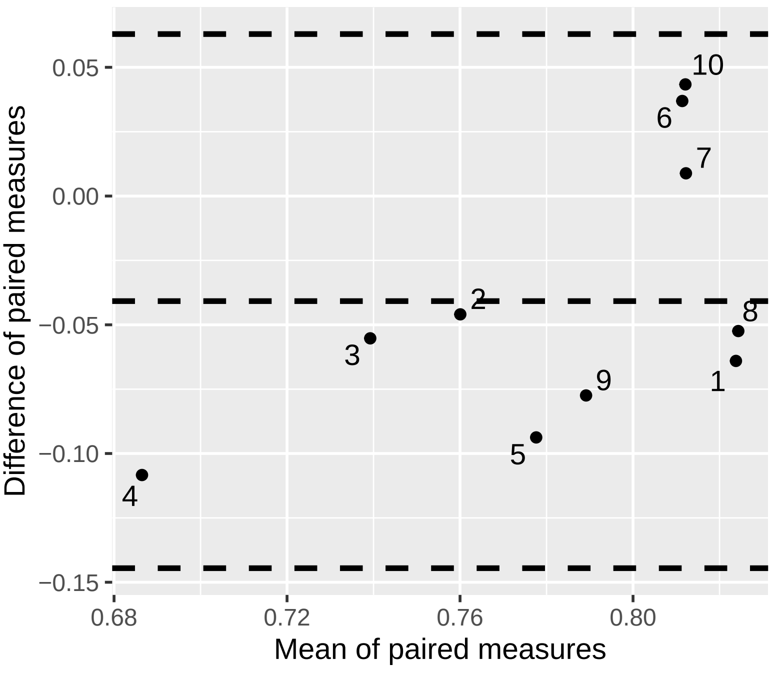

TIRM glcm differenceaverage 8,16 mm

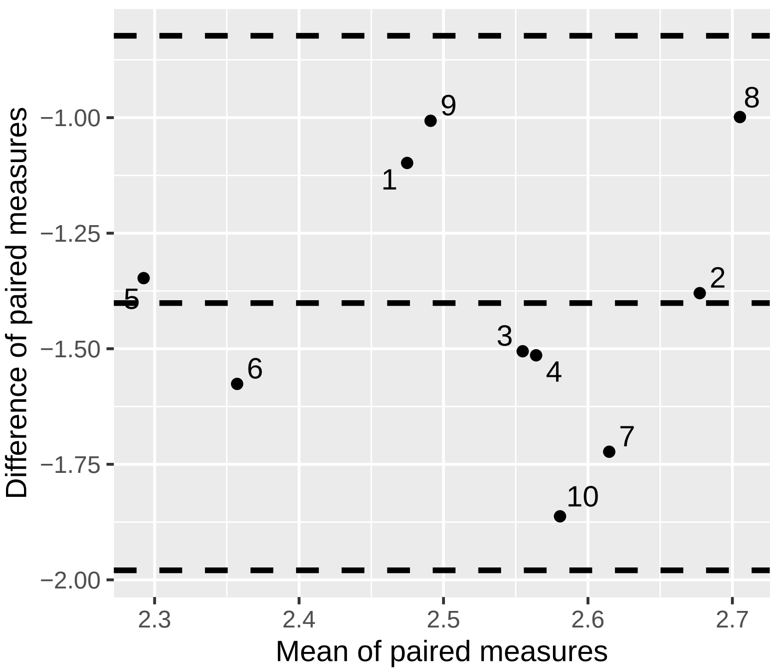


TIRM glcm id 8,16 mm

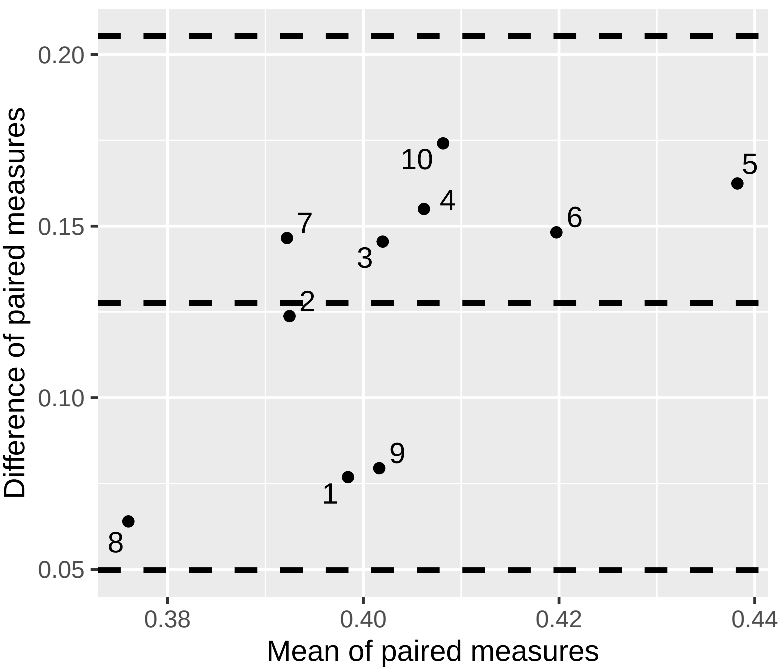


TIRM glcm differenceentropy 8,16 mm

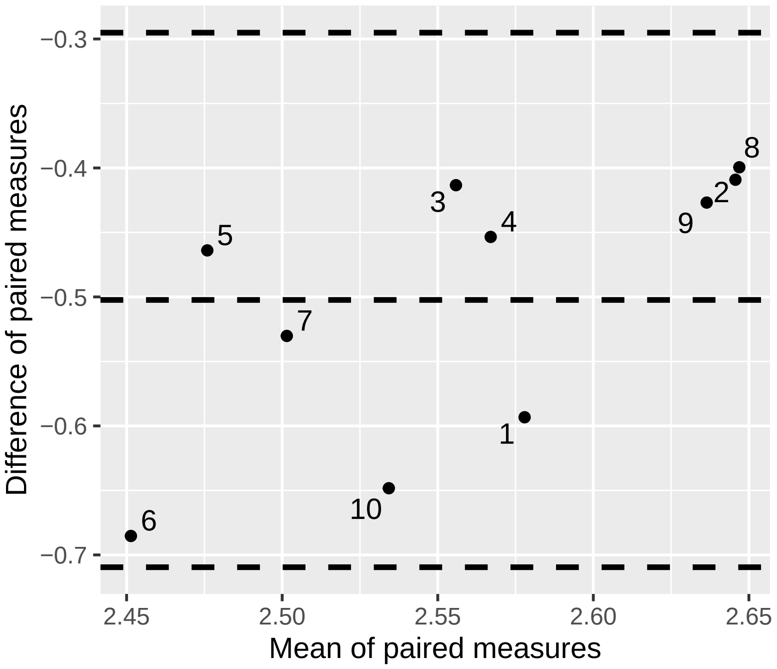


TIRM glcm idm 8,16 mm

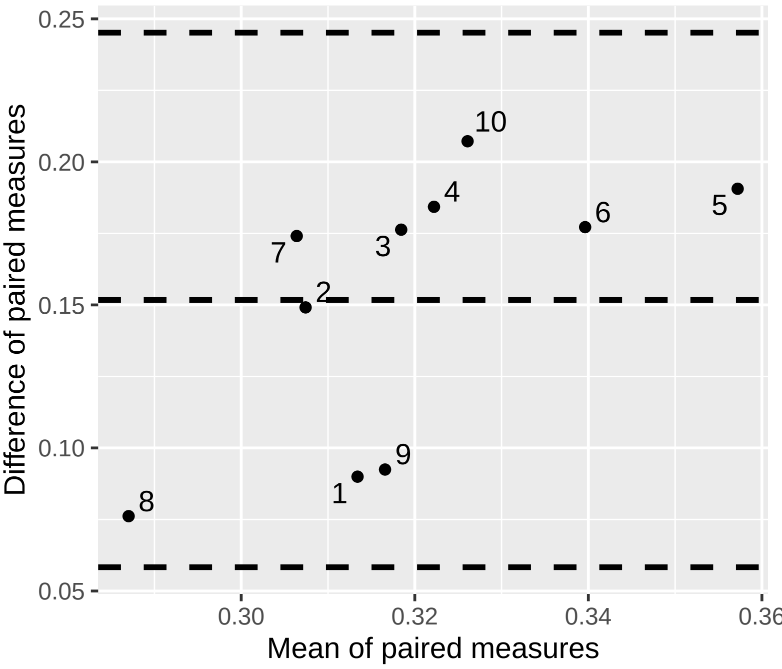


TIRM glcm differencevariance 8,16 mm

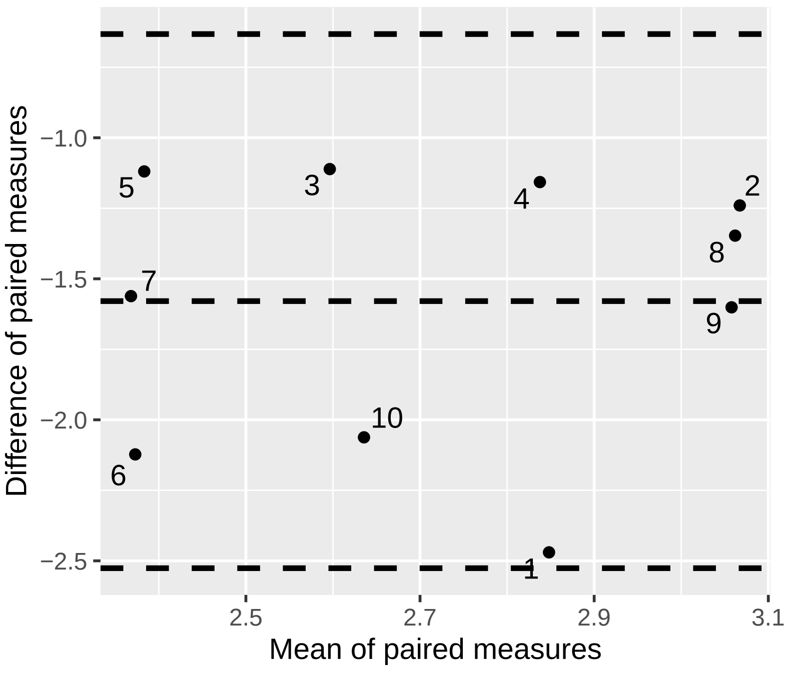


TIRM glcm idmn 8,16 mm

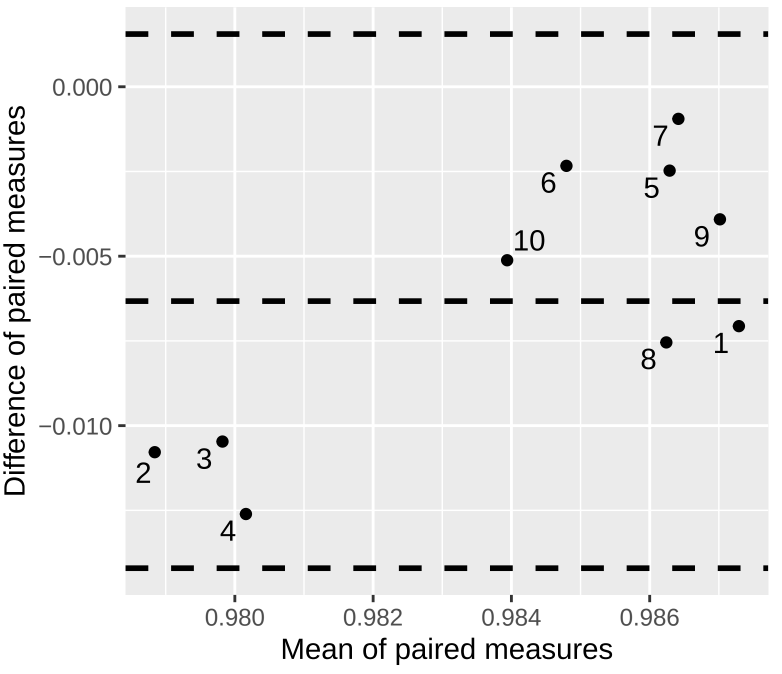

TIRM glcm idn 8,16 mm

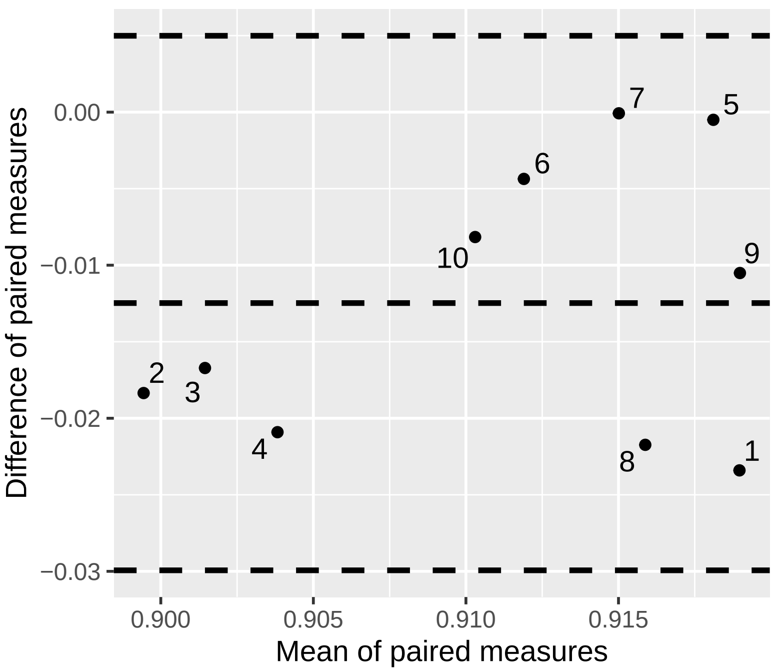


TIRM glcm inversevariance 8,16 mm

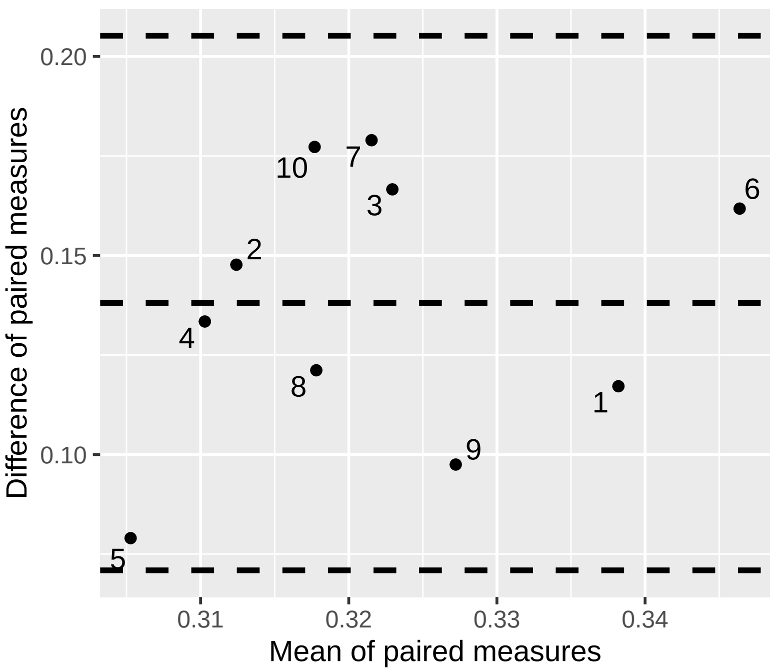


TIRM glcm imc1 8,16 mm

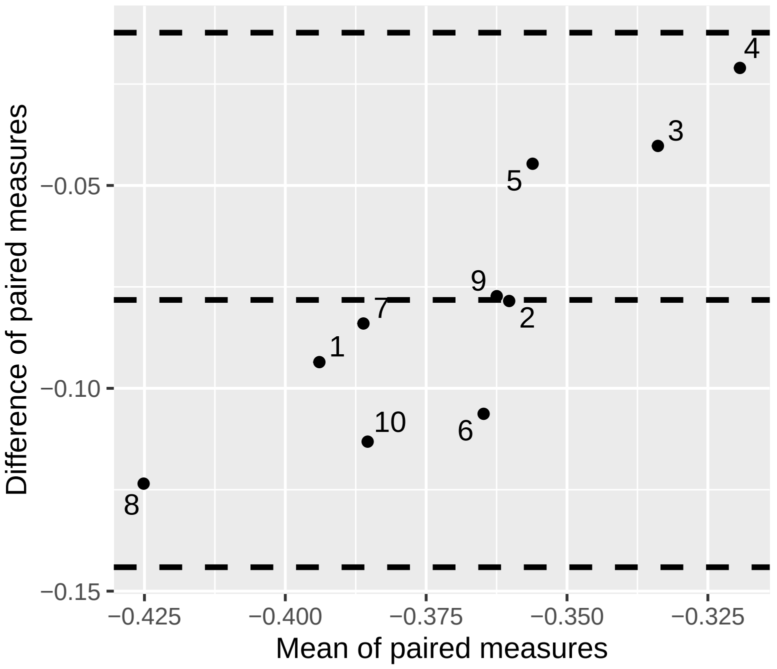


TIRM glcm jointaverage 8,16 mm

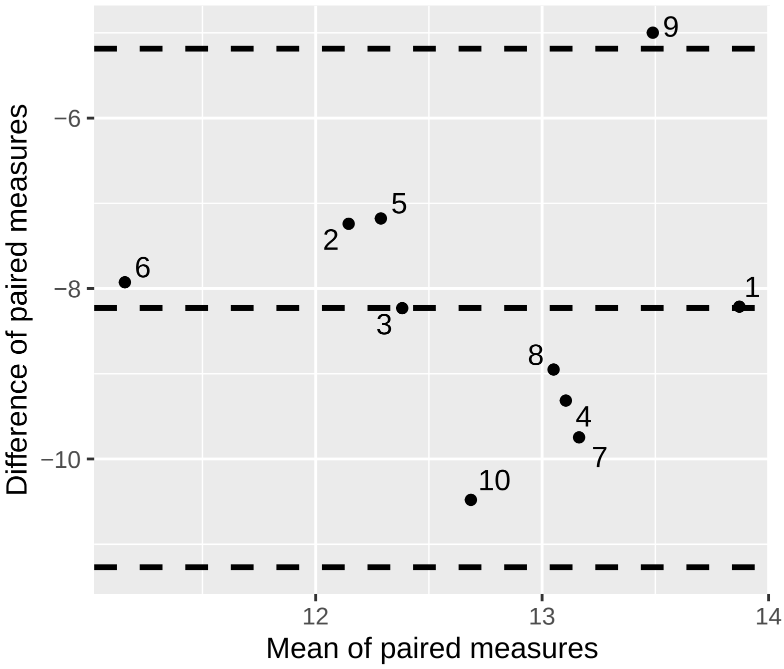


TIRM glcm imc2 8,16 mm

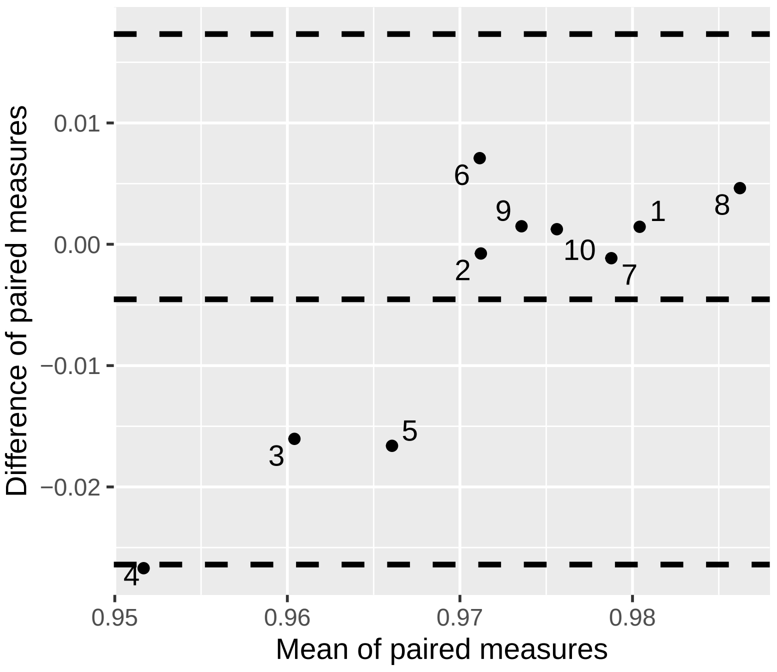


TIRM glcm jointenergy 8,16 mm

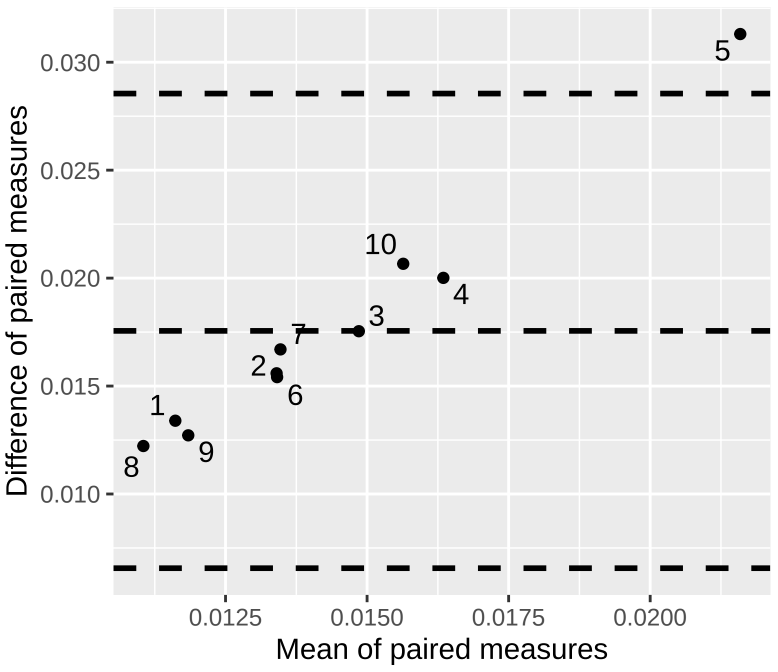

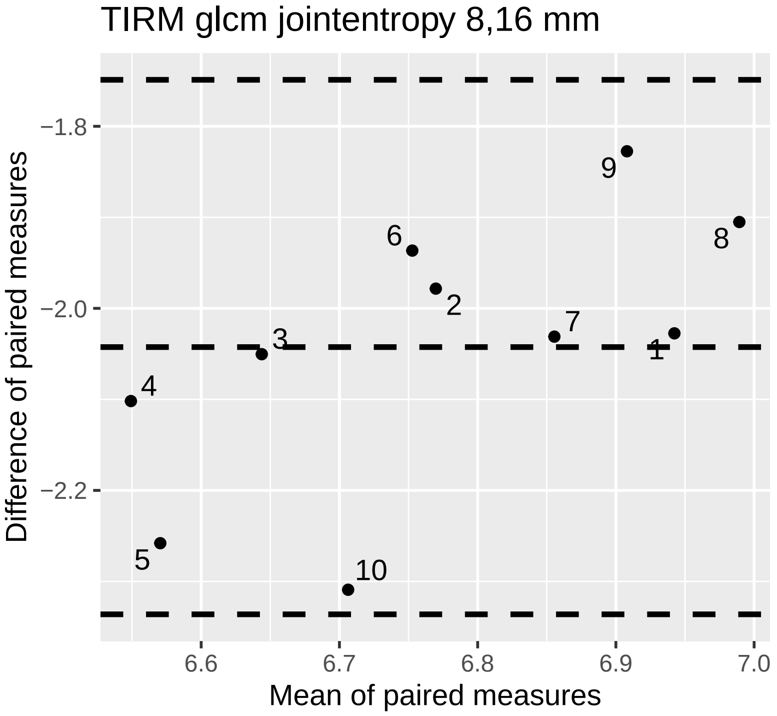
TIRM glcm jointentropy 8,16 mm
Difference of paired measures
-1.8
-2.0
-2.2
Mean of paired measures
6.6
6.7
6.8
6.9
7.0

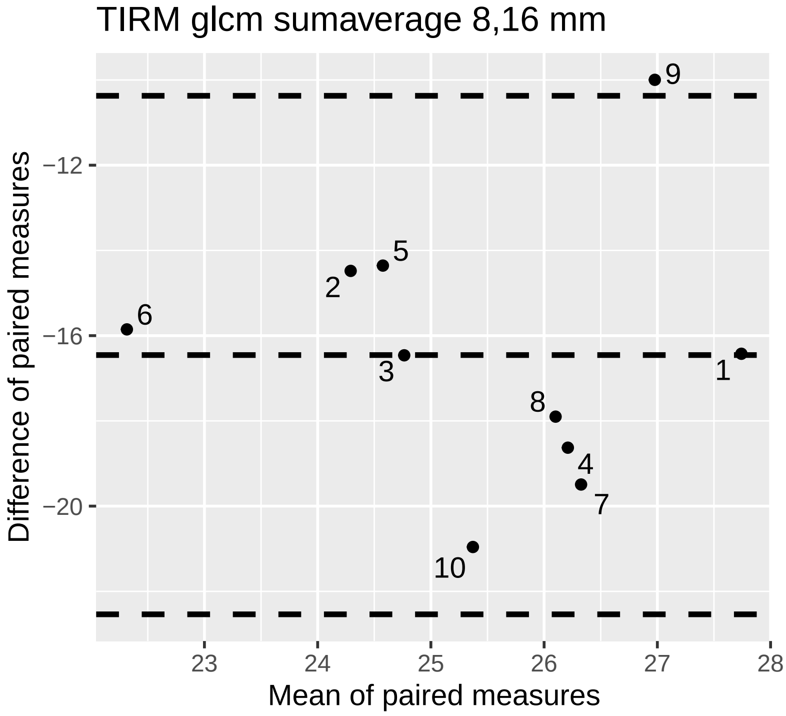
TIRM glcm sumaverage 8,16 mm
Difference of paired measures
-12
-16
-20
Mean of paired measures
23
24
25
26
27
28

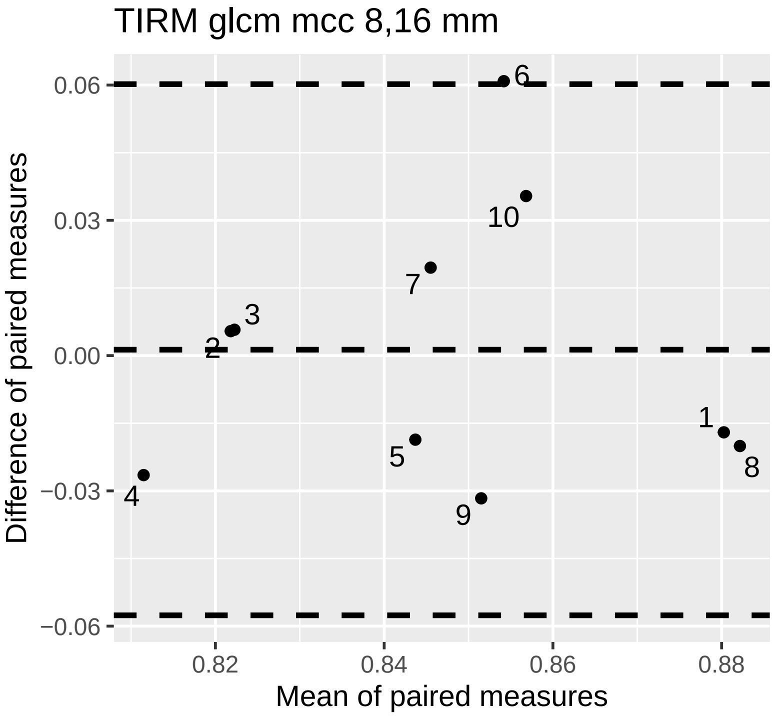
TIRM glcm mcc 8,16 mm
Difference of paired measures
0.06
0.03
0.00
-0.03
-0.06
Mean of paired measures
0.82
0.84
0.86
0.88

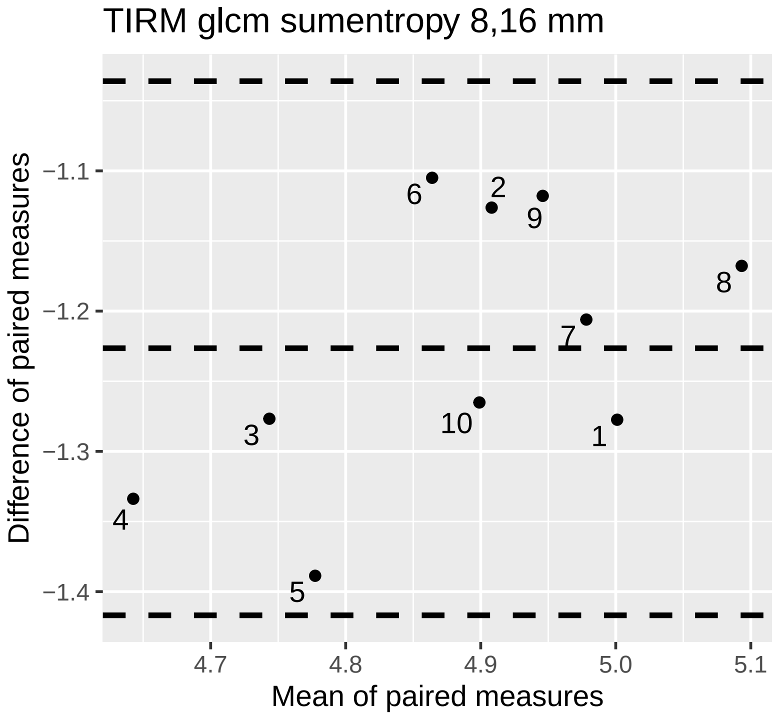
TIRM glcm sumentropy 8,16 mm
Difference of paired measures
-1.1
-1.2
-1.3
-1.4
Mean of paired measures
4.7
4.8
4.9
5.0
5.1

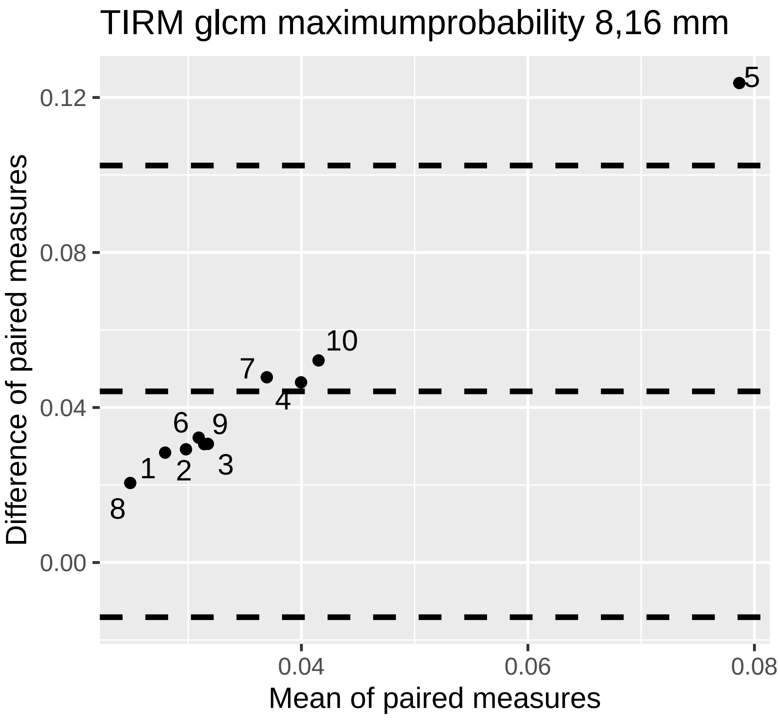
TIRM glcm maximumprobability 8,16 mm
Difference of paired measures
0.12
0.08
0.04
0.00
Mean of paired measures
0.04
0.06
0.08

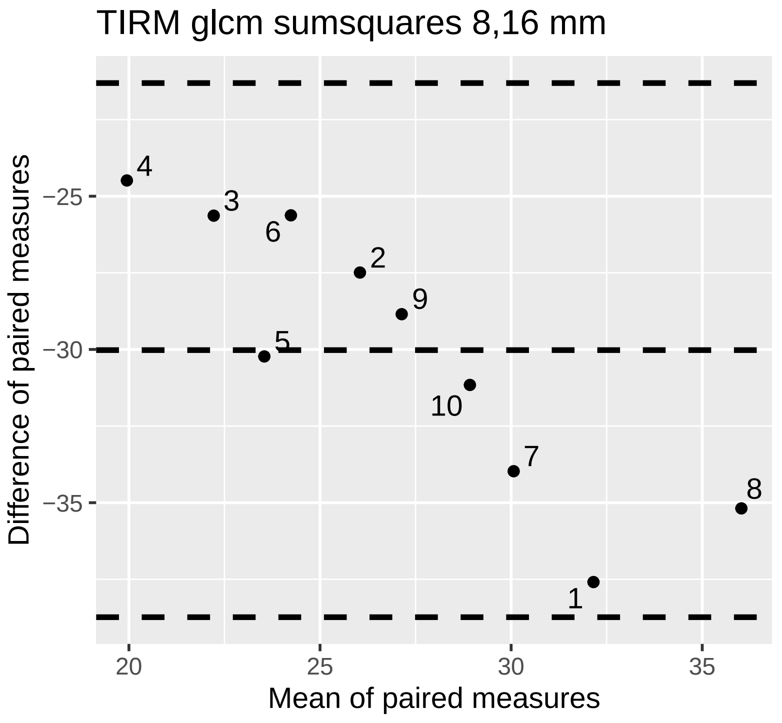
TIRM glcm sumsquares 8,16 mm
Difference of paired measures
-25
-30
-35
Mean of paired measures
20
25
30
35

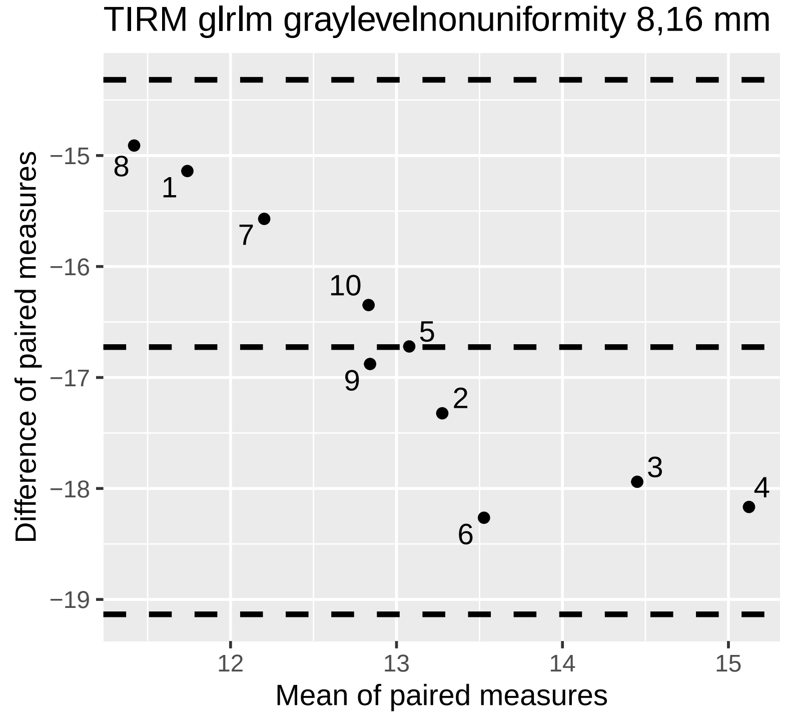
TIRM glrlm graylevelnonuniformity 8,16 mm
Difference of paired measures
Mean of paired measures
-15
-16
-17
-18
-19
12
13
14
15
1
2
3
4
5
6
7
8
9
10

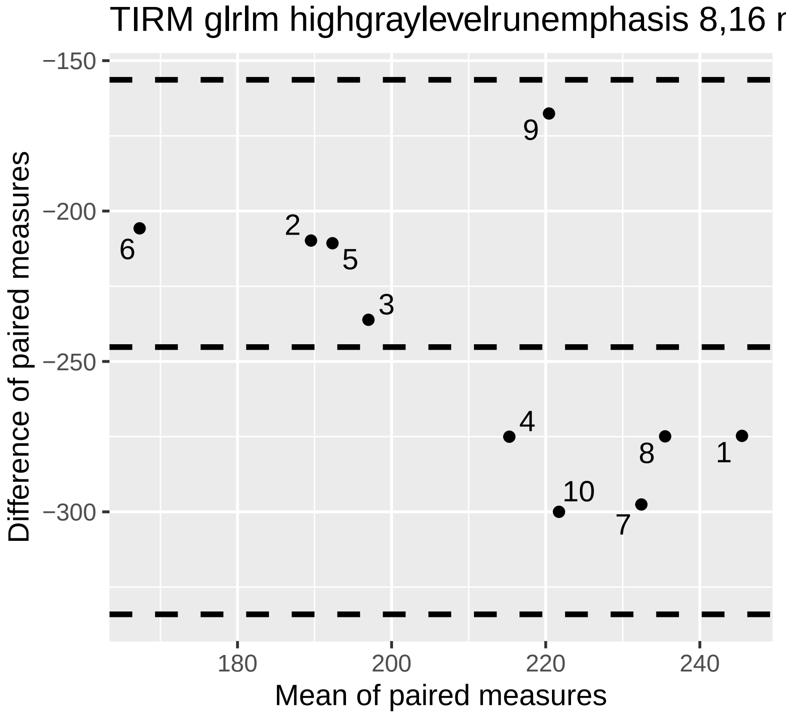
TIRM glrlm highgraylevelrunemphasis 8,16 m
Difference of paired measures
Mean of paired measures
-150
-200
-250
-300
180
200
220
240
1
2
3
4
5
6
7
8
9
10

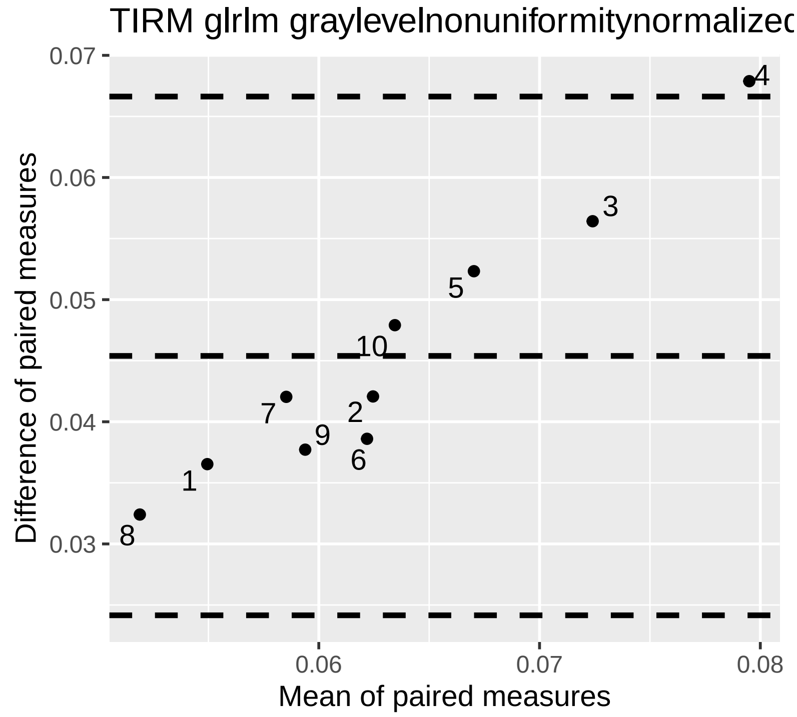
TIRM glrlm graylevelnonuniformitynormalizec
Difference of paired measures
Mean of paired measures
0.07
0.06
0.05
0.04
0.03
0.06
0.07
0.08
1
2
3
4
5
6
7
8
9
10

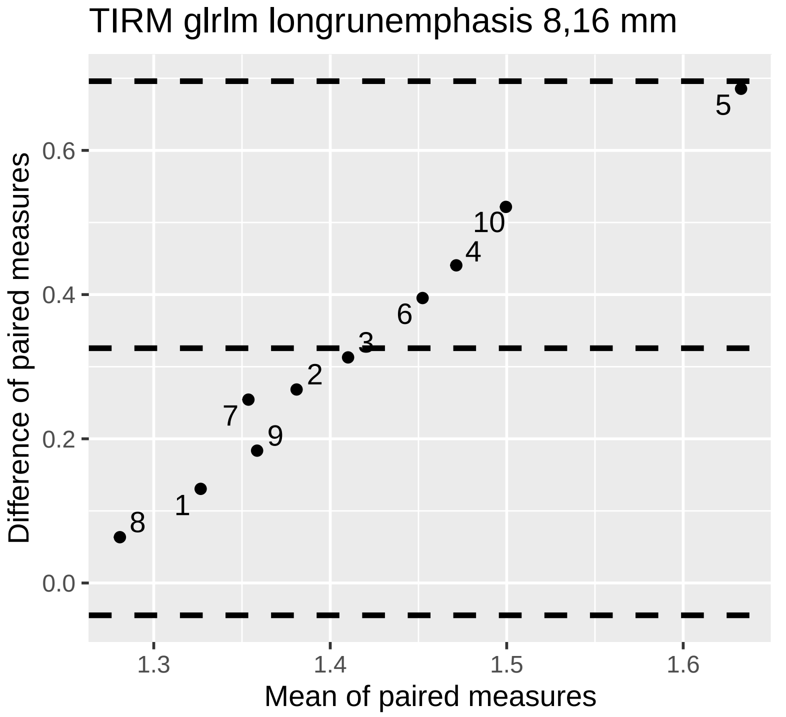
TIRM glrlm longrunemphasis 8,16 mm
Difference of paired measures
Mean of paired measures
0.6
0.4
0.2
0.0
1.3
1.4
1.5
1.6
1
2
3
4
5
6
7
8
9
10

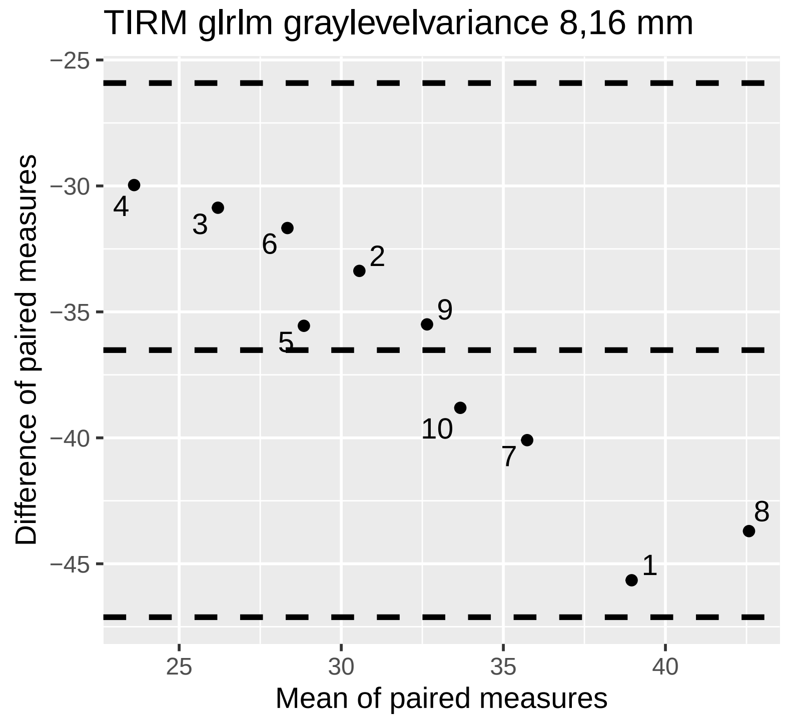
TIRM glrlm graylevelvariance 8,16 mm
Difference of paired measures
Mean of paired measures
-25
-30
-35
-40
-45
25
30
35
40
1
2
3
4
5
6
7
8
9
10

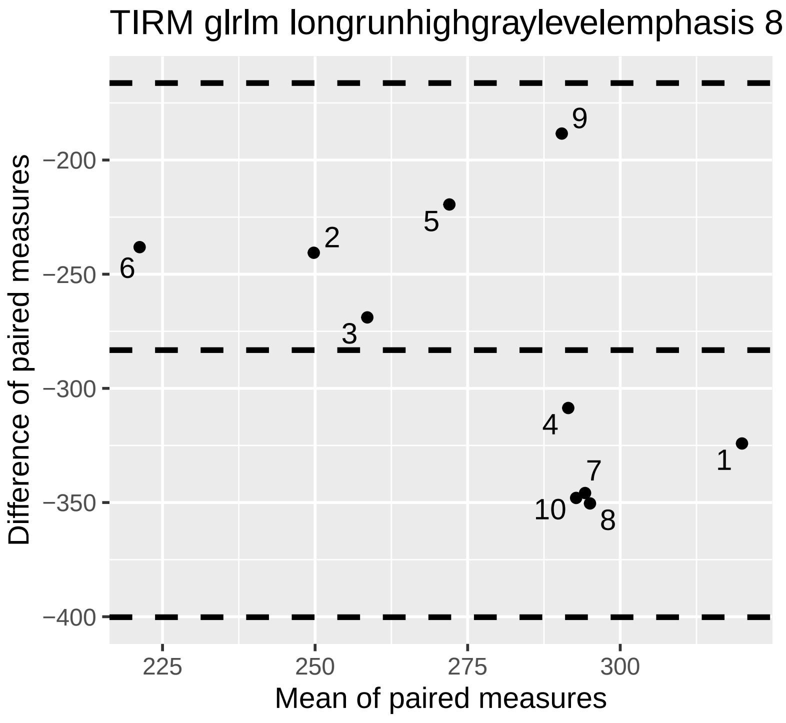
TIRM glrlm longrunhighgraylevelemphasis 8
Difference of paired measures
Mean of paired measures
-200
-250
-300
-350
-400
225
250
275
300
1
2
3
4
5
6
7
8
9
10

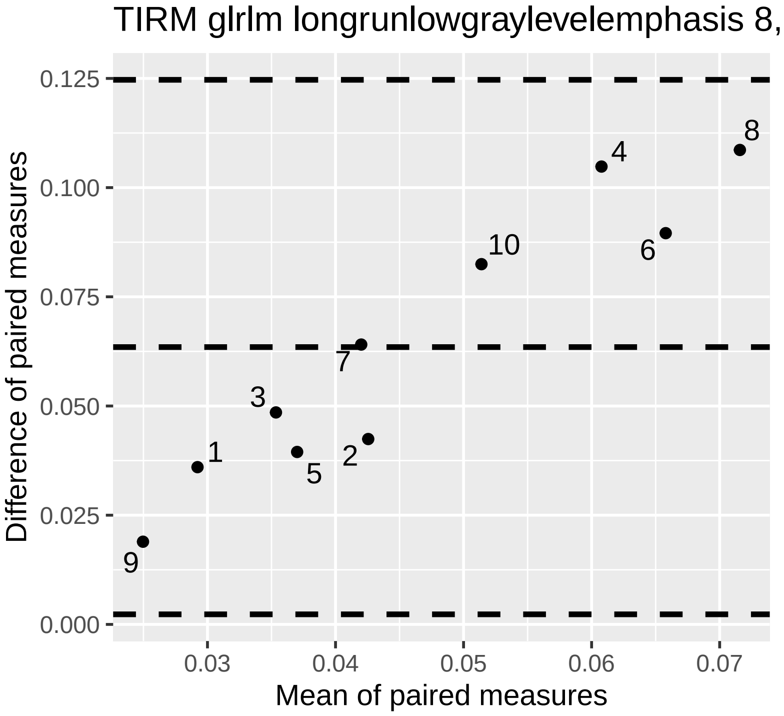
TIRM glrlm longrunlowgraylevelemphasis 8,
Difference of paired measures
Mean of paired measures
0.125
0.100
0.075
0.050
0.025
0.000
0.03
0.04
0.05
0.06
0.07

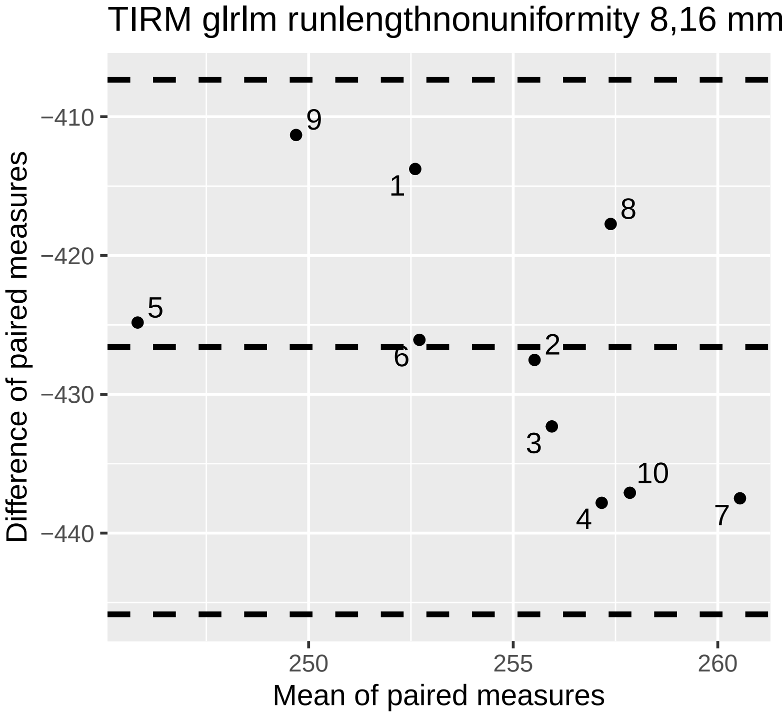
TIRM glrlm runlengthnonuniformity 8,16 mm
Difference of paired measures
Mean of paired measures
−410
−420
−430
−440
250
255
260

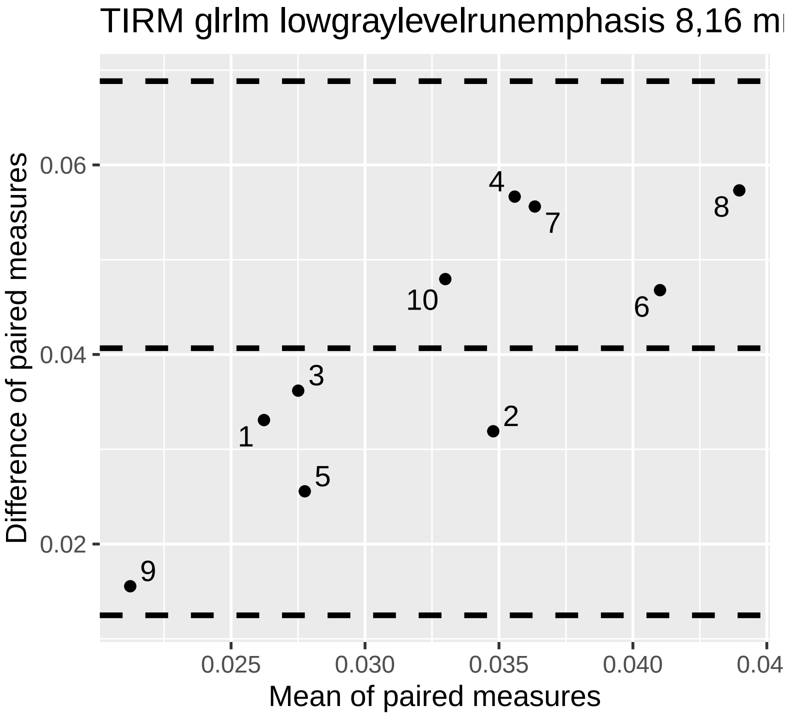
TIRM glrlm lowgraylevelrunemphasis 8,16 m
Difference of paired measures
Mean of paired measures
0.06
0.04
0.02
0.025
0.030
0.035
0.040
0.04

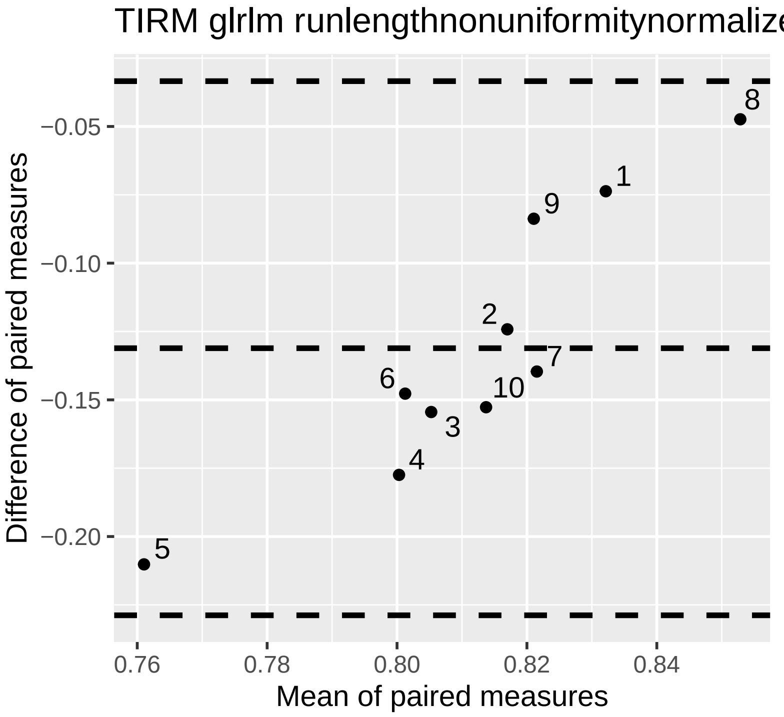
TIRM glrlm runlengthnonuniformitynormalize
Difference of paired measures
Mean of paired measures
−0.05
−0.10
−0.15
−0.20
0.76
0.78
0.80
0.82
0.84

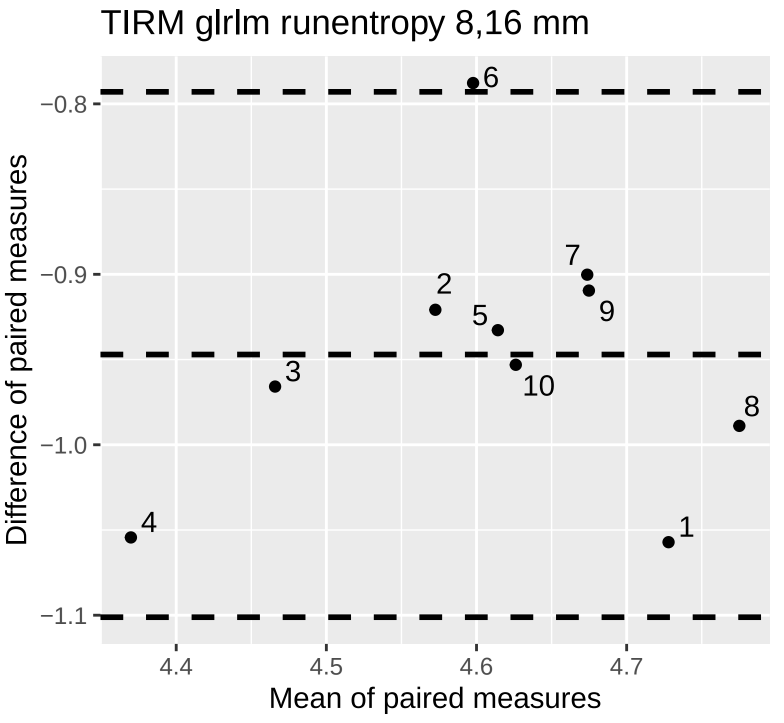
TIRM glrlm runentropy 8,16 mm
Difference of paired measures
Mean of paired measures
−0.8
−0.9
−1.0
−1.1
4.4
4.5
4.6
4.7

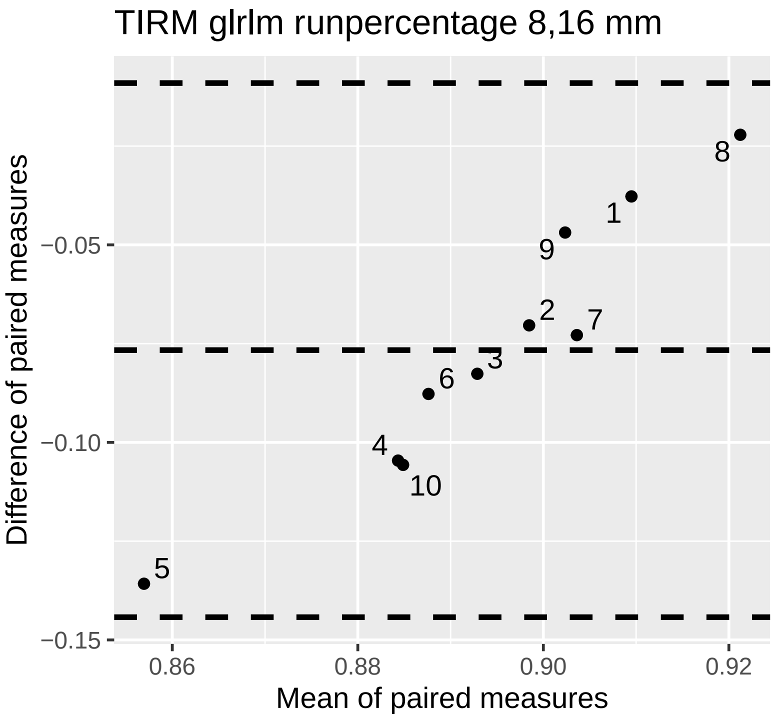
TIRM glrlm runpercentage 8,16 mm
Difference of paired measures
Mean of paired measures
−0.05
−0.10
−0.15
0.86
0.88
0.90
0.92

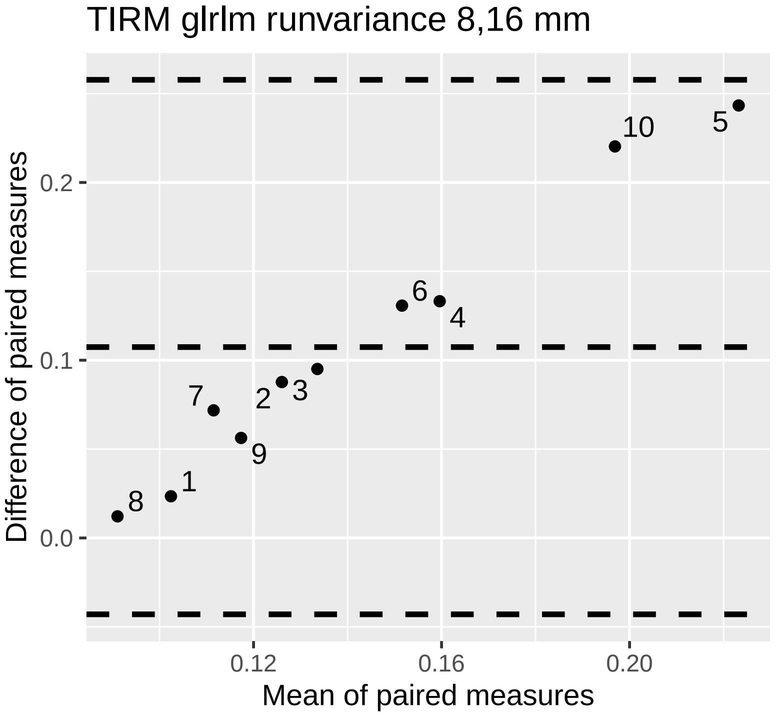
TIRM glrlm runvariance 8,16 mm
Difference of paired measures
Mean of paired measures
0.0
0.1
0.2
0.12
0.16
0.20

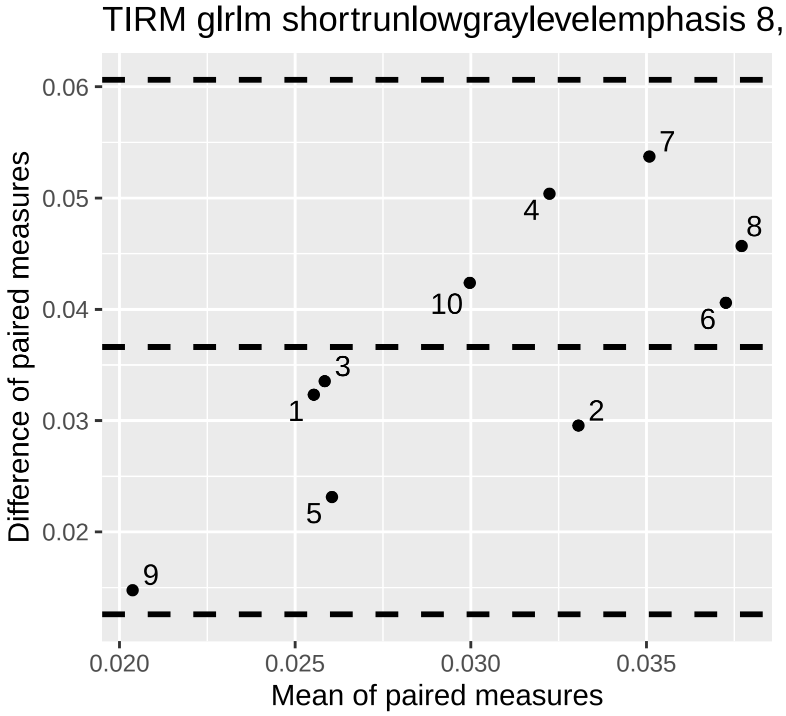
TIRM glrlm shortrunlowgraylevelemphasis 8,
Difference of paired measures
Mean of paired measures
0.02
0.03
0.04
0.05
0.06
0.020
0.025
0.030
0.035

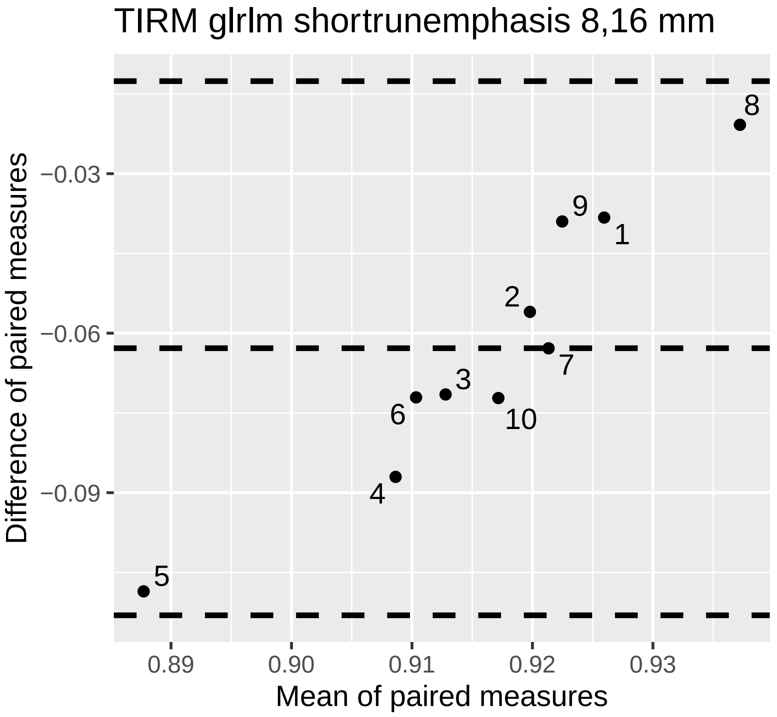
TIRM glrlm shortrunemphasis 8,16 mm
Difference of paired measures
Mean of paired measures
−0.03
−0.06
−0.09
0.89
0.90
0.91
0.92
0.93

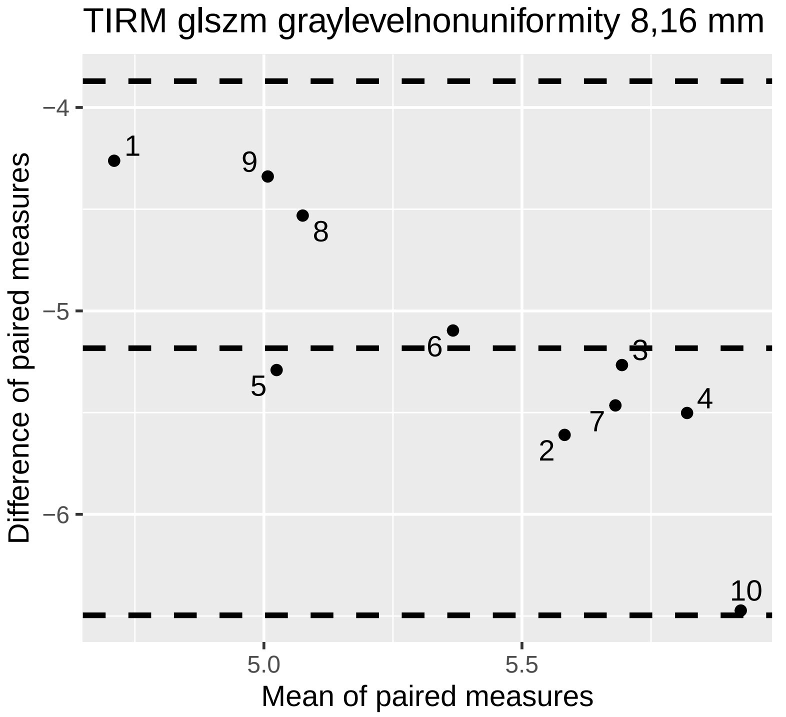
TIRM glszm graylevelnonuniformity 8,16 mm
Difference of paired measures
Mean of paired measures
−4
−5
−6
5.0
5.5

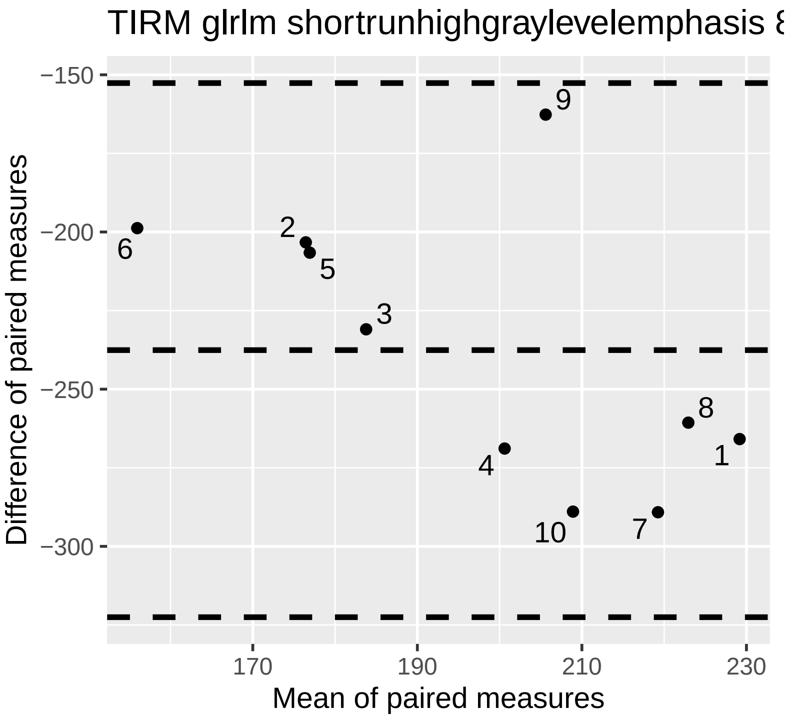
TIRM glrlm shortrunhighgraylevelemphasis 8
Difference of paired measures
Mean of paired measures
−150
−200
−250
−300
170
190
210
230

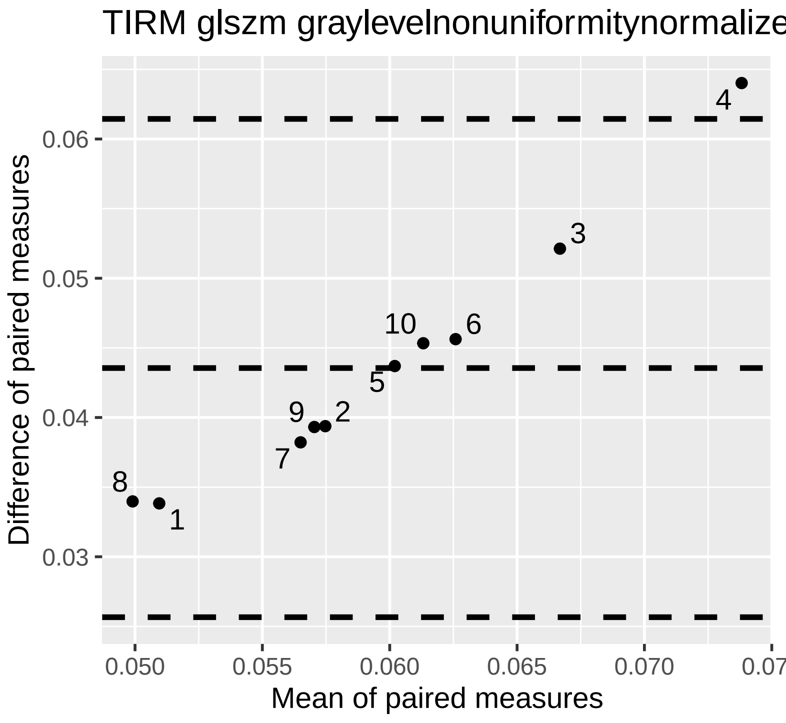
TIRM glszm graylevelnonuniformitynormalize
Difference of paired measures
Mean of paired measures
0.03
0.04
0.05
0.06
0.050
0.055
0.060
0.065
0.070
0.07

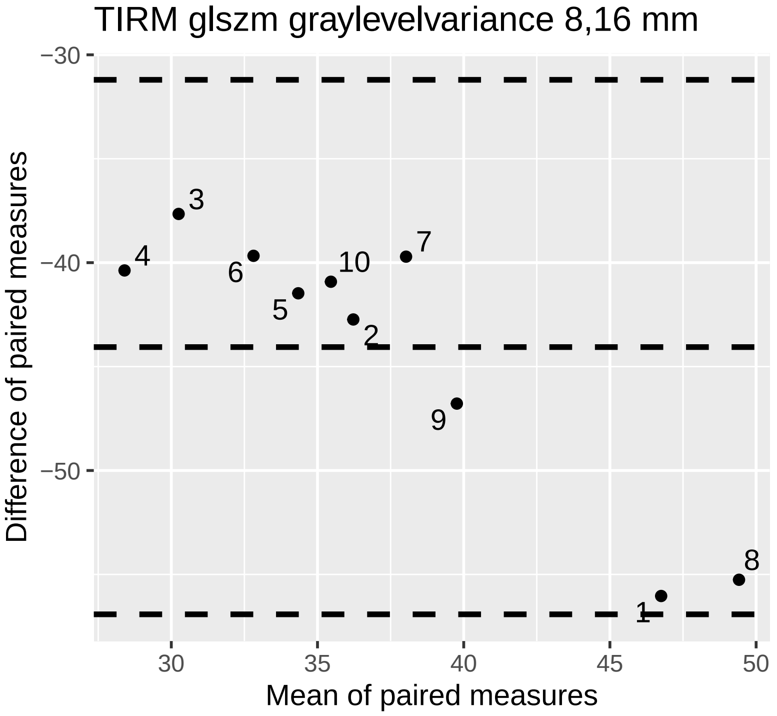
TIRM glszm graylevelvariance 8,16 mm
Difference of paired measures
Mean of paired measures
−30
−40
−50
30
35
40
45
50
1
2
3
4
5
6
7
8
9
10

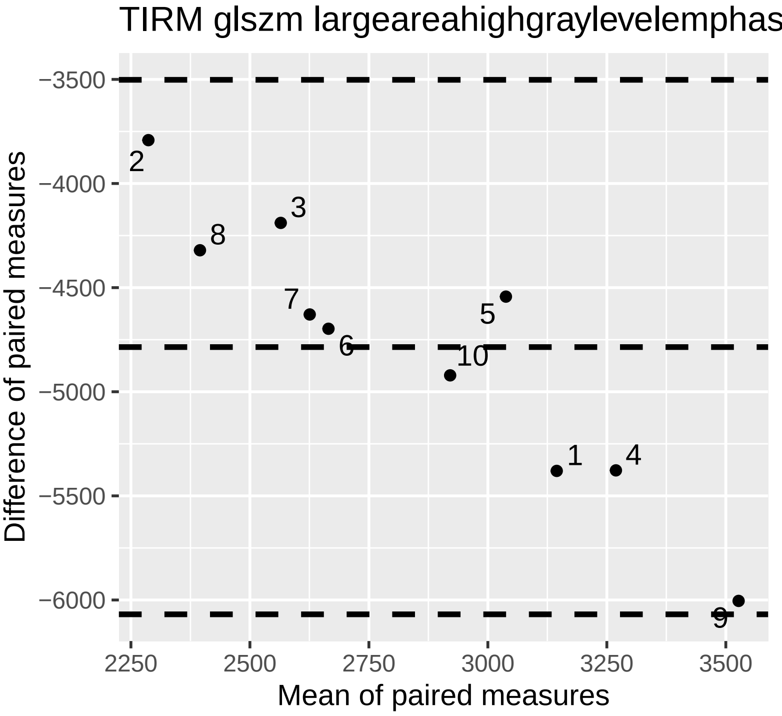
TIRM glszm largeareahighgraylevelemphas
Difference of paired measures
Mean of paired measures
−3500
−4000
−4500
−5000
−5500
−6000
2250
2500
2750
3000
3250
3500
1
2
3
4
5
6
7
8
9
10

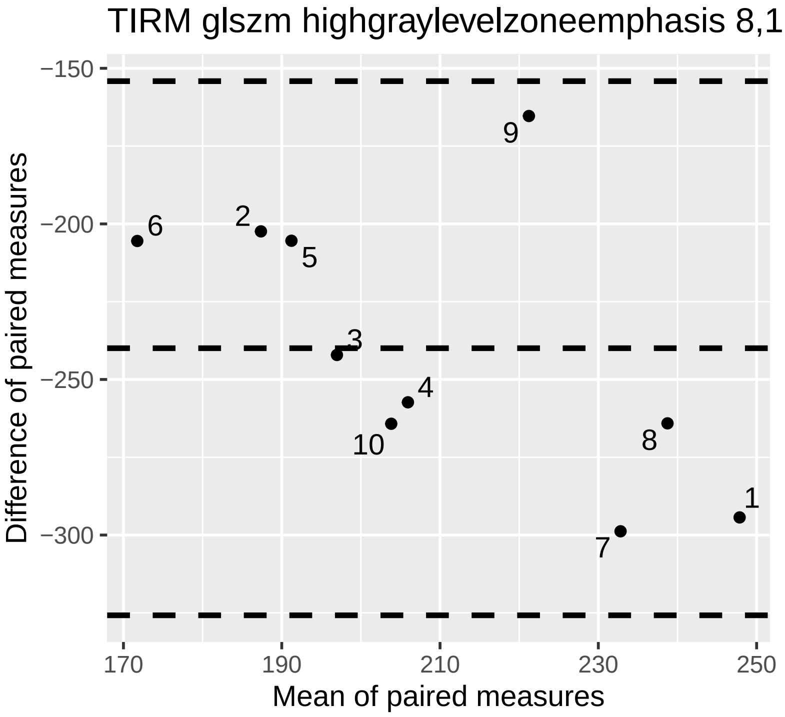
TIRM glszm highgraylevelzoneemphasis 8,1
Difference of paired measures
Mean of paired measures
−150
−200
−250
−300
170
190
210
230
250
1
2
3
4
5
6
7
8
9
10

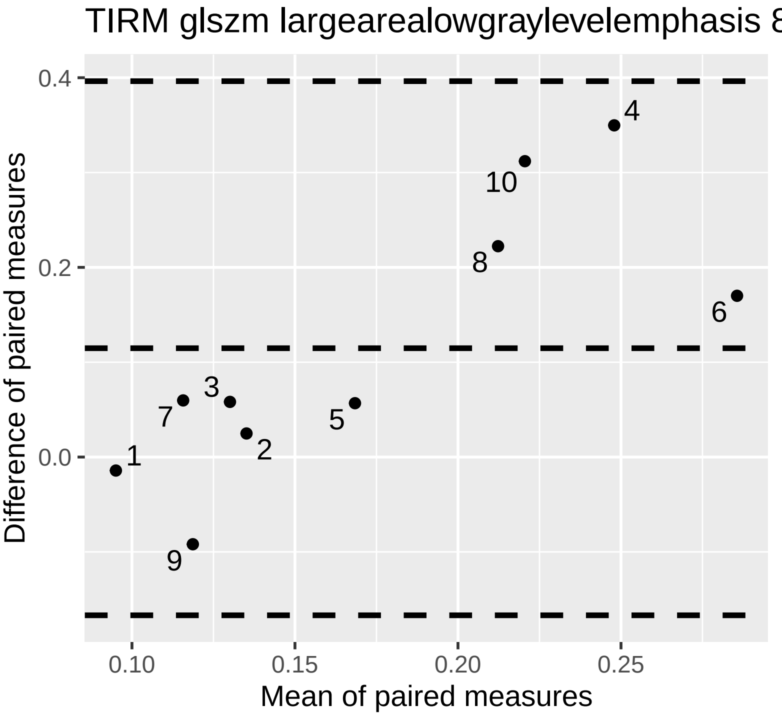
TIRM glszm largearealowgraylevelemphasis 8
Difference of paired measures
Mean of paired measures
0.4
0.2
0.0
0.10
0.15
0.20
0.25
1
2
3
4
5
6
7
8
9
10

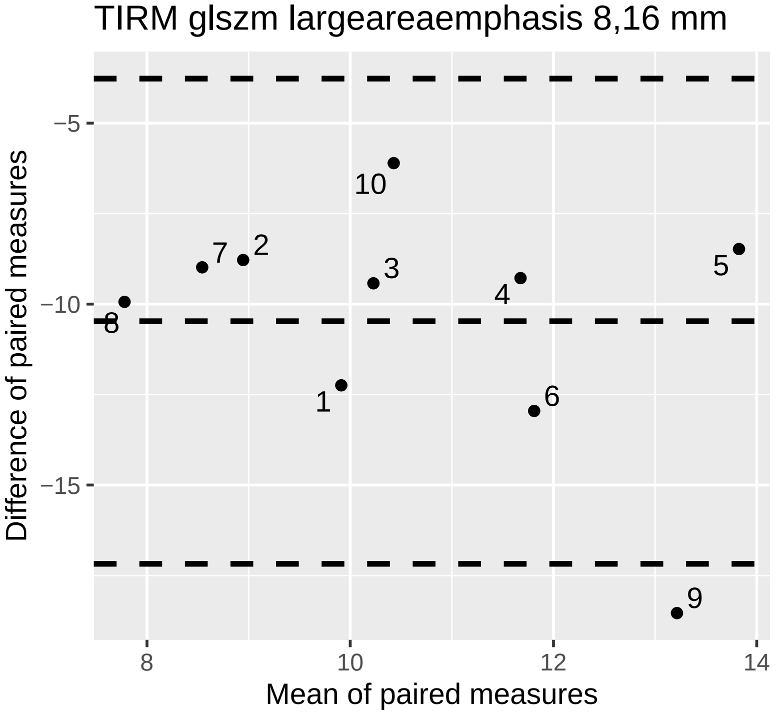
TIRM glszm largeareaemphasis 8,16 mm
Difference of paired measures
Mean of paired measures
−5
−10
−15
8
10
12
14
1
2
3
4
5
6
7
8
9
10

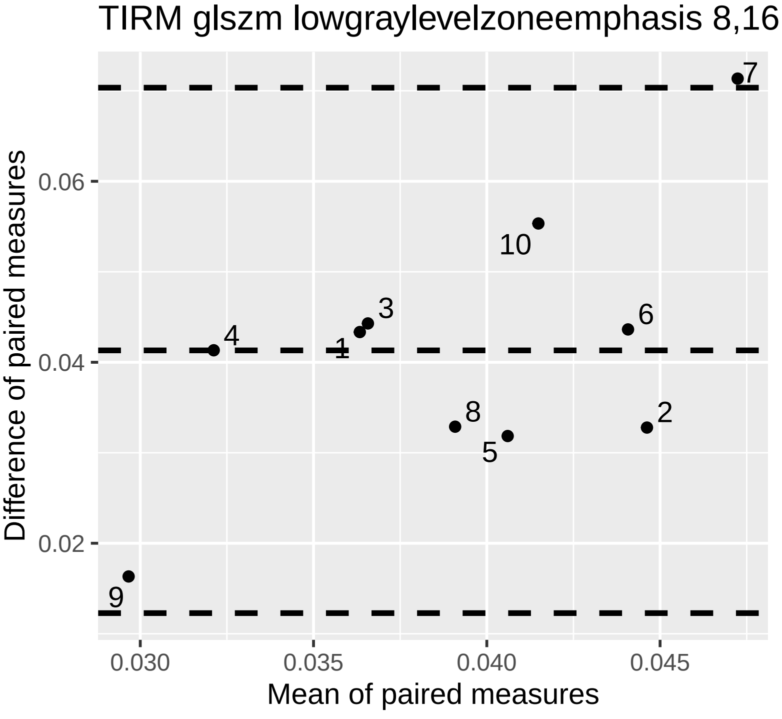
TIRM glszm lowgraylevelzoneemphasis 8,16
Difference of paired measures
Mean of paired measures
0.06
0.04
0.02
0.030
0.035
0.040
0.045
1
2
3
4
5
6
7
8
9
10

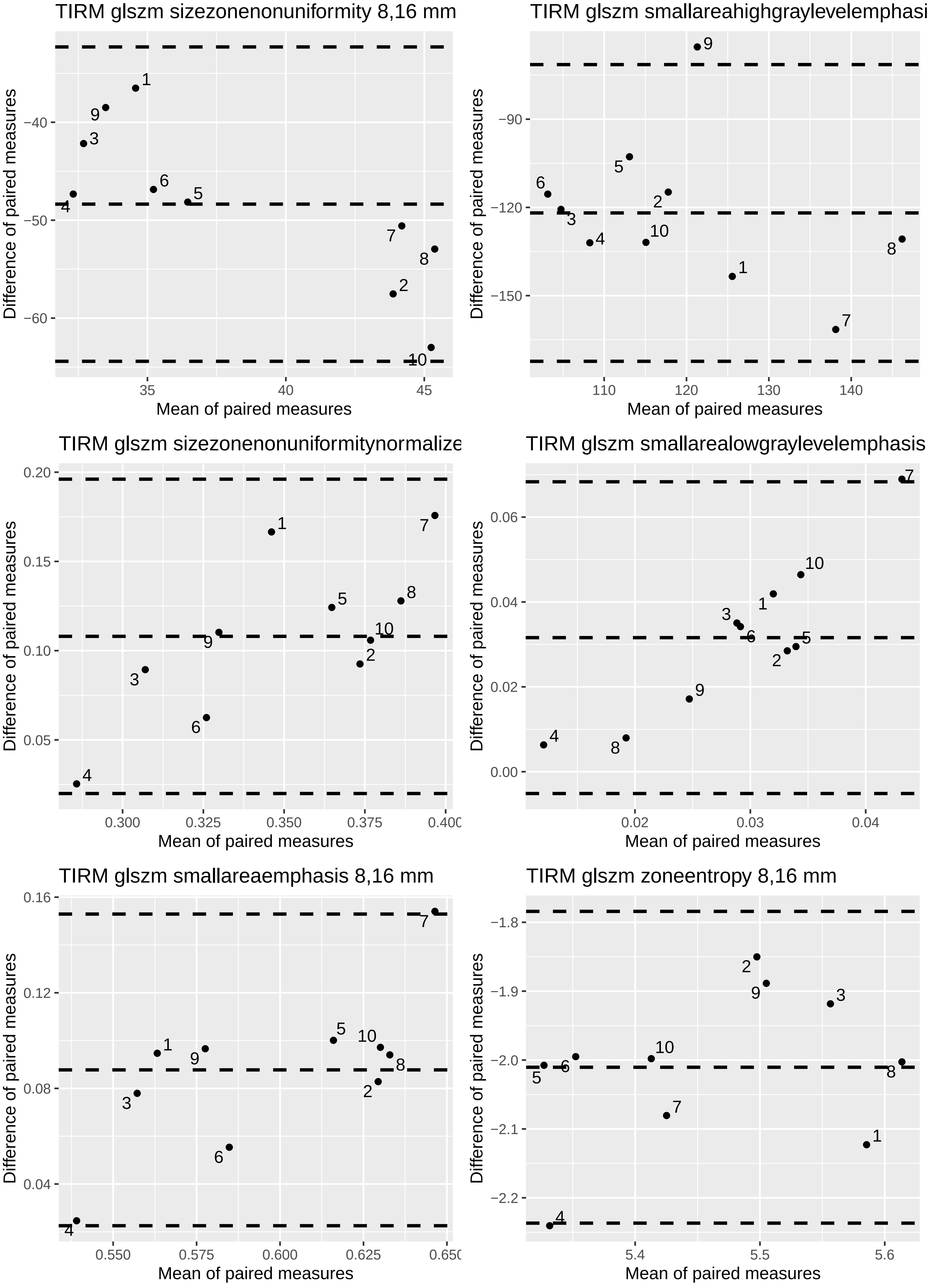
TIRM glszm sizezonenonuniformity 8,16 mm
TIRM glszm smallareahighgraylevelemphasi
TIRM glszm sizezonenonuniformitynormalize
TIRM glszm smallarealowgraylevelemphasis
TIRM glszm smallareaemphasis 8,16 mm
TIRM glszm zoneentropy 8,16 mm
Difference of paired measures
Mean of paired measures

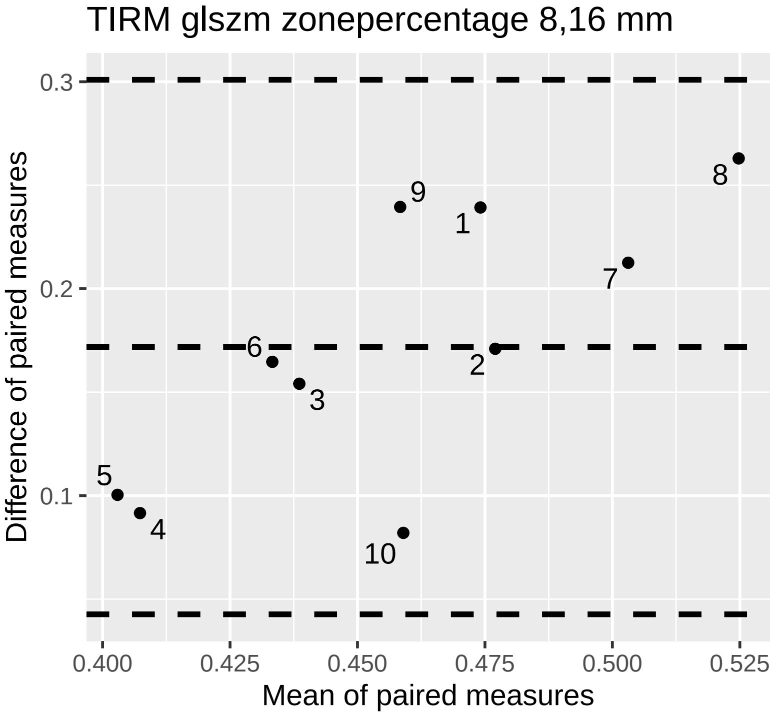
TIRM glszm zonepercentage 8,16 mm
Difference of paired measures
Mean of paired measures

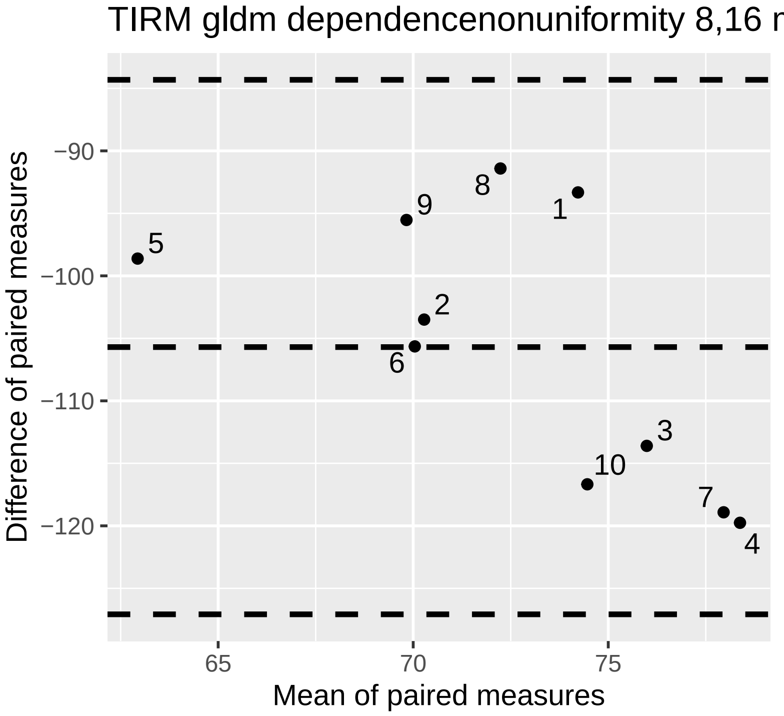
TIRM gldm dependencenonuniformity 8,16 m
Difference of paired measures
Mean of paired measures

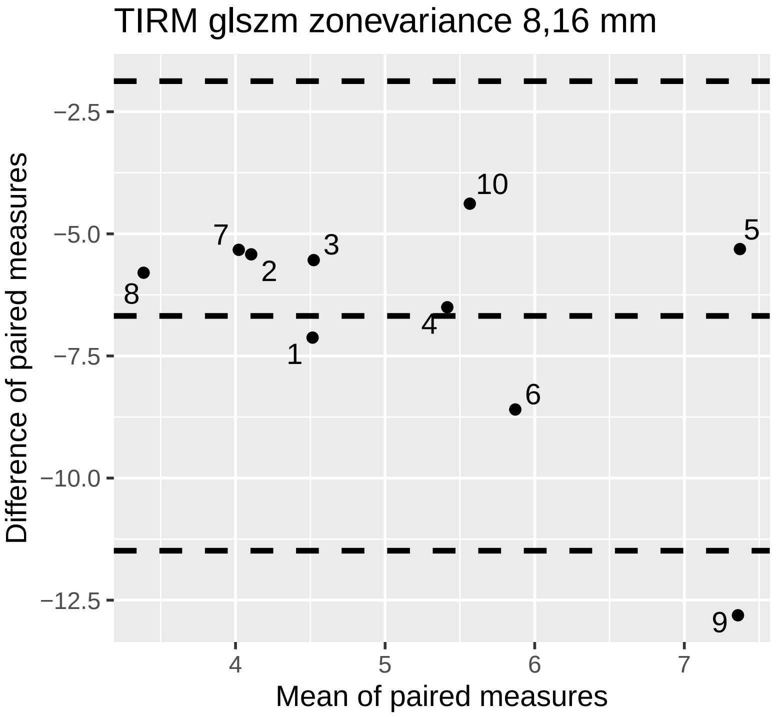
TIRM glszm zonevariance 8,16 mm
Difference of paired measures
Mean of paired measures

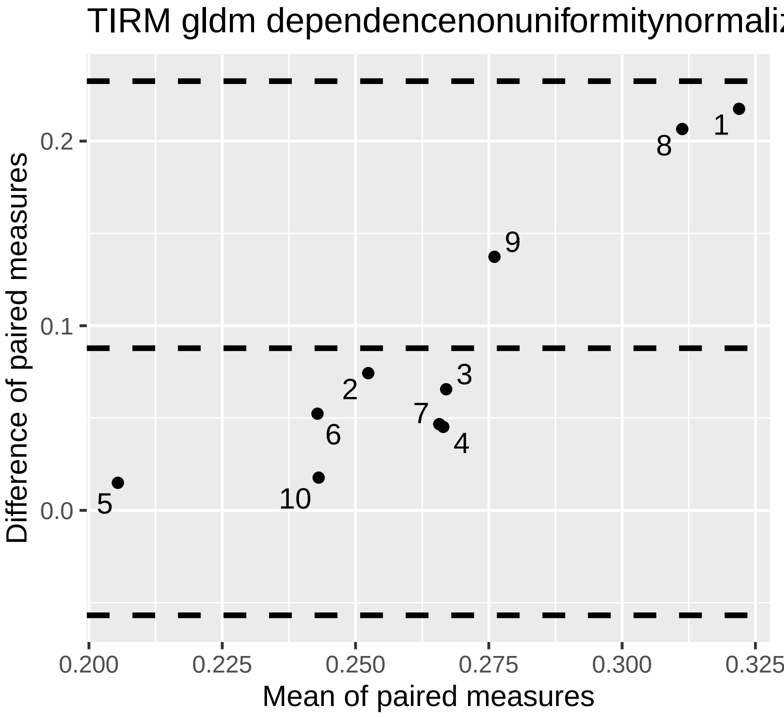
TIRM gldm dependencenonuniformitynormaliz
Difference of paired measures
Mean of paired measures

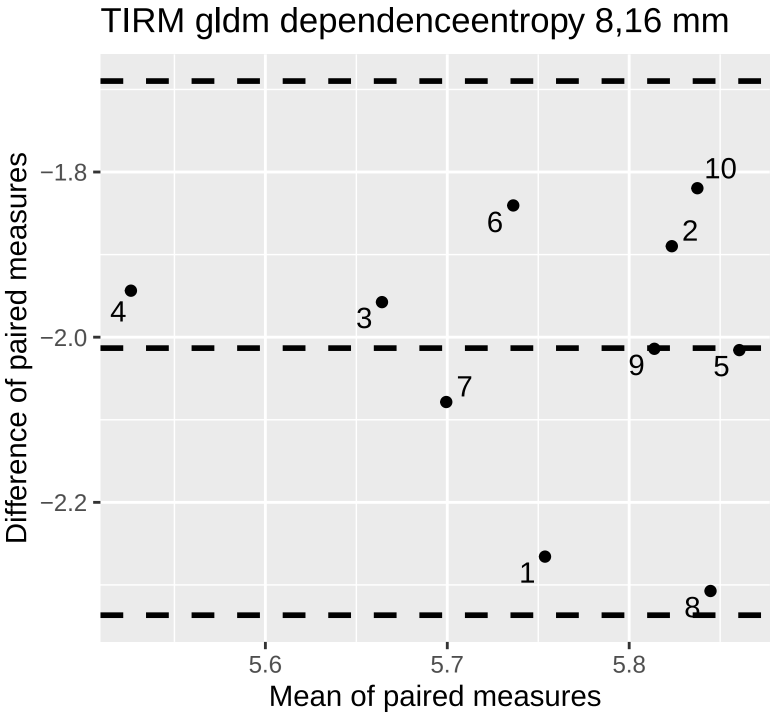
TIRM gldm dependenceentropy 8,16 mm
Difference of paired measures
Mean of paired measures

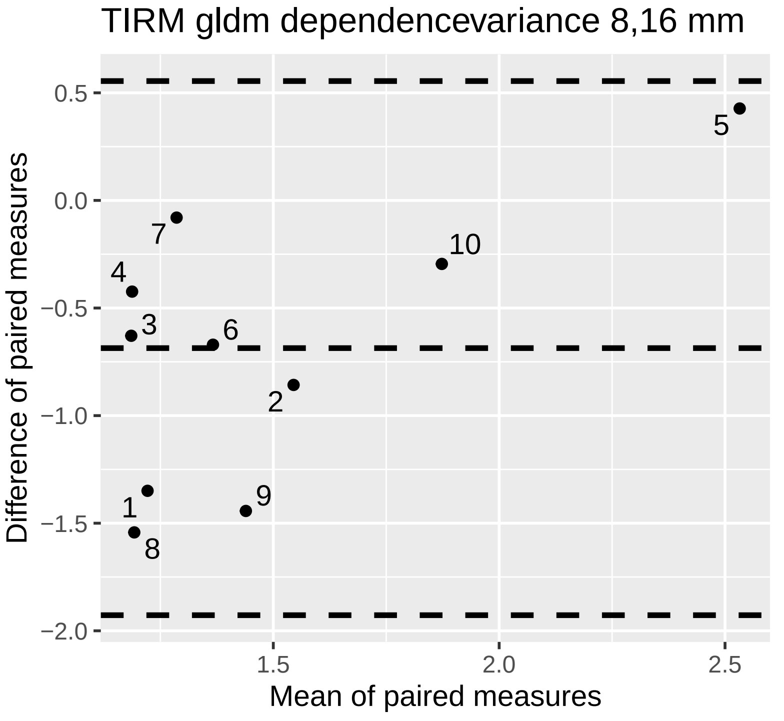
TIRM gldm dependencevariance 8,16 mm
Difference of paired measures
Mean of paired measures

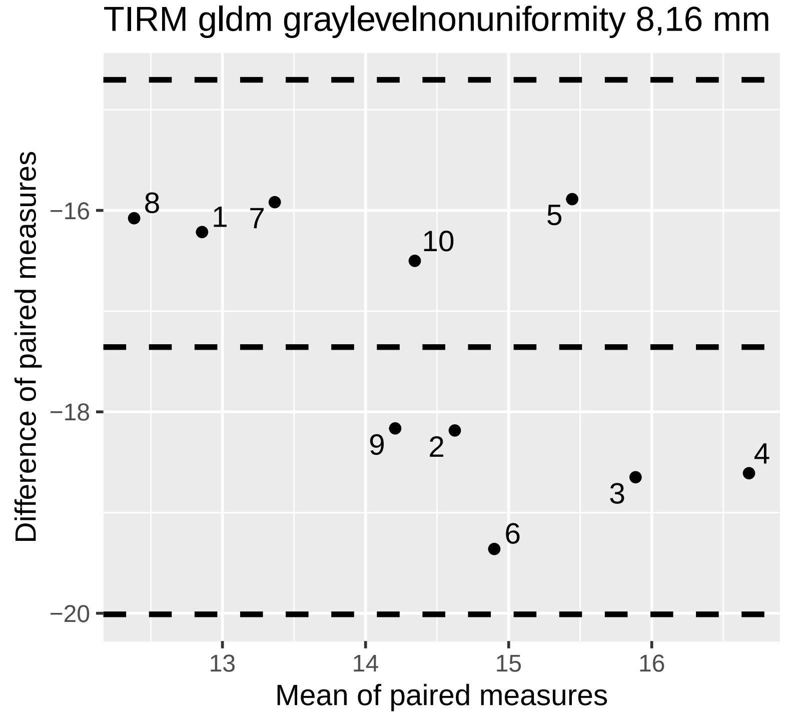
TIRM gldm graylevelnonuniformity 8,16 mm
Difference of paired measures
−16
−18
−20
Mean of paired measures
13
14
15
16

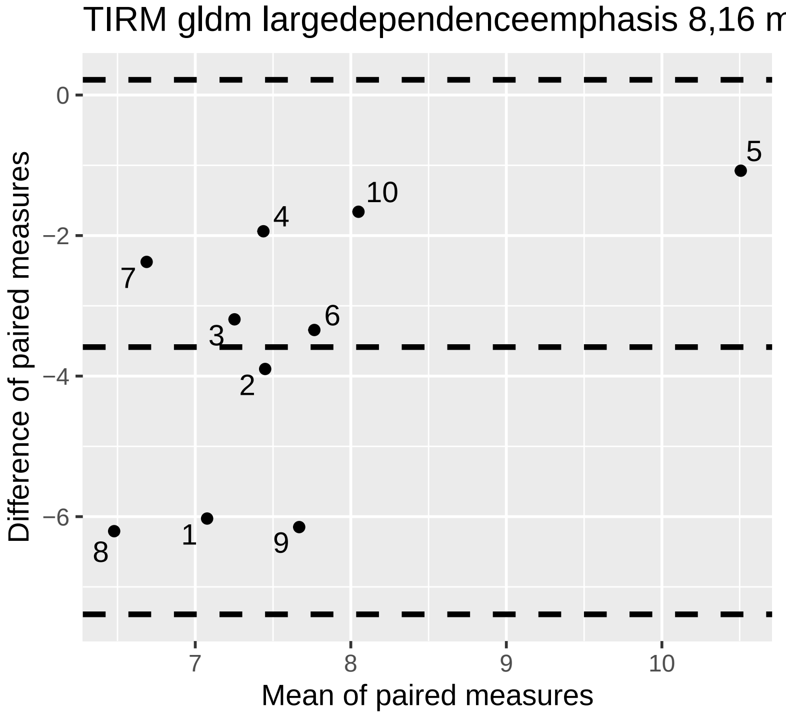
TIRM gldm largedependenceemphasis 8,16 m
Difference of paired measures
0
−2
−4
−6
Mean of paired measures
7
8
9
10

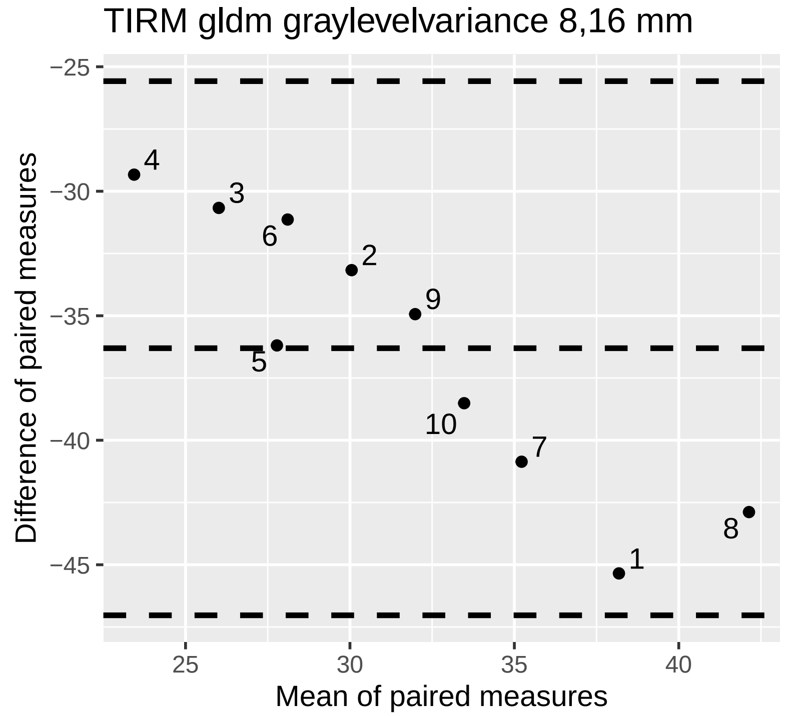
TIRM gldm graylevelvariance 8,16 mm
Difference of paired measures
−25
−30
−35
−40
−45
Mean of paired measures
25
30
35
40

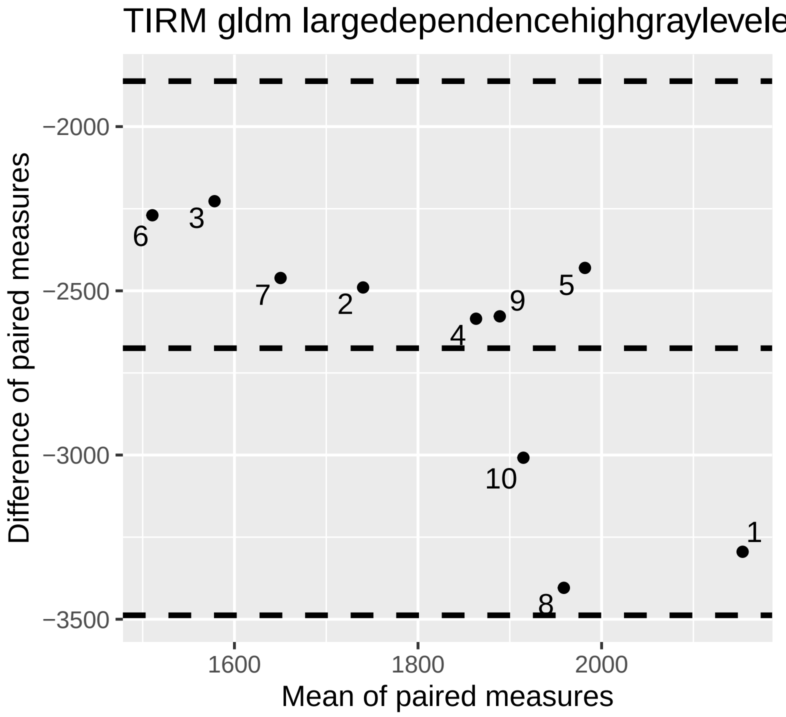
TIRM gldm largedependencehighgraylevele
Difference of paired measures
−2000
−2500
−3000
−3500
Mean of paired measures
1600
1800
2000

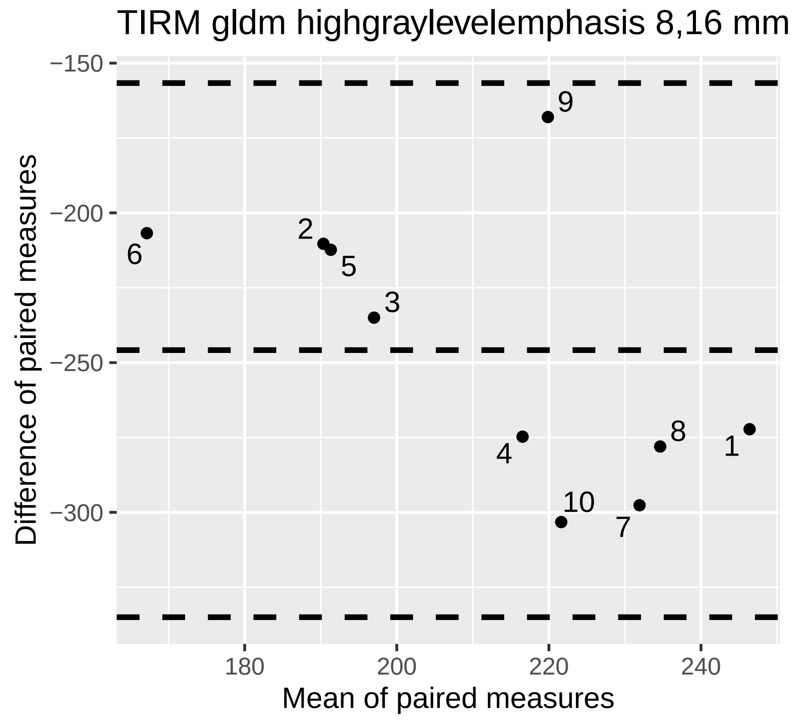
TIRM gldm highgraylevelemphasis 8,16 mm
Difference of paired measures
−150
−200
−250
−300
Mean of paired measures
180
200
220
240

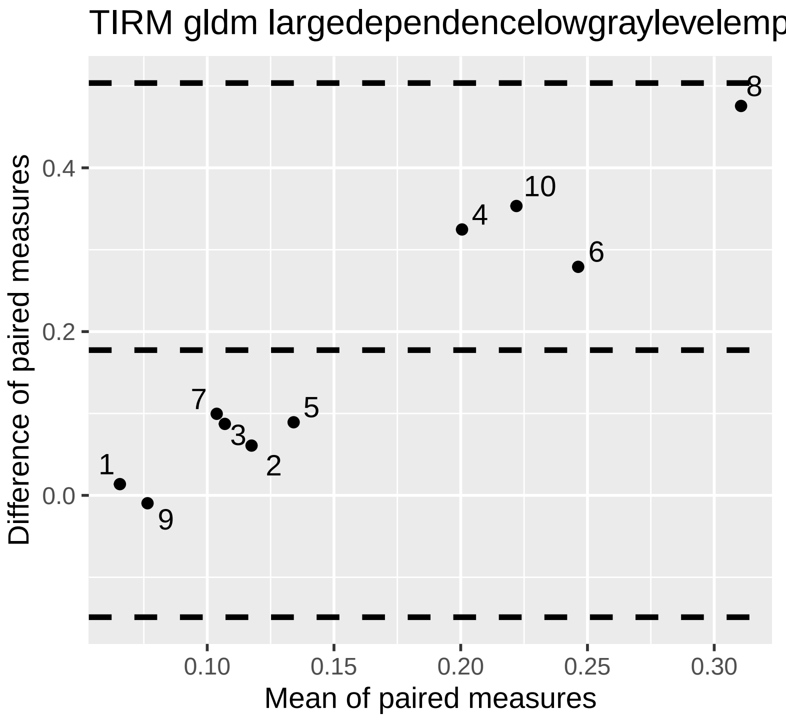
TIRM gldm largedependencelowgraylevelemp
Difference of paired measures
0.4
0.2
0.0
Mean of paired measures
0.10
0.15
0.20
0.25
0.30

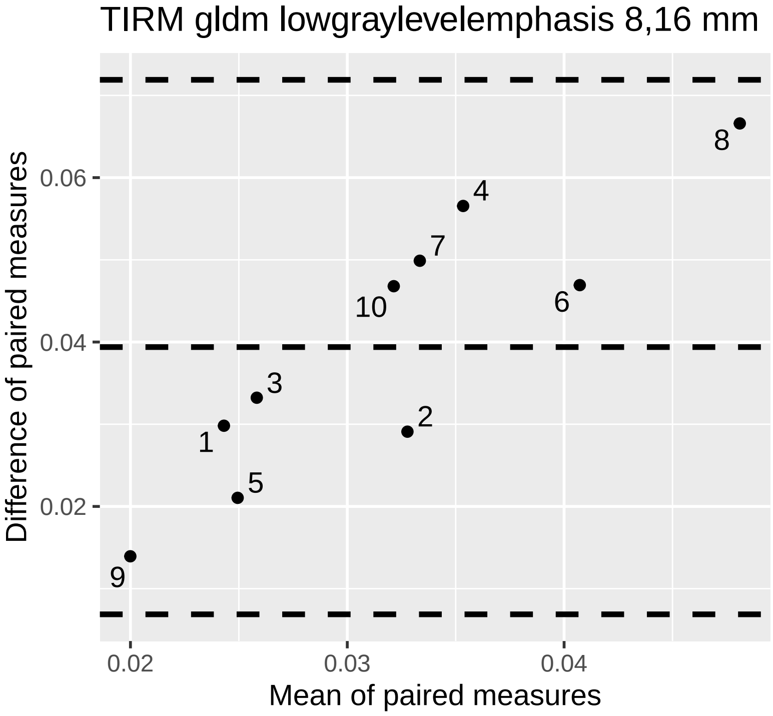
TIRM gldm lowgraylevelemphasis 8,16 mm
Difference of paired measures
Mean of paired measures
0.02
0.04
0.06
0.02
0.03
0.04
1
2
3
4
5
6
7
8
9
10

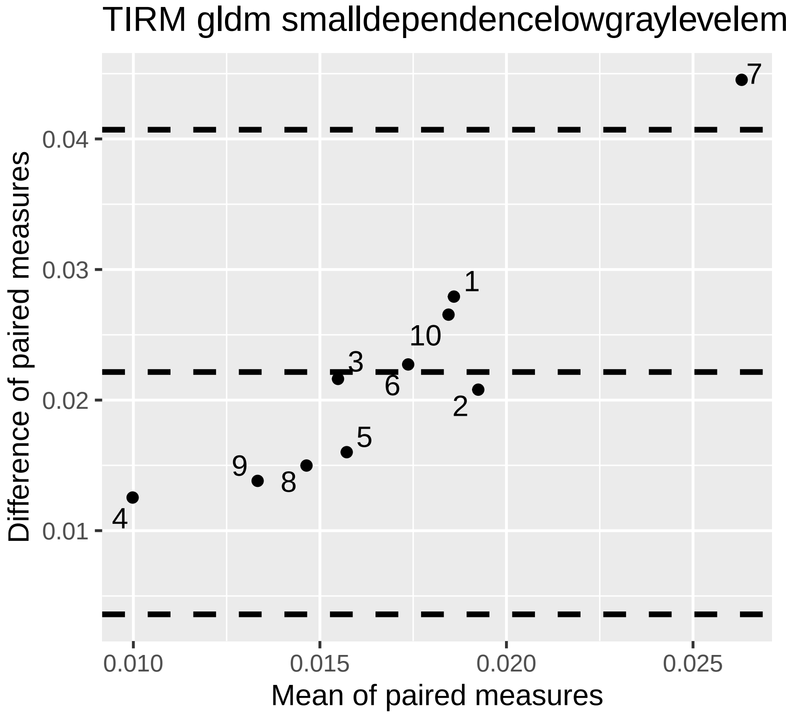
TIRM gldm smalldependencelowgraylevelem
Difference of paired measures
Mean of paired measures
0.01
0.02
0.03
0.04
0.010
0.015
0.020
0.025
1
2
3
4
5
6
7
8
9
10

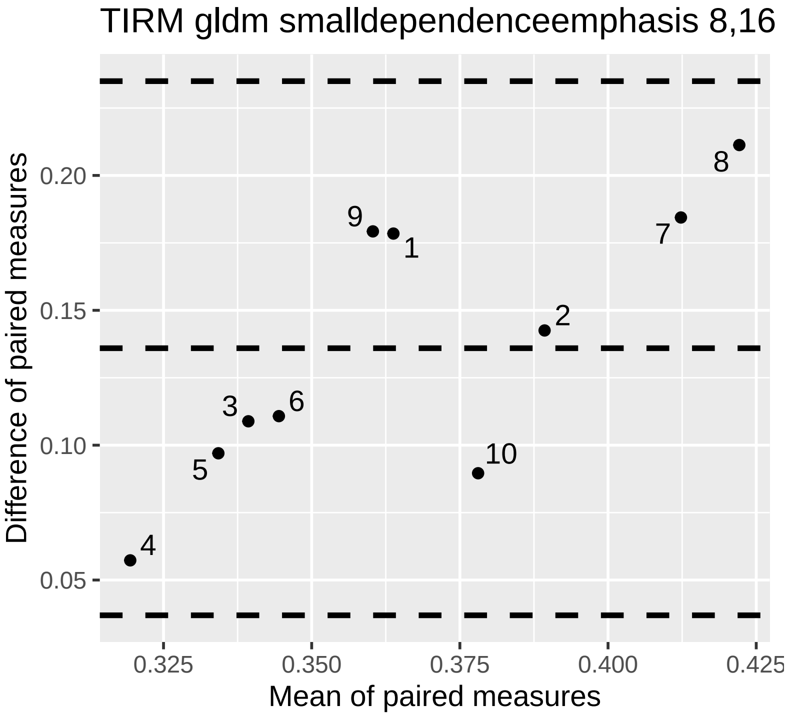
TIRM gldm smalldependenceemphasis 8,16
Difference of paired measures
Mean of paired measures
0.05
0.10
0.15
0.20
0.325
0.350
0.375
0.400
0.425
1
2
3
4
5
6
7
8
9
10

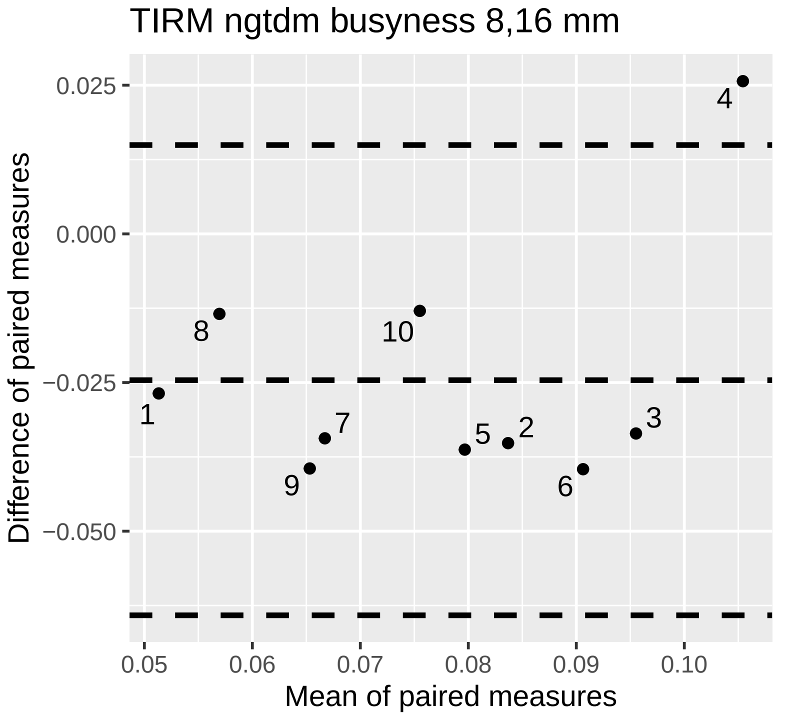
TIRM ngtdm busyness 8,16 mm
Difference of paired measures
Mean of paired measures
0.025
0.000
−0.025
−0.050
0.05
0.06
0.07
0.08
0.09
0.10
1
2
3
4
5
6
7
8
9
10

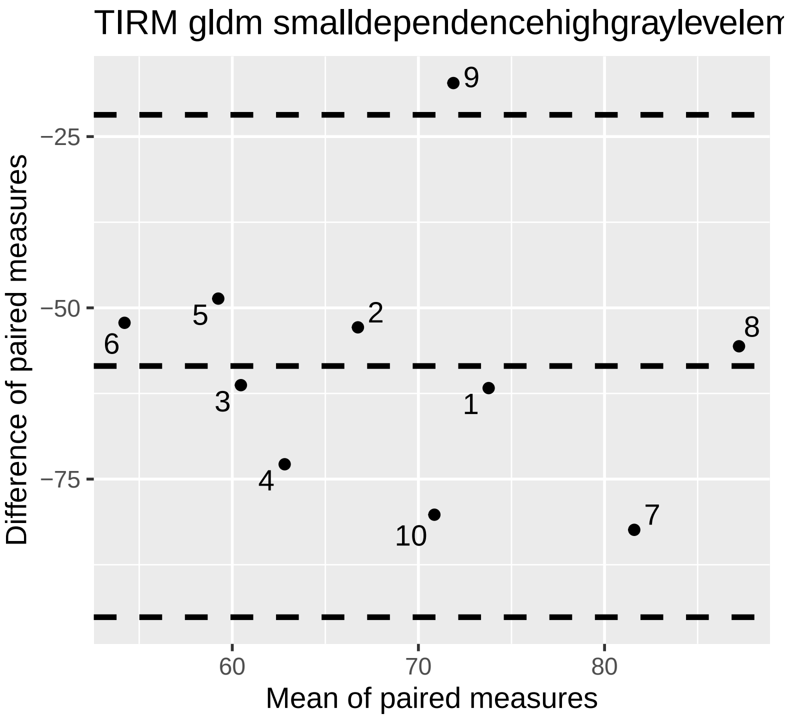
TIRM gldm smalldependencehighgraylevelem
Difference of paired measures
Mean of paired measures
−25
−50
−75
60
70
80
1
2
3
4
5
6
7
8
9
10

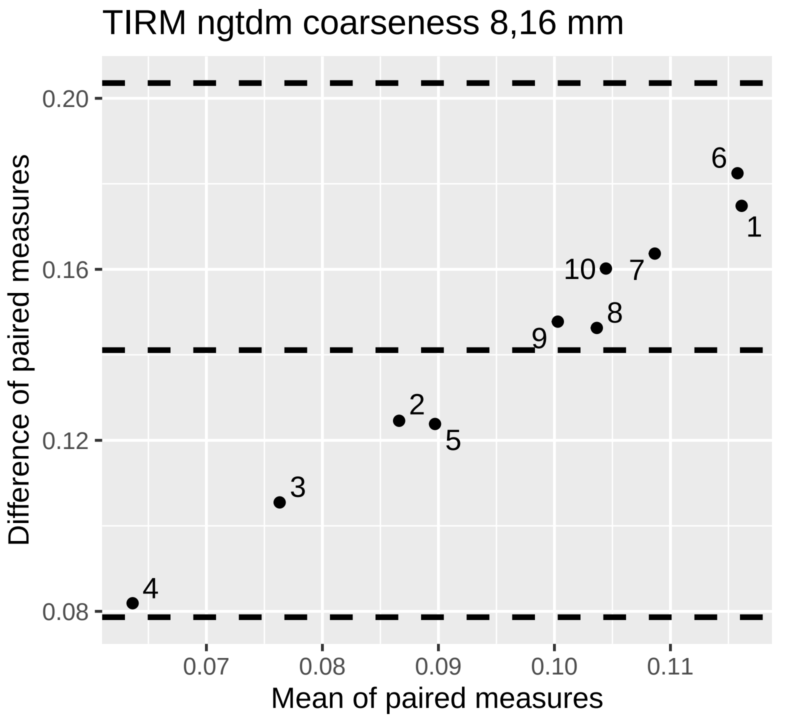
TIRM ngtdm coarseness 8,16 mm
Difference of paired measures
Mean of paired measures
0.08
0.12
0.16
0.20
0.07
0.08
0.09
0.10
0.11
1
2
3
4
5
6
7
8
9
10

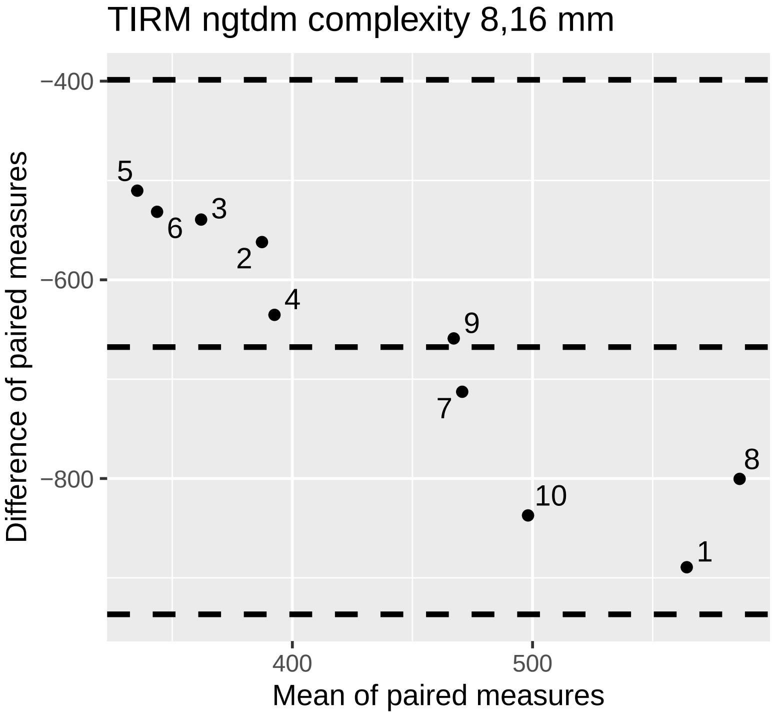
TIRM ngtdm complexity 8,16 mm
Difference of paired measures
Mean of paired measures
−400
−600
−800
400
500

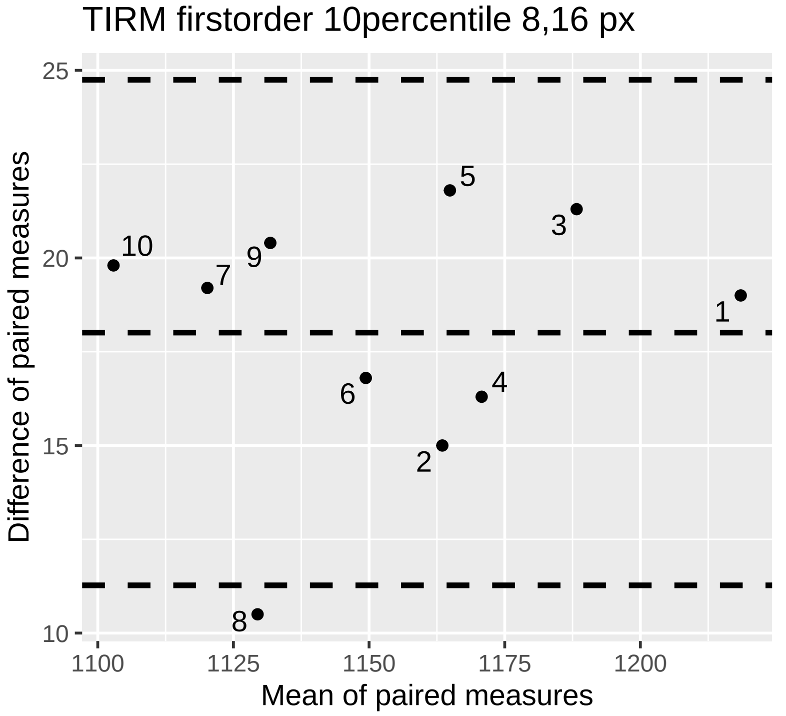
TIRM firstorder 10percentile 8,16 px
Difference of paired measures
Mean of paired measures
25
20
15
10
1100
1125
1150
1175
1200

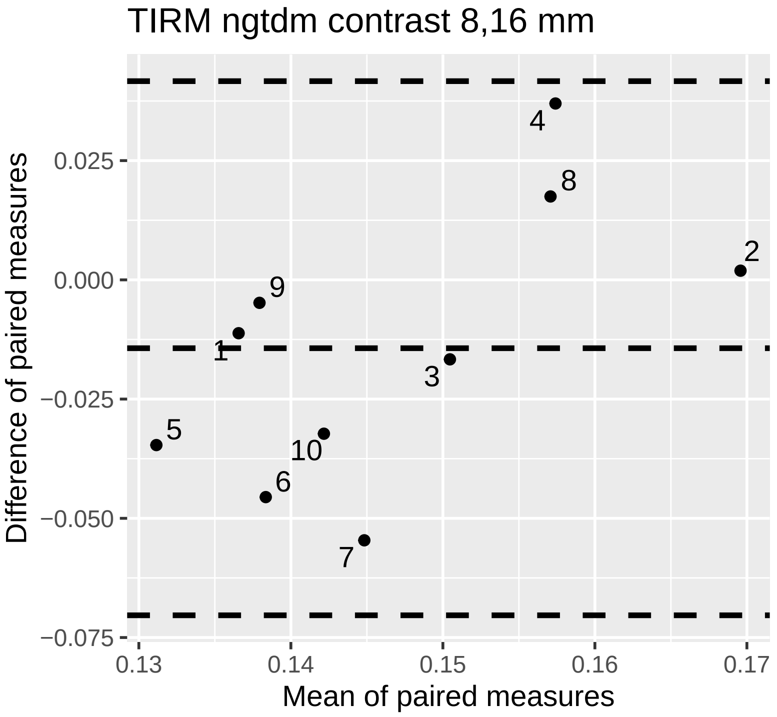
TIRM ngtdm contrast 8,16 mm
Difference of paired measures
Mean of paired measures
0.025
0.000
−0.025
−0.050
−0.075
0.13
0.14
0.15
0.16
0.17

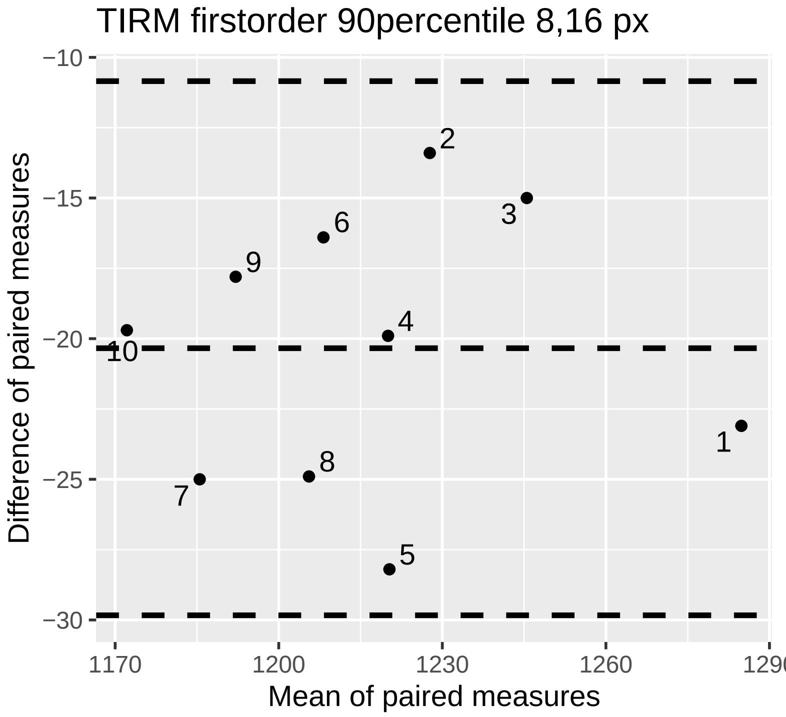
TIRM firstorder 90percentile 8,16 px
Difference of paired measures
Mean of paired measures
−10
−15
−20
−25
−30
1170
1200
1230
1260

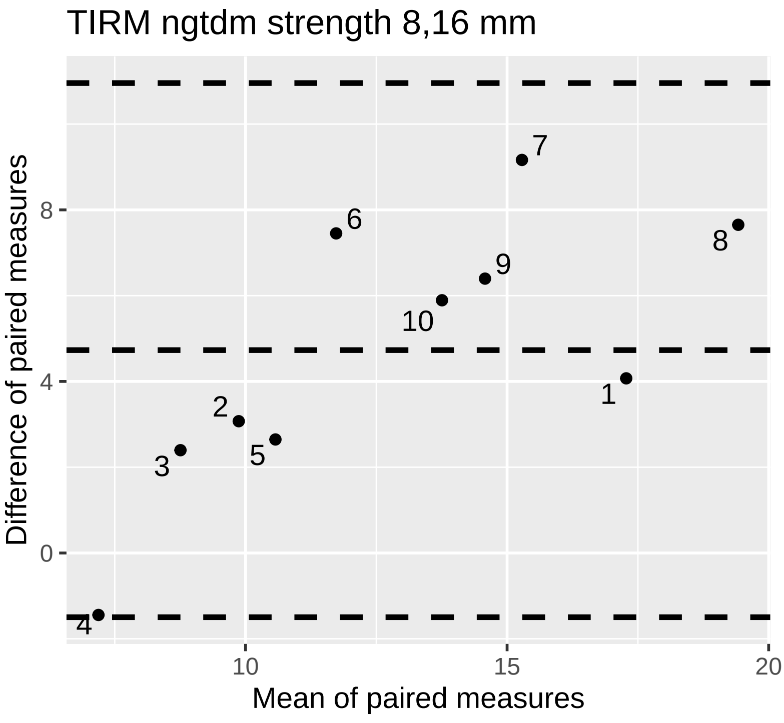
TIRM ngtdm strength 8,16 mm
Difference of paired measures
Mean of paired measures
8
4
0
10
15
20

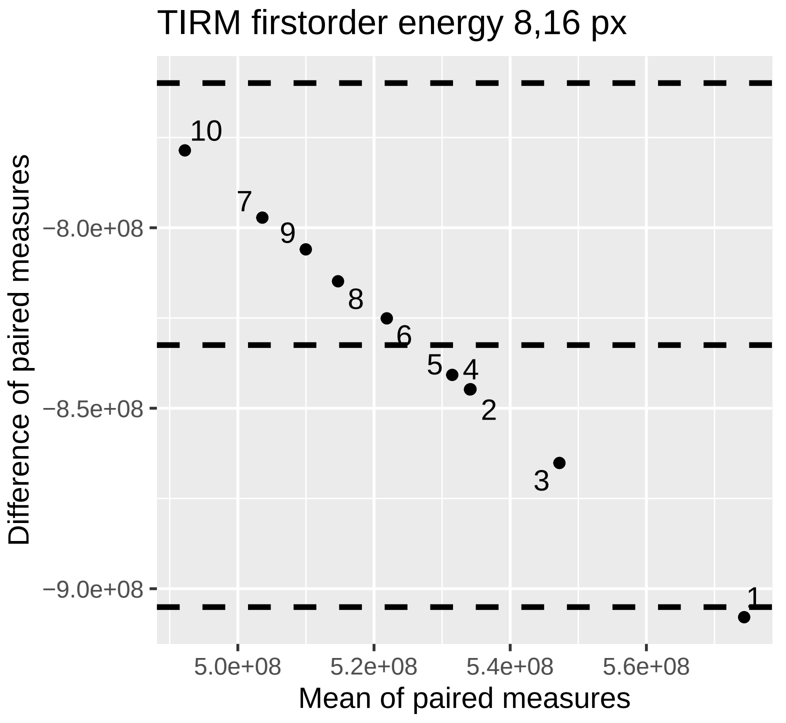
TIRM firstorder energy 8,16 px
Difference of paired measures
Mean of paired measures
−8.0e+08
−8.5e+08
−9.0e+08
5.0e+08
5.2e+08
5.4e+08
5.6e+08

TIRM firstorder entropy 8,16 px

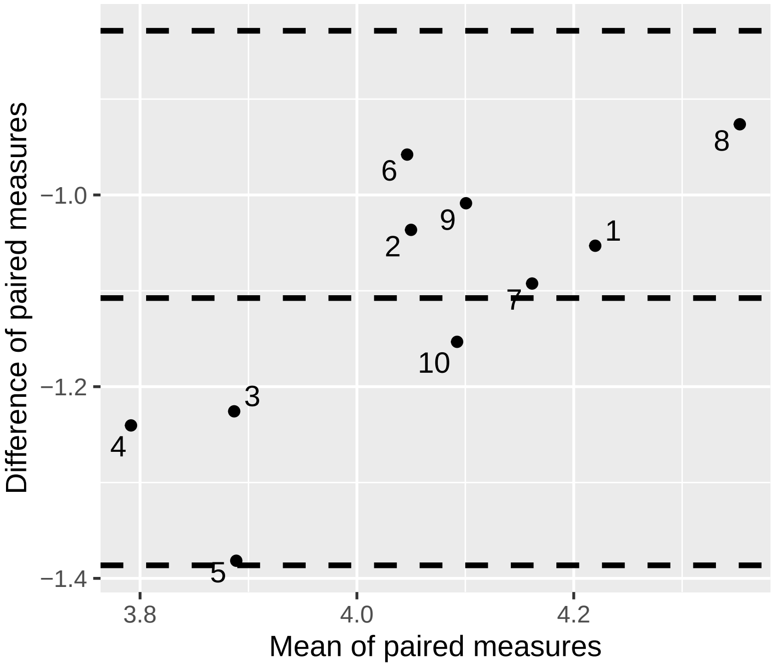


TIRM firstorder maximum 8,16 px

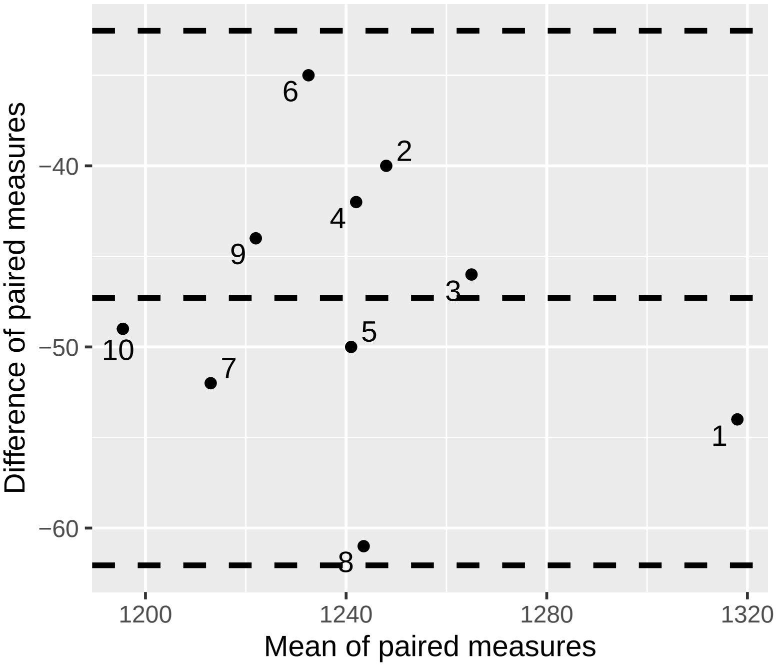


TIRM firstorder interquartilerange 8,16 px

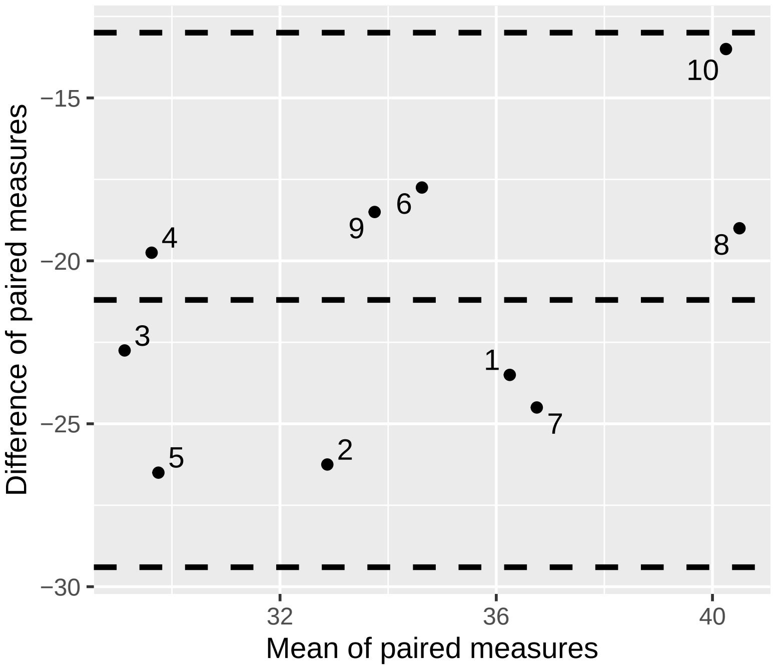


TIRM firstorder meanabsolutedeviation 8,16 p

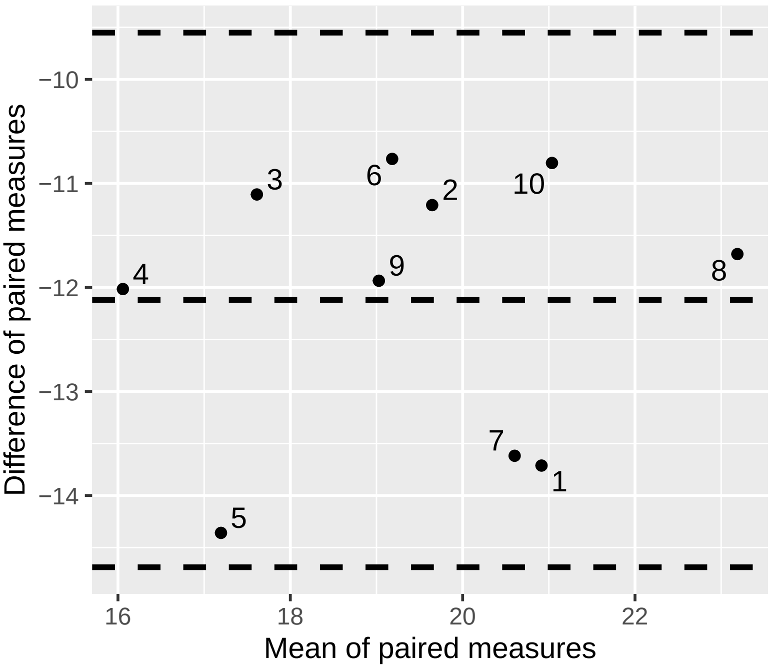


TIRM firstorder kurtosis 8,16 px

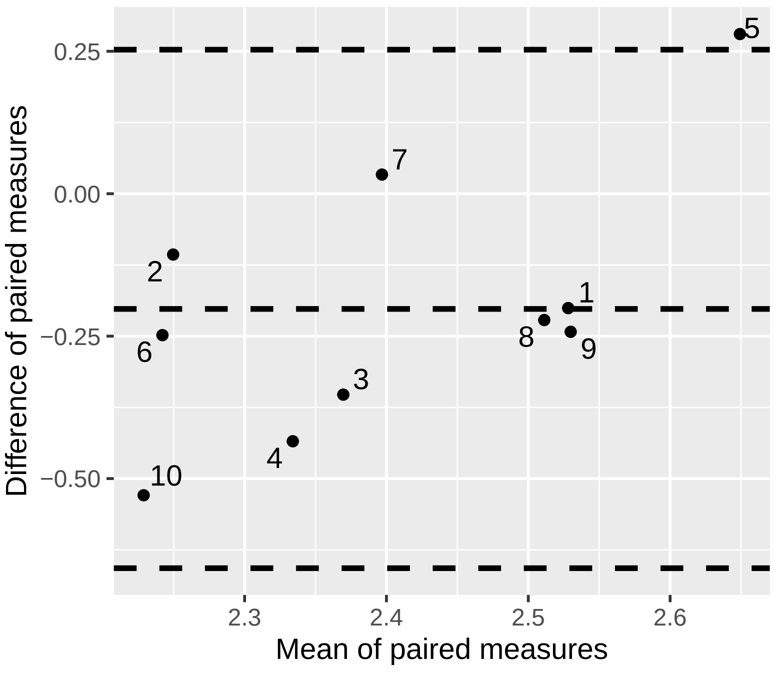


TIRM firstorder mean 8,16 px

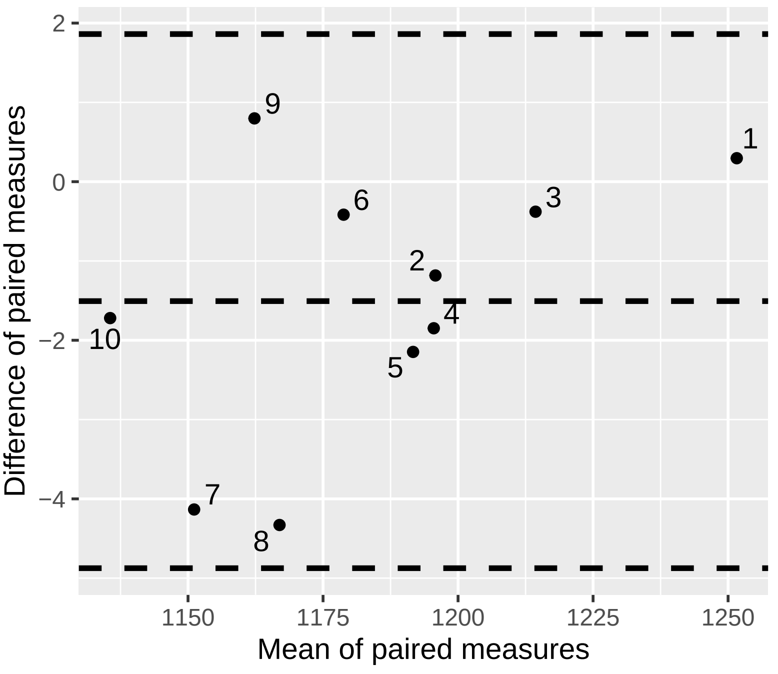

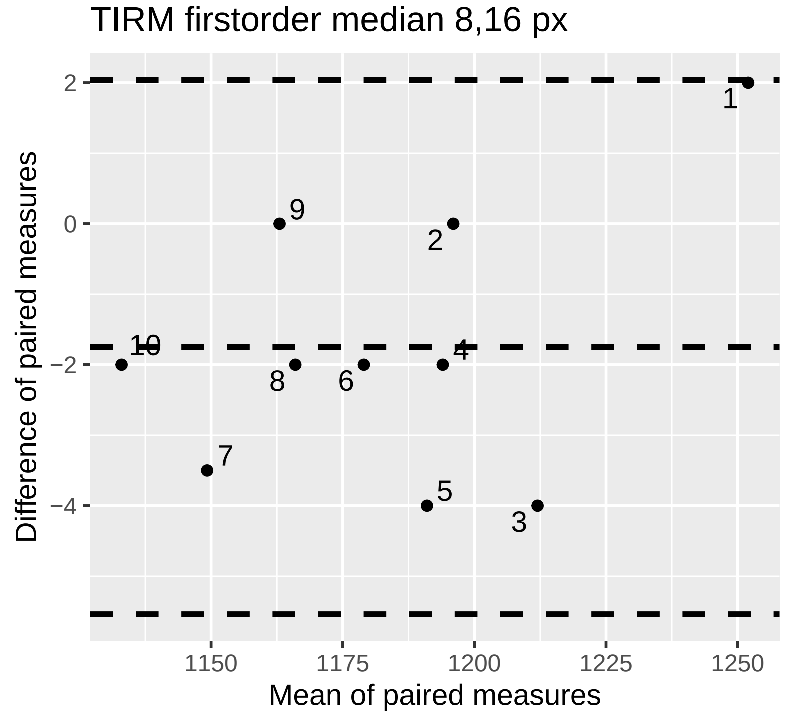
TIRM firstorder median 8,16 px
Difference of paired measures
Mean of paired measures

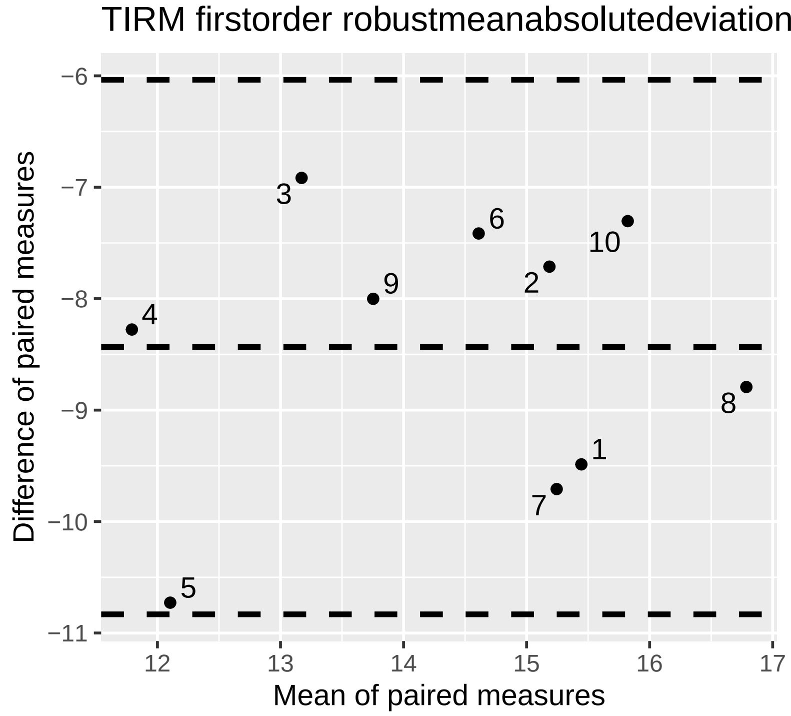
TIRM firstorder robustmeanabsolutedeviation
Difference of paired measures
Mean of paired measures

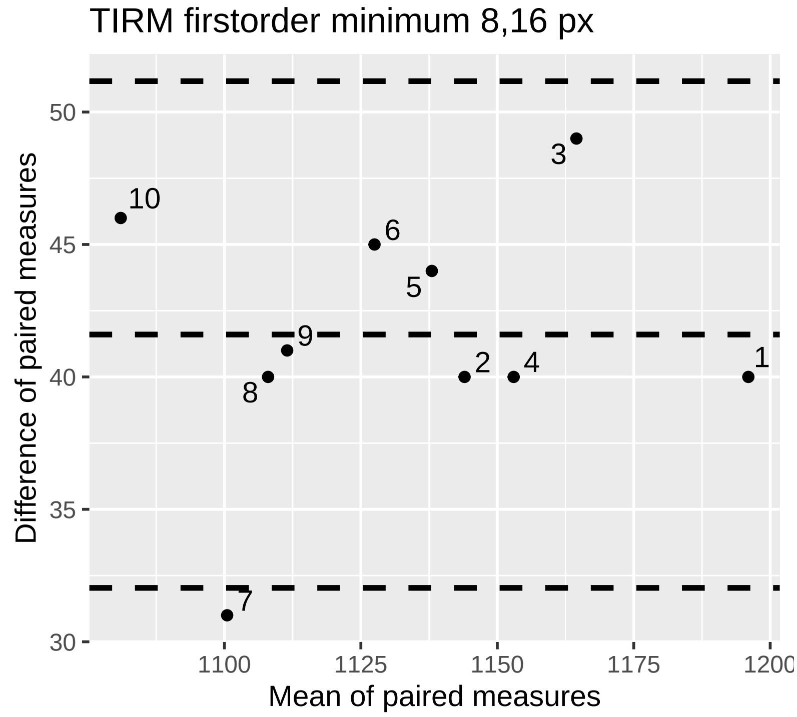
TIRM firstorder minimum 8,16 px
Difference of paired measures
Mean of paired measures

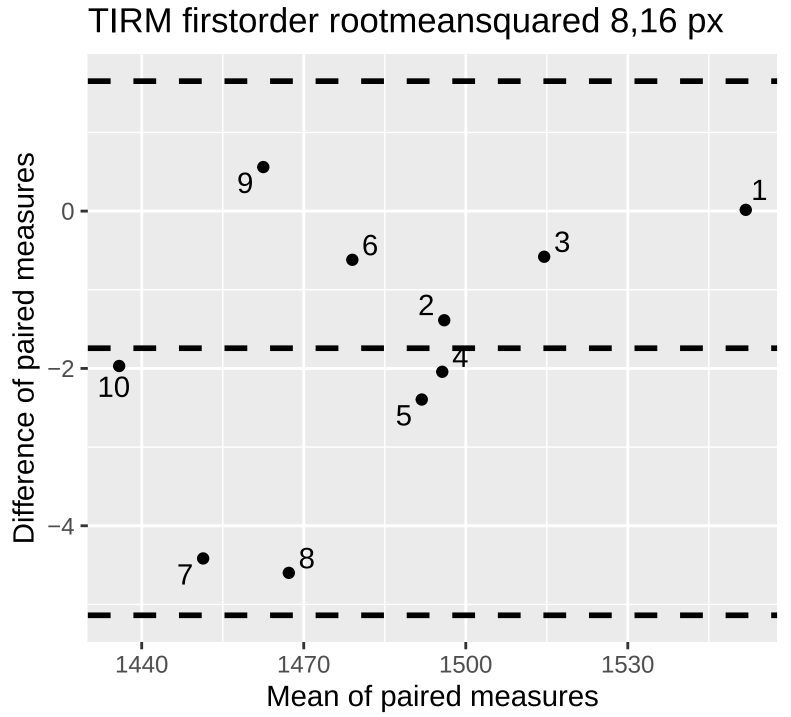
TIRM firstorder rootmeansquared 8,16 px
Difference of paired measures
Mean of paired measures

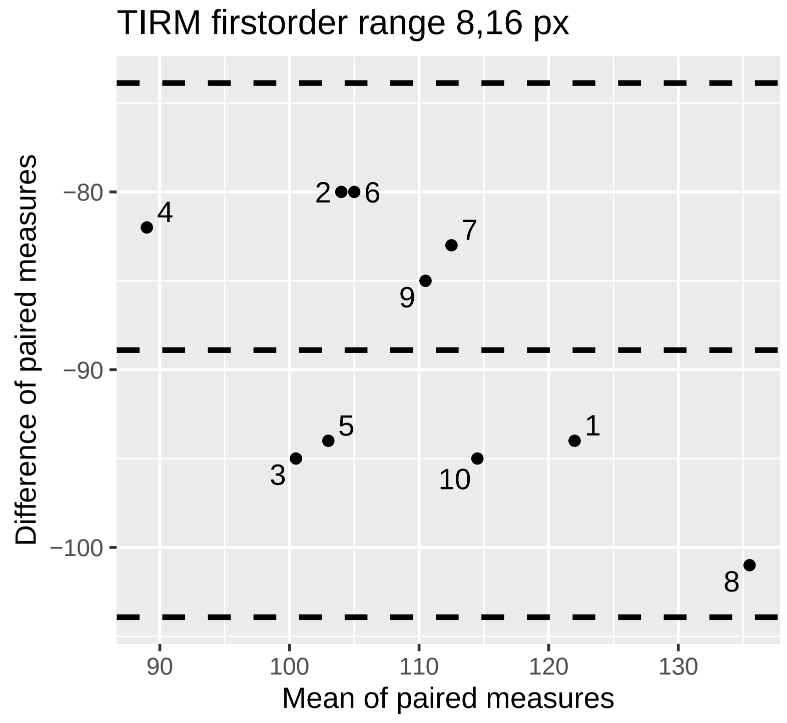
TIRM firstorder range 8,16 px
Difference of paired measures
Mean of paired measures

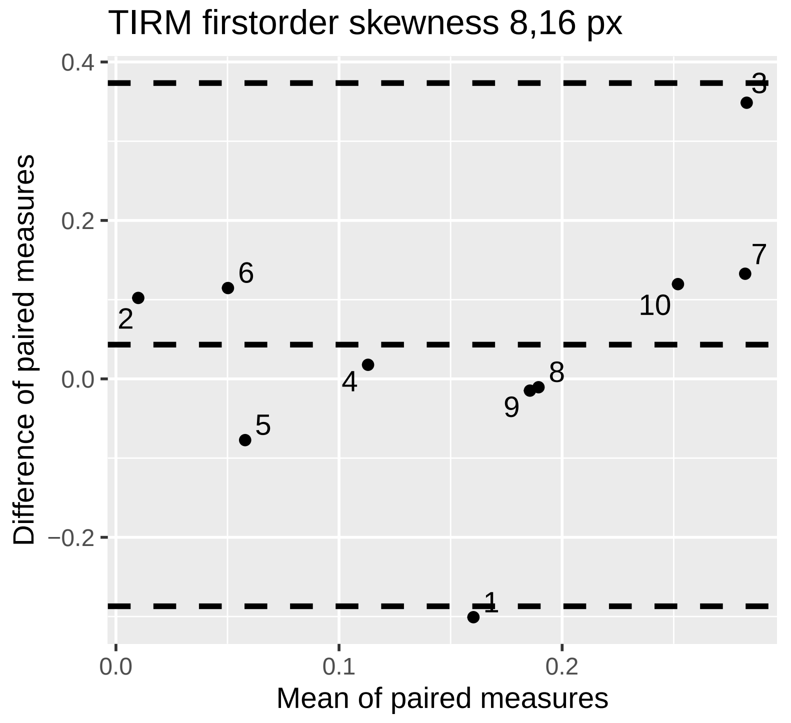
TIRM firstorder skewness 8,16 px
Difference of paired measures
Mean of paired measures

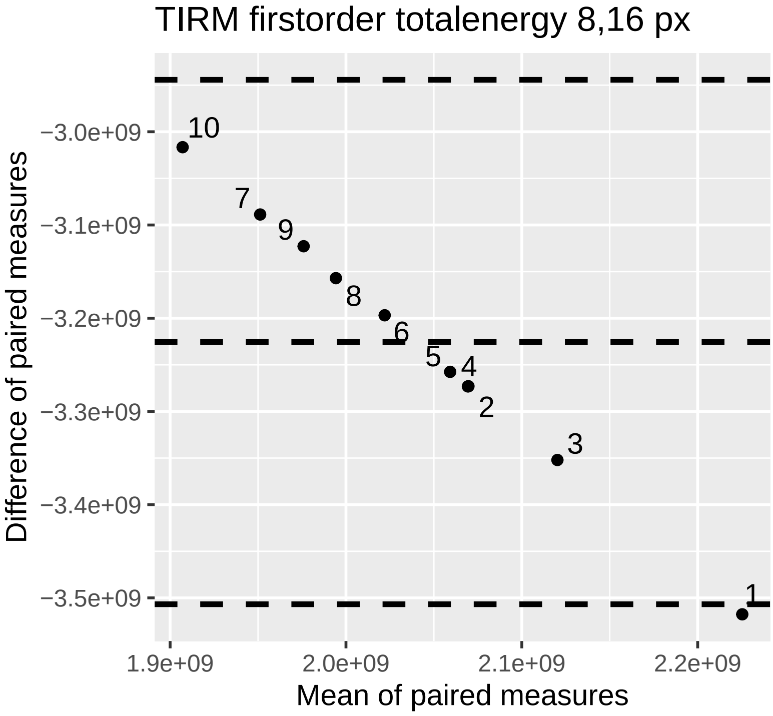
TIRM firstorder totalenergy 8,16 px
Difference of paired measures
−3.0e+09
−3.1e+09
−3.2e+09
−3.3e+09
−3.4e+09
−3.5e+09
1.9e+09
2.0e+09
2.1e+09
2.2e+09
Mean of paired measures

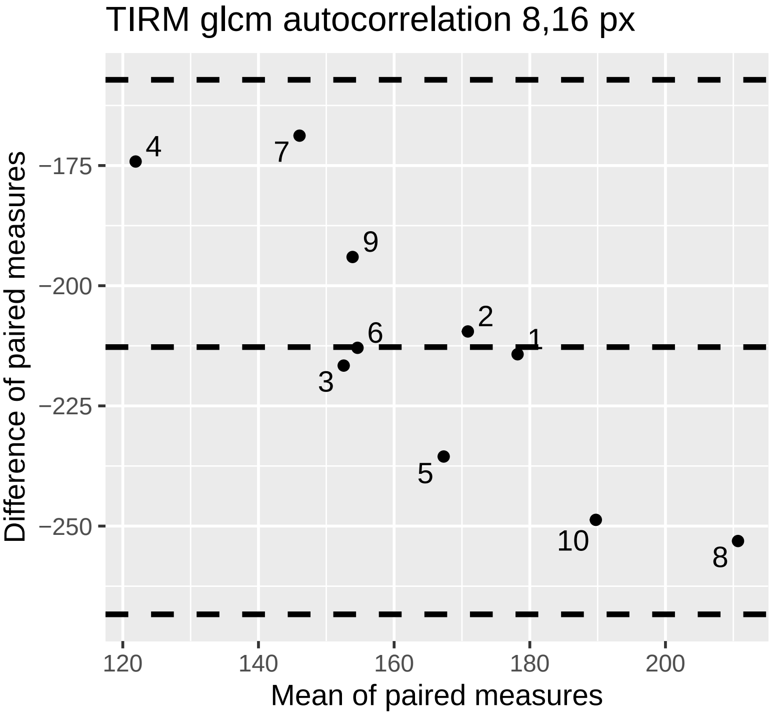
TIRM glcm autocorrelation 8,16 px
Difference of paired measures
−175
−200
−225
−250
120
140
160
180
200
Mean of paired measures

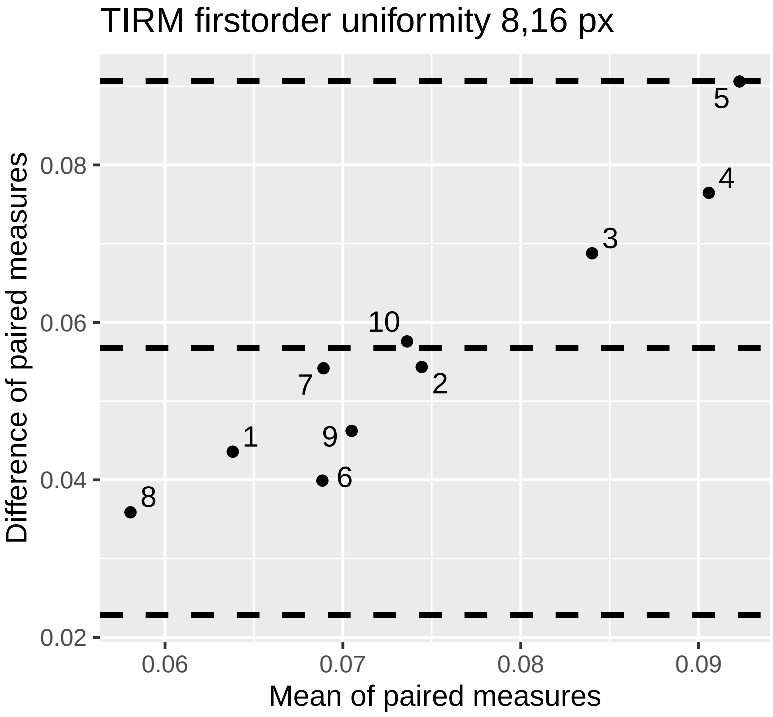
TIRM firstorder uniformity 8,16 px
Difference of paired measures
0.08
0.06
0.04
0.02
0.06
0.07
0.08
0.09
Mean of paired measures

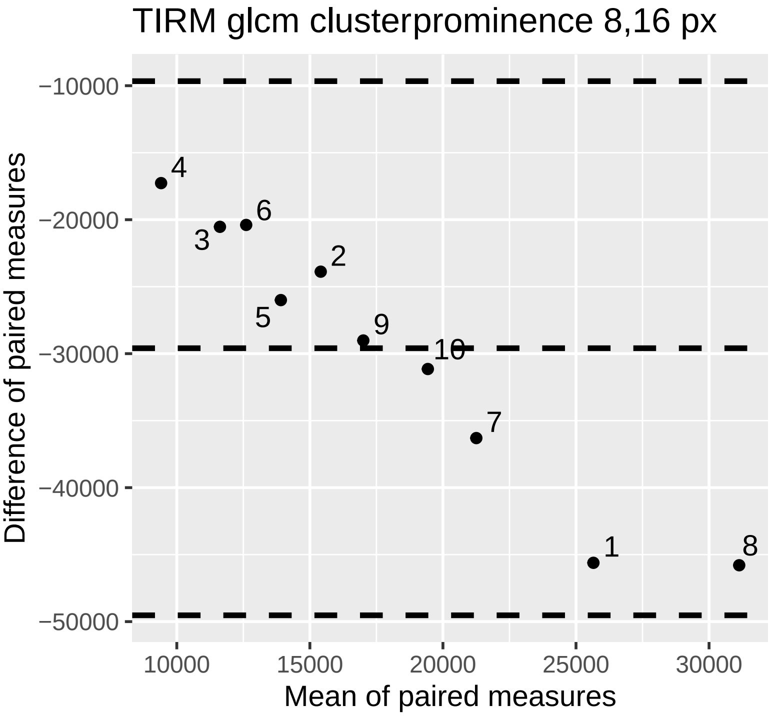
TIRM glcm clusterprominence 8,16 px
Difference of paired measures
−10000
−20000
−30000
−40000
−50000
10000
15000
20000
25000
30000
Mean of paired measures

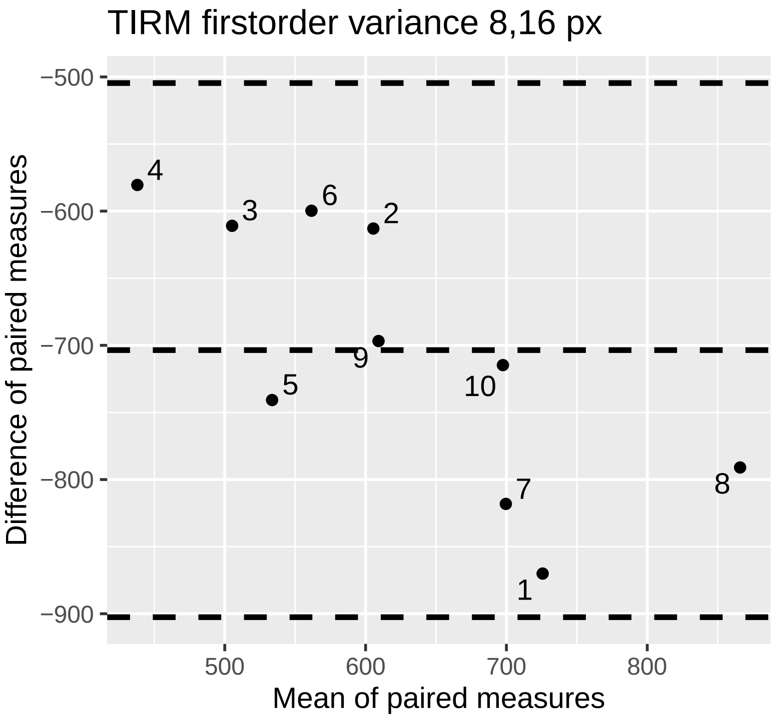
TIRM firstorder variance 8,16 px
Difference of paired measures
−500
−600
−700
−800
−900
500
600
700
800
Mean of paired measures

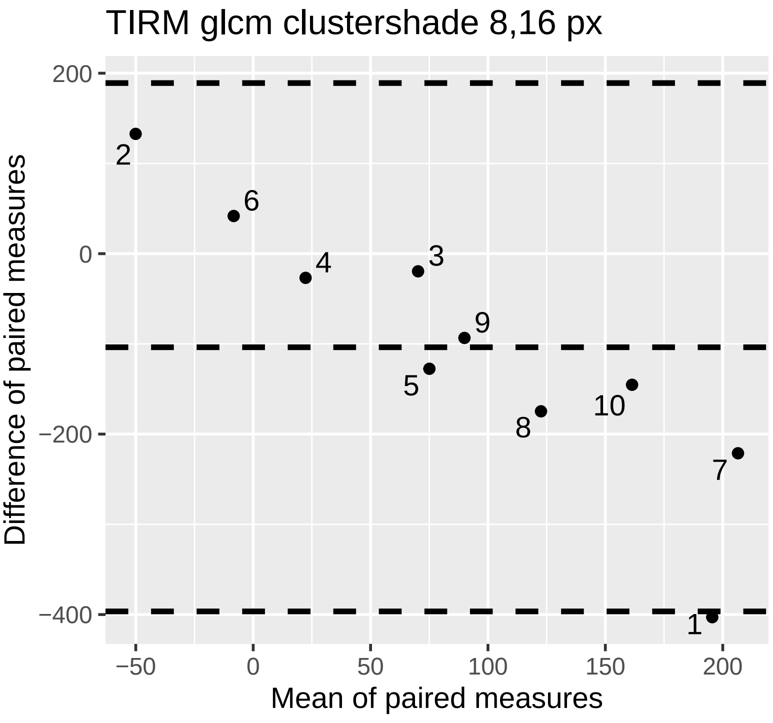
TIRM glcm clustershade 8,16 px
Difference of paired measures
200
0
−200
−400
−50
0
50
100
150
200
Mean of paired measures

TIRM glcm clustertendency 8,16 px

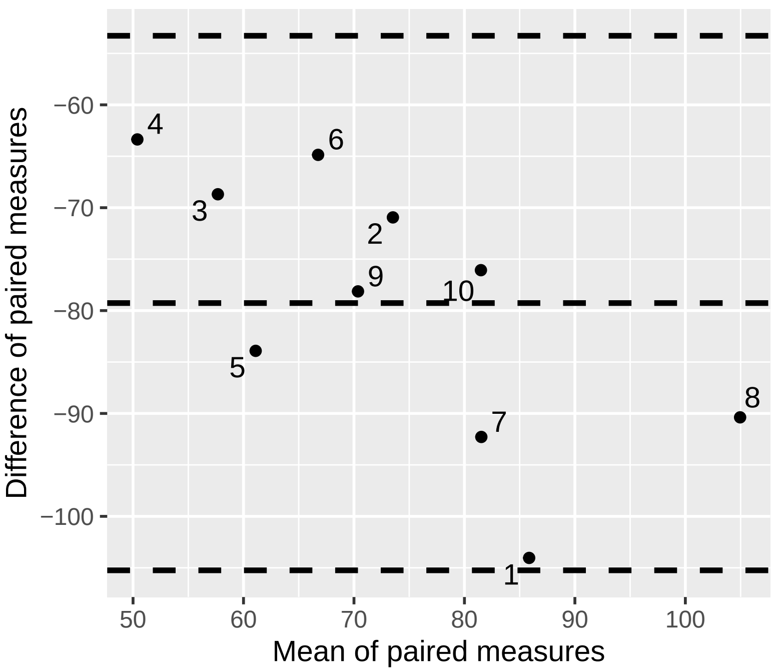


TIRM glcm differenceaverage 8,16 px

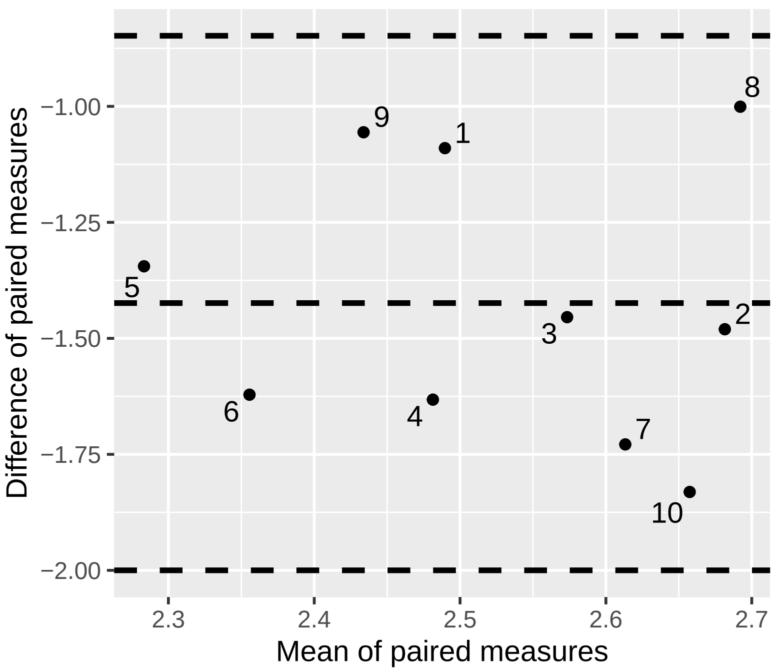


TIRM glcm contrast 8,16 px

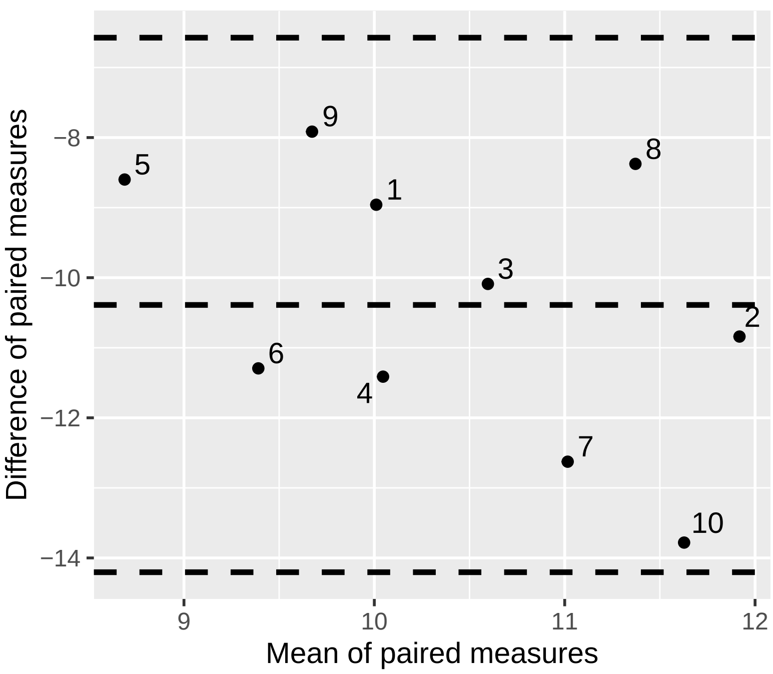


TIRM glcm differenceentropy 8,16 px

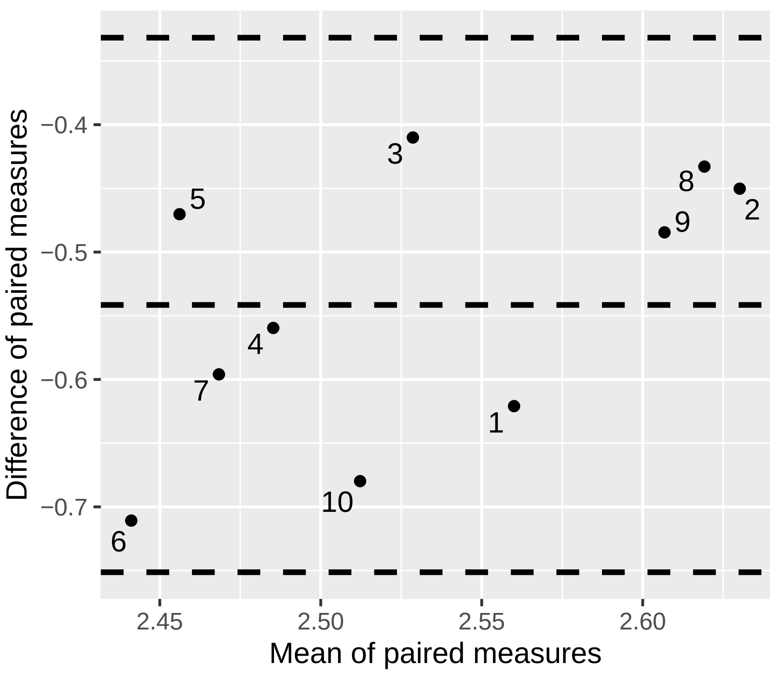


TIRM glcm correlation 8,16 px

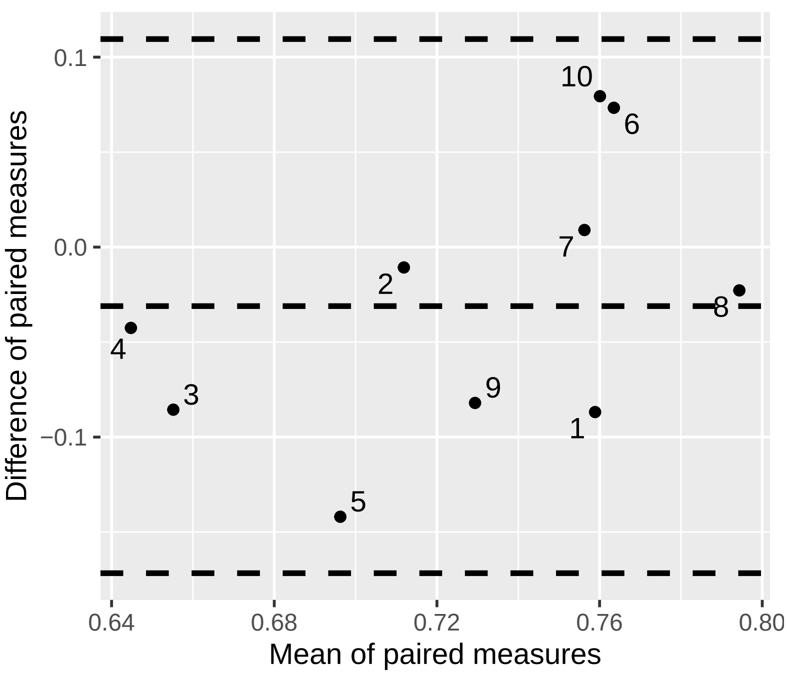


TIRM glcm differencevariance 8,16 px

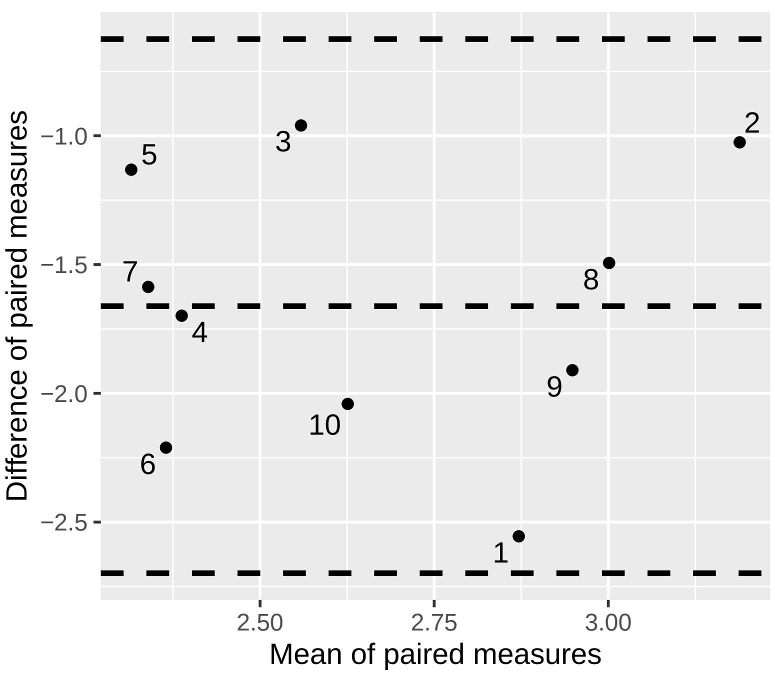

TIRM glcm id 8,16 px

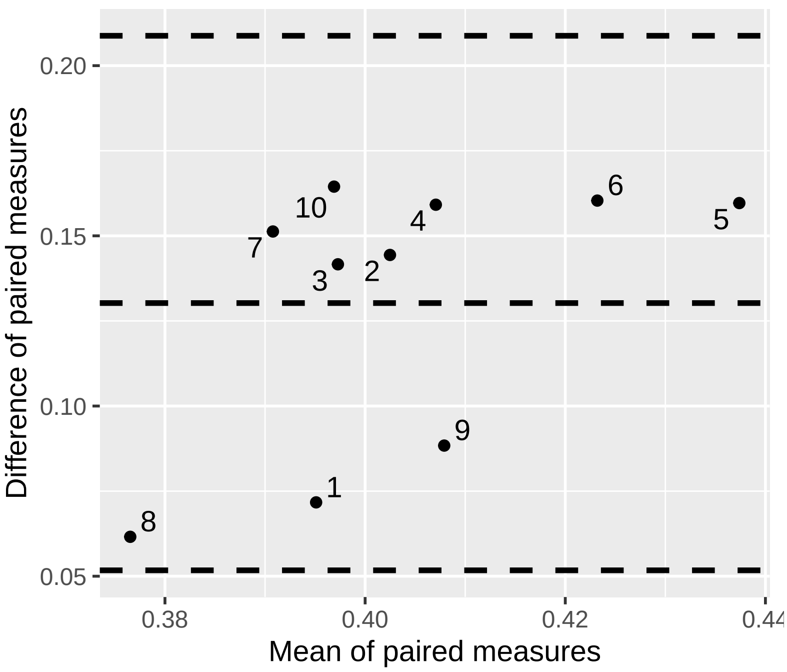


TIRM glcm idn 8,16 px

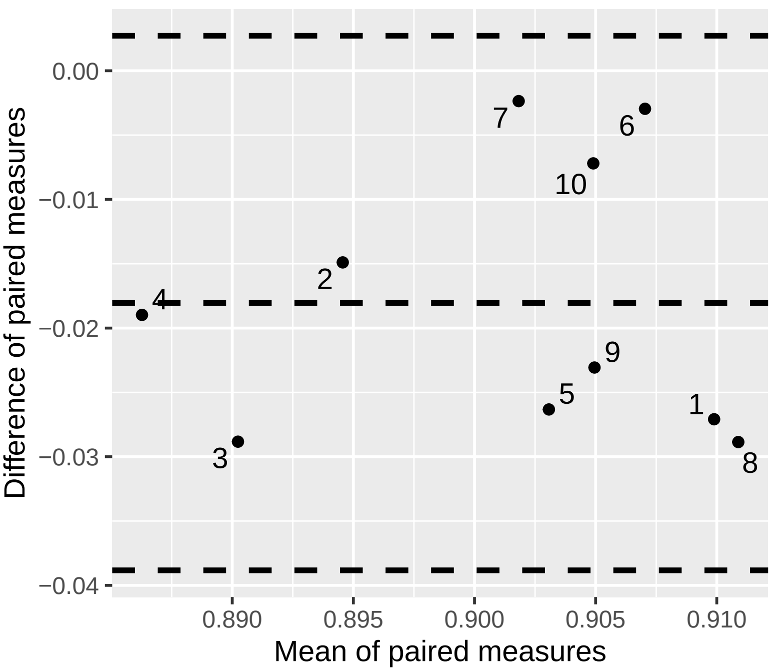


TIRM glcm idm 8,16 px

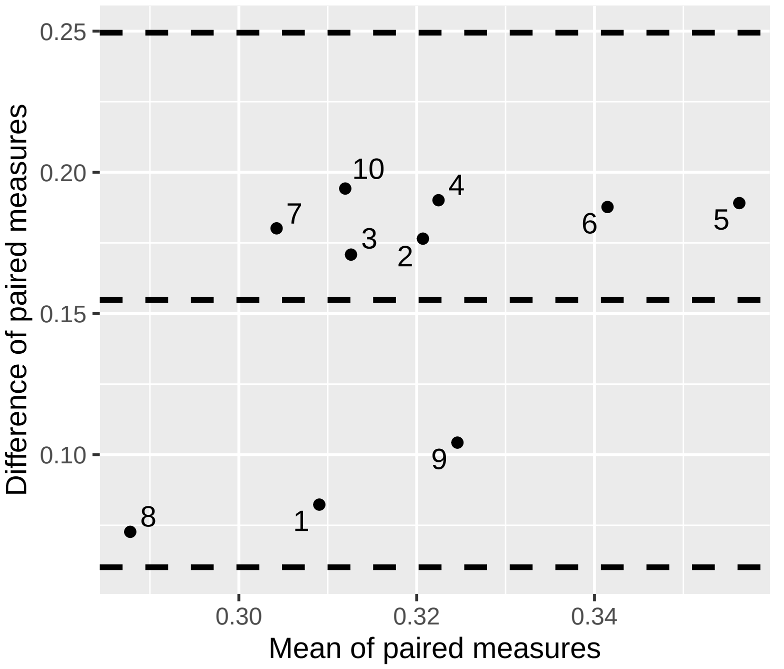


TIRM glcm imc1 8,16 px

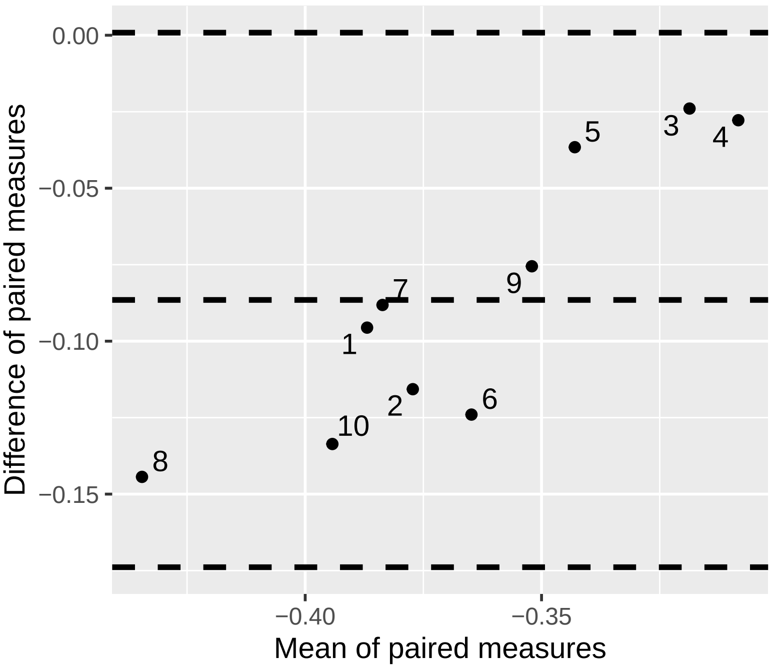


TIRM glcm idmn 8,16 px

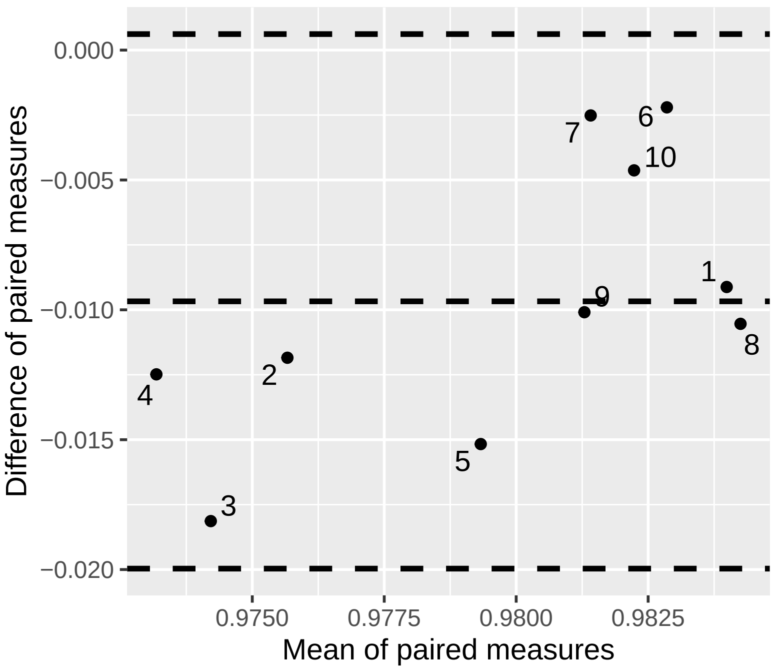


TIRM glcm imc2 8,16 px

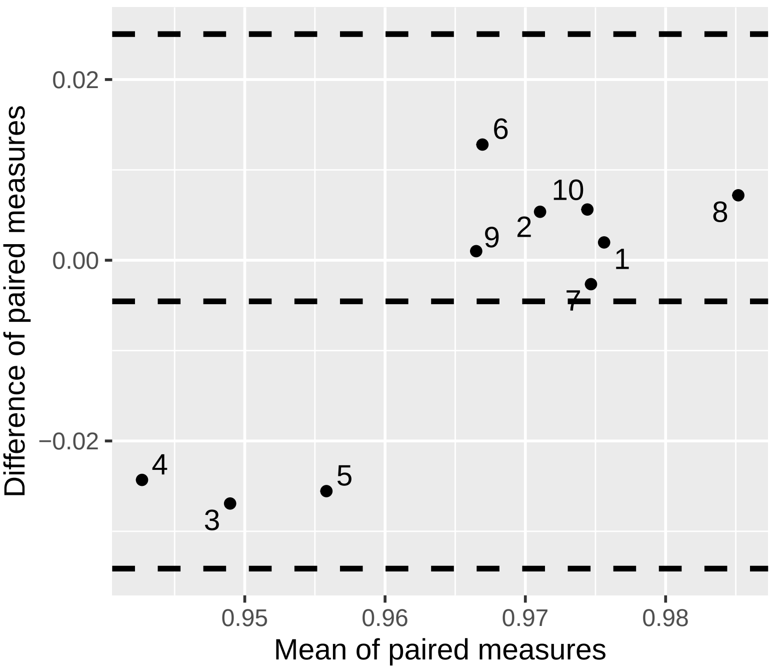

TIRM glcm inversevariance 8,16 px

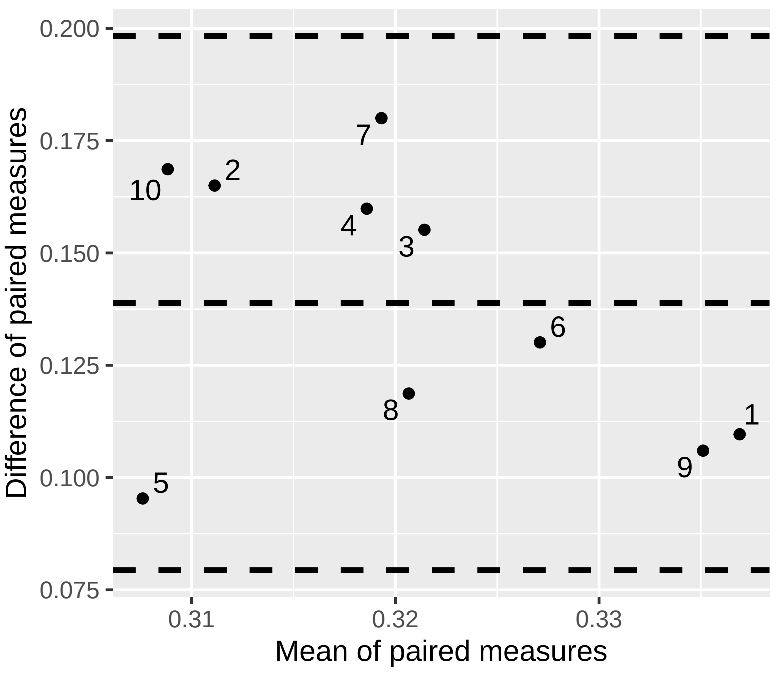


TIRM glcm jointentropy 8,16 px

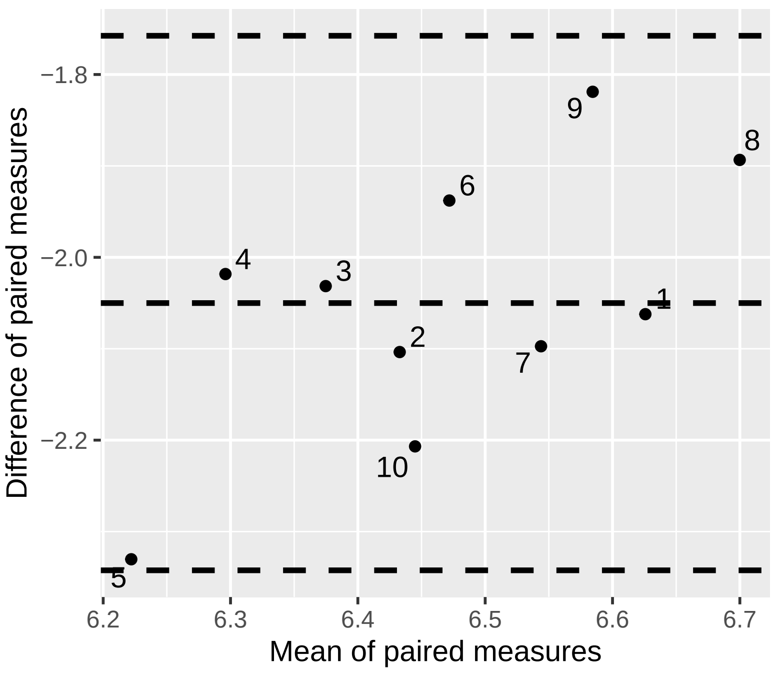


TIRM glcm jointaverage 8,16 px

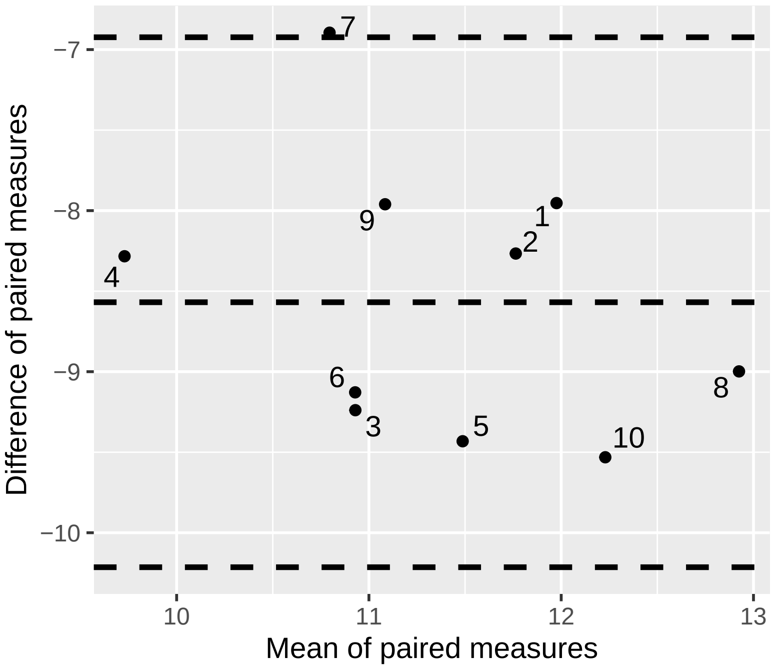


TIRM glcm mcc 8,16 px

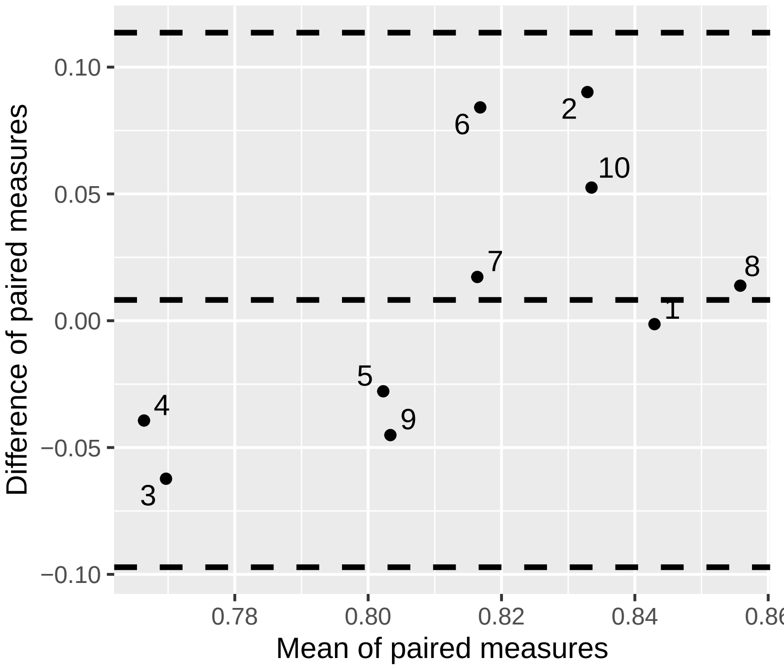


TIRM glcm jointenergy 8,16 px

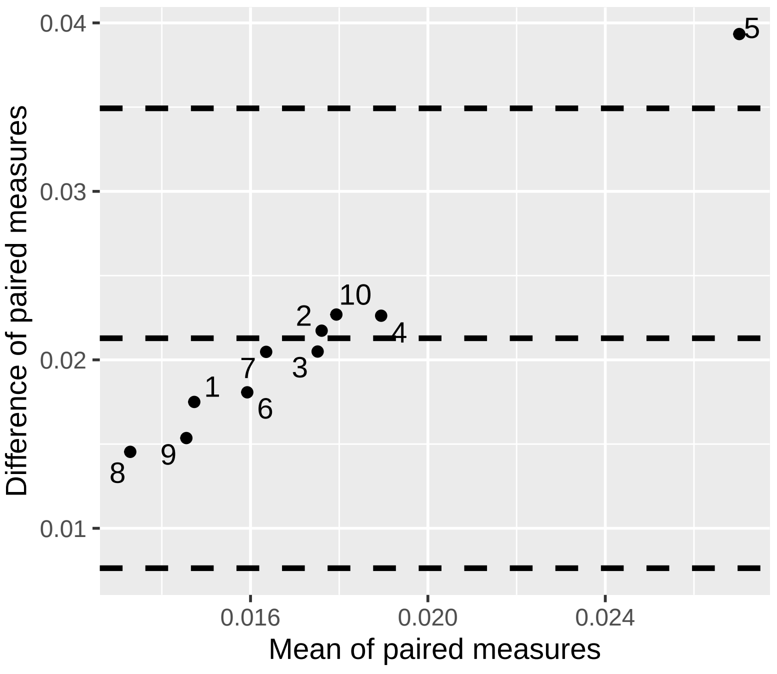


TIRM glcm maximumprobability 8,16 px

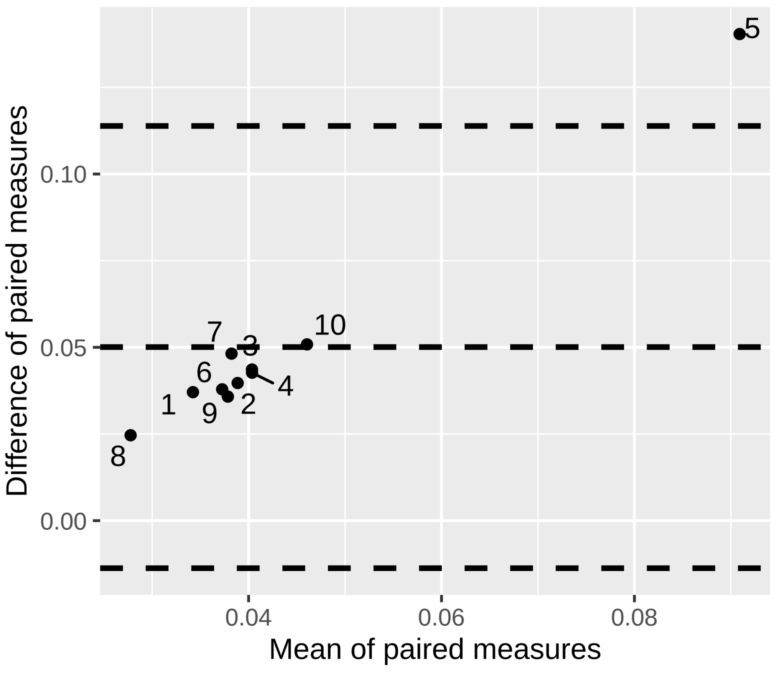

TIRM glcm sumaverage 8,16 px

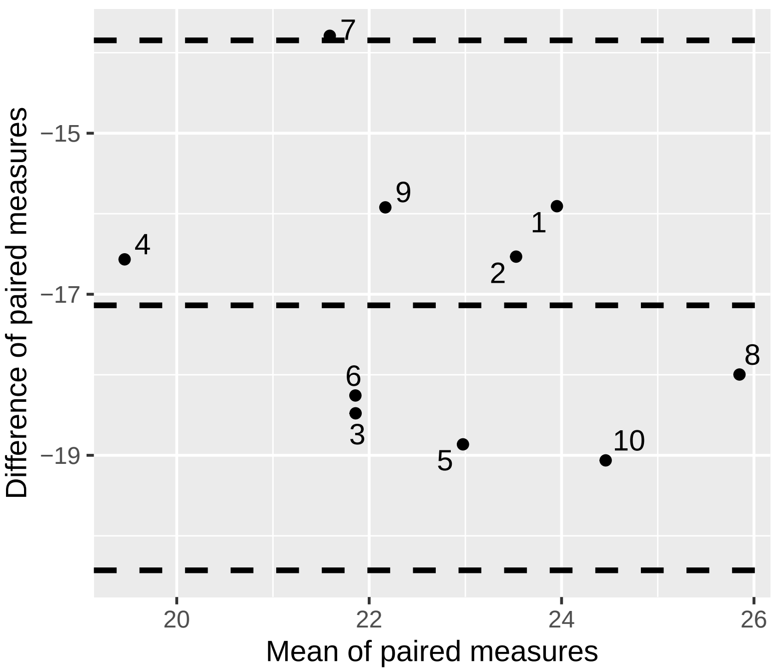


TIRM glrlm graylevelnonuniformity 8,16 px

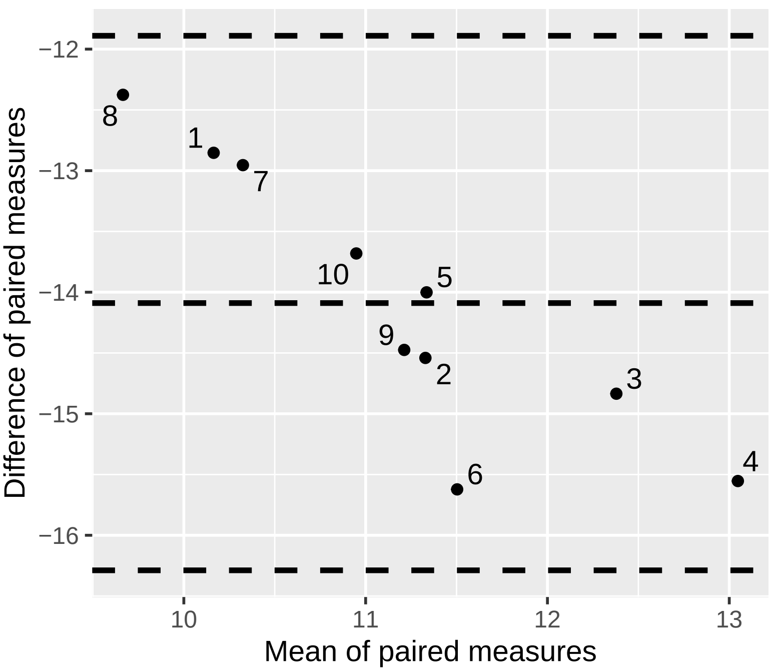


TIRM glcm sumentropy 8,16 px

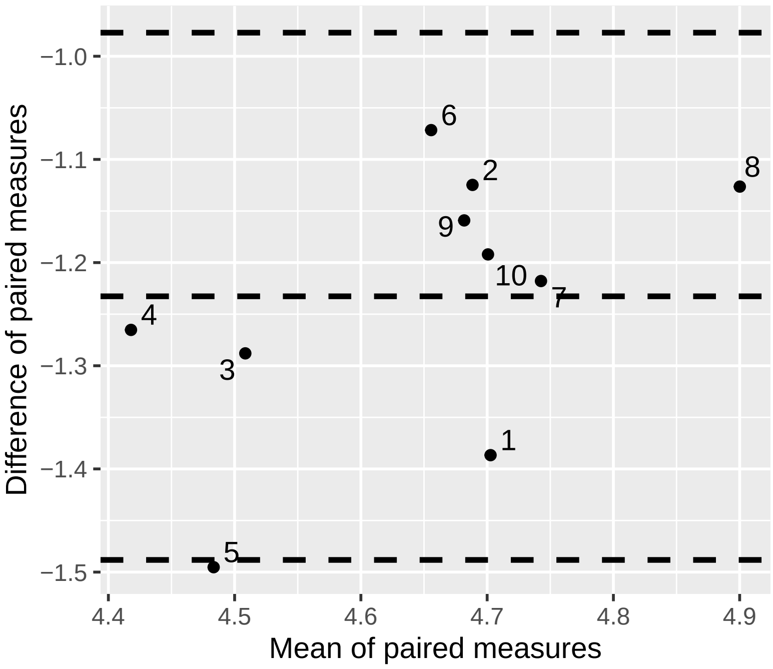


TIRM glrlm graylevelnonuniformitynormalized

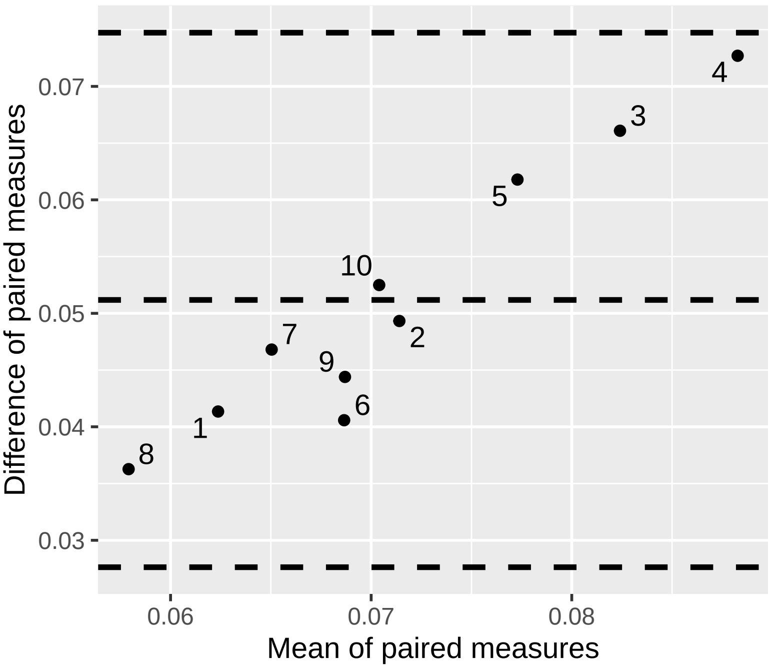


TIRM glcm sumsquares 8,16 px

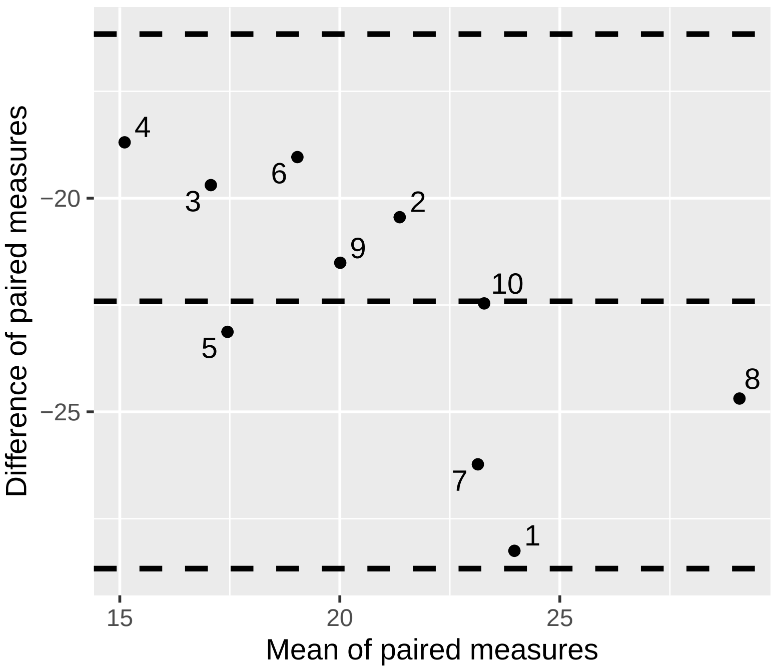


TIRM glrlm graylevelvariance 8,16 px

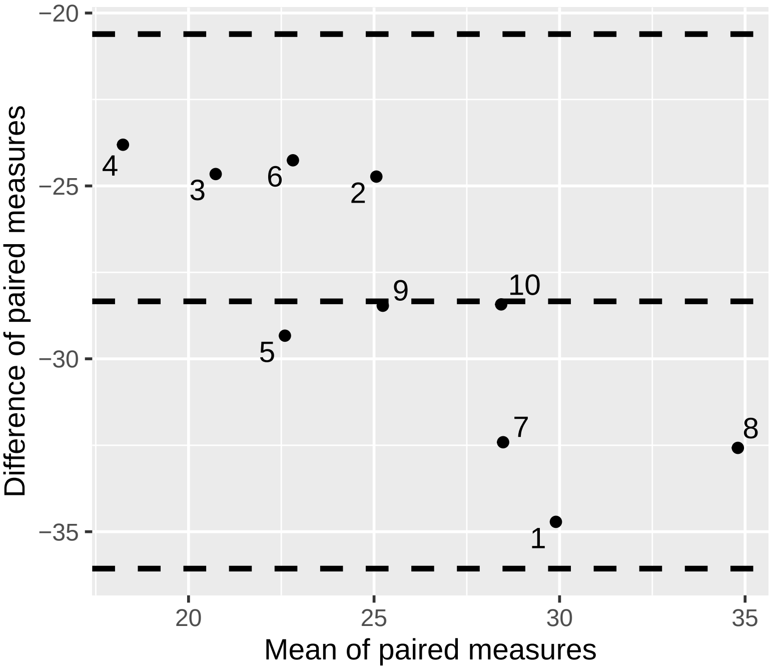

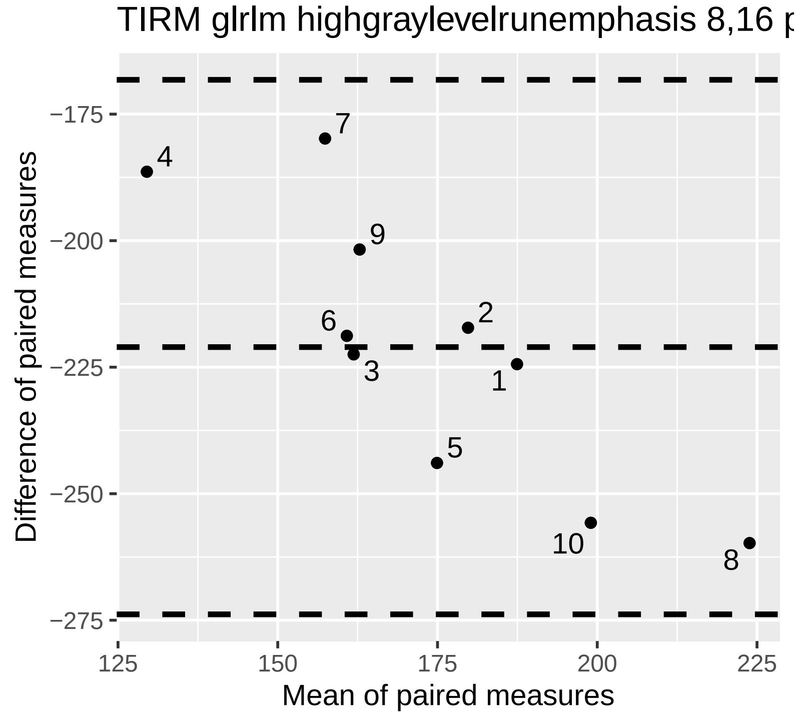
TIRM glrlm highgraylevelrunemphasis 8,16 p
Difference of paired measures
Mean of paired measures
−175
−200
−225
−250
−275
125
150
175
200
225
7
4
9
6
2
3
1
5
10
8

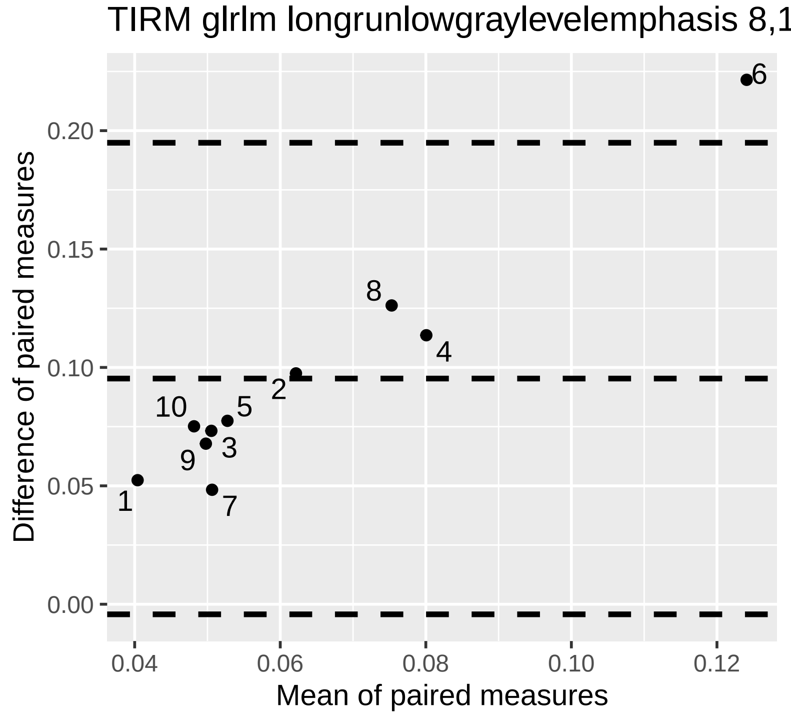
TIRM glrlm longrunlowgraylevelemphasis 8,1
Difference of paired measures
Mean of paired measures
0.20
0.15
0.10
0.05
0.00
0.04
0.06
0.08
0.10
0.12
6
8
4
2
10
5
9
3
1
7

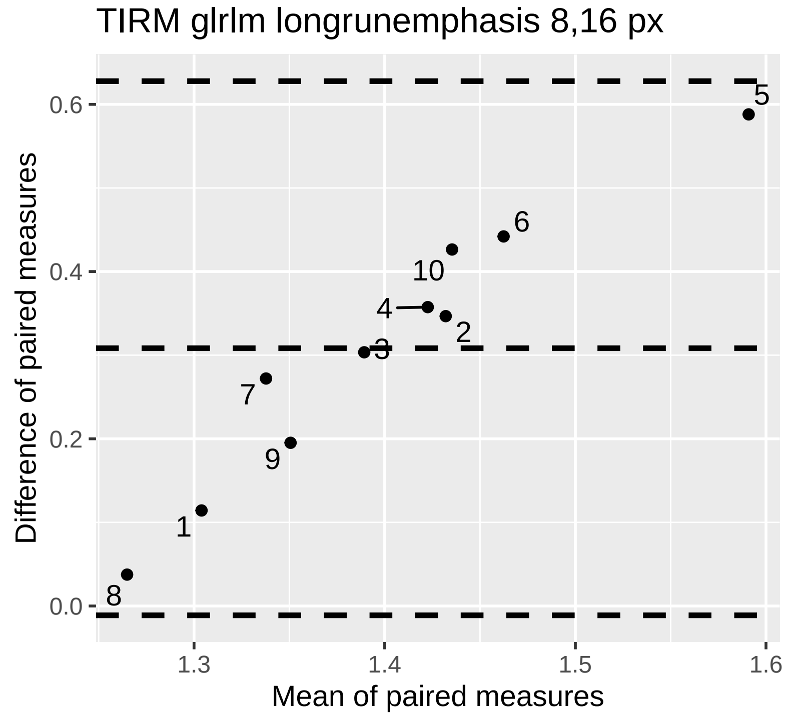
TIRM glrlm longrunemphasis 8,16 px
Difference of paired measures
Mean of paired measures
0.6
0.4
0.2
0.0
1.3
1.4
1.5
1.6
5
6
10
4
2
3
7
9
1
8

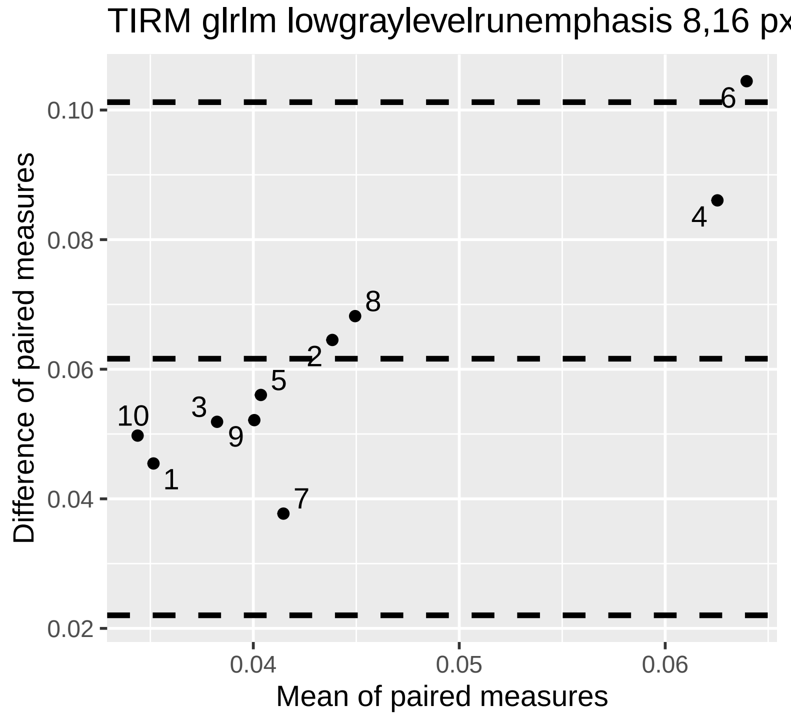
TIRM glrlm lowgraylevelrunemphasis 8,16 px
Difference of paired measures
Mean of paired measures
0.10
0.08
0.06
0.04
0.02
0.04
0.05
0.06
6
4
8
2
5
10
3
9
1
7

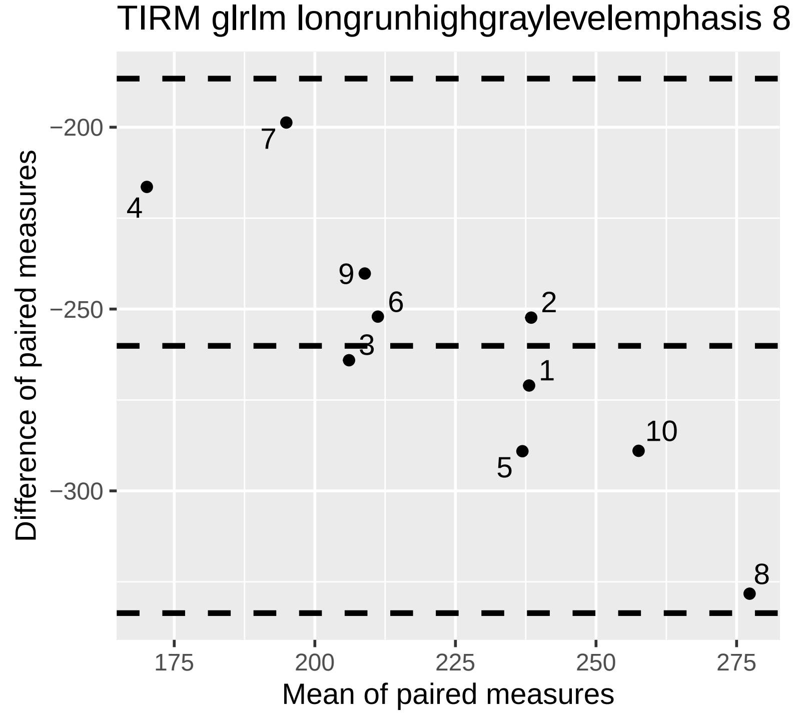
TIRM glrlm longrunhighgraylevelemphasis 8
Difference of paired measures
Mean of paired measures
−200
−250
−300
175
200
225
250
275
7
4
9
6
2
3
1
10
5
8

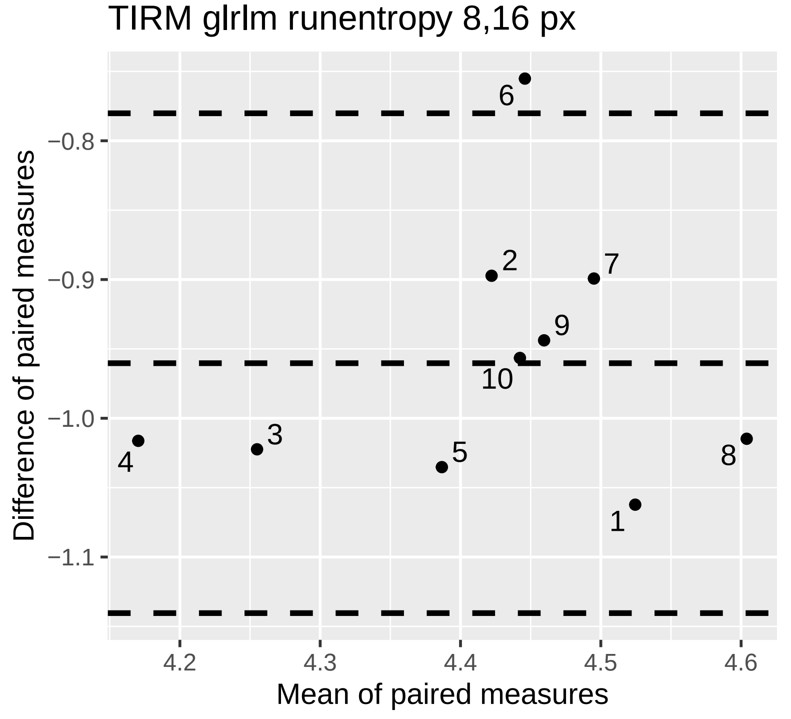
TIRM glrlm runentropy 8,16 px
Difference of paired measures
Mean of paired measures
−0.8
−0.9
−1.0
−1.1
4.2
4.3
4.4
4.5
4.6
6
2
7
9
10
4
3
5
8
1

TIRM glrlm runlengthnonuniformity 8,16 px

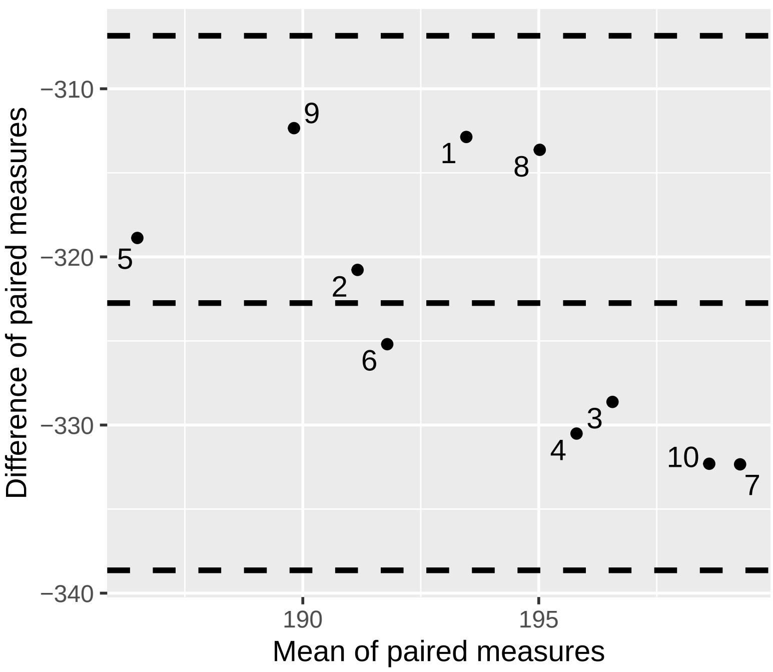


TIRM glrlm runvariance 8,16 px

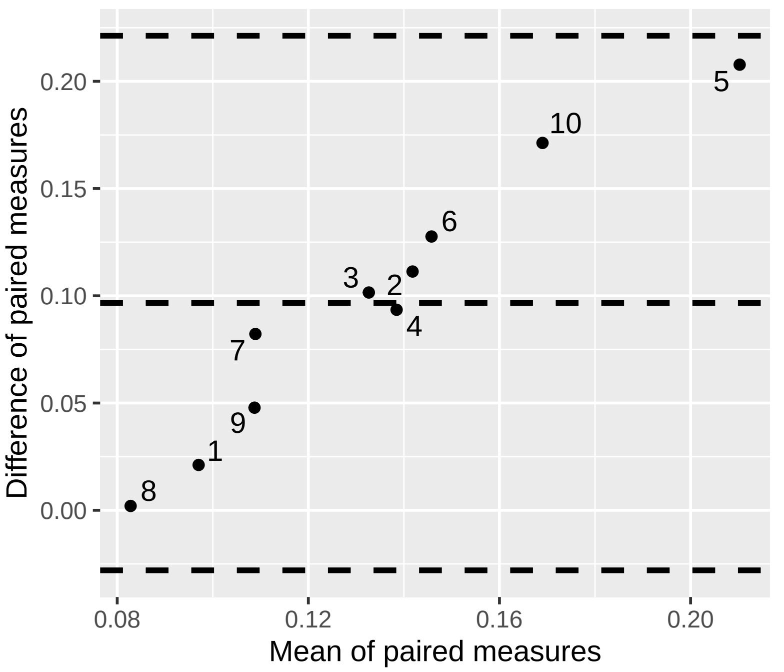


TIRM glrlm runlengthnonuniformitynormalize

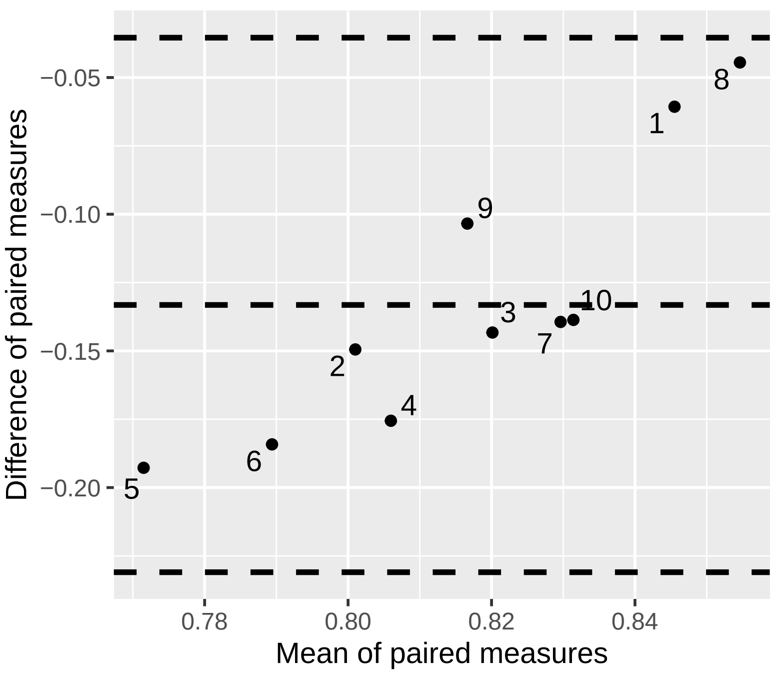


TIRM glrlm shortrunemphasis 8,16 px

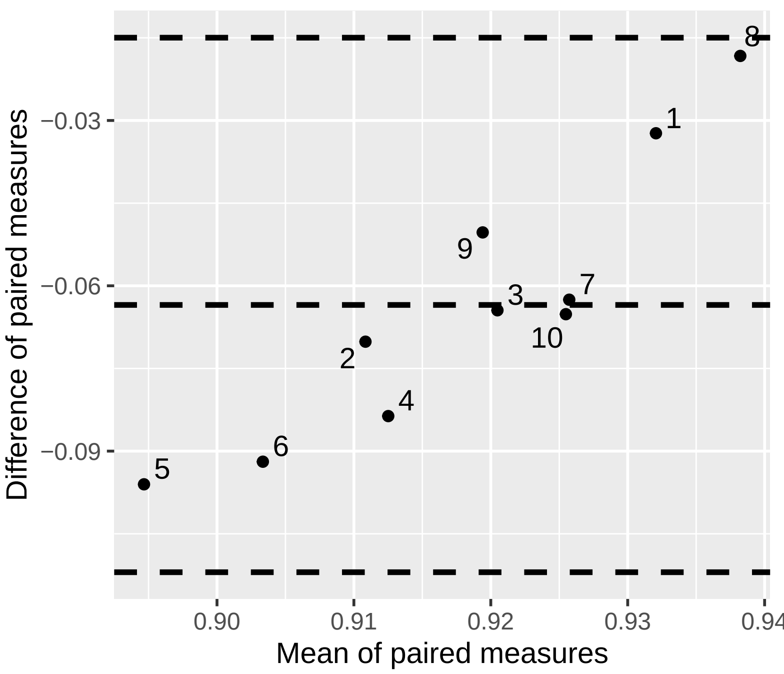


TIRM glrlm runpercentage 8,16 px

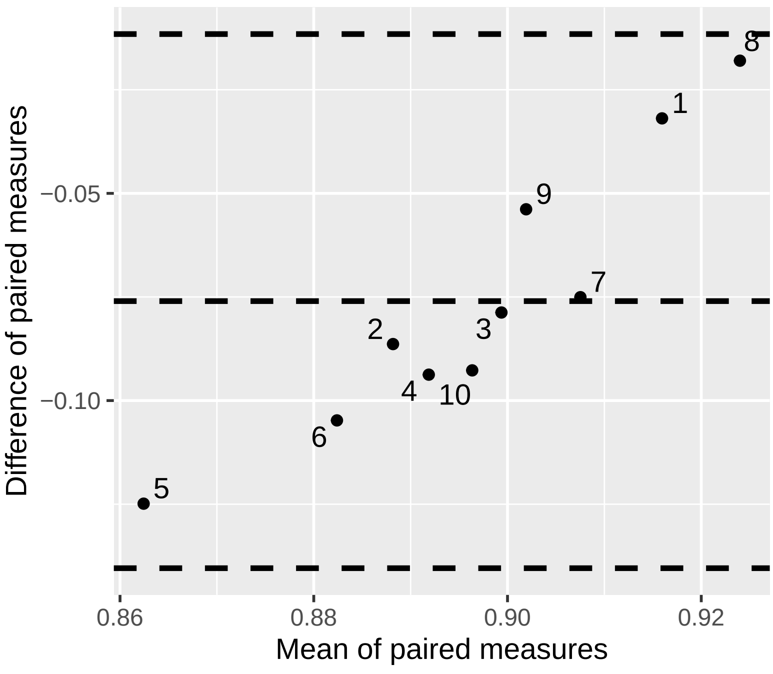


TIRM glrlm shortrunhighgraylevelemphasis 8

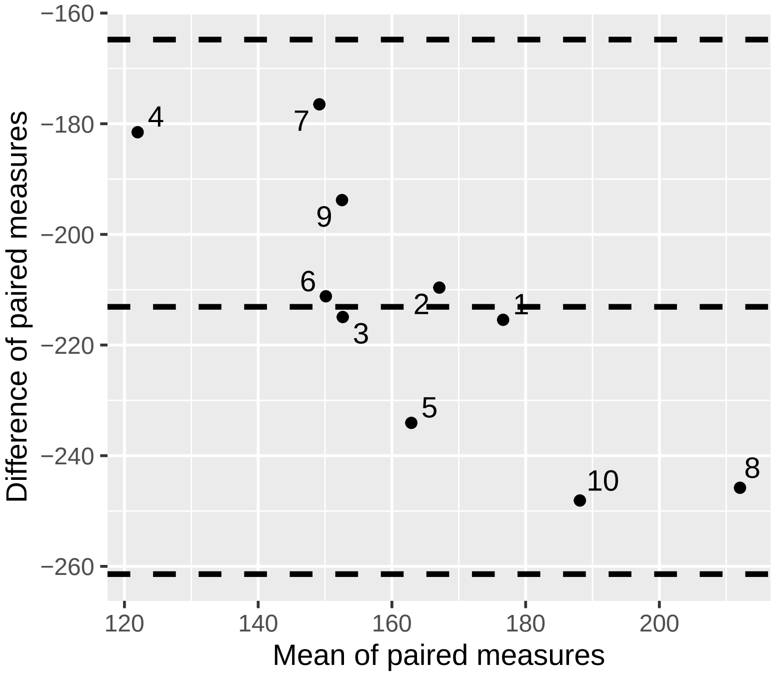

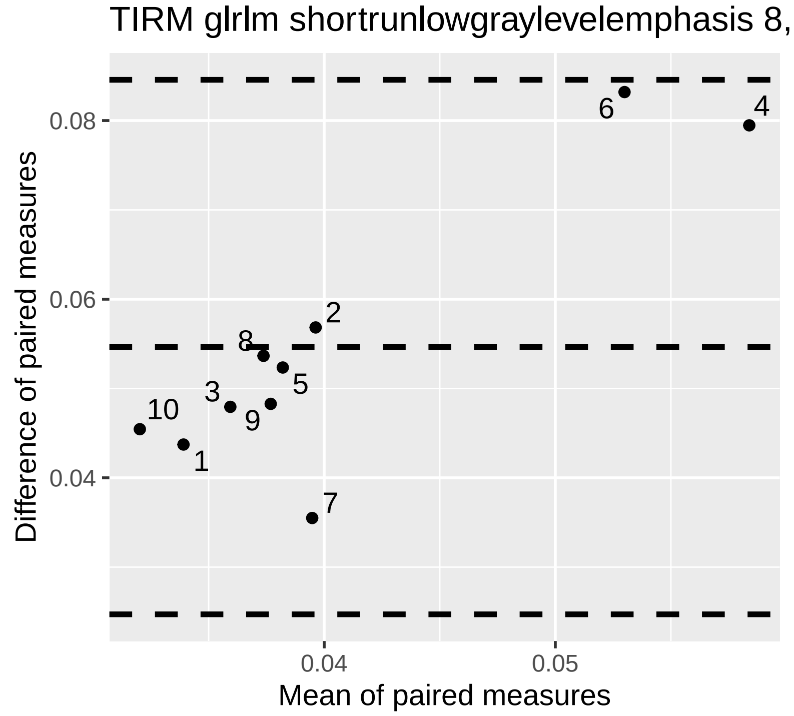
TIRM glrlm shortrunlowgraylevelemphasis 8,
0.08
0.06
0.04
Difference of paired measures
0.04
0.05
Mean of paired measures
1
2
3
4
5
6
7
8
9
10

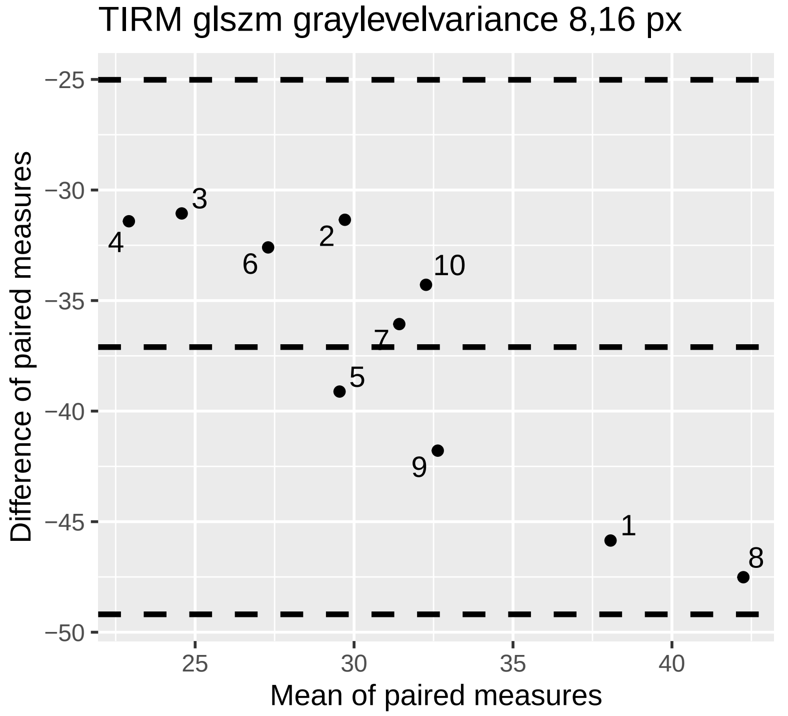
TIRM glszm graylevelvariance 8,16 px
-25
-30
-35
-40
-45
-50
Difference of paired measures
25
30
35
40
Mean of paired measures
1
2
3
4
5
6
7
8
9
10

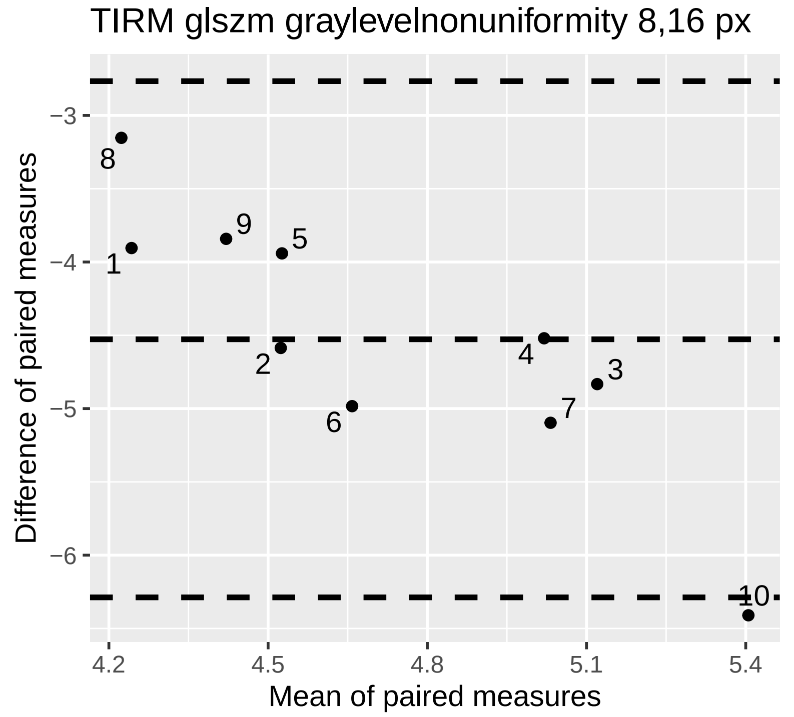
TIRM glszm graylevelnonuniformity 8,16 px
-3
-4
-5
-6
Difference of paired measures
4.2
4.5
4.8
5.1
5.4
Mean of paired measures
1
2
3
4
5
6
7
8
9
10

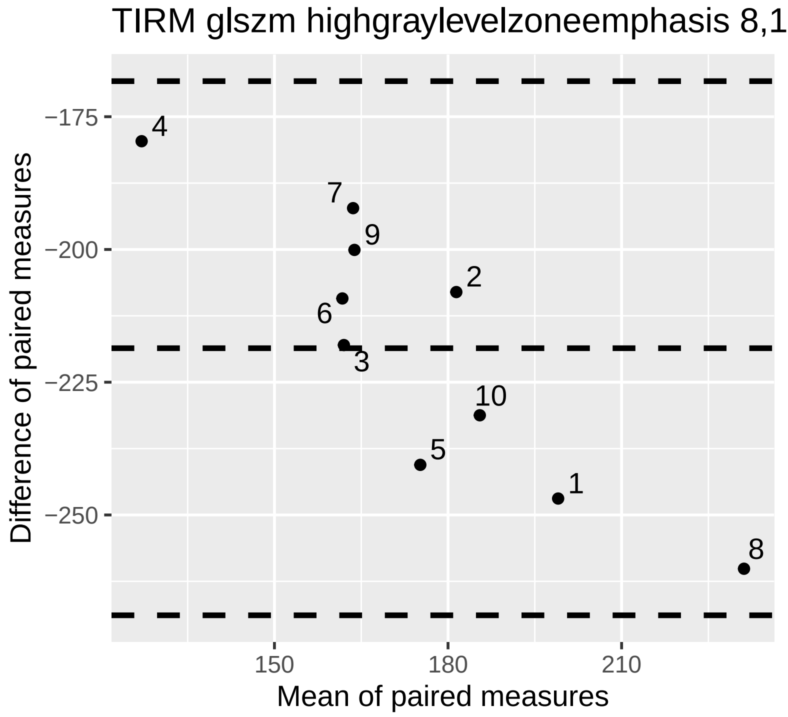
TIRM glszm highgraylevelzoneemphasis 8,1
-175
-200
-225
-250
Difference of paired measures
150
180
210
Mean of paired measures
1
2
3
4
5
6
7
8
9
10

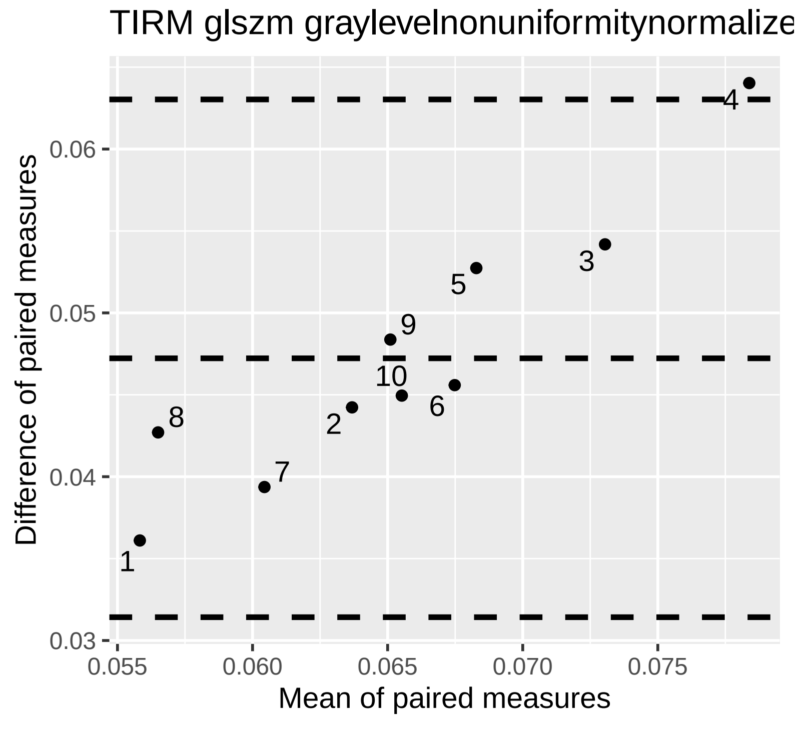
TIRM glszm graylevelnonuniformitynormalize
0.06
0.05
0.04
0.03
Difference of paired measures
0.055
0.060
0.065
0.070
0.075
Mean of paired measures
1
2
3
4
5
6
7
8
9
10

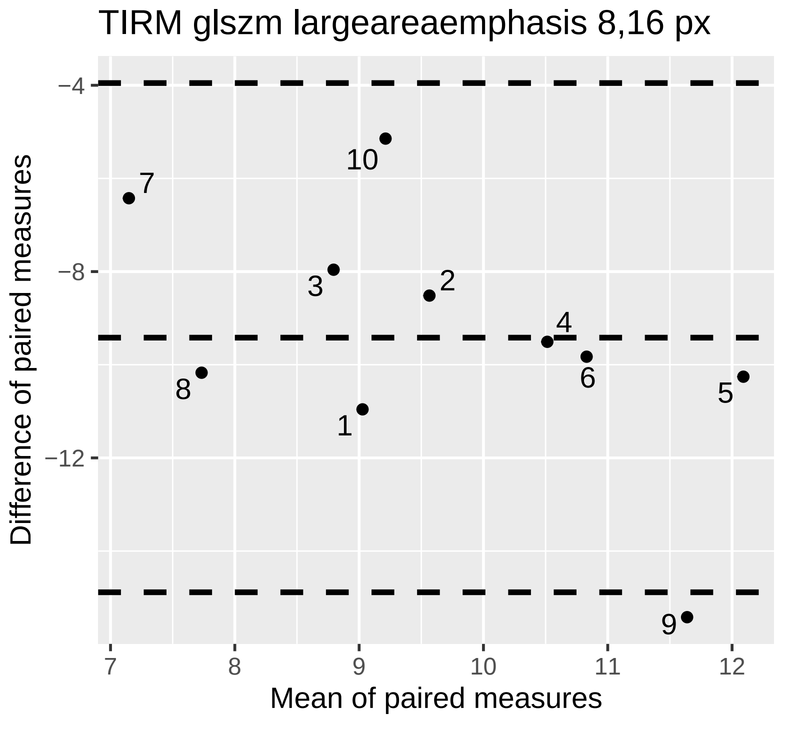
TIRM glszm largeareaemphasis 8,16 px
-4
-8
-12
Difference of paired measures
7
8
9
10
11
12
Mean of paired measures
1
2
3
4
5
6
7
8
9
10

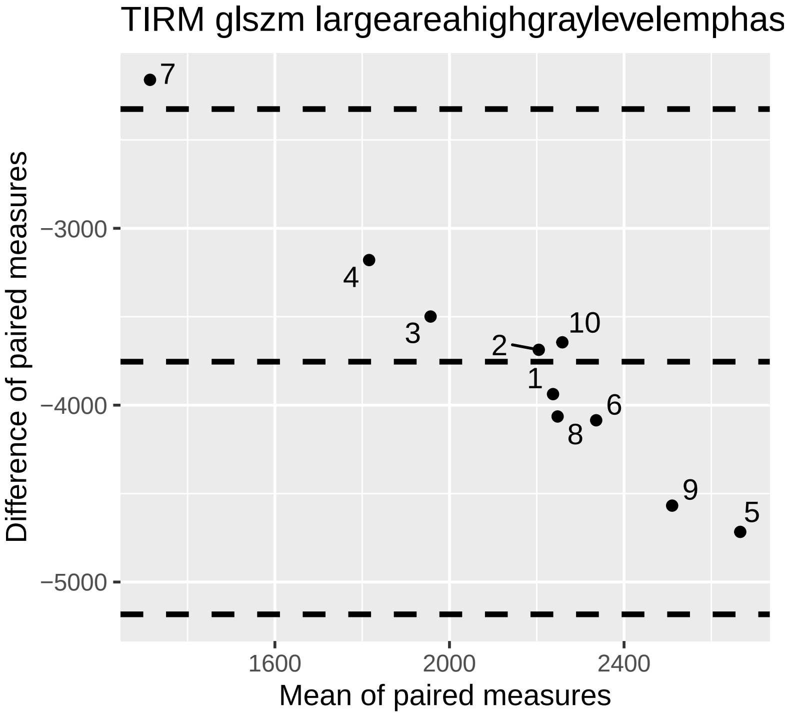
TIRM glszm largeareahighgraylevelemphas
Difference of paired measures
Mean of paired measures
−3000
−4000
−5000
1600
2000
2400
1
2
3
4
5
6
7
8
9
10

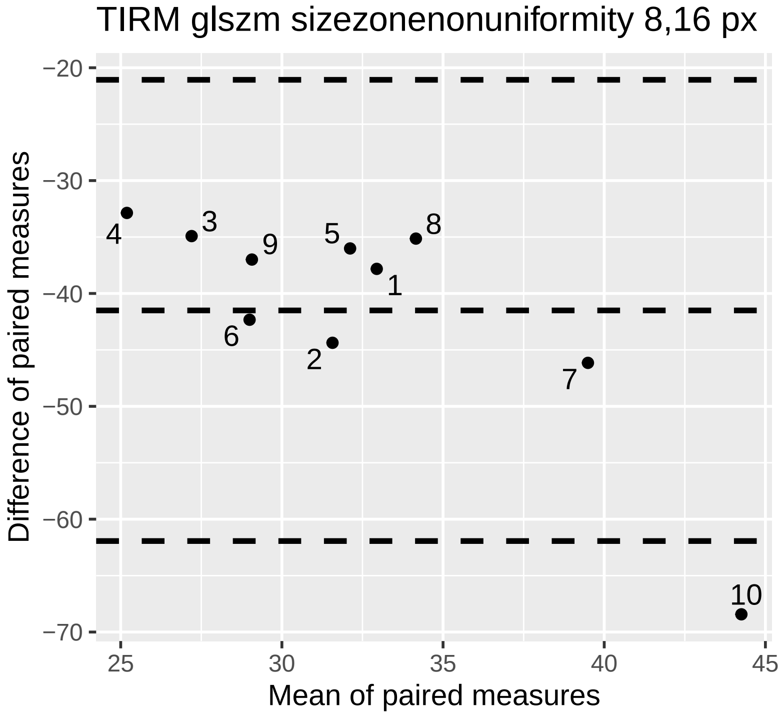
TIRM glszm sizezonenonuniformity 8,16 px
Difference of paired measures
Mean of paired measures
−20
−30
−40
−50
−60
−70
25
30
35
40
45
1
2
3
4
5
6
7
8
9
10

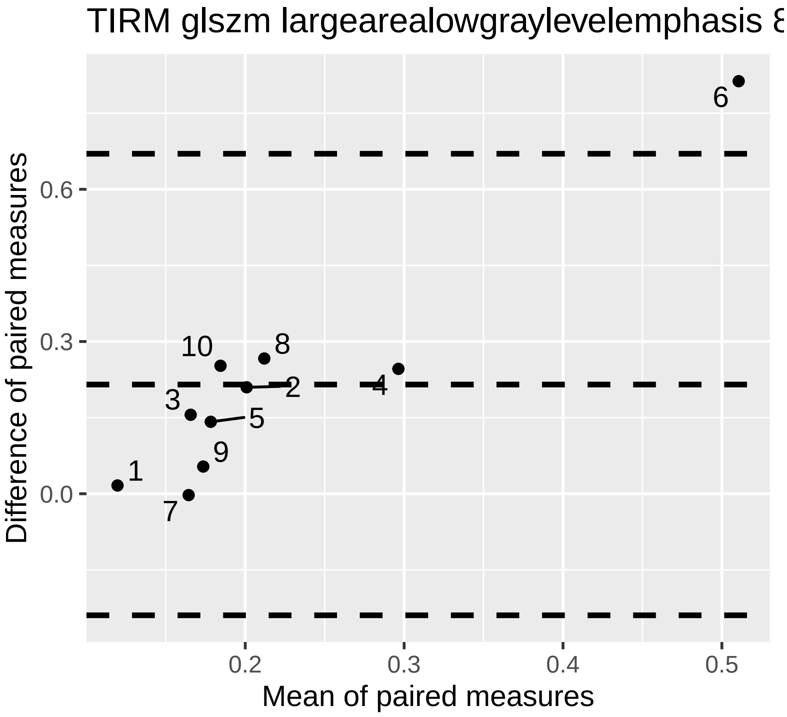
TIRM glszm largearealowgraylevelemphasis 8
Difference of paired measures
Mean of paired measures
0.6
0.3
0.0
0.2
0.3
0.4
0.5
1
2
3
4
5
6
7
8
9
10

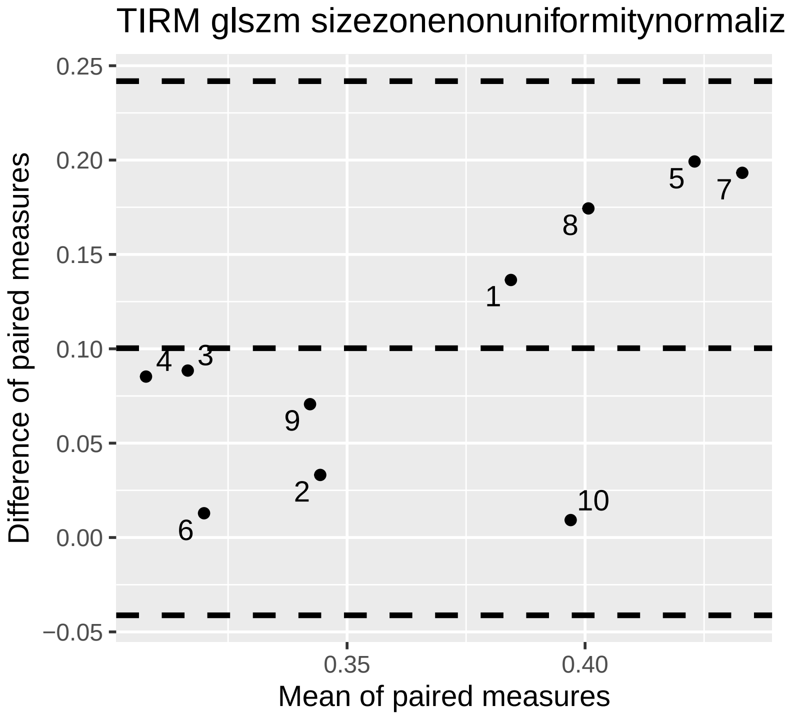
TIRM glszm sizezonenonuniformitynormaliz
Difference of paired measures
Mean of paired measures
0.25
0.20
0.15
0.10
0.05
0.00
−0.05
0.35
0.40
1
2
3
4
5
6
7
8
9
10

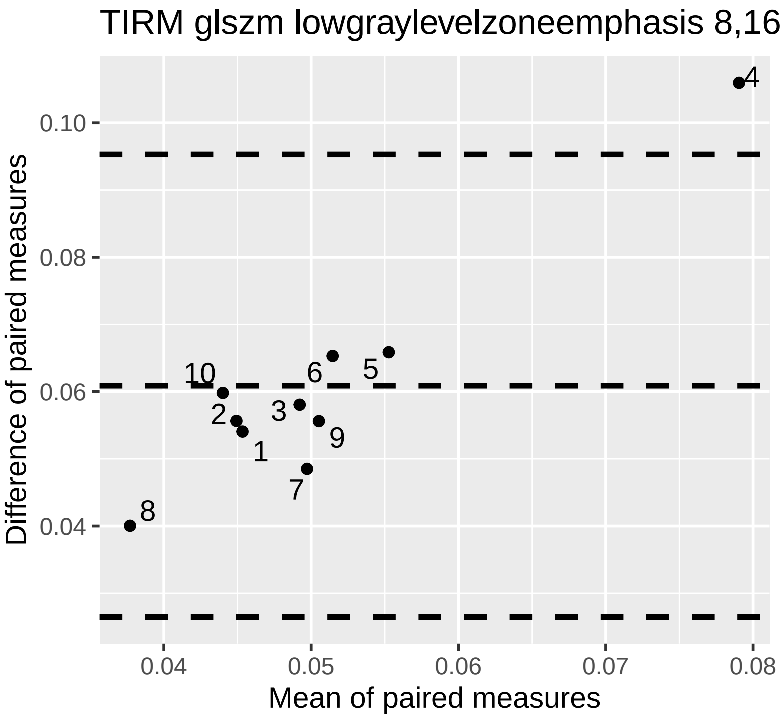
TIRM glszm lowgraylevelzoneemphasis 8,16
Difference of paired measures
Mean of paired measures
0.10
0.08
0.06
0.04
0.04
0.05
0.06
0.07
0.08
1
2
3
4
5
6
7
8
9
10

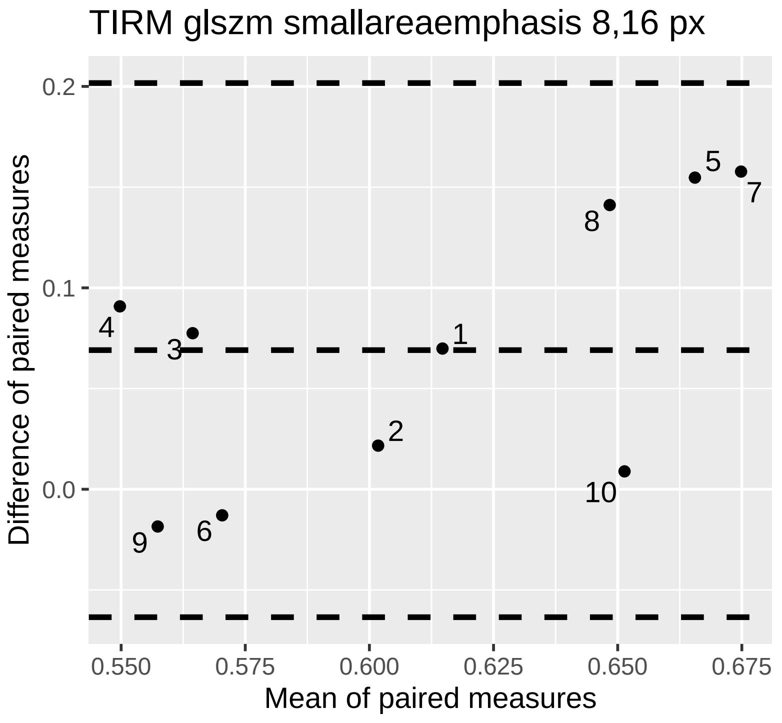
TIRM glszm smallareaemphasis 8,16 px
Difference of paired measures
Mean of paired measures
0.2
0.1
0.0
0.550
0.575
0.600
0.625
0.650
0.675
1
2
3
4
5
6
7
8
9
10

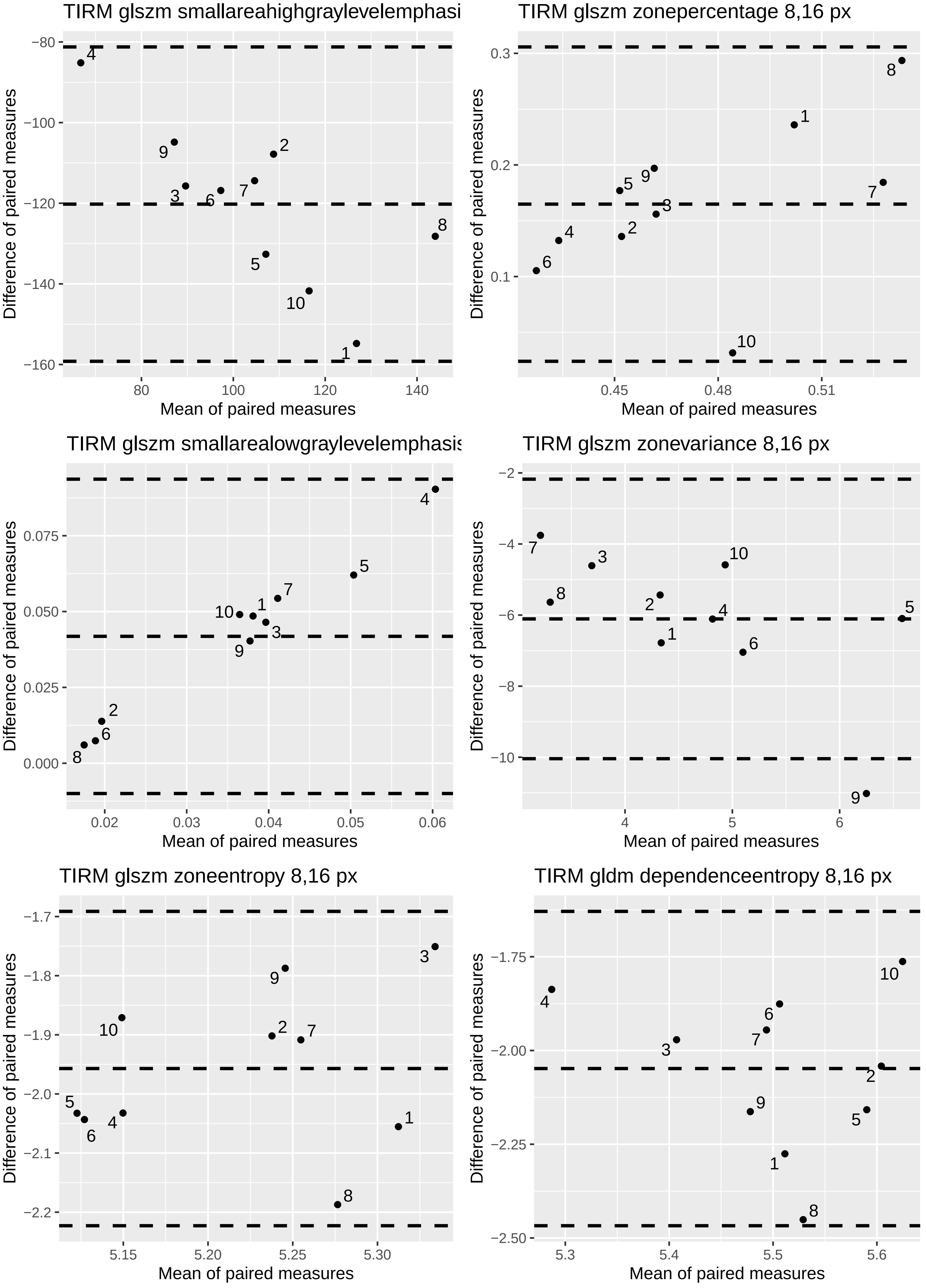
TIRM glszm smallareahighgraylevelemphasi
TIRM glszm zonepercentage 8,16 px
TIRM glszm smallarealowgraylevelemphasis
TIRM glszm zonevariance 8,16 px
TIRM glszm zoneentropy 8,16 px
TIRM gldm dependenceentropy 8,16 px
Difference of paired measures
Mean of paired measures

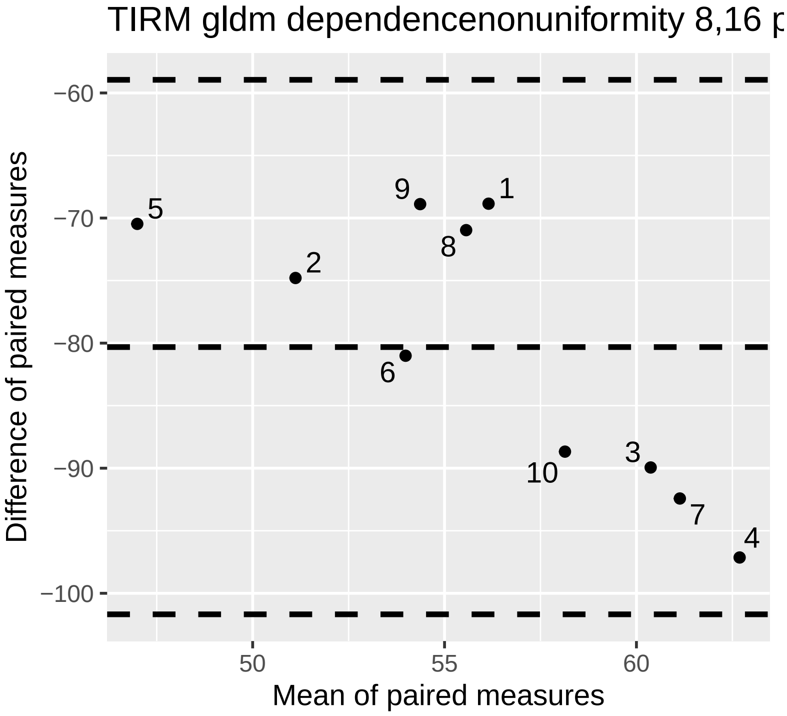
TIRM gldm dependencenonuniformity 8,16 p
Difference of paired measures
Mean of paired measures
−60
−70
−80
−90
−100
50
55
60

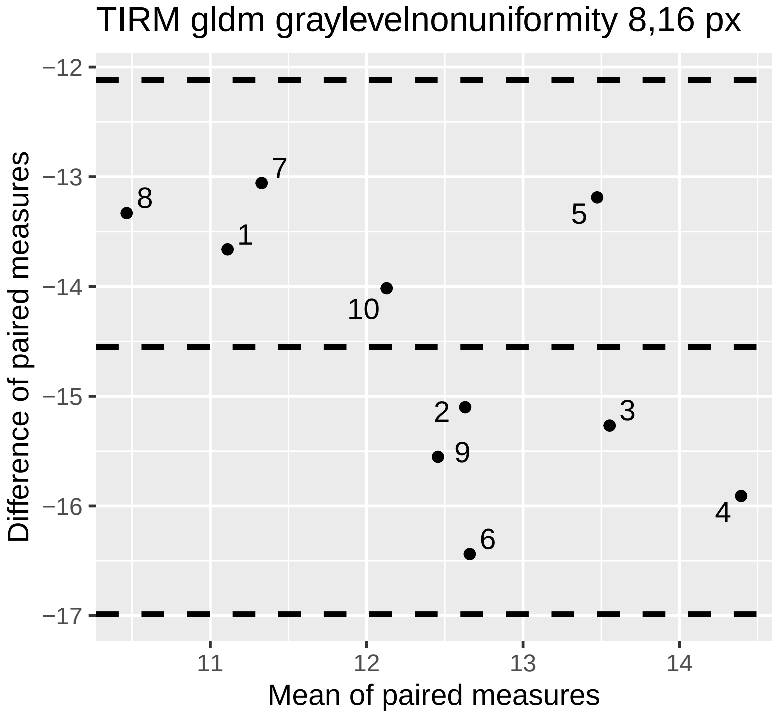
TIRM gldm graylevelnonuniformity 8,16 px
Difference of paired measures
Mean of paired measures
−12
−13
−14
−15
−16
−17
11
12
13
14

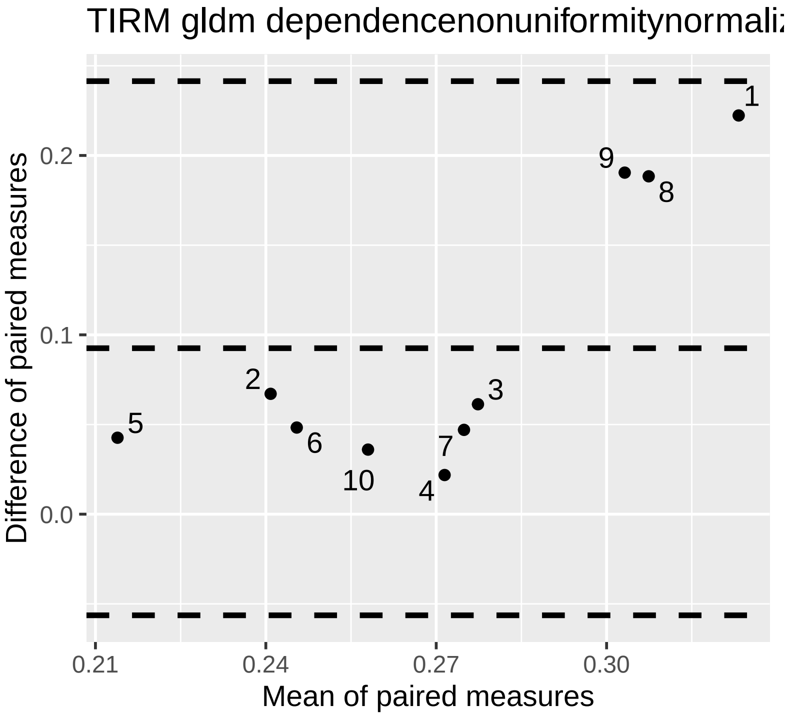
TIRM gldm dependencenonuniformitynormaliz
Difference of paired measures
Mean of paired measures
0.2
0.1
0.0
0.21
0.24
0.27
0.30

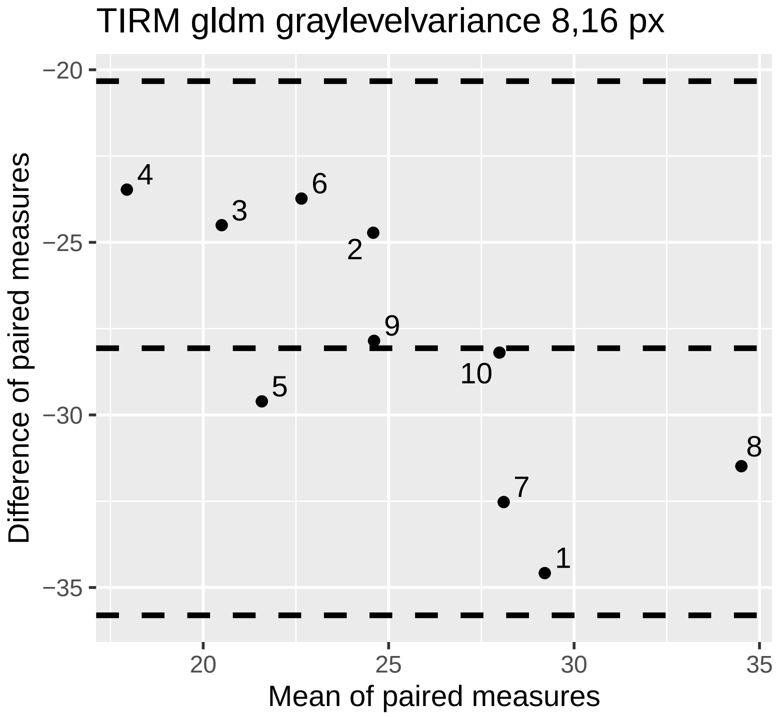
TIRM gldm graylevelvariance 8,16 px
Difference of paired measures
Mean of paired measures
−20
−25
−30
−35
20
25
30
35

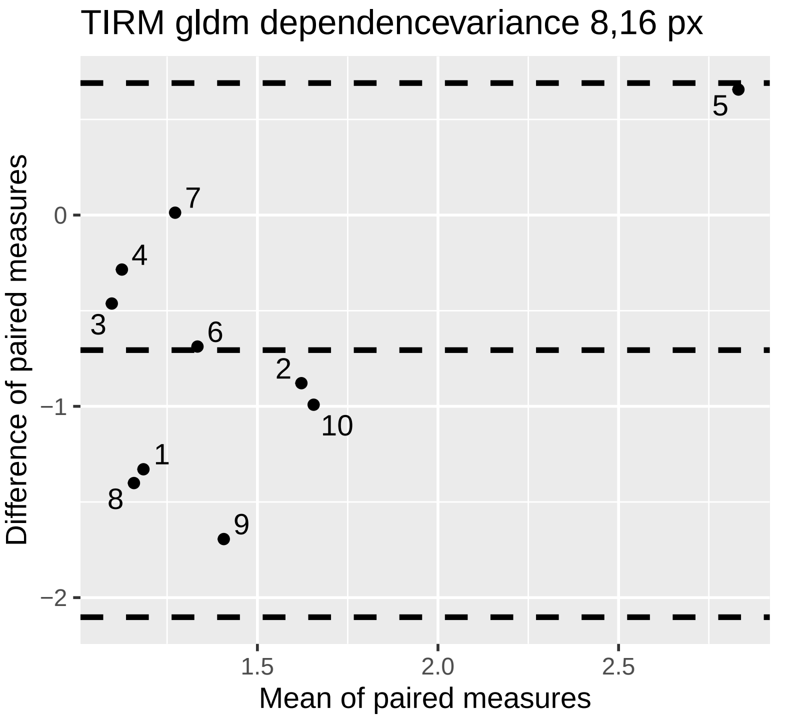
TIRM gldm dependencevariance 8,16 px
Difference of paired measures
Mean of paired measures
0
−1
−2
1.5
2.0
2.5

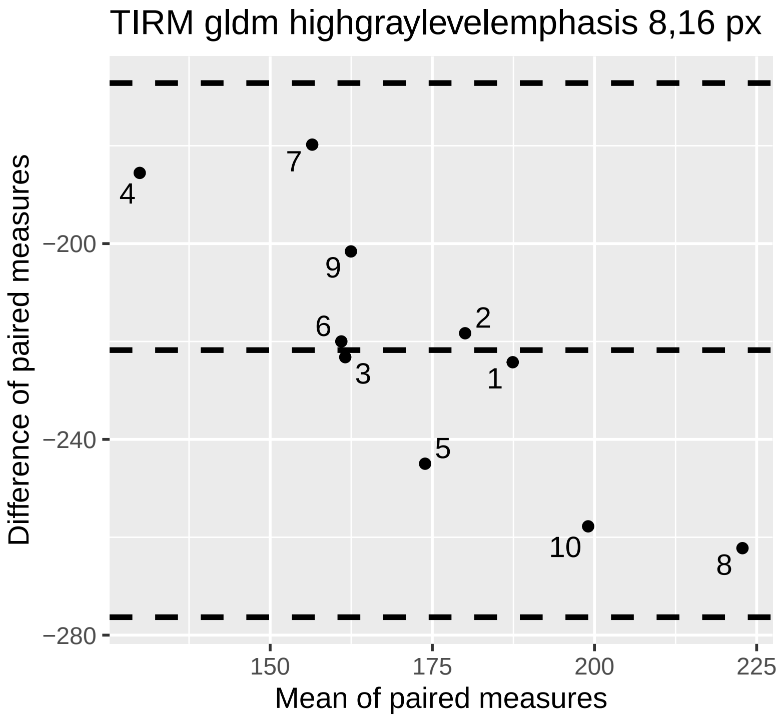
TIRM gldm highgraylevelemphasis 8,16 px
Difference of paired measures
Mean of paired measures
−200
−240
−280
150
175
200
225

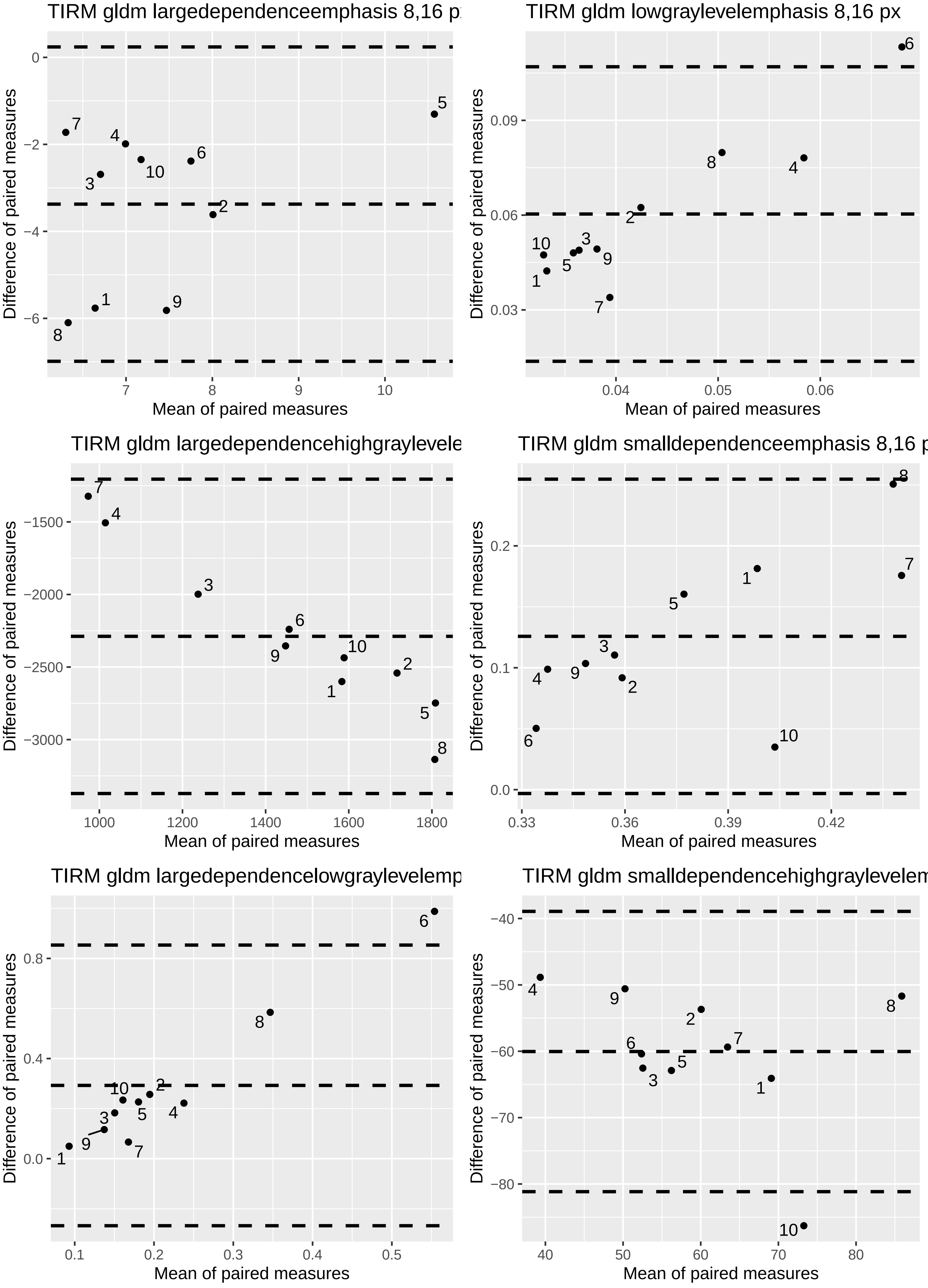

TIRM gldm largedependenceemphasis 8,16 px
Difference of paired measures
Mean of paired measures
TIRM gldm lowgraylevelemphasis 8,16 px
Difference of paired measures
Mean of paired measures
TIRM gldm largedependencehighgraylevele
Difference of paired measures
Mean of paired measures
TIRM gldm smalldependenceemphasis 8,16 p
Difference of paired measures
Mean of paired measures
TIRM gldm largedependencelowgraylevelemp
Difference of paired measures
Mean of paired measures
TIRM gldm smalldependencehighgraylevelem
Difference of paired measures
Mean of paired measures

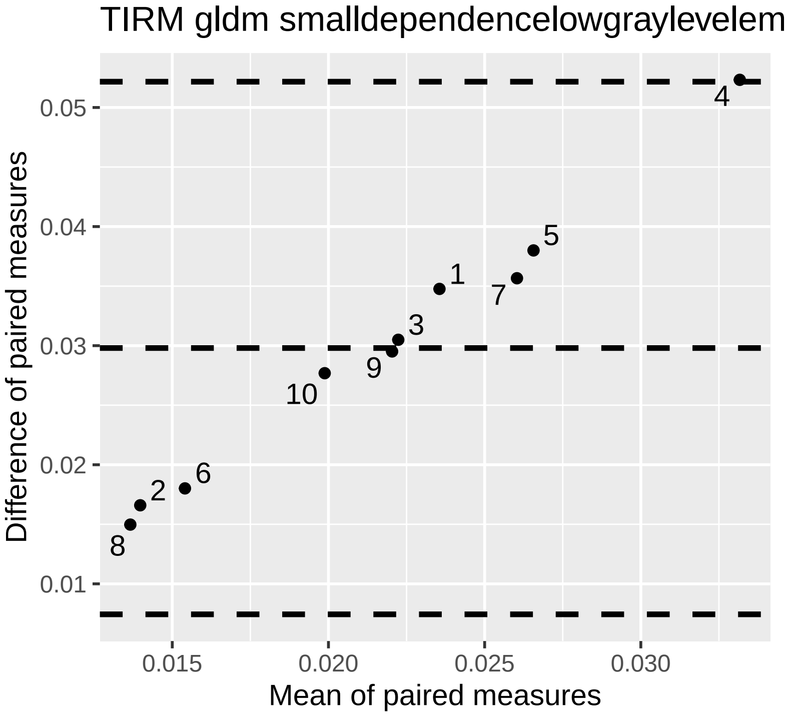
TIRM gldm smalldependencelowgraylevelem
Difference of paired measures
Mean of paired measures

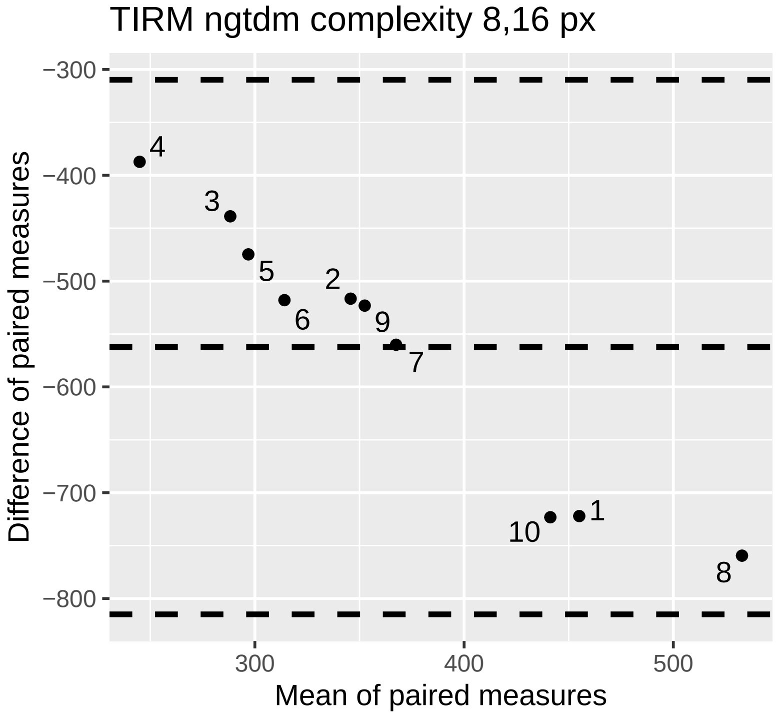
TIRM ngtdm complexity 8,16 px
Difference of paired measures
Mean of paired measures

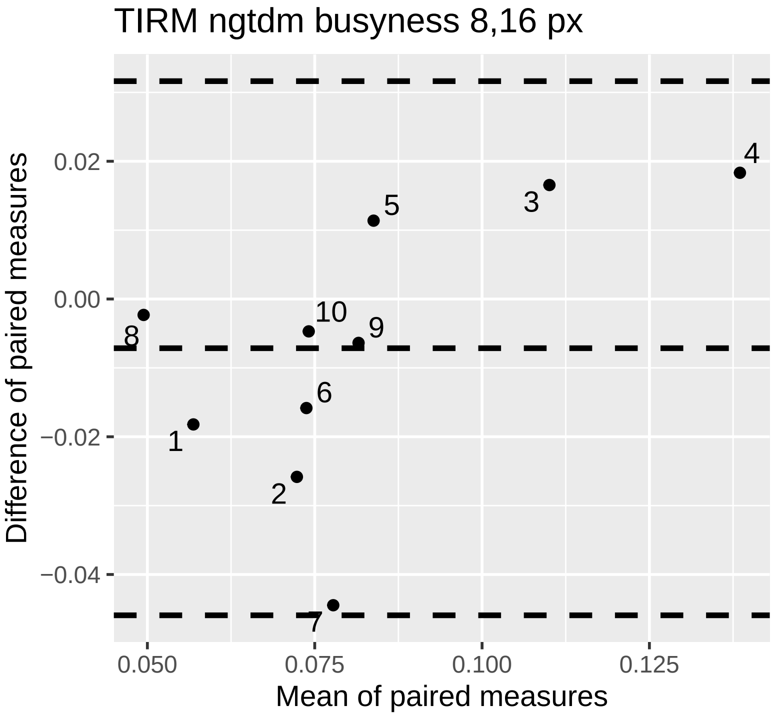
TIRM ngtdm busyness 8,16 px
Difference of paired measures
Mean of paired measures

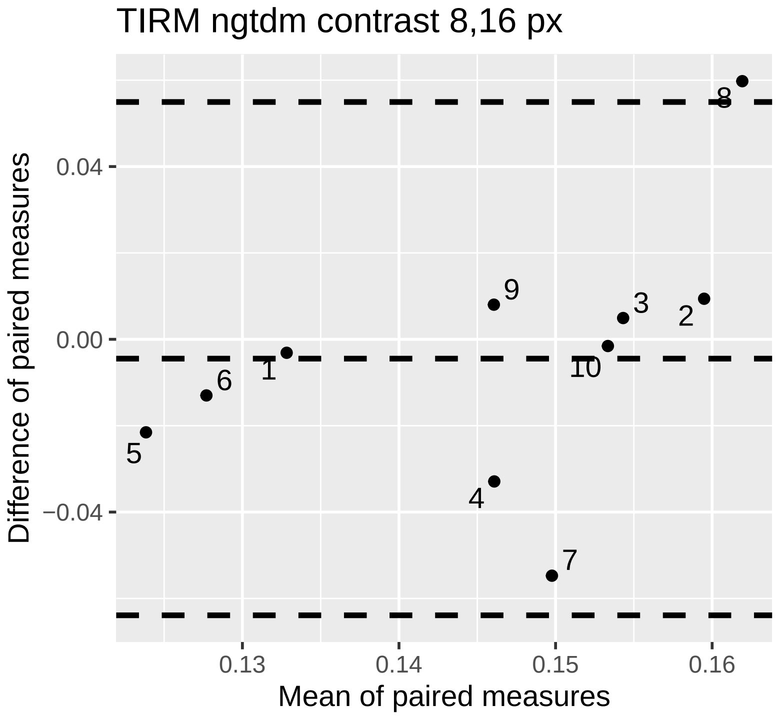
TIRM ngtdm contrast 8,16 px
Difference of paired measures
Mean of paired measures

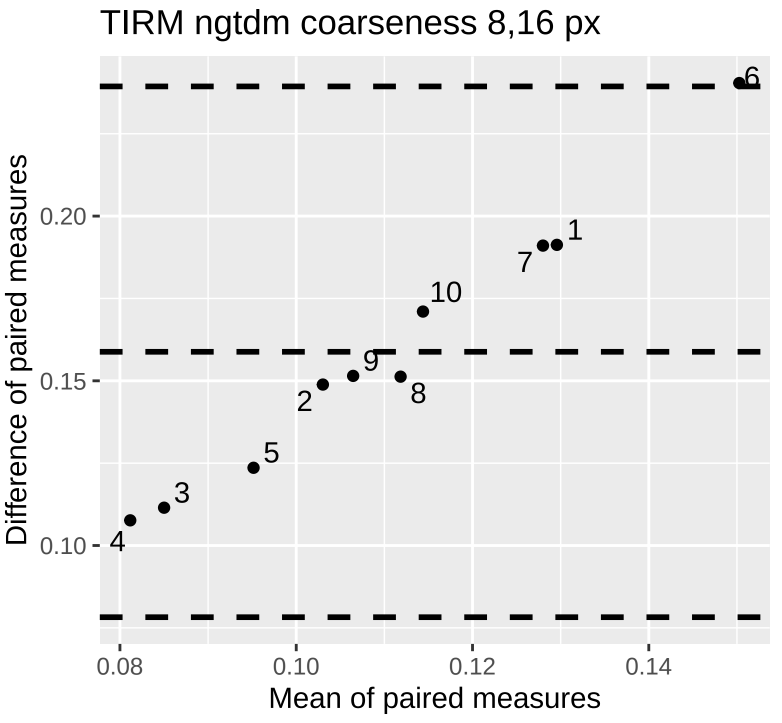
TIRM ngtdm coarseness 8,16 px
Difference of paired measures
Mean of paired measures

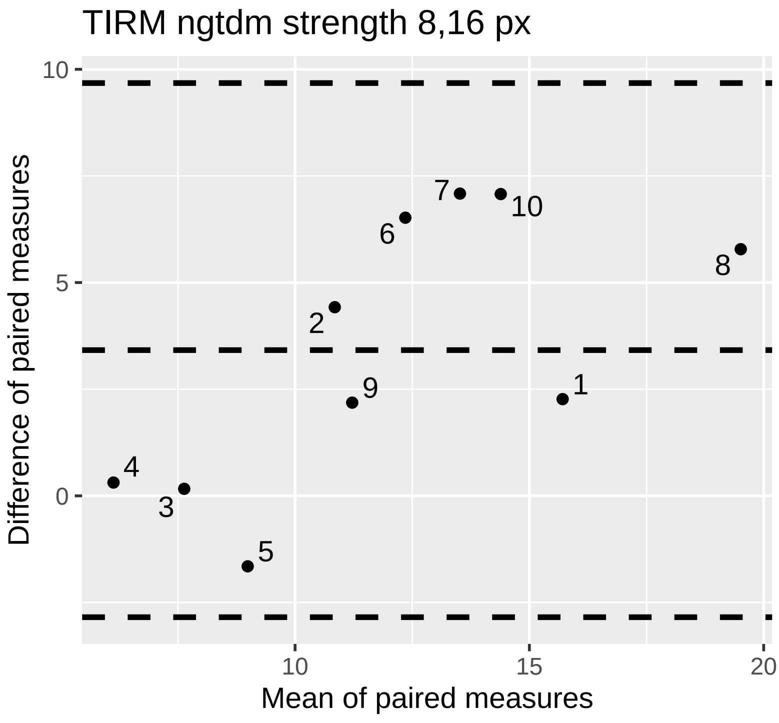
TIRM ngtdm strength 8,16 px
Difference of paired measures
Mean of paired measures
