# Supplementary material for: Stability of Radiomic Features across Different Region of Interest Sizes—A CT and MR Phantom Study
Source: Tomography. 2021 Jun 8;7(2):238–52. doi: 10.3390/tomography7020022 (PMC8293351; doi:10.3390/tomography7020022)
Supplement: Supplementary file 1 [file tomography-07-00022-s001.zip › table_S1.pdf]

T1w MR images: first-order features

feature	image_type	mri_sequence	mm_or_px	mean4	median4	mean8	median8	mean16	median16	mwu4.8	mwu4.16	mwu8.16	occc4.8.16	occc8.16
L1_original_firstorder_mean	MRI	T1	px	1036.27857	1031.10714	1036.97647	1031.2941	1038.9149	1033.20192	1	1	1	0.98763635	0.99370191
L1_original_firstorder_rootmeansquared	MRI	T1	px	1336.30033	1331.12324	1337.00808	1331.3214	1338.97934	1333.27256	1	1	1	0.98750917	0.99355609
L1_original_firstorder_median	MRI	T1	px	1035.55	1030.75	1036.3	1031	1038.7	1033	1	1	1	0.98072296	0.99172766
L1_original_firstorder_10percentile	MRI	T1	px	1027.08	1022.3	1026.2	1021.5	1021.91	1016.7	1	1	1	0.96756885	0.97397784
L1_original_firstorder_median	MRI	T1	mm	1033.05	1028.25	1039.6	1035	1040.1	1035.5	1	1	1	0.94650006	0.9801116
L1_original_firstorder_mean	MRI	T1	mm	1032.8931	1027.69828	1039.95903	1034.5859	1041.27678	1036.79427	1	1	1	0.93937712	0.98039055
L1_original_firstorder_rootmeansquared	MRI	T1	mm	1332.92801	1327.72531	1340.02702	1334.6574	1341.60201	1337.14516	1	1	1	0.93693332	0.97907918
L1_original_firstorder_90percentile	MRI	T1	px	1044.93	1038.95	1048.7	1042.5	1055.75	1050.8	1	1	1	0.9320733	0.94913014
L1_original_firstorder_minimum	MRI	T1	px	1023.7	1020.5	1016.6	1013	1005.2	1001.5	1	0.22408897	0.57331595	0.7827675	0.7839347
L1_original_firstorder_maximum	MRI	T1	px	1050.1	1046.5	1059.2	1055	1072.2	1065	1	0.08346806	1	0.75724134	0.83488801
L1_original_firstorder_10percentile	MRI	T1	mm	1020.75	1016.85	1022.82	1017.8	1003.05	999.5	1	0.18687342	0.09292004	0.75209283	0.65144133
L1_original_firstorder_90percentile	MRI	T1	mm	1044.64	1037.65	1057.88	1052.5	1080.9	1078.5	0.84010359	0.01311196	0.10203947	0.56422723	0.61479518
L1_original_firstorder_minimum	MRI	T1	mm	1010.9	1008.5	1005.7	1000.5	959.9	958.5	1	0.00123406	0.00292277	0.31908497	0.24370695
L1_original_firstorder_maximum	MRI	T1	mm	1056.8	1051.5	1074.1	1065.5	1124.5	1117.5	0.41659504	0.00045465	0.00685303	0.28843214	0.29837524
L1_original_firstorder_meanabsolutedeviation	MRI	T1	px	6.11836735	6.14795918	7.26143791	7.2168397	10.5991679	10.1831546	0.25954232	6.4951E-05	6.4951E-05	0.07980034	0.09791028
L1_original_firstorder_variance	MRI	T1	px	58.0709184	53.5127551	84.66213	84.559016	172.891177	158.748313	0.17283336	6.4951E-05	6.4951E-05	0.06874839	0.10587933
L1_original_firstorder_robustmeanabsolutedeviation	MRI	T1	px	4.09033058	4.53	5.2320366	5.3308725	7.66913004	7.32682628	0.01253545	6.4951E-05	6.4951E-05	0.05392819	0.0830371
L1_original_firstorder_range	MRI	T1	px	26.4	24.5	42.6	42	67	67.5	0.00454814	6.4951E-05	0.00125917	0.05209229	0.08683747
L1_original_firstorder_entropy	MRI	T1	px	2.34041553	2.32578551	2.78611537	2.777104	3.3830897	3.35467988	0.00045465	6.4951E-05	6.4951E-05	0.04199721	0.04976166
L1_original_firstorder_interquartilerange	MRI	T1	px	10.25	10.625	11.75	11.75	18.575	17.5	1	0.00103592	0.00098962	0.03474606	0.02338502
L1_original_firstorder_skewness	MRI	T1	mm	0.03623548	-0.0049792	0.04641476	0.0474293	0.11576092	0.12592319	1	1	1	0.02721194	-0.0651801
L1_original_firstorder_uniformity	MRI	T1	px	0.22755102	0.2244898	0.17085736	0.1730104	0.10930566	0.11076183	0.00146092	0.00108381	6.4951E-05	0.0265658	0.01790883
L1_original_firstorder_kurtosis	MRI	T1	px	2.26968332	2.37869303	2.99262136	3.0113473	2.70498159	2.67224234	0.00045465	0.06897746	0.17283336	0.02317362	0.2332649
L1_original_firstorder_robustmeanabsolutedeviation	MRI	T1	mm	5.41121789	5.22986457	7.99557347	7.6343496	17.8350549	19.1686389	0.0001299	6.4951E-05	6.4951E-05	0.01912245	0.01744138
L1_original_firstorder_meanabsolutedeviation	MRI	T1	mm	7.61848989	7.42003567	10.9450639	10.654583	24.1916692	25.3406771	6.4951E-05	6.4951E-05	6.4951E-05	0.0154724	0.01723723
L1_original_firstorder_interquartilerange	MRI	T1	mm	12.225	12.5	18.85	18	42.2	45.5	0.00106566	0.00107774	0.00105967	0.01510302	0.01686419
L1_original_firstorder_entropy	MRI	T1	mm	2.87354992	2.85505654	3.42951749	3.418453	4.56016463	4.61719212	6.4951E-05	6.4951E-05	6.4951E-05	0.01454931	0.01556855
L1_original_firstorder_uniformity	MRI	T1	mm	0.15951249	0.16171225	0.106115	0.1081527	0.04784796	0.04525364	0.00108991	0.00108991	6.4951E-05	0.01372521	0.01386873
L1_original_firstorder_variance	MRI	T1	mm	93.1900119	91.6507134	182.565072	176.83722	873.529375	926.619326	6.4951E-05	6.4951E-05	6.4951E-05	0.01044777	0.01324213
L1_original_firstorder_range	MRI	T1	mm	45.9	46.5	68.4	69	164.6	162	0.00107169	0.00108381	0.00108381	0.00492902	0.00522378
L1_original_firstorder_energy	MRI	T1	px	25005184.7	24806453.5	91186260	90393266	372997547	369744204	6.4951E-05	6.4951E-05	6.4951E-05	0.00043066	0.00082792
L1_original_firstorder_totalenergy	MRI	T1	px	30218668	29978502.2	110198239	109239909	450766078	446834426	6.4951E-05	6.4951E-05	6.4951E-05	0.00043066	0.00082792
L1_original_firstorder_energy	MRI	T1	mm	103070267	102245688	407705093	404357435	3395306463	3372087320	6.4951E-05	6.4951E-05	6.4951E-05	0.00016672	0.00028958
L1_original_firstorder_totalenergy	MRI	T1	mm	124560015	123563514	492710012	488664381	4103214597	4075154354	6.4951E-05	6.4951E-05	6.4951E-05	0.00016672	0.00028958
L1_original_firstorder_kurtosis	MRI	T1	mm	3.03284498	2.92600728	2.66196973	2.6475502	2.51687088	2.3093884	0.05358419	0.08813787	1	-0.0235212	-0.1669117
L1_original_firstorder_skewness	MRI	T1	px	0.10710725	0.11347417	0.24235945	0.3090438	0.0142816	-0.051429	1	1	0.11126026	-0.0339086	0.00307461

T1w MR images: GLCM-features

feature	image_type	mri_sequence	mm_or_px	mean4	median4	mean8	median8	mean16	median16	mwu4.8	mwu4.16	mwu8.16	occc4.8.16	occc8.16
L1_original_glcm_imc1	MRI	T1	mm	-0.2116019	-0.2082906	-0.1913943	-0.1878812	-0.193021	-0.2019988	1	1	1	0.47820236	0.47952812
L1_original_glcm_imc2	MRI	T1	mm	0.82690987	0.82614717	0.8468272	0.8459317	0.87240947	0.89330766	1	0.17283336	0.99296369	0.44617592	0.42467302
L1_original_glcm_imc2	MRI	T1	px	0.90512182	0.91333442	0.84236947	0.8613536	0.84472756	0.84438511	0.08813787	0.02331724	1	0.28836103	0.72935323
L1_original_glcm_id	MRI	T1	px	0.49233185	0.4984375	0.49965677	0.5015052	0.48287811	0.48026724	1	1	0.99296369	0.27131401	0.41154967
L1_original_glcm_mcc	MRI	T1	mm	0.58249574	0.58133899	0.68497374	0.6861442	0.66247217	0.65633538	0.0063002	0.04104873	1	0.25066187	0.41722181
L1_original_glcm_idm	MRI	T1	px	0.42790833	0.4365017	0.43704257	0.4373714	0.41606286	0.41200785	1	1	0.99296369	0.25016004	0.41437177
L1_original_glcm_differenceaverage	MRI	T1	px	1.625625	1.534375	1.60504696	1.594542	1.71625	1.73490991	1	1	0.45153608	0.17982541	0.43136236
L1_original_glcm_contrast	MRI	T1	px	4.288125	3.771875	4.27484496	4.2977918	4.84562078	4.9232897	1	0.73803286	0.31455541	0.15357481	0.39493955
L1_original_glcm_inversevariance	MRI	T1	px	0.4276224	0.45283854	0.42700793	0.4232717	0.41451354	0.41262117	1	1	1	0.13253413	0.5767077
L1_original_glcm_differencevariance	MRI	T1	px	1.34373438	1.24507813	1.62542369	1.682212	1.86000014	1.87948131	0.37807703	0.13938384	0.01727684	0.10144978	0.17202334
L1_original_glcm_correlation	MRI	T1	mm	0.33533413	0.32361296	0.64374654	0.6284485	0.5084634	0.50836515	6.4951E-05	0.00435169	6.4951E-05	0.06473463	0.03694784
L1_original_glcm_correlation	MRI	T1	px	0.09069045	0.08814762	0.35867138	0.3608902	0.63023352	0.63250312	0.01253545	6.4951E-05	6.4951E-05	0.0578487	0.07631213
L1_original_glcm_clustershade	MRI	T1	px	1.69795547	0.85966406	5.43265774	6.003501	7.83740451	7.25354153	0.25954232	1	1	0.05640185	0.19617071
L1_original_glcm_sumsquares	MRI	T1	px	2.42105859	2.39958984	3.41278377	3.4022421	6.69917676	6.23809607	0.17283336	6.4951E-05	6.4951E-05	0.05505042	0.12070322
L1_original_glcm_clustertendency	MRI	T1	px	5.39610938	5.88898438	9.37629011	9.1590864	21.9510863	20.1686744	0.05358419	6.4951E-05	6.4951E-05	0.05212857	0.10880416
L1_original_glcm_mcc	MRI	T1	px	0.78447742	0.78923497	0.60583228	0.6197817	0.6761135	0.67989512	0.00194852	0.05358419	0.21277793	0.04504646	0.35140829
L1_original_glcm_clustershade	MRI	T1	mm	1.29556942	2.1462643	12.1260291	6.5624121	103.875915	103.843253	1	6.4951E-05	0.00045465	0.03651866	0.03717846
L1_original_glcm_clusterprominence	MRI	T1	px	80.0874456	72.7641709	260.558847	266.73929	1297.66557	1134.51136	0.00902812	6.4951E-05	6.4951E-05	0.03607839	0.08556072
L1_original_glcm_jointaverage	MRI	T1	px	3.4878125	3.6703125	5.06373919	4.765298	7.7840266	7.41013338	0.0063002	6.4951E-05	0.0002598	0.03346018	-0.0035437
L1_original_glcm_sumaverage	MRI	T1	px	6.975625	7.340625	10.1274784	9.5305959	15.5680532	14.8202668	0.0063002	6.4951E-05	0.0002598	0.03346018	-0.0035437
L1_original_glcm_sumentropy	MRI	T1	px	2.46556732	2.57416893	3.35078211	3.3623857	4.14150169	4.09683723	6.4951E-05	6.4951E-05	6.4951E-05	0.02472121	0.06065593
L1_original_glcm_imc1	MRI	T1	px	-0.4318135	-0.4295263	-0.2330627	-0.2495141	-0.1913208	-0.1891011	6.4951E-05	6.4951E-05	0.05358419	0.02428859	0.35292747
L1_original_glcm_inversevariance	MRI	T1	mm	0.42516608	0.43324112	0.41674548	0.4177074	0.2087178	0.1778039	1	6.4951E-05	6.4951E-05	0.02052864	0.00147944
L1_original_glcm_sumentropy	MRI	T1	mm	3.4007459	3.37684556	4.18824524	4.1756247	5.21926786	5.27575793	6.4951E-05	6.4951E-05	6.4951E-05	0.01794007	0.02411158
L1_original_glcm_idn	MRI	T1	px	0.81150513	0.80636068	0.86517232	0.8624702	0.90009503	0.90118017	0.00045465	6.4951E-05	0.00045465	0.01785063	0.10103246
L1_original_glcm_clustertendency	MRI	T1	mm	9.58781251	8.99156376	23.0915298	22.051249	93.3569878	99.3894354	6.4951E-05	6.4951E-05	6.4951E-05	0.01570694	0.0221673
L1_original_glcm_maximumprobability	MRI	T1	px	0.149375	0.140625	0.09200842	0.0923049	0.04399528	0.04269426	0.00107169	0.00107774	0.00108991	0.01506916	0.03638291
L1_original_glcm_autocorrelation	MRI	T1	px	12.95375	13.646875	28.1148122	23.653358	65.7608896	59.4020728	0.00435169	6.4951E-05	0.0002598	0.01480756	-0.0116749
L1_original_glcm_differenceentropy	MRI	T1	px	1.84273689	1.87603459	2.17651259	2.1734914	2.32605456	2.33327009	0.00123406	6.4951E-05	0.00045465	0.01424916	0.14784716
L1_original_glcm_sumsquares	MRI	T1	mm	3.54139323	3.50272137	6.99130508	6.6542581	30.5553249	32.5989984	6.4951E-05	6.4951E-05	6.4951E-05	0.01137104	0.0154984
L1_original_glcm_idmn	MRI	T1	px	0.91415504	0.91314043	0.95685401	0.9559353	0.97814172	0.97877124	0.00077941	6.4951E-05	0.0001299	0.00997672	0.08014083
L1_original_glcm_jointentropy	MRI	T1	px	3.57179769	3.59533905	4.88976912	4.8871066	6.06825216	6.04681322	6.4951E-05	6.4951E-05	6.4951E-05	0.00939342	0.02244311
L1_original_glcm_jointenergy	MRI	T1	px	0.09497656	0.09058594	0.04188705	0.0428842	0.01902757	0.01898952	6.4951E-05	6.4951E-05	6.4951E-05	0.00924881	0.02364675
L1_original_glcm_clusterprominence	MRI	T1	mm	276.517956	226.487432	1435.82022	1231.1203	23549.3527	25601.6802	6.4951E-05	6.4951E-05	6.4951E-05	0.00683694	0.01146228
L1_original_glcm_jointentropy	MRI	T1	mm	5.04741347	5.05355939	6.13366416	6.0882556	8.05910097	8.1522203	6.4951E-05	6.4951E-05	6.4951E-05	0.00655352	0.00936376
L1_original_glcm_jointenergy	MRI	T1	mm	0.03771379	0.03808258	0.01816228	0.0187501	0.00484898	0.00446566	6.4951E-05	6.4951E-05	6.4951E-05	0.00461543	0.00666808
L1_original_glcm_differenceentropy	MRI	T1	mm	2.21875808	2.21882006	2.32845041	2.3327381	3.11632978	3.10296874	0.01253545	6.4951E-05	6.4951E-05	0.00388349	0.0027938
L1_original_glcm_jointaverage	MRI	T1	mm	5.28970231	5.64621007	7.78790025	7.5904659	17.2177977	16.7155143	6.4951E-05	6.4951E-05	6.4951E-05	0.00312273	-0.0105358
L1_original_glcm_sumaverage	MRI	T1	mm	10.5794046	11.2924201	15.5758005	15.180932	34.4355953	33.4310286	6.4951E-05	6.4951E-05	6.4951E-05	0.00312273	-0.0105358
L1_original_glcm_contrast	MRI	T1	mm	4.57776043	4.41237578	4.87369048	4.8220443	28.8643119	31.705282	0.73803286	6.4951E-05	6.4951E-05	0.00114137	0.00125203
L1_original_glcm_autocorrelation	MRI	T1	mm	29.8409401	33.1553394	65.7948317	62.01874	314.883537	294.046632	6.4951E-05	6.4951E-05	6.4951E-05	0.0007796	-0.0078006
L1_original_glcm_differenceaverage	MRI	T1	mm	1.66068146	1.63836957	1.72401067	1.7044335	4.3716661	4.73316153	0.99296369	6.4951E-05	6.4951E-05	-0.0003217	0.00048517
L1_original_glcm_differencevariance	MRI	T1	mm	1.74821904	1.65994964	1.86150335	1.8822051	6.00219149	5.38651869	0.25954232	6.4951E-05	6.4951E-05	-0.0004806	-0.001304
L1_original_glcm_maximumprobability	MRI	T1	mm	0.08210515	0.08215062	0.04170977	0.041133	0.01188714	0.01131564	6.4951E-05	6.4951E-05	6.4951E-05	-0.0009915	-0.0050058
L1_original_glcm_idm	MRI	T1	mm	0.42697517	0.4244147	0.41405758	0.4174484	0.20426729	0.17180835	1	6.4951E-05	6.4951E-05	-0.0027074	-0.002831
L1_original_glcm_id	MRI	T1	mm	0.49111798	0.48981001	0.48096737	0.4829835	0.29668272	0.26842484	1	6.4951E-05	6.4951E-05	-0.0054357	-0.0025512
L1_original_glcm_idmn	MRI	T1	mm	0.95896023	0.96152214	0.97893861	0.979063	0.97644256	0.97604746	6.4951E-05	6.4951E-05	1	-0.0446943	-0.1453246
L1_original_glcm_idn	MRI	T1	mm	0.86770933	0.87004243	0.90128475	0.9013491	0.89168259	0.88656741	6.4951E-05	0.00435169	1	-0.0681244	-0.1254339

T1w MR images: GLDM-features

feature	image_type	mri_sequence	mm_or_px	mean4	median4	mean8	median8	mean16	median16	mwu4.8	mwu4.16	mwu8.16	occc4.8.16	occc8.16
L1_original_gldm_smalldependenceemphasis	MRI	T1	px	0.51442262	0.51165675	0.41297222	0.4071732	0.38180882	0.39035256	0.25954232	0.05358419	1	0.21143371	0.51981289
L1_original_gldm_largedependenceemphasis	MRI	T1	px	4.85714286	4.64285714	6.52941176	6.7058824	6.63461538	6.53846154	0.03117625	0.00123406	1	0.14513674	0.36454773
L1_original_gldm_dependencevariance	MRI	T1	px	0.93877551	0.7755102	1.26120723	1.2287582	1.21767751	1.24237241	0.45330951	0.841895	1	0.1373906	0.0068719
L1_original_gldm_largedependencelowgraylevelemphasis	MRI	T1	px	0.96827984	0.73345704	0.73753353	0.4464223	0.18613258	0.17174284	0.85884085	6.4951E-05	0.05358419	0.13466516	0.02929254
L1_original_gldm_dependencenonuniformitynormalized	MRI	T1	px	0.38061224	0.36734694	0.27543253	0.2764321	0.26531527	0.26497781	0.00346374	0.00146883	1	0.11177773	0.33104591
L1_original_gldm_dependencevariance	MRI	T1	mm	1.2117717	1.20868014	1.23154728	1.2011683	1.83789152	1.49598931	1	0.02158732	0.0063002	0.10200983	-0.0050875
L1_original_gldm_smalldependencehighgraylevelemphasis	MRI	T1	px	9.41220437	9.14285714	14.5875784	13.046092	26.6597731	25.1760897	0.17283336	0.0002598	0.0063002	0.10019979	0.03744153
L1_original_gldm_lowgraylevelemphasis	MRI	T1	px	0.20923619	0.20101656	0.10625553	0.0758936	0.03545707	0.03836252	0.02331724	6.4951E-05	0.00077941	0.06411427	-0.0053943
L1_original_gldm_graylevelvariance	MRI	T1	px	2.39897959	2.42346939	3.49396386	3.4702038	6.99111733	6.49180612	0.13938384	6.4951E-05	6.4951E-05	0.05411126	0.09823739
L1_original_gldm_highgraylevelemphasis	MRI	T1	px	15.4857143	17.3571429	30.6921569	26.882353	68.4456731	62.2956731	0.0063002	6.4951E-05	0.0002598	0.02198259	-0.0105117
L1_original_gldm_largedependencelowgraylevelemphasis	MRI	T1	mm	0.42961881	0.34825374	0.17162088	0.1681648	0.04505818	0.04706903	0.00077941	6.4951E-05	6.4951E-05	0.0123139	-0.0151817
L1_original_gldm_smalldependencelowgraylevelemphasis	MRI	T1	px	0.10853715	0.10968499	0.04824033	0.0426935	0.01810366	0.0185636	0.00194852	6.4951E-05	6.4951E-05	0.01023528	-0.0421868
L1_original_gldm_graylevelvariance	MRI	T1	mm	3.79036861	3.67449465	7.36790157	7.094374	34.9881566	37.1269566	6.4951E-05	6.4951E-05	6.4951E-05	0.00950006	0.01246247
L1_original_gldm_largedependencehighgraylevelemphasis	MRI	T1	px	59.5571429	66.8571429	170.998039	147.37255	448.566827	410.247596	0.00077941	6.4951E-05	0.0001299	0.00948752	-0.0063034
L1_original_gldm_dependenceentropy	MRI	T1	mm	4.26965065	4.30578175	5.21929835	5.2260107	6.74342548	6.71182312	6.4951E-05	6.4951E-05	6.4951E-05	0.0051298	0.00520843
L1_original_gldm_smalldependencehighgraylevelemphasis	MRI	T1	mm	14.2566614	12.9176461	27.5316246	25.031115	108.552576	105.738196	0.00077941	6.4951E-05	6.4951E-05	0.00416562	-0.0062516
L1_original_gldm_graylevelnonuniformity	MRI	T1	mm	9.25172414	9.37931034	24.0881057	24.550661	90.2412513	85.3483563	0.00108991	0.00108991	6.4951E-05	0.00303253	0.00460176
L1_original_gldm_dependencenonuniformity	MRI	T1	px	5.32857143	5.14285714	14.0470588	14.098039	55.1855769	55.1153846	0.00108381	0.00108991	0.00108991	0.00234627	0.0026799
L1_original_gldm_graylevelnonuniformity	MRI	T1	px	3.18571429	3.14285714	8.71372549	8.8235294	22.7355769	23.0384615	0.00107774	0.00107774	6.4951E-05	0.0022102	0.00379172
L1_original_gldm_highgraylevelemphasis	MRI	T1	mm	32.2034483	35.25	68.6462555	64.550661	335.068611	314.396341	6.4951E-05	6.4951E-05	6.4951E-05	0.00159958	-0.0060556
L1_original_gldm_dependenceentropy	MRI	T1	px	2.95882644	3.0121884	4.14103095	4.1505376	5.15880779	5.13402596	0.00108381	0.00108381	6.4951E-05	0.00083265	0.0078604
L1_original_gldm_dependencenonuniformity	MRI	T1	mm	16.8	16.6724138	59.9867841	59.770925	427.120891	463.956522	6.4951E-05	6.4951E-05	6.4951E-05	-0.000204	7.6089E-05
L1_original_gldm_smalldependencelowgraylevelemphasis	MRI	T1	mm	0.05064862	0.0460265	0.01991117	0.0193554	0.00292983	0.00291628	6.4951E-05	6.4951E-05	6.4951E-05	-0.0012322	-0.013425
L1_original_gldm_lowgraylevelemphasis	MRI	T1	mm	0.09451444	0.07858674	0.03554246	0.0347022	0.00661112	0.00675929	6.4951E-05	6.4951E-05	6.4951E-05	-0.0050752	-0.0165712
L1_original_gldm_largedependencehighgraylevelemphasis	MRI	T1	mm	193.162069	181.5	441.546696	435.15859	3077.24199	3027.96129	6.4951E-05	6.4951E-05	6.4951E-05	-0.0068385	-0.0078646
L1_original_gldm_largedependenceemphasis	MRI	T1	mm	6.3	6.72413793	6.56651982	6.4845815	9.01749735	7.58271474	1	0.00456911	0.00459014	-0.0433761	-0.0215638
L1_original_gldm_smalldependenceemphasis	MRI	T1	mm	0.41493582	0.41584531	0.39107691	0.3962023	0.32632687	0.34874712	1	0.11126026	0.00292277	-0.0638254	-0.0558544
L1_original_gldm_dependencenonuniformitynormalized	MRI	T1	mm	0.28965517	0.28745541	0.26425896	0.263308	0.22646919	0.24600028	0.03117625	6.4951E-05	0.00435169	-0.0840961	0.00956418

T1w MR images: GLRLM-features

feature	image_type	mri_sequence	mm_or_px	mean4	median4	mean8	median8	mean16	median16	mwu4.8	mwu4.16	mwu8.16	occc4.8.16	occc8.16
L1_original_glrlm_shortrunemphasis	MRI	T1	px	0.89796005	0.89182692	0.87392023	0.8712086	0.87010789	0.87095208	0.22538083	0.01316258	1	0.28581105	0.44614424
L1_original_glrlm_runlengthnonuniformitynormalized	MRI	T1	px	0.77721115	0.75905063	0.72744094	0.7200013	0.71489962	0.71601903	0.2699582	0.00195861	1	0.27497008	0.46038884
L1_original_glrlm_runpercentage	MRI	T1	px	0.87857143	0.875	0.83921569	0.8357843	0.834375	0.83473558	0.04305059	0.00341421	1	0.1779704	0.46889029
L1_original_glrlm_longrunemphasis	MRI	T1	px	1.46001166	1.43269231	1.66765508	1.680525	1.67971289	1.66829853	0.03455178	0.02151171	1	0.15851839	0.49628252
L1_original_glrlm_runvariance	MRI	T1	px	0.13860276	0.12191815	0.2307817	0.2407387	0.23633352	0.22801258	0.00785654	0.00602134	1	0.09338856	0.53499582
L1_original_glrlm_longrunlowgraylevelemphasis	MRI	T1	px	0.30304585	0.26860588	0.17949922	0.1261909	0.05531494	0.05796201	0.13938384	6.4951E-05	0.00194852	0.08370084	0.01470321
L1_original_glrlm_graylevelvariance	MRI	T1	px	2.54078457	2.53558713	3.7382599	3.7754867	7.26259891	6.66959566	0.06897746	6.4951E-05	6.4951E-05	0.06793494	0.10424465
L1_original_glrlm_lowgraylevelrunemphasis	MRI	T1	px	0.21148719	0.20086817	0.10666948	0.078362	0.03704801	0.03987637	0.00902812	6.4951E-05	6.4951E-05	0.05220725	-0.017157
L1_original_glrlm_graylevelnonuniformitynormalized	MRI	T1	px	0.21471731	0.21897559	0.16117185	0.1638194	0.10661106	0.10902356	0.00045465	6.4951E-05	6.4951E-05	0.04041708	0.03486719
L1_original_glrlm_shortrunlowgraylevelemphasis	MRI	T1	px	0.1904976	0.17723587	0.09234752	0.0701154	0.03338867	0.03568866	0.00194852	6.4951E-05	6.4951E-05	0.03567768	-0.0315382
L1_original_glrlm_shortrunhighgraylevelemphasis	MRI	T1	px	14.7281277	15.8527072	28.0919598	24.990398	59.9055341	54.3728133	0.01253545	6.4951E-05	0.00045465	0.0274593	-0.0128508
L1_original_glrlm_highgraylevelrunemphasis	MRI	T1	px	15.9555619	17.4891255	31.4330859	28.069389	68.6830688	62.3647025	0.0063002	6.4951E-05	0.0002598	0.02257461	-0.0149808
L1_original_glrlm_runentropy	MRI	T1	mm	3.45650499	3.50230736	4.1245888	4.1278608	4.87065387	4.88101779	6.4951E-05	6.4951E-05	6.4951E-05	0.0191024	0.02802895
L1_original_glrlm_graylevelnonuniformitynormalized	MRI	T1	mm	0.15139103	0.15295041	0.10356795	0.1050023	0.04754568	0.04508841	6.4951E-05	6.4951E-05	6.4951E-05	0.01667146	0.01590606
L1_original_glrlm_longrunhighgraylevelemphasis	MRI	T1	px	21.3727065	24.0347985	48.504114	43.026806	114.368098	106.700507	0.00123406	6.4951E-05	0.0001299	0.01563818	-0.0131765
L1_original_glrlm_runentropy	MRI	T1	px	2.67239241	2.69145998	3.38184594	3.4250969	4.07958924	4.08281864	6.4951E-05	6.4951E-05	6.4951E-05	0.01321361	0.02717878
L1_original_glrlm_graylevelvariance	MRI	T1	mm	4.07982187	3.94121469	7.67881554	7.4240009	35.3337084	37.6261908	6.4951E-05	6.4951E-05	6.4951E-05	0.01003219	0.01337205
L1_original_glrlm_shortrunemphasis	MRI	T1	mm	0.87670385	0.88177133	0.87206699	0.872831	0.95042088	0.95653872	1	6.4951E-05	6.4951E-05	0.00339445	-0.0013659
L1_original_glrlm_graylevelnonuniformity	MRI	T1	mm	7.41913686	7.44334186	19.6642916	19.803693	83.8442012	80.1874119	6.4951E-05	6.4951E-05	6.4951E-05	0.00289911	0.00403664
L1_original_glrlm_graylevelnonuniformity	MRI	T1	px	2.64433816	2.65480769	6.89501876	6.9153865	18.4980657	18.8443237	6.4951E-05	6.4951E-05	6.4951E-05	0.00263224	0.00410303
L1_original_glrlm_longrunhighgraylevelemphasis	MRI	T1	mm	52.1226112	56.4344303	113.490183	111.92769	420.334263	401.80567	6.4951E-05	6.4951E-05	6.4951E-05	0.002022	-0.0080377
L1_original_glrlm_highgraylevelrunemphasis	MRI	T1	mm	32.606369	35.5982636	68.8177095	64.015529	334.442112	313.587592	6.4951E-05	6.4951E-05	6.4951E-05	0.00156533	-0.0060261
L1_original_glrlm_shortrunhighgraylevelemphasis	MRI	T1	mm	28.8491308	30.8645815	60.2099463	54.836572	317.619501	296.265261	6.4951E-05	6.4951E-05	6.4951E-05	0.00136523	-0.0056957
L1_original_glrlm_runlengthnonuniformity	MRI	T1	px	9.66566434	9.39498834	31.3590774	30.820295	124.389271	124.596475	0.00108991	0.00108991	6.4951E-05	0.00126838	0.00260493
L1_original_glrlm_runlengthnonuniformity	MRI	T1	mm	36.0184436	36.4628545	136.800818	136.86373	1582.50743	1627.53089	6.4951E-05	6.4951E-05	6.4951E-05	-3.887E-05	-2.942E-05
L1_original_glrlm_runlengthnonuniformitynormalized	MRI	T1	mm	0.73121354	0.74001293	0.71854202	0.7182387	0.88724697	0.9033071	1	6.4951E-05	6.4951E-05	-0.0006925	-0.0021995
L1_original_glrlm_longrunlowgraylevelemphasis	MRI	T1	mm	0.14024166	0.11947989	0.05422026	0.0536043	0.00801304	0.00831226	0.0001299	6.4951E-05	6.4951E-05	-0.0053571	-0.0124323
L1_original_glrlm_lowgraylevelrunemphasis	MRI	T1	mm	0.09926958	0.08454934	0.03770799	0.0372191	0.00672686	0.00690934	6.4951E-05	6.4951E-05	6.4951E-05	-0.0061865	-0.0161829
L1_original_glrlm_shortrunlowgraylevelemphasis	MRI	T1	mm	0.0905962	0.07740186	0.03445975	0.0340442	0.00647439	0.00660836	6.4951E-05	6.4951E-05	6.4951E-05	-0.0064096	-0.0175598
L1_original_glrlm_runpercentage	MRI	T1	mm	0.84396552	0.84267241	0.83656388	0.8386564	0.93593686	0.94357207	1	0.00108991	0.00108991	-0.0075898	-0.0025497
L1_original_glrlm_longrunemphasis	MRI	T1	mm	1.63631786	1.65449844	1.66852279	1.6536202	1.25281671	1.23561818	1	6.4951E-05	6.4951E-05	-0.013065	-0.003511
L1_original_glrlm_runvariance	MRI	T1	mm	0.21709664	0.22213708	0.23290189	0.2310267	0.08886102	0.08512454	1	6.4951E-05	6.4951E-05	-0.0295906	-0.0050597

T1w MR images: GLSZM-features

feature	image_type	mri_sequence	mm_or_px	mean4	median4	mean8	median8	mean16	median16	mwu4.8	mwu4.16	mwu8.16	occc4.8.16	occc8.16
L1_original_glszm_smallareaemphasis	MRI	T1	px	0.70936748	0.7191358	0.65185524	0.6664364	0.61421781	0.6181103	1	0.53311745	0.31455541	0.32258105	0.52673419
L1_original_glszm_sizezonenonuniformitynormalized	MRI	T1	px	0.54379509	0.50617284	0.41533152	0.4285294	0.36036196	0.36489792	0.3228742	0.0019688	0.31455541	0.18794672	0.40111686
L1_original_glszm_largearealowgraylevelemphasis	MRI	T1	px	0.79069177	0.55508378	0.71049936	0.4546576	0.22320062	0.21677078	1	0.00123406	0.03117625	0.17363615	0.05026191
L1_original_glszm_largeareaemphasis	MRI	T1	px	3.84064935	3.30555556	7.35941441	7.4823718	8.15388657	7.92306122	0.01019637	0.00108991	1	0.17333158	0.64867601
L1_original_glszm_zonepercentage	MRI	T1	px	0.62142857	0.64285714	0.5	0.5098039	0.47211538	0.47596154	0.06469418	0.00315832	1	0.16390227	0.52480382
L1_original_glszm_zonevariance	MRI	T1	px	1.10226465	0.71757984	3.185157	3.3007427	3.6100026	3.39937618	0.00891945	0.0019688	1	0.15519255	0.52796705
L1_original_glszm_graylevelvariance	MRI	T1	px	2.94373712	2.76571429	4.70019993	5.0022184	8.46408865	8.26968889	0.04104873	6.4951E-05	6.4951E-05	0.10014951	0.13395071
L1_original_glszm_smallareaemphasis	MRI	T1	mm	0.63659326	0.62908812	0.62731373	0.6269775	0.59167627	0.58940444	1	0.73803286	0.08813787	0.082408	-0.0660963
L1_original_glszm_graylevelnonuniformitynormalized	MRI	T1	px	0.2023798	0.19677501	0.14140999	0.1373203	0.0982375	0.09885663	0.01316258	0.00108991	6.4951E-05	0.07282432	0.09716644
L1_original_glszm_sizezonenonuniformitynormalized	MRI	T1	mm	0.40647451	0.38431776	0.3724862	0.3724637	0.33146786	0.33031474	1	0.13938384	0.04104873	0.06575087	-0.078462
L1_original_glszm_smallareahighgraylevelemphasis	MRI	T1	px	13.4356375	12.7570988	24.1400014	21.700702	43.525754	40.4353853	0.02331724	6.4951E-05	0.00292277	0.05918965	-0.0115511
L1_original_glszm_largearealowgraylevelemphasis	MRI	T1	mm	0.45225321	0.42656534	0.21538834	0.2255919	0.08521245	0.07139295	0.00435169	6.4951E-05	0.00194852	0.03709864	0.0244175
L1_original_glszm_sizezonenonuniformity	MRI	T1	px	4.85183261	4.55555556	10.7583079	10.585284	35.5255657	37.1959184	0.00602134	0.00108991	6.4951E-05	0.02426322	0.0380435
L1_original_glszm_highgraylevelzoneemphasis	MRI	T1	px	17.1962229	17.4222222	34.3445104	33.040385	70.0786013	63.8946314	0.00902812	6.4951E-05	0.00045465	0.02354178	-0.026988
L1_original_glszm_graylevelnonuniformitynormalized	MRI	T1	mm	0.13195309	0.13072705	0.09493572	0.0943676	0.04408654	0.04247377	6.4951E-05	6.4951E-05	6.4951E-05	0.02159266	0.01710138
L1_original_glszm_lowgraylevelzoneemphasis	MRI	T1	px	0.21826308	0.19051713	0.11641034	0.0983948	0.044304	0.04620914	0.00435169	6.4951E-05	0.0001299	0.01998031	-0.0593655
L1_original_glszm_zoneentropy	MRI	T1	px	2.77486411	2.77867746	3.81982532	3.8628191	5.00271416	5.03488367	0.00108381	0.00108991	0.00108991	0.01417578	0.01566064
L1_original_glszm_largeareahighgraylevelemphasis	MRI	T1	px	49.0109776	44.5181818	188.850113	187.64967	545.050653	481.301923	6.4951E-05	6.4951E-05	6.4951E-05	0.01255151	-0.0027118
L1_original_glszm_graylevelnonuniformity	MRI	T1	px	1.74525974	1.68571429	3.60442524	3.6025641	9.63117894	9.76576923	0.00146883	0.00108991	6.4951E-05	0.0124245	0.01967564
L1_original_glszm_graylevelvariance	MRI	T1	mm	5.19762331	5.07912147	9.00151421	9.0380899	40.6834507	41.6087239	6.4951E-05	6.4951E-05	6.4951E-05	0.00950848	0.01268558
L1_original_glszm_smallarealowgraylevelemphasis	MRI	T1	mm	0.08607554	0.09187597	0.03693047	0.0370544	0.00610619	0.00608154	0.0001299	6.4951E-05	6.4951E-05	0.00470138	-0.0197739
L1_original_glszm_zoneentropy	MRI	T1	mm	4.03852153	4.08759826	5.04355592	5.0734999	6.59960674	6.59585685	6.4951E-05	6.4951E-05	6.4951E-05	0.00448717	0.00574293
L1_original_glszm_highgraylevelzoneemphasis	MRI	T1	mm	33.8400934	35.4430199	69.8657912	64.259047	328.317729	307.524721	6.4951E-05	6.4951E-05	6.4951E-05	0.00331559	-0.0033961
L1_original_glszm_smallareahighgraylevelemphasis	MRI	T1	mm	22.4887811	20.5564106	44.4534151	41.798703	193.561611	180.268874	6.4951E-05	6.4951E-05	6.4951E-05	0.00141504	-0.0060257
L1_original_glszm_graylevelnonuniformity	MRI	T1	mm	3.91408466	3.54440961	10.3169614	10.321716	32.9436064	33.1886732	6.4951E-05	6.4951E-05	6.4951E-05	-0.0005042	-0.0004779
L1_original_glszm_sizezonenonuniformity	MRI	T1	mm	12.3328866	11.5250278	40.6814075	41.515695	251.209909	270.127877	6.4951E-05	6.4951E-05	6.4951E-05	-0.0029296	-0.0026649
L1_original_glszm_lowgraylevelzoneemphasis	MRI	T1	mm	0.11709386	0.11821975	0.04701752	0.0472685	0.00865241	0.00860258	6.4951E-05	6.4951E-05	6.4951E-05	-0.0054865	-0.0198747
L1_original_glszm_zonevariance	MRI	T1	mm	2.89700346	2.96119038	3.57596734	3.2858561	12.0458981	6.44077866	1	0.00077941	0.00077941	-0.0074517	0.03009657
L1_original_glszm_smallarealowgraylevelemphasis	MRI	T1	px	0.1520165	0.1641259	0.08216351	0.0722942	0.03278773	0.032365	0.11126026	0.0001299	6.4951E-05	-0.0080738	-0.0629918
L1_original_glszm_largeareahighgraylevelemphasis	MRI	T1	mm	226.872917	204.907001	522.812239	532.14432	6335.44141	4623.55246	0.00045465	6.4951E-05	6.4951E-05	-0.0088935	-0.0051426
L1_original_glszm_largeareaemphasis	MRI	T1	mm	6.97553223	6.68965517	7.96085783	7.8968407	18.6158685	11.4952753	0.73803286	0.00123406	0.0002598	-0.0354897	0.02076031
L1_original_glszm_zonepercentage	MRI	T1	mm	0.50862069	0.51724138	0.47973568	0.4801762	0.39994698	0.43186638	1	0.03422267	0.00262473	-0.0902933	-0.0292022

T1w MR images: NGTDM-features

feature	image_type	mri_sequence	mm_or_px	mean4	median4	mean8	median8	mean16	median16	mwu4.8	mwu4.16	mwu8.16	occc4.8.16	occc8.16
L1_original_ngtdm_busyness	MRI	T1	px	0.25841676	0.20519126	0.26559426	0.2136883	0.25392165	0.24862322	1	1	1	0.42697335	0.50452419
L1_original_ngtdm_strength	MRI	T1	mm	3.40697708	3.3843236	2.86560946	2.7266897	1.90047211	1.8618846	0.73803286	0.00077941	0.00194852	0.21232262	0.16308186
L1_original_ngtdm_strength	MRI	T1	px	4.36211509	4.1017899	3.55406742	3.9122407	2.89611205	2.98228113	1	0.85884085	0.73803286	0.20564277	0.44620638
L1_original_ngtdm_busyness	MRI	T1	mm	0.21914472	0.19547692	0.25926843	0.2544922	0.37532531	0.37328462	0.45153608	0.0063002	0.00194852	0.1362017	0.04163776
L1_original_ngtdm_complexity	MRI	T1	px	17.7167367	16.093423	34.7470042	32.838011	91.6727739	92.658412	0.01727684	6.4951E-05	6.4951E-05	0.05339334	0.06437824
L1_original_ngtdm_coarseness	MRI	T1	mm	0.11316938	0.11637821	0.03905657	0.0383386	0.00532675	0.0054206	6.4951E-05	6.4951E-05	6.4951E-05	0.00355105	0.00081507
L1_original_ngtdm_complexity	MRI	T1	mm	41.1785564	39.7239854	99.1862193	96.458743	997.559477	1041.08515	6.4951E-05	6.4951E-05	6.4951E-05	0.00215001	0.00361932
L1_original_ngtdm_coarseness	MRI	T1	px	0.34448323	0.35640612	0.1246519	0.1262089	0.04190112	0.04164459	6.4951E-05	6.4951E-05	6.4951E-05	-0.0022803	0.00291086
L1_original_ngtdm_contrast	MRI	T1	px	0.18394761	0.16455249	0.11029052	0.1168989	0.08755535	0.08854945	0.00123406	6.4951E-05	0.03117625	-0.0540874	0.19727827
L1_original_ngtdm_contrast	MRI	T1	mm	0.10977146	0.10816887	0.08636887	0.086566	0.1554614	0.16840815	0.06897746	0.02331724	6.4951E-05	-0.05729	-0.0346628
