# Supplementary material for: Stability of Radiomic Features across Different Region of Interest Sizes—A CT and MR Phantom Study
Source: Tomography. 2021 Jun 8;7(2):238–52. doi: 10.3390/tomography7020022 (PMC8293351; doi:10.3390/tomography7020022)
Supplement: Supplementary file 1 [file tomography-07-00022-s001.zip › table_S2.pdf]

T2w TIRM MR images: first-order features

feature	image_type	mri_sequence	mm_or_px	mean4	median4	mean8	median8	mean16	median16	mwu4.8	mwu4.16	mwu8.16	occc4.8.16	occc8.16
L1_original_firstorder_median	MRI	TIRM	mm	1182.5	1182	1182	1182.5	1183.1	1183.5	1	1	1	0.99812997	0.99803973
L1_original_firstorder_mean	MRI	TIRM	mm	1182.85882	1184.32353	1183.0875	1184.1953	1184.71693	1184.40035	1	1	1	0.99735661	0.99765184
L1_original_firstorder_rootmeansquared	MRI	TIRM	mm	1482.89563	1484.35474	1483.19903	1484.2852	1485.13592	1484.7772	1	1	1	0.99694194	0.99712367
L1_original_firstorder_median	MRI	TIRM	px	1177.9	1179	1182.65	1183.5	1184.4	1186.5	1	1	1	0.98614967	0.99703534
L1_original_firstorder_mean	MRI	TIRM	px	1178.02857	1180.07143	1183.618	1184.6	1185.12436	1185.88173	1	1	1	0.98539353	0.99766221
L1_original_firstorder_rootmeansquared	MRI	TIRM	px	1478.06303	1480.10731	1483.71008	1484.6716	1485.45324	1486.17871	1	1	1	0.98456133	0.9972951
L1_original_firstorder_10percentile	MRI	TIRM	px	1165.8	1167.35	1162.97	1164.4	1144.96	1147.5	1	1	1	0.90045767	0.87579153
L1_original_firstorder_10percentile	MRI	TIRM	mm	1170.22	1172.5	1160.43	1163.8	1139.18	1140.5	1	0.37807703	1	0.8190308	0.83563717
L1_original_firstorder_90percentile	MRI	TIRM	px	1190.19	1192.9	1206.02	1203.1	1226.36	1224	1	0.13942986	1	0.75793938	0.82576107
L1_original_firstorder_90percentile	MRI	TIRM	mm	1195.5	1197.1	1207.63	1206.2	1231.84	1229.9	1	0.11438889	0.63073459	0.74607354	0.77812266
L1_original_firstorder_minimum	MRI	TIRM	px	1161.4	1162	1153.2	1155	1111.6	1110.5	1	0.06082121	0.16983626	0.62118591	0.56676025
L1_original_firstorder_minimum	MRI	TIRM	mm	1164.1	1166	1145.8	1150.5	1104.9	1109.5	1	0.00902812	0.2053569	0.537683	0.55442262
L1_original_firstorder_maximum	MRI	TIRM	px	1195.3	1198.5	1218.4	1215.5	1265.7	1264.5	0.67284242	0.00602134	0.02331724	0.44931282	0.4847089
L1_original_firstorder_maximum	MRI	TIRM	mm	1201.8	1199.5	1222.1	1220.5	1274	1273.5	0.67284242	0.00348036	0.01727684	0.42938347	0.444356
L1_original_firstorder_kurtosis	MRI	TIRM	px	2.18185168	2.0473954	2.30282914	2.2983156	2.50505676	2.52750863	0.25954232	0.01253545	0.21277793	0.21692822	0.13837237
L1_original_firstorder_skewness	MRI	TIRM	mm	0.04546944	-0.0165259	0.0868313	0.0948944	0.19846801	0.20550936	1	0.37807703	1	0.13061682	0.08871807
L1_original_firstorder_uniformity	MRI	TIRM	px	0.17142857	0.16836735	0.10288	0.0988	0.04612651	0.04712909	0.00261167	0.00108381	6.4951E-05	0.04649932	0.02798605
L1_original_firstorder_robustmeanabsolutedeviation	MRI	TIRM	px	5.71173554	5.88082645	10.1744152	10.546104	18.6086865	18.6800398	0.0002598	6.4951E-05	6.4951E-05	0.04080048	0.05987147
L1_original_firstorder_meanabsolutedeviation	MRI	TIRM	px	8.34897959	8.03571429	13.38648	13.7972	25.5065996	25.1230633	0.0002598	6.4951E-05	6.4951E-05	0.03798442	0.05156897
L1_original_firstorder_interquartilerange	MRI	TIRM	px	14.55	14.75	23.75	24.5	44.95	44.75	0.00769226	0.00108381	0.00105967	0.03798029	0.05231635
L1_original_firstorder_interquartilerange	MRI	TIRM	mm	14.5	13.5	26.275	25.625	49.75	50	0.00452725	0.00106566	0.00106566	0.03077251	0.04675791
L1_original_firstorder_variance	MRI	TIRM	px	101.455102	92.377551	272.40516	276.1282	975.958067	934.785229	0.00045465	6.4951E-05	6.4951E-05	0.02970136	0.04940794
L1_original_firstorder_meanabsolutedeviation	MRI	TIRM	mm	8.4283737	8.15224913	14.770459	15.049561	28.7620491	28.3553559	0.0002598	6.4951E-05	6.4951E-05	0.02675416	0.04120963
L1_original_firstorder_uniformity	MRI	TIRM	mm	0.16747405	0.16608997	0.09047852	0.0854492	0.04082441	0.04116159	0.00108991	0.00108991	6.4951E-05	0.02665255	0.02708749
L1_original_firstorder_entropy	MRI	TIRM	px	2.69062019	2.67255358	3.50532347	3.5495625	4.61287538	4.59223249	0.00108381	0.00108381	6.4951E-05	0.02426358	0.03617753
L1_original_firstorder_robustmeanabsolutedeviation	MRI	TIRM	mm	6.23577185	6.01201546	10.7554497	11.114282	20.9207391	20.9028576	0.00045465	6.4951E-05	6.4951E-05	0.02404774	0.03972419
L1_original_firstorder_variance	MRI	TIRM	mm	108.704498	100.605536	330.250342	341.44519	1243.33432	1198.74978	0.0002598	6.4951E-05	6.4951E-05	0.02235478	0.03692661
L1_original_firstorder_range	MRI	TIRM	px	33.9	32.5	65.2	66	154.1	151.5	0.00146883	0.00108991	6.4951E-05	0.02105895	0.0357611
L1_original_firstorder_entropy	MRI	TIRM	mm	2.73206933	2.71601606	3.6787391	3.7053903	4.77961583	4.76723543	6.4951E-05	6.4951E-05	6.4951E-05	0.02076274	0.03353012
L1_original_firstorder_kurtosis	MRI	TIRM	mm	2.33145081	2.36346599	2.44982139	2.4635817	2.48843287	2.51255185	1	1	1	0.02018668	0.1610479
L1_original_firstorder_range	MRI	TIRM	mm	37.7	36.5	76.3	74.5	169.1	166	0.00107774	0.00108381	0.00107774	0.01364726	0.02156894
L1_original_firstorder_totalenergy	MRI	TIRM	px	118562996	118834807	426665563	427026462	3652206879	3654232196	6.4951E-05	6.4951E-05	6.4951E-05	0.0003653	0.00060998
L1_original_firstorder_energy	MRI	TIRM	px	30600767.4	30670941	110121152	110214282	942624065	943146651	6.4951E-05	6.4951E-05	6.4951E-05	0.00036529	0.00060997
L1_original_firstorder_totalenergy	MRI	TIRM	mm	144906095	145127701	545751595	546310608	4847590664	4843197009	6.4951E-05	6.4951E-05	6.4951E-05	0.00034056	0.00057898
L1_original_firstorder_energy	MRI	TIRM	mm	37399845.4	37457035.5	140856913	141001172	1251149173	1250014994	6.4951E-05	6.4951E-05	6.4951E-05	0.00034056	0.00057898
L1_original_firstorder_skewness	MRI	TIRM	px	0.08439154	0.0804244	0.1799005	0.15001	0.13670014	0.15027683	1	1	1	-0.1069832	0.14568385

T2w TIRM MR images: GLCM-features

feature	image_type	mri_sequence	mm_or_px	mean4	median4	mean8	median8	mean16	median16	mwu4.8	mwu4.16	mwu8.16	occc4.8.16	occc8.16
L1_original_glcm_mcc	MRI	TIRM	mm	0.83676518	0.83852692	0.8476234	0.8454718	0.84631669	0.83743937	1	1	1	0.45826204	0.42430243
L1_original_glcm_imc2	MRI	TIRM	px	0.96426029	0.96897526	0.96390899	0.9733484	0.96845787	0.96848283	1	1	1	0.39637048	0.48637137
L1_original_glcm_imc2	MRI	TIRM	mm	0.94873165	0.94987926	0.96924322	0.9745047	0.97377878	0.9735988	0.21277793	0.03117625	1	0.32726666	0.4937678
L1_original_glcm_imc1	MRI	TIRM	mm	-0.4739104	-0.4559883	-0.407854	-0.40953	-0.3296345	-0.3263196	0.21277793	6.4951E-05	0.00123406	0.17494363	0.15099102
L1_original_glcm_mcc	MRI	TIRM	px	0.9106618	0.90449161	0.81809816	0.8336377	0.80990297	0.80749602	0.02331724	0.0063002	1	0.1490859	0.08996001
L1_original_glcm_imc1	MRI	TIRM	px	-0.5573892	-0.559685	-0.4096106	-0.4272796	-0.3230825	-0.3220261	0.00435169	6.4951E-05	0.0063002	0.09530942	0.1610391
L1_original_glcm_maximumprobability	MRI	TIRM	px	0.1328125	0.125	0.06822465	0.0602046	0.01814946	0.01878039	0.01015535	0.00108381	6.4951E-05	0.08987157	0.02096311
L1_original_glcm_maximumprobability	MRI	TIRM	mm	0.12049242	0.11590909	0.05946918	0.0470485	0.01530163	0.0153199	0.01003291	0.00107169	0.00108991	0.06403778	0.01163933
L1_original_glcm_correlation	MRI	TIRM	px	0.30613049	0.35740196	0.71152529	0.7110025	0.74258365	0.73930462	6.4951E-05	6.4951E-05	1	0.04625097	0.27382992
L1_original_glcm_correlation	MRI	TIRM	mm	0.33229831	0.34065468	0.76323312	0.7710999	0.80403951	0.80036432	6.4951E-05	6.4951E-05	1	0.04397543	0.36197272
L1_original_glcm_inversevariance	MRI	TIRM	mm	0.39614498	0.41046843	0.39100558	0.39152	0.25294965	0.25040051	1	6.4951E-05	6.4951E-05	0.03718633	-0.0134655
L1_original_glcm_clustertendency	MRI	TIRM	px	9.862875	9.05367188	33.7305837	34.07852	112.998821	109.213132	0.0002598	6.4951E-05	6.4951E-05	0.03334727	0.06040369
L1_original_glcm_id	MRI	TIRM	px	0.4626369	0.46864583	0.46859003	0.4713885	0.33833774	0.33665903	1	6.4951E-05	6.4951E-05	0.03205087	-0.0121057
L1_original_glcm_sumsquares	MRI	TIRM	px	3.67353125	3.28136719	9.74243843	9.6789105	32.1567813	31.1743947	0.00045465	6.4951E-05	6.4951E-05	0.03155033	0.05285189
L1_original_glcm_clustershade	MRI	TIRM	px	2.92779844	1.9078125	36.6678989	25.770987	140.372637	137.763426	0.04104873	0.17283336	0.45153608	0.03136919	0.0921736
L1_original_glcm_idm	MRI	TIRM	px	0.39320293	0.40408795	0.39651663	0.4035051	0.24171782	0.24002785	1	6.4951E-05	6.4951E-05	0.02677169	-0.0165453
L1_original_glcm_id	MRI	TIRM	mm	0.4713079	0.47959596	0.4672956	0.4701022	0.33971291	0.33729425	1	6.4951E-05	6.4951E-05	0.02568027	-0.0127348
L1_original_glcm_clustertendency	MRI	TIRM	mm	10.765247	9.25457214	42.6752523	43.657555	152.529196	147.346571	0.0001299	6.4951E-05	6.4951E-05	0.02469313	0.04431114
L1_original_glcm_sumsquares	MRI	TIRM	mm	3.91549736	3.39332853	12.0188117	12.506289	42.0405661	40.6757543	0.0001299	6.4951E-05	6.4951E-05	0.02300358	0.03933943
L1_original_glcm_differencevariance	MRI	TIRM	mm	1.57748875	1.53173037	1.93331783	1.932216	3.51249048	3.55049883	0.73803286	6.4951E-05	6.4951E-05	0.02272112	0.01893012
L1_original_glcm_idm	MRI	TIRM	mm	0.39967306	0.4127613	0.3953071	0.4000093	0.24356858	0.24089754	1	6.4951E-05	6.4951E-05	0.02191837	-0.0161945
L1_original_glcm_clusterprominence	MRI	TIRM	px	237.111243	179.731208	2947.02079	2674.5674	32541.6225	29433.5939	0.0001299	6.4951E-05	6.4951E-05	0.01998672	0.03889151
L1_original_glcm_idmn	MRI	TIRM	px	0.93267049	0.93420944	0.97500559	0.9776134	0.98467893	0.98425282	6.4951E-05	6.4951E-05	0.00123406	0.01984452	0.13856794
L1_original_glcm_differencevariance	MRI	TIRM	px	1.4983125	1.435	1.829377	1.6771679	3.49085346	3.55843865	0.63073459	6.4951E-05	6.4951E-05	0.01758393	0.02067469
L1_original_glcm_jointenergy	MRI	TIRM	px	0.08452344	0.08386719	0.02802787	0.0271747	0.00674692	0.00680684	6.4951E-05	6.4951E-05	6.4951E-05	0.01475913	0.00948374
L1_original_glcm_clusterprominence	MRI	TIRM	mm	311.80851	190.584897	4536.06809	4253.5498	59236.1495	54527.3516	6.4951E-05	6.4951E-05	6.4951E-05	0.01460288	0.02785438
L1_original_glcm_clustershade	MRI	TIRM	mm	-3.3627703	-1.3511201	49.577088	41.458017	394.60785	359.638219	0.13938384	0.00194852	0.01253545	0.01306975	0.03920302
L1_original_glcm_differenceaverage	MRI	TIRM	mm	1.76787879	1.75473485	1.83065697	1.8045532	3.23182083	3.25606903	1	6.4951E-05	6.4951E-05	0.01030806	-0.0044618
L1_original_glcm_jointenergy	MRI	TIRM	mm	0.07047079	0.07173295	0.0230996	0.0215081	0.00554378	0.00554442	6.4951E-05	6.4951E-05	6.4951E-05	0.01019406	0.00959066
L1_original_glcm_differenceaverage	MRI	TIRM	px	1.76625	1.665625	1.81418932	1.797619	3.23811813	3.24499203	1	6.4951E-05	6.4951E-05	0.00820778	-0.0016437
L1_original_glcm_autocorrelation	MRI	TIRM	px	20.92625	22.478125	58.1996698	59.262158	270.959433	268.343672	6.4951E-05	6.4951E-05	6.4951E-05	0.008029	0.01755834
L1_original_glcm_sumentropy	MRI	TIRM	mm	2.78229735	2.78044135	4.27208277	4.3282608	5.49854666	5.48829128	6.4951E-05	6.4951E-05	6.4951E-05	0.00793505	0.02007154
L1_original_glcm_sumentropy	MRI	TIRM	px	2.57593396	2.60211065	4.03183516	4.1034502	5.26452125	5.2560758	6.4951E-05	6.4951E-05	6.4951E-05	0.00789389	0.02017793
L1_original_glcm_contrast	MRI	TIRM	mm	4.89674242	4.80776515	5.39999469	5.4228584	15.6330681	15.7245561	1	6.4951E-05	6.4951E-05	0.00750304	-0.001528
L1_original_glcm_contrast	MRI	TIRM	px	4.83125	4.403125	5.23917004	5.1338563	15.6283041	15.6001542	1	6.4951E-05	6.4951E-05	0.00720467	0.0027647
L1_original_glcm_jointaverage	MRI	TIRM	px	4.396875	4.5609375	7.10037835	7.2258471	15.6691313	15.7229637	6.4951E-05	6.4951E-05	6.4951E-05	0.00686414	0.01650454
L1_original_glcm_sumaverage	MRI	TIRM	px	8.79375	9.121875	14.2007567	14.451694	31.3382626	31.4459274	6.4951E-05	6.4951E-05	6.4951E-05	0.00686414	0.01650454
L1_original_glcm_idn	MRI	TIRM	px	0.82915803	0.82844855	0.89233585	0.8948831	0.91039206	0.90850968	6.4951E-05	6.4951E-05	0.00435169	0.00682206	0.15763749
L1_original_glcm_jointentropy	MRI	TIRM	px	3.70307213	3.69215295	5.44463925	5.4381377	7.49465667	7.48928957	6.4951E-05	6.4951E-05	6.4951E-05	0.00374756	0.00774684
L1_original_glcm_jointentropy	MRI	TIRM	mm	3.97934772	3.94457669	5.74745949	5.7825107	7.79000051	7.79032631	6.4951E-05	6.4951E-05	6.4951E-05	0.00370409	0.00853701
L1_original_glcm_jointaverage	MRI	TIRM	mm	4.71121212	4.43522727	8.62002254	8.4866897	16.84756	17.0116227	0.00108991	0.00108991	6.4951E-05	0.00322948	6.7998E-05
L1_original_glcm_sumaverage	MRI	TIRM	mm	9.42242424	8.87045455	17.2400451	16.973379	33.69512	34.0232455	0.00108991	0.00108991	6.4951E-05	0.00322948	6.7998E-05
L1_original_glcm_autocorrelation	MRI	TIRM	mm	24.1995833	21.7869318	84.6879629	80.607854	319.176087	319.433122	6.4951E-05	6.4951E-05	6.4951E-05	0.00300633	0.00220204
L1_original_glcm_differenceentropy	MRI	TIRM	mm	2.01657018	1.97893172	2.30812035	2.3107743	2.81045825	2.82034972	0.00194852	6.4951E-05	6.4951E-05	0.00108742	0.01570805
L1_original_glcm_idmn	MRI	TIRM	mm	0.93885344	0.94351054	0.98091788	0.9830486	0.98724356	0.98669342	6.4951E-05	6.4951E-05	0.00123406	0.00065603	0.18929686
L1_original_glcm_inversevariance	MRI	TIRM	px	0.43329635	0.45323003	0.39008714	0.3926439	0.25124899	0.25189318	0.73803286	6.4951E-05	6.4951E-05	-0.0013439	-0.0127942
L1_original_glcm_idn	MRI	TIRM	mm	0.83778703	0.84235121	0.90514914	0.9067421	0.91762215	0.91471641	6.4951E-05	6.4951E-05	0.03117625	-0.0029128	0.22967344
L1_original_glcm_differenceentropy	MRI	TIRM	px	1.82875052	1.87809917	2.26004017	2.2352952	2.8015281	2.81606476	6.4951E-05	6.4951E-05	6.4951E-05	-0.0091724	0.01328262

T2w TIRM MR images: GLDM-features

feature	image_type	mri_sequence	mm_or_px	mean4	median4	mean8	median8	mean16	median16	mwu4.8	mwu4.16	mwu8.16	occc4.8.16	occc8.16
L1_original_gldm_dependencevariance	MRI	TIRM	px	0.64693878	0.48979592	1.11568	0.9864	1.8217113	1.85383016	0.18535318	0.01003291	0.01727684	0.32099315	0.21893694
L1_original_gldm_dependencevariance	MRI	TIRM	mm	0.81937716	0.74740484	1.13974609	1.003418	1.82611971	1.9300256	1	0.01019637	0.03117625	0.26686815	0.15833155
L1_original_gldm_largedependenceemphasis	MRI	TIRM	px	3.91428571	3.42857143	5.708	5.72	9.08149883	9.16159251	0.15248931	0.00257278	0.01019637	0.15050108	0.08949828
L1_original_gldm_largedependenceemphasis	MRI	TIRM	mm	4.57647059	4.70588235	5.84375	5.578125	9.43068783	9.41798942	0.3228742	6.4951E-05	0.00785654	0.09868635	0.03525528
L1_original_gldm_dependencenonuniformitynormalized	MRI	TIRM	mm	0.3467128	0.32525952	0.30913086	0.2893066	0.22130897	0.21595451	0.3223332	0.00108991	0.00785654	0.07916772	-0.0361115
L1_original_gldm_dependencenonuniformitynormalized	MRI	TIRM	px	0.39693878	0.3877551	0.31784	0.2904	0.22531029	0.21725562	0.53023469	0.00106566	0.00077941	0.07766097	-0.0461362
L1_original_gldm_smalldependenceemphasis	MRI	TIRM	mm	0.50014379	0.50816993	0.43430714	0.4364138	0.29836158	0.28990909	0.17283336	6.4951E-05	6.4951E-05	0.05790636	0.04668173
L1_original_gldm_smalldependencehighgraylevelemphasis	MRI	TIRM	px	14.1877817	11.5634921	30.231201	27.545144	90.2708906	87.2906391	0.00194852	6.4951E-05	6.4951E-05	0.03676785	0.07353806
L1_original_gldm_graylevelvariance	MRI	TIRM	px	4.0877551	3.72959184	11.13384	11.3122	39.2016728	37.740173	0.0002598	6.4951E-05	6.4951E-05	0.02652383	0.04717256
L1_original_gldm_graylevelvariance	MRI	TIRM	mm	4.34463668	3.91695502	13.4860107	13.842773	49.7898802	48.0433607	0.00146883	0.00108991	6.4951E-05	0.02141597	0.03665277
L1_original_gldm_smalldependenceemphasis	MRI	TIRM	px	0.55027381	0.5515873	0.4422951	0.4166167	0.31650448	0.30839083	0.05387113	0.00106566	0.0001299	0.02038471	0.04267348
L1_original_gldm_smalldependencehighgraylevelemphasis	MRI	TIRM	mm	13.4787859	12.8635621	39.637764	37.624872	98.1261457	96.2007771	0.0002598	6.4951E-05	6.4951E-05	0.01791607	0.00979649
L1_original_gldm_smalldependencelowgraylevelemphasis	MRI	TIRM	px	0.10241849	0.10451581	0.03655308	0.0371384	0.00674976	0.0066936	0.0001299	6.4951E-05	6.4951E-05	0.0144908	0.01124184
L1_original_gldm_highgraylevelemphasis	MRI	TIRM	px	23.8	24	62.576	64.13	284.33911	281.197892	6.4951E-05	6.4951E-05	6.4951E-05	0.00869441	0.01828524
L1_original_gldm_graylevelnonuniformity	MRI	TIRM	px	2.4	2.35714286	5.144	4.94	19.6960187	20.1241218	0.00106566	0.00106566	6.4951E-05	0.0061775	0.01004364
L1_original_gldm_graylevelnonuniformity	MRI	TIRM	mm	2.84705882	2.82352941	5.790625	5.46875	23.1474427	23.3386243	0.00108991	0.00108991	6.4951E-05	0.0055966	0.00885556
L1_original_gldm_highgraylevelemphasis	MRI	TIRM	mm	26.7647059	23.7352941	88.7640625	84.132813	334.593827	334.200176	0.00108991	0.00108991	6.4951E-05	0.00331075	0.00263091
L1_original_gldm_smalldependencelowgraylevelemphasis	MRI	TIRM	mm	0.09253253	0.08200169	0.02798585	0.0275135	0.00583622	0.00558863	6.4951E-05	6.4951E-05	6.4951E-05	0.00304113	-0.012865
L1_original_gldm_largedependencehighgraylevelemphasis	MRI	TIRM	px	82.7	79.25	319.176	297.5	2607.58501	2716.04684	6.4951E-05	6.4951E-05	6.4951E-05	0.00253859	0.00548044
L1_original_gldm_dependenceentropy	MRI	TIRM	mm	3.3358485	3.38158049	4.7493367	4.7489143	6.76257909	6.75781768	0.00107169	0.00107169	6.4951E-05	0.00214461	0.00200999
L1_original_gldm_dependenceentropy	MRI	TIRM	px	3.09117876	3.09306921	4.47911739	4.4663701	6.52724275	6.53283525	0.00104775	0.00104775	6.4951E-05	0.00094391	-0.0006754
L1_original_gldm_largedependencehighgraylevelemphasis	MRI	TIRM	mm	121.223529	142.088235	486.673438	479.99219	3161.47637	3166.80423	6.4951E-05	6.4951E-05	6.4951E-05	-0.0001383	-0.0007817
L1_original_gldm_dependencenonuniformity	MRI	TIRM	mm	5.89411765	5.52941176	19.784375	18.515625	125.482187	122.446208	0.00108381	0.00108991	0.00108991	-0.0005054	-0.0014761
L1_original_gldm_dependencenonuniformity	MRI	TIRM	px	5.55714286	5.42857143	15.892	14.52	96.2074941	92.7681499	0.00106566	0.00106566	6.4951E-05	-0.0005406	-0.0020584
L1_original_gldm_lowgraylevelemphasis	MRI	TIRM	mm	0.15138948	0.14589429	0.05144514	0.0514321	0.01205751	0.01121489	6.4951E-05	6.4951E-05	6.4951E-05	-0.0025854	0.00247609
L1_original_gldm_lowgraylevelemphasis	MRI	TIRM	px	0.16654947	0.1662869	0.0736866	0.0618078	0.0133397	0.01187392	0.0001299	6.4951E-05	6.4951E-05	-0.0136271	-0.0016047
L1_original_gldm_largedependencelowgraylevelemphasis	MRI	TIRM	mm	0.48643282	0.49972247	0.247055	0.1661394	0.06967589	0.06806273	0.11126026	6.4951E-05	0.00902812	-0.0283419	-0.0089241
L1_original_gldm_largedependencelowgraylevelemphasis	MRI	TIRM	px	0.51946855	0.27935962	0.36862101	0.2858365	0.07589779	0.06673937	1	6.4951E-05	0.0002598	-0.0534446	-0.0274185

T2w TIRM MR images: GLRLM-features

feature	image_type	mri_sequence	mm_or_px	mean4	median4	mean8	median8	mean16	median16	mwu4.8	mwu4.16	mwu8.16	occc4.8.16	occc8.16
L1_original_glrlm_runvariance	MRI	TIRM	px	0.1106389	0.08797666	0.18185861	0.1843185	0.08524423	0.08409735	0.22538083	0.45330951	0.00123406	0.20532154	0.0538202
L1_original_glrlm_runvariance	MRI	TIRM	mm	0.13693787	0.14218537	0.195021	0.1755005	0.08762889	0.08649773	0.73803286	0.04104873	0.0001299	0.19780176	0.03440354
L1_original_glrlm_longrunemphasis	MRI	TIRM	px	1.37974553	1.31381119	1.55313358	1.571163	1.24481827	1.24499386	0.27044272	0.10329322	0.0001299	0.17781349	0.03054569
L1_original_glrlm_longrunemphasis	MRI	TIRM	mm	1.439375	1.43236607	1.5794761	1.5408373	1.25386478	1.25240231	0.85884085	0.00292277	6.4951E-05	0.15782414	0.0195656
L1_original_glrlm_runpercentage	MRI	TIRM	px	0.90178571	0.91071429	0.859	0.855	0.9350027	0.93388579	0.10054336	0.15083022	0.00108991	0.12658058	0.00989554
L1_original_glrlm_runpercentage	MRI	TIRM	mm	0.88382353	0.88235294	0.85585938	0.8574219	0.93250577	0.9329806	0.22408897	0.00194845	0.00108991	0.10793589	0.00245296
L1_original_glrlm_shortrunemphasis	MRI	TIRM	px	0.91576788	0.9215472	0.88654091	0.8905882	0.95001445	0.94876631	0.27044272	0.08389955	6.4951E-05	0.09423363	0.00914707
L1_original_glrlm_shortrunemphasis	MRI	TIRM	mm	0.90435039	0.90345601	0.88493013	0.8854909	0.947758	0.94770587	0.73803286	6.4951E-05	6.4951E-05	0.08995386	0.00251269
L1_original_glrlm_runlengthnonuniformitynormalized	MRI	TIRM	px	0.82109681	0.82482742	0.7499866	0.7542085	0.88317966	0.87918824	0.10329322	0.10329322	6.4951E-05	0.07528338	0.00236766
L1_original_glrlm_runlengthnonuniformitynormalized	MRI	TIRM	mm	0.7897941	0.78860961	0.74705605	0.7445345	0.87817312	0.87781187	0.31455541	6.4951E-05	6.4951E-05	0.07157141	-0.0048051
L1_original_glrlm_graylevelvariance	MRI	TIRM	px	4.21089684	3.83456462	11.4595278	11.63767	39.797571	38.4473931	0.0001299	6.4951E-05	6.4951E-05	0.02517203	0.04651084
L1_original_glrlm_graylevelnonuniformitynormalized	MRI	TIRM	px	0.16576353	0.16431653	0.09683371	0.0934801	0.04565407	0.04644793	0.0001299	6.4951E-05	6.4951E-05	0.02312043	0.03380342
L1_original_glrlm_graylevelvariance	MRI	TIRM	mm	4.48585724	4.23069755	13.8546915	14.070181	50.3730518	48.8194811	0.0001299	6.4951E-05	6.4951E-05	0.02134767	0.03780837
L1_original_glrlm_graylevelnonuniformitynormalized	MRI	TIRM	mm	0.16140394	0.16298329	0.08587387	0.0824909	0.04048143	0.04069386	6.4951E-05	6.4951E-05	6.4951E-05	0.0194971	0.03304143
L1_original_glrlm_runentropy	MRI	TIRM	mm	2.97368469	2.93577015	4.13626567	4.1744681	5.08331116	5.09165444	6.4951E-05	6.4951E-05	6.4951E-05	0.01028066	0.02770255
L1_original_glrlm_runentropy	MRI	TIRM	px	2.87968144	2.87925757	3.94041131	3.980582	4.90071694	4.91247835	6.4951E-05	6.4951E-05	6.4951E-05	0.01024174	0.02893812
L1_original_glrlm_shortrunhighgraylevelemphasis	MRI	TIRM	px	22.2088479	21.2944712	56.7750055	58.271227	269.871186	266.007885	6.4951E-05	6.4951E-05	6.4951E-05	0.00956161	0.01955486
L1_original_glrlm_highgraylevelrunemphasis	MRI	TIRM	px	23.974577	23.3679654	63.2298496	64.719309	284.25526	280.750816	6.4951E-05	6.4951E-05	6.4951E-05	0.00913507	0.01929797
L1_original_glrlm_longrunhighgraylevelemphasis	MRI	TIRM	px	31.4321061	32.6684461	93.8991636	93.972362	354.01968	351.383297	6.4951E-05	6.4951E-05	6.4951E-05	0.0074542	0.01844849
L1_original_glrlm_shortrunhighgraylevelemphasis	MRI	TIRM	mm	24.1548689	21.5352936	79.1697951	74.156208	316.746455	317.152233	6.4951E-05	6.4951E-05	6.4951E-05	0.00407488	0.00334403
L1_original_glrlm_graylevelnonuniformity	MRI	TIRM	mm	2.41929864	2.45665266	4.68578739	4.5158227	21.4116405	21.3582582	6.4951E-05	6.4951E-05	6.4951E-05	0.00382426	0.00654009
L1_original_glrlm_graylevelnonuniformity	MRI	TIRM	px	2.08075036	2.10029554	4.1462232	4.0165349	18.2354481	18.3926659	6.4951E-05	6.4951E-05	6.4951E-05	0.00353152	0.00701935
L1_original_glrlm_highgraylevelrunemphasis	MRI	TIRM	mm	26.715122	23.5668727	89.0883535	84.138468	334.306369	333.931683	6.4951E-05	6.4951E-05	6.4951E-05	0.00345246	0.00269468
L1_original_glrlm_longrunhighgraylevelemphasis	MRI	TIRM	mm	38.3126667	38.8148416	136.926752	126.78766	420.174898	419.398616	6.4951E-05	6.4951E-05	6.4951E-05	0.00045465	-0.0018993
L1_original_glrlm_runlengthnonuniformity	MRI	TIRM	px	10.5380206	10.5769231	32.4260903	32.356098	355.171323	353.110959	0.00108991	0.00108991	6.4951E-05	3.474E-05	-2.968E-06
L1_original_glrlm_runlengthnonuniformity	MRI	TIRM	mm	11.9886493	11.8753892	41.2251694	40.773434	467.821115	467.76505	6.4951E-05	6.4951E-05	6.4951E-05	-7.631E-06	-5.567E-05
L1_original_glrlm_shortrunlowgraylevelemphasis	MRI	TIRM	mm	0.14806459	0.13758162	0.04861709	0.0495016	0.01200763	0.01117954	6.4951E-05	6.4951E-05	6.4951E-05	-0.0006134	-0.0061
L1_original_glrlm_lowgraylevelrunemphasis	MRI	TIRM	mm	0.15767197	0.14715152	0.05307227	0.0538565	0.01241104	0.01157333	6.4951E-05	6.4951E-05	6.4951E-05	-0.0018026	-0.0021614
L1_original_glrlm_longrunlowgraylevelemphasis	MRI	TIRM	mm	0.20211856	0.20649098	0.07781021	0.068908	0.01431721	0.01336102	6.4951E-05	6.4951E-05	6.4951E-05	-0.0077251	0.00206612
L1_original_glrlm_shortrunlowgraylevelemphasis	MRI	TIRM	px	0.15935597	0.15819426	0.06788363	0.063014	0.01324299	0.0119976	6.4951E-05	6.4951E-05	6.4951E-05	-0.0101338	0.00770983
L1_original_glrlm_lowgraylevelrunemphasis	MRI	TIRM	px	0.17091632	0.171348	0.07530319	0.0672499	0.01368215	0.01232558	6.4951E-05	6.4951E-05	6.4951E-05	-0.0135526	0.00322263
L1_original_glrlm_longrunlowgraylevelemphasis	MRI	TIRM	px	0.22300825	0.19911322	0.11104438	0.0893123	0.01573206	0.01396837	0.00194852	6.4951E-05	6.4951E-05	-0.0248553	-0.0060358

T2w TIRM MR images: GLSZM-features

feature	image_type	mri_sequence	mm_or_px	mean4	median4	mean8	median8	mean16	median16	mwu4.8	mwu4.16	mwu8.16	occc4.8.16	occc8.16
L1_original_glszm_smallareaemphasis	MRI	TIRM	mm	0.68560813	0.68469066	0.64153369	0.6459771	0.55378906	0.56152227	0.84099943	0.00261167	0.00194852	0.13516056	0.20705672
L1_original_glszm_smallareaemphasis	MRI	TIRM	px	0.71656622	0.7191358	0.64438846	0.6311135	0.57531573	0.57882312	0.45067023	0.00591604	0.13938384	0.07665907	0.19706654
L1_original_glszm_sizezonenonuniformitynormalized	MRI	TIRM	mm	0.45844872	0.45454545	0.40326533	0.4233129	0.29523716	0.29868239	0.84099943	0.00108381	0.00123406	0.07592168	0.10717717
L1_original_glszm_sizezonenonuniformitynormalized	MRI	TIRM	px	0.51236235	0.50617284	0.41704879	0.3895755	0.31674141	0.31484823	0.22365878	0.00192825	0.00292277	0.05329566	0.09836754
L1_original_glszm_zonepercentage	MRI	TIRM	mm	0.61176471	0.64705882	0.54375	0.5390625	0.37195767	0.36155203	0.31100711	0.00096706	0.00107774	0.05017427	0.02518116
L1_original_glszm_smallarealowgraylevelemphasis	MRI	TIRM	px	0.13907718	0.14436845	0.05688873	0.0616631	0.01505722	0.01482035	0.05358419	6.4951E-05	6.4951E-05	0.04984395	0.0158575
L1_original_glszm_smallareahighgraylevelemphasis	MRI	TIRM	px	18.7133671	16.4409028	44.7528176	43.197104	164.971796	162.2606	0.00045465	6.4951E-05	6.4951E-05	0.02298449	0.04745993
L1_original_glszm_graylevelnonuniformitynormalized	MRI	TIRM	mm	0.15129789	0.15702479	0.08142285	0.0796028	0.03787066	0.03806551	0.00107169	0.00107169	6.4951E-05	0.0191757	0.02904108
L1_original_glszm_zonepercentage	MRI	TIRM	px	0.65714286	0.64285714	0.556	0.54	0.3911007	0.38407494	0.03281645	0.00097829	0.00104775	0.01808294	0.00512096
L1_original_glszm_graylevelvariance	MRI	TIRM	px	4.6070869	4.28395062	12.5163928	12.565007	49.6197887	49.2541887	6.4951E-05	6.4951E-05	6.4951E-05	0.01792846	0.03331751
L1_original_glszm_graylevelvariance	MRI	TIRM	mm	4.99158951	4.64253086	15.1147192	14.928524	59.1753601	56.7540141	0.00146883	0.00108991	6.4951E-05	0.01790806	0.03334155
L1_original_glszm_zonevariance	MRI	TIRM	px	0.68742223	0.6875	1.60101059	1.4809242	7.70858615	7.47691739	0.03422267	0.00106566	6.4951E-05	0.01776731	0.01353279
L1_original_glszm_largeareaemphasis	MRI	TIRM	mm	3.75285354	3.54545455	5.39959556	4.9444444	15.8732455	15.4881446	0.18649301	0.00108381	6.4951E-05	0.01490695	0.01637914
L1_original_glszm_graylevelnonuniformitynormalized	MRI	TIRM	px	0.15924092	0.16049383	0.08904106	0.0886429	0.0418144	0.0417411	0.0010068	0.0010068	6.4951E-05	0.01269789	0.0274814
L1_original_glszm_largeareaemphasis	MRI	TIRM	px	3.0940404	3.11111111	4.9461638	5.0612536	14.3646063	14.1646341	0.03422267	0.00106566	6.4951E-05	0.0126568	0.01319979
L1_original_glszm_zonevariance	MRI	TIRM	mm	0.97159159	0.99260331	1.87286886	1.4835731	8.55367204	7.9173925	0.3223332	0.00108381	6.4951E-05	0.0122447	0.01418654
L1_original_glszm_highgraylevelzoneemphasis	MRI	TIRM	px	24.6975758	24.25	65.7532469	65.634793	284.35252	278.222561	6.4951E-05	6.4951E-05	6.4951E-05	0.01176906	0.02373496
L1_original_glszm_smallareahighgraylevelemphasis	MRI	TIRM	mm	18.258427	17.9253788	58.3886632	55.601833	180.269925	174.729331	6.4951E-05	6.4951E-05	6.4951E-05	0.00917994	0.00403546
L1_original_glszm_lowgraylevelzoneemphasis	MRI	TIRM	px	0.18449481	0.1711704	0.08116666	0.0761135	0.02028173	0.01950192	6.4951E-05	6.4951E-05	6.4951E-05	0.00690098	0.02220279
L1_original_glszm_smallarealowgraylevelemphasis	MRI	TIRM	mm	0.13751737	0.13573307	0.04486287	0.0469017	0.01327219	0.01168252	0.00077941	6.4951E-05	0.00077941	0.00598042	-0.0184045
L1_original_glszm_sizezonenonuniformity	MRI	TIRM	mm	4.83065657	5	14.1650764	13.996622	62.5233017	59.5891206	0.00108381	0.00108381	6.4951E-05	0.00522181	0.01189917
L1_original_glszm_highgraylevelzoneemphasis	MRI	TIRM	mm	26.6077273	23.9545455	89.7940122	84.788462	329.750639	326.32439	0.00108991	0.00108991	6.4951E-05	0.0045661	0.00424703
L1_original_glszm_zoneentropy	MRI	TIRM	mm	3.05843464	3.09579526	4.45550256	4.4689479	6.46595015	6.45059503	0.00107169	0.00107169	6.4951E-05	0.00449006	0.00389486
L1_original_glszm_sizezonenonuniformity	MRI	TIRM	px	4.78873737	4.55555556	11.7481077	10.305714	53.2512484	50.9448864	0.00106566	0.00106566	6.4951E-05	0.00259062	0.00627918
L1_original_glszm_largeareahighgraylevelemphasis	MRI	TIRM	mm	101.402096	107.177273	450.966278	457.39145	5236.38199	5161.65945	6.4951E-05	6.4951E-05	6.4951E-05	0.0021888	0.00460871
L1_original_glszm_lowgraylevelzoneemphasis	MRI	TIRM	mm	0.17770781	0.16204096	0.05983624	0.0583604	0.01852416	0.01808169	0.00108991	0.00108991	6.4951E-05	0.00208921	-0.0251262
L1_original_glszm_graylevelnonuniformity	MRI	TIRM	mm	1.56641414	1.57272727	2.79673685	2.8138528	7.98018786	8.12070107	0.00107169	0.00107169	6.4951E-05	0.00140753	0.00418384
L1_original_glszm_largeareahighgraylevelemphasis	MRI	TIRM	px	66.0538131	72.1666667	277.641557	251.03226	4031.86407	4143.51799	6.4951E-05	6.4951E-05	6.4951E-05	0.00068531	0.00176087
L1_original_glszm_zoneentropy	MRI	TIRM	px	2.89321075	2.93481544	4.24243805	4.2491723	6.19950162	6.17732086	0.00108381	0.00108381	6.4951E-05	0.00059125	0.00078163
L1_original_glszm_graylevelnonuniformity	MRI	TIRM	px	1.44050505	1.44444444	2.45379475	2.4919355	6.98083901	6.98353659	0.0010068	0.0010068	6.4951E-05	-0.0009843	-0.0037221
L1_original_glszm_largearealowgraylevelemphasis	MRI	TIRM	mm	0.42721612	0.43215333	0.23031906	0.177979	0.11556251	0.10165011	0.02331724	6.4951E-05	0.25954232	-0.0138372	-0.0609861
L1_original_glszm_largearealowgraylevelemphasis	MRI	TIRM	px	0.45380649	0.32896802	0.32819915	0.2774702	0.11294018	0.1057107	1	6.4951E-05	0.00123406	-0.1002988	-0.0207205

T2w TIRM MR images: NGTDM-features

feature	image_type	mri_sequence	mm_or_px	mean4	median4	mean8	median8	mean16	median16	mwu4.8	mwu4.16	mwu8.16	occc4.8.16	occc8.16
L1_original_ngtdm_strength	MRI	TIRM	px	8.55317522	7.81918493	13.7379113	14.336072	10.3216114	9.89564017	0.21277793	1	0.85884085	0.48404565	0.54918855
L1_original_ngtdm_strength	MRI	TIRM	mm	7.54219224	7.00920209	15.2083079	16.079883	10.4783946	9.97520512	0.01727684	0.03117625	0.13938384	0.35882963	0.43808838
L1_original_ngtdm_busyness	MRI	TIRM	px	0.13790401	0.09830244	0.07824627	0.068796	0.08539226	0.08320258	0.25954232	0.99296369	1	0.16054924	0.71671452
L1_original_ngtdm_busyness	MRI	TIRM	mm	0.14000744	0.13200771	0.06477454	0.0638076	0.0893752	0.08881455	0.01727684	0.13938384	0.08813787	0.15225807	0.28081047
L1_original_ngtdm_contrast	MRI	TIRM	mm	0.21411682	0.2211828	0.13938543	0.1332484	0.15372228	0.15338809	0.00077941	0.00902812	0.73803286	0.08387081	-0.1391634
L1_original_ngtdm_contrast	MRI	TIRM	px	0.21352504	0.20407301	0.14329828	0.1406689	0.14777267	0.1469515	0.00045465	0.0001299	1	0.06915636	-0.133636
L1_original_ngtdm_coarseness	MRI	TIRM	mm	0.33247154	0.33955082	0.16707554	0.1754874	0.02597123	0.02547657	6.4951E-05	6.4951E-05	6.4951E-05	0.02068375	0.00401358
L1_original_ngtdm_complexity	MRI	TIRM	px	24.5759874	20.740971	82.725671	83.514521	645.076767	609.075086	6.4951E-05	6.4951E-05	6.4951E-05	0.01131812	0.02141473
L1_original_ngtdm_complexity	MRI	TIRM	mm	27.8037554	25.4460509	106.903487	99.341471	774.587327	753.431638	6.4951E-05	6.4951E-05	6.4951E-05	0.00831445	0.01451444
L1_original_ngtdm_coarseness	MRI	TIRM	px	0.41166216	0.42728853	0.18988882	0.1848361	0.0310852	0.0303921	0.0001299	6.4951E-05	6.4951E-05	0.00796277	0.00234416
