# Supplementary material for: Stability of Radiomic Features across Different Region of Interest Sizes—A CT and MR Phantom Study
Source: Tomography. 2021 Jun 8;7(2):238–52. doi: 10.3390/tomography7020022 (PMC8293351; doi:10.3390/tomography7020022)
Supplement: Supplementary file 1 [file tomography-07-00022-s001.zip › table_S3.pdf]

CT images: first-order features

feature	image_type	mm_or_px	mean4	median4	mean8	median8	mean16	median16	mwu4.8	mwu4.16	mwu8.16	occc4.8.16	occc8.16
L1_original_firstorder_entropy	CT	mm	0.95736376	0.96254439	0.95341801	0.9588809	0.94865933	0.9514916	1	1	1	0.69507366	0.6596546
L1_original_firstorder_uniformity	CT	mm	0.53196581	0.52573661	0.54260913	0.5376857	0.54429061	0.54211707	0.99296369	0.63073459	1	0.58690926	0.69394105
L1_original_firstorder_mean	CT	px	22.0393732	21.6010753	21.9188486	21.44055	21.4172643	21.4365405	1	1	1	0.42813934	0.38962574
L1_original_firstorder_rootmeansquared	CT	px	1022.0693	1021.63681	1021.94936	1021.4751	1021.44729	1021.46617	1	1	1	0.42723608	0.38871834
L1_original_firstorder_median	CT	mm	21.8	21.5	21.6	22	21.5	21.5	1	1	1	0.40978189	0.80160321
L1_original_firstorder_10percentile	CT	px	12.21	11.85	11.91	11.4	11.4	11	1	1	1	0.39607278	0.44611937
L1_original_firstorder_rootmeansquared	CT	mm	1021.88953	1021.77413	1021.47794	1021.5852	1021.53508	1021.5702	1	1	1	0.36010789	0.59248886
L1_original_firstorder_mean	CT	mm	21.8569642	21.7380282	21.4477	21.555066	21.506601	21.5409275	1	1	1	0.35995459	0.58924738
L1_original_firstorder_entropy	CT	px	0.90267162	0.95015711	0.94768471	0.9651743	0.94821555	0.95253669	1	1	1	0.35292465	0.62868044
L1_original_firstorder_uniformity	CT	px	0.56417094	0.53407848	0.5408007	0.5336825	0.54596913	0.54267995	1	1	1	0.34679477	0.58836537
L1_original_firstorder_median	CT	px	22.2	21.5	21.8	21	21.2	21	1	1	1	0.32543902	0.1464255
L1_original_firstorder_skewness	CT	px	-0.1593989	-0.1219349	0.08328239	0.0880568	0.03570548	0.02714924	1	1	1	0.30831487	0.31199062
L1_original_firstorder_90percentile	CT	px	31.44	31.45	32.1	31	31.6	31.5	1	1	1	0.28667381	0.34087883
L1_original_firstorder_90percentile	CT	mm	32.68	32	31.6	32	31.6	32	0.62933977	0.46639309	1	0.25029506	0.70588235
L1_original_firstorder_variance	CT	px	61.1922949	54.8858047	62.3539051	64.538657	61.3293807	60.6709376	1	1	1	0.22440454	0.31512958
L1_original_firstorder_meanabsolutedeviation	CT	mm	6.58665284	6.51335521	6.30906604	6.2799573	6.11982453	6.16024463	0.99296369	0.11126026	0.02331724	0.18403953	0.19630216
L1_original_firstorder_variance	CT	mm	66.5607747	64.4130137	61.7830246	60.581203	58.1835917	58.936645	1	0.11126026	0.03117625	0.16637636	0.18360403
L1_original_firstorder_robustmeanabsolutedeviation	CT	mm	4.85450246	4.77112124	4.61455927	4.6560866	4.46163506	4.50755306	1	0.45153608	0.25954232	0.15975635	0.26461382
L1_original_firstorder_meanabsolutedeviation	CT	px	6.26149631	6.16496763	6.34623604	6.3407053	6.28769942	6.2454613	1	1	1	0.15595152	0.30149004
L1_original_firstorder_10percentile	CT	mm	11.42	11.1	11.3	11	12	12	1	0.176093	0.00970064	0.15088438	0
L1_original_firstorder_robustmeanabsolutedeviation	CT	px	4.67265917	4.71579835	4.66229133	4.626495	4.60065944	4.60918952	1	1	1	0.15013462	0.35464458
L1_original_firstorder_interquartilerange	CT	px	10.525	10.625	10.575	10.75	10.8	11	1	1	1	0.14939863	0.44420522
L1_original_firstorder_kurtosis	CT	px	2.73081997	2.38842061	2.86510499	2.7961233	2.91827579	2.87101995	1	0.73803286	1	0.12469191	0.48483455
L1_original_firstorder_kurtosis	CT	mm	2.73392417	2.70000566	2.90383135	2.9347769	2.89801158	2.89474512	0.31455541	0.08813787	1	0.10312783	0.31919818
L1_original_firstorder_maximum	CT	px	36.6	37.5	43.7	43.5	47.2	48	0.0096713	0.00155356	0.08649655	0.09420038	0.26384997
L1_original_firstorder_interquartilerange	CT	mm	11.4	11	10.9	11	10.6	11	1	1	0.86634305	0.08635005	-0.1946472
L1_original_firstorder_minimum	CT	mm	1.9	2.5	-5.3	-5.5	-8.3	-7.5	0.0012113	0.00095592	0.03546175	0.06624691	0.23210162
L1_original_firstorder_range	CT	px	30.6	31.5	41.7	42.5	52.1	50.5	0.00682353	0.00096706	0.00188492	0.0628466	0.12199569
L1_original_firstorder_range	CT	mm	42.8	42.5	53.4	52.5	62.6	62.5	0.00105369	0.00107774	0.005074	0.05717702	0.03903991
L1_original_firstorder_minimum	CT	px	6	6	2	2	-4.9	-4	0.06325957	0.00104775	0.00278062	0.05097183	0.03524912
L1_original_firstorder_maximum	CT	mm	44.7	44.5	48.1	48	54.3	54	0.2100106	0.00218357	0.01061548	0.031522	0.04061029
L1_original_firstorder_skewness	CT	mm	0.10987572	0.06114555	0.02610955	0.0421395	0.01289748	0.01480492	1	0.21277793	1	0.03094507	0.70844273
L1_original_firstorder_totalenergy	CT	mm	34436172.7	34317207.1	278549189	278543837	2234942911	2235281771	6.4951E-05	6.4951E-05	6.4951E-05	7.6749E-08	1.9113E-07
L1_original_firstorder_energy	CT	mm	372484291	371197481	3012971214	3.013E+09	2.4175E+10	2.4178E+10	6.4951E-05	6.4951E-05	6.4951E-05	7.6749E-08	1.9113E-07
L1_original_firstorder_totalenergy	CT	px	2800777.09	2804893.11	22573879.1	22629456	177734243	177873469	6.4951E-05	6.4951E-05	6.4951E-05	-4.262E-06	-1.037E-05
L1_original_firstorder_energy	CT	px	30295046.9	30339568.5	244173922	244775075	1922490458	1923996421	6.4951E-05	6.4951E-05	6.4951E-05	-4.262E-06	-1.037E-05

CT images: GLCM-features

feature	image_type	mm_or_px	mean4	median4	mean8	median8	mean16	median16	mwu4.8	mwu4.16	mwu8.16	occc4.8.16	occc8.16
L1_original_glcm_jointentropy	CT	mm	1.88768628	1.89939115	1.88910396	1.8982336	1.88124326	1.88583253	1	1	1	0.66386215	0.64194179
L1_original_glcm_sumentropy	CT	mm	1.47629178	1.48699797	1.47333543	1.4797184	1.46868954	1.47028056	1	1	1	0.6385108	0.60406615
L1_original_glcm_sumsquares	CT	mm	0.23408112	0.23548014	0.23095038	0.2325787	0.22980133	0.2309011	1	1	1	0.61914083	0.66084215
L1_original_glcm_jointenergy	CT	mm	0.29553829	0.29483142	0.30356728	0.2990238	0.3052255	0.30222219	1	1	1	0.60017364	0.6944847
L1_original_glcm_contrast	CT	px	0.41181972	0.42068284	0.40911055	0.4047887	0.40895469	0.4155996	1	1	1	0.579992	0.60619579
L1_original_glcm_differenceaverage	CT	px	0.41181972	0.42068284	0.40767263	0.4047887	0.40722757	0.41392448	1	1	1	0.56096295	0.59585927
L1_original_glcm_idm	CT	px	0.79409014	0.78965858	0.79630748	0.7976056	0.79655892	0.79325982	1	1	1	0.55679092	0.59343007
L1_original_glcm_id	CT	px	0.79409014	0.78965858	0.79640334	0.7976056	0.79667407	0.79341048	1	1	1	0.55394295	0.59174002
L1_original_glcm_inversevariance	CT	px	0.41181972	0.42068284	0.40641444	0.4047887	0.40571635	0.41195485	1	1	1	0.54183458	0.58428026
L1_original_glcm_id	CT	mm	0.79544469	0.79717081	0.7959346	0.7945484	0.79727898	0.79653917	1	1	1	0.54028843	0.68919068
L1_original_glcm_idm	CT	mm	0.79540161	0.79717081	0.7958277	0.7944503	0.79718722	0.79645911	1	1	1	0.54027276	0.68742707
L1_original_glcm_differenceaverage	CT	mm	0.409326	0.40576065	0.40866529	0.4113937	0.40590084	0.40732196	1	1	1	0.54016814	0.68472198
L1_original_glcm_inversevariance	CT	mm	0.40876064	0.40565839	0.40726225	0.4101064	0.40469648	0.40627118	1	1	1	0.53986841	0.69599128
L1_original_glcm_contrast	CT	mm	0.40997213	0.40635768	0.41026877	0.4128649	0.40727726	0.40852285	1	1	1	0.53836546	0.67029192
L1_original_glcm_clusterprominence	CT	mm	0.55563842	0.55419482	0.56435487	0.5623473	0.55938405	0.55829256	1	1	1	0.51108352	0.33259485
L1_original_glcm_clustertendency	CT	mm	0.52635234	0.52827826	0.51353275	0.5169553	0.51192807	0.5131122	1	1	1	0.50476826	0.55715627
L1_original_glcm_differencevariance	CT	mm	0.23851823	0.23759429	0.24069958	0.2411741	0.24021586	0.24034377	0.31455541	0.63073459	1	0.48772415	0.63812195
L1_original_glcm_maximumprobability	CT	mm	0.41710423	0.42593818	0.44326972	0.436267	0.44731097	0.44225936	1	0.73803286	1	0.4692604	0.70186515
L1_original_glcm_maximumprobability	CT	px	0.46889051	0.46259997	0.43302527	0.4383765	0.44569919	0.43971803	1	1	1	0.45465583	0.51191833
L1_original_glcm_differenceentropy	CT	mm	0.96682059	0.96378571	0.97559118	0.9768876	0.97412314	0.97428008	0.17283336	0.06897746	1	0.45217124	0.60366613
L1_original_glcm_clustershade	CT	mm	0.14870255	0.16127367	0.1704935	0.1664745	0.17741013	0.17426875	1	1	1	0.38073442	0.61833612
L1_original_glcm_clustershade	CT	px	0.07605837	0.12572515	0.13407977	0.1678484	0.16775574	0.16776815	1	0.99296369	1	0.37227349	0.36411431
L1_original_glcm_sumsquares	CT	px	0.213306	0.22327468	0.2286722	0.2314286	0.22983406	0.23104867	1	1	1	0.30366794	0.52693375
L1_original_glcm_jointenergy	CT	px	0.3525419	0.33937879	0.30905867	0.3063224	0.30687465	0.30169977	0.99296369	1	1	0.27925327	0.50635551
L1_original_glcm_jointentropy	CT	px	1.70083362	1.73415953	1.85703424	1.8809061	1.88221601	1.89139498	0.63073459	0.17283336	1	0.22969427	0.50620146
L1_original_glcm_correlation	CT	mm	0.12387906	0.12214582	0.111684	0.1104311	0.11394504	0.11415121	0.63073459	0.85884085	1	0.12877984	-0.1844691
L1_original_glcm_differencevariance	CT	px	0.2208461	0.21760546	0.23813632	0.2378012	0.24004394	0.24151133	0.00435169	0.00077941	0.73803286	0.08849975	0.51776121
L1_original_glcm_differenceentropy	CT	px	0.9115512	0.90271328	0.96575625	0.9642988	0.97354808	0.97659288	0.00292277	0.00077941	0.73803286	0.08509437	0.51729599
L1_original_glcm_sumentropy	CT	px	1.2890139	1.31156748	1.44529962	1.4748437	1.46762186	1.47014107	0.11126026	0.03117625	1	0.08455116	0.43249827
L1_original_glcm_idn	CT	px	0.86272676	0.85977239	0.87135028	0.8666253	0.89827946	0.89662991	1	6.4951E-05	0.00435169	0.07628005	-0.018293
L1_original_glcm_mcc	CT	mm	0.13807601	0.13518985	0.11969468	0.1171714	0.11624109	0.11598108	0.25954232	0.02331724	1	0.07447944	-0.2673497
L1_original_glcm_imc1	CT	mm	-0.0246434	-0.0235237	-0.0187266	-0.0183893	-0.0181357	-0.0182266	0.08813787	0.06897746	1	0.05824245	-0.2014361
L1_original_glcm_imc2	CT	mm	0.16442797	0.16061896	0.13840518	0.1376276	0.13510977	0.13463845	0.05358419	0.04104873	1	0.04744449	-0.33806
L1_original_glcm_clustertendency	CT	px	0.44140427	0.45330736	0.50557825	0.5199502	0.51038153	0.51216728	0.53525731	0.31455541	1	0.04099457	0.4266269
L1_original_glcm_sumaverage	CT	px	2.8170951	2.79905568	3.13713347	2.8117992	4.6941102	4.70500739	1	6.4951E-05	0.00435169	0.02603659	0.01953618
L1_original_glcm_jointaverage	CT	px	1.40854755	1.39952784	1.56856673	1.4058996	2.3470551	2.3525037	1	6.4951E-05	0.00435169	0.02603659	0.01953618
L1_original_glcm_autocorrelation	CT	px	2.01973279	1.99638628	2.64126779	2.0129701	5.53529363	5.55928023	1	6.4951E-05	0.00435169	0.02175096	0.02320792
L1_original_glcm_imc2	CT	px	0.29723587	0.30356788	0.16895345	0.1804029	0.13937666	0.13832095	0.0001299	6.4951E-05	1	0.01974763	0.14167518
L1_original_glcm_autocorrelation	CT	mm	2.68366477	1.95681544	5.54550421	5.5713763	5.53675223	5.55238288	0.08813787	0.11126026	1	0.01006565	0.69309295
L1_original_glcm_imc1	CT	px	-0.0839864	-0.0815354	-0.0255733	-0.0265994	-0.019335	-0.0196927	0.00045465	6.4951E-05	0.85884085	0.00949984	0.12926523
L1_original_glcm_sumaverage	CT	mm	3.15449616	2.77665226	4.69862436	4.7097667	4.69486172	4.70191577	0.06897746	0.11126026	1	0.00808968	0.70045157
L1_original_glcm_jointaverage	CT	mm	1.57724808	1.38832613	2.34931218	2.3548834	2.34743086	2.35095789	0.06897746	0.11126026	1	0.00808968	0.70045157
L1_original_glcm_mcc	CT	px	0.24911189	0.25723289	0.1409513	0.1458452	0.11932345	0.11602467	0.0001299	6.4951E-05	1	0.00437841	0.08212925
L1_original_glcm_clusterprominence	CT	px	0.44130211	0.47744669	0.53784124	0.5549073	0.56382826	0.55630102	0.31455541	0.01253545	1	-0.0017824	0.36015353
L1_original_glcm_idmn	CT	mm	0.92672184	0.92023126	0.96077171	0.9589635	0.97281749	0.97568217	0.00292277	6.4951E-05	0.00194852	-0.0019259	0.01665087
L1_original_glcm_idmn	CT	px	0.91763606	0.91586343	0.92699116	0.9199752	0.95918424	0.95851735	1	6.4951E-05	0.00435169	-0.007343	-0.0199385
L1_original_glcm_idn	CT	mm	0.87077517	0.8670521	0.9000009	0.897634	0.91492712	0.91763605	0.00292277	6.4951E-05	0.00194852	-0.009761	-0.0206308
L1_original_glcm_correlation	CT	px	0.0275006	0.03475595	0.1027175	0.1064209	0.11034248	0.11042709	0.25954232	0.00902812	1	-0.1318347	0.26875027

CT images: GLDM-features

feature	image_type	mm_or_px	mean4	median4	mean8	median8	mean16	median16	mwu4.8	mwu4.16	mwu8.16	occc4.8.16	occc8.16
L1_original_gldm_graylevelvariance	CT	mm	0.23468686	0.23713169	0.23091438	0.2330154	0.22961396	0.23076375	1	1	1	0.65065368	0.68650398
L1_original_gldm_dependencevariance	CT	mm	24.3605677	23.7691331	26.7265476	26.276685	25.6828707	25.3486695	1	1	1	0.4438942	0.50148071
L1_original_gldm_graylevelvariance	CT	px	0.21791453	0.23296076	0.23101386	0.2353672	0.22923553	0.23064953	1	1	1	0.36558897	0.61849637
L1_original_gldm_dependencenonuniformitynormalized	CT	mm	0.05879241	0.05800608	0.05294218	0.0530183	0.05400856	0.0542725	0.08813787	0.13938384	1	0.24555669	0.49355044
L1_original_gldm_dependenceentropy	CT	mm	5.00334905	4.99820719	5.03982134	5.0401247	4.96000842	4.96043384	0.85884085	1	6.4951E-05	0.23612933	0.0975559
L1_original_gldm_largedependencelowgraylevelemphasis	CT	px	61.4776988	63.6215054	123.804833	130.56323	51.8050432	50.6744888	0.05358419	1	0.13938384	0.139975	0.05566208
L1_original_gldm_dependencevariance	CT	px	11.3236926	9.70098619	25.794016	24.299242	26.7739109	25.7526501	0.00077941	6.4951E-05	1	0.09112738	0.38734748
L1_original_gldm_smalldependencehighgraylevelemphasis	CT	px	0.09970805	0.06048182	0.05703746	0.0456862	0.07328288	0.07317401	0.45153608	1	0.13938384	0.05307971	-0.0216332
L1_original_gldm_largedependenceemphasis	CT	mm	186.527002	187.394335	239.056293	236.34488	267.378852	265.997652	6.4951E-05	6.4951E-05	0.00045465	0.04925591	0.13643167
L1_original_gldm_largedependenceemphasis	CT	px	80.4442394	76.8872679	175.769844	173.72385	231.801505	226.825274	6.4951E-05	6.4951E-05	6.4951E-05	0.03668995	0.11335932
L1_original_gldm_lowgraylevelemphasis	CT	px	0.69779905	0.69274194	0.6146702	0.6977942	0.20325581	0.20241097	1	6.4951E-05	0.00077941	0.0254282	0.00456839
L1_original_gldm_largedependencelowgraylevelemphasis	CT	mm	131.737938	144.124284	53.3762311	52.5292	59.889521	59.3103664	0.13938384	0.13938384	0.00435169	0.02455787	0.2286636
L1_original_gldm_highgraylevelemphasis	CT	px	2.2088038	2.22903226	2.86071329	2.2088234	5.73355209	5.74572906	1	6.4951E-05	0.0063002	0.02441617	0.0251526
L1_original_gldm_dependencenonuniformitynormalized	CT	px	0.12268044	0.12433611	0.06108793	0.0634397	0.05318925	0.05377661	6.4951E-05	6.4951E-05	0.21277793	0.01081483	0.24900523
L1_original_gldm_highgraylevelemphasis	CT	mm	2.89210081	2.1597153	5.74917525	5.7886605	5.73758659	5.75306953	0.13938384	0.13938384	1	0.00971678	0.70175571
L1_original_gldm_lowgraylevelemphasis	CT	mm	0.61529294	0.71007117	0.20273715	0.2016945	0.20276723	0.20234086	0.13938384	0.13938384	1	0.00139832	0.6798136
L1_original_gldm_dependencenonuniformity	CT	px	3.56135164	3.56527094	14.2734592	14.969849	98.0198202	99.1372035	6.4951E-05	6.4951E-05	6.4951E-05	0.00100802	0.00215596
L1_original_gldm_smalldependencelowgraylevelemphasis	CT	px	0.0181152	0.01830842	0.00820695	0.0077144	0.00289212	0.00282086	0.0002598	6.4951E-05	6.4951E-05	0.00086497	0.01955431
L1_original_gldm_graylevelnonuniformity	CT	px	16.3531065	15.7482759	126.45107	124.63326	1005.91524	1001.51581	0.00108991	0.00108991	6.4951E-05	0.00038514	0.00065411
L1_original_gldm_dependencenonuniformity	CT	mm	20.9637835	20.5915493	152.86865	153.45222	1251.17043	1257.33144	6.4951E-05	6.4951E-05	6.4951E-05	0.00015948	0.00026932
L1_original_gldm_graylevelnonuniformity	CT	mm	189.760685	188.0081	1566.84846	1557.1334	12609.0638	12560.0383	6.4951E-05	6.4951E-05	6.4951E-05	4.974E-05	9.3029E-05
L1_original_gldm_largedependencehighgraylevelemphasis	CT	px	156.310402	138.948157	410.700637	324.79134	1148.62914	1152.22605	0.00077941	6.4951E-05	6.4951E-05	-0.0029399	-0.0078166
L1_original_gldm_largedependencehighgraylevelemphasis	CT	mm	445.913613	329.93193	1186.31268	1188.8316	1320.01017	1320.37413	6.4951E-05	6.4951E-05	6.4951E-05	-0.0032056	0.0131292
L1_original_gldm_smalldependenceemphasis	CT	mm	0.01462295	0.01513841	0.01061818	0.0104812	0.00855169	0.00841315	0.00045465	6.4951E-05	6.4951E-05	-0.0036212	0.03768144
L1_original_gldm_dependenceentropy	CT	px	3.67045129	3.712751	4.87772094	4.8190094	5.03948215	5.04240513	6.4951E-05	6.4951E-05	0.21277793	-0.0057171	0.14940487
L1_original_gldm_smalldependencelowgraylevelemphasis	CT	mm	0.00662955	0.00673857	0.00290594	0.0028205	0.00227946	0.0022421	6.4951E-05	6.4951E-05	0.00902812	-0.0169611	0.1097383
L1_original_gldm_smalldependenceemphasis	CT	px	0.03443377	0.02950891	0.01635451	0.0163036	0.01121384	0.01089161	0.00194852	6.4951E-05	0.00045465	-0.0209243	0.09564538
L1_original_gldm_smalldependencehighgraylevelemphasis	CT	mm	0.05395038	0.0466587	0.06822193	0.067207	0.05566091	0.05479166	0.17283336	0.85884085	0.0001299	-0.0823641	0.06768665

CT images: GLRLM-features

feature	image_type	mm_or_px	mean4	median4	mean8	median8	mean16	median16	mwu4.8	mwu4.16	mwu8.16	occc4.8.16	occc8.16
L1_original_glrlm_graylevelvariance	CT	mm	0.24874376	0.24716855	0.25639235	0.2561985	0.25649795	0.25642316	0.04104873	0.08813787	1	0.14333718	0.23675854
L1_original_glrlm_longrunlowgraylevelemphasis	CT	px	1.74465814	1.77308889	2.97346869	3.2575713	1.4771488	1.43108241	0.13938384	1	0.13938384	0.13673141	0.08602101
L1_original_glrlm_graylevelvariance	CT	px	0.23190806	0.24192385	0.24995433	0.2471399	0.25545762	0.25369781	0.13938384	6.4951E-05	0.11126026	0.13125711	0.44347597
L1_original_glrlm_graylevelnonuniformitynormalized	CT	mm	0.5053271	0.50566291	0.49890785	0.4985366	0.49744081	0.49753634	0.02331724	0.00435169	0.01727684	0.08920448	0.22937515
L1_original_glrlm_graylevelnonuniformitynormalized	CT	px	0.53618389	0.51615229	0.50595082	0.5057203	0.500557	0.50135649	0.13938384	0.00045465	0.63073459	0.08551983	0.37228294
L1_original_glrlm_longrunlowgraylevelemphasis	CT	mm	3.25873585	3.62090162	1.56103495	1.5231226	1.97821332	1.94524069	0.13938384	0.13938384	0.0001299	0.0512665	0.13931603
L1_original_glrlm_runvariance	CT	mm	1.32907156	1.3076071	2.53748183	2.4641284	3.5329792	3.46190427	6.4951E-05	6.4951E-05	0.0001299	0.03445747	0.11545805
L1_original_glrlm_longrunemphasis	CT	mm	4.86792622	4.8882908	7.15696265	7.0187899	8.96055085	8.85294491	6.4951E-05	6.4951E-05	6.4951E-05	0.03005744	0.09943189
L1_original_glrlm_runpercentage	CT	mm	0.54892784	0.54616855	0.47810792	0.4806144	0.44056961	0.44198442	6.4951E-05	6.4951E-05	6.4951E-05	0.02905028	0.08336154
L1_original_glrlm_runpercentage	CT	px	0.71988356	0.7271903	0.56788134	0.5668536	0.48803134	0.4936304	6.4951E-05	6.4951E-05	6.4951E-05	0.02706039	0.07187249
L1_original_glrlm_shortrunemphasis	CT	px	0.73002503	0.7379403	0.62298249	0.615052	0.57234144	0.57474921	6.4951E-05	6.4951E-05	6.4951E-05	0.02543216	0.07347687
L1_original_glrlm_runlengthnonuniformitynormalized	CT	mm	0.36922698	0.36599786	0.32318844	0.3232968	0.29758721	0.29804917	6.4951E-05	6.4951E-05	6.4951E-05	0.02489006	0.03642074
L1_original_glrlm_shortrunemphasis	CT	mm	0.60575677	0.60356617	0.56565924	0.5658461	0.53904872	0.53931048	6.4951E-05	6.4951E-05	6.4951E-05	0.02358644	0.01564135
L1_original_glrlm_longrunemphasis	CT	px	2.41467125	2.35419987	4.46146841	4.442112	6.77906951	6.561514	6.4951E-05	6.4951E-05	6.4951E-05	0.02265386	0.0511425
L1_original_glrlm_runlengthnonuniformitynormalized	CT	px	0.54639711	0.55363298	0.3877986	0.3839465	0.32987377	0.33276506	6.4951E-05	6.4951E-05	6.4951E-05	0.01984636	0.08028783
L1_original_glrlm_runvariance	CT	px	0.35270318	0.34571169	1.16879388	1.1414781	2.31981909	2.23340791	6.4951E-05	6.4951E-05	6.4951E-05	0.0191434	0.05093948
L1_original_glrlm_shortrunhighgraylevelemphasis	CT	px	1.71362364	1.68708771	2.08472559	1.6015521	3.83570821	3.82988096	1	6.4951E-05	0.13938384	0.01723222	0.00580701
L1_original_glrlm_lowgraylevelrunemphasis	CT	px	0.68296259	0.68309453	0.57351722	0.6625692	0.1905337	0.18968302	1	6.4951E-05	0.0002598	0.01269177	-0.0009497
L1_original_glrlm_highgraylevelrunemphasis	CT	px	2.26814963	2.26762189	3.09598744	2.3497233	6.24273857	6.24689	1	6.4951E-05	0.00045465	0.00803712	0.00443429
L1_original_glrlm_runentropy	CT	mm	2.70769171	2.708665	3.03042326	3.024263	3.18077241	3.17643222	6.4951E-05	6.4951E-05	6.4951E-05	0.00234213	0.01722195
L1_original_glrlm_runlengthnonuniformity	CT	px	11.7345627	11.5954998	52.6262497	51.76357	302.918095	308.97766	6.4951E-05	6.4951E-05	6.4951E-05	0.00120182	0.00208773
L1_original_glrlm_shortrunlowgraylevelemphasis	CT	px	0.48412538	0.47360452	0.33002455	0.3741527	0.10289247	0.10222616	6.4951E-05	6.4951E-05	6.4951E-05	0.00041643	-0.0077842
L1_original_glrlm_runlengthnonuniformity	CT	mm	73.9575979	73.4897223	454.696495	457.15674	3092.3831	3106.73047	6.4951E-05	6.4951E-05	6.4951E-05	0.00019492	0.00035852
L1_original_glrlm_graylevelnonuniformity	CT	mm	98.9423371	98.8768996	688.863959	692.68344	5077.59332	5096.84228	6.4951E-05	6.4951E-05	6.4951E-05	4.9004E-05	0.00010001
L1_original_glrlm_graylevelnonuniformity	CT	px	11.1754941	11.2328853	67.174953	67.562443	450.176649	453.570941	6.4951E-05	6.4951E-05	6.4951E-05	1.1272E-05	9.9164E-05
L1_original_glrlm_highgraylevelrunemphasis	CT	mm	3.12051144	2.34503375	6.28479225	6.2906632	6.36920306	6.36831848	0.00435169	6.4951E-05	6.4951E-05	-0.0010077	0.04162678
L1_original_glrlm_lowgraylevelrunemphasis	CT	mm	0.57203991	0.66374156	0.18928088	0.1889853	0.1865181	0.18671078	0.00194852	6.4951E-05	0.00435169	-0.0013292	0.06905783
L1_original_glrlm_runentropy	CT	px	1.89518452	1.87452557	2.60190479	2.6014686	2.98367898	2.96911975	6.4951E-05	6.4951E-05	6.4951E-05	-0.0022178	0.00931402
L1_original_glrlm_shortrunlowgraylevelemphasis	CT	mm	0.3194938	0.36225616	0.10079908	0.1004142	0.09347254	0.0933615	0.0001299	6.4951E-05	6.4951E-05	-0.0036241	0.05565234
L1_original_glrlm_shortrunhighgraylevelemphasis	CT	mm	2.03343785	1.5632337	3.82215923	3.8308939	3.72499991	3.73103716	0.13938384	0.13938384	0.00194852	-0.006244	-0.0192674
L1_original_glrlm_longrunhighgraylevelemphasis	CT	mm	12.6147036	9.2305881	36.9491592	36.663604	45.3613518	45.1382984	6.4951E-05	6.4951E-05	6.4951E-05	-0.0065487	0.03945308
L1_original_glrlm_longrunhighgraylevelemphasis	CT	px	5.09472367	4.97628212	11.4226781	9.0537472	35.0639649	34.5164806	0.0001299	6.4951E-05	6.4951E-05	-0.0075458	-0.0179312

<u>CT images: GLSZM-features</u>

feature	image_type	mm_or_px	mean4	median4	mean8	median8	mean16	median16	mwu4.8	mwu4.16	mwu8.16	occc4.8.16	occc8.16
L1_original_glszm_smallarealowgraylevelemphasis	CT	px	0.0240691	0.00348759	0.12669162	0.0083387	0.34834737	0.31547625	1	6.4951E-05	0.17283336	0.1311646	0.33970939
L1_original_glszm_smallarealowgraylevelemphasis	CT	mm	0.10569814	0.07063439	0.47945664	0.5039683	0.60540848	0.60833085	0.00077941	6.4951E-05	0.31455541	0.08394301	0.21586801
L1_original_glszm_sizezonenonuniformity	CT	px	1	1	1.05	1	2.29785714	2.35714286	1	0.00136058	0.00409271	0.04332199	0.08885291
L1_original_glszm_sizezonenonuniformitynormalized	CT	px	0.45	0.5	0.4125	0.4375	0.33251861	0.33333333	1	0.05815018	0.5349342	0.01922242	0.12749347
L1_original_glszm_sizezonenonuniformity	CT	mm	1.12	1	3.84424242	3.5	23.6373274	23.038115	0.00071762	0.0005247	0.00108381	0.00495875	0.01680368
L1_original_glszm_graylevelnonuniformity	CT	mm	1.75666667	1.33333333	4.08197691	3.8035714	22.3631525	21.565812	0.0022881	0.00095592	0.00107169	0.00209075	0.01508754
L1_original_glszm_smallareaemphasis	CT	mm	0.22277746	0.28252365	0.63839572	0.6475696	0.77576947	0.7763123	0.00045465	6.4951E-05	0.00045465	0.00188677	0.03717308
L1_original_glszm_largeareahighgraylevelemphasis	CT	mm	57486.2667	50378.6667	2826431.8	2881250	36951608.4	37601803.3	6.4951E-05	6.4951E-05	6.4951E-05	0.00161696	0.00368354
L1_original_glszm_largeareaemphasis	CT	mm	23177.31	21240	550950.303	567911.81	7227545.35	7296048.56	6.4951E-05	6.4951E-05	6.4951E-05	0.00155033	0.00369584
L1_original_glszm_largearealowgraylevelemphasis	CT	mm	16085.4412	13936.1979	120442.911	125045.46	1583514.25	1579490.39	6.4951E-05	6.4951E-05	6.4951E-05	0.00132781	0.00377547
L1_original_glszm_zonevariance	CT	mm	6569.40028	7803.44444	420919.023	438265.68	6888776.99	6951898.72	6.4951E-05	6.4951E-05	6.4951E-05	0.00105507	0.00213261
L1_original_glszm_largeareahighgraylevelemphasis	CT	px	421.25	433	27102.95	25291	1538400.76	1447959.88	6.4951E-05	6.4951E-05	6.4951E-05	6.5099E-05	0.00014114
L1_original_glszm_zonevariance	CT	px	33.25	27.6111111	2428.52708	2710.2361	195659.27	199536.843	0.00107169	0.00107169	6.4951E-05	-0.0002738	-0.0005687
L1_original_glszm_largeareaemphasis	CT	px	211.2	214.5	11775.1083	12211.083	297777.188	281242.917	0.00108991	0.00108991	6.4951E-05	-0.0004214	-0.000845
L1_original_glszm_largearealowgraylevelemphasis	CT	px	158.6875	163	8392.8794	9492.8333	64797.9708	60780.7986	6.4951E-05	6.4951E-05	6.4951E-05	-0.0018599	-0.0042342
L1_original_glszm_zonepercentage	CT	px	0.079862	0.07019704	0.01155633	0.010643	0.00369051	0.00352834	0.00105967	0.00105967	6.4951E-05	-0.0043641	0.03674573
L1_original_glszm_graylevelnonuniformity	CT	px	1.2	1	1.33333333	1	3.09293651	3	1	0.00450241	0.01029485	-0.0047071	0.0535427
L1_original_glszm_sizezonenonuniformitynormalized	CT	mm	0.369	0.33333333	0.43440983	0.4375	0.56672763	0.56844682	1	0.0010242	0.0059949	-0.0076095	0.01373659
L1_original_glszm_zoneentropy	CT	px	1.17548875	1	1.32548875	1.25	2.18057547	2.18200581	1	0.00117221	0.00456213	-0.0118916	0.01125255
L1_original_glszm_smallareaemphasis	CT	px	0.07858126	0.00713545	0.15672	0.0333137	0.5076394	0.53009307	1	0.0019688	0.00292277	-0.0176735	-0.0101048
L1_original_glszm_zonepercentage	CT	mm	0.0089571	0.00847464	0.00301243	0.0027773	0.00179134	0.00170602	6.4951E-05	6.4951E-05	0.0001299	-0.0239239	0.12250538
L1_original_glszm_graylevelvariance	CT	px	0.24166667	0.25	0.32361111	0.25	0.71112999	0.73343411	1	0.0007619	0.00813362	-0.0354547	-0.0776519
L1_original_glszm_graylevelvariance	CT	mm	0.30280556	0.23611111	0.7660646	0.7755102	0.9041133	0.88302433	0.00578616	0.00188831	1	-0.0407084	0.07808598
L1_original_glszm_smallareahighgraylevelemphasis	CT	mm	0.6911096	0.33351314	2.08707644	1.8750012	2.48516577	2.34289642	0.04104873	0.00123406	1	-0.0700273	0.33745275
L1_original_glszm_smallareahighgraylevelemphasis	CT	px	0.29662994	0.02406254	0.27687095	0.1332139	1.94126726	1.90509738	1	0.00194852	0.00194852	-0.0733945	-0.0622596
L1_original_glszm_highgraylevelzoneemphasis	CT	px	2.55	2.5	2.91666667	2.5	4.90623016	5.4375	1	0.00260493	0.02255499	-0.0831955	-0.1759886
L1_original_glszm_lowgraylevelzoneemphasis	CT	mm	0.49949074	0.4537037	0.59766875	0.6388889	0.7257752	0.71060141	0.26608883	0.00252156	0.22538083	-0.0871934	0.2890403
L1_original_glszm_lowgraylevelzoneemphasis	CT	px	0.6125	0.625	0.58564815	0.625	0.49839837	0.44212963	1	0.49129219	0.88428146	-0.0885238	-0.1201446
L1_original_glszm_highgraylevelzoneemphasis	CT	mm	3.29833333	3.25	4.23689033	3.7142857	3.5866914	3.59166667	0.44737208	1	1	-0.1120379	0.44794428
L1_original_glszm_graylevelnonuniformitynormalized	CT	mm	0.52772222	0.52777778	0.47113958	0.4533833	0.54839865	0.53662516	0.81942311	1	0.22494999	-0.1265792	0.02592911
L1_original_glszm_zoneentropy	CT	mm	1.51258381	1.5849625	1.97700335	2.055241	2.16342711	2.19633354	0.12218235	0.01261247	1	-0.1492447	0.23245152
L1_original_glszm_graylevelnonuniformitynormalized	CT	px	0.51666667	0.5	0.49444444	0.5	0.44631258	0.44428571	1	0.14167238	1	-0.2276782	-0.3793337

CT images: NGTDM-features

feature	image_type	mm_or_px	mean4	median4	mean8	median8	mean16	median16	mwu4.8	mwu4.16	mwu8.16	occc4.8.16	occc8.16
L1_original_ngtdm_busyness	CT	mm	254.586868	203.944847	106.40333	113.35572	546.856563	475.440109	1	0.05358419	6.4951E-05	0.05663788	-8.282E-05
L1_original_ngtdm_complexity	CT	mm	0.65413314	0.40454316	1.72704762	1.6190703	2.57422033	2.82634111	0.01253545	0.00077941	0.25954232	0.02995839	0.09441546
L1_original_ngtdm_complexity	CT	px	0.40087575	0.40609111	0.64532445	0.4051798	1.59252376	1.61602485	1	6.4951E-05	0.01727684	0.02416077	0.03042143
L1_original_ngtdm_contrast	CT	px	0.08939774	0.09343883	0.0802582	0.0889654	0.0307837	0.03146123	1	6.4951E-05	0.00435169	0.00407752	-0.0127667
L1_original_ngtdm_coarseness	CT	px	0.1763346	0.18187224	0.0214084	0.0213634	0.00273731	0.00268605	6.4951E-05	6.4951E-05	6.4951E-05	0.00098068	0.00038794
L1_original_ngtdm_coarseness	CT	mm	0.01394257	0.01386116	0.00173078	0.0017106	0.00021623	0.00021473	6.4951E-05	6.4951E-05	6.4951E-05	0.00016688	0.00015079
L1_original_ngtdm_busyness	CT	px	10.9190974	10.5020189	748.317724	112.01832	71.0101666	72.2819995	0.01253545	6.4951E-05	1	0.00015764	2.8787E-05
L1_original_ngtdm_strength	CT	px	0.17510096	0.17649233	0.02987554	0.0214971	0.00825056	0.00819919	6.4951E-05	6.4951E-05	6.4951E-05	-0.0024864	-0.0035051
L1_original_ngtdm_strength	CT	mm	0.01976724	0.01401757	0.00573146	0.0052776	0.00115708	0.00127225	6.4951E-05	6.4951E-05	6.4951E-05	-0.0054084	0.00405059
L1_original_ngtdm_contrast	CT	mm	0.08139477	0.09121411	0.02951236	0.0314225	0.01833119	0.01574845	0.00123406	6.4951E-05	0.00292277	-0.0054368	-0.0018118
