# Supplementary figures and images for: Stability of Radiomic Features across Different Region of Interest Sizes—A CT and MR Phantom Study
Source: Tomography. 2021 Jun 8;7(2):238–52. doi: 10.3390/tomography7020022 (PMC8293351; doi:10.3390/tomography7020022)

T1 OCCC<sub>8,16</sub>T1 OCCC<sub>4-16</sub>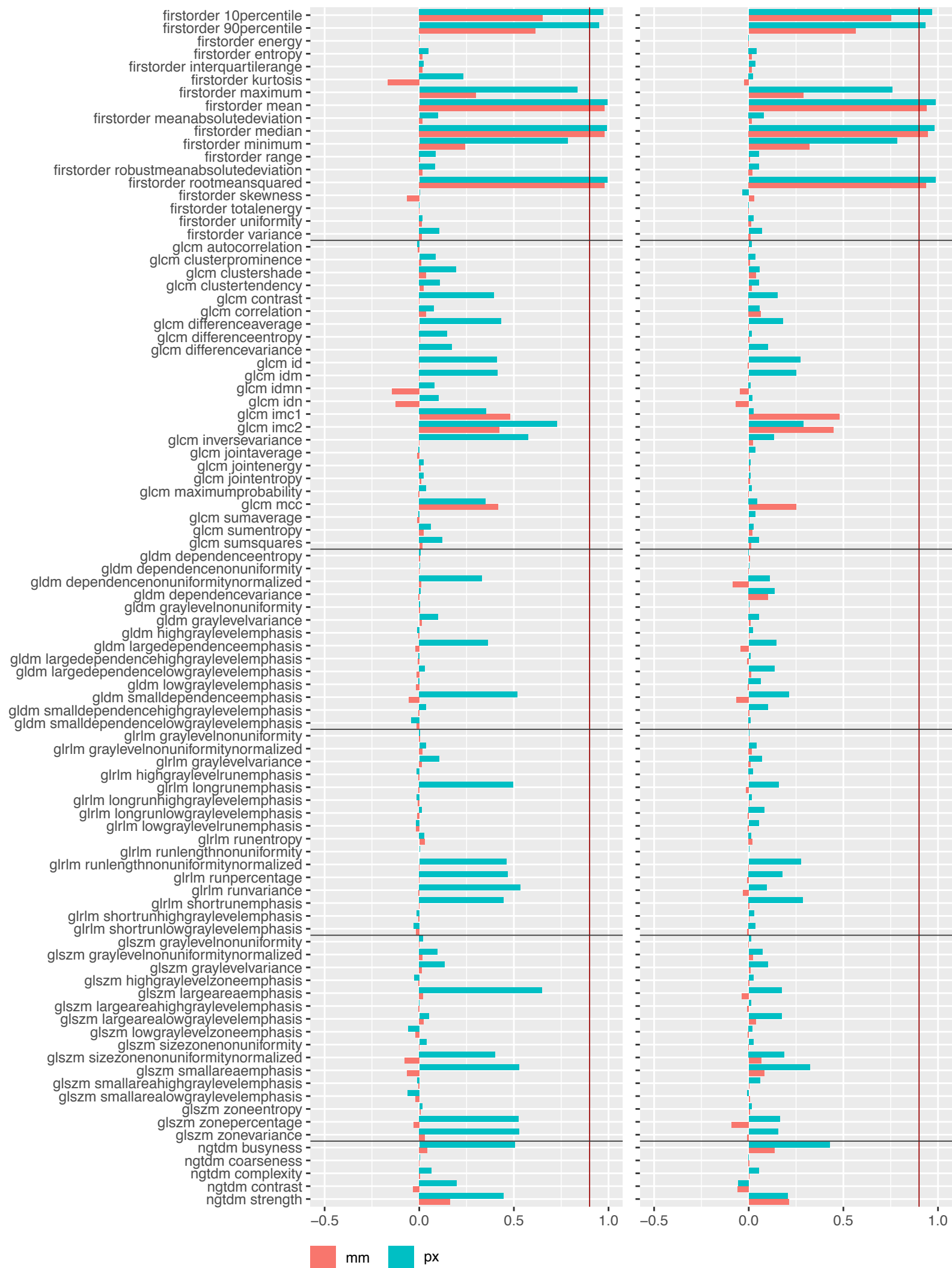

Supplement: Supplementary file 1 [file tomography-07-00022-s001.zip › figure_S1.pdf]

TIRM OCCC<sub>8,16</sub>TIRM OCCC<sub>4-16</sub>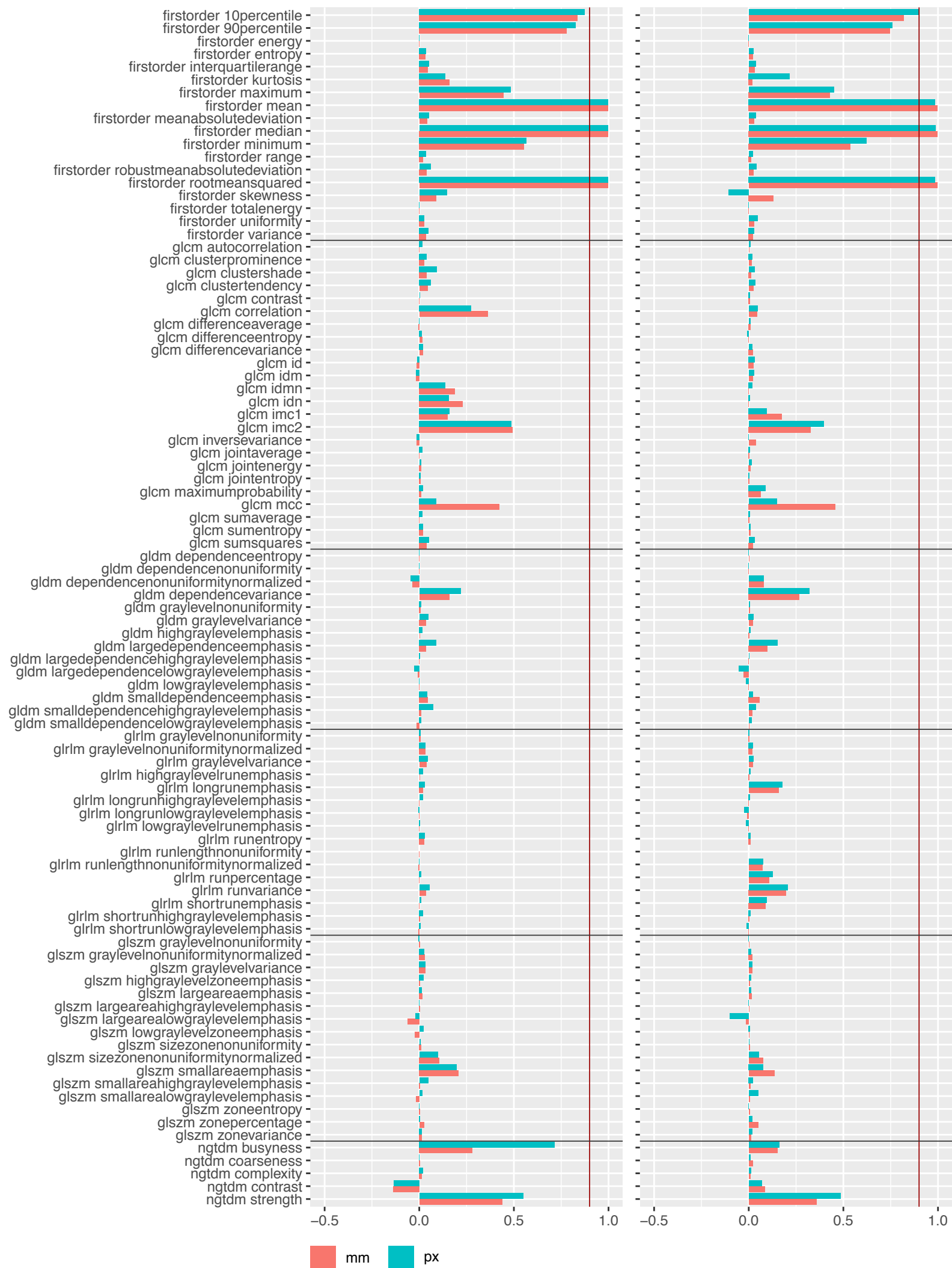

Supplement: Supplementary file 1 [file tomography-07-00022-s001.zip › figure_S2.pdf]

CT OCCC<sub>8,16</sub>CT OCCC<sub>4-16</sub>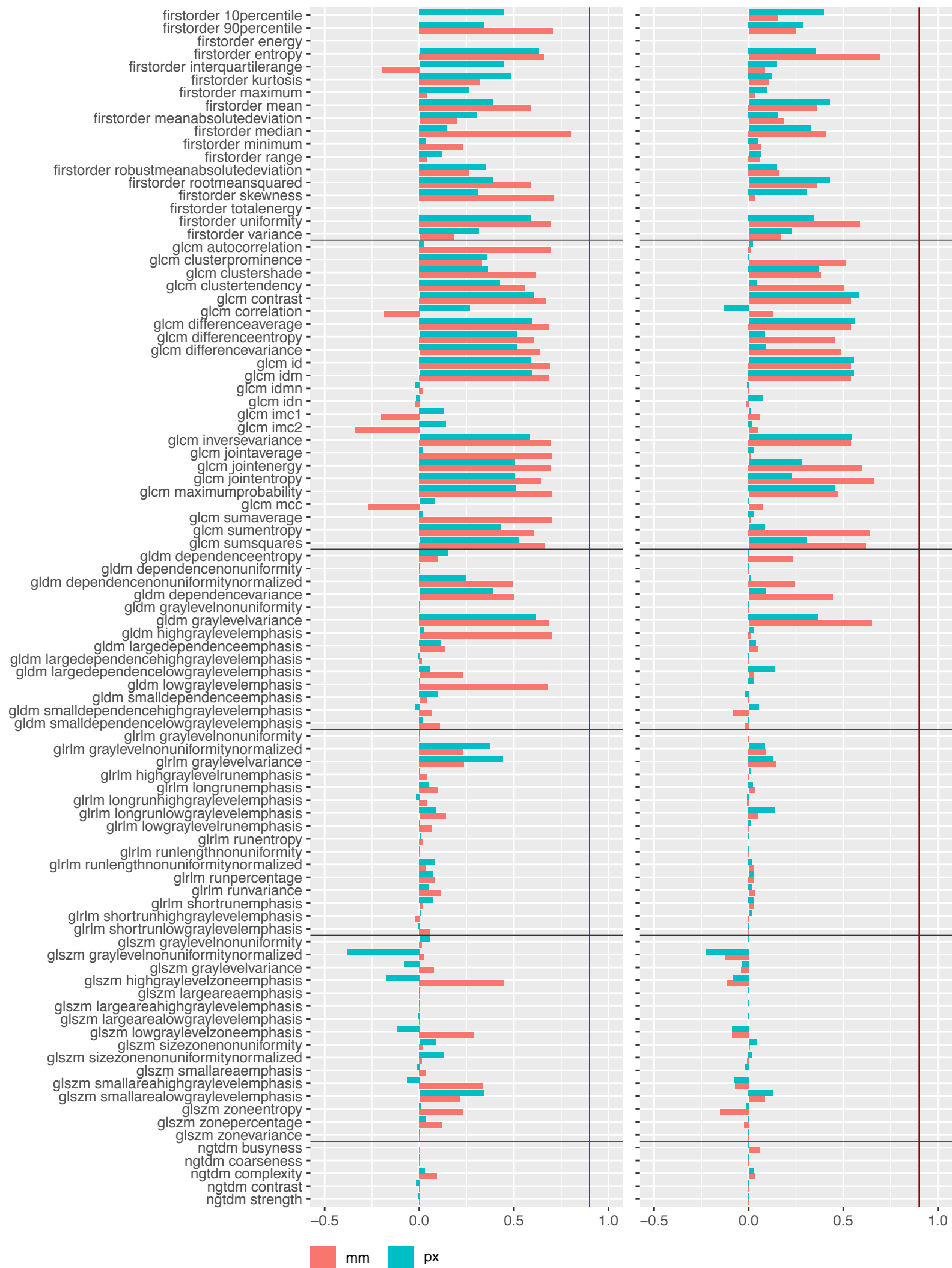

Supplement: Supplementary file 1 [file tomography-07-00022-s001.zip › figure_S3.pdf]
